# Future climate scenarios reshape the suitability of major malaria vector taxa in South America

**DOI:** 10.1007/s00484-026-03244-y

**Published:** 2026-06-23

**Authors:** Janderson Batista Rodrigues Alencar, Izabel Cristina de Oliveira Bentes, Francisco Augusto da Silva Ferreira, Adriano Nobre Arcos, Beatriz Ronchi-Teles, Fabrício Beggiato Baccaro

**Affiliations:** 1 Programa de Pós-Graduação em Biologia (Ecologia), Programa Institucional de Pós-Doutorado (PIPD/CAPES), Instituto Nacional de Pesquisas da Amazônia, Constelação Cruzeiro do Sul Street, Aleixo, Manaus, 69060-062 ZIP, AM Brazil; 2 Laboratório de Malária e Dengue – LMD, Instituto Nacional de Pesquisas da Amazônia, Manaus, Amazonas Brasil; 3 Coordenação de Biodiversidade, Instituto Nacional de Pesquisas da Amazonia, Manaus, Amazonas, Brasil; 4 Sinteses da Biodiversidade Amazônica – INCT SinBiAm, Universidade Federal do Pará, Belém, Pará Brazil; 5 Coordenaçáo de Tecnologia e Inovação, Instituto Nacional de Pesquisas da Amazônia, INPA Campus III, Manaus, Amazonas 69060-062 Brazil; 6 Department of Biological Sciences, Columbian College of Arts and Sciences, The George Washington University, Washington, DC, 20052 USA

**Keywords:** *Anopheles*, Ecological niche, Climate change, Epidemiology

## Abstract

Malaria remains a major health burden in South America, but the extent to which climate change will reshape vector communities is poorly understood. We modeled the distributions of 15 key *Anopheles* species, representing primary, secondary, and unclassified vectors, under current and future climate scenarios (SSP2–4.5, SSP5–8.5) using ensemble ecological niche models calibrated with 20 climatic and physiographic predictors. We quantified niche breadth and overlap and achieved high predictive performance. Analyses in environmental space revealed moderate overlap between primary and secondary vectors and lower overlap between primary and unclassified vectors, although ecological breadth did not differ significantly among classes. Future projections revealed divergent trajectories among *Anopheles* species. Climate-sensitive primary vectors such as *An. darlingi* is projected to undergo severe contractions and fragmentation, particularly within the Amazon Basin, whereas *An. albimanus* and *An. aquasalis* are predicted to expand southward under SSP5–8.5. Several secondary or unclassified species (*An. janconnae*, *An. marajoara*, *An. argyritarsis*) also showed potential for expansion. These results suggest a major reorganization of vector assemblages, with potential epidemiological implications. Understanding this reshuffling and clarifying the roles of emerging vectors will be crucial for guiding future malaria control and surveillance strategies in South America.

**Supplementary Information:**

The online version contains supplementary material available at 10.1007/s00484-026-03244-y.

## Introduction

Malaria remains a major global public health challenge, with the World Health Organization (WHO) estimating 282 million cases and 610,000 deaths worldwide across 80 endemic countries (WHO 2025). In South America, the malaria burden is concentrated mainly in the Amazon region, where transmission occurs through the bites of *Anopheles* mosquitoes infected with *Plasmodium* parasites and is strongly shaped by local environmental conditions (Fonseca et al. 2023). *Anopheles darlingi* is a critical vector in this region, with its distribution strongly influenced by climatic and environmental variables such as temperature, humidity, and water availability (Laporta et al. 2015; Ferreira et al. 2021).

In South America, around 90 *Anopheles* species have been recorded, although only a minority are recognized as epidemiologically important malaria vectors (Consoli and Oliveira 1994; Sinka et al. 2010, 2011; Kirchgatter et al. 2014; Tadei et al. 2017; Multini et al. 2020). Here, we use the term “primary vectors” in accordance with the established malaria literature, referring to species or well-characterized taxa with consistently documented roles in human malaria transmission in natural settings. These include *An. darlingi*, which is widespread in the Amazon, as well as *An. albimanus*, and *An. aquasalis*, all of which play key roles in regional malaria transmission (Consoli and Oliveira 1994; Sinka et al. 2010, 2011; Kirchgatter et al. 2014; Tadei et al. 2017; Multini et al. 2020). In contrast, species such as *An. braziliensis*,* An. deaneorum*,* An. marajoara*,* An. neivai*,* An. nuneztovari* s.l., *An. oswaldoi* s.l., *An. pseudopunctipennis*, and *An. triannulatus* s.l. are generally regarded as secondary vectors, with contributions to malaria transmission that may vary across space and time according to local ecological and epidemiological conditions (Carvajal et al. 1989; Consoli and Oliveira 1994; Sinka et al. 2010; Laporta et al. 2015). Most South American *Anopheles* species, however, do not have confirmed vector status. Importantly, this should not be interpreted as evidence of absent vector competence, but rather as reflecting limited, context-dependent, or still incomplete epidemiological evidence across much of the region.

Epidemiological categories describe the documented relevance of mosquito taxa to malaria transmission. However, these taxa can also be understood as components of ecological assemblages structured by species-specific responses to environmental gradients and biotic interactions (Gilbert et al. 2008; Trivellone et al. 2022). Illustrative examples from the Peruvian and Brazilian Amazon show that larval occurrence and community composition can shift along gradients of deforestation, human presence, shade, and water conditions, consistent with fine-scale niche differentiation within local communities (Vittor et al. 2009). In the northern Brazilian Amazon, larval surveys across fish-farming tanks, ponds, and streams reveal multi-species assemblages in which dominant taxa differ in their associations with shade and water conditions, consistent with niche partitioning within local communities (Barbosa and Scarpassa 2023). More broadly, South American mosquito assemblages may also reflect resource partitioning and context-dependent, asymmetrical competition, indicating that vector communities are structured by multiple ecological processes beyond epidemiological classification alone (Laporta and Sallum 2014). This ecological perspective provides the basis for interpreting modeled differences among taxa not only in terms of vector status, but also in terms of environmentally structured overlap and potential community reorganization under climate change.

Climate change can alter malaria transmission by reshaping mosquito habitats and life cycles. Because elevation is effectively stable over the approximately centennial timescale, it may serve as a topographic proxy capturing broad physiographic contrasts across South America, particularly between the Andes and the lower Amazon Basin, whereas temperature- and precipitation-related variables capture the temporal component of change (Stanton et al. 2012; Lawler et al. 2015). Under this framework, and assuming niche conservatism over the modelled time horizon, each species occupies a particular environmental niche, and projected range shifts reflect temporal changes in where suitable environmental conditions occur, rather than rapid change in the niche itself (Colwell and Rangel 2009; Wiens et al. 2009). In South America, *An*. *darlingi* is the primary vector and remains central to transmission across much of the continent (Tadei et al. 2017; Hertig 2019). Projections for 2070 suggest reductions in its climatic suitability, interpreted mainly as a response to decreased precipitation and water availability, indicating low tolerance for drier environments (Laporta et al. 2015).

Climate-driven changes in environmental conditions may reorganize vector communities under future scenarios. Classifications such as “primary” and “secondary” vectors are therefore not absolute, and other taxa, including *An. aquasalis* and members of the *An. albitarsis* s.l. complex, may gain epidemiological importance under environmental change (Laporta et al. 2015). Evidence from other tropical regions likewise suggests that mosquito species can expand their ranges or increase in local importance when climatic conditions become favourable, as reported for *An. gambiae* under high-emission scenarios in Africa (Akpan et al. 2019). Although such examples are not directly transferable to South America because vector assemblages differ, they illustrate a broader principle across tropical systems: warming and altered rainfall regimes can shift the suitability, distribution, and relative importance of vectors in transmission. These uncertainties underscore the need for integrative, regionally grounded approaches based on the taxa and environmental conditions of the focal region.

At the same time, human-induced environmental changes, including deforestation, hydroelectric development, land-use change, and urbanisation, create new mosquito larval habitats and increase opportunities for vector–human contact (Tadei et al. 2017; Hertig 2019; Ferreira et al. 2021). Combined with climatic variation, these processes may intensify malaria transmission where suitable habitats and vector populations overlap spatially. However, quantitative assessments of how these interacting drivers shape future vector distributions in South America also remain limited (Laporta et al. 2015).

Species distribution models (SDMs) provide a robust framework for predicting potential ranges under current and future environmental scenarios. In line with Hutchinson’s niche–biotope duality, they link environmental and geographic space through reciprocal projections based on the same environmental dimensions (Colwell and Rangel 2009). By integrating bioclimatic and topographic predictors, SDMs identify major drivers of vector distributions, estimate climatic suitability, and forecast range shifts (Peterson et al. 2011; Peterson and Soberón 2012; Zhu et al. 2013). They also enable the assessment of overlap in both geographic and environmental space. Similar approaches have been used for South American hygropetric insect lineages to compare occupied environmental space and quantify niche segregation among taxa (Alencar et al. 2025a).

This study aims to assess how climate change may influence the suitability of habitats and the distributions of species richness for major *Anopheles* vector species across South America. Specifically, our objectives are: (1) to develop SDMs for 15 broadly distributed *Anopheles* vectors using contemporary occurrence data and environmental variables; (2) to quantify and compare niche breadth and overlap among species classified according to their malaria vector status, distinguishing between Primary (epidemiologically dominant) and Secondary (locally relevant or emerging) vectors; and (3) to project shifts in species distributions under moderate (SSP2-4.5) and high-emission (SSP5-8.5) climate scenarios, assessing species-specific patterns of range change, including expansion, contraction, fragmentation, and persistence throughout the 21 st century. We hypothesise that climate change will favour the expansion of non-primary *Anopheles* species, increasing their potential contribution to malaria transmission across South America. Conversely, climate-sensitive taxa such as *An. darlingi* (Primary vector) are likely to experience range contractions, particularly in areas affected by intensified water scarcity. These contrasting responses may reshape vector community composition, with reduced transmission where *An. darlingi* currently predominates, but elevated risk in regions such as the Amazon, where climatic shifts could foster niche replacement and the persistence of multiple vector species.

## Materials and methods

### Occurrence records and data cleaning

Occurrence records for *Anopheles* were compiled from peer-reviewed literature (taxonomic revisions and checklists) and the Global Biodiversity Information Facility (GBIF, 2025); download: 10.15468/dl.zfaw79). The original GBIF download contained 578,292 raw occurrences. We first restricted the dataset to the focal South American taxa, yielding approximately 25,800 candidate records, of which about 15,200 corresponded to species-level records with valid geographic coordinates. To account for taxonomic changes through time and reduce the influence of misidentification, we performed taxonomic harmonization by reconciling synonyms and historical names against an updated taxonomic backbone and by removing records that could not be confidently assigned at the species level(Harbach 2018). We then applied quality-control procedures to remove problematic coordinates and collapse duplicate occurrences, reducing the dataset to approximately 3,940 unique species–coordinate records. To reduce spatial sampling bias, occurrences were spatially thinned using the CELLSIZE approach, retaining one record per ~ 9 km grid cell (Fourcade et al. 2014; Velazco et al. 2019). After harmonization, filtering, coordinate cleaning, duplicate removal, and thinning, the final dataset comprised 2,726 spatially independent occurrences.

**Fig. 1:**
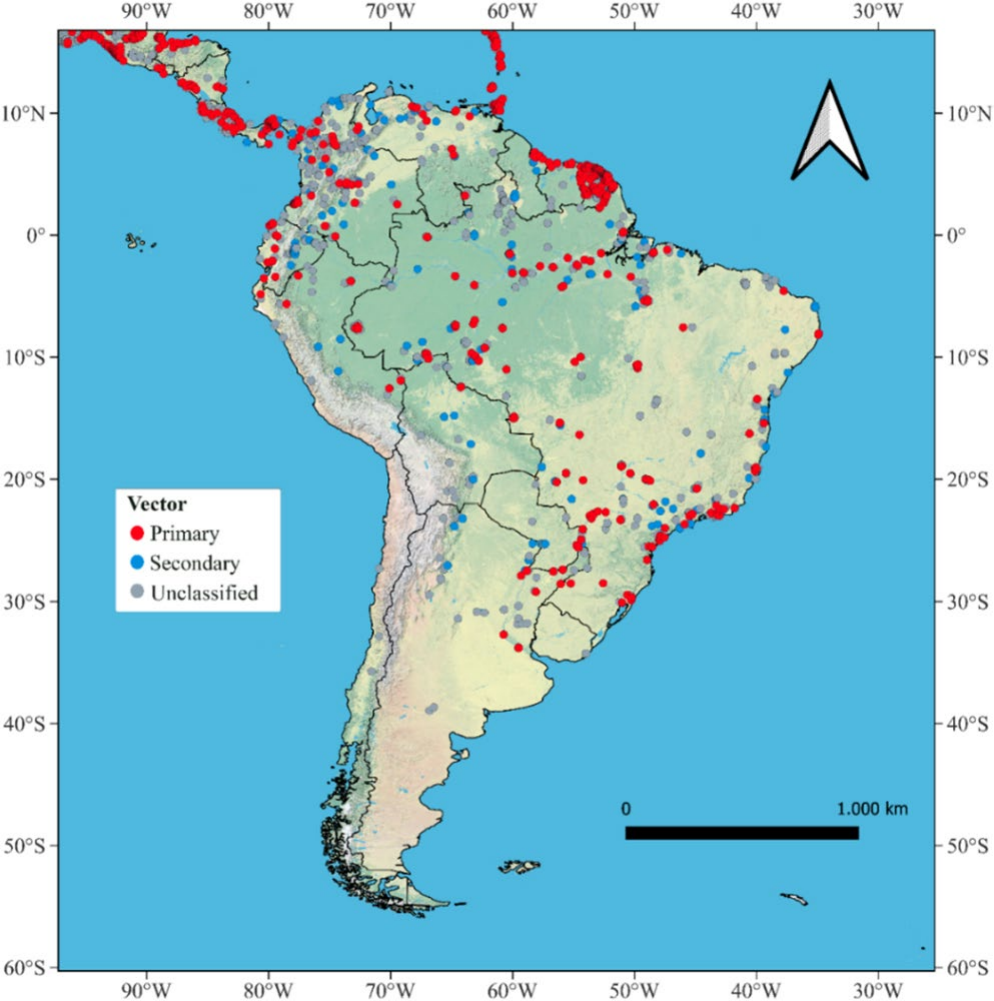
Spatial distribution of occurrence records for *Anopheles* species across South America. Points indicate the locations of primary malaria vectors (red), secondary vectors (blue), and unclassified species (grey)

Several taxa treated here represent sensu lato species groups; therefore, projections for these groups reflect pooled climatic envelopes of closely related lineages rather than the distribution of any single biological species. This may smooth lineage-specific ecological differences and mask finer epidemiological contrasts among cryptic taxa, including variation in habitat associations or vector importance. To ensure consistent nomenclature throughout the manuscript, we followed Harbach (2018) and treated *Anopheles* as a single genus with subgenera. For ecological niche modelling, we included only South American species with ≥ 20 spatially independent records (Wisz et al. 2008; van Proosdij et al. 2016; Sampaio and Cavalcante 2023), yielding a final dataset of 15 species. Figure 1 summarises the geographic distribution of occurrences, and Supplementary Files S1 and S2 provide detailed source information and vector-status classification.

A total of 20 environmental variables were selected as predictors for modelling *Anopheles* distributions: 19 BIOCLIM bioclimatic variables (Booth et al. 2014) obtained from the WorldClim database (https://www.worldclim.org/) and elevation (Fick and Hijmans 2017). Climatic variables included temperature- and precipitation-based metrics representing annual trends, seasonal variation, and extremes. Elevation was included for its influence on precipitation, vegetation, and habitat conditions affecting insect biology (Beirne 1970; Hodkinson 2005; Régnière et al. 2012; Khaliq et al. 2014). Predictors were standardized to a 2.5 arc-minute resolution. Multicollinearity was reduced using a variance inflation factor threshold (VIF > 10; Marquardt 1970), and 10 predictors were retained for the final model set: mean diurnal range (BIO2), isothermality (BIO3), mean temperature of the wettest quarter (BIO8), mean temperature of the driest quarter (BIO9), precipitation of the wettest month (BIO13), precipitation of the driest month (BIO14), precipitation seasonality (BIO15), precipitation of the warmest quarter (BIO18), precipitation of the coldest quarter (BIO19), and elevation (see Supplementary File S3).

Future distributions were projected using climate projections from the MIROC6 general circulation model (CMIP6), selected because it was among the better-performing CMIP6 models in an evaluation of large-scale atmospheric circulation biases across continental regions, including South America (Cannon 2020). We considered two Shared Socioeconomic Pathways, SSP2-4.5 and SSP5-8.5, to represent intermediate and very high scenarios, respectively, thereby capturing a broader range of potential future climatic responses. Consistent with IPCC AR6, global mean temperature averaged over 2081–2100 is very likely to be 2.1–3.5 °C higher under SSP2-4.5 and 3.3–5.7 °C higher under SSP5-8.5 relative to 1850–1900 (IPCC 2021).

## Ecological niche models

We modelled current and future climatic suitability for *Anopheles* using four algorithms spanning complementary modelling assumptions: Maximum Entropy (default settings; MXD), which contrasts presences against background conditions (Phillips et al. 2006; Phillips 2017); DOMAIN (DOM), a presence-only method based on environmental similarity (Hijmans et al. 2017); Random Forest (RDF) (Liaw and Wiener 2002); and Generalized Linear Models (GLM) (McCullagh and Nelder 1989), both fitted with presences and pseudo-absences. Using multiple algorithms reduced reliance on any single set of assumptions and improved robustness to method-specific biases (Pimenta et al. 2022).

Species-specific calibration areas (“M”) were delineated by buffering occurrence records using the maximum inter-point geographic distance for each species, following the accessible-area rationale (Barve et al. 2011; Peterson et al. 2011). Within each calibration area, pseudo-absences were generated for algorithms requiring absence information (GLM and RDF) using a 1:1 ratio relative to presence records, whereas background points for MXD were sampled from the same calibration region. To reduce the influence of sampling bias, pseudo-absences were restricted to cells with lower suitability predicted by an initial BIOCLIM model fitted with the same predictor set (Engler et al. 2004). Ensemble predictions were generated by averaging only the best-performing models (TSS ≥ 0.85), and binary maps were derived using the threshold that maximized the Sørensen similarity index (Allouche et al. 2006; Araújo et al. 2007; Thuiller et al. 2019; Velazco et al. 2019).

Models were assessed using five-fold cross-validation, with occurrences partitioned into five subsets and each subset used once for evaluation while the remaining subsets were used for calibration (Fielding and Bell 1997). Predictive performance was summarized using the True Skill Statistic (TSS) and the Sørensen index, with values ≥ 0.8 interpreted as strong model (Jiménez-Valverde and Lobo 2007; Leroy et al. 2018). Spatial autocorrelation in evaluation outputs were quantified using Moran’s I, reported as mean ± SD across cross-validation replicates; values ranged from 0.067 to 0.497, indicating generally low to moderate spatial structure. Model transferability was further assessed with the Multivariate Environmental Similarity Surface (MESS; see Supplementary File S4) and Mobility-Oriented Parity (MOP), which identify low environmental similarity and novel climatic conditions in projection areas, respectively (Owens et al. 2013). Mean MESS values ranged from 6.916 ± 0.610 to −28.426 ± 52.561, indicating species-specific extrapolation risk, whereas MOP showed high environmental similarity across most projected areas, with > 98% of cells showing similarity > 0.9 to the calibration domain.

We used binarized ensemble models to estimate potential *Anopheles* species richness and spatial overlap across South America under present and future climates. Species-richness values for each scenario were obtained by stacking binary suitability projections across all modelled species. To summarize continental-scale redistribution in a simple and reproducible way, South America was divided into four broad geographic sectors—NW, NE, SW, and SE (Supplementary Fig. S1). This subdivision was adopted as a descriptive spatial framework only, rather than as a biogeographic or ecological regionalization, and therefore was not used to mask, constrain, or redefine the underlying model predictions. Within each sector, we summarized occupied area (km²), its proportional contribution to the total occupied area of each scenario (%), and descriptive richness metrics (mean, median, and maximum richness). This approach enabled direct comparison of large-scale shifts in richness and overlap among scenarios, whereas the ecological interpretation of these patterns was based on the distribution of major South American biomes within and across sectors, as discussed later. Niche models were built in R using *ENMTML* (Andrade et al. 2020), and final maps were prepared in QGIS version 3.34.12–Prizren.

## Environmental space analysis and niche overlap among vector classes

We assessed differences in niche occupation among vector classes (Primary, Secondary, and Unclassified) using the same 20 environmental predictors applied in the ENM, 19 bioclimatic variables from WorldClim and elevation, standardized at a 2.5 arc-min resolution. To reduce collinearity and summarize the main gradients of environmental variation, we performed a principal component analysis (PCA) and retained the first seven axes, which accounted for > 95% of the total variation (De Marco and Nóbrega 2018). For each vector class, species occurrences were projected into PCA space, and their occupied area was delineated using convex hull polygons as an estimate of realized environmental space. No additional outlier exclusion was applied at this stage. Therefore, convex hulls represent the full extent of the filtered occurrences in environmental space (Cornwell et al. 2006).

Niche overlap between classes was quantified using two complementary indices: Schoener’s D and Warren’s I (Schoener 1970; Warren et al. 2008), both widely used in ecological niche modelling to assess similarity in occupied environmental space. Both metrics range from 0 to 1, where values close to 0 indicate low overlap and values close to 1 indicate highly similar niche distributions. Schoener’s D is based on absolute differences in predicted probabilities across grid cells, whereas Warren’s I is derived from the Hellinger distance and emphasizes overall distributional similarity (Broennimann et al. 2012). Differences in standardized diversity (B*; sensu Colwell and Coddington 1994) and convex hull area (as a proxy for the extent of occupied PCA space) were evaluated by calculating group-wise means and testing for significance using Welch’s t-tests (Ruxton 2006). B* provides a standardized measure of how evenly species occurrences are distributed across environmental space, with values closer to zero indicating restricted or clustered occupation and values closer to one reflecting broader and more heterogeneous use of conditions. Convex hull area, in turn, captures the absolute extent of the occupied space. Together, these complementary metrics allow us to assess both the breadth of environmental space occupied and the internal structure of diversity within groups.

To visualize differences in environmental space occupation among vector classes, we plotted occurrences on the first two PCA axes and applied a 2D kernel density estimation for each class. The density surfaces were filled by class, with darker shades indicating areas of overlap between classes. This approach highlights both the extent of occupied space and regions of environmental similarity. All analyses were conducted in R version 4.4.3 (R Core Team 2025) using the packages *terra* and *ecospat* (Di Cola et al. 2017; Hijmans 2025).

## Results

We compiled occurrence data for 87 *Anopheles* species across South America, but modelled only the 15 that met the eligibility threshold of ≥ 20 unique records. Of these, four were classified as primary vectors, eight as secondary vectors, and three as Unclassified because the available literature did not provide sufficient epidemiological evidence for formal vector assignment. This category should be interpreted cautiously, as it reflects uncertainty in current knowledge rather than a shared epidemiological role. The number of unique records used for model calibration ranged from 20 for *An. deaneorum* to 823 for *An. pseudopunctipennis*, with nine species represented by more than 100 records and only three by fewer than 30 (Table 1).

**Table 1:** Performance of ensemble distribution models for 15 *Anopheles* species, evaluated by True Skill Statistic (TSS), and Sørensen similarity index. Values represent mean ± standard deviation

Species	Vector	*n*	TSS	Sørensen
*Anopheles albimanus*	Primary	576	0.898 ± 0.011	0.949 ± 0.006
*Anopheles albitarsis s.l.*	Primary	115	0.887 ± 0.050	0.945 ± 0.024
*Anopheles aquasalis*	Primary	175	0.949 ± 0.037	0.975 ± 0.018
*Anopheles argyritarsis*	Unclassified	110	0.918 ± 0.038	0.959 ± 0.019
*Anopheles braziliensis*	Secondary	98	0.888 ± 0.043	0.942 ± 0.025
*Anopheles darlingi*	Primary	190	0.911 ± 0.071	0.952 ± 0.041
*Anopheles deaneorum*	Secondary	20	0.900 ± 0.137	0.949 ± 0.070
*Anopheles intermedius*	Unclassified	37	0.921 ± 0.072	0.961 ± 0.036
*Anopheles janconnae*	Unclassified	26	0.960 ± 0.089	0.982 ± 0.041
*Anopheles marajoara*	Secondary	27	0.847 ± 0.166	0.93 ± 0.0710
*Anopheles neivai*	Secondary	74	0.919 ± 0.029	0.959 ± 0.017
*Anopheles nuneztovari* s.l	Secondary	113	0.876 ± 0.065	0.935 ± 0.037
*Anopheles oswaldoi* s.l	Secondary	169	0.905 ± 0.013	0.953 ± 0.007
*Anopheles pseudopunctipennis*	Secondary	823	0.915 ± 0.006	0.957 ± 0.003
*Anopheles triannulatus* s.l	Secondary	173	0.913 ± 0.036	0.956 ± 0.019

Overall, the ensemble distribution models achieved very high predictive performance for all 15 species analyzed. TSS values ranged from 0.85 for *An. marajoara* to 0.96 for *An. janconnae*, reflecting strong discrimination between suitable and unsuitable habitats. Similarly, Sørensen similarity indices ranged from 0.93 to 0.98, indicating a high degree of congruence between predicted and observed species distributions. Model sensitivity (True Positive Rate, TPR) was optimal for all species, consistently at 0.99, indicating high model capacity to identify true presences. Specificity (True Negative Rate, TNR) also approached precision, averaging 0.99. Despite these high values, caution is warranted for sparsely sampled taxa, particularly *An. deaneorum* (*n* = 20), *An. janconnae* (*n* = 26), *An. marajoara* (*n* = 27), and *An. intermedius* (*n* = 37), because high discrimination metrics do not by themselves exclude overfitting under limited sampling.

## Environmental space partitioning across vector classes

**Fig. 2:**
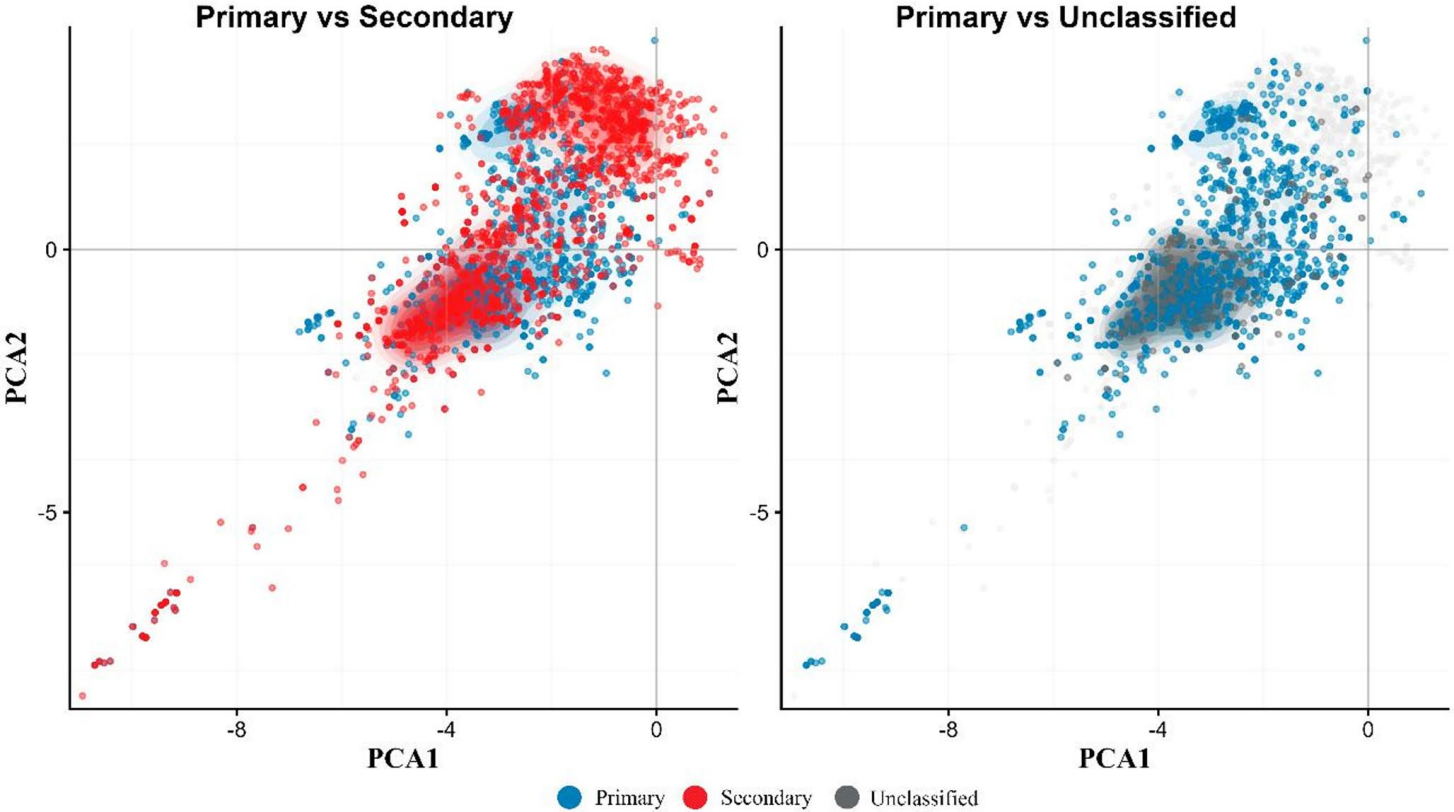
Environmental niche space of vector classes. Left panel: comparison between *Primary* (blue) and *Secondary* (red) vectors. Right panel: comparison between *Primary* (blue) and *Unclassified* (dark gray) vectors

The comparison between primary and secondary classes revealed moderate overlap in environmental space (Schoener’s D = 0.570; Hellinger’s I = 0.773), based on 1,605 and 2,022 occurrence records, respectively (Fig. 2). Here, D and I quantify overlap between the class-level density/hypervolume estimated in PCA environmental space. Standardized diversity (B*) showed similar means between groups (0.490 in primary vs. 0.567 in secondary species), with no statistically significant differences (Welch’s t-test: t = − 0.701, df = 5.79, *p* = 0.511). Likewise, the convex hull area in PCA space did not differ between groups (Welch’s t-test: t = 0.509, df = 4.39, *p* = 0.635). In the comparison between primary and unclassified classes (1,605 vs. 236 records), overlap was lower (D = 0.453; I = 0.672). Because the Unclassified group had fewer records, these estimates should be interpreted cautiously, as a smaller sample size may affect the stability of environmental overlap and occupied-space metrics. Mean B* values were 0.490 for primary and 0.680 for unclassified species, but differences were not significant (Welch’s t-test: t = − 0.997, df = 3.19, *p* = 0.388). Similarly, convex hull area did not differ between groups (Welch’s t-test: t = 1.319, df = 4.84, *p* = 0.246) (Fig. 2; Supplementary S5).

**Fig. 3:**
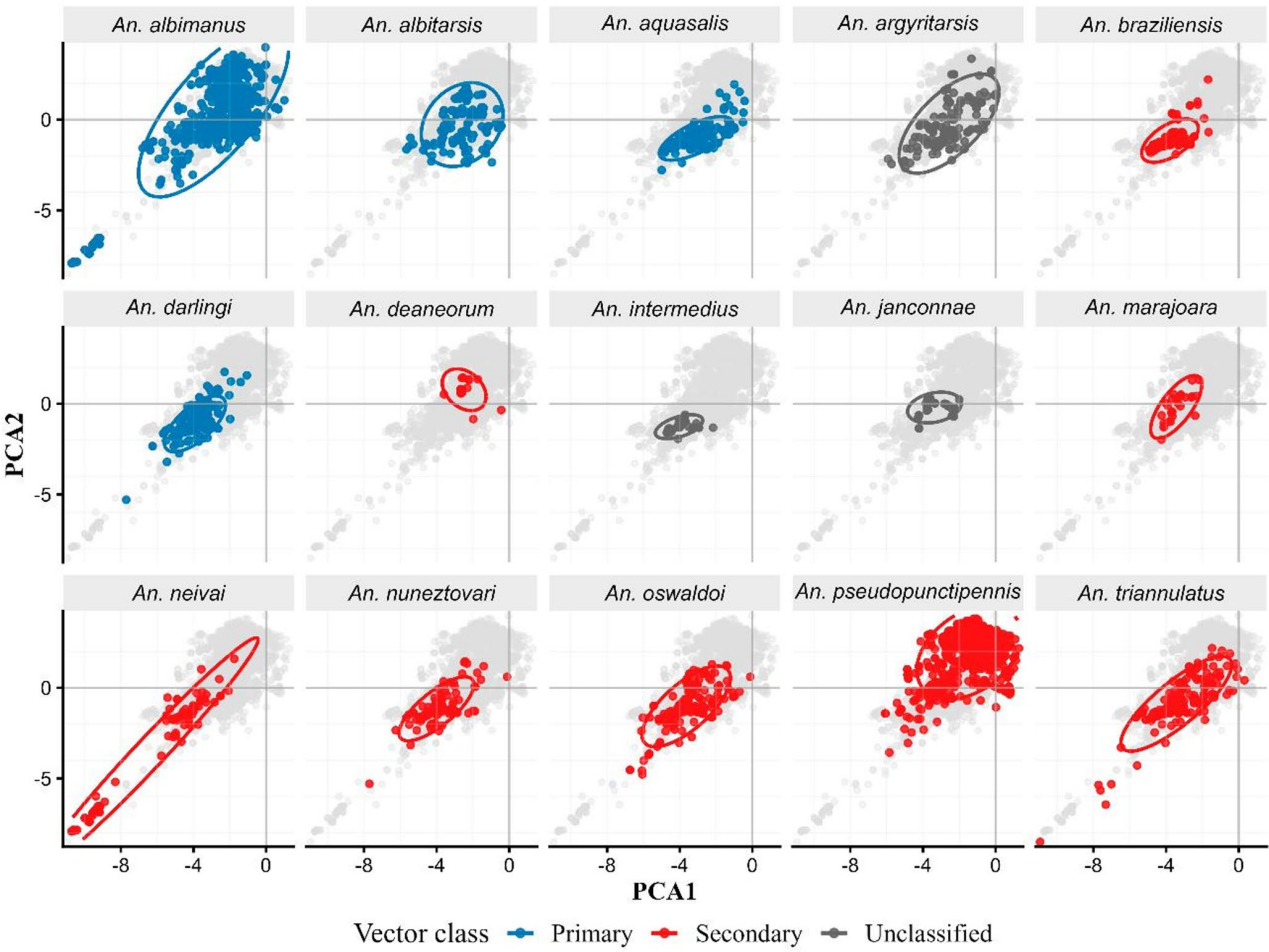
Niche space occupancy of *Anopheles* species across vector classes. Scatterplots represent species occurrences projected onto the first two PCA axes. Each panel shows the distribution of one species (colored points) against the background of all other species (gray points) in the niche space. Colors represent vector classes: red = Primary vectors, blue = Secondary vectors, dark gray = Unclassified species

At the species level, most taxa exhibited distinguishable 95% confidence ellipses in PCA space, although overlap varied widely among species pairs. Some taxa showed more concentrated ellipses, such as *An. darlingi* and *An. nuneztovari* s.l., indicating lower environmental dispersion in their occurrences, whereas *An. albimanus* (primary), *An. pseudopunctipennis* (secondary), *An. triannulatus* s.l. (secondary), and *An. argyritarsis* (unclassified) displayed more diffuse niches (Fig. 3). Despite these differences, several taxa shared portions of climatic space, indicating partial affinity in the environmental conditions they occupy (Fig. 3; Supplementary S6 and S7). Because these species-level patterns are primarily descriptive and the total number of modelled species was small, the statistical power to detect sampling-related effects was limited. Accordingly, the standardized niche-breadth metric B* showed no detected association with the number of occurrence records (Pearson *r* = − 0.19, *p* = 0.504; Spearman ρ = −0.35, *p* = 0.206), and this result should be interpreted cautiously.

### Current and future potential distribution across South America

Projections under two greenhouse gas emission scenarios, the intermediate SSP2-4.5 and the high-end SSP5-8.5, for two future periods (2021–2040 and 2081–2100), revealed substantial spatial shifts in the potential climatic suitability of *Anopheles* species across South America (see Supplementary Table S8 for species-level summaries of projected range change by scenario). These projections indicated marked species-specific ecological responses to future climate conditions. At the same time, broad-scale richness patterns remained unevenly distributed across the continent under both present and future scenarios (Table 2, see Supplementary Figure S2). Under present conditions, the NE had the largest occupied area and the highest overall richness, whereas the SW consistently represented the least diverse portion of the continent. This spatial asymmetry indicates that current multi-species suitability is concentrated primarily in northern and northeastern South America, while southern and southwestern regions support comparatively fewer overlapping suitable areas.

**Table 2:** Spatial distribution of predicted *Anopheles* species richness across South American quadrants under present and future climate scenarios. Values summarize the spatial distribution of species-richness maps generated by stacking binary suitability maps for all modelled species under present and future scenarios. South America was divided into four quadrants: NW (northwestern South America), NE (northeastern South America), SW (southwestern South America), and SE (southeastern South America)

Scenario	Quadrant	Area (km²)	%	Mean richness	Median richness	Max richness
Present	NW	4,502,319	28%	4.08	4	14
Present	NE	7,429,777	46%	5.05	4	14
Present	SW	1,978,455	12%	1.17	1	10
Present	SE	2,242,882	14%	2.78	2	14
Scenario total occupied area		16,153,433	100%			
SSP2-4.5, 2021–2040	NW	4,622,952	28%	4.23	4	14
SSP2-4.5, 2021–2040	NE	7,462,604	45%	4.87	4	14
SSP2-4.5, 2021–2040	SW	2,134,423	13%	1.41	1	10
SSP2-4.5, 2021–2040	SE	2,258,760	14%	4.03	4	13
Scenario total occupied area		16,478,739	100%			
SSP2-4.5, 2081–2100	NW	4,865,927	29%	4.22	4	14
SSP2-4.5, 2081–2100	NE	7,469,392	44%	4.73	4	14
SSP2-4.5, 2081–2100	SW	2,241,107	13%	1.92	2	10
SSP2-4.5, 2081–2100	SE	2,264,743	13%	6.20	6	13
Scenario total occupied area		16,841,170	100%			
SSP5-8.5, 2021–2040	NW	4,623,886	28%	4.18	4	14
SSP5-8.5, 2021–2040	NE	7,462,605	45%	4.64	4	14
SSP5-8.5, 2021–2040	SW	2,135,834	13%	1.41	1	10
SSP5-8.5, 2021–2040	SE	2,257,067	14%	4.06	4	13
Scenario total occupied area		16,479,392	100%			
SSP5-8.5, 2081–2100	NW	4,937,267	29%	3.13	3	13
SSP5-8.5, 2081–2100	NE	7,428,612	44%	3.64	3	13
SSP5-8.5, 2081–2100	SW	2,283,322	13%	1.97	2	10
SSP5-8.5, 2081–2100	SE	2,265,670	13%	6.63	7	13
Scenario total occupied area		16,914,871	100%			

Future projections showed that these richness patterns were not spatially static. Although total occupied area changed only moderately among scenarios, the internal distribution of richness shifted substantially. In particular, the SE showed the strongest increase in richness under future climates, especially in the end-of-century projections, whereas the NE and NW lost relative dominance under the most severe scenario. Together, these results indicate a spatial reorganization of multi-species suitability across South America, with climate change altering not only the extent of suitable area but also the geographic concentration of richness and overlap.

## Primary vectors

**Fig. 4:**
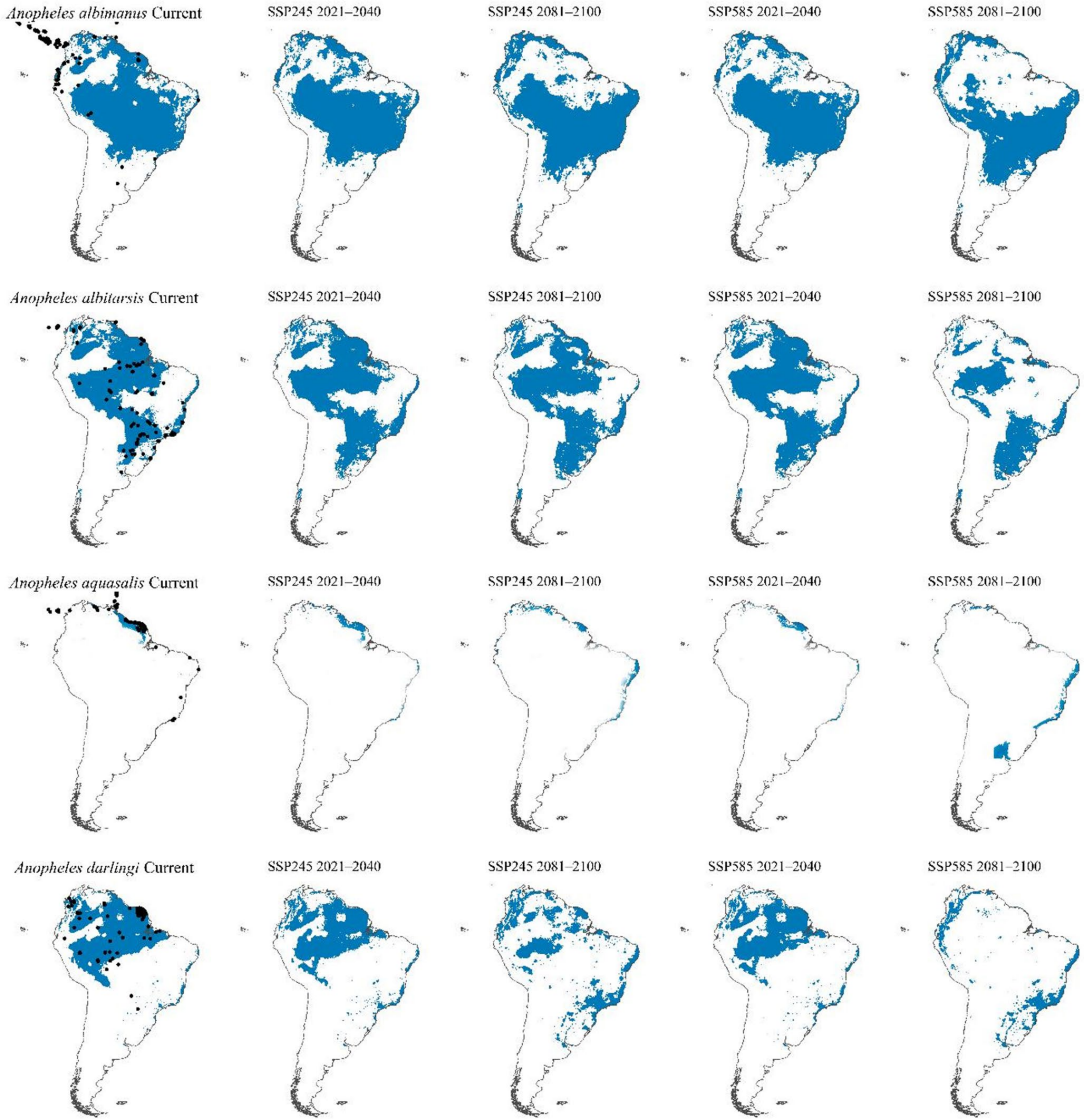
Spatial projections of climatic suitability for primary *Anopheles* species across South America under current and future climate scenarios. Each row shows one species in alphabetical order. Columns show the current potential distribution, then projections under SSP245 (stabilization) and SSP585 (high-end emissions) for the near future (2021–2040) and late century (2081–2100). Maps are binary ecological niche models: blue = predicted presence, white = unsuitable. Black dots are occurrence records and appear only on the current map

Primary malaria vectors are projected to undergo pronounced changes in climatic suitability. *Anopheles albimanus*, which currently maintains a relatively stable distribution, shows a clear southward expansion by 2081–2100 under SSP5-8.5 (Fig. 4). *An. albitarsis s.l.* is expected to contract under both scenarios, with the extreme SSP5-8.5 leading to severe retractions along the Atlantic coast, leaving two isolated regions: southern Amazonia and a southern corridor spanning Brazil, Paraguay, Uruguay, and Argentina (Fig. 4). *An. aquasalis* reveals a latitudinal shift from its Amazonian range toward the Atlantic coast, where future suitable habitats consolidate into a longitudinal band extending from northern South America to northern Argentina under SSP5-8.5 (Fig. 4).

*Anopheles darlingi*, the most important malaria vector in South America, is projected to experience substantial habitat reductions under both emission scenarios (Fig. 4). By 2081–2100, SSP5-8.5 predicts near-complete loss of Amazonian suitability, leaving only small fragments along northern coastal areas and scattered inland patches. These reductions contrast with moderate fragmentation projected under SSP2-4.5, but in all cases, habitat contraction is severe.

## Secondary vectors

**Fig. 5:**
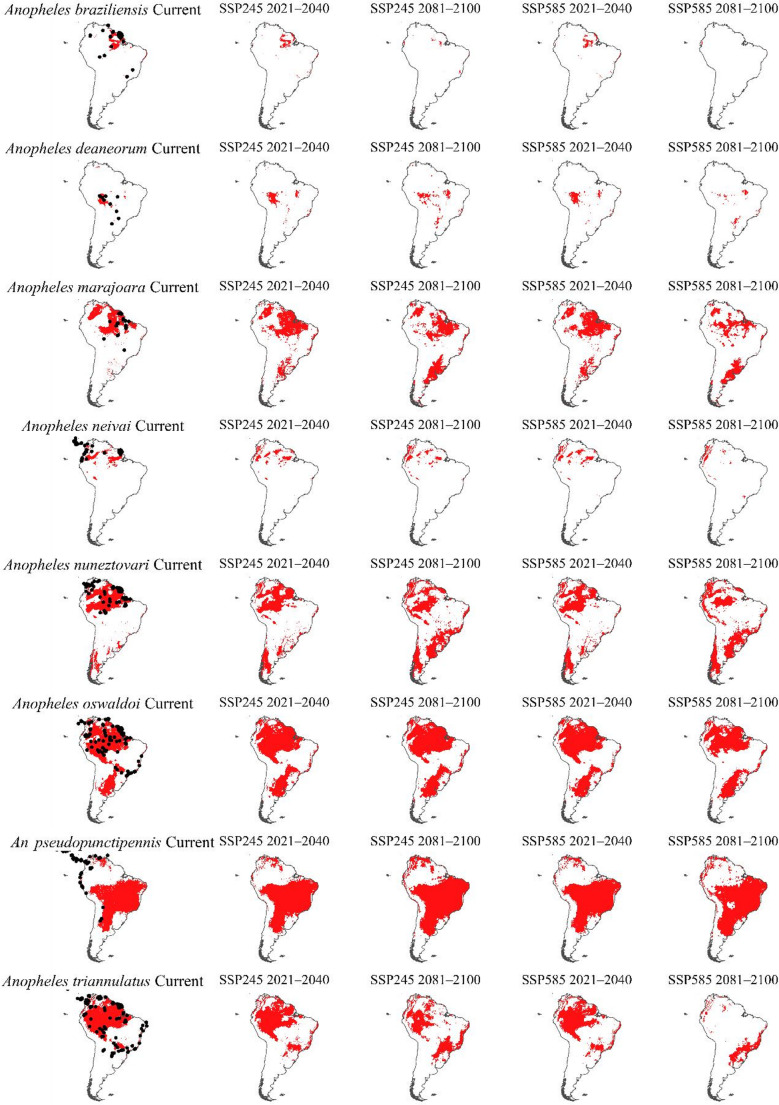
Spatial projections of climatic suitability for secondary *Anopheles* species across South America under current and future climate scenarios. Each row shows one species in alphabetical order. Columns show the current potential distribution, then projections under SSP245 (stabilization) and SSP585 (high-end emissions) for the near future (2021–2040) and late century (2081–2100). Maps are binary ecological niche models: red = predicted presence, white = unsuitable. Black dots are occurrence records and appear only on the current map

Among secondary vectors, heterogeneous responses emerge. *An. braziliensis* maintains relative stability through 2040, but severe reductions occur by 2100, leaving only small, isolated patches along Pacific and Atlantic coasts under SSP5-8.5 (Fig. 4). *An. deaneorum* and *An. intermedius* show moderate changes, with slight expansions under SSP2-4.5 but reductions under SSP5-8.5 by the century’s end (Fig. 5). In contrast, *An. marajoara* shows clear expansion of suitable habitats, predominantly southward under SSP5-8.5 (Fig. 5).

**Fig. 6:**
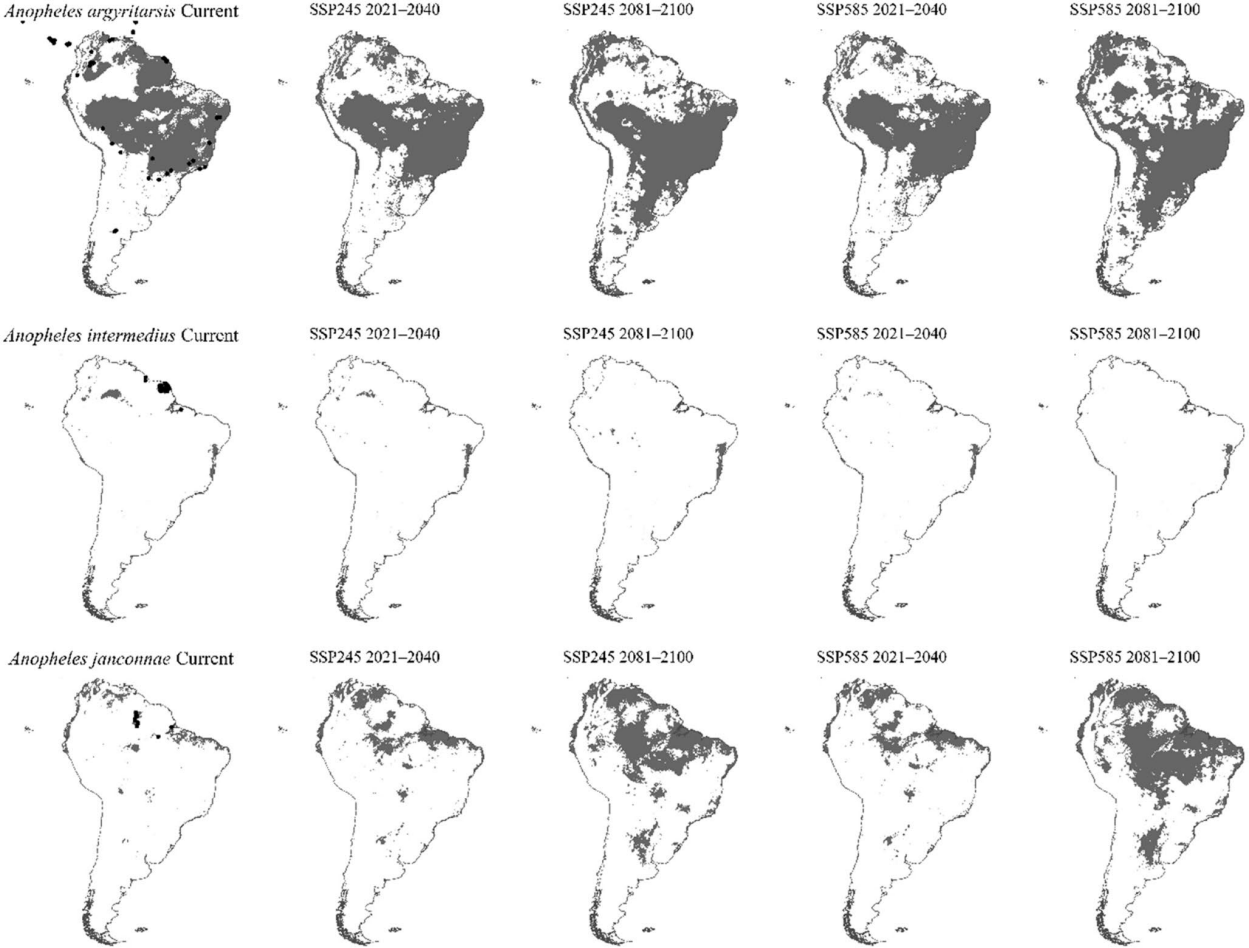
Spatial projections of climatic suitability for unclassified *Anopheles* species across South America under current and future climate scenarios. Each row shows one species in alphabetical order. Columns show the current potential distribution, then projections under SSP245 (stabilization) and SSP585 (high-end emissions) for the near future (2021–2040) and late century (2081–2100). Maps are binary ecological niche models: gray = predicted presence, white = unsuitable. Black dots are occurrence records and appear only on the current map

Projections for *An. neivai* indicate consistent northward contraction, culminating in severe reductions by 2081–2100 under SSP5-8.5, leaving only small patches along the northern Pacific coast. *An. nuneztovari s.l.* exhibits fragmentation between northern and central Amazonia, with Atlantic coast expansions forming a continuous north–south corridor. Additional expansions are projected in southern South America under both scenarios. *An. oswaldoi s.l.* remains relatively stable, with two main habitat patches: northern Amazonia and southern South America. *An. pseudopunctipennis* also shows stability, with only minor reductions in northern regions and small central gaps (Fig. 6). Conversely, *An. triannulatus s.l.* suffers severe losses under both scenarios, with future suitability restricted to the southeastern Atlantic coast and fragmented Andean corridors (Fig. 5).

### Unclassified vectors

Unclassified species exhibit a mix of expansions and moderate reductions. *An. argyritarsis* shows significant expansion under SSP5-8.5, with increasingly fragmented distributions around the Amazon and substantial extensions into southern Brazil, Paraguay, Uruguay, and Argentina by 2100. *An. janconnae* demonstrates future expansions, predominantly southward under SSP5-8.5 (Fig. 6).

## Discussion

Our study provides continent-wide projections of potential present and future climatic suitability for epidemiologically relevant *Anopheles* vector species (and species complexes) in South America. By combining ensemble distribution models with analyses in environmental space, we show that taxa differ markedly in their climatic associations and projected responses to climate change. Contractions and shifts in suitability for several vectors under the high-end scenario, coupled with expansions of others, indicate substantial reorganization in the composition of local vector communities. At the community level, present richness was concentrated mainly in the NE, whereas the SW consistently showed the lowest richness; under future scenarios, the SE exhibited the strongest increase in richness, especially toward the end of the century, while the NE and NW lost relative dominance under the most severe scenario. Together, these results suggest that malaria risk is unlikely to be shaped by the persistence of individual species alone, but rather by the dynamic restructuring of *Anopheles* assemblages. This community-level perspective complements the traditional Primary–Secondary framing by emphasizing turnover, changing overlap, and the potential reshuffling of multi-vector communities under climate change.

### Environmental space partitioning

Environmental space partitioning indicated moderate overlap between primary and secondary vector classes and lower overlap between primary and unclassified taxa (Fig. 2). However, standardized diversity (B*) and convex hull area did not differ detectably among epidemiological classes, which is expected because vector status is an epidemiological attribute related to transmission competence rather than an ecological trait that determines habitat use. Likewise, Schoener’s* D and Warren’s I describe similarity in occupied climatic space, but do not by themselves demonstrate competition or other biotic interactions among taxa. Instead, ecological breadth and differentiation emerged mainly at the species level, with most taxa occupying distinct portions of environmental space. For example, *An. darlingi* and *An. nuneztovari* s.l. showed relatively concentrated ellipses, consistent with constrained climatic associations, whereas *An. albimanus*, *An. pseudopunctipennis*, and *An. triannulatus* s.l. occupied broader and more diffuse ranges in multivariate space.

These patterns align with metacommunity studies of Culicidae showing that landscape composition and environmental turnover structure mosquito assemblages and drive community shifts across habitats, largely independent of their role as disease vectors (Gilbert et al. 2008; Zittra et al. 2016; Trivellone et al. 2022). Although coarse-resolution predictors can smooth fine-scale heterogeneity and overlook microhabitats critical for breeding or survival (Dormann et al. 2018), the analyses still capture major climatic and physiographic gradients structuring vector distributions at regional scales. Taken together, the evidence suggests that ecological differentiation operates primarily at the species level and that epidemiological classification alone does not capture these ecological patterns. Importantly, overlap metrics derived from suitability surfaces provide a useful, first-order proxy for shared climatic space and potential co-occurrence, but they do not by themselves demonstrate mechanistic niche partitioning or competitive interactions, which require independent ecological and behavioral evidence.

### Species-specific distributional shifts

Future projections revealed divergent ecological trajectories among primary South American malaria vectors. Expansive species such as *An. albimanus* and *An. aquasalis* are predicted to shift southward, especially under SSP5-8.5 by 2100, taking advantage of newly suitable areas. By contrast, vectors with narrower climatic tolerances, such as *An. darlingi* is expected to undergo severe contractions and fragmentation, particularly in the Amazon Basin, where they are currently major malaria vectors. These findings are consistent with Laporta et al. (2015), who demonstrated that *An. darlingi* exhibits low tolerance to drier environments, leading to significant habitat loss under future climates, whereas generalist species within the *Albitarsis* complex show strong potential for broad spatial and temporal expansion. Likewise, secondary or unclassified species such as *An. janconnae*, *An. marajoara*, and *An. argyritarsis* are projected to expand considerably, in some cases occupying areas vacated by primary vectors. However, these species-specific projections remain uncertain, especially for cryptic species complexes and for taxa represented by few records, and expansion of climatically suitable areas should not be taken as direct evidence of increased malaria transmission, particularly for species with uncertain epidemiological status. Collectively, these responses underscore pronounced interspecific differences in climate sensitivity and adaptive potential. For species complexes, vector competence is often documented for only a subset of member taxa; therefore, epidemiological implications should be treated as hypotheses requiring targeted entomological validation.

Richness patterns are more interpretable when viewed at the quadrant level, because major South American biomes do not map neatly onto a single geographic sector. In particular, the NW and NE both encompass large portions of the Amazon, while the Atlantic Forest extends across both the NE and SE, and drier formations associated with Cerrado and Caatinga also occur within the broader eastern and northeastern domain. Thus, the quadrant framework captures broad spatial reorganization in climatic suitability without implying that biome boundaries coincide with quadrant boundaries. Under present conditions, the highest richness was concentrated in the NE, consistent with the extensive overlap of climatically suitable conditions across Amazonian, Atlantic, and transitional environments in this sector. By contrast, future projections indicate that the SE emerges as one of the principal centres of potential *Anopheles* richness under the high-emission SSP5-8.5 scenario by the end of the century. This shift is plausibly explained by the persistence of environmentally heterogeneous and comparatively humid conditions in southeastern South America, where Atlantic Forest and transitional landscapes may continue to provide climatically suitable space for multiple taxa, while some northern areas lose relative dominance under stronger warming and drying. In this sense, climate change appears to reorganize, rather than simply reduce, the geography of multi-species suitability, shifting the concentration of potential vector assemblages from the northern and northeastern sectors toward the southeast.

### Epidemiological implications and limitations

Despite the high evaluation metrics, caution is warranted for taxa represented by few occurrence records, because high discrimination scores do not by themselves exclude overfitting under limited sampling (van Proosdij et al. 2016). As in other correlative SDM approaches, our framework is also limited by the exclusive use of abiotic predictors, without explicitly incorporating biotic interactions, microhabitat structure, or host availability, all of which can shape vector dynamics locally (Elith and Leathwick 2009; Dormann et al. 2018). Although we adopted widely recommended practices in predictor screening, bias mitigation, and ensemble modelling (Araujo and New 2007; Fourcade et al. 2014; Norberg et al. 2019; Thuiller et al. 2019; Velazco et al. 2019), uncertainty remains, especially for poorly sampled taxa and projections into novel climates. Even so, our results are consistent with other climate-driven insect SDM studies (Alencar et al. 2022, 2024, 2025b, 2026; Cruz et al. 2023), supporting the value of SDMs for anticipating broad-scale shifts in vector suitability under global change. We also emphasise that factors related to vector competence remain insufficiently understood, particularly within species complexes, which limits their incorporation into predictive models. Therefore, further studies are required to better elucidate this biological component, which is essential for understanding the dynamics of malaria transmission.

Primary vectors, including *An. darlingi*, are projected to contract most strongly under SSP5-8.5, raising concerns about declining suitability in parts of the Amazon. In contrast, several secondary and unclassified taxa show expansion or persistence, potentially forming new corridors of suitability along the Atlantic coast and into southern South America. These patterns may influence malaria transmission: reductions in suitability for *An. darlingi*, historically a key Amazonian vector (Tadei et al. 2017; Ferreira et al. 2021; Sallum et al. 2025), could reduce transmission locally, but expansions of other taxa could offset these declines if competent vectors become locally dominant. This reinforces the need to anticipate malaria risk as an emergent property of shifting multi-vector communities under climate change. Finally, these projections represent potential climatic suitability, not realized transmission risk, which also depends on human exposure, vector abundance, land use, control measures, parasite dynamics, and species-specific competence. Even so, the present results remain valuable by identifying broad-scale spatial shifts in suitability and overlap among vector taxa, thereby providing an important basis for hypothesis testing,

## Supplementary Information

Below is the link to the electronic supplementary material.

Supplementary Material 1 (CSV 2.87 MB)

Supplementary Material 2 (PDF 15.2 MB)

## Data Availability

Data generated or analysed during this study are provided in full within the published article and its supplementary materials.
