# Supplementary material for: Future climate scenarios reshape the suitability of major malaria vector taxa in South America
Source: Int J Biometeorol. 2026 Jun 23;70(7):195. doi: 10.1007/s00484-026-03244-y (PMC13290823; doi:10.1007/s00484-026-03244-y)
Supplement: Supplementary file 2 — Supplementary Material 2 (PDF 15.2 MB) [file 484_2026_3244_MOESM2_ESM.pdf]

Supplementary File S1

Dataset title: Shifting frontlines of malaria: Climate-driven redistribution of Anopheles vectors in South America

Table S1. Occurrence records used to model climatic suitability of major malaria vector taxa in South America

species	longitude	latitude	Vector	Souce
Anopheles aberrans	97.996656	19.01668	Unclassified	GBIF: https://doi.org/10.15468/dl.zfaw79
Anopheles aberrans	100.389404	19.896706	Unclassified	GBIF: https://doi.org/10.15468/dl.zfaw79
Anopheles aberrans	100.508752	20.071488	Unclassified	GBIF: https://doi.org/10.15468/dl.zfaw79
Anopheles aberrans	102.058493	12.785252	Unclassified	GBIF: https://doi.org/10.15468/dl.zfaw79
Anopheles aberrans	102.208368	13.969362	Unclassified	GBIF: https://doi.org/10.15468/dl.zfaw79
Anopheles aberrans	103.528317	16.32556	Unclassified	GBIF: https://doi.org/10.15468/dl.zfaw79
Anopheles acanthotorynus	-53.24036	3.91451	Unclassified	GBIF: https://doi.org/10.15468/dl.zfaw79
Anopheles acanthotorynus	-53.23477	3.92404	Unclassified	GBIF: https://doi.org/10.15468/dl.zfaw79
Anopheles acanthotorynus	-52.464115	4.563611	Unclassified	GBIF: https://doi.org/10.15468/dl.zfaw79
Anopheles acanthotorynus	-52.334495	4.941608	Unclassified	GBIF: https://doi.org/10.15468/dl.zfaw79
Anopheles acanthotorynus	-52.323296	4.936976	Unclassified	GBIF: https://doi.org/10.15468/dl.zfaw79
Anopheles acanthotorynus	-51.81813	3.88265	Unclassified	GBIF: https://doi.org/10.15468/dl.zfaw79
Anopheles aconitus	80.66931	7.696631	Unclassified	GBIF: https://doi.org/10.15468/dl.zfaw79
Anopheles aconitus	81.173004	23.5863	Unclassified	GBIF: https://doi.org/10.15468/dl.zfaw79
Anopheles aconitus	84.14	27.63	Unclassified	GBIF: https://doi.org/10.15468/dl.zfaw79
Anopheles aconitus	85.92	27	Unclassified	GBIF: https://doi.org/10.15468/dl.zfaw79
Anopheles aconitus	92.66	11.74	Unclassified	GBIF: https://doi.org/10.15468/dl.zfaw79
Anopheles aconitus	95.323611	5.549444	Unclassified	GBIF: https://doi.org/10.15468/dl.zfaw79
Anopheles aconitus	97.809343	18.851386	Unclassified	GBIF: https://doi.org/10.15468/dl.zfaw79
Anopheles aconitus	97.928027	18.082159	Unclassified	GBIF: https://doi.org/10.15468/dl.zfaw79
Anopheles aconitus	97.938127	18.131016	Unclassified	GBIF: https://doi.org/10.15468/dl.zfaw79
Anopheles aconitus	97.951992	18.183508	Unclassified	GBIF: https://doi.org/10.15468/dl.zfaw79
Anopheles aconitus	97.982849	18.328586	Unclassified	GBIF: https://doi.org/10.15468/dl.zfaw79
Anopheles aconitus	98.004753	18.860719	Unclassified	GBIF: https://doi.org/10.15468/dl.zfaw79
Anopheles aconitus	98.116084	19.533555	Unclassified	GBIF: https://doi.org/10.15468/dl.zfaw79
Anopheles aconitus	98.368106	14.679777	Unclassified	GBIF: https://doi.org/10.15468/dl.zfaw79
Anopheles aconitus	98.37818	18.49362	Unclassified	GBIF: https://doi.org/10.15468/dl.zfaw79
Anopheles aconitus	98.38201	18.48188	Unclassified	GBIF: https://doi.org/10.15468/dl.zfaw79
Anopheles aconitus	98.391201	8.333326	Unclassified	GBIF: https://doi.org/10.15468/dl.zfaw79
Anopheles aconitus	98.407947	19.477815	Unclassified	GBIF: https://doi.org/10.15468/dl.zfaw79
Anopheles aconitus	98.425492	19.365813	Unclassified	GBIF: https://doi.org/10.15468/dl.zfaw79
Anopheles aconitus	98.438844	19.359529	Unclassified	GBIF: https://doi.org/10.15468/dl.zfaw79
Anopheles aconitus	98.459221	18.420279	Unclassified	GBIF: https://doi.org/10.15468/dl.zfaw79
Anopheles aconitus	98.477145	19.291865	Unclassified	GBIF: https://doi.org/10.15468/dl.zfaw79
Anopheles aconitus	98.490191	17.788002	Unclassified	GBIF: https://doi.org/10.15468/dl.zfaw79
Anopheles aconitus	98.492188	17.040433	Unclassified	GBIF: https://doi.org/10.15468/dl.zfaw79

Anopheles aconitus	98.492807	9.451781	Unclassified	GBIF: https://doi.org/10.15468/dl.zfaw79
Anopheles aconitus	98.564605	14.636853	Unclassified	GBIF: https://doi.org/10.15468/dl.zfaw79
Anopheles aconitus	98.62446	8.58293	Unclassified	GBIF: https://doi.org/10.15468/dl.zfaw79
Anopheles aconitus	98.66423	18.50429	Unclassified	GBIF: https://doi.org/10.15468/dl.zfaw79
Anopheles aconitus	98.6747	18.48171	Unclassified	GBIF: https://doi.org/10.15468/dl.zfaw79
Anopheles aconitus	98.67657	18.49346	Unclassified	GBIF: https://doi.org/10.15468/dl.zfaw79
Anopheles aconitus	98.67941	18.49347	Unclassified	GBIF: https://doi.org/10.15468/dl.zfaw79
Anopheles aconitus	98.6813	18.49347	Unclassified	GBIF: https://doi.org/10.15468/dl.zfaw79
Anopheles aconitus	98.696078	16.504844	Unclassified	GBIF: https://doi.org/10.15468/dl.zfaw79
Anopheles aconitus	98.720925	14.670656	Unclassified	GBIF: https://doi.org/10.15468/dl.zfaw79
Anopheles aconitus	98.795965	8.284143	Unclassified	GBIF: https://doi.org/10.15468/dl.zfaw79
Anopheles aconitus	98.836142	16.435759	Unclassified	GBIF: https://doi.org/10.15468/dl.zfaw79
Anopheles aconitus	98.838979	10.523317	Unclassified	GBIF: https://doi.org/10.15468/dl.zfaw79
Anopheles aconitus	98.844326	16.04819	Unclassified	GBIF: https://doi.org/10.15468/dl.zfaw79
Anopheles aconitus	98.90083	18.90584	Unclassified	GBIF: https://doi.org/10.15468/dl.zfaw79
Anopheles aconitus	98.90387	18.57506	Unclassified	GBIF: https://doi.org/10.15468/dl.zfaw79
Anopheles aconitus	98.90558	18.91308	Unclassified	GBIF: https://doi.org/10.15468/dl.zfaw79
Anopheles aconitus	98.90558	18.91036	Unclassified	GBIF: https://doi.org/10.15468/dl.zfaw79
Anopheles aconitus	98.93293	19.38666	Unclassified	GBIF: https://doi.org/10.15468/dl.zfaw79
Anopheles aconitus	98.93316	18.79831	Unclassified	GBIF: https://doi.org/10.15468/dl.zfaw79
Anopheles aconitus	98.940808	16.179539	Unclassified	GBIF: https://doi.org/10.15468/dl.zfaw79
Anopheles aconitus	98.9417	18.81187	Unclassified	GBIF: https://doi.org/10.15468/dl.zfaw79
Anopheles aconitus	98.94455	18.81187	Unclassified	GBIF: https://doi.org/10.15468/dl.zfaw79
Anopheles aconitus	98.94834	18.81187	Unclassified	GBIF: https://doi.org/10.15468/dl.zfaw79
Anopheles aconitus	98.9533	9.03	Unclassified	GBIF: https://doi.org/10.15468/dl.zfaw79
Anopheles aconitus	98.96256	18.87514	Unclassified	GBIF: https://doi.org/10.15468/dl.zfaw79
Anopheles aconitus	98.96256	18.87695	Unclassified	GBIF: https://doi.org/10.15468/dl.zfaw79
Anopheles aconitus	98.98257	19.16918	Unclassified	GBIF: https://doi.org/10.15468/dl.zfaw79
Anopheles aconitus	98.988528	14.335889	Unclassified	GBIF: https://doi.org/10.15468/dl.zfaw79
Anopheles aconitus	99.00054	18.79561	Unclassified	GBIF: https://doi.org/10.15468/dl.zfaw79
Anopheles aconitus	99.001515	10.516334	Unclassified	GBIF: https://doi.org/10.15468/dl.zfaw79
Anopheles aconitus	99.077778	1.559722	Unclassified	GBIF: https://doi.org/10.15468/dl.zfaw79
Anopheles aconitus	99.097464	10.473811	Unclassified	GBIF: https://doi.org/10.15468/dl.zfaw79
Anopheles aconitus	99.105666	10.407784	Unclassified	GBIF: https://doi.org/10.15468/dl.zfaw79
Anopheles aconitus	99.110267	10.50094	Unclassified	GBIF: https://doi.org/10.15468/dl.zfaw79
Anopheles aconitus	99.11209	10.488277	Unclassified	GBIF: https://doi.org/10.15468/dl.zfaw79
Anopheles aconitus	99.118995	10.549008	Unclassified	GBIF: https://doi.org/10.15468/dl.zfaw79
Anopheles aconitus	99.126704	10.469278	Unclassified	GBIF: https://doi.org/10.15468/dl.zfaw79
Anopheles aconitus	99.149627	13.893048	Unclassified	GBIF: https://doi.org/10.15468/dl.zfaw79
Anopheles aconitus	99.181219	10.671592	Unclassified	GBIF: https://doi.org/10.15468/dl.zfaw79
Anopheles aconitus	99.19213	16.500795	Unclassified	GBIF: https://doi.org/10.15468/dl.zfaw79
Anopheles aconitus	99.201877	10.857277	Unclassified	GBIF: https://doi.org/10.15468/dl.zfaw79

Anopheles aconitus	99.211781	14.046716	Unclassified	GBIF: https://doi.org/10.15468/dl.zfaw79
Anopheles aconitus	99.237565	16.403682	Unclassified	GBIF: https://doi.org/10.15468/dl.zfaw79
Anopheles aconitus	99.24081	18.92017	Unclassified	GBIF: https://doi.org/10.15468/dl.zfaw79
Anopheles aconitus	99.254742	13.458063	Unclassified	GBIF: https://doi.org/10.15468/dl.zfaw79
Anopheles aconitus	99.2725	1.378056	Unclassified	GBIF: https://doi.org/10.15468/dl.zfaw79
Anopheles aconitus	99.296459	12.920093	Unclassified	GBIF: https://doi.org/10.15468/dl.zfaw79
Anopheles aconitus	99.316465	16.600952	Unclassified	GBIF: https://doi.org/10.15468/dl.zfaw79
Anopheles aconitus	99.359444	0.798056	Unclassified	GBIF: https://doi.org/10.15468/dl.zfaw79
Anopheles aconitus	99.361265	14.392985	Unclassified	GBIF: https://doi.org/10.15468/dl.zfaw79
Anopheles aconitus	99.377469	14.5429	Unclassified	GBIF: https://doi.org/10.15468/dl.zfaw79
Anopheles aconitus	99.42	13.29	Unclassified	GBIF: https://doi.org/10.15468/dl.zfaw79
Anopheles aconitus	99.445328	12.649994	Unclassified	GBIF: https://doi.org/10.15468/dl.zfaw79
Anopheles aconitus	99.600483	12.815447	Unclassified	GBIF: https://doi.org/10.15468/dl.zfaw79
Anopheles aconitus	99.653077	12.777445	Unclassified	GBIF: https://doi.org/10.15468/dl.zfaw79
Anopheles aconitus	99.708503	12.718746	Unclassified	GBIF: https://doi.org/10.15468/dl.zfaw79
Anopheles aconitus	99.71	12.9	Unclassified	GBIF: https://doi.org/10.15468/dl.zfaw79
Anopheles aconitus	99.744448	13.199366	Unclassified	GBIF: https://doi.org/10.15468/dl.zfaw79
Anopheles aconitus	99.79323	8.3346	Unclassified	GBIF: https://doi.org/10.15468/dl.zfaw79
Anopheles aconitus	99.81042	8.13246	Unclassified	GBIF: https://doi.org/10.15468/dl.zfaw79
Anopheles aconitus	99.8965	19.00492	Unclassified	GBIF: https://doi.org/10.15468/dl.zfaw79
Anopheles aconitus	99.91724	9.57275	Unclassified	GBIF: https://doi.org/10.15468/dl.zfaw79
Anopheles aconitus	99.92635	9.57272	Unclassified	GBIF: https://doi.org/10.15468/dl.zfaw79
Anopheles aconitus	99.93536	9.53653	Unclassified	GBIF: https://doi.org/10.15468/dl.zfaw79
Anopheles aconitus	99.93539	9.54557	Unclassified	GBIF: https://doi.org/10.15468/dl.zfaw79
Anopheles aconitus	99.93541	9.55461	Unclassified	GBIF: https://doi.org/10.15468/dl.zfaw79
Anopheles aconitus	99.94396	18.82756	Unclassified	GBIF: https://doi.org/10.15468/dl.zfaw79
Anopheles aconitus	99.94416	9.42346	Unclassified	GBIF: https://doi.org/10.15468/dl.zfaw79
Anopheles aconitus	99.94447	9.5365	Unclassified	GBIF: https://doi.org/10.15468/dl.zfaw79
Anopheles aconitus	99.94491	18.82756	Unclassified	GBIF: https://doi.org/10.15468/dl.zfaw79
Anopheles aconitus	99.9831	18.70356	Unclassified	GBIF: https://doi.org/10.15468/dl.zfaw79
Anopheles aconitus	100.012778	0.537222	Unclassified	GBIF: https://doi.org/10.15468/dl.zfaw79
Anopheles aconitus	100.02696	9.69905	Unclassified	GBIF: https://doi.org/10.15468/dl.zfaw79
Anopheles aconitus	100.02699	9.70809	Unclassified	GBIF: https://doi.org/10.15468/dl.zfaw79
Anopheles aconitus	100.3675	-0.300278	Unclassified	GBIF: https://doi.org/10.15468/dl.zfaw79
Anopheles aconitus	100.389404	19.896706	Unclassified	GBIF: https://doi.org/10.15468/dl.zfaw79
Anopheles aconitus	100.4927	18.42009	Unclassified	GBIF: https://doi.org/10.15468/dl.zfaw79
Anopheles aconitus	100.508752	20.071488	Unclassified	GBIF: https://doi.org/10.15468/dl.zfaw79
Anopheles aconitus	100.53101	13.739892	Unclassified	GBIF: https://doi.org/10.15468/dl.zfaw79
Anopheles aconitus	100.53426	14.06371	Unclassified	GBIF: https://doi.org/10.15468/dl.zfaw79
Anopheles aconitus	100.53429	14.06823	Unclassified	GBIF: https://doi.org/10.15468/dl.zfaw79
Anopheles aconitus	100.54337	14.04105	Unclassified	GBIF: https://doi.org/10.15468/dl.zfaw79
Anopheles aconitus	100.54608	13.75178	Unclassified	GBIF: https://doi.org/10.15468/dl.zfaw79

Anopheles aconitus	100.54812	14.0591	Unclassified	GBIF: https://doi.org/10.15468/dl.zfaw79
Anopheles aconitus	100.58727	18.41028	Unclassified	GBIF: https://doi.org/10.15468/dl.zfaw79
Anopheles aconitus	100.72826	14.61101	Unclassified	GBIF: https://doi.org/10.15468/dl.zfaw79
Anopheles aconitus	100.85772	14.66424	Unclassified	GBIF: https://doi.org/10.15468/dl.zfaw79
Anopheles aconitus	100.89131	14.68476	Unclassified	GBIF: https://doi.org/10.15468/dl.zfaw79
Anopheles aconitus	100.89854	14.6612	Unclassified	GBIF: https://doi.org/10.15468/dl.zfaw79
Anopheles aconitus	100.95415	14.01007	Unclassified	GBIF: https://doi.org/10.15468/dl.zfaw79
Anopheles aconitus	100.96246	14.43655	Unclassified	GBIF: https://doi.org/10.15468/dl.zfaw79
Anopheles aconitus	101	5	Unclassified	GBIF: https://doi.org/10.15468/dl.zfaw79
Anopheles aconitus	101.05673	14.60383	Unclassified	GBIF: https://doi.org/10.15468/dl.zfaw79
Anopheles aconitus	101.268126	15.693213	Unclassified	GBIF: https://doi.org/10.15468/dl.zfaw79
Anopheles aconitus	101.50725	14.60882	Unclassified	GBIF: https://doi.org/10.15468/dl.zfaw79
Anopheles aconitus	101.555706	6.256998	Unclassified	GBIF: https://doi.org/10.15468/dl.zfaw79
Anopheles aconitus	101.576794	14.46852	Unclassified	GBIF: https://doi.org/10.15468/dl.zfaw79
Anopheles aconitus	101.665313	6.076102	Unclassified	GBIF: https://doi.org/10.15468/dl.zfaw79
Anopheles aconitus	101.71016	5.94843	Unclassified	GBIF: https://doi.org/10.15468/dl.zfaw79
Anopheles aconitus	101.737005	5.843329	Unclassified	GBIF: https://doi.org/10.15468/dl.zfaw79
Anopheles aconitus	101.794338	13.49568	Unclassified	GBIF: https://doi.org/10.15468/dl.zfaw79
Anopheles aconitus	101.866043	14.358637	Unclassified	GBIF: https://doi.org/10.15468/dl.zfaw79
Anopheles aconitus	101.8966	14.19572	Unclassified	GBIF: https://doi.org/10.15468/dl.zfaw79
Anopheles aconitus	101.914358	14.389737	Unclassified	GBIF: https://doi.org/10.15468/dl.zfaw79
Anopheles aconitus	101.92694	14.39404	Unclassified	GBIF: https://doi.org/10.15468/dl.zfaw79
Anopheles aconitus	102.02336	12.60527	Unclassified	GBIF: https://doi.org/10.15468/dl.zfaw79
Anopheles aconitus	102.03245	12.6144	Unclassified	GBIF: https://doi.org/10.15468/dl.zfaw79
Anopheles aconitus	102.03256	12.60537	Unclassified	GBIF: https://doi.org/10.15468/dl.zfaw79
Anopheles aconitus	102.07513	13.67163	Unclassified	GBIF: https://doi.org/10.15468/dl.zfaw79
Anopheles aconitus	102.127301	17.32058	Unclassified	GBIF: https://doi.org/10.15468/dl.zfaw79
Anopheles aconitus	102.192464	13.280892	Unclassified	GBIF: https://doi.org/10.15468/dl.zfaw79
Anopheles aconitus	102.404232	14.335548	Unclassified	GBIF: https://doi.org/10.15468/dl.zfaw79
Anopheles aconitus	102.413476	13.824381	Unclassified	GBIF: https://doi.org/10.15468/dl.zfaw79
Anopheles aconitus	102.462267	12.595272	Unclassified	GBIF: https://doi.org/10.15468/dl.zfaw79
Anopheles aconitus	102.574818	14.044675	Unclassified	GBIF: https://doi.org/10.15468/dl.zfaw79
Anopheles aconitus	102.618292	12.230746	Unclassified	GBIF: https://doi.org/10.15468/dl.zfaw79
Anopheles aconitus	102.692968	12.387497	Unclassified	GBIF: https://doi.org/10.15468/dl.zfaw79
Anopheles aconitus	102.967142	14.369102	Unclassified	GBIF: https://doi.org/10.15468/dl.zfaw79
Anopheles aconitus	103.38384	15.05659	Unclassified	GBIF: https://doi.org/10.15468/dl.zfaw79
Anopheles aconitus	103.38386	15.05388	Unclassified	GBIF: https://doi.org/10.15468/dl.zfaw79
Anopheles aconitus	103.444973	14.449552	Unclassified	GBIF: https://doi.org/10.15468/dl.zfaw79
Anopheles aconitus	103.588787	16.376585	Unclassified	GBIF: https://doi.org/10.15468/dl.zfaw79
Anopheles aconitus	103.63334	17.130957	Unclassified	GBIF: https://doi.org/10.15468/dl.zfaw79
Anopheles aconitus	103.728408	16.926085	Unclassified	GBIF: https://doi.org/10.15468/dl.zfaw79
Anopheles aconitus	104.063014	14.433857	Unclassified	GBIF: https://doi.org/10.15468/dl.zfaw79

Anopheles aconitus	104.208742	14.503051	Unclassified	GBIF: https://doi.org/10.15468/dl.zfaw79
Anopheles aconitus	104.24534	16.83593	Unclassified	GBIF: https://doi.org/10.15468/dl.zfaw79
Anopheles aconitus	104.312034	14.554037	Unclassified	GBIF: https://doi.org/10.15468/dl.zfaw79
Anopheles aconitus	104.320394	16.718235	Unclassified	GBIF: https://doi.org/10.15468/dl.zfaw79
Anopheles aconitus	104.409567	16.802636	Unclassified	GBIF: https://doi.org/10.15468/dl.zfaw79
Anopheles aconitus	104.69114	38.073254	Unclassified	GBIF: https://doi.org/10.15468/dl.zfaw79
Anopheles aconitus	104.691789	14.480704	Unclassified	GBIF: https://doi.org/10.15468/dl.zfaw79
Anopheles aconitus	104.724816	16.195812	Unclassified	GBIF: https://doi.org/10.15468/dl.zfaw79
Anopheles aconitus	104.769864	16.22415	Unclassified	GBIF: https://doi.org/10.15468/dl.zfaw79
Anopheles aconitus	104.844444	14.473205	Unclassified	GBIF: https://doi.org/10.15468/dl.zfaw79
Anopheles aconitus	104.92	13.77	Unclassified	GBIF: https://doi.org/10.15468/dl.zfaw79
Anopheles aconitus	104.92039	13.76981	Unclassified	GBIF: https://doi.org/10.15468/dl.zfaw79
Anopheles aconitus	105.137188	14.552966	Unclassified	GBIF: https://doi.org/10.15468/dl.zfaw79
Anopheles aconitus	105.311586	15.697635	Unclassified	GBIF: https://doi.org/10.15468/dl.zfaw79
Anopheles aconitus	105.36191	15.656875	Unclassified	GBIF: https://doi.org/10.15468/dl.zfaw79
Anopheles aconitus	105.506659	15.695174	Unclassified	GBIF: https://doi.org/10.15468/dl.zfaw79
Anopheles aconitus	105.91339	16.517347	Unclassified	GBIF: https://doi.org/10.15468/dl.zfaw79
Anopheles aconitus	106.75	-6.13	Unclassified	GBIF: https://doi.org/10.15468/dl.zfaw79
Anopheles aconitus	106.78333	-6.58333	Unclassified	GBIF: https://doi.org/10.15468/dl.zfaw79
Anopheles aconitus	106.826056	-6.182496	Unclassified	GBIF: https://doi.org/10.15468/dl.zfaw79
Anopheles aconitus	106.845278	-6.204167	Unclassified	GBIF: https://doi.org/10.15468/dl.zfaw79
Anopheles aconitus	106.932333	-6.680078	Unclassified	GBIF: https://doi.org/10.15468/dl.zfaw79
Anopheles aconitus	108.53	12.16	Unclassified	GBIF: https://doi.org/10.15468/dl.zfaw79
Anopheles aconitus	108.59	13.37	Unclassified	GBIF: https://doi.org/10.15468/dl.zfaw79
Anopheles aconitus	109.02	13.22	Unclassified	GBIF: https://doi.org/10.15468/dl.zfaw79
Anopheles aconitus	109.023056	-7.697778	Unclassified	GBIF: https://doi.org/10.15468/dl.zfaw79
Anopheles aconitus	109.13714	-7.681584	Unclassified	GBIF: https://doi.org/10.15468/dl.zfaw79
Anopheles aconitus	109.146389	-7.684283	Unclassified	GBIF: https://doi.org/10.15468/dl.zfaw79
Anopheles aconitus	110.217656	-7.479992	Unclassified	GBIF: https://doi.org/10.15468/dl.zfaw79
Anopheles aconitus	110.218997	-7.46058	Unclassified	GBIF: https://doi.org/10.15468/dl.zfaw79
Anopheles aconitus	110.438056	-7.004722	Unclassified	GBIF: https://doi.org/10.15468/dl.zfaw79
Anopheles aconitus	110.818812	-7.559209	Unclassified	GBIF: https://doi.org/10.15468/dl.zfaw79
Anopheles aconitus	112.321944	-7.621667	Unclassified	GBIF: https://doi.org/10.15468/dl.zfaw79
Anopheles aconitus	112.749444	-7.256111	Unclassified	GBIF: https://doi.org/10.15468/dl.zfaw79
Anopheles aconitus	114.005833	-7.705556	Unclassified	GBIF: https://doi.org/10.15468/dl.zfaw79
Anopheles aconitus	116.854444	-1.2375	Unclassified	GBIF: https://doi.org/10.15468/dl.zfaw79
Anopheles aconitus	119.62	-2.37	Unclassified	GBIF: https://doi.org/10.15468/dl.zfaw79
Anopheles aconitus	119.872251	-0.819373	Unclassified	GBIF: https://doi.org/10.15468/dl.zfaw79
Anopheles aconitus	122.038574	-5.309061	Unclassified	GBIF: https://doi.org/10.15468/dl.zfaw79
Anopheles ahomi	92.857876	26.056061	Unclassified	GBIF: https://doi.org/10.15468/dl.zfaw79
Anopheles aitkenii	75.9	11.74	Unclassified	GBIF: https://doi.org/10.15468/dl.zfaw79
Anopheles aitkenii	76	11.8	Unclassified	GBIF: https://doi.org/10.15468/dl.zfaw79

Anopheles aitkenii	84.809736	27.540286	Unclassified	GBIF: https://doi.org/10.15468/dl.zfaw79
Anopheles aitkenii	84.989986	27.279238	Unclassified	GBIF: https://doi.org/10.15468/dl.zfaw79
Anopheles aitkenii	98.503333	3.195	Unclassified	GBIF: https://doi.org/10.15468/dl.zfaw79
Anopheles aitkenii	98.540942	18.541976	Unclassified	GBIF: https://doi.org/10.15468/dl.zfaw79
Anopheles aitkenii	98.921222	18.803611	Unclassified	GBIF: https://doi.org/10.15468/dl.zfaw79
Anopheles aitkenii	98.944538	18.8118	Unclassified	GBIF: https://doi.org/10.15468/dl.zfaw79
Anopheles aitkenii	99.004682	18.787044	Unclassified	GBIF: https://doi.org/10.15468/dl.zfaw79
Anopheles aitkenii	99.045584	18.926528	Unclassified	GBIF: https://doi.org/10.15468/dl.zfaw79
Anopheles aitkenii	99.359444	0.798056	Unclassified	GBIF: https://doi.org/10.15468/dl.zfaw79
Anopheles aitkenii	99.547059	7.54491	Unclassified	GBIF: https://doi.org/10.15468/dl.zfaw79
Anopheles aitkenii	99.547186	7.528619	Unclassified	GBIF: https://doi.org/10.15468/dl.zfaw79
Anopheles aitkenii	99.558616	7.549903	Unclassified	GBIF: https://doi.org/10.15468/dl.zfaw79
Anopheles aitkenii	99.560509	7.52389	Unclassified	GBIF: https://doi.org/10.15468/dl.zfaw79
Anopheles aitkenii	99.564025	7.547976	Unclassified	GBIF: https://doi.org/10.15468/dl.zfaw79
Anopheles aitkenii	99.564665	7.521019	Unclassified	GBIF: https://doi.org/10.15468/dl.zfaw79
Anopheles aitkenii	99.565704	7.557181	Unclassified	GBIF: https://doi.org/10.15468/dl.zfaw79
Anopheles aitkenii	99.570274	7.540336	Unclassified	GBIF: https://doi.org/10.15468/dl.zfaw79
Anopheles aitkenii	99.571468	7.56602	Unclassified	GBIF: https://doi.org/10.15468/dl.zfaw79
Anopheles aitkenii	99.582179	7.552467	Unclassified	GBIF: https://doi.org/10.15468/dl.zfaw79
Anopheles aitkenii	99.591853	7.559183	Unclassified	GBIF: https://doi.org/10.15468/dl.zfaw79
Anopheles aitkenii	99.593762	7.528875	Unclassified	GBIF: https://doi.org/10.15468/dl.zfaw79
Anopheles aitkenii	99.601074	7.527655	Unclassified	GBIF: https://doi.org/10.15468/dl.zfaw79
Anopheles aitkenii	99.601616	7.556635	Unclassified	GBIF: https://doi.org/10.15468/dl.zfaw79
Anopheles aitkenii	99.794747	7.540405	Unclassified	GBIF: https://doi.org/10.15468/dl.zfaw79
Anopheles aitkenii	99.794963	7.535938	Unclassified	GBIF: https://doi.org/10.15468/dl.zfaw79
Anopheles aitkenii	99.795837	7.540615	Unclassified	GBIF: https://doi.org/10.15468/dl.zfaw79
Anopheles aitkenii	103.938983	-4.880763	Unclassified	GBIF: https://doi.org/10.15468/dl.zfaw79
Anopheles aitkenii	104.69114	38.073254	Unclassified	GBIF: https://doi.org/10.15468/dl.zfaw79
Anopheles aitkenii	106.931389	-6.679722	Unclassified	GBIF: https://doi.org/10.15468/dl.zfaw79
Anopheles aitkenii	108.905278	-7.724444	Unclassified	GBIF: https://doi.org/10.15468/dl.zfaw79
Anopheles aitkenii	109.023056	-7.697778	Unclassified	GBIF: https://doi.org/10.15468/dl.zfaw79
Anopheles aitkenii	110.333	1.4167	Unclassified	GBIF: https://doi.org/10.15468/dl.zfaw79
Anopheles aitkenii	128.1025	-3.623333	Unclassified	GBIF: https://doi.org/10.15468/dl.zfaw79
Anopheles aitkenii	128.189722	-3.058611	Unclassified	GBIF: https://doi.org/10.15468/dl.zfaw79
Anopheles aitkenii	128.19	-3.058889	Unclassified	GBIF: https://doi.org/10.15468/dl.zfaw79
Anopheles albertoi	-49.08	-20.03	Unclassified	GBIF: https://doi.org/10.15468/dl.zfaw79
Anopheles albimanus	-109.708055	23.061389	Primary	GBIF: https://doi.org/10.15468/dl.zfaw79
Anopheles albimanus	-109.699594	23.059465	Primary	GBIF: https://doi.org/10.15468/dl.zfaw79
Anopheles albimanus	-109.68387	23.05045	Primary	GBIF: https://doi.org/10.15468/dl.zfaw79
Anopheles albimanus	-109.303278	25.973861	Primary	GBIF: https://doi.org/10.15468/dl.zfaw79
Anopheles albimanus	-109.049722	25.6	Primary	GBIF: https://doi.org/10.15468/dl.zfaw79
Anopheles albimanus	-108.8305	26.016139	Primary	GBIF: https://doi.org/10.15468/dl.zfaw79

Anopheles albimanus	-108.21675	25.823417	Primary	GBIF: https://doi.org/10.15468/dl.zfaw79
Anopheles albimanus	-108.195139	25.217139	Primary	GBIF: https://doi.org/10.15468/dl.zfaw79
Anopheles albimanus	-107.701111	24.764722	Primary	GBIF: https://doi.org/10.15468/dl.zfaw79
Anopheles albimanus	-107.693389	25.093722	Primary	GBIF: https://doi.org/10.15468/dl.zfaw79
Anopheles albimanus	-107.367778	24.323611	Primary	GBIF: https://doi.org/10.15468/dl.zfaw79
Anopheles albimanus	-106.780555	23.723056	Primary	GBIF: https://doi.org/10.15468/dl.zfaw79
Anopheles albimanus	-106.59775	23.792861	Primary	GBIF: https://doi.org/10.15468/dl.zfaw79
Anopheles albimanus	-106.415278	23.236111	Primary	GBIF: https://doi.org/10.15468/dl.zfaw79
Anopheles albimanus	-105.729194	22.532972	Primary	GBIF: https://doi.org/10.15468/dl.zfaw79
Anopheles albimanus	-105.686278	22.379917	Primary	GBIF: https://doi.org/10.15468/dl.zfaw79
Anopheles albimanus	-105.612444	22.011861	Primary	GBIF: https://doi.org/10.15468/dl.zfaw79
Anopheles albimanus	-105.447361	22.530306	Primary	GBIF: https://doi.org/10.15468/dl.zfaw79
Anopheles albimanus	-105.285333	21.627778	Primary	GBIF: https://doi.org/10.15468/dl.zfaw79
Anopheles albimanus	-105.285333	21.530583	Primary	GBIF: https://doi.org/10.15468/dl.zfaw79
Anopheles albimanus	-105.285278	21.539444	Primary	GBIF: https://doi.org/10.15468/dl.zfaw79
Anopheles albimanus	-105.285111	21.538139	Primary	GBIF: https://doi.org/10.15468/dl.zfaw79
Anopheles albimanus	-105.25044	20.61725	Primary	GBIF: https://doi.org/10.15468/dl.zfaw79
Anopheles albimanus	-105.231111	20.613333	Primary	GBIF: https://doi.org/10.15468/dl.zfaw79
Anopheles albimanus	-105.10044	22.86714	Primary	GBIF: https://doi.org/10.15468/dl.zfaw79
Anopheles albimanus	-105.078722	19.539194	Primary	GBIF: https://doi.org/10.15468/dl.zfaw79
Anopheles albimanus	-104.889361	19.352139	Primary	GBIF: https://doi.org/10.15468/dl.zfaw79
Anopheles albimanus	-104.815861	19.308389	Primary	GBIF: https://doi.org/10.15468/dl.zfaw79
Anopheles albimanus	-104.685833	19.221611	Primary	GBIF: https://doi.org/10.15468/dl.zfaw79
Anopheles albimanus	-104.682778	19.204167	Primary	GBIF: https://doi.org/10.15468/dl.zfaw79
Anopheles albimanus	-104.519639	19.5345	Primary	GBIF: https://doi.org/10.15468/dl.zfaw79
Anopheles albimanus	-104.519611	19.534194	Primary	GBIF: https://doi.org/10.15468/dl.zfaw79
Anopheles albimanus	-104.287833	19.049472	Primary	GBIF: https://doi.org/10.15468/dl.zfaw79
Anopheles albimanus	-104.259722	19.06	Primary	GBIF: https://doi.org/10.15468/dl.zfaw79
Anopheles albimanus	-104.070555	18.919444	Primary	GBIF: https://doi.org/10.15468/dl.zfaw79
Anopheles albimanus	-104.066389	18.930056	Primary	GBIF: https://doi.org/10.15468/dl.zfaw79
Anopheles albimanus	-104.066	18.927861	Primary	GBIF: https://doi.org/10.15468/dl.zfaw79
Anopheles albimanus	-104.065639	18.927361	Primary	GBIF: https://doi.org/10.15468/dl.zfaw79
Anopheles albimanus	-103.964889	18.85975	Primary	GBIF: https://doi.org/10.15468/dl.zfaw79
Anopheles albimanus	-103.962222	18.856944	Primary	GBIF: https://doi.org/10.15468/dl.zfaw79
Anopheles albimanus	-103.730556	19.243056	Primary	GBIF: https://doi.org/10.15468/dl.zfaw79
Anopheles albimanus	-103.715944	19.231028	Primary	GBIF: https://doi.org/10.15468/dl.zfaw79
Anopheles albimanus	-103.28373	18.68402	Primary	GBIF: https://doi.org/10.15468/dl.zfaw79
Anopheles albimanus	-102.402278	19.073583	Primary	GBIF: https://doi.org/10.15468/dl.zfaw79
Anopheles albimanus	-102.346305	17.988889	Primary	GBIF: https://doi.org/10.15468/dl.zfaw79
Anopheles albimanus	-102.27175	19.061722	Primary	GBIF: https://doi.org/10.15468/dl.zfaw79
Anopheles albimanus	-102.264444	19.055417	Primary	GBIF: https://doi.org/10.15468/dl.zfaw79
Anopheles albimanus	-102.201139	18.027722	Primary	GBIF: https://doi.org/10.15468/dl.zfaw79

Anopheles albimanus	-101.641389	17.695278	Primary	GBIF: https://doi.org/10.15468/dl.zfaw79
Anopheles albimanus	-101.593055	17.656944	Primary	GBIF: https://doi.org/10.15468/dl.zfaw79
Anopheles albimanus	-101.554	23.874361	Primary	GBIF: https://doi.org/10.15468/dl.zfaw79
Anopheles albimanus	-101.551111	17.644444	Primary	GBIF: https://doi.org/10.15468/dl.zfaw79
Anopheles albimanus	-101.543333	17.627222	Primary	GBIF: https://doi.org/10.15468/dl.zfaw79
Anopheles albimanus	-100.735889	18.899194	Primary	GBIF: https://doi.org/10.15468/dl.zfaw79
Anopheles albimanus	-100.730028	18.897028	Primary	GBIF: https://doi.org/10.15468/dl.zfaw79
Anopheles albimanus	-100.291667	25.9625	Primary	GBIF: https://doi.org/10.15468/dl.zfaw79
Anopheles albimanus	-100.199444	21.085278	Primary	GBIF: https://doi.org/10.15468/dl.zfaw79
Anopheles albimanus	-100.162778	21.073889	Primary	GBIF: https://doi.org/10.15468/dl.zfaw79
Anopheles albimanus	-100.140833	21.013611	Primary	GBIF: https://doi.org/10.15468/dl.zfaw79
Anopheles albimanus	-100.138611	16.961667	Primary	GBIF: https://doi.org/10.15468/dl.zfaw79
Anopheles albimanus	-100.0875	17.007222	Primary	GBIF: https://doi.org/10.15468/dl.zfaw79
Anopheles albimanus	-100.000661	25.705472	Primary	GBIF: https://doi.org/10.15468/dl.zfaw79
Anopheles albimanus	-100.00032	17.66741	Primary	GBIF: https://doi.org/10.15468/dl.zfaw79
Anopheles albimanus	-99.996667	21.931944	Primary	GBIF: https://doi.org/10.15468/dl.zfaw79
Anopheles albimanus	-99.91699	16.85078	Primary	GBIF: https://doi.org/10.15468/dl.zfaw79
Anopheles albimanus	-99.897778	16.871111	Primary	GBIF: https://doi.org/10.15468/dl.zfaw79
Anopheles albimanus	-99.548417	27.479833	Primary	GBIF: https://doi.org/10.15468/dl.zfaw79
Anopheles albimanus	-99.508333	18.308333	Primary	GBIF: https://doi.org/10.15468/dl.zfaw79
Anopheles albimanus	-99.465555	18.294444	Primary	GBIF: https://doi.org/10.15468/dl.zfaw79
Anopheles albimanus	-99.392222	21.957778	Primary	GBIF: https://doi.org/10.15468/dl.zfaw79
Anopheles albimanus	-99.391667	21.920278	Primary	GBIF: https://doi.org/10.15468/dl.zfaw79
Anopheles albimanus	-99.256944	21.975	Primary	GBIF: https://doi.org/10.15468/dl.zfaw79
Anopheles albimanus	-99.224722	16.766944	Primary	GBIF: https://doi.org/10.15468/dl.zfaw79
Anopheles albimanus	-99.191111	19.406667	Primary	GBIF: https://doi.org/10.15468/dl.zfaw79
Anopheles albimanus	-99.121944	24.075	Primary	GBIF: https://doi.org/10.15468/dl.zfaw79
Anopheles albimanus	-99.009722	21.981944	Primary	GBIF: https://doi.org/10.15468/dl.zfaw79
Anopheles albimanus	-98.791667	21.261111	Primary	GBIF: https://doi.org/10.15468/dl.zfaw79
Anopheles albimanus	-98.70031	22.80049	Primary	GBIF: https://doi.org/10.15468/dl.zfaw79
Anopheles albimanus	-98.207778	23.7675	Primary	GBIF: https://doi.org/10.15468/dl.zfaw79
Anopheles albimanus	-97.95029	22.28385	Primary	GBIF: https://doi.org/10.15468/dl.zfaw79
Anopheles albimanus	-97.868611	22.255278	Primary	GBIF: https://doi.org/10.15468/dl.zfaw79
Anopheles albimanus	-97.85029	22.21719	Primary	GBIF: https://doi.org/10.15468/dl.zfaw79
Anopheles albimanus	-97.836111	22.183333	Primary	GBIF: https://doi.org/10.15468/dl.zfaw79
Anopheles albimanus	-97.793694	22.969	Primary	GBIF: https://doi.org/10.15468/dl.zfaw79
Anopheles albimanus	-97.759694	23.026111	Primary	GBIF: https://doi.org/10.15468/dl.zfaw79
Anopheles albimanus	-97.743889	16.165556	Primary	GBIF: https://doi.org/10.15468/dl.zfaw79
Anopheles albimanus	-97.696103	26.190631	Primary	GBIF: https://doi.org/10.15468/dl.zfaw79
Anopheles albimanus	-97.631101	26.132576	Primary	GBIF: https://doi.org/10.15468/dl.zfaw79
Anopheles albimanus	-97.497484	25.901747	Primary	GBIF: https://doi.org/10.15468/dl.zfaw79
Anopheles albimanus	-97.49748	25.90175	Primary	GBIF: https://doi.org/10.15468/dl.zfaw79

Anopheles albimanus	-97.453722	25.602556	Primary	GBIF: https://doi.org/10.15468/dl.zfaw79
Anopheles albimanus	-97.403055	20.958889	Primary	GBIF: https://doi.org/10.15468/dl.zfaw79
Anopheles albimanus	-97.001389	20.140278	Primary	GBIF: https://doi.org/10.15468/dl.zfaw79
Anopheles albimanus	-96.9275	19.54	Primary	GBIF: https://doi.org/10.15468/dl.zfaw79
Anopheles albimanus	-96.914444	18.844167	Primary	GBIF: https://doi.org/10.15468/dl.zfaw79
Anopheles albimanus	-96.849167	20.168889	Primary	GBIF: https://doi.org/10.15468/dl.zfaw79
Anopheles albimanus	-96.726667	20.160556	Primary	GBIF: https://doi.org/10.15468/dl.zfaw79
Anopheles albimanus	-96.490278	15.665833	Primary	GBIF: https://doi.org/10.15468/dl.zfaw79
Anopheles albimanus	-96.463889	15.827778	Primary	GBIF: https://doi.org/10.15468/dl.zfaw79
Anopheles albimanus	-96.360555	18.447778	Primary	GBIF: https://doi.org/10.15468/dl.zfaw79
Anopheles albimanus	-96.221389	19.184722	Primary	GBIF: https://doi.org/10.15468/dl.zfaw79
Anopheles albimanus	-96.137778	19.199167	Primary	GBIF: https://doi.org/10.15468/dl.zfaw79
Anopheles albimanus	-96.126389	16.663889	Primary	GBIF: https://doi.org/10.15468/dl.zfaw79
Anopheles albimanus	-96.107222	19.100833	Primary	GBIF: https://doi.org/10.15468/dl.zfaw79
Anopheles albimanus	-96.0325	18.176389	Primary	GBIF: https://doi.org/10.15468/dl.zfaw79
Anopheles albimanus	-95.949444	18.231667	Primary	GBIF: https://doi.org/10.15468/dl.zfaw79
Anopheles albimanus	-95.841944	18.304167	Primary	GBIF: https://doi.org/10.15468/dl.zfaw79
Anopheles albimanus	-95.795278	18.365	Primary	GBIF: https://doi.org/10.15468/dl.zfaw79
Anopheles albimanus	-95.760555	18.770278	Primary	GBIF: https://doi.org/10.15468/dl.zfaw79
Anopheles albimanus	-95.661111	18.611667	Primary	GBIF: https://doi.org/10.15468/dl.zfaw79
Anopheles albimanus	-95.406	16.091306	Primary	GBIF: https://doi.org/10.15468/dl.zfaw79
Anopheles albimanus	-95.23356	16.20082	Primary	GBIF: https://doi.org/10.15468/dl.zfaw79
Anopheles albimanus	-95.212222	18.449722	Primary	GBIF: https://doi.org/10.15468/dl.zfaw79
Anopheles albimanus	-95.176722	16.194111	Primary	GBIF: https://doi.org/10.15468/dl.zfaw79
Anopheles albimanus	-95.167889	16.183889	Primary	GBIF: https://doi.org/10.15468/dl.zfaw79
Anopheles albimanus	-95.121389	16.39	Primary	GBIF: https://doi.org/10.15468/dl.zfaw79
Anopheles albimanus	-95.120917	16.390194	Primary	GBIF: https://doi.org/10.15468/dl.zfaw79
Anopheles albimanus	-95.094278	16.564611	Primary	GBIF: https://doi.org/10.15468/dl.zfaw79
Anopheles albimanus	-95.03355	17.43409	Primary	GBIF: https://doi.org/10.15468/dl.zfaw79
Anopheles albimanus	-95.027861	16.357305	Primary	GBIF: https://doi.org/10.15468/dl.zfaw79
Anopheles albimanus	-95.026722	16.416667	Primary	GBIF: https://doi.org/10.15468/dl.zfaw79
Anopheles albimanus	-95.026694	16.406361	Primary	GBIF: https://doi.org/10.15468/dl.zfaw79
Anopheles albimanus	-95.025417	16.414555	Primary	GBIF: https://doi.org/10.15468/dl.zfaw79
Anopheles albimanus	-94.755	17.989444	Primary	GBIF: https://doi.org/10.15468/dl.zfaw79
Anopheles albimanus	-94.724722	17.6775	Primary	GBIF: https://doi.org/10.15468/dl.zfaw79
Anopheles albimanus	-94.674167	18.023889	Primary	GBIF: https://doi.org/10.15468/dl.zfaw79
Anopheles albimanus	-94.605	16.557778	Primary	GBIF: https://doi.org/10.15468/dl.zfaw79
Anopheles albimanus	-94.558611	17.988889	Primary	GBIF: https://doi.org/10.15468/dl.zfaw79
Anopheles albimanus	-94.4825	16.350833	Primary	GBIF: https://doi.org/10.15468/dl.zfaw79
Anopheles albimanus	-94.435278	18.137778	Primary	GBIF: https://doi.org/10.15468/dl.zfaw79
Anopheles albimanus	-94.21625	16.402806	Primary	GBIF: https://doi.org/10.15468/dl.zfaw79
Anopheles albimanus	-94.188555	16.355639	Primary	GBIF: https://doi.org/10.15468/dl.zfaw79

Anopheles albimanus	-94.156055	16.279083	Primary	GBIF: https://doi.org/10.15468/dl.zfaw79
Anopheles albimanus	-94.092778	17.91	Primary	GBIF: https://doi.org/10.15468/dl.zfaw79
Anopheles albimanus	-93.79225	16.119528	Primary	GBIF: https://doi.org/10.15468/dl.zfaw79
Anopheles albimanus	-93.776222	15.977472	Primary	GBIF: https://doi.org/10.15468/dl.zfaw79
Anopheles albimanus	-93.414444	15.654722	Primary	GBIF: https://doi.org/10.15468/dl.zfaw79
Anopheles albimanus	-93.411111	15.659722	Primary	GBIF: https://doi.org/10.15468/dl.zfaw79
Anopheles albimanus	-93.410555	15.675833	Primary	GBIF: https://doi.org/10.15468/dl.zfaw79
Anopheles albimanus	-93.410278	15.658333	Primary	GBIF: https://doi.org/10.15468/dl.zfaw79
Anopheles albimanus	-93.405555	15.659167	Primary	GBIF: https://doi.org/10.15468/dl.zfaw79
Anopheles albimanus	-93.398055	15.67	Primary	GBIF: https://doi.org/10.15468/dl.zfaw79
Anopheles albimanus	-93.391667	17.829167	Primary	GBIF: https://doi.org/10.15468/dl.zfaw79
Anopheles albimanus	-93.386111	15.644444	Primary	GBIF: https://doi.org/10.15468/dl.zfaw79
Anopheles albimanus	-93.384444	15.6425	Primary	GBIF: https://doi.org/10.15468/dl.zfaw79
Anopheles albimanus	-93.384167	15.643333	Primary	GBIF: https://doi.org/10.15468/dl.zfaw79
Anopheles albimanus	-93.38	15.675	Primary	GBIF: https://doi.org/10.15468/dl.zfaw79
Anopheles albimanus	-93.375555	18	Primary	GBIF: https://doi.org/10.15468/dl.zfaw79
Anopheles albimanus	-93.369444	15.675	Primary	GBIF: https://doi.org/10.15468/dl.zfaw79
Anopheles albimanus	-93.368333	15.674722	Primary	GBIF: https://doi.org/10.15468/dl.zfaw79
Anopheles albimanus	-93.212222	18.395556	Primary	GBIF: https://doi.org/10.15468/dl.zfaw79
Anopheles albimanus	-93.211111	15.498611	Primary	GBIF: https://doi.org/10.15468/dl.zfaw79
Anopheles albimanus	-93.191667	15.575	Primary	GBIF: https://doi.org/10.15468/dl.zfaw79
Anopheles albimanus	-93.191667	15.541667	Primary	GBIF: https://doi.org/10.15468/dl.zfaw79
Anopheles albimanus	-93.166667	15.449722	Primary	GBIF: https://doi.org/10.15468/dl.zfaw79
Anopheles albimanus	-93.163889	15.452222	Primary	GBIF: https://doi.org/10.15468/dl.zfaw79
Anopheles albimanus	-93.163055	15.450833	Primary	GBIF: https://doi.org/10.15468/dl.zfaw79
Anopheles albimanus	-93.161111	15.452778	Primary	GBIF: https://doi.org/10.15468/dl.zfaw79
Anopheles albimanus	-93.149444	15.459167	Primary	GBIF: https://doi.org/10.15468/dl.zfaw79
Anopheles albimanus	-93.147222	15.458333	Primary	GBIF: https://doi.org/10.15468/dl.zfaw79
Anopheles albimanus	-93.147222	15.458056	Primary	GBIF: https://doi.org/10.15468/dl.zfaw79
Anopheles albimanus	-93.141667	15.447222	Primary	GBIF: https://doi.org/10.15468/dl.zfaw79
Anopheles albimanus	-93.140278	15.449722	Primary	GBIF: https://doi.org/10.15468/dl.zfaw79
Anopheles albimanus	-93.133055	15.566389	Primary	GBIF: https://doi.org/10.15468/dl.zfaw79
Anopheles albimanus	-93.133055	15.449722	Primary	GBIF: https://doi.org/10.15468/dl.zfaw79
Anopheles albimanus	-93.122222	15.433333	Primary	GBIF: https://doi.org/10.15468/dl.zfaw79
Anopheles albimanus	-93.12	15.431667	Primary	GBIF: https://doi.org/10.15468/dl.zfaw79
Anopheles albimanus	-93.119167	15.431667	Primary	GBIF: https://doi.org/10.15468/dl.zfaw79
Anopheles albimanus	-93.116667	16.752778	Primary	GBIF: https://doi.org/10.15468/dl.zfaw79
Anopheles albimanus	-93.103611	15.58	Primary	GBIF: https://doi.org/10.15468/dl.zfaw79
Anopheles albimanus	-93.1025	15.398333	Primary	GBIF: https://doi.org/10.15468/dl.zfaw79
Anopheles albimanus	-93.1	15.4	Primary	GBIF: https://doi.org/10.15468/dl.zfaw79
Anopheles albimanus	-93.094444	15.438889	Primary	GBIF: https://doi.org/10.15468/dl.zfaw79
Anopheles albimanus	-93.094167	15.436944	Primary	GBIF: https://doi.org/10.15468/dl.zfaw79

Anopheles albimanus	-93.093889	15.438056	Primary	GBIF: https://doi.org/10.15468/dl.zfaw79
Anopheles albimanus	-93.078611	17.548056	Primary	GBIF: https://doi.org/10.15468/dl.zfaw79
Anopheles albimanus	-92.952778	17.548056	Primary	GBIF: https://doi.org/10.15468/dl.zfaw79
Anopheles albimanus	-92.928055	17.990556	Primary	GBIF: https://doi.org/10.15468/dl.zfaw79
Anopheles albimanus	-92.91684	17.98407	Primary	GBIF: https://doi.org/10.15468/dl.zfaw79
Anopheles albimanus	-92.908333	15.425	Primary	GBIF: https://doi.org/10.15468/dl.zfaw79
Anopheles albimanus	-92.903889	15.280833	Primary	GBIF: https://doi.org/10.15468/dl.zfaw79
Anopheles albimanus	-92.899167	15.266389	Primary	GBIF: https://doi.org/10.15468/dl.zfaw79
Anopheles albimanus	-92.897222	15.269444	Primary	GBIF: https://doi.org/10.15468/dl.zfaw79
Anopheles albimanus	-92.888055	15.275278	Primary	GBIF: https://doi.org/10.15468/dl.zfaw79
Anopheles albimanus	-92.883333	15.283333	Primary	GBIF: https://doi.org/10.15468/dl.zfaw79
Anopheles albimanus	-92.883333	15.195278	Primary	GBIF: https://doi.org/10.15468/dl.zfaw79
Anopheles albimanus	-92.883055	15.2	Primary	GBIF: https://doi.org/10.15468/dl.zfaw79
Anopheles albimanus	-92.866667	15.327778	Primary	GBIF: https://doi.org/10.15468/dl.zfaw79
Anopheles albimanus	-92.858333	15.258333	Primary	GBIF: https://doi.org/10.15468/dl.zfaw79
Anopheles albimanus	-92.843889	15.178333	Primary	GBIF: https://doi.org/10.15468/dl.zfaw79
Anopheles albimanus	-92.842139	15.177111	Primary	GBIF: https://doi.org/10.15468/dl.zfaw79
Anopheles albimanus	-92.841667	15.275	Primary	GBIF: https://doi.org/10.15468/dl.zfaw79
Anopheles albimanus	-92.840611	15.175694	Primary	GBIF: https://doi.org/10.15468/dl.zfaw79
Anopheles albimanus	-92.84	15.176111	Primary	GBIF: https://doi.org/10.15468/dl.zfaw79
Anopheles albimanus	-92.839944	15.176472	Primary	GBIF: https://doi.org/10.15468/dl.zfaw79
Anopheles albimanus	-92.839722	15.176389	Primary	GBIF: https://doi.org/10.15468/dl.zfaw79
Anopheles albimanus	-92.838889	15.176667	Primary	GBIF: https://doi.org/10.15468/dl.zfaw79
Anopheles albimanus	-92.837778	15.166389	Primary	GBIF: https://doi.org/10.15468/dl.zfaw79
Anopheles albimanus	-92.808333	15.258333	Primary	GBIF: https://doi.org/10.15468/dl.zfaw79
Anopheles albimanus	-92.791667	15.258333	Primary	GBIF: https://doi.org/10.15468/dl.zfaw79
Anopheles albimanus	-92.758333	15.191667	Primary	GBIF: https://doi.org/10.15468/dl.zfaw79
Anopheles albimanus	-92.75	15.175833	Primary	GBIF: https://doi.org/10.15468/dl.zfaw79
Anopheles albimanus	-92.727778	15.157222	Primary	GBIF: https://doi.org/10.15468/dl.zfaw79
Anopheles albimanus	-92.727778	15.151944	Primary	GBIF: https://doi.org/10.15468/dl.zfaw79
Anopheles albimanus	-92.726833	15.15225	Primary	GBIF: https://doi.org/10.15468/dl.zfaw79
Anopheles albimanus	-92.72275	15.156444	Primary	GBIF: https://doi.org/10.15468/dl.zfaw79
Anopheles albimanus	-92.722222	15.152778	Primary	GBIF: https://doi.org/10.15468/dl.zfaw79
Anopheles albimanus	-92.720555	15.151389	Primary	GBIF: https://doi.org/10.15468/dl.zfaw79
Anopheles albimanus	-92.719722	15.157222	Primary	GBIF: https://doi.org/10.15468/dl.zfaw79
Anopheles albimanus	-92.719444	15.156944	Primary	GBIF: https://doi.org/10.15468/dl.zfaw79
Anopheles albimanus	-92.719444	15.153333	Primary	GBIF: https://doi.org/10.15468/dl.zfaw79
Anopheles albimanus	-92.719444	15.157778	Primary	GBIF: https://doi.org/10.15468/dl.zfaw79
Anopheles albimanus	-92.719444	15.133333	Primary	GBIF: https://doi.org/10.15468/dl.zfaw79
Anopheles albimanus	-92.719444	15.157222	Primary	GBIF: https://doi.org/10.15468/dl.zfaw79
Anopheles albimanus	-92.718611	15.158333	Primary	GBIF: https://doi.org/10.15468/dl.zfaw79
Anopheles albimanus	-92.714889	17.201278	Primary	GBIF: https://doi.org/10.15468/dl.zfaw79

Anopheles albimanus	-92.702778	15.144444	Primary	GBIF: https://doi.org/10.15468/dl.zfaw79
Anopheles albimanus	-92.701944	15.144167	Primary	GBIF: https://doi.org/10.15468/dl.zfaw79
Anopheles albimanus	-92.7	15.172222	Primary	GBIF: https://doi.org/10.15468/dl.zfaw79
Anopheles albimanus	-92.699167	15.173889	Primary	GBIF: https://doi.org/10.15468/dl.zfaw79
Anopheles albimanus	-92.698611	15.173611	Primary	GBIF: https://doi.org/10.15468/dl.zfaw79
Anopheles albimanus	-92.690555	15.190556	Primary	GBIF: https://doi.org/10.15468/dl.zfaw79
Anopheles albimanus	-92.686111	15.198611	Primary	GBIF: https://doi.org/10.15468/dl.zfaw79
Anopheles albimanus	-92.676667	15.116389	Primary	GBIF: https://doi.org/10.15468/dl.zfaw79
Anopheles albimanus	-92.672222	15.133333	Primary	GBIF: https://doi.org/10.15468/dl.zfaw79
Anopheles albimanus	-92.6625	14.95	Primary	GBIF: https://doi.org/10.15468/dl.zfaw79
Anopheles albimanus	-92.65	18.41	Primary	GBIF: https://doi.org/10.15468/dl.zfaw79
Anopheles albimanus	-92.6479	18.4073	Primary	GBIF: https://doi.org/10.15468/dl.zfaw79
Anopheles albimanus	-92.641667	15.091667	Primary	GBIF: https://doi.org/10.15468/dl.zfaw79
Anopheles albimanus	-92.638889	15.091667	Primary	GBIF: https://doi.org/10.15468/dl.zfaw79
Anopheles albimanus	-92.6225	14.916389	Primary	GBIF: https://doi.org/10.15468/dl.zfaw79
Anopheles albimanus	-92.606667	15.066667	Primary	GBIF: https://doi.org/10.15468/dl.zfaw79
Anopheles albimanus	-92.593056	15.06	Primary	GBIF: https://doi.org/10.15468/dl.zfaw79
Anopheles albimanus	-92.593055	15.116667	Primary	GBIF: https://doi.org/10.15468/dl.zfaw79
Anopheles albimanus	-92.5925	15.064722	Primary	GBIF: https://doi.org/10.15468/dl.zfaw79
Anopheles albimanus	-92.588889	15.061111	Primary	GBIF: https://doi.org/10.15468/dl.zfaw79
Anopheles albimanus	-92.587778	15.061667	Primary	GBIF: https://doi.org/10.15468/dl.zfaw79
Anopheles albimanus	-92.568055	15.055833	Primary	GBIF: https://doi.org/10.15468/dl.zfaw79
Anopheles albimanus	-92.566667	15.058333	Primary	GBIF: https://doi.org/10.15468/dl.zfaw79
Anopheles albimanus	-92.563889	15.05	Primary	GBIF: https://doi.org/10.15468/dl.zfaw79
Anopheles albimanus	-92.562222	15.048611	Primary	GBIF: https://doi.org/10.15468/dl.zfaw79
Anopheles albimanus	-92.551111	15.018333	Primary	GBIF: https://doi.org/10.15468/dl.zfaw79
Anopheles albimanus	-92.547222	15.027778	Primary	GBIF: https://doi.org/10.15468/dl.zfaw79
Anopheles albimanus	-92.54	14.832778	Primary	GBIF: https://doi.org/10.15468/dl.zfaw79
Anopheles albimanus	-92.538889	15.022222	Primary	GBIF: https://doi.org/10.15468/dl.zfaw79
Anopheles albimanus	-92.538055	15.043056	Primary	GBIF: https://doi.org/10.15468/dl.zfaw79
Anopheles albimanus	-92.533333	15.041667	Primary	GBIF: https://doi.org/10.15468/dl.zfaw79
Anopheles albimanus	-92.533333	14.916667	Primary	GBIF: https://doi.org/10.15468/dl.zfaw79
Anopheles albimanus	-92.533333	14.819167	Primary	GBIF: https://doi.org/10.15468/dl.zfaw79
Anopheles albimanus	-92.5325	15.044444	Primary	GBIF: https://doi.org/10.15468/dl.zfaw79
Anopheles albimanus	-92.519722	14.851667	Primary	GBIF: https://doi.org/10.15468/dl.zfaw79
Anopheles albimanus	-92.509722	15.031389	Primary	GBIF: https://doi.org/10.15468/dl.zfaw79
Anopheles albimanus	-92.509167	14.8	Primary	GBIF: https://doi.org/10.15468/dl.zfaw79
Anopheles albimanus	-92.506944	14.797222	Primary	GBIF: https://doi.org/10.15468/dl.zfaw79
Anopheles albimanus	-92.496389	14.817778	Primary	GBIF: https://doi.org/10.15468/dl.zfaw79
Anopheles albimanus	-92.495	14.861944	Primary	GBIF: https://doi.org/10.15468/dl.zfaw79
Anopheles albimanus	-92.491667	14.791667	Primary	GBIF: https://doi.org/10.15468/dl.zfaw79
Anopheles albimanus	-92.491667	15.125	Primary	GBIF: https://doi.org/10.15468/dl.zfaw79

Anopheles albimanus	-92.491667	14.808333	Primary	GBIF: https://doi.org/10.15468/dl.zfaw79
Anopheles albimanus	-92.491111	14.791389	Primary	GBIF: https://doi.org/10.15468/dl.zfaw79
Anopheles albimanus	-92.481111	14.811667	Primary	GBIF: https://doi.org/10.15468/dl.zfaw79
Anopheles albimanus	-92.480833	14.814722	Primary	GBIF: https://doi.org/10.15468/dl.zfaw79
Anopheles albimanus	-92.480833	14.820556	Primary	GBIF: https://doi.org/10.15468/dl.zfaw79
Anopheles albimanus	-92.475833	15.035556	Primary	GBIF: https://doi.org/10.15468/dl.zfaw79
Anopheles albimanus	-92.475	14.791667	Primary	GBIF: https://doi.org/10.15468/dl.zfaw79
Anopheles albimanus	-92.469444	14.834444	Primary	GBIF: https://doi.org/10.15468/dl.zfaw79
Anopheles albimanus	-92.458333	14.758333	Primary	GBIF: https://doi.org/10.15468/dl.zfaw79
Anopheles albimanus	-92.449444	15.046944	Primary	GBIF: https://doi.org/10.15468/dl.zfaw79
Anopheles albimanus	-92.443333	14.925833	Primary	GBIF: https://doi.org/10.15468/dl.zfaw79
Anopheles albimanus	-92.443055	14.78	Primary	GBIF: https://doi.org/10.15468/dl.zfaw79
Anopheles albimanus	-92.441667	14.791667	Primary	GBIF: https://doi.org/10.15468/dl.zfaw79
Anopheles albimanus	-92.440555	14.731667	Primary	GBIF: https://doi.org/10.15468/dl.zfaw79
Anopheles albimanus	-92.425555	14.719167	Primary	GBIF: https://doi.org/10.15468/dl.zfaw79
Anopheles albimanus	-92.422222	14.719167	Primary	GBIF: https://doi.org/10.15468/dl.zfaw79
Anopheles albimanus	-92.411944	15.035833	Primary	GBIF: https://doi.org/10.15468/dl.zfaw79
Anopheles albimanus	-92.400194	14.784722	Primary	GBIF: https://doi.org/10.15468/dl.zfaw79
Anopheles albimanus	-92.4	14.783611	Primary	GBIF: https://doi.org/10.15468/dl.zfaw79
Anopheles albimanus	-92.391667	14.908333	Primary	GBIF: https://doi.org/10.15468/dl.zfaw79
Anopheles albimanus	-92.364722	14.827778	Primary	GBIF: https://doi.org/10.15468/dl.zfaw79
Anopheles albimanus	-92.363889	14.8275	Primary	GBIF: https://doi.org/10.15468/dl.zfaw79
Anopheles albimanus	-92.3625	14.828889	Primary	GBIF: https://doi.org/10.15468/dl.zfaw79
Anopheles albimanus	-92.341667	14.841667	Primary	GBIF: https://doi.org/10.15468/dl.zfaw79
Anopheles albimanus	-92.333889	17.555556	Primary	GBIF: https://doi.org/10.15468/dl.zfaw79
Anopheles albimanus	-92.328055	14.668333	Primary	GBIF: https://doi.org/10.15468/dl.zfaw79
Anopheles albimanus	-92.325	14.625	Primary	GBIF: https://doi.org/10.15468/dl.zfaw79
Anopheles albimanus	-92.325	14.708333	Primary	GBIF: https://doi.org/10.15468/dl.zfaw79
Anopheles albimanus	-92.314444	14.6275	Primary	GBIF: https://doi.org/10.15468/dl.zfaw79
Anopheles albimanus	-92.314305	14.627139	Primary	GBIF: https://doi.org/10.15468/dl.zfaw79
Anopheles albimanus	-92.308333	14.625	Primary	GBIF: https://doi.org/10.15468/dl.zfaw79
Anopheles albimanus	-92.303333	14.638889	Primary	GBIF: https://doi.org/10.15468/dl.zfaw79
Anopheles albimanus	-92.3025	14.638333	Primary	GBIF: https://doi.org/10.15468/dl.zfaw79
Anopheles albimanus	-92.295278	14.64	Primary	GBIF: https://doi.org/10.15468/dl.zfaw79
Anopheles albimanus	-92.284722	14.724167	Primary	GBIF: https://doi.org/10.15468/dl.zfaw79
Anopheles albimanus	-92.2825	14.590556	Primary	GBIF: https://doi.org/10.15468/dl.zfaw79
Anopheles albimanus	-92.281667	14.625	Primary	GBIF: https://doi.org/10.15468/dl.zfaw79
Anopheles albimanus	-92.275	14.925	Primary	GBIF: https://doi.org/10.15468/dl.zfaw79
Anopheles albimanus	-92.275	14.908333	Primary	GBIF: https://doi.org/10.15468/dl.zfaw79
Anopheles albimanus	-92.275	14.758333	Primary	GBIF: https://doi.org/10.15468/dl.zfaw79
Anopheles albimanus	-92.270555	14.625	Primary	GBIF: https://doi.org/10.15468/dl.zfaw79
Anopheles albimanus	-92.26611	14.9	Primary	GBIF: https://doi.org/10.15468/dl.zfaw79

Anopheles albimanus	-92.265555	14.635556	Primary	GBIF: https://doi.org/10.15468/dl.zfaw79
Anopheles albimanus	-92.260556	14.908056	Primary	GBIF: https://doi.org/10.15468/dl.zfaw79
Anopheles albimanus	-92.260555	14.908056	Primary	GBIF: https://doi.org/10.15468/dl.zfaw79
Anopheles albimanus	-92.260555	14.614722	Primary	GBIF: https://doi.org/10.15468/dl.zfaw79
Anopheles albimanus	-92.258611	14.628333	Primary	GBIF: https://doi.org/10.15468/dl.zfaw79
Anopheles albimanus	-92.258333	14.615278	Primary	GBIF: https://doi.org/10.15468/dl.zfaw79
Anopheles albimanus	-92.257778	14.580556	Primary	GBIF: https://doi.org/10.15468/dl.zfaw79
Anopheles albimanus	-92.257778	14.625278	Primary	GBIF: https://doi.org/10.15468/dl.zfaw79
Anopheles albimanus	-92.256305	14.6285	Primary	GBIF: https://doi.org/10.15468/dl.zfaw79
Anopheles albimanus	-92.255278	14.563056	Primary	GBIF: https://doi.org/10.15468/dl.zfaw79
Anopheles albimanus	-92.253611	14.6275	Primary	GBIF: https://doi.org/10.15468/dl.zfaw79
Anopheles albimanus	-92.249722	14.636944	Primary	GBIF: https://doi.org/10.15468/dl.zfaw79
Anopheles albimanus	-92.248889	14.563889	Primary	GBIF: https://doi.org/10.15468/dl.zfaw79
Anopheles albimanus	-92.241667	14.625	Primary	GBIF: https://doi.org/10.15468/dl.zfaw79
Anopheles albimanus	-92.236667	14.643056	Primary	GBIF: https://doi.org/10.15468/dl.zfaw79
Anopheles albimanus	-92.228889	14.603333	Primary	GBIF: https://doi.org/10.15468/dl.zfaw79
Anopheles albimanus	-92.226944	15.050278	Primary	GBIF: https://doi.org/10.15468/dl.zfaw79
Anopheles albimanus	-92.225	14.575	Primary	GBIF: https://doi.org/10.15468/dl.zfaw79
Anopheles albimanus	-92.223055	14.535556	Primary	GBIF: https://doi.org/10.15468/dl.zfaw79
Anopheles albimanus	-92.2175	14.598056	Primary	GBIF: https://doi.org/10.15468/dl.zfaw79
Anopheles albimanus	-92.216111	14.559167	Primary	GBIF: https://doi.org/10.15468/dl.zfaw79
Anopheles albimanus	-92.210278	14.681111	Primary	GBIF: https://doi.org/10.15468/dl.zfaw79
Anopheles albimanus	-92.208889	15.095833	Primary	GBIF: https://doi.org/10.15468/dl.zfaw79
Anopheles albimanus	-92.203333	14.703056	Primary	GBIF: https://doi.org/10.15468/dl.zfaw79
Anopheles albimanus	-92.2025	14.562222	Primary	GBIF: https://doi.org/10.15468/dl.zfaw79
Anopheles albimanus	-92.202222	14.566389	Primary	GBIF: https://doi.org/10.15468/dl.zfaw79
Anopheles albimanus	-92.200833	14.723611	Primary	GBIF: https://doi.org/10.15468/dl.zfaw79
Anopheles albimanus	-92.1925	14.591111	Primary	GBIF: https://doi.org/10.15468/dl.zfaw79
Anopheles albimanus	-92.186667	14.621389	Primary	GBIF: https://doi.org/10.15468/dl.zfaw79
Anopheles albimanus	-92.175278	14.64	Primary	GBIF: https://doi.org/10.15468/dl.zfaw79
Anopheles albimanus	-92.175	14.676111	Primary	GBIF: https://doi.org/10.15468/dl.zfaw79
Anopheles albimanus	-92.17194	15.3925	Primary	GBIF: https://doi.org/10.15468/dl.zfaw79
Anopheles albimanus	-92.17	15.39	Primary	GBIF: https://doi.org/10.15468/dl.zfaw79
Anopheles albimanus	-92.162222	15.4525	Primary	GBIF: https://doi.org/10.15468/dl.zfaw79
Anopheles albimanus	-92.150833	15.094444	Primary	GBIF: https://doi.org/10.15468/dl.zfaw79
Anopheles albimanus	-92.02	17.498611	Primary	GBIF: https://doi.org/10.15468/dl.zfaw79
Anopheles albimanus	-92.019222	17.7005	Primary	GBIF: https://doi.org/10.15468/dl.zfaw79
Anopheles albimanus	-92.016389	17.702806	Primary	GBIF: https://doi.org/10.15468/dl.zfaw79
Anopheles albimanus	-91.9575	16.943611	Primary	GBIF: https://doi.org/10.15468/dl.zfaw79
Anopheles albimanus	-91.906	14.30017	Primary	GBIF: https://doi.org/10.15468/dl.zfaw79
Anopheles albimanus	-91.899167	16.956111	Primary	GBIF: https://doi.org/10.15468/dl.zfaw79
Anopheles albimanus	-91.830833	18.643333	Primary	GBIF: https://doi.org/10.15468/dl.zfaw79

Anopheles albimanus	-91.62925	16.097361	Primary	GBIF: https://doi.org/10.15468/dl.zfaw79
Anopheles albimanus	-91.628889	16.096667	Primary	GBIF: https://doi.org/10.15468/dl.zfaw79
Anopheles albimanus	-91.426389	17.470833	Primary	GBIF: https://doi.org/10.15468/dl.zfaw79
Anopheles albimanus	-91.362778	18.184444	Primary	GBIF: https://doi.org/10.15468/dl.zfaw79
Anopheles albimanus	-91.21106	16.309	Primary	GBIF: https://doi.org/10.15468/dl.zfaw79
Anopheles albimanus	-91.21	16.31	Primary	GBIF: https://doi.org/10.15468/dl.zfaw79
Anopheles albimanus	-91.155555	16.120278	Primary	GBIF: https://doi.org/10.15468/dl.zfaw79
Anopheles albimanus	-91.148889	16.1375	Primary	GBIF: https://doi.org/10.15468/dl.zfaw79
Anopheles albimanus	-91.099722	16.0785	Primary	GBIF: https://doi.org/10.15468/dl.zfaw79
Anopheles albimanus	-91.096111	16.088889	Primary	GBIF: https://doi.org/10.15468/dl.zfaw79
Anopheles albimanus	-91.094555	16.081639	Primary	GBIF: https://doi.org/10.15468/dl.zfaw79
Anopheles albimanus	-91.094444	16.081111	Primary	GBIF: https://doi.org/10.15468/dl.zfaw79
Anopheles albimanus	-91.094444	16.0875	Primary	GBIF: https://doi.org/10.15468/dl.zfaw79
Anopheles albimanus	-91.094111	16.081694	Primary	GBIF: https://doi.org/10.15468/dl.zfaw79
Anopheles albimanus	-90.998	16.762583	Primary	GBIF: https://doi.org/10.15468/dl.zfaw79
Anopheles albimanus	-90.99175	16.763611	Primary	GBIF: https://doi.org/10.15468/dl.zfaw79
Anopheles albimanus	-90.970833	16.097222	Primary	GBIF: https://doi.org/10.15468/dl.zfaw79
Anopheles albimanus	-90.913	16.801694	Primary	GBIF: https://doi.org/10.15468/dl.zfaw79
Anopheles albimanus	-90.9025	16.804444	Primary	GBIF: https://doi.org/10.15468/dl.zfaw79
Anopheles albimanus	-90.890278	16.821111	Primary	GBIF: https://doi.org/10.15468/dl.zfaw79
Anopheles albimanus	-90.874167	16.117778	Primary	GBIF: https://doi.org/10.15468/dl.zfaw79
Anopheles albimanus	-90.867778	16.207222	Primary	GBIF: https://doi.org/10.15468/dl.zfaw79
Anopheles albimanus	-90.764167	16.335	Primary	GBIF: https://doi.org/10.15468/dl.zfaw79
Anopheles albimanus	-90.734444	18.606667	Primary	GBIF: https://doi.org/10.15468/dl.zfaw79
Anopheles albimanus	-90.720555	19.354722	Primary	GBIF: https://doi.org/10.15468/dl.zfaw79
Anopheles albimanus	-90.706944	16.410833	Primary	GBIF: https://doi.org/10.15468/dl.zfaw79
Anopheles albimanus	-90.702611	16.404139	Primary	GBIF: https://doi.org/10.15468/dl.zfaw79
Anopheles albimanus	-90.701111	19.502222	Primary	GBIF: https://doi.org/10.15468/dl.zfaw79
Anopheles albimanus	-90.687778	19.638333	Primary	GBIF: https://doi.org/10.15468/dl.zfaw79
Anopheles albimanus	-90.678833	16.554778	Primary	GBIF: https://doi.org/10.15468/dl.zfaw79
Anopheles albimanus	-90.653889	16.515	Primary	GBIF: https://doi.org/10.15468/dl.zfaw79
Anopheles albimanus	-90.650555	16.518889	Primary	GBIF: https://doi.org/10.15468/dl.zfaw79
Anopheles albimanus	-90.6425	16.471111	Primary	GBIF: https://doi.org/10.15468/dl.zfaw79
Anopheles albimanus	-90.621805	16.454806	Primary	GBIF: https://doi.org/10.15468/dl.zfaw79
Anopheles albimanus	-90.615139	16.449917	Primary	GBIF: https://doi.org/10.15468/dl.zfaw79
Anopheles albimanus	-90.614167	16.448333	Primary	GBIF: https://doi.org/10.15468/dl.zfaw79
Anopheles albimanus	-90.535833	16.269167	Primary	GBIF: https://doi.org/10.15468/dl.zfaw79
Anopheles albimanus	-90.535781	19.839924	Primary	GBIF: https://doi.org/10.15468/dl.zfaw79
Anopheles albimanus	-90.531667	19.842222	Primary	GBIF: https://doi.org/10.15468/dl.zfaw79
Anopheles albimanus	-90.450472	20.70875	Primary	GBIF: https://doi.org/10.15468/dl.zfaw79
Anopheles albimanus	-90.383333	20.858333	Primary	GBIF: https://doi.org/10.15468/dl.zfaw79
Anopheles albimanus	-90.380278	20.912778	Primary	GBIF: https://doi.org/10.15468/dl.zfaw79

Anopheles albimanus	-90.355	20.977222	Primary	GBIF: https://doi.org/10.15468/dl.zfaw79
Anopheles albimanus	-90.30001	16.53417	Primary	GBIF: https://doi.org/10.15468/dl.zfaw79
Anopheles albimanus	-90.261111	20.848889	Primary	GBIF: https://doi.org/10.15468/dl.zfaw79
Anopheles albimanus	-89.88333	16.91748	Primary	GBIF: https://doi.org/10.15468/dl.zfaw79
Anopheles albimanus	-89.66678	21.28391	Primary	GBIF: https://doi.org/10.15468/dl.zfaw79
Anopheles albimanus	-89.663333	21.284444	Primary	GBIF: https://doi.org/10.15468/dl.zfaw79
Anopheles albimanus	-89.621667	20.967778	Primary	GBIF: https://doi.org/10.15468/dl.zfaw79
Anopheles albimanus	-89.417778	18.588611	Primary	GBIF: https://doi.org/10.15468/dl.zfaw79
Anopheles albimanus	-89.376583	18.803861	Primary	GBIF: https://doi.org/10.15468/dl.zfaw79
Anopheles albimanus	-89.105889	18.521861	Primary	GBIF: https://doi.org/10.15468/dl.zfaw79
Anopheles albimanus	-89.09998	16.21752	Primary	GBIF: https://doi.org/10.15468/dl.zfaw79
Anopheles albimanus	-89.09998	13.70098	Primary	GBIF: https://doi.org/10.15468/dl.zfaw79
Anopheles albimanus	-89.08331	16.15085	Primary	GBIF: https://doi.org/10.15468/dl.zfaw79
Anopheles albimanus	-89.08331	16.25085	Primary	GBIF: https://doi.org/10.15468/dl.zfaw79
Anopheles albimanus	-89.06814	17.15798	Primary	GBIF: https://doi.org/10.15468/dl.zfaw79
Anopheles albimanus	-89.06665	16.16752	Primary	GBIF: https://doi.org/10.15468/dl.zfaw79
Anopheles albimanus	-89.06665	17.33412	Primary	GBIF: https://doi.org/10.15468/dl.zfaw79
Anopheles albimanus	-89.04998	17.26746	Primary	GBIF: https://doi.org/10.15468/dl.zfaw79
Anopheles albimanus	-89.00265	17.18575	Primary	GBIF: https://doi.org/10.15468/dl.zfaw79
Anopheles albimanus	-88.99998	16.21752	Primary	GBIF: https://doi.org/10.15468/dl.zfaw79
Anopheles albimanus	-88.98331	16.26751	Primary	GBIF: https://doi.org/10.15468/dl.zfaw79
Anopheles albimanus	-88.97432	15.29757	Primary	GBIF: https://doi.org/10.15468/dl.zfaw79
Anopheles albimanus	-88.96665	17.11747	Primary	GBIF: https://doi.org/10.15468/dl.zfaw79
Anopheles albimanus	-88.94998	16.23418	Primary	GBIF: https://doi.org/10.15468/dl.zfaw79
Anopheles albimanus	-88.94998	16.10086	Primary	GBIF: https://doi.org/10.15468/dl.zfaw79
Anopheles albimanus	-88.93331	16.23418	Primary	GBIF: https://doi.org/10.15468/dl.zfaw79
Anopheles albimanus	-88.93331	16.20085	Primary	GBIF: https://doi.org/10.15468/dl.zfaw79
Anopheles albimanus	-88.93331	16.16752	Primary	GBIF: https://doi.org/10.15468/dl.zfaw79
Anopheles albimanus	-88.929583	18.465	Primary	GBIF: https://doi.org/10.15468/dl.zfaw79
Anopheles albimanus	-88.92835	15.36123	Primary	GBIF: https://doi.org/10.15468/dl.zfaw79
Anopheles albimanus	-88.91664	16.18418	Primary	GBIF: https://doi.org/10.15468/dl.zfaw79
Anopheles albimanus	-88.90989	15.37946	Primary	GBIF: https://doi.org/10.15468/dl.zfaw79
Anopheles albimanus	-88.89998	16.23418	Primary	GBIF: https://doi.org/10.15468/dl.zfaw79
Anopheles albimanus	-88.88331	16.28418	Primary	GBIF: https://doi.org/10.15468/dl.zfaw79
Anopheles albimanus	-88.88331	16.25085	Primary	GBIF: https://doi.org/10.15468/dl.zfaw79
Anopheles albimanus	-88.88331	16.26751	Primary	GBIF: https://doi.org/10.15468/dl.zfaw79
Anopheles albimanus	-88.84998	17.06747	Primary	GBIF: https://doi.org/10.15468/dl.zfaw79
Anopheles albimanus	-88.83442	17.33523	Primary	GBIF: https://doi.org/10.15468/dl.zfaw79
Anopheles albimanus	-88.83331	17.50078	Primary	GBIF: https://doi.org/10.15468/dl.zfaw79
Anopheles albimanus	-88.83331	16.15085	Primary	GBIF: https://doi.org/10.15468/dl.zfaw79
Anopheles albimanus	-88.83331	16.13419	Primary	GBIF: https://doi.org/10.15468/dl.zfaw79
Anopheles albimanus	-88.81664	16.10086	Primary	GBIF: https://doi.org/10.15468/dl.zfaw79

Anopheles albimanus	-88.79998	16.16752	Primary	GBIF: https://doi.org/10.15468/dl.zfaw79
Anopheles albimanus	-88.79998	17.25079	Primary	GBIF: https://doi.org/10.15468/dl.zfaw79
Anopheles albimanus	-88.79998	16.11752	Primary	GBIF: https://doi.org/10.15468/dl.zfaw79
Anopheles albimanus	-88.74754	13.6907	Primary	GBIF: https://doi.org/10.15468/dl.zfaw79
Anopheles albimanus	-88.71664	17.1508	Primary	GBIF: https://doi.org/10.15468/dl.zfaw79
Anopheles albimanus	-88.64997	18.18408	Primary	GBIF: https://doi.org/10.15468/dl.zfaw79
Anopheles albimanus	-88.64997	17.1008	Primary	GBIF: https://doi.org/10.15468/dl.zfaw79
Anopheles albimanus	-88.64997	17.08414	Primary	GBIF: https://doi.org/10.15468/dl.zfaw79
Anopheles albimanus	-88.64997	18.16741	Primary	GBIF: https://doi.org/10.15468/dl.zfaw79
Anopheles albimanus	-88.61664	13.23434	Primary	GBIF: https://doi.org/10.15468/dl.zfaw79
Anopheles albimanus	-88.61664	17.1508	Primary	GBIF: https://doi.org/10.15468/dl.zfaw79
Anopheles albimanus	-88.59997	15.71754	Primary	GBIF: https://doi.org/10.15468/dl.zfaw79
Anopheles albimanus	-88.5833	17.51745	Primary	GBIF: https://doi.org/10.15468/dl.zfaw79
Anopheles albimanus	-88.554167	18.384722	Primary	GBIF: https://doi.org/10.15468/dl.zfaw79
Anopheles albimanus	-88.54853	15.63533	Primary	GBIF: https://doi.org/10.15468/dl.zfaw79
Anopheles albimanus	-88.5333	17.75077	Primary	GBIF: https://doi.org/10.15468/dl.zfaw79
Anopheles albimanus	-88.531667	18.419722	Primary	GBIF: https://doi.org/10.15468/dl.zfaw79
Anopheles albimanus	-88.529	18.424	Primary	GBIF: https://doi.org/10.15468/dl.zfaw79
Anopheles albimanus	-88.524	18.445	Primary	GBIF: https://doi.org/10.15468/dl.zfaw79
Anopheles albimanus	-88.518056	18.463694	Primary	GBIF: https://doi.org/10.15468/dl.zfaw79
Anopheles albimanus	-88.518	18.462	Primary	GBIF: https://doi.org/10.15468/dl.zfaw79
Anopheles albimanus	-88.51664	17.55078	Primary	GBIF: https://doi.org/10.15468/dl.zfaw79
Anopheles albimanus	-88.516	18.464	Primary	GBIF: https://doi.org/10.15468/dl.zfaw79
Anopheles albimanus	-88.478889	18.496111	Primary	GBIF: https://doi.org/10.15468/dl.zfaw79
Anopheles albimanus	-88.465278	18.551944	Primary	GBIF: https://doi.org/10.15468/dl.zfaw79
Anopheles albimanus	-88.44997	18.43407	Primary	GBIF: https://doi.org/10.15468/dl.zfaw79
Anopheles albimanus	-88.4333	17.38412	Primary	GBIF: https://doi.org/10.15468/dl.zfaw79
Anopheles albimanus	-88.4333	18.43407	Primary	GBIF: https://doi.org/10.15468/dl.zfaw79
Anopheles albimanus	-88.41663	17.51745	Primary	GBIF: https://doi.org/10.15468/dl.zfaw79
Anopheles albimanus	-88.41663	17.48412	Primary	GBIF: https://doi.org/10.15468/dl.zfaw79
Anopheles albimanus	-88.39997	17.46745	Primary	GBIF: https://doi.org/10.15468/dl.zfaw79
Anopheles albimanus	-88.39997	18.4674	Primary	GBIF: https://doi.org/10.15468/dl.zfaw79
Anopheles albimanus	-88.39997	17.48412	Primary	GBIF: https://doi.org/10.15468/dl.zfaw79
Anopheles albimanus	-88.3833	17.63411	Primary	GBIF: https://doi.org/10.15468/dl.zfaw79
Anopheles albimanus	-88.36519	17.42603	Primary	GBIF: https://doi.org/10.15468/dl.zfaw79
Anopheles albimanus	-88.34997	17.48412	Primary	GBIF: https://doi.org/10.15468/dl.zfaw79
Anopheles albimanus	-88.32	18.507	Primary	GBIF: https://doi.org/10.15468/dl.zfaw79
Anopheles albimanus	-88.32	18.51	Primary	GBIF: https://doi.org/10.15468/dl.zfaw79
Anopheles albimanus	-88.31663	17.8341	Primary	GBIF: https://doi.org/10.15468/dl.zfaw79
Anopheles albimanus	-88.31663	17.48412	Primary	GBIF: https://doi.org/10.15468/dl.zfaw79
Anopheles albimanus	-88.315	18.496	Primary	GBIF: https://doi.org/10.15468/dl.zfaw79
Anopheles albimanus	-88.31	18.5	Primary	GBIF: https://doi.org/10.15468/dl.zfaw79

Anopheles albimanus	-88.305278	18.503611	Primary	GBIF: https://doi.org/10.15468/dl.zfaw79
Anopheles albimanus	-88.266667	18.6875	Primary	GBIF: https://doi.org/10.15468/dl.zfaw79
Anopheles albimanus	-88.253333	18.709722	Primary	GBIF: https://doi.org/10.15468/dl.zfaw79
Anopheles albimanus	-88.24996	17.48412	Primary	GBIF: https://doi.org/10.15468/dl.zfaw79
Anopheles albimanus	-88.24996	17.58411	Primary	GBIF: https://doi.org/10.15468/dl.zfaw79
Anopheles albimanus	-88.21663	16.96748	Primary	GBIF: https://doi.org/10.15468/dl.zfaw79
Anopheles albimanus	-88.19996	17.50078	Primary	GBIF: https://doi.org/10.15468/dl.zfaw79
Anopheles albimanus	-88.195139	18.67275	Primary	GBIF: https://doi.org/10.15468/dl.zfaw79
Anopheles albimanus	-88.169167	19.143056	Primary	GBIF: https://doi.org/10.15468/dl.zfaw79
Anopheles albimanus	-88.162778	21.58	Primary	GBIF: https://doi.org/10.15468/dl.zfaw79
Anopheles albimanus	-88.159778	21.573833	Primary	GBIF: https://doi.org/10.15468/dl.zfaw79
Anopheles albimanus	-88.146389	21.596111	Primary	GBIF: https://doi.org/10.15468/dl.zfaw79
Anopheles albimanus	-88.102778	19.162778	Primary	GBIF: https://doi.org/10.15468/dl.zfaw79
Anopheles albimanus	-87.996417	19.491833	Primary	GBIF: https://doi.org/10.15468/dl.zfaw79
Anopheles albimanus	-87.993028	19.49625	Primary	GBIF: https://doi.org/10.15468/dl.zfaw79
Anopheles albimanus	-87.93389	15.85576	Primary	GBIF: https://doi.org/10.15468/dl.zfaw79
Anopheles albimanus	-87.81662	15.3509	Primary	GBIF: https://doi.org/10.15468/dl.zfaw79
Anopheles albimanus	-87.814	32.532	Primary	GBIF: https://doi.org/10.15468/dl.zfaw79
Anopheles albimanus	-87.735111	18.770889	Primary	GBIF: https://doi.org/10.15468/dl.zfaw79
Anopheles albimanus	-87.700555	21.482778	Primary	GBIF: https://doi.org/10.15468/dl.zfaw79
Anopheles albimanus	-87.692778	21.519167	Primary	GBIF: https://doi.org/10.15468/dl.zfaw79
Anopheles albimanus	-87.678611	21.509722	Primary	GBIF: https://doi.org/10.15468/dl.zfaw79
Anopheles albimanus	-87.618055	20.076389	Primary	GBIF: https://doi.org/10.15468/dl.zfaw79
Anopheles albimanus	-87.61555	15.58569	Primary	GBIF: https://doi.org/10.15468/dl.zfaw79
Anopheles albimanus	-87.46675	15.77591	Primary	GBIF: https://doi.org/10.15468/dl.zfaw79
Anopheles albimanus	-87.16661	12.48438	Primary	GBIF: https://doi.org/10.15468/dl.zfaw79
Anopheles albimanus	-87.04994	12.53438	Primary	GBIF: https://doi.org/10.15468/dl.zfaw79
Anopheles albimanus	-86.91671	20.41728	Primary	GBIF: https://doi.org/10.15468/dl.zfaw79
Anopheles albimanus	-86.833611	21.161111	Primary	GBIF: https://doi.org/10.15468/dl.zfaw79
Anopheles albimanus	-86.83327	15.75087	Primary	GBIF: https://doi.org/10.15468/dl.zfaw79
Anopheles albimanus	-86.77012	12.19568	Primary	GBIF: https://doi.org/10.15468/dl.zfaw79
Anopheles albimanus	-86.76093	12.19567	Primary	GBIF: https://doi.org/10.15468/dl.zfaw79
Anopheles albimanus	-86.5666	15.7842	Primary	GBIF: https://doi.org/10.15468/dl.zfaw79
Anopheles albimanus	-86.48	12.38	Primary	GBIF: https://doi.org/10.15468/dl.zfaw79
Anopheles albimanus	-86.33326	12.33439	Primary	GBIF: https://doi.org/10.15468/dl.zfaw79
Anopheles albimanus	-86.28326	12.15107	Primary	GBIF: https://doi.org/10.15468/dl.zfaw79
Anopheles albimanus	-86.28306	12.14058	Primary	GBIF: https://doi.org/10.15468/dl.zfaw79
Anopheles albimanus	-86.27382	12.15865	Primary	GBIF: https://doi.org/10.15468/dl.zfaw79
Anopheles albimanus	-86.09992	12.20107	Primary	GBIF: https://doi.org/10.15468/dl.zfaw79
Anopheles albimanus	-86.09907	12.1943	Primary	GBIF: https://doi.org/10.15468/dl.zfaw79
Anopheles albimanus	-86.01659	16.01753	Primary	GBIF: https://doi.org/10.15468/dl.zfaw79
Anopheles albimanus	-85.99992	15.8842	Primary	GBIF: https://doi.org/10.15468/dl.zfaw79

Anopheles albimanus	-85.96221	11.9316	Primary	GBIF: https://doi.org/10.15468/dl.zfaw79
Anopheles albimanus	-85.94992	11.93441	Primary	GBIF: https://doi.org/10.15468/dl.zfaw79
Anopheles albimanus	-85.94367	11.97674	Primary	GBIF: https://doi.org/10.15468/dl.zfaw79
Anopheles albimanus	-85.46658	10.35117	Primary	GBIF: https://doi.org/10.15468/dl.zfaw79
Anopheles albimanus	-85.44991	10.15118	Primary	GBIF: https://doi.org/10.15468/dl.zfaw79
Anopheles albimanus	-85.44588	10.62735	Primary	GBIF: https://doi.org/10.15468/dl.zfaw79
Anopheles albimanus	-85.35226	10.35029	Primary	GBIF: https://doi.org/10.15468/dl.zfaw79
Anopheles albimanus	-85.34689	10.33754	Primary	GBIF: https://doi.org/10.15468/dl.zfaw79
Anopheles albimanus	-85.33771	10.34563	Primary	GBIF: https://doi.org/10.15468/dl.zfaw79
Anopheles albimanus	-85.24095	10.06538	Primary	GBIF: https://doi.org/10.15468/dl.zfaw79
Anopheles albimanus	-84.7834	9.97263	Primary	GBIF: https://doi.org/10.15468/dl.zfaw79
Anopheles albimanus	-84.62054	9.7998	Primary	GBIF: https://doi.org/10.15468/dl.zfaw79
Anopheles albimanus	-84.24988	12.15107	Primary	GBIF: https://doi.org/10.15468/dl.zfaw79
Anopheles albimanus	-84.21789	10.01357	Primary	GBIF: https://doi.org/10.15468/dl.zfaw79
Anopheles albimanus	-84.15	12.17	Primary	GBIF: https://doi.org/10.15468/dl.zfaw79
Anopheles albimanus	-83.8666	9.25399	Primary	GBIF: https://doi.org/10.15468/dl.zfaw79
Anopheles albimanus	-83.77317	12.01019	Primary	GBIF: https://doi.org/10.15468/dl.zfaw79
Anopheles albimanus	-83.770278	12.011111	Primary	GBIF: https://doi.org/10.15468/dl.zfaw79
Anopheles albimanus	-83.74988	12.00108	Primary	GBIF: https://doi.org/10.15468/dl.zfaw79
Anopheles albimanus	-83.70633	22.630938	Primary	GBIF: https://doi.org/10.15468/dl.zfaw79
Anopheles albimanus	-83.58089	9.83989	Primary	GBIF: https://doi.org/10.15468/dl.zfaw79
Anopheles albimanus	-83.47515	8.89959	Primary	GBIF: https://doi.org/10.15468/dl.zfaw79
Anopheles albimanus	-83.39044	10.08891	Primary	GBIF: https://doi.org/10.15468/dl.zfaw79
Anopheles albimanus	-83.2832	10.08452	Primary	GBIF: https://doi.org/10.15468/dl.zfaw79
Anopheles albimanus	-83.28056	10.02643	Primary	GBIF: https://doi.org/10.15468/dl.zfaw79
Anopheles albimanus	-83.26653	10.03452	Primary	GBIF: https://doi.org/10.15468/dl.zfaw79
Anopheles albimanus	-83.05225	9.97368	Primary	GBIF: https://doi.org/10.15468/dl.zfaw79
Anopheles albimanus	-83.03413	9.99187	Primary	GBIF: https://doi.org/10.15468/dl.zfaw79
Anopheles albimanus	-82.96066	8.82889	Primary	GBIF: https://doi.org/10.15468/dl.zfaw79
Anopheles albimanus	-82.84597	8.49183	Primary	GBIF: https://doi.org/10.15468/dl.zfaw79
Anopheles albimanus	-82.84317	9.74177	Primary	GBIF: https://doi.org/10.15468/dl.zfaw79
Anopheles albimanus	-82.83319	9.73454	Primary	GBIF: https://doi.org/10.15468/dl.zfaw79
Anopheles albimanus	-82.74986	9.65121	Primary	GBIF: https://doi.org/10.15468/dl.zfaw79
Anopheles albimanus	-82.553	27.64	Primary	GBIF: https://doi.org/10.15468/dl.zfaw79
Anopheles albimanus	-82.5527	27.6405	Primary	GBIF: https://doi.org/10.15468/dl.zfaw79
Anopheles albimanus	-82.49989	22.88381	Primary	GBIF: https://doi.org/10.15468/dl.zfaw79
Anopheles albimanus	-82.4837	27.5784	Primary	GBIF: https://doi.org/10.15468/dl.zfaw79
Anopheles albimanus	-82.4249	27.565	Primary	GBIF: https://doi.org/10.15468/dl.zfaw79
Anopheles albimanus	-82.42017	9.29887	Primary	GBIF: https://doi.org/10.15468/dl.zfaw79
Anopheles albimanus	-82.41103	9.28987	Primary	GBIF: https://doi.org/10.15468/dl.zfaw79
Anopheles albimanus	-82.40192	9.2899	Primary	GBIF: https://doi.org/10.15468/dl.zfaw79
Anopheles albimanus	-82.36656	23.1338	Primary	GBIF: https://doi.org/10.15468/dl.zfaw79

Anopheles albimanus	-82.1913	8.91085	Primary	GBIF: https://doi.org/10.15468/dl.zfaw79
Anopheles albimanus	-81.39974	19.36442	Primary	GBIF: https://doi.org/10.15468/dl.zfaw79
Anopheles albimanus	-80.96	24.76	Primary	GBIF: https://doi.org/10.15468/dl.zfaw79
Anopheles albimanus	-80.65	-4.84	Primary	GBIF: https://doi.org/10.15468/dl.zfaw79
Anopheles albimanus	-80.554956	8.230176	Primary	GBIF: https://doi.org/10.15468/dl.zfaw79
Anopheles albimanus	-80.546092	8.241829	Primary	GBIF: https://doi.org/10.15468/dl.zfaw79
Anopheles albimanus	-80.39	-3.54	Primary	GBIF: https://doi.org/10.15468/dl.zfaw79
Anopheles albimanus	-80.20277	8.657308	Primary	GBIF: https://doi.org/10.15468/dl.zfaw79
Anopheles albimanus	-80.08494	-2.2196	Primary	GBIF: https://doi.org/10.15468/dl.zfaw79
Anopheles albimanus	-80	9	Primary	GBIF: https://doi.org/10.15468/dl.zfaw79
Anopheles albimanus	-79.9998	9.31796	Primary	GBIF: https://doi.org/10.15468/dl.zfaw79
Anopheles albimanus	-79.9998	7.46806	Primary	GBIF: https://doi.org/10.15468/dl.zfaw79
Anopheles albimanus	-79.98	32.77	Primary	GBIF: https://doi.org/10.15468/dl.zfaw79
Anopheles albimanus	-79.96228	9.14645	Primary	GBIF: https://doi.org/10.15468/dl.zfaw79
Anopheles albimanus	-79.932222	0.776944	Primary	GBIF: https://doi.org/10.15468/dl.zfaw79
Anopheles albimanus	-79.92	9.27	Primary	GBIF: https://doi.org/10.15468/dl.zfaw79
Anopheles albimanus	-79.918454	9.271745	Primary	GBIF: https://doi.org/10.15468/dl.zfaw79
Anopheles albimanus	-79.916667	9.266667	Primary	GBIF: https://doi.org/10.15468/dl.zfaw79
Anopheles albimanus	-79.91647	9.2013	Primary	GBIF: https://doi.org/10.15468/dl.zfaw79
Anopheles albimanus	-79.91639	9.27292	Primary	GBIF: https://doi.org/10.15468/dl.zfaw79
Anopheles albimanus	-79.8998	9.30129	Primary	GBIF: https://doi.org/10.15468/dl.zfaw79
Anopheles albimanus	-79.89612	-2.20138	Primary	GBIF: https://doi.org/10.15468/dl.zfaw79
Anopheles albimanus	-79.87061	9.35417	Primary	GBIF: https://doi.org/10.15468/dl.zfaw79
Anopheles albimanus	-79.834444	0.876667	Primary	GBIF: https://doi.org/10.15468/dl.zfaw79
Anopheles albimanus	-79.830556	0.877778	Primary	GBIF: https://doi.org/10.15468/dl.zfaw79
Anopheles albimanus	-79.829722	0.877778	Primary	GBIF: https://doi.org/10.15468/dl.zfaw79
Anopheles albimanus	-79.817354	9.037011	Primary	GBIF: https://doi.org/10.15468/dl.zfaw79
Anopheles albimanus	-79.81647	9.03464	Primary	GBIF: https://doi.org/10.15468/dl.zfaw79
Anopheles albimanus	-79.810278	0.8825	Primary	GBIF: https://doi.org/10.15468/dl.zfaw79
Anopheles albimanus	-79.7998	9.01797	Primary	GBIF: https://doi.org/10.15468/dl.zfaw79
Anopheles albimanus	-79.78313	8.88465	Primary	GBIF: https://doi.org/10.15468/dl.zfaw79
Anopheles albimanus	-79.78	8.88	Primary	GBIF: https://doi.org/10.15468/dl.zfaw79
Anopheles albimanus	-79.772129	8.884647	Primary	GBIF: https://doi.org/10.15468/dl.zfaw79
Anopheles albimanus	-79.7667	9.2833	Primary	GBIF: https://doi.org/10.15468/dl.zfaw79
Anopheles albimanus	-79.761713	9.121174	Primary	GBIF: https://doi.org/10.15468/dl.zfaw79
Anopheles albimanus	-79.70758	9.1185	Primary	GBIF: https://doi.org/10.15468/dl.zfaw79
Anopheles albimanus	-79.701944	0.903056	Primary	GBIF: https://doi.org/10.15468/dl.zfaw79
Anopheles albimanus	-79.7	9.12	Primary	GBIF: https://doi.org/10.15468/dl.zfaw79
Anopheles albimanus	-79.698516	9.116392	Primary	GBIF: https://doi.org/10.15468/dl.zfaw79
Anopheles albimanus	-79.69825	9.11665	Primary	GBIF: https://doi.org/10.15468/dl.zfaw79
Anopheles albimanus	-79.698173	9.11707	Primary	GBIF: https://doi.org/10.15468/dl.zfaw79
Anopheles albimanus	-79.698096	9.116642	Primary	GBIF: https://doi.org/10.15468/dl.zfaw79

Anopheles albimanus	-79.69783	9.118426	Primary	GBIF: https://doi.org/10.15468/dl.zfaw79
Anopheles albimanus	-79.697286	9.116358	Primary	GBIF: https://doi.org/10.15468/dl.zfaw79
Anopheles albimanus	-79.697143	9.117409	Primary	GBIF: https://doi.org/10.15468/dl.zfaw79
Anopheles albimanus	-79.696953	9.116524	Primary	GBIF: https://doi.org/10.15468/dl.zfaw79
Anopheles albimanus	-79.68552	-2.13666	Primary	GBIF: https://doi.org/10.15468/dl.zfaw79
Anopheles albimanus	-79.68	-2.1	Primary	GBIF: https://doi.org/10.15468/dl.zfaw79
Anopheles albimanus	-79.66646	9.06797	Primary	GBIF: https://doi.org/10.15468/dl.zfaw79
Anopheles albimanus	-79.66235	9.046	Primary	GBIF: https://doi.org/10.15468/dl.zfaw79
Anopheles albimanus	-79.66185	9.18163	Primary	GBIF: https://doi.org/10.15468/dl.zfaw79
Anopheles albimanus	-79.661111	0.922778	Primary	GBIF: https://doi.org/10.15468/dl.zfaw79
Anopheles albimanus	-79.659167	0.990833	Primary	GBIF: https://doi.org/10.15468/dl.zfaw79
Anopheles albimanus	-79.653056	0.916667	Primary	GBIF: https://doi.org/10.15468/dl.zfaw79
Anopheles albimanus	-79.65282	9.16352	Primary	GBIF: https://doi.org/10.15468/dl.zfaw79
Anopheles albimanus	-79.65275	9.1816	Primary	GBIF: https://doi.org/10.15468/dl.zfaw79
Anopheles albimanus	-79.65132	9.55234	Primary	GBIF: https://doi.org/10.15468/dl.zfaw79
Anopheles albimanus	-79.65	9.166667	Primary	GBIF: https://doi.org/10.15468/dl.zfaw79
Anopheles albimanus	-79.6425	0.948611	Primary	GBIF: https://doi.org/10.15468/dl.zfaw79
Anopheles albimanus	-79.62604	9.02778	Primary	GBIF: https://doi.org/10.15468/dl.zfaw79
Anopheles albimanus	-79.591869	8.997076	Primary	GBIF: https://doi.org/10.15468/dl.zfaw79
Anopheles albimanus	-79.59014	8.90104	Primary	GBIF: https://doi.org/10.15468/dl.zfaw79
Anopheles albimanus	-79.58976	9.00051	Primary	GBIF: https://doi.org/10.15468/dl.zfaw79
Anopheles albimanus	-79.58313	8.91798	Primary	GBIF: https://doi.org/10.15468/dl.zfaw79
Anopheles albimanus	-79.582975	9.001069	Primary	GBIF: https://doi.org/10.15468/dl.zfaw79
Anopheles albimanus	-79.581773	9.001578	Primary	GBIF: https://doi.org/10.15468/dl.zfaw79
Anopheles albimanus	-79.581528	9.001434	Primary	GBIF: https://doi.org/10.15468/dl.zfaw79
Anopheles albimanus	-79.5815	9.001126	Primary	GBIF: https://doi.org/10.15468/dl.zfaw79
Anopheles albimanus	-79.58143	9.000221	Primary	GBIF: https://doi.org/10.15468/dl.zfaw79
Anopheles albimanus	-79.580906	9.003006	Primary	GBIF: https://doi.org/10.15468/dl.zfaw79
Anopheles albimanus	-79.580743	9.000899	Primary	GBIF: https://doi.org/10.15468/dl.zfaw79
Anopheles albimanus	-79.580673	9.000979	Primary	GBIF: https://doi.org/10.15468/dl.zfaw79
Anopheles albimanus	-79.56646	8.98464	Primary	GBIF: https://doi.org/10.15468/dl.zfaw79
Anopheles albimanus	-79.54979	8.96797	Primary	GBIF: https://doi.org/10.15468/dl.zfaw79
Anopheles albimanus	-79.53313	8.96797	Primary	GBIF: https://doi.org/10.15468/dl.zfaw79
Anopheles albimanus	-79.51711	8.9731	Primary	GBIF: https://doi.org/10.15468/dl.zfaw79
Anopheles albimanus	-79.51646	8.98464	Primary	GBIF: https://doi.org/10.15468/dl.zfaw79
Anopheles albimanus	-79.48967	9.00916	Primary	GBIF: https://doi.org/10.15468/dl.zfaw79
Anopheles albimanus	-79.485565	9.00746	Primary	GBIF: https://doi.org/10.15468/dl.zfaw79
Anopheles albimanus	-79.48551	-1.10332	Primary	GBIF: https://doi.org/10.15468/dl.zfaw79
Anopheles albimanus	-79.45314	9.04517	Primary	GBIF: https://doi.org/10.15468/dl.zfaw79
Anopheles albimanus	-79.451373	9.05	Primary	GBIF: https://doi.org/10.15468/dl.zfaw79
Anopheles albimanus	-79.45	-3.42	Primary	GBIF: https://doi.org/10.15468/dl.zfaw79
Anopheles albimanus	-79.44404	9.04513	Primary	GBIF: https://doi.org/10.15468/dl.zfaw79

Anopheles albimanus	-79.42577	9.06314	Primary	GBIF: https://doi.org/10.15468/dl.zfaw79
Anopheles albimanus	-79.396389	-0.0175	Primary	GBIF: https://doi.org/10.15468/dl.zfaw79
Anopheles albimanus	-79.276389	-0.085	Primary	GBIF: https://doi.org/10.15468/dl.zfaw79
Anopheles albimanus	-79.11645	8.25135	Primary	GBIF: https://doi.org/10.15468/dl.zfaw79
Anopheles albimanus	-78.53	-5.64	Primary	GBIF: https://doi.org/10.15468/dl.zfaw79
Anopheles albimanus	-78.08	7.3	Primary	GBIF: https://doi.org/10.15468/dl.zfaw79
Anopheles albimanus	-78.00354	18.11689	Primary	GBIF: https://doi.org/10.15468/dl.zfaw79
Anopheles albimanus	-77.96	7.62	Primary	GBIF: https://doi.org/10.15468/dl.zfaw79
Anopheles albimanus	-77.956656	2.555017	Primary	GBIF: https://doi.org/10.15468/dl.zfaw79
Anopheles albimanus	-77.942977	2.549764	Primary	GBIF: https://doi.org/10.15468/dl.zfaw79
Anopheles albimanus	-77.93998	2.58304	Primary	GBIF: https://doi.org/10.15468/dl.zfaw79
Anopheles albimanus	-77.92985	2.60389	Primary	GBIF: https://doi.org/10.15468/dl.zfaw79
Anopheles albimanus	-77.888367	2.593774	Primary	GBIF: https://doi.org/10.15468/dl.zfaw79
Anopheles albimanus	-77.88505	2.58847	Primary	GBIF: https://doi.org/10.15468/dl.zfaw79
Anopheles albimanus	-77.88444	2.599734	Primary	GBIF: https://doi.org/10.15468/dl.zfaw79
Anopheles albimanus	-77.87693	2.597532	Primary	GBIF: https://doi.org/10.15468/dl.zfaw79
Anopheles albimanus	-77.875698	2.600023	Primary	GBIF: https://doi.org/10.15468/dl.zfaw79
Anopheles albimanus	-77.873733	2.56444	Primary	GBIF: https://doi.org/10.15468/dl.zfaw79
Anopheles albimanus	-77.870214	2.565049	Primary	GBIF: https://doi.org/10.15468/dl.zfaw79
Anopheles albimanus	-77.865815	2.558616	Primary	GBIF: https://doi.org/10.15468/dl.zfaw79
Anopheles albimanus	-77.84975	18.01737	Primary	GBIF: https://doi.org/10.15468/dl.zfaw79
Anopheles albimanus	-77.835088	2.553429	Primary	GBIF: https://doi.org/10.15468/dl.zfaw79
Anopheles albimanus	-77.827116	2.612266	Primary	GBIF: https://doi.org/10.15468/dl.zfaw79
Anopheles albimanus	-77.815819	2.613739	Primary	GBIF: https://doi.org/10.15468/dl.zfaw79
Anopheles albimanus	-77.813952	2.610693	Primary	GBIF: https://doi.org/10.15468/dl.zfaw79
Anopheles albimanus	-77.802114	2.614108	Primary	GBIF: https://doi.org/10.15468/dl.zfaw79
Anopheles albimanus	-77.802086	2.608661	Primary	GBIF: https://doi.org/10.15468/dl.zfaw79
Anopheles albimanus	-77.80153	2.61512	Primary	GBIF: https://doi.org/10.15468/dl.zfaw79
Anopheles albimanus	-77.791958	2.597238	Primary	GBIF: https://doi.org/10.15468/dl.zfaw79
Anopheles albimanus	-77.781658	2.584563	Primary	GBIF: https://doi.org/10.15468/dl.zfaw79
Anopheles albimanus	-77.7628	2.63368	Primary	GBIF: https://doi.org/10.15468/dl.zfaw79
Anopheles albimanus	-77.74969	2.615468	Primary	GBIF: https://doi.org/10.15468/dl.zfaw79
Anopheles albimanus	-77.683833	2.777421	Primary	GBIF: https://doi.org/10.15468/dl.zfaw79
Anopheles albimanus	-77.67683	2.737968	Primary	GBIF: https://doi.org/10.15468/dl.zfaw79
Anopheles albimanus	-77.676548	2.73597	Primary	GBIF: https://doi.org/10.15468/dl.zfaw79
Anopheles albimanus	-77.66848	2.713274	Primary	GBIF: https://doi.org/10.15468/dl.zfaw79
Anopheles albimanus	-77.663705	2.752645	Primary	GBIF: https://doi.org/10.15468/dl.zfaw79
Anopheles albimanus	-77.663169	2.758674	Primary	GBIF: https://doi.org/10.15468/dl.zfaw79
Anopheles albimanus	-77.659832	2.701077	Primary	GBIF: https://doi.org/10.15468/dl.zfaw79
Anopheles albimanus	-77.64286	2.67032	Primary	GBIF: https://doi.org/10.15468/dl.zfaw79
Anopheles albimanus	-77.634029	2.883271	Primary	GBIF: https://doi.org/10.15468/dl.zfaw79
Anopheles albimanus	-77.631669	2.880863	Primary	GBIF: https://doi.org/10.15468/dl.zfaw79

Anopheles albimanus	-77.629373	2.87585	Primary	GBIF: https://doi.org/10.15468/dl.zfaw79
Anopheles albimanus	-77.624947	2.660282	Primary	GBIF: https://doi.org/10.15468/dl.zfaw79
Anopheles albimanus	-77.623386	2.866411	Primary	GBIF: https://doi.org/10.15468/dl.zfaw79
Anopheles albimanus	-77.623091	2.659431	Primary	GBIF: https://doi.org/10.15468/dl.zfaw79
Anopheles albimanus	-77.592412	2.63895	Primary	GBIF: https://doi.org/10.15468/dl.zfaw79
Anopheles albimanus	-77.590486	2.640292	Primary	GBIF: https://doi.org/10.15468/dl.zfaw79
Anopheles albimanus	-77.49975	8.01803	Primary	GBIF: https://doi.org/10.15468/dl.zfaw79
Anopheles albimanus	-77.41829	25.05483	Primary	GBIF: https://doi.org/10.15468/dl.zfaw79
Anopheles albimanus	-77.37814	25.0284	Primary	GBIF: https://doi.org/10.15468/dl.zfaw79
Anopheles albimanus	-77.351111	25.051389	Primary	GBIF: https://doi.org/10.15468/dl.zfaw79
Anopheles albimanus	-77.34992	18.46276	Primary	GBIF: https://doi.org/10.15468/dl.zfaw79
Anopheles albimanus	-77.32	8.62	Primary	GBIF: https://doi.org/10.15468/dl.zfaw79
Anopheles albimanus	-77.26903	25.02108	Primary	GBIF: https://doi.org/10.15468/dl.zfaw79
Anopheles albimanus	-77.15886	18.32231	Primary	GBIF: https://doi.org/10.15468/dl.zfaw79
Anopheles albimanus	-76.97363	17.99999	Primary	GBIF: https://doi.org/10.15468/dl.zfaw79
Anopheles albimanus	-76.94972	17.98403	Primary	GBIF: https://doi.org/10.15468/dl.zfaw79
Anopheles albimanus	-76.91741	17.95267	Primary	GBIF: https://doi.org/10.15468/dl.zfaw79
Anopheles albimanus	-76.91266	18.03765	Primary	GBIF: https://doi.org/10.15468/dl.zfaw79
Anopheles albimanus	-76.90773	18.01782	Primary	GBIF: https://doi.org/10.15468/dl.zfaw79
Anopheles albimanus	-76.88916	17.95928	Primary	GBIF: https://doi.org/10.15468/dl.zfaw79
Anopheles albimanus	-76.88837	17.97374	Primary	GBIF: https://doi.org/10.15468/dl.zfaw79
Anopheles albimanus	-76.88011	17.9955	Primary	GBIF: https://doi.org/10.15468/dl.zfaw79
Anopheles albimanus	-76.87396	17.95039	Primary	GBIF: https://doi.org/10.15468/dl.zfaw79
Anopheles albimanus	-76.86583	17.9848	Primary	GBIF: https://doi.org/10.15468/dl.zfaw79
Anopheles albimanus	-76.81	18.018056	Primary	GBIF: https://doi.org/10.15468/dl.zfaw79
Anopheles albimanus	-76.79972	18.0007	Primary	GBIF: https://doi.org/10.15468/dl.zfaw79
Anopheles albimanus	-76.62717	17.90563	Primary	GBIF: https://doi.org/10.15468/dl.zfaw79
Anopheles albimanus	-76.5545	8.3425	Primary	GBIF: https://doi.org/10.15468/dl.zfaw79
Anopheles albimanus	-76.5516	8.3492	Primary	GBIF: https://doi.org/10.15468/dl.zfaw79
Anopheles albimanus	-76.5502	8.3446	Primary	GBIF: https://doi.org/10.15468/dl.zfaw79
Anopheles albimanus	-76.55	8.37	Primary	GBIF: https://doi.org/10.15468/dl.zfaw79
Anopheles albimanus	-76.55	8.35	Primary	GBIF: https://doi.org/10.15468/dl.zfaw79
Anopheles albimanus	-76.55	8.34	Primary	GBIF: https://doi.org/10.15468/dl.zfaw79
Anopheles albimanus	-76.5498	8.3676	Primary	GBIF: https://doi.org/10.15468/dl.zfaw79
Anopheles albimanus	-76.510355	3.234182	Primary	GBIF: https://doi.org/10.15468/dl.zfaw79
Anopheles albimanus	-76.33301	25.28371	Primary	GBIF: https://doi.org/10.15468/dl.zfaw79
Anopheles albimanus	-76.2905	17.94611	Primary	GBIF: https://doi.org/10.15468/dl.zfaw79
Anopheles albimanus	-76.28304	17.93404	Primary	GBIF: https://doi.org/10.15468/dl.zfaw79
Anopheles albimanus	-76.27154	17.93448	Primary	GBIF: https://doi.org/10.15468/dl.zfaw79
Anopheles albimanus	-76.24971	17.9507	Primary	GBIF: https://doi.org/10.15468/dl.zfaw79
Anopheles albimanus	-76.24532	25.20023	Primary	GBIF: https://doi.org/10.15468/dl.zfaw79
Anopheles albimanus	-75.78306	20.66723	Primary	GBIF: https://doi.org/10.15468/dl.zfaw79

Anopheles albimanus	-75.71639	20.96722	Primary	GBIF: https://doi.org/10.15468/dl.zfaw79
Anopheles albimanus	-75.64972	20.76723	Primary	GBIF: https://doi.org/10.15468/dl.zfaw79
Anopheles albimanus	-75.54972	20.66723	Primary	GBIF: https://doi.org/10.15468/dl.zfaw79
Anopheles albimanus	-75.16638	20.00059	Primary	GBIF: https://doi.org/10.15468/dl.zfaw79
Anopheles albimanus	-75.036235	5.152643	Primary	GBIF: https://doi.org/10.15468/dl.zfaw79
Anopheles albimanus	-74.5163	24.03376	Primary	GBIF: https://doi.org/10.15468/dl.zfaw79
Anopheles albimanus	-74.4663	24.03376	Primary	GBIF: https://doi.org/10.15468/dl.zfaw79
Anopheles albimanus	-74.21633	18.584	Primary	GBIF: https://doi.org/10.15468/dl.zfaw79
Anopheles albimanus	-74.183333	4.25	Primary	GBIF: https://doi.org/10.15468/dl.zfaw79
Anopheles albimanus	-73.25195	-3.77013	Primary	GBIF: https://doi.org/10.15468/dl.zfaw79
Anopheles albimanus	-73.04934	18.42393	Primary	GBIF: https://doi.org/10.15468/dl.zfaw79
Anopheles albimanus	-72.93	2.65	Primary	GBIF: https://doi.org/10.15468/dl.zfaw79
Anopheles albimanus	-72.88296	18.43401	Primary	GBIF: https://doi.org/10.15468/dl.zfaw79
Anopheles albimanus	-72.68296	19.45062	Primary	GBIF: https://doi.org/10.15468/dl.zfaw79
Anopheles albimanus	-72.64451	4.187754	Primary	GBIF: https://doi.org/10.15468/dl.zfaw79
Anopheles albimanus	-72.52399	18.761	Primary	GBIF: https://doi.org/10.15468/dl.zfaw79
Anopheles albimanus	-72.45611	18.54425	Primary	GBIF: https://doi.org/10.15468/dl.zfaw79
Anopheles albimanus	-72.42776	18.54115	Primary	GBIF: https://doi.org/10.15468/dl.zfaw79
Anopheles albimanus	-72.39962	19.70061	Primary	GBIF: https://doi.org/10.15468/dl.zfaw79
Anopheles albimanus	-72.39474	18.53348	Primary	GBIF: https://doi.org/10.15468/dl.zfaw79
Anopheles albimanus	-72.33295	18.534	Primary	GBIF: https://doi.org/10.15468/dl.zfaw79
Anopheles albimanus	-72.33044	18.52806	Primary	GBIF: https://doi.org/10.15468/dl.zfaw79
Anopheles albimanus	-72.32792	18.56866	Primary	GBIF: https://doi.org/10.15468/dl.zfaw79
Anopheles albimanus	-71.05653	19.70911	Primary	GBIF: https://doi.org/10.15468/dl.zfaw79
Anopheles albimanus	-70.40278	19.11548	Primary	GBIF: https://doi.org/10.15468/dl.zfaw79
Anopheles albimanus	-70.37631	19.13284	Primary	GBIF: https://doi.org/10.15468/dl.zfaw79
Anopheles albimanus	-70.37619	19.11748	Primary	GBIF: https://doi.org/10.15468/dl.zfaw79
Anopheles albimanus	-70.16624	18.66732	Primary	GBIF: https://doi.org/10.15468/dl.zfaw79
Anopheles albimanus	-70.16024	19.09458	Primary	GBIF: https://doi.org/10.15468/dl.zfaw79
Anopheles albimanus	-70.14939	18.47745	Primary	GBIF: https://doi.org/10.15468/dl.zfaw79
Anopheles albimanus	-70.14377	18.48652	Primary	GBIF: https://doi.org/10.15468/dl.zfaw79
Anopheles albimanus	-70.12085	18.45774	Primary	GBIF: https://doi.org/10.15468/dl.zfaw79
Anopheles albimanus	-70.11145	18.46864	Primary	GBIF: https://doi.org/10.15468/dl.zfaw79
Anopheles albimanus	-70.10261	18.41899	Primary	GBIF: https://doi.org/10.15468/dl.zfaw79
Anopheles albimanus	-70.101	-12.5694	Primary	GBIF: https://doi.org/10.15468/dl.zfaw79
Anopheles albimanus	-70.00988	18.42764	Primary	GBIF: https://doi.org/10.15468/dl.zfaw79
Anopheles albimanus	-69.99871	18.46114	Primary	GBIF: https://doi.org/10.15468/dl.zfaw79
Anopheles albimanus	-69.99777	18.46204	Primary	GBIF: https://doi.org/10.15468/dl.zfaw79
Anopheles albimanus	-69.99584	18.45663	Primary	GBIF: https://doi.org/10.15468/dl.zfaw79
Anopheles albimanus	-69.96575	18.49474	Primary	GBIF: https://doi.org/10.15468/dl.zfaw79
Anopheles albimanus	-69.94594	18.5093	Primary	GBIF: https://doi.org/10.15468/dl.zfaw79
Anopheles albimanus	-69.88564	18.57195	Primary	GBIF: https://doi.org/10.15468/dl.zfaw79

Anopheles albimanus	-69.84729	18.48266	Primary	GBIF: https://doi.org/10.15468/dl.zfaw79
Anopheles albimanus	-69.34391	19.20545	Primary	GBIF: https://doi.org/10.15468/dl.zfaw79
Anopheles albimanus	-69.30735	18.45629	Primary	GBIF: https://doi.org/10.15468/dl.zfaw79
Anopheles albimanus	-69.22473	19.00772	Primary	GBIF: https://doi.org/10.15468/dl.zfaw79
Anopheles albimanus	-69.167	-11.917	Primary	GBIF: https://doi.org/10.15468/dl.zfaw79
Anopheles albimanus	-68.24327	10.57093	Primary	GBIF: https://doi.org/10.15468/dl.zfaw79
Anopheles albimanus	-68.01857	10.46352	Primary	GBIF: https://doi.org/10.15468/dl.zfaw79
Anopheles albimanus	-67.8519	10.44685	Primary	GBIF: https://doi.org/10.15468/dl.zfaw79
Anopheles albimanus	-67.16617	18.46732	Primary	GBIF: https://doi.org/10.15468/dl.zfaw79
Anopheles albimanus	-67.16617	18.234	Primary	GBIF: https://doi.org/10.15468/dl.zfaw79
Anopheles albimanus	-67.1495	18.20067	Primary	GBIF: https://doi.org/10.15468/dl.zfaw79
Anopheles albimanus	-67.14616	18.20806	Primary	GBIF: https://doi.org/10.15468/dl.zfaw79
Anopheles albimanus	-67.086	18.02	Primary	GBIF: https://doi.org/10.15468/dl.zfaw79
Anopheles albimanus	-67.085962	18.020018	Primary	GBIF: https://doi.org/10.15468/dl.zfaw79
Anopheles albimanus	-67.082493	18.025667	Primary	GBIF: https://doi.org/10.15468/dl.zfaw79
Anopheles albimanus	-67.082	18.026	Primary	GBIF: https://doi.org/10.15468/dl.zfaw79
Anopheles albimanus	-67.08025	18.024028	Primary	GBIF: https://doi.org/10.15468/dl.zfaw79
Anopheles albimanus	-67.08	18.024	Primary	GBIF: https://doi.org/10.15468/dl.zfaw79
Anopheles albimanus	-67.076889	18.021261	Primary	GBIF: https://doi.org/10.15468/dl.zfaw79
Anopheles albimanus	-67.075	18.027	Primary	GBIF: https://doi.org/10.15468/dl.zfaw79
Anopheles albimanus	-67.075	18.025	Primary	GBIF: https://doi.org/10.15468/dl.zfaw79
Anopheles albimanus	-67.074566	18.024505	Primary	GBIF: https://doi.org/10.15468/dl.zfaw79
Anopheles albimanus	-67.074539	18.027481	Primary	GBIF: https://doi.org/10.15468/dl.zfaw79
Anopheles albimanus	-67.074486	18.031807	Primary	GBIF: https://doi.org/10.15468/dl.zfaw79
Anopheles albimanus	-67.074	18.032	Primary	GBIF: https://doi.org/10.15468/dl.zfaw79
Anopheles albimanus	-67.07324	18.016367	Primary	GBIF: https://doi.org/10.15468/dl.zfaw79
Anopheles albimanus	-67.073	18.016	Primary	GBIF: https://doi.org/10.15468/dl.zfaw79
Anopheles albimanus	-67.070481	18.036123	Primary	GBIF: https://doi.org/10.15468/dl.zfaw79
Anopheles albimanus	-67.070234	18.032874	Primary	GBIF: https://doi.org/10.15468/dl.zfaw79
Anopheles albimanus	-67.07	18.036	Primary	GBIF: https://doi.org/10.15468/dl.zfaw79
Anopheles albimanus	-67.07	18.033	Primary	GBIF: https://doi.org/10.15468/dl.zfaw79
Anopheles albimanus	-67.066235	18.03554	Primary	GBIF: https://doi.org/10.15468/dl.zfaw79
Anopheles albimanus	-67.066	18.036	Primary	GBIF: https://doi.org/10.15468/dl.zfaw79
Anopheles albimanus	-66.882706	17.968509	Primary	GBIF: https://doi.org/10.15468/dl.zfaw79
Anopheles albimanus	-66.8687	17.96955	Primary	GBIF: https://doi.org/10.15468/dl.zfaw79
Anopheles albimanus	-66.8495	18.03401	Primary	GBIF: https://doi.org/10.15468/dl.zfaw79
Anopheles albimanus	-66.841744	17.962708	Primary	GBIF: https://doi.org/10.15468/dl.zfaw79
Anopheles albimanus	-66.836	17.954	Primary	GBIF: https://doi.org/10.15468/dl.zfaw79
Anopheles albimanus	-66.835854	17.953607	Primary	GBIF: https://doi.org/10.15468/dl.zfaw79
Anopheles albimanus	-66.829438	17.970605	Primary	GBIF: https://doi.org/10.15468/dl.zfaw79
Anopheles albimanus	-66.71616	18.46732	Primary	GBIF: https://doi.org/10.15468/dl.zfaw79
Anopheles albimanus	-66.614353	18.011908	Primary	GBIF: https://doi.org/10.15468/dl.zfaw79

Anopheles albimanus	-66.5	18.25	Primary	GBIF: https://doi.org/10.15468/dl.zfaw79
Anopheles albimanus	-66.43282	18.45066	Primary	GBIF: https://doi.org/10.15468/dl.zfaw79
Anopheles albimanus	-66.43282	18.46732	Primary	GBIF: https://doi.org/10.15468/dl.zfaw79
Anopheles albimanus	-66.41615	18.45066	Primary	GBIF: https://doi.org/10.15468/dl.zfaw79
Anopheles albimanus	-66.39949	17.96735	Primary	GBIF: https://doi.org/10.15468/dl.zfaw79
Anopheles albimanus	-66.30095	18.46894	Primary	GBIF: https://doi.org/10.15468/dl.zfaw79
Anopheles albimanus	-66.29949	17.98401	Primary	GBIF: https://doi.org/10.15468/dl.zfaw79
Anopheles albimanus	-66.24948	18.46732	Primary	GBIF: https://doi.org/10.15468/dl.zfaw79
Anopheles albimanus	-66.18282	18.46732	Primary	GBIF: https://doi.org/10.15468/dl.zfaw79
Anopheles albimanus	-66.16615	18.11734	Primary	GBIF: https://doi.org/10.15468/dl.zfaw79
Anopheles albimanus	-66.16615	18.46732	Primary	GBIF: https://doi.org/10.15468/dl.zfaw79
Anopheles albimanus	-66.11615	18.46732	Primary	GBIF: https://doi.org/10.15468/dl.zfaw79
Anopheles albimanus	-66.11615	18.43399	Primary	GBIF: https://doi.org/10.15468/dl.zfaw79
Anopheles albimanus	-66.11615	18.45066	Primary	GBIF: https://doi.org/10.15468/dl.zfaw79
Anopheles albimanus	-66.08281	18.43399	Primary	GBIF: https://doi.org/10.15468/dl.zfaw79
Anopheles albimanus	-66.04948	18.41732	Primary	GBIF: https://doi.org/10.15468/dl.zfaw79
Anopheles albimanus	-66.0167	18.36667	Primary	GBIF: https://doi.org/10.15468/dl.zfaw79
Anopheles albimanus	-65.99948	18.45066	Primary	GBIF: https://doi.org/10.15468/dl.zfaw79
Anopheles albimanus	-65.96615	18.26733	Primary	GBIF: https://doi.org/10.15468/dl.zfaw79
Anopheles albimanus	-65.94948	18.38399	Primary	GBIF: https://doi.org/10.15468/dl.zfaw79
Anopheles albimanus	-65.89948	18.38399	Primary	GBIF: https://doi.org/10.15468/dl.zfaw79
Anopheles albimanus	-65.88281	18.43399	Primary	GBIF: https://doi.org/10.15468/dl.zfaw79
Anopheles albimanus	-65.873056	18.419722	Primary	GBIF: https://doi.org/10.15468/dl.zfaw79
Anopheles albimanus	-65.41613	18.134	Primary	GBIF: https://doi.org/10.15468/dl.zfaw79
Anopheles albimanus	-65.284224	18.299756	Primary	GBIF: https://doi.org/10.15468/dl.zfaw79
Anopheles albimanus	-65.284224	18.299715	Primary	GBIF: https://doi.org/10.15468/dl.zfaw79
Anopheles albimanus	-65.2828	18.31733	Primary	GBIF: https://doi.org/10.15468/dl.zfaw79
Anopheles albimanus	-64.79946	17.73402	Primary	GBIF: https://doi.org/10.15468/dl.zfaw79
Anopheles albimanus	-64.75036	18.36261	Primary	GBIF: https://doi.org/10.15468/dl.zfaw79
Anopheles albimanus	-64.70176	10.1302	Primary	GBIF: https://doi.org/10.15468/dl.zfaw79
Anopheles albimanus	-64.63509	10.21353	Primary	GBIF: https://doi.org/10.15468/dl.zfaw79
Anopheles albimanus	-63.036705	18.056453	Primary	GBIF: https://doi.org/10.15468/dl.zfaw79
Anopheles albimanus	-62.62989	17.16363	Primary	GBIF: https://doi.org/10.15468/dl.zfaw79
Anopheles albimanus	-62.62804	17.14826	Primary	GBIF: https://doi.org/10.15468/dl.zfaw79
Anopheles albimanus	-62.62516	17.17537	Primary	GBIF: https://doi.org/10.15468/dl.zfaw79
Anopheles albimanus	-62.62239	17.15276	Primary	GBIF: https://doi.org/10.15468/dl.zfaw79
Anopheles albimanus	-62.62044	17.1844	Primary	GBIF: https://doi.org/10.15468/dl.zfaw79
Anopheles albimanus	-62.61608	17.13405	Primary	GBIF: https://doi.org/10.15468/dl.zfaw79
Anopheles albimanus	-62.60253	17.20335	Primary	GBIF: https://doi.org/10.15468/dl.zfaw79
Anopheles albimanus	-62.23676	16.72413	Primary	GBIF: https://doi.org/10.15468/dl.zfaw79
Anopheles albimanus	-62.23105	16.744	Primary	GBIF: https://doi.org/10.15468/dl.zfaw79
Anopheles albimanus	-62.22447	16.74759	Primary	GBIF: https://doi.org/10.15468/dl.zfaw79

Anopheles albimanus	-62.21981	16.74034	Primary	GBIF: https://doi.org/10.15468/dl.zfaw79
Anopheles albimanus	-61.86951	17.12013	Primary	GBIF: https://doi.org/10.15468/dl.zfaw79
Anopheles albimanus	-61.86482	17.1192	Primary	GBIF: https://doi.org/10.15468/dl.zfaw79
Anopheles albimanus	-61.83413	17.06661	Primary	GBIF: https://doi.org/10.15468/dl.zfaw79
Anopheles albimanus	-61.82399	17.03491	Primary	GBIF: https://doi.org/10.15468/dl.zfaw79
Anopheles albimanus	-61.81783	17.11803	Primary	GBIF: https://doi.org/10.15468/dl.zfaw79
Anopheles albimanus	-61.80751	17.11526	Primary	GBIF: https://doi.org/10.15468/dl.zfaw79
Anopheles albimanus	-61.78986	17.07779	Primary	GBIF: https://doi.org/10.15468/dl.zfaw79
Anopheles albimanus	-61.78922	17.03742	Primary	GBIF: https://doi.org/10.15468/dl.zfaw79
Anopheles albimanus	-61.78273	17.03405	Primary	GBIF: https://doi.org/10.15468/dl.zfaw79
Anopheles albimanus	-61.76869	17.01561	Primary	GBIF: https://doi.org/10.15468/dl.zfaw79
Anopheles albimanus	-61.76819	17.09152	Primary	GBIF: https://doi.org/10.15468/dl.zfaw79
Anopheles albimanus	-61.7074	17.04957	Primary	GBIF: https://doi.org/10.15468/dl.zfaw79
Anopheles albimanus	-61.58702	16.16941	Primary	GBIF: https://doi.org/10.15468/dl.zfaw79
Anopheles albimanus	-61.54915	16.23242	Primary	GBIF: https://doi.org/10.15468/dl.zfaw79
Anopheles albimanus	-61.5398	16.19761	Primary	GBIF: https://doi.org/10.15468/dl.zfaw79
Anopheles albimanus	-61.53977	16.19759	Primary	GBIF: https://doi.org/10.15468/dl.zfaw79
Anopheles albimanus	-61.31766	15.95056	Primary	GBIF: https://doi.org/10.15468/dl.zfaw79
Anopheles albimanus	-61.282997	10.324386	Primary	GBIF: https://doi.org/10.15468/dl.zfaw79
Anopheles albimanus	-61.27166	15.86886	Primary	GBIF: https://doi.org/10.15468/dl.zfaw79
Anopheles albimanus	-61.01929	14.65485	Primary	GBIF: https://doi.org/10.15468/dl.zfaw79
Anopheles albimanus	-60.7333	-32.7	Primary	GBIF: https://doi.org/10.15468/dl.zfaw79
Anopheles albimanus	-59.30059	-27.86608	Primary	GBIF: https://doi.org/10.15468/dl.zfaw79
Anopheles albimanus	-54.192245	3.826958	Primary	GBIF: https://doi.org/10.15468/dl.zfaw79
Anopheles albimanus	-54.079444	5.505	Primary	GBIF: https://doi.org/10.15468/dl.zfaw79
Anopheles albimanus	-54.053915	3.383908	Primary	GBIF: https://doi.org/10.15468/dl.zfaw79
Anopheles albimanus	-54.031415	3.643348	Primary	GBIF: https://doi.org/10.15468/dl.zfaw79
Anopheles albimanus	-48.8293	-25.4754	Primary	GBIF: https://doi.org/10.15468/dl.zfaw79
Anopheles albimanus	-34.926846	-8.16108	Primary	GBIF: https://doi.org/10.15468/dl.zfaw79
Anopheles albimanus	11.0167	46.8667	Primary	GBIF: https://doi.org/10.15468/dl.zfaw79
Anopheles albitarsis	-82.64534	8.7735	Primary	GBIF: https://doi.org/10.15468/dl.zfaw79
Anopheles albitarsis	-80.139282	8.681183	Primary	GBIF: https://doi.org/10.15468/dl.zfaw79
Anopheles albitarsis	-80	9	Primary	GBIF: https://doi.org/10.15468/dl.zfaw79
Anopheles albitarsis	-79.91647	9.2013	Primary	GBIF: https://doi.org/10.15468/dl.zfaw79
Anopheles albitarsis	-79.817354	9.037011	Primary	GBIF: https://doi.org/10.15468/dl.zfaw79
Anopheles albitarsis	-79.7998	9.01797	Primary	GBIF: https://doi.org/10.15468/dl.zfaw79
Anopheles albitarsis	-79.779775	8.875191	Primary	GBIF: https://doi.org/10.15468/dl.zfaw79
Anopheles albitarsis	-79.777715	8.882654	Primary	GBIF: https://doi.org/10.15468/dl.zfaw79
Anopheles albitarsis	-79.774281	8.881297	Primary	GBIF: https://doi.org/10.15468/dl.zfaw79
Anopheles albitarsis	-79.773595	8.881975	Primary	GBIF: https://doi.org/10.15468/dl.zfaw79
Anopheles albitarsis	-79.773374	8.876812	Primary	GBIF: https://doi.org/10.15468/dl.zfaw79
Anopheles albitarsis	-79.772222	8.884689	Primary	GBIF: https://doi.org/10.15468/dl.zfaw79

Anopheles albitarsis	-79.769475	8.878583	Primary	GBIF: https://doi.org/10.15468/dl.zfaw79
Anopheles albitarsis	-79.5333	9.1667	Primary	GBIF: https://doi.org/10.15468/dl.zfaw79
Anopheles albitarsis	-74.77	8.11	Primary	GBIF: https://doi.org/10.15468/dl.zfaw79
Anopheles albitarsis	-74.71	7.61	Primary	GBIF: https://doi.org/10.15468/dl.zfaw79
Anopheles albitarsis	-74.71	7.68	Primary	GBIF: https://doi.org/10.15468/dl.zfaw79
Anopheles albitarsis	-74.66	7.72	Primary	GBIF: https://doi.org/10.15468/dl.zfaw79
Anopheles albitarsis	-73.61867	4.14669	Primary	GBIF: https://doi.org/10.15468/dl.zfaw79
Anopheles albitarsis	-73.61867	4.31336	Primary	GBIF: https://doi.org/10.15468/dl.zfaw79
Anopheles albitarsis	-72.89	-7.62	Primary	GBIF: https://doi.org/10.15468/dl.zfaw79
Anopheles albitarsis	-72.74	8.64	Primary	GBIF: https://doi.org/10.15468/dl.zfaw79
Anopheles albitarsis	-72.737	8.64	Primary	GBIF: https://doi.org/10.15468/dl.zfaw79
Anopheles albitarsis	-72.633	8.9	Primary	GBIF: https://doi.org/10.15468/dl.zfaw79
Anopheles albitarsis	-72.63	8.9	Primary	GBIF: https://doi.org/10.15468/dl.zfaw79
Anopheles albitarsis	-64.711403	-3.35417	Primary	GBIF: https://doi.org/10.15468/dl.zfaw79
Anopheles albitarsis	-64.27	-12.47	Primary	GBIF: https://doi.org/10.15468/dl.zfaw79
Anopheles albitarsis	-64.267	-12.467	Primary	GBIF: https://doi.org/10.15468/dl.zfaw79
Anopheles albitarsis	-63.38	-9.67	Primary	GBIF: https://doi.org/10.15468/dl.zfaw79
Anopheles albitarsis	-63.1	-6.98	Primary	GBIF: https://doi.org/10.15468/dl.zfaw79
Anopheles albitarsis	-63.07	-9.93	Primary	GBIF: https://doi.org/10.15468/dl.zfaw79
Anopheles albitarsis	-62.79	-10.29	Primary	GBIF: https://doi.org/10.15468/dl.zfaw79
Anopheles albitarsis	-62.76	-10.29	Primary	GBIF: https://doi.org/10.15468/dl.zfaw79
Anopheles albitarsis	-61.398783	10.21572	Primary	GBIF: https://doi.org/10.15468/dl.zfaw79
Anopheles albitarsis	-61.2	10.4	Primary	GBIF: https://doi.org/10.15468/dl.zfaw79
Anopheles albitarsis	-60.24	-1.49	Primary	GBIF: https://doi.org/10.15468/dl.zfaw79
Anopheles albitarsis	-60.02	-3.1	Primary	GBIF: https://doi.org/10.15468/dl.zfaw79
Anopheles albitarsis	-60.017	-3.1	Primary	GBIF: https://doi.org/10.15468/dl.zfaw79
Anopheles albitarsis	-59.95	-15	Primary	GBIF: https://doi.org/10.15468/dl.zfaw79
Anopheles albitarsis	-59.87	-14.88	Primary	GBIF: https://doi.org/10.15468/dl.zfaw79
Anopheles albitarsis	-59.85	-14.99	Primary	GBIF: https://doi.org/10.15468/dl.zfaw79
Anopheles albitarsis	-59.514	-33.778	Primary	GBIF: https://doi.org/10.15468/dl.zfaw79
Anopheles albitarsis	-59.51	-33.78	Primary	GBIF: https://doi.org/10.15468/dl.zfaw79
Anopheles albitarsis	-59.50063	-33.79953	Primary	GBIF: https://doi.org/10.15468/dl.zfaw79
Anopheles albitarsis	-58.833	-27.467	Primary	GBIF: https://doi.org/10.15468/dl.zfaw79
Anopheles albitarsis	-58.83	-27.47	Primary	GBIF: https://doi.org/10.15468/dl.zfaw79
Anopheles albitarsis	-58.083	-29.2	Primary	GBIF: https://doi.org/10.15468/dl.zfaw79
Anopheles albitarsis	-58.08	-29.2	Primary	GBIF: https://doi.org/10.15468/dl.zfaw79
Anopheles albitarsis	-57.735	-2.542	Primary	GBIF: https://doi.org/10.15468/dl.zfaw79
Anopheles albitarsis	-57.73	-2.54	Primary	GBIF: https://doi.org/10.15468/dl.zfaw79
Anopheles albitarsis	-56.673	-2.62	Primary	GBIF: https://doi.org/10.15468/dl.zfaw79
Anopheles albitarsis	-56.67	-2.62	Primary	GBIF: https://doi.org/10.15468/dl.zfaw79
Anopheles albitarsis	-56.66	-27.53	Primary	GBIF: https://doi.org/10.15468/dl.zfaw79
Anopheles albitarsis	-56.3783	-20.240601	Primary	GBIF: https://doi.org/10.15468/dl.zfaw79

Anopheles albitarsis	-56.13	-15.43	Primary	GBIF: https://doi.org/10.15468/dl.zfaw79
Anopheles albitarsis	-56.12	-15.36	Primary	GBIF: https://doi.org/10.15468/dl.zfaw79
Anopheles albitarsis	-56.05	-28.55	Primary	GBIF: https://doi.org/10.15468/dl.zfaw79
Anopheles albitarsis	-55.9833	-4.25	Primary	GBIF: https://doi.org/10.15468/dl.zfaw79
Anopheles albitarsis	-55.98	-4.25	Primary	GBIF: https://doi.org/10.15468/dl.zfaw79
Anopheles albitarsis	-55.883	-27.383	Primary	GBIF: https://doi.org/10.15468/dl.zfaw79
Anopheles albitarsis	-55.88	-27.38	Primary	GBIF: https://doi.org/10.15468/dl.zfaw79
Anopheles albitarsis	-55.833	-4.167	Primary	GBIF: https://doi.org/10.15468/dl.zfaw79
Anopheles albitarsis	-55.83	-4.17	Primary	GBIF: https://doi.org/10.15468/dl.zfaw79
Anopheles albitarsis	-55.63	-19.51	Primary	GBIF: https://doi.org/10.15468/dl.zfaw79
Anopheles albitarsis	-55.471	-1.869	Primary	GBIF: https://doi.org/10.15468/dl.zfaw79
Anopheles albitarsis	-55.47	-1.87	Primary	GBIF: https://doi.org/10.15468/dl.zfaw79
Anopheles albitarsis	-55.227778	-28.511389	Primary	GBIF: https://doi.org/10.15468/dl.zfaw79
Anopheles albitarsis	-54.9	-10.38	Primary	GBIF: https://doi.org/10.15468/dl.zfaw79
Anopheles albitarsis	-54.9	-10.383	Primary	GBIF: https://doi.org/10.15468/dl.zfaw79
Anopheles albitarsis	-54.752	-2.434	Primary	GBIF: https://doi.org/10.15468/dl.zfaw79
Anopheles albitarsis	-54.75	-2.43	Primary	GBIF: https://doi.org/10.15468/dl.zfaw79
Anopheles albitarsis	-54.75	-25.37	Primary	GBIF: https://doi.org/10.15468/dl.zfaw79
Anopheles albitarsis	-54.7	-25.48	Primary	GBIF: https://doi.org/10.15468/dl.zfaw79
Anopheles albitarsis	-54.66	-2.48	Primary	GBIF: https://doi.org/10.15468/dl.zfaw79
Anopheles albitarsis	-54.5833	-25.55	Primary	GBIF: https://doi.org/10.15468/dl.zfaw79
Anopheles albitarsis	-54.51	-16.37	Primary	GBIF: https://doi.org/10.15468/dl.zfaw79
Anopheles albitarsis	-54.42	-9.99	Primary	GBIF: https://doi.org/10.15468/dl.zfaw79
Anopheles albitarsis	-54.38	-24.93	Primary	GBIF: https://doi.org/10.15468/dl.zfaw79
Anopheles albitarsis	-54.25	-24.07	Primary	GBIF: https://doi.org/10.15468/dl.zfaw79
Anopheles albitarsis	-54.25	-24.0667	Primary	GBIF: https://doi.org/10.15468/dl.zfaw79
Anopheles albitarsis	-54.22	-20.08	Primary	GBIF: https://doi.org/10.15468/dl.zfaw79
Anopheles albitarsis	-54.1	-2.02	Primary	GBIF: https://doi.org/10.15468/dl.zfaw79
Anopheles albitarsis	-54.099	-2.017	Primary	GBIF: https://doi.org/10.15468/dl.zfaw79
Anopheles albitarsis	-53.66	-22.98	Primary	GBIF: https://doi.org/10.15468/dl.zfaw79
Anopheles albitarsis	-53.633	-2.133	Primary	GBIF: https://doi.org/10.15468/dl.zfaw79
Anopheles albitarsis	-53.63	-2.13	Primary	GBIF: https://doi.org/10.15468/dl.zfaw79
Anopheles albitarsis	-53.53	-23.11	Primary	GBIF: https://doi.org/10.15468/dl.zfaw79
Anopheles albitarsis	-53.52	-23.18	Primary	GBIF: https://doi.org/10.15468/dl.zfaw79
Anopheles albitarsis	-53.37	-22.85	Primary	GBIF: https://doi.org/10.15468/dl.zfaw79
Anopheles albitarsis	-53.23477	3.92404	Primary	GBIF: https://doi.org/10.15468/dl.zfaw79
Anopheles albitarsis	-53.205199	5.47958	Primary	GBIF: https://doi.org/10.15468/dl.zfaw79
Anopheles albitarsis	-53.03179	5.41668	Primary	GBIF: https://doi.org/10.15468/dl.zfaw79
Anopheles albitarsis	-53.02	-22.65	Primary	GBIF: https://doi.org/10.15468/dl.zfaw79
Anopheles albitarsis	-52.743	-1.476	Primary	GBIF: https://doi.org/10.15468/dl.zfaw79
Anopheles albitarsis	-52.74	-1.48	Primary	GBIF: https://doi.org/10.15468/dl.zfaw79
Anopheles albitarsis	-52.573333	-28.499167	Primary	GBIF: https://doi.org/10.15468/dl.zfaw79

Anopheles albitarsis	-52.509685	4.832668	Primary	GBIF: https://doi.org/10.15468/dl.zfaw79
Anopheles albitarsis	-52.492926	4.892476	Primary	GBIF: https://doi.org/10.15468/dl.zfaw79
Anopheles albitarsis	-52.47241	5.01171	Primary	GBIF: https://doi.org/10.15468/dl.zfaw79
Anopheles albitarsis	-52.45662	4.792945	Primary	GBIF: https://doi.org/10.15468/dl.zfaw79
Anopheles albitarsis	-52.3667	-22.7	Primary	GBIF: https://doi.org/10.15468/dl.zfaw79
Anopheles albitarsis	-52.333355	4.833348	Primary	GBIF: https://doi.org/10.15468/dl.zfaw79
Anopheles albitarsis	-52.33033	4.93461	Primary	GBIF: https://doi.org/10.15468/dl.zfaw79
Anopheles albitarsis	-52.323296	4.936976	Primary	GBIF: https://doi.org/10.15468/dl.zfaw79
Anopheles albitarsis	-51.15	-23.3	Primary	GBIF: https://doi.org/10.15468/dl.zfaw79
Anopheles albitarsis	-51.09	-18.96	Primary	GBIF: https://doi.org/10.15468/dl.zfaw79
Anopheles albitarsis	-51.03	-18.93	Primary	GBIF: https://doi.org/10.15468/dl.zfaw79
Anopheles albitarsis	-50.35	-19.52	Primary	GBIF: https://doi.org/10.15468/dl.zfaw79
Anopheles albitarsis	-50.269444	-29.886944	Primary	GBIF: https://doi.org/10.15468/dl.zfaw79
Anopheles albitarsis	-50.22	-29.64	Primary	GBIF: https://doi.org/10.15468/dl.zfaw79
Anopheles albitarsis	-50.22	-29.66	Primary	GBIF: https://doi.org/10.15468/dl.zfaw79
Anopheles albitarsis	-49.82	-10.64	Primary	GBIF: https://doi.org/10.15468/dl.zfaw79
Anopheles albitarsis	-49.75	-10.9	Primary	GBIF: https://doi.org/10.15468/dl.zfaw79
Anopheles albitarsis	-49.74	-10.72	Primary	GBIF: https://doi.org/10.15468/dl.zfaw79
Anopheles albitarsis	-49.71	-10.61	Primary	GBIF: https://doi.org/10.15468/dl.zfaw79
Anopheles albitarsis	-49.202	-5.365	Primary	GBIF: https://doi.org/10.15468/dl.zfaw79
Anopheles albitarsis	-49.07	-20.02	Primary	GBIF: https://doi.org/10.15468/dl.zfaw79
Anopheles albitarsis	-48.97	-26.58	Primary	GBIF: https://doi.org/10.15468/dl.zfaw79
Anopheles albitarsis	-48.967	-26.583	Primary	GBIF: https://doi.org/10.15468/dl.zfaw79
Anopheles albitarsis	-48.92	-5.28	Primary	GBIF: https://doi.org/10.15468/dl.zfaw79
Anopheles albitarsis	-48.91	-5.44	Primary	GBIF: https://doi.org/10.15468/dl.zfaw79
Anopheles albitarsis	-48.85	-20.04	Primary	GBIF: https://doi.org/10.15468/dl.zfaw79
Anopheles albitarsis	-48.72	-20.12	Primary	GBIF: https://doi.org/10.15468/dl.zfaw79
Anopheles albitarsis	-48.45	-22.07	Primary	GBIF: https://doi.org/10.15468/dl.zfaw79
Anopheles albitarsis	-47.885	-24.613	Primary	GBIF: https://doi.org/10.15468/dl.zfaw79
Anopheles albitarsis	-47.88	-24.61	Primary	GBIF: https://doi.org/10.15468/dl.zfaw79
Anopheles albitarsis	-47.551	-24.726	Primary	GBIF: https://doi.org/10.15468/dl.zfaw79
Anopheles albitarsis	-47.55	-24.73	Primary	GBIF: https://doi.org/10.15468/dl.zfaw79
Anopheles albitarsis	-46.02	-7.56	Primary	GBIF: https://doi.org/10.15468/dl.zfaw79
Anopheles albitarsis	-46.01	-7.56	Primary	GBIF: https://doi.org/10.15468/dl.zfaw79
Anopheles albitarsis	-45.5	-23	Primary	GBIF: https://doi.org/10.15468/dl.zfaw79
Anopheles albitarsis	-45.45	-22.96	Primary	GBIF: https://doi.org/10.15468/dl.zfaw79
Anopheles albitarsis	-45.24	-22.84	Primary	GBIF: https://doi.org/10.15468/dl.zfaw79
Anopheles albitarsis	-44.88	-20.77	Primary	GBIF: https://doi.org/10.15468/dl.zfaw79
Anopheles albitarsis	-43.70484	-22.75528	Primary	GBIF: https://doi.org/10.15468/dl.zfaw79
Anopheles albitarsis	-43.292801	-22.1619	Primary	GBIF: https://doi.org/10.15468/dl.zfaw79
Anopheles albitarsis	-43.23048	-22.84013	Primary	GBIF: https://doi.org/10.15468/dl.zfaw79
Anopheles albitarsis	-43.2075	-22.9028	Primary	GBIF: https://doi.org/10.15468/dl.zfaw79

Anopheles albitarsis	-42.99067	-22.91584	Primary	GBIF: https://doi.org/10.15468/dl.zfaw79
Anopheles albitarsis	-42.653099	-22.4625	Primary	GBIF: https://doi.org/10.15468/dl.zfaw79
Anopheles albitarsis	-40.12	-19.08	Primary	GBIF: https://doi.org/10.15468/dl.zfaw79
Anopheles albitarsis	-40.12	-19.35	Primary	GBIF: https://doi.org/10.15468/dl.zfaw79
Anopheles albitarsis	-40.05	-19.05	Primary	GBIF: https://doi.org/10.15468/dl.zfaw79
Anopheles albitarsis	-40	-19.14	Primary	GBIF: https://doi.org/10.15468/dl.zfaw79
Anopheles albitarsis	-39.933	-13.433	Primary	GBIF: https://doi.org/10.15468/dl.zfaw79
Anopheles albitarsis	-39.93	-13.43	Primary	GBIF: https://doi.org/10.15468/dl.zfaw79
Anopheles albitarsis	-39.38	-15.4	Primary	GBIF: https://doi.org/10.15468/dl.zfaw79
Anopheles albotaeniatus	96.1275	4.150278	Unclassified	GBIF: https://doi.org/10.15468/dl.zfaw79
Anopheles albotaeniatus	97.3175	5.058611	Unclassified	GBIF: https://doi.org/10.15468/dl.zfaw79
Anopheles albotaeniatus	97.975556	4.473056	Unclassified	GBIF: https://doi.org/10.15468/dl.zfaw79
Anopheles albotaeniatus	98.785	1.736667	Unclassified	GBIF: https://doi.org/10.15468/dl.zfaw79
Anopheles albotaeniatus	98.807778	3.531111	Unclassified	GBIF: https://doi.org/10.15468/dl.zfaw79
Anopheles albotaeniatus	99.359444	0.798056	Unclassified	GBIF: https://doi.org/10.15468/dl.zfaw79
Anopheles albotaeniatus	102.3425	-1.218611	Unclassified	GBIF: https://doi.org/10.15468/dl.zfaw79
Anopheles albotaeniatus	120.756903	-1.43938	Unclassified	GBIF: https://doi.org/10.15468/dl.zfaw79
Anopheles algeriensis	-8.931983	53.015862	Unclassified	GBIF: https://doi.org/10.15468/dl.zfaw79
Anopheles algeriensis	-8.789	38.072	Unclassified	GBIF: https://doi.org/10.15468/dl.zfaw79
Anopheles algeriensis	-8.517	38.38	Unclassified	GBIF: https://doi.org/10.15468/dl.zfaw79
Anopheles algeriensis	-7.953745	53.317867	Unclassified	GBIF: https://doi.org/10.15468/dl.zfaw79
Anopheles algeriensis	-7.953162	53.318592	Unclassified	GBIF: https://doi.org/10.15468/dl.zfaw79
Anopheles algeriensis	-4.304798	53.313086	Unclassified	GBIF: https://doi.org/10.15468/dl.zfaw79
Anopheles algeriensis	-4.291542	53.317837	Unclassified	GBIF: https://doi.org/10.15468/dl.zfaw79
Anopheles algeriensis	-4.260879	53.305836	Unclassified	GBIF: https://doi.org/10.15468/dl.zfaw79
Anopheles algeriensis	-4.26046	53.305862	Unclassified	GBIF: https://doi.org/10.15468/dl.zfaw79
Anopheles algeriensis	-4.260295	53.308994	Unclassified	GBIF: https://doi.org/10.15468/dl.zfaw79
Anopheles algeriensis	-4.252183	53.308311	Unclassified	GBIF: https://doi.org/10.15468/dl.zfaw79
Anopheles algeriensis	-4.252118	53.310497	Unclassified	GBIF: https://doi.org/10.15468/dl.zfaw79
Anopheles algeriensis	-4.252023	53.308701	Unclassified	GBIF: https://doi.org/10.15468/dl.zfaw79
Anopheles algeriensis	-4.250571	53.309627	Unclassified	GBIF: https://doi.org/10.15468/dl.zfaw79
Anopheles algeriensis	-4.250476	53.307831	Unclassified	GBIF: https://doi.org/10.15468/dl.zfaw79
Anopheles algeriensis	-4.250362	53.308498	Unclassified	GBIF: https://doi.org/10.15468/dl.zfaw79
Anopheles algeriensis	-4.250275	53.275468	Unclassified	GBIF: https://doi.org/10.15468/dl.zfaw79
Anopheles algeriensis	-4.250228	53.27457	Unclassified	GBIF: https://doi.org/10.15468/dl.zfaw79
Anopheles algeriensis	-4.249071	53.309655	Unclassified	GBIF: https://doi.org/10.15468/dl.zfaw79
Anopheles algeriensis	-4.24767	53.27409	Unclassified	GBIF: https://doi.org/10.15468/dl.zfaw79
Anopheles algeriensis	-4.24706	53.27392	Unclassified	GBIF: https://doi.org/10.15468/dl.zfaw79
Anopheles algeriensis	-4.24506	53.27306	Unclassified	GBIF: https://doi.org/10.15468/dl.zfaw79
Anopheles algeriensis	-2.980312	54.396437	Unclassified	GBIF: https://doi.org/10.15468/dl.zfaw79
Anopheles algeriensis	-2.8705	36.73076	Unclassified	GBIF: https://doi.org/10.15468/dl.zfaw79
Anopheles algeriensis	-2.870471	36.730836	Unclassified	GBIF: https://doi.org/10.15468/dl.zfaw79

Anopheles algeriensis	-2.704407	36.692	Unclassified	GBIF: https://doi.org/10.15468/dl.zfaw79
Anopheles algeriensis	-2.704183	36.691802	Unclassified	GBIF: https://doi.org/10.15468/dl.zfaw79
Anopheles algeriensis	-2.51	42.44	Unclassified	GBIF: https://doi.org/10.15468/dl.zfaw79
Anopheles algeriensis	-2.50962	42.44007	Unclassified	GBIF: https://doi.org/10.15468/dl.zfaw79
Anopheles algeriensis	-2.41	42.48	Unclassified	GBIF: https://doi.org/10.15468/dl.zfaw79
Anopheles algeriensis	-2.4071	42.48438	Unclassified	GBIF: https://doi.org/10.15468/dl.zfaw79
Anopheles algeriensis	-2.4071	42.4844	Unclassified	GBIF: https://doi.org/10.15468/dl.zfaw79
Anopheles algeriensis	0.6	52.55	Unclassified	GBIF: https://doi.org/10.15468/dl.zfaw79
Anopheles algeriensis	0.60359	52.5582	Unclassified	GBIF: https://doi.org/10.15468/dl.zfaw79
Anopheles algeriensis	1.468096	52.591956	Unclassified	GBIF: https://doi.org/10.15468/dl.zfaw79
Anopheles algeriensis	1.482356	52.771418	Unclassified	GBIF: https://doi.org/10.15468/dl.zfaw79
Anopheles algeriensis	1.489496	52.591776	Unclassified	GBIF: https://doi.org/10.15468/dl.zfaw79
Anopheles algeriensis	1.502657	52.737992	Unclassified	GBIF: https://doi.org/10.15468/dl.zfaw79
Anopheles algeriensis	1.504063	52.737051	Unclassified	GBIF: https://doi.org/10.15468/dl.zfaw79
Anopheles algeriensis	1.505541	52.737007	Unclassified	GBIF: https://doi.org/10.15468/dl.zfaw79
Anopheles algeriensis	1.50702	52.736963	Unclassified	GBIF: https://doi.org/10.15468/dl.zfaw79
Anopheles algeriensis	1.508535	52.755806	Unclassified	GBIF: https://doi.org/10.15468/dl.zfaw79
Anopheles algeriensis	1.508607	52.756703	Unclassified	GBIF: https://doi.org/10.15468/dl.zfaw79
Anopheles algeriensis	1.5667	52.733	Unclassified	GBIF: https://doi.org/10.15468/dl.zfaw79
Anopheles algeriensis	3	28	Unclassified	GBIF: https://doi.org/10.15468/dl.zfaw79
Anopheles algeriensis	3.087891	28	Unclassified	GBIF: https://doi.org/10.15468/dl.zfaw79
Anopheles algeriensis	3.088333	43.107498	Unclassified	GBIF: https://doi.org/10.15468/dl.zfaw79
Anopheles algeriensis	9	40	Unclassified	GBIF: https://doi.org/10.15468/dl.zfaw79
Anopheles algeriensis	9.381	41.666	Unclassified	GBIF: https://doi.org/10.15468/dl.zfaw79
Anopheles algeriensis	10.426171	51.083046	Unclassified	GBIF: https://doi.org/10.15468/dl.zfaw79
Anopheles algeriensis	11.94807	57.54828	Unclassified	GBIF: https://doi.org/10.15468/dl.zfaw79
Anopheles algeriensis	12.71	52.56	Unclassified	GBIF: https://doi.org/10.15468/dl.zfaw79
Anopheles algeriensis	12.88	53.19	Unclassified	GBIF: https://doi.org/10.15468/dl.zfaw79
Anopheles algeriensis	17.618	43.095	Unclassified	GBIF: https://doi.org/10.15468/dl.zfaw79
Anopheles algeriensis	18.22	56.93	Unclassified	GBIF: https://doi.org/10.15468/dl.zfaw79
Anopheles algeriensis	18.70463	57.7204	Unclassified	GBIF: https://doi.org/10.15468/dl.zfaw79
Anopheles algeriensis	19.18	57.97	Unclassified	GBIF: https://doi.org/10.15468/dl.zfaw79
Anopheles algeriensis	25.094158	45.82455	Unclassified	GBIF: https://doi.org/10.15468/dl.zfaw79
Anopheles algeriensis	25.50366	29.17801	Unclassified	GBIF: https://doi.org/10.15468/dl.zfaw79
Anopheles algeriensis	25.54116	29.20718	Unclassified	GBIF: https://doi.org/10.15468/dl.zfaw79
Anopheles algeriensis	25.712	29.2044	Unclassified	GBIF: https://doi.org/10.15468/dl.zfaw79
Anopheles algeriensis	29.516	45.151	Unclassified	GBIF: https://doi.org/10.15468/dl.zfaw79
Anopheles algeriensis	29.52	45.15	Unclassified	GBIF: https://doi.org/10.15468/dl.zfaw79
Anopheles algeriensis	29.63	45.06	Unclassified	GBIF: https://doi.org/10.15468/dl.zfaw79
Anopheles algeriensis	29.631	45.064	Unclassified	GBIF: https://doi.org/10.15468/dl.zfaw79
Anopheles algeriensis	35.59972	33.06556	Unclassified	GBIF: https://doi.org/10.15468/dl.zfaw79
Anopheles algeriensis	35.60528	32.89612	Unclassified	GBIF: https://doi.org/10.15468/dl.zfaw79

Anopheles amictus	115.704788	-32.699242	Unclassified	GBIF: https://doi.org/10.15468/dl.zfaw79
Anopheles amictus	119.75	-21.166666	Unclassified	GBIF: https://doi.org/10.15468/dl.zfaw79
Anopheles amictus	119.75	-21.16667	Unclassified	GBIF: https://doi.org/10.15468/dl.zfaw79
Anopheles amictus	123.1667	-17.63333	Unclassified	GBIF: https://doi.org/10.15468/dl.zfaw79
Anopheles amictus	123.166702	-17.633329	Unclassified	GBIF: https://doi.org/10.15468/dl.zfaw79
Anopheles amictus	127.66639	-18.22861	Unclassified	GBIF: https://doi.org/10.15468/dl.zfaw79
Anopheles amictus	127.8333	-15.1833	Unclassified	GBIF: https://doi.org/10.15468/dl.zfaw79
Anopheles amictus	128.100006	-15.466666	Unclassified	GBIF: https://doi.org/10.15468/dl.zfaw79
Anopheles amictus	128.10001	-15.46667	Unclassified	GBIF: https://doi.org/10.15468/dl.zfaw79
Anopheles amictus	130.983333	-12.466667	Unclassified	GBIF: https://doi.org/10.15468/dl.zfaw79
Anopheles amictus	131.100006	-13.233334	Unclassified	GBIF: https://doi.org/10.15468/dl.zfaw79
Anopheles amictus	131.10001	-13.23333	Unclassified	GBIF: https://doi.org/10.15468/dl.zfaw79
Anopheles amictus	131.105	-13.23999	Unclassified	GBIF: https://doi.org/10.15468/dl.zfaw79
Anopheles amictus	131.11	-13.24	Unclassified	GBIF: https://doi.org/10.15468/dl.zfaw79
Anopheles amictus	131.25	-12.633333	Unclassified	GBIF: https://doi.org/10.15468/dl.zfaw79
Anopheles amictus	131.25	-12.63333	Unclassified	GBIF: https://doi.org/10.15468/dl.zfaw79
Anopheles amictus	132.03334	-13.516666	Unclassified	GBIF: https://doi.org/10.15468/dl.zfaw79
Anopheles amictus	132.03334	-13.51667	Unclassified	GBIF: https://doi.org/10.15468/dl.zfaw79
Anopheles amictus	132.27	-14.47	Unclassified	GBIF: https://doi.org/10.15468/dl.zfaw79
Anopheles amictus	132.866699	-12.58333	Unclassified	GBIF: https://doi.org/10.15468/dl.zfaw79
Anopheles amictus	132.8667	-12.58333	Unclassified	GBIF: https://doi.org/10.15468/dl.zfaw79
Anopheles amictus	134.192547	-19.640987	Unclassified	GBIF: https://doi.org/10.15468/dl.zfaw79
Anopheles amictus	134.733337	-14.733334	Unclassified	GBIF: https://doi.org/10.15468/dl.zfaw79
Anopheles amictus	134.73334	-14.73333	Unclassified	GBIF: https://doi.org/10.15468/dl.zfaw79
Anopheles amictus	135.3833	-14.73333	Unclassified	GBIF: https://doi.org/10.15468/dl.zfaw79
Anopheles amictus	135.383301	-14.73333	Unclassified	GBIF: https://doi.org/10.15468/dl.zfaw79
Anopheles amictus	136.25	-16.16667	Unclassified	GBIF: https://doi.org/10.15468/dl.zfaw79
Anopheles amictus	139.483337	-20.733334	Unclassified	GBIF: https://doi.org/10.15468/dl.zfaw79
Anopheles amictus	139.48334	-20.73333	Unclassified	GBIF: https://doi.org/10.15468/dl.zfaw79
Anopheles amictus	139.671044	-7.666446	Unclassified	GBIF: https://doi.org/10.15468/dl.zfaw79
Anopheles amictus	140.404444	-8.495833	Unclassified	GBIF: https://doi.org/10.15468/dl.zfaw79
Anopheles amictus	140.407156	-8.500401	Unclassified	GBIF: https://doi.org/10.15468/dl.zfaw79
Anopheles amictus	141.066666	-17.666666	Unclassified	GBIF: https://doi.org/10.15468/dl.zfaw79
Anopheles amictus	141.06667	-17.66667	Unclassified	GBIF: https://doi.org/10.15468/dl.zfaw79
Anopheles amictus	141.449997	-16.65	Unclassified	GBIF: https://doi.org/10.15468/dl.zfaw79
Anopheles amictus	141.45	-16.65	Unclassified	GBIF: https://doi.org/10.15468/dl.zfaw79
Anopheles amictus	141.591	-15.198	Unclassified	GBIF: https://doi.org/10.15468/dl.zfaw79
Anopheles amictus	141.75	-15.466666	Unclassified	GBIF: https://doi.org/10.15468/dl.zfaw79
Anopheles amictus	141.75	-15.46667	Unclassified	GBIF: https://doi.org/10.15468/dl.zfaw79
Anopheles amictus	141.949997	-16.966667	Unclassified	GBIF: https://doi.org/10.15468/dl.zfaw79
Anopheles amictus	141.95	-16.96667	Unclassified	GBIF: https://doi.org/10.15468/dl.zfaw79
Anopheles amictus	142.216995	-17.533001	Unclassified	GBIF: https://doi.org/10.15468/dl.zfaw79

Anopheles amictus	142.217	-17.533	Unclassified	GBIF: https://doi.org/10.15468/dl.zfaw79
Anopheles amictus	142.676	-17.619	Unclassified	GBIF: https://doi.org/10.15468/dl.zfaw79
Anopheles amictus	143.03334	-22.383333	Unclassified	GBIF: https://doi.org/10.15468/dl.zfaw79
Anopheles amictus	143.03334	-22.38333	Unclassified	GBIF: https://doi.org/10.15468/dl.zfaw79
Anopheles amictus	143.13333	-20.73333	Unclassified	GBIF: https://doi.org/10.15468/dl.zfaw79
Anopheles amictus	143.133331	-20.733334	Unclassified	GBIF: https://doi.org/10.15468/dl.zfaw79
Anopheles amictus	143.28329	-20.51667	Unclassified	GBIF: https://doi.org/10.15468/dl.zfaw79
Anopheles amictus	143.283295	-20.51667	Unclassified	GBIF: https://doi.org/10.15468/dl.zfaw79
Anopheles amictus	143.5	-14.783334	Unclassified	GBIF: https://doi.org/10.15468/dl.zfaw79
Anopheles amictus	143.5	-14.78333	Unclassified	GBIF: https://doi.org/10.15468/dl.zfaw79
Anopheles amictus	143.55	-13.98333	Unclassified	GBIF: https://doi.org/10.15468/dl.zfaw79
Anopheles amictus	143.550003	-13.983334	Unclassified	GBIF: https://doi.org/10.15468/dl.zfaw79
Anopheles amictus	144.066666	-28.033333	Unclassified	GBIF: https://doi.org/10.15468/dl.zfaw79
Anopheles amictus	144.06667	-28.03333	Unclassified	GBIF: https://doi.org/10.15468/dl.zfaw79
Anopheles amictus	144.850006	-21.4	Unclassified	GBIF: https://doi.org/10.15468/dl.zfaw79
Anopheles amictus	144.85001	-21.4	Unclassified	GBIF: https://doi.org/10.15468/dl.zfaw79
Anopheles amictus	145.083298	-28.549999	Unclassified	GBIF: https://doi.org/10.15468/dl.zfaw79
Anopheles amictus	145.0833	-28.55	Unclassified	GBIF: https://doi.org/10.15468/dl.zfaw79
Anopheles amictus	145.43333	-17	Unclassified	GBIF: https://doi.org/10.15468/dl.zfaw79
Anopheles amictus	145.433334	-17	Unclassified	GBIF: https://doi.org/10.15468/dl.zfaw79
Anopheles amictus	145.46666	-24.433332	Unclassified	GBIF: https://doi.org/10.15468/dl.zfaw79
Anopheles amictus	145.46666	-24.43333	Unclassified	GBIF: https://doi.org/10.15468/dl.zfaw79
Anopheles amictus	145.68333	-28.06667	Unclassified	GBIF: https://doi.org/10.15468/dl.zfaw79
Anopheles amictus	145.683334	-28.066668	Unclassified	GBIF: https://doi.org/10.15468/dl.zfaw79
Anopheles amictus	145.76666	-16.91667	Unclassified	GBIF: https://doi.org/10.15468/dl.zfaw79
Anopheles amictus	145.766663	-16.916666	Unclassified	GBIF: https://doi.org/10.15468/dl.zfaw79
Anopheles amictus	145.78334	-28.6	Unclassified	GBIF: https://doi.org/10.15468/dl.zfaw79
Anopheles amictus	145.78334	-28.583334	Unclassified	GBIF: https://doi.org/10.15468/dl.zfaw79
Anopheles amictus	145.78334	-28.58333	Unclassified	GBIF: https://doi.org/10.15468/dl.zfaw79
Anopheles amictus	146.083328	-18.633333	Unclassified	GBIF: https://doi.org/10.15468/dl.zfaw79
Anopheles amictus	146.08333	-18.63333	Unclassified	GBIF: https://doi.org/10.15468/dl.zfaw79
Anopheles amictus	146.199997	-28.233334	Unclassified	GBIF: https://doi.org/10.15468/dl.zfaw79
Anopheles amictus	146.2	-28.23333	Unclassified	GBIF: https://doi.org/10.15468/dl.zfaw79
Anopheles amictus	146.25	-26.4	Unclassified	GBIF: https://doi.org/10.15468/dl.zfaw79
Anopheles amictus	146.25	-18.583334	Unclassified	GBIF: https://doi.org/10.15468/dl.zfaw79
Anopheles amictus	146.25	-18.58333	Unclassified	GBIF: https://doi.org/10.15468/dl.zfaw79
Anopheles amictus	146.76666	-19.26667	Unclassified	GBIF: https://doi.org/10.15468/dl.zfaw79
Anopheles amictus	146.766663	-19.266666	Unclassified	GBIF: https://doi.org/10.15468/dl.zfaw79
Anopheles amictus	146.800833	-19.282777	Unclassified	GBIF: https://doi.org/10.15468/dl.zfaw79
Anopheles amictus	146.816666	-19.266666	Unclassified	GBIF: https://doi.org/10.15468/dl.zfaw79
Anopheles amictus	146.816666	-19.26667	Unclassified	GBIF: https://doi.org/10.15468/dl.zfaw79
Anopheles amictus	146.81667	-19.26667	Unclassified	GBIF: https://doi.org/10.15468/dl.zfaw79

Anopheles amictus	146.816696	-19.26667	Unclassified	GBIF: https://doi.org/10.15468/dl.zfaw79
Anopheles amictus	146.8167	-19.26667	Unclassified	GBIF: https://doi.org/10.15468/dl.zfaw79
Anopheles amictus	146.81693	-19.25983	Unclassified	GBIF: https://doi.org/10.15468/dl.zfaw79
Anopheles amictus	148.16667	-20.01667	Unclassified	GBIF: https://doi.org/10.15468/dl.zfaw79
Anopheles amictus	148.166672	-20.016666	Unclassified	GBIF: https://doi.org/10.15468/dl.zfaw79
Anopheles amictus	148.39999	-25.25	Unclassified	GBIF: https://doi.org/10.15468/dl.zfaw79
Anopheles amictus	148.399994	-25.25	Unclassified	GBIF: https://doi.org/10.15468/dl.zfaw79
Anopheles amictus	150.63333	-26.73333	Unclassified	GBIF: https://doi.org/10.15468/dl.zfaw79
Anopheles amictus	150.633331	-26.733334	Unclassified	GBIF: https://doi.org/10.15468/dl.zfaw79
Anopheles amictus	150.76666	-28.9	Unclassified	GBIF: https://doi.org/10.15468/dl.zfaw79
Anopheles amictus	150.766663	-28.9	Unclassified	GBIF: https://doi.org/10.15468/dl.zfaw79
Anopheles amictus	151.116669	-25.366667	Unclassified	GBIF: https://doi.org/10.15468/dl.zfaw79
Anopheles amictus	151.11667	-25.36667	Unclassified	GBIF: https://doi.org/10.15468/dl.zfaw79
Anopheles amictus	152.88333	-27.4	Unclassified	GBIF: https://doi.org/10.15468/dl.zfaw79
Anopheles amictus	152.883331	-27.4	Unclassified	GBIF: https://doi.org/10.15468/dl.zfaw79
Anopheles amictus	153.01666	-27.4	Unclassified	GBIF: https://doi.org/10.15468/dl.zfaw79
Anopheles amictus	153.016663	-27.4	Unclassified	GBIF: https://doi.org/10.15468/dl.zfaw79
Anopheles amictus	153.18333	-27.46667	Unclassified	GBIF: https://doi.org/10.15468/dl.zfaw79
Anopheles amictus	153.183334	-27.466667	Unclassified	GBIF: https://doi.org/10.15468/dl.zfaw79
Anopheles amictus	153.418314	-27.973794	Unclassified	GBIF: https://doi.org/10.15468/dl.zfaw79
Anopheles annandalei	106.937306	-6.685415	Unclassified	GBIF: https://doi.org/10.15468/dl.zfaw79
Anopheles annandalei	107.79262	-7.406426	Unclassified	GBIF: https://doi.org/10.15468/dl.zfaw79
Anopheles annularis	71.48	34.01	Unclassified	GBIF: https://doi.org/10.15468/dl.zfaw79
Anopheles annularis	71.49	34	Unclassified	GBIF: https://doi.org/10.15468/dl.zfaw79
Anopheles annularis	81.173004	23.5863	Unclassified	GBIF: https://doi.org/10.15468/dl.zfaw79
Anopheles annularis	83.188455	28.090779	Unclassified	GBIF: https://doi.org/10.15468/dl.zfaw79
Anopheles annularis	83.189326	28.091636	Unclassified	GBIF: https://doi.org/10.15468/dl.zfaw79
Anopheles annularis	86.05	23.26	Unclassified	GBIF: https://doi.org/10.15468/dl.zfaw79
Anopheles annularis	97.826148	18.754803	Unclassified	GBIF: https://doi.org/10.15468/dl.zfaw79
Anopheles annularis	97.878886	18.378295	Unclassified	GBIF: https://doi.org/10.15468/dl.zfaw79
Anopheles annularis	97.982849	18.328586	Unclassified	GBIF: https://doi.org/10.15468/dl.zfaw79
Anopheles annularis	98.009414	17.54756	Unclassified	GBIF: https://doi.org/10.15468/dl.zfaw79
Anopheles annularis	98.019926	17.867552	Unclassified	GBIF: https://doi.org/10.15468/dl.zfaw79
Anopheles annularis	98.085099	18.581833	Unclassified	GBIF: https://doi.org/10.15468/dl.zfaw79
Anopheles annularis	98.143289	17.358119	Unclassified	GBIF: https://doi.org/10.15468/dl.zfaw79
Anopheles annularis	98.20844	17.865638	Unclassified	GBIF: https://doi.org/10.15468/dl.zfaw79
Anopheles annularis	98.256188	17.311012	Unclassified	GBIF: https://doi.org/10.15468/dl.zfaw79
Anopheles annularis	98.31223	17.822893	Unclassified	GBIF: https://doi.org/10.15468/dl.zfaw79
Anopheles annularis	98.459221	18.420279	Unclassified	GBIF: https://doi.org/10.15468/dl.zfaw79
Anopheles annularis	98.490191	17.788002	Unclassified	GBIF: https://doi.org/10.15468/dl.zfaw79
Anopheles annularis	98.590765	8.555189	Unclassified	GBIF: https://doi.org/10.15468/dl.zfaw79
Anopheles annularis	98.656019	16.371895	Unclassified	GBIF: https://doi.org/10.15468/dl.zfaw79

Anopheles annularis	98.710833	9.550218	Unclassified	GBIF: https://doi.org/10.15468/dl.zfaw79
Anopheles annularis	98.785	1.736667	Unclassified	GBIF: https://doi.org/10.15468/dl.zfaw79
Anopheles annularis	98.90558	18.91036	Unclassified	GBIF: https://doi.org/10.15468/dl.zfaw79
Anopheles annularis	98.96256	18.87514	Unclassified	GBIF: https://doi.org/10.15468/dl.zfaw79
Anopheles annularis	99.2725	1.378056	Unclassified	GBIF: https://doi.org/10.15468/dl.zfaw79
Anopheles annularis	99.359444	0.798056	Unclassified	GBIF: https://doi.org/10.15468/dl.zfaw79
Anopheles annularis	99.540469	20.259504	Unclassified	GBIF: https://doi.org/10.15468/dl.zfaw79
Anopheles annularis	99.645557	12.7855	Unclassified	GBIF: https://doi.org/10.15468/dl.zfaw79
Anopheles annularis	99.670083	13.218098	Unclassified	GBIF: https://doi.org/10.15468/dl.zfaw79
Anopheles annularis	99.687316	13.229365	Unclassified	GBIF: https://doi.org/10.15468/dl.zfaw79
Anopheles annularis	99.80425	18.98456	Unclassified	GBIF: https://doi.org/10.15468/dl.zfaw79
Anopheles annularis	99.86834	18.51434	Unclassified	GBIF: https://doi.org/10.15468/dl.zfaw79
Anopheles annularis	99.89557	19.00764	Unclassified	GBIF: https://doi.org/10.15468/dl.zfaw79
Anopheles annularis	99.92605	18.67673	Unclassified	GBIF: https://doi.org/10.15468/dl.zfaw79
Anopheles annularis	99.93536	9.53653	Unclassified	GBIF: https://doi.org/10.15468/dl.zfaw79
Anopheles annularis	99.93694	18.92971	Unclassified	GBIF: https://doi.org/10.15468/dl.zfaw79
Anopheles annularis	99.94302	18.82847	Unclassified	GBIF: https://doi.org/10.15468/dl.zfaw79
Anopheles annularis	99.94396	18.82756	Unclassified	GBIF: https://doi.org/10.15468/dl.zfaw79
Anopheles annularis	99.94491	18.82756	Unclassified	GBIF: https://doi.org/10.15468/dl.zfaw79
Anopheles annularis	100.129167	0.186111	Unclassified	GBIF: https://doi.org/10.15468/dl.zfaw79
Anopheles annularis	100.3667	-0.3167	Unclassified	GBIF: https://doi.org/10.15468/dl.zfaw79
Anopheles annularis	100.3675	-0.300278	Unclassified	GBIF: https://doi.org/10.15468/dl.zfaw79
Anopheles annularis	100.508752	20.071488	Unclassified	GBIF: https://doi.org/10.15468/dl.zfaw79
Anopheles annularis	100.53429	14.06823	Unclassified	GBIF: https://doi.org/10.15468/dl.zfaw79
Anopheles annularis	100.72826	14.61101	Unclassified	GBIF: https://doi.org/10.15468/dl.zfaw79
Anopheles annularis	100.85772	14.66424	Unclassified	GBIF: https://doi.org/10.15468/dl.zfaw79
Anopheles annularis	100.89854	14.6612	Unclassified	GBIF: https://doi.org/10.15468/dl.zfaw79
Anopheles annularis	100.95415	14.01007	Unclassified	GBIF: https://doi.org/10.15468/dl.zfaw79
Anopheles annularis	101.025167	16.866491	Unclassified	GBIF: https://doi.org/10.15468/dl.zfaw79
Anopheles annularis	101.501371	13.239733	Unclassified	GBIF: https://doi.org/10.15468/dl.zfaw79
Anopheles annularis	102.02336	12.60527	Unclassified	GBIF: https://doi.org/10.15468/dl.zfaw79
Anopheles annularis	102.03245	12.6144	Unclassified	GBIF: https://doi.org/10.15468/dl.zfaw79
Anopheles annularis	102.25934	16.63674	Unclassified	GBIF: https://doi.org/10.15468/dl.zfaw79
Anopheles annularis	102.34243	16.69156	Unclassified	GBIF: https://doi.org/10.15468/dl.zfaw79
Anopheles annularis	102.35176	16.72826	Unclassified	GBIF: https://doi.org/10.15468/dl.zfaw79
Anopheles annularis	102.574818	14.044675	Unclassified	GBIF: https://doi.org/10.15468/dl.zfaw79
Anopheles annularis	103.38384	15.05659	Unclassified	GBIF: https://doi.org/10.15468/dl.zfaw79
Anopheles annularis	103.38385	15.05478	Unclassified	GBIF: https://doi.org/10.15468/dl.zfaw79
Anopheles annularis	103.38386	15.05388	Unclassified	GBIF: https://doi.org/10.15468/dl.zfaw79
Anopheles annularis	103.42348	15.10387	Unclassified	GBIF: https://doi.org/10.15468/dl.zfaw79
Anopheles annularis	104.452058	14.487193	Unclassified	GBIF: https://doi.org/10.15468/dl.zfaw79
Anopheles annularis	106.807378	-6.172488	Unclassified	GBIF: https://doi.org/10.15468/dl.zfaw79

Anopheles annularis	106.845278	-6.204167	Unclassified	GBIF: https://doi.org/10.15468/dl.zfaw79
Anopheles annularis	106.932333	-6.680078	Unclassified	GBIF: https://doi.org/10.15468/dl.zfaw79
Anopheles annularis	106.937306	-6.685415	Unclassified	GBIF: https://doi.org/10.15468/dl.zfaw79
Anopheles annularis	107.02	13.55	Unclassified	GBIF: https://doi.org/10.15468/dl.zfaw79
Anopheles annularis	107.02456	13.54764	Unclassified	GBIF: https://doi.org/10.15468/dl.zfaw79
Anopheles annularis	108.216667	-7.349722	Unclassified	GBIF: https://doi.org/10.15468/dl.zfaw79
Anopheles annularis	108.232753	-7.359982	Unclassified	GBIF: https://doi.org/10.15468/dl.zfaw79
Anopheles annularis	108.59	13.37	Unclassified	GBIF: https://doi.org/10.15468/dl.zfaw79
Anopheles annularis	109.023056	-7.697778	Unclassified	GBIF: https://doi.org/10.15468/dl.zfaw79
Anopheles annularis	109.107279	-7.667544	Unclassified	GBIF: https://doi.org/10.15468/dl.zfaw79
Anopheles annularis	109.13714	-7.681584	Unclassified	GBIF: https://doi.org/10.15468/dl.zfaw79
Anopheles annularis	109.146389	-7.684283	Unclassified	GBIF: https://doi.org/10.15468/dl.zfaw79
Anopheles annularis	109.173139	-7.53588	Unclassified	GBIF: https://doi.org/10.15468/dl.zfaw79
Anopheles annularis	110.217656	-7.479992	Unclassified	GBIF: https://doi.org/10.15468/dl.zfaw79
Anopheles annularis	110.218997	-7.46058	Unclassified	GBIF: https://doi.org/10.15468/dl.zfaw79
Anopheles annularis	110.225311	-7.455138	Unclassified	GBIF: https://doi.org/10.15468/dl.zfaw79
Anopheles annularis	110.818812	-7.559209	Unclassified	GBIF: https://doi.org/10.15468/dl.zfaw79
Anopheles annularis	112.321944	-7.621667	Unclassified	GBIF: https://doi.org/10.15468/dl.zfaw79
Anopheles annularis	120.033108	15.28533	Unclassified	GBIF: https://doi.org/10.15468/dl.zfaw79
Anopheles annularis	120.112096	15.298921	Unclassified	GBIF: https://doi.org/10.15468/dl.zfaw79
Anopheles annularis	120.138846	15.288106	Unclassified	GBIF: https://doi.org/10.15468/dl.zfaw79
Anopheles annularis	120.160158	15.304059	Unclassified	GBIF: https://doi.org/10.15468/dl.zfaw79
Anopheles annularis	120.181611	-9.738741	Unclassified	GBIF: https://doi.org/10.15468/dl.zfaw79
Anopheles annularis	120.467506	-8.612898	Unclassified	GBIF: https://doi.org/10.15468/dl.zfaw79
Anopheles annularis	120.827407	23.714012	Unclassified	GBIF: https://doi.org/10.15468/dl.zfaw79
Anopheles annularis	120.880218	23.709715	Unclassified	GBIF: https://doi.org/10.15468/dl.zfaw79
Anopheles annularis	120.926424	23.646976	Unclassified	GBIF: https://doi.org/10.15468/dl.zfaw79
Anopheles annularis	121	16	Unclassified	GBIF: https://doi.org/10.15468/dl.zfaw79
Anopheles annularis	121.065918	12.347433	Unclassified	GBIF: https://doi.org/10.15468/dl.zfaw79
Anopheles annularis	121.120076	14.626434	Unclassified	GBIF: https://doi.org/10.15468/dl.zfaw79
Anopheles annularis	121.123808	14.624468	Unclassified	GBIF: https://doi.org/10.15468/dl.zfaw79
Anopheles annularis	121.124007	14.626103	Unclassified	GBIF: https://doi.org/10.15468/dl.zfaw79
Anopheles annularis	121.12454	14.625388	Unclassified	GBIF: https://doi.org/10.15468/dl.zfaw79
Anopheles annularis	121.12533	14.627316	Unclassified	GBIF: https://doi.org/10.15468/dl.zfaw79
Anopheles annularis	121.125341	14.625234	Unclassified	GBIF: https://doi.org/10.15468/dl.zfaw79
Anopheles annularis	121.127102	14.626149	Unclassified	GBIF: https://doi.org/10.15468/dl.zfaw79
Anopheles annularis	121.3833	14.4167	Unclassified	GBIF: https://doi.org/10.15468/dl.zfaw79
Anopheles annularis	131.222984	-4.015743	Unclassified	GBIF: https://doi.org/10.15468/dl.zfaw79
Anopheles annulatus	146.81667	-19.26667	Unclassified	GBIF: https://doi.org/10.15468/dl.zfaw79
Anopheles annulatus	146.8167	-19.26667	Unclassified	GBIF: https://doi.org/10.15468/dl.zfaw79
Anopheles annulipalpis	-59.520834	-33.796669	Unclassified	GBIF: https://doi.org/10.15468/dl.zfaw79
Anopheles annulipes	113.979	-23.868	Unclassified	GBIF: https://doi.org/10.15468/dl.zfaw79

Anopheles annulipes	113.980253	-23.866493	Unclassified	GBIF: https://doi.org/10.15468/dl.zfaw79
Anopheles annulipes	114.136	-25.756	Unclassified	GBIF: https://doi.org/10.15468/dl.zfaw79
Anopheles annulipes	114.13694	-25.755121	Unclassified	GBIF: https://doi.org/10.15468/dl.zfaw79
Anopheles annulipes	114.193	-26.428	Unclassified	GBIF: https://doi.org/10.15468/dl.zfaw79
Anopheles annulipes	114.193893	-26.426516	Unclassified	GBIF: https://doi.org/10.15468/dl.zfaw79
Anopheles annulipes	114.302	-24.472	Unclassified	GBIF: https://doi.org/10.15468/dl.zfaw79
Anopheles annulipes	114.303593	-24.470663	Unclassified	GBIF: https://doi.org/10.15468/dl.zfaw79
Anopheles annulipes	114.422	-25.658	Unclassified	GBIF: https://doi.org/10.15468/dl.zfaw79
Anopheles annulipes	114.423606	-25.657062	Unclassified	GBIF: https://doi.org/10.15468/dl.zfaw79
Anopheles annulipes	114.548	-24.428	Unclassified	GBIF: https://doi.org/10.15468/dl.zfaw79
Anopheles annulipes	114.549703	-24.426771	Unclassified	GBIF: https://doi.org/10.15468/dl.zfaw79
Anopheles annulipes	114.841	-24.302	Unclassified	GBIF: https://doi.org/10.15468/dl.zfaw79
Anopheles annulipes	114.842479	-24.300656	Unclassified	GBIF: https://doi.org/10.15468/dl.zfaw79
Anopheles annulipes	114.848	-25.665	Unclassified	GBIF: https://doi.org/10.15468/dl.zfaw79
Anopheles annulipes	114.849717	-25.663725	Unclassified	GBIF: https://doi.org/10.15468/dl.zfaw79
Anopheles annulipes	114.87	-24.244	Unclassified	GBIF: https://doi.org/10.15468/dl.zfaw79
Anopheles annulipes	114.87109	-24.243156	Unclassified	GBIF: https://doi.org/10.15468/dl.zfaw79
Anopheles annulipes	114.938	-24.774	Unclassified	GBIF: https://doi.org/10.15468/dl.zfaw79
Anopheles annulipes	114.939429	-24.77316	Unclassified	GBIF: https://doi.org/10.15468/dl.zfaw79
Anopheles annulipes	114.948	-25.009	Unclassified	GBIF: https://doi.org/10.15468/dl.zfaw79
Anopheles annulipes	114.949153	-25.007606	Unclassified	GBIF: https://doi.org/10.15468/dl.zfaw79
Anopheles annulipes	114.958	-24.891	Unclassified	GBIF: https://doi.org/10.15468/dl.zfaw79
Anopheles annulipes	114.95943	-24.889272	Unclassified	GBIF: https://doi.org/10.15468/dl.zfaw79
Anopheles annulipes	115.03	-25.071	Unclassified	GBIF: https://doi.org/10.15468/dl.zfaw79
Anopheles annulipes	115.031376	-25.070106	Unclassified	GBIF: https://doi.org/10.15468/dl.zfaw79
Anopheles annulipes	115.116	-25.741	Unclassified	GBIF: https://doi.org/10.15468/dl.zfaw79
Anopheles annulipes	115.117773	-25.740112	Unclassified	GBIF: https://doi.org/10.15468/dl.zfaw79
Anopheles annulipes	115.291	-25.792	Unclassified	GBIF: https://doi.org/10.15468/dl.zfaw79
Anopheles annulipes	115.29194	-25.790666	Unclassified	GBIF: https://doi.org/10.15468/dl.zfaw79
Anopheles annulipes	115.333336	-33.650002	Unclassified	GBIF: https://doi.org/10.15468/dl.zfaw79
Anopheles annulipes	115.33334	-33.65	Unclassified	GBIF: https://doi.org/10.15468/dl.zfaw79
Anopheles annulipes	115.35	-33.65	Unclassified	GBIF: https://doi.org/10.15468/dl.zfaw79
Anopheles annulipes	115.3833	-33.65	Unclassified	GBIF: https://doi.org/10.15468/dl.zfaw79
Anopheles annulipes	115.383301	-33.650002	Unclassified	GBIF: https://doi.org/10.15468/dl.zfaw79
Anopheles annulipes	115.502	-25.877	Unclassified	GBIF: https://doi.org/10.15468/dl.zfaw79
Anopheles annulipes	115.503718	-25.87522	Unclassified	GBIF: https://doi.org/10.15468/dl.zfaw79
Anopheles annulipes	115.524	-25.626	Unclassified	GBIF: https://doi.org/10.15468/dl.zfaw79
Anopheles annulipes	115.525826	-25.624829	Unclassified	GBIF: https://doi.org/10.15468/dl.zfaw79
Anopheles annulipes	115.531	-25.863	Unclassified	GBIF: https://doi.org/10.15468/dl.zfaw79
Anopheles annulipes	115.532774	-25.86122	Unclassified	GBIF: https://doi.org/10.15468/dl.zfaw79
Anopheles annulipes	115.6333	-31.11667	Unclassified	GBIF: https://doi.org/10.15468/dl.zfaw79
Anopheles annulipes	115.633301	-31.116671	Unclassified	GBIF: https://doi.org/10.15468/dl.zfaw79

Anopheles annulipes	115.728616	-33.246032	Unclassified	GBIF: https://doi.org/10.15468/dl.zfaw79
Anopheles annulipes	115.814217	-31.926708	Unclassified	GBIF: https://doi.org/10.15468/dl.zfaw79
Anopheles annulipes	115.849998	-31.950001	Unclassified	GBIF: https://doi.org/10.15468/dl.zfaw79
Anopheles annulipes	115.85	-31.95	Unclassified	GBIF: https://doi.org/10.15468/dl.zfaw79
Anopheles annulipes	115.97083	-31.91278	Unclassified	GBIF: https://doi.org/10.15468/dl.zfaw79
Anopheles annulipes	115.970833	-31.912777	Unclassified	GBIF: https://doi.org/10.15468/dl.zfaw79
Anopheles annulipes	116	-31.883333	Unclassified	GBIF: https://doi.org/10.15468/dl.zfaw79
Anopheles annulipes	116	-31.88333	Unclassified	GBIF: https://doi.org/10.15468/dl.zfaw79
Anopheles annulipes	116.03333	-34.53333	Unclassified	GBIF: https://doi.org/10.15468/dl.zfaw79
Anopheles annulipes	116.033333	-34.533333	Unclassified	GBIF: https://doi.org/10.15468/dl.zfaw79
Anopheles annulipes	116.389168	-33.834446	Unclassified	GBIF: https://doi.org/10.15468/dl.zfaw79
Anopheles annulipes	116.38917	-33.83445	Unclassified	GBIF: https://doi.org/10.15468/dl.zfaw79
Anopheles annulipes	116.8333	-31.6333	Unclassified	GBIF: https://doi.org/10.15468/dl.zfaw79
Anopheles annulipes	116.93	-31.62	Unclassified	GBIF: https://doi.org/10.15468/dl.zfaw79
Anopheles annulipes	117.026	-21.566	Unclassified	GBIF: https://doi.org/10.15468/dl.zfaw79
Anopheles annulipes	117.066666	-21.583334	Unclassified	GBIF: https://doi.org/10.15468/dl.zfaw79
Anopheles annulipes	117.066667	-21.583333	Unclassified	GBIF: https://doi.org/10.15468/dl.zfaw79
Anopheles annulipes	117.06667	-21.58333	Unclassified	GBIF: https://doi.org/10.15468/dl.zfaw79
Anopheles annulipes	118.966667	-31.333334	Unclassified	GBIF: https://doi.org/10.15468/dl.zfaw79
Anopheles annulipes	118.96667	-31.33333	Unclassified	GBIF: https://doi.org/10.15468/dl.zfaw79
Anopheles annulipes	119.75	-21.166666	Unclassified	GBIF: https://doi.org/10.15468/dl.zfaw79
Anopheles annulipes	119.75	-21.16667	Unclassified	GBIF: https://doi.org/10.15468/dl.zfaw79
Anopheles annulipes	122.399719	-28.624722	Unclassified	GBIF: https://doi.org/10.15468/dl.zfaw79
Anopheles annulipes	122.39972	-28.62472	Unclassified	GBIF: https://doi.org/10.15468/dl.zfaw79
Anopheles annulipes	123.6	-17.78	Unclassified	GBIF: https://doi.org/10.15468/dl.zfaw79
Anopheles annulipes	123.63333	-17.31667	Unclassified	GBIF: https://doi.org/10.15468/dl.zfaw79
Anopheles annulipes	123.633331	-17.316668	Unclassified	GBIF: https://doi.org/10.15468/dl.zfaw79
Anopheles annulipes	126.1	-17.36667	Unclassified	GBIF: https://doi.org/10.15468/dl.zfaw79
Anopheles annulipes	128.100006	-15.466666	Unclassified	GBIF: https://doi.org/10.15468/dl.zfaw79
Anopheles annulipes	128.10001	-15.46667	Unclassified	GBIF: https://doi.org/10.15468/dl.zfaw79
Anopheles annulipes	128.35	-15.5	Unclassified	GBIF: https://doi.org/10.15468/dl.zfaw79
Anopheles annulipes	128.73	-15.77	Unclassified	GBIF: https://doi.org/10.15468/dl.zfaw79
Anopheles annulipes	130.449997	-11.3	Unclassified	GBIF: https://doi.org/10.15468/dl.zfaw79
Anopheles annulipes	130.45	-11.3	Unclassified	GBIF: https://doi.org/10.15468/dl.zfaw79
Anopheles annulipes	130.733337	-25.299999	Unclassified	GBIF: https://doi.org/10.15468/dl.zfaw79
Anopheles annulipes	130.73334	-25.3	Unclassified	GBIF: https://doi.org/10.15468/dl.zfaw79
Anopheles annulipes	130.828	-12.452	Unclassified	GBIF: https://doi.org/10.15468/dl.zfaw79
Anopheles annulipes	130.85	-12.384	Unclassified	GBIF: https://doi.org/10.15468/dl.zfaw79
Anopheles annulipes	130.850006	-12.416667	Unclassified	GBIF: https://doi.org/10.15468/dl.zfaw79
Anopheles annulipes	130.8667	-12.45	Unclassified	GBIF: https://doi.org/10.15468/dl.zfaw79
Anopheles annulipes	130.899994	-12.4	Unclassified	GBIF: https://doi.org/10.15468/dl.zfaw79
Anopheles annulipes	130.91667	-12.41667	Unclassified	GBIF: https://doi.org/10.15468/dl.zfaw79

Anopheles annulipes	130.97	-12.7	Unclassified	GBIF: https://doi.org/10.15468/dl.zfaw79
Anopheles annulipes	130.983333	-12.466667	Unclassified	GBIF: https://doi.org/10.15468/dl.zfaw79
Anopheles annulipes	131	-12.983334	Unclassified	GBIF: https://doi.org/10.15468/dl.zfaw79
Anopheles annulipes	131	-12.98333	Unclassified	GBIF: https://doi.org/10.15468/dl.zfaw79
Anopheles annulipes	131.03334	-13.066667	Unclassified	GBIF: https://doi.org/10.15468/dl.zfaw79
Anopheles annulipes	131.03334	-13.06667	Unclassified	GBIF: https://doi.org/10.15468/dl.zfaw79
Anopheles annulipes	131.100006	-13.233334	Unclassified	GBIF: https://doi.org/10.15468/dl.zfaw79
Anopheles annulipes	131.10001	-13.23333	Unclassified	GBIF: https://doi.org/10.15468/dl.zfaw79
Anopheles annulipes	131.26669	-23.26667	Unclassified	GBIF: https://doi.org/10.15468/dl.zfaw79
Anopheles annulipes	131.266693	-23.26667	Unclassified	GBIF: https://doi.org/10.15468/dl.zfaw79
Anopheles annulipes	132.03334	-13.516666	Unclassified	GBIF: https://doi.org/10.15468/dl.zfaw79
Anopheles annulipes	132.03334	-13.51667	Unclassified	GBIF: https://doi.org/10.15468/dl.zfaw79
Anopheles annulipes	132.26669	-14.46667	Unclassified	GBIF: https://doi.org/10.15468/dl.zfaw79
Anopheles annulipes	132.266693	-14.46667	Unclassified	GBIF: https://doi.org/10.15468/dl.zfaw79
Anopheles annulipes	132.366669	-13.35	Unclassified	GBIF: https://doi.org/10.15468/dl.zfaw79
Anopheles annulipes	132.36667	-13.35	Unclassified	GBIF: https://doi.org/10.15468/dl.zfaw79
Anopheles annulipes	132.66667	-24.05	Unclassified	GBIF: https://doi.org/10.15468/dl.zfaw79
Anopheles annulipes	132.666672	-24.049999	Unclassified	GBIF: https://doi.org/10.15468/dl.zfaw79
Anopheles annulipes	132.699997	-24.049999	Unclassified	GBIF: https://doi.org/10.15468/dl.zfaw79
Anopheles annulipes	132.7	-24.05	Unclassified	GBIF: https://doi.org/10.15468/dl.zfaw79
Anopheles annulipes	132.72	-23.62	Unclassified	GBIF: https://doi.org/10.15468/dl.zfaw79
Anopheles annulipes	132.745695	-24.057084	Unclassified	GBIF: https://doi.org/10.15468/dl.zfaw79
Anopheles annulipes	132.817	-14.483	Unclassified	GBIF: https://doi.org/10.15468/dl.zfaw79
Anopheles annulipes	132.817001	-14.483	Unclassified	GBIF: https://doi.org/10.15468/dl.zfaw79
Anopheles annulipes	132.866699	-12.58333	Unclassified	GBIF: https://doi.org/10.15468/dl.zfaw79
Anopheles annulipes	132.8667	-12.58333	Unclassified	GBIF: https://doi.org/10.15468/dl.zfaw79
Anopheles annulipes	133.08	-13.08	Unclassified	GBIF: https://doi.org/10.15468/dl.zfaw79
Anopheles annulipes	133.866669	-23.700001	Unclassified	GBIF: https://doi.org/10.15468/dl.zfaw79
Anopheles annulipes	133.86667	-23.7	Unclassified	GBIF: https://doi.org/10.15468/dl.zfaw79
Anopheles annulipes	133.882996	-23.632999	Unclassified	GBIF: https://doi.org/10.15468/dl.zfaw79
Anopheles annulipes	133.883	-23.633	Unclassified	GBIF: https://doi.org/10.15468/dl.zfaw79
Anopheles annulipes	134.100006	-14.05	Unclassified	GBIF: https://doi.org/10.15468/dl.zfaw79
Anopheles annulipes	134.10001	-14.05	Unclassified	GBIF: https://doi.org/10.15468/dl.zfaw79
Anopheles annulipes	134.174625	-19.672384	Unclassified	GBIF: https://doi.org/10.15468/dl.zfaw79
Anopheles annulipes	134.33333	-14.61667	Unclassified	GBIF: https://doi.org/10.15468/dl.zfaw79
Anopheles annulipes	134.43	-24.17	Unclassified	GBIF: https://doi.org/10.15468/dl.zfaw79
Anopheles annulipes	134.733337	-14.733334	Unclassified	GBIF: https://doi.org/10.15468/dl.zfaw79
Anopheles annulipes	134.73334	-14.73333	Unclassified	GBIF: https://doi.org/10.15468/dl.zfaw79
Anopheles annulipes	135.05	-14.816	Unclassified	GBIF: https://doi.org/10.15468/dl.zfaw79
Anopheles annulipes	136.753	-12.216	Unclassified	GBIF: https://doi.org/10.15468/dl.zfaw79
Anopheles annulipes	136.76666	-12.16667	Unclassified	GBIF: https://doi.org/10.15468/dl.zfaw79
Anopheles annulipes	136.766663	-12.166667	Unclassified	GBIF: https://doi.org/10.15468/dl.zfaw79

Anopheles annulipes	137.948267	-35.8438	Unclassified	GBIF: https://doi.org/10.15468/dl.zfaw79
Anopheles annulipes	138.400703	-35.180737	Unclassified	GBIF: https://doi.org/10.15468/dl.zfaw79
Anopheles annulipes	138.400889	-35.062586	Unclassified	GBIF: https://doi.org/10.15468/dl.zfaw79
Anopheles annulipes	138.493126	-35.108465	Unclassified	GBIF: https://doi.org/10.15468/dl.zfaw79
Anopheles annulipes	138.551797	-35.039415	Unclassified	GBIF: https://doi.org/10.15468/dl.zfaw79
Anopheles annulipes	138.600006	-34.950001	Unclassified	GBIF: https://doi.org/10.15468/dl.zfaw79
Anopheles annulipes	138.60001	-34.95	Unclassified	GBIF: https://doi.org/10.15468/dl.zfaw79
Anopheles annulipes	138.65058	-34.992825	Unclassified	GBIF: https://doi.org/10.15468/dl.zfaw79
Anopheles annulipes	138.765976	-35.018681	Unclassified	GBIF: https://doi.org/10.15468/dl.zfaw79
Anopheles annulipes	138.777275	-34.872881	Unclassified	GBIF: https://doi.org/10.15468/dl.zfaw79
Anopheles annulipes	138.792876	-34.717691	Unclassified	GBIF: https://doi.org/10.15468/dl.zfaw79
Anopheles annulipes	138.992137	-35.006207	Unclassified	GBIF: https://doi.org/10.15468/dl.zfaw79
Anopheles annulipes	139.303073	-34.930166	Unclassified	GBIF: https://doi.org/10.15468/dl.zfaw79
Anopheles annulipes	139.303086	-34.930164	Unclassified	GBIF: https://doi.org/10.15468/dl.zfaw79
Anopheles annulipes	139.5	-17.75	Unclassified	GBIF: https://doi.org/10.15468/dl.zfaw79
Anopheles annulipes	139.598895	-34.5626	Unclassified	GBIF: https://doi.org/10.15468/dl.zfaw79
Anopheles annulipes	139.854197	-36.833435	Unclassified	GBIF: https://doi.org/10.15468/dl.zfaw79
Anopheles annulipes	139.884574	-36.975988	Unclassified	GBIF: https://doi.org/10.15468/dl.zfaw79
Anopheles annulipes	140.05	-20.85	Unclassified	GBIF: https://doi.org/10.15468/dl.zfaw79
Anopheles annulipes	140.050003	-20.85	Unclassified	GBIF: https://doi.org/10.15468/dl.zfaw79
Anopheles annulipes	141.116669	-25.866667	Unclassified	GBIF: https://doi.org/10.15468/dl.zfaw79
Anopheles annulipes	141.11667	-25.86667	Unclassified	GBIF: https://doi.org/10.15468/dl.zfaw79
Anopheles annulipes	141.199997	-28.299999	Unclassified	GBIF: https://doi.org/10.15468/dl.zfaw79
Anopheles annulipes	141.2	-28.3	Unclassified	GBIF: https://doi.org/10.15468/dl.zfaw79
Anopheles annulipes	141.449997	-16.65	Unclassified	GBIF: https://doi.org/10.15468/dl.zfaw79
Anopheles annulipes	141.45	-16.65	Unclassified	GBIF: https://doi.org/10.15468/dl.zfaw79
Anopheles annulipes	141.6034	-34.1847	Unclassified	GBIF: https://doi.org/10.15468/dl.zfaw79
Anopheles annulipes	141.705215	-31.089607	Unclassified	GBIF: https://doi.org/10.15468/dl.zfaw79
Anopheles annulipes	141.75	-15.466666	Unclassified	GBIF: https://doi.org/10.15468/dl.zfaw79
Anopheles annulipes	141.75	-15.47	Unclassified	GBIF: https://doi.org/10.15468/dl.zfaw79
Anopheles annulipes	141.75	-15.46667	Unclassified	GBIF: https://doi.org/10.15468/dl.zfaw79
Anopheles annulipes	141.816666	-15.633333	Unclassified	GBIF: https://doi.org/10.15468/dl.zfaw79
Anopheles annulipes	141.81667	-15.63333	Unclassified	GBIF: https://doi.org/10.15468/dl.zfaw79
Anopheles annulipes	141.88333	-12.68333	Unclassified	GBIF: https://doi.org/10.15468/dl.zfaw79
Anopheles annulipes	141.883331	-12.683333	Unclassified	GBIF: https://doi.org/10.15468/dl.zfaw79
Anopheles annulipes	141.89999	-34.1	Unclassified	GBIF: https://doi.org/10.15468/dl.zfaw79
Anopheles annulipes	141.899994	-34.099998	Unclassified	GBIF: https://doi.org/10.15468/dl.zfaw79
Anopheles annulipes	141.91667	-28.56667	Unclassified	GBIF: https://doi.org/10.15468/dl.zfaw79
Anopheles annulipes	141.916672	-28.566668	Unclassified	GBIF: https://doi.org/10.15468/dl.zfaw79
Anopheles annulipes	141.9167	-29.25	Unclassified	GBIF: https://doi.org/10.15468/dl.zfaw79
Anopheles annulipes	141.916702	-29.25	Unclassified	GBIF: https://doi.org/10.15468/dl.zfaw79
Anopheles annulipes	141.93333	-14.2	Unclassified	GBIF: https://doi.org/10.15468/dl.zfaw79

Anopheles annulipes	141.933334	-14.2	Unclassified	GBIF: https://doi.org/10.15468/dl.zfaw79
Anopheles annulipes	141.949997	-16.966667	Unclassified	GBIF: https://doi.org/10.15468/dl.zfaw79
Anopheles annulipes	141.95	-16.96667	Unclassified	GBIF: https://doi.org/10.15468/dl.zfaw79
Anopheles annulipes	141.983337	-27.4	Unclassified	GBIF: https://doi.org/10.15468/dl.zfaw79
Anopheles annulipes	141.98334	-27.4	Unclassified	GBIF: https://doi.org/10.15468/dl.zfaw79
Anopheles annulipes	141.9957	-35.4455	Unclassified	GBIF: https://doi.org/10.15468/dl.zfaw79
Anopheles annulipes	141.998043	-34.530554	Unclassified	GBIF: https://doi.org/10.15468/dl.zfaw79
Anopheles annulipes	142.020625	-36.486626	Unclassified	GBIF: https://doi.org/10.15468/dl.zfaw79
Anopheles annulipes	142.065839	-36.496659	Unclassified	GBIF: https://doi.org/10.15468/dl.zfaw79
Anopheles annulipes	142.066666	-34.166668	Unclassified	GBIF: https://doi.org/10.15468/dl.zfaw79
Anopheles annulipes	142.06667	-34.16667	Unclassified	GBIF: https://doi.org/10.15468/dl.zfaw79
Anopheles annulipes	142.078197	-36.504932	Unclassified	GBIF: https://doi.org/10.15468/dl.zfaw79
Anopheles annulipes	142.116669	-10.716666	Unclassified	GBIF: https://doi.org/10.15468/dl.zfaw79
Anopheles annulipes	142.11667	-10.71667	Unclassified	GBIF: https://doi.org/10.15468/dl.zfaw79
Anopheles annulipes	142.139641	-35.680373	Unclassified	GBIF: https://doi.org/10.15468/dl.zfaw79
Anopheles annulipes	142.15	-34.18	Unclassified	GBIF: https://doi.org/10.15468/dl.zfaw79
Anopheles annulipes	142.1625	-34.18549	Unclassified	GBIF: https://doi.org/10.15468/dl.zfaw79
Anopheles annulipes	142.16667	-34.18333	Unclassified	GBIF: https://doi.org/10.15468/dl.zfaw79
Anopheles annulipes	142.166672	-34.183334	Unclassified	GBIF: https://doi.org/10.15468/dl.zfaw79
Anopheles annulipes	142.1667	-34.18333	Unclassified	GBIF: https://doi.org/10.15468/dl.zfaw79
Anopheles annulipes	142.167967	-34.250568	Unclassified	GBIF: https://doi.org/10.15468/dl.zfaw79
Anopheles annulipes	142.169288	-34.291872	Unclassified	GBIF: https://doi.org/10.15468/dl.zfaw79
Anopheles annulipes	142.21666	-10.583333	Unclassified	GBIF: https://doi.org/10.15468/dl.zfaw79
Anopheles annulipes	142.21666	-10.58333	Unclassified	GBIF: https://doi.org/10.15468/dl.zfaw79
Anopheles annulipes	142.216995	-17.533001	Unclassified	GBIF: https://doi.org/10.15468/dl.zfaw79
Anopheles annulipes	142.217	-17.533	Unclassified	GBIF: https://doi.org/10.15468/dl.zfaw79
Anopheles annulipes	142.2597	-33.5835	Unclassified	GBIF: https://doi.org/10.15468/dl.zfaw79
Anopheles annulipes	142.28	-10.58	Unclassified	GBIF: https://doi.org/10.15468/dl.zfaw79
Anopheles annulipes	142.3	-31.28333	Unclassified	GBIF: https://doi.org/10.15468/dl.zfaw79
Anopheles annulipes	142.300003	-31.283331	Unclassified	GBIF: https://doi.org/10.15468/dl.zfaw79
Anopheles annulipes	142.45	-37.43	Unclassified	GBIF: https://doi.org/10.15468/dl.zfaw79
Anopheles annulipes	142.617342	-37.145898	Unclassified	GBIF: https://doi.org/10.15468/dl.zfaw79
Anopheles annulipes	142.618628	-37.15726	Unclassified	GBIF: https://doi.org/10.15468/dl.zfaw79
Anopheles annulipes	142.619833	-37.156687	Unclassified	GBIF: https://doi.org/10.15468/dl.zfaw79
Anopheles annulipes	142.7	-28.53333	Unclassified	GBIF: https://doi.org/10.15468/dl.zfaw79
Anopheles annulipes	142.822	-37.0254	Unclassified	GBIF: https://doi.org/10.15468/dl.zfaw79
Anopheles annulipes	142.986807	-34.807449	Unclassified	GBIF: https://doi.org/10.15468/dl.zfaw79
Anopheles annulipes	143.083328	-13.633333	Unclassified	GBIF: https://doi.org/10.15468/dl.zfaw79
Anopheles annulipes	143.08333	-13.63333	Unclassified	GBIF: https://doi.org/10.15468/dl.zfaw79
Anopheles annulipes	143.100006	-28.483299	Unclassified	GBIF: https://doi.org/10.15468/dl.zfaw79
Anopheles annulipes	143.10001	-28.4833	Unclassified	GBIF: https://doi.org/10.15468/dl.zfaw79
Anopheles annulipes	143.13333	-20.73333	Unclassified	GBIF: https://doi.org/10.15468/dl.zfaw79

Anopheles annulipes	143.133331	-20.733334	Unclassified	GBIF: https://doi.org/10.15468/dl.zfaw79
Anopheles annulipes	143.199997	-13.95	Unclassified	GBIF: https://doi.org/10.15468/dl.zfaw79
Anopheles annulipes	143.2	-13.95	Unclassified	GBIF: https://doi.org/10.15468/dl.zfaw79
Anopheles annulipes	143.21666	-13.933333	Unclassified	GBIF: https://doi.org/10.15468/dl.zfaw79
Anopheles annulipes	143.21666	-13.93333	Unclassified	GBIF: https://doi.org/10.15468/dl.zfaw79
Anopheles annulipes	143.26	-38.37	Unclassified	GBIF: https://doi.org/10.15468/dl.zfaw79
Anopheles annulipes	143.266667	-38.383333	Unclassified	GBIF: https://doi.org/10.15468/dl.zfaw79
Anopheles annulipes	143.28329	-20.51667	Unclassified	GBIF: https://doi.org/10.15468/dl.zfaw79
Anopheles annulipes	143.283295	-20.51667	Unclassified	GBIF: https://doi.org/10.15468/dl.zfaw79
Anopheles annulipes	143.3	-14.31667	Unclassified	GBIF: https://doi.org/10.15468/dl.zfaw79
Anopheles annulipes	143.300003	-14.316667	Unclassified	GBIF: https://doi.org/10.15468/dl.zfaw79
Anopheles annulipes	143.472485	-38.765772	Unclassified	GBIF: https://doi.org/10.15468/dl.zfaw79
Anopheles annulipes	143.5	-14.783334	Unclassified	GBIF: https://doi.org/10.15468/dl.zfaw79
Anopheles annulipes	143.5	-14.78333	Unclassified	GBIF: https://doi.org/10.15468/dl.zfaw79
Anopheles annulipes	143.55	-18.3	Unclassified	GBIF: https://doi.org/10.15468/dl.zfaw79
Anopheles annulipes	143.55	-35.33333	Unclassified	GBIF: https://doi.org/10.15468/dl.zfaw79
Anopheles annulipes	143.550003	-18.299999	Unclassified	GBIF: https://doi.org/10.15468/dl.zfaw79
Anopheles annulipes	143.550003	-35.333332	Unclassified	GBIF: https://doi.org/10.15468/dl.zfaw79
Anopheles annulipes	143.566	-35.333	Unclassified	GBIF: https://doi.org/10.15468/dl.zfaw79
Anopheles annulipes	143.583328	-14.733334	Unclassified	GBIF: https://doi.org/10.15468/dl.zfaw79
Anopheles annulipes	143.58333	-14.73333	Unclassified	GBIF: https://doi.org/10.15468/dl.zfaw79
Anopheles annulipes	143.866669	-27.766666	Unclassified	GBIF: https://doi.org/10.15468/dl.zfaw79
Anopheles annulipes	143.86667	-27.76667	Unclassified	GBIF: https://doi.org/10.15468/dl.zfaw79
Anopheles annulipes	143.88	-5.63	Unclassified	GBIF: https://doi.org/10.15468/dl.zfaw79
Anopheles annulipes	144	-35	Unclassified	GBIF: https://doi.org/10.15468/dl.zfaw79
Anopheles annulipes	144.05	-21.01667	Unclassified	GBIF: https://doi.org/10.15468/dl.zfaw79
Anopheles annulipes	144.050003	-21.016666	Unclassified	GBIF: https://doi.org/10.15468/dl.zfaw79
Anopheles annulipes	144.066666	-28.033333	Unclassified	GBIF: https://doi.org/10.15468/dl.zfaw79
Anopheles annulipes	144.06667	-28.03333	Unclassified	GBIF: https://doi.org/10.15468/dl.zfaw79
Anopheles annulipes	144.083328	-18.516666	Unclassified	GBIF: https://doi.org/10.15468/dl.zfaw79
Anopheles annulipes	144.08333	-18.51667	Unclassified	GBIF: https://doi.org/10.15468/dl.zfaw79
Anopheles annulipes	144.14999	-5.5	Unclassified	GBIF: https://doi.org/10.15468/dl.zfaw79
Anopheles annulipes	144.149994	-5.5	Unclassified	GBIF: https://doi.org/10.15468/dl.zfaw79
Anopheles annulipes	144.25	-28.08333	Unclassified	GBIF: https://doi.org/10.15468/dl.zfaw79
Anopheles annulipes	144.3	-18.11667	Unclassified	GBIF: https://doi.org/10.15468/dl.zfaw79
Anopheles annulipes	144.300003	-18.116667	Unclassified	GBIF: https://doi.org/10.15468/dl.zfaw79
Anopheles annulipes	144.316666	-27.516666	Unclassified	GBIF: https://doi.org/10.15468/dl.zfaw79
Anopheles annulipes	144.31667	-27.51667	Unclassified	GBIF: https://doi.org/10.15468/dl.zfaw79
Anopheles annulipes	144.322967	-38.212102	Unclassified	GBIF: https://doi.org/10.15468/dl.zfaw79
Anopheles annulipes	144.350006	-28.1	Unclassified	GBIF: https://doi.org/10.15468/dl.zfaw79
Anopheles annulipes	144.35001	-28.1	Unclassified	GBIF: https://doi.org/10.15468/dl.zfaw79
Anopheles annulipes	144.366669	-27.516666	Unclassified	GBIF: https://doi.org/10.15468/dl.zfaw79

Anopheles annulipes	144.36667	-27.51667	Unclassified	GBIF: https://doi.org/10.15468/dl.zfaw79
Anopheles annulipes	144.37	-35.97	Unclassified	GBIF: https://doi.org/10.15468/dl.zfaw79
Anopheles annulipes	144.383596	-38.153573	Unclassified	GBIF: https://doi.org/10.15468/dl.zfaw79
Anopheles annulipes	144.413353	-36.257121	Unclassified	GBIF: https://doi.org/10.15468/dl.zfaw79
Anopheles annulipes	144.449997	-27.799999	Unclassified	GBIF: https://doi.org/10.15468/dl.zfaw79
Anopheles annulipes	144.45	-27.8	Unclassified	GBIF: https://doi.org/10.15468/dl.zfaw79
Anopheles annulipes	144.55	-27.6	Unclassified	GBIF: https://doi.org/10.15468/dl.zfaw79
Anopheles annulipes	144.550003	-27.6	Unclassified	GBIF: https://doi.org/10.15468/dl.zfaw79
Anopheles annulipes	144.566	-36.25	Unclassified	GBIF: https://doi.org/10.15468/dl.zfaw79
Anopheles annulipes	144.566667	-36.25	Unclassified	GBIF: https://doi.org/10.15468/dl.zfaw79
Anopheles annulipes	144.575836	-36.256111	Unclassified	GBIF: https://doi.org/10.15468/dl.zfaw79
Anopheles annulipes	144.57584	-36.25611	Unclassified	GBIF: https://doi.org/10.15468/dl.zfaw79
Anopheles annulipes	144.583298	-27.76667	Unclassified	GBIF: https://doi.org/10.15468/dl.zfaw79
Anopheles annulipes	144.5833	-27.76667	Unclassified	GBIF: https://doi.org/10.15468/dl.zfaw79
Anopheles annulipes	144.616669	-5.8	Unclassified	GBIF: https://doi.org/10.15468/dl.zfaw79
Anopheles annulipes	144.61667	-5.8	Unclassified	GBIF: https://doi.org/10.15468/dl.zfaw79
Anopheles annulipes	144.66667	-5.91667	Unclassified	GBIF: https://doi.org/10.15468/dl.zfaw79
Anopheles annulipes	144.666672	-5.916667	Unclassified	GBIF: https://doi.org/10.15468/dl.zfaw79
Anopheles annulipes	144.6667	-27.0167	Unclassified	GBIF: https://doi.org/10.15468/dl.zfaw79
Anopheles annulipes	144.666702	-27.016701	Unclassified	GBIF: https://doi.org/10.15468/dl.zfaw79
Anopheles annulipes	144.699997	-27.799999	Unclassified	GBIF: https://doi.org/10.15468/dl.zfaw79
Anopheles annulipes	144.7	-27.8	Unclassified	GBIF: https://doi.org/10.15468/dl.zfaw79
Anopheles annulipes	144.75	-36.13	Unclassified	GBIF: https://doi.org/10.15468/dl.zfaw79
Anopheles annulipes	144.766767	-37.66766	Unclassified	GBIF: https://doi.org/10.15468/dl.zfaw79
Anopheles annulipes	144.916667	-6	Unclassified	GBIF: https://doi.org/10.15468/dl.zfaw79
Anopheles annulipes	144.91667	-6	Unclassified	GBIF: https://doi.org/10.15468/dl.zfaw79
Anopheles annulipes	144.93333	-17.35	Unclassified	GBIF: https://doi.org/10.15468/dl.zfaw79
Anopheles annulipes	144.933334	-17.35	Unclassified	GBIF: https://doi.org/10.15468/dl.zfaw79
Anopheles annulipes	144.969975	-36.630788	Unclassified	GBIF: https://doi.org/10.15468/dl.zfaw79
Anopheles annulipes	144.97	-37.82	Unclassified	GBIF: https://doi.org/10.15468/dl.zfaw79
Anopheles annulipes	144.9933	-37.886744	Unclassified	GBIF: https://doi.org/10.15468/dl.zfaw79
Anopheles annulipes	145.044998	-36.316113	Unclassified	GBIF: https://doi.org/10.15468/dl.zfaw79
Anopheles annulipes	145.045	-36.31611	Unclassified	GBIF: https://doi.org/10.15468/dl.zfaw79
Anopheles annulipes	145.053786	-37.71857	Unclassified	GBIF: https://doi.org/10.15468/dl.zfaw79
Anopheles annulipes	145.08104	-38.286999	Unclassified	GBIF: https://doi.org/10.15468/dl.zfaw79
Anopheles annulipes	145.11929	-38.182622	Unclassified	GBIF: https://doi.org/10.15468/dl.zfaw79
Anopheles annulipes	145.12122	-38.035183	Unclassified	GBIF: https://doi.org/10.15468/dl.zfaw79
Anopheles annulipes	145.125252	-38.038276	Unclassified	GBIF: https://doi.org/10.15468/dl.zfaw79
Anopheles annulipes	145.126648	-38.05458	Unclassified	GBIF: https://doi.org/10.15468/dl.zfaw79
Anopheles annulipes	145.12881	-38.039214	Unclassified	GBIF: https://doi.org/10.15468/dl.zfaw79
Anopheles annulipes	145.19768	-37.749183	Unclassified	GBIF: https://doi.org/10.15468/dl.zfaw79
Anopheles annulipes	145.2	-17.43	Unclassified	GBIF: https://doi.org/10.15468/dl.zfaw79

Anopheles annulipes	145.23	-37.82	Unclassified	GBIF: https://doi.org/10.15468/dl.zfaw79
Anopheles annulipes	145.23	-17.67	Unclassified	GBIF: https://doi.org/10.15468/dl.zfaw79
Anopheles annulipes	145.233337	-17.666666	Unclassified	GBIF: https://doi.org/10.15468/dl.zfaw79
Anopheles annulipes	145.23334	-17.66667	Unclassified	GBIF: https://doi.org/10.15468/dl.zfaw79
Anopheles annulipes	145.240336	-37.834745	Unclassified	GBIF: https://doi.org/10.15468/dl.zfaw79
Anopheles annulipes	145.25	-35.683331	Unclassified	GBIF: https://doi.org/10.15468/dl.zfaw79
Anopheles annulipes	145.25	-35.68333	Unclassified	GBIF: https://doi.org/10.15468/dl.zfaw79
Anopheles annulipes	145.316666	-16.299999	Unclassified	GBIF: https://doi.org/10.15468/dl.zfaw79
Anopheles annulipes	145.31667	-16.3	Unclassified	GBIF: https://doi.org/10.15468/dl.zfaw79
Anopheles annulipes	145.333328	-17.016666	Unclassified	GBIF: https://doi.org/10.15468/dl.zfaw79
Anopheles annulipes	145.333328	-16.683332	Unclassified	GBIF: https://doi.org/10.15468/dl.zfaw79
Anopheles annulipes	145.33333	-16.68333	Unclassified	GBIF: https://doi.org/10.15468/dl.zfaw79
Anopheles annulipes	145.33333	-17.01667	Unclassified	GBIF: https://doi.org/10.15468/dl.zfaw79
Anopheles annulipes	145.366669	-16.450001	Unclassified	GBIF: https://doi.org/10.15468/dl.zfaw79
Anopheles annulipes	145.36667	-16.45	Unclassified	GBIF: https://doi.org/10.15468/dl.zfaw79
Anopheles annulipes	145.366699	-16.816669	Unclassified	GBIF: https://doi.org/10.15468/dl.zfaw79
Anopheles annulipes	145.3667	-16.81667	Unclassified	GBIF: https://doi.org/10.15468/dl.zfaw79
Anopheles annulipes	145.37306	-37.66056	Unclassified	GBIF: https://doi.org/10.15468/dl.zfaw79
Anopheles annulipes	145.373062	-37.660557	Unclassified	GBIF: https://doi.org/10.15468/dl.zfaw79
Anopheles annulipes	145.381378	-38.103178	Unclassified	GBIF: https://doi.org/10.15468/dl.zfaw79
Anopheles annulipes	145.38333	-6.08333	Unclassified	GBIF: https://doi.org/10.15468/dl.zfaw79
Anopheles annulipes	145.383331	-6.083333	Unclassified	GBIF: https://doi.org/10.15468/dl.zfaw79
Anopheles annulipes	145.39999	-6.06667	Unclassified	GBIF: https://doi.org/10.15468/dl.zfaw79
Anopheles annulipes	145.399994	-6.066667	Unclassified	GBIF: https://doi.org/10.15468/dl.zfaw79
Anopheles annulipes	145.408759	-16.180312	Unclassified	GBIF: https://doi.org/10.15468/dl.zfaw79
Anopheles annulipes	145.411	-37.878	Unclassified	GBIF: https://doi.org/10.15468/dl.zfaw79
Anopheles annulipes	145.41667	-16.91667	Unclassified	GBIF: https://doi.org/10.15468/dl.zfaw79
Anopheles annulipes	145.416672	-16.916666	Unclassified	GBIF: https://doi.org/10.15468/dl.zfaw79
Anopheles annulipes	145.417062	-37.87392	Unclassified	GBIF: https://doi.org/10.15468/dl.zfaw79
Anopheles annulipes	145.417084	-37.873923	Unclassified	GBIF: https://doi.org/10.15468/dl.zfaw79
Anopheles annulipes	145.43	-19.22	Unclassified	GBIF: https://doi.org/10.15468/dl.zfaw79
Anopheles annulipes	145.4333	-17.1333	Unclassified	GBIF: https://doi.org/10.15468/dl.zfaw79
Anopheles annulipes	145.433304	-17.133301	Unclassified	GBIF: https://doi.org/10.15468/dl.zfaw79
Anopheles annulipes	145.449997	-16.416666	Unclassified	GBIF: https://doi.org/10.15468/dl.zfaw79
Anopheles annulipes	145.46666	-24.433332	Unclassified	GBIF: https://doi.org/10.15468/dl.zfaw79
Anopheles annulipes	145.46666	-24.43333	Unclassified	GBIF: https://doi.org/10.15468/dl.zfaw79
Anopheles annulipes	145.4833	-16.5	Unclassified	GBIF: https://doi.org/10.15468/dl.zfaw79
Anopheles annulipes	145.483337	-17.266666	Unclassified	GBIF: https://doi.org/10.15468/dl.zfaw79
Anopheles annulipes	145.48334	-17.26667	Unclassified	GBIF: https://doi.org/10.15468/dl.zfaw79
Anopheles annulipes	145.5	-26.6	Unclassified	GBIF: https://doi.org/10.15468/dl.zfaw79
Anopheles annulipes	145.52	-37.65	Unclassified	GBIF: https://doi.org/10.15468/dl.zfaw79
Anopheles annulipes	145.55	-37.62	Unclassified	GBIF: https://doi.org/10.15468/dl.zfaw79

Anopheles annulipes	145.55	-17.38333	Unclassified	GBIF: https://doi.org/10.15468/dl.zfaw79
Anopheles annulipes	145.550003	-17.383333	Unclassified	GBIF: https://doi.org/10.15468/dl.zfaw79
Anopheles annulipes	145.63333	-17.16667	Unclassified	GBIF: https://doi.org/10.15468/dl.zfaw79
Anopheles annulipes	145.633331	-17.166666	Unclassified	GBIF: https://doi.org/10.15468/dl.zfaw79
Anopheles annulipes	145.647884	-37.775243	Unclassified	GBIF: https://doi.org/10.15468/dl.zfaw79
Anopheles annulipes	145.64999	-35.91667	Unclassified	GBIF: https://doi.org/10.15468/dl.zfaw79
Anopheles annulipes	145.649994	-35.916668	Unclassified	GBIF: https://doi.org/10.15468/dl.zfaw79
Anopheles annulipes	145.68333	-28.06667	Unclassified	GBIF: https://doi.org/10.15468/dl.zfaw79
Anopheles annulipes	145.683334	-28.066668	Unclassified	GBIF: https://doi.org/10.15468/dl.zfaw79
Anopheles annulipes	145.7	-37.75	Unclassified	GBIF: https://doi.org/10.15468/dl.zfaw79
Anopheles annulipes	145.75	-5.3	Unclassified	GBIF: https://doi.org/10.15468/dl.zfaw79
Anopheles annulipes	145.76666	-17.03333	Unclassified	GBIF: https://doi.org/10.15468/dl.zfaw79
Anopheles annulipes	145.76666	-16.91667	Unclassified	GBIF: https://doi.org/10.15468/dl.zfaw79
Anopheles annulipes	145.766663	-16.916666	Unclassified	GBIF: https://doi.org/10.15468/dl.zfaw79
Anopheles annulipes	145.766663	-17.033333	Unclassified	GBIF: https://doi.org/10.15468/dl.zfaw79
Anopheles annulipes	145.7667	-16.91667	Unclassified	GBIF: https://doi.org/10.15468/dl.zfaw79
Anopheles annulipes	145.77	-19.62	Unclassified	GBIF: https://doi.org/10.15468/dl.zfaw79
Anopheles annulipes	145.78334	-28.6	Unclassified	GBIF: https://doi.org/10.15468/dl.zfaw79
Anopheles annulipes	145.866699	-6.283333	Unclassified	GBIF: https://doi.org/10.15468/dl.zfaw79
Anopheles annulipes	145.8667	-6.28333	Unclassified	GBIF: https://doi.org/10.15468/dl.zfaw79
Anopheles annulipes	145.916667	-6.333333	Unclassified	GBIF: https://doi.org/10.15468/dl.zfaw79
Anopheles annulipes	145.91667	-17.31667	Unclassified	GBIF: https://doi.org/10.15468/dl.zfaw79
Anopheles annulipes	145.91667	-6.33333	Unclassified	GBIF: https://doi.org/10.15468/dl.zfaw79
Anopheles annulipes	145.916672	-17.316668	Unclassified	GBIF: https://doi.org/10.15468/dl.zfaw79
Anopheles annulipes	145.9333	-17.1333	Unclassified	GBIF: https://doi.org/10.15468/dl.zfaw79
Anopheles annulipes	145.933304	-17.133301	Unclassified	GBIF: https://doi.org/10.15468/dl.zfaw79
Anopheles annulipes	145.949997	-30.083334	Unclassified	GBIF: https://doi.org/10.15468/dl.zfaw79
Anopheles annulipes	145.95	-30.08333	Unclassified	GBIF: https://doi.org/10.15468/dl.zfaw79
Anopheles annulipes	146.01666	-17.53333	Unclassified	GBIF: https://doi.org/10.15468/dl.zfaw79
Anopheles annulipes	146.016663	-17.533333	Unclassified	GBIF: https://doi.org/10.15468/dl.zfaw79
Anopheles annulipes	146.016667	-17.533333	Unclassified	GBIF: https://doi.org/10.15468/dl.zfaw79
Anopheles annulipes	146.03	-34.28	Unclassified	GBIF: https://doi.org/10.15468/dl.zfaw79
Anopheles annulipes	146.03334	-34.28333	Unclassified	GBIF: https://doi.org/10.15468/dl.zfaw79
Anopheles annulipes	146.083328	-18.633333	Unclassified	GBIF: https://doi.org/10.15468/dl.zfaw79
Anopheles annulipes	146.083328	-18.966667	Unclassified	GBIF: https://doi.org/10.15468/dl.zfaw79
Anopheles annulipes	146.08333	-18.63333	Unclassified	GBIF: https://doi.org/10.15468/dl.zfaw79
Anopheles annulipes	146.08333	-18.96667	Unclassified	GBIF: https://doi.org/10.15468/dl.zfaw79
Anopheles annulipes	146.116669	-17.6	Unclassified	GBIF: https://doi.org/10.15468/dl.zfaw79
Anopheles annulipes	146.11667	-17.6	Unclassified	GBIF: https://doi.org/10.15468/dl.zfaw79
Anopheles annulipes	146.25	-20.0667	Unclassified	GBIF: https://doi.org/10.15468/dl.zfaw79
Anopheles annulipes	146.25	-26.4	Unclassified	GBIF: https://doi.org/10.15468/dl.zfaw79
Anopheles annulipes	146.25	-18.583334	Unclassified	GBIF: https://doi.org/10.15468/dl.zfaw79

Anopheles annulipes	146.25	-18.58333	Unclassified	GBIF: https://doi.org/10.15468/dl.zfaw79
Anopheles annulipes	146.316696	-26.200001	Unclassified	GBIF: https://doi.org/10.15468/dl.zfaw79
Anopheles annulipes	146.3167	-26.2	Unclassified	GBIF: https://doi.org/10.15468/dl.zfaw79
Anopheles annulipes	146.387953	-38.354323	Unclassified	GBIF: https://doi.org/10.15468/dl.zfaw79
Anopheles annulipes	146.4	-36.82	Unclassified	GBIF: https://doi.org/10.15468/dl.zfaw79
Anopheles annulipes	146.53	-38.19	Unclassified	GBIF: https://doi.org/10.15468/dl.zfaw79
Anopheles annulipes	146.55	-34.75	Unclassified	GBIF: https://doi.org/10.15468/dl.zfaw79
Anopheles annulipes	146.550003	-34.75	Unclassified	GBIF: https://doi.org/10.15468/dl.zfaw79
Anopheles annulipes	146.58	-8.97	Unclassified	GBIF: https://doi.org/10.15468/dl.zfaw79
Anopheles annulipes	146.621105	-36.10808	Unclassified	GBIF: https://doi.org/10.15468/dl.zfaw79
Anopheles annulipes	146.732571	-19.361711	Unclassified	GBIF: https://doi.org/10.15468/dl.zfaw79
Anopheles annulipes	146.75	-19.43	Unclassified	GBIF: https://doi.org/10.15468/dl.zfaw79
Anopheles annulipes	146.76666	-19.26667	Unclassified	GBIF: https://doi.org/10.15468/dl.zfaw79
Anopheles annulipes	146.766663	-19.266666	Unclassified	GBIF: https://doi.org/10.15468/dl.zfaw79
Anopheles annulipes	146.785278	-41.226944	Unclassified	GBIF: https://doi.org/10.15468/dl.zfaw79
Anopheles annulipes	146.78528	-41.22694	Unclassified	GBIF: https://doi.org/10.15468/dl.zfaw79
Anopheles annulipes	146.816666	-19.266666	Unclassified	GBIF: https://doi.org/10.15468/dl.zfaw79
Anopheles annulipes	146.81667	-19.26667	Unclassified	GBIF: https://doi.org/10.15468/dl.zfaw79
Anopheles annulipes	146.816696	-19.26667	Unclassified	GBIF: https://doi.org/10.15468/dl.zfaw79
Anopheles annulipes	146.8167	-19.26667	Unclassified	GBIF: https://doi.org/10.15468/dl.zfaw79
Anopheles annulipes	146.87	-36.1	Unclassified	GBIF: https://doi.org/10.15468/dl.zfaw79
Anopheles annulipes	146.87389	-36.1	Unclassified	GBIF: https://doi.org/10.15468/dl.zfaw79
Anopheles annulipes	146.937652	-43.165848	Unclassified	GBIF: https://doi.org/10.15468/dl.zfaw79
Anopheles annulipes	147	-6.716667	Unclassified	GBIF: https://doi.org/10.15468/dl.zfaw79
Anopheles annulipes	147	-6.71667	Unclassified	GBIF: https://doi.org/10.15468/dl.zfaw79
Anopheles annulipes	147.01666	-41.36666	Unclassified	GBIF: https://doi.org/10.15468/dl.zfaw79
Anopheles annulipes	147.016663	-41.366665	Unclassified	GBIF: https://doi.org/10.15468/dl.zfaw79
Anopheles annulipes	147.05	-7.05	Unclassified	GBIF: https://doi.org/10.15468/dl.zfaw79
Anopheles annulipes	147.050003	-7.05	Unclassified	GBIF: https://doi.org/10.15468/dl.zfaw79
Anopheles annulipes	147.083298	-9.416667	Unclassified	GBIF: https://doi.org/10.15468/dl.zfaw79
Anopheles annulipes	147.0833	-9.41667	Unclassified	GBIF: https://doi.org/10.15468/dl.zfaw79
Anopheles annulipes	147.1	-9.42	Unclassified	GBIF: https://doi.org/10.15468/dl.zfaw79
Anopheles annulipes	147.1333	-9.45	Unclassified	GBIF: https://doi.org/10.15468/dl.zfaw79
Anopheles annulipes	147.133301	-9.466666	Unclassified	GBIF: https://doi.org/10.15468/dl.zfaw79
Anopheles annulipes	147.13333	-20.65	Unclassified	GBIF: https://doi.org/10.15468/dl.zfaw79
Anopheles annulipes	147.133331	-20.65	Unclassified	GBIF: https://doi.org/10.15468/dl.zfaw79
Anopheles annulipes	147.146587	-38.142207	Unclassified	GBIF: https://doi.org/10.15468/dl.zfaw79
Anopheles annulipes	147.151292	-19.479956	Unclassified	GBIF: https://doi.org/10.15468/dl.zfaw79
Anopheles annulipes	147.1667	-42.48333	Unclassified	GBIF: https://doi.org/10.15468/dl.zfaw79
Anopheles annulipes	147.166702	-42.48333	Unclassified	GBIF: https://doi.org/10.15468/dl.zfaw79
Anopheles annulipes	147.17	-19.75	Unclassified	GBIF: https://doi.org/10.15468/dl.zfaw79
Anopheles annulipes	147.177394	-9.456594	Unclassified	GBIF: https://doi.org/10.15468/dl.zfaw79

Anopheles annulipes	147.17911	-9.453208	Unclassified	GBIF: https://doi.org/10.15468/dl.zfaw79
Anopheles annulipes	147.18	-42.47	Unclassified	GBIF: https://doi.org/10.15468/dl.zfaw79
Anopheles annulipes	147.18014	-9.453885	Unclassified	GBIF: https://doi.org/10.15468/dl.zfaw79
Anopheles annulipes	147.180484	-9.455578	Unclassified	GBIF: https://doi.org/10.15468/dl.zfaw79
Anopheles annulipes	147.180484	-9.453885	Unclassified	GBIF: https://doi.org/10.15468/dl.zfaw79
Anopheles annulipes	147.183917	-9.451176	Unclassified	GBIF: https://doi.org/10.15468/dl.zfaw79
Anopheles annulipes	147.24	-38.07	Unclassified	GBIF: https://doi.org/10.15468/dl.zfaw79
Anopheles annulipes	147.24445	-38.066666	Unclassified	GBIF: https://doi.org/10.15468/dl.zfaw79
Anopheles annulipes	147.3	-41.88334	Unclassified	GBIF: https://doi.org/10.15468/dl.zfaw79
Anopheles annulipes	147.300003	-41.883335	Unclassified	GBIF: https://doi.org/10.15468/dl.zfaw79
Anopheles annulipes	147.350006	-19.549999	Unclassified	GBIF: https://doi.org/10.15468/dl.zfaw79
Anopheles annulipes	147.35001	-19.55	Unclassified	GBIF: https://doi.org/10.15468/dl.zfaw79
Anopheles annulipes	147.38	-38.02	Unclassified	GBIF: https://doi.org/10.15468/dl.zfaw79
Anopheles annulipes	147.38	-38.05	Unclassified	GBIF: https://doi.org/10.15468/dl.zfaw79
Anopheles annulipes	147.39999	-19.56667	Unclassified	GBIF: https://doi.org/10.15468/dl.zfaw79
Anopheles annulipes	147.399994	-19.566668	Unclassified	GBIF: https://doi.org/10.15468/dl.zfaw79
Anopheles annulipes	147.4106	-36.5624	Unclassified	GBIF: https://doi.org/10.15468/dl.zfaw79
Anopheles annulipes	147.46666	-19.6	Unclassified	GBIF: https://doi.org/10.15468/dl.zfaw79
Anopheles annulipes	147.46666	-26.483334	Unclassified	GBIF: https://doi.org/10.15468/dl.zfaw79
Anopheles annulipes	147.46666	-26.48333	Unclassified	GBIF: https://doi.org/10.15468/dl.zfaw79
Anopheles annulipes	147.53334	-41.049999	Unclassified	GBIF: https://doi.org/10.15468/dl.zfaw79
Anopheles annulipes	147.53334	-41.05	Unclassified	GBIF: https://doi.org/10.15468/dl.zfaw79
Anopheles annulipes	147.613	-40.893	Unclassified	GBIF: https://doi.org/10.15468/dl.zfaw79
Anopheles annulipes	147.63	-9.95	Unclassified	GBIF: https://doi.org/10.15468/dl.zfaw79
Anopheles annulipes	147.633331	-40.833332	Unclassified	GBIF: https://doi.org/10.15468/dl.zfaw79
Anopheles annulipes	147.96666	-32.033333	Unclassified	GBIF: https://doi.org/10.15468/dl.zfaw79
Anopheles annulipes	147.96666	-32.03333	Unclassified	GBIF: https://doi.org/10.15468/dl.zfaw79
Anopheles annulipes	148.100006	-31.549999	Unclassified	GBIF: https://doi.org/10.15468/dl.zfaw79
Anopheles annulipes	148.10001	-31.55	Unclassified	GBIF: https://doi.org/10.15468/dl.zfaw79
Anopheles annulipes	148.1105	-35.0011	Unclassified	GBIF: https://doi.org/10.15468/dl.zfaw79
Anopheles annulipes	148.16667	-23.51667	Unclassified	GBIF: https://doi.org/10.15468/dl.zfaw79
Anopheles annulipes	148.166672	-23.516666	Unclassified	GBIF: https://doi.org/10.15468/dl.zfaw79
Anopheles annulipes	148.25	-41.5	Unclassified	GBIF: https://doi.org/10.15468/dl.zfaw79
Anopheles annulipes	148.26666	-41.51667	Unclassified	GBIF: https://doi.org/10.15468/dl.zfaw79
Anopheles annulipes	148.266663	-41.516666	Unclassified	GBIF: https://doi.org/10.15468/dl.zfaw79
Anopheles annulipes	148.26669	-41.5	Unclassified	GBIF: https://doi.org/10.15468/dl.zfaw79
Anopheles annulipes	148.266693	-41.5	Unclassified	GBIF: https://doi.org/10.15468/dl.zfaw79
Anopheles annulipes	148.28334	-41.700001	Unclassified	GBIF: https://doi.org/10.15468/dl.zfaw79
Anopheles annulipes	148.28334	-42.133335	Unclassified	GBIF: https://doi.org/10.15468/dl.zfaw79
Anopheles annulipes	148.28334	-41.7	Unclassified	GBIF: https://doi.org/10.15468/dl.zfaw79
Anopheles annulipes	148.28334	-42.13334	Unclassified	GBIF: https://doi.org/10.15468/dl.zfaw79
Anopheles annulipes	148.316666	-21.200001	Unclassified	GBIF: https://doi.org/10.15468/dl.zfaw79

Anopheles annulipes	148.31667	-21.2	Unclassified	GBIF: https://doi.org/10.15468/dl.zfaw79
Anopheles annulipes	148.34515	-33.242773	Unclassified	GBIF: https://doi.org/10.15468/dl.zfaw79
Anopheles annulipes	148.39999	-25.25	Unclassified	GBIF: https://doi.org/10.15468/dl.zfaw79
Anopheles annulipes	148.399994	-25.25	Unclassified	GBIF: https://doi.org/10.15468/dl.zfaw79
Anopheles annulipes	148.4051	-36.7923	Unclassified	GBIF: https://doi.org/10.15468/dl.zfaw79
Anopheles annulipes	148.5	-21.13	Unclassified	GBIF: https://doi.org/10.15468/dl.zfaw79
Anopheles annulipes	148.53905	-37.50787	Unclassified	GBIF: https://doi.org/10.15468/dl.zfaw79
Anopheles annulipes	148.58	-37.07	Unclassified	GBIF: https://doi.org/10.15468/dl.zfaw79
Anopheles annulipes	148.600006	-20.583334	Unclassified	GBIF: https://doi.org/10.15468/dl.zfaw79
Anopheles annulipes	148.60001	-20.58333	Unclassified	GBIF: https://doi.org/10.15468/dl.zfaw79
Anopheles annulipes	148.63333	-21.15	Unclassified	GBIF: https://doi.org/10.15468/dl.zfaw79
Anopheles annulipes	148.633331	-21.15	Unclassified	GBIF: https://doi.org/10.15468/dl.zfaw79
Anopheles annulipes	148.6765	-33.8331	Unclassified	GBIF: https://doi.org/10.15468/dl.zfaw79
Anopheles annulipes	148.71666	-20.266666	Unclassified	GBIF: https://doi.org/10.15468/dl.zfaw79
Anopheles annulipes	148.71666	-20.26667	Unclassified	GBIF: https://doi.org/10.15468/dl.zfaw79
Anopheles annulipes	148.72	-37.7	Unclassified	GBIF: https://doi.org/10.15468/dl.zfaw79
Anopheles annulipes	148.78334	-26.583334	Unclassified	GBIF: https://doi.org/10.15468/dl.zfaw79
Anopheles annulipes	148.78334	-26.58333	Unclassified	GBIF: https://doi.org/10.15468/dl.zfaw79
Anopheles annulipes	148.8833	-28.7833	Unclassified	GBIF: https://doi.org/10.15468/dl.zfaw79
Anopheles annulipes	148.883301	-28.7833	Unclassified	GBIF: https://doi.org/10.15468/dl.zfaw79
Anopheles annulipes	148.91667	-34.83333	Unclassified	GBIF: https://doi.org/10.15468/dl.zfaw79
Anopheles annulipes	148.916672	-34.833332	Unclassified	GBIF: https://doi.org/10.15468/dl.zfaw79
Anopheles annulipes	149.12	-35.19	Unclassified	GBIF: https://doi.org/10.15468/dl.zfaw79
Anopheles annulipes	149.13333	-35.3	Unclassified	GBIF: https://doi.org/10.15468/dl.zfaw79
Anopheles annulipes	149.133331	-35.299999	Unclassified	GBIF: https://doi.org/10.15468/dl.zfaw79
Anopheles annulipes	149.15	-37.57	Unclassified	GBIF: https://doi.org/10.15468/dl.zfaw79
Anopheles annulipes	149.16	-21.04	Unclassified	GBIF: https://doi.org/10.15468/dl.zfaw79
Anopheles annulipes	149.16667	-21.73333	Unclassified	GBIF: https://doi.org/10.15468/dl.zfaw79
Anopheles annulipes	149.16667	-21.03333	Unclassified	GBIF: https://doi.org/10.15468/dl.zfaw79
Anopheles annulipes	149.166672	-21.033333	Unclassified	GBIF: https://doi.org/10.15468/dl.zfaw79
Anopheles annulipes	149.166672	-21.733334	Unclassified	GBIF: https://doi.org/10.15468/dl.zfaw79
Anopheles annulipes	149.18333	-21.1	Unclassified	GBIF: https://doi.org/10.15468/dl.zfaw79
Anopheles annulipes	149.183334	-21.1	Unclassified	GBIF: https://doi.org/10.15468/dl.zfaw79
Anopheles annulipes	149.199997	-21.133333	Unclassified	GBIF: https://doi.org/10.15468/dl.zfaw79
Anopheles annulipes	149.2	-21.13333	Unclassified	GBIF: https://doi.org/10.15468/dl.zfaw79
Anopheles annulipes	149.233337	-21.066668	Unclassified	GBIF: https://doi.org/10.15468/dl.zfaw79
Anopheles annulipes	149.489903	-36.110897	Unclassified	GBIF: https://doi.org/10.15468/dl.zfaw79
Anopheles annulipes	149.646152	-35.724831	Unclassified	GBIF: https://doi.org/10.15468/dl.zfaw79
Anopheles annulipes	149.71666	-34.75	Unclassified	GBIF: https://doi.org/10.15468/dl.zfaw79
Anopheles annulipes	149.7922	-35.6234	Unclassified	GBIF: https://doi.org/10.15468/dl.zfaw79
Anopheles annulipes	149.7925	-35.6224	Unclassified	GBIF: https://doi.org/10.15468/dl.zfaw79
Anopheles annulipes	149.7938	-35.6249	Unclassified	GBIF: https://doi.org/10.15468/dl.zfaw79

Anopheles annulipes	149.833328	-25.5	Unclassified	GBIF: https://doi.org/10.15468/dl.zfaw79
Anopheles annulipes	149.83333	-25.5	Unclassified	GBIF: https://doi.org/10.15468/dl.zfaw79
Anopheles annulipes	149.85	-33.7	Unclassified	GBIF: https://doi.org/10.15468/dl.zfaw79
Anopheles annulipes	149.8796	-35.7271	Unclassified	GBIF: https://doi.org/10.15468/dl.zfaw79
Anopheles annulipes	149.9044	-35.729	Unclassified	GBIF: https://doi.org/10.15468/dl.zfaw79
Anopheles annulipes	149.9054	-35.7292	Unclassified	GBIF: https://doi.org/10.15468/dl.zfaw79
Anopheles annulipes	149.9177	-35.7494	Unclassified	GBIF: https://doi.org/10.15468/dl.zfaw79
Anopheles annulipes	150.116669	-28.25	Unclassified	GBIF: https://doi.org/10.15468/dl.zfaw79
Anopheles annulipes	150.11667	-28.25	Unclassified	GBIF: https://doi.org/10.15468/dl.zfaw79
Anopheles annulipes	150.1285	-35.6414	Unclassified	GBIF: https://doi.org/10.15468/dl.zfaw79
Anopheles annulipes	150.13333	-36.21667	Unclassified	GBIF: https://doi.org/10.15468/dl.zfaw79
Anopheles annulipes	150.133331	-36.216667	Unclassified	GBIF: https://doi.org/10.15468/dl.zfaw79
Anopheles annulipes	150.1364	-35.6328	Unclassified	GBIF: https://doi.org/10.15468/dl.zfaw79
Anopheles annulipes	150.1369	-35.6312	Unclassified	GBIF: https://doi.org/10.15468/dl.zfaw79
Anopheles annulipes	150.1516	-35.5931	Unclassified	GBIF: https://doi.org/10.15468/dl.zfaw79
Anopheles annulipes	150.1562	-35.9315	Unclassified	GBIF: https://doi.org/10.15468/dl.zfaw79
Anopheles annulipes	150.1685	-35.7197	Unclassified	GBIF: https://doi.org/10.15468/dl.zfaw79
Anopheles annulipes	150.1791	-35.5414	Unclassified	GBIF: https://doi.org/10.15468/dl.zfaw79
Anopheles annulipes	150.18333	-33.75	Unclassified	GBIF: https://doi.org/10.15468/dl.zfaw79
Anopheles annulipes	150.183334	-33.75	Unclassified	GBIF: https://doi.org/10.15468/dl.zfaw79
Anopheles annulipes	150.199997	-23.916666	Unclassified	GBIF: https://doi.org/10.15468/dl.zfaw79
Anopheles annulipes	150.2	-23.91667	Unclassified	GBIF: https://doi.org/10.15468/dl.zfaw79
Anopheles annulipes	150.233337	-33.716667	Unclassified	GBIF: https://doi.org/10.15468/dl.zfaw79
Anopheles annulipes	150.23334	-33.71667	Unclassified	GBIF: https://doi.org/10.15468/dl.zfaw79
Anopheles annulipes	150.25	-35.73	Unclassified	GBIF: https://doi.org/10.15468/dl.zfaw79
Anopheles annulipes	150.27	-23.88	Unclassified	GBIF: https://doi.org/10.15468/dl.zfaw79
Anopheles annulipes	150.333328	-35.483334	Unclassified	GBIF: https://doi.org/10.15468/dl.zfaw79
Anopheles annulipes	150.33333	-35.48333	Unclassified	GBIF: https://doi.org/10.15468/dl.zfaw79
Anopheles annulipes	150.37	-35.43	Unclassified	GBIF: https://doi.org/10.15468/dl.zfaw79
Anopheles annulipes	150.3906	-35.5016	Unclassified	GBIF: https://doi.org/10.15468/dl.zfaw79
Anopheles annulipes	150.41667	-26.05	Unclassified	GBIF: https://doi.org/10.15468/dl.zfaw79
Anopheles annulipes	150.41667	-34.48333	Unclassified	GBIF: https://doi.org/10.15468/dl.zfaw79
Anopheles annulipes	150.416672	-34.483334	Unclassified	GBIF: https://doi.org/10.15468/dl.zfaw79
Anopheles annulipes	150.416672	-26.049999	Unclassified	GBIF: https://doi.org/10.15468/dl.zfaw79
Anopheles annulipes	150.43	-35.22	Unclassified	GBIF: https://doi.org/10.15468/dl.zfaw79
Anopheles annulipes	150.4323	-35.2259	Unclassified	GBIF: https://doi.org/10.15468/dl.zfaw79
Anopheles annulipes	150.43333	-25.03333	Unclassified	GBIF: https://doi.org/10.15468/dl.zfaw79
Anopheles annulipes	150.433334	-25.033333	Unclassified	GBIF: https://doi.org/10.15468/dl.zfaw79
Anopheles annulipes	150.46666	-33.483334	Unclassified	GBIF: https://doi.org/10.15468/dl.zfaw79
Anopheles annulipes	150.46666	-33.48333	Unclassified	GBIF: https://doi.org/10.15468/dl.zfaw79
Anopheles annulipes	150.483337	-34.400002	Unclassified	GBIF: https://doi.org/10.15468/dl.zfaw79
Anopheles annulipes	150.483337	-34.733334	Unclassified	GBIF: https://doi.org/10.15468/dl.zfaw79

Anopheles annulipes	150.48334	-34.73333	Unclassified	GBIF: https://doi.org/10.15468/dl.zfaw79
Anopheles annulipes	150.48334	-34.4	Unclassified	GBIF: https://doi.org/10.15468/dl.zfaw79
Anopheles annulipes	150.51666	-23.3	Unclassified	GBIF: https://doi.org/10.15468/dl.zfaw79
Anopheles annulipes	150.516663	-23.299999	Unclassified	GBIF: https://doi.org/10.15468/dl.zfaw79
Anopheles annulipes	150.5303	-35.2255	Unclassified	GBIF: https://doi.org/10.15468/dl.zfaw79
Anopheles annulipes	150.5667	-34.7	Unclassified	GBIF: https://doi.org/10.15468/dl.zfaw79
Anopheles annulipes	150.583298	-33.866661	Unclassified	GBIF: https://doi.org/10.15468/dl.zfaw79
Anopheles annulipes	150.5833	-33.86666	Unclassified	GBIF: https://doi.org/10.15468/dl.zfaw79
Anopheles annulipes	150.619446	-33.769444	Unclassified	GBIF: https://doi.org/10.15468/dl.zfaw79
Anopheles annulipes	150.61945	-33.76944	Unclassified	GBIF: https://doi.org/10.15468/dl.zfaw79
Anopheles annulipes	150.63333	-26.73333	Unclassified	GBIF: https://doi.org/10.15468/dl.zfaw79
Anopheles annulipes	150.633331	-26.733334	Unclassified	GBIF: https://doi.org/10.15468/dl.zfaw79
Anopheles annulipes	150.66667	-33.75	Unclassified	GBIF: https://doi.org/10.15468/dl.zfaw79
Anopheles annulipes	150.666672	-33.75	Unclassified	GBIF: https://doi.org/10.15468/dl.zfaw79
Anopheles annulipes	150.75	-29.25	Unclassified	GBIF: https://doi.org/10.15468/dl.zfaw79
Anopheles annulipes	150.76666	-28.9	Unclassified	GBIF: https://doi.org/10.15468/dl.zfaw79
Anopheles annulipes	150.766663	-28.9	Unclassified	GBIF: https://doi.org/10.15468/dl.zfaw79
Anopheles annulipes	150.8167	-9.7333	Unclassified	GBIF: https://doi.org/10.15468/dl.zfaw79
Anopheles annulipes	150.89166	-34.425	Unclassified	GBIF: https://doi.org/10.15468/dl.zfaw79
Anopheles annulipes	150.891663	-34.424999	Unclassified	GBIF: https://doi.org/10.15468/dl.zfaw79
Anopheles annulipes	150.89999	-29.25	Unclassified	GBIF: https://doi.org/10.15468/dl.zfaw79
Anopheles annulipes	150.899994	-29.25	Unclassified	GBIF: https://doi.org/10.15468/dl.zfaw79
Anopheles annulipes	150.972332	-33.958555	Unclassified	GBIF: https://doi.org/10.15468/dl.zfaw79
Anopheles annulipes	151	-33.733334	Unclassified	GBIF: https://doi.org/10.15468/dl.zfaw79
Anopheles annulipes	151	-33.73333	Unclassified	GBIF: https://doi.org/10.15468/dl.zfaw79
Anopheles annulipes	151.0058	-33.9775	Unclassified	GBIF: https://doi.org/10.15468/dl.zfaw79
Anopheles annulipes	151.03	-34.03	Unclassified	GBIF: https://doi.org/10.15468/dl.zfaw79
Anopheles annulipes	151.0598	-33.8248	Unclassified	GBIF: https://doi.org/10.15468/dl.zfaw79
Anopheles annulipes	151.066666	-34.133335	Unclassified	GBIF: https://doi.org/10.15468/dl.zfaw79
Anopheles annulipes	151.06667	-34.13334	Unclassified	GBIF: https://doi.org/10.15468/dl.zfaw79
Anopheles annulipes	151.0823	-33.8641	Unclassified	GBIF: https://doi.org/10.15468/dl.zfaw79
Anopheles annulipes	151.0833	-28.4167	Unclassified	GBIF: https://doi.org/10.15468/dl.zfaw79
Anopheles annulipes	151.084546	-29.66613	Unclassified	GBIF: https://doi.org/10.15468/dl.zfaw79
Anopheles annulipes	151.100006	-33.700001	Unclassified	GBIF: https://doi.org/10.15468/dl.zfaw79
Anopheles annulipes	151.10001	-33.7	Unclassified	GBIF: https://doi.org/10.15468/dl.zfaw79
Anopheles annulipes	151.116669	-23.833334	Unclassified	GBIF: https://doi.org/10.15468/dl.zfaw79
Anopheles annulipes	151.116669	-25.366667	Unclassified	GBIF: https://doi.org/10.15468/dl.zfaw79
Anopheles annulipes	151.11667	-23.83333	Unclassified	GBIF: https://doi.org/10.15468/dl.zfaw79
Anopheles annulipes	151.11667	-25.36667	Unclassified	GBIF: https://doi.org/10.15468/dl.zfaw79
Anopheles annulipes	151.116699	-25.366699	Unclassified	GBIF: https://doi.org/10.15468/dl.zfaw79
Anopheles annulipes	151.1167	-25.3667	Unclassified	GBIF: https://doi.org/10.15468/dl.zfaw79
Anopheles annulipes	151.12257	-25.3706	Unclassified	GBIF: https://doi.org/10.15468/dl.zfaw79

Anopheles annulipes	151.15542	-33.867687	Unclassified	GBIF: https://doi.org/10.15468/dl.zfaw79
Anopheles annulipes	151.16667	-23.63333	Unclassified	GBIF: https://doi.org/10.15468/dl.zfaw79
Anopheles annulipes	151.166672	-23.633333	Unclassified	GBIF: https://doi.org/10.15468/dl.zfaw79
Anopheles annulipes	151.16677	-33.81583	Unclassified	GBIF: https://doi.org/10.15468/dl.zfaw79
Anopheles annulipes	151.16804	-32.565	Unclassified	GBIF: https://doi.org/10.15468/dl.zfaw79
Anopheles annulipes	151.183	-33.516	Unclassified	GBIF: https://doi.org/10.15468/dl.zfaw79
Anopheles annulipes	151.1833	-33.88334	Unclassified	GBIF: https://doi.org/10.15468/dl.zfaw79
Anopheles annulipes	151.183304	-33.883339	Unclassified	GBIF: https://doi.org/10.15468/dl.zfaw79
Anopheles annulipes	151.199997	-24	Unclassified	GBIF: https://doi.org/10.15468/dl.zfaw79
Anopheles annulipes	151.2	-24	Unclassified	GBIF: https://doi.org/10.15468/dl.zfaw79
Anopheles annulipes	151.21666	-33.883335	Unclassified	GBIF: https://doi.org/10.15468/dl.zfaw79
Anopheles annulipes	151.21666	-33.88334	Unclassified	GBIF: https://doi.org/10.15468/dl.zfaw79
Anopheles annulipes	151.233	-33.716	Unclassified	GBIF: https://doi.org/10.15468/dl.zfaw79
Anopheles annulipes	151.233337	-23.883333	Unclassified	GBIF: https://doi.org/10.15468/dl.zfaw79
Anopheles annulipes	151.23334	-23.88333	Unclassified	GBIF: https://doi.org/10.15468/dl.zfaw79
Anopheles annulipes	151.25	-33.75	Unclassified	GBIF: https://doi.org/10.15468/dl.zfaw79
Anopheles annulipes	151.26666	-23.85	Unclassified	GBIF: https://doi.org/10.15468/dl.zfaw79
Anopheles annulipes	151.266663	-23.85	Unclassified	GBIF: https://doi.org/10.15468/dl.zfaw79
Anopheles annulipes	151.28334	-33.766666	Unclassified	GBIF: https://doi.org/10.15468/dl.zfaw79
Anopheles annulipes	151.28334	-33.76667	Unclassified	GBIF: https://doi.org/10.15468/dl.zfaw79
Anopheles annulipes	151.28499	-33.73193	Unclassified	GBIF: https://doi.org/10.15468/dl.zfaw79
Anopheles annulipes	151.2874	-33.7183	Unclassified	GBIF: https://doi.org/10.15468/dl.zfaw79
Anopheles annulipes	151.3	-33.71667	Unclassified	GBIF: https://doi.org/10.15468/dl.zfaw79
Anopheles annulipes	151.300003	-33.716667	Unclassified	GBIF: https://doi.org/10.15468/dl.zfaw79
Anopheles annulipes	151.333328	-23.933332	Unclassified	GBIF: https://doi.org/10.15468/dl.zfaw79
Anopheles annulipes	151.33333	-23.93333	Unclassified	GBIF: https://doi.org/10.15468/dl.zfaw79
Anopheles annulipes	151.3727	-33.346	Unclassified	GBIF: https://doi.org/10.15468/dl.zfaw79
Anopheles annulipes	151.600006	-32.700001	Unclassified	GBIF: https://doi.org/10.15468/dl.zfaw79
Anopheles annulipes	151.60001	-32.7	Unclassified	GBIF: https://doi.org/10.15468/dl.zfaw79
Anopheles annulipes	151.6902	-32.8475	Unclassified	GBIF: https://doi.org/10.15468/dl.zfaw79
Anopheles annulipes	151.699997	-26.833334	Unclassified	GBIF: https://doi.org/10.15468/dl.zfaw79
Anopheles annulipes	151.7	-26.83333	Unclassified	GBIF: https://doi.org/10.15468/dl.zfaw79
Anopheles annulipes	151.8	-28.58333	Unclassified	GBIF: https://doi.org/10.15468/dl.zfaw79
Anopheles annulipes	151.8	-26.85	Unclassified	GBIF: https://doi.org/10.15468/dl.zfaw79
Anopheles annulipes	151.800003	-28.583334	Unclassified	GBIF: https://doi.org/10.15468/dl.zfaw79
Anopheles annulipes	151.800003	-26.85	Unclassified	GBIF: https://doi.org/10.15468/dl.zfaw79
Anopheles annulipes	151.83	-29.15	Unclassified	GBIF: https://doi.org/10.15468/dl.zfaw79
Anopheles annulipes	152.03334	-28.216667	Unclassified	GBIF: https://doi.org/10.15468/dl.zfaw79
Anopheles annulipes	152.03334	-28.21667	Unclassified	GBIF: https://doi.org/10.15468/dl.zfaw79
Anopheles annulipes	152.1667	-28.23333	Unclassified	GBIF: https://doi.org/10.15468/dl.zfaw79
Anopheles annulipes	152.166702	-28.23333	Unclassified	GBIF: https://doi.org/10.15468/dl.zfaw79
Anopheles annulipes	152.25	-24.85	Unclassified	GBIF: https://doi.org/10.15468/dl.zfaw79

Anopheles annulipes	152.3	-26.76667	Unclassified	GBIF: https://doi.org/10.15468/dl.zfaw79
Anopheles annulipes	152.350006	-24.866667	Unclassified	GBIF: https://doi.org/10.15468/dl.zfaw79
Anopheles annulipes	152.35001	-24.86667	Unclassified	GBIF: https://doi.org/10.15468/dl.zfaw79
Anopheles annulipes	152.366669	-31.433332	Unclassified	GBIF: https://doi.org/10.15468/dl.zfaw79
Anopheles annulipes	152.36667	-31.43333	Unclassified	GBIF: https://doi.org/10.15468/dl.zfaw79
Anopheles annulipes	152.38333	-28.26667	Unclassified	GBIF: https://doi.org/10.15468/dl.zfaw79
Anopheles annulipes	152.383331	-28.266666	Unclassified	GBIF: https://doi.org/10.15468/dl.zfaw79
Anopheles annulipes	152.400904	-31.941731	Unclassified	GBIF: https://doi.org/10.15468/dl.zfaw79
Anopheles annulipes	152.436599	-32.218856	Unclassified	GBIF: https://doi.org/10.15468/dl.zfaw79
Anopheles annulipes	152.449997	-31.916666	Unclassified	GBIF: https://doi.org/10.15468/dl.zfaw79
Anopheles annulipes	152.45	-31.91667	Unclassified	GBIF: https://doi.org/10.15468/dl.zfaw79
Anopheles annulipes	152.45486	-32.225884	Unclassified	GBIF: https://doi.org/10.15468/dl.zfaw79
Anopheles annulipes	152.454864	-32.225878	Unclassified	GBIF: https://doi.org/10.15468/dl.zfaw79
Anopheles annulipes	152.459457	-27.669208	Unclassified	GBIF: https://doi.org/10.15468/dl.zfaw79
Anopheles annulipes	152.516216	-29.096968	Unclassified	GBIF: https://doi.org/10.15468/dl.zfaw79
Anopheles annulipes	152.68333	-26.46667	Unclassified	GBIF: https://doi.org/10.15468/dl.zfaw79
Anopheles annulipes	152.683334	-26.466667	Unclassified	GBIF: https://doi.org/10.15468/dl.zfaw79
Anopheles annulipes	152.695797	-25.78988	Unclassified	GBIF: https://doi.org/10.15468/dl.zfaw79
Anopheles annulipes	152.709923	-26.540926	Unclassified	GBIF: https://doi.org/10.15468/dl.zfaw79
Anopheles annulipes	152.734591	-26.712849	Unclassified	GBIF: https://doi.org/10.15468/dl.zfaw79
Anopheles annulipes	152.757055	-26.914774	Unclassified	GBIF: https://doi.org/10.15468/dl.zfaw79
Anopheles annulipes	152.75709	-26.908169	Unclassified	GBIF: https://doi.org/10.15468/dl.zfaw79
Anopheles annulipes	152.88333	-27.4	Unclassified	GBIF: https://doi.org/10.15468/dl.zfaw79
Anopheles annulipes	152.883331	-27.4	Unclassified	GBIF: https://doi.org/10.15468/dl.zfaw79
Anopheles annulipes	152.918558	-26.822887	Unclassified	GBIF: https://doi.org/10.15468/dl.zfaw79
Anopheles annulipes	152.945369	-27.493463	Unclassified	GBIF: https://doi.org/10.15468/dl.zfaw79
Anopheles annulipes	152.945418	-27.493357	Unclassified	GBIF: https://doi.org/10.15468/dl.zfaw79
Anopheles annulipes	152.945588	-27.195168	Unclassified	GBIF: https://doi.org/10.15468/dl.zfaw79
Anopheles annulipes	152.949997	-27.433331	Unclassified	GBIF: https://doi.org/10.15468/dl.zfaw79
Anopheles annulipes	152.95	-27.43333	Unclassified	GBIF: https://doi.org/10.15468/dl.zfaw79
Anopheles annulipes	152.956987	-26.964577	Unclassified	GBIF: https://doi.org/10.15468/dl.zfaw79
Anopheles annulipes	152.958555	-26.955248	Unclassified	GBIF: https://doi.org/10.15468/dl.zfaw79
Anopheles annulipes	152.961933	-26.771211	Unclassified	GBIF: https://doi.org/10.15468/dl.zfaw79
Anopheles annulipes	152.962005	-26.878118	Unclassified	GBIF: https://doi.org/10.15468/dl.zfaw79
Anopheles annulipes	152.96666	-26.683332	Unclassified	GBIF: https://doi.org/10.15468/dl.zfaw79
Anopheles annulipes	152.96666	-26.68333	Unclassified	GBIF: https://doi.org/10.15468/dl.zfaw79
Anopheles annulipes	152.977303	-27.406882	Unclassified	GBIF: https://doi.org/10.15468/dl.zfaw79
Anopheles annulipes	153	-26.283333	Unclassified	GBIF: https://doi.org/10.15468/dl.zfaw79
Anopheles annulipes	153	-26.28333	Unclassified	GBIF: https://doi.org/10.15468/dl.zfaw79
Anopheles annulipes	153.01666	-27.46667	Unclassified	GBIF: https://doi.org/10.15468/dl.zfaw79
Anopheles annulipes	153.016663	-27.466667	Unclassified	GBIF: https://doi.org/10.15468/dl.zfaw79
Anopheles annulipes	153.021692	-26.446933	Unclassified	GBIF: https://doi.org/10.15468/dl.zfaw79

Anopheles annulipes	153.03329	-27.2333	Unclassified	GBIF: https://doi.org/10.15468/dl.zfaw79
Anopheles annulipes	153.033295	-27.233299	Unclassified	GBIF: https://doi.org/10.15468/dl.zfaw79
Anopheles annulipes	153.0333	-27.46667	Unclassified	GBIF: https://doi.org/10.15468/dl.zfaw79
Anopheles annulipes	153.03334	-26.266666	Unclassified	GBIF: https://doi.org/10.15468/dl.zfaw79
Anopheles annulipes	153.03334	-26.26667	Unclassified	GBIF: https://doi.org/10.15468/dl.zfaw79
Anopheles annulipes	153.040011	-26.615305	Unclassified	GBIF: https://doi.org/10.15468/dl.zfaw79
Anopheles annulipes	153.042062	-26.750508	Unclassified	GBIF: https://doi.org/10.15468/dl.zfaw79
Anopheles annulipes	153.049584	-26.606853	Unclassified	GBIF: https://doi.org/10.15468/dl.zfaw79
Anopheles annulipes	153.050437	-26.869064	Unclassified	GBIF: https://doi.org/10.15468/dl.zfaw79
Anopheles annulipes	153.050609	-27.312677	Unclassified	GBIF: https://doi.org/10.15468/dl.zfaw79
Anopheles annulipes	153.052	-31.063	Unclassified	GBIF: https://doi.org/10.15468/dl.zfaw79
Anopheles annulipes	153.053272	-26.567402	Unclassified	GBIF: https://doi.org/10.15468/dl.zfaw79
Anopheles annulipes	153.058493	-26.646694	Unclassified	GBIF: https://doi.org/10.15468/dl.zfaw79
Anopheles annulipes	153.06202	-26.646148	Unclassified	GBIF: https://doi.org/10.15468/dl.zfaw79
Anopheles annulipes	153.071983	-26.843878	Unclassified	GBIF: https://doi.org/10.15468/dl.zfaw79
Anopheles annulipes	153.078214	-26.563274	Unclassified	GBIF: https://doi.org/10.15468/dl.zfaw79
Anopheles annulipes	153.080812	-26.567363	Unclassified	GBIF: https://doi.org/10.15468/dl.zfaw79
Anopheles annulipes	153.083997	-26.814483	Unclassified	GBIF: https://doi.org/10.15468/dl.zfaw79
Anopheles annulipes	153.100006	-26.383333	Unclassified	GBIF: https://doi.org/10.15468/dl.zfaw79
Anopheles annulipes	153.10001	-26.38333	Unclassified	GBIF: https://doi.org/10.15468/dl.zfaw79
Anopheles annulipes	153.104647	-26.752915	Unclassified	GBIF: https://doi.org/10.15468/dl.zfaw79
Anopheles annulipes	153.110157	-26.839911	Unclassified	GBIF: https://doi.org/10.15468/dl.zfaw79
Anopheles annulipes	153.117704	-26.732815	Unclassified	GBIF: https://doi.org/10.15468/dl.zfaw79
Anopheles annulipes	153.13	-30.3	Unclassified	GBIF: https://doi.org/10.15468/dl.zfaw79
Anopheles annulipes	153.1333	-27.5	Unclassified	GBIF: https://doi.org/10.15468/dl.zfaw79
Anopheles annulipes	153.133301	-27.5	Unclassified	GBIF: https://doi.org/10.15468/dl.zfaw79
Anopheles annulipes	153.13333	-26.8	Unclassified	GBIF: https://doi.org/10.15468/dl.zfaw79
Anopheles annulipes	153.13333	-30.3	Unclassified	GBIF: https://doi.org/10.15468/dl.zfaw79
Anopheles annulipes	153.133331	-26.799999	Unclassified	GBIF: https://doi.org/10.15468/dl.zfaw79
Anopheles annulipes	153.133331	-30.299999	Unclassified	GBIF: https://doi.org/10.15468/dl.zfaw79
Anopheles annulipes	153.16525	-28.01688	Unclassified	GBIF: https://doi.org/10.15468/dl.zfaw79
Anopheles annulipes	153.18333	-25.31667	Unclassified	GBIF: https://doi.org/10.15468/dl.zfaw79
Anopheles annulipes	153.18333	-27.46667	Unclassified	GBIF: https://doi.org/10.15468/dl.zfaw79
Anopheles annulipes	153.183334	-25.316668	Unclassified	GBIF: https://doi.org/10.15468/dl.zfaw79
Anopheles annulipes	153.183334	-27.466667	Unclassified	GBIF: https://doi.org/10.15468/dl.zfaw79
Anopheles annulipes	153.25	-27.866671	Unclassified	GBIF: https://doi.org/10.15468/dl.zfaw79
Anopheles annulipes	153.25	-27.86667	Unclassified	GBIF: https://doi.org/10.15468/dl.zfaw79
Anopheles annulipes	153.296784	-28.810523	Unclassified	GBIF: https://doi.org/10.15468/dl.zfaw79
Anopheles annulipes	153.3	-27.58333	Unclassified	GBIF: https://doi.org/10.15468/dl.zfaw79
Anopheles annulipes	153.3	-24.95	Unclassified	GBIF: https://doi.org/10.15468/dl.zfaw79
Anopheles annulipes	153.300003	-24.950001	Unclassified	GBIF: https://doi.org/10.15468/dl.zfaw79
Anopheles annulipes	153.300003	-27.583334	Unclassified	GBIF: https://doi.org/10.15468/dl.zfaw79

Anopheles annulipes	153.314362	-27.669709	Unclassified	GBIF: https://doi.org/10.15468/dl.zfaw79
Anopheles annulipes	153.32	-28.68	Unclassified	GBIF: https://doi.org/10.15468/dl.zfaw79
Anopheles annulipes	153.366669	-27.083334	Unclassified	GBIF: https://doi.org/10.15468/dl.zfaw79
Anopheles annulipes	153.36667	-27.08333	Unclassified	GBIF: https://doi.org/10.15468/dl.zfaw79
Anopheles annulipes	153.401639	-28.258211	Unclassified	GBIF: https://doi.org/10.15468/dl.zfaw79
Anopheles annulipes	153.5	-28.5	Unclassified	GBIF: https://doi.org/10.15468/dl.zfaw79
Anopheles annulipes	153.556243	-29.002791	Unclassified	GBIF: https://doi.org/10.15468/dl.zfaw79
Anopheles annulipes	159.066666	-31.533333	Unclassified	GBIF: https://doi.org/10.15468/dl.zfaw79
Anopheles annulipes	159.06667	-31.53333	Unclassified	GBIF: https://doi.org/10.15468/dl.zfaw79
Anopheles annulipes	159.08	-31.55	Unclassified	GBIF: https://doi.org/10.15468/dl.zfaw79
Anopheles anomalophyllus	-83.58089	9.83989	Unclassified	GBIF: https://doi.org/10.15468/dl.zfaw79
Anopheles anomalophyllus	-83.4332	9.23457	Unclassified	GBIF: https://doi.org/10.15468/dl.zfaw79
Anopheles anomalophyllus	-82.93319	9.51788	Unclassified	GBIF: https://doi.org/10.15468/dl.zfaw79
Anopheles anomalophyllus	-82.76652	9.61788	Unclassified	GBIF: https://doi.org/10.15468/dl.zfaw79
Anopheles anomalophyllus	-82.41103	9.28987	Unclassified	GBIF: https://doi.org/10.15468/dl.zfaw79
Anopheles antunesi	-54.355206	-11.524631	Unclassified	GBIF: https://doi.org/10.15468/dl.zfaw79
Anopheles antunesi	-48.8293	-25.4754	Unclassified	GBIF: https://doi.org/10.15468/dl.zfaw79
Anopheles antunesi	-45.89059	-23.65587	Unclassified	GBIF: https://doi.org/10.15468/dl.zfaw79
Anopheles antunesi	-44.62	-22.42	Unclassified	GBIF: https://doi.org/10.15468/dl.zfaw79
Anopheles antunesi	9.277168	55.026557	Unclassified	GBIF: https://doi.org/10.15468/dl.zfaw79
Anopheles antunesi	11.24422	55.68773	Unclassified	GBIF: https://doi.org/10.15468/dl.zfaw79
Anopheles antunesi	11.77348	55.22138	Unclassified	GBIF: https://doi.org/10.15468/dl.zfaw79
Anopheles antunesi	12.627485	55.595293	Unclassified	GBIF: https://doi.org/10.15468/dl.zfaw79
Anopheles apicimacula	-97.823333	16.280555	Unclassified	GBIF: https://doi.org/10.15468/dl.zfaw79
Anopheles apicimacula	-97.1745578	19.6178911	Unclassified	GBIF: https://doi.org/10.15468/dl.zfaw79
Anopheles apicimacula	-96.997778	18.896944	Unclassified	GBIF: https://doi.org/10.15468/dl.zfaw79
Anopheles apicimacula	-96.660833	15.958611	Unclassified	GBIF: https://doi.org/10.15468/dl.zfaw79
Anopheles apicimacula	-96.322222	15.834167	Unclassified	GBIF: https://doi.org/10.15468/dl.zfaw79
Anopheles apicimacula	-95.035555	18.504167	Unclassified	GBIF: https://doi.org/10.15468/dl.zfaw79
Anopheles apicimacula	-94.913333	17.948333	Unclassified	GBIF: https://doi.org/10.15468/dl.zfaw79
Anopheles apicimacula	-94.674167	18.023889	Unclassified	GBIF: https://doi.org/10.15468/dl.zfaw79
Anopheles apicimacula	-94.345278	17.986667	Unclassified	GBIF: https://doi.org/10.15468/dl.zfaw79
Anopheles apicimacula	-93.311111	17.136111	Unclassified	GBIF: https://doi.org/10.15468/dl.zfaw79
Anopheles apicimacula	-93.209167	15.686667	Unclassified	GBIF: https://doi.org/10.15468/dl.zfaw79
Anopheles apicimacula	-93.118333	17.508611	Unclassified	GBIF: https://doi.org/10.15468/dl.zfaw79
Anopheles apicimacula	-92.952778	17.548056	Unclassified	GBIF: https://doi.org/10.15468/dl.zfaw79
Anopheles apicimacula	-92.825555	17.5925	Unclassified	GBIF: https://doi.org/10.15468/dl.zfaw79
Anopheles apicimacula	-92.7775	17.460278	Unclassified	GBIF: https://doi.org/10.15468/dl.zfaw79
Anopheles apicimacula	-92.26611	14.9	Unclassified	GBIF: https://doi.org/10.15468/dl.zfaw79
Anopheles apicimacula	-92.260556	14.908056	Unclassified	GBIF: https://doi.org/10.15468/dl.zfaw79
Anopheles apicimacula	-92.260555	14.908056	Unclassified	GBIF: https://doi.org/10.15468/dl.zfaw79
Anopheles apicimacula	-92.215555	15.088333	Unclassified	GBIF: https://doi.org/10.15468/dl.zfaw79

Anopheles apicimacula	-92.208889	15.095833	Unclassified	GBIF: https://doi.org/10.15468/dl.zfaw79
Anopheles apicimacula	-91.982222	17.509167	Unclassified	GBIF: https://doi.org/10.15468/dl.zfaw79
Anopheles apicimacula	-91.426389	17.470833	Unclassified	GBIF: https://doi.org/10.15468/dl.zfaw79
Anopheles apicimacula	-91.155555	16.120278	Unclassified	GBIF: https://doi.org/10.15468/dl.zfaw79
Anopheles apicimacula	-91.148889	16.1375	Unclassified	GBIF: https://doi.org/10.15468/dl.zfaw79
Anopheles apicimacula	-91.096111	16.088889	Unclassified	GBIF: https://doi.org/10.15468/dl.zfaw79
Anopheles apicimacula	-91.094444	16.0875	Unclassified	GBIF: https://doi.org/10.15468/dl.zfaw79
Anopheles apicimacula	-90.970833	16.097222	Unclassified	GBIF: https://doi.org/10.15468/dl.zfaw79
Anopheles apicimacula	-90.890278	16.821111	Unclassified	GBIF: https://doi.org/10.15468/dl.zfaw79
Anopheles apicimacula	-90.8250619	16.1272713	Unclassified	GBIF: https://doi.org/10.15468/dl.zfaw79
Anopheles apicimacula	-90.764167	16.335	Unclassified	GBIF: https://doi.org/10.15468/dl.zfaw79
Anopheles apicimacula	-90.650555	16.518889	Unclassified	GBIF: https://doi.org/10.15468/dl.zfaw79
Anopheles apicimacula	-90.535833	16.269167	Unclassified	GBIF: https://doi.org/10.15468/dl.zfaw79
Anopheles apicimacula	-89.417778	18.588611	Unclassified	GBIF: https://doi.org/10.15468/dl.zfaw79
Anopheles apicimacula	-89.414167	18.593056	Unclassified	GBIF: https://doi.org/10.15468/dl.zfaw79
Anopheles apicimacula	-89.18293	13.79568	Unclassified	GBIF: https://doi.org/10.15468/dl.zfaw79
Anopheles apicimacula	-89.115667	18.618778	Unclassified	GBIF: https://doi.org/10.15468/dl.zfaw79
Anopheles apicimacula	-89.09998	16.21752	Unclassified	GBIF: https://doi.org/10.15468/dl.zfaw79
Anopheles apicimacula	-88.99998	16.21752	Unclassified	GBIF: https://doi.org/10.15468/dl.zfaw79
Anopheles apicimacula	-88.98331	16.26751	Unclassified	GBIF: https://doi.org/10.15468/dl.zfaw79
Anopheles apicimacula	-88.94998	16.23418	Unclassified	GBIF: https://doi.org/10.15468/dl.zfaw79
Anopheles apicimacula	-88.757989	15.814745	Unclassified	GBIF: https://doi.org/10.15468/dl.zfaw79
Anopheles apicimacula	-87.16792	15.68594	Unclassified	GBIF: https://doi.org/10.15468/dl.zfaw79
Anopheles apicimacula	-84.9887	10.71831	Unclassified	GBIF: https://doi.org/10.15468/dl.zfaw79
Anopheles apicimacula	-84.74954	10.30282	Unclassified	GBIF: https://doi.org/10.15468/dl.zfaw79
Anopheles apicimacula	-84.60757	9.7813	Unclassified	GBIF: https://doi.org/10.15468/dl.zfaw79
Anopheles apicimacula	-84.60065	10.22071	Unclassified	GBIF: https://doi.org/10.15468/dl.zfaw79
Anopheles apicimacula	-84.12563	9.56617	Unclassified	GBIF: https://doi.org/10.15468/dl.zfaw79
Anopheles apicimacula	-84.01352	10.42731	Unclassified	GBIF: https://doi.org/10.15468/dl.zfaw79
Anopheles apicimacula	-83.88956	9.52326	Unclassified	GBIF: https://doi.org/10.15468/dl.zfaw79
Anopheles apicimacula	-83.85765	10.19145	Unclassified	GBIF: https://doi.org/10.15468/dl.zfaw79
Anopheles apicimacula	-83.85495	9.28522	Unclassified	GBIF: https://doi.org/10.15468/dl.zfaw79
Anopheles apicimacula	-83.80278	9.76877	Unclassified	GBIF: https://doi.org/10.15468/dl.zfaw79
Anopheles apicimacula	-83.79152	12.01	Unclassified	GBIF: https://doi.org/10.15468/dl.zfaw79
Anopheles apicimacula	-83.78639	9.76016	Unclassified	GBIF: https://doi.org/10.15468/dl.zfaw79
Anopheles apicimacula	-83.7365	8.62476	Unclassified	GBIF: https://doi.org/10.15468/dl.zfaw79
Anopheles apicimacula	-83.71291	9.37264	Unclassified	GBIF: https://doi.org/10.15468/dl.zfaw79
Anopheles apicimacula	-83.59116	8.48145	Unclassified	GBIF: https://doi.org/10.15468/dl.zfaw79
Anopheles apicimacula	-83.5321	8.55783	Unclassified	GBIF: https://doi.org/10.15468/dl.zfaw79
Anopheles apicimacula	-83.51382	8.70033	Unclassified	GBIF: https://doi.org/10.15468/dl.zfaw79
Anopheles apicimacula	-83.44947	9.97413	Unclassified	GBIF: https://doi.org/10.15468/dl.zfaw79
Anopheles apicimacula	-83.42538	8.53788	Unclassified	GBIF: https://doi.org/10.15468/dl.zfaw79

Anopheles apicimacula	-83.22719	8.68191	Unclassified	GBIF: https://doi.org/10.15468/dl.zfaw79
Anopheles apicimacula	-83.17953	8.65474	Unclassified	GBIF: https://doi.org/10.15468/dl.zfaw79
Anopheles apicimacula	-83.02599	9.67567	Unclassified	GBIF: https://doi.org/10.15468/dl.zfaw79
Anopheles apicimacula	-83.01074	9.03423	Unclassified	GBIF: https://doi.org/10.15468/dl.zfaw79
Anopheles apicimacula	-82.80723	8.93706	Unclassified	GBIF: https://doi.org/10.15468/dl.zfaw79
Anopheles apicimacula	-82.80338	8.90461	Unclassified	GBIF: https://doi.org/10.15468/dl.zfaw79
Anopheles apicimacula	-82.659379	9.629605	Unclassified	GBIF: https://doi.org/10.15468/dl.zfaw79
Anopheles apicimacula	-82.42017	9.29887	Unclassified	GBIF: https://doi.org/10.15468/dl.zfaw79
Anopheles apicimacula	-82.1913	8.91085	Unclassified	GBIF: https://doi.org/10.15468/dl.zfaw79
Anopheles apicimacula	-80	9	Unclassified	GBIF: https://doi.org/10.15468/dl.zfaw79
Anopheles apicimacula	-79.96164	9.36349	Unclassified	GBIF: https://doi.org/10.15468/dl.zfaw79
Anopheles apicimacula	-79.95345	9.366458	Unclassified	GBIF: https://doi.org/10.15468/dl.zfaw79
Anopheles apicimacula	-79.906218	9.169671	Unclassified	GBIF: https://doi.org/10.15468/dl.zfaw79
Anopheles apicimacula	-79.8998	9.28462	Unclassified	GBIF: https://doi.org/10.15468/dl.zfaw79
Anopheles apicimacula	-79.8998	9.36795	Unclassified	GBIF: https://doi.org/10.15468/dl.zfaw79
Anopheles apicimacula	-79.895022	9.198953	Unclassified	GBIF: https://doi.org/10.15468/dl.zfaw79
Anopheles apicimacula	-79.88313	9.38462	Unclassified	GBIF: https://doi.org/10.15468/dl.zfaw79
Anopheles apicimacula	-79.78313	8.88465	Unclassified	GBIF: https://doi.org/10.15468/dl.zfaw79
Anopheles apicimacula	-79.772129	8.884647	Unclassified	GBIF: https://doi.org/10.15468/dl.zfaw79
Anopheles apicimacula	-79.761713	9.121174	Unclassified	GBIF: https://doi.org/10.15468/dl.zfaw79
Anopheles apicimacula	-79.697814	9.116557	Unclassified	GBIF: https://doi.org/10.15468/dl.zfaw79
Anopheles apicimacula	-79.696988	9.116663	Unclassified	GBIF: https://doi.org/10.15468/dl.zfaw79
Anopheles apicimacula	-79.65282	9.16352	Unclassified	GBIF: https://doi.org/10.15468/dl.zfaw79
Anopheles apicimacula	-79.59014	8.90104	Unclassified	GBIF: https://doi.org/10.15468/dl.zfaw79
Anopheles apicimacula	-79.58313	8.91798	Unclassified	GBIF: https://doi.org/10.15468/dl.zfaw79
Anopheles apicimacula	-79.54979	8.96797	Unclassified	GBIF: https://doi.org/10.15468/dl.zfaw79
Anopheles apicimacula	-79.396389	-0.0175	Unclassified	GBIF: https://doi.org/10.15468/dl.zfaw79
Anopheles apicimacula	-79.276389	-0.085	Unclassified	GBIF: https://doi.org/10.15468/dl.zfaw79
Anopheles apicimacula	-79.276111	-0.086111	Unclassified	GBIF: https://doi.org/10.15468/dl.zfaw79
Anopheles apicimacula	-79.276111	-0.088056	Unclassified	GBIF: https://doi.org/10.15468/dl.zfaw79
Anopheles apicimacula	-79.2725	-0.086111	Unclassified	GBIF: https://doi.org/10.15468/dl.zfaw79
Anopheles apicimacula	-78.1831	7.58472	Unclassified	GBIF: https://doi.org/10.15468/dl.zfaw79
Anopheles apicimacula	-77.956656	2.555017	Unclassified	GBIF: https://doi.org/10.15468/dl.zfaw79
Anopheles apicimacula	-77.942977	2.549764	Unclassified	GBIF: https://doi.org/10.15468/dl.zfaw79
Anopheles apicimacula	-77.93998	2.58304	Unclassified	GBIF: https://doi.org/10.15468/dl.zfaw79
Anopheles apicimacula	-77.92985	2.60389	Unclassified	GBIF: https://doi.org/10.15468/dl.zfaw79
Anopheles apicimacula	-77.888367	2.593774	Unclassified	GBIF: https://doi.org/10.15468/dl.zfaw79
Anopheles apicimacula	-77.88444	2.599734	Unclassified	GBIF: https://doi.org/10.15468/dl.zfaw79
Anopheles apicimacula	-77.87693	2.597532	Unclassified	GBIF: https://doi.org/10.15468/dl.zfaw79
Anopheles apicimacula	-77.875698	2.600023	Unclassified	GBIF: https://doi.org/10.15468/dl.zfaw79
Anopheles apicimacula	-77.873733	2.56444	Unclassified	GBIF: https://doi.org/10.15468/dl.zfaw79
Anopheles apicimacula	-77.870214	2.565049	Unclassified	GBIF: https://doi.org/10.15468/dl.zfaw79

Anopheles apicimacula	-77.865815	2.558616	Unclassified	GBIF: https://doi.org/10.15468/dl.zfaw79
Anopheles apicimacula	-77.835088	2.553429	Unclassified	GBIF: https://doi.org/10.15468/dl.zfaw79
Anopheles apicimacula	-77.827116	2.612266	Unclassified	GBIF: https://doi.org/10.15468/dl.zfaw79
Anopheles apicimacula	-77.815819	2.613739	Unclassified	GBIF: https://doi.org/10.15468/dl.zfaw79
Anopheles apicimacula	-77.813952	2.610693	Unclassified	GBIF: https://doi.org/10.15468/dl.zfaw79
Anopheles apicimacula	-77.802114	2.614108	Unclassified	GBIF: https://doi.org/10.15468/dl.zfaw79
Anopheles apicimacula	-77.802086	2.608661	Unclassified	GBIF: https://doi.org/10.15468/dl.zfaw79
Anopheles apicimacula	-77.80153	2.61512	Unclassified	GBIF: https://doi.org/10.15468/dl.zfaw79
Anopheles apicimacula	-77.791958	2.597238	Unclassified	GBIF: https://doi.org/10.15468/dl.zfaw79
Anopheles apicimacula	-77.7628	2.63368	Unclassified	GBIF: https://doi.org/10.15468/dl.zfaw79
Anopheles apicimacula	-77.74969	2.615468	Unclassified	GBIF: https://doi.org/10.15468/dl.zfaw79
Anopheles apicimacula	-77.683833	2.777421	Unclassified	GBIF: https://doi.org/10.15468/dl.zfaw79
Anopheles apicimacula	-77.67683	2.737968	Unclassified	GBIF: https://doi.org/10.15468/dl.zfaw79
Anopheles apicimacula	-77.676548	2.73597	Unclassified	GBIF: https://doi.org/10.15468/dl.zfaw79
Anopheles apicimacula	-77.66848	2.713274	Unclassified	GBIF: https://doi.org/10.15468/dl.zfaw79
Anopheles apicimacula	-77.663169	2.758674	Unclassified	GBIF: https://doi.org/10.15468/dl.zfaw79
Anopheles apicimacula	-77.659832	2.701077	Unclassified	GBIF: https://doi.org/10.15468/dl.zfaw79
Anopheles apicimacula	-77.64286	2.67032	Unclassified	GBIF: https://doi.org/10.15468/dl.zfaw79
Anopheles apicimacula	-77.631669	2.880863	Unclassified	GBIF: https://doi.org/10.15468/dl.zfaw79
Anopheles apicimacula	-77.624947	2.660282	Unclassified	GBIF: https://doi.org/10.15468/dl.zfaw79
Anopheles apicimacula	-77.623091	2.659431	Unclassified	GBIF: https://doi.org/10.15468/dl.zfaw79
Anopheles apicimacula	-77.592412	2.63895	Unclassified	GBIF: https://doi.org/10.15468/dl.zfaw79
Anopheles apicimacula	-77.27	5.7	Unclassified	GBIF: https://doi.org/10.15468/dl.zfaw79
Anopheles apicimacula	-77.2672	5.7	Unclassified	GBIF: https://doi.org/10.15468/dl.zfaw79
Anopheles apicimacula	-76.99	3.82	Unclassified	GBIF: https://doi.org/10.15468/dl.zfaw79
Anopheles apicimacula	-76.9833	3.8167	Unclassified	GBIF: https://doi.org/10.15468/dl.zfaw79
Anopheles apicimacula	-76.98	3.82	Unclassified	GBIF: https://doi.org/10.15468/dl.zfaw79
Anopheles apicimacula	-76.87	7.8	Unclassified	GBIF: https://doi.org/10.15468/dl.zfaw79
Anopheles apicimacula	-76.79	4.98	Unclassified	GBIF: https://doi.org/10.15468/dl.zfaw79
Anopheles apicimacula	-76.7839	8.4258	Unclassified	GBIF: https://doi.org/10.15468/dl.zfaw79
Anopheles apicimacula	-76.78	8.43	Unclassified	GBIF: https://doi.org/10.15468/dl.zfaw79
Anopheles apicimacula	-76.65	5.55	Unclassified	GBIF: https://doi.org/10.15468/dl.zfaw79
Anopheles apicimacula	-76.64	5.55	Unclassified	GBIF: https://doi.org/10.15468/dl.zfaw79
Anopheles apicimacula	-76.58	7.86	Unclassified	GBIF: https://doi.org/10.15468/dl.zfaw79
Anopheles apicimacula	-76.5502	8.3446	Unclassified	GBIF: https://doi.org/10.15468/dl.zfaw79
Anopheles apicimacula	-76.55	8.66	Unclassified	GBIF: https://doi.org/10.15468/dl.zfaw79
Anopheles apicimacula	-76.55	8.34	Unclassified	GBIF: https://doi.org/10.15468/dl.zfaw79
Anopheles apicimacula	-75.23	7.58	Unclassified	GBIF: https://doi.org/10.15468/dl.zfaw79
Anopheles apicimacula	-73.61867	4.14669	Unclassified	GBIF: https://doi.org/10.15468/dl.zfaw79
Anopheles apicimacula	-72.64451	4.187754	Unclassified	GBIF: https://doi.org/10.15468/dl.zfaw79
Anopheles apicimacula	-61.297045	10.38824	Unclassified	GBIF: https://doi.org/10.15468/dl.zfaw79
Anopheles apicimacula	-61.271348	10.51485	Unclassified	GBIF: https://doi.org/10.15468/dl.zfaw79

Anopheles apicimacula	-61.265687	10.567702	Unclassified	GBIF: https://doi.org/10.15468/dl.zfaw79
Anopheles apicimacula	-61.25	10.5	Unclassified	GBIF: https://doi.org/10.15468/dl.zfaw79
Anopheles apicimacula	-61.207835	10.586586	Unclassified	GBIF: https://doi.org/10.15468/dl.zfaw79
Anopheles apicimacula	-61.175559	10.305938	Unclassified	GBIF: https://doi.org/10.15468/dl.zfaw79
Anopheles apicimacula	-60.95912	10.77802	Unclassified	GBIF: https://doi.org/10.15468/dl.zfaw79
Anopheles apicimacula	-58.1682	6.79681	Unclassified	GBIF: https://doi.org/10.15468/dl.zfaw79
Anopheles apicimacula	-55.47799	5.794231	Unclassified	GBIF: https://doi.org/10.15468/dl.zfaw79
Anopheles apicimacula	-54.415345	4.619739	Unclassified	GBIF: https://doi.org/10.15468/dl.zfaw79
Anopheles apicimacula	-54.41527	4.618084	Unclassified	GBIF: https://doi.org/10.15468/dl.zfaw79
Anopheles apicimacula	-52.593636	3.256962	Unclassified	GBIF: https://doi.org/10.15468/dl.zfaw79
Anopheles apoci	54.237076	32.906025	Unclassified	GBIF: https://doi.org/10.15468/dl.zfaw79
Anopheles aquasalis	-82.74986	9.65121	Primary	GBIF: https://doi.org/10.15468/dl.zfaw79
Anopheles aquasalis	-82.1913	8.91085	Primary	GBIF: https://doi.org/10.15468/dl.zfaw79
Anopheles aquasalis	-80.05294	9.27331	Primary	GBIF: https://doi.org/10.15468/dl.zfaw79
Anopheles aquasalis	-79.96647	9.30129	Primary	GBIF: https://doi.org/10.15468/dl.zfaw79
Anopheles aquasalis	-79.91647	9.26796	Primary	GBIF: https://doi.org/10.15468/dl.zfaw79
Anopheles aquasalis	-79.91647	9.2013	Primary	GBIF: https://doi.org/10.15468/dl.zfaw79
Anopheles aquasalis	-79.91639	9.27292	Primary	GBIF: https://doi.org/10.15468/dl.zfaw79
Anopheles aquasalis	-79.901247	9.350783	Primary	GBIF: https://doi.org/10.15468/dl.zfaw79
Anopheles aquasalis	-79.900903	9.351121	Primary	GBIF: https://doi.org/10.15468/dl.zfaw79
Anopheles aquasalis	-79.8998	9.30129	Primary	GBIF: https://doi.org/10.15468/dl.zfaw79
Anopheles aquasalis	-79.8998	9.36795	Primary	GBIF: https://doi.org/10.15468/dl.zfaw79
Anopheles aquasalis	-79.8998	9.33462	Primary	GBIF: https://doi.org/10.15468/dl.zfaw79
Anopheles aquasalis	-79.87061	9.35417	Primary	GBIF: https://doi.org/10.15468/dl.zfaw79
Anopheles aquasalis	-79.83313	9.40128	Primary	GBIF: https://doi.org/10.15468/dl.zfaw79
Anopheles aquasalis	-79.779166	9.123994	Primary	GBIF: https://doi.org/10.15468/dl.zfaw79
Anopheles aquasalis	-79.65132	9.55234	Primary	GBIF: https://doi.org/10.15468/dl.zfaw79
Anopheles aquasalis	-79.61646	9.01797	Primary	GBIF: https://doi.org/10.15468/dl.zfaw79
Anopheles aquasalis	-79.58313	8.98464	Primary	GBIF: https://doi.org/10.15468/dl.zfaw79
Anopheles aquasalis	-79.54979	8.96797	Primary	GBIF: https://doi.org/10.15468/dl.zfaw79
Anopheles aquasalis	-79.509795	8.981643	Primary	GBIF: https://doi.org/10.15468/dl.zfaw79
Anopheles aquasalis	-75.94	9.35	Primary	GBIF: https://doi.org/10.15468/dl.zfaw79
Anopheles aquasalis	-68.24327	10.57093	Primary	GBIF: https://doi.org/10.15468/dl.zfaw79
Anopheles aquasalis	-68.01857	10.46352	Primary	GBIF: https://doi.org/10.15468/dl.zfaw79
Anopheles aquasalis	-67.8519	10.44685	Primary	GBIF: https://doi.org/10.15468/dl.zfaw79
Anopheles aquasalis	-67.35187	9.91351	Primary	GBIF: https://doi.org/10.15468/dl.zfaw79
Anopheles aquasalis	-67.05186	9.38016	Primary	GBIF: https://doi.org/10.15468/dl.zfaw79
Anopheles aquasalis	-63.51837	9.74686	Primary	GBIF: https://doi.org/10.15468/dl.zfaw79
Anopheles aquasalis	-62.23676	16.72413	Primary	GBIF: https://doi.org/10.15468/dl.zfaw79
Anopheles aquasalis	-61.88964	17.05517	Primary	GBIF: https://doi.org/10.15468/dl.zfaw79
Anopheles aquasalis	-61.88456	17.11841	Primary	GBIF: https://doi.org/10.15468/dl.zfaw79
Anopheles aquasalis	-61.7961	12.00318	Primary	GBIF: https://doi.org/10.15468/dl.zfaw79

Anopheles aquasalis	-61.78986	17.07779	Primary	GBIF: https://doi.org/10.15468/dl.zfaw79
Anopheles aquasalis	-61.78922	17.03742	Primary	GBIF: https://doi.org/10.15468/dl.zfaw79
Anopheles aquasalis	-61.74879	17.0417	Primary	GBIF: https://doi.org/10.15468/dl.zfaw79
Anopheles aquasalis	-61.73543	16.36015	Primary	GBIF: https://doi.org/10.15468/dl.zfaw79
Anopheles aquasalis	-61.72259	12.0119	Primary	GBIF: https://doi.org/10.15468/dl.zfaw79
Anopheles aquasalis	-61.64807	12.21949	Primary	GBIF: https://doi.org/10.15468/dl.zfaw79
Anopheles aquasalis	-61.62367	16.27811	Primary	GBIF: https://doi.org/10.15468/dl.zfaw79
Anopheles aquasalis	-61.62092	12.13799	Primary	GBIF: https://doi.org/10.15468/dl.zfaw79
Anopheles aquasalis	-61.61607	12.11764	Primary	GBIF: https://doi.org/10.15468/dl.zfaw79
Anopheles aquasalis	-61.58702	16.16941	Primary	GBIF: https://doi.org/10.15468/dl.zfaw79
Anopheles aquasalis	-61.57702	16.25972	Primary	GBIF: https://doi.org/10.15468/dl.zfaw79
Anopheles aquasalis	-61.56512	10.66839	Primary	GBIF: https://doi.org/10.15468/dl.zfaw79
Anopheles aquasalis	-61.55981	16.05173	Primary	GBIF: https://doi.org/10.15468/dl.zfaw79
Anopheles aquasalis	-61.54915	16.23242	Primary	GBIF: https://doi.org/10.15468/dl.zfaw79
Anopheles aquasalis	-61.5398	16.19761	Primary	GBIF: https://doi.org/10.15468/dl.zfaw79
Anopheles aquasalis	-61.53977	16.19759	Primary	GBIF: https://doi.org/10.15468/dl.zfaw79
Anopheles aquasalis	-61.5301	16.27748	Primary	GBIF: https://doi.org/10.15468/dl.zfaw79
Anopheles aquasalis	-61.52902	16.42207	Primary	GBIF: https://doi.org/10.15468/dl.zfaw79
Anopheles aquasalis	-61.51511	10.65173	Primary	GBIF: https://doi.org/10.15468/dl.zfaw79
Anopheles aquasalis	-61.49316	16.21395	Primary	GBIF: https://doi.org/10.15468/dl.zfaw79
Anopheles aquasalis	-61.48273	15.58412	Primary	GBIF: https://doi.org/10.15468/dl.zfaw79
Anopheles aquasalis	-61.47921	15.58121	Primary	GBIF: https://doi.org/10.15468/dl.zfaw79
Anopheles aquasalis	-61.47009	15.55403	Primary	GBIF: https://doi.org/10.15468/dl.zfaw79
Anopheles aquasalis	-61.47002	15.56307	Primary	GBIF: https://doi.org/10.15468/dl.zfaw79
Anopheles aquasalis	-61.46606	15.58412	Primary	GBIF: https://doi.org/10.15468/dl.zfaw79
Anopheles aquasalis	-61.46524	16.20472	Primary	GBIF: https://doi.org/10.15468/dl.zfaw79
Anopheles aquasalis	-61.44844	10.65173	Primary	GBIF: https://doi.org/10.15468/dl.zfaw79
Anopheles aquasalis	-61.44547	16.3311	Primary	GBIF: https://doi.org/10.15468/dl.zfaw79
Anopheles aquasalis	-61.44314	15.41827	Primary	GBIF: https://doi.org/10.15468/dl.zfaw79
Anopheles aquasalis	-61.416667	10.6	Primary	GBIF: https://doi.org/10.15468/dl.zfaw79
Anopheles aquasalis	-61.41511	10.65173	Primary	GBIF: https://doi.org/10.15468/dl.zfaw79
Anopheles aquasalis	-61.4	10.633333	Primary	GBIF: https://doi.org/10.15468/dl.zfaw79
Anopheles aquasalis	-61.381743	10.605738	Primary	GBIF: https://doi.org/10.15468/dl.zfaw79
Anopheles aquasalis	-61.37142	16.23113	Primary	GBIF: https://doi.org/10.15468/dl.zfaw79
Anopheles aquasalis	-61.34844	10.60172	Primary	GBIF: https://doi.org/10.15468/dl.zfaw79
Anopheles aquasalis	-61.33642	15.94167	Primary	GBIF: https://doi.org/10.15468/dl.zfaw79
Anopheles aquasalis	-61.31766	15.95056	Primary	GBIF: https://doi.org/10.15468/dl.zfaw79
Anopheles aquasalis	-61.31562	10.7892	Primary	GBIF: https://doi.org/10.15468/dl.zfaw79
Anopheles aquasalis	-61.30497	10.332492	Primary	GBIF: https://doi.org/10.15468/dl.zfaw79
Anopheles aquasalis	-61.293984	10.321684	Primary	GBIF: https://doi.org/10.15468/dl.zfaw79
Anopheles aquasalis	-61.293984	10.313577	Primary	GBIF: https://doi.org/10.15468/dl.zfaw79
Anopheles aquasalis	-61.291237	10.332492	Primary	GBIF: https://doi.org/10.15468/dl.zfaw79

Anopheles aquasalis	-61.291237	10.340598	Primary	GBIF: https://doi.org/10.15468/dl.zfaw79
Anopheles aquasalis	-61.282997	10.337896	Primary	GBIF: https://doi.org/10.15468/dl.zfaw79
Anopheles aquasalis	-61.282997	10.35681	Primary	GBIF: https://doi.org/10.15468/dl.zfaw79
Anopheles aquasalis	-61.27477	15.49837	Primary	GBIF: https://doi.org/10.15468/dl.zfaw79
Anopheles aquasalis	-61.259506	10.396587	Primary	GBIF: https://doi.org/10.15468/dl.zfaw79
Anopheles aquasalis	-61.2	10.4	Primary	GBIF: https://doi.org/10.15468/dl.zfaw79
Anopheles aquasalis	-61.19843	10.58506	Primary	GBIF: https://doi.org/10.15468/dl.zfaw79
Anopheles aquasalis	-61.08801	14.49374	Primary	GBIF: https://doi.org/10.15468/dl.zfaw79
Anopheles aquasalis	-61.06097	13.85638	Primary	GBIF: https://doi.org/10.15468/dl.zfaw79
Anopheles aquasalis	-61.04177	13.93937	Primary	GBIF: https://doi.org/10.15468/dl.zfaw79
Anopheles aquasalis	-61.02954	14.80954	Primary	GBIF: https://doi.org/10.15468/dl.zfaw79
Anopheles aquasalis	-61.02776	13.95463	Primary	GBIF: https://doi.org/10.15468/dl.zfaw79
Anopheles aquasalis	-61.01929	14.65485	Primary	GBIF: https://doi.org/10.15468/dl.zfaw79
Anopheles aquasalis	-61.01828	13.76386	Primary	GBIF: https://doi.org/10.15468/dl.zfaw79
Anopheles aquasalis	-61.01509	10.20171	Primary	GBIF: https://doi.org/10.15468/dl.zfaw79
Anopheles aquasalis	-61	11	Primary	GBIF: https://doi.org/10.15468/dl.zfaw79
Anopheles aquasalis	-60.97153	14.0364	Primary	GBIF: https://doi.org/10.15468/dl.zfaw79
Anopheles aquasalis	-60.96957	14.04813	Primary	GBIF: https://doi.org/10.15468/dl.zfaw79
Anopheles aquasalis	-60.96952	14.05355	Primary	GBIF: https://doi.org/10.15468/dl.zfaw79
Anopheles aquasalis	-60.95454	14.0724	Primary	GBIF: https://doi.org/10.15468/dl.zfaw79
Anopheles aquasalis	-60.94841	10.83506	Primary	GBIF: https://doi.org/10.15468/dl.zfaw79
Anopheles aquasalis	-60.94679	14.71846	Primary	GBIF: https://doi.org/10.15468/dl.zfaw79
Anopheles aquasalis	-60.90411	13.81443	Primary	GBIF: https://doi.org/10.15468/dl.zfaw79
Anopheles aquasalis	-60.83746	11.16583	Primary	GBIF: https://doi.org/10.15468/dl.zfaw79
Anopheles aquasalis	-60.81	11.16563	Primary	GBIF: https://doi.org/10.15468/dl.zfaw79
Anopheles aquasalis	-58.2682	6.4968	Primary	GBIF: https://doi.org/10.15468/dl.zfaw79
Anopheles aquasalis	-58.1682	6.79681	Primary	GBIF: https://doi.org/10.15468/dl.zfaw79
Anopheles aquasalis	-57.78485	6.51347	Primary	GBIF: https://doi.org/10.15468/dl.zfaw79
Anopheles aquasalis	-57.51817	6.24679	Primary	GBIF: https://doi.org/10.15468/dl.zfaw79
Anopheles aquasalis	-56.993055	5.94158	Primary	GBIF: https://doi.org/10.15468/dl.zfaw79
Anopheles aquasalis	-56.98475	5.94684	Primary	GBIF: https://doi.org/10.15468/dl.zfaw79
Anopheles aquasalis	-56.33472	5.91351	Primary	GBIF: https://doi.org/10.15468/dl.zfaw79
Anopheles aquasalis	-56.33472	5.89684	Primary	GBIF: https://doi.org/10.15468/dl.zfaw79
Anopheles aquasalis	-55.47799	5.794231	Primary	GBIF: https://doi.org/10.15468/dl.zfaw79
Anopheles aquasalis	-55.16801	5.83018	Primary	GBIF: https://doi.org/10.15468/dl.zfaw79
Anopheles aquasalis	-55.15134	5.81351	Primary	GBIF: https://doi.org/10.15468/dl.zfaw79
Anopheles aquasalis	-55.13468	5.93018	Primary	GBIF: https://doi.org/10.15468/dl.zfaw79
Anopheles aquasalis	-54.40131	5.61351	Primary	GBIF: https://doi.org/10.15468/dl.zfaw79
Anopheles aquasalis	-54.388355	4.280298	Primary	GBIF: https://doi.org/10.15468/dl.zfaw79
Anopheles aquasalis	-54.383048	4.27418	Primary	GBIF: https://doi.org/10.15468/dl.zfaw79
Anopheles aquasalis	-54.082518	5.409066	Primary	GBIF: https://doi.org/10.15468/dl.zfaw79
Anopheles aquasalis	-54.045577	5.480947	Primary	GBIF: https://doi.org/10.15468/dl.zfaw79

Anopheles aquasalis	-54.04329	5.484058 Primary	GBIF: https://doi.org/10.15468/dl.zfaw79
Anopheles aquasalis	-54.031415	3.643348 Primary	GBIF: https://doi.org/10.15468/dl.zfaw79
Anopheles aquasalis	-54.028939	5.503342 Primary	GBIF: https://doi.org/10.15468/dl.zfaw79
Anopheles aquasalis	-54.02004	5.50717 Primary	GBIF: https://doi.org/10.15468/dl.zfaw79
Anopheles aquasalis	-53.916695	3.383348 Primary	GBIF: https://doi.org/10.15468/dl.zfaw79
Anopheles aquasalis	-53.910915	5.631878 Primary	GBIF: https://doi.org/10.15468/dl.zfaw79
Anopheles aquasalis	-53.909572	5.736301 Primary	GBIF: https://doi.org/10.15468/dl.zfaw79
Anopheles aquasalis	-53.814272	5.593271 Primary	GBIF: https://doi.org/10.15468/dl.zfaw79
Anopheles aquasalis	-53.778092	5.66898 Primary	GBIF: https://doi.org/10.15468/dl.zfaw79
Anopheles aquasalis	-53.74599	5.638017 Primary	GBIF: https://doi.org/10.15468/dl.zfaw79
Anopheles aquasalis	-53.71799	5.61346 Primary	GBIF: https://doi.org/10.15468/dl.zfaw79
Anopheles aquasalis	-53.674595	5.394268 Primary	GBIF: https://doi.org/10.15468/dl.zfaw79
Anopheles aquasalis	-53.66805	5.61258 Primary	GBIF: https://doi.org/10.15468/dl.zfaw79
Anopheles aquasalis	-53.36797	4.8801 Primary	GBIF: https://doi.org/10.15468/dl.zfaw79
Anopheles aquasalis	-53.30643	5.48769 Primary	GBIF: https://doi.org/10.15468/dl.zfaw79
Anopheles aquasalis	-53.24036	3.91451 Primary	GBIF: https://doi.org/10.15468/dl.zfaw79
Anopheles aquasalis	-53.240025	5.469148 Primary	GBIF: https://doi.org/10.15468/dl.zfaw79
Anopheles aquasalis	-53.23783	5.50331 Primary	GBIF: https://doi.org/10.15468/dl.zfaw79
Anopheles aquasalis	-53.23477	3.92404 Primary	GBIF: https://doi.org/10.15468/dl.zfaw79
Anopheles aquasalis	-53.21796	5.48012 Primary	GBIF: https://doi.org/10.15468/dl.zfaw79
Anopheles aquasalis	-53.205199	5.47958 Primary	GBIF: https://doi.org/10.15468/dl.zfaw79
Anopheles aquasalis	-53.13625	5.45004 Primary	GBIF: https://doi.org/10.15468/dl.zfaw79
Anopheles aquasalis	-53.08936	5.42827 Primary	GBIF: https://doi.org/10.15468/dl.zfaw79
Anopheles aquasalis	-53.083355	5.416688 Primary	GBIF: https://doi.org/10.15468/dl.zfaw79
Anopheles aquasalis	-53.0745	5.4234 Primary	GBIF: https://doi.org/10.15468/dl.zfaw79
Anopheles aquasalis	-53.050025	5.062018 Primary	GBIF: https://doi.org/10.15468/dl.zfaw79
Anopheles aquasalis	-53.03179	5.41668 Primary	GBIF: https://doi.org/10.15468/dl.zfaw79
Anopheles aquasalis	-53.02915	5.01314 Primary	GBIF: https://doi.org/10.15468/dl.zfaw79
Anopheles aquasalis	-52.99943	5.40638 Primary	GBIF: https://doi.org/10.15468/dl.zfaw79
Anopheles aquasalis	-52.983355	5.383348 Primary	GBIF: https://doi.org/10.15468/dl.zfaw79
Anopheles aquasalis	-52.958187	5.377037 Primary	GBIF: https://doi.org/10.15468/dl.zfaw79
Anopheles aquasalis	-52.950025	5.316688 Primary	GBIF: https://doi.org/10.15468/dl.zfaw79
Anopheles aquasalis	-52.900025	5.166684 Primary	GBIF: https://doi.org/10.15468/dl.zfaw79
Anopheles aquasalis	-52.846531	5.347491 Primary	GBIF: https://doi.org/10.15468/dl.zfaw79
Anopheles aquasalis	-52.8307	5.29361 Primary	GBIF: https://doi.org/10.15468/dl.zfaw79
Anopheles aquasalis	-52.76898	5.21603 Primary	GBIF: https://doi.org/10.15468/dl.zfaw79
Anopheles aquasalis	-52.756745	5.153398 Primary	GBIF: https://doi.org/10.15468/dl.zfaw79
Anopheles aquasalis	-52.738605	5.115338 Primary	GBIF: https://doi.org/10.15468/dl.zfaw79
Anopheles aquasalis	-52.700025	5.016688 Primary	GBIF: https://doi.org/10.15468/dl.zfaw79
Anopheles aquasalis	-52.66885	5.15907 Primary	GBIF: https://doi.org/10.15468/dl.zfaw79
Anopheles aquasalis	-52.650278	5.159722 Primary	GBIF: https://doi.org/10.15468/dl.zfaw79
Anopheles aquasalis	-52.643374	5.166931 Primary	GBIF: https://doi.org/10.15468/dl.zfaw79

Anopheles aquasalis	-52.63905	5.15894	Primary	GBIF: https://doi.org/10.15468/dl.zfaw79
Anopheles aquasalis	-52.593636	3.256962	Primary	GBIF: https://doi.org/10.15468/dl.zfaw79
Anopheles aquasalis	-52.57423	5.03313	Primary	GBIF: https://doi.org/10.15468/dl.zfaw79
Anopheles aquasalis	-52.52501	5.02128	Primary	GBIF: https://doi.org/10.15468/dl.zfaw79
Anopheles aquasalis	-52.509685	4.832668	Primary	GBIF: https://doi.org/10.15468/dl.zfaw79
Anopheles aquasalis	-52.508494	4.99794	Primary	GBIF: https://doi.org/10.15468/dl.zfaw79
Anopheles aquasalis	-52.500025	4.950018	Primary	GBIF: https://doi.org/10.15468/dl.zfaw79
Anopheles aquasalis	-52.492926	4.892476	Primary	GBIF: https://doi.org/10.15468/dl.zfaw79
Anopheles aquasalis	-52.473837	5.013996	Primary	GBIF: https://doi.org/10.15468/dl.zfaw79
Anopheles aquasalis	-52.47241	5.01171	Primary	GBIF: https://doi.org/10.15468/dl.zfaw79
Anopheles aquasalis	-52.47027	4.572008	Primary	GBIF: https://doi.org/10.15468/dl.zfaw79
Anopheles aquasalis	-52.464045	4.988308	Primary	GBIF: https://doi.org/10.15468/dl.zfaw79
Anopheles aquasalis	-52.45662	4.792945	Primary	GBIF: https://doi.org/10.15468/dl.zfaw79
Anopheles aquasalis	-52.443289	4.83079	Primary	GBIF: https://doi.org/10.15468/dl.zfaw79
Anopheles aquasalis	-52.434423	4.780484	Primary	GBIF: https://doi.org/10.15468/dl.zfaw79
Anopheles aquasalis	-52.431005	4.907008	Primary	GBIF: https://doi.org/10.15468/dl.zfaw79
Anopheles aquasalis	-52.41792	4.7801	Primary	GBIF: https://doi.org/10.15468/dl.zfaw79
Anopheles aquasalis	-52.406535	4.910838	Primary	GBIF: https://doi.org/10.15468/dl.zfaw79
Anopheles aquasalis	-52.40423	4.94154	Primary	GBIF: https://doi.org/10.15468/dl.zfaw79
Anopheles aquasalis	-52.383355	4.883348	Primary	GBIF: https://doi.org/10.15468/dl.zfaw79
Anopheles aquasalis	-52.371136	4.878629	Primary	GBIF: https://doi.org/10.15468/dl.zfaw79
Anopheles aquasalis	-52.361061	4.81862	Primary	GBIF: https://doi.org/10.15468/dl.zfaw79
Anopheles aquasalis	-52.360475	4.819828	Primary	GBIF: https://doi.org/10.15468/dl.zfaw79
Anopheles aquasalis	-52.35913	4.90255	Primary	GBIF: https://doi.org/10.15468/dl.zfaw79
Anopheles aquasalis	-52.357135	4.886868	Primary	GBIF: https://doi.org/10.15468/dl.zfaw79
Anopheles aquasalis	-52.351905	4.788018	Primary	GBIF: https://doi.org/10.15468/dl.zfaw79
Anopheles aquasalis	-52.35125	4.88011	Primary	GBIF: https://doi.org/10.15468/dl.zfaw79
Anopheles aquasalis	-52.348155	4.886318	Primary	GBIF: https://doi.org/10.15468/dl.zfaw79
Anopheles aquasalis	-52.34352	4.77908	Primary	GBIF: https://doi.org/10.15468/dl.zfaw79
Anopheles aquasalis	-52.3435	4.88147	Primary	GBIF: https://doi.org/10.15468/dl.zfaw79
Anopheles aquasalis	-52.34241	4.89956	Primary	GBIF: https://doi.org/10.15468/dl.zfaw79
Anopheles aquasalis	-52.341575	4.823418	Primary	GBIF: https://doi.org/10.15468/dl.zfaw79
Anopheles aquasalis	-52.34122	4.89382	Primary	GBIF: https://doi.org/10.15468/dl.zfaw79
Anopheles aquasalis	-52.340858	4.894185	Primary	GBIF: https://doi.org/10.15468/dl.zfaw79
Anopheles aquasalis	-52.34003	4.81928	Primary	GBIF: https://doi.org/10.15468/dl.zfaw79
Anopheles aquasalis	-52.33772	4.87499	Primary	GBIF: https://doi.org/10.15468/dl.zfaw79
Anopheles aquasalis	-52.33696	4.83273	Primary	GBIF: https://doi.org/10.15468/dl.zfaw79
Anopheles aquasalis	-52.33554	4.92061	Primary	GBIF: https://doi.org/10.15468/dl.zfaw79
Anopheles aquasalis	-52.33458	4.93011	Primary	GBIF: https://doi.org/10.15468/dl.zfaw79
Anopheles aquasalis	-52.33458	4.8301	Primary	GBIF: https://doi.org/10.15468/dl.zfaw79
Anopheles aquasalis	-52.334495	4.941608	Primary	GBIF: https://doi.org/10.15468/dl.zfaw79
Anopheles aquasalis	-52.334255	4.788748	Primary	GBIF: https://doi.org/10.15468/dl.zfaw79

Anopheles aquasalis	-52.334095	3.160908	Primary	GBIF: https://doi.org/10.15468/dl.zfaw79
Anopheles aquasalis	-52.33348	4.93332	Primary	GBIF: https://doi.org/10.15468/dl.zfaw79
Anopheles aquasalis	-52.333355	4.833348	Primary	GBIF: https://doi.org/10.15468/dl.zfaw79
Anopheles aquasalis	-52.333355	4.850018	Primary	GBIF: https://doi.org/10.15468/dl.zfaw79
Anopheles aquasalis	-52.333115	4.816198	Primary	GBIF: https://doi.org/10.15468/dl.zfaw79
Anopheles aquasalis	-52.332371	4.767255	Primary	GBIF: https://doi.org/10.15468/dl.zfaw79
Anopheles aquasalis	-52.332154	4.943859	Primary	GBIF: https://doi.org/10.15468/dl.zfaw79
Anopheles aquasalis	-52.331442	4.935653	Primary	GBIF: https://doi.org/10.15468/dl.zfaw79
Anopheles aquasalis	-52.330987	4.850608	Primary	GBIF: https://doi.org/10.15468/dl.zfaw79
Anopheles aquasalis	-52.33033	4.93461	Primary	GBIF: https://doi.org/10.15468/dl.zfaw79
Anopheles aquasalis	-52.330005	4.921208	Primary	GBIF: https://doi.org/10.15468/dl.zfaw79
Anopheles aquasalis	-52.328392	3.172584	Primary	GBIF: https://doi.org/10.15468/dl.zfaw79
Anopheles aquasalis	-52.326965	4.883998	Primary	GBIF: https://doi.org/10.15468/dl.zfaw79
Anopheles aquasalis	-52.326495	4.915138	Primary	GBIF: https://doi.org/10.15468/dl.zfaw79
Anopheles aquasalis	-52.32628	4.727944	Primary	GBIF: https://doi.org/10.15468/dl.zfaw79
Anopheles aquasalis	-52.325665	4.944568	Primary	GBIF: https://doi.org/10.15468/dl.zfaw79
Anopheles aquasalis	-52.325445	4.933458	Primary	GBIF: https://doi.org/10.15468/dl.zfaw79
Anopheles aquasalis	-52.324865	4.935998	Primary	GBIF: https://doi.org/10.15468/dl.zfaw79
Anopheles aquasalis	-52.323685	4.920628	Primary	GBIF: https://doi.org/10.15468/dl.zfaw79
Anopheles aquasalis	-52.323296	4.936976	Primary	GBIF: https://doi.org/10.15468/dl.zfaw79
Anopheles aquasalis	-52.323248	4.921181	Primary	GBIF: https://doi.org/10.15468/dl.zfaw79
Anopheles aquasalis	-52.32266	4.84545	Primary	GBIF: https://doi.org/10.15468/dl.zfaw79
Anopheles aquasalis	-52.322317	4.931876	Primary	GBIF: https://doi.org/10.15468/dl.zfaw79
Anopheles aquasalis	-52.321842	3.171168	Primary	GBIF: https://doi.org/10.15468/dl.zfaw79
Anopheles aquasalis	-52.32059	4.73866	Primary	GBIF: https://doi.org/10.15468/dl.zfaw79
Anopheles aquasalis	-52.320395	4.903308	Primary	GBIF: https://doi.org/10.15468/dl.zfaw79
Anopheles aquasalis	-52.320214	4.939807	Primary	GBIF: https://doi.org/10.15468/dl.zfaw79
Anopheles aquasalis	-52.319958	4.943506	Primary	GBIF: https://doi.org/10.15468/dl.zfaw79
Anopheles aquasalis	-52.319815	4.849048	Primary	GBIF: https://doi.org/10.15468/dl.zfaw79
Anopheles aquasalis	-52.3192	4.88391	Primary	GBIF: https://doi.org/10.15468/dl.zfaw79
Anopheles aquasalis	-52.318795	3.170558	Primary	GBIF: https://doi.org/10.15468/dl.zfaw79
Anopheles aquasalis	-52.316695	4.516688	Primary	GBIF: https://doi.org/10.15468/dl.zfaw79
Anopheles aquasalis	-52.315858	4.873629	Primary	GBIF: https://doi.org/10.15468/dl.zfaw79
Anopheles aquasalis	-52.313549	4.923477	Primary	GBIF: https://doi.org/10.15468/dl.zfaw79
Anopheles aquasalis	-52.312455	4.927798	Primary	GBIF: https://doi.org/10.15468/dl.zfaw79
Anopheles aquasalis	-52.30826	4.83195	Primary	GBIF: https://doi.org/10.15468/dl.zfaw79
Anopheles aquasalis	-52.307803	4.890296	Primary	GBIF: https://doi.org/10.15468/dl.zfaw79
Anopheles aquasalis	-52.30742	4.94114	Primary	GBIF: https://doi.org/10.15468/dl.zfaw79
Anopheles aquasalis	-52.307335	4.833438	Primary	GBIF: https://doi.org/10.15468/dl.zfaw79
Anopheles aquasalis	-52.307198	4.627222	Primary	GBIF: https://doi.org/10.15468/dl.zfaw79
Anopheles aquasalis	-52.306695	4.940978	Primary	GBIF: https://doi.org/10.15468/dl.zfaw79
Anopheles aquasalis	-52.304705	4.946748	Primary	GBIF: https://doi.org/10.15468/dl.zfaw79

Anopheles aquasalis	-52.30125	4.89677	Primary	GBIF: https://doi.org/10.15468/dl.zfaw79
Anopheles aquasalis	-52.29797	4.90772	Primary	GBIF: https://doi.org/10.15468/dl.zfaw79
Anopheles aquasalis	-52.292208	4.926687	Primary	GBIF: https://doi.org/10.15468/dl.zfaw79
Anopheles aquasalis	-52.291065	4.890268	Primary	GBIF: https://doi.org/10.15468/dl.zfaw79
Anopheles aquasalis	-52.29017	4.92854	Primary	GBIF: https://doi.org/10.15468/dl.zfaw79
Anopheles aquasalis	-52.290025	4.896962	Primary	GBIF: https://doi.org/10.15468/dl.zfaw79
Anopheles aquasalis	-52.28941	4.9089	Primary	GBIF: https://doi.org/10.15468/dl.zfaw79
Anopheles aquasalis	-52.28775	4.93178	Primary	GBIF: https://doi.org/10.15468/dl.zfaw79
Anopheles aquasalis	-52.28725	4.93915	Primary	GBIF: https://doi.org/10.15468/dl.zfaw79
Anopheles aquasalis	-52.28659	4.89308	Primary	GBIF: https://doi.org/10.15468/dl.zfaw79
Anopheles aquasalis	-52.284085	4.920338	Primary	GBIF: https://doi.org/10.15468/dl.zfaw79
Anopheles aquasalis	-52.28402	4.91235	Primary	GBIF: https://doi.org/10.15468/dl.zfaw79
Anopheles aquasalis	-52.283358	4.921685	Primary	GBIF: https://doi.org/10.15468/dl.zfaw79
Anopheles aquasalis	-52.281692	4.875851	Primary	GBIF: https://doi.org/10.15468/dl.zfaw79
Anopheles aquasalis	-52.279846	4.717854	Primary	GBIF: https://doi.org/10.15468/dl.zfaw79
Anopheles aquasalis	-52.279143	4.718157	Primary	GBIF: https://doi.org/10.15468/dl.zfaw79
Anopheles aquasalis	-52.276221	4.886853	Primary	GBIF: https://doi.org/10.15468/dl.zfaw79
Anopheles aquasalis	-52.27475	4.85384	Primary	GBIF: https://doi.org/10.15468/dl.zfaw79
Anopheles aquasalis	-52.272123	4.912182	Primary	GBIF: https://doi.org/10.15468/dl.zfaw79
Anopheles aquasalis	-52.26791	4.91344	Primary	GBIF: https://doi.org/10.15468/dl.zfaw79
Anopheles aquasalis	-52.265025	4.901129	Primary	GBIF: https://doi.org/10.15468/dl.zfaw79
Anopheles aquasalis	-52.259305	4.911678	Primary	GBIF: https://doi.org/10.15468/dl.zfaw79
Anopheles aquasalis	-52.256515	4.857598	Primary	GBIF: https://doi.org/10.15468/dl.zfaw79
Anopheles aquasalis	-52.251475	4.881148	Primary	GBIF: https://doi.org/10.15468/dl.zfaw79
Anopheles aquasalis	-52.25125	4.88011	Primary	GBIF: https://doi.org/10.15468/dl.zfaw79
Anopheles aquasalis	-52.153995	4.315458	Primary	GBIF: https://doi.org/10.15468/dl.zfaw79
Anopheles aquasalis	-52.129162	4.314002	Primary	GBIF: https://doi.org/10.15468/dl.zfaw79
Anopheles aquasalis	-52.037094	4.486403	Primary	GBIF: https://doi.org/10.15468/dl.zfaw79
Anopheles aquasalis	-51.855225	3.870678	Primary	GBIF: https://doi.org/10.15468/dl.zfaw79
Anopheles aquasalis	-51.808359	3.891601	Primary	GBIF: https://doi.org/10.15468/dl.zfaw79
Anopheles aquasalis	-51.806695	3.888638	Primary	GBIF: https://doi.org/10.15468/dl.zfaw79
Anopheles aquasalis	-51.801158	3.888398	Primary	GBIF: https://doi.org/10.15468/dl.zfaw79
Anopheles aquasalis	-48.48369	-1.45007	Primary	GBIF: https://doi.org/10.15468/dl.zfaw79
Anopheles aquasalis	-43.2075	-22.9028	Primary	GBIF: https://doi.org/10.15468/dl.zfaw79
Anopheles aquasalis	-43.0416	-22.96705	Primary	GBIF: https://doi.org/10.15468/dl.zfaw79
Anopheles aquasalis	-42.653099	-22.4625	Primary	GBIF: https://doi.org/10.15468/dl.zfaw79
Anopheles aquasalis	-40.56705	-16.26713	Primary	GBIF: https://doi.org/10.15468/dl.zfaw79
Anopheles aquasalis	-37.76658	-4.56678	Primary	GBIF: https://doi.org/10.15468/dl.zfaw79
Anopheles aquasalis	-34.89979	-8.05016	Primary	GBIF: https://doi.org/10.15468/dl.zfaw79
Anopheles aquasalis	7.114444	48.171665	Primary	GBIF: https://doi.org/10.15468/dl.zfaw79
Anopheles arabiensis	-22.8833	16.1333	Unclassified	GBIF: https://doi.org/10.15468/dl.zfaw79
Anopheles arabiensis	-17.05	14.95	Unclassified	GBIF: https://doi.org/10.15468/dl.zfaw79

Anopheles arabiensis	-16.86667	14.81667	Unclassified	GBIF: https://doi.org/10.15468/dl.zfaw79
Anopheles arabiensis	-16.75	13.33333	Unclassified	GBIF: https://doi.org/10.15468/dl.zfaw79
Anopheles arabiensis	-16.71667	15.43333	Unclassified	GBIF: https://doi.org/10.15468/dl.zfaw79
Anopheles arabiensis	-16.66667	14.53333	Unclassified	GBIF: https://doi.org/10.15468/dl.zfaw79
Anopheles arabiensis	-16.5928	13.2806	Unclassified	GBIF: https://doi.org/10.15468/dl.zfaw79
Anopheles arabiensis	-16.5919	13.2256	Unclassified	GBIF: https://doi.org/10.15468/dl.zfaw79
Anopheles arabiensis	-16.5761	13.1794	Unclassified	GBIF: https://doi.org/10.15468/dl.zfaw79
Anopheles arabiensis	-16.5	16.01667	Unclassified	GBIF: https://doi.org/10.15468/dl.zfaw79
Anopheles arabiensis	-16.46667	14.66667	Unclassified	GBIF: https://doi.org/10.15468/dl.zfaw79
Anopheles arabiensis	-16.4	15.95	Unclassified	GBIF: https://doi.org/10.15468/dl.zfaw79
Anopheles arabiensis	-16.38333	13.68333	Unclassified	GBIF: https://doi.org/10.15468/dl.zfaw79
Anopheles arabiensis	-16.38333	15.98333	Unclassified	GBIF: https://doi.org/10.15468/dl.zfaw79
Anopheles arabiensis	-16.36667	13.25	Unclassified	GBIF: https://doi.org/10.15468/dl.zfaw79
Anopheles arabiensis	-16.36667	12.65	Unclassified	GBIF: https://doi.org/10.15468/dl.zfaw79
Anopheles arabiensis	-16.35	14.46667	Unclassified	GBIF: https://doi.org/10.15468/dl.zfaw79
Anopheles arabiensis	-16.33333	13.86667	Unclassified	GBIF: https://doi.org/10.15468/dl.zfaw79
Anopheles arabiensis	-16.31667	12.7	Unclassified	GBIF: https://doi.org/10.15468/dl.zfaw79
Anopheles arabiensis	-16.28333	13.46667	Unclassified	GBIF: https://doi.org/10.15468/dl.zfaw79
Anopheles arabiensis	-16.25	14.26667	Unclassified	GBIF: https://doi.org/10.15468/dl.zfaw79
Anopheles arabiensis	-16.0836	13.6183	Unclassified	GBIF: https://doi.org/10.15468/dl.zfaw79
Anopheles arabiensis	-16.06667	14.16667	Unclassified	GBIF: https://doi.org/10.15468/dl.zfaw79
Anopheles arabiensis	-16	13.33333	Unclassified	GBIF: https://doi.org/10.15468/dl.zfaw79
Anopheles arabiensis	-15.91667	13.91667	Unclassified	GBIF: https://doi.org/10.15468/dl.zfaw79
Anopheles arabiensis	-15.8	13.36667	Unclassified	GBIF: https://doi.org/10.15468/dl.zfaw79
Anopheles arabiensis	-15.78333	13.48333	Unclassified	GBIF: https://doi.org/10.15468/dl.zfaw79
Anopheles arabiensis	-15.75	13.41667	Unclassified	GBIF: https://doi.org/10.15468/dl.zfaw79
Anopheles arabiensis	-15.71667	13.43333	Unclassified	GBIF: https://doi.org/10.15468/dl.zfaw79
Anopheles arabiensis	-15.7	16.41667	Unclassified	GBIF: https://doi.org/10.15468/dl.zfaw79
Anopheles arabiensis	-15.58333	13.5	Unclassified	GBIF: https://doi.org/10.15468/dl.zfaw79
Anopheles arabiensis	-15.56667	13.48333	Unclassified	GBIF: https://doi.org/10.15468/dl.zfaw79
Anopheles arabiensis	-15.56667	13.56667	Unclassified	GBIF: https://doi.org/10.15468/dl.zfaw79
Anopheles arabiensis	-15.53333	13.2	Unclassified	GBIF: https://doi.org/10.15468/dl.zfaw79
Anopheles arabiensis	-15.5	13.5	Unclassified	GBIF: https://doi.org/10.15468/dl.zfaw79
Anopheles arabiensis	-15.03333	16.58333	Unclassified	GBIF: https://doi.org/10.15468/dl.zfaw79
Anopheles arabiensis	-15	13.6	Unclassified	GBIF: https://doi.org/10.15468/dl.zfaw79
Anopheles arabiensis	-14.93333	13.58333	Unclassified	GBIF: https://doi.org/10.15468/dl.zfaw79
Anopheles arabiensis	-14.91667	12.93333	Unclassified	GBIF: https://doi.org/10.15468/dl.zfaw79
Anopheles arabiensis	-14.88333	15.28333	Unclassified	GBIF: https://doi.org/10.15468/dl.zfaw79
Anopheles arabiensis	-14.83333	13.51667	Unclassified	GBIF: https://doi.org/10.15468/dl.zfaw79
Anopheles arabiensis	-14.75	16.55	Unclassified	GBIF: https://doi.org/10.15468/dl.zfaw79
Anopheles arabiensis	-14.7	16.01667	Unclassified	GBIF: https://doi.org/10.15468/dl.zfaw79
Anopheles arabiensis	-14.66667	13.43333	Unclassified	GBIF: https://doi.org/10.15468/dl.zfaw79

Anopheles arabiensis	-14.65	16.51667	Unclassified	GBIF: https://doi.org/10.15468/dl.zfaw79
Anopheles arabiensis	-14.45	12.33333	Unclassified	GBIF: https://doi.org/10.15468/dl.zfaw79
Anopheles arabiensis	-14.25	13.38333	Unclassified	GBIF: https://doi.org/10.15468/dl.zfaw79
Anopheles arabiensis	-14.25	13.33333	Unclassified	GBIF: https://doi.org/10.15468/dl.zfaw79
Anopheles arabiensis	-14.23333	16.08333	Unclassified	GBIF: https://doi.org/10.15468/dl.zfaw79
Anopheles arabiensis	-13.66667	15.65	Unclassified	GBIF: https://doi.org/10.15468/dl.zfaw79
Anopheles arabiensis	-11.55	13.75	Unclassified	GBIF: https://doi.org/10.15468/dl.zfaw79
Anopheles arabiensis	-11.46667	14.43333	Unclassified	GBIF: https://doi.org/10.15468/dl.zfaw79
Anopheles arabiensis	-10.91667	14.46667	Unclassified	GBIF: https://doi.org/10.15468/dl.zfaw79
Anopheles arabiensis	-10.83333	13.81667	Unclassified	GBIF: https://doi.org/10.15468/dl.zfaw79
Anopheles arabiensis	-10.75	13.45	Unclassified	GBIF: https://doi.org/10.15468/dl.zfaw79
Anopheles arabiensis	-10.75	14.8	Unclassified	GBIF: https://doi.org/10.15468/dl.zfaw79
Anopheles arabiensis	-10.58333	15.06667	Unclassified	GBIF: https://doi.org/10.15468/dl.zfaw79
Anopheles arabiensis	-10.5	13.25	Unclassified	GBIF: https://doi.org/10.15468/dl.zfaw79
Anopheles arabiensis	-9.75	14.55	Unclassified	GBIF: https://doi.org/10.15468/dl.zfaw79
Anopheles arabiensis	-9.5	12.91667	Unclassified	GBIF: https://doi.org/10.15468/dl.zfaw79
Anopheles arabiensis	-9.13333	15.06667	Unclassified	GBIF: https://doi.org/10.15468/dl.zfaw79
Anopheles arabiensis	-8.33333	13.36667	Unclassified	GBIF: https://doi.org/10.15468/dl.zfaw79
Anopheles arabiensis	-8.31667	11.41667	Unclassified	GBIF: https://doi.org/10.15468/dl.zfaw79
Anopheles arabiensis	-8.28333	11.7	Unclassified	GBIF: https://doi.org/10.15468/dl.zfaw79
Anopheles arabiensis	-8.26667	12.41667	Unclassified	GBIF: https://doi.org/10.15468/dl.zfaw79
Anopheles arabiensis	-8.26667	12.8	Unclassified	GBIF: https://doi.org/10.15468/dl.zfaw79
Anopheles arabiensis	-8.21667	12.5	Unclassified	GBIF: https://doi.org/10.15468/dl.zfaw79
Anopheles arabiensis	-8.18333	11.66667	Unclassified	GBIF: https://doi.org/10.15468/dl.zfaw79
Anopheles arabiensis	-8.08333	13.91667	Unclassified	GBIF: https://doi.org/10.15468/dl.zfaw79
Anopheles arabiensis	-8.05	12.8	Unclassified	GBIF: https://doi.org/10.15468/dl.zfaw79
Anopheles arabiensis	-8	12.46667	Unclassified	GBIF: https://doi.org/10.15468/dl.zfaw79
Anopheles arabiensis	-7.98333	12.66667	Unclassified	GBIF: https://doi.org/10.15468/dl.zfaw79
Anopheles arabiensis	-7.95	12.68333	Unclassified	GBIF: https://doi.org/10.15468/dl.zfaw79
Anopheles arabiensis	-7.91667	12.51667	Unclassified	GBIF: https://doi.org/10.15468/dl.zfaw79
Anopheles arabiensis	-7.75	14.33333	Unclassified	GBIF: https://doi.org/10.15468/dl.zfaw79
Anopheles arabiensis	-7.63333	12.91667	Unclassified	GBIF: https://doi.org/10.15468/dl.zfaw79
Anopheles arabiensis	-7.5	16.5	Unclassified	GBIF: https://doi.org/10.15468/dl.zfaw79
Anopheles arabiensis	-7.46667	14.56667	Unclassified	GBIF: https://doi.org/10.15468/dl.zfaw79
Anopheles arabiensis	-7.41667	14.58333	Unclassified	GBIF: https://doi.org/10.15468/dl.zfaw79
Anopheles arabiensis	-7.3	13.86667	Unclassified	GBIF: https://doi.org/10.15468/dl.zfaw79
Anopheles arabiensis	-7.28333	13.86667	Unclassified	GBIF: https://doi.org/10.15468/dl.zfaw79
Anopheles arabiensis	-7.17	13.55	Unclassified	GBIF: https://doi.org/10.15468/dl.zfaw79
Anopheles arabiensis	-7.1	13.85	Unclassified	GBIF: https://doi.org/10.15468/dl.zfaw79
Anopheles arabiensis	-6.63333	14.15	Unclassified	GBIF: https://doi.org/10.15468/dl.zfaw79
Anopheles arabiensis	-6.63333	13.01667	Unclassified	GBIF: https://doi.org/10.15468/dl.zfaw79
Anopheles arabiensis	-6.45	13.3	Unclassified	GBIF: https://doi.org/10.15468/dl.zfaw79

Anopheles arabiensis	-6.35	13.4	Unclassified	GBIF: https://doi.org/10.15468/dl.zfaw79
Anopheles arabiensis	-6.3	13.46667	Unclassified	GBIF: https://doi.org/10.15468/dl.zfaw79
Anopheles arabiensis	-6.21667	13.33333	Unclassified	GBIF: https://doi.org/10.15468/dl.zfaw79
Anopheles arabiensis	-6.18333	14.88333	Unclassified	GBIF: https://doi.org/10.15468/dl.zfaw79
Anopheles arabiensis	-6.08333	13.68333	Unclassified	GBIF: https://doi.org/10.15468/dl.zfaw79
Anopheles arabiensis	-6.01667	14.73333	Unclassified	GBIF: https://doi.org/10.15468/dl.zfaw79
Anopheles arabiensis	-5.91667	13.21667	Unclassified	GBIF: https://doi.org/10.15468/dl.zfaw79
Anopheles arabiensis	-5.78333	12.8	Unclassified	GBIF: https://doi.org/10.15468/dl.zfaw79
Anopheles arabiensis	-5.58333	12.21667	Unclassified	GBIF: https://doi.org/10.15468/dl.zfaw79
Anopheles arabiensis	-5.3	13.11667	Unclassified	GBIF: https://doi.org/10.15468/dl.zfaw79
Anopheles arabiensis	-5.03333	12.26667	Unclassified	GBIF: https://doi.org/10.15468/dl.zfaw79
Anopheles arabiensis	-5.03333	12.71667	Unclassified	GBIF: https://doi.org/10.15468/dl.zfaw79
Anopheles arabiensis	-4.96667	11.16667	Unclassified	GBIF: https://doi.org/10.15468/dl.zfaw79
Anopheles arabiensis	-4.91667	10.28333	Unclassified	GBIF: https://doi.org/10.15468/dl.zfaw79
Anopheles arabiensis	-4.55	11.58333	Unclassified	GBIF: https://doi.org/10.15468/dl.zfaw79
Anopheles arabiensis	-4.46667	11.26667	Unclassified	GBIF: https://doi.org/10.15468/dl.zfaw79
Anopheles arabiensis	-4.38333	11.38333	Unclassified	GBIF: https://doi.org/10.15468/dl.zfaw79
Anopheles arabiensis	-4.33333	13.86667	Unclassified	GBIF: https://doi.org/10.15468/dl.zfaw79
Anopheles arabiensis	-4.3	11.18333	Unclassified	GBIF: https://doi.org/10.15468/dl.zfaw79
Anopheles arabiensis	-4.25	11.48333	Unclassified	GBIF: https://doi.org/10.15468/dl.zfaw79
Anopheles arabiensis	-4.23333	11.15	Unclassified	GBIF: https://doi.org/10.15468/dl.zfaw79
Anopheles arabiensis	-4.2	11.43333	Unclassified	GBIF: https://doi.org/10.15468/dl.zfaw79
Anopheles arabiensis	-4.2	11.13333	Unclassified	GBIF: https://doi.org/10.15468/dl.zfaw79
Anopheles arabiensis	-4.11667	14.55	Unclassified	GBIF: https://doi.org/10.15468/dl.zfaw79
Anopheles arabiensis	-4.05	11.06667	Unclassified	GBIF: https://doi.org/10.15468/dl.zfaw79
Anopheles arabiensis	-4	11.51667	Unclassified	GBIF: https://doi.org/10.15468/dl.zfaw79
Anopheles arabiensis	-3.81667	11.7	Unclassified	GBIF: https://doi.org/10.15468/dl.zfaw79
Anopheles arabiensis	-3.78333	14.41667	Unclassified	GBIF: https://doi.org/10.15468/dl.zfaw79
Anopheles arabiensis	-3.66667	10.91667	Unclassified	GBIF: https://doi.org/10.15468/dl.zfaw79
Anopheles arabiensis	-3.63333	11.3	Unclassified	GBIF: https://doi.org/10.15468/dl.zfaw79
Anopheles arabiensis	-3.56667	14.4	Unclassified	GBIF: https://doi.org/10.15468/dl.zfaw79
Anopheles arabiensis	-3.51667	11.13333	Unclassified	GBIF: https://doi.org/10.15468/dl.zfaw79
Anopheles arabiensis	-3.477567	10.795097	Unclassified	GBIF: https://doi.org/10.15468/dl.zfaw79
Anopheles arabiensis	-3.472538	10.858087	Unclassified	GBIF: https://doi.org/10.15468/dl.zfaw79
Anopheles arabiensis	-3.453171	10.92178	Unclassified	GBIF: https://doi.org/10.15468/dl.zfaw79
Anopheles arabiensis	-3.453132	10.920139	Unclassified	GBIF: https://doi.org/10.15468/dl.zfaw79
Anopheles arabiensis	-3.453089	10.921504	Unclassified	GBIF: https://doi.org/10.15468/dl.zfaw79
Anopheles arabiensis	-3.452748	10.920485	Unclassified	GBIF: https://doi.org/10.15468/dl.zfaw79
Anopheles arabiensis	-3.445582	10.814674	Unclassified	GBIF: https://doi.org/10.15468/dl.zfaw79
Anopheles arabiensis	-3.445563	10.814736	Unclassified	GBIF: https://doi.org/10.15468/dl.zfaw79
Anopheles arabiensis	-3.41667	13.16667	Unclassified	GBIF: https://doi.org/10.15468/dl.zfaw79
Anopheles arabiensis	-3.399661	10.743503	Unclassified	GBIF: https://doi.org/10.15468/dl.zfaw79

Anopheles arabiensis	-3.393933	10.808007	Unclassified	GBIF: https://doi.org/10.15468/dl.zfaw79
Anopheles arabiensis	-3.387473	10.914349	Unclassified	GBIF: https://doi.org/10.15468/dl.zfaw79
Anopheles arabiensis	-3.385578	10.912662	Unclassified	GBIF: https://doi.org/10.15468/dl.zfaw79
Anopheles arabiensis	-3.384354	10.988509	Unclassified	GBIF: https://doi.org/10.15468/dl.zfaw79
Anopheles arabiensis	-3.384267	10.988254	Unclassified	GBIF: https://doi.org/10.15468/dl.zfaw79
Anopheles arabiensis	-3.384052	10.98831	Unclassified	GBIF: https://doi.org/10.15468/dl.zfaw79
Anopheles arabiensis	-3.383707	10.988053	Unclassified	GBIF: https://doi.org/10.15468/dl.zfaw79
Anopheles arabiensis	-3.38333	16.25	Unclassified	GBIF: https://doi.org/10.15468/dl.zfaw79
Anopheles arabiensis	-3.342176	10.781939	Unclassified	GBIF: https://doi.org/10.15468/dl.zfaw79
Anopheles arabiensis	-3.341232	10.782837	Unclassified	GBIF: https://doi.org/10.15468/dl.zfaw79
Anopheles arabiensis	-3.340944	10.782752	Unclassified	GBIF: https://doi.org/10.15468/dl.zfaw79
Anopheles arabiensis	-3.331853	10.856236	Unclassified	GBIF: https://doi.org/10.15468/dl.zfaw79
Anopheles arabiensis	-3.33127	10.827468	Unclassified	GBIF: https://doi.org/10.15468/dl.zfaw79
Anopheles arabiensis	-3.330952	10.826591	Unclassified	GBIF: https://doi.org/10.15468/dl.zfaw79
Anopheles arabiensis	-3.330898	10.825726	Unclassified	GBIF: https://doi.org/10.15468/dl.zfaw79
Anopheles arabiensis	-3.330522	10.824695	Unclassified	GBIF: https://doi.org/10.15468/dl.zfaw79
Anopheles arabiensis	-3.330331	10.825128	Unclassified	GBIF: https://doi.org/10.15468/dl.zfaw79
Anopheles arabiensis	-3.330326	10.825814	Unclassified	GBIF: https://doi.org/10.15468/dl.zfaw79
Anopheles arabiensis	-3.329987	10.892908	Unclassified	GBIF: https://doi.org/10.15468/dl.zfaw79
Anopheles arabiensis	-3.329921	10.891975	Unclassified	GBIF: https://doi.org/10.15468/dl.zfaw79
Anopheles arabiensis	-3.326126	10.891635	Unclassified	GBIF: https://doi.org/10.15468/dl.zfaw79
Anopheles arabiensis	-3.325897	10.893405	Unclassified	GBIF: https://doi.org/10.15468/dl.zfaw79
Anopheles arabiensis	-3.321167	10.852973	Unclassified	GBIF: https://doi.org/10.15468/dl.zfaw79
Anopheles arabiensis	-3.32079	10.852812	Unclassified	GBIF: https://doi.org/10.15468/dl.zfaw79
Anopheles arabiensis	-3.315687	10.852369	Unclassified	GBIF: https://doi.org/10.15468/dl.zfaw79
Anopheles arabiensis	-3.313747	10.707981	Unclassified	GBIF: https://doi.org/10.15468/dl.zfaw79
Anopheles arabiensis	-3.295975	10.669052	Unclassified	GBIF: https://doi.org/10.15468/dl.zfaw79
Anopheles arabiensis	-3.29583	10.668199	Unclassified	GBIF: https://doi.org/10.15468/dl.zfaw79
Anopheles arabiensis	-3.295755	10.667941	Unclassified	GBIF: https://doi.org/10.15468/dl.zfaw79
Anopheles arabiensis	-3.295173	10.668426	Unclassified	GBIF: https://doi.org/10.15468/dl.zfaw79
Anopheles arabiensis	-3.2879	10.711338	Unclassified	GBIF: https://doi.org/10.15468/dl.zfaw79
Anopheles arabiensis	-3.287385	10.711307	Unclassified	GBIF: https://doi.org/10.15468/dl.zfaw79
Anopheles arabiensis	-3.286987	10.712953	Unclassified	GBIF: https://doi.org/10.15468/dl.zfaw79
Anopheles arabiensis	-3.28698	10.712808	Unclassified	GBIF: https://doi.org/10.15468/dl.zfaw79
Anopheles arabiensis	-3.256134	10.749538	Unclassified	GBIF: https://doi.org/10.15468/dl.zfaw79
Anopheles arabiensis	-3.255155	10.749496	Unclassified	GBIF: https://doi.org/10.15468/dl.zfaw79
Anopheles arabiensis	-3.223079	10.733179	Unclassified	GBIF: https://doi.org/10.15468/dl.zfaw79
Anopheles arabiensis	-3.220803	10.732259	Unclassified	GBIF: https://doi.org/10.15468/dl.zfaw79
Anopheles arabiensis	-3.212989	10.738675	Unclassified	GBIF: https://doi.org/10.15468/dl.zfaw79
Anopheles arabiensis	-3.212977	10.739225	Unclassified	GBIF: https://doi.org/10.15468/dl.zfaw79
Anopheles arabiensis	-3.212624	10.739121	Unclassified	GBIF: https://doi.org/10.15468/dl.zfaw79
Anopheles arabiensis	-3.2	11	Unclassified	GBIF: https://doi.org/10.15468/dl.zfaw79

Anopheles arabiensis	-3.19443	10.963547	Unclassified	GBIF: https://doi.org/10.15468/dl.zfaw79
Anopheles arabiensis	-3.194278	10.974277	Unclassified	GBIF: https://doi.org/10.15468/dl.zfaw79
Anopheles arabiensis	-3.192815	10.963358	Unclassified	GBIF: https://doi.org/10.15468/dl.zfaw79
Anopheles arabiensis	-3.190162	10.96256	Unclassified	GBIF: https://doi.org/10.15468/dl.zfaw79
Anopheles arabiensis	-3.165778	10.738544	Unclassified	GBIF: https://doi.org/10.15468/dl.zfaw79
Anopheles arabiensis	-3.165707	10.738	Unclassified	GBIF: https://doi.org/10.15468/dl.zfaw79
Anopheles arabiensis	-3.165506	10.737966	Unclassified	GBIF: https://doi.org/10.15468/dl.zfaw79
Anopheles arabiensis	-2.96667	15	Unclassified	GBIF: https://doi.org/10.15468/dl.zfaw79
Anopheles arabiensis	-2.9	11.78333	Unclassified	GBIF: https://doi.org/10.15468/dl.zfaw79
Anopheles arabiensis	-2.61667	12.3	Unclassified	GBIF: https://doi.org/10.15468/dl.zfaw79
Anopheles arabiensis	-2.45	12.21667	Unclassified	GBIF: https://doi.org/10.15468/dl.zfaw79
Anopheles arabiensis	-2.38333	12.25	Unclassified	GBIF: https://doi.org/10.15468/dl.zfaw79
Anopheles arabiensis	-2.33333	13.51667	Unclassified	GBIF: https://doi.org/10.15468/dl.zfaw79
Anopheles arabiensis	-1.8	12.3	Unclassified	GBIF: https://doi.org/10.15468/dl.zfaw79
Anopheles arabiensis	-1.75	12.41667	Unclassified	GBIF: https://doi.org/10.15468/dl.zfaw79
Anopheles arabiensis	-1.66667	12.33333	Unclassified	GBIF: https://doi.org/10.15468/dl.zfaw79
Anopheles arabiensis	-1.63333	14.15	Unclassified	GBIF: https://doi.org/10.15468/dl.zfaw79
Anopheles arabiensis	-1.58333	12.36667	Unclassified	GBIF: https://doi.org/10.15468/dl.zfaw79
Anopheles arabiensis	-1.33333	15.8	Unclassified	GBIF: https://doi.org/10.15468/dl.zfaw79
Anopheles arabiensis	-0.33333	16.91667	Unclassified	GBIF: https://doi.org/10.15468/dl.zfaw79
Anopheles arabiensis	-0.03333	14.05	Unclassified	GBIF: https://doi.org/10.15468/dl.zfaw79
Anopheles arabiensis	0.21667	11.81667	Unclassified	GBIF: https://doi.org/10.15468/dl.zfaw79
Anopheles arabiensis	0.41667	10.16667	Unclassified	GBIF: https://doi.org/10.15468/dl.zfaw79
Anopheles arabiensis	0.46667	12.25	Unclassified	GBIF: https://doi.org/10.15468/dl.zfaw79
Anopheles arabiensis	0.61667	18.06667	Unclassified	GBIF: https://doi.org/10.15468/dl.zfaw79
Anopheles arabiensis	0.708	9.376	Unclassified	GBIF: https://doi.org/10.15468/dl.zfaw79
Anopheles arabiensis	0.70801	9.37601	Unclassified	GBIF: https://doi.org/10.15468/dl.zfaw79
Anopheles arabiensis	1.032	9.563	Unclassified	GBIF: https://doi.org/10.15468/dl.zfaw79
Anopheles arabiensis	1.03333	12.46667	Unclassified	GBIF: https://doi.org/10.15468/dl.zfaw79
Anopheles arabiensis	1.33333	18.41667	Unclassified	GBIF: https://doi.org/10.15468/dl.zfaw79
Anopheles arabiensis	1.8	12.15	Unclassified	GBIF: https://doi.org/10.15468/dl.zfaw79
Anopheles arabiensis	1.99834	10.9721	Unclassified	GBIF: https://doi.org/10.15468/dl.zfaw79
Anopheles arabiensis	2.00043	10.2309	Unclassified	GBIF: https://doi.org/10.15468/dl.zfaw79
Anopheles arabiensis	2.08333	13.53333	Unclassified	GBIF: https://doi.org/10.15468/dl.zfaw79
Anopheles arabiensis	2.185	7.78491	Unclassified	GBIF: https://doi.org/10.15468/dl.zfaw79
Anopheles arabiensis	2.3	6.88333	Unclassified	GBIF: https://doi.org/10.15468/dl.zfaw79
Anopheles arabiensis	3.93333	7.38333	Unclassified	GBIF: https://doi.org/10.15468/dl.zfaw79
Anopheles arabiensis	4.18333	12.5	Unclassified	GBIF: https://doi.org/10.15468/dl.zfaw79
Anopheles arabiensis	5.68333	6.31667	Unclassified	GBIF: https://doi.org/10.15468/dl.zfaw79
Anopheles arabiensis	5.76667	5.91667	Unclassified	GBIF: https://doi.org/10.15468/dl.zfaw79
Anopheles arabiensis	6.26667	7.01667	Unclassified	GBIF: https://doi.org/10.15468/dl.zfaw79
Anopheles arabiensis	7	13.73333	Unclassified	GBIF: https://doi.org/10.15468/dl.zfaw79

Anopheles arabiensis	7.23333	10.38333	Unclassified	GBIF: https://doi.org/10.15468/dl.zfaw79
Anopheles arabiensis	7.33333	10.5	Unclassified	GBIF: https://doi.org/10.15468/dl.zfaw79
Anopheles arabiensis	7.65	11.8	Unclassified	GBIF: https://doi.org/10.15468/dl.zfaw79
Anopheles arabiensis	7.81667	12.58333	Unclassified	GBIF: https://doi.org/10.15468/dl.zfaw79
Anopheles arabiensis	8	13.66667	Unclassified	GBIF: https://doi.org/10.15468/dl.zfaw79
Anopheles arabiensis	8	17	Unclassified	GBIF: https://doi.org/10.15468/dl.zfaw79
Anopheles arabiensis	8.33333	15.05	Unclassified	GBIF: https://doi.org/10.15468/dl.zfaw79
Anopheles arabiensis	8.51667	12	Unclassified	GBIF: https://doi.org/10.15468/dl.zfaw79
Anopheles arabiensis	8.75	12.33333	Unclassified	GBIF: https://doi.org/10.15468/dl.zfaw79
Anopheles arabiensis	8.81667	14.91667	Unclassified	GBIF: https://doi.org/10.15468/dl.zfaw79
Anopheles arabiensis	8.899	39.912	Unclassified	GBIF: https://doi.org/10.15468/dl.zfaw79
Anopheles arabiensis	8.89901	39.912	Unclassified	GBIF: https://doi.org/10.15468/dl.zfaw79
Anopheles arabiensis	8.89902	39.912	Unclassified	GBIF: https://doi.org/10.15468/dl.zfaw79
Anopheles arabiensis	8.89903	39.912	Unclassified	GBIF: https://doi.org/10.15468/dl.zfaw79
Anopheles arabiensis	8.993	40.156	Unclassified	GBIF: https://doi.org/10.15468/dl.zfaw79
Anopheles arabiensis	8.99301	40.156	Unclassified	GBIF: https://doi.org/10.15468/dl.zfaw79
Anopheles arabiensis	8.99303	40.156	Unclassified	GBIF: https://doi.org/10.15468/dl.zfaw79
Anopheles arabiensis	9.41667	0.5	Unclassified	GBIF: https://doi.org/10.15468/dl.zfaw79
Anopheles arabiensis	9.91667	10.16667	Unclassified	GBIF: https://doi.org/10.15468/dl.zfaw79
Anopheles arabiensis	13.98333	9.75	Unclassified	GBIF: https://doi.org/10.15468/dl.zfaw79
Anopheles arabiensis	14.35	-17.4333	Unclassified	GBIF: https://doi.org/10.15468/dl.zfaw79
Anopheles arabiensis	14.98333	12.16667	Unclassified	GBIF: https://doi.org/10.15468/dl.zfaw79
Anopheles arabiensis	15.8	-17.78333	Unclassified	GBIF: https://doi.org/10.15468/dl.zfaw79
Anopheles arabiensis	21.65	-18.05	Unclassified	GBIF: https://doi.org/10.15468/dl.zfaw79
Anopheles arabiensis	21.75	-18.08333	Unclassified	GBIF: https://doi.org/10.15468/dl.zfaw79
Anopheles arabiensis	23.3867	-19.1183	Unclassified	GBIF: https://doi.org/10.15468/dl.zfaw79
Anopheles arabiensis	23.41667	-25.41667	Unclassified	GBIF: https://doi.org/10.15468/dl.zfaw79
Anopheles arabiensis	23.41667	-20	Unclassified	GBIF: https://doi.org/10.15468/dl.zfaw79
Anopheles arabiensis	23.7	-25.45	Unclassified	GBIF: https://doi.org/10.15468/dl.zfaw79
Anopheles arabiensis	23.7	-25.5	Unclassified	GBIF: https://doi.org/10.15468/dl.zfaw79
Anopheles arabiensis	23.8683	-19.1217	Unclassified	GBIF: https://doi.org/10.15468/dl.zfaw79
Anopheles arabiensis	23.9333	-25.65	Unclassified	GBIF: https://doi.org/10.15468/dl.zfaw79
Anopheles arabiensis	24.5	-18.16667	Unclassified	GBIF: https://doi.org/10.15468/dl.zfaw79
Anopheles arabiensis	25.15	-17.8167	Unclassified	GBIF: https://doi.org/10.15468/dl.zfaw79
Anopheles arabiensis	26	-18.63333	Unclassified	GBIF: https://doi.org/10.15468/dl.zfaw79
Anopheles arabiensis	26.01667	-20.08333	Unclassified	GBIF: https://doi.org/10.15468/dl.zfaw79
Anopheles arabiensis	26.31667	-18.33333	Unclassified	GBIF: https://doi.org/10.15468/dl.zfaw79
Anopheles arabiensis	26.6	-18.05	Unclassified	GBIF: https://doi.org/10.15468/dl.zfaw79
Anopheles arabiensis	27.41667	-17.75	Unclassified	GBIF: https://doi.org/10.15468/dl.zfaw79
Anopheles arabiensis	27.86667	-19.08333	Unclassified	GBIF: https://doi.org/10.15468/dl.zfaw79
Anopheles arabiensis	28.43333	-10.55	Unclassified	GBIF: https://doi.org/10.15468/dl.zfaw79
Anopheles arabiensis	28.8333	-16.05	Unclassified	GBIF: https://doi.org/10.15468/dl.zfaw79

Anopheles arabiensis	28.9432	-18.2203	Unclassified	GBIF: https://doi.org/10.15468/dl.zfaw79
Anopheles arabiensis	29	-2.83333	Unclassified	GBIF: https://doi.org/10.15468/dl.zfaw79
Anopheles arabiensis	29.16667	-3.36667	Unclassified	GBIF: https://doi.org/10.15468/dl.zfaw79
Anopheles arabiensis	29.2933	-3.1875	Unclassified	GBIF: https://doi.org/10.15468/dl.zfaw79
Anopheles arabiensis	29.36667	-17.91667	Unclassified	GBIF: https://doi.org/10.15468/dl.zfaw79
Anopheles arabiensis	29.7814	-0.885566	Unclassified	GBIF: https://doi.org/10.15468/dl.zfaw79
Anopheles arabiensis	30	-3.33333	Unclassified	GBIF: https://doi.org/10.15468/dl.zfaw79
Anopheles arabiensis	30.015	-0.85	Unclassified	GBIF: https://doi.org/10.15468/dl.zfaw79
Anopheles arabiensis	30.21667	19.91667	Unclassified	GBIF: https://doi.org/10.15468/dl.zfaw79
Anopheles arabiensis	30.23333	19.83333	Unclassified	GBIF: https://doi.org/10.15468/dl.zfaw79
Anopheles arabiensis	30.23333	0.9	Unclassified	GBIF: https://doi.org/10.15468/dl.zfaw79
Anopheles arabiensis	30.25	19.68333	Unclassified	GBIF: https://doi.org/10.15468/dl.zfaw79
Anopheles arabiensis	30.26667	0.95	Unclassified	GBIF: https://doi.org/10.15468/dl.zfaw79
Anopheles arabiensis	30.3	19.48333	Unclassified	GBIF: https://doi.org/10.15468/dl.zfaw79
Anopheles arabiensis	30.33333	-23.83333	Unclassified	GBIF: https://doi.org/10.15468/dl.zfaw79
Anopheles arabiensis	30.33333	-15.66667	Unclassified	GBIF: https://doi.org/10.15468/dl.zfaw79
Anopheles arabiensis	30.443	19.2362	Unclassified	GBIF: https://doi.org/10.15468/dl.zfaw79
Anopheles arabiensis	30.4455	19.2881	Unclassified	GBIF: https://doi.org/10.15468/dl.zfaw79
Anopheles arabiensis	30.4455	19.288	Unclassified	GBIF: https://doi.org/10.15468/dl.zfaw79
Anopheles arabiensis	30.4473	19.2273	Unclassified	GBIF: https://doi.org/10.15468/dl.zfaw79
Anopheles arabiensis	30.4487	19.2056	Unclassified	GBIF: https://doi.org/10.15468/dl.zfaw79
Anopheles arabiensis	30.4491	19.2405	Unclassified	GBIF: https://doi.org/10.15468/dl.zfaw79
Anopheles arabiensis	30.4511	19.291	Unclassified	GBIF: https://doi.org/10.15468/dl.zfaw79
Anopheles arabiensis	30.4519	19.2857	Unclassified	GBIF: https://doi.org/10.15468/dl.zfaw79
Anopheles arabiensis	30.4521	19.2752	Unclassified	GBIF: https://doi.org/10.15468/dl.zfaw79
Anopheles arabiensis	30.4522	19.2739	Unclassified	GBIF: https://doi.org/10.15468/dl.zfaw79
Anopheles arabiensis	30.4522	19.2778	Unclassified	GBIF: https://doi.org/10.15468/dl.zfaw79
Anopheles arabiensis	30.4523	19.2737	Unclassified	GBIF: https://doi.org/10.15468/dl.zfaw79
Anopheles arabiensis	30.4523	19.2785	Unclassified	GBIF: https://doi.org/10.15468/dl.zfaw79
Anopheles arabiensis	30.4524	19.274	Unclassified	GBIF: https://doi.org/10.15468/dl.zfaw79
Anopheles arabiensis	30.4524	19.2742	Unclassified	GBIF: https://doi.org/10.15468/dl.zfaw79
Anopheles arabiensis	30.4524	19.2786	Unclassified	GBIF: https://doi.org/10.15468/dl.zfaw79
Anopheles arabiensis	30.4525	19.2737	Unclassified	GBIF: https://doi.org/10.15468/dl.zfaw79
Anopheles arabiensis	30.4533	19.2866	Unclassified	GBIF: https://doi.org/10.15468/dl.zfaw79
Anopheles arabiensis	30.4533	19.2155	Unclassified	GBIF: https://doi.org/10.15468/dl.zfaw79
Anopheles arabiensis	30.4534	19.2171	Unclassified	GBIF: https://doi.org/10.15468/dl.zfaw79
Anopheles arabiensis	30.4534	19.217	Unclassified	GBIF: https://doi.org/10.15468/dl.zfaw79
Anopheles arabiensis	30.4541	19.2895	Unclassified	GBIF: https://doi.org/10.15468/dl.zfaw79
Anopheles arabiensis	30.4541	19.2898	Unclassified	GBIF: https://doi.org/10.15468/dl.zfaw79
Anopheles arabiensis	30.4541	19.2897	Unclassified	GBIF: https://doi.org/10.15468/dl.zfaw79
Anopheles arabiensis	30.4541	19.2896	Unclassified	GBIF: https://doi.org/10.15468/dl.zfaw79
Anopheles arabiensis	30.4542	19.2896	Unclassified	GBIF: https://doi.org/10.15468/dl.zfaw79

Anopheles arabiensis	30.4542	19.29	Unclassified	GBIF: https://doi.org/10.15468/dl.zfaw79
Anopheles arabiensis	30.4542	19.2902	Unclassified	GBIF: https://doi.org/10.15468/dl.zfaw79
Anopheles arabiensis	30.4542	19.292	Unclassified	GBIF: https://doi.org/10.15468/dl.zfaw79
Anopheles arabiensis	30.4543	19.2923	Unclassified	GBIF: https://doi.org/10.15468/dl.zfaw79
Anopheles arabiensis	30.4543	19.2897	Unclassified	GBIF: https://doi.org/10.15468/dl.zfaw79
Anopheles arabiensis	30.4543	19.2899	Unclassified	GBIF: https://doi.org/10.15468/dl.zfaw79
Anopheles arabiensis	30.4543	19.29	Unclassified	GBIF: https://doi.org/10.15468/dl.zfaw79
Anopheles arabiensis	30.4558	19.2214	Unclassified	GBIF: https://doi.org/10.15468/dl.zfaw79
Anopheles arabiensis	30.4565	19.2675	Unclassified	GBIF: https://doi.org/10.15468/dl.zfaw79
Anopheles arabiensis	30.4584	19.2409	Unclassified	GBIF: https://doi.org/10.15468/dl.zfaw79
Anopheles arabiensis	30.4585	19.2409	Unclassified	GBIF: https://doi.org/10.15468/dl.zfaw79
Anopheles arabiensis	30.4585	19.2405	Unclassified	GBIF: https://doi.org/10.15468/dl.zfaw79
Anopheles arabiensis	30.4592	19.241	Unclassified	GBIF: https://doi.org/10.15468/dl.zfaw79
Anopheles arabiensis	30.4593	19.241	Unclassified	GBIF: https://doi.org/10.15468/dl.zfaw79
Anopheles arabiensis	30.4593	19.2326	Unclassified	GBIF: https://doi.org/10.15468/dl.zfaw79
Anopheles arabiensis	30.4594	19.241	Unclassified	GBIF: https://doi.org/10.15468/dl.zfaw79
Anopheles arabiensis	30.4596	19.233	Unclassified	GBIF: https://doi.org/10.15468/dl.zfaw79
Anopheles arabiensis	30.4596	19.2322	Unclassified	GBIF: https://doi.org/10.15468/dl.zfaw79
Anopheles arabiensis	30.4597	19.232	Unclassified	GBIF: https://doi.org/10.15468/dl.zfaw79
Anopheles arabiensis	30.4597	19.2325	Unclassified	GBIF: https://doi.org/10.15468/dl.zfaw79
Anopheles arabiensis	30.4597	19.2314	Unclassified	GBIF: https://doi.org/10.15468/dl.zfaw79
Anopheles arabiensis	30.4598	19.2577	Unclassified	GBIF: https://doi.org/10.15468/dl.zfaw79
Anopheles arabiensis	30.4598	19.2576	Unclassified	GBIF: https://doi.org/10.15468/dl.zfaw79
Anopheles arabiensis	30.4598	19.2325	Unclassified	GBIF: https://doi.org/10.15468/dl.zfaw79
Anopheles arabiensis	30.4598	19.2321	Unclassified	GBIF: https://doi.org/10.15468/dl.zfaw79
Anopheles arabiensis	30.4599	19.2573	Unclassified	GBIF: https://doi.org/10.15468/dl.zfaw79
Anopheles arabiensis	30.4599	19.2574	Unclassified	GBIF: https://doi.org/10.15468/dl.zfaw79
Anopheles arabiensis	30.4599	19.2576	Unclassified	GBIF: https://doi.org/10.15468/dl.zfaw79
Anopheles arabiensis	30.4599	19.2575	Unclassified	GBIF: https://doi.org/10.15468/dl.zfaw79
Anopheles arabiensis	30.4599	19.2323	Unclassified	GBIF: https://doi.org/10.15468/dl.zfaw79
Anopheles arabiensis	30.4599	19.2317	Unclassified	GBIF: https://doi.org/10.15468/dl.zfaw79
Anopheles arabiensis	30.46	19.2576	Unclassified	GBIF: https://doi.org/10.15468/dl.zfaw79
Anopheles arabiensis	30.46	19.2573	Unclassified	GBIF: https://doi.org/10.15468/dl.zfaw79
Anopheles arabiensis	30.46	19.2312	Unclassified	GBIF: https://doi.org/10.15468/dl.zfaw79
Anopheles arabiensis	30.46	19.2572	Unclassified	GBIF: https://doi.org/10.15468/dl.zfaw79
Anopheles arabiensis	30.46	19.2575	Unclassified	GBIF: https://doi.org/10.15468/dl.zfaw79
Anopheles arabiensis	30.46	19.2571	Unclassified	GBIF: https://doi.org/10.15468/dl.zfaw79
Anopheles arabiensis	30.46	19.2574	Unclassified	GBIF: https://doi.org/10.15468/dl.zfaw79
Anopheles arabiensis	30.4602	19.2571	Unclassified	GBIF: https://doi.org/10.15468/dl.zfaw79
Anopheles arabiensis	30.4602	19.2315	Unclassified	GBIF: https://doi.org/10.15468/dl.zfaw79
Anopheles arabiensis	30.4605	19.2297	Unclassified	GBIF: https://doi.org/10.15468/dl.zfaw79
Anopheles arabiensis	30.4605	19.2562	Unclassified	GBIF: https://doi.org/10.15468/dl.zfaw79

Anopheles arabiensis	30.4605	19.2315	Unclassified	GBIF: https://doi.org/10.15468/dl.zfaw79
Anopheles arabiensis	30.4606	19.2558	Unclassified	GBIF: https://doi.org/10.15468/dl.zfaw79
Anopheles arabiensis	30.4606	19.2312	Unclassified	GBIF: https://doi.org/10.15468/dl.zfaw79
Anopheles arabiensis	30.4606	19.256	Unclassified	GBIF: https://doi.org/10.15468/dl.zfaw79
Anopheles arabiensis	30.4606	19.2563	Unclassified	GBIF: https://doi.org/10.15468/dl.zfaw79
Anopheles arabiensis	30.4608	19.2491	Unclassified	GBIF: https://doi.org/10.15468/dl.zfaw79
Anopheles arabiensis	30.4608	19.249	Unclassified	GBIF: https://doi.org/10.15468/dl.zfaw79
Anopheles arabiensis	30.4609	19.2489	Unclassified	GBIF: https://doi.org/10.15468/dl.zfaw79
Anopheles arabiensis	30.4609	19.2455	Unclassified	GBIF: https://doi.org/10.15468/dl.zfaw79
Anopheles arabiensis	30.461	19.2487	Unclassified	GBIF: https://doi.org/10.15468/dl.zfaw79
Anopheles arabiensis	30.461	19.245	Unclassified	GBIF: https://doi.org/10.15468/dl.zfaw79
Anopheles arabiensis	30.461	19.2454	Unclassified	GBIF: https://doi.org/10.15468/dl.zfaw79
Anopheles arabiensis	30.461	19.2466	Unclassified	GBIF: https://doi.org/10.15468/dl.zfaw79
Anopheles arabiensis	30.4611	19.2408	Unclassified	GBIF: https://doi.org/10.15468/dl.zfaw79
Anopheles arabiensis	30.4611	19.2488	Unclassified	GBIF: https://doi.org/10.15468/dl.zfaw79
Anopheles arabiensis	30.4612	19.249	Unclassified	GBIF: https://doi.org/10.15468/dl.zfaw79
Anopheles arabiensis	30.4612	19.2486	Unclassified	GBIF: https://doi.org/10.15468/dl.zfaw79
Anopheles arabiensis	30.4612	19.2487	Unclassified	GBIF: https://doi.org/10.15468/dl.zfaw79
Anopheles arabiensis	30.4612	19.2488	Unclassified	GBIF: https://doi.org/10.15468/dl.zfaw79
Anopheles arabiensis	30.4615	19.228	Unclassified	GBIF: https://doi.org/10.15468/dl.zfaw79
Anopheles arabiensis	30.4616	19.2281	Unclassified	GBIF: https://doi.org/10.15468/dl.zfaw79
Anopheles arabiensis	30.4616	19.228	Unclassified	GBIF: https://doi.org/10.15468/dl.zfaw79
Anopheles arabiensis	30.4617	19.2489	Unclassified	GBIF: https://doi.org/10.15468/dl.zfaw79
Anopheles arabiensis	30.4618	19.2279	Unclassified	GBIF: https://doi.org/10.15468/dl.zfaw79
Anopheles arabiensis	30.4619	19.2282	Unclassified	GBIF: https://doi.org/10.15468/dl.zfaw79
Anopheles arabiensis	30.4644	19.2407	Unclassified	GBIF: https://doi.org/10.15468/dl.zfaw79
Anopheles arabiensis	30.4647	19.219	Unclassified	GBIF: https://doi.org/10.15468/dl.zfaw79
Anopheles arabiensis	30.4647	19.2077	Unclassified	GBIF: https://doi.org/10.15468/dl.zfaw79
Anopheles arabiensis	30.4649	19.2408	Unclassified	GBIF: https://doi.org/10.15468/dl.zfaw79
Anopheles arabiensis	30.465	19.2407	Unclassified	GBIF: https://doi.org/10.15468/dl.zfaw79
Anopheles arabiensis	30.4651	19.2407	Unclassified	GBIF: https://doi.org/10.15468/dl.zfaw79
Anopheles arabiensis	30.4652	19.2405	Unclassified	GBIF: https://doi.org/10.15468/dl.zfaw79
Anopheles arabiensis	30.4652	19.2402	Unclassified	GBIF: https://doi.org/10.15468/dl.zfaw79
Anopheles arabiensis	30.4653	19.2404	Unclassified	GBIF: https://doi.org/10.15468/dl.zfaw79
Anopheles arabiensis	30.4653	19.24	Unclassified	GBIF: https://doi.org/10.15468/dl.zfaw79
Anopheles arabiensis	30.4653	19.2405	Unclassified	GBIF: https://doi.org/10.15468/dl.zfaw79
Anopheles arabiensis	30.4653	19.2398	Unclassified	GBIF: https://doi.org/10.15468/dl.zfaw79
Anopheles arabiensis	30.4657	19.2242	Unclassified	GBIF: https://doi.org/10.15468/dl.zfaw79
Anopheles arabiensis	30.466	19.2197	Unclassified	GBIF: https://doi.org/10.15468/dl.zfaw79
Anopheles arabiensis	30.46667	-16.08333	Unclassified	GBIF: https://doi.org/10.15468/dl.zfaw79
Anopheles arabiensis	30.4668	19.1896	Unclassified	GBIF: https://doi.org/10.15468/dl.zfaw79
Anopheles arabiensis	30.4712	19.1989	Unclassified	GBIF: https://doi.org/10.15468/dl.zfaw79

Anopheles arabiensis	30.4721	19.2166	Unclassified	GBIF: https://doi.org/10.15468/dl.zfaw79
Anopheles arabiensis	30.4788	19.1881	Unclassified	GBIF: https://doi.org/10.15468/dl.zfaw79
Anopheles arabiensis	30.479	19.2072	Unclassified	GBIF: https://doi.org/10.15468/dl.zfaw79
Anopheles arabiensis	30.4791	19.1903	Unclassified	GBIF: https://doi.org/10.15468/dl.zfaw79
Anopheles arabiensis	30.48	19.1882	Unclassified	GBIF: https://doi.org/10.15468/dl.zfaw79
Anopheles arabiensis	30.4844	19.1882	Unclassified	GBIF: https://doi.org/10.15468/dl.zfaw79
Anopheles arabiensis	30.4847	19.1879	Unclassified	GBIF: https://doi.org/10.15468/dl.zfaw79
Anopheles arabiensis	30.5	1.05	Unclassified	GBIF: https://doi.org/10.15468/dl.zfaw79
Anopheles arabiensis	30.58333	-22.75	Unclassified	GBIF: https://doi.org/10.15468/dl.zfaw79
Anopheles arabiensis	30.8892	-23.5362	Unclassified	GBIF: https://doi.org/10.15468/dl.zfaw79
Anopheles arabiensis	30.95	-16.33333	Unclassified	GBIF: https://doi.org/10.15468/dl.zfaw79
Anopheles arabiensis	31.01667	-26.75	Unclassified	GBIF: https://doi.org/10.15468/dl.zfaw79
Anopheles arabiensis	31.03333	-22.88333	Unclassified	GBIF: https://doi.org/10.15468/dl.zfaw79
Anopheles arabiensis	31.0396	-22.8896	Unclassified	GBIF: https://doi.org/10.15468/dl.zfaw79
Anopheles arabiensis	31.2	-20.68333	Unclassified	GBIF: https://doi.org/10.15468/dl.zfaw79
Anopheles arabiensis	31.21667	-20.71667	Unclassified	GBIF: https://doi.org/10.15468/dl.zfaw79
Anopheles arabiensis	31.25	-23.01667	Unclassified	GBIF: https://doi.org/10.15468/dl.zfaw79
Anopheles arabiensis	31.36667	-23.08333	Unclassified	GBIF: https://doi.org/10.15468/dl.zfaw79
Anopheles arabiensis	31.43333	-23.4	Unclassified	GBIF: https://doi.org/10.15468/dl.zfaw79
Anopheles arabiensis	31.43333	-23.11667	Unclassified	GBIF: https://doi.org/10.15468/dl.zfaw79
Anopheles arabiensis	31.56667	-0.63333	Unclassified	GBIF: https://doi.org/10.15468/dl.zfaw79
Anopheles arabiensis	31.58333	-24.98333	Unclassified	GBIF: https://doi.org/10.15468/dl.zfaw79
Anopheles arabiensis	31.61667	-20.9	Unclassified	GBIF: https://doi.org/10.15468/dl.zfaw79
Anopheles arabiensis	31.61667	-21.05	Unclassified	GBIF: https://doi.org/10.15468/dl.zfaw79
Anopheles arabiensis	31.61667	-21.21667	Unclassified	GBIF: https://doi.org/10.15468/dl.zfaw79
Anopheles arabiensis	31.63333	-21.11667	Unclassified	GBIF: https://doi.org/10.15468/dl.zfaw79
Anopheles arabiensis	31.63333	-25.91667	Unclassified	GBIF: https://doi.org/10.15468/dl.zfaw79
Anopheles arabiensis	31.66667	-21.41667	Unclassified	GBIF: https://doi.org/10.15468/dl.zfaw79
Anopheles arabiensis	31.68333	-26.68333	Unclassified	GBIF: https://doi.org/10.15468/dl.zfaw79
Anopheles arabiensis	31.75	-17.21667	Unclassified	GBIF: https://doi.org/10.15468/dl.zfaw79
Anopheles arabiensis	31.75	-21.03333	Unclassified	GBIF: https://doi.org/10.15468/dl.zfaw79
Anopheles arabiensis	31.7827	-24.6314	Unclassified	GBIF: https://doi.org/10.15468/dl.zfaw79
Anopheles arabiensis	31.8055	18.4931	Unclassified	GBIF: https://doi.org/10.15468/dl.zfaw79
Anopheles arabiensis	31.81	18.48	Unclassified	GBIF: https://doi.org/10.15468/dl.zfaw79
Anopheles arabiensis	31.8104	18.4794	Unclassified	GBIF: https://doi.org/10.15468/dl.zfaw79
Anopheles arabiensis	31.8107	18.4813	Unclassified	GBIF: https://doi.org/10.15468/dl.zfaw79
Anopheles arabiensis	31.8138	18.5026	Unclassified	GBIF: https://doi.org/10.15468/dl.zfaw79
Anopheles arabiensis	31.8166	18.4871	Unclassified	GBIF: https://doi.org/10.15468/dl.zfaw79
Anopheles arabiensis	31.81667	-21	Unclassified	GBIF: https://doi.org/10.15468/dl.zfaw79
Anopheles arabiensis	31.8181	18.4767	Unclassified	GBIF: https://doi.org/10.15468/dl.zfaw79
Anopheles arabiensis	31.8181	18.4766	Unclassified	GBIF: https://doi.org/10.15468/dl.zfaw79
Anopheles arabiensis	31.8201	18.485	Unclassified	GBIF: https://doi.org/10.15468/dl.zfaw79

Anopheles arabiensis	31.8221	18.5051	Unclassified	GBIF: https://doi.org/10.15468/dl.zfaw79
Anopheles arabiensis	31.8259	18.4929	Unclassified	GBIF: https://doi.org/10.15468/dl.zfaw79
Anopheles arabiensis	31.8274	18.4886	Unclassified	GBIF: https://doi.org/10.15468/dl.zfaw79
Anopheles arabiensis	31.8323	18.524	Unclassified	GBIF: https://doi.org/10.15468/dl.zfaw79
Anopheles arabiensis	31.8328	18.5205	Unclassified	GBIF: https://doi.org/10.15468/dl.zfaw79
Anopheles arabiensis	31.8331	18.5247	Unclassified	GBIF: https://doi.org/10.15468/dl.zfaw79
Anopheles arabiensis	31.83333	-25.93333	Unclassified	GBIF: https://doi.org/10.15468/dl.zfaw79
Anopheles arabiensis	31.83333	-26.83333	Unclassified	GBIF: https://doi.org/10.15468/dl.zfaw79
Anopheles arabiensis	31.8347	18.5415	Unclassified	GBIF: https://doi.org/10.15468/dl.zfaw79
Anopheles arabiensis	31.8348	18.5042	Unclassified	GBIF: https://doi.org/10.15468/dl.zfaw79
Anopheles arabiensis	31.8349	18.5042	Unclassified	GBIF: https://doi.org/10.15468/dl.zfaw79
Anopheles arabiensis	31.835	18.5042	Unclassified	GBIF: https://doi.org/10.15468/dl.zfaw79
Anopheles arabiensis	31.8352	18.504	Unclassified	GBIF: https://doi.org/10.15468/dl.zfaw79
Anopheles arabiensis	31.8379	18.5279	Unclassified	GBIF: https://doi.org/10.15468/dl.zfaw79
Anopheles arabiensis	31.8379	18.5282	Unclassified	GBIF: https://doi.org/10.15468/dl.zfaw79
Anopheles arabiensis	31.838	18.5253	Unclassified	GBIF: https://doi.org/10.15468/dl.zfaw79
Anopheles arabiensis	31.8381	18.5249	Unclassified	GBIF: https://doi.org/10.15468/dl.zfaw79
Anopheles arabiensis	31.8383	18.5248	Unclassified	GBIF: https://doi.org/10.15468/dl.zfaw79
Anopheles arabiensis	31.839	18.5253	Unclassified	GBIF: https://doi.org/10.15468/dl.zfaw79
Anopheles arabiensis	31.8447	18.5413	Unclassified	GBIF: https://doi.org/10.15468/dl.zfaw79
Anopheles arabiensis	31.8457	18.5264	Unclassified	GBIF: https://doi.org/10.15468/dl.zfaw79
Anopheles arabiensis	31.8457	18.5263	Unclassified	GBIF: https://doi.org/10.15468/dl.zfaw79
Anopheles arabiensis	31.846	18.5263	Unclassified	GBIF: https://doi.org/10.15468/dl.zfaw79
Anopheles arabiensis	31.8462	18.5262	Unclassified	GBIF: https://doi.org/10.15468/dl.zfaw79
Anopheles arabiensis	31.8462	18.5263	Unclassified	GBIF: https://doi.org/10.15468/dl.zfaw79
Anopheles arabiensis	31.8466	18.5263	Unclassified	GBIF: https://doi.org/10.15468/dl.zfaw79
Anopheles arabiensis	31.848	18.5518	Unclassified	GBIF: https://doi.org/10.15468/dl.zfaw79
Anopheles arabiensis	31.8514	18.5138	Unclassified	GBIF: https://doi.org/10.15468/dl.zfaw79
Anopheles arabiensis	31.8514	18.5139	Unclassified	GBIF: https://doi.org/10.15468/dl.zfaw79
Anopheles arabiensis	31.8524	18.5499	Unclassified	GBIF: https://doi.org/10.15468/dl.zfaw79
Anopheles arabiensis	31.8551	18.5565	Unclassified	GBIF: https://doi.org/10.15468/dl.zfaw79
Anopheles arabiensis	31.8562	18.5589	Unclassified	GBIF: https://doi.org/10.15468/dl.zfaw79
Anopheles arabiensis	31.8569	18.529	Unclassified	GBIF: https://doi.org/10.15468/dl.zfaw79
Anopheles arabiensis	31.8586	18.5654	Unclassified	GBIF: https://doi.org/10.15468/dl.zfaw79
Anopheles arabiensis	31.862	18.5305	Unclassified	GBIF: https://doi.org/10.15468/dl.zfaw79
Anopheles arabiensis	31.8622	18.5309	Unclassified	GBIF: https://doi.org/10.15468/dl.zfaw79
Anopheles arabiensis	31.8629	18.5418	Unclassified	GBIF: https://doi.org/10.15468/dl.zfaw79
Anopheles arabiensis	31.8635	18.5311	Unclassified	GBIF: https://doi.org/10.15468/dl.zfaw79
Anopheles arabiensis	31.8666	18.5392	Unclassified	GBIF: https://doi.org/10.15468/dl.zfaw79
Anopheles arabiensis	31.86667	-26.8	Unclassified	GBIF: https://doi.org/10.15468/dl.zfaw79
Anopheles arabiensis	31.8685	18.5346	Unclassified	GBIF: https://doi.org/10.15468/dl.zfaw79
Anopheles arabiensis	31.8689	18.5463	Unclassified	GBIF: https://doi.org/10.15468/dl.zfaw79

Anopheles arabiensis	31.8689	18.5462	Unclassified	GBIF: https://doi.org/10.15468/dl.zfaw79
Anopheles arabiensis	31.869	18.5462	Unclassified	GBIF: https://doi.org/10.15468/dl.zfaw79
Anopheles arabiensis	31.869	18.5461	Unclassified	GBIF: https://doi.org/10.15468/dl.zfaw79
Anopheles arabiensis	31.8703	18.5411	Unclassified	GBIF: https://doi.org/10.15468/dl.zfaw79
Anopheles arabiensis	31.8706	18.5605	Unclassified	GBIF: https://doi.org/10.15468/dl.zfaw79
Anopheles arabiensis	31.8706	18.5396	Unclassified	GBIF: https://doi.org/10.15468/dl.zfaw79
Anopheles arabiensis	31.8719	18.5627	Unclassified	GBIF: https://doi.org/10.15468/dl.zfaw79
Anopheles arabiensis	31.872	18.5627	Unclassified	GBIF: https://doi.org/10.15468/dl.zfaw79
Anopheles arabiensis	31.8733	18.5643	Unclassified	GBIF: https://doi.org/10.15468/dl.zfaw79
Anopheles arabiensis	31.8735	18.563	Unclassified	GBIF: https://doi.org/10.15468/dl.zfaw79
Anopheles arabiensis	31.8752	18.5504	Unclassified	GBIF: https://doi.org/10.15468/dl.zfaw79
Anopheles arabiensis	31.8753	18.5656	Unclassified	GBIF: https://doi.org/10.15468/dl.zfaw79
Anopheles arabiensis	31.8758	18.5303	Unclassified	GBIF: https://doi.org/10.15468/dl.zfaw79
Anopheles arabiensis	31.882	18.5723	Unclassified	GBIF: https://doi.org/10.15468/dl.zfaw79
Anopheles arabiensis	31.8823	18.5664	Unclassified	GBIF: https://doi.org/10.15468/dl.zfaw79
Anopheles arabiensis	31.88333	-25.68333	Unclassified	GBIF: https://doi.org/10.15468/dl.zfaw79
Anopheles arabiensis	31.8848	18.5801	Unclassified	GBIF: https://doi.org/10.15468/dl.zfaw79
Anopheles arabiensis	31.885	18.5802	Unclassified	GBIF: https://doi.org/10.15468/dl.zfaw79
Anopheles arabiensis	31.8852	18.5702	Unclassified	GBIF: https://doi.org/10.15468/dl.zfaw79
Anopheles arabiensis	31.8861	18.5435	Unclassified	GBIF: https://doi.org/10.15468/dl.zfaw79
Anopheles arabiensis	31.8861	18.5465	Unclassified	GBIF: https://doi.org/10.15468/dl.zfaw79
Anopheles arabiensis	31.8866	18.5593	Unclassified	GBIF: https://doi.org/10.15468/dl.zfaw79
Anopheles arabiensis	31.8866	18.5467	Unclassified	GBIF: https://doi.org/10.15468/dl.zfaw79
Anopheles arabiensis	31.8867	18.5465	Unclassified	GBIF: https://doi.org/10.15468/dl.zfaw79
Anopheles arabiensis	31.8867	18.5462	Unclassified	GBIF: https://doi.org/10.15468/dl.zfaw79
Anopheles arabiensis	31.8868	18.5591	Unclassified	GBIF: https://doi.org/10.15468/dl.zfaw79
Anopheles arabiensis	31.887	18.5466	Unclassified	GBIF: https://doi.org/10.15468/dl.zfaw79
Anopheles arabiensis	31.8872	18.5633	Unclassified	GBIF: https://doi.org/10.15468/dl.zfaw79
Anopheles arabiensis	31.891	18.5733	Unclassified	GBIF: https://doi.org/10.15468/dl.zfaw79
Anopheles arabiensis	31.8919	18.5763	Unclassified	GBIF: https://doi.org/10.15468/dl.zfaw79
Anopheles arabiensis	31.8932	18.5522	Unclassified	GBIF: https://doi.org/10.15468/dl.zfaw79
Anopheles arabiensis	31.8934	18.5592	Unclassified	GBIF: https://doi.org/10.15468/dl.zfaw79
Anopheles arabiensis	31.8935	18.5489	Unclassified	GBIF: https://doi.org/10.15468/dl.zfaw79
Anopheles arabiensis	31.8957	18.5801	Unclassified	GBIF: https://doi.org/10.15468/dl.zfaw79
Anopheles arabiensis	31.8958	18.5801	Unclassified	GBIF: https://doi.org/10.15468/dl.zfaw79
Anopheles arabiensis	31.8975	18.5777	Unclassified	GBIF: https://doi.org/10.15468/dl.zfaw79
Anopheles arabiensis	31.8976	18.5778	Unclassified	GBIF: https://doi.org/10.15468/dl.zfaw79
Anopheles arabiensis	31.8979	18.5776	Unclassified	GBIF: https://doi.org/10.15468/dl.zfaw79
Anopheles arabiensis	31.898	18.5777	Unclassified	GBIF: https://doi.org/10.15468/dl.zfaw79
Anopheles arabiensis	31.8992	18.5867	Unclassified	GBIF: https://doi.org/10.15468/dl.zfaw79
Anopheles arabiensis	31.8993	18.5868	Unclassified	GBIF: https://doi.org/10.15468/dl.zfaw79
Anopheles arabiensis	31.8994	18.5866	Unclassified	GBIF: https://doi.org/10.15468/dl.zfaw79

Anopheles arabiensis	31.9008	18.5642	Unclassified	GBIF: https://doi.org/10.15468/dl.zfaw79
Anopheles arabiensis	31.9031	18.5579	Unclassified	GBIF: https://doi.org/10.15468/dl.zfaw79
Anopheles arabiensis	31.9061	18.5786	Unclassified	GBIF: https://doi.org/10.15468/dl.zfaw79
Anopheles arabiensis	31.9064	18.5786	Unclassified	GBIF: https://doi.org/10.15468/dl.zfaw79
Anopheles arabiensis	31.9068	18.5749	Unclassified	GBIF: https://doi.org/10.15468/dl.zfaw79
Anopheles arabiensis	31.9115	18.5672	Unclassified	GBIF: https://doi.org/10.15468/dl.zfaw79
Anopheles arabiensis	31.9164	18.5933	Unclassified	GBIF: https://doi.org/10.15468/dl.zfaw79
Anopheles arabiensis	31.9165	18.5932	Unclassified	GBIF: https://doi.org/10.15468/dl.zfaw79
Anopheles arabiensis	31.9166	18.5934	Unclassified	GBIF: https://doi.org/10.15468/dl.zfaw79
Anopheles arabiensis	31.9166	18.5933	Unclassified	GBIF: https://doi.org/10.15468/dl.zfaw79
Anopheles arabiensis	31.9166	18.5932	Unclassified	GBIF: https://doi.org/10.15468/dl.zfaw79
Anopheles arabiensis	31.91667	-21.28333	Unclassified	GBIF: https://doi.org/10.15468/dl.zfaw79
Anopheles arabiensis	31.91667	-17.33333	Unclassified	GBIF: https://doi.org/10.15468/dl.zfaw79
Anopheles arabiensis	31.9168	18.593	Unclassified	GBIF: https://doi.org/10.15468/dl.zfaw79
Anopheles arabiensis	31.9234	18.589	Unclassified	GBIF: https://doi.org/10.15468/dl.zfaw79
Anopheles arabiensis	31.9234	18.5889	Unclassified	GBIF: https://doi.org/10.15468/dl.zfaw79
Anopheles arabiensis	31.9235	18.5889	Unclassified	GBIF: https://doi.org/10.15468/dl.zfaw79
Anopheles arabiensis	31.924	18.589	Unclassified	GBIF: https://doi.org/10.15468/dl.zfaw79
Anopheles arabiensis	31.924	18.5891	Unclassified	GBIF: https://doi.org/10.15468/dl.zfaw79
Anopheles arabiensis	31.9267	18.5795	Unclassified	GBIF: https://doi.org/10.15468/dl.zfaw79
Anopheles arabiensis	31.9269	18.5799	Unclassified	GBIF: https://doi.org/10.15468/dl.zfaw79
Anopheles arabiensis	31.9269	18.5801	Unclassified	GBIF: https://doi.org/10.15468/dl.zfaw79
Anopheles arabiensis	31.927	18.5802	Unclassified	GBIF: https://doi.org/10.15468/dl.zfaw79
Anopheles arabiensis	31.927	18.58	Unclassified	GBIF: https://doi.org/10.15468/dl.zfaw79
Anopheles arabiensis	31.9272	18.5803	Unclassified	GBIF: https://doi.org/10.15468/dl.zfaw79
Anopheles arabiensis	31.9272	18.5806	Unclassified	GBIF: https://doi.org/10.15468/dl.zfaw79
Anopheles arabiensis	31.9272	18.5804	Unclassified	GBIF: https://doi.org/10.15468/dl.zfaw79
Anopheles arabiensis	31.9275	18.5898	Unclassified	GBIF: https://doi.org/10.15468/dl.zfaw79
Anopheles arabiensis	31.9279	18.5798	Unclassified	GBIF: https://doi.org/10.15468/dl.zfaw79
Anopheles arabiensis	31.9279	18.5799	Unclassified	GBIF: https://doi.org/10.15468/dl.zfaw79
Anopheles arabiensis	31.9284	18.5899	Unclassified	GBIF: https://doi.org/10.15468/dl.zfaw79
Anopheles arabiensis	31.9284	18.59	Unclassified	GBIF: https://doi.org/10.15468/dl.zfaw79
Anopheles arabiensis	31.9286	18.59	Unclassified	GBIF: https://doi.org/10.15468/dl.zfaw79
Anopheles arabiensis	31.931	18.5906	Unclassified	GBIF: https://doi.org/10.15468/dl.zfaw79
Anopheles arabiensis	31.93333	-26.66667	Unclassified	GBIF: https://doi.org/10.15468/dl.zfaw79
Anopheles arabiensis	31.93333	-25.43333	Unclassified	GBIF: https://doi.org/10.15468/dl.zfaw79
Anopheles arabiensis	31.93333	-26.83333	Unclassified	GBIF: https://doi.org/10.15468/dl.zfaw79
Anopheles arabiensis	31.95	-27.01667	Unclassified	GBIF: https://doi.org/10.15468/dl.zfaw79
Anopheles arabiensis	31.96667	-28.03333	Unclassified	GBIF: https://doi.org/10.15468/dl.zfaw79
Anopheles arabiensis	31.96667	-28.56667	Unclassified	GBIF: https://doi.org/10.15468/dl.zfaw79
Anopheles arabiensis	32	-27	Unclassified	GBIF: https://doi.org/10.15468/dl.zfaw79
Anopheles arabiensis	32.01667	-26.75	Unclassified	GBIF: https://doi.org/10.15468/dl.zfaw79

Anopheles arabiensis	32.05	-25.71667	Unclassified	GBIF: https://doi.org/10.15468/dl.zfaw79
Anopheles arabiensis	32.15	-21.03333	Unclassified	GBIF: https://doi.org/10.15468/dl.zfaw79
Anopheles arabiensis	32.16667	-21	Unclassified	GBIF: https://doi.org/10.15468/dl.zfaw79
Anopheles arabiensis	32.18333	-20.91667	Unclassified	GBIF: https://doi.org/10.15468/dl.zfaw79
Anopheles arabiensis	32.23333	-21.08333	Unclassified	GBIF: https://doi.org/10.15468/dl.zfaw79
Anopheles arabiensis	32.23333	-21.1	Unclassified	GBIF: https://doi.org/10.15468/dl.zfaw79
Anopheles arabiensis	32.25	-21.08333	Unclassified	GBIF: https://doi.org/10.15468/dl.zfaw79
Anopheles arabiensis	32.25	-27.03333	Unclassified	GBIF: https://doi.org/10.15468/dl.zfaw79
Anopheles arabiensis	32.26667	-27.38333	Unclassified	GBIF: https://doi.org/10.15468/dl.zfaw79
Anopheles arabiensis	32.26667	-27.13333	Unclassified	GBIF: https://doi.org/10.15468/dl.zfaw79
Anopheles arabiensis	32.31667	-27.03333	Unclassified	GBIF: https://doi.org/10.15468/dl.zfaw79
Anopheles arabiensis	32.31667	-26.9	Unclassified	GBIF: https://doi.org/10.15468/dl.zfaw79
Anopheles arabiensis	32.33333	-26.3	Unclassified	GBIF: https://doi.org/10.15468/dl.zfaw79
Anopheles arabiensis	32.33333	-26.53333	Unclassified	GBIF: https://doi.org/10.15468/dl.zfaw79
Anopheles arabiensis	32.5	-25.86667	Unclassified	GBIF: https://doi.org/10.15468/dl.zfaw79
Anopheles arabiensis	32.5	-27	Unclassified	GBIF: https://doi.org/10.15468/dl.zfaw79
Anopheles arabiensis	32.5	0.16667	Unclassified	GBIF: https://doi.org/10.15468/dl.zfaw79
Anopheles arabiensis	32.55	-25.5	Unclassified	GBIF: https://doi.org/10.15468/dl.zfaw79
Anopheles arabiensis	32.55	-27.05	Unclassified	GBIF: https://doi.org/10.15468/dl.zfaw79
Anopheles arabiensis	32.55	-27.08333	Unclassified	GBIF: https://doi.org/10.15468/dl.zfaw79
Anopheles arabiensis	32.56667	-26.83333	Unclassified	GBIF: https://doi.org/10.15468/dl.zfaw79
Anopheles arabiensis	32.58333	-25.96667	Unclassified	GBIF: https://doi.org/10.15468/dl.zfaw79
Anopheles arabiensis	32.66667	-25.9	Unclassified	GBIF: https://doi.org/10.15468/dl.zfaw79
Anopheles arabiensis	32.7833	-19.1833	Unclassified	GBIF: https://doi.org/10.15468/dl.zfaw79
Anopheles arabiensis	32.86667	-25.25	Unclassified	GBIF: https://doi.org/10.15468/dl.zfaw79
Anopheles arabiensis	33	-25.05	Unclassified	GBIF: https://doi.org/10.15468/dl.zfaw79
Anopheles arabiensis	33	-2.83333	Unclassified	GBIF: https://doi.org/10.15468/dl.zfaw79
Anopheles arabiensis	33.214	0.448213	Unclassified	GBIF: https://doi.org/10.15468/dl.zfaw79
Anopheles arabiensis	33.25	14.91667	Unclassified	GBIF: https://doi.org/10.15468/dl.zfaw79
Anopheles arabiensis	33.36667	16.7	Unclassified	GBIF: https://doi.org/10.15468/dl.zfaw79
Anopheles arabiensis	33.5	-16.16667	Unclassified	GBIF: https://doi.org/10.15468/dl.zfaw79
Anopheles arabiensis	33.5	14.5	Unclassified	GBIF: https://doi.org/10.15468/dl.zfaw79
Anopheles arabiensis	33.83333	13.5	Unclassified	GBIF: https://doi.org/10.15468/dl.zfaw79
Anopheles arabiensis	34	-0.06667	Unclassified	GBIF: https://doi.org/10.15468/dl.zfaw79
Anopheles arabiensis	34	0	Unclassified	GBIF: https://doi.org/10.15468/dl.zfaw79
Anopheles arabiensis	34.01	-0.03	Unclassified	GBIF: https://doi.org/10.15468/dl.zfaw79
Anopheles arabiensis	34.0261	0.77	Unclassified	GBIF: https://doi.org/10.15468/dl.zfaw79
Anopheles arabiensis	34.05	0.1	Unclassified	GBIF: https://doi.org/10.15468/dl.zfaw79
Anopheles arabiensis	34.11	0.01	Unclassified	GBIF: https://doi.org/10.15468/dl.zfaw79
Anopheles arabiensis	34.14	-0.16	Unclassified	GBIF: https://doi.org/10.15468/dl.zfaw79
Anopheles arabiensis	34.15	0.51	Unclassified	GBIF: https://doi.org/10.15468/dl.zfaw79
Anopheles arabiensis	34.1847	0.681882	Unclassified	GBIF: https://doi.org/10.15468/dl.zfaw79

Anopheles arabiensis	34.23	0.58	Unclassified	GBIF: https://doi.org/10.15468/dl.zfaw79
Anopheles arabiensis	34.25	-0.66667	Unclassified	GBIF: https://doi.org/10.15468/dl.zfaw79
Anopheles arabiensis	34.38333	-0.16667	Unclassified	GBIF: https://doi.org/10.15468/dl.zfaw79
Anopheles arabiensis	34.4	15.83333	Unclassified	GBIF: https://doi.org/10.15468/dl.zfaw79
Anopheles arabiensis	34.42	0.62	Unclassified	GBIF: https://doi.org/10.15468/dl.zfaw79
Anopheles arabiensis	34.5	-0.5	Unclassified	GBIF: https://doi.org/10.15468/dl.zfaw79
Anopheles arabiensis	34.58333	8.25	Unclassified	GBIF: https://doi.org/10.15468/dl.zfaw79
Anopheles arabiensis	34.66667	11.66667	Unclassified	GBIF: https://doi.org/10.15468/dl.zfaw79
Anopheles arabiensis	34.68333	-0.06667	Unclassified	GBIF: https://doi.org/10.15468/dl.zfaw79
Anopheles arabiensis	34.75	2.2	Unclassified	GBIF: https://doi.org/10.15468/dl.zfaw79
Anopheles arabiensis	34.75	-12.75	Unclassified	GBIF: https://doi.org/10.15468/dl.zfaw79
Anopheles arabiensis	34.8717	-13.4217	Unclassified	GBIF: https://doi.org/10.15468/dl.zfaw79
Anopheles arabiensis	34.93	-0.16	Unclassified	GBIF: https://doi.org/10.15468/dl.zfaw79
Anopheles arabiensis	34.94	-0.13	Unclassified	GBIF: https://doi.org/10.15468/dl.zfaw79
Anopheles arabiensis	34.98333	-13.46667	Unclassified	GBIF: https://doi.org/10.15468/dl.zfaw79
Anopheles arabiensis	35	8	Unclassified	GBIF: https://doi.org/10.15468/dl.zfaw79
Anopheles arabiensis	35	-21	Unclassified	GBIF: https://doi.org/10.15468/dl.zfaw79
Anopheles arabiensis	35.13333	14.2	Unclassified	GBIF: https://doi.org/10.15468/dl.zfaw79
Anopheles arabiensis	35.13333	-13	Unclassified	GBIF: https://doi.org/10.15468/dl.zfaw79
Anopheles arabiensis	35.15	-3.33333	Unclassified	GBIF: https://doi.org/10.15468/dl.zfaw79
Anopheles arabiensis	35.46667	-18.28333	Unclassified	GBIF: https://doi.org/10.15468/dl.zfaw79
Anopheles arabiensis	35.63333	3.1	Unclassified	GBIF: https://doi.org/10.15468/dl.zfaw79
Anopheles arabiensis	35.7	-1.33333	Unclassified	GBIF: https://doi.org/10.15468/dl.zfaw79
Anopheles arabiensis	35.73333	-4.13333	Unclassified	GBIF: https://doi.org/10.15468/dl.zfaw79
Anopheles arabiensis	35.75	-3.91667	Unclassified	GBIF: https://doi.org/10.15468/dl.zfaw79
Anopheles arabiensis	35.83333	-3.5	Unclassified	GBIF: https://doi.org/10.15468/dl.zfaw79
Anopheles arabiensis	35.93333	-11.16667	Unclassified	GBIF: https://doi.org/10.15468/dl.zfaw79
Anopheles arabiensis	35.98333	14.98333	Unclassified	GBIF: https://doi.org/10.15468/dl.zfaw79
Anopheles arabiensis	36	0.5	Unclassified	GBIF: https://doi.org/10.15468/dl.zfaw79
Anopheles arabiensis	36	4	Unclassified	GBIF: https://doi.org/10.15468/dl.zfaw79
Anopheles arabiensis	36.28333	0.63333	Unclassified	GBIF: https://doi.org/10.15468/dl.zfaw79
Anopheles arabiensis	36.31667	8.48333	Unclassified	GBIF: https://doi.org/10.15468/dl.zfaw79
Anopheles arabiensis	36.33333	8	Unclassified	GBIF: https://doi.org/10.15468/dl.zfaw79
Anopheles arabiensis	36.3363	-8.1536	Unclassified	GBIF: https://doi.org/10.15468/dl.zfaw79
Anopheles arabiensis	36.36	-8.16668	Unclassified	GBIF: https://doi.org/10.15468/dl.zfaw79
Anopheles arabiensis	36.3623	-8.17202	Unclassified	GBIF: https://doi.org/10.15468/dl.zfaw79
Anopheles arabiensis	36.3627	-8.15718	Unclassified	GBIF: https://doi.org/10.15468/dl.zfaw79
Anopheles arabiensis	36.3644	-8.15527	Unclassified	GBIF: https://doi.org/10.15468/dl.zfaw79
Anopheles arabiensis	36.365	-8.16375	Unclassified	GBIF: https://doi.org/10.15468/dl.zfaw79
Anopheles arabiensis	36.36667	14.36667	Unclassified	GBIF: https://doi.org/10.15468/dl.zfaw79
Anopheles arabiensis	36.3681	-8.1713	Unclassified	GBIF: https://doi.org/10.15468/dl.zfaw79
Anopheles arabiensis	36.3694	-8.15503	Unclassified	GBIF: https://doi.org/10.15468/dl.zfaw79

Anopheles arabiensis	36.3715	-8.16998	Unclassified	GBIF: https://doi.org/10.15468/dl.zfaw79
Anopheles arabiensis	36.3807	-8.18135	Unclassified	GBIF: https://doi.org/10.15468/dl.zfaw79
Anopheles arabiensis	36.384	-8.1683	Unclassified	GBIF: https://doi.org/10.15468/dl.zfaw79
Anopheles arabiensis	36.386	-8.18618	Unclassified	GBIF: https://doi.org/10.15468/dl.zfaw79
Anopheles arabiensis	36.387	-8.18568	Unclassified	GBIF: https://doi.org/10.15468/dl.zfaw79
Anopheles arabiensis	36.3871	-8.17	Unclassified	GBIF: https://doi.org/10.15468/dl.zfaw79
Anopheles arabiensis	36.3872	-8.17	Unclassified	GBIF: https://doi.org/10.15468/dl.zfaw79
Anopheles arabiensis	36.3872	-8.1701	Unclassified	GBIF: https://doi.org/10.15468/dl.zfaw79
Anopheles arabiensis	36.3876	-8.18327	Unclassified	GBIF: https://doi.org/10.15468/dl.zfaw79
Anopheles arabiensis	36.3883	-8.17953	Unclassified	GBIF: https://doi.org/10.15468/dl.zfaw79
Anopheles arabiensis	36.3885	-8.1748	Unclassified	GBIF: https://doi.org/10.15468/dl.zfaw79
Anopheles arabiensis	36.3885	-8.1747	Unclassified	GBIF: https://doi.org/10.15468/dl.zfaw79
Anopheles arabiensis	36.3886	-8.1747	Unclassified	GBIF: https://doi.org/10.15468/dl.zfaw79
Anopheles arabiensis	36.3886	-8.1748	Unclassified	GBIF: https://doi.org/10.15468/dl.zfaw79
Anopheles arabiensis	36.3893	-8.17788	Unclassified	GBIF: https://doi.org/10.15468/dl.zfaw79
Anopheles arabiensis	36.3898	-8.1835	Unclassified	GBIF: https://doi.org/10.15468/dl.zfaw79
Anopheles arabiensis	36.3903	-8.1797	Unclassified	GBIF: https://doi.org/10.15468/dl.zfaw79
Anopheles arabiensis	36.3907	-8.15435	Unclassified	GBIF: https://doi.org/10.15468/dl.zfaw79
Anopheles arabiensis	36.3908	-8.17755	Unclassified	GBIF: https://doi.org/10.15468/dl.zfaw79
Anopheles arabiensis	36.3916	-8.22153	Unclassified	GBIF: https://doi.org/10.15468/dl.zfaw79
Anopheles arabiensis	36.3916	-8.1606	Unclassified	GBIF: https://doi.org/10.15468/dl.zfaw79
Anopheles arabiensis	36.3917	-8.15608	Unclassified	GBIF: https://doi.org/10.15468/dl.zfaw79
Anopheles arabiensis	36.3919	-8.17112	Unclassified	GBIF: https://doi.org/10.15468/dl.zfaw79
Anopheles arabiensis	36.3919	-8.17193	Unclassified	GBIF: https://doi.org/10.15468/dl.zfaw79
Anopheles arabiensis	36.3927	-8.17417	Unclassified	GBIF: https://doi.org/10.15468/dl.zfaw79
Anopheles arabiensis	36.3932	-8.1754	Unclassified	GBIF: https://doi.org/10.15468/dl.zfaw79
Anopheles arabiensis	36.3932	-8.1756	Unclassified	GBIF: https://doi.org/10.15468/dl.zfaw79
Anopheles arabiensis	36.3933	-8.1754	Unclassified	GBIF: https://doi.org/10.15468/dl.zfaw79
Anopheles arabiensis	36.3934	-8.1755	Unclassified	GBIF: https://doi.org/10.15468/dl.zfaw79
Anopheles arabiensis	36.3934	-8.1756	Unclassified	GBIF: https://doi.org/10.15468/dl.zfaw79
Anopheles arabiensis	36.3935	-8.1754	Unclassified	GBIF: https://doi.org/10.15468/dl.zfaw79
Anopheles arabiensis	36.3938	-8.15408	Unclassified	GBIF: https://doi.org/10.15468/dl.zfaw79
Anopheles arabiensis	36.3942	-8.15687	Unclassified	GBIF: https://doi.org/10.15468/dl.zfaw79
Anopheles arabiensis	36.3943	-8.15422	Unclassified	GBIF: https://doi.org/10.15468/dl.zfaw79
Anopheles arabiensis	36.3943	-8.15415	Unclassified	GBIF: https://doi.org/10.15468/dl.zfaw79
Anopheles arabiensis	36.3947	-8.1657	Unclassified	GBIF: https://doi.org/10.15468/dl.zfaw79
Anopheles arabiensis	36.3947	-8.1658	Unclassified	GBIF: https://doi.org/10.15468/dl.zfaw79
Anopheles arabiensis	36.3948	-8.1658	Unclassified	GBIF: https://doi.org/10.15468/dl.zfaw79
Anopheles arabiensis	36.3949	-8.166	Unclassified	GBIF: https://doi.org/10.15468/dl.zfaw79
Anopheles arabiensis	36.395	-8.15722	Unclassified	GBIF: https://doi.org/10.15468/dl.zfaw79
Anopheles arabiensis	36.3952	-8.15354	Unclassified	GBIF: https://doi.org/10.15468/dl.zfaw79
Anopheles arabiensis	36.3952	-8.1626	Unclassified	GBIF: https://doi.org/10.15468/dl.zfaw79

Anopheles arabiensis	36.3952	-8.15473	Unclassified	GBIF: https://doi.org/10.15468/dl.zfaw79
Anopheles arabiensis	36.3956	-8.15948	Unclassified	GBIF: https://doi.org/10.15468/dl.zfaw79
Anopheles arabiensis	36.3956	-8.15978	Unclassified	GBIF: https://doi.org/10.15468/dl.zfaw79
Anopheles arabiensis	36.3956	-8.15543	Unclassified	GBIF: https://doi.org/10.15468/dl.zfaw79
Anopheles arabiensis	36.3956	-8.1708	Unclassified	GBIF: https://doi.org/10.15468/dl.zfaw79
Anopheles arabiensis	36.3956	-8.1709	Unclassified	GBIF: https://doi.org/10.15468/dl.zfaw79
Anopheles arabiensis	36.3958	-8.15662	Unclassified	GBIF: https://doi.org/10.15468/dl.zfaw79
Anopheles arabiensis	36.3959	-8.15623	Unclassified	GBIF: https://doi.org/10.15468/dl.zfaw79
Anopheles arabiensis	36.3959	-8.15885	Unclassified	GBIF: https://doi.org/10.15468/dl.zfaw79
Anopheles arabiensis	36.3962	-8.15373	Unclassified	GBIF: https://doi.org/10.15468/dl.zfaw79
Anopheles arabiensis	36.3963	-8.1744	Unclassified	GBIF: https://doi.org/10.15468/dl.zfaw79
Anopheles arabiensis	36.3963	-8.15737	Unclassified	GBIF: https://doi.org/10.15468/dl.zfaw79
Anopheles arabiensis	36.3963	-8.15403	Unclassified	GBIF: https://doi.org/10.15468/dl.zfaw79
Anopheles arabiensis	36.3963	-8.1743	Unclassified	GBIF: https://doi.org/10.15468/dl.zfaw79
Anopheles arabiensis	36.3966	-8.1542	Unclassified	GBIF: https://doi.org/10.15468/dl.zfaw79
Anopheles arabiensis	36.3968	-8.15583	Unclassified	GBIF: https://doi.org/10.15468/dl.zfaw79
Anopheles arabiensis	36.397	-8.19995	Unclassified	GBIF: https://doi.org/10.15468/dl.zfaw79
Anopheles arabiensis	36.3971	-8.15353	Unclassified	GBIF: https://doi.org/10.15468/dl.zfaw79
Anopheles arabiensis	36.3972	-8.15433	Unclassified	GBIF: https://doi.org/10.15468/dl.zfaw79
Anopheles arabiensis	36.3974	-8.15251	Unclassified	GBIF: https://doi.org/10.15468/dl.zfaw79
Anopheles arabiensis	36.3975	-8.15543	Unclassified	GBIF: https://doi.org/10.15468/dl.zfaw79
Anopheles arabiensis	36.3975	-8.15263	Unclassified	GBIF: https://doi.org/10.15468/dl.zfaw79
Anopheles arabiensis	36.3975	-8.1525	Unclassified	GBIF: https://doi.org/10.15468/dl.zfaw79
Anopheles arabiensis	36.3976	-8.15645	Unclassified	GBIF: https://doi.org/10.15468/dl.zfaw79
Anopheles arabiensis	36.3976	-8.15592	Unclassified	GBIF: https://doi.org/10.15468/dl.zfaw79
Anopheles arabiensis	36.3979	-8.15625	Unclassified	GBIF: https://doi.org/10.15468/dl.zfaw79
Anopheles arabiensis	36.3979	-8.15916	Unclassified	GBIF: https://doi.org/10.15468/dl.zfaw79
Anopheles arabiensis	36.3982	-8.15192	Unclassified	GBIF: https://doi.org/10.15468/dl.zfaw79
Anopheles arabiensis	36.3982	-8.15523	Unclassified	GBIF: https://doi.org/10.15468/dl.zfaw79
Anopheles arabiensis	36.3983	-8.15672	Unclassified	GBIF: https://doi.org/10.15468/dl.zfaw79
Anopheles arabiensis	36.3986	-8.15187	Unclassified	GBIF: https://doi.org/10.15468/dl.zfaw79
Anopheles arabiensis	36.3986	-8.15317	Unclassified	GBIF: https://doi.org/10.15468/dl.zfaw79
Anopheles arabiensis	36.3991	-8.15375	Unclassified	GBIF: https://doi.org/10.15468/dl.zfaw79
Anopheles arabiensis	36.3992	-8.15548	Unclassified	GBIF: https://doi.org/10.15468/dl.zfaw79
Anopheles arabiensis	36.3992	-8.15252	Unclassified	GBIF: https://doi.org/10.15468/dl.zfaw79
Anopheles arabiensis	36.3993	-8.22125	Unclassified	GBIF: https://doi.org/10.15468/dl.zfaw79
Anopheles arabiensis	36.3994	-8.15638	Unclassified	GBIF: https://doi.org/10.15468/dl.zfaw79
Anopheles arabiensis	36.3996	-8.15255	Unclassified	GBIF: https://doi.org/10.15468/dl.zfaw79
Anopheles arabiensis	36.3998	-8.16152	Unclassified	GBIF: https://doi.org/10.15468/dl.zfaw79
Anopheles arabiensis	36.4001	-8.1516	Unclassified	GBIF: https://doi.org/10.15468/dl.zfaw79
Anopheles arabiensis	36.4002	-8.15532	Unclassified	GBIF: https://doi.org/10.15468/dl.zfaw79
Anopheles arabiensis	36.4005	-8.15118	Unclassified	GBIF: https://doi.org/10.15468/dl.zfaw79

Anopheles arabiensis	36.4007	-8.15705	Unclassified	GBIF: https://doi.org/10.15468/dl.zfaw79
Anopheles arabiensis	36.401	-8.23767	Unclassified	GBIF: https://doi.org/10.15468/dl.zfaw79
Anopheles arabiensis	36.401	-8.22303	Unclassified	GBIF: https://doi.org/10.15468/dl.zfaw79
Anopheles arabiensis	36.4013	-8.23812	Unclassified	GBIF: https://doi.org/10.15468/dl.zfaw79
Anopheles arabiensis	36.4015	-8.15153	Unclassified	GBIF: https://doi.org/10.15468/dl.zfaw79
Anopheles arabiensis	36.4025	-8.15143	Unclassified	GBIF: https://doi.org/10.15468/dl.zfaw79
Anopheles arabiensis	36.4026	-8.1722	Unclassified	GBIF: https://doi.org/10.15468/dl.zfaw79
Anopheles arabiensis	36.4026	-8.1721	Unclassified	GBIF: https://doi.org/10.15468/dl.zfaw79
Anopheles arabiensis	36.4027	-8.1721	Unclassified	GBIF: https://doi.org/10.15468/dl.zfaw79
Anopheles arabiensis	36.4029	-8.2021	Unclassified	GBIF: https://doi.org/10.15468/dl.zfaw79
Anopheles arabiensis	36.4038	-8.1729	Unclassified	GBIF: https://doi.org/10.15468/dl.zfaw79
Anopheles arabiensis	36.4047	-8.20558	Unclassified	GBIF: https://doi.org/10.15468/dl.zfaw79
Anopheles arabiensis	36.405	-8.15203	Unclassified	GBIF: https://doi.org/10.15468/dl.zfaw79
Anopheles arabiensis	36.4051	-8.14951	Unclassified	GBIF: https://doi.org/10.15468/dl.zfaw79
Anopheles arabiensis	36.409	-8.1473	Unclassified	GBIF: https://doi.org/10.15468/dl.zfaw79
Anopheles arabiensis	36.4122	-8.1634	Unclassified	GBIF: https://doi.org/10.15468/dl.zfaw79
Anopheles arabiensis	36.4122	-8.1633	Unclassified	GBIF: https://doi.org/10.15468/dl.zfaw79
Anopheles arabiensis	36.4137	-8.2236	Unclassified	GBIF: https://doi.org/10.15468/dl.zfaw79
Anopheles arabiensis	36.4139	-8.2236	Unclassified	GBIF: https://doi.org/10.15468/dl.zfaw79
Anopheles arabiensis	36.414	-8.2236	Unclassified	GBIF: https://doi.org/10.15468/dl.zfaw79
Anopheles arabiensis	36.4141	-8.2236	Unclassified	GBIF: https://doi.org/10.15468/dl.zfaw79
Anopheles arabiensis	36.41667	7.45	Unclassified	GBIF: https://doi.org/10.15468/dl.zfaw79
Anopheles arabiensis	36.4173	-8.14743	Unclassified	GBIF: https://doi.org/10.15468/dl.zfaw79
Anopheles arabiensis	36.4175	-8.1485	Unclassified	GBIF: https://doi.org/10.15468/dl.zfaw79
Anopheles arabiensis	36.4178	-8.22123	Unclassified	GBIF: https://doi.org/10.15468/dl.zfaw79
Anopheles arabiensis	36.4191	-8.22228	Unclassified	GBIF: https://doi.org/10.15468/dl.zfaw79
Anopheles arabiensis	36.4193	-8.14423	Unclassified	GBIF: https://doi.org/10.15468/dl.zfaw79
Anopheles arabiensis	36.4193	-8.1442	Unclassified	GBIF: https://doi.org/10.15468/dl.zfaw79
Anopheles arabiensis	36.4209	-8.14275	Unclassified	GBIF: https://doi.org/10.15468/dl.zfaw79
Anopheles arabiensis	36.4221	-8.1767	Unclassified	GBIF: https://doi.org/10.15468/dl.zfaw79
Anopheles arabiensis	36.4221	-8.1768	Unclassified	GBIF: https://doi.org/10.15468/dl.zfaw79
Anopheles arabiensis	36.4222	-8.1768	Unclassified	GBIF: https://doi.org/10.15468/dl.zfaw79
Anopheles arabiensis	36.4222	-8.1769	Unclassified	GBIF: https://doi.org/10.15468/dl.zfaw79
Anopheles arabiensis	36.424	-8.1382	Unclassified	GBIF: https://doi.org/10.15468/dl.zfaw79
Anopheles arabiensis	36.4329	-8.1785	Unclassified	GBIF: https://doi.org/10.15468/dl.zfaw79
Anopheles arabiensis	36.4441	-8.2197	Unclassified	GBIF: https://doi.org/10.15468/dl.zfaw79
Anopheles arabiensis	36.4442	-8.2195	Unclassified	GBIF: https://doi.org/10.15468/dl.zfaw79
Anopheles arabiensis	36.4442	-8.2196	Unclassified	GBIF: https://doi.org/10.15468/dl.zfaw79
Anopheles arabiensis	36.4442	-8.2197	Unclassified	GBIF: https://doi.org/10.15468/dl.zfaw79
Anopheles arabiensis	36.4443	-8.2197	Unclassified	GBIF: https://doi.org/10.15468/dl.zfaw79
Anopheles arabiensis	36.4443	-8.2196	Unclassified	GBIF: https://doi.org/10.15468/dl.zfaw79
Anopheles arabiensis	36.4462	-8.1836	Unclassified	GBIF: https://doi.org/10.15468/dl.zfaw79

Anopheles arabiensis	36.4463	-8.1836	Unclassified	GBIF: https://doi.org/10.15468/dl.zfaw79
Anopheles arabiensis	36.4464	-8.1837	Unclassified	GBIF: https://doi.org/10.15468/dl.zfaw79
Anopheles arabiensis	36.4464	-8.1836	Unclassified	GBIF: https://doi.org/10.15468/dl.zfaw79
Anopheles arabiensis	36.4577	-8.1824	Unclassified	GBIF: https://doi.org/10.15468/dl.zfaw79
Anopheles arabiensis	36.4578	-8.1824	Unclassified	GBIF: https://doi.org/10.15468/dl.zfaw79
Anopheles arabiensis	36.4619	-8.1974	Unclassified	GBIF: https://doi.org/10.15468/dl.zfaw79
Anopheles arabiensis	36.462	-8.1974	Unclassified	GBIF: https://doi.org/10.15468/dl.zfaw79
Anopheles arabiensis	36.462	-8.1975	Unclassified	GBIF: https://doi.org/10.15468/dl.zfaw79
Anopheles arabiensis	36.4621	-8.1975	Unclassified	GBIF: https://doi.org/10.15468/dl.zfaw79
Anopheles arabiensis	36.4621	-8.1974	Unclassified	GBIF: https://doi.org/10.15468/dl.zfaw79
Anopheles arabiensis	36.4631	-8.2461	Unclassified	GBIF: https://doi.org/10.15468/dl.zfaw79
Anopheles arabiensis	36.4789	-8.2404	Unclassified	GBIF: https://doi.org/10.15468/dl.zfaw79
Anopheles arabiensis	36.479	-8.2404	Unclassified	GBIF: https://doi.org/10.15468/dl.zfaw79
Anopheles arabiensis	36.4814	-8.2688	Unclassified	GBIF: https://doi.org/10.15468/dl.zfaw79
Anopheles arabiensis	36.4817	-8.2375	Unclassified	GBIF: https://doi.org/10.15468/dl.zfaw79
Anopheles arabiensis	36.4818	-8.2376	Unclassified	GBIF: https://doi.org/10.15468/dl.zfaw79
Anopheles arabiensis	36.4818	-8.2375	Unclassified	GBIF: https://doi.org/10.15468/dl.zfaw79
Anopheles arabiensis	36.4818	-8.21	Unclassified	GBIF: https://doi.org/10.15468/dl.zfaw79
Anopheles arabiensis	36.4818	-8.2098	Unclassified	GBIF: https://doi.org/10.15468/dl.zfaw79
Anopheles arabiensis	36.4819	-8.2098	Unclassified	GBIF: https://doi.org/10.15468/dl.zfaw79
Anopheles arabiensis	36.4823	-8.2669	Unclassified	GBIF: https://doi.org/10.15468/dl.zfaw79
Anopheles arabiensis	36.4853	-8.2191	Unclassified	GBIF: https://doi.org/10.15468/dl.zfaw79
Anopheles arabiensis	36.4871	-8.08422	Unclassified	GBIF: https://doi.org/10.15468/dl.zfaw79
Anopheles arabiensis	36.4896	-8.2256	Unclassified	GBIF: https://doi.org/10.15468/dl.zfaw79
Anopheles arabiensis	36.4897	-8.2255	Unclassified	GBIF: https://doi.org/10.15468/dl.zfaw79
Anopheles arabiensis	36.4898	-8.2255	Unclassified	GBIF: https://doi.org/10.15468/dl.zfaw79
Anopheles arabiensis	36.4905	-8.2255	Unclassified	GBIF: https://doi.org/10.15468/dl.zfaw79
Anopheles arabiensis	36.4905	-8.2256	Unclassified	GBIF: https://doi.org/10.15468/dl.zfaw79
Anopheles arabiensis	36.4906	-8.2255	Unclassified	GBIF: https://doi.org/10.15468/dl.zfaw79
Anopheles arabiensis	36.4906	-8.2256	Unclassified	GBIF: https://doi.org/10.15468/dl.zfaw79
Anopheles arabiensis	36.4948	-8.08026	Unclassified	GBIF: https://doi.org/10.15468/dl.zfaw79
Anopheles arabiensis	36.4989	-8.08888	Unclassified	GBIF: https://doi.org/10.15468/dl.zfaw79
Anopheles arabiensis	36.499	-8.09943	Unclassified	GBIF: https://doi.org/10.15468/dl.zfaw79
Anopheles arabiensis	36.4999	-8.09793	Unclassified	GBIF: https://doi.org/10.15468/dl.zfaw79
Anopheles arabiensis	36.5	-7.45	Unclassified	GBIF: https://doi.org/10.15468/dl.zfaw79
Anopheles arabiensis	36.5022	-8.09957	Unclassified	GBIF: https://doi.org/10.15468/dl.zfaw79
Anopheles arabiensis	36.5071	-8.10013	Unclassified	GBIF: https://doi.org/10.15468/dl.zfaw79
Anopheles arabiensis	36.5083	-8.10252	Unclassified	GBIF: https://doi.org/10.15468/dl.zfaw79
Anopheles arabiensis	36.5084	-8.1015	Unclassified	GBIF: https://doi.org/10.15468/dl.zfaw79
Anopheles arabiensis	36.5086	-8.10095	Unclassified	GBIF: https://doi.org/10.15468/dl.zfaw79
Anopheles arabiensis	36.5088	-8.10113	Unclassified	GBIF: https://doi.org/10.15468/dl.zfaw79
Anopheles arabiensis	36.5088	-8.08348	Unclassified	GBIF: https://doi.org/10.15468/dl.zfaw79

Anopheles arabiensis	36.5089	-8.10162	Unclassified	GBIF: https://doi.org/10.15468/dl.zfaw79
Anopheles arabiensis	36.5089	-8.10205	Unclassified	GBIF: https://doi.org/10.15468/dl.zfaw79
Anopheles arabiensis	36.509	-8.1024	Unclassified	GBIF: https://doi.org/10.15468/dl.zfaw79
Anopheles arabiensis	36.509	-8.09308	Unclassified	GBIF: https://doi.org/10.15468/dl.zfaw79
Anopheles arabiensis	36.5091	-8.10053	Unclassified	GBIF: https://doi.org/10.15468/dl.zfaw79
Anopheles arabiensis	36.5092	-8.10068	Unclassified	GBIF: https://doi.org/10.15468/dl.zfaw79
Anopheles arabiensis	36.5093	-8.1007	Unclassified	GBIF: https://doi.org/10.15468/dl.zfaw79
Anopheles arabiensis	36.5093	-8.09962	Unclassified	GBIF: https://doi.org/10.15468/dl.zfaw79
Anopheles arabiensis	36.5097	-8.09598	Unclassified	GBIF: https://doi.org/10.15468/dl.zfaw79
Anopheles arabiensis	36.5098	-8.09598	Unclassified	GBIF: https://doi.org/10.15468/dl.zfaw79
Anopheles arabiensis	36.51	-8.10127	Unclassified	GBIF: https://doi.org/10.15468/dl.zfaw79
Anopheles arabiensis	36.5102	-8.10118	Unclassified	GBIF: https://doi.org/10.15468/dl.zfaw79
Anopheles arabiensis	36.5103	-8.10002	Unclassified	GBIF: https://doi.org/10.15468/dl.zfaw79
Anopheles arabiensis	36.5103	-8.09778	Unclassified	GBIF: https://doi.org/10.15468/dl.zfaw79
Anopheles arabiensis	36.5104	-8.09688	Unclassified	GBIF: https://doi.org/10.15468/dl.zfaw79
Anopheles arabiensis	36.5105	-8.09998	Unclassified	GBIF: https://doi.org/10.15468/dl.zfaw79
Anopheles arabiensis	36.5107	-8.10348	Unclassified	GBIF: https://doi.org/10.15468/dl.zfaw79
Anopheles arabiensis	36.5107	-8.09993	Unclassified	GBIF: https://doi.org/10.15468/dl.zfaw79
Anopheles arabiensis	36.5108	-8.10322	Unclassified	GBIF: https://doi.org/10.15468/dl.zfaw79
Anopheles arabiensis	36.5108	-8.10037	Unclassified	GBIF: https://doi.org/10.15468/dl.zfaw79
Anopheles arabiensis	36.511	-8.10002	Unclassified	GBIF: https://doi.org/10.15468/dl.zfaw79
Anopheles arabiensis	36.5111	-8.098	Unclassified	GBIF: https://doi.org/10.15468/dl.zfaw79
Anopheles arabiensis	36.5111	-8.10248	Unclassified	GBIF: https://doi.org/10.15468/dl.zfaw79
Anopheles arabiensis	36.5111	-8.09627	Unclassified	GBIF: https://doi.org/10.15468/dl.zfaw79
Anopheles arabiensis	36.5114	-8.09942	Unclassified	GBIF: https://doi.org/10.15468/dl.zfaw79
Anopheles arabiensis	36.5115	-8.10038	Unclassified	GBIF: https://doi.org/10.15468/dl.zfaw79
Anopheles arabiensis	36.5116	-8.0941	Unclassified	GBIF: https://doi.org/10.15468/dl.zfaw79
Anopheles arabiensis	36.5121	-8.09948	Unclassified	GBIF: https://doi.org/10.15468/dl.zfaw79
Anopheles arabiensis	36.5122	-8.10003	Unclassified	GBIF: https://doi.org/10.15468/dl.zfaw79
Anopheles arabiensis	36.5128	-8.09644	Unclassified	GBIF: https://doi.org/10.15468/dl.zfaw79
Anopheles arabiensis	36.5131	-8.1007	Unclassified	GBIF: https://doi.org/10.15468/dl.zfaw79
Anopheles arabiensis	36.5168	-8.1001	Unclassified	GBIF: https://doi.org/10.15468/dl.zfaw79
Anopheles arabiensis	36.5173	-8.09877	Unclassified	GBIF: https://doi.org/10.15468/dl.zfaw79
Anopheles arabiensis	36.5177	-8.09588	Unclassified	GBIF: https://doi.org/10.15468/dl.zfaw79
Anopheles arabiensis	36.5182	-8.09975	Unclassified	GBIF: https://doi.org/10.15468/dl.zfaw79
Anopheles arabiensis	36.5189	-8.09853	Unclassified	GBIF: https://doi.org/10.15468/dl.zfaw79
Anopheles arabiensis	36.5199	-8.10857	Unclassified	GBIF: https://doi.org/10.15468/dl.zfaw79
Anopheles arabiensis	36.5201	-8.09785	Unclassified	GBIF: https://doi.org/10.15468/dl.zfaw79
Anopheles arabiensis	36.5202	-8.1081	Unclassified	GBIF: https://doi.org/10.15468/dl.zfaw79
Anopheles arabiensis	36.5202	-8.0986	Unclassified	GBIF: https://doi.org/10.15468/dl.zfaw79
Anopheles arabiensis	36.5202	-8.10823	Unclassified	GBIF: https://doi.org/10.15468/dl.zfaw79
Anopheles arabiensis	36.5205	-8.1017	Unclassified	GBIF: https://doi.org/10.15468/dl.zfaw79

Anopheles arabiensis	36.5206	-8.10062	Unclassified	GBIF: https://doi.org/10.15468/dl.zfaw79
Anopheles arabiensis	36.5208	-8.1011	Unclassified	GBIF: https://doi.org/10.15468/dl.zfaw79
Anopheles arabiensis	36.5209	-8.09753	Unclassified	GBIF: https://doi.org/10.15468/dl.zfaw79
Anopheles arabiensis	36.521	-8.0977	Unclassified	GBIF: https://doi.org/10.15468/dl.zfaw79
Anopheles arabiensis	36.5214	-8.1098	Unclassified	GBIF: https://doi.org/10.15468/dl.zfaw79
Anopheles arabiensis	36.5227	-8.09815	Unclassified	GBIF: https://doi.org/10.15468/dl.zfaw79
Anopheles arabiensis	36.5232	-8.09953	Unclassified	GBIF: https://doi.org/10.15468/dl.zfaw79
Anopheles arabiensis	36.5234	-8.108	Unclassified	GBIF: https://doi.org/10.15468/dl.zfaw79
Anopheles arabiensis	36.524	-8.08778	Unclassified	GBIF: https://doi.org/10.15468/dl.zfaw79
Anopheles arabiensis	36.524	-8.10003	Unclassified	GBIF: https://doi.org/10.15468/dl.zfaw79
Anopheles arabiensis	36.5243	-8.10058	Unclassified	GBIF: https://doi.org/10.15468/dl.zfaw79
Anopheles arabiensis	36.5264	-8.09977	Unclassified	GBIF: https://doi.org/10.15468/dl.zfaw79
Anopheles arabiensis	36.5283	-8.09658	Unclassified	GBIF: https://doi.org/10.15468/dl.zfaw79
Anopheles arabiensis	36.5283	-8.10648	Unclassified	GBIF: https://doi.org/10.15468/dl.zfaw79
Anopheles arabiensis	36.5289	-8.09548	Unclassified	GBIF: https://doi.org/10.15468/dl.zfaw79
Anopheles arabiensis	36.5296	-8.0651	Unclassified	GBIF: https://doi.org/10.15468/dl.zfaw79
Anopheles arabiensis	36.5308	-8.06853	Unclassified	GBIF: https://doi.org/10.15468/dl.zfaw79
Anopheles arabiensis	36.5323	-8.09362	Unclassified	GBIF: https://doi.org/10.15468/dl.zfaw79
Anopheles arabiensis	36.5336	-8.06665	Unclassified	GBIF: https://doi.org/10.15468/dl.zfaw79
Anopheles arabiensis	36.5343	-8.11875	Unclassified	GBIF: https://doi.org/10.15468/dl.zfaw79
Anopheles arabiensis	36.549	-8.06653	Unclassified	GBIF: https://doi.org/10.15468/dl.zfaw79
Anopheles arabiensis	36.5505	-8.11457	Unclassified	GBIF: https://doi.org/10.15468/dl.zfaw79
Anopheles arabiensis	36.5521	-8.11192	Unclassified	GBIF: https://doi.org/10.15468/dl.zfaw79
Anopheles arabiensis	36.5587	-8.13008	Unclassified	GBIF: https://doi.org/10.15468/dl.zfaw79
Anopheles arabiensis	36.6202	-8.41346	Unclassified	GBIF: https://doi.org/10.15468/dl.zfaw79
Anopheles arabiensis	36.6224	-8.41348	Unclassified	GBIF: https://doi.org/10.15468/dl.zfaw79
Anopheles arabiensis	36.6255	-8.41094	Unclassified	GBIF: https://doi.org/10.15468/dl.zfaw79
Anopheles arabiensis	36.6257	-8.41097	Unclassified	GBIF: https://doi.org/10.15468/dl.zfaw79
Anopheles arabiensis	36.6262	-8.41262	Unclassified	GBIF: https://doi.org/10.15468/dl.zfaw79
Anopheles arabiensis	36.6264	-8.4106	Unclassified	GBIF: https://doi.org/10.15468/dl.zfaw79
Anopheles arabiensis	36.6268	-8.41439	Unclassified	GBIF: https://doi.org/10.15468/dl.zfaw79
Anopheles arabiensis	36.6271	-8.41431	Unclassified	GBIF: https://doi.org/10.15468/dl.zfaw79
Anopheles arabiensis	36.6275	-8.40995	Unclassified	GBIF: https://doi.org/10.15468/dl.zfaw79
Anopheles arabiensis	36.6276	-8.41333	Unclassified	GBIF: https://doi.org/10.15468/dl.zfaw79
Anopheles arabiensis	36.6278	-8.40854	Unclassified	GBIF: https://doi.org/10.15468/dl.zfaw79
Anopheles arabiensis	36.6279	-8.41206	Unclassified	GBIF: https://doi.org/10.15468/dl.zfaw79
Anopheles arabiensis	36.6279	-8.40844	Unclassified	GBIF: https://doi.org/10.15468/dl.zfaw79
Anopheles arabiensis	36.6279	-8.41268	Unclassified	GBIF: https://doi.org/10.15468/dl.zfaw79
Anopheles arabiensis	36.6281	-8.40974	Unclassified	GBIF: https://doi.org/10.15468/dl.zfaw79
Anopheles arabiensis	36.6284	-8.4114	Unclassified	GBIF: https://doi.org/10.15468/dl.zfaw79
Anopheles arabiensis	36.6284	-8.41319	Unclassified	GBIF: https://doi.org/10.15468/dl.zfaw79
Anopheles arabiensis	36.6284	-8.4119	Unclassified	GBIF: https://doi.org/10.15468/dl.zfaw79

Anopheles arabiensis	36.6285	-8.41076	Unclassified	GBIF: https://doi.org/10.15468/dl.zfaw79
Anopheles arabiensis	36.6285	-8.40835	Unclassified	GBIF: https://doi.org/10.15468/dl.zfaw79
Anopheles arabiensis	36.6287	-8.41079	Unclassified	GBIF: https://doi.org/10.15468/dl.zfaw79
Anopheles arabiensis	36.6289	-8.41265	Unclassified	GBIF: https://doi.org/10.15468/dl.zfaw79
Anopheles arabiensis	36.629	-8.4089	Unclassified	GBIF: https://doi.org/10.15468/dl.zfaw79
Anopheles arabiensis	36.6296	-8.40965	Unclassified	GBIF: https://doi.org/10.15468/dl.zfaw79
Anopheles arabiensis	36.6297	-8.41156	Unclassified	GBIF: https://doi.org/10.15468/dl.zfaw79
Anopheles arabiensis	36.6299	-8.40929	Unclassified	GBIF: https://doi.org/10.15468/dl.zfaw79
Anopheles arabiensis	36.6299	-8.40894	Unclassified	GBIF: https://doi.org/10.15468/dl.zfaw79
Anopheles arabiensis	36.6302	-8.40816	Unclassified	GBIF: https://doi.org/10.15468/dl.zfaw79
Anopheles arabiensis	36.6304	-8.40884	Unclassified	GBIF: https://doi.org/10.15468/dl.zfaw79
Anopheles arabiensis	36.6304	-8.40857	Unclassified	GBIF: https://doi.org/10.15468/dl.zfaw79
Anopheles arabiensis	36.6306	-8.40844	Unclassified	GBIF: https://doi.org/10.15468/dl.zfaw79
Anopheles arabiensis	36.6306	-8.40676	Unclassified	GBIF: https://doi.org/10.15468/dl.zfaw79
Anopheles arabiensis	36.631	-8.41112	Unclassified	GBIF: https://doi.org/10.15468/dl.zfaw79
Anopheles arabiensis	36.6314	-8.41114	Unclassified	GBIF: https://doi.org/10.15468/dl.zfaw79
Anopheles arabiensis	36.6315	-8.41109	Unclassified	GBIF: https://doi.org/10.15468/dl.zfaw79
Anopheles arabiensis	36.6321	-8.40645	Unclassified	GBIF: https://doi.org/10.15468/dl.zfaw79
Anopheles arabiensis	36.63333	-8.16667	Unclassified	GBIF: https://doi.org/10.15468/dl.zfaw79
Anopheles arabiensis	36.6667	15.1333	Unclassified	GBIF: https://doi.org/10.15468/dl.zfaw79
Anopheles arabiensis	36.683	-8.14607	Unclassified	GBIF: https://doi.org/10.15468/dl.zfaw79
Anopheles arabiensis	36.68333	-8.16667	Unclassified	GBIF: https://doi.org/10.15468/dl.zfaw79
Anopheles arabiensis	36.691	-8.47978	Unclassified	GBIF: https://doi.org/10.15468/dl.zfaw79
Anopheles arabiensis	36.6915	-8.48041	Unclassified	GBIF: https://doi.org/10.15468/dl.zfaw79
Anopheles arabiensis	36.6918	-8.48184	Unclassified	GBIF: https://doi.org/10.15468/dl.zfaw79
Anopheles arabiensis	36.6919	-8.47922	Unclassified	GBIF: https://doi.org/10.15468/dl.zfaw79
Anopheles arabiensis	36.692	-8.47982	Unclassified	GBIF: https://doi.org/10.15468/dl.zfaw79
Anopheles arabiensis	36.692	-8.47976	Unclassified	GBIF: https://doi.org/10.15468/dl.zfaw79
Anopheles arabiensis	36.6926	-8.48814	Unclassified	GBIF: https://doi.org/10.15468/dl.zfaw79
Anopheles arabiensis	36.6932	-8.4814	Unclassified	GBIF: https://doi.org/10.15468/dl.zfaw79
Anopheles arabiensis	36.6935	-8.48621	Unclassified	GBIF: https://doi.org/10.15468/dl.zfaw79
Anopheles arabiensis	36.6937	-8.48751	Unclassified	GBIF: https://doi.org/10.15468/dl.zfaw79
Anopheles arabiensis	36.694	-8.48647	Unclassified	GBIF: https://doi.org/10.15468/dl.zfaw79
Anopheles arabiensis	36.6941	-8.48625	Unclassified	GBIF: https://doi.org/10.15468/dl.zfaw79
Anopheles arabiensis	36.6945	-8.48585	Unclassified	GBIF: https://doi.org/10.15468/dl.zfaw79
Anopheles arabiensis	36.6947	-8.49933	Unclassified	GBIF: https://doi.org/10.15468/dl.zfaw79
Anopheles arabiensis	36.6948	-8.48607	Unclassified	GBIF: https://doi.org/10.15468/dl.zfaw79
Anopheles arabiensis	36.6948	-8.48604	Unclassified	GBIF: https://doi.org/10.15468/dl.zfaw79
Anopheles arabiensis	36.6949	-8.48582	Unclassified	GBIF: https://doi.org/10.15468/dl.zfaw79
Anopheles arabiensis	36.6949	-8.50027	Unclassified	GBIF: https://doi.org/10.15468/dl.zfaw79
Anopheles arabiensis	36.6949	-8.48596	Unclassified	GBIF: https://doi.org/10.15468/dl.zfaw79
Anopheles arabiensis	36.6953	-8.4898	Unclassified	GBIF: https://doi.org/10.15468/dl.zfaw79

Anopheles arabiensis	36.6954	-8.51941	Unclassified	GBIF: https://doi.org/10.15468/dl.zfaw79
Anopheles arabiensis	36.6958	-8.50058	Unclassified	GBIF: https://doi.org/10.15468/dl.zfaw79
Anopheles arabiensis	36.6959	-8.52399	Unclassified	GBIF: https://doi.org/10.15468/dl.zfaw79
Anopheles arabiensis	36.6962	-8.51988	Unclassified	GBIF: https://doi.org/10.15468/dl.zfaw79
Anopheles arabiensis	36.6968	-8.51633	Unclassified	GBIF: https://doi.org/10.15468/dl.zfaw79
Anopheles arabiensis	36.6969	-8.5165	Unclassified	GBIF: https://doi.org/10.15468/dl.zfaw79
Anopheles arabiensis	36.6973	-8.53006	Unclassified	GBIF: https://doi.org/10.15468/dl.zfaw79
Anopheles arabiensis	36.6974	-8.53049	Unclassified	GBIF: https://doi.org/10.15468/dl.zfaw79
Anopheles arabiensis	36.6978	-8.5073	Unclassified	GBIF: https://doi.org/10.15468/dl.zfaw79
Anopheles arabiensis	36.698	-8.51669	Unclassified	GBIF: https://doi.org/10.15468/dl.zfaw79
Anopheles arabiensis	36.6981	-8.50765	Unclassified	GBIF: https://doi.org/10.15468/dl.zfaw79
Anopheles arabiensis	36.6998	-8.51657	Unclassified	GBIF: https://doi.org/10.15468/dl.zfaw79
Anopheles arabiensis	36.6998	-8.51674	Unclassified	GBIF: https://doi.org/10.15468/dl.zfaw79
Anopheles arabiensis	36.6999	-8.51649	Unclassified	GBIF: https://doi.org/10.15468/dl.zfaw79
Anopheles arabiensis	36.7	-8.51642	Unclassified	GBIF: https://doi.org/10.15468/dl.zfaw79
Anopheles arabiensis	36.7	-8.51646	Unclassified	GBIF: https://doi.org/10.15468/dl.zfaw79
Anopheles arabiensis	36.7001	-8.51633	Unclassified	GBIF: https://doi.org/10.15468/dl.zfaw79
Anopheles arabiensis	36.7005	-8.51308	Unclassified	GBIF: https://doi.org/10.15468/dl.zfaw79
Anopheles arabiensis	36.7012	-8.53656	Unclassified	GBIF: https://doi.org/10.15468/dl.zfaw79
Anopheles arabiensis	36.7027	-8.5365	Unclassified	GBIF: https://doi.org/10.15468/dl.zfaw79
Anopheles arabiensis	36.7028	-8.53652	Unclassified	GBIF: https://doi.org/10.15468/dl.zfaw79
Anopheles arabiensis	36.7217	-8.61079	Unclassified	GBIF: https://doi.org/10.15468/dl.zfaw79
Anopheles arabiensis	36.7217	-8.61201	Unclassified	GBIF: https://doi.org/10.15468/dl.zfaw79
Anopheles arabiensis	36.7246	-8.60832	Unclassified	GBIF: https://doi.org/10.15468/dl.zfaw79
Anopheles arabiensis	36.7248	-8.60858	Unclassified	GBIF: https://doi.org/10.15468/dl.zfaw79
Anopheles arabiensis	36.7252	-8.60745	Unclassified	GBIF: https://doi.org/10.15468/dl.zfaw79
Anopheles arabiensis	36.7252	-8.60697	Unclassified	GBIF: https://doi.org/10.15468/dl.zfaw79
Anopheles arabiensis	36.7253	-8.86173	Unclassified	GBIF: https://doi.org/10.15468/dl.zfaw79
Anopheles arabiensis	36.7255	-8.60777	Unclassified	GBIF: https://doi.org/10.15468/dl.zfaw79
Anopheles arabiensis	36.7256	-8.86295	Unclassified	GBIF: https://doi.org/10.15468/dl.zfaw79
Anopheles arabiensis	36.7269	-8.86636	Unclassified	GBIF: https://doi.org/10.15468/dl.zfaw79
Anopheles arabiensis	36.727	-8.86573	Unclassified	GBIF: https://doi.org/10.15468/dl.zfaw79
Anopheles arabiensis	36.7272	-8.86899	Unclassified	GBIF: https://doi.org/10.15468/dl.zfaw79
Anopheles arabiensis	36.7273	-8.87002	Unclassified	GBIF: https://doi.org/10.15468/dl.zfaw79
Anopheles arabiensis	36.7273	-8.86715	Unclassified	GBIF: https://doi.org/10.15468/dl.zfaw79
Anopheles arabiensis	36.7281	-8.87319	Unclassified	GBIF: https://doi.org/10.15468/dl.zfaw79
Anopheles arabiensis	36.7284	-8.87186	Unclassified	GBIF: https://doi.org/10.15468/dl.zfaw79
Anopheles arabiensis	36.7285	-8.60201	Unclassified	GBIF: https://doi.org/10.15468/dl.zfaw79
Anopheles arabiensis	36.7286	-8.87508	Unclassified	GBIF: https://doi.org/10.15468/dl.zfaw79
Anopheles arabiensis	36.7289	-8.87616	Unclassified	GBIF: https://doi.org/10.15468/dl.zfaw79
Anopheles arabiensis	36.729	-8.87831	Unclassified	GBIF: https://doi.org/10.15468/dl.zfaw79
Anopheles arabiensis	36.7291	-8.87648	Unclassified	GBIF: https://doi.org/10.15468/dl.zfaw79

Anopheles arabiensis	36.7294	-8.87979	Unclassified	GBIF: https://doi.org/10.15468/dl.zfaw79
Anopheles arabiensis	36.7297	-8.87908	Unclassified	GBIF: https://doi.org/10.15468/dl.zfaw79
Anopheles arabiensis	36.73	-8.88001	Unclassified	GBIF: https://doi.org/10.15468/dl.zfaw79
Anopheles arabiensis	36.73	-8.87801	Unclassified	GBIF: https://doi.org/10.15468/dl.zfaw79
Anopheles arabiensis	36.7301	-8.88224	Unclassified	GBIF: https://doi.org/10.15468/dl.zfaw79
Anopheles arabiensis	36.7301	-8.6021	Unclassified	GBIF: https://doi.org/10.15468/dl.zfaw79
Anopheles arabiensis	36.7308	-8.88222	Unclassified	GBIF: https://doi.org/10.15468/dl.zfaw79
Anopheles arabiensis	36.7308	-8.88076	Unclassified	GBIF: https://doi.org/10.15468/dl.zfaw79
Anopheles arabiensis	36.7316	-8.8882	Unclassified	GBIF: https://doi.org/10.15468/dl.zfaw79
Anopheles arabiensis	36.7318	-8.88838	Unclassified	GBIF: https://doi.org/10.15468/dl.zfaw79
Anopheles arabiensis	36.732	-8.59858	Unclassified	GBIF: https://doi.org/10.15468/dl.zfaw79
Anopheles arabiensis	36.7329	-8.89041	Unclassified	GBIF: https://doi.org/10.15468/dl.zfaw79
Anopheles arabiensis	36.7333	-8.89005	Unclassified	GBIF: https://doi.org/10.15468/dl.zfaw79
Anopheles arabiensis	36.7341	-8.89544	Unclassified	GBIF: https://doi.org/10.15468/dl.zfaw79
Anopheles arabiensis	36.7344	-8.8955	Unclassified	GBIF: https://doi.org/10.15468/dl.zfaw79
Anopheles arabiensis	36.7364	-8.89992	Unclassified	GBIF: https://doi.org/10.15468/dl.zfaw79
Anopheles arabiensis	36.7368	-8.90165	Unclassified	GBIF: https://doi.org/10.15468/dl.zfaw79
Anopheles arabiensis	36.7369	-8.90213	Unclassified	GBIF: https://doi.org/10.15468/dl.zfaw79
Anopheles arabiensis	36.7583	-8.95023	Unclassified	GBIF: https://doi.org/10.15468/dl.zfaw79
Anopheles arabiensis	36.7583	-8.95011	Unclassified	GBIF: https://doi.org/10.15468/dl.zfaw79
Anopheles arabiensis	36.7583	-8.97886	Unclassified	GBIF: https://doi.org/10.15468/dl.zfaw79
Anopheles arabiensis	36.7585	-8.9497	Unclassified	GBIF: https://doi.org/10.15468/dl.zfaw79
Anopheles arabiensis	36.7586	-8.94939	Unclassified	GBIF: https://doi.org/10.15468/dl.zfaw79
Anopheles arabiensis	36.7586	-8.94952	Unclassified	GBIF: https://doi.org/10.15468/dl.zfaw79
Anopheles arabiensis	36.7588	-8.94738	Unclassified	GBIF: https://doi.org/10.15468/dl.zfaw79
Anopheles arabiensis	36.7588	-8.94926	Unclassified	GBIF: https://doi.org/10.15468/dl.zfaw79
Anopheles arabiensis	36.7588	-8.98038	Unclassified	GBIF: https://doi.org/10.15468/dl.zfaw79
Anopheles arabiensis	36.7589	-8.94892	Unclassified	GBIF: https://doi.org/10.15468/dl.zfaw79
Anopheles arabiensis	36.759	-8.97857	Unclassified	GBIF: https://doi.org/10.15468/dl.zfaw79
Anopheles arabiensis	36.7592	-8.9797	Unclassified	GBIF: https://doi.org/10.15468/dl.zfaw79
Anopheles arabiensis	36.7593	-8.97919	Unclassified	GBIF: https://doi.org/10.15468/dl.zfaw79
Anopheles arabiensis	36.7594	-8.97905	Unclassified	GBIF: https://doi.org/10.15468/dl.zfaw79
Anopheles arabiensis	36.7595	-8.97895	Unclassified	GBIF: https://doi.org/10.15468/dl.zfaw79
Anopheles arabiensis	36.7596	-8.96189	Unclassified	GBIF: https://doi.org/10.15468/dl.zfaw79
Anopheles arabiensis	36.7596	-8.97968	Unclassified	GBIF: https://doi.org/10.15468/dl.zfaw79
Anopheles arabiensis	36.7596	-8.97864	Unclassified	GBIF: https://doi.org/10.15468/dl.zfaw79
Anopheles arabiensis	36.7598	-8.97867	Unclassified	GBIF: https://doi.org/10.15468/dl.zfaw79
Anopheles arabiensis	36.7598	-8.94694	Unclassified	GBIF: https://doi.org/10.15468/dl.zfaw79
Anopheles arabiensis	36.7599	-8.96194	Unclassified	GBIF: https://doi.org/10.15468/dl.zfaw79
Anopheles arabiensis	36.76	-8.97787	Unclassified	GBIF: https://doi.org/10.15468/dl.zfaw79
Anopheles arabiensis	36.76	-8.97795	Unclassified	GBIF: https://doi.org/10.15468/dl.zfaw79
Anopheles arabiensis	36.7601	-8.97771	Unclassified	GBIF: https://doi.org/10.15468/dl.zfaw79

Anopheles arabiensis	36.7602	-8.97755	Unclassified	GBIF: https://doi.org/10.15468/dl.zfaw79
Anopheles arabiensis	36.7603	-8.96996	Unclassified	GBIF: https://doi.org/10.15468/dl.zfaw79
Anopheles arabiensis	36.7604	-8.95647	Unclassified	GBIF: https://doi.org/10.15468/dl.zfaw79
Anopheles arabiensis	36.7605	-8.97616	Unclassified	GBIF: https://doi.org/10.15468/dl.zfaw79
Anopheles arabiensis	36.7605	-8.96944	Unclassified	GBIF: https://doi.org/10.15468/dl.zfaw79
Anopheles arabiensis	36.7606	-8.96154	Unclassified	GBIF: https://doi.org/10.15468/dl.zfaw79
Anopheles arabiensis	36.7606	-8.9614	Unclassified	GBIF: https://doi.org/10.15468/dl.zfaw79
Anopheles arabiensis	36.7606	-8.96226	Unclassified	GBIF: https://doi.org/10.15468/dl.zfaw79
Anopheles arabiensis	36.7606	-8.971	Unclassified	GBIF: https://doi.org/10.15468/dl.zfaw79
Anopheles arabiensis	36.7606	-8.97091	Unclassified	GBIF: https://doi.org/10.15468/dl.zfaw79
Anopheles arabiensis	36.7607	-8.96221	Unclassified	GBIF: https://doi.org/10.15468/dl.zfaw79
Anopheles arabiensis	36.7607	-8.96222	Unclassified	GBIF: https://doi.org/10.15468/dl.zfaw79
Anopheles arabiensis	36.7608	-8.96141	Unclassified	GBIF: https://doi.org/10.15468/dl.zfaw79
Anopheles arabiensis	36.7608	-8.96845	Unclassified	GBIF: https://doi.org/10.15468/dl.zfaw79
Anopheles arabiensis	36.7609	-8.97061	Unclassified	GBIF: https://doi.org/10.15468/dl.zfaw79
Anopheles arabiensis	36.7609	-8.96219	Unclassified	GBIF: https://doi.org/10.15468/dl.zfaw79
Anopheles arabiensis	36.7609	-8.96313	Unclassified	GBIF: https://doi.org/10.15468/dl.zfaw79
Anopheles arabiensis	36.7609	-8.97426	Unclassified	GBIF: https://doi.org/10.15468/dl.zfaw79
Anopheles arabiensis	36.7609	-8.97112	Unclassified	GBIF: https://doi.org/10.15468/dl.zfaw79
Anopheles arabiensis	36.761	-8.96127	Unclassified	GBIF: https://doi.org/10.15468/dl.zfaw79
Anopheles arabiensis	36.761	-8.96253	Unclassified	GBIF: https://doi.org/10.15468/dl.zfaw79
Anopheles arabiensis	36.7611	-8.96687	Unclassified	GBIF: https://doi.org/10.15468/dl.zfaw79
Anopheles arabiensis	36.7611	-8.9629	Unclassified	GBIF: https://doi.org/10.15468/dl.zfaw79
Anopheles arabiensis	36.762	-8.94141	Unclassified	GBIF: https://doi.org/10.15468/dl.zfaw79
Anopheles arabiensis	36.7623	-8.94154	Unclassified	GBIF: https://doi.org/10.15468/dl.zfaw79
Anopheles arabiensis	36.7624	-8.9427	Unclassified	GBIF: https://doi.org/10.15468/dl.zfaw79
Anopheles arabiensis	36.7625	-8.94399	Unclassified	GBIF: https://doi.org/10.15468/dl.zfaw79
Anopheles arabiensis	36.7626	-8.94097	Unclassified	GBIF: https://doi.org/10.15468/dl.zfaw79
Anopheles arabiensis	36.7626	-8.94084	Unclassified	GBIF: https://doi.org/10.15468/dl.zfaw79
Anopheles arabiensis	36.7626	-8.94384	Unclassified	GBIF: https://doi.org/10.15468/dl.zfaw79
Anopheles arabiensis	36.7629	-8.94436	Unclassified	GBIF: https://doi.org/10.15468/dl.zfaw79
Anopheles arabiensis	36.7629	-8.94523	Unclassified	GBIF: https://doi.org/10.15468/dl.zfaw79
Anopheles arabiensis	36.7629	-8.94396	Unclassified	GBIF: https://doi.org/10.15468/dl.zfaw79
Anopheles arabiensis	36.763	-8.94315	Unclassified	GBIF: https://doi.org/10.15468/dl.zfaw79
Anopheles arabiensis	36.763	-8.93949	Unclassified	GBIF: https://doi.org/10.15468/dl.zfaw79
Anopheles arabiensis	36.7631	-8.93964	Unclassified	GBIF: https://doi.org/10.15468/dl.zfaw79
Anopheles arabiensis	36.7633	-8.94135	Unclassified	GBIF: https://doi.org/10.15468/dl.zfaw79
Anopheles arabiensis	36.7633	-8.93885	Unclassified	GBIF: https://doi.org/10.15468/dl.zfaw79
Anopheles arabiensis	36.7633	-8.94359	Unclassified	GBIF: https://doi.org/10.15468/dl.zfaw79
Anopheles arabiensis	36.7634	-8.94563	Unclassified	GBIF: https://doi.org/10.15468/dl.zfaw79
Anopheles arabiensis	36.7634	-8.93906	Unclassified	GBIF: https://doi.org/10.15468/dl.zfaw79
Anopheles arabiensis	36.7635	-8.93905	Unclassified	GBIF: https://doi.org/10.15468/dl.zfaw79

Anopheles arabiensis	36.7635	-8.94143	Unclassified	GBIF: https://doi.org/10.15468/dl.zfaw79
Anopheles arabiensis	36.7636	-8.94478	Unclassified	GBIF: https://doi.org/10.15468/dl.zfaw79
Anopheles arabiensis	36.7639	-8.94364	Unclassified	GBIF: https://doi.org/10.15468/dl.zfaw79
Anopheles arabiensis	36.7639	-8.93876	Unclassified	GBIF: https://doi.org/10.15468/dl.zfaw79
Anopheles arabiensis	36.7642	-8.93836	Unclassified	GBIF: https://doi.org/10.15468/dl.zfaw79
Anopheles arabiensis	36.7645	-8.94355	Unclassified	GBIF: https://doi.org/10.15468/dl.zfaw79
Anopheles arabiensis	36.7821	-9.07734	Unclassified	GBIF: https://doi.org/10.15468/dl.zfaw79
Anopheles arabiensis	36.7826	-9.07687	Unclassified	GBIF: https://doi.org/10.15468/dl.zfaw79
Anopheles arabiensis	36.7827	-9.07739	Unclassified	GBIF: https://doi.org/10.15468/dl.zfaw79
Anopheles arabiensis	36.7829	-9.0767	Unclassified	GBIF: https://doi.org/10.15468/dl.zfaw79
Anopheles arabiensis	36.7831	-9.07527	Unclassified	GBIF: https://doi.org/10.15468/dl.zfaw79
Anopheles arabiensis	36.7832	-9.07705	Unclassified	GBIF: https://doi.org/10.15468/dl.zfaw79
Anopheles arabiensis	36.7834	-9.07646	Unclassified	GBIF: https://doi.org/10.15468/dl.zfaw79
Anopheles arabiensis	36.7834	-9.07759	Unclassified	GBIF: https://doi.org/10.15468/dl.zfaw79
Anopheles arabiensis	36.7835	-9.07558	Unclassified	GBIF: https://doi.org/10.15468/dl.zfaw79
Anopheles arabiensis	36.7838	-9.07466	Unclassified	GBIF: https://doi.org/10.15468/dl.zfaw79
Anopheles arabiensis	36.7847	-9.0738	Unclassified	GBIF: https://doi.org/10.15468/dl.zfaw79
Anopheles arabiensis	36.7848	-9.07434	Unclassified	GBIF: https://doi.org/10.15468/dl.zfaw79
Anopheles arabiensis	36.7849	-9.07358	Unclassified	GBIF: https://doi.org/10.15468/dl.zfaw79
Anopheles arabiensis	36.785	-8.92968	Unclassified	GBIF: https://doi.org/10.15468/dl.zfaw79
Anopheles arabiensis	36.7851	-9.07644	Unclassified	GBIF: https://doi.org/10.15468/dl.zfaw79
Anopheles arabiensis	36.7852	-9.0769	Unclassified	GBIF: https://doi.org/10.15468/dl.zfaw79
Anopheles arabiensis	36.7854	-9.07348	Unclassified	GBIF: https://doi.org/10.15468/dl.zfaw79
Anopheles arabiensis	36.7856	-9.07356	Unclassified	GBIF: https://doi.org/10.15468/dl.zfaw79
Anopheles arabiensis	36.7859	-9.07388	Unclassified	GBIF: https://doi.org/10.15468/dl.zfaw79
Anopheles arabiensis	36.7859	-9.07729	Unclassified	GBIF: https://doi.org/10.15468/dl.zfaw79
Anopheles arabiensis	36.7859	-9.07451	Unclassified	GBIF: https://doi.org/10.15468/dl.zfaw79
Anopheles arabiensis	36.7859	-9.0737	Unclassified	GBIF: https://doi.org/10.15468/dl.zfaw79
Anopheles arabiensis	36.786	-9.07408	Unclassified	GBIF: https://doi.org/10.15468/dl.zfaw79
Anopheles arabiensis	36.7866	-9.07505	Unclassified	GBIF: https://doi.org/10.15468/dl.zfaw79
Anopheles arabiensis	36.787	-9.07426	Unclassified	GBIF: https://doi.org/10.15468/dl.zfaw79
Anopheles arabiensis	36.7882	-9.07257	Unclassified	GBIF: https://doi.org/10.15468/dl.zfaw79
Anopheles arabiensis	36.7885	-9.07051	Unclassified	GBIF: https://doi.org/10.15468/dl.zfaw79
Anopheles arabiensis	36.7887	-9.07042	Unclassified	GBIF: https://doi.org/10.15468/dl.zfaw79
Anopheles arabiensis	36.789	-9.07032	Unclassified	GBIF: https://doi.org/10.15468/dl.zfaw79
Anopheles arabiensis	36.7929	-8.92785	Unclassified	GBIF: https://doi.org/10.15468/dl.zfaw79
Anopheles arabiensis	36.7929	-8.92721	Unclassified	GBIF: https://doi.org/10.15468/dl.zfaw79
Anopheles arabiensis	36.7932	-8.92747	Unclassified	GBIF: https://doi.org/10.15468/dl.zfaw79
Anopheles arabiensis	36.7932	-8.92722	Unclassified	GBIF: https://doi.org/10.15468/dl.zfaw79
Anopheles arabiensis	36.7933	-9.06546	Unclassified	GBIF: https://doi.org/10.15468/dl.zfaw79
Anopheles arabiensis	36.7934	-8.92707	Unclassified	GBIF: https://doi.org/10.15468/dl.zfaw79
Anopheles arabiensis	36.7934	-8.92783	Unclassified	GBIF: https://doi.org/10.15468/dl.zfaw79

Anopheles arabiensis	36.7935	-8.92746	Unclassified	GBIF: https://doi.org/10.15468/dl.zfaw79
Anopheles arabiensis	36.7936	-9.06553	Unclassified	GBIF: https://doi.org/10.15468/dl.zfaw79
Anopheles arabiensis	36.7936	-8.92743	Unclassified	GBIF: https://doi.org/10.15468/dl.zfaw79
Anopheles arabiensis	36.7937	-9.06571	Unclassified	GBIF: https://doi.org/10.15468/dl.zfaw79
Anopheles arabiensis	36.7937	-8.92736	Unclassified	GBIF: https://doi.org/10.15468/dl.zfaw79
Anopheles arabiensis	36.7938	-8.85932	Unclassified	GBIF: https://doi.org/10.15468/dl.zfaw79
Anopheles arabiensis	36.7939	-8.92751	Unclassified	GBIF: https://doi.org/10.15468/dl.zfaw79
Anopheles arabiensis	36.7939	-8.86088	Unclassified	GBIF: https://doi.org/10.15468/dl.zfaw79
Anopheles arabiensis	36.7942	-8.8591	Unclassified	GBIF: https://doi.org/10.15468/dl.zfaw79
Anopheles arabiensis	36.7943	-8.92795	Unclassified	GBIF: https://doi.org/10.15468/dl.zfaw79
Anopheles arabiensis	36.7944	-8.86067	Unclassified	GBIF: https://doi.org/10.15468/dl.zfaw79
Anopheles arabiensis	36.7944	-8.86049	Unclassified	GBIF: https://doi.org/10.15468/dl.zfaw79
Anopheles arabiensis	36.7944	-8.86068	Unclassified	GBIF: https://doi.org/10.15468/dl.zfaw79
Anopheles arabiensis	36.7945	-8.85907	Unclassified	GBIF: https://doi.org/10.15468/dl.zfaw79
Anopheles arabiensis	36.7945	-8.86044	Unclassified	GBIF: https://doi.org/10.15468/dl.zfaw79
Anopheles arabiensis	36.7945	-8.86145	Unclassified	GBIF: https://doi.org/10.15468/dl.zfaw79
Anopheles arabiensis	36.7946	-8.86239	Unclassified	GBIF: https://doi.org/10.15468/dl.zfaw79
Anopheles arabiensis	36.7947	-8.86247	Unclassified	GBIF: https://doi.org/10.15468/dl.zfaw79
Anopheles arabiensis	36.7948	-8.85937	Unclassified	GBIF: https://doi.org/10.15468/dl.zfaw79
Anopheles arabiensis	36.7948	-8.86027	Unclassified	GBIF: https://doi.org/10.15468/dl.zfaw79
Anopheles arabiensis	36.7949	-8.8596	Unclassified	GBIF: https://doi.org/10.15468/dl.zfaw79
Anopheles arabiensis	36.795	-8.85911	Unclassified	GBIF: https://doi.org/10.15468/dl.zfaw79
Anopheles arabiensis	36.7952	-8.86047	Unclassified	GBIF: https://doi.org/10.15468/dl.zfaw79
Anopheles arabiensis	36.7952	-8.92645	Unclassified	GBIF: https://doi.org/10.15468/dl.zfaw79
Anopheles arabiensis	36.7952	-8.86009	Unclassified	GBIF: https://doi.org/10.15468/dl.zfaw79
Anopheles arabiensis	36.7952	-8.92633	Unclassified	GBIF: https://doi.org/10.15468/dl.zfaw79
Anopheles arabiensis	36.7952	-8.92602	Unclassified	GBIF: https://doi.org/10.15468/dl.zfaw79
Anopheles arabiensis	36.7953	-8.85946	Unclassified	GBIF: https://doi.org/10.15468/dl.zfaw79
Anopheles arabiensis	36.7955	-8.8606	Unclassified	GBIF: https://doi.org/10.15468/dl.zfaw79
Anopheles arabiensis	36.7955	-8.86062	Unclassified	GBIF: https://doi.org/10.15468/dl.zfaw79
Anopheles arabiensis	36.7956	-8.92642	Unclassified	GBIF: https://doi.org/10.15468/dl.zfaw79
Anopheles arabiensis	36.7956	-8.85999	Unclassified	GBIF: https://doi.org/10.15468/dl.zfaw79
Anopheles arabiensis	36.7957	-8.92884	Unclassified	GBIF: https://doi.org/10.15468/dl.zfaw79
Anopheles arabiensis	36.7957	-8.92671	Unclassified	GBIF: https://doi.org/10.15468/dl.zfaw79
Anopheles arabiensis	36.7957	-8.92614	Unclassified	GBIF: https://doi.org/10.15468/dl.zfaw79
Anopheles arabiensis	36.7959	-8.92469	Unclassified	GBIF: https://doi.org/10.15468/dl.zfaw79
Anopheles arabiensis	36.7959	-8.9263	Unclassified	GBIF: https://doi.org/10.15468/dl.zfaw79
Anopheles arabiensis	36.7959	-8.92805	Unclassified	GBIF: https://doi.org/10.15468/dl.zfaw79
Anopheles arabiensis	36.796	-8.92727	Unclassified	GBIF: https://doi.org/10.15468/dl.zfaw79
Anopheles arabiensis	36.796	-8.92653	Unclassified	GBIF: https://doi.org/10.15468/dl.zfaw79
Anopheles arabiensis	36.7961	-8.85901	Unclassified	GBIF: https://doi.org/10.15468/dl.zfaw79
Anopheles arabiensis	36.7962	-8.92703	Unclassified	GBIF: https://doi.org/10.15468/dl.zfaw79

Anopheles arabiensis	36.7964	-8.92563	Unclassified	GBIF: https://doi.org/10.15468/dl.zfaw79
Anopheles arabiensis	36.7966	-8.92609	Unclassified	GBIF: https://doi.org/10.15468/dl.zfaw79
Anopheles arabiensis	36.7971	-8.8577	Unclassified	GBIF: https://doi.org/10.15468/dl.zfaw79
Anopheles arabiensis	36.7978	-8.85704	Unclassified	GBIF: https://doi.org/10.15468/dl.zfaw79
Anopheles arabiensis	36.7979	-8.92526	Unclassified	GBIF: https://doi.org/10.15468/dl.zfaw79
Anopheles arabiensis	36.7979	-8.85906	Unclassified	GBIF: https://doi.org/10.15468/dl.zfaw79
Anopheles arabiensis	36.798	-8.92528	Unclassified	GBIF: https://doi.org/10.15468/dl.zfaw79
Anopheles arabiensis	36.7981	-8.85908	Unclassified	GBIF: https://doi.org/10.15468/dl.zfaw79
Anopheles arabiensis	36.7981	-8.92494	Unclassified	GBIF: https://doi.org/10.15468/dl.zfaw79
Anopheles arabiensis	36.7981	-8.85903	Unclassified	GBIF: https://doi.org/10.15468/dl.zfaw79
Anopheles arabiensis	36.7982	-8.85949	Unclassified	GBIF: https://doi.org/10.15468/dl.zfaw79
Anopheles arabiensis	36.7983	-8.92521	Unclassified	GBIF: https://doi.org/10.15468/dl.zfaw79
Anopheles arabiensis	36.7986	-8.92577	Unclassified	GBIF: https://doi.org/10.15468/dl.zfaw79
Anopheles arabiensis	36.799	-8.92581	Unclassified	GBIF: https://doi.org/10.15468/dl.zfaw79
Anopheles arabiensis	36.7991	-8.85892	Unclassified	GBIF: https://doi.org/10.15468/dl.zfaw79
Anopheles arabiensis	36.7993	-8.85772	Unclassified	GBIF: https://doi.org/10.15468/dl.zfaw79
Anopheles arabiensis	36.7999	-8.85777	Unclassified	GBIF: https://doi.org/10.15468/dl.zfaw79
Anopheles arabiensis	36.8005	-8.85862	Unclassified	GBIF: https://doi.org/10.15468/dl.zfaw79
Anopheles arabiensis	36.8009	-8.85853	Unclassified	GBIF: https://doi.org/10.15468/dl.zfaw79
Anopheles arabiensis	36.801	-8.8584	Unclassified	GBIF: https://doi.org/10.15468/dl.zfaw79
Anopheles arabiensis	36.8207	-8.94651	Unclassified	GBIF: https://doi.org/10.15468/dl.zfaw79
Anopheles arabiensis	36.8228	-8.94604	Unclassified	GBIF: https://doi.org/10.15468/dl.zfaw79
Anopheles arabiensis	36.8238	-8.9465	Unclassified	GBIF: https://doi.org/10.15468/dl.zfaw79
Anopheles arabiensis	36.8238	-8.94484	Unclassified	GBIF: https://doi.org/10.15468/dl.zfaw79
Anopheles arabiensis	36.8241	-8.94541	Unclassified	GBIF: https://doi.org/10.15468/dl.zfaw79
Anopheles arabiensis	36.8245	-8.94524	Unclassified	GBIF: https://doi.org/10.15468/dl.zfaw79
Anopheles arabiensis	36.8246	-8.9449	Unclassified	GBIF: https://doi.org/10.15468/dl.zfaw79
Anopheles arabiensis	36.8246	-8.94519	Unclassified	GBIF: https://doi.org/10.15468/dl.zfaw79
Anopheles arabiensis	36.8277	-8.94283	Unclassified	GBIF: https://doi.org/10.15468/dl.zfaw79
Anopheles arabiensis	36.828	-8.94255	Unclassified	GBIF: https://doi.org/10.15468/dl.zfaw79
Anopheles arabiensis	36.8282	-8.94079	Unclassified	GBIF: https://doi.org/10.15468/dl.zfaw79
Anopheles arabiensis	36.8283	-8.94098	Unclassified	GBIF: https://doi.org/10.15468/dl.zfaw79
Anopheles arabiensis	36.8284	-8.94036	Unclassified	GBIF: https://doi.org/10.15468/dl.zfaw79
Anopheles arabiensis	36.8285	-8.94045	Unclassified	GBIF: https://doi.org/10.15468/dl.zfaw79
Anopheles arabiensis	36.8297	-8.94003	Unclassified	GBIF: https://doi.org/10.15468/dl.zfaw79
Anopheles arabiensis	36.8298	-9.05593	Unclassified	GBIF: https://doi.org/10.15468/dl.zfaw79
Anopheles arabiensis	36.83	-8.97644	Unclassified	GBIF: https://doi.org/10.15468/dl.zfaw79
Anopheles arabiensis	36.8301	-8.97724	Unclassified	GBIF: https://doi.org/10.15468/dl.zfaw79
Anopheles arabiensis	36.8301	-9.05644	Unclassified	GBIF: https://doi.org/10.15468/dl.zfaw79
Anopheles arabiensis	36.8303	-8.97838	Unclassified	GBIF: https://doi.org/10.15468/dl.zfaw79
Anopheles arabiensis	36.8303	-9.05621	Unclassified	GBIF: https://doi.org/10.15468/dl.zfaw79
Anopheles arabiensis	36.8304	-8.97848	Unclassified	GBIF: https://doi.org/10.15468/dl.zfaw79

Anopheles arabiensis	36.8304	-8.97823	Unclassified	GBIF: https://doi.org/10.15468/dl.zfaw79
Anopheles arabiensis	36.8304	-8.97701	Unclassified	GBIF: https://doi.org/10.15468/dl.zfaw79
Anopheles arabiensis	36.8304	-8.97853	Unclassified	GBIF: https://doi.org/10.15468/dl.zfaw79
Anopheles arabiensis	36.8304	-8.97886	Unclassified	GBIF: https://doi.org/10.15468/dl.zfaw79
Anopheles arabiensis	36.8305	-8.98102	Unclassified	GBIF: https://doi.org/10.15468/dl.zfaw79
Anopheles arabiensis	36.8305	-8.93661	Unclassified	GBIF: https://doi.org/10.15468/dl.zfaw79
Anopheles arabiensis	36.8305	-8.977	Unclassified	GBIF: https://doi.org/10.15468/dl.zfaw79
Anopheles arabiensis	36.8305	-8.93707	Unclassified	GBIF: https://doi.org/10.15468/dl.zfaw79
Anopheles arabiensis	36.8306	-8.93868	Unclassified	GBIF: https://doi.org/10.15468/dl.zfaw79
Anopheles arabiensis	36.8307	-8.93798	Unclassified	GBIF: https://doi.org/10.15468/dl.zfaw79
Anopheles arabiensis	36.8309	-8.93835	Unclassified	GBIF: https://doi.org/10.15468/dl.zfaw79
Anopheles arabiensis	36.8309	-8.93771	Unclassified	GBIF: https://doi.org/10.15468/dl.zfaw79
Anopheles arabiensis	36.8311	-8.93834	Unclassified	GBIF: https://doi.org/10.15468/dl.zfaw79
Anopheles arabiensis	36.8312	-8.98139	Unclassified	GBIF: https://doi.org/10.15468/dl.zfaw79
Anopheles arabiensis	36.8312	-9.05634	Unclassified	GBIF: https://doi.org/10.15468/dl.zfaw79
Anopheles arabiensis	36.8312	-9.05636	Unclassified	GBIF: https://doi.org/10.15468/dl.zfaw79
Anopheles arabiensis	36.8314	-9.05547	Unclassified	GBIF: https://doi.org/10.15468/dl.zfaw79
Anopheles arabiensis	36.8315	-8.97386	Unclassified	GBIF: https://doi.org/10.15468/dl.zfaw79
Anopheles arabiensis	36.8315	-8.97399	Unclassified	GBIF: https://doi.org/10.15468/dl.zfaw79
Anopheles arabiensis	36.8315	-9.05545	Unclassified	GBIF: https://doi.org/10.15468/dl.zfaw79
Anopheles arabiensis	36.8318	-8.97923	Unclassified	GBIF: https://doi.org/10.15468/dl.zfaw79
Anopheles arabiensis	36.8318	-8.97471	Unclassified	GBIF: https://doi.org/10.15468/dl.zfaw79
Anopheles arabiensis	36.8319	-8.96922	Unclassified	GBIF: https://doi.org/10.15468/dl.zfaw79
Anopheles arabiensis	36.8319	-9.05633	Unclassified	GBIF: https://doi.org/10.15468/dl.zfaw79
Anopheles arabiensis	36.832	-8.97227	Unclassified	GBIF: https://doi.org/10.15468/dl.zfaw79
Anopheles arabiensis	36.832	-8.95112	Unclassified	GBIF: https://doi.org/10.15468/dl.zfaw79
Anopheles arabiensis	36.832	-8.94806	Unclassified	GBIF: https://doi.org/10.15468/dl.zfaw79
Anopheles arabiensis	36.8321	-8.97456	Unclassified	GBIF: https://doi.org/10.15468/dl.zfaw79
Anopheles arabiensis	36.8321	-8.94788	Unclassified	GBIF: https://doi.org/10.15468/dl.zfaw79
Anopheles arabiensis	36.8321	-9.05573	Unclassified	GBIF: https://doi.org/10.15468/dl.zfaw79
Anopheles arabiensis	36.8322	-8.94797	Unclassified	GBIF: https://doi.org/10.15468/dl.zfaw79
Anopheles arabiensis	36.8322	-8.94498	Unclassified	GBIF: https://doi.org/10.15468/dl.zfaw79
Anopheles arabiensis	36.8322	-9.05489	Unclassified	GBIF: https://doi.org/10.15468/dl.zfaw79
Anopheles arabiensis	36.8323	-9.05496	Unclassified	GBIF: https://doi.org/10.15468/dl.zfaw79
Anopheles arabiensis	36.8324	-8.97219	Unclassified	GBIF: https://doi.org/10.15468/dl.zfaw79
Anopheles arabiensis	36.8324	-8.94741	Unclassified	GBIF: https://doi.org/10.15468/dl.zfaw79
Anopheles arabiensis	36.8324	-8.94414	Unclassified	GBIF: https://doi.org/10.15468/dl.zfaw79
Anopheles arabiensis	36.8326	-8.97997	Unclassified	GBIF: https://doi.org/10.15468/dl.zfaw79
Anopheles arabiensis	36.8326	-8.96994	Unclassified	GBIF: https://doi.org/10.15468/dl.zfaw79
Anopheles arabiensis	36.8327	-8.9469	Unclassified	GBIF: https://doi.org/10.15468/dl.zfaw79
Anopheles arabiensis	36.8327	-9.05633	Unclassified	GBIF: https://doi.org/10.15468/dl.zfaw79
Anopheles arabiensis	36.8328	-8.94812	Unclassified	GBIF: https://doi.org/10.15468/dl.zfaw79

Anopheles arabiensis	36.8329	-9.05728	Unclassified	GBIF: https://doi.org/10.15468/dl.zfaw79
Anopheles arabiensis	36.833	-9.05507	Unclassified	GBIF: https://doi.org/10.15468/dl.zfaw79
Anopheles arabiensis	36.833	-9.05463	Unclassified	GBIF: https://doi.org/10.15468/dl.zfaw79
Anopheles arabiensis	36.833	-9.05706	Unclassified	GBIF: https://doi.org/10.15468/dl.zfaw79
Anopheles arabiensis	36.8331	-8.9477	Unclassified	GBIF: https://doi.org/10.15468/dl.zfaw79
Anopheles arabiensis	36.8331	-9.05568	Unclassified	GBIF: https://doi.org/10.15468/dl.zfaw79
Anopheles arabiensis	36.8331	-8.98039	Unclassified	GBIF: https://doi.org/10.15468/dl.zfaw79
Anopheles arabiensis	36.8331	-9.0569	Unclassified	GBIF: https://doi.org/10.15468/dl.zfaw79
Anopheles arabiensis	36.8332	-9.05526	Unclassified	GBIF: https://doi.org/10.15468/dl.zfaw79
Anopheles arabiensis	36.8332	-9.05433	Unclassified	GBIF: https://doi.org/10.15468/dl.zfaw79
Anopheles arabiensis	36.8332	-9.05529	Unclassified	GBIF: https://doi.org/10.15468/dl.zfaw79
Anopheles arabiensis	36.8332	-9.05689	Unclassified	GBIF: https://doi.org/10.15468/dl.zfaw79
Anopheles arabiensis	36.8332	-9.05468	Unclassified	GBIF: https://doi.org/10.15468/dl.zfaw79
Anopheles arabiensis	36.8333	-9.05435	Unclassified	GBIF: https://doi.org/10.15468/dl.zfaw79
Anopheles arabiensis	36.8333	-9.05458	Unclassified	GBIF: https://doi.org/10.15468/dl.zfaw79
Anopheles arabiensis	36.8333	-8.97923	Unclassified	GBIF: https://doi.org/10.15468/dl.zfaw79
Anopheles arabiensis	36.8333	-9.05465	Unclassified	GBIF: https://doi.org/10.15468/dl.zfaw79
Anopheles arabiensis	36.8334	-9.05633	Unclassified	GBIF: https://doi.org/10.15468/dl.zfaw79
Anopheles arabiensis	36.8334	-9.05579	Unclassified	GBIF: https://doi.org/10.15468/dl.zfaw79
Anopheles arabiensis	36.8335	-9.05468	Unclassified	GBIF: https://doi.org/10.15468/dl.zfaw79
Anopheles arabiensis	36.8337	-9.05624	Unclassified	GBIF: https://doi.org/10.15468/dl.zfaw79
Anopheles arabiensis	36.8343	-8.9694	Unclassified	GBIF: https://doi.org/10.15468/dl.zfaw79
Anopheles arabiensis	36.8345	-8.97002	Unclassified	GBIF: https://doi.org/10.15468/dl.zfaw79
Anopheles arabiensis	36.8391	-9.00898	Unclassified	GBIF: https://doi.org/10.15468/dl.zfaw79
Anopheles arabiensis	36.8395	-9.00987	Unclassified	GBIF: https://doi.org/10.15468/dl.zfaw79
Anopheles arabiensis	36.8403	-9.0091	Unclassified	GBIF: https://doi.org/10.15468/dl.zfaw79
Anopheles arabiensis	36.8403	-9.00864	Unclassified	GBIF: https://doi.org/10.15468/dl.zfaw79
Anopheles arabiensis	36.8404	-9.0097	Unclassified	GBIF: https://doi.org/10.15468/dl.zfaw79
Anopheles arabiensis	36.8408	-9.01154	Unclassified	GBIF: https://doi.org/10.15468/dl.zfaw79
Anopheles arabiensis	36.8409	-9.01049	Unclassified	GBIF: https://doi.org/10.15468/dl.zfaw79
Anopheles arabiensis	36.8409	-9.00861	Unclassified	GBIF: https://doi.org/10.15468/dl.zfaw79
Anopheles arabiensis	36.8411	-9.01087	Unclassified	GBIF: https://doi.org/10.15468/dl.zfaw79
Anopheles arabiensis	36.8415	-9.00913	Unclassified	GBIF: https://doi.org/10.15468/dl.zfaw79
Anopheles arabiensis	36.8415	-9.01008	Unclassified	GBIF: https://doi.org/10.15468/dl.zfaw79
Anopheles arabiensis	36.8417	-9.00938	Unclassified	GBIF: https://doi.org/10.15468/dl.zfaw79
Anopheles arabiensis	36.8418	-9.01017	Unclassified	GBIF: https://doi.org/10.15468/dl.zfaw79
Anopheles arabiensis	36.842	-9.01096	Unclassified	GBIF: https://doi.org/10.15468/dl.zfaw79
Anopheles arabiensis	36.8421	-9.01353	Unclassified	GBIF: https://doi.org/10.15468/dl.zfaw79
Anopheles arabiensis	36.8424	-9.0113	Unclassified	GBIF: https://doi.org/10.15468/dl.zfaw79
Anopheles arabiensis	36.8424	-9.01108	Unclassified	GBIF: https://doi.org/10.15468/dl.zfaw79
Anopheles arabiensis	36.8425	-9.0116	Unclassified	GBIF: https://doi.org/10.15468/dl.zfaw79
Anopheles arabiensis	36.8425	-9.01315	Unclassified	GBIF: https://doi.org/10.15468/dl.zfaw79

Anopheles arabiensis	36.8426	-9.01142	Unclassified	GBIF: https://doi.org/10.15468/dl.zfaw79
Anopheles arabiensis	36.8427	-9.01066	Unclassified	GBIF: https://doi.org/10.15468/dl.zfaw79
Anopheles arabiensis	36.8428	-9.01075	Unclassified	GBIF: https://doi.org/10.15468/dl.zfaw79
Anopheles arabiensis	36.843	-9.01287	Unclassified	GBIF: https://doi.org/10.15468/dl.zfaw79
Anopheles arabiensis	36.843	-9.01133	Unclassified	GBIF: https://doi.org/10.15468/dl.zfaw79
Anopheles arabiensis	36.8432	-9.011	Unclassified	GBIF: https://doi.org/10.15468/dl.zfaw79
Anopheles arabiensis	36.8432	-9.01382	Unclassified	GBIF: https://doi.org/10.15468/dl.zfaw79
Anopheles arabiensis	36.8432	-9.01347	Unclassified	GBIF: https://doi.org/10.15468/dl.zfaw79
Anopheles arabiensis	36.8432	-9.01217	Unclassified	GBIF: https://doi.org/10.15468/dl.zfaw79
Anopheles arabiensis	36.8433	-9.00995	Unclassified	GBIF: https://doi.org/10.15468/dl.zfaw79
Anopheles arabiensis	36.8433	-9.01365	Unclassified	GBIF: https://doi.org/10.15468/dl.zfaw79
Anopheles arabiensis	36.8434	-9.01093	Unclassified	GBIF: https://doi.org/10.15468/dl.zfaw79
Anopheles arabiensis	36.8434	-9.01277	Unclassified	GBIF: https://doi.org/10.15468/dl.zfaw79
Anopheles arabiensis	36.8435	-9.01364	Unclassified	GBIF: https://doi.org/10.15468/dl.zfaw79
Anopheles arabiensis	36.8436	-9.01295	Unclassified	GBIF: https://doi.org/10.15468/dl.zfaw79
Anopheles arabiensis	36.8438	-9.01186	Unclassified	GBIF: https://doi.org/10.15468/dl.zfaw79
Anopheles arabiensis	36.8439	-9.01133	Unclassified	GBIF: https://doi.org/10.15468/dl.zfaw79
Anopheles arabiensis	36.8439	-9.01086	Unclassified	GBIF: https://doi.org/10.15468/dl.zfaw79
Anopheles arabiensis	36.844	-9.01083	Unclassified	GBIF: https://doi.org/10.15468/dl.zfaw79
Anopheles arabiensis	36.844	-9.01039	Unclassified	GBIF: https://doi.org/10.15468/dl.zfaw79
Anopheles arabiensis	36.8442	-9.01051	Unclassified	GBIF: https://doi.org/10.15468/dl.zfaw79
Anopheles arabiensis	36.8442	-9.01055	Unclassified	GBIF: https://doi.org/10.15468/dl.zfaw79
Anopheles arabiensis	36.8443	-9.01071	Unclassified	GBIF: https://doi.org/10.15468/dl.zfaw79
Anopheles arabiensis	36.8445	-9.00941	Unclassified	GBIF: https://doi.org/10.15468/dl.zfaw79
Anopheles arabiensis	36.8445	-9.00996	Unclassified	GBIF: https://doi.org/10.15468/dl.zfaw79
Anopheles arabiensis	36.8446	-9.00973	Unclassified	GBIF: https://doi.org/10.15468/dl.zfaw79
Anopheles arabiensis	36.8446	-9.00318	Unclassified	GBIF: https://doi.org/10.15468/dl.zfaw79
Anopheles arabiensis	36.8447	-9.01019	Unclassified	GBIF: https://doi.org/10.15468/dl.zfaw79
Anopheles arabiensis	36.8448	-9.00997	Unclassified	GBIF: https://doi.org/10.15468/dl.zfaw79
Anopheles arabiensis	36.8449	-9.01038	Unclassified	GBIF: https://doi.org/10.15468/dl.zfaw79
Anopheles arabiensis	36.845	-9.0096	Unclassified	GBIF: https://doi.org/10.15468/dl.zfaw79
Anopheles arabiensis	36.845	-9.00961	Unclassified	GBIF: https://doi.org/10.15468/dl.zfaw79
Anopheles arabiensis	36.8451	-9.00894	Unclassified	GBIF: https://doi.org/10.15468/dl.zfaw79
Anopheles arabiensis	36.8452	-9.00895	Unclassified	GBIF: https://doi.org/10.15468/dl.zfaw79
Anopheles arabiensis	36.8454	-9.00909	Unclassified	GBIF: https://doi.org/10.15468/dl.zfaw79
Anopheles arabiensis	36.8454	-9.00923	Unclassified	GBIF: https://doi.org/10.15468/dl.zfaw79
Anopheles arabiensis	36.8456	-9.00863	Unclassified	GBIF: https://doi.org/10.15468/dl.zfaw79
Anopheles arabiensis	36.8461	-9.00832	Unclassified	GBIF: https://doi.org/10.15468/dl.zfaw79
Anopheles arabiensis	36.9	0.37	Unclassified	GBIF: https://doi.org/10.15468/dl.zfaw79
Anopheles arabiensis	37	7.13333	Unclassified	GBIF: https://doi.org/10.15468/dl.zfaw79
Anopheles arabiensis	37	9.1	Unclassified	GBIF: https://doi.org/10.15468/dl.zfaw79
Anopheles arabiensis	37.3	-0.66667	Unclassified	GBIF: https://doi.org/10.15468/dl.zfaw79

Anopheles arabiensis	37.58333	8.21667	Unclassified	GBIF: https://doi.org/10.15468/dl.zfaw79
Anopheles arabiensis	37.66667	-3.38333	Unclassified	GBIF: https://doi.org/10.15468/dl.zfaw79
Anopheles arabiensis	37.68333	-0.5	Unclassified	GBIF: https://doi.org/10.15468/dl.zfaw79
Anopheles arabiensis	37.7	6.15	Unclassified	GBIF: https://doi.org/10.15468/dl.zfaw79
Anopheles arabiensis	37.93333	6.63333	Unclassified	GBIF: https://doi.org/10.15468/dl.zfaw79
Anopheles arabiensis	38.01667	-4.66667	Unclassified	GBIF: https://doi.org/10.15468/dl.zfaw79
Anopheles arabiensis	38.01667	-5.08333	Unclassified	GBIF: https://doi.org/10.15468/dl.zfaw79
Anopheles arabiensis	38.03333	-5.41667	Unclassified	GBIF: https://doi.org/10.15468/dl.zfaw79
Anopheles arabiensis	38.06667	-4.38333	Unclassified	GBIF: https://doi.org/10.15468/dl.zfaw79
Anopheles arabiensis	38.08333	10.03333	Unclassified	GBIF: https://doi.org/10.15468/dl.zfaw79
Anopheles arabiensis	38.08333	-4.65	Unclassified	GBIF: https://doi.org/10.15468/dl.zfaw79
Anopheles arabiensis	38.2	10.1	Unclassified	GBIF: https://doi.org/10.15468/dl.zfaw79
Anopheles arabiensis	38.28333	-4.88333	Unclassified	GBIF: https://doi.org/10.15468/dl.zfaw79
Anopheles arabiensis	38.41667	-5.16667	Unclassified	GBIF: https://doi.org/10.15468/dl.zfaw79
Anopheles arabiensis	38.55	-5.08333	Unclassified	GBIF: https://doi.org/10.15468/dl.zfaw79
Anopheles arabiensis	38.55	-5.3	Unclassified	GBIF: https://doi.org/10.15468/dl.zfaw79
Anopheles arabiensis	38.55	-4.3	Unclassified	GBIF: https://doi.org/10.15468/dl.zfaw79
Anopheles arabiensis	38.55	-5.16667	Unclassified	GBIF: https://doi.org/10.15468/dl.zfaw79
Anopheles arabiensis	38.66667	7.61667	Unclassified	GBIF: https://doi.org/10.15468/dl.zfaw79
Anopheles arabiensis	38.76667	-5.16667	Unclassified	GBIF: https://doi.org/10.15468/dl.zfaw79
Anopheles arabiensis	38.82	8.15	Unclassified	GBIF: https://doi.org/10.15468/dl.zfaw79
Anopheles arabiensis	38.83333	-5.18333	Unclassified	GBIF: https://doi.org/10.15468/dl.zfaw79
Anopheles arabiensis	38.91667	-6.43333	Unclassified	GBIF: https://doi.org/10.15468/dl.zfaw79
Anopheles arabiensis	39.08333	-5.11667	Unclassified	GBIF: https://doi.org/10.15468/dl.zfaw79
Anopheles arabiensis	39.1	8.45	Unclassified	GBIF: https://doi.org/10.15468/dl.zfaw79
Anopheles arabiensis	39.15	-4.86667	Unclassified	GBIF: https://doi.org/10.15468/dl.zfaw79
Anopheles arabiensis	39.2	-6.16667	Unclassified	GBIF: https://doi.org/10.15468/dl.zfaw79
Anopheles arabiensis	39.2	8.43333	Unclassified	GBIF: https://doi.org/10.15468/dl.zfaw79
Anopheles arabiensis	39.2	8.5	Unclassified	GBIF: https://doi.org/10.15468/dl.zfaw79
Anopheles arabiensis	39.21667	-4.6	Unclassified	GBIF: https://doi.org/10.15468/dl.zfaw79
Anopheles arabiensis	39.25	8.41667	Unclassified	GBIF: https://doi.org/10.15468/dl.zfaw79
Anopheles arabiensis	39.33333	8.43333	Unclassified	GBIF: https://doi.org/10.15468/dl.zfaw79
Anopheles arabiensis	39.47	-4.17	Unclassified	GBIF: https://doi.org/10.15468/dl.zfaw79
Anopheles arabiensis	39.5	8.5	Unclassified	GBIF: https://doi.org/10.15468/dl.zfaw79
Anopheles arabiensis	39.63333	-1.25	Unclassified	GBIF: https://doi.org/10.15468/dl.zfaw79
Anopheles arabiensis	39.75	-5	Unclassified	GBIF: https://doi.org/10.15468/dl.zfaw79
Anopheles arabiensis	39.83333	-3.61667	Unclassified	GBIF: https://doi.org/10.15468/dl.zfaw79
Anopheles arabiensis	39.91	-3.89	Unclassified	GBIF: https://doi.org/10.15468/dl.zfaw79
Anopheles arabiensis	39.91667	9.05	Unclassified	GBIF: https://doi.org/10.15468/dl.zfaw79
Anopheles arabiensis	40	9.01667	Unclassified	GBIF: https://doi.org/10.15468/dl.zfaw79
Anopheles arabiensis	40	-3.1	Unclassified	GBIF: https://doi.org/10.15468/dl.zfaw79
Anopheles arabiensis	40.11	-3.25	Unclassified	GBIF: https://doi.org/10.15468/dl.zfaw79

Anopheles arabiensis	40.2	9.2	Unclassified	GBIF: https://doi.org/10.15468/dl.zfaw79
Anopheles arabiensis	40.53333	10.46667	Unclassified	GBIF: https://doi.org/10.15468/dl.zfaw79
Anopheles arabiensis	41.25	11.5	Unclassified	GBIF: https://doi.org/10.15468/dl.zfaw79
Anopheles arabiensis	42.1	9.3	Unclassified	GBIF: https://doi.org/10.15468/dl.zfaw79
Anopheles arabiensis	42.18	9.28	Unclassified	GBIF: https://doi.org/10.15468/dl.zfaw79
Anopheles arabiensis	42.75	17.25	Unclassified	GBIF: https://doi.org/10.15468/dl.zfaw79
Anopheles arabiensis	43.28333	13.75	Unclassified	GBIF: https://doi.org/10.15468/dl.zfaw79
Anopheles arabiensis	43.5	-21.7	Unclassified	GBIF: https://doi.org/10.15468/dl.zfaw79
Anopheles arabiensis	43.68333	-23.33333	Unclassified	GBIF: https://doi.org/10.15468/dl.zfaw79
Anopheles arabiensis	43.75	-21.73333	Unclassified	GBIF: https://doi.org/10.15468/dl.zfaw79
Anopheles arabiensis	44.05	-18.01667	Unclassified	GBIF: https://doi.org/10.15468/dl.zfaw79
Anopheles arabiensis	44.28333	-20.31667	Unclassified	GBIF: https://doi.org/10.15468/dl.zfaw79
Anopheles arabiensis	44.33333	-23.5	Unclassified	GBIF: https://doi.org/10.15468/dl.zfaw79
Anopheles arabiensis	44.33333	-22.88333	Unclassified	GBIF: https://doi.org/10.15468/dl.zfaw79
Anopheles arabiensis	44.33333	-21.43333	Unclassified	GBIF: https://doi.org/10.15468/dl.zfaw79
Anopheles arabiensis	44.48333	-16.71667	Unclassified	GBIF: https://doi.org/10.15468/dl.zfaw79
Anopheles arabiensis	44.5	-22.5	Unclassified	GBIF: https://doi.org/10.15468/dl.zfaw79
Anopheles arabiensis	44.5	-19.66667	Unclassified	GBIF: https://doi.org/10.15468/dl.zfaw79
Anopheles arabiensis	44.51667	-22.9	Unclassified	GBIF: https://doi.org/10.15468/dl.zfaw79
Anopheles arabiensis	44.61667	-18.66667	Unclassified	GBIF: https://doi.org/10.15468/dl.zfaw79
Anopheles arabiensis	44.75	-24.66667	Unclassified	GBIF: https://doi.org/10.15468/dl.zfaw79
Anopheles arabiensis	44.78333	-19.11667	Unclassified	GBIF: https://doi.org/10.15468/dl.zfaw79
Anopheles arabiensis	44.9	-17.81667	Unclassified	GBIF: https://doi.org/10.15468/dl.zfaw79
Anopheles arabiensis	44.93333	-21.03333	Unclassified	GBIF: https://doi.org/10.15468/dl.zfaw79
Anopheles arabiensis	44.96667	-19.75	Unclassified	GBIF: https://doi.org/10.15468/dl.zfaw79
Anopheles arabiensis	45	13	Unclassified	GBIF: https://doi.org/10.15468/dl.zfaw79
Anopheles arabiensis	45.03333	-20.41667	Unclassified	GBIF: https://doi.org/10.15468/dl.zfaw79
Anopheles arabiensis	45.33333	-24.2	Unclassified	GBIF: https://doi.org/10.15468/dl.zfaw79
Anopheles arabiensis	45.35	2.03333	Unclassified	GBIF: https://doi.org/10.15468/dl.zfaw79
Anopheles arabiensis	45.45	-22.53333	Unclassified	GBIF: https://doi.org/10.15468/dl.zfaw79
Anopheles arabiensis	45.48333	-19.51667	Unclassified	GBIF: https://doi.org/10.15468/dl.zfaw79
Anopheles arabiensis	45.58333	-20.33333	Unclassified	GBIF: https://doi.org/10.15468/dl.zfaw79
Anopheles arabiensis	45.63333	13.33333	Unclassified	GBIF: https://doi.org/10.15468/dl.zfaw79
Anopheles arabiensis	45.65	-17.66667	Unclassified	GBIF: https://doi.org/10.15468/dl.zfaw79
Anopheles arabiensis	45.76667	-23.73333	Unclassified	GBIF: https://doi.org/10.15468/dl.zfaw79
Anopheles arabiensis	46.03333	-18.73333	Unclassified	GBIF: https://doi.org/10.15468/dl.zfaw79
Anopheles arabiensis	46.11667	-23.25	Unclassified	GBIF: https://doi.org/10.15468/dl.zfaw79
Anopheles arabiensis	46.15	-22.38333	Unclassified	GBIF: https://doi.org/10.15468/dl.zfaw79
Anopheles arabiensis	46.28333	-19.55	Unclassified	GBIF: https://doi.org/10.15468/dl.zfaw79
Anopheles arabiensis	46.33333	-15.66667	Unclassified	GBIF: https://doi.org/10.15468/dl.zfaw79
Anopheles arabiensis	46.41667	-24.91667	Unclassified	GBIF: https://doi.org/10.15468/dl.zfaw79
Anopheles arabiensis	46.48333	-19	Unclassified	GBIF: https://doi.org/10.15468/dl.zfaw79

Anopheles arabiensis	46.5	-25.01667	Unclassified	GBIF: https://doi.org/10.15468/dl.zfaw79
Anopheles arabiensis	46.5	-15.6	Unclassified	GBIF: https://doi.org/10.15468/dl.zfaw79
Anopheles arabiensis	46.66667	-16.08333	Unclassified	GBIF: https://doi.org/10.15468/dl.zfaw79
Anopheles arabiensis	46.71667	-16.43333	Unclassified	GBIF: https://doi.org/10.15468/dl.zfaw79
Anopheles arabiensis	46.71667	-19.16667	Unclassified	GBIF: https://doi.org/10.15468/dl.zfaw79
Anopheles arabiensis	46.75	-18.86667	Unclassified	GBIF: https://doi.org/10.15468/dl.zfaw79
Anopheles arabiensis	46.83333	-19.11667	Unclassified	GBIF: https://doi.org/10.15468/dl.zfaw79
Anopheles arabiensis	46.83333	-16.95	Unclassified	GBIF: https://doi.org/10.15468/dl.zfaw79
Anopheles arabiensis	46.83333	-19.83333	Unclassified	GBIF: https://doi.org/10.15468/dl.zfaw79
Anopheles arabiensis	46.85	-18.95	Unclassified	GBIF: https://doi.org/10.15468/dl.zfaw79
Anopheles arabiensis	46.91667	-17.55	Unclassified	GBIF: https://doi.org/10.15468/dl.zfaw79
Anopheles arabiensis	46.95	-17.3	Unclassified	GBIF: https://doi.org/10.15468/dl.zfaw79
Anopheles arabiensis	46.966667	-16.133333	Unclassified	GBIF: https://doi.org/10.15468/dl.zfaw79
Anopheles arabiensis	47.01667	-23.58333	Unclassified	GBIF: https://doi.org/10.15468/dl.zfaw79
Anopheles arabiensis	47.03333	-17.96667	Unclassified	GBIF: https://doi.org/10.15468/dl.zfaw79
Anopheles arabiensis	47.05	-16.33333	Unclassified	GBIF: https://doi.org/10.15468/dl.zfaw79
Anopheles arabiensis	47.08333	-21.45	Unclassified	GBIF: https://doi.org/10.15468/dl.zfaw79
Anopheles arabiensis	47.1	-18.2	Unclassified	GBIF: https://doi.org/10.15468/dl.zfaw79
Anopheles arabiensis	47.11667	-18.33333	Unclassified	GBIF: https://doi.org/10.15468/dl.zfaw79
Anopheles arabiensis	47.11667	-18.31667	Unclassified	GBIF: https://doi.org/10.15468/dl.zfaw79
Anopheles arabiensis	47.15	-18.68333	Unclassified	GBIF: https://doi.org/10.15468/dl.zfaw79
Anopheles arabiensis	47.21667	-21.11667	Unclassified	GBIF: https://doi.org/10.15468/dl.zfaw79
Anopheles arabiensis	47.33333	-22.83333	Unclassified	GBIF: https://doi.org/10.15468/dl.zfaw79
Anopheles arabiensis	47.33333	-19.08333	Unclassified	GBIF: https://doi.org/10.15468/dl.zfaw79
Anopheles arabiensis	47.43333	-18.8	Unclassified	GBIF: https://doi.org/10.15468/dl.zfaw79
Anopheles arabiensis	47.43333	-21.55	Unclassified	GBIF: https://doi.org/10.15468/dl.zfaw79
Anopheles arabiensis	47.45	-21.86667	Unclassified	GBIF: https://doi.org/10.15468/dl.zfaw79
Anopheles arabiensis	47.46667	-22.8	Unclassified	GBIF: https://doi.org/10.15468/dl.zfaw79
Anopheles arabiensis	47.5	-18.86667	Unclassified	GBIF: https://doi.org/10.15468/dl.zfaw79
Anopheles arabiensis	47.58333	-18.95	Unclassified	GBIF: https://doi.org/10.15468/dl.zfaw79
Anopheles arabiensis	47.58333	-23.35	Unclassified	GBIF: https://doi.org/10.15468/dl.zfaw79
Anopheles arabiensis	47.61667	-18.91667	Unclassified	GBIF: https://doi.org/10.15468/dl.zfaw79
Anopheles arabiensis	47.65	-21.31667	Unclassified	GBIF: https://doi.org/10.15468/dl.zfaw79
Anopheles arabiensis	47.65	-16.05	Unclassified	GBIF: https://doi.org/10.15468/dl.zfaw79
Anopheles arabiensis	47.66667	-15.51667	Unclassified	GBIF: https://doi.org/10.15468/dl.zfaw79
Anopheles arabiensis	47.68333	-18.96667	Unclassified	GBIF: https://doi.org/10.15468/dl.zfaw79
Anopheles arabiensis	47.68333	-18.7	Unclassified	GBIF: https://doi.org/10.15468/dl.zfaw79
Anopheles arabiensis	47.83333	-14.63333	Unclassified	GBIF: https://doi.org/10.15468/dl.zfaw79
Anopheles arabiensis	47.83333	-19.26667	Unclassified	GBIF: https://doi.org/10.15468/dl.zfaw79
Anopheles arabiensis	47.83333	-22.83333	Unclassified	GBIF: https://doi.org/10.15468/dl.zfaw79
Anopheles arabiensis	47.85	-18.85	Unclassified	GBIF: https://doi.org/10.15468/dl.zfaw79
Anopheles arabiensis	47.86667	-18.18333	Unclassified	GBIF: https://doi.org/10.15468/dl.zfaw79

Anopheles arabiensis	47.86667	-18.58333	Unclassified	GBIF: https://doi.org/10.15468/dl.zfaw79
Anopheles arabiensis	47.98333	-18.85	Unclassified	GBIF: https://doi.org/10.15468/dl.zfaw79
Anopheles arabiensis	48	-14.83333	Unclassified	GBIF: https://doi.org/10.15468/dl.zfaw79
Anopheles arabiensis	48	-18.91667	Unclassified	GBIF: https://doi.org/10.15468/dl.zfaw79
Anopheles arabiensis	48.01667	-18.81667	Unclassified	GBIF: https://doi.org/10.15468/dl.zfaw79
Anopheles arabiensis	48.01667	-19.15	Unclassified	GBIF: https://doi.org/10.15468/dl.zfaw79
Anopheles arabiensis	48.05	-19.13333	Unclassified	GBIF: https://doi.org/10.15468/dl.zfaw79
Anopheles arabiensis	48.05	-18.91667	Unclassified	GBIF: https://doi.org/10.15468/dl.zfaw79
Anopheles arabiensis	48.1	-19.08333	Unclassified	GBIF: https://doi.org/10.15468/dl.zfaw79
Anopheles arabiensis	48.1	-14.16667	Unclassified	GBIF: https://doi.org/10.15468/dl.zfaw79
Anopheles arabiensis	48.1	-19.03333	Unclassified	GBIF: https://doi.org/10.15468/dl.zfaw79
Anopheles arabiensis	48.11667	-19.01667	Unclassified	GBIF: https://doi.org/10.15468/dl.zfaw79
Anopheles arabiensis	48.1667	-19.3667	Unclassified	GBIF: https://doi.org/10.15468/dl.zfaw79
Anopheles arabiensis	48.2	-19.06667	Unclassified	GBIF: https://doi.org/10.15468/dl.zfaw79
Anopheles arabiensis	48.2	-19.36667	Unclassified	GBIF: https://doi.org/10.15468/dl.zfaw79
Anopheles arabiensis	48.21667	-13.35	Unclassified	GBIF: https://doi.org/10.15468/dl.zfaw79
Anopheles arabiensis	48.23333	-18.18333	Unclassified	GBIF: https://doi.org/10.15468/dl.zfaw79
Anopheles arabiensis	48.25	-18.78333	Unclassified	GBIF: https://doi.org/10.15468/dl.zfaw79
Anopheles arabiensis	48.28333	-18.18333	Unclassified	GBIF: https://doi.org/10.15468/dl.zfaw79
Anopheles arabiensis	48.33333	-19	Unclassified	GBIF: https://doi.org/10.15468/dl.zfaw79
Anopheles arabiensis	48.35	-18.96667	Unclassified	GBIF: https://doi.org/10.15468/dl.zfaw79
Anopheles arabiensis	48.43333	-19.16667	Unclassified	GBIF: https://doi.org/10.15468/dl.zfaw79
Anopheles arabiensis	48.46667	-17.81667	Unclassified	GBIF: https://doi.org/10.15468/dl.zfaw79
Anopheles arabiensis	48.46667	-18.9	Unclassified	GBIF: https://doi.org/10.15468/dl.zfaw79
Anopheles arabiensis	48.53333	-18.91667	Unclassified	GBIF: https://doi.org/10.15468/dl.zfaw79
Anopheles arabiensis	48.55	-15.23333	Unclassified	GBIF: https://doi.org/10.15468/dl.zfaw79
Anopheles arabiensis	48.6	-18.98333	Unclassified	GBIF: https://doi.org/10.15468/dl.zfaw79
Anopheles arabiensis	48.63333	-14.05	Unclassified	GBIF: https://doi.org/10.15468/dl.zfaw79
Anopheles arabiensis	48.75	-18.96667	Unclassified	GBIF: https://doi.org/10.15468/dl.zfaw79
Anopheles arabiensis	48.83333	-18.91667	Unclassified	GBIF: https://doi.org/10.15468/dl.zfaw79
Anopheles arabiensis	48.85	-18.86667	Unclassified	GBIF: https://doi.org/10.15468/dl.zfaw79
Anopheles arabiensis	48.85	-18.9	Unclassified	GBIF: https://doi.org/10.15468/dl.zfaw79
Anopheles arabiensis	48.85	-18.88333	Unclassified	GBIF: https://doi.org/10.15468/dl.zfaw79
Anopheles arabiensis	48.85	-18.91667	Unclassified	GBIF: https://doi.org/10.15468/dl.zfaw79
Anopheles arabiensis	49.05	-13.16667	Unclassified	GBIF: https://doi.org/10.15468/dl.zfaw79
Anopheles arabiensis	49.18333	-17.41667	Unclassified	GBIF: https://doi.org/10.15468/dl.zfaw79
Anopheles arabiensis	49.2	-12.7	Unclassified	GBIF: https://doi.org/10.15468/dl.zfaw79
Anopheles arabiensis	49.28333	-12.31667	Unclassified	GBIF: https://doi.org/10.15468/dl.zfaw79
Anopheles arabiensis	49.31667	-18.05	Unclassified	GBIF: https://doi.org/10.15468/dl.zfaw79
Anopheles arabiensis	49.38333	-18.16667	Unclassified	GBIF: https://doi.org/10.15468/dl.zfaw79
Anopheles arabiensis	49.41667	-17.2	Unclassified	GBIF: https://doi.org/10.15468/dl.zfaw79
Anopheles arabiensis	49.42	-17.81	Unclassified	GBIF: https://doi.org/10.15468/dl.zfaw79

Anopheles arabiensis	49.58333	-16.88333	Unclassified	GBIF: https://doi.org/10.15468/dl.zfaw79
Anopheles arabiensis	49.66667	-14.65	Unclassified	GBIF: https://doi.org/10.15468/dl.zfaw79
Anopheles arabiensis	49.76667	-16.16667	Unclassified	GBIF: https://doi.org/10.15468/dl.zfaw79
Anopheles arabiensis	50.26667	-14.88333	Unclassified	GBIF: https://doi.org/10.15468/dl.zfaw79
Anopheles arabiensis	55.36667	-21	Unclassified	GBIF: https://doi.org/10.15468/dl.zfaw79
Anopheles arabiensis	55.53241	-21.13317	Unclassified	GBIF: https://doi.org/10.15468/dl.zfaw79
Anopheles arabiensis	57.5	-20.25	Unclassified	GBIF: https://doi.org/10.15468/dl.zfaw79
Anopheles ardensis	28.92	-28.77	Unclassified	GBIF: https://doi.org/10.15468/dl.zfaw79
Anopheles ardensis	30.85	-29.766667	Unclassified	GBIF: https://doi.org/10.15468/dl.zfaw79
Anopheles argenteolobatus	28.1825	-25.671111	Unclassified	GBIF: https://doi.org/10.15468/dl.zfaw79
Anopheles argyritarsis	-104.893889	21.516111	Unclassified	GBIF: https://doi.org/10.15468/dl.zfaw79
Anopheles argyritarsis	-102.050833	19.1575	Unclassified	GBIF: https://doi.org/10.15468/dl.zfaw79
Anopheles argyritarsis	-99.500833	17.551389	Unclassified	GBIF: https://doi.org/10.15468/dl.zfaw79
Anopheles argyritarsis	-99.50031	17.55075	Unclassified	GBIF: https://doi.org/10.15468/dl.zfaw79
Anopheles argyritarsis	-97.180833	18.803333	Unclassified	GBIF: https://doi.org/10.15468/dl.zfaw79
Anopheles argyritarsis	-97.010833	20.1175	Unclassified	GBIF: https://doi.org/10.15468/dl.zfaw79
Anopheles argyritarsis	-96.997778	18.896944	Unclassified	GBIF: https://doi.org/10.15468/dl.zfaw79
Anopheles argyritarsis	-96.979167	18.85	Unclassified	GBIF: https://doi.org/10.15468/dl.zfaw79
Anopheles argyritarsis	-96.941944	18.914444	Unclassified	GBIF: https://doi.org/10.15468/dl.zfaw79
Anopheles argyritarsis	-96.931667	18.891111	Unclassified	GBIF: https://doi.org/10.15468/dl.zfaw79
Anopheles argyritarsis	-96.9275	19.54	Unclassified	GBIF: https://doi.org/10.15468/dl.zfaw79
Anopheles argyritarsis	-96.9152792	18.8471579	Unclassified	GBIF: https://doi.org/10.15468/dl.zfaw79
Anopheles argyritarsis	-96.914444	18.844167	Unclassified	GBIF: https://doi.org/10.15468/dl.zfaw79
Anopheles argyritarsis	-96.854167	19.927222	Unclassified	GBIF: https://doi.org/10.15468/dl.zfaw79
Anopheles argyritarsis	-96.849167	20.168889	Unclassified	GBIF: https://doi.org/10.15468/dl.zfaw79
Anopheles argyritarsis	-96.340833	17.465278	Unclassified	GBIF: https://doi.org/10.15468/dl.zfaw79
Anopheles argyritarsis	-94.872778	18.2325	Unclassified	GBIF: https://doi.org/10.15468/dl.zfaw79
Anopheles argyritarsis	-94.674167	18.023889	Unclassified	GBIF: https://doi.org/10.15468/dl.zfaw79
Anopheles argyritarsis	-93.784472	16.10775	Unclassified	GBIF: https://doi.org/10.15468/dl.zfaw79
Anopheles argyritarsis	-93.755	16.086389	Unclassified	GBIF: https://doi.org/10.15468/dl.zfaw79
Anopheles argyritarsis	-92.952778	17.548056	Unclassified	GBIF: https://doi.org/10.15468/dl.zfaw79
Anopheles argyritarsis	-92.918889	17.581389	Unclassified	GBIF: https://doi.org/10.15468/dl.zfaw79
Anopheles argyritarsis	-92.825555	17.5925	Unclassified	GBIF: https://doi.org/10.15468/dl.zfaw79
Anopheles argyritarsis	-92.7775	17.460278	Unclassified	GBIF: https://doi.org/10.15468/dl.zfaw79
Anopheles argyritarsis	-92.7277968	15.6629297	Unclassified	GBIF: https://doi.org/10.15468/dl.zfaw79
Anopheles argyritarsis	-92.260555	14.908056	Unclassified	GBIF: https://doi.org/10.15468/dl.zfaw79
Anopheles argyritarsis	-92.17194	15.3925	Unclassified	GBIF: https://doi.org/10.15468/dl.zfaw79
Anopheles argyritarsis	-92.17	15.39	Unclassified	GBIF: https://doi.org/10.15468/dl.zfaw79
Anopheles argyritarsis	-92.000111	17.392194	Unclassified	GBIF: https://doi.org/10.15468/dl.zfaw79
Anopheles argyritarsis	-91.629083	16.097139	Unclassified	GBIF: https://doi.org/10.15468/dl.zfaw79
Anopheles argyritarsis	-91.628889	16.096667	Unclassified	GBIF: https://doi.org/10.15468/dl.zfaw79
Anopheles argyritarsis	-91.187222	17.794167	Unclassified	GBIF: https://doi.org/10.15468/dl.zfaw79

Anopheles argyritarsis	-91.096111	16.088889	Unclassified	GBIF: https://doi.org/10.15468/dl.zfaw79
Anopheles argyritarsis	-91.094667	16.082333	Unclassified	GBIF: https://doi.org/10.15468/dl.zfaw79
Anopheles argyritarsis	-91.094444	16.081111	Unclassified	GBIF: https://doi.org/10.15468/dl.zfaw79
Anopheles argyritarsis	-89.04998	17.26746	Unclassified	GBIF: https://doi.org/10.15468/dl.zfaw79
Anopheles argyritarsis	-88.98331	16.98414	Unclassified	GBIF: https://doi.org/10.15468/dl.zfaw79
Anopheles argyritarsis	-88.96665	17.01747	Unclassified	GBIF: https://doi.org/10.15468/dl.zfaw79
Anopheles argyritarsis	-88.64997	17.08414	Unclassified	GBIF: https://doi.org/10.15468/dl.zfaw79
Anopheles argyritarsis	-88.64997	17.1008	Unclassified	GBIF: https://doi.org/10.15468/dl.zfaw79
Anopheles argyritarsis	-88.39997	18.4674	Unclassified	GBIF: https://doi.org/10.15468/dl.zfaw79
Anopheles argyritarsis	-88.3333	17.65077	Unclassified	GBIF: https://doi.org/10.15468/dl.zfaw79
Anopheles argyritarsis	-84.84207	10.50069	Unclassified	GBIF: https://doi.org/10.15468/dl.zfaw79
Anopheles argyritarsis	-84.6689	10.45074	Unclassified	GBIF: https://doi.org/10.15468/dl.zfaw79
Anopheles argyritarsis	-84.47248	10.09693	Unclassified	GBIF: https://doi.org/10.15468/dl.zfaw79
Anopheles argyritarsis	-84.25465	9.97774	Unclassified	GBIF: https://doi.org/10.15468/dl.zfaw79
Anopheles argyritarsis	-84.24546	9.98669	Unclassified	GBIF: https://doi.org/10.15468/dl.zfaw79
Anopheles argyritarsis	-83.86217	9.81414	Unclassified	GBIF: https://doi.org/10.15468/dl.zfaw79
Anopheles argyritarsis	-83.7225	9.43582	Unclassified	GBIF: https://doi.org/10.15468/dl.zfaw79
Anopheles argyritarsis	-83.71291	9.37264	Unclassified	GBIF: https://doi.org/10.15468/dl.zfaw79
Anopheles argyritarsis	-83.66952	9.85623	Unclassified	GBIF: https://doi.org/10.15468/dl.zfaw79
Anopheles argyritarsis	-83.64385	9.85206	Unclassified	GBIF: https://doi.org/10.15468/dl.zfaw79
Anopheles argyritarsis	-83.45172	9.98453	Unclassified	GBIF: https://doi.org/10.15468/dl.zfaw79
Anopheles argyritarsis	-80	9	Unclassified	GBIF: https://doi.org/10.15468/dl.zfaw79
Anopheles argyritarsis	-79.78313	8.88465	Unclassified	GBIF: https://doi.org/10.15468/dl.zfaw79
Anopheles argyritarsis	-79.779166	9.123994	Unclassified	GBIF: https://doi.org/10.15468/dl.zfaw79
Anopheles argyritarsis	-79.772129	8.884647	Unclassified	GBIF: https://doi.org/10.15468/dl.zfaw79
Anopheles argyritarsis	-79.761713	9.121174	Unclassified	GBIF: https://doi.org/10.15468/dl.zfaw79
Anopheles argyritarsis	-79.611099	9.019542	Unclassified	GBIF: https://doi.org/10.15468/dl.zfaw79
Anopheles argyritarsis	-79.516667	9.016667	Unclassified	GBIF: https://doi.org/10.15468/dl.zfaw79
Anopheles argyritarsis	-79.44404	9.04513	Unclassified	GBIF: https://doi.org/10.15468/dl.zfaw79
Anopheles argyritarsis	-79.41624	9.16256	Unclassified	GBIF: https://doi.org/10.15468/dl.zfaw79
Anopheles argyritarsis	-79.28827	9.09507	Unclassified	GBIF: https://doi.org/10.15468/dl.zfaw79
Anopheles argyritarsis	-77.15037	1.08301	Unclassified	GBIF: https://doi.org/10.15468/dl.zfaw79
Anopheles argyritarsis	-73.71966	3.56736	Unclassified	GBIF: https://doi.org/10.15468/dl.zfaw79
Anopheles argyritarsis	-73.64164	4.14812	Unclassified	GBIF: https://doi.org/10.15468/dl.zfaw79
Anopheles argyritarsis	-73.61867	4.14669	Unclassified	GBIF: https://doi.org/10.15468/dl.zfaw79
Anopheles argyritarsis	-73.46129	4.25631	Unclassified	GBIF: https://doi.org/10.15468/dl.zfaw79
Anopheles argyritarsis	-73.25037	7.11636	Unclassified	GBIF: https://doi.org/10.15468/dl.zfaw79
Anopheles argyritarsis	-72.73531	4.28003	Unclassified	GBIF: https://doi.org/10.15468/dl.zfaw79
Anopheles argyritarsis	-72.64451	4.187754	Unclassified	GBIF: https://doi.org/10.15468/dl.zfaw79
Anopheles argyritarsis	-70.101	-12.5694	Unclassified	GBIF: https://doi.org/10.15468/dl.zfaw79
Anopheles argyritarsis	-68.30192	9.9135	Unclassified	GBIF: https://doi.org/10.15468/dl.zfaw79
Anopheles argyritarsis	-67.60189	10.24685	Unclassified	GBIF: https://doi.org/10.15468/dl.zfaw79

Anopheles argyritarsis	-67.51833	-16.40362	Unclassified	GBIF: https://doi.org/10.15468/dl.zfaw79
Anopheles argyritarsis	-67.47695	10.28788	Unclassified	GBIF: https://doi.org/10.15468/dl.zfaw79
Anopheles argyritarsis	-67.42213	10.29666	Unclassified	GBIF: https://doi.org/10.15468/dl.zfaw79
Anopheles argyritarsis	-66.8125	-38.944172	Unclassified	GBIF: https://doi.org/10.15468/dl.zfaw79
Anopheles argyritarsis	-66.460937	-38.670214	Unclassified	GBIF: https://doi.org/10.15468/dl.zfaw79
Anopheles argyritarsis	-65.16824	-18.67031	Unclassified	GBIF: https://doi.org/10.15468/dl.zfaw79
Anopheles argyritarsis	-62.22447	16.74759	Unclassified	GBIF: https://doi.org/10.15468/dl.zfaw79
Anopheles argyritarsis	-62.21137	16.7376	Unclassified	GBIF: https://doi.org/10.15468/dl.zfaw79
Anopheles argyritarsis	-62.15969	16.75728	Unclassified	GBIF: https://doi.org/10.15468/dl.zfaw79
Anopheles argyritarsis	-61.88931	17.1112	Unclassified	GBIF: https://doi.org/10.15468/dl.zfaw79
Anopheles argyritarsis	-61.86287	17.13094	Unclassified	GBIF: https://doi.org/10.15468/dl.zfaw79
Anopheles argyritarsis	-61.79681	17.02481	Unclassified	GBIF: https://doi.org/10.15468/dl.zfaw79
Anopheles argyritarsis	-61.78986	17.07779	Unclassified	GBIF: https://doi.org/10.15468/dl.zfaw79
Anopheles argyritarsis	-61.75442	12.07985	Unclassified	GBIF: https://doi.org/10.15468/dl.zfaw79
Anopheles argyritarsis	-61.74964	12.12052	Unclassified	GBIF: https://doi.org/10.15468/dl.zfaw79
Anopheles argyritarsis	-61.73543	16.36015	Unclassified	GBIF: https://doi.org/10.15468/dl.zfaw79
Anopheles argyritarsis	-61.7316	12.0481	Unclassified	GBIF: https://doi.org/10.15468/dl.zfaw79
Anopheles argyritarsis	-61.71763	12.08872	Unclassified	GBIF: https://doi.org/10.15468/dl.zfaw79
Anopheles argyritarsis	-61.70405	12.04798	Unclassified	GBIF: https://doi.org/10.15468/dl.zfaw79
Anopheles argyritarsis	-61.70383	12.09318	Unclassified	GBIF: https://doi.org/10.15468/dl.zfaw79
Anopheles argyritarsis	-61.66654	12.2015	Unclassified	GBIF: https://doi.org/10.15468/dl.zfaw79
Anopheles argyritarsis	-61.58702	16.16941	Unclassified	GBIF: https://doi.org/10.15468/dl.zfaw79
Anopheles argyritarsis	-61.5398	16.19761	Unclassified	GBIF: https://doi.org/10.15468/dl.zfaw79
Anopheles argyritarsis	-61.53977	16.19759	Unclassified	GBIF: https://doi.org/10.15468/dl.zfaw79
Anopheles argyritarsis	-61.38786	15.33654	Unclassified	GBIF: https://doi.org/10.15468/dl.zfaw79
Anopheles argyritarsis	-61.37848	15.34551	Unclassified	GBIF: https://doi.org/10.15468/dl.zfaw79
Anopheles argyritarsis	-61.35971	15.36345	Unclassified	GBIF: https://doi.org/10.15468/dl.zfaw79
Anopheles argyritarsis	-61.34137	15.32716	Unclassified	GBIF: https://doi.org/10.15468/dl.zfaw79
Anopheles argyritarsis	-61.3327	15.24576	Unclassified	GBIF: https://doi.org/10.15468/dl.zfaw79
Anopheles argyritarsis	-61.31286	15.39925	Unclassified	GBIF: https://doi.org/10.15468/dl.zfaw79
Anopheles argyritarsis	-61.28528	15.35385	Unclassified	GBIF: https://doi.org/10.15468/dl.zfaw79
Anopheles argyritarsis	-61.2665	15.37178	Unclassified	GBIF: https://doi.org/10.15468/dl.zfaw79
Anopheles argyritarsis	-61.26628	15.39889	Unclassified	GBIF: https://doi.org/10.15468/dl.zfaw79
Anopheles argyritarsis	-61.26613	15.41696	Unclassified	GBIF: https://doi.org/10.15468/dl.zfaw79
Anopheles argyritarsis	-61.01929	14.65485	Unclassified	GBIF: https://doi.org/10.15468/dl.zfaw79
Anopheles argyritarsis	-57.37601	-25.30194	Unclassified	GBIF: https://doi.org/10.15468/dl.zfaw79
Anopheles argyritarsis	-56.3783	-20.240601	Unclassified	GBIF: https://doi.org/10.15468/dl.zfaw79
Anopheles argyritarsis	-54.57	-25.59	Unclassified	GBIF: https://doi.org/10.15468/dl.zfaw79
Anopheles argyritarsis	-53.24993	5.42263	Unclassified	GBIF: https://doi.org/10.15468/dl.zfaw79
Anopheles argyritarsis	-53.03462	5.41345	Unclassified	GBIF: https://doi.org/10.15468/dl.zfaw79
Anopheles argyritarsis	-52.76898	5.21603	Unclassified	GBIF: https://doi.org/10.15468/dl.zfaw79
Anopheles argyritarsis	-52.47027	4.572008	Unclassified	GBIF: https://doi.org/10.15468/dl.zfaw79

Anopheles argyritarsis	-52.3667	-24.05	Unclassified	GBIF: https://doi.org/10.15468/dl.zfaw79
Anopheles argyritarsis	-52.34415	4.83505	Unclassified	GBIF: https://doi.org/10.15468/dl.zfaw79
Anopheles argyritarsis	-51.15	-23.3	Unclassified	GBIF: https://doi.org/10.15468/dl.zfaw79
Anopheles argyritarsis	-46.20086	-21.53884	Unclassified	GBIF: https://doi.org/10.15468/dl.zfaw79
Anopheles argyritarsis	-46.2	-21.54	Unclassified	GBIF: https://doi.org/10.15468/dl.zfaw79
Anopheles argyritarsis	-45.12166	-20.7952	Unclassified	GBIF: https://doi.org/10.15468/dl.zfaw79
Anopheles argyritarsis	-45.12	-20.8	Unclassified	GBIF: https://doi.org/10.15468/dl.zfaw79
Anopheles argyritarsis	-45.06	-20.82	Unclassified	GBIF: https://doi.org/10.15468/dl.zfaw79
Anopheles argyritarsis	-43.2075	-22.9028	Unclassified	GBIF: https://doi.org/10.15468/dl.zfaw79
Anopheles argyritarsis	-41.7869	-22.3708	Unclassified	GBIF: https://doi.org/10.15468/dl.zfaw79
Anopheles argyritarsis	-40.56705	-16.26713	Unclassified	GBIF: https://doi.org/10.15468/dl.zfaw79
Anopheles argyritarsis	-38.4921	-9.8096	Unclassified	GBIF: https://doi.org/10.15468/dl.zfaw79
Anopheles argyritarsis	-38.49	-9.81	Unclassified	GBIF: https://doi.org/10.15468/dl.zfaw79
Anopheles argyritarsis	-37.68822	-9.65569	Unclassified	GBIF: https://doi.org/10.15468/dl.zfaw79
Anopheles argyritarsis	-37.6882	-9.6557	Unclassified	GBIF: https://doi.org/10.15468/dl.zfaw79
Anopheles argyritarsis	-37.688	-9.656	Unclassified	GBIF: https://doi.org/10.15468/dl.zfaw79
Anopheles argyritarsis	-37.67822	-9.65608	Unclassified	GBIF: https://doi.org/10.15468/dl.zfaw79
Anopheles argyritarsis	-37.6782	-9.6561	Unclassified	GBIF: https://doi.org/10.15468/dl.zfaw79
Anopheles argyropus	98.293605	17.875966	Unclassified	GBIF: https://doi.org/10.15468/dl.zfaw79
Anopheles argyropus	98.393148	8.248306	Unclassified	GBIF: https://doi.org/10.15468/dl.zfaw79
Anopheles argyropus	98.795965	8.284143	Unclassified	GBIF: https://doi.org/10.15468/dl.zfaw79
Anopheles argyropus	98.96256	18.87514	Unclassified	GBIF: https://doi.org/10.15468/dl.zfaw79
Anopheles argyropus	98.96256	18.87695	Unclassified	GBIF: https://doi.org/10.15468/dl.zfaw79
Anopheles argyropus	99.566076	15.220405	Unclassified	GBIF: https://doi.org/10.15468/dl.zfaw79
Anopheles argyropus	99.690027	8.027579	Unclassified	GBIF: https://doi.org/10.15468/dl.zfaw79
Anopheles argyropus	100.389404	19.896706	Unclassified	GBIF: https://doi.org/10.15468/dl.zfaw79
Anopheles argyropus	100.508752	20.071488	Unclassified	GBIF: https://doi.org/10.15468/dl.zfaw79
Anopheles argyropus	100.53429	14.06823	Unclassified	GBIF: https://doi.org/10.15468/dl.zfaw79
Anopheles argyropus	100.95037	13.21027	Unclassified	GBIF: https://doi.org/10.15468/dl.zfaw79
Anopheles argyropus	100.95415	14.01007	Unclassified	GBIF: https://doi.org/10.15468/dl.zfaw79
Anopheles argyropus	101.501371	13.239733	Unclassified	GBIF: https://doi.org/10.15468/dl.zfaw79
Anopheles argyropus	102.02336	12.60527	Unclassified	GBIF: https://doi.org/10.15468/dl.zfaw79
Anopheles argyropus	102.381435	12.215942	Unclassified	GBIF: https://doi.org/10.15468/dl.zfaw79
Anopheles argyropus	102.618292	12.230746	Unclassified	GBIF: https://doi.org/10.15468/dl.zfaw79
Anopheles argyropus	102.62282	12.238014	Unclassified	GBIF: https://doi.org/10.15468/dl.zfaw79
Anopheles argyropus	102.666441	12.187784	Unclassified	GBIF: https://doi.org/10.15468/dl.zfaw79
Anopheles argyropus	102.783061	11.957611	Unclassified	GBIF: https://doi.org/10.15468/dl.zfaw79
Anopheles argyropus	102.808502	11.888004	Unclassified	GBIF: https://doi.org/10.15468/dl.zfaw79
Anopheles argyropus	102.829025	11.845686	Unclassified	GBIF: https://doi.org/10.15468/dl.zfaw79
Anopheles argyropus	102.885517	11.777426	Unclassified	GBIF: https://doi.org/10.15468/dl.zfaw79
Anopheles argyropus	102.907592	11.701079	Unclassified	GBIF: https://doi.org/10.15468/dl.zfaw79
Anopheles argyropus	103.38384	15.05659	Unclassified	GBIF: https://doi.org/10.15468/dl.zfaw79

Anopheles artemievi	72.45	41.26	Unclassified	GBIF: https://doi.org/10.15468/dl.zfaw79
Anopheles arthuri	-65.5	-10.64	Unclassified	GBIF: https://doi.org/10.15468/dl.zfaw79
Anopheles arthuri	-63.56	-10.27	Unclassified	GBIF: https://doi.org/10.15468/dl.zfaw79
Anopheles arthuri	-62.23	-9.19	Unclassified	GBIF: https://doi.org/10.15468/dl.zfaw79
Anopheles arthuri	-51.025	-18.906	Unclassified	GBIF: https://doi.org/10.15468/dl.zfaw79
Anopheles arthuri	-51.02	-18.91	Unclassified	GBIF: https://doi.org/10.15468/dl.zfaw79
Anopheles arthuri	-50.92	-21.98	Unclassified	GBIF: https://doi.org/10.15468/dl.zfaw79
Anopheles arthuri	-50.92	-21.68	Unclassified	GBIF: https://doi.org/10.15468/dl.zfaw79
Anopheles arthuri	-49.08	-20.03	Unclassified	GBIF: https://doi.org/10.15468/dl.zfaw79
Anopheles arthuri	-44.974	-20.712	Unclassified	GBIF: https://doi.org/10.15468/dl.zfaw79
Anopheles arthuri	-44.97	-20.71	Unclassified	GBIF: https://doi.org/10.15468/dl.zfaw79
Anopheles arthuri	-44.92	-20.75	Unclassified	GBIF: https://doi.org/10.15468/dl.zfaw79
Anopheles arthuri	-44.916	-20.746	Unclassified	GBIF: https://doi.org/10.15468/dl.zfaw79
Anopheles arthuri	-44.915	-20.746	Unclassified	GBIF: https://doi.org/10.15468/dl.zfaw79
Anopheles arthuri	-44.88	-20.77	Unclassified	GBIF: https://doi.org/10.15468/dl.zfaw79
Anopheles arthuri	-44.8782	-20.76842	Unclassified	GBIF: https://doi.org/10.15468/dl.zfaw79
Anopheles arthuri	-44.77	-20.51	Unclassified	GBIF: https://doi.org/10.15468/dl.zfaw79
Anopheles arthuri	-43.2	-21.54	Unclassified	GBIF: https://doi.org/10.15468/dl.zfaw79
Anopheles arthuri	-41	-3.89	Unclassified	GBIF: https://doi.org/10.15468/dl.zfaw79
Anopheles arthuri	-40.9	-4.1	Unclassified	GBIF: https://doi.org/10.15468/dl.zfaw79
Anopheles arthuri	-40.9	-3.84	Unclassified	GBIF: https://doi.org/10.15468/dl.zfaw79
Anopheles arthuri	-40.09	-19.99	Unclassified	GBIF: https://doi.org/10.15468/dl.zfaw79
Anopheles asiaticus	98.249585	8.587786	Unclassified	GBIF: https://doi.org/10.15468/dl.zfaw79
Anopheles asiaticus	98.59142	8.57402	Unclassified	GBIF: https://doi.org/10.15468/dl.zfaw79
Anopheles asiaticus	98.59158	8.56576	Unclassified	GBIF: https://doi.org/10.15468/dl.zfaw79
Anopheles asiaticus	98.62414	8.59946	Unclassified	GBIF: https://doi.org/10.15468/dl.zfaw79
Anopheles asiaticus	98.6243	8.59119	Unclassified	GBIF: https://doi.org/10.15468/dl.zfaw79
Anopheles asiaticus	98.64189	8.58327	Unclassified	GBIF: https://doi.org/10.15468/dl.zfaw79
Anopheles asiaticus	98.94046	16.83508	Unclassified	GBIF: https://doi.org/10.15468/dl.zfaw79
Anopheles asiaticus	99.257729	10.888848	Unclassified	GBIF: https://doi.org/10.15468/dl.zfaw79
Anopheles asiaticus	99.690027	8.027579	Unclassified	GBIF: https://doi.org/10.15468/dl.zfaw79
Anopheles asiaticus	99.76886	8.14009	Unclassified	GBIF: https://doi.org/10.15468/dl.zfaw79
Anopheles asiaticus	100.389404	19.896706	Unclassified	GBIF: https://doi.org/10.15468/dl.zfaw79
Anopheles asiaticus	100.508752	20.071488	Unclassified	GBIF: https://doi.org/10.15468/dl.zfaw79
Anopheles atacamensis	-71.54775	-35.733593	Unclassified	GBIF: https://doi.org/10.15468/dl.zfaw79
Anopheles atacamensis	-70.917	-27.3	Unclassified	GBIF: https://doi.org/10.15468/dl.zfaw79
Anopheles atratipes	115.633333	-33.333333	Unclassified	GBIF: https://doi.org/10.15468/dl.zfaw79
Anopheles atratipes	137.948267	-35.8438	Unclassified	GBIF: https://doi.org/10.15468/dl.zfaw79
Anopheles atratipes	145.22	-39.02	Unclassified	GBIF: https://doi.org/10.15468/dl.zfaw79
Anopheles atratipes	146.160852	-43.428465	Unclassified	GBIF: https://doi.org/10.15468/dl.zfaw79
Anopheles atratipes	146.3	-39.02	Unclassified	GBIF: https://doi.org/10.15468/dl.zfaw79
Anopheles atratipes	146.8903	-38.2616	Unclassified	GBIF: https://doi.org/10.15468/dl.zfaw79

Anopheles atratipes	148.45	-37.7	Unclassified	GBIF: https://doi.org/10.15468/dl.zfaw79
Anopheles atratipes	148.7	-37.7	Unclassified	GBIF: https://doi.org/10.15468/dl.zfaw79
Anopheles atratipes	151.230236	-33.633333	Unclassified	GBIF: https://doi.org/10.15468/dl.zfaw79
Anopheles atratipes	152.4	-32.433	Unclassified	GBIF: https://doi.org/10.15468/dl.zfaw79
Anopheles atratipes	152.956976	-26.964609	Unclassified	GBIF: https://doi.org/10.15468/dl.zfaw79
Anopheles atratipes	152.961977	-26.771259	Unclassified	GBIF: https://doi.org/10.15468/dl.zfaw79
Anopheles atratipes	153.01666	-27.46667	Unclassified	GBIF: https://doi.org/10.15468/dl.zfaw79
Anopheles atratipes	153.016663	-27.466667	Unclassified	GBIF: https://doi.org/10.15468/dl.zfaw79
Anopheles atratipes	153.021705	-26.446931	Unclassified	GBIF: https://doi.org/10.15468/dl.zfaw79
Anopheles atratipes	153.04001	-26.615275	Unclassified	GBIF: https://doi.org/10.15468/dl.zfaw79
Anopheles atratipes	153.049698	-26.60679	Unclassified	GBIF: https://doi.org/10.15468/dl.zfaw79
Anopheles atratipes	153.05	-27.53333	Unclassified	GBIF: https://doi.org/10.15468/dl.zfaw79
Anopheles atratipes	153.050003	-27.533333	Unclassified	GBIF: https://doi.org/10.15468/dl.zfaw79
Anopheles atratipes	153.050285	-26.868939	Unclassified	GBIF: https://doi.org/10.15468/dl.zfaw79
Anopheles atratipes	153.053238	-26.567411	Unclassified	GBIF: https://doi.org/10.15468/dl.zfaw79
Anopheles atratipes	153.071998	-26.843985	Unclassified	GBIF: https://doi.org/10.15468/dl.zfaw79
Anopheles atratipes	153.080826	-26.567411	Unclassified	GBIF: https://doi.org/10.15468/dl.zfaw79
Anopheles atratipes	153.084012	-26.81462	Unclassified	GBIF: https://doi.org/10.15468/dl.zfaw79
Anopheles atratipes	153.101132	-26.80349	Unclassified	GBIF: https://doi.org/10.15468/dl.zfaw79
Anopheles atratipes	153.116669	-27.233334	Unclassified	GBIF: https://doi.org/10.15468/dl.zfaw79
Anopheles atratipes	153.11667	-27.23333	Unclassified	GBIF: https://doi.org/10.15468/dl.zfaw79
Anopheles atratipes	153.117747	-26.732738	Unclassified	GBIF: https://doi.org/10.15468/dl.zfaw79
Anopheles atratipes	153.13333	-26.8	Unclassified	GBIF: https://doi.org/10.15468/dl.zfaw79
Anopheles atratipes	153.133331	-26.799999	Unclassified	GBIF: https://doi.org/10.15468/dl.zfaw79
Anopheles atratipes	153.402	-27.499	Unclassified	GBIF: https://doi.org/10.15468/dl.zfaw79
Anopheles atratipes	153.404	-27.499	Unclassified	GBIF: https://doi.org/10.15468/dl.zfaw79
Anopheles atroparvus	-8.184	37.951	Unclassified	GBIF: https://doi.org/10.15468/dl.zfaw79
Anopheles atroparvus	-8.04	37.038	Unclassified	GBIF: https://doi.org/10.15468/dl.zfaw79
Anopheles atroparvus	-7.562	41.118	Unclassified	GBIF: https://doi.org/10.15468/dl.zfaw79
Anopheles atroparvus	-7.039	41.14	Unclassified	GBIF: https://doi.org/10.15468/dl.zfaw79
Anopheles atroparvus	-7.011	41.152	Unclassified	GBIF: https://doi.org/10.15468/dl.zfaw79
Anopheles atroparvus	-6.75	37.16	Unclassified	GBIF: https://doi.org/10.15468/dl.zfaw79
Anopheles atroparvus	-4.252183	53.308311	Unclassified	GBIF: https://doi.org/10.15468/dl.zfaw79
Anopheles atroparvus	-3.657158	50.253262	Unclassified	GBIF: https://doi.org/10.15468/dl.zfaw79
Anopheles atroparvus	-3.083265	51.558875	Unclassified	GBIF: https://doi.org/10.15468/dl.zfaw79
Anopheles atroparvus	-3.081134	51.468976	Unclassified	GBIF: https://doi.org/10.15468/dl.zfaw79
Anopheles atroparvus	-3.074116	53.59075	Unclassified	GBIF: https://doi.org/10.15468/dl.zfaw79
Anopheles atroparvus	-3.018242	53.055939	Unclassified	GBIF: https://doi.org/10.15468/dl.zfaw79
Anopheles atroparvus	-2.939038	51.560118	Unclassified	GBIF: https://doi.org/10.15468/dl.zfaw79
Anopheles atroparvus	-2.87	42.45	Unclassified	GBIF: https://doi.org/10.15468/dl.zfaw79
Anopheles atroparvus	-2.86518	42.4537	Unclassified	GBIF: https://doi.org/10.15468/dl.zfaw79
Anopheles atroparvus	-2.86518	42.45372	Unclassified	GBIF: https://doi.org/10.15468/dl.zfaw79

Anopheles atroparvus	-2.856083	51.36743	Unclassified	GBIF: https://doi.org/10.15468/dl.zfaw79
Anopheles atroparvus	-2.805785	51.460267	Unclassified	GBIF: https://doi.org/10.15468/dl.zfaw79
Anopheles atroparvus	-2.800285	51.458507	Unclassified	GBIF: https://doi.org/10.15468/dl.zfaw79
Anopheles atroparvus	-2.796881	51.461407	Unclassified	GBIF: https://doi.org/10.15468/dl.zfaw79
Anopheles atroparvus	-2.794801	51.561184	Unclassified	GBIF: https://doi.org/10.15468/dl.zfaw79
Anopheles atroparvus	-2.785808	51.45784	Unclassified	GBIF: https://doi.org/10.15468/dl.zfaw79
Anopheles atroparvus	-2.639361	51.956847	Unclassified	GBIF: https://doi.org/10.15468/dl.zfaw79
Anopheles atroparvus	-2.6	42.44	Unclassified	GBIF: https://doi.org/10.15468/dl.zfaw79
Anopheles atroparvus	-2.59667	42.4362	Unclassified	GBIF: https://doi.org/10.15468/dl.zfaw79
Anopheles atroparvus	-2.51	42.44	Unclassified	GBIF: https://doi.org/10.15468/dl.zfaw79
Anopheles atroparvus	-2.50962	42.44007	Unclassified	GBIF: https://doi.org/10.15468/dl.zfaw79
Anopheles atroparvus	-2.50962	42.4401	Unclassified	GBIF: https://doi.org/10.15468/dl.zfaw79
Anopheles atroparvus	-2.41	42.48	Unclassified	GBIF: https://doi.org/10.15468/dl.zfaw79
Anopheles atroparvus	-2.4071	42.4844	Unclassified	GBIF: https://doi.org/10.15468/dl.zfaw79
Anopheles atroparvus	-2.200351	52.512219	Unclassified	GBIF: https://doi.org/10.15468/dl.zfaw79
Anopheles atroparvus	-1.96	42.27	Unclassified	GBIF: https://doi.org/10.15468/dl.zfaw79
Anopheles atroparvus	-1.95622	42.2726	Unclassified	GBIF: https://doi.org/10.15468/dl.zfaw79
Anopheles atroparvus	-1.495699	50.913127	Unclassified	GBIF: https://doi.org/10.15468/dl.zfaw79
Anopheles atroparvus	-1.213033	52.927801	Unclassified	GBIF: https://doi.org/10.15468/dl.zfaw79
Anopheles atroparvus	-1.105335	53.495603	Unclassified	GBIF: https://doi.org/10.15468/dl.zfaw79
Anopheles atroparvus	-1.07407	45.98136	Unclassified	GBIF: https://doi.org/10.15468/dl.zfaw79
Anopheles atroparvus	-0.888	51.569	Unclassified	GBIF: https://doi.org/10.15468/dl.zfaw79
Anopheles atroparvus	-0.719085	51.273188	Unclassified	GBIF: https://doi.org/10.15468/dl.zfaw79
Anopheles atroparvus	-0.1806	51.99938	Unclassified	GBIF: https://doi.org/10.15468/dl.zfaw79
Anopheles atroparvus	0.37832	50.93337	Unclassified	GBIF: https://doi.org/10.15468/dl.zfaw79
Anopheles atroparvus	0.37876	50.93582	Unclassified	GBIF: https://doi.org/10.15468/dl.zfaw79
Anopheles atroparvus	0.37943	50.93471	Unclassified	GBIF: https://doi.org/10.15468/dl.zfaw79
Anopheles atroparvus	0.478	51.454	Unclassified	GBIF: https://doi.org/10.15468/dl.zfaw79
Anopheles atroparvus	0.483	51.478	Unclassified	GBIF: https://doi.org/10.15468/dl.zfaw79
Anopheles atroparvus	0.49	51.467	Unclassified	GBIF: https://doi.org/10.15468/dl.zfaw79
Anopheles atroparvus	0.544727	51.759345	Unclassified	GBIF: https://doi.org/10.15468/dl.zfaw79
Anopheles atroparvus	0.79	51.37	Unclassified	GBIF: https://doi.org/10.15468/dl.zfaw79
Anopheles atroparvus	0.7909	51.3743	Unclassified	GBIF: https://doi.org/10.15468/dl.zfaw79
Anopheles atroparvus	1.00991	52.370676	Unclassified	GBIF: https://doi.org/10.15468/dl.zfaw79
Anopheles atroparvus	1.468096	52.591956	Unclassified	GBIF: https://doi.org/10.15468/dl.zfaw79
Anopheles atroparvus	1.577	52.566	Unclassified	GBIF: https://doi.org/10.15468/dl.zfaw79
Anopheles atroparvus	1.577	52.661	Unclassified	GBIF: https://doi.org/10.15468/dl.zfaw79
Anopheles atroparvus	1.622834	52.677263	Unclassified	GBIF: https://doi.org/10.15468/dl.zfaw79
Anopheles atroparvus	1.90291	52.62565	Unclassified	GBIF: https://doi.org/10.15468/dl.zfaw79
Anopheles atroparvus	1.90292	52.62567	Unclassified	GBIF: https://doi.org/10.15468/dl.zfaw79
Anopheles atroparvus	2.48	42.16	Unclassified	GBIF: https://doi.org/10.15468/dl.zfaw79
Anopheles atroparvus	3.251229	51.338514	Unclassified	GBIF: https://doi.org/10.15468/dl.zfaw79

Anopheles atroparvus	4.1935	51.21351	Unclassified	GBIF: https://doi.org/10.15468/dl.zfaw79
Anopheles atroparvus	4.21772	51.25156	Unclassified	GBIF: https://doi.org/10.15468/dl.zfaw79
Anopheles atroparvus	4.316129	51.360051	Unclassified	GBIF: https://doi.org/10.15468/dl.zfaw79
Anopheles atroparvus	4.316443	51.360409	Unclassified	GBIF: https://doi.org/10.15468/dl.zfaw79
Anopheles atroparvus	4.329038	51.376206	Unclassified	GBIF: https://doi.org/10.15468/dl.zfaw79
Anopheles atroparvus	4.445191	51.547904	Unclassified	GBIF: https://doi.org/10.15468/dl.zfaw79
Anopheles atroparvus	4.657632	52.581192	Unclassified	GBIF: https://doi.org/10.15468/dl.zfaw79
Anopheles atroparvus	4.67501	50.618214	Unclassified	GBIF: https://doi.org/10.15468/dl.zfaw79
Anopheles atroparvus	4.920388	51.687935	Unclassified	GBIF: https://doi.org/10.15468/dl.zfaw79
Anopheles atroparvus	4.955437	52.679439	Unclassified	GBIF: https://doi.org/10.15468/dl.zfaw79
Anopheles atroparvus	6.732649	52.561966	Unclassified	GBIF: https://doi.org/10.15468/dl.zfaw79
Anopheles atroparvus	6.735153	52.561848	Unclassified	GBIF: https://doi.org/10.15468/dl.zfaw79
Anopheles atroparvus	6.751778	52.344037	Unclassified	GBIF: https://doi.org/10.15468/dl.zfaw79
Anopheles atroparvus	9.98933	55.78776	Unclassified	GBIF: https://doi.org/10.15468/dl.zfaw79
Anopheles atroparvus	10.648333	56.412756	Unclassified	GBIF: https://doi.org/10.15468/dl.zfaw79
Anopheles atroparvus	10.906	45.149	Unclassified	GBIF: https://doi.org/10.15468/dl.zfaw79
Anopheles atroparvus	10.918	45.182	Unclassified	GBIF: https://doi.org/10.15468/dl.zfaw79
Anopheles atroparvus	10.981	45.149	Unclassified	GBIF: https://doi.org/10.15468/dl.zfaw79
Anopheles atroparvus	12.132	44.55	Unclassified	GBIF: https://doi.org/10.15468/dl.zfaw79
Anopheles atroparvus	21.186548	54.81129	Unclassified	GBIF: https://doi.org/10.15468/dl.zfaw79
Anopheles atroparvus	30.560657	46.349259	Unclassified	GBIF: https://doi.org/10.15468/dl.zfaw79
Anopheles atroparvus	30.561922	46.350012	Unclassified	GBIF: https://doi.org/10.15468/dl.zfaw79
Anopheles atroparvus	30.564007	46.349657	Unclassified	GBIF: https://doi.org/10.15468/dl.zfaw79
Anopheles atroparvus	30.565666	46.347174	Unclassified	GBIF: https://doi.org/10.15468/dl.zfaw79
Anopheles atroparvus	30.565666	46.349545	Unclassified	GBIF: https://doi.org/10.15468/dl.zfaw79
Anopheles atroparvus	30.569601	46.346693	Unclassified	GBIF: https://doi.org/10.15468/dl.zfaw79
Anopheles atroparvus	30.575575	46.350341	Unclassified	GBIF: https://doi.org/10.15468/dl.zfaw79
Anopheles atroparvus	30.579846	46.350105	Unclassified	GBIF: https://doi.org/10.15468/dl.zfaw79
Anopheles atroparvus	30.582815	46.411009	Unclassified	GBIF: https://doi.org/10.15468/dl.zfaw79
Anopheles atroparvus	30.596523	46.397805	Unclassified	GBIF: https://doi.org/10.15468/dl.zfaw79
Anopheles atroparvus	30.59737	46.412597	Unclassified	GBIF: https://doi.org/10.15468/dl.zfaw79
Anopheles atroparvus	30.62052	46.405252	Unclassified	GBIF: https://doi.org/10.15468/dl.zfaw79
Anopheles atroparvus	30.635941	46.401213	Unclassified	GBIF: https://doi.org/10.15468/dl.zfaw79
Anopheles atroparvus	30.63635	46.4054	Unclassified	GBIF: https://doi.org/10.15468/dl.zfaw79
Anopheles atroparvus	30.639417	46.38294	Unclassified	GBIF: https://doi.org/10.15468/dl.zfaw79
Anopheles atroparvus	30.641115	46.384677	Unclassified	GBIF: https://doi.org/10.15468/dl.zfaw79
Anopheles atroparvus	30.642146	46.382395	Unclassified	GBIF: https://doi.org/10.15468/dl.zfaw79
Anopheles atroparvus	30.684149	46.53576	Unclassified	GBIF: https://doi.org/10.15468/dl.zfaw79
Anopheles atroparvus	30.684149	46.538993	Unclassified	GBIF: https://doi.org/10.15468/dl.zfaw79
Anopheles atroparvus	30.690698	46.54216	Unclassified	GBIF: https://doi.org/10.15468/dl.zfaw79
Anopheles atroparvus	30.69188	46.526331	Unclassified	GBIF: https://doi.org/10.15468/dl.zfaw79
Anopheles atroparvus	30.69738	46.545525	Unclassified	GBIF: https://doi.org/10.15468/dl.zfaw79

Anopheles atroparvus	30.698694	46.53725	Unclassified	GBIF: https://doi.org/10.15468/dl.zfaw79
Anopheles atroparvus	30.701518	46.530932	Unclassified	GBIF: https://doi.org/10.15468/dl.zfaw79
Anopheles atropos	-94.956603	29.204528	Unclassified	GBIF: https://doi.org/10.15468/dl.zfaw79
Anopheles atropos	-94.956599	29.204534	Unclassified	GBIF: https://doi.org/10.15468/dl.zfaw79
Anopheles atropos	-94.728076	29.372278	Unclassified	GBIF: https://doi.org/10.15468/dl.zfaw79
Anopheles atropos	-91.23417	30.28889	Unclassified	GBIF: https://doi.org/10.15468/dl.zfaw79
Anopheles atropos	-88.388742	36.235732	Unclassified	GBIF: https://doi.org/10.15468/dl.zfaw79
Anopheles atropos	-87.308548	30.347578	Unclassified	GBIF: https://doi.org/10.15468/dl.zfaw79
Anopheles atropos	-86.833611	21.161111	Unclassified	GBIF: https://doi.org/10.15468/dl.zfaw79
Anopheles atropos	-86.243	30.415	Unclassified	GBIF: https://doi.org/10.15468/dl.zfaw79
Anopheles atropos	-86.1582	30.3295	Unclassified	GBIF: https://doi.org/10.15468/dl.zfaw79
Anopheles atropos	-85.647718	30.181853	Unclassified	GBIF: https://doi.org/10.15468/dl.zfaw79
Anopheles atropos	-82.6843	27.4685	Unclassified	GBIF: https://doi.org/10.15468/dl.zfaw79
Anopheles atropos	-82.684	27.468	Unclassified	GBIF: https://doi.org/10.15468/dl.zfaw79
Anopheles atropos	-82.6837	27.5111	Unclassified	GBIF: https://doi.org/10.15468/dl.zfaw79
Anopheles atropos	-82.6835	27.5111	Unclassified	GBIF: https://doi.org/10.15468/dl.zfaw79
Anopheles atropos	-82.6574	27.5031	Unclassified	GBIF: https://doi.org/10.15468/dl.zfaw79
Anopheles atropos	-82.657	27.503	Unclassified	GBIF: https://doi.org/10.15468/dl.zfaw79
Anopheles atropos	-82.6305	28.461	Unclassified	GBIF: https://doi.org/10.15468/dl.zfaw79
Anopheles atropos	-82.629	27.5322	Unclassified	GBIF: https://doi.org/10.15468/dl.zfaw79
Anopheles atropos	-82.6165	28.4817	Unclassified	GBIF: https://doi.org/10.15468/dl.zfaw79
Anopheles atropos	-82.5932	27.5695	Unclassified	GBIF: https://doi.org/10.15468/dl.zfaw79
Anopheles atropos	-82.593	27.57	Unclassified	GBIF: https://doi.org/10.15468/dl.zfaw79
Anopheles atropos	-82.5905	27.5288	Unclassified	GBIF: https://doi.org/10.15468/dl.zfaw79
Anopheles atropos	-82.588	27.529	Unclassified	GBIF: https://doi.org/10.15468/dl.zfaw79
Anopheles atropos	-82.577	28.5731	Unclassified	GBIF: https://doi.org/10.15468/dl.zfaw79
Anopheles atropos	-82.553	27.64	Unclassified	GBIF: https://doi.org/10.15468/dl.zfaw79
Anopheles atropos	-82.5527	27.6405	Unclassified	GBIF: https://doi.org/10.15468/dl.zfaw79
Anopheles atropos	-82.551	27.475	Unclassified	GBIF: https://doi.org/10.15468/dl.zfaw79
Anopheles atropos	-82.549	27.439	Unclassified	GBIF: https://doi.org/10.15468/dl.zfaw79
Anopheles atropos	-82.5489	27.4394	Unclassified	GBIF: https://doi.org/10.15468/dl.zfaw79
Anopheles atropos	-82.548	27.591	Unclassified	GBIF: https://doi.org/10.15468/dl.zfaw79
Anopheles atropos	-82.548	27.5909	Unclassified	GBIF: https://doi.org/10.15468/dl.zfaw79
Anopheles atropos	-82.536	27.603	Unclassified	GBIF: https://doi.org/10.15468/dl.zfaw79
Anopheles atropos	-82.5357	27.6031	Unclassified	GBIF: https://doi.org/10.15468/dl.zfaw79
Anopheles atropos	-82.5353	27.6164	Unclassified	GBIF: https://doi.org/10.15468/dl.zfaw79
Anopheles atropos	-82.529	27.601	Unclassified	GBIF: https://doi.org/10.15468/dl.zfaw79
Anopheles atropos	-82.525	27.427	Unclassified	GBIF: https://doi.org/10.15468/dl.zfaw79
Anopheles atropos	-82.5249	27.5469	Unclassified	GBIF: https://doi.org/10.15468/dl.zfaw79
Anopheles atropos	-82.504	27.407	Unclassified	GBIF: https://doi.org/10.15468/dl.zfaw79
Anopheles atropos	-82.502348	27.8469	Unclassified	GBIF: https://doi.org/10.15468/dl.zfaw79
Anopheles atropos	-82.499	27.449	Unclassified	GBIF: https://doi.org/10.15468/dl.zfaw79

Anopheles atropos	-82.497	27.628	Unclassified	GBIF: https://doi.org/10.15468/dl.zfaw79
Anopheles atropos	-82.4968	27.628	Unclassified	GBIF: https://doi.org/10.15468/dl.zfaw79
Anopheles atropos	-82.4798	27.4197	Unclassified	GBIF: https://doi.org/10.15468/dl.zfaw79
Anopheles atropos	-82.468	27.5161	Unclassified	GBIF: https://doi.org/10.15468/dl.zfaw79
Anopheles atropos	-82.440543	27.809792	Unclassified	GBIF: https://doi.org/10.15468/dl.zfaw79
Anopheles atropos	-82.432	27.513	Unclassified	GBIF: https://doi.org/10.15468/dl.zfaw79
Anopheles atropos	-82.4262	27.4195	Unclassified	GBIF: https://doi.org/10.15468/dl.zfaw79
Anopheles atropos	-82.426	27.419	Unclassified	GBIF: https://doi.org/10.15468/dl.zfaw79
Anopheles atropos	-82.4249	27.565	Unclassified	GBIF: https://doi.org/10.15468/dl.zfaw79
Anopheles atropos	-82.42	27.575	Unclassified	GBIF: https://doi.org/10.15468/dl.zfaw79
Anopheles atropos	-82.4197	27.5751	Unclassified	GBIF: https://doi.org/10.15468/dl.zfaw79
Anopheles atropos	-82.4171	27.6395	Unclassified	GBIF: https://doi.org/10.15468/dl.zfaw79
Anopheles atropos	-82.402	27.485	Unclassified	GBIF: https://doi.org/10.15468/dl.zfaw79
Anopheles atropos	-82.3766	27.4265	Unclassified	GBIF: https://doi.org/10.15468/dl.zfaw79
Anopheles atropos	-82.362	27.489	Unclassified	GBIF: https://doi.org/10.15468/dl.zfaw79
Anopheles atropos	-82.324	27.475	Unclassified	GBIF: https://doi.org/10.15468/dl.zfaw79
Anopheles atropos	-82.304	27.464	Unclassified	GBIF: https://doi.org/10.15468/dl.zfaw79
Anopheles atropos	-82.2642	28.6428	Unclassified	GBIF: https://doi.org/10.15468/dl.zfaw79
Anopheles atropos	-82.243	27.347	Unclassified	GBIF: https://doi.org/10.15468/dl.zfaw79
Anopheles atropos	-82.1999	27.5915	Unclassified	GBIF: https://doi.org/10.15468/dl.zfaw79
Anopheles atropos	-82.1672	26.4846	Unclassified	GBIF: https://doi.org/10.15468/dl.zfaw79
Anopheles atropos	-82.1563	26.4707	Unclassified	GBIF: https://doi.org/10.15468/dl.zfaw79
Anopheles atropos	-82.1227	27.6136	Unclassified	GBIF: https://doi.org/10.15468/dl.zfaw79
Anopheles atropos	-82.1226	28.0041	Unclassified	GBIF: https://doi.org/10.15468/dl.zfaw79
Anopheles atropos	-82.0981	27.6031	Unclassified	GBIF: https://doi.org/10.15468/dl.zfaw79
Anopheles atropos	-82.098	27.603	Unclassified	GBIF: https://doi.org/10.15468/dl.zfaw79
Anopheles atropos	-82.0156	26.5923	Unclassified	GBIF: https://doi.org/10.15468/dl.zfaw79
Anopheles atropos	-81.9783	26.5377	Unclassified	GBIF: https://doi.org/10.15468/dl.zfaw79
Anopheles atropos	-81.9349	26.4876	Unclassified	GBIF: https://doi.org/10.15468/dl.zfaw79
Anopheles atropos	-81.92	26.5619	Unclassified	GBIF: https://doi.org/10.15468/dl.zfaw79
Anopheles atropos	-81.8974	26.541	Unclassified	GBIF: https://doi.org/10.15468/dl.zfaw79
Anopheles atropos	-81.8963	26.4805	Unclassified	GBIF: https://doi.org/10.15468/dl.zfaw79
Anopheles atropos	-81.8914	26.6784	Unclassified	GBIF: https://doi.org/10.15468/dl.zfaw79
Anopheles atropos	-81.891	26.5197	Unclassified	GBIF: https://doi.org/10.15468/dl.zfaw79
Anopheles atropos	-81.8662	26.675	Unclassified	GBIF: https://doi.org/10.15468/dl.zfaw79
Anopheles atropos	-81.8622	26.4808	Unclassified	GBIF: https://doi.org/10.15468/dl.zfaw79
Anopheles atropos	-81.854	26.5486	Unclassified	GBIF: https://doi.org/10.15468/dl.zfaw79
Anopheles atropos	-81.8441	26.6533	Unclassified	GBIF: https://doi.org/10.15468/dl.zfaw79
Anopheles atropos	-81.8285	26.4757	Unclassified	GBIF: https://doi.org/10.15468/dl.zfaw79
Anopheles atropos	-81.8261	26.485	Unclassified	GBIF: https://doi.org/10.15468/dl.zfaw79
Anopheles atropos	-81.8206	26.4707	Unclassified	GBIF: https://doi.org/10.15468/dl.zfaw79
Anopheles atropos	-81.8206	26.4755	Unclassified	GBIF: https://doi.org/10.15468/dl.zfaw79

Anopheles atropos	-81.8168	26.676	Unclassified	GBIF: https://doi.org/10.15468/dl.zfaw79
Anopheles atropos	-81.816	26.6811	Unclassified	GBIF: https://doi.org/10.15468/dl.zfaw79
Anopheles atropos	-81.813	26.6673	Unclassified	GBIF: https://doi.org/10.15468/dl.zfaw79
Anopheles atropos	-81.8123	26.615	Unclassified	GBIF: https://doi.org/10.15468/dl.zfaw79
Anopheles atropos	-81.8083	26.6559	Unclassified	GBIF: https://doi.org/10.15468/dl.zfaw79
Anopheles atropos	-81.8079	26.6512	Unclassified	GBIF: https://doi.org/10.15468/dl.zfaw79
Anopheles atropos	-81.8074	26.6535	Unclassified	GBIF: https://doi.org/10.15468/dl.zfaw79
Anopheles atropos	-81.8063	26.4851	Unclassified	GBIF: https://doi.org/10.15468/dl.zfaw79
Anopheles atropos	-81.7812	26.4613	Unclassified	GBIF: https://doi.org/10.15468/dl.zfaw79
Anopheles atropos	-81.775	26.3348	Unclassified	GBIF: https://doi.org/10.15468/dl.zfaw79
Anopheles atropos	-81.7692	26.7208	Unclassified	GBIF: https://doi.org/10.15468/dl.zfaw79
Anopheles atropos	-81.7104	26.6394	Unclassified	GBIF: https://doi.org/10.15468/dl.zfaw79
Anopheles atropos	-81.6685	26.62	Unclassified	GBIF: https://doi.org/10.15468/dl.zfaw79
Anopheles atropos	-81.6029	26.6111	Unclassified	GBIF: https://doi.org/10.15468/dl.zfaw79
Anopheles atropos	-81.4328	30.2479	Unclassified	GBIF: https://doi.org/10.15468/dl.zfaw79
Anopheles atropos	-81.43	30.39	Unclassified	GBIF: https://doi.org/10.15468/dl.zfaw79
Anopheles atropos	-81.4182	30.2362	Unclassified	GBIF: https://doi.org/10.15468/dl.zfaw79
Anopheles atropos	-81.4035	30.1941	Unclassified	GBIF: https://doi.org/10.15468/dl.zfaw79
Anopheles atropos	-81.402	30.2515	Unclassified	GBIF: https://doi.org/10.15468/dl.zfaw79
Anopheles atropos	-81.4009	30.2517	Unclassified	GBIF: https://doi.org/10.15468/dl.zfaw79
Anopheles atropos	-81.3758	29.9075	Unclassified	GBIF: https://doi.org/10.15468/dl.zfaw79
Anopheles atropos	-81.3748	30.03	Unclassified	GBIF: https://doi.org/10.15468/dl.zfaw79
Anopheles atropos	-81.3701	29.9996	Unclassified	GBIF: https://doi.org/10.15468/dl.zfaw79
Anopheles atropos	-81.3559	29.848	Unclassified	GBIF: https://doi.org/10.15468/dl.zfaw79
Anopheles atropos	-81.3355	29.9522	Unclassified	GBIF: https://doi.org/10.15468/dl.zfaw79
Anopheles atropos	-81.3311	29.9106	Unclassified	GBIF: https://doi.org/10.15468/dl.zfaw79
Anopheles atropos	-81.274	29.842	Unclassified	GBIF: https://doi.org/10.15468/dl.zfaw79
Anopheles atropos	-81.2245	28.8728	Unclassified	GBIF: https://doi.org/10.15468/dl.zfaw79
Anopheles atropos	-81.1018	29.3293	Unclassified	GBIF: https://doi.org/10.15468/dl.zfaw79
Anopheles atropos	-80.96	24.76	Unclassified	GBIF: https://doi.org/10.15468/dl.zfaw79
Anopheles atropos	-80.9463	28.9997	Unclassified	GBIF: https://doi.org/10.15468/dl.zfaw79
Anopheles atropos	-80.8924	28.9477	Unclassified	GBIF: https://doi.org/10.15468/dl.zfaw79
Anopheles atropos	-80.8742	28.8751	Unclassified	GBIF: https://doi.org/10.15468/dl.zfaw79
Anopheles atropos	-80.8471	28.8597	Unclassified	GBIF: https://doi.org/10.15468/dl.zfaw79
Anopheles atropos	-80.3973	27.6386	Unclassified	GBIF: https://doi.org/10.15468/dl.zfaw79
Anopheles atropos	-80.371969	25.173142	Unclassified	GBIF: https://doi.org/10.15468/dl.zfaw79
Anopheles atropos	-80.193659	25.774266	Unclassified	GBIF: https://doi.org/10.15468/dl.zfaw79
Anopheles atropos	-77.89	34.51	Unclassified	GBIF: https://doi.org/10.15468/dl.zfaw79
Anopheles atropos	81.3873	24.7172	Unclassified	GBIF: https://doi.org/10.15468/dl.zfaw79
Anopheles azaniae	43.88	13.65	Unclassified	GBIF: https://doi.org/10.15468/dl.zfaw79
Anopheles aztecus	-102.927778	20.381111	Unclassified	GBIF: https://doi.org/10.15468/dl.zfaw79
Anopheles aztecus	-102.287222	20.043333	Unclassified	GBIF: https://doi.org/10.15468/dl.zfaw79

Anopheles aztecus	-101.609444	19.515833	Unclassified	GBIF: https://doi.org/10.15468/dl.zfaw79
Anopheles aztecus	-101.398333	19.563333	Unclassified	GBIF: https://doi.org/10.15468/dl.zfaw79
Anopheles aztecus	-101.185278	19.701667	Unclassified	GBIF: https://doi.org/10.15468/dl.zfaw79
Anopheles aztecus	-101.066667	20.372222	Unclassified	GBIF: https://doi.org/10.15468/dl.zfaw79
Anopheles aztecus	-100.878333	20.213889	Unclassified	GBIF: https://doi.org/10.15468/dl.zfaw79
Anopheles aztecus	-100.721667	20.031389	Unclassified	GBIF: https://doi.org/10.15468/dl.zfaw79
Anopheles aztecus	-99.52463	19.11194	Unclassified	GBIF: https://doi.org/10.15468/dl.zfaw79
Anopheles aztecus	-99.488611	19.158611	Unclassified	GBIF: https://doi.org/10.15468/dl.zfaw79
Anopheles aztecus	-99.456111	19.222778	Unclassified	GBIF: https://doi.org/10.15468/dl.zfaw79
Anopheles aztecus	-99.191111	19.406667	Unclassified	GBIF: https://doi.org/10.15468/dl.zfaw79
Anopheles aztecus	-99.15031	19.40066	Unclassified	GBIF: https://doi.org/10.15468/dl.zfaw79
Anopheles aztecus	-99.138889	19.275	Unclassified	GBIF: https://doi.org/10.15468/dl.zfaw79
Anopheles aztecus	-99.133611	19.223056	Unclassified	GBIF: https://doi.org/10.15468/dl.zfaw79
Anopheles aztecus	-99.10031	19.26733	Unclassified	GBIF: https://doi.org/10.15468/dl.zfaw79
Anopheles aztecus	-99.054167	19.304167	Unclassified	GBIF: https://doi.org/10.15468/dl.zfaw79
Anopheles aztecus	-98.3925	20.146111	Unclassified	GBIF: https://doi.org/10.15468/dl.zfaw79
Anopheles baezai	98.795965	8.284143	Unclassified	GBIF: https://doi.org/10.15468/dl.zfaw79
Anopheles baezai	99.690027	8.027579	Unclassified	GBIF: https://doi.org/10.15468/dl.zfaw79
Anopheles baezai	102.574818	14.044675	Unclassified	GBIF: https://doi.org/10.15468/dl.zfaw79
Anopheles baezai	110.333	1.4167	Unclassified	GBIF: https://doi.org/10.15468/dl.zfaw79
Anopheles baezai	115.2167	5.35	Unclassified	GBIF: https://doi.org/10.15468/dl.zfaw79
Anopheles baezai	124.936655	10.746991	Unclassified	GBIF: https://doi.org/10.15468/dl.zfaw79
Anopheles baileyi	98.482444	18.555833	Unclassified	GBIF: https://doi.org/10.15468/dl.zfaw79
Anopheles baileyi	98.554944	18.570583	Unclassified	GBIF: https://doi.org/10.15468/dl.zfaw79
Anopheles baileyi	100.508752	20.071488	Unclassified	GBIF: https://doi.org/10.15468/dl.zfaw79
Anopheles baileyi	102.574818	14.044675	Unclassified	GBIF: https://doi.org/10.15468/dl.zfaw79
Anopheles baileyi	104.69114	38.073254	Unclassified	GBIF: https://doi.org/10.15468/dl.zfaw79
Anopheles baimaii	92.66	11.74	Unclassified	GBIF: https://doi.org/10.15468/dl.zfaw79
Anopheles baimaii	97.95548	18.37789	Unclassified	GBIF: https://doi.org/10.15468/dl.zfaw79
Anopheles baimaii	97.95596	18.29656	Unclassified	GBIF: https://doi.org/10.15468/dl.zfaw79
Anopheles baimaii	97.96386	18.55778	Unclassified	GBIF: https://doi.org/10.15468/dl.zfaw79
Anopheles baimaii	97.96575	18.24239	Unclassified	GBIF: https://doi.org/10.15468/dl.zfaw79
Anopheles baimaii	98.02305	18.14328	Unclassified	GBIF: https://doi.org/10.15468/dl.zfaw79
Anopheles baimaii	98.03245	18.15237	Unclassified	GBIF: https://doi.org/10.15468/dl.zfaw79
Anopheles baimaii	98.04195	18.14338	Unclassified	GBIF: https://doi.org/10.15468/dl.zfaw79
Anopheles baimaii	98.08935	18.11649	Unclassified	GBIF: https://doi.org/10.15468/dl.zfaw79
Anopheles baimaii	98.37308	8.28871	Unclassified	GBIF: https://doi.org/10.15468/dl.zfaw79
Anopheles baimaii	98.62414	8.59946	Unclassified	GBIF: https://doi.org/10.15468/dl.zfaw79
Anopheles baimaii	98.754286	14.714072	Unclassified	GBIF: https://doi.org/10.15468/dl.zfaw79
Anopheles baimaii	98.795965	8.284143	Unclassified	GBIF: https://doi.org/10.15468/dl.zfaw79
Anopheles baimaii	98.8133	8.855	Unclassified	GBIF: https://doi.org/10.15468/dl.zfaw79
Anopheles baimaii	98.9533	9.03	Unclassified	GBIF: https://doi.org/10.15468/dl.zfaw79

Anopheles baimaii	98.9789	9.23889	Unclassified	GBIF: https://doi.org/10.15468/dl.zfaw79
Anopheles baimaii	99.157197	14.135369	Unclassified	GBIF: https://doi.org/10.15468/dl.zfaw79
Anopheles baimaii	99.184979	14.11817	Unclassified	GBIF: https://doi.org/10.15468/dl.zfaw79
Anopheles baimaii	99.211781	14.046716	Unclassified	GBIF: https://doi.org/10.15468/dl.zfaw79
Anopheles baimaii	99.240469	14.02318	Unclassified	GBIF: https://doi.org/10.15468/dl.zfaw79
Anopheles baimaii	99.268212	13.988792	Unclassified	GBIF: https://doi.org/10.15468/dl.zfaw79
Anopheles baimaii	101.501371	13.239733	Unclassified	GBIF: https://doi.org/10.15468/dl.zfaw79
Anopheles balabacensis	98.833869	18.795167	Unclassified	GBIF: https://doi.org/10.15468/dl.zfaw79
Anopheles balabacensis	98.978309	18.794867	Unclassified	GBIF: https://doi.org/10.15468/dl.zfaw79
Anopheles balabacensis	99.530603	7.556705	Unclassified	GBIF: https://doi.org/10.15468/dl.zfaw79
Anopheles balabacensis	99.550667	7.552206	Unclassified	GBIF: https://doi.org/10.15468/dl.zfaw79
Anopheles balabacensis	99.554608	7.51817	Unclassified	GBIF: https://doi.org/10.15468/dl.zfaw79
Anopheles balabacensis	99.575962	7.589625	Unclassified	GBIF: https://doi.org/10.15468/dl.zfaw79
Anopheles balabacensis	99.578111	7.534813	Unclassified	GBIF: https://doi.org/10.15468/dl.zfaw79
Anopheles balabacensis	99.586171	7.53414	Unclassified	GBIF: https://doi.org/10.15468/dl.zfaw79
Anopheles balabacensis	99.588894	7.522688	Unclassified	GBIF: https://doi.org/10.15468/dl.zfaw79
Anopheles balabacensis	99.593971	7.581991	Unclassified	GBIF: https://doi.org/10.15468/dl.zfaw79
Anopheles balabacensis	99.599313	7.548077	Unclassified	GBIF: https://doi.org/10.15468/dl.zfaw79
Anopheles balabacensis	99.613239	7.544507	Unclassified	GBIF: https://doi.org/10.15468/dl.zfaw79
Anopheles balabacensis	99.61474	7.580491	Unclassified	GBIF: https://doi.org/10.15468/dl.zfaw79
Anopheles balabacensis	102.73	12.15	Unclassified	GBIF: https://doi.org/10.15468/dl.zfaw79
Anopheles balabacensis	116.04	5.33	Unclassified	GBIF: https://doi.org/10.15468/dl.zfaw79
Anopheles balabacensis	116.0414	5.33192	Unclassified	GBIF: https://doi.org/10.15468/dl.zfaw79
Anopheles balabacensis	116.6262	5.97634	Unclassified	GBIF: https://doi.org/10.15468/dl.zfaw79
Anopheles balabacensis	116.63	5.98	Unclassified	GBIF: https://doi.org/10.15468/dl.zfaw79
Anopheles balabacensis	116.67802	6.73986	Unclassified	GBIF: https://doi.org/10.15468/dl.zfaw79
Anopheles balabacensis	116.68	6.74	Unclassified	GBIF: https://doi.org/10.15468/dl.zfaw79
Anopheles balabacensis	116.72552	6.30238	Unclassified	GBIF: https://doi.org/10.15468/dl.zfaw79
Anopheles balabacensis	116.73	6.3	Unclassified	GBIF: https://doi.org/10.15468/dl.zfaw79
Anopheles balabacensis	116.74675	6.78297	Unclassified	GBIF: https://doi.org/10.15468/dl.zfaw79
Anopheles balabacensis	116.75	6.96	Unclassified	GBIF: https://doi.org/10.15468/dl.zfaw79
Anopheles balabacensis	116.75	6.78	Unclassified	GBIF: https://doi.org/10.15468/dl.zfaw79
Anopheles balabacensis	116.75076	6.96205	Unclassified	GBIF: https://doi.org/10.15468/dl.zfaw79
Anopheles balabacensis	116.78628	5.68577	Unclassified	GBIF: https://doi.org/10.15468/dl.zfaw79
Anopheles balabacensis	116.78635	6.76837	Unclassified	GBIF: https://doi.org/10.15468/dl.zfaw79
Anopheles balabacensis	116.79	6.77	Unclassified	GBIF: https://doi.org/10.15468/dl.zfaw79
Anopheles balabacensis	116.79	6.81	Unclassified	GBIF: https://doi.org/10.15468/dl.zfaw79
Anopheles balabacensis	116.79	5.69	Unclassified	GBIF: https://doi.org/10.15468/dl.zfaw79
Anopheles balabacensis	116.79096	6.80918	Unclassified	GBIF: https://doi.org/10.15468/dl.zfaw79
Anopheles balabacensis	116.81	6.74	Unclassified	GBIF: https://doi.org/10.15468/dl.zfaw79
Anopheles balabacensis	116.81166	6.7388	Unclassified	GBIF: https://doi.org/10.15468/dl.zfaw79
Anopheles balabacensis	116.81754	6.7593	Unclassified	GBIF: https://doi.org/10.15468/dl.zfaw79

Anopheles balabacensis	116.82	6.76	Unclassified	GBIF: https://doi.org/10.15468/dl.zfaw79
Anopheles balabacensis	116.98878	6.60844	Unclassified	GBIF: https://doi.org/10.15468/dl.zfaw79
Anopheles balabacensis	116.99	6.61	Unclassified	GBIF: https://doi.org/10.15468/dl.zfaw79
Anopheles balabacensis	117.06585	7.21578	Unclassified	GBIF: https://doi.org/10.15468/dl.zfaw79
Anopheles balabacensis	117.07	7.22	Unclassified	GBIF: https://doi.org/10.15468/dl.zfaw79
Anopheles balabacensis	117.1	7.16	Unclassified	GBIF: https://doi.org/10.15468/dl.zfaw79
Anopheles balabacensis	117.10292	7.15585	Unclassified	GBIF: https://doi.org/10.15468/dl.zfaw79
Anopheles balabacensis	117.6917	4.949	Unclassified	GBIF: https://doi.org/10.15468/dl.zfaw79
Anopheles bambusicolus	-76.22	-0.66	Unclassified	GBIF: https://doi.org/10.15468/dl.zfaw79
Anopheles bambusicolus	-76.215556	-0.663889	Unclassified	GBIF: https://doi.org/10.15468/dl.zfaw79
Anopheles bambusicolus	-76.215	-0.664722	Unclassified	GBIF: https://doi.org/10.15468/dl.zfaw79
Anopheles bambusicolus	-76.14	-0.66	Unclassified	GBIF: https://doi.org/10.15468/dl.zfaw79
Anopheles bambusicolus	-73.61867	4.14669	Unclassified	GBIF: https://doi.org/10.15468/dl.zfaw79
Anopheles bambusicolus	-72.055	4.256111	Unclassified	GBIF: https://doi.org/10.15468/dl.zfaw79
Anopheles bancroftii	119.966516	-1.170781	Unclassified	GBIF: https://doi.org/10.15468/dl.zfaw79
Anopheles bancroftii	119.968603	-1.171993	Unclassified	GBIF: https://doi.org/10.15468/dl.zfaw79
Anopheles bancroftii	121.974109	-5.31601	Unclassified	GBIF: https://doi.org/10.15468/dl.zfaw79
Anopheles bancroftii	122.038574	-5.309061	Unclassified	GBIF: https://doi.org/10.15468/dl.zfaw79
Anopheles bancroftii	125.828845	-14.818816	Unclassified	GBIF: https://doi.org/10.15468/dl.zfaw79
Anopheles bancroftii	126.63333	-14.3	Unclassified	GBIF: https://doi.org/10.15468/dl.zfaw79
Anopheles bancroftii	126.633331	-14.3	Unclassified	GBIF: https://doi.org/10.15468/dl.zfaw79
Anopheles bancroftii	128.681527	-2.868118	Unclassified	GBIF: https://doi.org/10.15468/dl.zfaw79
Anopheles bancroftii	128.684444	-2.867778	Unclassified	GBIF: https://doi.org/10.15468/dl.zfaw79
Anopheles bancroftii	129.55	-14.1	Unclassified	GBIF: https://doi.org/10.15468/dl.zfaw79
Anopheles bancroftii	129.550003	-14.1	Unclassified	GBIF: https://doi.org/10.15468/dl.zfaw79
Anopheles bancroftii	130.6833	-13.75	Unclassified	GBIF: https://doi.org/10.15468/dl.zfaw79
Anopheles bancroftii	130.683304	-13.75	Unclassified	GBIF: https://doi.org/10.15468/dl.zfaw79
Anopheles bancroftii	130.73331	-13.5833	Unclassified	GBIF: https://doi.org/10.15468/dl.zfaw79
Anopheles bancroftii	130.816666	-13.5	Unclassified	GBIF: https://doi.org/10.15468/dl.zfaw79
Anopheles bancroftii	130.81667	-13.5	Unclassified	GBIF: https://doi.org/10.15468/dl.zfaw79
Anopheles bancroftii	130.828	-12.452	Unclassified	GBIF: https://doi.org/10.15468/dl.zfaw79
Anopheles bancroftii	130.833298	-12.45	Unclassified	GBIF: https://doi.org/10.15468/dl.zfaw79
Anopheles bancroftii	130.8333	-12.45	Unclassified	GBIF: https://doi.org/10.15468/dl.zfaw79
Anopheles bancroftii	130.833333	-13.466667	Unclassified	GBIF: https://doi.org/10.15468/dl.zfaw79
Anopheles bancroftii	130.833333	-12.466667	Unclassified	GBIF: https://doi.org/10.15468/dl.zfaw79
Anopheles bancroftii	130.84244	-12.46152	Unclassified	GBIF: https://doi.org/10.15468/dl.zfaw79
Anopheles bancroftii	130.861	-12.385	Unclassified	GBIF: https://doi.org/10.15468/dl.zfaw79
Anopheles bancroftii	130.865	-12.381	Unclassified	GBIF: https://doi.org/10.15468/dl.zfaw79
Anopheles bancroftii	130.8667	-12.45	Unclassified	GBIF: https://doi.org/10.15468/dl.zfaw79
Anopheles bancroftii	130.899994	-12.4	Unclassified	GBIF: https://doi.org/10.15468/dl.zfaw79
Anopheles bancroftii	130.912074	-12.406917	Unclassified	GBIF: https://doi.org/10.15468/dl.zfaw79
Anopheles bancroftii	130.91667	-12.41667	Unclassified	GBIF: https://doi.org/10.15468/dl.zfaw79

Anopheles bancroftii	130.91667	-12.43333	Unclassified	GBIF: https://doi.org/10.15468/dl.zfaw79
Anopheles bancroftii	130.916672	-12.433333	Unclassified	GBIF: https://doi.org/10.15468/dl.zfaw79
Anopheles bancroftii	130.932	-12.399	Unclassified	GBIF: https://doi.org/10.15468/dl.zfaw79
Anopheles bancroftii	130.933334	-12.4	Unclassified	GBIF: https://doi.org/10.15468/dl.zfaw79
Anopheles bancroftii	130.96666	-12.7	Unclassified	GBIF: https://doi.org/10.15468/dl.zfaw79
Anopheles bancroftii	131	-12.983334	Unclassified	GBIF: https://doi.org/10.15468/dl.zfaw79
Anopheles bancroftii	131	-12.98333	Unclassified	GBIF: https://doi.org/10.15468/dl.zfaw79
Anopheles bancroftii	131.024287	-12.435758	Unclassified	GBIF: https://doi.org/10.15468/dl.zfaw79
Anopheles bancroftii	131.03334	-13.066667	Unclassified	GBIF: https://doi.org/10.15468/dl.zfaw79
Anopheles bancroftii	131.03334	-13.06667	Unclassified	GBIF: https://doi.org/10.15468/dl.zfaw79
Anopheles bancroftii	131.089996	-13.222	Unclassified	GBIF: https://doi.org/10.15468/dl.zfaw79
Anopheles bancroftii	131.09	-13.222	Unclassified	GBIF: https://doi.org/10.15468/dl.zfaw79
Anopheles bancroftii	131.100006	-13.233334	Unclassified	GBIF: https://doi.org/10.15468/dl.zfaw79
Anopheles bancroftii	131.10001	-13.23333	Unclassified	GBIF: https://doi.org/10.15468/dl.zfaw79
Anopheles bancroftii	131.25	-12.633333	Unclassified	GBIF: https://doi.org/10.15468/dl.zfaw79
Anopheles bancroftii	131.25	-12.63333	Unclassified	GBIF: https://doi.org/10.15468/dl.zfaw79
Anopheles bancroftii	131.254	-12.9167	Unclassified	GBIF: https://doi.org/10.15468/dl.zfaw79
Anopheles bancroftii	131.28334	-12.566667	Unclassified	GBIF: https://doi.org/10.15468/dl.zfaw79
Anopheles bancroftii	131.28334	-12.56667	Unclassified	GBIF: https://doi.org/10.15468/dl.zfaw79
Anopheles bancroftii	131.366669	-12.783334	Unclassified	GBIF: https://doi.org/10.15468/dl.zfaw79
Anopheles bancroftii	131.36667	-12.78333	Unclassified	GBIF: https://doi.org/10.15468/dl.zfaw79
Anopheles bancroftii	131.3667	-12.78333	Unclassified	GBIF: https://doi.org/10.15468/dl.zfaw79
Anopheles bancroftii	131.669998	-13.201	Unclassified	GBIF: https://doi.org/10.15468/dl.zfaw79
Anopheles bancroftii	131.67	-13.201	Unclassified	GBIF: https://doi.org/10.15468/dl.zfaw79
Anopheles bancroftii	132.366669	-13.35	Unclassified	GBIF: https://doi.org/10.15468/dl.zfaw79
Anopheles bancroftii	132.36667	-13.35	Unclassified	GBIF: https://doi.org/10.15468/dl.zfaw79
Anopheles bancroftii	132.64999	-12.75	Unclassified	GBIF: https://doi.org/10.15468/dl.zfaw79
Anopheles bancroftii	132.649994	-12.75	Unclassified	GBIF: https://doi.org/10.15468/dl.zfaw79
Anopheles bancroftii	132.817	-14.483	Unclassified	GBIF: https://doi.org/10.15468/dl.zfaw79
Anopheles bancroftii	132.817001	-14.483	Unclassified	GBIF: https://doi.org/10.15468/dl.zfaw79
Anopheles bancroftii	132.866699	-12.58333	Unclassified	GBIF: https://doi.org/10.15468/dl.zfaw79
Anopheles bancroftii	132.8667	-12.58333	Unclassified	GBIF: https://doi.org/10.15468/dl.zfaw79
Anopheles bancroftii	133.066666	-12.333333	Unclassified	GBIF: https://doi.org/10.15468/dl.zfaw79
Anopheles bancroftii	133.06667	-12.33333	Unclassified	GBIF: https://doi.org/10.15468/dl.zfaw79
Anopheles bancroftii	133.75	-14.866667	Unclassified	GBIF: https://doi.org/10.15468/dl.zfaw79
Anopheles bancroftii	133.75	-14.86667	Unclassified	GBIF: https://doi.org/10.15468/dl.zfaw79
Anopheles bancroftii	134.100006	-14.05	Unclassified	GBIF: https://doi.org/10.15468/dl.zfaw79
Anopheles bancroftii	134.10001	-14.05	Unclassified	GBIF: https://doi.org/10.15468/dl.zfaw79
Anopheles bancroftii	134.533	-14.733	Unclassified	GBIF: https://doi.org/10.15468/dl.zfaw79
Anopheles bancroftii	134.733337	-14.733334	Unclassified	GBIF: https://doi.org/10.15468/dl.zfaw79
Anopheles bancroftii	134.73334	-14.73333	Unclassified	GBIF: https://doi.org/10.15468/dl.zfaw79
Anopheles bancroftii	135.3833	-14.73333	Unclassified	GBIF: https://doi.org/10.15468/dl.zfaw79

Anopheles bancroftii	135.383301	-14.73333	Unclassified	GBIF: https://doi.org/10.15468/dl.zfaw79
Anopheles bancroftii	136.46666	-13.9	Unclassified	GBIF: https://doi.org/10.15468/dl.zfaw79
Anopheles bancroftii	136.76666	-12.16667	Unclassified	GBIF: https://doi.org/10.15468/dl.zfaw79
Anopheles bancroftii	136.766663	-12.166667	Unclassified	GBIF: https://doi.org/10.15468/dl.zfaw79
Anopheles bancroftii	140.083298	-17.4667	Unclassified	GBIF: https://doi.org/10.15468/dl.zfaw79
Anopheles bancroftii	140.276667	-6.080278	Unclassified	GBIF: https://doi.org/10.15468/dl.zfaw79
Anopheles bancroftii	140.28329	-6.08333	Unclassified	GBIF: https://doi.org/10.15468/dl.zfaw79
Anopheles bancroftii	140.283295	-6.083333	Unclassified	GBIF: https://doi.org/10.15468/dl.zfaw79
Anopheles bancroftii	140.333298	-8.4667	Unclassified	GBIF: https://doi.org/10.15468/dl.zfaw79
Anopheles bancroftii	140.3333	-8.4667	Unclassified	GBIF: https://doi.org/10.15468/dl.zfaw79
Anopheles bancroftii	140.404444	-8.495833	Unclassified	GBIF: https://doi.org/10.15468/dl.zfaw79
Anopheles bancroftii	140.407156	-8.500401	Unclassified	GBIF: https://doi.org/10.15468/dl.zfaw79
Anopheles bancroftii	140.670742	-2.591103	Unclassified	GBIF: https://doi.org/10.15468/dl.zfaw79
Anopheles bancroftii	141.067	-17.383	Unclassified	GBIF: https://doi.org/10.15468/dl.zfaw79
Anopheles bancroftii	141.591	-15.198	Unclassified	GBIF: https://doi.org/10.15468/dl.zfaw79
Anopheles bancroftii	141.591003	-15.198	Unclassified	GBIF: https://doi.org/10.15468/dl.zfaw79
Anopheles bancroftii	141.733337	-15.466666	Unclassified	GBIF: https://doi.org/10.15468/dl.zfaw79
Anopheles bancroftii	141.73334	-15.46667	Unclassified	GBIF: https://doi.org/10.15468/dl.zfaw79
Anopheles bancroftii	141.75	-15.466666	Unclassified	GBIF: https://doi.org/10.15468/dl.zfaw79
Anopheles bancroftii	141.75	-15.46667	Unclassified	GBIF: https://doi.org/10.15468/dl.zfaw79
Anopheles bancroftii	141.93333	-14.2	Unclassified	GBIF: https://doi.org/10.15468/dl.zfaw79
Anopheles bancroftii	141.933334	-14.2	Unclassified	GBIF: https://doi.org/10.15468/dl.zfaw79
Anopheles bancroftii	142.116669	-10.716666	Unclassified	GBIF: https://doi.org/10.15468/dl.zfaw79
Anopheles bancroftii	142.11667	-10.71667	Unclassified	GBIF: https://doi.org/10.15468/dl.zfaw79
Anopheles bancroftii	142.14471	-10.11555	Unclassified	GBIF: https://doi.org/10.15468/dl.zfaw79
Anopheles bancroftii	142.26666	-10.18333	Unclassified	GBIF: https://doi.org/10.15468/dl.zfaw79
Anopheles bancroftii	142.266663	-10.183333	Unclassified	GBIF: https://doi.org/10.15468/dl.zfaw79
Anopheles bancroftii	142.316666	-10.9	Unclassified	GBIF: https://doi.org/10.15468/dl.zfaw79
Anopheles bancroftii	142.31667	-10.9	Unclassified	GBIF: https://doi.org/10.15468/dl.zfaw79
Anopheles bancroftii	142.366669	-11.15	Unclassified	GBIF: https://doi.org/10.15468/dl.zfaw79
Anopheles bancroftii	142.36667	-11.15	Unclassified	GBIF: https://doi.org/10.15468/dl.zfaw79
Anopheles bancroftii	142.38333	-10.88333	Unclassified	GBIF: https://doi.org/10.15468/dl.zfaw79
Anopheles bancroftii	142.383331	-10.883333	Unclassified	GBIF: https://doi.org/10.15468/dl.zfaw79
Anopheles bancroftii	142.46666	-10.8	Unclassified	GBIF: https://doi.org/10.15468/dl.zfaw79
Anopheles bancroftii	142.467	-10.8	Unclassified	GBIF: https://doi.org/10.15468/dl.zfaw79
Anopheles bancroftii	142.66667	-9.38333	Unclassified	GBIF: https://doi.org/10.15468/dl.zfaw79
Anopheles bancroftii	142.666672	-9.383333	Unclassified	GBIF: https://doi.org/10.15468/dl.zfaw79
Anopheles bancroftii	143.3	-12.73333	Unclassified	GBIF: https://doi.org/10.15468/dl.zfaw79
Anopheles bancroftii	143.300003	-12.733334	Unclassified	GBIF: https://doi.org/10.15468/dl.zfaw79
Anopheles bancroftii	143.316666	-12.716666	Unclassified	GBIF: https://doi.org/10.15468/dl.zfaw79
Anopheles bancroftii	143.31667	-12.71667	Unclassified	GBIF: https://doi.org/10.15468/dl.zfaw79
Anopheles bancroftii	143.55	-13.98333	Unclassified	GBIF: https://doi.org/10.15468/dl.zfaw79

Anopheles bancroftii	143.550003	-13.983334	Unclassified	GBIF: https://doi.org/10.15468/dl.zfaw79
Anopheles bancroftii	143.583328	-14.733334	Unclassified	GBIF: https://doi.org/10.15468/dl.zfaw79
Anopheles bancroftii	143.58333	-14.73333	Unclassified	GBIF: https://doi.org/10.15468/dl.zfaw79
Anopheles bancroftii	144.066666	-4.066667	Unclassified	GBIF: https://doi.org/10.15468/dl.zfaw79
Anopheles bancroftii	144.06667	-4.06667	Unclassified	GBIF: https://doi.org/10.15468/dl.zfaw79
Anopheles bancroftii	144.25	-5.833333	Unclassified	GBIF: https://doi.org/10.15468/dl.zfaw79
Anopheles bancroftii	144.25	-5.83333	Unclassified	GBIF: https://doi.org/10.15468/dl.zfaw79
Anopheles bancroftii	144.66667	-5.91667	Unclassified	GBIF: https://doi.org/10.15468/dl.zfaw79
Anopheles bancroftii	144.666672	-5.916667	Unclassified	GBIF: https://doi.org/10.15468/dl.zfaw79
Anopheles bancroftii	145.199997	-17.416666	Unclassified	GBIF: https://doi.org/10.15468/dl.zfaw79
Anopheles bancroftii	145.2	-17.41667	Unclassified	GBIF: https://doi.org/10.15468/dl.zfaw79
Anopheles bancroftii	145.316666	-16.25	Unclassified	GBIF: https://doi.org/10.15468/dl.zfaw79
Anopheles bancroftii	145.316666	-16.299999	Unclassified	GBIF: https://doi.org/10.15468/dl.zfaw79
Anopheles bancroftii	145.31667	-16.3	Unclassified	GBIF: https://doi.org/10.15468/dl.zfaw79
Anopheles bancroftii	145.31667	-16.25	Unclassified	GBIF: https://doi.org/10.15468/dl.zfaw79
Anopheles bancroftii	145.333328	-16.683332	Unclassified	GBIF: https://doi.org/10.15468/dl.zfaw79
Anopheles bancroftii	145.33333	-16.68333	Unclassified	GBIF: https://doi.org/10.15468/dl.zfaw79
Anopheles bancroftii	145.366669	-16.583334	Unclassified	GBIF: https://doi.org/10.15468/dl.zfaw79
Anopheles bancroftii	145.366669	-16.450001	Unclassified	GBIF: https://doi.org/10.15468/dl.zfaw79
Anopheles bancroftii	145.36667	-16.58333	Unclassified	GBIF: https://doi.org/10.15468/dl.zfaw79
Anopheles bancroftii	145.36667	-16.45	Unclassified	GBIF: https://doi.org/10.15468/dl.zfaw79
Anopheles bancroftii	145.38333	-6.08333	Unclassified	GBIF: https://doi.org/10.15468/dl.zfaw79
Anopheles bancroftii	145.383331	-6.083333	Unclassified	GBIF: https://doi.org/10.15468/dl.zfaw79
Anopheles bancroftii	145.39999	-6.06667	Unclassified	GBIF: https://doi.org/10.15468/dl.zfaw79
Anopheles bancroftii	145.399994	-6.066667	Unclassified	GBIF: https://doi.org/10.15468/dl.zfaw79
Anopheles bancroftii	145.41667	-16.91667	Unclassified	GBIF: https://doi.org/10.15468/dl.zfaw79
Anopheles bancroftii	145.416672	-16.916666	Unclassified	GBIF: https://doi.org/10.15468/dl.zfaw79
Anopheles bancroftii	145.423	-16.997	Unclassified	GBIF: https://doi.org/10.15468/dl.zfaw79
Anopheles bancroftii	145.423004	-16.997	Unclassified	GBIF: https://doi.org/10.15468/dl.zfaw79
Anopheles bancroftii	145.4333	-17.1333	Unclassified	GBIF: https://doi.org/10.15468/dl.zfaw79
Anopheles bancroftii	145.433304	-17.133301	Unclassified	GBIF: https://doi.org/10.15468/dl.zfaw79
Anopheles bancroftii	145.483337	-17.266666	Unclassified	GBIF: https://doi.org/10.15468/dl.zfaw79
Anopheles bancroftii	145.48334	-17.26667	Unclassified	GBIF: https://doi.org/10.15468/dl.zfaw79
Anopheles bancroftii	145.567778	-17.110833	Unclassified	GBIF: https://doi.org/10.15468/dl.zfaw79
Anopheles bancroftii	145.602	-17.141	Unclassified	GBIF: https://doi.org/10.15468/dl.zfaw79
Anopheles bancroftii	145.63333	-17.16667	Unclassified	GBIF: https://doi.org/10.15468/dl.zfaw79
Anopheles bancroftii	145.63333	-17.25	Unclassified	GBIF: https://doi.org/10.15468/dl.zfaw79
Anopheles bancroftii	145.633331	-17.25	Unclassified	GBIF: https://doi.org/10.15468/dl.zfaw79
Anopheles bancroftii	145.633331	-17.166666	Unclassified	GBIF: https://doi.org/10.15468/dl.zfaw79
Anopheles bancroftii	145.733337	-16.9	Unclassified	GBIF: https://doi.org/10.15468/dl.zfaw79
Anopheles bancroftii	145.73334	-16.9	Unclassified	GBIF: https://doi.org/10.15468/dl.zfaw79
Anopheles bancroftii	145.76666	-17.03333	Unclassified	GBIF: https://doi.org/10.15468/dl.zfaw79

Anopheles bancroftii	145.76666	-16.91667	Unclassified	GBIF: https://doi.org/10.15468/dl.zfaw79
Anopheles bancroftii	145.766663	-16.916666	Unclassified	GBIF: https://doi.org/10.15468/dl.zfaw79
Anopheles bancroftii	145.766663	-17.033333	Unclassified	GBIF: https://doi.org/10.15468/dl.zfaw79
Anopheles bancroftii	145.79807	-16.948556	Unclassified	GBIF: https://doi.org/10.15468/dl.zfaw79
Anopheles bancroftii	146.03329	-17.56667	Unclassified	GBIF: https://doi.org/10.15468/dl.zfaw79
Anopheles bancroftii	146.033295	-17.566669	Unclassified	GBIF: https://doi.org/10.15468/dl.zfaw79
Anopheles bancroftii	146.083328	-17.566668	Unclassified	GBIF: https://doi.org/10.15468/dl.zfaw79
Anopheles bancroftii	146.08333	-17.56667	Unclassified	GBIF: https://doi.org/10.15468/dl.zfaw79
Anopheles bancroftii	146.100006	-17.783333	Unclassified	GBIF: https://doi.org/10.15468/dl.zfaw79
Anopheles bancroftii	146.10001	-17.78333	Unclassified	GBIF: https://doi.org/10.15468/dl.zfaw79
Anopheles bancroftii	146.251007	-18.799	Unclassified	GBIF: https://doi.org/10.15468/dl.zfaw79
Anopheles bancroftii	146.25101	-18.799	Unclassified	GBIF: https://doi.org/10.15468/dl.zfaw79
Anopheles bancroftii	146.55	-18.66667	Unclassified	GBIF: https://doi.org/10.15468/dl.zfaw79
Anopheles bancroftii	146.550003	-18.666666	Unclassified	GBIF: https://doi.org/10.15468/dl.zfaw79
Anopheles bancroftii	146.68333	-19.45	Unclassified	GBIF: https://doi.org/10.15468/dl.zfaw79
Anopheles bancroftii	146.683334	-19.450001	Unclassified	GBIF: https://doi.org/10.15468/dl.zfaw79
Anopheles bancroftii	146.733307	-19.450001	Unclassified	GBIF: https://doi.org/10.15468/dl.zfaw79
Anopheles bancroftii	146.73331	-19.45	Unclassified	GBIF: https://doi.org/10.15468/dl.zfaw79
Anopheles bancroftii	146.800833	-19.282777	Unclassified	GBIF: https://doi.org/10.15468/dl.zfaw79
Anopheles bancroftii	146.816666	-19.266666	Unclassified	GBIF: https://doi.org/10.15468/dl.zfaw79
Anopheles bancroftii	146.816666	-19.26667	Unclassified	GBIF: https://doi.org/10.15468/dl.zfaw79
Anopheles bancroftii	146.81667	-19.26667	Unclassified	GBIF: https://doi.org/10.15468/dl.zfaw79
Anopheles bancroftii	147.100006	-19.5	Unclassified	GBIF: https://doi.org/10.15468/dl.zfaw79
Anopheles bancroftii	147.10001	-19.5	Unclassified	GBIF: https://doi.org/10.15468/dl.zfaw79
Anopheles bancroftii	147.21666	-19.783333	Unclassified	GBIF: https://doi.org/10.15468/dl.zfaw79
Anopheles bancroftii	147.21666	-19.78333	Unclassified	GBIF: https://doi.org/10.15468/dl.zfaw79
Anopheles bancroftii	147.350006	-19.549999	Unclassified	GBIF: https://doi.org/10.15468/dl.zfaw79
Anopheles bancroftii	147.35001	-19.55	Unclassified	GBIF: https://doi.org/10.15468/dl.zfaw79
Anopheles bancroftii	147.55	-19.65	Unclassified	GBIF: https://doi.org/10.15468/dl.zfaw79
Anopheles bancroftii	147.550003	-19.65	Unclassified	GBIF: https://doi.org/10.15468/dl.zfaw79
Anopheles bancroftii	148.366699	-8.7667	Unclassified	GBIF: https://doi.org/10.15468/dl.zfaw79
Anopheles bancroftii	148.3667	-8.7667	Unclassified	GBIF: https://doi.org/10.15468/dl.zfaw79
Anopheles bancroftii	149.16	-21.04	Unclassified	GBIF: https://doi.org/10.15468/dl.zfaw79
Anopheles bancroftii	149.233337	-21.066668	Unclassified	GBIF: https://doi.org/10.15468/dl.zfaw79
Anopheles bancroftii	149.852642	-9.976595	Unclassified	GBIF: https://doi.org/10.15468/dl.zfaw79
Anopheles bancroftii	150.449997	-23.4	Unclassified	GBIF: https://doi.org/10.15468/dl.zfaw79
Anopheles bancroftii	150.45	-23.4	Unclassified	GBIF: https://doi.org/10.15468/dl.zfaw79
Anopheles bancroftii	150.51361	-23.37804	Unclassified	GBIF: https://doi.org/10.15468/dl.zfaw79
Anopheles bancroftii	150.53334	-23.366667	Unclassified	GBIF: https://doi.org/10.15468/dl.zfaw79
Anopheles bancroftii	150.53334	-23.36667	Unclassified	GBIF: https://doi.org/10.15468/dl.zfaw79
Anopheles bancroftii	151.12257	-25.3706	Unclassified	GBIF: https://doi.org/10.15468/dl.zfaw79
Anopheles bancroftii	151.949997	-25	Unclassified	GBIF: https://doi.org/10.15468/dl.zfaw79

Anopheles bancroftii	151.95	-25	Unclassified	GBIF: https://doi.org/10.15468/dl.zfaw79
Anopheles bancroftii	152.25	-24.85	Unclassified	GBIF: https://doi.org/10.15468/dl.zfaw79
Anopheles bancroftii	152.350006	-27.117001	Unclassified	GBIF: https://doi.org/10.15468/dl.zfaw79
Anopheles bancroftii	152.35001	-27.117	Unclassified	GBIF: https://doi.org/10.15468/dl.zfaw79
Anopheles bancroftii	152.68333	-26.46667	Unclassified	GBIF: https://doi.org/10.15468/dl.zfaw79
Anopheles bancroftii	152.683334	-26.466667	Unclassified	GBIF: https://doi.org/10.15468/dl.zfaw79
Anopheles bancroftii	152.695503	-25.790197	Unclassified	GBIF: https://doi.org/10.15468/dl.zfaw79
Anopheles bancroftii	152.69574	-25.790092	Unclassified	GBIF: https://doi.org/10.15468/dl.zfaw79
Anopheles bancroftii	152.699997	-25.533333	Unclassified	GBIF: https://doi.org/10.15468/dl.zfaw79
Anopheles bancroftii	152.7	-25.53333	Unclassified	GBIF: https://doi.org/10.15468/dl.zfaw79
Anopheles bancroftii	152.76666	-25.3	Unclassified	GBIF: https://doi.org/10.15468/dl.zfaw79
Anopheles bancroftii	152.766663	-25.299999	Unclassified	GBIF: https://doi.org/10.15468/dl.zfaw79
Anopheles bancroftii	152.88333	-27.4	Unclassified	GBIF: https://doi.org/10.15468/dl.zfaw79
Anopheles bancroftii	152.883331	-27.4	Unclassified	GBIF: https://doi.org/10.15468/dl.zfaw79
Anopheles bancroftii	152.949997	-27.166667	Unclassified	GBIF: https://doi.org/10.15468/dl.zfaw79
Anopheles bancroftii	152.961828	-26.878008	Unclassified	GBIF: https://doi.org/10.15468/dl.zfaw79
Anopheles bancroftii	152.96666	-27.450001	Unclassified	GBIF: https://doi.org/10.15468/dl.zfaw79
Anopheles bancroftii	152.96666	-27.45	Unclassified	GBIF: https://doi.org/10.15468/dl.zfaw79
Anopheles bancroftii	153.01666	-27.46667	Unclassified	GBIF: https://doi.org/10.15468/dl.zfaw79
Anopheles bancroftii	153.016663	-27.466667	Unclassified	GBIF: https://doi.org/10.15468/dl.zfaw79
Anopheles bancroftii	153.021659	-26.446942	Unclassified	GBIF: https://doi.org/10.15468/dl.zfaw79
Anopheles bancroftii	153.03334	-27.566668	Unclassified	GBIF: https://doi.org/10.15468/dl.zfaw79
Anopheles bancroftii	153.03334	-27.56667	Unclassified	GBIF: https://doi.org/10.15468/dl.zfaw79
Anopheles bancroftii	153.05	-26.68333	Unclassified	GBIF: https://doi.org/10.15468/dl.zfaw79
Anopheles bancroftii	153.050003	-26.683332	Unclassified	GBIF: https://doi.org/10.15468/dl.zfaw79
Anopheles bancroftii	153.053791	-27.212088	Unclassified	GBIF: https://doi.org/10.15468/dl.zfaw79
Anopheles bancroftii	153.072068	-26.84392	Unclassified	GBIF: https://doi.org/10.15468/dl.zfaw79
Anopheles bancroftii	153.13333	-27.48333	Unclassified	GBIF: https://doi.org/10.15468/dl.zfaw79
Anopheles bancroftii	153.133331	-27.483334	Unclassified	GBIF: https://doi.org/10.15468/dl.zfaw79
Anopheles bancroftii	153.25	-27.75	Unclassified	GBIF: https://doi.org/10.15468/dl.zfaw79
Anopheles bancroftii	153.402	-27.499	Unclassified	GBIF: https://doi.org/10.15468/dl.zfaw79
Anopheles bancroftii	153.402	-27.483	Unclassified	GBIF: https://doi.org/10.15468/dl.zfaw79
Anopheles bancroftii	153.404	-27.499	Unclassified	GBIF: https://doi.org/10.15468/dl.zfaw79
Anopheles bancroftii	153.41667	-27.48333	Unclassified	GBIF: https://doi.org/10.15468/dl.zfaw79
Anopheles barberellus	-10.366666	6.4	Unclassified	GBIF: https://doi.org/10.15468/dl.zfaw79
Anopheles barberellus	5.0659	9.30023	Unclassified	GBIF: https://doi.org/10.15468/dl.zfaw79
Anopheles barberellus	6.36593	4.684	Unclassified	GBIF: https://doi.org/10.15468/dl.zfaw79
Anopheles barberellus	6.73259	7.80037	Unclassified	GBIF: https://doi.org/10.15468/dl.zfaw79
Anopheles barberellus	7.01594	4.85065	Unclassified	GBIF: https://doi.org/10.15468/dl.zfaw79
Anopheles barberellus	7.54928	6.4505	Unclassified	GBIF: https://doi.org/10.15468/dl.zfaw79
Anopheles barberellus	8.89929	9.86685	Unclassified	GBIF: https://doi.org/10.15468/dl.zfaw79
Anopheles barberellus	12.04935	9.4669	Unclassified	GBIF: https://doi.org/10.15468/dl.zfaw79

Anopheles barberi	-110.856389	30.778056	Unclassified	GBIF: https://doi.org/10.15468/dl.zfaw79
Anopheles barberi	-97.581	33.376	Unclassified	GBIF: https://doi.org/10.15468/dl.zfaw79
Anopheles barberi	-97.580944	33.376482	Unclassified	GBIF: https://doi.org/10.15468/dl.zfaw79
Anopheles barberi	-97.58	29.59	Unclassified	GBIF: https://doi.org/10.15468/dl.zfaw79
Anopheles barberi	-97.5311	32.7112	Unclassified	GBIF: https://doi.org/10.15468/dl.zfaw79
Anopheles barberi	-97.4658	32.9353	Unclassified	GBIF: https://doi.org/10.15468/dl.zfaw79
Anopheles barberi	-97.1607	32.6628	Unclassified	GBIF: https://doi.org/10.15468/dl.zfaw79
Anopheles barberi	-97.0878	32.8638	Unclassified	GBIF: https://doi.org/10.15468/dl.zfaw79
Anopheles barberi	-95.930609	41.285325	Unclassified	GBIF: https://doi.org/10.15468/dl.zfaw79
Anopheles barberi	-93.78	41.78	Unclassified	GBIF: https://doi.org/10.15468/dl.zfaw79
Anopheles barberi	-93.69	41.58	Unclassified	GBIF: https://doi.org/10.15468/dl.zfaw79
Anopheles barberi	-93.68	41.58	Unclassified	GBIF: https://doi.org/10.15468/dl.zfaw79
Anopheles barberi	-93.67	41.64	Unclassified	GBIF: https://doi.org/10.15468/dl.zfaw79
Anopheles barberi	-93.66	42.04	Unclassified	GBIF: https://doi.org/10.15468/dl.zfaw79
Anopheles barberi	-93.66	42.03	Unclassified	GBIF: https://doi.org/10.15468/dl.zfaw79
Anopheles barberi	-93.62	42.06	Unclassified	GBIF: https://doi.org/10.15468/dl.zfaw79
Anopheles barberi	-93.61	41.61	Unclassified	GBIF: https://doi.org/10.15468/dl.zfaw79
Anopheles barberi	-93.61	41.62	Unclassified	GBIF: https://doi.org/10.15468/dl.zfaw79
Anopheles barberi	-93.6	42.04	Unclassified	GBIF: https://doi.org/10.15468/dl.zfaw79
Anopheles barberi	-93.58	41.54	Unclassified	GBIF: https://doi.org/10.15468/dl.zfaw79
Anopheles barberi	-93.56	41.62	Unclassified	GBIF: https://doi.org/10.15468/dl.zfaw79
Anopheles barberi	-92.58306	40.19472	Unclassified	GBIF: https://doi.org/10.15468/dl.zfaw79
Anopheles barberi	-92.41	42.52	Unclassified	GBIF: https://doi.org/10.15468/dl.zfaw79
Anopheles barberi	-92.31	42.46	Unclassified	GBIF: https://doi.org/10.15468/dl.zfaw79
Anopheles barberi	-91.74	42.07	Unclassified	GBIF: https://doi.org/10.15468/dl.zfaw79
Anopheles barberi	-91.71	42.01	Unclassified	GBIF: https://doi.org/10.15468/dl.zfaw79
Anopheles barberi	-91.63	41.99	Unclassified	GBIF: https://doi.org/10.15468/dl.zfaw79
Anopheles barberi	-91.183289	30.495605	Unclassified	GBIF: https://doi.org/10.15468/dl.zfaw79
Anopheles barberi	-90.68	41.53	Unclassified	GBIF: https://doi.org/10.15468/dl.zfaw79
Anopheles barberi	-90.53	41.54	Unclassified	GBIF: https://doi.org/10.15468/dl.zfaw79
Anopheles barberi	-90.15083	38.62444	Unclassified	GBIF: https://doi.org/10.15468/dl.zfaw79
Anopheles barberi	-90.14	42.09	Unclassified	GBIF: https://doi.org/10.15468/dl.zfaw79
Anopheles barberi	-89.862778	38.539444	Unclassified	GBIF: https://doi.org/10.15468/dl.zfaw79
Anopheles barberi	-89.42	43.04	Unclassified	GBIF: https://doi.org/10.15468/dl.zfaw79
Anopheles barberi	-89.2797	37.6399	Unclassified	GBIF: https://doi.org/10.15468/dl.zfaw79
Anopheles barberi	-88.569012	47.121872	Unclassified	GBIF: https://doi.org/10.15468/dl.zfaw79
Anopheles barberi	-88.388742	36.235732	Unclassified	GBIF: https://doi.org/10.15468/dl.zfaw79
Anopheles barberi	-88.182	31.82	Unclassified	GBIF: https://doi.org/10.15468/dl.zfaw79
Anopheles barberi	-88.181662	31.82037	Unclassified	GBIF: https://doi.org/10.15468/dl.zfaw79
Anopheles barberi	-87.9062	41.7246	Unclassified	GBIF: https://doi.org/10.15468/dl.zfaw79
Anopheles barberi	-87.9062	41.8871	Unclassified	GBIF: https://doi.org/10.15468/dl.zfaw79
Anopheles barberi	-87.8966	41.7874	Unclassified	GBIF: https://doi.org/10.15468/dl.zfaw79

Anopheles barberi	-87.888711	42.042804	Unclassified	GBIF: https://doi.org/10.15468/dl.zfaw79
Anopheles barberi	-87.8851	41.7397	Unclassified	GBIF: https://doi.org/10.15468/dl.zfaw79
Anopheles barberi	-87.8399	41.8308	Unclassified	GBIF: https://doi.org/10.15468/dl.zfaw79
Anopheles barberi	-87.8258	41.8755	Unclassified	GBIF: https://doi.org/10.15468/dl.zfaw79
Anopheles barberi	-87.8194	41.7374	Unclassified	GBIF: https://doi.org/10.15468/dl.zfaw79
Anopheles barberi	-87.8191	41.9027	Unclassified	GBIF: https://doi.org/10.15468/dl.zfaw79
Anopheles barberi	-87.8052	41.8373	Unclassified	GBIF: https://doi.org/10.15468/dl.zfaw79
Anopheles barberi	-87.614274	45.107763	Unclassified	GBIF: https://doi.org/10.15468/dl.zfaw79
Anopheles barberi	-87.51	44.21	Unclassified	GBIF: https://doi.org/10.15468/dl.zfaw79
Anopheles barberi	-87.450338	32.907758	Unclassified	GBIF: https://doi.org/10.15468/dl.zfaw79
Anopheles barberi	-87.45	32.908	Unclassified	GBIF: https://doi.org/10.15468/dl.zfaw79
Anopheles barberi	-87.443	32.909	Unclassified	GBIF: https://doi.org/10.15468/dl.zfaw79
Anopheles barberi	-87.442949	32.90911	Unclassified	GBIF: https://doi.org/10.15468/dl.zfaw79
Anopheles barberi	-87.409	32.974	Unclassified	GBIF: https://doi.org/10.15468/dl.zfaw79
Anopheles barberi	-87.408875	32.97357	Unclassified	GBIF: https://doi.org/10.15468/dl.zfaw79
Anopheles barberi	-87.406101	32.916037	Unclassified	GBIF: https://doi.org/10.15468/dl.zfaw79
Anopheles barberi	-86.530554	43.625286	Unclassified	GBIF: https://doi.org/10.15468/dl.zfaw79
Anopheles barberi	-86.3963	30.3972	Unclassified	GBIF: https://doi.org/10.15468/dl.zfaw79
Anopheles barberi	-86.3226	39.8735	Unclassified	GBIF: https://doi.org/10.15468/dl.zfaw79
Anopheles barberi	-86.3172	39.739	Unclassified	GBIF: https://doi.org/10.15468/dl.zfaw79
Anopheles barberi	-86.196	39.7309	Unclassified	GBIF: https://doi.org/10.15468/dl.zfaw79
Anopheles barberi	-86.1797	39.8503	Unclassified	GBIF: https://doi.org/10.15468/dl.zfaw79
Anopheles barberi	-86.1233	39.6788	Unclassified	GBIF: https://doi.org/10.15468/dl.zfaw79
Anopheles barberi	-86.0561	39.7552	Unclassified	GBIF: https://doi.org/10.15468/dl.zfaw79
Anopheles barberi	-85.9932	39.6672	Unclassified	GBIF: https://doi.org/10.15468/dl.zfaw79
Anopheles barberi	-85.9878	39.8913	Unclassified	GBIF: https://doi.org/10.15468/dl.zfaw79
Anopheles barberi	-85.9762	39.736	Unclassified	GBIF: https://doi.org/10.15468/dl.zfaw79
Anopheles barberi	-85.800051	43.419743	Unclassified	GBIF: https://doi.org/10.15468/dl.zfaw79
Anopheles barberi	-84.3	44.0833	Unclassified	GBIF: https://doi.org/10.15468/dl.zfaw79
Anopheles barberi	-83.86	41.48	Unclassified	GBIF: https://doi.org/10.15468/dl.zfaw79
Anopheles barberi	-83.8574	41.4847	Unclassified	GBIF: https://doi.org/10.15468/dl.zfaw79
Anopheles barberi	-83.8355	41.6229	Unclassified	GBIF: https://doi.org/10.15468/dl.zfaw79
Anopheles barberi	-83.8	41.45	Unclassified	GBIF: https://doi.org/10.15468/dl.zfaw79
Anopheles barberi	-83.7993	41.4456	Unclassified	GBIF: https://doi.org/10.15468/dl.zfaw79
Anopheles barberi	-83.7923	41.5692	Unclassified	GBIF: https://doi.org/10.15468/dl.zfaw79
Anopheles barberi	-83.7563	41.6759	Unclassified	GBIF: https://doi.org/10.15468/dl.zfaw79
Anopheles barberi	-83.72	41.62	Unclassified	GBIF: https://doi.org/10.15468/dl.zfaw79
Anopheles barberi	-83.67	41.69	Unclassified	GBIF: https://doi.org/10.15468/dl.zfaw79
Anopheles barberi	-83.6687	41.6934	Unclassified	GBIF: https://doi.org/10.15468/dl.zfaw79
Anopheles barberi	-83.6011	41.7232	Unclassified	GBIF: https://doi.org/10.15468/dl.zfaw79
Anopheles barberi	-83.6009	41.7229	Unclassified	GBIF: https://doi.org/10.15468/dl.zfaw79
Anopheles barberi	-83.5984	41.6403	Unclassified	GBIF: https://doi.org/10.15468/dl.zfaw79

Anopheles barberi	-83.576	41.6874	Unclassified	GBIF: https://doi.org/10.15468/dl.zfaw79
Anopheles barberi	-83.4708	41.6631	Unclassified	GBIF: https://doi.org/10.15468/dl.zfaw79
Anopheles barberi	-83.4708	41.663	Unclassified	GBIF: https://doi.org/10.15468/dl.zfaw79
Anopheles barberi	-83.4371	41.6444	Unclassified	GBIF: https://doi.org/10.15468/dl.zfaw79
Anopheles barberi	-83.337	41.6738	Unclassified	GBIF: https://doi.org/10.15468/dl.zfaw79
Anopheles barberi	-83.3115	41.4028	Unclassified	GBIF: https://doi.org/10.15468/dl.zfaw79
Anopheles barberi	-82.790033	42.212625	Unclassified	GBIF: https://doi.org/10.15468/dl.zfaw79
Anopheles barberi	-82.783286	40.139244	Unclassified	GBIF: https://doi.org/10.15468/dl.zfaw79
Anopheles barberi	-82.6837	27.5111	Unclassified	GBIF: https://doi.org/10.15468/dl.zfaw79
Anopheles barberi	-82.6574	27.5031	Unclassified	GBIF: https://doi.org/10.15468/dl.zfaw79
Anopheles barberi	-82.5527	27.6405	Unclassified	GBIF: https://doi.org/10.15468/dl.zfaw79
Anopheles barberi	-82.5125	41.95833	Unclassified	GBIF: https://doi.org/10.15468/dl.zfaw79
Anopheles barberi	-82.1227	27.6136	Unclassified	GBIF: https://doi.org/10.15468/dl.zfaw79
Anopheles barberi	-82.0981	27.6031	Unclassified	GBIF: https://doi.org/10.15468/dl.zfaw79
Anopheles barberi	-82.00951	29.70074	Unclassified	GBIF: https://doi.org/10.15468/dl.zfaw79
Anopheles barberi	-81.2992	41.7358	Unclassified	GBIF: https://doi.org/10.15468/dl.zfaw79
Anopheles barberi	-79.921309	32.827356	Unclassified	GBIF: https://doi.org/10.15468/dl.zfaw79
Anopheles barberi	-79.4833	35.029	Unclassified	GBIF: https://doi.org/10.15468/dl.zfaw79
Anopheles barberi	-78.343755	39.675359	Unclassified	GBIF: https://doi.org/10.15468/dl.zfaw79
Anopheles barberi	-77.83417	39.11111	Unclassified	GBIF: https://doi.org/10.15468/dl.zfaw79
Anopheles barberi	-77.209318	38.683963	Unclassified	GBIF: https://doi.org/10.15468/dl.zfaw79
Anopheles barberi	-77.17	40.31	Unclassified	GBIF: https://doi.org/10.15468/dl.zfaw79
Anopheles barberi	-77.05	38.96	Unclassified	GBIF: https://doi.org/10.15468/dl.zfaw79
Anopheles barberi	-76.924	38.9927	Unclassified	GBIF: https://doi.org/10.15468/dl.zfaw79
Anopheles barberi	-76.9164	38.9869	Unclassified	GBIF: https://doi.org/10.15468/dl.zfaw79
Anopheles barberi	-76.8718	39.0035	Unclassified	GBIF: https://doi.org/10.15468/dl.zfaw79
Anopheles barberi	-76.8578	38.9532	Unclassified	GBIF: https://doi.org/10.15468/dl.zfaw79
Anopheles barberi	-76.7742	38.9662	Unclassified	GBIF: https://doi.org/10.15468/dl.zfaw79
Anopheles barberi	-76.7586	39.0427	Unclassified	GBIF: https://doi.org/10.15468/dl.zfaw79
Anopheles barberi	-76.748577	38.539288	Unclassified	GBIF: https://doi.org/10.15468/dl.zfaw79
Anopheles barberi	-76.662803	37.332009	Unclassified	GBIF: https://doi.org/10.15468/dl.zfaw79
Anopheles barberi	-76.547	36.644	Unclassified	GBIF: https://doi.org/10.15468/dl.zfaw79
Anopheles barberi	-76.527	42.469	Unclassified	GBIF: https://doi.org/10.15468/dl.zfaw79
Anopheles barberi	-76.49	36.795	Unclassified	GBIF: https://doi.org/10.15468/dl.zfaw79
Anopheles barberi	-74.94781	39.377097	Unclassified	GBIF: https://doi.org/10.15468/dl.zfaw79
Anopheles barberi	-73.969583	41.504816	Unclassified	GBIF: https://doi.org/10.15468/dl.zfaw79
Anopheles barberi	-73.033186	41.550539	Unclassified	GBIF: https://doi.org/10.15468/dl.zfaw79
Anopheles barberi	-72.829031	41.85401	Unclassified	GBIF: https://doi.org/10.15468/dl.zfaw79
Anopheles barberi	-72.575595	41.295795	Unclassified	GBIF: https://doi.org/10.15468/dl.zfaw79
Anopheles barberi	-72.402565	41.043	Unclassified	GBIF: https://doi.org/10.15468/dl.zfaw79
Anopheles barberi	-72.220399	42.42152	Unclassified	GBIF: https://doi.org/10.15468/dl.zfaw79
Anopheles barberi	-71.35	42.175	Unclassified	GBIF: https://doi.org/10.15468/dl.zfaw79

Anopheles barberi	-71.289	42.246	Unclassified	GBIF: https://doi.org/10.15468/dl.zfaw79
Anopheles barberi	-71.2237	42.1946	Unclassified	GBIF: https://doi.org/10.15468/dl.zfaw79
Anopheles barberi	-71.12	42.222	Unclassified	GBIF: https://doi.org/10.15468/dl.zfaw79
Anopheles barberi	-71.0427	42.1224	Unclassified	GBIF: https://doi.org/10.15468/dl.zfaw79
Anopheles barberi	-71.03	42.167	Unclassified	GBIF: https://doi.org/10.15468/dl.zfaw79
Anopheles barberi	-71.017	42.152	Unclassified	GBIF: https://doi.org/10.15468/dl.zfaw79
Anopheles barberi	-71.001	42.271	Unclassified	GBIF: https://doi.org/10.15468/dl.zfaw79
Anopheles barberi	-64.8232	6.574	Unclassified	GBIF: https://doi.org/10.15468/dl.zfaw79
Anopheles barbirostris	73.65	33.12	Unclassified	GBIF: https://doi.org/10.15468/dl.zfaw79
Anopheles barbirostris	80.66931	7.696631	Unclassified	GBIF: https://doi.org/10.15468/dl.zfaw79
Anopheles barbirostris	84.88	19.3	Unclassified	GBIF: https://doi.org/10.15468/dl.zfaw79
Anopheles barbirostris	84.883	19.3017	Unclassified	GBIF: https://doi.org/10.15468/dl.zfaw79
Anopheles barbirostris	92.66	11.74	Unclassified	GBIF: https://doi.org/10.15468/dl.zfaw79
Anopheles barbirostris	92.76	9.16	Unclassified	GBIF: https://doi.org/10.15468/dl.zfaw79
Anopheles barbirostris	92.9	12.65	Unclassified	GBIF: https://doi.org/10.15468/dl.zfaw79
Anopheles barbirostris	93.36	7.97	Unclassified	GBIF: https://doi.org/10.15468/dl.zfaw79
Anopheles barbirostris	93.78	7.03	Unclassified	GBIF: https://doi.org/10.15468/dl.zfaw79
Anopheles barbirostris	95.29	27.2	Unclassified	GBIF: https://doi.org/10.15468/dl.zfaw79
Anopheles barbirostris	95.323611	5.549444	Unclassified	GBIF: https://doi.org/10.15468/dl.zfaw79
Anopheles barbirostris	97.773802	17.838145	Unclassified	GBIF: https://doi.org/10.15468/dl.zfaw79
Anopheles barbirostris	97.809343	18.851386	Unclassified	GBIF: https://doi.org/10.15468/dl.zfaw79
Anopheles barbirostris	97.826148	18.754803	Unclassified	GBIF: https://doi.org/10.15468/dl.zfaw79
Anopheles barbirostris	97.839458	18.611205	Unclassified	GBIF: https://doi.org/10.15468/dl.zfaw79
Anopheles barbirostris	97.850588	18.64742	Unclassified	GBIF: https://doi.org/10.15468/dl.zfaw79
Anopheles barbirostris	97.886859	17.714105	Unclassified	GBIF: https://doi.org/10.15468/dl.zfaw79
Anopheles barbirostris	97.891199	19.198597	Unclassified	GBIF: https://doi.org/10.15468/dl.zfaw79
Anopheles barbirostris	97.896109	18.199462	Unclassified	GBIF: https://doi.org/10.15468/dl.zfaw79
Anopheles barbirostris	97.896331	18.164219	Unclassified	GBIF: https://doi.org/10.15468/dl.zfaw79
Anopheles barbirostris	97.898905	18.798621	Unclassified	GBIF: https://doi.org/10.15468/dl.zfaw79
Anopheles barbirostris	97.900708	17.607539	Unclassified	GBIF: https://doi.org/10.15468/dl.zfaw79
Anopheles barbirostris	97.904747	18.479659	Unclassified	GBIF: https://doi.org/10.15468/dl.zfaw79
Anopheles barbirostris	97.911148	18.214007	Unclassified	GBIF: https://doi.org/10.15468/dl.zfaw79
Anopheles barbirostris	97.91318	17.570554	Unclassified	GBIF: https://doi.org/10.15468/dl.zfaw79
Anopheles barbirostris	97.914317	18.123971	Unclassified	GBIF: https://doi.org/10.15468/dl.zfaw79
Anopheles barbirostris	97.924769	19.057216	Unclassified	GBIF: https://doi.org/10.15468/dl.zfaw79
Anopheles barbirostris	97.924988	17.960138	Unclassified	GBIF: https://doi.org/10.15468/dl.zfaw79
Anopheles barbirostris	97.928027	18.082159	Unclassified	GBIF: https://doi.org/10.15468/dl.zfaw79
Anopheles barbirostris	97.928655	18.745478	Unclassified	GBIF: https://doi.org/10.15468/dl.zfaw79
Anopheles barbirostris	97.930401	19.068093	Unclassified	GBIF: https://doi.org/10.15468/dl.zfaw79
Anopheles barbirostris	97.934712	18.381326	Unclassified	GBIF: https://doi.org/10.15468/dl.zfaw79
Anopheles barbirostris	97.938127	18.131016	Unclassified	GBIF: https://doi.org/10.15468/dl.zfaw79
Anopheles barbirostris	97.941246	17.947884	Unclassified	GBIF: https://doi.org/10.15468/dl.zfaw79

Anopheles barbirostris	97.945471	18.938958	Unclassified	GBIF: https://doi.org/10.15468/dl.zfaw79
Anopheles barbirostris	97.94749	19.070902	Unclassified	GBIF: https://doi.org/10.15468/dl.zfaw79
Anopheles barbirostris	97.948904	18.382309	Unclassified	GBIF: https://doi.org/10.15468/dl.zfaw79
Anopheles barbirostris	97.960614	18.164577	Unclassified	GBIF: https://doi.org/10.15468/dl.zfaw79
Anopheles barbirostris	97.982849	18.328586	Unclassified	GBIF: https://doi.org/10.15468/dl.zfaw79
Anopheles barbirostris	98.004753	18.860719	Unclassified	GBIF: https://doi.org/10.15468/dl.zfaw79
Anopheles barbirostris	98.00489	18.350394	Unclassified	GBIF: https://doi.org/10.15468/dl.zfaw79
Anopheles barbirostris	98.009414	17.54756	Unclassified	GBIF: https://doi.org/10.15468/dl.zfaw79
Anopheles barbirostris	98.042889	17.451921	Unclassified	GBIF: https://doi.org/10.15468/dl.zfaw79
Anopheles barbirostris	98.062728	17.439362	Unclassified	GBIF: https://doi.org/10.15468/dl.zfaw79
Anopheles barbirostris	98.077687	18.373036	Unclassified	GBIF: https://doi.org/10.15468/dl.zfaw79
Anopheles barbirostris	98.099076	17.515451	Unclassified	GBIF: https://doi.org/10.15468/dl.zfaw79
Anopheles barbirostris	98.143289	17.358119	Unclassified	GBIF: https://doi.org/10.15468/dl.zfaw79
Anopheles barbirostris	98.151179	17.582298	Unclassified	GBIF: https://doi.org/10.15468/dl.zfaw79
Anopheles barbirostris	98.159761	17.61602	Unclassified	GBIF: https://doi.org/10.15468/dl.zfaw79
Anopheles barbirostris	98.161384	19.501423	Unclassified	GBIF: https://doi.org/10.15468/dl.zfaw79
Anopheles barbirostris	98.167296	18.040862	Unclassified	GBIF: https://doi.org/10.15468/dl.zfaw79
Anopheles barbirostris	98.189487	18.350047	Unclassified	GBIF: https://doi.org/10.15468/dl.zfaw79
Anopheles barbirostris	98.20166	17.900948	Unclassified	GBIF: https://doi.org/10.15468/dl.zfaw79
Anopheles barbirostris	98.206252	18.445299	Unclassified	GBIF: https://doi.org/10.15468/dl.zfaw79
Anopheles barbirostris	98.20844	17.865638	Unclassified	GBIF: https://doi.org/10.15468/dl.zfaw79
Anopheles barbirostris	98.21222	18.11704	Unclassified	GBIF: https://doi.org/10.15468/dl.zfaw79
Anopheles barbirostris	98.221983	17.936853	Unclassified	GBIF: https://doi.org/10.15468/dl.zfaw79
Anopheles barbirostris	98.227472	17.223143	Unclassified	GBIF: https://doi.org/10.15468/dl.zfaw79
Anopheles barbirostris	98.2331	17.22678	Unclassified	GBIF: https://doi.org/10.15468/dl.zfaw79
Anopheles barbirostris	98.249585	8.587786	Unclassified	GBIF: https://doi.org/10.15468/dl.zfaw79
Anopheles barbirostris	98.256188	17.311012	Unclassified	GBIF: https://doi.org/10.15468/dl.zfaw79
Anopheles barbirostris	98.259209	19.493173	Unclassified	GBIF: https://doi.org/10.15468/dl.zfaw79
Anopheles barbirostris	98.268263	9.163782	Unclassified	GBIF: https://doi.org/10.15468/dl.zfaw79
Anopheles barbirostris	98.270466	8.785356	Unclassified	GBIF: https://doi.org/10.15468/dl.zfaw79
Anopheles barbirostris	98.270636	17.248612	Unclassified	GBIF: https://doi.org/10.15468/dl.zfaw79
Anopheles barbirostris	98.280448	8.795324	Unclassified	GBIF: https://doi.org/10.15468/dl.zfaw79
Anopheles barbirostris	98.29335	19.120191	Unclassified	GBIF: https://doi.org/10.15468/dl.zfaw79
Anopheles barbirostris	98.301521	17.2903	Unclassified	GBIF: https://doi.org/10.15468/dl.zfaw79
Anopheles barbirostris	98.320916	9.037794	Unclassified	GBIF: https://doi.org/10.15468/dl.zfaw79
Anopheles barbirostris	98.335418	17.800191	Unclassified	GBIF: https://doi.org/10.15468/dl.zfaw79
Anopheles barbirostris	98.340133	18.555785	Unclassified	GBIF: https://doi.org/10.15468/dl.zfaw79
Anopheles barbirostris	98.344341	17.681818	Unclassified	GBIF: https://doi.org/10.15468/dl.zfaw79
Anopheles barbirostris	98.362877	8.438202	Unclassified	GBIF: https://doi.org/10.15468/dl.zfaw79
Anopheles barbirostris	98.369411	17.247138	Unclassified	GBIF: https://doi.org/10.15468/dl.zfaw79
Anopheles barbirostris	98.373113	8.286264	Unclassified	GBIF: https://doi.org/10.15468/dl.zfaw79
Anopheles barbirostris	98.375289	8.610302	Unclassified	GBIF: https://doi.org/10.15468/dl.zfaw79

Anopheles barbirostris	98.380422	8.820827	Unclassified	GBIF: https://doi.org/10.15468/dl.zfaw79
Anopheles barbirostris	98.382218	8.834397	Unclassified	GBIF: https://doi.org/10.15468/dl.zfaw79
Anopheles barbirostris	98.38406	8.253719	Unclassified	GBIF: https://doi.org/10.15468/dl.zfaw79
Anopheles barbirostris	98.391201	8.333326	Unclassified	GBIF: https://doi.org/10.15468/dl.zfaw79
Anopheles barbirostris	98.3922	17.182128	Unclassified	GBIF: https://doi.org/10.15468/dl.zfaw79
Anopheles barbirostris	98.393148	8.248306	Unclassified	GBIF: https://doi.org/10.15468/dl.zfaw79
Anopheles barbirostris	98.403062	9.062374	Unclassified	GBIF: https://doi.org/10.15468/dl.zfaw79
Anopheles barbirostris	98.404687	8.422892	Unclassified	GBIF: https://doi.org/10.15468/dl.zfaw79
Anopheles barbirostris	98.407775	9.330989	Unclassified	GBIF: https://doi.org/10.15468/dl.zfaw79
Anopheles barbirostris	98.421046	9.008118	Unclassified	GBIF: https://doi.org/10.15468/dl.zfaw79
Anopheles barbirostris	98.428423	18.570699	Unclassified	GBIF: https://doi.org/10.15468/dl.zfaw79
Anopheles barbirostris	98.428512	19.31883	Unclassified	GBIF: https://doi.org/10.15468/dl.zfaw79
Anopheles barbirostris	98.429807	9.220681	Unclassified	GBIF: https://doi.org/10.15468/dl.zfaw79
Anopheles barbirostris	98.430765	17.098101	Unclassified	GBIF: https://doi.org/10.15468/dl.zfaw79
Anopheles barbirostris	98.431969	8.402129	Unclassified	GBIF: https://doi.org/10.15468/dl.zfaw79
Anopheles barbirostris	98.438505	8.277316	Unclassified	GBIF: https://doi.org/10.15468/dl.zfaw79
Anopheles barbirostris	98.440819	8.563142	Unclassified	GBIF: https://doi.org/10.15468/dl.zfaw79
Anopheles barbirostris	98.459221	18.420279	Unclassified	GBIF: https://doi.org/10.15468/dl.zfaw79
Anopheles barbirostris	98.461775	17.120149	Unclassified	GBIF: https://doi.org/10.15468/dl.zfaw79
Anopheles barbirostris	98.462582	8.492644	Unclassified	GBIF: https://doi.org/10.15468/dl.zfaw79
Anopheles barbirostris	98.466359	8.493532	Unclassified	GBIF: https://doi.org/10.15468/dl.zfaw79
Anopheles barbirostris	98.471798	8.502584	Unclassified	GBIF: https://doi.org/10.15468/dl.zfaw79
Anopheles barbirostris	98.485906	8.819177	Unclassified	GBIF: https://doi.org/10.15468/dl.zfaw79
Anopheles barbirostris	98.490191	17.788002	Unclassified	GBIF: https://doi.org/10.15468/dl.zfaw79
Anopheles barbirostris	98.492188	17.040433	Unclassified	GBIF: https://doi.org/10.15468/dl.zfaw79
Anopheles barbirostris	98.4925	16.981409	Unclassified	GBIF: https://doi.org/10.15468/dl.zfaw79
Anopheles barbirostris	98.498753	8.511742	Unclassified	GBIF: https://doi.org/10.15468/dl.zfaw79
Anopheles barbirostris	98.504416	16.70335	Unclassified	GBIF: https://doi.org/10.15468/dl.zfaw79
Anopheles barbirostris	98.507149	14.740746	Unclassified	GBIF: https://doi.org/10.15468/dl.zfaw79
Anopheles barbirostris	98.512302	16.9438	Unclassified	GBIF: https://doi.org/10.15468/dl.zfaw79
Anopheles barbirostris	98.515936	8.802031	Unclassified	GBIF: https://doi.org/10.15468/dl.zfaw79
Anopheles barbirostris	98.517059	17.055351	Unclassified	GBIF: https://doi.org/10.15468/dl.zfaw79
Anopheles barbirostris	98.527557	16.828149	Unclassified	GBIF: https://doi.org/10.15468/dl.zfaw79
Anopheles barbirostris	98.527969	17.01478	Unclassified	GBIF: https://doi.org/10.15468/dl.zfaw79
Anopheles barbirostris	98.53177	8.46466	Unclassified	GBIF: https://doi.org/10.15468/dl.zfaw79
Anopheles barbirostris	98.539884	17.069085	Unclassified	GBIF: https://doi.org/10.15468/dl.zfaw79
Anopheles barbirostris	98.555653	17.025727	Unclassified	GBIF: https://doi.org/10.15468/dl.zfaw79
Anopheles barbirostris	98.565348	8.531644	Unclassified	GBIF: https://doi.org/10.15468/dl.zfaw79
Anopheles barbirostris	98.574929	17.05002	Unclassified	GBIF: https://doi.org/10.15468/dl.zfaw79
Anopheles barbirostris	98.578127	16.879784	Unclassified	GBIF: https://doi.org/10.15468/dl.zfaw79
Anopheles barbirostris	98.5863	17.0803	Unclassified	GBIF: https://doi.org/10.15468/dl.zfaw79
Anopheles barbirostris	98.588792	17.155507	Unclassified	GBIF: https://doi.org/10.15468/dl.zfaw79

Anopheles barbirostris	98.59043	17.121171	Unclassified	GBIF: https://doi.org/10.15468/dl.zfaw79
Anopheles barbirostris	98.601544	16.028639	Unclassified	GBIF: https://doi.org/10.15468/dl.zfaw79
Anopheles barbirostris	98.601768	16.798476	Unclassified	GBIF: https://doi.org/10.15468/dl.zfaw79
Anopheles barbirostris	98.605142	17.026647	Unclassified	GBIF: https://doi.org/10.15468/dl.zfaw79
Anopheles barbirostris	98.615629	14.940706	Unclassified	GBIF: https://doi.org/10.15468/dl.zfaw79
Anopheles barbirostris	98.623192	16.879872	Unclassified	GBIF: https://doi.org/10.15468/dl.zfaw79
Anopheles barbirostris	98.62446	8.58293	Unclassified	GBIF: https://doi.org/10.15468/dl.zfaw79
Anopheles barbirostris	98.635298	19.646503	Unclassified	GBIF: https://doi.org/10.15468/dl.zfaw79
Anopheles barbirostris	98.635782	17.166444	Unclassified	GBIF: https://doi.org/10.15468/dl.zfaw79
Anopheles barbirostris	98.646075	17.192676	Unclassified	GBIF: https://doi.org/10.15468/dl.zfaw79
Anopheles barbirostris	98.647404	16.47584	Unclassified	GBIF: https://doi.org/10.15468/dl.zfaw79
Anopheles barbirostris	98.650163	16.504772	Unclassified	GBIF: https://doi.org/10.15468/dl.zfaw79
Anopheles barbirostris	98.653801	16.033791	Unclassified	GBIF: https://doi.org/10.15468/dl.zfaw79
Anopheles barbirostris	98.659846	8.533548	Unclassified	GBIF: https://doi.org/10.15468/dl.zfaw79
Anopheles barbirostris	98.66423	18.50429	Unclassified	GBIF: https://doi.org/10.15468/dl.zfaw79
Anopheles barbirostris	98.668094	16.42977	Unclassified	GBIF: https://doi.org/10.15468/dl.zfaw79
Anopheles barbirostris	98.668482	9.687029	Unclassified	GBIF: https://doi.org/10.15468/dl.zfaw79
Anopheles barbirostris	98.670826	9.773319	Unclassified	GBIF: https://doi.org/10.15468/dl.zfaw79
Anopheles barbirostris	98.673505	14.79252	Unclassified	GBIF: https://doi.org/10.15468/dl.zfaw79
Anopheles barbirostris	98.680047	15.98863	Unclassified	GBIF: https://doi.org/10.15468/dl.zfaw79
Anopheles barbirostris	98.682859	9.756881	Unclassified	GBIF: https://doi.org/10.15468/dl.zfaw79
Anopheles barbirostris	98.684802	16.523811	Unclassified	GBIF: https://doi.org/10.15468/dl.zfaw79
Anopheles barbirostris	98.689719	16.378275	Unclassified	GBIF: https://doi.org/10.15468/dl.zfaw79
Anopheles barbirostris	98.692486	16.405399	Unclassified	GBIF: https://doi.org/10.15468/dl.zfaw79
Anopheles barbirostris	98.693435	16.397265	Unclassified	GBIF: https://doi.org/10.15468/dl.zfaw79
Anopheles barbirostris	98.69414	9.778821	Unclassified	GBIF: https://doi.org/10.15468/dl.zfaw79
Anopheles barbirostris	98.694385	16.38913	Unclassified	GBIF: https://doi.org/10.15468/dl.zfaw79
Anopheles barbirostris	98.696078	16.504844	Unclassified	GBIF: https://doi.org/10.15468/dl.zfaw79
Anopheles barbirostris	98.700735	16.522931	Unclassified	GBIF: https://doi.org/10.15468/dl.zfaw79
Anopheles barbirostris	98.701922	9.761124	Unclassified	GBIF: https://doi.org/10.15468/dl.zfaw79
Anopheles barbirostris	98.703903	16.286991	Unclassified	GBIF: https://doi.org/10.15468/dl.zfaw79
Anopheles barbirostris	98.704053	18.189085	Unclassified	GBIF: https://doi.org/10.15468/dl.zfaw79
Anopheles barbirostris	98.710439	16.297848	Unclassified	GBIF: https://doi.org/10.15468/dl.zfaw79
Anopheles barbirostris	98.714214	9.727315	Unclassified	GBIF: https://doi.org/10.15468/dl.zfaw79
Anopheles barbirostris	98.719566	16.461485	Unclassified	GBIF: https://doi.org/10.15468/dl.zfaw79
Anopheles barbirostris	98.719946	14.674302	Unclassified	GBIF: https://doi.org/10.15468/dl.zfaw79
Anopheles barbirostris	98.720646	16.361143	Unclassified	GBIF: https://doi.org/10.15468/dl.zfaw79
Anopheles barbirostris	98.720925	14.670656	Unclassified	GBIF: https://doi.org/10.15468/dl.zfaw79
Anopheles barbirostris	98.72352	16.315042	Unclassified	GBIF: https://doi.org/10.15468/dl.zfaw79
Anopheles barbirostris	98.731831	16.398221	Unclassified	GBIF: https://doi.org/10.15468/dl.zfaw79
Anopheles barbirostris	98.73197	9.747006	Unclassified	GBIF: https://doi.org/10.15468/dl.zfaw79
Anopheles barbirostris	98.732199	16.809535	Unclassified	GBIF: https://doi.org/10.15468/dl.zfaw79

Anopheles barbirostris	98.733785	16.33856	Unclassified	GBIF: https://doi.org/10.15468/dl.zfaw79
Anopheles barbirostris	98.737832	16.807734	Unclassified	GBIF: https://doi.org/10.15468/dl.zfaw79
Anopheles barbirostris	98.739244	16.457895	Unclassified	GBIF: https://doi.org/10.15468/dl.zfaw79
Anopheles barbirostris	98.743875	16.414867	Unclassified	GBIF: https://doi.org/10.15468/dl.zfaw79
Anopheles barbirostris	98.752602	9.677865	Unclassified	GBIF: https://doi.org/10.15468/dl.zfaw79
Anopheles barbirostris	98.754286	14.714072	Unclassified	GBIF: https://doi.org/10.15468/dl.zfaw79
Anopheles barbirostris	98.760464	16.365653	Unclassified	GBIF: https://doi.org/10.15468/dl.zfaw79
Anopheles barbirostris	98.763621	16.426822	Unclassified	GBIF: https://doi.org/10.15468/dl.zfaw79
Anopheles barbirostris	98.769608	16.918056	Unclassified	GBIF: https://doi.org/10.15468/dl.zfaw79
Anopheles barbirostris	98.787977	16.445064	Unclassified	GBIF: https://doi.org/10.15468/dl.zfaw79
Anopheles barbirostris	98.789103	9.74699	Unclassified	GBIF: https://doi.org/10.15468/dl.zfaw79
Anopheles barbirostris	98.789505	16.674094	Unclassified	GBIF: https://doi.org/10.15468/dl.zfaw79
Anopheles barbirostris	98.797448	18.578654	Unclassified	GBIF: https://doi.org/10.15468/dl.zfaw79
Anopheles barbirostris	98.798255	18.539229	Unclassified	GBIF: https://doi.org/10.15468/dl.zfaw79
Anopheles barbirostris	98.799267	9.827039	Unclassified	GBIF: https://doi.org/10.15468/dl.zfaw79
Anopheles barbirostris	98.799834	16.418367	Unclassified	GBIF: https://doi.org/10.15468/dl.zfaw79
Anopheles barbirostris	98.800258	9.814136	Unclassified	GBIF: https://doi.org/10.15468/dl.zfaw79
Anopheles barbirostris	98.800365	9.814721	Unclassified	GBIF: https://doi.org/10.15468/dl.zfaw79
Anopheles barbirostris	98.801398	9.792276	Unclassified	GBIF: https://doi.org/10.15468/dl.zfaw79
Anopheles barbirostris	98.807778	3.531111	Unclassified	GBIF: https://doi.org/10.15468/dl.zfaw79
Anopheles barbirostris	98.809225	16.741822	Unclassified	GBIF: https://doi.org/10.15468/dl.zfaw79
Anopheles barbirostris	98.8133	8.855	Unclassified	GBIF: https://doi.org/10.15468/dl.zfaw79
Anopheles barbirostris	98.823354	18.524566	Unclassified	GBIF: https://doi.org/10.15468/dl.zfaw79
Anopheles barbirostris	98.827227	9.763324	Unclassified	GBIF: https://doi.org/10.15468/dl.zfaw79
Anopheles barbirostris	98.83079	9.825765	Unclassified	GBIF: https://doi.org/10.15468/dl.zfaw79
Anopheles barbirostris	98.832284	9.840487	Unclassified	GBIF: https://doi.org/10.15468/dl.zfaw79
Anopheles barbirostris	98.839905	9.867426	Unclassified	GBIF: https://doi.org/10.15468/dl.zfaw79
Anopheles barbirostris	98.847827	9.891034	Unclassified	GBIF: https://doi.org/10.15468/dl.zfaw79
Anopheles barbirostris	98.86661	9.889881	Unclassified	GBIF: https://doi.org/10.15468/dl.zfaw79
Anopheles barbirostris	98.88308	18.608419	Unclassified	GBIF: https://doi.org/10.15468/dl.zfaw79
Anopheles barbirostris	98.889634	10.123784	Unclassified	GBIF: https://doi.org/10.15468/dl.zfaw79
Anopheles barbirostris	98.889704	18.627403	Unclassified	GBIF: https://doi.org/10.15468/dl.zfaw79
Anopheles barbirostris	98.889884	18.540126	Unclassified	GBIF: https://doi.org/10.15468/dl.zfaw79
Anopheles barbirostris	98.89701	10.22258	Unclassified	GBIF: https://doi.org/10.15468/dl.zfaw79
Anopheles barbirostris	98.899195	10.120829	Unclassified	GBIF: https://doi.org/10.15468/dl.zfaw79
Anopheles barbirostris	98.90083	18.90584	Unclassified	GBIF: https://doi.org/10.15468/dl.zfaw79
Anopheles barbirostris	98.902384	10.208372	Unclassified	GBIF: https://doi.org/10.15468/dl.zfaw79
Anopheles barbirostris	98.90558	18.91308	Unclassified	GBIF: https://doi.org/10.15468/dl.zfaw79
Anopheles barbirostris	98.907484	10.131621	Unclassified	GBIF: https://doi.org/10.15468/dl.zfaw79
Anopheles barbirostris	98.908454	10.231891	Unclassified	GBIF: https://doi.org/10.15468/dl.zfaw79
Anopheles barbirostris	98.908575	10.141897	Unclassified	GBIF: https://doi.org/10.15468/dl.zfaw79
Anopheles barbirostris	98.91442	10.007	Unclassified	GBIF: https://doi.org/10.15468/dl.zfaw79

Anopheles barbirostris	98.914825	18.556314	Unclassified	GBIF: https://doi.org/10.15468/dl.zfaw79
Anopheles barbirostris	98.91691	10.23508	Unclassified	GBIF: https://doi.org/10.15468/dl.zfaw79
Anopheles barbirostris	98.924014	10.245558	Unclassified	GBIF: https://doi.org/10.15468/dl.zfaw79
Anopheles barbirostris	98.926143	10.227229	Unclassified	GBIF: https://doi.org/10.15468/dl.zfaw79
Anopheles barbirostris	98.927016	10.14075	Unclassified	GBIF: https://doi.org/10.15468/dl.zfaw79
Anopheles barbirostris	98.933523	10.198913	Unclassified	GBIF: https://doi.org/10.15468/dl.zfaw79
Anopheles barbirostris	98.934357	10.207495	Unclassified	GBIF: https://doi.org/10.15468/dl.zfaw79
Anopheles barbirostris	98.93458	10.489564	Unclassified	GBIF: https://doi.org/10.15468/dl.zfaw79
Anopheles barbirostris	98.93491	10.16105	Unclassified	GBIF: https://doi.org/10.15468/dl.zfaw79
Anopheles barbirostris	98.938648	19.587218	Unclassified	GBIF: https://doi.org/10.15468/dl.zfaw79
Anopheles barbirostris	98.94455	18.81187	Unclassified	GBIF: https://doi.org/10.15468/dl.zfaw79
Anopheles barbirostris	98.945507	10.328036	Unclassified	GBIF: https://doi.org/10.15468/dl.zfaw79
Anopheles barbirostris	98.94566	10.193877	Unclassified	GBIF: https://doi.org/10.15468/dl.zfaw79
Anopheles barbirostris	98.952698	10.263799	Unclassified	GBIF: https://doi.org/10.15468/dl.zfaw79
Anopheles barbirostris	98.9533	9.03	Unclassified	GBIF: https://doi.org/10.15468/dl.zfaw79
Anopheles barbirostris	98.958039	10.515357	Unclassified	GBIF: https://doi.org/10.15468/dl.zfaw79
Anopheles barbirostris	98.96256	18.87514	Unclassified	GBIF: https://doi.org/10.15468/dl.zfaw79
Anopheles barbirostris	98.96256	18.87695	Unclassified	GBIF: https://doi.org/10.15468/dl.zfaw79
Anopheles barbirostris	98.96731	9.96144	Unclassified	GBIF: https://doi.org/10.15468/dl.zfaw79
Anopheles barbirostris	98.972071	10.767742	Unclassified	GBIF: https://doi.org/10.15468/dl.zfaw79
Anopheles barbirostris	98.975234	10.725473	Unclassified	GBIF: https://doi.org/10.15468/dl.zfaw79
Anopheles barbirostris	98.977655	10.13297	Unclassified	GBIF: https://doi.org/10.15468/dl.zfaw79
Anopheles barbirostris	98.97885	9.8723	Unclassified	GBIF: https://doi.org/10.15468/dl.zfaw79
Anopheles barbirostris	98.9789	9.23889	Unclassified	GBIF: https://doi.org/10.15468/dl.zfaw79
Anopheles barbirostris	98.97972	10.213745	Unclassified	GBIF: https://doi.org/10.15468/dl.zfaw79
Anopheles barbirostris	98.980197	10.795057	Unclassified	GBIF: https://doi.org/10.15468/dl.zfaw79
Anopheles barbirostris	98.98251	9.91825	Unclassified	GBIF: https://doi.org/10.15468/dl.zfaw79
Anopheles barbirostris	98.98257	19.16918	Unclassified	GBIF: https://doi.org/10.15468/dl.zfaw79
Anopheles barbirostris	98.98494	10.35961	Unclassified	GBIF: https://doi.org/10.15468/dl.zfaw79
Anopheles barbirostris	98.985302	10.504337	Unclassified	GBIF: https://doi.org/10.15468/dl.zfaw79
Anopheles barbirostris	98.988056	14.335333	Unclassified	GBIF: https://doi.org/10.15468/dl.zfaw79
Anopheles barbirostris	98.988139	14.335778	Unclassified	GBIF: https://doi.org/10.15468/dl.zfaw79
Anopheles barbirostris	98.98845	9.88797	Unclassified	GBIF: https://doi.org/10.15468/dl.zfaw79
Anopheles barbirostris	98.988667	14.335583	Unclassified	GBIF: https://doi.org/10.15468/dl.zfaw79
Anopheles barbirostris	98.988669	14.336786	Unclassified	GBIF: https://doi.org/10.15468/dl.zfaw79
Anopheles barbirostris	98.99154	9.73591	Unclassified	GBIF: https://doi.org/10.15468/dl.zfaw79
Anopheles barbirostris	98.991845	10.762198	Unclassified	GBIF: https://doi.org/10.15468/dl.zfaw79
Anopheles barbirostris	98.992975	10.261541	Unclassified	GBIF: https://doi.org/10.15468/dl.zfaw79
Anopheles barbirostris	98.995442	10.389929	Unclassified	GBIF: https://doi.org/10.15468/dl.zfaw79
Anopheles barbirostris	98.998231	10.227831	Unclassified	GBIF: https://doi.org/10.15468/dl.zfaw79
Anopheles barbirostris	98.998904	14.49628	Unclassified	GBIF: https://doi.org/10.15468/dl.zfaw79
Anopheles barbirostris	99.00054	18.79561	Unclassified	GBIF: https://doi.org/10.15468/dl.zfaw79

Anopheles barbirostris	99.001513	10.247715	Unclassified	GBIF: https://doi.org/10.15468/dl.zfaw79
Anopheles barbirostris	99.001515	10.516334	Unclassified	GBIF: https://doi.org/10.15468/dl.zfaw79
Anopheles barbirostris	99.00155	9.90412	Unclassified	GBIF: https://doi.org/10.15468/dl.zfaw79
Anopheles barbirostris	99.005085	10.074342	Unclassified	GBIF: https://doi.org/10.15468/dl.zfaw79
Anopheles barbirostris	99.005167	10.320975	Unclassified	GBIF: https://doi.org/10.15468/dl.zfaw79
Anopheles barbirostris	99.00538	9.92306	Unclassified	GBIF: https://doi.org/10.15468/dl.zfaw79
Anopheles barbirostris	99.005419	10.781285	Unclassified	GBIF: https://doi.org/10.15468/dl.zfaw79
Anopheles barbirostris	99.007111	10.710313	Unclassified	GBIF: https://doi.org/10.15468/dl.zfaw79
Anopheles barbirostris	99.007533	10.103959	Unclassified	GBIF: https://doi.org/10.15468/dl.zfaw79
Anopheles barbirostris	99.01705	10.493723	Unclassified	GBIF: https://doi.org/10.15468/dl.zfaw79
Anopheles barbirostris	99.017267	10.418451	Unclassified	GBIF: https://doi.org/10.15468/dl.zfaw79
Anopheles barbirostris	99.019064	10.246246	Unclassified	GBIF: https://doi.org/10.15468/dl.zfaw79
Anopheles barbirostris	99.023304	10.387686	Unclassified	GBIF: https://doi.org/10.15468/dl.zfaw79
Anopheles barbirostris	99.026978	9.791408	Unclassified	GBIF: https://doi.org/10.15468/dl.zfaw79
Anopheles barbirostris	99.028919	10.362578	Unclassified	GBIF: https://doi.org/10.15468/dl.zfaw79
Anopheles barbirostris	99.032063	10.172115	Unclassified	GBIF: https://doi.org/10.15468/dl.zfaw79
Anopheles barbirostris	99.03216	10.098729	Unclassified	GBIF: https://doi.org/10.15468/dl.zfaw79
Anopheles barbirostris	99.032913	10.140303	Unclassified	GBIF: https://doi.org/10.15468/dl.zfaw79
Anopheles barbirostris	99.03435	9.98155	Unclassified	GBIF: https://doi.org/10.15468/dl.zfaw79
Anopheles barbirostris	99.034395	10.322782	Unclassified	GBIF: https://doi.org/10.15468/dl.zfaw79
Anopheles barbirostris	99.034918	19.420037	Unclassified	GBIF: https://doi.org/10.15468/dl.zfaw79
Anopheles barbirostris	99.04182	10.841428	Unclassified	GBIF: https://doi.org/10.15468/dl.zfaw79
Anopheles barbirostris	99.042069	10.841473	Unclassified	GBIF: https://doi.org/10.15468/dl.zfaw79
Anopheles barbirostris	99.043353	9.7952	Unclassified	GBIF: https://doi.org/10.15468/dl.zfaw79
Anopheles barbirostris	99.045168	16.528478	Unclassified	GBIF: https://doi.org/10.15468/dl.zfaw79
Anopheles barbirostris	99.045396	10.621095	Unclassified	GBIF: https://doi.org/10.15468/dl.zfaw79
Anopheles barbirostris	99.045745	10.449499	Unclassified	GBIF: https://doi.org/10.15468/dl.zfaw79
Anopheles barbirostris	99.046281	10.406894	Unclassified	GBIF: https://doi.org/10.15468/dl.zfaw79
Anopheles barbirostris	99.046629	10.088851	Unclassified	GBIF: https://doi.org/10.15468/dl.zfaw79
Anopheles barbirostris	99.050601	10.860462	Unclassified	GBIF: https://doi.org/10.15468/dl.zfaw79
Anopheles barbirostris	99.051912	9.730896	Unclassified	GBIF: https://doi.org/10.15468/dl.zfaw79
Anopheles barbirostris	99.056022	10.426884	Unclassified	GBIF: https://doi.org/10.15468/dl.zfaw79
Anopheles barbirostris	99.056241	9.893162	Unclassified	GBIF: https://doi.org/10.15468/dl.zfaw79
Anopheles barbirostris	99.056348	10.501858	Unclassified	GBIF: https://doi.org/10.15468/dl.zfaw79
Anopheles barbirostris	99.056372	10.63481	Unclassified	GBIF: https://doi.org/10.15468/dl.zfaw79
Anopheles barbirostris	99.058182	10.536226	Unclassified	GBIF: https://doi.org/10.15468/dl.zfaw79
Anopheles barbirostris	99.059934	10.133748	Unclassified	GBIF: https://doi.org/10.15468/dl.zfaw79
Anopheles barbirostris	99.06229	10.37095	Unclassified	GBIF: https://doi.org/10.15468/dl.zfaw79
Anopheles barbirostris	99.062369	10.537866	Unclassified	GBIF: https://doi.org/10.15468/dl.zfaw79
Anopheles barbirostris	99.062502	10.288631	Unclassified	GBIF: https://doi.org/10.15468/dl.zfaw79
Anopheles barbirostris	99.065092	10.721264	Unclassified	GBIF: https://doi.org/10.15468/dl.zfaw79
Anopheles barbirostris	99.065485	10.492812	Unclassified	GBIF: https://doi.org/10.15468/dl.zfaw79

Anopheles barbirostris	99.06551	10.609484	Unclassified	GBIF: https://doi.org/10.15468/dl.zfaw79
Anopheles barbirostris	99.069993	10.215147	Unclassified	GBIF: https://doi.org/10.15468/dl.zfaw79
Anopheles barbirostris	99.075506	10.361666	Unclassified	GBIF: https://doi.org/10.15468/dl.zfaw79
Anopheles barbirostris	99.075515	10.398748	Unclassified	GBIF: https://doi.org/10.15468/dl.zfaw79
Anopheles barbirostris	99.079223	10.411568	Unclassified	GBIF: https://doi.org/10.15468/dl.zfaw79
Anopheles barbirostris	99.081334	9.951966	Unclassified	GBIF: https://doi.org/10.15468/dl.zfaw79
Anopheles barbirostris	99.082009	10.772276	Unclassified	GBIF: https://doi.org/10.15468/dl.zfaw79
Anopheles barbirostris	99.083573	10.574847	Unclassified	GBIF: https://doi.org/10.15468/dl.zfaw79
Anopheles barbirostris	99.08692	10.56566	Unclassified	GBIF: https://doi.org/10.15468/dl.zfaw79
Anopheles barbirostris	99.093247	10.075561	Unclassified	GBIF: https://doi.org/10.15468/dl.zfaw79
Anopheles barbirostris	99.095757	9.721792	Unclassified	GBIF: https://doi.org/10.15468/dl.zfaw79
Anopheles barbirostris	99.097464	10.473811	Unclassified	GBIF: https://doi.org/10.15468/dl.zfaw79
Anopheles barbirostris	99.098238	10.440082	Unclassified	GBIF: https://doi.org/10.15468/dl.zfaw79
Anopheles barbirostris	99.098669	10.053614	Unclassified	GBIF: https://doi.org/10.15468/dl.zfaw79
Anopheles barbirostris	99.10014	10.50879	Unclassified	GBIF: https://doi.org/10.15468/dl.zfaw79
Anopheles barbirostris	99.104758	10.42316	Unclassified	GBIF: https://doi.org/10.15468/dl.zfaw79
Anopheles barbirostris	99.105666	10.407784	Unclassified	GBIF: https://doi.org/10.15468/dl.zfaw79
Anopheles barbirostris	99.106562	10.354422	Unclassified	GBIF: https://doi.org/10.15468/dl.zfaw79
Anopheles barbirostris	99.107244	10.618508	Unclassified	GBIF: https://doi.org/10.15468/dl.zfaw79
Anopheles barbirostris	99.10809	10.6182	Unclassified	GBIF: https://doi.org/10.15468/dl.zfaw79
Anopheles barbirostris	99.110267	10.50094	Unclassified	GBIF: https://doi.org/10.15468/dl.zfaw79
Anopheles barbirostris	99.110336	10.371534	Unclassified	GBIF: https://doi.org/10.15468/dl.zfaw79
Anopheles barbirostris	99.111026	16.427828	Unclassified	GBIF: https://doi.org/10.15468/dl.zfaw79
Anopheles barbirostris	99.11209	10.488277	Unclassified	GBIF: https://doi.org/10.15468/dl.zfaw79
Anopheles barbirostris	99.113393	10.504991	Unclassified	GBIF: https://doi.org/10.15468/dl.zfaw79
Anopheles barbirostris	99.116314	10.798231	Unclassified	GBIF: https://doi.org/10.15468/dl.zfaw79
Anopheles barbirostris	99.116609	10.35261	Unclassified	GBIF: https://doi.org/10.15468/dl.zfaw79
Anopheles barbirostris	99.118274	16.522995	Unclassified	GBIF: https://doi.org/10.15468/dl.zfaw79
Anopheles barbirostris	99.118551	10.653785	Unclassified	GBIF: https://doi.org/10.15468/dl.zfaw79
Anopheles barbirostris	99.118995	10.549008	Unclassified	GBIF: https://doi.org/10.15468/dl.zfaw79
Anopheles barbirostris	99.119516	10.842566	Unclassified	GBIF: https://doi.org/10.15468/dl.zfaw79
Anopheles barbirostris	99.119962	10.511094	Unclassified	GBIF: https://doi.org/10.15468/dl.zfaw79
Anopheles barbirostris	99.123269	14.052543	Unclassified	GBIF: https://doi.org/10.15468/dl.zfaw79
Anopheles barbirostris	99.125713	10.477258	Unclassified	GBIF: https://doi.org/10.15468/dl.zfaw79
Anopheles barbirostris	99.126278	15.158887	Unclassified	GBIF: https://doi.org/10.15468/dl.zfaw79
Anopheles barbirostris	99.126704	10.469278	Unclassified	GBIF: https://doi.org/10.15468/dl.zfaw79
Anopheles barbirostris	99.127358	10.765704	Unclassified	GBIF: https://doi.org/10.15468/dl.zfaw79
Anopheles barbirostris	99.127583	10.383356	Unclassified	GBIF: https://doi.org/10.15468/dl.zfaw79
Anopheles barbirostris	99.128413	14.026883	Unclassified	GBIF: https://doi.org/10.15468/dl.zfaw79
Anopheles barbirostris	99.128996	10.458158	Unclassified	GBIF: https://doi.org/10.15468/dl.zfaw79
Anopheles barbirostris	99.129484	10.560625	Unclassified	GBIF: https://doi.org/10.15468/dl.zfaw79
Anopheles barbirostris	99.131592	10.47153	Unclassified	GBIF: https://doi.org/10.15468/dl.zfaw79

Anopheles barbirostris	99.135314	9.793432	Unclassified	GBIF: https://doi.org/10.15468/dl.zfaw79
Anopheles barbirostris	99.136015	10.726162	Unclassified	GBIF: https://doi.org/10.15468/dl.zfaw79
Anopheles barbirostris	99.136612	16.662018	Unclassified	GBIF: https://doi.org/10.15468/dl.zfaw79
Anopheles barbirostris	99.145206	10.944996	Unclassified	GBIF: https://doi.org/10.15468/dl.zfaw79
Anopheles barbirostris	99.14522	10.5501	Unclassified	GBIF: https://doi.org/10.15468/dl.zfaw79
Anopheles barbirostris	99.145698	10.884027	Unclassified	GBIF: https://doi.org/10.15468/dl.zfaw79
Anopheles barbirostris	99.151931	13.89761	Unclassified	GBIF: https://doi.org/10.15468/dl.zfaw79
Anopheles barbirostris	99.162173	10.820339	Unclassified	GBIF: https://doi.org/10.15468/dl.zfaw79
Anopheles barbirostris	99.163427	10.537782	Unclassified	GBIF: https://doi.org/10.15468/dl.zfaw79
Anopheles barbirostris	99.168901	10.765006	Unclassified	GBIF: https://doi.org/10.15468/dl.zfaw79
Anopheles barbirostris	99.169124	10.92481	Unclassified	GBIF: https://doi.org/10.15468/dl.zfaw79
Anopheles barbirostris	99.170721	10.74963	Unclassified	GBIF: https://doi.org/10.15468/dl.zfaw79
Anopheles barbirostris	99.171917	10.842589	Unclassified	GBIF: https://doi.org/10.15468/dl.zfaw79
Anopheles barbirostris	99.178055	10.778567	Unclassified	GBIF: https://doi.org/10.15468/dl.zfaw79
Anopheles barbirostris	99.178938	-1.627301	Unclassified	GBIF: https://doi.org/10.15468/dl.zfaw79
Anopheles barbirostris	99.179473	16.557488	Unclassified	GBIF: https://doi.org/10.15468/dl.zfaw79
Anopheles barbirostris	99.180595	13.914555	Unclassified	GBIF: https://doi.org/10.15468/dl.zfaw79
Anopheles barbirostris	99.181219	10.671592	Unclassified	GBIF: https://doi.org/10.15468/dl.zfaw79
Anopheles barbirostris	99.18506	14.21763	Unclassified	GBIF: https://doi.org/10.15468/dl.zfaw79
Anopheles barbirostris	99.190995	10.780432	Unclassified	GBIF: https://doi.org/10.15468/dl.zfaw79
Anopheles barbirostris	99.19213	16.500795	Unclassified	GBIF: https://doi.org/10.15468/dl.zfaw79
Anopheles barbirostris	99.201877	10.857277	Unclassified	GBIF: https://doi.org/10.15468/dl.zfaw79
Anopheles barbirostris	99.211781	14.046716	Unclassified	GBIF: https://doi.org/10.15468/dl.zfaw79
Anopheles barbirostris	99.211901	10.781259	Unclassified	GBIF: https://doi.org/10.15468/dl.zfaw79
Anopheles barbirostris	99.222021	16.522682	Unclassified	GBIF: https://doi.org/10.15468/dl.zfaw79
Anopheles barbirostris	99.224479	10.905932	Unclassified	GBIF: https://doi.org/10.15468/dl.zfaw79
Anopheles barbirostris	99.228177	16.384708	Unclassified	GBIF: https://doi.org/10.15468/dl.zfaw79
Anopheles barbirostris	99.228932	13.888759	Unclassified	GBIF: https://doi.org/10.15468/dl.zfaw79
Anopheles barbirostris	99.23568	10.871475	Unclassified	GBIF: https://doi.org/10.15468/dl.zfaw79
Anopheles barbirostris	99.236468	10.8714	Unclassified	GBIF: https://doi.org/10.15468/dl.zfaw79
Anopheles barbirostris	99.237565	16.403682	Unclassified	GBIF: https://doi.org/10.15468/dl.zfaw79
Anopheles barbirostris	99.239482	10.955789	Unclassified	GBIF: https://doi.org/10.15468/dl.zfaw79
Anopheles barbirostris	99.240437	16.460193	Unclassified	GBIF: https://doi.org/10.15468/dl.zfaw79
Anopheles barbirostris	99.254742	13.458063	Unclassified	GBIF: https://doi.org/10.15468/dl.zfaw79
Anopheles barbirostris	99.257729	10.888848	Unclassified	GBIF: https://doi.org/10.15468/dl.zfaw79
Anopheles barbirostris	99.268212	13.988792	Unclassified	GBIF: https://doi.org/10.15468/dl.zfaw79
Anopheles barbirostris	99.274324	13.37981	Unclassified	GBIF: https://doi.org/10.15468/dl.zfaw79
Anopheles barbirostris	99.276019	10.901084	Unclassified	GBIF: https://doi.org/10.15468/dl.zfaw79
Anopheles barbirostris	99.278195	10.798224	Unclassified	GBIF: https://doi.org/10.15468/dl.zfaw79
Anopheles barbirostris	99.280002	13.783525	Unclassified	GBIF: https://doi.org/10.15468/dl.zfaw79
Anopheles barbirostris	99.28079	15.150611	Unclassified	GBIF: https://doi.org/10.15468/dl.zfaw79
Anopheles barbirostris	99.282505	16.393683	Unclassified	GBIF: https://doi.org/10.15468/dl.zfaw79

Anopheles barbirostris	99.28559	10.923141	Unclassified	GBIF: https://doi.org/10.15468/dl.zfaw79
Anopheles barbirostris	99.286853	10.628456	Unclassified	GBIF: https://doi.org/10.15468/dl.zfaw79
Anopheles barbirostris	99.29494	10.9579	Unclassified	GBIF: https://doi.org/10.15468/dl.zfaw79
Anopheles barbirostris	99.296171	13.616696	Unclassified	GBIF: https://doi.org/10.15468/dl.zfaw79
Anopheles barbirostris	99.296317	10.939305	Unclassified	GBIF: https://doi.org/10.15468/dl.zfaw79
Anopheles barbirostris	99.305819	16.33037	Unclassified	GBIF: https://doi.org/10.15468/dl.zfaw79
Anopheles barbirostris	99.311222	13.45774	Unclassified	GBIF: https://doi.org/10.15468/dl.zfaw79
Anopheles barbirostris	99.316465	16.600952	Unclassified	GBIF: https://doi.org/10.15468/dl.zfaw79
Anopheles barbirostris	99.318085	10.854425	Unclassified	GBIF: https://doi.org/10.15468/dl.zfaw79
Anopheles barbirostris	99.321218	14.763239	Unclassified	GBIF: https://doi.org/10.15468/dl.zfaw79
Anopheles barbirostris	99.321609	10.7269	Unclassified	GBIF: https://doi.org/10.15468/dl.zfaw79
Anopheles barbirostris	99.322367	10.865695	Unclassified	GBIF: https://doi.org/10.15468/dl.zfaw79
Anopheles barbirostris	99.330744	10.716942	Unclassified	GBIF: https://doi.org/10.15468/dl.zfaw79
Anopheles barbirostris	99.3317	14.793109	Unclassified	GBIF: https://doi.org/10.15468/dl.zfaw79
Anopheles barbirostris	99.334443	10.754018	Unclassified	GBIF: https://doi.org/10.15468/dl.zfaw79
Anopheles barbirostris	99.336998	14.78976	Unclassified	GBIF: https://doi.org/10.15468/dl.zfaw79
Anopheles barbirostris	99.337604	19.772169	Unclassified	GBIF: https://doi.org/10.15468/dl.zfaw79
Anopheles barbirostris	99.343494	10.931574	Unclassified	GBIF: https://doi.org/10.15468/dl.zfaw79
Anopheles barbirostris	99.347572	10.921539	Unclassified	GBIF: https://doi.org/10.15468/dl.zfaw79
Anopheles barbirostris	99.358923	14.772592	Unclassified	GBIF: https://doi.org/10.15468/dl.zfaw79
Anopheles barbirostris	99.359444	0.798056	Unclassified	GBIF: https://doi.org/10.15468/dl.zfaw79
Anopheles barbirostris	99.360871	10.673497	Unclassified	GBIF: https://doi.org/10.15468/dl.zfaw79
Anopheles barbirostris	99.361265	14.392985	Unclassified	GBIF: https://doi.org/10.15468/dl.zfaw79
Anopheles barbirostris	99.361599	10.960042	Unclassified	GBIF: https://doi.org/10.15468/dl.zfaw79
Anopheles barbirostris	99.365582	10.789255	Unclassified	GBIF: https://doi.org/10.15468/dl.zfaw79
Anopheles barbirostris	99.370419	13.421992	Unclassified	GBIF: https://doi.org/10.15468/dl.zfaw79
Anopheles barbirostris	99.371973	10.776531	Unclassified	GBIF: https://doi.org/10.15468/dl.zfaw79
Anopheles barbirostris	99.378541	14.028376	Unclassified	GBIF: https://doi.org/10.15468/dl.zfaw79
Anopheles barbirostris	99.379248	14.49769	Unclassified	GBIF: https://doi.org/10.15468/dl.zfaw79
Anopheles barbirostris	99.379358	13.671355	Unclassified	GBIF: https://doi.org/10.15468/dl.zfaw79
Anopheles barbirostris	99.379726	15.934121	Unclassified	GBIF: https://doi.org/10.15468/dl.zfaw79
Anopheles barbirostris	99.382518	14.75967	Unclassified	GBIF: https://doi.org/10.15468/dl.zfaw79
Anopheles barbirostris	99.384151	19.971647	Unclassified	GBIF: https://doi.org/10.15468/dl.zfaw79
Anopheles barbirostris	99.38563	14.664028	Unclassified	GBIF: https://doi.org/10.15468/dl.zfaw79
Anopheles barbirostris	99.386491	13.695979	Unclassified	GBIF: https://doi.org/10.15468/dl.zfaw79
Anopheles barbirostris	99.399574	13.461019	Unclassified	GBIF: https://doi.org/10.15468/dl.zfaw79
Anopheles barbirostris	99.406118	11.011515	Unclassified	GBIF: https://doi.org/10.15468/dl.zfaw79
Anopheles barbirostris	99.409712	14.634444	Unclassified	GBIF: https://doi.org/10.15468/dl.zfaw79
Anopheles barbirostris	99.41094	16.986531	Unclassified	GBIF: https://doi.org/10.15468/dl.zfaw79
Anopheles barbirostris	99.415127	13.35709	Unclassified	GBIF: https://doi.org/10.15468/dl.zfaw79
Anopheles barbirostris	99.453259	13.330879	Unclassified	GBIF: https://doi.org/10.15468/dl.zfaw79
Anopheles barbirostris	99.457395	15.464941	Unclassified	GBIF: https://doi.org/10.15468/dl.zfaw79

Anopheles barbirostris	99.461506	15.135703	Unclassified	GBIF: https://doi.org/10.15468/dl.zfaw79
Anopheles barbirostris	99.46518	13.314495	Unclassified	GBIF: https://doi.org/10.15468/dl.zfaw79
Anopheles barbirostris	99.479395	15.302162	Unclassified	GBIF: https://doi.org/10.15468/dl.zfaw79
Anopheles barbirostris	99.496263	11.223328	Unclassified	GBIF: https://doi.org/10.15468/dl.zfaw79
Anopheles barbirostris	99.55524	12.774639	Unclassified	GBIF: https://doi.org/10.15468/dl.zfaw79
Anopheles barbirostris	99.561028	12.890586	Unclassified	GBIF: https://doi.org/10.15468/dl.zfaw79
Anopheles barbirostris	99.562456	13.49195	Unclassified	GBIF: https://doi.org/10.15468/dl.zfaw79
Anopheles barbirostris	99.574603	12.844239	Unclassified	GBIF: https://doi.org/10.15468/dl.zfaw79
Anopheles barbirostris	99.580969	20.243946	Unclassified	GBIF: https://doi.org/10.15468/dl.zfaw79
Anopheles barbirostris	99.600483	12.815447	Unclassified	GBIF: https://doi.org/10.15468/dl.zfaw79
Anopheles barbirostris	99.605885	13.14367	Unclassified	GBIF: https://doi.org/10.15468/dl.zfaw79
Anopheles barbirostris	99.606919	13.188879	Unclassified	GBIF: https://doi.org/10.15468/dl.zfaw79
Anopheles barbirostris	99.62131	8.799615	Unclassified	GBIF: https://doi.org/10.15468/dl.zfaw79
Anopheles barbirostris	99.629186	13.235844	Unclassified	GBIF: https://doi.org/10.15468/dl.zfaw79
Anopheles barbirostris	99.632275	12.7236	Unclassified	GBIF: https://doi.org/10.15468/dl.zfaw79
Anopheles barbirostris	99.645557	12.7855	Unclassified	GBIF: https://doi.org/10.15468/dl.zfaw79
Anopheles barbirostris	99.64971	13.232429	Unclassified	GBIF: https://doi.org/10.15468/dl.zfaw79
Anopheles barbirostris	99.653077	12.777445	Unclassified	GBIF: https://doi.org/10.15468/dl.zfaw79
Anopheles barbirostris	99.670083	13.218098	Unclassified	GBIF: https://doi.org/10.15468/dl.zfaw79
Anopheles barbirostris	99.677746	12.589749	Unclassified	GBIF: https://doi.org/10.15468/dl.zfaw79
Anopheles barbirostris	99.687316	13.229365	Unclassified	GBIF: https://doi.org/10.15468/dl.zfaw79
Anopheles barbirostris	99.687803	12.664836	Unclassified	GBIF: https://doi.org/10.15468/dl.zfaw79
Anopheles barbirostris	99.690795	13.478912	Unclassified	GBIF: https://doi.org/10.15468/dl.zfaw79
Anopheles barbirostris	99.708503	12.718746	Unclassified	GBIF: https://doi.org/10.15468/dl.zfaw79
Anopheles barbirostris	99.71	12.9	Unclassified	GBIF: https://doi.org/10.15468/dl.zfaw79
Anopheles barbirostris	99.744448	13.199366	Unclassified	GBIF: https://doi.org/10.15468/dl.zfaw79
Anopheles barbirostris	99.74737	19.01191	Unclassified	GBIF: https://doi.org/10.15468/dl.zfaw79
Anopheles barbirostris	99.79323	8.3346	Unclassified	GBIF: https://doi.org/10.15468/dl.zfaw79
Anopheles barbirostris	99.81042	8.13246	Unclassified	GBIF: https://doi.org/10.15468/dl.zfaw79
Anopheles barbirostris	99.89557	19.00764	Unclassified	GBIF: https://doi.org/10.15468/dl.zfaw79
Anopheles barbirostris	99.89914	18.96605	Unclassified	GBIF: https://doi.org/10.15468/dl.zfaw79
Anopheles barbirostris	99.89924	18.98412	Unclassified	GBIF: https://doi.org/10.15468/dl.zfaw79
Anopheles barbirostris	99.91839	19.01114	Unclassified	GBIF: https://doi.org/10.15468/dl.zfaw79
Anopheles barbirostris	99.92217	18.65958	Unclassified	GBIF: https://doi.org/10.15468/dl.zfaw79
Anopheles barbirostris	99.92605	18.67673	Unclassified	GBIF: https://doi.org/10.15468/dl.zfaw79
Anopheles barbirostris	99.92632	9.56368	Unclassified	GBIF: https://doi.org/10.15468/dl.zfaw79
Anopheles barbirostris	99.93536	9.53653	Unclassified	GBIF: https://doi.org/10.15468/dl.zfaw79
Anopheles barbirostris	99.93539	9.54557	Unclassified	GBIF: https://doi.org/10.15468/dl.zfaw79
Anopheles barbirostris	99.93541	9.55461	Unclassified	GBIF: https://doi.org/10.15468/dl.zfaw79
Anopheles barbirostris	99.93699	18.93875	Unclassified	GBIF: https://doi.org/10.15468/dl.zfaw79
Anopheles barbirostris	99.94302	18.82847	Unclassified	GBIF: https://doi.org/10.15468/dl.zfaw79
Anopheles barbirostris	99.94396	18.82756	Unclassified	GBIF: https://doi.org/10.15468/dl.zfaw79

Anopheles barbirostris	99.94447	9.5365	Unclassified	GBIF: https://doi.org/10.15468/dl.zfaw79
Anopheles barbirostris	99.94491	18.82756	Unclassified	GBIF: https://doi.org/10.15468/dl.zfaw79
Anopheles barbirostris	99.95348	9.5003	Unclassified	GBIF: https://doi.org/10.15468/dl.zfaw79
Anopheles barbirostris	99.95372	18.70732	Unclassified	GBIF: https://doi.org/10.15468/dl.zfaw79
Anopheles barbirostris	99.95373	18.70823	Unclassified	GBIF: https://doi.org/10.15468/dl.zfaw79
Anopheles barbirostris	99.95658	18.70911	Unclassified	GBIF: https://doi.org/10.15468/dl.zfaw79
Anopheles barbirostris	99.961881	10.302612	Unclassified	GBIF: https://doi.org/10.15468/dl.zfaw79
Anopheles barbirostris	99.96817	8.68014	Unclassified	GBIF: https://doi.org/10.15468/dl.zfaw79
Anopheles barbirostris	99.971127	9.956608	Unclassified	GBIF: https://doi.org/10.15468/dl.zfaw79
Anopheles barbirostris	99.97367	18.71264	Unclassified	GBIF: https://doi.org/10.15468/dl.zfaw79
Anopheles barbirostris	99.97769	18.75238	Unclassified	GBIF: https://doi.org/10.15468/dl.zfaw79
Anopheles barbirostris	99.97863	18.75147	Unclassified	GBIF: https://doi.org/10.15468/dl.zfaw79
Anopheles barbirostris	99.97958	18.75147	Unclassified	GBIF: https://doi.org/10.15468/dl.zfaw79
Anopheles barbirostris	99.98053	18.75146	Unclassified	GBIF: https://doi.org/10.15468/dl.zfaw79
Anopheles barbirostris	99.98811	18.74962	Unclassified	GBIF: https://doi.org/10.15468/dl.zfaw79
Anopheles barbirostris	99.99189	18.74779	Unclassified	GBIF: https://doi.org/10.15468/dl.zfaw79
Anopheles barbirostris	99.9938	18.74959	Unclassified	GBIF: https://doi.org/10.15468/dl.zfaw79
Anopheles barbirostris	100.00212	18.71249	Unclassified	GBIF: https://doi.org/10.15468/dl.zfaw79
Anopheles barbirostris	100.02696	9.69905	Unclassified	GBIF: https://doi.org/10.15468/dl.zfaw79
Anopheles barbirostris	100.318685	17.012445	Unclassified	GBIF: https://doi.org/10.15468/dl.zfaw79
Anopheles barbirostris	100.382235	17.343596	Unclassified	GBIF: https://doi.org/10.15468/dl.zfaw79
Anopheles barbirostris	100.508752	20.071488	Unclassified	GBIF: https://doi.org/10.15468/dl.zfaw79
Anopheles barbirostris	100.510863	16.847539	Unclassified	GBIF: https://doi.org/10.15468/dl.zfaw79
Anopheles barbirostris	100.519977	17.460058	Unclassified	GBIF: https://doi.org/10.15468/dl.zfaw79
Anopheles barbirostris	100.624451	16.741845	Unclassified	GBIF: https://doi.org/10.15468/dl.zfaw79
Anopheles barbirostris	100.624841	17.543373	Unclassified	GBIF: https://doi.org/10.15468/dl.zfaw79
Anopheles barbirostris	100.63	18.53	Unclassified	GBIF: https://doi.org/10.15468/dl.zfaw79
Anopheles barbirostris	100.63537	18.49119	Unclassified	GBIF: https://doi.org/10.15468/dl.zfaw79
Anopheles barbirostris	100.664328	17.011719	Unclassified	GBIF: https://doi.org/10.15468/dl.zfaw79
Anopheles barbirostris	100.7247	18.89696	Unclassified	GBIF: https://doi.org/10.15468/dl.zfaw79
Anopheles barbirostris	100.751699	16.913396	Unclassified	GBIF: https://doi.org/10.15468/dl.zfaw79
Anopheles barbirostris	100.75383	18.95993	Unclassified	GBIF: https://doi.org/10.15468/dl.zfaw79
Anopheles barbirostris	100.76313	18.94177	Unclassified	GBIF: https://doi.org/10.15468/dl.zfaw79
Anopheles barbirostris	100.765506	16.984662	Unclassified	GBIF: https://doi.org/10.15468/dl.zfaw79
Anopheles barbirostris	100.766375	16.923139	Unclassified	GBIF: https://doi.org/10.15468/dl.zfaw79
Anopheles barbirostris	100.78557	16.821839	Unclassified	GBIF: https://doi.org/10.15468/dl.zfaw79
Anopheles barbirostris	100.791695	19.118503	Unclassified	GBIF: https://doi.org/10.15468/dl.zfaw79
Anopheles barbirostris	100.802838	16.89936	Unclassified	GBIF: https://doi.org/10.15468/dl.zfaw79
Anopheles barbirostris	100.82944	17.101114	Unclassified	GBIF: https://doi.org/10.15468/dl.zfaw79
Anopheles barbirostris	100.835435	17.030126	Unclassified	GBIF: https://doi.org/10.15468/dl.zfaw79
Anopheles barbirostris	100.841548	16.886403	Unclassified	GBIF: https://doi.org/10.15468/dl.zfaw79
Anopheles barbirostris	100.874163	16.380101	Unclassified	GBIF: https://doi.org/10.15468/dl.zfaw79

Anopheles barbirostris	100.881387	16.222448	Unclassified	GBIF: https://doi.org/10.15468/dl.zfaw79
Anopheles barbirostris	100.89312	15.813364	Unclassified	GBIF: https://doi.org/10.15468/dl.zfaw79
Anopheles barbirostris	100.93455	13.19321	Unclassified	GBIF: https://doi.org/10.15468/dl.zfaw79
Anopheles barbirostris	100.937892	16.579187	Unclassified	GBIF: https://doi.org/10.15468/dl.zfaw79
Anopheles barbirostris	100.946318	17.74737	Unclassified	GBIF: https://doi.org/10.15468/dl.zfaw79
Anopheles barbirostris	100.95415	14.01007	Unclassified	GBIF: https://doi.org/10.15468/dl.zfaw79
Anopheles barbirostris	100.96035	16.971298	Unclassified	GBIF: https://doi.org/10.15468/dl.zfaw79
Anopheles barbirostris	100.965507	12.989845	Unclassified	GBIF: https://doi.org/10.15468/dl.zfaw79
Anopheles barbirostris	100.967629	12.915851	Unclassified	GBIF: https://doi.org/10.15468/dl.zfaw79
Anopheles barbirostris	100.978356	16.87099	Unclassified	GBIF: https://doi.org/10.15468/dl.zfaw79
Anopheles barbirostris	100.979437	19.604616	Unclassified	GBIF: https://doi.org/10.15468/dl.zfaw79
Anopheles barbirostris	101.006342	15.55663	Unclassified	GBIF: https://doi.org/10.15468/dl.zfaw79
Anopheles barbirostris	101.012616	16.733908	Unclassified	GBIF: https://doi.org/10.15468/dl.zfaw79
Anopheles barbirostris	101.048138	13.309745	Unclassified	GBIF: https://doi.org/10.15468/dl.zfaw79
Anopheles barbirostris	101.053087	12.891937	Unclassified	GBIF: https://doi.org/10.15468/dl.zfaw79
Anopheles barbirostris	101.0847	12.8573	Unclassified	GBIF: https://doi.org/10.15468/dl.zfaw79
Anopheles barbirostris	101.097471	13.359047	Unclassified	GBIF: https://doi.org/10.15468/dl.zfaw79
Anopheles barbirostris	101.104708	13.235179	Unclassified	GBIF: https://doi.org/10.15468/dl.zfaw79
Anopheles barbirostris	101.108928	13.296596	Unclassified	GBIF: https://doi.org/10.15468/dl.zfaw79
Anopheles barbirostris	101.124122	19.506643	Unclassified	GBIF: https://doi.org/10.15468/dl.zfaw79
Anopheles barbirostris	101.20263	19.279811	Unclassified	GBIF: https://doi.org/10.15468/dl.zfaw79
Anopheles barbirostris	101.234592	14.56598	Unclassified	GBIF: https://doi.org/10.15468/dl.zfaw79
Anopheles barbirostris	101.251101	15.747735	Unclassified	GBIF: https://doi.org/10.15468/dl.zfaw79
Anopheles barbirostris	101.252322	15.805219	Unclassified	GBIF: https://doi.org/10.15468/dl.zfaw79
Anopheles barbirostris	101.268126	15.693213	Unclassified	GBIF: https://doi.org/10.15468/dl.zfaw79
Anopheles barbirostris	101.30242	6.25942	Unclassified	GBIF: https://doi.org/10.15468/dl.zfaw79
Anopheles barbirostris	101.39096	14.53559	Unclassified	GBIF: https://doi.org/10.15468/dl.zfaw79
Anopheles barbirostris	101.399898	13.143124	Unclassified	GBIF: https://doi.org/10.15468/dl.zfaw79
Anopheles barbirostris	101.44148	6.214395	Unclassified	GBIF: https://doi.org/10.15468/dl.zfaw79
Anopheles barbirostris	101.443397	6.275781	Unclassified	GBIF: https://doi.org/10.15468/dl.zfaw79
Anopheles barbirostris	101.4474	14.60728	Unclassified	GBIF: https://doi.org/10.15468/dl.zfaw79
Anopheles barbirostris	101.478599	6.190669	Unclassified	GBIF: https://doi.org/10.15468/dl.zfaw79
Anopheles barbirostris	101.48209	14.3901	Unclassified	GBIF: https://doi.org/10.15468/dl.zfaw79
Anopheles barbirostris	101.492865	6.169472	Unclassified	GBIF: https://doi.org/10.15468/dl.zfaw79
Anopheles barbirostris	101.501371	13.239733	Unclassified	GBIF: https://doi.org/10.15468/dl.zfaw79
Anopheles barbirostris	101.50725	14.60882	Unclassified	GBIF: https://doi.org/10.15468/dl.zfaw79
Anopheles barbirostris	101.532439	13.300862	Unclassified	GBIF: https://doi.org/10.15468/dl.zfaw79
Anopheles barbirostris	101.5363	13.493259	Unclassified	GBIF: https://doi.org/10.15468/dl.zfaw79
Anopheles barbirostris	101.539376	6.208986	Unclassified	GBIF: https://doi.org/10.15468/dl.zfaw79
Anopheles barbirostris	101.541033	14.491603	Unclassified	GBIF: https://doi.org/10.15468/dl.zfaw79
Anopheles barbirostris	101.549551	16.993522	Unclassified	GBIF: https://doi.org/10.15468/dl.zfaw79
Anopheles barbirostris	101.569413	13.04301	Unclassified	GBIF: https://doi.org/10.15468/dl.zfaw79

Anopheles barbirostris	101.572512	6.168986	Unclassified	GBIF: https://doi.org/10.15468/dl.zfaw79
Anopheles barbirostris	101.576794	14.46852	Unclassified	GBIF: https://doi.org/10.15468/dl.zfaw79
Anopheles barbirostris	101.584419	16.763201	Unclassified	GBIF: https://doi.org/10.15468/dl.zfaw79
Anopheles barbirostris	101.585851	13.286359	Unclassified	GBIF: https://doi.org/10.15468/dl.zfaw79
Anopheles barbirostris	101.597225	6.034226	Unclassified	GBIF: https://doi.org/10.15468/dl.zfaw79
Anopheles barbirostris	101.625213	6.029105	Unclassified	GBIF: https://doi.org/10.15468/dl.zfaw79
Anopheles barbirostris	101.637721	6.030815	Unclassified	GBIF: https://doi.org/10.15468/dl.zfaw79
Anopheles barbirostris	101.651256	6.076325	Unclassified	GBIF: https://doi.org/10.15468/dl.zfaw79
Anopheles barbirostris	101.653968	6.007651	Unclassified	GBIF: https://doi.org/10.15468/dl.zfaw79
Anopheles barbirostris	101.656504	13.0873	Unclassified	GBIF: https://doi.org/10.15468/dl.zfaw79
Anopheles barbirostris	101.660153	13.133261	Unclassified	GBIF: https://doi.org/10.15468/dl.zfaw79
Anopheles barbirostris	101.660782	5.966934	Unclassified	GBIF: https://doi.org/10.15468/dl.zfaw79
Anopheles barbirostris	101.665313	6.076102	Unclassified	GBIF: https://doi.org/10.15468/dl.zfaw79
Anopheles barbirostris	101.672168	13.001317	Unclassified	GBIF: https://doi.org/10.15468/dl.zfaw79
Anopheles barbirostris	101.67662	13.072638	Unclassified	GBIF: https://doi.org/10.15468/dl.zfaw79
Anopheles barbirostris	101.683613	16.171731	Unclassified	GBIF: https://doi.org/10.15468/dl.zfaw79
Anopheles barbirostris	101.692791	12.948708	Unclassified	GBIF: https://doi.org/10.15468/dl.zfaw79
Anopheles barbirostris	101.698576	13.309993	Unclassified	GBIF: https://doi.org/10.15468/dl.zfaw79
Anopheles barbirostris	101.702026	13.371384	Unclassified	GBIF: https://doi.org/10.15468/dl.zfaw79
Anopheles barbirostris	101.713136	5.811958	Unclassified	GBIF: https://doi.org/10.15468/dl.zfaw79
Anopheles barbirostris	101.715982	5.978439	Unclassified	GBIF: https://doi.org/10.15468/dl.zfaw79
Anopheles barbirostris	101.737005	5.843329	Unclassified	GBIF: https://doi.org/10.15468/dl.zfaw79
Anopheles barbirostris	101.741003	13.129274	Unclassified	GBIF: https://doi.org/10.15468/dl.zfaw79
Anopheles barbirostris	101.742215	13.41702	Unclassified	GBIF: https://doi.org/10.15468/dl.zfaw79
Anopheles barbirostris	101.742333	13.015038	Unclassified	GBIF: https://doi.org/10.15468/dl.zfaw79
Anopheles barbirostris	101.748221	6.03947	Unclassified	GBIF: https://doi.org/10.15468/dl.zfaw79
Anopheles barbirostris	101.75755	14.362739	Unclassified	GBIF: https://doi.org/10.15468/dl.zfaw79
Anopheles barbirostris	101.759407	13.407947	Unclassified	GBIF: https://doi.org/10.15468/dl.zfaw79
Anopheles barbirostris	101.766781	5.957333	Unclassified	GBIF: https://doi.org/10.15468/dl.zfaw79
Anopheles barbirostris	101.775659	13.11003	Unclassified	GBIF: https://doi.org/10.15468/dl.zfaw79
Anopheles barbirostris	101.794338	13.49568	Unclassified	GBIF: https://doi.org/10.15468/dl.zfaw79
Anopheles barbirostris	101.821927	12.884111	Unclassified	GBIF: https://doi.org/10.15468/dl.zfaw79
Anopheles barbirostris	101.82418	14.32755	Unclassified	GBIF: https://doi.org/10.15468/dl.zfaw79
Anopheles barbirostris	101.82424	14.33206	Unclassified	GBIF: https://doi.org/10.15468/dl.zfaw79
Anopheles barbirostris	101.8335	14.33195	Unclassified	GBIF: https://doi.org/10.15468/dl.zfaw79
Anopheles barbirostris	101.847691	13.166991	Unclassified	GBIF: https://doi.org/10.15468/dl.zfaw79
Anopheles barbirostris	101.85134	14.27755	Unclassified	GBIF: https://doi.org/10.15468/dl.zfaw79
Anopheles barbirostris	101.86049	14.26841	Unclassified	GBIF: https://doi.org/10.15468/dl.zfaw79
Anopheles barbirostris	101.86975	14.2683	Unclassified	GBIF: https://doi.org/10.15468/dl.zfaw79
Anopheles barbirostris	101.87901	14.26818	Unclassified	GBIF: https://doi.org/10.15468/dl.zfaw79
Anopheles barbirostris	101.879012	13.063376	Unclassified	GBIF: https://doi.org/10.15468/dl.zfaw79
Anopheles barbirostris	101.87958	14.31334	Unclassified	GBIF: https://doi.org/10.15468/dl.zfaw79

Anopheles barbirostris	101.885153	12.8463	Unclassified	GBIF: https://doi.org/10.15468/dl.zfaw79
Anopheles barbirostris	101.88815	14.25904	Unclassified	GBIF: https://doi.org/10.15468/dl.zfaw79
Anopheles barbirostris	101.8885	14.28613	Unclassified	GBIF: https://doi.org/10.15468/dl.zfaw79
Anopheles barbirostris	101.88896	14.32225	Unclassified	GBIF: https://doi.org/10.15468/dl.zfaw79
Anopheles barbirostris	101.89313	14.28608	Unclassified	GBIF: https://doi.org/10.15468/dl.zfaw79
Anopheles barbirostris	101.8966	14.19572	Unclassified	GBIF: https://doi.org/10.15468/dl.zfaw79
Anopheles barbirostris	101.89672	14.20475	Unclassified	GBIF: https://doi.org/10.15468/dl.zfaw79
Anopheles barbirostris	101.89741	14.25893	Unclassified	GBIF: https://doi.org/10.15468/dl.zfaw79
Anopheles barbirostris	101.89753	14.26796	Unclassified	GBIF: https://doi.org/10.15468/dl.zfaw79
Anopheles barbirostris	101.90586	14.19561	Unclassified	GBIF: https://doi.org/10.15468/dl.zfaw79
Anopheles barbirostris	101.90598	14.20464	Unclassified	GBIF: https://doi.org/10.15468/dl.zfaw79
Anopheles barbirostris	101.90621	14.2227	Unclassified	GBIF: https://doi.org/10.15468/dl.zfaw79
Anopheles barbirostris	101.907779	13.227123	Unclassified	GBIF: https://doi.org/10.15468/dl.zfaw79
Anopheles barbirostris	101.908958	13.37005	Unclassified	GBIF: https://doi.org/10.15468/dl.zfaw79
Anopheles barbirostris	101.914358	14.389737	Unclassified	GBIF: https://doi.org/10.15468/dl.zfaw79
Anopheles barbirostris	101.91523	14.20452	Unclassified	GBIF: https://doi.org/10.15468/dl.zfaw79
Anopheles barbirostris	101.916117	12.586819	Unclassified	GBIF: https://doi.org/10.15468/dl.zfaw79
Anopheles barbirostris	101.922512	14.204013	Unclassified	GBIF: https://doi.org/10.15468/dl.zfaw79
Anopheles barbirostris	101.92694	14.39404	Unclassified	GBIF: https://doi.org/10.15468/dl.zfaw79
Anopheles barbirostris	101.961118	13.464004	Unclassified	GBIF: https://doi.org/10.15468/dl.zfaw79
Anopheles barbirostris	101.969375	13.128144	Unclassified	GBIF: https://doi.org/10.15468/dl.zfaw79
Anopheles barbirostris	102.0244	12.51496	Unclassified	GBIF: https://doi.org/10.15468/dl.zfaw79
Anopheles barbirostris	102.03245	12.6144	Unclassified	GBIF: https://doi.org/10.15468/dl.zfaw79
Anopheles barbirostris	102.033679	13.960971	Unclassified	GBIF: https://doi.org/10.15468/dl.zfaw79
Anopheles barbirostris	102.039792	14.056518	Unclassified	GBIF: https://doi.org/10.15468/dl.zfaw79
Anopheles barbirostris	102.056339	12.623627	Unclassified	GBIF: https://doi.org/10.15468/dl.zfaw79
Anopheles barbirostris	102.069882	12.964291	Unclassified	GBIF: https://doi.org/10.15468/dl.zfaw79
Anopheles barbirostris	102.076399	14.449597	Unclassified	GBIF: https://doi.org/10.15468/dl.zfaw79
Anopheles barbirostris	102.08925	12.47051	Unclassified	GBIF: https://doi.org/10.15468/dl.zfaw79
Anopheles barbirostris	102.09824	12.48867	Unclassified	GBIF: https://doi.org/10.15468/dl.zfaw79
Anopheles barbirostris	102.121997	13.034346	Unclassified	GBIF: https://doi.org/10.15468/dl.zfaw79
Anopheles barbirostris	102.1518	13.209082	Unclassified	GBIF: https://doi.org/10.15468/dl.zfaw79
Anopheles barbirostris	102.166581	13.206536	Unclassified	GBIF: https://doi.org/10.15468/dl.zfaw79
Anopheles barbirostris	102.179683	13.189519	Unclassified	GBIF: https://doi.org/10.15468/dl.zfaw79
Anopheles barbirostris	102.192464	13.280892	Unclassified	GBIF: https://doi.org/10.15468/dl.zfaw79
Anopheles barbirostris	102.193808	13.163479	Unclassified	GBIF: https://doi.org/10.15468/dl.zfaw79
Anopheles barbirostris	102.207242	13.116654	Unclassified	GBIF: https://doi.org/10.15468/dl.zfaw79
Anopheles barbirostris	102.208368	13.969362	Unclassified	GBIF: https://doi.org/10.15468/dl.zfaw79
Anopheles barbirostris	102.25724	12.928392	Unclassified	GBIF: https://doi.org/10.15468/dl.zfaw79
Anopheles barbirostris	102.25934	16.63674	Unclassified	GBIF: https://doi.org/10.15468/dl.zfaw79
Anopheles barbirostris	102.26109	13.081099	Unclassified	GBIF: https://doi.org/10.15468/dl.zfaw79
Anopheles barbirostris	102.26383	12.817941	Unclassified	GBIF: https://doi.org/10.15468/dl.zfaw79

Anopheles barbirostris	102.264346	14.389798	Unclassified	GBIF: https://doi.org/10.15468/dl.zfaw79
Anopheles barbirostris	102.265793	12.904092	Unclassified	GBIF: https://doi.org/10.15468/dl.zfaw79
Anopheles barbirostris	102.26631	16.77185	Unclassified	GBIF: https://doi.org/10.15468/dl.zfaw79
Anopheles barbirostris	102.26657	16.75379	Unclassified	GBIF: https://doi.org/10.15468/dl.zfaw79
Anopheles barbirostris	102.26696	16.7267	Unclassified	GBIF: https://doi.org/10.15468/dl.zfaw79
Anopheles barbirostris	102.268136	13.026972	Unclassified	GBIF: https://doi.org/10.15468/dl.zfaw79
Anopheles barbirostris	102.269907	12.864386	Unclassified	GBIF: https://doi.org/10.15468/dl.zfaw79
Anopheles barbirostris	102.274245	12.973739	Unclassified	GBIF: https://doi.org/10.15468/dl.zfaw79
Anopheles barbirostris	102.275149	13.059567	Unclassified	GBIF: https://doi.org/10.15468/dl.zfaw79
Anopheles barbirostris	102.27786	16.61847	Unclassified	GBIF: https://doi.org/10.15468/dl.zfaw79
Anopheles barbirostris	102.279299	12.7615	Unclassified	GBIF: https://doi.org/10.15468/dl.zfaw79
Anopheles barbirostris	102.280828	12.646369	Unclassified	GBIF: https://doi.org/10.15468/dl.zfaw79
Anopheles barbirostris	102.283668	13.038879	Unclassified	GBIF: https://doi.org/10.15468/dl.zfaw79
Anopheles barbirostris	102.287214	12.881731	Unclassified	GBIF: https://doi.org/10.15468/dl.zfaw79
Anopheles barbirostris	102.288544	13.182565	Unclassified	GBIF: https://doi.org/10.15468/dl.zfaw79
Anopheles barbirostris	102.293068	12.053376	Unclassified	GBIF: https://doi.org/10.15468/dl.zfaw79
Anopheles barbirostris	102.293509	12.981167	Unclassified	GBIF: https://doi.org/10.15468/dl.zfaw79
Anopheles barbirostris	102.301726	13.157409	Unclassified	GBIF: https://doi.org/10.15468/dl.zfaw79
Anopheles barbirostris	102.302237	14.254161	Unclassified	GBIF: https://doi.org/10.15468/dl.zfaw79
Anopheles barbirostris	102.302612	12.991199	Unclassified	GBIF: https://doi.org/10.15468/dl.zfaw79
Anopheles barbirostris	102.311755	13.335478	Unclassified	GBIF: https://doi.org/10.15468/dl.zfaw79
Anopheles barbirostris	102.326459	12.914657	Unclassified	GBIF: https://doi.org/10.15468/dl.zfaw79
Anopheles barbirostris	102.326726	13.061912	Unclassified	GBIF: https://doi.org/10.15468/dl.zfaw79
Anopheles barbirostris	102.331768	12.494625	Unclassified	GBIF: https://doi.org/10.15468/dl.zfaw79
Anopheles barbirostris	102.331948	13.175793	Unclassified	GBIF: https://doi.org/10.15468/dl.zfaw79
Anopheles barbirostris	102.33368	16.64629	Unclassified	GBIF: https://doi.org/10.15468/dl.zfaw79
Anopheles barbirostris	102.338285	12.237169	Unclassified	GBIF: https://doi.org/10.15468/dl.zfaw79
Anopheles barbirostris	102.34243	16.69156	Unclassified	GBIF: https://doi.org/10.15468/dl.zfaw79
Anopheles barbirostris	102.34305	16.64641	Unclassified	GBIF: https://doi.org/10.15468/dl.zfaw79
Anopheles barbirostris	102.34474	12.484815	Unclassified	GBIF: https://doi.org/10.15468/dl.zfaw79
Anopheles barbirostris	102.365687	12.684681	Unclassified	GBIF: https://doi.org/10.15468/dl.zfaw79
Anopheles barbirostris	102.367458	13.12537	Unclassified	GBIF: https://doi.org/10.15468/dl.zfaw79
Anopheles barbirostris	102.368323	13.049691	Unclassified	GBIF: https://doi.org/10.15468/dl.zfaw79
Anopheles barbirostris	102.374354	12.914239	Unclassified	GBIF: https://doi.org/10.15468/dl.zfaw79
Anopheles barbirostris	102.381249	12.511373	Unclassified	GBIF: https://doi.org/10.15468/dl.zfaw79
Anopheles barbirostris	102.381435	12.215942	Unclassified	GBIF: https://doi.org/10.15468/dl.zfaw79
Anopheles barbirostris	102.381567	12.661349	Unclassified	GBIF: https://doi.org/10.15468/dl.zfaw79
Anopheles barbirostris	102.386576	13.041499	Unclassified	GBIF: https://doi.org/10.15468/dl.zfaw79
Anopheles barbirostris	102.390085	13.083338	Unclassified	GBIF: https://doi.org/10.15468/dl.zfaw79
Anopheles barbirostris	102.391101	12.861658	Unclassified	GBIF: https://doi.org/10.15468/dl.zfaw79
Anopheles barbirostris	102.392028	12.204587	Unclassified	GBIF: https://doi.org/10.15468/dl.zfaw79
Anopheles barbirostris	102.392236	12.965914	Unclassified	GBIF: https://doi.org/10.15468/dl.zfaw79

Anopheles barbirostris	102.404232	14.335548	Unclassified	GBIF: https://doi.org/10.15468/dl.zfaw79
Anopheles barbirostris	102.404323	13.043731	Unclassified	GBIF: https://doi.org/10.15468/dl.zfaw79
Anopheles barbirostris	102.409248	12.992124	Unclassified	GBIF: https://doi.org/10.15468/dl.zfaw79
Anopheles barbirostris	102.418115	13.046579	Unclassified	GBIF: https://doi.org/10.15468/dl.zfaw79
Anopheles barbirostris	102.421872	12.275075	Unclassified	GBIF: https://doi.org/10.15468/dl.zfaw79
Anopheles barbirostris	102.441214	12.677288	Unclassified	GBIF: https://doi.org/10.15468/dl.zfaw79
Anopheles barbirostris	102.445247	12.549032	Unclassified	GBIF: https://doi.org/10.15468/dl.zfaw79
Anopheles barbirostris	102.462267	12.595272	Unclassified	GBIF: https://doi.org/10.15468/dl.zfaw79
Anopheles barbirostris	102.462574	12.981753	Unclassified	GBIF: https://doi.org/10.15468/dl.zfaw79
Anopheles barbirostris	102.470532	12.894781	Unclassified	GBIF: https://doi.org/10.15468/dl.zfaw79
Anopheles barbirostris	102.472119	12.62247	Unclassified	GBIF: https://doi.org/10.15468/dl.zfaw79
Anopheles barbirostris	102.475205	12.933475	Unclassified	GBIF: https://doi.org/10.15468/dl.zfaw79
Anopheles barbirostris	102.476382	12.562879	Unclassified	GBIF: https://doi.org/10.15468/dl.zfaw79
Anopheles barbirostris	102.48322	12.616251	Unclassified	GBIF: https://doi.org/10.15468/dl.zfaw79
Anopheles barbirostris	102.486248	12.205174	Unclassified	GBIF: https://doi.org/10.15468/dl.zfaw79
Anopheles barbirostris	102.530348	12.400742	Unclassified	GBIF: https://doi.org/10.15468/dl.zfaw79
Anopheles barbirostris	102.550476	12.606033	Unclassified	GBIF: https://doi.org/10.15468/dl.zfaw79
Anopheles barbirostris	102.564458	12.390208	Unclassified	GBIF: https://doi.org/10.15468/dl.zfaw79
Anopheles barbirostris	102.578558	11.67832	Unclassified	GBIF: https://doi.org/10.15468/dl.zfaw79
Anopheles barbirostris	102.580014	12.59546	Unclassified	GBIF: https://doi.org/10.15468/dl.zfaw79
Anopheles barbirostris	102.580471	12.546671	Unclassified	GBIF: https://doi.org/10.15468/dl.zfaw79
Anopheles barbirostris	102.589953	12.516034	Unclassified	GBIF: https://doi.org/10.15468/dl.zfaw79
Anopheles barbirostris	102.593272	12.455523	Unclassified	GBIF: https://doi.org/10.15468/dl.zfaw79
Anopheles barbirostris	102.595932	17.100737	Unclassified	GBIF: https://doi.org/10.15468/dl.zfaw79
Anopheles barbirostris	102.618292	12.230746	Unclassified	GBIF: https://doi.org/10.15468/dl.zfaw79
Anopheles barbirostris	102.62282	12.238014	Unclassified	GBIF: https://doi.org/10.15468/dl.zfaw79
Anopheles barbirostris	102.629235	12.444094	Unclassified	GBIF: https://doi.org/10.15468/dl.zfaw79
Anopheles barbirostris	102.632075	12.435083	Unclassified	GBIF: https://doi.org/10.15468/dl.zfaw79
Anopheles barbirostris	102.6369	12.409824	Unclassified	GBIF: https://doi.org/10.15468/dl.zfaw79
Anopheles barbirostris	102.658145	12.295247	Unclassified	GBIF: https://doi.org/10.15468/dl.zfaw79
Anopheles barbirostris	102.660827	12.199483	Unclassified	GBIF: https://doi.org/10.15468/dl.zfaw79
Anopheles barbirostris	102.666093	12.227541	Unclassified	GBIF: https://doi.org/10.15468/dl.zfaw79
Anopheles barbirostris	102.666441	12.187784	Unclassified	GBIF: https://doi.org/10.15468/dl.zfaw79
Anopheles barbirostris	102.679426	12.173435	Unclassified	GBIF: https://doi.org/10.15468/dl.zfaw79
Anopheles barbirostris	102.679598	12.153556	Unclassified	GBIF: https://doi.org/10.15468/dl.zfaw79
Anopheles barbirostris	102.680198	12.474401	Unclassified	GBIF: https://doi.org/10.15468/dl.zfaw79
Anopheles barbirostris	102.681637	14.189385	Unclassified	GBIF: https://doi.org/10.15468/dl.zfaw79
Anopheles barbirostris	102.692968	12.387497	Unclassified	GBIF: https://doi.org/10.15468/dl.zfaw79
Anopheles barbirostris	102.701124	12.106745	Unclassified	GBIF: https://doi.org/10.15468/dl.zfaw79
Anopheles barbirostris	102.704913	12.09322	Unclassified	GBIF: https://doi.org/10.15468/dl.zfaw79
Anopheles barbirostris	102.73	12.15	Unclassified	GBIF: https://doi.org/10.15468/dl.zfaw79
Anopheles barbirostris	102.731724	12.071752	Unclassified	GBIF: https://doi.org/10.15468/dl.zfaw79

Anopheles barbirostris	102.778824	11.926632	Unclassified	GBIF: https://doi.org/10.15468/dl.zfaw79
Anopheles barbirostris	102.808502	11.888004	Unclassified	GBIF: https://doi.org/10.15468/dl.zfaw79
Anopheles barbirostris	102.817793	11.873617	Unclassified	GBIF: https://doi.org/10.15468/dl.zfaw79
Anopheles barbirostris	102.829025	11.845686	Unclassified	GBIF: https://doi.org/10.15468/dl.zfaw79
Anopheles barbirostris	102.943857	14.279438	Unclassified	GBIF: https://doi.org/10.15468/dl.zfaw79
Anopheles barbirostris	102.967142	14.369102	Unclassified	GBIF: https://doi.org/10.15468/dl.zfaw79
Anopheles barbirostris	103.128814	14.371502	Unclassified	GBIF: https://doi.org/10.15468/dl.zfaw79
Anopheles barbirostris	103.38384	15.05659	Unclassified	GBIF: https://doi.org/10.15468/dl.zfaw79
Anopheles barbirostris	103.38386	15.05388	Unclassified	GBIF: https://doi.org/10.15468/dl.zfaw79
Anopheles barbirostris	103.444973	14.449552	Unclassified	GBIF: https://doi.org/10.15468/dl.zfaw79
Anopheles barbirostris	103.59543	17.039013	Unclassified	GBIF: https://doi.org/10.15468/dl.zfaw79
Anopheles barbirostris	103.703396	14.484479	Unclassified	GBIF: https://doi.org/10.15468/dl.zfaw79
Anopheles barbirostris	103.709242	14.473979	Unclassified	GBIF: https://doi.org/10.15468/dl.zfaw79
Anopheles barbirostris	103.711539	14.517386	Unclassified	GBIF: https://doi.org/10.15468/dl.zfaw79
Anopheles barbirostris	103.728408	16.926085	Unclassified	GBIF: https://doi.org/10.15468/dl.zfaw79
Anopheles barbirostris	103.81026	1.352825	Unclassified	GBIF: https://doi.org/10.15468/dl.zfaw79
Anopheles barbirostris	103.847257	14.480587	Unclassified	GBIF: https://doi.org/10.15468/dl.zfaw79
Anopheles barbirostris	103.873492	14.453018	Unclassified	GBIF: https://doi.org/10.15468/dl.zfaw79
Anopheles barbirostris	103.920707	14.47843	Unclassified	GBIF: https://doi.org/10.15468/dl.zfaw79
Anopheles barbirostris	104.116595	16.637982	Unclassified	GBIF: https://doi.org/10.15468/dl.zfaw79
Anopheles barbirostris	104.140872	16.660682	Unclassified	GBIF: https://doi.org/10.15468/dl.zfaw79
Anopheles barbirostris	104.160468	16.763269	Unclassified	GBIF: https://doi.org/10.15468/dl.zfaw79
Anopheles barbirostris	104.208742	14.503051	Unclassified	GBIF: https://doi.org/10.15468/dl.zfaw79
Anopheles barbirostris	104.211418	14.506937	Unclassified	GBIF: https://doi.org/10.15468/dl.zfaw79
Anopheles barbirostris	104.24534	16.83593	Unclassified	GBIF: https://doi.org/10.15468/dl.zfaw79
Anopheles barbirostris	104.312034	14.554037	Unclassified	GBIF: https://doi.org/10.15468/dl.zfaw79
Anopheles barbirostris	104.320394	16.718235	Unclassified	GBIF: https://doi.org/10.15468/dl.zfaw79
Anopheles barbirostris	104.363153	14.530674	Unclassified	GBIF: https://doi.org/10.15468/dl.zfaw79
Anopheles barbirostris	104.370933	16.178594	Unclassified	GBIF: https://doi.org/10.15468/dl.zfaw79
Anopheles barbirostris	104.375223	14.528899	Unclassified	GBIF: https://doi.org/10.15468/dl.zfaw79
Anopheles barbirostris	104.452058	14.487193	Unclassified	GBIF: https://doi.org/10.15468/dl.zfaw79
Anopheles barbirostris	104.455708	16.414059	Unclassified	GBIF: https://doi.org/10.15468/dl.zfaw79
Anopheles barbirostris	104.490682	16.916538	Unclassified	GBIF: https://doi.org/10.15468/dl.zfaw79
Anopheles barbirostris	104.517268	16.517264	Unclassified	GBIF: https://doi.org/10.15468/dl.zfaw79
Anopheles barbirostris	104.528418	14.504845	Unclassified	GBIF: https://doi.org/10.15468/dl.zfaw79
Anopheles barbirostris	104.542243	16.818064	Unclassified	GBIF: https://doi.org/10.15468/dl.zfaw79
Anopheles barbirostris	104.595483	16.305895	Unclassified	GBIF: https://doi.org/10.15468/dl.zfaw79
Anopheles barbirostris	104.623555	16.311371	Unclassified	GBIF: https://doi.org/10.15468/dl.zfaw79
Anopheles barbirostris	104.627336	16.29149	Unclassified	GBIF: https://doi.org/10.15468/dl.zfaw79
Anopheles barbirostris	104.703049	16.361218	Unclassified	GBIF: https://doi.org/10.15468/dl.zfaw79
Anopheles barbirostris	104.724816	16.195812	Unclassified	GBIF: https://doi.org/10.15468/dl.zfaw79
Anopheles barbirostris	104.732978	16.38657	Unclassified	GBIF: https://doi.org/10.15468/dl.zfaw79

Anopheles barbirostris	104.8182	16.382142	Unclassified	GBIF: https://doi.org/10.15468/dl.zfaw79
Anopheles barbirostris	104.84929	16.137176	Unclassified	GBIF: https://doi.org/10.15468/dl.zfaw79
Anopheles barbirostris	104.979029	14.476281	Unclassified	GBIF: https://doi.org/10.15468/dl.zfaw79
Anopheles barbirostris	104.991524	14.483618	Unclassified	GBIF: https://doi.org/10.15468/dl.zfaw79
Anopheles barbirostris	105.137188	14.552966	Unclassified	GBIF: https://doi.org/10.15468/dl.zfaw79
Anopheles barbirostris	105.137874	14.427188	Unclassified	GBIF: https://doi.org/10.15468/dl.zfaw79
Anopheles barbirostris	105.234915	15.572064	Unclassified	GBIF: https://doi.org/10.15468/dl.zfaw79
Anopheles barbirostris	105.257916	14.536822	Unclassified	GBIF: https://doi.org/10.15468/dl.zfaw79
Anopheles barbirostris	105.268966	15.568951	Unclassified	GBIF: https://doi.org/10.15468/dl.zfaw79
Anopheles barbirostris	105.311586	15.697635	Unclassified	GBIF: https://doi.org/10.15468/dl.zfaw79
Anopheles barbirostris	105.338703	14.558419	Unclassified	GBIF: https://doi.org/10.15468/dl.zfaw79
Anopheles barbirostris	105.36191	15.656875	Unclassified	GBIF: https://doi.org/10.15468/dl.zfaw79
Anopheles barbirostris	105.374034	14.591819	Unclassified	GBIF: https://doi.org/10.15468/dl.zfaw79
Anopheles barbirostris	105.393231	14.941686	Unclassified	GBIF: https://doi.org/10.15468/dl.zfaw79
Anopheles barbirostris	105.464749	14.95448	Unclassified	GBIF: https://doi.org/10.15468/dl.zfaw79
Anopheles barbirostris	105.606057	15.549659	Unclassified	GBIF: https://doi.org/10.15468/dl.zfaw79
Anopheles barbirostris	106.78333	-6.58333	Unclassified	GBIF: https://doi.org/10.15468/dl.zfaw79
Anopheles barbirostris	106.791892	-6.595193	Unclassified	GBIF: https://doi.org/10.15468/dl.zfaw79
Anopheles barbirostris	106.795015	-6.145732	Unclassified	GBIF: https://doi.org/10.15468/dl.zfaw79
Anopheles barbirostris	106.842727	-6.214125	Unclassified	GBIF: https://doi.org/10.15468/dl.zfaw79
Anopheles barbirostris	106.845278	-6.204167	Unclassified	GBIF: https://doi.org/10.15468/dl.zfaw79
Anopheles barbirostris	106.932333	-6.680078	Unclassified	GBIF: https://doi.org/10.15468/dl.zfaw79
Anopheles barbirostris	107.29	21	Unclassified	GBIF: https://doi.org/10.15468/dl.zfaw79
Anopheles barbirostris	107.600278	-6.924762	Unclassified	GBIF: https://doi.org/10.15468/dl.zfaw79
Anopheles barbirostris	108.510278	-6.576111	Unclassified	GBIF: https://doi.org/10.15468/dl.zfaw79
Anopheles barbirostris	108.59	13.37	Unclassified	GBIF: https://doi.org/10.15468/dl.zfaw79
Anopheles barbirostris	108.905278	-7.724444	Unclassified	GBIF: https://doi.org/10.15468/dl.zfaw79
Anopheles barbirostris	109.023056	-7.697778	Unclassified	GBIF: https://doi.org/10.15468/dl.zfaw79
Anopheles barbirostris	109.13714	-7.681584	Unclassified	GBIF: https://doi.org/10.15468/dl.zfaw79
Anopheles barbirostris	109.6	19.6	Unclassified	GBIF: https://doi.org/10.15468/dl.zfaw79
Anopheles barbirostris	109.6	18.3	Unclassified	GBIF: https://doi.org/10.15468/dl.zfaw79
Anopheles barbirostris	110.217656	-7.479992	Unclassified	GBIF: https://doi.org/10.15468/dl.zfaw79
Anopheles barbirostris	110.28	1.28	Unclassified	GBIF: https://doi.org/10.15468/dl.zfaw79
Anopheles barbirostris	110.28	1.25	Unclassified	GBIF: https://doi.org/10.15468/dl.zfaw79
Anopheles barbirostris	110.333	1.4167	Unclassified	GBIF: https://doi.org/10.15468/dl.zfaw79
Anopheles barbirostris	110.818812	-7.559209	Unclassified	GBIF: https://doi.org/10.15468/dl.zfaw79
Anopheles barbirostris	112.749444	-7.256111	Unclassified	GBIF: https://doi.org/10.15468/dl.zfaw79
Anopheles barbirostris	115.2167	5.35	Unclassified	GBIF: https://doi.org/10.15468/dl.zfaw79
Anopheles barbirostris	116.8333	-1.2833	Unclassified	GBIF: https://doi.org/10.15468/dl.zfaw79
Anopheles barbirostris	118.802871	9.915757	Unclassified	GBIF: https://doi.org/10.15468/dl.zfaw79
Anopheles barbirostris	119.872251	-0.819373	Unclassified	GBIF: https://doi.org/10.15468/dl.zfaw79
Anopheles barbirostris	119.966516	-1.170781	Unclassified	GBIF: https://doi.org/10.15468/dl.zfaw79

Anopheles barbirostris	119.968603	-1.171993	Unclassified	GBIF: https://doi.org/10.15468/dl.zfaw79
Anopheles barbirostris	120.267798	15.902717	Unclassified	GBIF: https://doi.org/10.15468/dl.zfaw79
Anopheles barbirostris	120.40565	16.240515	Unclassified	GBIF: https://doi.org/10.15468/dl.zfaw79
Anopheles barbirostris	120.411747	16.223892	Unclassified	GBIF: https://doi.org/10.15468/dl.zfaw79
Anopheles barbirostris	120.411899	16.237991	Unclassified	GBIF: https://doi.org/10.15468/dl.zfaw79
Anopheles barbirostris	120.420556	16.21	Unclassified	GBIF: https://doi.org/10.15468/dl.zfaw79
Anopheles barbirostris	120.422963	16.216198	Unclassified	GBIF: https://doi.org/10.15468/dl.zfaw79
Anopheles barbirostris	121.037248	14.666877	Unclassified	GBIF: https://doi.org/10.15468/dl.zfaw79
Anopheles barbirostris	121.037702	14.671363	Unclassified	GBIF: https://doi.org/10.15468/dl.zfaw79
Anopheles barbirostris	121.0549	14.3125	Unclassified	GBIF: https://doi.org/10.15468/dl.zfaw79
Anopheles barbirostris	121.065918	12.347433	Unclassified	GBIF: https://doi.org/10.15468/dl.zfaw79
Anopheles barbirostris	121.067613	12.352748	Unclassified	GBIF: https://doi.org/10.15468/dl.zfaw79
Anopheles barbirostris	121.238905	16.268563	Unclassified	GBIF: https://doi.org/10.15468/dl.zfaw79
Anopheles barbirostris	121.974109	-5.31601	Unclassified	GBIF: https://doi.org/10.15468/dl.zfaw79
Anopheles barbirostris	122.017222	0.981944	Unclassified	GBIF: https://doi.org/10.15468/dl.zfaw79
Anopheles barbirostris	122.038574	-5.309061	Unclassified	GBIF: https://doi.org/10.15468/dl.zfaw79
Anopheles barbirostris	122.188	7.024	Unclassified	GBIF: https://doi.org/10.15468/dl.zfaw79
Anopheles barbirostris	124.675556	11.299167	Unclassified	GBIF: https://doi.org/10.15468/dl.zfaw79
Anopheles barbirostris	124.685761	11.302971	Unclassified	GBIF: https://doi.org/10.15468/dl.zfaw79
Anopheles barbirostris	124.757122	11.243803	Unclassified	GBIF: https://doi.org/10.15468/dl.zfaw79
Anopheles barbirostris	124.833333	10.833333	Unclassified	GBIF: https://doi.org/10.15468/dl.zfaw79
Anopheles barbirostris	124.884524	10.138572	Unclassified	GBIF: https://doi.org/10.15468/dl.zfaw79
Anopheles barbirostris	124.88499	10.139394	Unclassified	GBIF: https://doi.org/10.15468/dl.zfaw79
Anopheles barbirostris	124.983333	10.883333	Unclassified	GBIF: https://doi.org/10.15468/dl.zfaw79
Anopheles barbirostris	126.780278	-3.389444	Unclassified	GBIF: https://doi.org/10.15468/dl.zfaw79
Anopheles barbirostris	128.684444	-2.867778	Unclassified	GBIF: https://doi.org/10.15468/dl.zfaw79
Anopheles barbumbrosus	85.002554	27.296121	Unclassified	GBIF: https://doi.org/10.15468/dl.zfaw79
Anopheles barbumbrosus	95.323611	5.549444	Unclassified	GBIF: https://doi.org/10.15468/dl.zfaw79
Anopheles barbumbrosus	98.459667	18.502778	Unclassified	GBIF: https://doi.org/10.15468/dl.zfaw79
Anopheles barbumbrosus	98.578056	18.541972	Unclassified	GBIF: https://doi.org/10.15468/dl.zfaw79
Anopheles barbumbrosus	98.601861	18.528194	Unclassified	GBIF: https://doi.org/10.15468/dl.zfaw79
Anopheles barbumbrosus	98.619694	18.570389	Unclassified	GBIF: https://doi.org/10.15468/dl.zfaw79
Anopheles barbumbrosus	98.62446	8.58293	Unclassified	GBIF: https://doi.org/10.15468/dl.zfaw79
Anopheles barbumbrosus	98.82057	14.40306	Unclassified	GBIF: https://doi.org/10.15468/dl.zfaw79
Anopheles barbumbrosus	98.83911	14.42116	Unclassified	GBIF: https://doi.org/10.15468/dl.zfaw79
Anopheles barbumbrosus	98.87621	14.43022	Unclassified	GBIF: https://doi.org/10.15468/dl.zfaw79
Anopheles barbumbrosus	98.89989	18.90584	Unclassified	GBIF: https://doi.org/10.15468/dl.zfaw79
Anopheles barbumbrosus	98.93293	19.38666	Unclassified	GBIF: https://doi.org/10.15468/dl.zfaw79
Anopheles barbumbrosus	98.97828	14.27654	Unclassified	GBIF: https://doi.org/10.15468/dl.zfaw79
Anopheles barbumbrosus	98.98257	19.16918	Unclassified	GBIF: https://doi.org/10.15468/dl.zfaw79
Anopheles barbumbrosus	99.06171	14.24037	Unclassified	GBIF: https://doi.org/10.15468/dl.zfaw79
Anopheles barbumbrosus	99.06171	14.24941	Unclassified	GBIF: https://doi.org/10.15468/dl.zfaw79

Anopheles barbumbrosus	99.08024	14.22228	Unclassified	GBIF: https://doi.org/10.15468/dl.zfaw79
Anopheles barbumbrosus	99.088655	14.194176	Unclassified	GBIF: https://doi.org/10.15468/dl.zfaw79
Anopheles barbumbrosus	99.19213	16.500795	Unclassified	GBIF: https://doi.org/10.15468/dl.zfaw79
Anopheles barbumbrosus	99.2725	1.378056	Unclassified	GBIF: https://doi.org/10.15468/dl.zfaw79
Anopheles barbumbrosus	99.338339	13.039994	Unclassified	GBIF: https://doi.org/10.15468/dl.zfaw79
Anopheles barbumbrosus	99.359444	0.798056	Unclassified	GBIF: https://doi.org/10.15468/dl.zfaw79
Anopheles barbumbrosus	99.379358	13.671355	Unclassified	GBIF: https://doi.org/10.15468/dl.zfaw79
Anopheles barbumbrosus	99.408709	13.848447	Unclassified	GBIF: https://doi.org/10.15468/dl.zfaw79
Anopheles barbumbrosus	99.67948	18.66875	Unclassified	GBIF: https://doi.org/10.15468/dl.zfaw79
Anopheles barbumbrosus	99.744448	13.199366	Unclassified	GBIF: https://doi.org/10.15468/dl.zfaw79
Anopheles barbumbrosus	99.80425	18.98456	Unclassified	GBIF: https://doi.org/10.15468/dl.zfaw79
Anopheles barbumbrosus	99.84085	18.70425	Unclassified	GBIF: https://doi.org/10.15468/dl.zfaw79
Anopheles barbumbrosus	99.86834	18.51434	Unclassified	GBIF: https://doi.org/10.15468/dl.zfaw79
Anopheles barbumbrosus	99.87074	18.98426	Unclassified	GBIF: https://doi.org/10.15468/dl.zfaw79
Anopheles barbumbrosus	99.89309	18.72027	Unclassified	GBIF: https://doi.org/10.15468/dl.zfaw79
Anopheles barbumbrosus	99.89454	18.99228	Unclassified	GBIF: https://doi.org/10.15468/dl.zfaw79
Anopheles barbumbrosus	99.89914	18.96605	Unclassified	GBIF: https://doi.org/10.15468/dl.zfaw79
Anopheles barbumbrosus	99.89924	18.98412	Unclassified	GBIF: https://doi.org/10.15468/dl.zfaw79
Anopheles barbumbrosus	99.93681	18.90712	Unclassified	GBIF: https://doi.org/10.15468/dl.zfaw79
Anopheles barbumbrosus	99.93683	18.91164	Unclassified	GBIF: https://doi.org/10.15468/dl.zfaw79
Anopheles barbumbrosus	99.99285	18.74869	Unclassified	GBIF: https://doi.org/10.15468/dl.zfaw79
Anopheles barbumbrosus	100.3675	-0.300278	Unclassified	GBIF: https://doi.org/10.15468/dl.zfaw79
Anopheles barbumbrosus	100.49262	18.41105	Unclassified	GBIF: https://doi.org/10.15468/dl.zfaw79
Anopheles barbumbrosus	100.4927	18.42009	Unclassified	GBIF: https://doi.org/10.15468/dl.zfaw79
Anopheles barbumbrosus	100.59782	18.52765	Unclassified	GBIF: https://doi.org/10.15468/dl.zfaw79
Anopheles barbumbrosus	100.63537	18.49119	Unclassified	GBIF: https://doi.org/10.15468/dl.zfaw79
Anopheles barbumbrosus	100.7247	18.89696	Unclassified	GBIF: https://doi.org/10.15468/dl.zfaw79
Anopheles barbumbrosus	100.75383	18.95993	Unclassified	GBIF: https://doi.org/10.15468/dl.zfaw79
Anopheles barbumbrosus	100.76313	18.94177	Unclassified	GBIF: https://doi.org/10.15468/dl.zfaw79
Anopheles barbumbrosus	100.79171	18.95053	Unclassified	GBIF: https://doi.org/10.15468/dl.zfaw79
Anopheles barbumbrosus	101.048138	13.309745	Unclassified	GBIF: https://doi.org/10.15468/dl.zfaw79
Anopheles barbumbrosus	101.252322	15.805219	Unclassified	GBIF: https://doi.org/10.15468/dl.zfaw79
Anopheles barbumbrosus	101.39037	14.48139	Unclassified	GBIF: https://doi.org/10.15468/dl.zfaw79
Anopheles barbumbrosus	101.39096	14.53559	Unclassified	GBIF: https://doi.org/10.15468/dl.zfaw79
Anopheles barbumbrosus	101.39906	14.42709	Unclassified	GBIF: https://doi.org/10.15468/dl.zfaw79
Anopheles barbumbrosus	101.4474	14.60728	Unclassified	GBIF: https://doi.org/10.15468/dl.zfaw79
Anopheles barbumbrosus	101.48209	14.3901	Unclassified	GBIF: https://doi.org/10.15468/dl.zfaw79
Anopheles barbumbrosus	101.8335	14.33195	Unclassified	GBIF: https://doi.org/10.15468/dl.zfaw79
Anopheles barbumbrosus	101.89753	14.26796	Unclassified	GBIF: https://doi.org/10.15468/dl.zfaw79
Anopheles barbumbrosus	101.914358	14.389737	Unclassified	GBIF: https://doi.org/10.15468/dl.zfaw79
Anopheles barbumbrosus	102.19419	12.95941	Unclassified	GBIF: https://doi.org/10.15468/dl.zfaw79
Anopheles barbumbrosus	102.19879	12.95946	Unclassified	GBIF: https://doi.org/10.15468/dl.zfaw79

Anopheles barbumbrosus	102.20335	12.96403	Unclassified	GBIF: https://doi.org/10.15468/dl.zfaw79
Anopheles barbumbrosus	102.2034	12.95951	Unclassified	GBIF: https://doi.org/10.15468/dl.zfaw79
Anopheles barbumbrosus	102.21261	12.95961	Unclassified	GBIF: https://doi.org/10.15468/dl.zfaw79
Anopheles barbumbrosus	102.25759	16.72658	Unclassified	GBIF: https://doi.org/10.15468/dl.zfaw79
Anopheles barbumbrosus	102.26631	16.77185	Unclassified	GBIF: https://doi.org/10.15468/dl.zfaw79
Anopheles barbumbrosus	102.574818	14.044675	Unclassified	GBIF: https://doi.org/10.15468/dl.zfaw79
Anopheles barbumbrosus	103.444973	14.449552	Unclassified	GBIF: https://doi.org/10.15468/dl.zfaw79
Anopheles barbumbrosus	104.69114	38.073254	Unclassified	GBIF: https://doi.org/10.15468/dl.zfaw79
Anopheles barbumbrosus	108.905278	-7.724444	Unclassified	GBIF: https://doi.org/10.15468/dl.zfaw79
Anopheles barbumbrosus	116.79	6.77	Unclassified	GBIF: https://doi.org/10.15468/dl.zfaw79
Anopheles barbumbrosus	122.038574	-5.309061	Unclassified	GBIF: https://doi.org/10.15468/dl.zfaw79
Anopheles barbumbrosus	128.1025	-3.623333	Unclassified	GBIF: https://doi.org/10.15468/dl.zfaw79
Anopheles barbumbrosus	128.19	-3.058889	Unclassified	GBIF: https://doi.org/10.15468/dl.zfaw79
Anopheles barbumbrosus	128.4167	2.3333	Unclassified	GBIF: https://doi.org/10.15468/dl.zfaw79
Anopheles barbumbrosus	128.592778	-2.852222	Unclassified	GBIF: https://doi.org/10.15468/dl.zfaw79
Anopheles barbumbrosus	128.681527	-2.868118	Unclassified	GBIF: https://doi.org/10.15468/dl.zfaw79
Anopheles barbumbrosus	128.684444	-2.867778	Unclassified	GBIF: https://doi.org/10.15468/dl.zfaw79
Anopheles barbumbrosus	128.922778	-3.339167	Unclassified	GBIF: https://doi.org/10.15468/dl.zfaw79
Anopheles barbumbrosus	129.054098	-2.831123	Unclassified	GBIF: https://doi.org/10.15468/dl.zfaw79
Anopheles barbumbrosus	129.054722	-2.830556	Unclassified	GBIF: https://doi.org/10.15468/dl.zfaw79
Anopheles beklemishevi	10.033258	59.744871	Unclassified	GBIF: https://doi.org/10.15468/dl.zfaw79
Anopheles beklemishevi	11.068906	63.238392	Unclassified	GBIF: https://doi.org/10.15468/dl.zfaw79
Anopheles beklemishevi	14.13552	61.86726	Unclassified	GBIF: https://doi.org/10.15468/dl.zfaw79
Anopheles beklemishevi	16.511281	61.875424	Unclassified	GBIF: https://doi.org/10.15468/dl.zfaw79
Anopheles beklemishevi	18.25	66.87	Unclassified	GBIF: https://doi.org/10.15468/dl.zfaw79
Anopheles beklemishevi	18.4	59.37	Unclassified	GBIF: https://doi.org/10.15468/dl.zfaw79
Anopheles beklemishevi	19.84261	66.597345	Unclassified	GBIF: https://doi.org/10.15468/dl.zfaw79
Anopheles beklemishevi	24.227725	68.147009	Unclassified	GBIF: https://doi.org/10.15468/dl.zfaw79
Anopheles beklemishevi	24.91	67.66	Unclassified	GBIF: https://doi.org/10.15468/dl.zfaw79
Anopheles beklemishevi	25.745404	62.250136	Unclassified	GBIF: https://doi.org/10.15468/dl.zfaw79
Anopheles beklemishevi	25.825323	65.116732	Unclassified	GBIF: https://doi.org/10.15468/dl.zfaw79
Anopheles beklemishevi	27.49	69.07	Unclassified	GBIF: https://doi.org/10.15468/dl.zfaw79
Anopheles beklemishevi	27.64	64.87	Unclassified	GBIF: https://doi.org/10.15468/dl.zfaw79
Anopheles beklemishevi	27.81	65.08	Unclassified	GBIF: https://doi.org/10.15468/dl.zfaw79
Anopheles beklemishevi	29.23	66.31	Unclassified	GBIF: https://doi.org/10.15468/dl.zfaw79
Anopheles beklemishevi	29.24398	69.16016	Unclassified	GBIF: https://doi.org/10.15468/dl.zfaw79
Anopheles beklemishevi	29.247	69.1647	Unclassified	GBIF: https://doi.org/10.15468/dl.zfaw79
Anopheles beklemishevi	29.31	66.37	Unclassified	GBIF: https://doi.org/10.15468/dl.zfaw79
Anopheles beklemishevi	34.59837	64.95553	Unclassified	GBIF: https://doi.org/10.15468/dl.zfaw79
Anopheles beklemishevi	53.684025	63.562626	Unclassified	GBIF: https://doi.org/10.15468/dl.zfaw79
Anopheles beklemishevi	82.6	57.9	Unclassified	GBIF: https://doi.org/10.15468/dl.zfaw79
Anopheles beklemishevi	88.2	57.3	Unclassified	GBIF: https://doi.org/10.15468/dl.zfaw79

Anopheles belenrae	116.58015	35.41003	Unclassified	GBIF: https://doi.org/10.15468/dl.zfaw79
Anopheles belenrae	121.50015	39.77003	Unclassified	GBIF: https://doi.org/10.15468/dl.zfaw79
Anopheles belenrae	126.48333	37.75	Unclassified	GBIF: https://doi.org/10.15468/dl.zfaw79
Anopheles belenrae	126.53	37.74	Unclassified	GBIF: https://doi.org/10.15468/dl.zfaw79
Anopheles belenrae	126.62282	35.90998	Unclassified	GBIF: https://doi.org/10.15468/dl.zfaw79
Anopheles belenrae	126.65192	37.71319	Unclassified	GBIF: https://doi.org/10.15468/dl.zfaw79
Anopheles belenrae	126.69833	37.66833	Unclassified	GBIF: https://doi.org/10.15468/dl.zfaw79
Anopheles belenrae	126.7	37.85	Unclassified	GBIF: https://doi.org/10.15468/dl.zfaw79
Anopheles belenrae	126.71667	37.81667	Unclassified	GBIF: https://doi.org/10.15468/dl.zfaw79
Anopheles belenrae	126.72362	37.93156	Unclassified	GBIF: https://doi.org/10.15468/dl.zfaw79
Anopheles belenrae	126.73367	37.90517	Unclassified	GBIF: https://doi.org/10.15468/dl.zfaw79
Anopheles belenrae	126.7373	37.90802	Unclassified	GBIF: https://doi.org/10.15468/dl.zfaw79
Anopheles belenrae	126.74159	37.91911	Unclassified	GBIF: https://doi.org/10.15468/dl.zfaw79
Anopheles belenrae	126.74172	37.92204	Unclassified	GBIF: https://doi.org/10.15468/dl.zfaw79
Anopheles belenrae	126.75911	37.87861	Unclassified	GBIF: https://doi.org/10.15468/dl.zfaw79
Anopheles belenrae	126.79071	37.92292	Unclassified	GBIF: https://doi.org/10.15468/dl.zfaw79
Anopheles belenrae	126.80189	37.89508	Unclassified	GBIF: https://doi.org/10.15468/dl.zfaw79
Anopheles belenrae	126.80287	37.75586	Unclassified	GBIF: https://doi.org/10.15468/dl.zfaw79
Anopheles belenrae	126.8032	35.12422	Unclassified	GBIF: https://doi.org/10.15468/dl.zfaw79
Anopheles belenrae	126.82971	37.89038	Unclassified	GBIF: https://doi.org/10.15468/dl.zfaw79
Anopheles belenrae	126.8324	37.89359	Unclassified	GBIF: https://doi.org/10.15468/dl.zfaw79
Anopheles belenrae	126.9491	37.97792	Unclassified	GBIF: https://doi.org/10.15468/dl.zfaw79
Anopheles belenrae	127.01256	38.04484	Unclassified	GBIF: https://doi.org/10.15468/dl.zfaw79
Anopheles belenrae	127.03031	36.95795	Unclassified	GBIF: https://doi.org/10.15468/dl.zfaw79
Anopheles belenrae	127.0383	36.96114	Unclassified	GBIF: https://doi.org/10.15468/dl.zfaw79
Anopheles belenrae	127.10795	38.04618	Unclassified	GBIF: https://doi.org/10.15468/dl.zfaw79
Anopheles belenrae	127.15597	37.37981	Unclassified	GBIF: https://doi.org/10.15468/dl.zfaw79
Anopheles belenrae	127.19567	37.4765	Unclassified	GBIF: https://doi.org/10.15468/dl.zfaw79
Anopheles belenrae	127.23071	38.03663	Unclassified	GBIF: https://doi.org/10.15468/dl.zfaw79
Anopheles belenrae	127.24153	37.246	Unclassified	GBIF: https://doi.org/10.15468/dl.zfaw79
Anopheles belenrae	127.25975	38.02564	Unclassified	GBIF: https://doi.org/10.15468/dl.zfaw79
Anopheles belenrae	127.26153	37.96286	Unclassified	GBIF: https://doi.org/10.15468/dl.zfaw79
Anopheles belenrae	127.31883	37.90394	Unclassified	GBIF: https://doi.org/10.15468/dl.zfaw79
Anopheles belenrae	127.52522	38.04956	Unclassified	GBIF: https://doi.org/10.15468/dl.zfaw79
Anopheles belenrae	127.52703	38.08531	Unclassified	GBIF: https://doi.org/10.15468/dl.zfaw79
Anopheles belenrae	127.52881	38.17025	Unclassified	GBIF: https://doi.org/10.15468/dl.zfaw79
Anopheles belenrae	127.62317	36.77192	Unclassified	GBIF: https://doi.org/10.15468/dl.zfaw79
Anopheles belenrae	127.70719	37.86369	Unclassified	GBIF: https://doi.org/10.15468/dl.zfaw79
Anopheles belenrae	127.95293	37.39079	Unclassified	GBIF: https://doi.org/10.15468/dl.zfaw79
Anopheles belenrae	127.95644	37.40658	Unclassified	GBIF: https://doi.org/10.15468/dl.zfaw79
Anopheles belenrae	127.96047	37.12158	Unclassified	GBIF: https://doi.org/10.15468/dl.zfaw79
Anopheles belenrae	127.97342	37.45056	Unclassified	GBIF: https://doi.org/10.15468/dl.zfaw79

Anopheles belenrae	128.0045	36.47881	Unclassified	GBIF: https://doi.org/10.15468/dl.zfaw79
Anopheles belenrae	128.13942	36.928	Unclassified	GBIF: https://doi.org/10.15468/dl.zfaw79
Anopheles belenrae	128.21264	36.46786	Unclassified	GBIF: https://doi.org/10.15468/dl.zfaw79
Anopheles belenrae	128.84192	37.72811	Unclassified	GBIF: https://doi.org/10.15468/dl.zfaw79
Anopheles belenrae	128.94711	36.91592	Unclassified	GBIF: https://doi.org/10.15468/dl.zfaw79
Anopheles belenrae	129.19161	37.13942	Unclassified	GBIF: https://doi.org/10.15468/dl.zfaw79
Anopheles belenrae	129.24178	35.80222	Unclassified	GBIF: https://doi.org/10.15468/dl.zfaw79
Anopheles belenrae	129.31133	37.15372	Unclassified	GBIF: https://doi.org/10.15468/dl.zfaw79
Anopheles belenrae	129.3775	36.96881	Unclassified	GBIF: https://doi.org/10.15468/dl.zfaw79
Anopheles belenrae	129.39436	36.9015	Unclassified	GBIF: https://doi.org/10.15468/dl.zfaw79
Anopheles bellator	-75.383	0.75	Primary	GBIF: https://doi.org/10.15468/dl.zfaw79
Anopheles bellator	-75.38	0.75	Primary	GBIF: https://doi.org/10.15468/dl.zfaw79
Anopheles bellator	-61.2631	10.37305	Primary	GBIF: https://doi.org/10.15468/dl.zfaw79
Anopheles bellator	-61.25	10.733333	Primary	GBIF: https://doi.org/10.15468/dl.zfaw79
Anopheles bellator	-61.2	10.4	Primary	GBIF: https://doi.org/10.15468/dl.zfaw79
Anopheles bellator	-61.19843	10.58506	Primary	GBIF: https://doi.org/10.15468/dl.zfaw79
Anopheles bellator	-61.17	10.47	Primary	GBIF: https://doi.org/10.15468/dl.zfaw79
Anopheles bellator	-61.16068	10.70696	Primary	GBIF: https://doi.org/10.15468/dl.zfaw79
Anopheles bellator	-61.14	10.46	Primary	GBIF: https://doi.org/10.15468/dl.zfaw79
Anopheles bellator	-61.132198	10.587123	Primary	GBIF: https://doi.org/10.15468/dl.zfaw79
Anopheles bellator	-61.11641	10.47166	Primary	GBIF: https://doi.org/10.15468/dl.zfaw79
Anopheles bellator	-61.11509	10.58506	Primary	GBIF: https://doi.org/10.15468/dl.zfaw79
Anopheles bellator	-58.08486	6.36346	Primary	GBIF: https://doi.org/10.15468/dl.zfaw79
Anopheles bellator	-48.5427	-25.5128	Primary	GBIF: https://doi.org/10.15468/dl.zfaw79
Anopheles bellator	-47.98	-24.95	Primary	GBIF: https://doi.org/10.15468/dl.zfaw79
Anopheles bellator	-47.978	-24.953	Primary	GBIF: https://doi.org/10.15468/dl.zfaw79
Anopheles bellator	-47.95	-24.748	Primary	GBIF: https://doi.org/10.15468/dl.zfaw79
Anopheles bellator	-47.95	-24.75	Primary	GBIF: https://doi.org/10.15468/dl.zfaw79
Anopheles bellator	-47.851	-24.888	Primary	GBIF: https://doi.org/10.15468/dl.zfaw79
Anopheles bellator	-47.85	-24.89	Primary	GBIF: https://doi.org/10.15468/dl.zfaw79
Anopheles benarrochi	-77.5832	-3.314419	Unclassified	GBIF: https://doi.org/10.15468/dl.zfaw79
Anopheles benarrochi	-76.8	-2.58	Unclassified	GBIF: https://doi.org/10.15468/dl.zfaw79
Anopheles benarrochi	-76.46115	-4.658039	Unclassified	GBIF: https://doi.org/10.15468/dl.zfaw79
Anopheles benarrochi	-76.40269	-2.905164	Unclassified	GBIF: https://doi.org/10.15468/dl.zfaw79
Anopheles benarrochi	-76.4	-2.91	Unclassified	GBIF: https://doi.org/10.15468/dl.zfaw79
Anopheles benarrochi	-76.35569	-3.670834	Unclassified	GBIF: https://doi.org/10.15468/dl.zfaw79
Anopheles benarrochi	-73.75197	-3.92013	Unclassified	GBIF: https://doi.org/10.15468/dl.zfaw79
Anopheles benarrochi	-73.61867	4.14669	Unclassified	GBIF: https://doi.org/10.15468/dl.zfaw79
Anopheles benarrochi	-73.32	-3.898	Unclassified	GBIF: https://doi.org/10.15468/dl.zfaw79
Anopheles benarrochi	-72.89	-7.62	Unclassified	GBIF: https://doi.org/10.15468/dl.zfaw79
Anopheles benarrochi	-72.886	-7.62	Unclassified	GBIF: https://doi.org/10.15468/dl.zfaw79
Anopheles benarrochi	-72.82	-7.596	Unclassified	GBIF: https://doi.org/10.15468/dl.zfaw79

Anopheles benarrochi	-72.82	-7.6	Unclassified	GBIF: https://doi.org/10.15468/dl.zfaw79
Anopheles benarrochi	-72.68232	-7.7712	Unclassified	GBIF: https://doi.org/10.15468/dl.zfaw79
Anopheles benarrochi	-72.68	-7.77	Unclassified	GBIF: https://doi.org/10.15468/dl.zfaw79
Anopheles benarrochi	-70.3	-13.08	Unclassified	GBIF: https://doi.org/10.15468/dl.zfaw79
Anopheles benarrochi	-69.17	-11.72	Unclassified	GBIF: https://doi.org/10.15468/dl.zfaw79
Anopheles benarrochi	-67.143	-9.702	Unclassified	GBIF: https://doi.org/10.15468/dl.zfaw79
Anopheles benarrochi	-67.14	-9.7	Unclassified	GBIF: https://doi.org/10.15468/dl.zfaw79
Anopheles benarrochi	-67.13481	-9.68431	Unclassified	GBIF: https://doi.org/10.15468/dl.zfaw79
Anopheles benarrochi	-67.13	-9.68	Unclassified	GBIF: https://doi.org/10.15468/dl.zfaw79
Anopheles benarrochi	-65.2828	18.31733	Unclassified	GBIF: https://doi.org/10.15468/dl.zfaw79
Anopheles benarrochi	-62.29	-9.25	Unclassified	GBIF: https://doi.org/10.15468/dl.zfaw79
Anopheles benarrochi	-62.26	-9.22	Unclassified	GBIF: https://doi.org/10.15468/dl.zfaw79
Anopheles benarrochi	-62.25	-9.25	Unclassified	GBIF: https://doi.org/10.15468/dl.zfaw79
Anopheles benarrochi	-60.82	-7.596	Unclassified	GBIF: https://doi.org/10.15468/dl.zfaw79
Anopheles benarrochi	-60.82	-7.6	Unclassified	GBIF: https://doi.org/10.15468/dl.zfaw79
Anopheles benarrochi	-43.292801	-22.1619	Unclassified	GBIF: https://doi.org/10.15468/dl.zfaw79
Anopheles benarrochi	-41.7869	-22.3708	Unclassified	GBIF: https://doi.org/10.15468/dl.zfaw79
Anopheles bengalensis	98.477889	18.553611	Unclassified	GBIF: https://doi.org/10.15468/dl.zfaw79
Anopheles bengalensis	98.502	18.536056	Unclassified	GBIF: https://doi.org/10.15468/dl.zfaw79
Anopheles bengalensis	98.51073	8.45674	Unclassified	GBIF: https://doi.org/10.15468/dl.zfaw79
Anopheles bengalensis	98.535028	18.574194	Unclassified	GBIF: https://doi.org/10.15468/dl.zfaw79
Anopheles bengalensis	98.82057	14.40306	Unclassified	GBIF: https://doi.org/10.15468/dl.zfaw79
Anopheles bengalensis	98.93316	18.79831	Unclassified	GBIF: https://doi.org/10.15468/dl.zfaw79
Anopheles bengalensis	98.93411	18.79921	Unclassified	GBIF: https://doi.org/10.15468/dl.zfaw79
Anopheles bengalensis	99.00054	18.79561	Unclassified	GBIF: https://doi.org/10.15468/dl.zfaw79
Anopheles bengalensis	99.496263	11.223328	Unclassified	GBIF: https://doi.org/10.15468/dl.zfaw79
Anopheles bengalensis	99.67948	18.66875	Unclassified	GBIF: https://doi.org/10.15468/dl.zfaw79
Anopheles bengalensis	99.690027	8.027579	Unclassified	GBIF: https://doi.org/10.15468/dl.zfaw79
Anopheles bengalensis	99.80425	18.98456	Unclassified	GBIF: https://doi.org/10.15468/dl.zfaw79
Anopheles bengalensis	99.89309	18.72027	Unclassified	GBIF: https://doi.org/10.15468/dl.zfaw79
Anopheles bengalensis	99.89454	18.99228	Unclassified	GBIF: https://doi.org/10.15468/dl.zfaw79
Anopheles bengalensis	99.90211	18.98772	Unclassified	GBIF: https://doi.org/10.15468/dl.zfaw79
Anopheles bengalensis	99.94543	15.59635	Unclassified	GBIF: https://doi.org/10.15468/dl.zfaw79
Anopheles bengalensis	100.4927	18.42009	Unclassified	GBIF: https://doi.org/10.15468/dl.zfaw79
Anopheles bengalensis	100.508752	20.071488	Unclassified	GBIF: https://doi.org/10.15468/dl.zfaw79
Anopheles bengalensis	101.39037	14.48139	Unclassified	GBIF: https://doi.org/10.15468/dl.zfaw79
Anopheles bengalensis	102.15868	12.8416	Unclassified	GBIF: https://doi.org/10.15468/dl.zfaw79
Anopheles bengalensis	102.18498	12.95931	Unclassified	GBIF: https://doi.org/10.15468/dl.zfaw79
Anopheles bengalensis	102.18943	12.97291	Unclassified	GBIF: https://doi.org/10.15468/dl.zfaw79
Anopheles bengalensis	102.19419	12.95941	Unclassified	GBIF: https://doi.org/10.15468/dl.zfaw79
Anopheles bengalensis	102.19879	12.95946	Unclassified	GBIF: https://doi.org/10.15468/dl.zfaw79
Anopheles bengalensis	102.20335	12.96403	Unclassified	GBIF: https://doi.org/10.15468/dl.zfaw79

Anopheles bengalensis	102.2036	12.94145	Unclassified	GBIF: https://doi.org/10.15468/dl.zfaw79
Anopheles bengalensis	102.20375	12.9279	Unclassified	GBIF: https://doi.org/10.15468/dl.zfaw79
Anopheles bengalensis	102.20825	12.93698	Unclassified	GBIF: https://doi.org/10.15468/dl.zfaw79
Anopheles bengalensis	104.455708	16.414059	Unclassified	GBIF: https://doi.org/10.15468/dl.zfaw79
Anopheles bironelli	128.684444	-2.867778	Unclassified	GBIF: https://doi.org/10.15468/dl.zfaw79
Anopheles bradleyi	-97.868611	22.255278	Unclassified	GBIF: https://doi.org/10.15468/dl.zfaw79
Anopheles bradleyi	-97.836111	22.183333	Unclassified	GBIF: https://doi.org/10.15468/dl.zfaw79
Anopheles bradleyi	-97.788889	20.977778	Unclassified	GBIF: https://doi.org/10.15468/dl.zfaw79
Anopheles bradleyi	-97.716667	24.626389	Unclassified	GBIF: https://doi.org/10.15468/dl.zfaw79
Anopheles bradleyi	-97.692778	25.940556	Unclassified	GBIF: https://doi.org/10.15468/dl.zfaw79
Anopheles bradleyi	-97.304069	20.965877	Unclassified	GBIF: https://doi.org/10.15468/dl.zfaw79
Anopheles bradleyi	-97.2957	27.431074	Unclassified	GBIF: https://doi.org/10.15468/dl.zfaw79
Anopheles bradleyi	-94.092778	17.91	Unclassified	GBIF: https://doi.org/10.15468/dl.zfaw79
Anopheles bradleyi	-93.92861	29.89861	Unclassified	GBIF: https://doi.org/10.15468/dl.zfaw79
Anopheles bradleyi	-91.426389	17.470833	Unclassified	GBIF: https://doi.org/10.15468/dl.zfaw79
Anopheles bradleyi	-90.531667	19.842222	Unclassified	GBIF: https://doi.org/10.15468/dl.zfaw79
Anopheles bradleyi	-89.663333	21.284444	Unclassified	GBIF: https://doi.org/10.15468/dl.zfaw79
Anopheles bradleyi	-89.07771	30.428126	Unclassified	GBIF: https://doi.org/10.15468/dl.zfaw79
Anopheles bradleyi	-86.833611	21.161111	Unclassified	GBIF: https://doi.org/10.15468/dl.zfaw79
Anopheles bradleyi	-85.647718	30.181853	Unclassified	GBIF: https://doi.org/10.15468/dl.zfaw79
Anopheles bradleyi	-82.72	34.89	Unclassified	GBIF: https://doi.org/10.15468/dl.zfaw79
Anopheles bradleyi	-82.6528	28.4509	Unclassified	GBIF: https://doi.org/10.15468/dl.zfaw79
Anopheles bradleyi	-82.6165	28.4817	Unclassified	GBIF: https://doi.org/10.15468/dl.zfaw79
Anopheles bradleyi	-82.577	28.5731	Unclassified	GBIF: https://doi.org/10.15468/dl.zfaw79
Anopheles bradleyi	-82.555	28.4927	Unclassified	GBIF: https://doi.org/10.15468/dl.zfaw79
Anopheles bradleyi	-82.548	27.5909	Unclassified	GBIF: https://doi.org/10.15468/dl.zfaw79
Anopheles bradleyi	-82.3848	28.571	Unclassified	GBIF: https://doi.org/10.15468/dl.zfaw79
Anopheles bradleyi	-82.3539	28.5431	Unclassified	GBIF: https://doi.org/10.15468/dl.zfaw79
Anopheles bradleyi	-82.2642	28.6428	Unclassified	GBIF: https://doi.org/10.15468/dl.zfaw79
Anopheles bradleyi	-82.1746	28.4942	Unclassified	GBIF: https://doi.org/10.15468/dl.zfaw79
Anopheles bradleyi	-81.506186	27.595867	Unclassified	GBIF: https://doi.org/10.15468/dl.zfaw79
Anopheles bradleyi	-81.481	29.9774	Unclassified	GBIF: https://doi.org/10.15468/dl.zfaw79
Anopheles bradleyi	-81.47	31.36	Unclassified	GBIF: https://doi.org/10.15468/dl.zfaw79
Anopheles bradleyi	-81.4328	30.2479	Unclassified	GBIF: https://doi.org/10.15468/dl.zfaw79
Anopheles bradleyi	-81.4182	30.2362	Unclassified	GBIF: https://doi.org/10.15468/dl.zfaw79
Anopheles bradleyi	-81.402	30.2515	Unclassified	GBIF: https://doi.org/10.15468/dl.zfaw79
Anopheles bradleyi	-81.4009	30.2517	Unclassified	GBIF: https://doi.org/10.15468/dl.zfaw79
Anopheles bradleyi	-81	34	Unclassified	GBIF: https://doi.org/10.15468/dl.zfaw79
Anopheles bradleyi	-79.98	32.77	Unclassified	GBIF: https://doi.org/10.15468/dl.zfaw79
Anopheles bradleyi	-79.95	33.2	Unclassified	GBIF: https://doi.org/10.15468/dl.zfaw79
Anopheles bradleyi	-76.6604	37.332009	Unclassified	GBIF: https://doi.org/10.15468/dl.zfaw79
Anopheles braziliensis	-73.71966	3.56736	Secondary	GBIF: https://doi.org/10.15468/dl.zfaw79

Anopheles braziliensis	-73.64164	4.14812	Secondary	GBIF: https://doi.org/10.15468/dl.zfaw79
Anopheles braziliensis	-67.143	-9.702	Secondary	GBIF: https://doi.org/10.15468/dl.zfaw79
Anopheles braziliensis	-67.14	-9.7	Secondary	GBIF: https://doi.org/10.15468/dl.zfaw79
Anopheles braziliensis	-64.98	7.06	Secondary	GBIF: https://doi.org/10.15468/dl.zfaw79
Anopheles braziliensis	-64.97694	7.06194	Secondary	GBIF: https://doi.org/10.15468/dl.zfaw79
Anopheles braziliensis	-63.9039	-8.76194	Secondary	GBIF: https://doi.org/10.15468/dl.zfaw79
Anopheles braziliensis	-61.340676	10.313577	Secondary	GBIF: https://doi.org/10.15468/dl.zfaw79
Anopheles braziliensis	-61.332436	10.321684	Secondary	GBIF: https://doi.org/10.15468/dl.zfaw79
Anopheles braziliensis	-61.315956	10.313577	Secondary	GBIF: https://doi.org/10.15468/dl.zfaw79
Anopheles braziliensis	-61.307717	10.327088	Secondary	GBIF: https://doi.org/10.15468/dl.zfaw79
Anopheles braziliensis	-61.302224	10.321684	Secondary	GBIF: https://doi.org/10.15468/dl.zfaw79
Anopheles braziliensis	-61.288491	10.327088	Secondary	GBIF: https://doi.org/10.15468/dl.zfaw79
Anopheles braziliensis	-59.9004	3.14959	Secondary	GBIF: https://doi.org/10.15468/dl.zfaw79
Anopheles braziliensis	-59.8004	3.38292	Secondary	GBIF: https://doi.org/10.15468/dl.zfaw79
Anopheles braziliensis	-59.78373	3.08292	Secondary	GBIF: https://doi.org/10.15468/dl.zfaw79
Anopheles braziliensis	-59.073	-3.094	Secondary	GBIF: https://doi.org/10.15468/dl.zfaw79
Anopheles braziliensis	-59.07	-3.09	Secondary	GBIF: https://doi.org/10.15468/dl.zfaw79
Anopheles braziliensis	-58.30039	5.96627	Secondary	GBIF: https://doi.org/10.15468/dl.zfaw79
Anopheles braziliensis	-58.30039	5.9996	Secondary	GBIF: https://doi.org/10.15468/dl.zfaw79
Anopheles braziliensis	-57.80039	6.46627	Secondary	GBIF: https://doi.org/10.15468/dl.zfaw79
Anopheles braziliensis	-54.383048	4.27418	Secondary	GBIF: https://doi.org/10.15468/dl.zfaw79
Anopheles braziliensis	-54.198355	3.822518	Secondary	GBIF: https://doi.org/10.15468/dl.zfaw79
Anopheles braziliensis	-54.082518	5.409066	Secondary	GBIF: https://doi.org/10.15468/dl.zfaw79
Anopheles braziliensis	-54.07664	3.67331	Secondary	GBIF: https://doi.org/10.15468/dl.zfaw79
Anopheles braziliensis	-54.045577	5.480947	Secondary	GBIF: https://doi.org/10.15468/dl.zfaw79
Anopheles braziliensis	-54.04329	5.484058	Secondary	GBIF: https://doi.org/10.15468/dl.zfaw79
Anopheles braziliensis	-54.031415	3.643348	Secondary	GBIF: https://doi.org/10.15468/dl.zfaw79
Anopheles braziliensis	-54.028939	5.503342	Secondary	GBIF: https://doi.org/10.15468/dl.zfaw79
Anopheles braziliensis	-54.02004	5.50717	Secondary	GBIF: https://doi.org/10.15468/dl.zfaw79
Anopheles braziliensis	-53.909572	5.736301	Secondary	GBIF: https://doi.org/10.15468/dl.zfaw79
Anopheles braziliensis	-53.83532	3.32363	Secondary	GBIF: https://doi.org/10.15468/dl.zfaw79
Anopheles braziliensis	-53.778092	5.66898	Secondary	GBIF: https://doi.org/10.15468/dl.zfaw79
Anopheles braziliensis	-53.74599	5.638017	Secondary	GBIF: https://doi.org/10.15468/dl.zfaw79
Anopheles braziliensis	-53.674595	5.394268	Secondary	GBIF: https://doi.org/10.15468/dl.zfaw79
Anopheles braziliensis	-53.30643	5.48769	Secondary	GBIF: https://doi.org/10.15468/dl.zfaw79
Anopheles braziliensis	-53.288005	4.825571	Secondary	GBIF: https://doi.org/10.15468/dl.zfaw79
Anopheles braziliensis	-53.24036	3.91451	Secondary	GBIF: https://doi.org/10.15468/dl.zfaw79
Anopheles braziliensis	-53.23477	3.92404	Secondary	GBIF: https://doi.org/10.15468/dl.zfaw79
Anopheles braziliensis	-53.20944	3.623127	Secondary	GBIF: https://doi.org/10.15468/dl.zfaw79
Anopheles braziliensis	-53.205199	5.47958	Secondary	GBIF: https://doi.org/10.15468/dl.zfaw79
Anopheles braziliensis	-53.17007	5.52203	Secondary	GBIF: https://doi.org/10.15468/dl.zfaw79
Anopheles braziliensis	-53.132825	3.608848	Secondary	GBIF: https://doi.org/10.15468/dl.zfaw79

Anopheles braziliensis	-53.08936	5.42827	Secondary	GBIF: https://doi.org/10.15468/dl.zfaw79
Anopheles braziliensis	-53.083355	5.416688	Secondary	GBIF: https://doi.org/10.15468/dl.zfaw79
Anopheles braziliensis	-53.050025	5.062018	Secondary	GBIF: https://doi.org/10.15468/dl.zfaw79
Anopheles braziliensis	-53.03179	5.41668	Secondary	GBIF: https://doi.org/10.15468/dl.zfaw79
Anopheles braziliensis	-53.02915	5.01314	Secondary	GBIF: https://doi.org/10.15468/dl.zfaw79
Anopheles braziliensis	-52.99943	5.40638	Secondary	GBIF: https://doi.org/10.15468/dl.zfaw79
Anopheles braziliensis	-52.990525	5.386458	Secondary	GBIF: https://doi.org/10.15468/dl.zfaw79
Anopheles braziliensis	-52.983355	5.383348	Secondary	GBIF: https://doi.org/10.15468/dl.zfaw79
Anopheles braziliensis	-52.958187	5.377037	Secondary	GBIF: https://doi.org/10.15468/dl.zfaw79
Anopheles braziliensis	-52.957798	5.395708	Secondary	GBIF: https://doi.org/10.15468/dl.zfaw79
Anopheles braziliensis	-52.950025	5.316688	Secondary	GBIF: https://doi.org/10.15468/dl.zfaw79
Anopheles braziliensis	-52.900025	5.166684	Secondary	GBIF: https://doi.org/10.15468/dl.zfaw79
Anopheles braziliensis	-52.873971	2.247312	Secondary	GBIF: https://doi.org/10.15468/dl.zfaw79
Anopheles braziliensis	-52.873058	2.257084	Secondary	GBIF: https://doi.org/10.15468/dl.zfaw79
Anopheles braziliensis	-52.846531	5.347491	Secondary	GBIF: https://doi.org/10.15468/dl.zfaw79
Anopheles braziliensis	-52.834808	2.293751	Secondary	GBIF: https://doi.org/10.15468/dl.zfaw79
Anopheles braziliensis	-52.783359	4.833351	Secondary	GBIF: https://doi.org/10.15468/dl.zfaw79
Anopheles braziliensis	-52.7585	5.16303	Secondary	GBIF: https://doi.org/10.15468/dl.zfaw79
Anopheles braziliensis	-52.756745	5.153398	Secondary	GBIF: https://doi.org/10.15468/dl.zfaw79
Anopheles braziliensis	-52.738605	5.115338	Secondary	GBIF: https://doi.org/10.15468/dl.zfaw79
Anopheles braziliensis	-52.700025	5.016688	Secondary	GBIF: https://doi.org/10.15468/dl.zfaw79
Anopheles braziliensis	-52.66885	5.15907	Secondary	GBIF: https://doi.org/10.15468/dl.zfaw79
Anopheles braziliensis	-52.66862	5.16483	Secondary	GBIF: https://doi.org/10.15468/dl.zfaw79
Anopheles braziliensis	-52.650278	5.159722	Secondary	GBIF: https://doi.org/10.15468/dl.zfaw79
Anopheles braziliensis	-52.643374	5.166931	Secondary	GBIF: https://doi.org/10.15468/dl.zfaw79
Anopheles braziliensis	-52.63905	5.15894	Secondary	GBIF: https://doi.org/10.15468/dl.zfaw79
Anopheles braziliensis	-52.57423	5.03313	Secondary	GBIF: https://doi.org/10.15468/dl.zfaw79
Anopheles braziliensis	-52.52501	5.02128	Secondary	GBIF: https://doi.org/10.15468/dl.zfaw79
Anopheles braziliensis	-52.516695	4.916688	Secondary	GBIF: https://doi.org/10.15468/dl.zfaw79
Anopheles braziliensis	-52.515503	4.831218	Secondary	GBIF: https://doi.org/10.15468/dl.zfaw79
Anopheles braziliensis	-52.509685	4.832668	Secondary	GBIF: https://doi.org/10.15468/dl.zfaw79
Anopheles braziliensis	-52.508494	4.99794	Secondary	GBIF: https://doi.org/10.15468/dl.zfaw79
Anopheles braziliensis	-52.500025	4.950018	Secondary	GBIF: https://doi.org/10.15468/dl.zfaw79
Anopheles braziliensis	-52.492926	4.892476	Secondary	GBIF: https://doi.org/10.15468/dl.zfaw79
Anopheles braziliensis	-52.491355	4.561737	Secondary	GBIF: https://doi.org/10.15468/dl.zfaw79
Anopheles braziliensis	-52.489897	4.563033	Secondary	GBIF: https://doi.org/10.15468/dl.zfaw79
Anopheles braziliensis	-52.483355	4.666688	Secondary	GBIF: https://doi.org/10.15468/dl.zfaw79
Anopheles braziliensis	-52.475633	4.564139	Secondary	GBIF: https://doi.org/10.15468/dl.zfaw79
Anopheles braziliensis	-52.473837	5.013996	Secondary	GBIF: https://doi.org/10.15468/dl.zfaw79
Anopheles braziliensis	-52.473699	4.574534	Secondary	GBIF: https://doi.org/10.15468/dl.zfaw79
Anopheles braziliensis	-52.473185	4.57464	Secondary	GBIF: https://doi.org/10.15468/dl.zfaw79
Anopheles braziliensis	-52.473154	4.550587	Secondary	GBIF: https://doi.org/10.15468/dl.zfaw79

Anopheles braziliensis	-52.473105	4.552939	Secondary	GBIF: https://doi.org/10.15468/dl.zfaw79
Anopheles braziliensis	-52.47241	5.01171	Secondary	GBIF: https://doi.org/10.15468/dl.zfaw79
Anopheles braziliensis	-52.47175	4.573209	Secondary	GBIF: https://doi.org/10.15468/dl.zfaw79
Anopheles braziliensis	-52.47027	4.572008	Secondary	GBIF: https://doi.org/10.15468/dl.zfaw79
Anopheles braziliensis	-52.468501	4.571289	Secondary	GBIF: https://doi.org/10.15468/dl.zfaw79
Anopheles braziliensis	-52.46505	4.968779	Secondary	GBIF: https://doi.org/10.15468/dl.zfaw79
Anopheles braziliensis	-52.46407	4.563566	Secondary	GBIF: https://doi.org/10.15468/dl.zfaw79
Anopheles braziliensis	-52.464045	4.988308	Secondary	GBIF: https://doi.org/10.15468/dl.zfaw79
Anopheles braziliensis	-52.45662	4.792945	Secondary	GBIF: https://doi.org/10.15468/dl.zfaw79
Anopheles braziliensis	-52.443289	4.83079	Secondary	GBIF: https://doi.org/10.15468/dl.zfaw79
Anopheles braziliensis	-52.443015	4.976488	Secondary	GBIF: https://doi.org/10.15468/dl.zfaw79
Anopheles braziliensis	-52.434423	4.780484	Secondary	GBIF: https://doi.org/10.15468/dl.zfaw79
Anopheles braziliensis	-52.422625	4.782958	Secondary	GBIF: https://doi.org/10.15468/dl.zfaw79
Anopheles braziliensis	-52.413525	4.563701	Secondary	GBIF: https://doi.org/10.15468/dl.zfaw79
Anopheles braziliensis	-52.406535	4.910838	Secondary	GBIF: https://doi.org/10.15468/dl.zfaw79
Anopheles braziliensis	-52.40423	4.94154	Secondary	GBIF: https://doi.org/10.15468/dl.zfaw79
Anopheles braziliensis	-52.40147	4.71699	Secondary	GBIF: https://doi.org/10.15468/dl.zfaw79
Anopheles braziliensis	-52.383355	4.883348	Secondary	GBIF: https://doi.org/10.15468/dl.zfaw79
Anopheles braziliensis	-52.361061	4.81862	Secondary	GBIF: https://doi.org/10.15468/dl.zfaw79
Anopheles braziliensis	-52.360475	4.819828	Secondary	GBIF: https://doi.org/10.15468/dl.zfaw79
Anopheles braziliensis	-52.35913	4.90255	Secondary	GBIF: https://doi.org/10.15468/dl.zfaw79
Anopheles braziliensis	-52.357135	4.886868	Secondary	GBIF: https://doi.org/10.15468/dl.zfaw79
Anopheles braziliensis	-52.351905	4.788018	Secondary	GBIF: https://doi.org/10.15468/dl.zfaw79
Anopheles braziliensis	-52.34913	4.83642	Secondary	GBIF: https://doi.org/10.15468/dl.zfaw79
Anopheles braziliensis	-52.348155	4.886318	Secondary	GBIF: https://doi.org/10.15468/dl.zfaw79
Anopheles braziliensis	-52.346175	4.889698	Secondary	GBIF: https://doi.org/10.15468/dl.zfaw79
Anopheles braziliensis	-52.34352	4.77908	Secondary	GBIF: https://doi.org/10.15468/dl.zfaw79
Anopheles braziliensis	-52.3435	4.88147	Secondary	GBIF: https://doi.org/10.15468/dl.zfaw79
Anopheles braziliensis	-52.34241	4.89956	Secondary	GBIF: https://doi.org/10.15468/dl.zfaw79
Anopheles braziliensis	-52.341575	4.823418	Secondary	GBIF: https://doi.org/10.15468/dl.zfaw79
Anopheles braziliensis	-52.34122	4.89382	Secondary	GBIF: https://doi.org/10.15468/dl.zfaw79
Anopheles braziliensis	-52.34003	4.81928	Secondary	GBIF: https://doi.org/10.15468/dl.zfaw79
Anopheles braziliensis	-52.33768	4.85645	Secondary	GBIF: https://doi.org/10.15468/dl.zfaw79
Anopheles braziliensis	-52.337483	4.875631	Secondary	GBIF: https://doi.org/10.15468/dl.zfaw79
Anopheles braziliensis	-52.33696	4.83273	Secondary	GBIF: https://doi.org/10.15468/dl.zfaw79
Anopheles braziliensis	-52.334255	4.788748	Secondary	GBIF: https://doi.org/10.15468/dl.zfaw79
Anopheles braziliensis	-52.333705	4.852888	Secondary	GBIF: https://doi.org/10.15468/dl.zfaw79
Anopheles braziliensis	-52.333605	3.167747	Secondary	GBIF: https://doi.org/10.15468/dl.zfaw79
Anopheles braziliensis	-52.333355	4.850018	Secondary	GBIF: https://doi.org/10.15468/dl.zfaw79
Anopheles braziliensis	-52.333355	4.733348	Secondary	GBIF: https://doi.org/10.15468/dl.zfaw79
Anopheles braziliensis	-52.333115	4.816198	Secondary	GBIF: https://doi.org/10.15468/dl.zfaw79
Anopheles braziliensis	-52.332371	4.767255	Secondary	GBIF: https://doi.org/10.15468/dl.zfaw79

Anopheles braziliensis	-52.33163	4.62747	Secondary	GBIF: https://doi.org/10.15468/dl.zfaw79
Anopheles braziliensis	-52.330987	4.850608	Secondary	GBIF: https://doi.org/10.15468/dl.zfaw79
Anopheles braziliensis	-52.326965	4.883998	Secondary	GBIF: https://doi.org/10.15468/dl.zfaw79
Anopheles braziliensis	-52.32628	4.727944	Secondary	GBIF: https://doi.org/10.15468/dl.zfaw79
Anopheles braziliensis	-52.32267	4.73133	Secondary	GBIF: https://doi.org/10.15468/dl.zfaw79
Anopheles braziliensis	-52.32266	4.84545	Secondary	GBIF: https://doi.org/10.15468/dl.zfaw79
Anopheles braziliensis	-52.32059	4.73866	Secondary	GBIF: https://doi.org/10.15468/dl.zfaw79
Anopheles braziliensis	-52.320395	4.903308	Secondary	GBIF: https://doi.org/10.15468/dl.zfaw79
Anopheles braziliensis	-52.319815	4.849048	Secondary	GBIF: https://doi.org/10.15468/dl.zfaw79
Anopheles braziliensis	-52.3192	4.88391	Secondary	GBIF: https://doi.org/10.15468/dl.zfaw79
Anopheles braziliensis	-52.318795	3.170558	Secondary	GBIF: https://doi.org/10.15468/dl.zfaw79
Anopheles braziliensis	-52.316695	4.516688	Secondary	GBIF: https://doi.org/10.15468/dl.zfaw79
Anopheles braziliensis	-52.312455	4.927798	Secondary	GBIF: https://doi.org/10.15468/dl.zfaw79
Anopheles braziliensis	-52.311136	4.925018	Secondary	GBIF: https://doi.org/10.15468/dl.zfaw79
Anopheles braziliensis	-52.30826	4.83195	Secondary	GBIF: https://doi.org/10.15468/dl.zfaw79
Anopheles braziliensis	-52.307335	4.833438	Secondary	GBIF: https://doi.org/10.15468/dl.zfaw79
Anopheles braziliensis	-52.307198	4.627222	Secondary	GBIF: https://doi.org/10.15468/dl.zfaw79
Anopheles braziliensis	-52.306695	4.940978	Secondary	GBIF: https://doi.org/10.15468/dl.zfaw79
Anopheles braziliensis	-52.303835	4.889818	Secondary	GBIF: https://doi.org/10.15468/dl.zfaw79
Anopheles braziliensis	-52.291065	4.890268	Secondary	GBIF: https://doi.org/10.15468/dl.zfaw79
Anopheles braziliensis	-52.29017	4.92854	Secondary	GBIF: https://doi.org/10.15468/dl.zfaw79
Anopheles braziliensis	-52.28659	4.89308	Secondary	GBIF: https://doi.org/10.15468/dl.zfaw79
Anopheles braziliensis	-52.28402	4.91235	Secondary	GBIF: https://doi.org/10.15468/dl.zfaw79
Anopheles braziliensis	-52.279846	4.717854	Secondary	GBIF: https://doi.org/10.15468/dl.zfaw79
Anopheles braziliensis	-52.279143	4.718157	Secondary	GBIF: https://doi.org/10.15468/dl.zfaw79
Anopheles braziliensis	-52.276221	4.886853	Secondary	GBIF: https://doi.org/10.15468/dl.zfaw79
Anopheles braziliensis	-52.27475	4.85384	Secondary	GBIF: https://doi.org/10.15468/dl.zfaw79
Anopheles braziliensis	-52.272123	4.912182	Secondary	GBIF: https://doi.org/10.15468/dl.zfaw79
Anopheles braziliensis	-52.259305	4.911678	Secondary	GBIF: https://doi.org/10.15468/dl.zfaw79
Anopheles braziliensis	-52.256515	4.857598	Secondary	GBIF: https://doi.org/10.15468/dl.zfaw79
Anopheles braziliensis	-52.251475	4.881148	Secondary	GBIF: https://doi.org/10.15468/dl.zfaw79
Anopheles braziliensis	-52.153995	4.315458	Secondary	GBIF: https://doi.org/10.15468/dl.zfaw79
Anopheles braziliensis	-52.129162	4.314002	Secondary	GBIF: https://doi.org/10.15468/dl.zfaw79
Anopheles braziliensis	-52.037094	4.486403	Secondary	GBIF: https://doi.org/10.15468/dl.zfaw79
Anopheles braziliensis	-51.886575	3.802423	Secondary	GBIF: https://doi.org/10.15468/dl.zfaw79
Anopheles braziliensis	-51.88333	3.8	Secondary	GBIF: https://doi.org/10.15468/dl.zfaw79
Anopheles braziliensis	-51.855225	3.870678	Secondary	GBIF: https://doi.org/10.15468/dl.zfaw79
Anopheles braziliensis	-51.838914	3.833407	Secondary	GBIF: https://doi.org/10.15468/dl.zfaw79
Anopheles braziliensis	-51.838247	3.833546	Secondary	GBIF: https://doi.org/10.15468/dl.zfaw79
Anopheles braziliensis	-51.838081	3.834323	Secondary	GBIF: https://doi.org/10.15468/dl.zfaw79
Anopheles braziliensis	-51.815192	3.875351	Secondary	GBIF: https://doi.org/10.15468/dl.zfaw79
Anopheles braziliensis	-51.808359	3.891601	Secondary	GBIF: https://doi.org/10.15468/dl.zfaw79

Anopheles braziliensis	-51.806695	3.888638	Secondary	GBIF: https://doi.org/10.15468/dl.zfaw79
Anopheles braziliensis	-51.805914	3.898851	Secondary	GBIF: https://doi.org/10.15468/dl.zfaw79
Anopheles braziliensis	-51.802995	3.889048	Secondary	GBIF: https://doi.org/10.15468/dl.zfaw79
Anopheles braziliensis	-51.801756	3.895797	Secondary	GBIF: https://doi.org/10.15468/dl.zfaw79
Anopheles braziliensis	-51.801414	3.898407	Secondary	GBIF: https://doi.org/10.15468/dl.zfaw79
Anopheles braziliensis	-51.801158	3.888398	Secondary	GBIF: https://doi.org/10.15468/dl.zfaw79
Anopheles braziliensis	-51.800609	3.896323	Secondary	GBIF: https://doi.org/10.15468/dl.zfaw79
Anopheles braziliensis	-51.798581	3.895712	Secondary	GBIF: https://doi.org/10.15468/dl.zfaw79
Anopheles braziliensis	-51.795969	3.878796	Secondary	GBIF: https://doi.org/10.15468/dl.zfaw79
Anopheles braziliensis	-50.97217	0.21314	Secondary	GBIF: https://doi.org/10.15468/dl.zfaw79
Anopheles braziliensis	-50.97	0.21	Secondary	GBIF: https://doi.org/10.15468/dl.zfaw79
Anopheles braziliensis	-48.53389	-22.17611	Secondary	GBIF: https://doi.org/10.15468/dl.zfaw79
Anopheles braziliensis	-48.43	-22.09	Secondary	GBIF: https://doi.org/10.15468/dl.zfaw79
Anopheles braziliensis	-44.566668	-17.900001	Secondary	GBIF: https://doi.org/10.15468/dl.zfaw79
Anopheles brevipalpis	105.164167	-2.064444	Unclassified	GBIF: https://doi.org/10.15468/dl.zfaw79
Anopheles brohieri	6.73259	7.80037	Unclassified	GBIF: https://doi.org/10.15468/dl.zfaw79
Anopheles brohieri	7.36594	6.96712	Unclassified	GBIF: https://doi.org/10.15468/dl.zfaw79
Anopheles brohieri	7.44927	10.50013	Unclassified	GBIF: https://doi.org/10.15468/dl.zfaw79
Anopheles brohieri	13.41	-1.64	Unclassified	GBIF: https://doi.org/10.15468/dl.zfaw79
Anopheles brunnipes	6.73259	7.80037	Unclassified	GBIF: https://doi.org/10.15468/dl.zfaw79
Anopheles brunnipes	7.44927	10.50013	Unclassified	GBIF: https://doi.org/10.15468/dl.zfaw79
Anopheles brunnipes	7.4826	10.63345	Unclassified	GBIF: https://doi.org/10.15468/dl.zfaw79
Anopheles brunnipes	7.54928	6.4505	Unclassified	GBIF: https://doi.org/10.15468/dl.zfaw79
Anopheles brunnipes	11.6	-0.2	Unclassified	GBIF: https://doi.org/10.15468/dl.zfaw79
Anopheles brunnipes	38.155594	-17.27115	Unclassified	GBIF: https://doi.org/10.15468/dl.zfaw79
Anopheles bulkleyi	101.501371	13.239733	Unclassified	GBIF: https://doi.org/10.15468/dl.zfaw79
Anopheles bulkleyi	101.904816	6.31097	Unclassified	GBIF: https://doi.org/10.15468/dl.zfaw79
Anopheles bulkleyi	104.22271	14.615236	Unclassified	GBIF: https://doi.org/10.15468/dl.zfaw79
Anopheles buxtoni	10.39932	6.16719	Unclassified	GBIF: https://doi.org/10.15468/dl.zfaw79
Anopheles bwambae	30.16	0.83	Unclassified	GBIF: https://doi.org/10.15468/dl.zfaw79
Anopheles bwambae	30.18333	0.8	Unclassified	GBIF: https://doi.org/10.15468/dl.zfaw79
Anopheles calderoni	-79.683	-2.1	Unclassified	GBIF: https://doi.org/10.15468/dl.zfaw79
Anopheles calderoni	-79.68	-2.1	Unclassified	GBIF: https://doi.org/10.15468/dl.zfaw79
Anopheles calderoni	-79.405	-0.030833	Unclassified	GBIF: https://doi.org/10.15468/dl.zfaw79
Anopheles calderoni	-79.396389	-0.0175	Unclassified	GBIF: https://doi.org/10.15468/dl.zfaw79
Anopheles calderoni	-79.276389	-0.085	Unclassified	GBIF: https://doi.org/10.15468/dl.zfaw79
Anopheles calderoni	-79.276111	-0.088056	Unclassified	GBIF: https://doi.org/10.15468/dl.zfaw79
Anopheles calderoni	-79.276111	-0.086111	Unclassified	GBIF: https://doi.org/10.15468/dl.zfaw79
Anopheles calderoni	-79.2725	-0.086111	Unclassified	GBIF: https://doi.org/10.15468/dl.zfaw79
Anopheles calderoni	-79.080833	-0.271667	Unclassified	GBIF: https://doi.org/10.15468/dl.zfaw79
Anopheles calderoni	-79.058056	-0.296389	Unclassified	GBIF: https://doi.org/10.15468/dl.zfaw79
Anopheles calderoni	-78.7333	1.6167	Unclassified	GBIF: https://doi.org/10.15468/dl.zfaw79

Anopheles calderoni	-78.73	1.62	Unclassified	GBIF: https://doi.org/10.15468/dl.zfaw79
Anopheles calderoni	-76.47	4.14	Unclassified	GBIF: https://doi.org/10.15468/dl.zfaw79
Anopheles calderoni	-76.35	3.88	Unclassified	GBIF: https://doi.org/10.15468/dl.zfaw79
Anopheles calderoni	-76.3481	3.8756	Unclassified	GBIF: https://doi.org/10.15468/dl.zfaw79
Anopheles calderoni	-76.348	3.876	Unclassified	GBIF: https://doi.org/10.15468/dl.zfaw79
Anopheles calderoni	-76.34	3.88	Unclassified	GBIF: https://doi.org/10.15468/dl.zfaw79
Anopheles calderoni	-76.28	4.14	Unclassified	GBIF: https://doi.org/10.15468/dl.zfaw79
Anopheles calderoni	-76.279	4.1434	Unclassified	GBIF: https://doi.org/10.15468/dl.zfaw79
Anopheles calderoni	-75.9028	4.9433	Unclassified	GBIF: https://doi.org/10.15468/dl.zfaw79
Anopheles calderoni	-75.9	4.94	Unclassified	GBIF: https://doi.org/10.15468/dl.zfaw79
Anopheles calderoni	-75.86	4.88	Unclassified	GBIF: https://doi.org/10.15468/dl.zfaw79
Anopheles calderoni	-75.8577	4.878	Unclassified	GBIF: https://doi.org/10.15468/dl.zfaw79
Anopheles calderoni	-75.85	-1	Unclassified	GBIF: https://doi.org/10.15468/dl.zfaw79
Anopheles calderoni	-75.85	4.88	Unclassified	GBIF: https://doi.org/10.15468/dl.zfaw79
Anopheles calderoni	-72.64451	4.187754	Unclassified	GBIF: https://doi.org/10.15468/dl.zfaw79
Anopheles campestris	98.065354	17.903965	Unclassified	GBIF: https://doi.org/10.15468/dl.zfaw79
Anopheles campestris	98.357238	18.510428	Unclassified	GBIF: https://doi.org/10.15468/dl.zfaw79
Anopheles campestris	98.38286	17.076825	Unclassified	GBIF: https://doi.org/10.15468/dl.zfaw79
Anopheles campestris	98.393148	8.248306	Unclassified	GBIF: https://doi.org/10.15468/dl.zfaw79
Anopheles campestris	98.411169	8.927604	Unclassified	GBIF: https://doi.org/10.15468/dl.zfaw79
Anopheles campestris	98.532184	14.559572	Unclassified	GBIF: https://doi.org/10.15468/dl.zfaw79
Anopheles campestris	98.557819	14.670573	Unclassified	GBIF: https://doi.org/10.15468/dl.zfaw79
Anopheles campestris	98.564605	14.636853	Unclassified	GBIF: https://doi.org/10.15468/dl.zfaw79
Anopheles campestris	98.615629	14.940706	Unclassified	GBIF: https://doi.org/10.15468/dl.zfaw79
Anopheles campestris	98.620219	14.974166	Unclassified	GBIF: https://doi.org/10.15468/dl.zfaw79
Anopheles campestris	98.655633	14.929016	Unclassified	GBIF: https://doi.org/10.15468/dl.zfaw79
Anopheles campestris	98.662471	14.716557	Unclassified	GBIF: https://doi.org/10.15468/dl.zfaw79
Anopheles campestris	98.668136	14.655988	Unclassified	GBIF: https://doi.org/10.15468/dl.zfaw79
Anopheles campestris	98.670826	9.773319	Unclassified	GBIF: https://doi.org/10.15468/dl.zfaw79
Anopheles campestris	98.682859	9.756881	Unclassified	GBIF: https://doi.org/10.15468/dl.zfaw79
Anopheles campestris	98.69414	9.778821	Unclassified	GBIF: https://doi.org/10.15468/dl.zfaw79
Anopheles campestris	98.701922	9.761124	Unclassified	GBIF: https://doi.org/10.15468/dl.zfaw79
Anopheles campestris	98.707889	14.56204	Unclassified	GBIF: https://doi.org/10.15468/dl.zfaw79
Anopheles campestris	98.71649	14.608132	Unclassified	GBIF: https://doi.org/10.15468/dl.zfaw79
Anopheles campestris	98.719946	14.674302	Unclassified	GBIF: https://doi.org/10.15468/dl.zfaw79
Anopheles campestris	98.720925	14.670656	Unclassified	GBIF: https://doi.org/10.15468/dl.zfaw79
Anopheles campestris	98.722985	14.611756	Unclassified	GBIF: https://doi.org/10.15468/dl.zfaw79
Anopheles campestris	98.723911	14.613566	Unclassified	GBIF: https://doi.org/10.15468/dl.zfaw79
Anopheles campestris	98.73197	9.747006	Unclassified	GBIF: https://doi.org/10.15468/dl.zfaw79
Anopheles campestris	98.739665	14.639804	Unclassified	GBIF: https://doi.org/10.15468/dl.zfaw79
Anopheles campestris	98.745317	14.570191	Unclassified	GBIF: https://doi.org/10.15468/dl.zfaw79
Anopheles campestris	98.754286	14.714072	Unclassified	GBIF: https://doi.org/10.15468/dl.zfaw79

Anopheles campestris	98.789103	9.74699	Unclassified	GBIF: https://doi.org/10.15468/dl.zfaw79
Anopheles campestris	98.795965	8.284143	Unclassified	GBIF: https://doi.org/10.15468/dl.zfaw79
Anopheles campestris	98.827227	9.763324	Unclassified	GBIF: https://doi.org/10.15468/dl.zfaw79
Anopheles campestris	98.83079	9.825765	Unclassified	GBIF: https://doi.org/10.15468/dl.zfaw79
Anopheles campestris	98.832284	9.840487	Unclassified	GBIF: https://doi.org/10.15468/dl.zfaw79
Anopheles campestris	98.838156	14.19671	Unclassified	GBIF: https://doi.org/10.15468/dl.zfaw79
Anopheles campestris	98.839905	9.867426	Unclassified	GBIF: https://doi.org/10.15468/dl.zfaw79
Anopheles campestris	98.889634	10.123784	Unclassified	GBIF: https://doi.org/10.15468/dl.zfaw79
Anopheles campestris	98.90558	18.91308	Unclassified	GBIF: https://doi.org/10.15468/dl.zfaw79
Anopheles campestris	98.908575	10.141897	Unclassified	GBIF: https://doi.org/10.15468/dl.zfaw79
Anopheles campestris	98.923176	14.364739	Unclassified	GBIF: https://doi.org/10.15468/dl.zfaw79
Anopheles campestris	98.934357	10.207495	Unclassified	GBIF: https://doi.org/10.15468/dl.zfaw79
Anopheles campestris	98.93458	10.489564	Unclassified	GBIF: https://doi.org/10.15468/dl.zfaw79
Anopheles campestris	98.938173	14.215543	Unclassified	GBIF: https://doi.org/10.15468/dl.zfaw79
Anopheles campestris	98.945507	10.328036	Unclassified	GBIF: https://doi.org/10.15468/dl.zfaw79
Anopheles campestris	98.94834	18.81187	Unclassified	GBIF: https://doi.org/10.15468/dl.zfaw79
Anopheles campestris	98.952698	10.263799	Unclassified	GBIF: https://doi.org/10.15468/dl.zfaw79
Anopheles campestris	98.958039	10.515357	Unclassified	GBIF: https://doi.org/10.15468/dl.zfaw79
Anopheles campestris	98.975234	10.725473	Unclassified	GBIF: https://doi.org/10.15468/dl.zfaw79
Anopheles campestris	98.97972	10.213745	Unclassified	GBIF: https://doi.org/10.15468/dl.zfaw79
Anopheles campestris	98.9806	14.517659	Unclassified	GBIF: https://doi.org/10.15468/dl.zfaw79
Anopheles campestris	98.98494	10.35961	Unclassified	GBIF: https://doi.org/10.15468/dl.zfaw79
Anopheles campestris	98.988401	14.335196	Unclassified	GBIF: https://doi.org/10.15468/dl.zfaw79
Anopheles campestris	98.988985	14.336084	Unclassified	GBIF: https://doi.org/10.15468/dl.zfaw79
Anopheles campestris	98.991845	10.762198	Unclassified	GBIF: https://doi.org/10.15468/dl.zfaw79
Anopheles campestris	98.992975	10.261541	Unclassified	GBIF: https://doi.org/10.15468/dl.zfaw79
Anopheles campestris	98.995442	10.389929	Unclassified	GBIF: https://doi.org/10.15468/dl.zfaw79
Anopheles campestris	98.998904	14.49628	Unclassified	GBIF: https://doi.org/10.15468/dl.zfaw79
Anopheles campestris	99.005419	10.781285	Unclassified	GBIF: https://doi.org/10.15468/dl.zfaw79
Anopheles campestris	99.007111	10.710313	Unclassified	GBIF: https://doi.org/10.15468/dl.zfaw79
Anopheles campestris	99.014309	10.509098	Unclassified	GBIF: https://doi.org/10.15468/dl.zfaw79
Anopheles campestris	99.01705	10.493723	Unclassified	GBIF: https://doi.org/10.15468/dl.zfaw79
Anopheles campestris	99.034395	10.322782	Unclassified	GBIF: https://doi.org/10.15468/dl.zfaw79
Anopheles campestris	99.03499	14.453069	Unclassified	GBIF: https://doi.org/10.15468/dl.zfaw79
Anopheles campestris	99.045396	10.621095	Unclassified	GBIF: https://doi.org/10.15468/dl.zfaw79
Anopheles campestris	99.050601	10.860462	Unclassified	GBIF: https://doi.org/10.15468/dl.zfaw79
Anopheles campestris	99.056022	10.426884	Unclassified	GBIF: https://doi.org/10.15468/dl.zfaw79
Anopheles campestris	99.058182	10.536226	Unclassified	GBIF: https://doi.org/10.15468/dl.zfaw79
Anopheles campestris	99.06551	10.609484	Unclassified	GBIF: https://doi.org/10.15468/dl.zfaw79
Anopheles campestris	99.083191	14.458198	Unclassified	GBIF: https://doi.org/10.15468/dl.zfaw79
Anopheles campestris	99.088655	14.194176	Unclassified	GBIF: https://doi.org/10.15468/dl.zfaw79
Anopheles campestris	99.093247	10.075561	Unclassified	GBIF: https://doi.org/10.15468/dl.zfaw79

Anopheles campestris	99.097464	10.473811	Unclassified	GBIF: https://doi.org/10.15468/dl.zfaw79
Anopheles campestris	99.100668	14.114603	Unclassified	GBIF: https://doi.org/10.15468/dl.zfaw79
Anopheles campestris	99.123269	14.052543	Unclassified	GBIF: https://doi.org/10.15468/dl.zfaw79
Anopheles campestris	99.128413	14.026883	Unclassified	GBIF: https://doi.org/10.15468/dl.zfaw79
Anopheles campestris	99.129484	10.560625	Unclassified	GBIF: https://doi.org/10.15468/dl.zfaw79
Anopheles campestris	99.129673	14.609172	Unclassified	GBIF: https://doi.org/10.15468/dl.zfaw79
Anopheles campestris	99.130313	10.358031	Unclassified	GBIF: https://doi.org/10.15468/dl.zfaw79
Anopheles campestris	99.149627	13.893048	Unclassified	GBIF: https://doi.org/10.15468/dl.zfaw79
Anopheles campestris	99.151931	13.89761	Unclassified	GBIF: https://doi.org/10.15468/dl.zfaw79
Anopheles campestris	99.173763	13.987069	Unclassified	GBIF: https://doi.org/10.15468/dl.zfaw79
Anopheles campestris	99.180595	13.914555	Unclassified	GBIF: https://doi.org/10.15468/dl.zfaw79
Anopheles campestris	99.184979	14.11817	Unclassified	GBIF: https://doi.org/10.15468/dl.zfaw79
Anopheles campestris	99.187126	14.026089	Unclassified	GBIF: https://doi.org/10.15468/dl.zfaw79
Anopheles campestris	99.201877	10.857277	Unclassified	GBIF: https://doi.org/10.15468/dl.zfaw79
Anopheles campestris	99.208309	14.3017	Unclassified	GBIF: https://doi.org/10.15468/dl.zfaw79
Anopheles campestris	99.211657	13.911086	Unclassified	GBIF: https://doi.org/10.15468/dl.zfaw79
Anopheles campestris	99.211781	14.046716	Unclassified	GBIF: https://doi.org/10.15468/dl.zfaw79
Anopheles campestris	99.228932	13.888759	Unclassified	GBIF: https://doi.org/10.15468/dl.zfaw79
Anopheles campestris	99.240469	14.02318	Unclassified	GBIF: https://doi.org/10.15468/dl.zfaw79
Anopheles campestris	99.242146	13.855902	Unclassified	GBIF: https://doi.org/10.15468/dl.zfaw79
Anopheles campestris	99.254742	13.458063	Unclassified	GBIF: https://doi.org/10.15468/dl.zfaw79
Anopheles campestris	99.263446	13.869443	Unclassified	GBIF: https://doi.org/10.15468/dl.zfaw79
Anopheles campestris	99.268026	13.827844	Unclassified	GBIF: https://doi.org/10.15468/dl.zfaw79
Anopheles campestris	99.268212	13.988792	Unclassified	GBIF: https://doi.org/10.15468/dl.zfaw79
Anopheles campestris	99.274324	13.37981	Unclassified	GBIF: https://doi.org/10.15468/dl.zfaw79
Anopheles campestris	99.276019	10.901084	Unclassified	GBIF: https://doi.org/10.15468/dl.zfaw79
Anopheles campestris	99.280002	13.783525	Unclassified	GBIF: https://doi.org/10.15468/dl.zfaw79
Anopheles campestris	99.28559	10.923141	Unclassified	GBIF: https://doi.org/10.15468/dl.zfaw79
Anopheles campestris	99.296317	10.939305	Unclassified	GBIF: https://doi.org/10.15468/dl.zfaw79
Anopheles campestris	99.305642	13.598615	Unclassified	GBIF: https://doi.org/10.15468/dl.zfaw79
Anopheles campestris	99.305819	16.33037	Unclassified	GBIF: https://doi.org/10.15468/dl.zfaw79
Anopheles campestris	99.317018	13.790713	Unclassified	GBIF: https://doi.org/10.15468/dl.zfaw79
Anopheles campestris	99.319101	14.225909	Unclassified	GBIF: https://doi.org/10.15468/dl.zfaw79
Anopheles campestris	99.321218	14.763239	Unclassified	GBIF: https://doi.org/10.15468/dl.zfaw79
Anopheles campestris	99.322804	14.758513	Unclassified	GBIF: https://doi.org/10.15468/dl.zfaw79
Anopheles campestris	99.3317	14.793109	Unclassified	GBIF: https://doi.org/10.15468/dl.zfaw79
Anopheles campestris	99.333372	14.021586	Unclassified	GBIF: https://doi.org/10.15468/dl.zfaw79
Anopheles campestris	99.338339	13.039994	Unclassified	GBIF: https://doi.org/10.15468/dl.zfaw79
Anopheles campestris	99.357918	13.701094	Unclassified	GBIF: https://doi.org/10.15468/dl.zfaw79
Anopheles campestris	99.358923	14.772592	Unclassified	GBIF: https://doi.org/10.15468/dl.zfaw79
Anopheles campestris	99.361265	14.392985	Unclassified	GBIF: https://doi.org/10.15468/dl.zfaw79
Anopheles campestris	99.363747	14.632549	Unclassified	GBIF: https://doi.org/10.15468/dl.zfaw79

Anopheles campestris	99.37404	13.475408	Unclassified	GBIF: https://doi.org/10.15468/dl.zfaw79
Anopheles campestris	99.377469	14.5429	Unclassified	GBIF: https://doi.org/10.15468/dl.zfaw79
Anopheles campestris	99.378541	14.028376	Unclassified	GBIF: https://doi.org/10.15468/dl.zfaw79
Anopheles campestris	99.379248	14.49769	Unclassified	GBIF: https://doi.org/10.15468/dl.zfaw79
Anopheles campestris	99.379358	13.671355	Unclassified	GBIF: https://doi.org/10.15468/dl.zfaw79
Anopheles campestris	99.382518	14.75967	Unclassified	GBIF: https://doi.org/10.15468/dl.zfaw79
Anopheles campestris	99.383244	13.427679	Unclassified	GBIF: https://doi.org/10.15468/dl.zfaw79
Anopheles campestris	99.38563	14.664028	Unclassified	GBIF: https://doi.org/10.15468/dl.zfaw79
Anopheles campestris	99.386491	13.695979	Unclassified	GBIF: https://doi.org/10.15468/dl.zfaw79
Anopheles campestris	99.399574	13.461019	Unclassified	GBIF: https://doi.org/10.15468/dl.zfaw79
Anopheles campestris	99.406118	11.011515	Unclassified	GBIF: https://doi.org/10.15468/dl.zfaw79
Anopheles campestris	99.408709	13.848447	Unclassified	GBIF: https://doi.org/10.15468/dl.zfaw79
Anopheles campestris	99.409712	14.634444	Unclassified	GBIF: https://doi.org/10.15468/dl.zfaw79
Anopheles campestris	99.413953	13.396582	Unclassified	GBIF: https://doi.org/10.15468/dl.zfaw79
Anopheles campestris	99.415127	13.35709	Unclassified	GBIF: https://doi.org/10.15468/dl.zfaw79
Anopheles campestris	99.419449	13.330236	Unclassified	GBIF: https://doi.org/10.15468/dl.zfaw79
Anopheles campestris	99.42	13.29	Unclassified	GBIF: https://doi.org/10.15468/dl.zfaw79
Anopheles campestris	99.431421	13.30249	Unclassified	GBIF: https://doi.org/10.15468/dl.zfaw79
Anopheles campestris	99.469207	7.674829	Unclassified	GBIF: https://doi.org/10.15468/dl.zfaw79
Anopheles campestris	99.496263	11.223328	Unclassified	GBIF: https://doi.org/10.15468/dl.zfaw79
Anopheles campestris	99.505395	13.331615	Unclassified	GBIF: https://doi.org/10.15468/dl.zfaw79
Anopheles campestris	99.57656	12.275771	Unclassified	GBIF: https://doi.org/10.15468/dl.zfaw79
Anopheles campestris	99.582399	13.355311	Unclassified	GBIF: https://doi.org/10.15468/dl.zfaw79
Anopheles campestris	99.587705	18.702434	Unclassified	GBIF: https://doi.org/10.15468/dl.zfaw79
Anopheles campestris	99.594187	13.266671	Unclassified	GBIF: https://doi.org/10.15468/dl.zfaw79
Anopheles campestris	99.63	13.91	Unclassified	GBIF: https://doi.org/10.15468/dl.zfaw79
Anopheles campestris	99.632275	12.7236	Unclassified	GBIF: https://doi.org/10.15468/dl.zfaw79
Anopheles campestris	99.64971	13.232429	Unclassified	GBIF: https://doi.org/10.15468/dl.zfaw79
Anopheles campestris	99.652327	13.31654	Unclassified	GBIF: https://doi.org/10.15468/dl.zfaw79
Anopheles campestris	99.653077	12.777445	Unclassified	GBIF: https://doi.org/10.15468/dl.zfaw79
Anopheles campestris	99.655115	13.267426	Unclassified	GBIF: https://doi.org/10.15468/dl.zfaw79
Anopheles campestris	99.687316	13.229365	Unclassified	GBIF: https://doi.org/10.15468/dl.zfaw79
Anopheles campestris	99.690027	8.027579	Unclassified	GBIF: https://doi.org/10.15468/dl.zfaw79
Anopheles campestris	99.943064	15.46883	Unclassified	GBIF: https://doi.org/10.15468/dl.zfaw79
Anopheles campestris	99.94396	18.82756	Unclassified	GBIF: https://doi.org/10.15468/dl.zfaw79
Anopheles campestris	99.94491	18.82756	Unclassified	GBIF: https://doi.org/10.15468/dl.zfaw79
Anopheles campestris	99.95278	18.70913	Unclassified	GBIF: https://doi.org/10.15468/dl.zfaw79
Anopheles campestris	99.95373	18.70823	Unclassified	GBIF: https://doi.org/10.15468/dl.zfaw79
Anopheles campestris	99.95658	18.70911	Unclassified	GBIF: https://doi.org/10.15468/dl.zfaw79
Anopheles campestris	99.99189	18.74779	Unclassified	GBIF: https://doi.org/10.15468/dl.zfaw79
Anopheles campestris	99.9938	18.74959	Unclassified	GBIF: https://doi.org/10.15468/dl.zfaw79
Anopheles campestris	100.53426	14.06371	Unclassified	GBIF: https://doi.org/10.15468/dl.zfaw79

Anopheles campestris	100.53429	14.06823	Unclassified	GBIF: https://doi.org/10.15468/dl.zfaw79
Anopheles campestris	100.54337	14.04105	Unclassified	GBIF: https://doi.org/10.15468/dl.zfaw79
Anopheles campestris	100.5434	14.04557	Unclassified	GBIF: https://doi.org/10.15468/dl.zfaw79
Anopheles campestris	100.54352	14.06365	Unclassified	GBIF: https://doi.org/10.15468/dl.zfaw79
Anopheles campestris	100.54608	13.75178	Unclassified	GBIF: https://doi.org/10.15468/dl.zfaw79
Anopheles campestris	100.54812	14.0591	Unclassified	GBIF: https://doi.org/10.15468/dl.zfaw79
Anopheles campestris	100.55725	14.04096	Unclassified	GBIF: https://doi.org/10.15468/dl.zfaw79
Anopheles campestris	100.56651	14.0409	Unclassified	GBIF: https://doi.org/10.15468/dl.zfaw79
Anopheles campestris	100.58933	14.25949	Unclassified	GBIF: https://doi.org/10.15468/dl.zfaw79
Anopheles campestris	100.63	18.53	Unclassified	GBIF: https://doi.org/10.15468/dl.zfaw79
Anopheles campestris	100.72826	14.61101	Unclassified	GBIF: https://doi.org/10.15468/dl.zfaw79
Anopheles campestris	100.85772	14.66424	Unclassified	GBIF: https://doi.org/10.15468/dl.zfaw79
Anopheles campestris	100.89854	14.6612	Unclassified	GBIF: https://doi.org/10.15468/dl.zfaw79
Anopheles campestris	100.9323	12.90401	Unclassified	GBIF: https://doi.org/10.15468/dl.zfaw79
Anopheles campestris	100.93455	13.19321	Unclassified	GBIF: https://doi.org/10.15468/dl.zfaw79
Anopheles campestris	100.95415	14.01007	Unclassified	GBIF: https://doi.org/10.15468/dl.zfaw79
Anopheles campestris	101.007553	13.311877	Unclassified	GBIF: https://doi.org/10.15468/dl.zfaw79
Anopheles campestris	101.268126	15.693213	Unclassified	GBIF: https://doi.org/10.15468/dl.zfaw79
Anopheles campestris	101.438401	14.625377	Unclassified	GBIF: https://doi.org/10.15468/dl.zfaw79
Anopheles campestris	101.501371	13.239733	Unclassified	GBIF: https://doi.org/10.15468/dl.zfaw79
Anopheles campestris	101.569413	13.04301	Unclassified	GBIF: https://doi.org/10.15468/dl.zfaw79
Anopheles campestris	101.585851	13.286359	Unclassified	GBIF: https://doi.org/10.15468/dl.zfaw79
Anopheles campestris	101.660153	13.133261	Unclassified	GBIF: https://doi.org/10.15468/dl.zfaw79
Anopheles campestris	101.741003	13.129274	Unclassified	GBIF: https://doi.org/10.15468/dl.zfaw79
Anopheles campestris	101.742333	13.015038	Unclassified	GBIF: https://doi.org/10.15468/dl.zfaw79
Anopheles campestris	101.758915	13.097968	Unclassified	GBIF: https://doi.org/10.15468/dl.zfaw79
Anopheles campestris	101.767664	5.964042	Unclassified	GBIF: https://doi.org/10.15468/dl.zfaw79
Anopheles campestris	101.82424	14.33206	Unclassified	GBIF: https://doi.org/10.15468/dl.zfaw79
Anopheles campestris	101.8335	14.33195	Unclassified	GBIF: https://doi.org/10.15468/dl.zfaw79
Anopheles campestris	101.86975	14.2683	Unclassified	GBIF: https://doi.org/10.15468/dl.zfaw79
Anopheles campestris	101.875857	14.158867	Unclassified	GBIF: https://doi.org/10.15468/dl.zfaw79
Anopheles campestris	101.87901	14.26818	Unclassified	GBIF: https://doi.org/10.15468/dl.zfaw79
Anopheles campestris	101.881875	14.193961	Unclassified	GBIF: https://doi.org/10.15468/dl.zfaw79
Anopheles campestris	101.887067	14.240018	Unclassified	GBIF: https://doi.org/10.15468/dl.zfaw79
Anopheles campestris	101.88815	14.25904	Unclassified	GBIF: https://doi.org/10.15468/dl.zfaw79
Anopheles campestris	101.88896	14.32225	Unclassified	GBIF: https://doi.org/10.15468/dl.zfaw79
Anopheles campestris	101.891961	14.260731	Unclassified	GBIF: https://doi.org/10.15468/dl.zfaw79
Anopheles campestris	101.893175	14.283296	Unclassified	GBIF: https://doi.org/10.15468/dl.zfaw79
Anopheles campestris	101.8966	14.19572	Unclassified	GBIF: https://doi.org/10.15468/dl.zfaw79
Anopheles campestris	101.89672	14.20475	Unclassified	GBIF: https://doi.org/10.15468/dl.zfaw79
Anopheles campestris	101.89753	14.26796	Unclassified	GBIF: https://doi.org/10.15468/dl.zfaw79
Anopheles campestris	101.907779	13.227123	Unclassified	GBIF: https://doi.org/10.15468/dl.zfaw79

Anopheles campestris	101.922512	14.204013	Unclassified	GBIF: https://doi.org/10.15468/dl.zfaw79
Anopheles campestris	101.940633	13.509772	Unclassified	GBIF: https://doi.org/10.15468/dl.zfaw79
Anopheles campestris	101.961118	13.464004	Unclassified	GBIF: https://doi.org/10.15468/dl.zfaw79
Anopheles campestris	101.975685	14.077272	Unclassified	GBIF: https://doi.org/10.15468/dl.zfaw79
Anopheles campestris	101.992168	13.381358	Unclassified	GBIF: https://doi.org/10.15468/dl.zfaw79
Anopheles campestris	102.004328	14.040758	Unclassified	GBIF: https://doi.org/10.15468/dl.zfaw79
Anopheles campestris	102.019523	14.145245	Unclassified	GBIF: https://doi.org/10.15468/dl.zfaw79
Anopheles campestris	102.02286	13.350541	Unclassified	GBIF: https://doi.org/10.15468/dl.zfaw79
Anopheles campestris	102.0244	12.51496	Unclassified	GBIF: https://doi.org/10.15468/dl.zfaw79
Anopheles campestris	102.033384	13.49167	Unclassified	GBIF: https://doi.org/10.15468/dl.zfaw79
Anopheles campestris	102.033679	13.960971	Unclassified	GBIF: https://doi.org/10.15468/dl.zfaw79
Anopheles campestris	102.03642	12.912429	Unclassified	GBIF: https://doi.org/10.15468/dl.zfaw79
Anopheles campestris	102.037568	14.090165	Unclassified	GBIF: https://doi.org/10.15468/dl.zfaw79
Anopheles campestris	102.039792	14.056518	Unclassified	GBIF: https://doi.org/10.15468/dl.zfaw79
Anopheles campestris	102.047735	14.018942	Unclassified	GBIF: https://doi.org/10.15468/dl.zfaw79
Anopheles campestris	102.055482	13.322303	Unclassified	GBIF: https://doi.org/10.15468/dl.zfaw79
Anopheles campestris	102.066645	13.986658	Unclassified	GBIF: https://doi.org/10.15468/dl.zfaw79
Anopheles campestris	102.07895	13.295772	Unclassified	GBIF: https://doi.org/10.15468/dl.zfaw79
Anopheles campestris	102.122565	13.70952	Unclassified	GBIF: https://doi.org/10.15468/dl.zfaw79
Anopheles campestris	102.127301	17.32058	Unclassified	GBIF: https://doi.org/10.15468/dl.zfaw79
Anopheles campestris	102.147146	13.338472	Unclassified	GBIF: https://doi.org/10.15468/dl.zfaw79
Anopheles campestris	102.162099	13.328166	Unclassified	GBIF: https://doi.org/10.15468/dl.zfaw79
Anopheles campestris	102.207242	13.116654	Unclassified	GBIF: https://doi.org/10.15468/dl.zfaw79
Anopheles campestris	102.208368	13.969362	Unclassified	GBIF: https://doi.org/10.15468/dl.zfaw79
Anopheles campestris	102.243543	13.967055	Unclassified	GBIF: https://doi.org/10.15468/dl.zfaw79
Anopheles campestris	102.248019	13.296443	Unclassified	GBIF: https://doi.org/10.15468/dl.zfaw79
Anopheles campestris	102.26719	13.442497	Unclassified	GBIF: https://doi.org/10.15468/dl.zfaw79
Anopheles campestris	102.280828	12.646369	Unclassified	GBIF: https://doi.org/10.15468/dl.zfaw79
Anopheles campestris	102.287214	12.881731	Unclassified	GBIF: https://doi.org/10.15468/dl.zfaw79
Anopheles campestris	102.304465	13.410379	Unclassified	GBIF: https://doi.org/10.15468/dl.zfaw79
Anopheles campestris	102.328466	13.492841	Unclassified	GBIF: https://doi.org/10.15468/dl.zfaw79
Anopheles campestris	102.331768	12.494625	Unclassified	GBIF: https://doi.org/10.15468/dl.zfaw79
Anopheles campestris	102.34049	13.407148	Unclassified	GBIF: https://doi.org/10.15468/dl.zfaw79
Anopheles campestris	102.34243	16.69156	Unclassified	GBIF: https://doi.org/10.15468/dl.zfaw79
Anopheles campestris	102.344765	13.932048	Unclassified	GBIF: https://doi.org/10.15468/dl.zfaw79
Anopheles campestris	102.349325	12.304173	Unclassified	GBIF: https://doi.org/10.15468/dl.zfaw79
Anopheles campestris	102.390085	13.083338	Unclassified	GBIF: https://doi.org/10.15468/dl.zfaw79
Anopheles campestris	102.413476	13.824381	Unclassified	GBIF: https://doi.org/10.15468/dl.zfaw79
Anopheles campestris	102.462267	12.595272	Unclassified	GBIF: https://doi.org/10.15468/dl.zfaw79
Anopheles campestris	102.470532	12.894781	Unclassified	GBIF: https://doi.org/10.15468/dl.zfaw79
Anopheles campestris	102.472119	12.62247	Unclassified	GBIF: https://doi.org/10.15468/dl.zfaw79
Anopheles campestris	102.574818	14.044675	Unclassified	GBIF: https://doi.org/10.15468/dl.zfaw79

Anopheles campestris	102.618292	12.230746	Unclassified	GBIF: https://doi.org/10.15468/dl.zfaw79
Anopheles campestris	102.663431	13.941306	Unclassified	GBIF: https://doi.org/10.15468/dl.zfaw79
Anopheles campestris	102.680198	12.474401	Unclassified	GBIF: https://doi.org/10.15468/dl.zfaw79
Anopheles campestris	103.220715	16.600609	Unclassified	GBIF: https://doi.org/10.15468/dl.zfaw79
Anopheles campestris	103.30278	16.37926	Unclassified	GBIF: https://doi.org/10.15468/dl.zfaw79
Anopheles campestris	103.38384	15.05659	Unclassified	GBIF: https://doi.org/10.15468/dl.zfaw79
Anopheles campestris	103.754088	17.850781	Unclassified	GBIF: https://doi.org/10.15468/dl.zfaw79
Anopheles campestris	103.873492	14.453018	Unclassified	GBIF: https://doi.org/10.15468/dl.zfaw79
Anopheles campestris	103.912857	16.92786	Unclassified	GBIF: https://doi.org/10.15468/dl.zfaw79
Anopheles campestris	104.063014	14.433857	Unclassified	GBIF: https://doi.org/10.15468/dl.zfaw79
Anopheles campestris	104.208742	14.503051	Unclassified	GBIF: https://doi.org/10.15468/dl.zfaw79
Anopheles campestris	104.211418	14.506937	Unclassified	GBIF: https://doi.org/10.15468/dl.zfaw79
Anopheles campestris	104.22271	14.615236	Unclassified	GBIF: https://doi.org/10.15468/dl.zfaw79
Anopheles campestris	104.24534	16.83593	Unclassified	GBIF: https://doi.org/10.15468/dl.zfaw79
Anopheles campestris	104.312034	14.554037	Unclassified	GBIF: https://doi.org/10.15468/dl.zfaw79
Anopheles campestris	104.375223	14.528899	Unclassified	GBIF: https://doi.org/10.15468/dl.zfaw79
Anopheles campestris	104.409567	16.802636	Unclassified	GBIF: https://doi.org/10.15468/dl.zfaw79
Anopheles campestris	104.506653	16.637468	Unclassified	GBIF: https://doi.org/10.15468/dl.zfaw79
Anopheles campestris	104.691789	14.480704	Unclassified	GBIF: https://doi.org/10.15468/dl.zfaw79
Anopheles campestris	105.311586	15.697635	Unclassified	GBIF: https://doi.org/10.15468/dl.zfaw79
Anopheles canorii	-53.40888	4.61955	Unclassified	GBIF: https://doi.org/10.15468/dl.zfaw79
Anopheles canorii	-53.24036	3.91451	Unclassified	GBIF: https://doi.org/10.15468/dl.zfaw79
Anopheles canorii	-53.23477	3.92404	Unclassified	GBIF: https://doi.org/10.15468/dl.zfaw79
Anopheles canorii	-52.67012	3.61844	Unclassified	GBIF: https://doi.org/10.15468/dl.zfaw79
Anopheles carnevalei	9.59	2.22	Unclassified	GBIF: https://doi.org/10.15468/dl.zfaw79
Anopheles carnevalei	11.59	-0.19	Unclassified	GBIF: https://doi.org/10.15468/dl.zfaw79
Anopheles carnevalei	11.59327	-0.19124	Unclassified	GBIF: https://doi.org/10.15468/dl.zfaw79
Anopheles carnevalei	11.6	-0.2	Unclassified	GBIF: https://doi.org/10.15468/dl.zfaw79
Anopheles carnevalei	11.66	-0.33	Unclassified	GBIF: https://doi.org/10.15468/dl.zfaw79
Anopheles carnevalei	12.948474	6.294169	Unclassified	GBIF: https://doi.org/10.15468/dl.zfaw79
Anopheles chiriquiensis	-84.369122	10.228106	Unclassified	GBIF: https://doi.org/10.15468/dl.zfaw79
Anopheles cinctus	3.01587	6.90045	Unclassified	GBIF: https://doi.org/10.15468/dl.zfaw79
Anopheles cinctus	3.33254	6.58381	Unclassified	GBIF: https://doi.org/10.15468/dl.zfaw79
Anopheles cinctus	3.39921	6.45049	Unclassified	GBIF: https://doi.org/10.15468/dl.zfaw79
Anopheles cinctus	3.93255	6.81712	Unclassified	GBIF: https://doi.org/10.15468/dl.zfaw79
Anopheles cinctus	5.69925	5.90055	Unclassified	GBIF: https://doi.org/10.15468/dl.zfaw79
Anopheles cinctus	5.86592	6.0672	Unclassified	GBIF: https://doi.org/10.15468/dl.zfaw79
Anopheles claviger	-9.511509	53.797515	Unclassified	GBIF: https://doi.org/10.15468/dl.zfaw79
Anopheles claviger	-9.503163	52.058739	Unclassified	GBIF: https://doi.org/10.15468/dl.zfaw79
Anopheles claviger	-9.476532	52.783902	Unclassified	GBIF: https://doi.org/10.15468/dl.zfaw79
Anopheles claviger	-9.404934	53.423222	Unclassified	GBIF: https://doi.org/10.15468/dl.zfaw79
Anopheles claviger	-9.301554	52.942749	Unclassified	GBIF: https://doi.org/10.15468/dl.zfaw79

Anopheles claviger	-9.235004	54.114876	Unclassified	GBIF: https://doi.org/10.15468/dl.zfaw79
Anopheles claviger	-9.169522	53.341295	Unclassified	GBIF: https://doi.org/10.15468/dl.zfaw79
Anopheles claviger	-9.147701	51.585037	Unclassified	GBIF: https://doi.org/10.15468/dl.zfaw79
Anopheles claviger	-9.120909	53.662535	Unclassified	GBIF: https://doi.org/10.15468/dl.zfaw79
Anopheles claviger	-9.041932	52.959107	Unclassified	GBIF: https://doi.org/10.15468/dl.zfaw79
Anopheles claviger	-9.024355	51.873787	Unclassified	GBIF: https://doi.org/10.15468/dl.zfaw79
Anopheles claviger	-8.931983	53.015862	Unclassified	GBIF: https://doi.org/10.15468/dl.zfaw79
Anopheles claviger	-8.87463	52.610893	Unclassified	GBIF: https://doi.org/10.15468/dl.zfaw79
Anopheles claviger	-8.835211	53.094348	Unclassified	GBIF: https://doi.org/10.15468/dl.zfaw79
Anopheles claviger	-8.709155	51.634792	Unclassified	GBIF: https://doi.org/10.15468/dl.zfaw79
Anopheles claviger	-8.60257	53.498966	Unclassified	GBIF: https://doi.org/10.15468/dl.zfaw79
Anopheles claviger	-8.549525	52.523565	Unclassified	GBIF: https://doi.org/10.15468/dl.zfaw79
Anopheles claviger	-8.482623	53.209694	Unclassified	GBIF: https://doi.org/10.15468/dl.zfaw79
Anopheles claviger	-8.451	40.224	Unclassified	GBIF: https://doi.org/10.15468/dl.zfaw79
Anopheles claviger	-8.451	40.229	Unclassified	GBIF: https://doi.org/10.15468/dl.zfaw79
Anopheles claviger	-8.374241	53.390463	Unclassified	GBIF: https://doi.org/10.15468/dl.zfaw79
Anopheles claviger	-8.241992	53.083214	Unclassified	GBIF: https://doi.org/10.15468/dl.zfaw79
Anopheles claviger	-7.953745	53.317867	Unclassified	GBIF: https://doi.org/10.15468/dl.zfaw79
Anopheles claviger	-7.946895	53.32291	Unclassified	GBIF: https://doi.org/10.15468/dl.zfaw79
Anopheles claviger	-7.863137	53.88343	Unclassified	GBIF: https://doi.org/10.15468/dl.zfaw79
Anopheles claviger	-7.85	52.1333	Unclassified	GBIF: https://doi.org/10.15468/dl.zfaw79
Anopheles claviger	-7.615882	53.315854	Unclassified	GBIF: https://doi.org/10.15468/dl.zfaw79
Anopheles claviger	-7.551213	53.899632	Unclassified	GBIF: https://doi.org/10.15468/dl.zfaw79
Anopheles claviger	-7.513828	53.252135	Unclassified	GBIF: https://doi.org/10.15468/dl.zfaw79
Anopheles claviger	-7.382261	53.596102	Unclassified	GBIF: https://doi.org/10.15468/dl.zfaw79
Anopheles claviger	-7.358626	52.577995	Unclassified	GBIF: https://doi.org/10.15468/dl.zfaw79
Anopheles claviger	-7.037534	53.923061	Unclassified	GBIF: https://doi.org/10.15468/dl.zfaw79
Anopheles claviger	-6.959803	52.77458	Unclassified	GBIF: https://doi.org/10.15468/dl.zfaw79
Anopheles claviger	-6.950078	52.377289	Unclassified	GBIF: https://doi.org/10.15468/dl.zfaw79
Anopheles claviger	-6.852393	53.195035	Unclassified	GBIF: https://doi.org/10.15468/dl.zfaw79
Anopheles claviger	-6.559447	53.140631	Unclassified	GBIF: https://doi.org/10.15468/dl.zfaw79
Anopheles claviger	-6.525177	52.348483	Unclassified	GBIF: https://doi.org/10.15468/dl.zfaw79
Anopheles claviger	-6.511346	52.345164	Unclassified	GBIF: https://doi.org/10.15468/dl.zfaw79
Anopheles claviger	-6.416344	52.190315	Unclassified	GBIF: https://doi.org/10.15468/dl.zfaw79
Anopheles claviger	-6.395767	53.321856	Unclassified	GBIF: https://doi.org/10.15468/dl.zfaw79
Anopheles claviger	-6.363011	52.388139	Unclassified	GBIF: https://doi.org/10.15468/dl.zfaw79
Anopheles claviger	-6.358232	53.244064	Unclassified	GBIF: https://doi.org/10.15468/dl.zfaw79
Anopheles claviger	-6.320913	53.009917	Unclassified	GBIF: https://doi.org/10.15468/dl.zfaw79
Anopheles claviger	-6.197576	53.359463	Unclassified	GBIF: https://doi.org/10.15468/dl.zfaw79
Anopheles claviger	-6.03902	53.066243	Unclassified	GBIF: https://doi.org/10.15468/dl.zfaw79
Anopheles claviger	-5.934989	54.601092	Unclassified	GBIF: https://doi.org/10.15468/dl.zfaw79
Anopheles claviger	-5.929782	54.59696	Unclassified	GBIF: https://doi.org/10.15468/dl.zfaw79

Anopheles claviger	-5.929736	54.597858	Unclassified	GBIF: https://doi.org/10.15468/dl.zfaw79
Anopheles claviger	-5.929691	54.598756	Unclassified	GBIF: https://doi.org/10.15468/dl.zfaw79
Anopheles claviger	-5.834589	54.641118	Unclassified	GBIF: https://doi.org/10.15468/dl.zfaw79
Anopheles claviger	-5.24179	51.895484	Unclassified	GBIF: https://doi.org/10.15468/dl.zfaw79
Anopheles claviger	-5.229118	51.901229	Unclassified	GBIF: https://doi.org/10.15468/dl.zfaw79
Anopheles claviger	-5.220526	51.89292	Unclassified	GBIF: https://doi.org/10.15468/dl.zfaw79
Anopheles claviger	-5.193938	51.938167	Unclassified	GBIF: https://doi.org/10.15468/dl.zfaw79
Anopheles claviger	-5.189581	51.938285	Unclassified	GBIF: https://doi.org/10.15468/dl.zfaw79
Anopheles claviger	-5.08407	51.96988	Unclassified	GBIF: https://doi.org/10.15468/dl.zfaw79
Anopheles claviger	-5.048931	51.945613	Unclassified	GBIF: https://doi.org/10.15468/dl.zfaw79
Anopheles claviger	-5.045533	51.658793	Unclassified	GBIF: https://doi.org/10.15468/dl.zfaw79
Anopheles claviger	-5.038761	51.945876	Unclassified	GBIF: https://doi.org/10.15468/dl.zfaw79
Anopheles claviger	-5.037309	51.945913	Unclassified	GBIF: https://doi.org/10.15468/dl.zfaw79
Anopheles claviger	-4.99417	51.997384	Unclassified	GBIF: https://doi.org/10.15468/dl.zfaw79
Anopheles claviger	-4.903866	52.020332	Unclassified	GBIF: https://doi.org/10.15468/dl.zfaw79
Anopheles claviger	-4.721552	51.65776	Unclassified	GBIF: https://doi.org/10.15468/dl.zfaw79
Anopheles claviger	-4.720108	51.657794	Unclassified	GBIF: https://doi.org/10.15468/dl.zfaw79
Anopheles claviger	-4.652051	52.075736	Unclassified	GBIF: https://doi.org/10.15468/dl.zfaw79
Anopheles claviger	-4.528059	53.267651	Unclassified	GBIF: https://doi.org/10.15468/dl.zfaw79
Anopheles claviger	-4.527161	52.908461	Unclassified	GBIF: https://doi.org/10.15468/dl.zfaw79
Anopheles claviger	-4.500967	53.407135	Unclassified	GBIF: https://doi.org/10.15468/dl.zfaw79
Anopheles claviger	-4.498109	52.894689	Unclassified	GBIF: https://doi.org/10.15468/dl.zfaw79
Anopheles claviger	-4.48162	52.892338	Unclassified	GBIF: https://doi.org/10.15468/dl.zfaw79
Anopheles claviger	-4.413232	57.503577	Unclassified	GBIF: https://doi.org/10.15468/dl.zfaw79
Anopheles claviger	-4.37264	53.20144	Unclassified	GBIF: https://doi.org/10.15468/dl.zfaw79
Anopheles claviger	-4.37217	53.20118	Unclassified	GBIF: https://doi.org/10.15468/dl.zfaw79
Anopheles claviger	-4.337236	53.343473	Unclassified	GBIF: https://doi.org/10.15468/dl.zfaw79
Anopheles claviger	-4.33283	50.58178	Unclassified	GBIF: https://doi.org/10.15468/dl.zfaw79
Anopheles claviger	-4.320496	52.126796	Unclassified	GBIF: https://doi.org/10.15468/dl.zfaw79
Anopheles claviger	-4.304847	53.313985	Unclassified	GBIF: https://doi.org/10.15468/dl.zfaw79
Anopheles claviger	-4.300154	53.310479	Unclassified	GBIF: https://doi.org/10.15468/dl.zfaw79
Anopheles claviger	-4.298655	53.310508	Unclassified	GBIF: https://doi.org/10.15468/dl.zfaw79
Anopheles claviger	-4.294782	53.32227	Unclassified	GBIF: https://doi.org/10.15468/dl.zfaw79
Anopheles claviger	-4.293051	53.009419	Unclassified	GBIF: https://doi.org/10.15468/dl.zfaw79
Anopheles claviger	-4.292993	53.31691	Unclassified	GBIF: https://doi.org/10.15468/dl.zfaw79
Anopheles claviger	-4.289705	53.311579	Unclassified	GBIF: https://doi.org/10.15468/dl.zfaw79
Anopheles claviger	-4.288425	51.699379	Unclassified	GBIF: https://doi.org/10.15468/dl.zfaw79
Anopheles claviger	-4.288205	53.311608	Unclassified	GBIF: https://doi.org/10.15468/dl.zfaw79
Anopheles claviger	-4.28267	50.40324	Unclassified	GBIF: https://doi.org/10.15468/dl.zfaw79
Anopheles claviger	-4.262408	51.627491	Unclassified	GBIF: https://doi.org/10.15468/dl.zfaw79
Anopheles claviger	-4.260295	53.308994	Unclassified	GBIF: https://doi.org/10.15468/dl.zfaw79
Anopheles claviger	-4.257244	51.625342	Unclassified	GBIF: https://doi.org/10.15468/dl.zfaw79

Anopheles claviger	-4.253003	51.627222	Unclassified	GBIF: https://doi.org/10.15468/dl.zfaw79
Anopheles claviger	-4.246534	53.289924	Unclassified	GBIF: https://doi.org/10.15468/dl.zfaw79
Anopheles claviger	-4.245082	53.29085	Unclassified	GBIF: https://doi.org/10.15468/dl.zfaw79
Anopheles claviger	-4.242642	53.272915	Unclassified	GBIF: https://doi.org/10.15468/dl.zfaw79
Anopheles claviger	-4.241097	53.272045	Unclassified	GBIF: https://doi.org/10.15468/dl.zfaw79
Anopheles claviger	-4.240976	51.632398	Unclassified	GBIF: https://doi.org/10.15468/dl.zfaw79
Anopheles claviger	-4.23919	53.3156	Unclassified	GBIF: https://doi.org/10.15468/dl.zfaw79
Anopheles claviger	-4.203781	52.20412	Unclassified	GBIF: https://doi.org/10.15468/dl.zfaw79
Anopheles claviger	-4.174845	53.252144	Unclassified	GBIF: https://doi.org/10.15468/dl.zfaw79
Anopheles claviger	-4.09883	50.73238	Unclassified	GBIF: https://doi.org/10.15468/dl.zfaw79
Anopheles claviger	-4.09679	50.68367	Unclassified	GBIF: https://doi.org/10.15468/dl.zfaw79
Anopheles claviger	-4.06186	50.72971	Unclassified	GBIF: https://doi.org/10.15468/dl.zfaw79
Anopheles claviger	-3.957096	57.089186	Unclassified	GBIF: https://doi.org/10.15468/dl.zfaw79
Anopheles claviger	-3.952914	52.539876	Unclassified	GBIF: https://doi.org/10.15468/dl.zfaw79
Anopheles claviger	-3.952192	51.637557	Unclassified	GBIF: https://doi.org/10.15468/dl.zfaw79
Anopheles claviger	-3.947505	52.217172	Unclassified	GBIF: https://doi.org/10.15468/dl.zfaw79
Anopheles claviger	-3.946082	52.218095	Unclassified	GBIF: https://doi.org/10.15468/dl.zfaw79
Anopheles claviger	-3.887018	51.634127	Unclassified	GBIF: https://doi.org/10.15468/dl.zfaw79
Anopheles claviger	-3.85695	50.05147	Unclassified	GBIF: https://doi.org/10.15468/dl.zfaw79
Anopheles claviger	-3.804204	51.550002	Unclassified	GBIF: https://doi.org/10.15468/dl.zfaw79
Anopheles claviger	-3.73056	53.219419	Unclassified	GBIF: https://doi.org/10.15468/dl.zfaw79
Anopheles claviger	-3.729027	53.218542	Unclassified	GBIF: https://doi.org/10.15468/dl.zfaw79
Anopheles claviger	-3.721487	53.254611	Unclassified	GBIF: https://doi.org/10.15468/dl.zfaw79
Anopheles claviger	-3.68175	51.540266	Unclassified	GBIF: https://doi.org/10.15468/dl.zfaw79
Anopheles claviger	-3.669942	51.821752	Unclassified	GBIF: https://doi.org/10.15468/dl.zfaw79
Anopheles claviger	-3.663558	51.786324	Unclassified	GBIF: https://doi.org/10.15468/dl.zfaw79
Anopheles claviger	-3.660047	51.552131	Unclassified	GBIF: https://doi.org/10.15468/dl.zfaw79
Anopheles claviger	-3.46912	50.67484	Unclassified	GBIF: https://doi.org/10.15468/dl.zfaw79
Anopheles claviger	-3.46911	50.67925	Unclassified	GBIF: https://doi.org/10.15468/dl.zfaw79
Anopheles claviger	-3.46876	50.67709	Unclassified	GBIF: https://doi.org/10.15468/dl.zfaw79
Anopheles claviger	-3.442154	52.207349	Unclassified	GBIF: https://doi.org/10.15468/dl.zfaw79
Anopheles claviger	-3.350987	52.193143	Unclassified	GBIF: https://doi.org/10.15468/dl.zfaw79
Anopheles claviger	-3.349443	52.190464	Unclassified	GBIF: https://doi.org/10.15468/dl.zfaw79
Anopheles claviger	-3.348035	52.192278	Unclassified	GBIF: https://doi.org/10.15468/dl.zfaw79
Anopheles claviger	-3.347953	52.189582	Unclassified	GBIF: https://doi.org/10.15468/dl.zfaw79
Anopheles claviger	-3.334469	52.178946	Unclassified	GBIF: https://doi.org/10.15468/dl.zfaw79
Anopheles claviger	-3.24209	54.164387	Unclassified	GBIF: https://doi.org/10.15468/dl.zfaw79
Anopheles claviger	-3.21422	56.321431	Unclassified	GBIF: https://doi.org/10.15468/dl.zfaw79
Anopheles claviger	-3.178369	51.74993	Unclassified	GBIF: https://doi.org/10.15468/dl.zfaw79
Anopheles claviger	-3.178106	52.128464	Unclassified	GBIF: https://doi.org/10.15468/dl.zfaw79
Anopheles claviger	-3.074116	53.59075	Unclassified	GBIF: https://doi.org/10.15468/dl.zfaw79
Anopheles claviger	-3.040816	53.465201	Unclassified	GBIF: https://doi.org/10.15468/dl.zfaw79

Anopheles claviger	-3.011605	53.091055	Unclassified	GBIF: https://doi.org/10.15468/dl.zfaw79
Anopheles claviger	-3.007937	53.026766	Unclassified	GBIF: https://doi.org/10.15468/dl.zfaw79
Anopheles claviger	-3.000428	53.030875	Unclassified	GBIF: https://doi.org/10.15468/dl.zfaw79
Anopheles claviger	-2.998082	53.02613	Unclassified	GBIF: https://doi.org/10.15468/dl.zfaw79
Anopheles claviger	-2.986238	53.026544	Unclassified	GBIF: https://doi.org/10.15468/dl.zfaw79
Anopheles claviger	-2.939038	51.560118	Unclassified	GBIF: https://doi.org/10.15468/dl.zfaw79
Anopheles claviger	-2.913743	54.207771	Unclassified	GBIF: https://doi.org/10.15468/dl.zfaw79
Anopheles claviger	-2.899652	51.354964	Unclassified	GBIF: https://doi.org/10.15468/dl.zfaw79
Anopheles claviger	-2.882	53.227	Unclassified	GBIF: https://doi.org/10.15468/dl.zfaw79
Anopheles claviger	-2.827815	51.381076	Unclassified	GBIF: https://doi.org/10.15468/dl.zfaw79
Anopheles claviger	-2.825587	51.44889	Unclassified	GBIF: https://doi.org/10.15468/dl.zfaw79
Anopheles claviger	-2.823366	51.381377	Unclassified	GBIF: https://doi.org/10.15468/dl.zfaw79
Anopheles claviger	-2.822717	51.377245	Unclassified	GBIF: https://doi.org/10.15468/dl.zfaw79
Anopheles claviger	-2.819267	52.9096	Unclassified	GBIF: https://doi.org/10.15468/dl.zfaw79
Anopheles claviger	-2.805558	54.163605	Unclassified	GBIF: https://doi.org/10.15468/dl.zfaw79
Anopheles claviger	-2.804654	51.461444	Unclassified	GBIF: https://doi.org/10.15468/dl.zfaw79
Anopheles claviger	-2.800875	51.459312	Unclassified	GBIF: https://doi.org/10.15468/dl.zfaw79
Anopheles claviger	-2.794801	51.561184	Unclassified	GBIF: https://doi.org/10.15468/dl.zfaw79
Anopheles claviger	-2.784602	51.458883	Unclassified	GBIF: https://doi.org/10.15468/dl.zfaw79
Anopheles claviger	-2.780292	51.459361	Unclassified	GBIF: https://doi.org/10.15468/dl.zfaw79
Anopheles claviger	-2.77889	51.461528	Unclassified	GBIF: https://doi.org/10.15468/dl.zfaw79
Anopheles claviger	-2.752595	52.923525	Unclassified	GBIF: https://doi.org/10.15468/dl.zfaw79
Anopheles claviger	-2.704765	54.315677	Unclassified	GBIF: https://doi.org/10.15468/dl.zfaw79
Anopheles claviger	-2.683	42.853	Unclassified	GBIF: https://doi.org/10.15468/dl.zfaw79
Anopheles claviger	-2.658	43.347	Unclassified	GBIF: https://doi.org/10.15468/dl.zfaw79
Anopheles claviger	-2.6	42.44	Unclassified	GBIF: https://doi.org/10.15468/dl.zfaw79
Anopheles claviger	-2.59667	42.43623	Unclassified	GBIF: https://doi.org/10.15468/dl.zfaw79
Anopheles claviger	-2.592855	51.617675	Unclassified	GBIF: https://doi.org/10.15468/dl.zfaw79
Anopheles claviger	-2.533	42.891	Unclassified	GBIF: https://doi.org/10.15468/dl.zfaw79
Anopheles claviger	-2.51	42.44	Unclassified	GBIF: https://doi.org/10.15468/dl.zfaw79
Anopheles claviger	-2.50962	42.44007	Unclassified	GBIF: https://doi.org/10.15468/dl.zfaw79
Anopheles claviger	-2.50962	42.4401	Unclassified	GBIF: https://doi.org/10.15468/dl.zfaw79
Anopheles claviger	-2.41	42.48	Unclassified	GBIF: https://doi.org/10.15468/dl.zfaw79
Anopheles claviger	-2.4071	42.48438	Unclassified	GBIF: https://doi.org/10.15468/dl.zfaw79
Anopheles claviger	-1.97517	50.696592	Unclassified	GBIF: https://doi.org/10.15468/dl.zfaw79
Anopheles claviger	-1.965967	50.696141	Unclassified	GBIF: https://doi.org/10.15468/dl.zfaw79
Anopheles claviger	-1.930617	50.66465	Unclassified	GBIF: https://doi.org/10.15468/dl.zfaw79
Anopheles claviger	-1.840157	54.301005	Unclassified	GBIF: https://doi.org/10.15468/dl.zfaw79
Anopheles claviger	-1.738889	46.994446	Unclassified	GBIF: https://doi.org/10.15468/dl.zfaw79
Anopheles claviger	-1.564855	54.542529	Unclassified	GBIF: https://doi.org/10.15468/dl.zfaw79
Anopheles claviger	-1.53724	46.2364	Unclassified	GBIF: https://doi.org/10.15468/dl.zfaw79
Anopheles claviger	-1.51466	46.21101	Unclassified	GBIF: https://doi.org/10.15468/dl.zfaw79

Anopheles claviger	-1.43646	46.20142	Unclassified	GBIF: https://doi.org/10.15468/dl.zfaw79
Anopheles claviger	-1.39	51.72	Unclassified	GBIF: https://doi.org/10.15468/dl.zfaw79
Anopheles claviger	-1.38617	51.71962	Unclassified	GBIF: https://doi.org/10.15468/dl.zfaw79
Anopheles claviger	-1.36051	53.517464	Unclassified	GBIF: https://doi.org/10.15468/dl.zfaw79
Anopheles claviger	-1.323881	53.449848	Unclassified	GBIF: https://doi.org/10.15468/dl.zfaw79
Anopheles claviger	-1.32386	45.97605	Unclassified	GBIF: https://doi.org/10.15468/dl.zfaw79
Anopheles claviger	-1.3216	46.15647	Unclassified	GBIF: https://doi.org/10.15468/dl.zfaw79
Anopheles claviger	-1.316711	50.692712	Unclassified	GBIF: https://doi.org/10.15468/dl.zfaw79
Anopheles claviger	-1.30563	45.93966	Unclassified	GBIF: https://doi.org/10.15468/dl.zfaw79
Anopheles claviger	-1.2253	45.83295	Unclassified	GBIF: https://doi.org/10.15468/dl.zfaw79
Anopheles claviger	-1.21646	45.88134	Unclassified	GBIF: https://doi.org/10.15468/dl.zfaw79
Anopheles claviger	-1.211727	52.999708	Unclassified	GBIF: https://doi.org/10.15468/dl.zfaw79
Anopheles claviger	-1.19082	45.75932	Unclassified	GBIF: https://doi.org/10.15468/dl.zfaw79
Anopheles claviger	-1.18614	46.19378	Unclassified	GBIF: https://doi.org/10.15468/dl.zfaw79
Anopheles claviger	-1.175062	53.359025	Unclassified	GBIF: https://doi.org/10.15468/dl.zfaw79
Anopheles claviger	-1.1735	46.16242	Unclassified	GBIF: https://doi.org/10.15468/dl.zfaw79
Anopheles claviger	-1.173322	53.448901	Unclassified	GBIF: https://doi.org/10.15468/dl.zfaw79
Anopheles claviger	-1.16992	45.70639	Unclassified	GBIF: https://doi.org/10.15468/dl.zfaw79
Anopheles claviger	-1.157371	52.729646	Unclassified	GBIF: https://doi.org/10.15468/dl.zfaw79
Anopheles claviger	-1.152484	52.981321	Unclassified	GBIF: https://doi.org/10.15468/dl.zfaw79
Anopheles claviger	-1.15	51.38333	Unclassified	GBIF: https://doi.org/10.15468/dl.zfaw79
Anopheles claviger	-1.11418	46.13445	Unclassified	GBIF: https://doi.org/10.15468/dl.zfaw79
Anopheles claviger	-1.11298	46.24843	Unclassified	GBIF: https://doi.org/10.15468/dl.zfaw79
Anopheles claviger	-1.10665	45.68573	Unclassified	GBIF: https://doi.org/10.15468/dl.zfaw79
Anopheles claviger	-1.10572	46.10681	Unclassified	GBIF: https://doi.org/10.15468/dl.zfaw79
Anopheles claviger	-1.090287	53.49086	Unclassified	GBIF: https://doi.org/10.15468/dl.zfaw79
Anopheles claviger	-1.08866	46.15805	Unclassified	GBIF: https://doi.org/10.15468/dl.zfaw79
Anopheles claviger	-1.08303	46.07222	Unclassified	GBIF: https://doi.org/10.15468/dl.zfaw79
Anopheles claviger	-1.07835	45.94427	Unclassified	GBIF: https://doi.org/10.15468/dl.zfaw79
Anopheles claviger	-1.077416	53.253588	Unclassified	GBIF: https://doi.org/10.15468/dl.zfaw79
Anopheles claviger	-1.07453	45.83521	Unclassified	GBIF: https://doi.org/10.15468/dl.zfaw79
Anopheles claviger	-1.07407	45.98136	Unclassified	GBIF: https://doi.org/10.15468/dl.zfaw79
Anopheles claviger	-1.07161	46.12145	Unclassified	GBIF: https://doi.org/10.15468/dl.zfaw79
Anopheles claviger	-1.071324	53.258036	Unclassified	GBIF: https://doi.org/10.15468/dl.zfaw79
Anopheles claviger	-1.06146	45.91337	Unclassified	GBIF: https://doi.org/10.15468/dl.zfaw79
Anopheles claviger	-1.05406	45.79461	Unclassified	GBIF: https://doi.org/10.15468/dl.zfaw79
Anopheles claviger	-1.05196	45.88864	Unclassified	GBIF: https://doi.org/10.15468/dl.zfaw79
Anopheles claviger	-1.05186	46.10285	Unclassified	GBIF: https://doi.org/10.15468/dl.zfaw79
Anopheles claviger	-1.04896	46.07716	Unclassified	GBIF: https://doi.org/10.15468/dl.zfaw79
Anopheles claviger	-1.03464	46.03977	Unclassified	GBIF: https://doi.org/10.15468/dl.zfaw79
Anopheles claviger	-1.02777	45.9435	Unclassified	GBIF: https://doi.org/10.15468/dl.zfaw79
Anopheles claviger	-1.02738	45.9882	Unclassified	GBIF: https://doi.org/10.15468/dl.zfaw79

Anopheles claviger	-1.01762	45.63437	Unclassified	GBIF: https://doi.org/10.15468/dl.zfaw79
Anopheles claviger	-1.0082	45.87041	Unclassified	GBIF: https://doi.org/10.15468/dl.zfaw79
Anopheles claviger	-1.00276	45.90977	Unclassified	GBIF: https://doi.org/10.15468/dl.zfaw79
Anopheles claviger	-0.98648	45.97359	Unclassified	GBIF: https://doi.org/10.15468/dl.zfaw79
Anopheles claviger	-0.978	51.454	Unclassified	GBIF: https://doi.org/10.15468/dl.zfaw79
Anopheles claviger	-0.9722	45.94288	Unclassified	GBIF: https://doi.org/10.15468/dl.zfaw79
Anopheles claviger	-0.972	51.456	Unclassified	GBIF: https://doi.org/10.15468/dl.zfaw79
Anopheles claviger	-0.962151	50.828167	Unclassified	GBIF: https://doi.org/10.15468/dl.zfaw79
Anopheles claviger	-0.962	50.828	Unclassified	GBIF: https://doi.org/10.15468/dl.zfaw79
Anopheles claviger	-0.96	50.83	Unclassified	GBIF: https://doi.org/10.15468/dl.zfaw79
Anopheles claviger	-0.95956	45.56436	Unclassified	GBIF: https://doi.org/10.15468/dl.zfaw79
Anopheles claviger	-0.95359	45.90259	Unclassified	GBIF: https://doi.org/10.15468/dl.zfaw79
Anopheles claviger	-0.95335	45.8676	Unclassified	GBIF: https://doi.org/10.15468/dl.zfaw79
Anopheles claviger	-0.95187	45.79151	Unclassified	GBIF: https://doi.org/10.15468/dl.zfaw79
Anopheles claviger	-0.90453	45.91977	Unclassified	GBIF: https://doi.org/10.15468/dl.zfaw79
Anopheles claviger	-0.888	51.569	Unclassified	GBIF: https://doi.org/10.15468/dl.zfaw79
Anopheles claviger	-0.87454	45.96376	Unclassified	GBIF: https://doi.org/10.15468/dl.zfaw79
Anopheles claviger	-0.73035	45.41802	Unclassified	GBIF: https://doi.org/10.15468/dl.zfaw79
Anopheles claviger	-0.71381	45.87244	Unclassified	GBIF: https://doi.org/10.15468/dl.zfaw79
Anopheles claviger	-0.70551	45.85057	Unclassified	GBIF: https://doi.org/10.15468/dl.zfaw79
Anopheles claviger	-0.67722	45.88655	Unclassified	GBIF: https://doi.org/10.15468/dl.zfaw79
Anopheles claviger	-0.67207	45.81803	Unclassified	GBIF: https://doi.org/10.15468/dl.zfaw79
Anopheles claviger	-0.553861	50.856955	Unclassified	GBIF: https://doi.org/10.15468/dl.zfaw79
Anopheles claviger	-0.38754	46.07346	Unclassified	GBIF: https://doi.org/10.15468/dl.zfaw79
Anopheles claviger	-0.362707	52.595469	Unclassified	GBIF: https://doi.org/10.15468/dl.zfaw79
Anopheles claviger	-0.276285	52.540297	Unclassified	GBIF: https://doi.org/10.15468/dl.zfaw79
Anopheles claviger	-0.1537	51.535	Unclassified	GBIF: https://doi.org/10.15468/dl.zfaw79
Anopheles claviger	-0.11	52.27	Unclassified	GBIF: https://doi.org/10.15468/dl.zfaw79
Anopheles claviger	0.09	52.42	Unclassified	GBIF: https://doi.org/10.15468/dl.zfaw79
Anopheles claviger	0.227774	51.861972	Unclassified	GBIF: https://doi.org/10.15468/dl.zfaw79
Anopheles claviger	0.279576	52.310595	Unclassified	GBIF: https://doi.org/10.15468/dl.zfaw79
Anopheles claviger	0.28638	52.31451	Unclassified	GBIF: https://doi.org/10.15468/dl.zfaw79
Anopheles claviger	0.479	51.483	Unclassified	GBIF: https://doi.org/10.15468/dl.zfaw79
Anopheles claviger	0.55	51.463	Unclassified	GBIF: https://doi.org/10.15468/dl.zfaw79
Anopheles claviger	0.60359	52.5582	Unclassified	GBIF: https://doi.org/10.15468/dl.zfaw79
Anopheles claviger	0.647486	52.218836	Unclassified	GBIF: https://doi.org/10.15468/dl.zfaw79
Anopheles claviger	0.648137	52.217473	Unclassified	GBIF: https://doi.org/10.15468/dl.zfaw79
Anopheles claviger	0.649206	52.235434	Unclassified	GBIF: https://doi.org/10.15468/dl.zfaw79
Anopheles claviger	0.653817	52.226788	Unclassified	GBIF: https://doi.org/10.15468/dl.zfaw79
Anopheles claviger	0.657399	52.213218	Unclassified	GBIF: https://doi.org/10.15468/dl.zfaw79
Anopheles claviger	0.66027	52.212254	Unclassified	GBIF: https://doi.org/10.15468/dl.zfaw79
Anopheles claviger	0.664978	52.217543	Unclassified	GBIF: https://doi.org/10.15468/dl.zfaw79

Anopheles claviger	0.677379	52.216813	Unclassified	GBIF: https://doi.org/10.15468/dl.zfaw79
Anopheles claviger	0.71	51.396	Unclassified	GBIF: https://doi.org/10.15468/dl.zfaw79
Anopheles claviger	0.784	51.377	Unclassified	GBIF: https://doi.org/10.15468/dl.zfaw79
Anopheles claviger	0.789	51.384	Unclassified	GBIF: https://doi.org/10.15468/dl.zfaw79
Anopheles claviger	0.79	51.37	Unclassified	GBIF: https://doi.org/10.15468/dl.zfaw79
Anopheles claviger	0.7909	51.3743	Unclassified	GBIF: https://doi.org/10.15468/dl.zfaw79
Anopheles claviger	0.820565	51.387417	Unclassified	GBIF: https://doi.org/10.15468/dl.zfaw79
Anopheles claviger	0.866711	52.428196	Unclassified	GBIF: https://doi.org/10.15468/dl.zfaw79
Anopheles claviger	0.889	51.343	Unclassified	GBIF: https://doi.org/10.15468/dl.zfaw79
Anopheles claviger	1.209972	52.828121	Unclassified	GBIF: https://doi.org/10.15468/dl.zfaw79
Anopheles claviger	1.217502	52.748191	Unclassified	GBIF: https://doi.org/10.15468/dl.zfaw79
Anopheles claviger	1.254408	52.906051	Unclassified	GBIF: https://doi.org/10.15468/dl.zfaw79
Anopheles claviger	1.408374	52.612129	Unclassified	GBIF: https://doi.org/10.15468/dl.zfaw79
Anopheles claviger	1.453154	52.599139	Unclassified	GBIF: https://doi.org/10.15468/dl.zfaw79
Anopheles claviger	1.468096	52.591956	Unclassified	GBIF: https://doi.org/10.15468/dl.zfaw79
Anopheles claviger	1.475207	52.681688	Unclassified	GBIF: https://doi.org/10.15468/dl.zfaw79
Anopheles claviger	1.487864	52.682665	Unclassified	GBIF: https://doi.org/10.15468/dl.zfaw79
Anopheles claviger	1.512933	52.736788	Unclassified	GBIF: https://doi.org/10.15468/dl.zfaw79
Anopheles claviger	1.514523	52.756528	Unclassified	GBIF: https://doi.org/10.15468/dl.zfaw79
Anopheles claviger	1.61667	52.76667	Unclassified	GBIF: https://doi.org/10.15468/dl.zfaw79
Anopheles claviger	1.65319	52.72194	Unclassified	GBIF: https://doi.org/10.15468/dl.zfaw79
Anopheles claviger	1.90297	52.62567	Unclassified	GBIF: https://doi.org/10.15468/dl.zfaw79
Anopheles claviger	2.355411	50.752045	Unclassified	GBIF: https://doi.org/10.15468/dl.zfaw79
Anopheles claviger	2.384766	28.155094	Unclassified	GBIF: https://doi.org/10.15468/dl.zfaw79
Anopheles claviger	2.589614	51.094682	Unclassified	GBIF: https://doi.org/10.15468/dl.zfaw79
Anopheles claviger	2.607135	51.11332	Unclassified	GBIF: https://doi.org/10.15468/dl.zfaw79
Anopheles claviger	2.65145	50.971851	Unclassified	GBIF: https://doi.org/10.15468/dl.zfaw79
Anopheles claviger	2.65461	50.905441	Unclassified	GBIF: https://doi.org/10.15468/dl.zfaw79
Anopheles claviger	2.65741	50.967541	Unclassified	GBIF: https://doi.org/10.15468/dl.zfaw79
Anopheles claviger	2.69963	50.921501	Unclassified	GBIF: https://doi.org/10.15468/dl.zfaw79
Anopheles claviger	2.70109	51.095711	Unclassified	GBIF: https://doi.org/10.15468/dl.zfaw79
Anopheles claviger	2.72965	51.154789	Unclassified	GBIF: https://doi.org/10.15468/dl.zfaw79
Anopheles claviger	2.73007	51.10223	Unclassified	GBIF: https://doi.org/10.15468/dl.zfaw79
Anopheles claviger	2.736328	27.922369	Unclassified	GBIF: https://doi.org/10.15468/dl.zfaw79
Anopheles claviger	2.74015	51.053539	Unclassified	GBIF: https://doi.org/10.15468/dl.zfaw79
Anopheles claviger	2.74068	51.041939	Unclassified	GBIF: https://doi.org/10.15468/dl.zfaw79
Anopheles claviger	2.77039	51.15683	Unclassified	GBIF: https://doi.org/10.15468/dl.zfaw79
Anopheles claviger	2.78676	50.812061	Unclassified	GBIF: https://doi.org/10.15468/dl.zfaw79
Anopheles claviger	2.80801	51.065411	Unclassified	GBIF: https://doi.org/10.15468/dl.zfaw79
Anopheles claviger	2.82524	51.151051	Unclassified	GBIF: https://doi.org/10.15468/dl.zfaw79
Anopheles claviger	2.85481	50.72364	Unclassified	GBIF: https://doi.org/10.15468/dl.zfaw79
Anopheles claviger	2.85957	50.985901	Unclassified	GBIF: https://doi.org/10.15468/dl.zfaw79

Anopheles claviger	2.86	50.87	Unclassified	GBIF: https://doi.org/10.15468/dl.zfaw79
Anopheles claviger	2.861	50.868	Unclassified	GBIF: https://doi.org/10.15468/dl.zfaw79
Anopheles claviger	2.8611	50.868038	Unclassified	GBIF: https://doi.org/10.15468/dl.zfaw79
Anopheles claviger	2.8654	50.887241	Unclassified	GBIF: https://doi.org/10.15468/dl.zfaw79
Anopheles claviger	2.86668	51.20387	Unclassified	GBIF: https://doi.org/10.15468/dl.zfaw79
Anopheles claviger	2.87125	51.040569	Unclassified	GBIF: https://doi.org/10.15468/dl.zfaw79
Anopheles claviger	2.88284	51.104881	Unclassified	GBIF: https://doi.org/10.15468/dl.zfaw79
Anopheles claviger	2.89304	51.049912	Unclassified	GBIF: https://doi.org/10.15468/dl.zfaw79
Anopheles claviger	2.91655	50.979752	Unclassified	GBIF: https://doi.org/10.15468/dl.zfaw79
Anopheles claviger	2.97443	51.033428	Unclassified	GBIF: https://doi.org/10.15468/dl.zfaw79
Anopheles claviger	2.98186	51.226212	Unclassified	GBIF: https://doi.org/10.15468/dl.zfaw79
Anopheles claviger	2.99277	50.78923	Unclassified	GBIF: https://doi.org/10.15468/dl.zfaw79
Anopheles claviger	3	28	Unclassified	GBIF: https://doi.org/10.15468/dl.zfaw79
Anopheles claviger	3.017715	36.789885	Unclassified	GBIF: https://doi.org/10.15468/dl.zfaw79
Anopheles claviger	3.02395	42.47816	Unclassified	GBIF: https://doi.org/10.15468/dl.zfaw79
Anopheles claviger	3.02922	50.81094	Unclassified	GBIF: https://doi.org/10.15468/dl.zfaw79
Anopheles claviger	3.0886	51.01619	Unclassified	GBIF: https://doi.org/10.15468/dl.zfaw79
Anopheles claviger	3.107566	51.293774	Unclassified	GBIF: https://doi.org/10.15468/dl.zfaw79
Anopheles claviger	3.11435	50.908199	Unclassified	GBIF: https://doi.org/10.15468/dl.zfaw79
Anopheles claviger	3.12744	51.278099	Unclassified	GBIF: https://doi.org/10.15468/dl.zfaw79
Anopheles claviger	3.13092	51.27824	Unclassified	GBIF: https://doi.org/10.15468/dl.zfaw79
Anopheles claviger	3.132	51.311	Unclassified	GBIF: https://doi.org/10.15468/dl.zfaw79
Anopheles claviger	3.15852	50.78125	Unclassified	GBIF: https://doi.org/10.15468/dl.zfaw79
Anopheles claviger	3.166291	51.316528	Unclassified	GBIF: https://doi.org/10.15468/dl.zfaw79
Anopheles claviger	3.16872	51.326191	Unclassified	GBIF: https://doi.org/10.15468/dl.zfaw79
Anopheles claviger	3.1699	50.76562	Unclassified	GBIF: https://doi.org/10.15468/dl.zfaw79
Anopheles claviger	3.17945	51.338646	Unclassified	GBIF: https://doi.org/10.15468/dl.zfaw79
Anopheles claviger	3.2036	51.33791	Unclassified	GBIF: https://doi.org/10.15468/dl.zfaw79
Anopheles claviger	3.20429	51.328185	Unclassified	GBIF: https://doi.org/10.15468/dl.zfaw79
Anopheles claviger	3.24082	51.232121	Unclassified	GBIF: https://doi.org/10.15468/dl.zfaw79
Anopheles claviger	3.26107	51.271969	Unclassified	GBIF: https://doi.org/10.15468/dl.zfaw79
Anopheles claviger	3.27088	50.833752	Unclassified	GBIF: https://doi.org/10.15468/dl.zfaw79
Anopheles claviger	3.32158	50.813431	Unclassified	GBIF: https://doi.org/10.15468/dl.zfaw79
Anopheles claviger	3.34593	51.12664	Unclassified	GBIF: https://doi.org/10.15468/dl.zfaw79
Anopheles claviger	3.35054	51.359169	Unclassified	GBIF: https://doi.org/10.15468/dl.zfaw79
Anopheles claviger	3.35294	51.35952	Unclassified	GBIF: https://doi.org/10.15468/dl.zfaw79
Anopheles claviger	3.374444	51.072778	Unclassified	GBIF: https://doi.org/10.15468/dl.zfaw79
Anopheles claviger	3.39798	50.6404	Unclassified	GBIF: https://doi.org/10.15468/dl.zfaw79
Anopheles claviger	3.4	51.5	Unclassified	GBIF: https://doi.org/10.15468/dl.zfaw79
Anopheles claviger	3.40211	50.6558	Unclassified	GBIF: https://doi.org/10.15468/dl.zfaw79
Anopheles claviger	3.46793	50.796001	Unclassified	GBIF: https://doi.org/10.15468/dl.zfaw79
Anopheles claviger	3.51012	50.774681	Unclassified	GBIF: https://doi.org/10.15468/dl.zfaw79

Anopheles claviger	3.51232	50.64975	Unclassified	GBIF: https://doi.org/10.15468/dl.zfaw79
Anopheles claviger	3.52686	50.50441	Unclassified	GBIF: https://doi.org/10.15468/dl.zfaw79
Anopheles claviger	3.54632	50.919399	Unclassified	GBIF: https://doi.org/10.15468/dl.zfaw79
Anopheles claviger	3.55143	51.098969	Unclassified	GBIF: https://doi.org/10.15468/dl.zfaw79
Anopheles claviger	3.607737	51.157362	Unclassified	GBIF: https://doi.org/10.15468/dl.zfaw79
Anopheles claviger	3.61982	50.962921	Unclassified	GBIF: https://doi.org/10.15468/dl.zfaw79
Anopheles claviger	3.621299	51.155676	Unclassified	GBIF: https://doi.org/10.15468/dl.zfaw79
Anopheles claviger	3.62722	51.134159	Unclassified	GBIF: https://doi.org/10.15468/dl.zfaw79
Anopheles claviger	3.641383	51.087742	Unclassified	GBIF: https://doi.org/10.15468/dl.zfaw79
Anopheles claviger	3.641769	51.087876	Unclassified	GBIF: https://doi.org/10.15468/dl.zfaw79
Anopheles claviger	3.641769	51.087769	Unclassified	GBIF: https://doi.org/10.15468/dl.zfaw79
Anopheles claviger	3.642027	51.088011	Unclassified	GBIF: https://doi.org/10.15468/dl.zfaw79
Anopheles claviger	3.65422	51.14027	Unclassified	GBIF: https://doi.org/10.15468/dl.zfaw79
Anopheles claviger	3.664257	51.050824	Unclassified	GBIF: https://doi.org/10.15468/dl.zfaw79
Anopheles claviger	3.66617	51.050571	Unclassified	GBIF: https://doi.org/10.15468/dl.zfaw79
Anopheles claviger	3.66664	51.066139	Unclassified	GBIF: https://doi.org/10.15468/dl.zfaw79
Anopheles claviger	3.677913	51.067052	Unclassified	GBIF: https://doi.org/10.15468/dl.zfaw79
Anopheles claviger	3.69062	50.44035	Unclassified	GBIF: https://doi.org/10.15468/dl.zfaw79
Anopheles claviger	3.70604	50.977139	Unclassified	GBIF: https://doi.org/10.15468/dl.zfaw79
Anopheles claviger	3.70683	51.189941	Unclassified	GBIF: https://doi.org/10.15468/dl.zfaw79
Anopheles claviger	3.722536	51.056364	Unclassified	GBIF: https://doi.org/10.15468/dl.zfaw79
Anopheles claviger	3.723995	51.054695	Unclassified	GBIF: https://doi.org/10.15468/dl.zfaw79
Anopheles claviger	3.72614	50.544689	Unclassified	GBIF: https://doi.org/10.15468/dl.zfaw79
Anopheles claviger	3.749229	51.062153	Unclassified	GBIF: https://doi.org/10.15468/dl.zfaw79
Anopheles claviger	3.749229	51.062207	Unclassified	GBIF: https://doi.org/10.15468/dl.zfaw79
Anopheles claviger	3.749266	51.066617	Unclassified	GBIF: https://doi.org/10.15468/dl.zfaw79
Anopheles claviger	3.749658	51.062531	Unclassified	GBIF: https://doi.org/10.15468/dl.zfaw79
Anopheles claviger	3.75279	50.808571	Unclassified	GBIF: https://doi.org/10.15468/dl.zfaw79
Anopheles claviger	3.75788	51.24929	Unclassified	GBIF: https://doi.org/10.15468/dl.zfaw79
Anopheles claviger	3.75898	51.259048	Unclassified	GBIF: https://doi.org/10.15468/dl.zfaw79
Anopheles claviger	3.76342	51.221561	Unclassified	GBIF: https://doi.org/10.15468/dl.zfaw79
Anopheles claviger	3.78058	51.15981	Unclassified	GBIF: https://doi.org/10.15468/dl.zfaw79
Anopheles claviger	3.795664	51.001428	Unclassified	GBIF: https://doi.org/10.15468/dl.zfaw79
Anopheles claviger	3.795921	51.000618	Unclassified	GBIF: https://doi.org/10.15468/dl.zfaw79
Anopheles claviger	3.798239	51.00186	Unclassified	GBIF: https://doi.org/10.15468/dl.zfaw79
Anopheles claviger	3.800299	51.056057	Unclassified	GBIF: https://doi.org/10.15468/dl.zfaw79
Anopheles claviger	3.80459	51.030802	Unclassified	GBIF: https://doi.org/10.15468/dl.zfaw79
Anopheles claviger	3.819823	51.02118	Unclassified	GBIF: https://doi.org/10.15468/dl.zfaw79
Anopheles claviger	3.820617	51.066137	Unclassified	GBIF: https://doi.org/10.15468/dl.zfaw79
Anopheles claviger	3.82418	51.085079	Unclassified	GBIF: https://doi.org/10.15468/dl.zfaw79
Anopheles claviger	3.83608	51.11195	Unclassified	GBIF: https://doi.org/10.15468/dl.zfaw79
Anopheles claviger	3.83611	51.11228	Unclassified	GBIF: https://doi.org/10.15468/dl.zfaw79

Anopheles claviger	3.8808	50.674721	Unclassified	GBIF: https://doi.org/10.15468/dl.zfaw79
Anopheles claviger	3.8835	50.586349	Unclassified	GBIF: https://doi.org/10.15468/dl.zfaw79
Anopheles claviger	3.89067	51.105679	Unclassified	GBIF: https://doi.org/10.15468/dl.zfaw79
Anopheles claviger	3.89326	51.17263	Unclassified	GBIF: https://doi.org/10.15468/dl.zfaw79
Anopheles claviger	3.92336	50.698738	Unclassified	GBIF: https://doi.org/10.15468/dl.zfaw79
Anopheles claviger	3.92491	50.41203	Unclassified	GBIF: https://doi.org/10.15468/dl.zfaw79
Anopheles claviger	3.92787	51.070042	Unclassified	GBIF: https://doi.org/10.15468/dl.zfaw79
Anopheles claviger	3.94216	51.18763	Unclassified	GBIF: https://doi.org/10.15468/dl.zfaw79
Anopheles claviger	3.947327	51.046831	Unclassified	GBIF: https://doi.org/10.15468/dl.zfaw79
Anopheles claviger	3.949129	51.046075	Unclassified	GBIF: https://doi.org/10.15468/dl.zfaw79
Anopheles claviger	3.95	51.11	Unclassified	GBIF: https://doi.org/10.15468/dl.zfaw79
Anopheles claviger	3.952	51.107	Unclassified	GBIF: https://doi.org/10.15468/dl.zfaw79
Anopheles claviger	3.9537	51.183769	Unclassified	GBIF: https://doi.org/10.15468/dl.zfaw79
Anopheles claviger	3.96275	50.8965	Unclassified	GBIF: https://doi.org/10.15468/dl.zfaw79
Anopheles claviger	3.963313	51.065049	Unclassified	GBIF: https://doi.org/10.15468/dl.zfaw79
Anopheles claviger	3.98287	51.081501	Unclassified	GBIF: https://doi.org/10.15468/dl.zfaw79
Anopheles claviger	3.9979	51.0555	Unclassified	GBIF: https://doi.org/10.15468/dl.zfaw79
Anopheles claviger	4.01675	51.103222	Unclassified	GBIF: https://doi.org/10.15468/dl.zfaw79
Anopheles claviger	4.04	50.69	Unclassified	GBIF: https://doi.org/10.15468/dl.zfaw79
Anopheles claviger	4.042	50.693	Unclassified	GBIF: https://doi.org/10.15468/dl.zfaw79
Anopheles claviger	4.04224	50.69278	Unclassified	GBIF: https://doi.org/10.15468/dl.zfaw79
Anopheles claviger	4.0506	51.12513	Unclassified	GBIF: https://doi.org/10.15468/dl.zfaw79
Anopheles claviger	4.06593	51.042309	Unclassified	GBIF: https://doi.org/10.15468/dl.zfaw79
Anopheles claviger	4.06978	50.858452	Unclassified	GBIF: https://doi.org/10.15468/dl.zfaw79
Anopheles claviger	4.070881	51.896405	Unclassified	GBIF: https://doi.org/10.15468/dl.zfaw79
Anopheles claviger	4.1383	51.149269	Unclassified	GBIF: https://doi.org/10.15468/dl.zfaw79
Anopheles claviger	4.14315	51.223381	Unclassified	GBIF: https://doi.org/10.15468/dl.zfaw79
Anopheles claviger	4.16294	51.175098	Unclassified	GBIF: https://doi.org/10.15468/dl.zfaw79
Anopheles claviger	4.16423	51.171139	Unclassified	GBIF: https://doi.org/10.15468/dl.zfaw79
Anopheles claviger	4.17235	50.606861	Unclassified	GBIF: https://doi.org/10.15468/dl.zfaw79
Anopheles claviger	4.1848	51.203041	Unclassified	GBIF: https://doi.org/10.15468/dl.zfaw79
Anopheles claviger	4.1935	51.21351	Unclassified	GBIF: https://doi.org/10.15468/dl.zfaw79
Anopheles claviger	4.19417	51.10524	Unclassified	GBIF: https://doi.org/10.15468/dl.zfaw79
Anopheles claviger	4.19777	50.89613	Unclassified	GBIF: https://doi.org/10.15468/dl.zfaw79
Anopheles claviger	4.20551	51.06319	Unclassified	GBIF: https://doi.org/10.15468/dl.zfaw79
Anopheles claviger	4.20593	50.802399	Unclassified	GBIF: https://doi.org/10.15468/dl.zfaw79
Anopheles claviger	4.21592	51.175461	Unclassified	GBIF: https://doi.org/10.15468/dl.zfaw79
Anopheles claviger	4.21772	51.25156	Unclassified	GBIF: https://doi.org/10.15468/dl.zfaw79
Anopheles claviger	4.2288	51.320259	Unclassified	GBIF: https://doi.org/10.15468/dl.zfaw79
Anopheles claviger	4.25849	51.06432	Unclassified	GBIF: https://doi.org/10.15468/dl.zfaw79
Anopheles claviger	4.26662	50.987331	Unclassified	GBIF: https://doi.org/10.15468/dl.zfaw79
Anopheles claviger	4.2674	51.115528	Unclassified	GBIF: https://doi.org/10.15468/dl.zfaw79

Anopheles claviger	4.2765	51.27883	Unclassified	GBIF: https://doi.org/10.15468/dl.zfaw79
Anopheles claviger	4.29414	51.190632	Unclassified	GBIF: https://doi.org/10.15468/dl.zfaw79
Anopheles claviger	4.29872	51.268799	Unclassified	GBIF: https://doi.org/10.15468/dl.zfaw79
Anopheles claviger	4.302015	51.457668	Unclassified	GBIF: https://doi.org/10.15468/dl.zfaw79
Anopheles claviger	4.30696	50.031528	Unclassified	GBIF: https://doi.org/10.15468/dl.zfaw79
Anopheles claviger	4.31861	50.717999	Unclassified	GBIF: https://doi.org/10.15468/dl.zfaw79
Anopheles claviger	4.345138	51.329414	Unclassified	GBIF: https://doi.org/10.15468/dl.zfaw79
Anopheles claviger	4.34853	50.079689	Unclassified	GBIF: https://doi.org/10.15468/dl.zfaw79
Anopheles claviger	4.34877	51.236321	Unclassified	GBIF: https://doi.org/10.15468/dl.zfaw79
Anopheles claviger	4.35	52.15	Unclassified	GBIF: https://doi.org/10.15468/dl.zfaw79
Anopheles claviger	4.36024	51.26369	Unclassified	GBIF: https://doi.org/10.15468/dl.zfaw79
Anopheles claviger	4.36981	51.165871	Unclassified	GBIF: https://doi.org/10.15468/dl.zfaw79
Anopheles claviger	4.37552	51.23079	Unclassified	GBIF: https://doi.org/10.15468/dl.zfaw79
Anopheles claviger	4.380262	50.814067	Unclassified	GBIF: https://doi.org/10.15468/dl.zfaw79
Anopheles claviger	4.383314	50.791038	Unclassified	GBIF: https://doi.org/10.15468/dl.zfaw79
Anopheles claviger	4.384642	50.835989	Unclassified	GBIF: https://doi.org/10.15468/dl.zfaw79
Anopheles claviger	4.38988	50.431431	Unclassified	GBIF: https://doi.org/10.15468/dl.zfaw79
Anopheles claviger	4.39225	51.032398	Unclassified	GBIF: https://doi.org/10.15468/dl.zfaw79
Anopheles claviger	4.4	52.1	Unclassified	GBIF: https://doi.org/10.15468/dl.zfaw79
Anopheles claviger	4.403774	50.809406	Unclassified	GBIF: https://doi.org/10.15468/dl.zfaw79
Anopheles claviger	4.42332	51.029701	Unclassified	GBIF: https://doi.org/10.15468/dl.zfaw79
Anopheles claviger	4.448678	50.610374	Unclassified	GBIF: https://doi.org/10.15468/dl.zfaw79
Anopheles claviger	4.44907	51.115051	Unclassified	GBIF: https://doi.org/10.15468/dl.zfaw79
Anopheles claviger	4.452836	50.745225	Unclassified	GBIF: https://doi.org/10.15468/dl.zfaw79
Anopheles claviger	4.462611	50.610138	Unclassified	GBIF: https://doi.org/10.15468/dl.zfaw79
Anopheles claviger	4.466111	51.168889	Unclassified	GBIF: https://doi.org/10.15468/dl.zfaw79
Anopheles claviger	4.47338	51.24585	Unclassified	GBIF: https://doi.org/10.15468/dl.zfaw79
Anopheles claviger	4.483333	50.733333	Unclassified	GBIF: https://doi.org/10.15468/dl.zfaw79
Anopheles claviger	4.49128	50.09161	Unclassified	GBIF: https://doi.org/10.15468/dl.zfaw79
Anopheles claviger	4.5	52.15	Unclassified	GBIF: https://doi.org/10.15468/dl.zfaw79
Anopheles claviger	4.5	52.05	Unclassified	GBIF: https://doi.org/10.15468/dl.zfaw79
Anopheles claviger	4.50526	51.227379	Unclassified	GBIF: https://doi.org/10.15468/dl.zfaw79
Anopheles claviger	4.507212	51.055363	Unclassified	GBIF: https://doi.org/10.15468/dl.zfaw79
Anopheles claviger	4.51056	51.056252	Unclassified	GBIF: https://doi.org/10.15468/dl.zfaw79
Anopheles claviger	4.51305	51.000481	Unclassified	GBIF: https://doi.org/10.15468/dl.zfaw79
Anopheles claviger	4.51324	51.138672	Unclassified	GBIF: https://doi.org/10.15468/dl.zfaw79
Anopheles claviger	4.51494	49.985889	Unclassified	GBIF: https://doi.org/10.15468/dl.zfaw79
Anopheles claviger	4.55701	50.932209	Unclassified	GBIF: https://doi.org/10.15468/dl.zfaw79
Anopheles claviger	4.56	50.691	Unclassified	GBIF: https://doi.org/10.15468/dl.zfaw79
Anopheles claviger	4.56	50.69	Unclassified	GBIF: https://doi.org/10.15468/dl.zfaw79
Anopheles claviger	4.56994	50.947449	Unclassified	GBIF: https://doi.org/10.15468/dl.zfaw79
Anopheles claviger	4.58032	50.534939	Unclassified	GBIF: https://doi.org/10.15468/dl.zfaw79

Anopheles claviger	4.59191	50.76918	Unclassified	GBIF: https://doi.org/10.15468/dl.zfaw79
Anopheles claviger	4.60218	51.22261	Unclassified	GBIF: https://doi.org/10.15468/dl.zfaw79
Anopheles claviger	4.63093	51.384941	Unclassified	GBIF: https://doi.org/10.15468/dl.zfaw79
Anopheles claviger	4.631667	52.660833	Unclassified	GBIF: https://doi.org/10.15468/dl.zfaw79
Anopheles claviger	4.643676	52.621369	Unclassified	GBIF: https://doi.org/10.15468/dl.zfaw79
Anopheles claviger	4.67661	51.068588	Unclassified	GBIF: https://doi.org/10.15468/dl.zfaw79
Anopheles claviger	4.696	50.519	Unclassified	GBIF: https://doi.org/10.15468/dl.zfaw79
Anopheles claviger	4.7177	51.312241	Unclassified	GBIF: https://doi.org/10.15468/dl.zfaw79
Anopheles claviger	4.727929	50.516626	Unclassified	GBIF: https://doi.org/10.15468/dl.zfaw79
Anopheles claviger	4.728668	51.616443	Unclassified	GBIF: https://doi.org/10.15468/dl.zfaw79
Anopheles claviger	4.732051	51.571167	Unclassified	GBIF: https://doi.org/10.15468/dl.zfaw79
Anopheles claviger	4.75	52.45	Unclassified	GBIF: https://doi.org/10.15468/dl.zfaw79
Anopheles claviger	4.75044	51.45504	Unclassified	GBIF: https://doi.org/10.15468/dl.zfaw79
Anopheles claviger	4.76747	50.889999	Unclassified	GBIF: https://doi.org/10.15468/dl.zfaw79
Anopheles claviger	4.80215	50.998039	Unclassified	GBIF: https://doi.org/10.15468/dl.zfaw79
Anopheles claviger	4.82815	51.510003	Unclassified	GBIF: https://doi.org/10.15468/dl.zfaw79
Anopheles claviger	4.829792	51.481786	Unclassified	GBIF: https://doi.org/10.15468/dl.zfaw79
Anopheles claviger	4.843645	51.527245	Unclassified	GBIF: https://doi.org/10.15468/dl.zfaw79
Anopheles claviger	4.84562	50.975498	Unclassified	GBIF: https://doi.org/10.15468/dl.zfaw79
Anopheles claviger	4.84673	50.541431	Unclassified	GBIF: https://doi.org/10.15468/dl.zfaw79
Anopheles claviger	4.85011	50.85368	Unclassified	GBIF: https://doi.org/10.15468/dl.zfaw79
Anopheles claviger	4.851911	51.510636	Unclassified	GBIF: https://doi.org/10.15468/dl.zfaw79
Anopheles claviger	4.85388	50.919128	Unclassified	GBIF: https://doi.org/10.15468/dl.zfaw79
Anopheles claviger	4.854655	51.510037	Unclassified	GBIF: https://doi.org/10.15468/dl.zfaw79
Anopheles claviger	4.86543	51.25943	Unclassified	GBIF: https://doi.org/10.15468/dl.zfaw79
Anopheles claviger	4.870661	51.407097	Unclassified	GBIF: https://doi.org/10.15468/dl.zfaw79
Anopheles claviger	4.87515	50.53159	Unclassified	GBIF: https://doi.org/10.15468/dl.zfaw79
Anopheles claviger	4.88963	51.001789	Unclassified	GBIF: https://doi.org/10.15468/dl.zfaw79
Anopheles claviger	4.89	50.037	Unclassified	GBIF: https://doi.org/10.15468/dl.zfaw79
Anopheles claviger	4.89	50.04	Unclassified	GBIF: https://doi.org/10.15468/dl.zfaw79
Anopheles claviger	4.89048	50.03664	Unclassified	GBIF: https://doi.org/10.15468/dl.zfaw79
Anopheles claviger	4.89712	50.938229	Unclassified	GBIF: https://doi.org/10.15468/dl.zfaw79
Anopheles claviger	4.9	52.45	Unclassified	GBIF: https://doi.org/10.15468/dl.zfaw79
Anopheles claviger	4.92196	50.854679	Unclassified	GBIF: https://doi.org/10.15468/dl.zfaw79
Anopheles claviger	4.93018	51.196499	Unclassified	GBIF: https://doi.org/10.15468/dl.zfaw79
Anopheles claviger	4.940385	51.713181	Unclassified	GBIF: https://doi.org/10.15468/dl.zfaw79
Anopheles claviger	4.94829	51.20118	Unclassified	GBIF: https://doi.org/10.15468/dl.zfaw79
Anopheles claviger	4.95	52.45	Unclassified	GBIF: https://doi.org/10.15468/dl.zfaw79
Anopheles claviger	4.95421	51.245739	Unclassified	GBIF: https://doi.org/10.15468/dl.zfaw79
Anopheles claviger	4.96155	51.344261	Unclassified	GBIF: https://doi.org/10.15468/dl.zfaw79
Anopheles claviger	4.9671	51.358158	Unclassified	GBIF: https://doi.org/10.15468/dl.zfaw79
Anopheles claviger	4.98179	50.775188	Unclassified	GBIF: https://doi.org/10.15468/dl.zfaw79

Anopheles claviger	4.99479	51.42572	Unclassified	GBIF: https://doi.org/10.15468/dl.zfaw79
Anopheles claviger	4.99526	50.750469	Unclassified	GBIF: https://doi.org/10.15468/dl.zfaw79
Anopheles claviger	5	52.1	Unclassified	GBIF: https://doi.org/10.15468/dl.zfaw79
Anopheles claviger	5.00904	50.997269	Unclassified	GBIF: https://doi.org/10.15468/dl.zfaw79
Anopheles claviger	5.04329	51.41589	Unclassified	GBIF: https://doi.org/10.15468/dl.zfaw79
Anopheles claviger	5.045001	50.338611	Unclassified	GBIF: https://doi.org/10.15468/dl.zfaw79
Anopheles claviger	5.04547	50.33894	Unclassified	GBIF: https://doi.org/10.15468/dl.zfaw79
Anopheles claviger	5.04574	50.3392	Unclassified	GBIF: https://doi.org/10.15468/dl.zfaw79
Anopheles claviger	5.05	52.85	Unclassified	GBIF: https://doi.org/10.15468/dl.zfaw79
Anopheles claviger	5.063758	51.507972	Unclassified	GBIF: https://doi.org/10.15468/dl.zfaw79
Anopheles claviger	5.06892	51.450142	Unclassified	GBIF: https://doi.org/10.15468/dl.zfaw79
Anopheles claviger	5.071389	50.335833	Unclassified	GBIF: https://doi.org/10.15468/dl.zfaw79
Anopheles claviger	5.07164	50.33586	Unclassified	GBIF: https://doi.org/10.15468/dl.zfaw79
Anopheles claviger	5.106667	52.7675	Unclassified	GBIF: https://doi.org/10.15468/dl.zfaw79
Anopheles claviger	5.13359	50.94508	Unclassified	GBIF: https://doi.org/10.15468/dl.zfaw79
Anopheles claviger	5.14754	50.409809	Unclassified	GBIF: https://doi.org/10.15468/dl.zfaw79
Anopheles claviger	5.157614	51.910312	Unclassified	GBIF: https://doi.org/10.15468/dl.zfaw79
Anopheles claviger	5.16745	50.991829	Unclassified	GBIF: https://doi.org/10.15468/dl.zfaw79
Anopheles claviger	5.17407	51.15456	Unclassified	GBIF: https://doi.org/10.15468/dl.zfaw79
Anopheles claviger	5.17456	50.32671	Unclassified	GBIF: https://doi.org/10.15468/dl.zfaw79
Anopheles claviger	5.18043	51.304001	Unclassified	GBIF: https://doi.org/10.15468/dl.zfaw79
Anopheles claviger	5.186295	51.273408	Unclassified	GBIF: https://doi.org/10.15468/dl.zfaw79
Anopheles claviger	5.191059	51.289381	Unclassified	GBIF: https://doi.org/10.15468/dl.zfaw79
Anopheles claviger	5.191271	51.289262	Unclassified	GBIF: https://doi.org/10.15468/dl.zfaw79
Anopheles claviger	5.2	52.4	Unclassified	GBIF: https://doi.org/10.15468/dl.zfaw79
Anopheles claviger	5.210526	51.703698	Unclassified	GBIF: https://doi.org/10.15468/dl.zfaw79
Anopheles claviger	5.219	50.494	Unclassified	GBIF: https://doi.org/10.15468/dl.zfaw79
Anopheles claviger	5.22	50.49	Unclassified	GBIF: https://doi.org/10.15468/dl.zfaw79
Anopheles claviger	5.238264	51.578804	Unclassified	GBIF: https://doi.org/10.15468/dl.zfaw79
Anopheles claviger	5.23999	50.841702	Unclassified	GBIF: https://doi.org/10.15468/dl.zfaw79
Anopheles claviger	5.252241	51.487272	Unclassified	GBIF: https://doi.org/10.15468/dl.zfaw79
Anopheles claviger	5.25975	50.58533	Unclassified	GBIF: https://doi.org/10.15468/dl.zfaw79
Anopheles claviger	5.266012	49.967696	Unclassified	GBIF: https://doi.org/10.15468/dl.zfaw79
Anopheles claviger	5.272691	51.856713	Unclassified	GBIF: https://doi.org/10.15468/dl.zfaw79
Anopheles claviger	5.29123	50.781521	Unclassified	GBIF: https://doi.org/10.15468/dl.zfaw79
Anopheles claviger	5.293109	50.019611	Unclassified	GBIF: https://doi.org/10.15468/dl.zfaw79
Anopheles claviger	5.3	51.6	Unclassified	GBIF: https://doi.org/10.15468/dl.zfaw79
Anopheles claviger	5.31507	50.950611	Unclassified	GBIF: https://doi.org/10.15468/dl.zfaw79
Anopheles claviger	5.331307	51.279689	Unclassified	GBIF: https://doi.org/10.15468/dl.zfaw79
Anopheles claviger	5.367234	51.554448	Unclassified	GBIF: https://doi.org/10.15468/dl.zfaw79
Anopheles claviger	5.37005	50.430809	Unclassified	GBIF: https://doi.org/10.15468/dl.zfaw79
Anopheles claviger	5.406042	52.258875	Unclassified	GBIF: https://doi.org/10.15468/dl.zfaw79

Anopheles claviger	5.41562	50.9347	Unclassified	GBIF: https://doi.org/10.15468/dl.zfaw79
Anopheles claviger	5.41704	51.249722	Unclassified	GBIF: https://doi.org/10.15468/dl.zfaw79
Anopheles claviger	5.42833	50.55122	Unclassified	GBIF: https://doi.org/10.15468/dl.zfaw79
Anopheles claviger	5.431648	51.662106	Unclassified	GBIF: https://doi.org/10.15468/dl.zfaw79
Anopheles claviger	5.48693	51.080631	Unclassified	GBIF: https://doi.org/10.15468/dl.zfaw79
Anopheles claviger	5.49848	51.19516	Unclassified	GBIF: https://doi.org/10.15468/dl.zfaw79
Anopheles claviger	5.5	52	Unclassified	GBIF: https://doi.org/10.15468/dl.zfaw79
Anopheles claviger	5.513855	52.191867	Unclassified	GBIF: https://doi.org/10.15468/dl.zfaw79
Anopheles claviger	5.52	50.21	Unclassified	GBIF: https://doi.org/10.15468/dl.zfaw79
Anopheles claviger	5.52043	50.55032	Unclassified	GBIF: https://doi.org/10.15468/dl.zfaw79
Anopheles claviger	5.524	50.215	Unclassified	GBIF: https://doi.org/10.15468/dl.zfaw79
Anopheles claviger	5.52428	50.21492	Unclassified	GBIF: https://doi.org/10.15468/dl.zfaw79
Anopheles claviger	5.525863	49.602492	Unclassified	GBIF: https://doi.org/10.15468/dl.zfaw79
Anopheles claviger	5.533	49.568	Unclassified	GBIF: https://doi.org/10.15468/dl.zfaw79
Anopheles claviger	5.555685	51.273449	Unclassified	GBIF: https://doi.org/10.15468/dl.zfaw79
Anopheles claviger	5.567426	51.692348	Unclassified	GBIF: https://doi.org/10.15468/dl.zfaw79
Anopheles claviger	5.5784	50.590839	Unclassified	GBIF: https://doi.org/10.15468/dl.zfaw79
Anopheles claviger	5.57869	50.285149	Unclassified	GBIF: https://doi.org/10.15468/dl.zfaw79
Anopheles claviger	5.58333	50.583333	Unclassified	GBIF: https://doi.org/10.15468/dl.zfaw79
Anopheles claviger	5.583333	50.583333	Unclassified	GBIF: https://doi.org/10.15468/dl.zfaw79
Anopheles claviger	5.594991	49.600962	Unclassified	GBIF: https://doi.org/10.15468/dl.zfaw79
Anopheles claviger	5.598795	50.594179	Unclassified	GBIF: https://doi.org/10.15468/dl.zfaw79
Anopheles claviger	5.599402	52.159657	Unclassified	GBIF: https://doi.org/10.15468/dl.zfaw79
Anopheles claviger	5.599782	50.594534	Unclassified	GBIF: https://doi.org/10.15468/dl.zfaw79
Anopheles claviger	5.60019	50.594631	Unclassified	GBIF: https://doi.org/10.15468/dl.zfaw79
Anopheles claviger	5.600308	49.586939	Unclassified	GBIF: https://doi.org/10.15468/dl.zfaw79
Anopheles claviger	5.600426	50.595324	Unclassified	GBIF: https://doi.org/10.15468/dl.zfaw79
Anopheles claviger	5.604288	50.595075	Unclassified	GBIF: https://doi.org/10.15468/dl.zfaw79
Anopheles claviger	5.61205	51.00111	Unclassified	GBIF: https://doi.org/10.15468/dl.zfaw79
Anopheles claviger	5.618889	50.995	Unclassified	GBIF: https://doi.org/10.15468/dl.zfaw79
Anopheles claviger	5.619141	50.991833	Unclassified	GBIF: https://doi.org/10.15468/dl.zfaw79
Anopheles claviger	5.619444	50.987778	Unclassified	GBIF: https://doi.org/10.15468/dl.zfaw79
Anopheles claviger	5.626299	51.75687	Unclassified	GBIF: https://doi.org/10.15468/dl.zfaw79
Anopheles claviger	5.651684	51.330556	Unclassified	GBIF: https://doi.org/10.15468/dl.zfaw79
Anopheles claviger	5.659102	50.768922	Unclassified	GBIF: https://doi.org/10.15468/dl.zfaw79
Anopheles claviger	5.666945	50.758611	Unclassified	GBIF: https://doi.org/10.15468/dl.zfaw79
Anopheles claviger	5.67007	49.63533	Unclassified	GBIF: https://doi.org/10.15468/dl.zfaw79
Anopheles claviger	5.675229	51.621419	Unclassified	GBIF: https://doi.org/10.15468/dl.zfaw79
Anopheles claviger	5.68738	50.53413	Unclassified	GBIF: https://doi.org/10.15468/dl.zfaw79
Anopheles claviger	5.6891	51.01456	Unclassified	GBIF: https://doi.org/10.15468/dl.zfaw79
Anopheles claviger	5.69522	51.01628	Unclassified	GBIF: https://doi.org/10.15468/dl.zfaw79
Anopheles claviger	5.69702	50.603981	Unclassified	GBIF: https://doi.org/10.15468/dl.zfaw79

Anopheles claviger	5.697141	51.014011	Unclassified	GBIF: https://doi.org/10.15468/dl.zfaw79
Anopheles claviger	5.713044	51.490301	Unclassified	GBIF: https://doi.org/10.15468/dl.zfaw79
Anopheles claviger	5.71609	50.466282	Unclassified	GBIF: https://doi.org/10.15468/dl.zfaw79
Anopheles claviger	5.73625	49.723289	Unclassified	GBIF: https://doi.org/10.15468/dl.zfaw79
Anopheles claviger	5.73707	50.441731	Unclassified	GBIF: https://doi.org/10.15468/dl.zfaw79
Anopheles claviger	5.74493	51.136261	Unclassified	GBIF: https://doi.org/10.15468/dl.zfaw79
Anopheles claviger	5.752702	50.941506	Unclassified	GBIF: https://doi.org/10.15468/dl.zfaw79
Anopheles claviger	5.79171	49.664982	Unclassified	GBIF: https://doi.org/10.15468/dl.zfaw79
Anopheles claviger	5.80508	49.552422	Unclassified	GBIF: https://doi.org/10.15468/dl.zfaw79
Anopheles claviger	5.80532	49.551178	Unclassified	GBIF: https://doi.org/10.15468/dl.zfaw79
Anopheles claviger	5.81766	50.62735	Unclassified	GBIF: https://doi.org/10.15468/dl.zfaw79
Anopheles claviger	5.819679	51.241594	Unclassified	GBIF: https://doi.org/10.15468/dl.zfaw79
Anopheles claviger	5.82093	51.040867	Unclassified	GBIF: https://doi.org/10.15468/dl.zfaw79
Anopheles claviger	5.82689	45.82792	Unclassified	GBIF: https://doi.org/10.15468/dl.zfaw79
Anopheles claviger	5.8663	49.6657	Unclassified	GBIF: https://doi.org/10.15468/dl.zfaw79
Anopheles claviger	5.88244	50.516811	Unclassified	GBIF: https://doi.org/10.15468/dl.zfaw79
Anopheles claviger	5.889355	50.928274	Unclassified	GBIF: https://doi.org/10.15468/dl.zfaw79
Anopheles claviger	5.9	50.75	Unclassified	GBIF: https://doi.org/10.15468/dl.zfaw79
Anopheles claviger	5.9	50.9	Unclassified	GBIF: https://doi.org/10.15468/dl.zfaw79
Anopheles claviger	5.90309	58.797879	Unclassified	GBIF: https://doi.org/10.15468/dl.zfaw79
Anopheles claviger	5.915484	51.06764	Unclassified	GBIF: https://doi.org/10.15468/dl.zfaw79
Anopheles claviger	5.920039	50.40286	Unclassified	GBIF: https://doi.org/10.15468/dl.zfaw79
Anopheles claviger	5.922243	51.685979	Unclassified	GBIF: https://doi.org/10.15468/dl.zfaw79
Anopheles claviger	5.9302	49.9069	Unclassified	GBIF: https://doi.org/10.15468/dl.zfaw79
Anopheles claviger	5.9387	50.67429	Unclassified	GBIF: https://doi.org/10.15468/dl.zfaw79
Anopheles claviger	5.95	51.15	Unclassified	GBIF: https://doi.org/10.15468/dl.zfaw79
Anopheles claviger	5.952974	52.578129	Unclassified	GBIF: https://doi.org/10.15468/dl.zfaw79
Anopheles claviger	5.960879	51.755233	Unclassified	GBIF: https://doi.org/10.15468/dl.zfaw79
Anopheles claviger	5.961349	51.098362	Unclassified	GBIF: https://doi.org/10.15468/dl.zfaw79
Anopheles claviger	5.970929	50.962274	Unclassified	GBIF: https://doi.org/10.15468/dl.zfaw79
Anopheles claviger	5.971738	51.761642	Unclassified	GBIF: https://doi.org/10.15468/dl.zfaw79
Anopheles claviger	5.97433	45.55149	Unclassified	GBIF: https://doi.org/10.15468/dl.zfaw79
Anopheles claviger	5.983333	50.483333	Unclassified	GBIF: https://doi.org/10.15468/dl.zfaw79
Anopheles claviger	5.991353	51.49971	Unclassified	GBIF: https://doi.org/10.15468/dl.zfaw79
Anopheles claviger	6.00036	45.48279	Unclassified	GBIF: https://doi.org/10.15468/dl.zfaw79
Anopheles claviger	6.020765	51.419283	Unclassified	GBIF: https://doi.org/10.15468/dl.zfaw79
Anopheles claviger	6.03336	50.713879	Unclassified	GBIF: https://doi.org/10.15468/dl.zfaw79
Anopheles claviger	6.054728	51.436379	Unclassified	GBIF: https://doi.org/10.15468/dl.zfaw79
Anopheles claviger	6.07321	50.63235	Unclassified	GBIF: https://doi.org/10.15468/dl.zfaw79
Anopheles claviger	6.088271	52.133136	Unclassified	GBIF: https://doi.org/10.15468/dl.zfaw79
Anopheles claviger	6.09752	50.251019	Unclassified	GBIF: https://doi.org/10.15468/dl.zfaw79
Anopheles claviger	6.1	52.1	Unclassified	GBIF: https://doi.org/10.15468/dl.zfaw79

Anopheles claviger	6.163694	51.446105	Unclassified	GBIF: https://doi.org/10.15468/dl.zfaw79
Anopheles claviger	6.186546	51.403971	Unclassified	GBIF: https://doi.org/10.15468/dl.zfaw79
Anopheles claviger	6.2	51.85	Unclassified	GBIF: https://doi.org/10.15468/dl.zfaw79
Anopheles claviger	6.203273	51.391001	Unclassified	GBIF: https://doi.org/10.15468/dl.zfaw79
Anopheles claviger	6.208249	52.501411	Unclassified	GBIF: https://doi.org/10.15468/dl.zfaw79
Anopheles claviger	6.28095	50.421459	Unclassified	GBIF: https://doi.org/10.15468/dl.zfaw79
Anopheles claviger	6.28148	50.294472	Unclassified	GBIF: https://doi.org/10.15468/dl.zfaw79
Anopheles claviger	6.538623	52.329441	Unclassified	GBIF: https://doi.org/10.15468/dl.zfaw79
Anopheles claviger	6.568326	52.398116	Unclassified	GBIF: https://doi.org/10.15468/dl.zfaw79
Anopheles claviger	6.603509	50.005163	Unclassified	GBIF: https://doi.org/10.15468/dl.zfaw79
Anopheles claviger	6.61532	48.78231	Unclassified	GBIF: https://doi.org/10.15468/dl.zfaw79
Anopheles claviger	6.622425	52.280929	Unclassified	GBIF: https://doi.org/10.15468/dl.zfaw79
Anopheles claviger	6.622733	52.286742	Unclassified	GBIF: https://doi.org/10.15468/dl.zfaw79
Anopheles claviger	6.64659	53.049049	Unclassified	GBIF: https://doi.org/10.15468/dl.zfaw79
Anopheles claviger	6.649461	53.03491	Unclassified	GBIF: https://doi.org/10.15468/dl.zfaw79
Anopheles claviger	6.658962	53.028428	Unclassified	GBIF: https://doi.org/10.15468/dl.zfaw79
Anopheles claviger	6.661147	53.052131	Unclassified	GBIF: https://doi.org/10.15468/dl.zfaw79
Anopheles claviger	6.693449	53.040099	Unclassified	GBIF: https://doi.org/10.15468/dl.zfaw79
Anopheles claviger	6.696974	52.978948	Unclassified	GBIF: https://doi.org/10.15468/dl.zfaw79
Anopheles claviger	6.720815	52.324651	Unclassified	GBIF: https://doi.org/10.15468/dl.zfaw79
Anopheles claviger	6.723035	52.21605	Unclassified	GBIF: https://doi.org/10.15468/dl.zfaw79
Anopheles claviger	6.742664	52.326263	Unclassified	GBIF: https://doi.org/10.15468/dl.zfaw79
Anopheles claviger	6.746959	52.399408	Unclassified	GBIF: https://doi.org/10.15468/dl.zfaw79
Anopheles claviger	6.811631	52.1689	Unclassified	GBIF: https://doi.org/10.15468/dl.zfaw79
Anopheles claviger	6.829195	52.251809	Unclassified	GBIF: https://doi.org/10.15468/dl.zfaw79
Anopheles claviger	6.862198	52.334091	Unclassified	GBIF: https://doi.org/10.15468/dl.zfaw79
Anopheles claviger	6.900104	52.442647	Unclassified	GBIF: https://doi.org/10.15468/dl.zfaw79
Anopheles claviger	6.915288	52.269325	Unclassified	GBIF: https://doi.org/10.15468/dl.zfaw79
Anopheles claviger	6.925196	52.406189	Unclassified	GBIF: https://doi.org/10.15468/dl.zfaw79
Anopheles claviger	6.929726	52.376736	Unclassified	GBIF: https://doi.org/10.15468/dl.zfaw79
Anopheles claviger	6.931602	52.39051	Unclassified	GBIF: https://doi.org/10.15468/dl.zfaw79
Anopheles claviger	6.941757	52.294257	Unclassified	GBIF: https://doi.org/10.15468/dl.zfaw79
Anopheles claviger	6.94884	52.323926	Unclassified	GBIF: https://doi.org/10.15468/dl.zfaw79
Anopheles claviger	6.949732	52.353435	Unclassified	GBIF: https://doi.org/10.15468/dl.zfaw79
Anopheles claviger	6.951305	52.382197	Unclassified	GBIF: https://doi.org/10.15468/dl.zfaw79
Anopheles claviger	6.957609	52.347452	Unclassified	GBIF: https://doi.org/10.15468/dl.zfaw79
Anopheles claviger	6.972977	52.367958	Unclassified	GBIF: https://doi.org/10.15468/dl.zfaw79
Anopheles claviger	6.976824	52.34582	Unclassified	GBIF: https://doi.org/10.15468/dl.zfaw79
Anopheles claviger	6.996751	52.350126	Unclassified	GBIF: https://doi.org/10.15468/dl.zfaw79
Anopheles claviger	7.00598	52.354845	Unclassified	GBIF: https://doi.org/10.15468/dl.zfaw79
Anopheles claviger	7.006011	52.231745	Unclassified	GBIF: https://doi.org/10.15468/dl.zfaw79
Anopheles claviger	7.018392	52.236807	Unclassified	GBIF: https://doi.org/10.15468/dl.zfaw79

Anopheles claviger	7.020909	52.283831	Unclassified	GBIF: https://doi.org/10.15468/dl.zfaw79
Anopheles claviger	9.080996	42.100753	Unclassified	GBIF: https://doi.org/10.15468/dl.zfaw79
Anopheles claviger	9.090843	47.985958	Unclassified	GBIF: https://doi.org/10.15468/dl.zfaw79
Anopheles claviger	9.399	41.969	Unclassified	GBIF: https://doi.org/10.15468/dl.zfaw79
Anopheles claviger	9.63603	59.220138	Unclassified	GBIF: https://doi.org/10.15468/dl.zfaw79
Anopheles claviger	9.639458	53.696826	Unclassified	GBIF: https://doi.org/10.15468/dl.zfaw79
Anopheles claviger	9.775784	56.641542	Unclassified	GBIF: https://doi.org/10.15468/dl.zfaw79
Anopheles claviger	10.022533	59.976073	Unclassified	GBIF: https://doi.org/10.15468/dl.zfaw79
Anopheles claviger	10.022549	59.976086	Unclassified	GBIF: https://doi.org/10.15468/dl.zfaw79
Anopheles claviger	10.426171	51.083046	Unclassified	GBIF: https://doi.org/10.15468/dl.zfaw79
Anopheles claviger	10.606114	59.896759	Unclassified	GBIF: https://doi.org/10.15468/dl.zfaw79
Anopheles claviger	11.206003	60.24832	Unclassified	GBIF: https://doi.org/10.15468/dl.zfaw79
Anopheles claviger	12.01572	57.43398	Unclassified	GBIF: https://doi.org/10.15468/dl.zfaw79
Anopheles claviger	12.317306	51.291794	Unclassified	GBIF: https://doi.org/10.15468/dl.zfaw79
Anopheles claviger	13.06653	57.8086	Unclassified	GBIF: https://doi.org/10.15468/dl.zfaw79
Anopheles claviger	13.07544	57.81287	Unclassified	GBIF: https://doi.org/10.15468/dl.zfaw79
Anopheles claviger	13.07667	57.8085	Unclassified	GBIF: https://doi.org/10.15468/dl.zfaw79
Anopheles claviger	13.07751	57.80638	Unclassified	GBIF: https://doi.org/10.15468/dl.zfaw79
Anopheles claviger	13.23092	55.56882	Unclassified	GBIF: https://doi.org/10.15468/dl.zfaw79
Anopheles claviger	13.462715	52.621185	Unclassified	GBIF: https://doi.org/10.15468/dl.zfaw79
Anopheles claviger	13.466835	52.638271	Unclassified	GBIF: https://doi.org/10.15468/dl.zfaw79
Anopheles claviger	13.466986	58.260725	Unclassified	GBIF: https://doi.org/10.15468/dl.zfaw79
Anopheles claviger	13.488636	52.573429	Unclassified	GBIF: https://doi.org/10.15468/dl.zfaw79
Anopheles claviger	14.023	46.688	Unclassified	GBIF: https://doi.org/10.15468/dl.zfaw79
Anopheles claviger	14.15	58.4	Unclassified	GBIF: https://doi.org/10.15468/dl.zfaw79
Anopheles claviger	14.15664	55.99507	Unclassified	GBIF: https://doi.org/10.15468/dl.zfaw79
Anopheles claviger	14.26812	40.851798	Unclassified	GBIF: https://doi.org/10.15468/dl.zfaw79
Anopheles claviger	14.31037	57.77871	Unclassified	GBIF: https://doi.org/10.15468/dl.zfaw79
Anopheles claviger	15.001	40.405	Unclassified	GBIF: https://doi.org/10.15468/dl.zfaw79
Anopheles claviger	15.00504	40.426986	Unclassified	GBIF: https://doi.org/10.15468/dl.zfaw79
Anopheles claviger	15.313	44.816	Unclassified	GBIF: https://doi.org/10.15468/dl.zfaw79
Anopheles claviger	16.01778	57.16538	Unclassified	GBIF: https://doi.org/10.15468/dl.zfaw79
Anopheles claviger	16.01809	57.16018	Unclassified	GBIF: https://doi.org/10.15468/dl.zfaw79
Anopheles claviger	16.44447	58.50586	Unclassified	GBIF: https://doi.org/10.15468/dl.zfaw79
Anopheles claviger	16.46719	60.48354	Unclassified	GBIF: https://doi.org/10.15468/dl.zfaw79
Anopheles claviger	16.52	43.505	Unclassified	GBIF: https://doi.org/10.15468/dl.zfaw79
Anopheles claviger	16.524433	61.877881	Unclassified	GBIF: https://doi.org/10.15468/dl.zfaw79
Anopheles claviger	17.011024	52.402752	Unclassified	GBIF: https://doi.org/10.15468/dl.zfaw79
Anopheles claviger	17.33303	60.65202	Unclassified	GBIF: https://doi.org/10.15468/dl.zfaw79
Anopheles claviger	17.57539	59.85638	Unclassified	GBIF: https://doi.org/10.15468/dl.zfaw79
Anopheles claviger	17.57565	59.85644	Unclassified	GBIF: https://doi.org/10.15468/dl.zfaw79
Anopheles claviger	17.658	59.80061	Unclassified	GBIF: https://doi.org/10.15468/dl.zfaw79

Anopheles claviger	17.66482	59.80995	Unclassified	GBIF: https://doi.org/10.15468/dl.zfaw79
Anopheles claviger	17.75	60.42	Unclassified	GBIF: https://doi.org/10.15468/dl.zfaw79
Anopheles claviger	17.79651	62.92545	Unclassified	GBIF: https://doi.org/10.15468/dl.zfaw79
Anopheles claviger	17.96248	59.21152	Unclassified	GBIF: https://doi.org/10.15468/dl.zfaw79
Anopheles claviger	18.37636	57.28398	Unclassified	GBIF: https://doi.org/10.15468/dl.zfaw79
Anopheles claviger	18.55	60	Unclassified	GBIF: https://doi.org/10.15468/dl.zfaw79
Anopheles claviger	18.97445	47.597642	Unclassified	GBIF: https://doi.org/10.15468/dl.zfaw79
Anopheles claviger	19.3962	47.225273	Unclassified	GBIF: https://doi.org/10.15468/dl.zfaw79
Anopheles claviger	20.031	60.072	Unclassified	GBIF: https://doi.org/10.15468/dl.zfaw79
Anopheles claviger	20.902977	42.602634	Unclassified	GBIF: https://doi.org/10.15468/dl.zfaw79
Anopheles claviger	21.679133	60.169563	Unclassified	GBIF: https://doi.org/10.15468/dl.zfaw79
Anopheles claviger	22.15	58.341667	Unclassified	GBIF: https://doi.org/10.15468/dl.zfaw79
Anopheles claviger	22.909073	61.736933	Unclassified	GBIF: https://doi.org/10.15468/dl.zfaw79
Anopheles claviger	26.0896	38.3222	Unclassified	GBIF: https://doi.org/10.15468/dl.zfaw79
Anopheles claviger	26.69414	58.39218	Unclassified	GBIF: https://doi.org/10.15468/dl.zfaw79
Anopheles claviger	26.6944	58.396	Unclassified	GBIF: https://doi.org/10.15468/dl.zfaw79
Anopheles claviger	26.715279	58.390003	Unclassified	GBIF: https://doi.org/10.15468/dl.zfaw79
Anopheles claviger	26.71556	58.39028	Unclassified	GBIF: https://doi.org/10.15468/dl.zfaw79
Anopheles claviger	26.72	58.32	Unclassified	GBIF: https://doi.org/10.15468/dl.zfaw79
Anopheles claviger	26.723056	58.3175	Unclassified	GBIF: https://doi.org/10.15468/dl.zfaw79
Anopheles claviger	26.723057	58.317497	Unclassified	GBIF: https://doi.org/10.15468/dl.zfaw79
Anopheles claviger	26.9	58.5	Unclassified	GBIF: https://doi.org/10.15468/dl.zfaw79
Anopheles claviger	26.9	58.495556	Unclassified	GBIF: https://doi.org/10.15468/dl.zfaw79
Anopheles claviger	26.93218	59.38999	Unclassified	GBIF: https://doi.org/10.15468/dl.zfaw79
Anopheles claviger	27.41947	59.01583	Unclassified	GBIF: https://doi.org/10.15468/dl.zfaw79
Anopheles claviger	30.315866	59.939095	Unclassified	GBIF: https://doi.org/10.15468/dl.zfaw79
Anopheles claviger	30.582815	46.411009	Unclassified	GBIF: https://doi.org/10.15468/dl.zfaw79
Anopheles claviger	30.661834	46.539625	Unclassified	GBIF: https://doi.org/10.15468/dl.zfaw79
Anopheles claviger	30.662298	46.540956	Unclassified	GBIF: https://doi.org/10.15468/dl.zfaw79
Anopheles claviger	30.665578	46.539411	Unclassified	GBIF: https://doi.org/10.15468/dl.zfaw79
Anopheles claviger	30.665836	46.540692	Unclassified	GBIF: https://doi.org/10.15468/dl.zfaw79
Anopheles claviger	35.22332	33.06695	Unclassified	GBIF: https://doi.org/10.15468/dl.zfaw79
Anopheles claviger	35.303333	32.699444	Unclassified	GBIF: https://doi.org/10.15468/dl.zfaw79
Anopheles claviger	35.37055	32.91001	Unclassified	GBIF: https://doi.org/10.15468/dl.zfaw79
Anopheles claviger	35.568867	38.932076	Unclassified	GBIF: https://doi.org/10.15468/dl.zfaw79
Anopheles claviger	35.64416	33.2614	Unclassified	GBIF: https://doi.org/10.15468/dl.zfaw79
Anopheles claviger	35.7275	33.1614	Unclassified	GBIF: https://doi.org/10.15468/dl.zfaw79
Anopheles claviger	35.72889	33.1614	Unclassified	GBIF: https://doi.org/10.15468/dl.zfaw79
Anopheles claviger	35.74417	33.15584	Unclassified	GBIF: https://doi.org/10.15468/dl.zfaw79
Anopheles claviger	35.74417	33.13918	Unclassified	GBIF: https://doi.org/10.15468/dl.zfaw79
Anopheles claviger	37.287151	58.504621	Unclassified	GBIF: https://doi.org/10.15468/dl.zfaw79
Anopheles claviger	42.758873	59.973488	Unclassified	GBIF: https://doi.org/10.15468/dl.zfaw79

Anopheles claviger	50.211826	58.463743	Unclassified	GBIF: https://doi.org/10.15468/dl.zfaw79
Anopheles claviger	50.8364	61.668793	Unclassified	GBIF: https://doi.org/10.15468/dl.zfaw79
Anopheles claviger	54.237076	32.906025	Unclassified	GBIF: https://doi.org/10.15468/dl.zfaw79
Anopheles claviger	70.942154	38.569332	Unclassified	GBIF: https://doi.org/10.15468/dl.zfaw79
Anopheles claviger	104.69114	38.073254	Unclassified	GBIF: https://doi.org/10.15468/dl.zfaw79
Anopheles colledgei	145.26666	-16.45	Unclassified	GBIF: https://doi.org/10.15468/dl.zfaw79
Anopheles colledgei	145.325275	-16.470575	Unclassified	GBIF: https://doi.org/10.15468/dl.zfaw79
Anopheles colledgei	145.366669	-16.450001	Unclassified	GBIF: https://doi.org/10.15468/dl.zfaw79
Anopheles colledgei	145.36667	-16.45	Unclassified	GBIF: https://doi.org/10.15468/dl.zfaw79
Anopheles colledgei	145.372778	-16.4625	Unclassified	GBIF: https://doi.org/10.15468/dl.zfaw79
Anopheles colledgei	145.38	-16.47	Unclassified	GBIF: https://doi.org/10.15468/dl.zfaw79
Anopheles coluzzii	-8.29	12.17	Unclassified	GBIF: https://doi.org/10.15468/dl.zfaw79
Anopheles coluzzii	-7.23	14.17	Unclassified	GBIF: https://doi.org/10.15468/dl.zfaw79
Anopheles coluzzii	-7.21	13.66	Unclassified	GBIF: https://doi.org/10.15468/dl.zfaw79
Anopheles coluzzii	-7.17	13.55	Unclassified	GBIF: https://doi.org/10.15468/dl.zfaw79
Anopheles coluzzii	-7.1	13.85	Unclassified	GBIF: https://doi.org/10.15468/dl.zfaw79
Anopheles coluzzii	-5.780209	9.167037	Unclassified	GBIF: https://doi.org/10.15468/dl.zfaw79
Anopheles coluzzii	-5.77936	9.167506	Unclassified	GBIF: https://doi.org/10.15468/dl.zfaw79
Anopheles coluzzii	-5.77879	9.166932	Unclassified	GBIF: https://doi.org/10.15468/dl.zfaw79
Anopheles coluzzii	-5.761025	9.256636	Unclassified	GBIF: https://doi.org/10.15468/dl.zfaw79
Anopheles coluzzii	-5.761023	9.256631	Unclassified	GBIF: https://doi.org/10.15468/dl.zfaw79
Anopheles coluzzii	-5.76009	9.255415	Unclassified	GBIF: https://doi.org/10.15468/dl.zfaw79
Anopheles coluzzii	-5.759272	9.254687	Unclassified	GBIF: https://doi.org/10.15468/dl.zfaw79
Anopheles coluzzii	-5.759272	9.25472	Unclassified	GBIF: https://doi.org/10.15468/dl.zfaw79
Anopheles coluzzii	-5.756273	9.210743	Unclassified	GBIF: https://doi.org/10.15468/dl.zfaw79
Anopheles coluzzii	-5.751178	8.884021	Unclassified	GBIF: https://doi.org/10.15468/dl.zfaw79
Anopheles coluzzii	-5.750521	8.885467	Unclassified	GBIF: https://doi.org/10.15468/dl.zfaw79
Anopheles coluzzii	-5.749401	8.884694	Unclassified	GBIF: https://doi.org/10.15468/dl.zfaw79
Anopheles coluzzii	-5.748971	9.060317	Unclassified	GBIF: https://doi.org/10.15468/dl.zfaw79
Anopheles coluzzii	-5.748191	9.06072	Unclassified	GBIF: https://doi.org/10.15468/dl.zfaw79
Anopheles coluzzii	-5.748167	9.060317	Unclassified	GBIF: https://doi.org/10.15468/dl.zfaw79
Anopheles coluzzii	-5.747939	9.060538	Unclassified	GBIF: https://doi.org/10.15468/dl.zfaw79
Anopheles coluzzii	-5.740076	9.235419	Unclassified	GBIF: https://doi.org/10.15468/dl.zfaw79
Anopheles coluzzii	-5.739538	9.234096	Unclassified	GBIF: https://doi.org/10.15468/dl.zfaw79
Anopheles coluzzii	-5.711436	8.9917	Unclassified	GBIF: https://doi.org/10.15468/dl.zfaw79
Anopheles coluzzii	-5.710799	8.991622	Unclassified	GBIF: https://doi.org/10.15468/dl.zfaw79
Anopheles coluzzii	-5.710585	8.991108	Unclassified	GBIF: https://doi.org/10.15468/dl.zfaw79
Anopheles coluzzii	-5.710578	8.991117	Unclassified	GBIF: https://doi.org/10.15468/dl.zfaw79
Anopheles coluzzii	-5.68714	9.160268	Unclassified	GBIF: https://doi.org/10.15468/dl.zfaw79
Anopheles coluzzii	-5.668768	9.237244	Unclassified	GBIF: https://doi.org/10.15468/dl.zfaw79
Anopheles coluzzii	-5.668763	9.237229	Unclassified	GBIF: https://doi.org/10.15468/dl.zfaw79
Anopheles coluzzii	-5.668521	9.236631	Unclassified	GBIF: https://doi.org/10.15468/dl.zfaw79

Anopheles coluzzii	-5.668462	9.237755	Unclassified	GBIF: https://doi.org/10.15468/dl.zfaw79
Anopheles coluzzii	-5.667822	9.23615	Unclassified	GBIF: https://doi.org/10.15468/dl.zfaw79
Anopheles coluzzii	-5.667792	9.236133	Unclassified	GBIF: https://doi.org/10.15468/dl.zfaw79
Anopheles coluzzii	-5.653322	8.948459	Unclassified	GBIF: https://doi.org/10.15468/dl.zfaw79
Anopheles coluzzii	-5.653316	8.948413	Unclassified	GBIF: https://doi.org/10.15468/dl.zfaw79
Anopheles coluzzii	-5.652866	8.949516	Unclassified	GBIF: https://doi.org/10.15468/dl.zfaw79
Anopheles coluzzii	-5.652802	8.949554	Unclassified	GBIF: https://doi.org/10.15468/dl.zfaw79
Anopheles coluzzii	-5.652115	8.948168	Unclassified	GBIF: https://doi.org/10.15468/dl.zfaw79
Anopheles coluzzii	-5.652092	8.948231	Unclassified	GBIF: https://doi.org/10.15468/dl.zfaw79
Anopheles coluzzii	-5.651703	8.949318	Unclassified	GBIF: https://doi.org/10.15468/dl.zfaw79
Anopheles coluzzii	-5.651652	8.949291	Unclassified	GBIF: https://doi.org/10.15468/dl.zfaw79
Anopheles coluzzii	-5.618424	9.257512	Unclassified	GBIF: https://doi.org/10.15468/dl.zfaw79
Anopheles coluzzii	-5.617164	9.258627	Unclassified	GBIF: https://doi.org/10.15468/dl.zfaw79
Anopheles coluzzii	-5.610981	9.295961	Unclassified	GBIF: https://doi.org/10.15468/dl.zfaw79
Anopheles coluzzii	-5.610591	9.294421	Unclassified	GBIF: https://doi.org/10.15468/dl.zfaw79
Anopheles coluzzii	-5.610008	9.297116	Unclassified	GBIF: https://doi.org/10.15468/dl.zfaw79
Anopheles coluzzii	-5.609976	9.297136	Unclassified	GBIF: https://doi.org/10.15468/dl.zfaw79
Anopheles coluzzii	-5.606349	9.325325	Unclassified	GBIF: https://doi.org/10.15468/dl.zfaw79
Anopheles coluzzii	-5.58837	9.352946	Unclassified	GBIF: https://doi.org/10.15468/dl.zfaw79
Anopheles coluzzii	-5.587198	9.351439	Unclassified	GBIF: https://doi.org/10.15468/dl.zfaw79
Anopheles coluzzii	-5.584327	9.278919	Unclassified	GBIF: https://doi.org/10.15468/dl.zfaw79
Anopheles coluzzii	-5.584318	9.278908	Unclassified	GBIF: https://doi.org/10.15468/dl.zfaw79
Anopheles coluzzii	-5.584316	9.27892	Unclassified	GBIF: https://doi.org/10.15468/dl.zfaw79
Anopheles coluzzii	-5.584312	9.278941	Unclassified	GBIF: https://doi.org/10.15468/dl.zfaw79
Anopheles coluzzii	-5.584309	9.278933	Unclassified	GBIF: https://doi.org/10.15468/dl.zfaw79
Anopheles coluzzii	-5.583271	9.279775	Unclassified	GBIF: https://doi.org/10.15468/dl.zfaw79
Anopheles coluzzii	-5.583236	9.27985	Unclassified	GBIF: https://doi.org/10.15468/dl.zfaw79
Anopheles coluzzii	-5.583193	9.279842	Unclassified	GBIF: https://doi.org/10.15468/dl.zfaw79
Anopheles coluzzii	-5.583192	9.279868	Unclassified	GBIF: https://doi.org/10.15468/dl.zfaw79
Anopheles coluzzii	-5.58177	9.279226	Unclassified	GBIF: https://doi.org/10.15468/dl.zfaw79
Anopheles coluzzii	-5.581748	9.279259	Unclassified	GBIF: https://doi.org/10.15468/dl.zfaw79
Anopheles coluzzii	-5.581733	9.279249	Unclassified	GBIF: https://doi.org/10.15468/dl.zfaw79
Anopheles coluzzii	-5.581431	9.27938	Unclassified	GBIF: https://doi.org/10.15468/dl.zfaw79
Anopheles coluzzii	-5.580081	9.279904	Unclassified	GBIF: https://doi.org/10.15468/dl.zfaw79
Anopheles coluzzii	-5.579754	9.280038	Unclassified	GBIF: https://doi.org/10.15468/dl.zfaw79
Anopheles coluzzii	-5.579752	9.280037	Unclassified	GBIF: https://doi.org/10.15468/dl.zfaw79
Anopheles coluzzii	-5.567923	9.296866	Unclassified	GBIF: https://doi.org/10.15468/dl.zfaw79
Anopheles coluzzii	-5.53589	9.282552	Unclassified	GBIF: https://doi.org/10.15468/dl.zfaw79
Anopheles coluzzii	-5.514423	9.440627	Unclassified	GBIF: https://doi.org/10.15468/dl.zfaw79
Anopheles coluzzii	-5.514406	9.440621	Unclassified	GBIF: https://doi.org/10.15468/dl.zfaw79
Anopheles coluzzii	-5.513508	9.440792	Unclassified	GBIF: https://doi.org/10.15468/dl.zfaw79
Anopheles coluzzii	-5.513502	9.440762	Unclassified	GBIF: https://doi.org/10.15468/dl.zfaw79

Anopheles coluzzii	-5.513059	9.440866	Unclassified	GBIF: https://doi.org/10.15468/dl.zfaw79
Anopheles coluzzii	-5.513028	9.440838	Unclassified	GBIF: https://doi.org/10.15468/dl.zfaw79
Anopheles coluzzii	-5.500079	8.989065	Unclassified	GBIF: https://doi.org/10.15468/dl.zfaw79
Anopheles coluzzii	-5.499924	8.988782	Unclassified	GBIF: https://doi.org/10.15468/dl.zfaw79
Anopheles coluzzii	-5.499923	8.98879	Unclassified	GBIF: https://doi.org/10.15468/dl.zfaw79
Anopheles coluzzii	-5.499404	9.394611	Unclassified	GBIF: https://doi.org/10.15468/dl.zfaw79
Anopheles coluzzii	-5.499126	9.394285	Unclassified	GBIF: https://doi.org/10.15468/dl.zfaw79
Anopheles coluzzii	-5.498792	9.394007	Unclassified	GBIF: https://doi.org/10.15468/dl.zfaw79
Anopheles coluzzii	-5.488097	9.504177	Unclassified	GBIF: https://doi.org/10.15468/dl.zfaw79
Anopheles coluzzii	-5.487454	9.031845	Unclassified	GBIF: https://doi.org/10.15468/dl.zfaw79
Anopheles coluzzii	-5.486658	9.033219	Unclassified	GBIF: https://doi.org/10.15468/dl.zfaw79
Anopheles coluzzii	-5.486353	9.031134	Unclassified	GBIF: https://doi.org/10.15468/dl.zfaw79
Anopheles coluzzii	-5.48625	9.032432	Unclassified	GBIF: https://doi.org/10.15468/dl.zfaw79
Anopheles coluzzii	-5.472067	9.449943	Unclassified	GBIF: https://doi.org/10.15468/dl.zfaw79
Anopheles coluzzii	-5.471982	9.449938	Unclassified	GBIF: https://doi.org/10.15468/dl.zfaw79
Anopheles coluzzii	-5.471737	9.450453	Unclassified	GBIF: https://doi.org/10.15468/dl.zfaw79
Anopheles coluzzii	-5.471684	9.450406	Unclassified	GBIF: https://doi.org/10.15468/dl.zfaw79
Anopheles coluzzii	-5.470364	9.44996	Unclassified	GBIF: https://doi.org/10.15468/dl.zfaw79
Anopheles coluzzii	-5.470309	9.449927	Unclassified	GBIF: https://doi.org/10.15468/dl.zfaw79
Anopheles coluzzii	-5.470115	9.448979	Unclassified	GBIF: https://doi.org/10.15468/dl.zfaw79
Anopheles coluzzii	-4.4	11.3	Unclassified	GBIF: https://doi.org/10.15468/dl.zfaw79
Anopheles coluzzii	-3.553645	10.706175	Unclassified	GBIF: https://doi.org/10.15468/dl.zfaw79
Anopheles coluzzii	-3.552666	10.705635	Unclassified	GBIF: https://doi.org/10.15468/dl.zfaw79
Anopheles coluzzii	-3.552098	10.706196	Unclassified	GBIF: https://doi.org/10.15468/dl.zfaw79
Anopheles coluzzii	-3.551186	10.707987	Unclassified	GBIF: https://doi.org/10.15468/dl.zfaw79
Anopheles coluzzii	-3.523797	10.69716	Unclassified	GBIF: https://doi.org/10.15468/dl.zfaw79
Anopheles coluzzii	-3.523793	10.697185	Unclassified	GBIF: https://doi.org/10.15468/dl.zfaw79
Anopheles coluzzii	-3.52369	10.694744	Unclassified	GBIF: https://doi.org/10.15468/dl.zfaw79
Anopheles coluzzii	-3.486179	10.746839	Unclassified	GBIF: https://doi.org/10.15468/dl.zfaw79
Anopheles coluzzii	-3.479137	10.79523	Unclassified	GBIF: https://doi.org/10.15468/dl.zfaw79
Anopheles coluzzii	-3.478208	10.7946	Unclassified	GBIF: https://doi.org/10.15468/dl.zfaw79
Anopheles coluzzii	-3.477823	10.79342	Unclassified	GBIF: https://doi.org/10.15468/dl.zfaw79
Anopheles coluzzii	-3.477669	10.795122	Unclassified	GBIF: https://doi.org/10.15468/dl.zfaw79
Anopheles coluzzii	-3.477567	10.795097	Unclassified	GBIF: https://doi.org/10.15468/dl.zfaw79
Anopheles coluzzii	-3.477545	10.795111	Unclassified	GBIF: https://doi.org/10.15468/dl.zfaw79
Anopheles coluzzii	-3.47312	10.858201	Unclassified	GBIF: https://doi.org/10.15468/dl.zfaw79
Anopheles coluzzii	-3.454403	10.919474	Unclassified	GBIF: https://doi.org/10.15468/dl.zfaw79
Anopheles coluzzii	-3.453448	10.919592	Unclassified	GBIF: https://doi.org/10.15468/dl.zfaw79
Anopheles coluzzii	-3.453352	10.922321	Unclassified	GBIF: https://doi.org/10.15468/dl.zfaw79
Anopheles coluzzii	-3.453351	10.922379	Unclassified	GBIF: https://doi.org/10.15468/dl.zfaw79
Anopheles coluzzii	-3.453336	10.922344	Unclassified	GBIF: https://doi.org/10.15468/dl.zfaw79
Anopheles coluzzii	-3.453327	10.921791	Unclassified	GBIF: https://doi.org/10.15468/dl.zfaw79

Anopheles coluzzii	-3.453218	10.921763	Unclassified	GBIF: https://doi.org/10.15468/dl.zfaw79
Anopheles coluzzii	-3.453194	10.919507	Unclassified	GBIF: https://doi.org/10.15468/dl.zfaw79
Anopheles coluzzii	-3.453171	10.92178	Unclassified	GBIF: https://doi.org/10.15468/dl.zfaw79
Anopheles coluzzii	-3.453155	10.921741	Unclassified	GBIF: https://doi.org/10.15468/dl.zfaw79
Anopheles coluzzii	-3.453132	10.920139	Unclassified	GBIF: https://doi.org/10.15468/dl.zfaw79
Anopheles coluzzii	-3.453089	10.921504	Unclassified	GBIF: https://doi.org/10.15468/dl.zfaw79
Anopheles coluzzii	-3.452838	10.92036	Unclassified	GBIF: https://doi.org/10.15468/dl.zfaw79
Anopheles coluzzii	-3.452748	10.920485	Unclassified	GBIF: https://doi.org/10.15468/dl.zfaw79
Anopheles coluzzii	-3.452622	10.922154	Unclassified	GBIF: https://doi.org/10.15468/dl.zfaw79
Anopheles coluzzii	-3.452468	10.922771	Unclassified	GBIF: https://doi.org/10.15468/dl.zfaw79
Anopheles coluzzii	-3.452465	10.922756	Unclassified	GBIF: https://doi.org/10.15468/dl.zfaw79
Anopheles coluzzii	-3.446161	10.815725	Unclassified	GBIF: https://doi.org/10.15468/dl.zfaw79
Anopheles coluzzii	-3.446023	10.815473	Unclassified	GBIF: https://doi.org/10.15468/dl.zfaw79
Anopheles coluzzii	-3.445563	10.814736	Unclassified	GBIF: https://doi.org/10.15468/dl.zfaw79
Anopheles coluzzii	-3.444826	10.814347	Unclassified	GBIF: https://doi.org/10.15468/dl.zfaw79
Anopheles coluzzii	-3.44193	10.823981	Unclassified	GBIF: https://doi.org/10.15468/dl.zfaw79
Anopheles coluzzii	-3.418195	10.754379	Unclassified	GBIF: https://doi.org/10.15468/dl.zfaw79
Anopheles coluzzii	-3.418023	10.754454	Unclassified	GBIF: https://doi.org/10.15468/dl.zfaw79
Anopheles coluzzii	-3.417711	10.754363	Unclassified	GBIF: https://doi.org/10.15468/dl.zfaw79
Anopheles coluzzii	-3.417681	10.755274	Unclassified	GBIF: https://doi.org/10.15468/dl.zfaw79
Anopheles coluzzii	-3.417667	10.754263	Unclassified	GBIF: https://doi.org/10.15468/dl.zfaw79
Anopheles coluzzii	-3.417289	10.75448	Unclassified	GBIF: https://doi.org/10.15468/dl.zfaw79
Anopheles coluzzii	-3.403001	10.790794	Unclassified	GBIF: https://doi.org/10.15468/dl.zfaw79
Anopheles coluzzii	-3.40297	10.794655	Unclassified	GBIF: https://doi.org/10.15468/dl.zfaw79
Anopheles coluzzii	-3.402937	10.791297	Unclassified	GBIF: https://doi.org/10.15468/dl.zfaw79
Anopheles coluzzii	-3.399661	10.743503	Unclassified	GBIF: https://doi.org/10.15468/dl.zfaw79
Anopheles coluzzii	-3.399188	10.744949	Unclassified	GBIF: https://doi.org/10.15468/dl.zfaw79
Anopheles coluzzii	-3.39867	10.745877	Unclassified	GBIF: https://doi.org/10.15468/dl.zfaw79
Anopheles coluzzii	-3.398204	10.744567	Unclassified	GBIF: https://doi.org/10.15468/dl.zfaw79
Anopheles coluzzii	-3.39768	10.744274	Unclassified	GBIF: https://doi.org/10.15468/dl.zfaw79
Anopheles coluzzii	-3.393422	10.808199	Unclassified	GBIF: https://doi.org/10.15468/dl.zfaw79
Anopheles coluzzii	-3.393395	10.80714	Unclassified	GBIF: https://doi.org/10.15468/dl.zfaw79
Anopheles coluzzii	-3.393386	10.808234	Unclassified	GBIF: https://doi.org/10.15468/dl.zfaw79
Anopheles coluzzii	-3.387575	10.914597	Unclassified	GBIF: https://doi.org/10.15468/dl.zfaw79
Anopheles coluzzii	-3.387539	10.914582	Unclassified	GBIF: https://doi.org/10.15468/dl.zfaw79
Anopheles coluzzii	-3.387539	10.914585	Unclassified	GBIF: https://doi.org/10.15468/dl.zfaw79
Anopheles coluzzii	-3.387528	10.914534	Unclassified	GBIF: https://doi.org/10.15468/dl.zfaw79
Anopheles coluzzii	-3.387473	10.914349	Unclassified	GBIF: https://doi.org/10.15468/dl.zfaw79
Anopheles coluzzii	-3.386719	10.915201	Unclassified	GBIF: https://doi.org/10.15468/dl.zfaw79
Anopheles coluzzii	-3.386642	10.913802	Unclassified	GBIF: https://doi.org/10.15468/dl.zfaw79
Anopheles coluzzii	-3.386605	10.913293	Unclassified	GBIF: https://doi.org/10.15468/dl.zfaw79
Anopheles coluzzii	-3.386602	10.913787	Unclassified	GBIF: https://doi.org/10.15468/dl.zfaw79

Anopheles coluzzii	-3.386599	10.913746	Unclassified	GBIF: https://doi.org/10.15468/dl.zfaw79
Anopheles coluzzii	-3.385578	10.912662	Unclassified	GBIF: https://doi.org/10.15468/dl.zfaw79
Anopheles coluzzii	-3.385274	10.913341	Unclassified	GBIF: https://doi.org/10.15468/dl.zfaw79
Anopheles coluzzii	-3.385207	10.913335	Unclassified	GBIF: https://doi.org/10.15468/dl.zfaw79
Anopheles coluzzii	-3.384354	10.988509	Unclassified	GBIF: https://doi.org/10.15468/dl.zfaw79
Anopheles coluzzii	-3.384346	10.988543	Unclassified	GBIF: https://doi.org/10.15468/dl.zfaw79
Anopheles coluzzii	-3.384298	10.988608	Unclassified	GBIF: https://doi.org/10.15468/dl.zfaw79
Anopheles coluzzii	-3.384278	10.98826	Unclassified	GBIF: https://doi.org/10.15468/dl.zfaw79
Anopheles coluzzii	-3.384052	10.98831	Unclassified	GBIF: https://doi.org/10.15468/dl.zfaw79
Anopheles coluzzii	-3.383707	10.988053	Unclassified	GBIF: https://doi.org/10.15468/dl.zfaw79
Anopheles coluzzii	-3.38348	10.987875	Unclassified	GBIF: https://doi.org/10.15468/dl.zfaw79
Anopheles coluzzii	-3.383304	10.987865	Unclassified	GBIF: https://doi.org/10.15468/dl.zfaw79
Anopheles coluzzii	-3.383283	10.987871	Unclassified	GBIF: https://doi.org/10.15468/dl.zfaw79
Anopheles coluzzii	-3.342176	10.781939	Unclassified	GBIF: https://doi.org/10.15468/dl.zfaw79
Anopheles coluzzii	-3.342055	10.781851	Unclassified	GBIF: https://doi.org/10.15468/dl.zfaw79
Anopheles coluzzii	-3.341995	10.78187	Unclassified	GBIF: https://doi.org/10.15468/dl.zfaw79
Anopheles coluzzii	-3.341794	10.781939	Unclassified	GBIF: https://doi.org/10.15468/dl.zfaw79
Anopheles coluzzii	-3.341607	10.781834	Unclassified	GBIF: https://doi.org/10.15468/dl.zfaw79
Anopheles coluzzii	-3.341232	10.782837	Unclassified	GBIF: https://doi.org/10.15468/dl.zfaw79
Anopheles coluzzii	-3.340944	10.782752	Unclassified	GBIF: https://doi.org/10.15468/dl.zfaw79
Anopheles coluzzii	-3.340141	10.783414	Unclassified	GBIF: https://doi.org/10.15468/dl.zfaw79
Anopheles coluzzii	-3.332069	10.854748	Unclassified	GBIF: https://doi.org/10.15468/dl.zfaw79
Anopheles coluzzii	-3.331853	10.856236	Unclassified	GBIF: https://doi.org/10.15468/dl.zfaw79
Anopheles coluzzii	-3.330952	10.826591	Unclassified	GBIF: https://doi.org/10.15468/dl.zfaw79
Anopheles coluzzii	-3.330898	10.825726	Unclassified	GBIF: https://doi.org/10.15468/dl.zfaw79
Anopheles coluzzii	-3.33082	10.855686	Unclassified	GBIF: https://doi.org/10.15468/dl.zfaw79
Anopheles coluzzii	-3.330809	10.855686	Unclassified	GBIF: https://doi.org/10.15468/dl.zfaw79
Anopheles coluzzii	-3.330522	10.824695	Unclassified	GBIF: https://doi.org/10.15468/dl.zfaw79
Anopheles coluzzii	-3.330331	10.825128	Unclassified	GBIF: https://doi.org/10.15468/dl.zfaw79
Anopheles coluzzii	-3.330326	10.825814	Unclassified	GBIF: https://doi.org/10.15468/dl.zfaw79
Anopheles coluzzii	-3.330062	10.82558	Unclassified	GBIF: https://doi.org/10.15468/dl.zfaw79
Anopheles coluzzii	-3.329992	10.892918	Unclassified	GBIF: https://doi.org/10.15468/dl.zfaw79
Anopheles coluzzii	-3.329923	10.855973	Unclassified	GBIF: https://doi.org/10.15468/dl.zfaw79
Anopheles coluzzii	-3.329921	10.891975	Unclassified	GBIF: https://doi.org/10.15468/dl.zfaw79
Anopheles coluzzii	-3.329804	10.824567	Unclassified	GBIF: https://doi.org/10.15468/dl.zfaw79
Anopheles coluzzii	-3.329531	10.892519	Unclassified	GBIF: https://doi.org/10.15468/dl.zfaw79
Anopheles coluzzii	-3.3295	10.892524	Unclassified	GBIF: https://doi.org/10.15468/dl.zfaw79
Anopheles coluzzii	-3.328943	10.892494	Unclassified	GBIF: https://doi.org/10.15468/dl.zfaw79
Anopheles coluzzii	-3.327488	10.894405	Unclassified	GBIF: https://doi.org/10.15468/dl.zfaw79
Anopheles coluzzii	-3.32624	10.891821	Unclassified	GBIF: https://doi.org/10.15468/dl.zfaw79
Anopheles coluzzii	-3.326126	10.891635	Unclassified	GBIF: https://doi.org/10.15468/dl.zfaw79
Anopheles coluzzii	-3.32612	10.891774	Unclassified	GBIF: https://doi.org/10.15468/dl.zfaw79

Anopheles coluzzii	-3.325897	10.893405	Unclassified	GBIF: https://doi.org/10.15468/dl.zfaw79
Anopheles coluzzii	-3.325444	10.892378	Unclassified	GBIF: https://doi.org/10.15468/dl.zfaw79
Anopheles coluzzii	-3.324404	10.890882	Unclassified	GBIF: https://doi.org/10.15468/dl.zfaw79
Anopheles coluzzii	-3.324326	10.890826	Unclassified	GBIF: https://doi.org/10.15468/dl.zfaw79
Anopheles coluzzii	-3.323908	10.891245	Unclassified	GBIF: https://doi.org/10.15468/dl.zfaw79
Anopheles coluzzii	-3.323896	10.89124	Unclassified	GBIF: https://doi.org/10.15468/dl.zfaw79
Anopheles coluzzii	-3.323332	10.89123	Unclassified	GBIF: https://doi.org/10.15468/dl.zfaw79
Anopheles coluzzii	-3.322304	10.891803	Unclassified	GBIF: https://doi.org/10.15468/dl.zfaw79
Anopheles coluzzii	-3.321167	10.852973	Unclassified	GBIF: https://doi.org/10.15468/dl.zfaw79
Anopheles coluzzii	-3.320809	10.853105	Unclassified	GBIF: https://doi.org/10.15468/dl.zfaw79
Anopheles coluzzii	-3.32079	10.852812	Unclassified	GBIF: https://doi.org/10.15468/dl.zfaw79
Anopheles coluzzii	-3.320505	10.85249	Unclassified	GBIF: https://doi.org/10.15468/dl.zfaw79
Anopheles coluzzii	-3.316595	10.708401	Unclassified	GBIF: https://doi.org/10.15468/dl.zfaw79
Anopheles coluzzii	-3.316576	10.708411	Unclassified	GBIF: https://doi.org/10.15468/dl.zfaw79
Anopheles coluzzii	-3.31581	10.852056	Unclassified	GBIF: https://doi.org/10.15468/dl.zfaw79
Anopheles coluzzii	-3.315796	10.851316	Unclassified	GBIF: https://doi.org/10.15468/dl.zfaw79
Anopheles coluzzii	-3.315789	10.851345	Unclassified	GBIF: https://doi.org/10.15468/dl.zfaw79
Anopheles coluzzii	-3.31578	10.852071	Unclassified	GBIF: https://doi.org/10.15468/dl.zfaw79
Anopheles coluzzii	-3.315769	10.852078	Unclassified	GBIF: https://doi.org/10.15468/dl.zfaw79
Anopheles coluzzii	-3.31576	10.851339	Unclassified	GBIF: https://doi.org/10.15468/dl.zfaw79
Anopheles coluzzii	-3.315749	10.849297	Unclassified	GBIF: https://doi.org/10.15468/dl.zfaw79
Anopheles coluzzii	-3.315724	10.849249	Unclassified	GBIF: https://doi.org/10.15468/dl.zfaw79
Anopheles coluzzii	-3.315689	10.852419	Unclassified	GBIF: https://doi.org/10.15468/dl.zfaw79
Anopheles coluzzii	-3.315687	10.852369	Unclassified	GBIF: https://doi.org/10.15468/dl.zfaw79
Anopheles coluzzii	-3.314975	10.708011	Unclassified	GBIF: https://doi.org/10.15468/dl.zfaw79
Anopheles coluzzii	-3.314939	10.708003	Unclassified	GBIF: https://doi.org/10.15468/dl.zfaw79
Anopheles coluzzii	-3.313999	10.854832	Unclassified	GBIF: https://doi.org/10.15468/dl.zfaw79
Anopheles coluzzii	-3.313747	10.707981	Unclassified	GBIF: https://doi.org/10.15468/dl.zfaw79
Anopheles coluzzii	-3.296218	10.669313	Unclassified	GBIF: https://doi.org/10.15468/dl.zfaw79
Anopheles coluzzii	-3.295975	10.669052	Unclassified	GBIF: https://doi.org/10.15468/dl.zfaw79
Anopheles coluzzii	-3.295874	10.668288	Unclassified	GBIF: https://doi.org/10.15468/dl.zfaw79
Anopheles coluzzii	-3.29583	10.668199	Unclassified	GBIF: https://doi.org/10.15468/dl.zfaw79
Anopheles coluzzii	-3.295726	10.667961	Unclassified	GBIF: https://doi.org/10.15468/dl.zfaw79
Anopheles coluzzii	-3.29565	10.667751	Unclassified	GBIF: https://doi.org/10.15468/dl.zfaw79
Anopheles coluzzii	-3.295205	10.668353	Unclassified	GBIF: https://doi.org/10.15468/dl.zfaw79
Anopheles coluzzii	-3.2952	10.668362	Unclassified	GBIF: https://doi.org/10.15468/dl.zfaw79
Anopheles coluzzii	-3.295173	10.668426	Unclassified	GBIF: https://doi.org/10.15468/dl.zfaw79
Anopheles coluzzii	-3.289028	10.761363	Unclassified	GBIF: https://doi.org/10.15468/dl.zfaw79
Anopheles coluzzii	-3.288167	10.712289	Unclassified	GBIF: https://doi.org/10.15468/dl.zfaw79
Anopheles coluzzii	-3.288133	10.712286	Unclassified	GBIF: https://doi.org/10.15468/dl.zfaw79
Anopheles coluzzii	-3.288112	10.712303	Unclassified	GBIF: https://doi.org/10.15468/dl.zfaw79
Anopheles coluzzii	-3.2879	10.711338	Unclassified	GBIF: https://doi.org/10.15468/dl.zfaw79

Anopheles coluzzii	-3.287375	10.7113	Unclassified	GBIF: https://doi.org/10.15468/dl.zfaw79
Anopheles coluzzii	-3.2873	10.711253	Unclassified	GBIF: https://doi.org/10.15468/dl.zfaw79
Anopheles coluzzii	-3.28722	10.761557	Unclassified	GBIF: https://doi.org/10.15468/dl.zfaw79
Anopheles coluzzii	-3.28713	10.762614	Unclassified	GBIF: https://doi.org/10.15468/dl.zfaw79
Anopheles coluzzii	-3.286994	10.712789	Unclassified	GBIF: https://doi.org/10.15468/dl.zfaw79
Anopheles coluzzii	-3.286987	10.712953	Unclassified	GBIF: https://doi.org/10.15468/dl.zfaw79
Anopheles coluzzii	-3.284901	10.760504	Unclassified	GBIF: https://doi.org/10.15468/dl.zfaw79
Anopheles coluzzii	-3.284695	10.761533	Unclassified	GBIF: https://doi.org/10.15468/dl.zfaw79
Anopheles coluzzii	-3.284679	10.761879	Unclassified	GBIF: https://doi.org/10.15468/dl.zfaw79
Anopheles coluzzii	-3.256245	10.749499	Unclassified	GBIF: https://doi.org/10.15468/dl.zfaw79
Anopheles coluzzii	-3.256209	10.749487	Unclassified	GBIF: https://doi.org/10.15468/dl.zfaw79
Anopheles coluzzii	-3.256147	10.749527	Unclassified	GBIF: https://doi.org/10.15468/dl.zfaw79
Anopheles coluzzii	-3.256134	10.749538	Unclassified	GBIF: https://doi.org/10.15468/dl.zfaw79
Anopheles coluzzii	-3.255973	10.745221	Unclassified	GBIF: https://doi.org/10.15468/dl.zfaw79
Anopheles coluzzii	-3.255946	10.745223	Unclassified	GBIF: https://doi.org/10.15468/dl.zfaw79
Anopheles coluzzii	-3.255915	10.745237	Unclassified	GBIF: https://doi.org/10.15468/dl.zfaw79
Anopheles coluzzii	-3.255906	10.745241	Unclassified	GBIF: https://doi.org/10.15468/dl.zfaw79
Anopheles coluzzii	-3.25589	10.745253	Unclassified	GBIF: https://doi.org/10.15468/dl.zfaw79
Anopheles coluzzii	-3.255249	10.749508	Unclassified	GBIF: https://doi.org/10.15468/dl.zfaw79
Anopheles coluzzii	-3.255217	10.749533	Unclassified	GBIF: https://doi.org/10.15468/dl.zfaw79
Anopheles coluzzii	-3.255191	10.749473	Unclassified	GBIF: https://doi.org/10.15468/dl.zfaw79
Anopheles coluzzii	-3.254559	10.74582	Unclassified	GBIF: https://doi.org/10.15468/dl.zfaw79
Anopheles coluzzii	-3.220803	10.732259	Unclassified	GBIF: https://doi.org/10.15468/dl.zfaw79
Anopheles coluzzii	-3.212989	10.738675	Unclassified	GBIF: https://doi.org/10.15468/dl.zfaw79
Anopheles coluzzii	-3.212977	10.739225	Unclassified	GBIF: https://doi.org/10.15468/dl.zfaw79
Anopheles coluzzii	-3.212783	10.738704	Unclassified	GBIF: https://doi.org/10.15468/dl.zfaw79
Anopheles coluzzii	-3.212624	10.739121	Unclassified	GBIF: https://doi.org/10.15468/dl.zfaw79
Anopheles coluzzii	-3.208671	10.741957	Unclassified	GBIF: https://doi.org/10.15468/dl.zfaw79
Anopheles coluzzii	-3.208146	10.742015	Unclassified	GBIF: https://doi.org/10.15468/dl.zfaw79
Anopheles coluzzii	-3.202662	10.693626	Unclassified	GBIF: https://doi.org/10.15468/dl.zfaw79
Anopheles coluzzii	-3.202138	10.694553	Unclassified	GBIF: https://doi.org/10.15468/dl.zfaw79
Anopheles coluzzii	-3.202049	10.693036	Unclassified	GBIF: https://doi.org/10.15468/dl.zfaw79
Anopheles coluzzii	-3.202032	10.69303	Unclassified	GBIF: https://doi.org/10.15468/dl.zfaw79
Anopheles coluzzii	-3.20203	10.693042	Unclassified	GBIF: https://doi.org/10.15468/dl.zfaw79
Anopheles coluzzii	-3.196218	10.972128	Unclassified	GBIF: https://doi.org/10.15468/dl.zfaw79
Anopheles coluzzii	-3.195154	10.969519	Unclassified	GBIF: https://doi.org/10.15468/dl.zfaw79
Anopheles coluzzii	-3.194461	10.962637	Unclassified	GBIF: https://doi.org/10.15468/dl.zfaw79
Anopheles coluzzii	-3.19443	10.963547	Unclassified	GBIF: https://doi.org/10.15468/dl.zfaw79
Anopheles coluzzii	-3.194416	10.960994	Unclassified	GBIF: https://doi.org/10.15468/dl.zfaw79
Anopheles coluzzii	-3.194094	10.963303	Unclassified	GBIF: https://doi.org/10.15468/dl.zfaw79
Anopheles coluzzii	-3.194082	10.963314	Unclassified	GBIF: https://doi.org/10.15468/dl.zfaw79
Anopheles coluzzii	-3.19374	10.968435	Unclassified	GBIF: https://doi.org/10.15468/dl.zfaw79

Anopheles coluzzii	-3.192815	10.963358	Unclassified	GBIF: https://doi.org/10.15468/dl.zfaw79
Anopheles coluzzii	-3.191842	10.963724	Unclassified	GBIF: https://doi.org/10.15468/dl.zfaw79
Anopheles coluzzii	-3.191255	10.96408	Unclassified	GBIF: https://doi.org/10.15468/dl.zfaw79
Anopheles coluzzii	-3.190162	10.96256	Unclassified	GBIF: https://doi.org/10.15468/dl.zfaw79
Anopheles coluzzii	-3.189137	10.961069	Unclassified	GBIF: https://doi.org/10.15468/dl.zfaw79
Anopheles coluzzii	-3.18912	10.961106	Unclassified	GBIF: https://doi.org/10.15468/dl.zfaw79
Anopheles coluzzii	-3.189106	10.961109	Unclassified	GBIF: https://doi.org/10.15468/dl.zfaw79
Anopheles coluzzii	-3.166394	10.737602	Unclassified	GBIF: https://doi.org/10.15468/dl.zfaw79
Anopheles coluzzii	-3.165778	10.738544	Unclassified	GBIF: https://doi.org/10.15468/dl.zfaw79
Anopheles coluzzii	-3.165736	10.738519	Unclassified	GBIF: https://doi.org/10.15468/dl.zfaw79
Anopheles coluzzii	-3.165726	10.737976	Unclassified	GBIF: https://doi.org/10.15468/dl.zfaw79
Anopheles coluzzii	-3.165707	10.738	Unclassified	GBIF: https://doi.org/10.15468/dl.zfaw79
Anopheles coluzzii	-3.165506	10.737966	Unclassified	GBIF: https://doi.org/10.15468/dl.zfaw79
Anopheles coluzzii	0.117	5.883	Unclassified	GBIF: https://doi.org/10.15468/dl.zfaw79
Anopheles coluzzii	0.11701	5.88301	Unclassified	GBIF: https://doi.org/10.15468/dl.zfaw79
Anopheles coluzzii	0.586	5.781	Unclassified	GBIF: https://doi.org/10.15468/dl.zfaw79
Anopheles coluzzii	0.58601	5.78101	Unclassified	GBIF: https://doi.org/10.15468/dl.zfaw79
Anopheles coluzzii	0.708	9.376	Unclassified	GBIF: https://doi.org/10.15468/dl.zfaw79
Anopheles coluzzii	0.70801	9.37601	Unclassified	GBIF: https://doi.org/10.15468/dl.zfaw79
Anopheles coluzzii	1.032	9.563	Unclassified	GBIF: https://doi.org/10.15468/dl.zfaw79
Anopheles coluzzii	1.03201	9.56301	Unclassified	GBIF: https://doi.org/10.15468/dl.zfaw79
Anopheles coluzzii	1.245	6.534	Unclassified	GBIF: https://doi.org/10.15468/dl.zfaw79
Anopheles coluzzii	1.24501	6.53401	Unclassified	GBIF: https://doi.org/10.15468/dl.zfaw79
Anopheles coluzzii	1.88538	6.40966	Unclassified	GBIF: https://doi.org/10.15468/dl.zfaw79
Anopheles coluzzii	1.99834	10.9721	Unclassified	GBIF: https://doi.org/10.15468/dl.zfaw79
Anopheles coluzzii	2.00043	10.2309	Unclassified	GBIF: https://doi.org/10.15468/dl.zfaw79
Anopheles coluzzii	2.07151	7.18012	Unclassified	GBIF: https://doi.org/10.15468/dl.zfaw79
Anopheles coluzzii	2.185	7.78491	Unclassified	GBIF: https://doi.org/10.15468/dl.zfaw79
Anopheles coluzzii	2.20307	7.01729	Unclassified	GBIF: https://doi.org/10.15468/dl.zfaw79
Anopheles coluzzii	2.46442	6.36483	Unclassified	GBIF: https://doi.org/10.15468/dl.zfaw79
Anopheles coluzzii	2.46472	6.365	Unclassified	GBIF: https://doi.org/10.15468/dl.zfaw79
Anopheles coluzzii	2.485	8.03361	Unclassified	GBIF: https://doi.org/10.15468/dl.zfaw79
Anopheles coluzzii	2.60389	7.36028	Unclassified	GBIF: https://doi.org/10.15468/dl.zfaw79
Anopheles coluzzii	2.6285	6.49825	Unclassified	GBIF: https://doi.org/10.15468/dl.zfaw79
Anopheles coluzzii	2.75928	11.0863	Unclassified	GBIF: https://doi.org/10.15468/dl.zfaw79
Anopheles coluzzii	3.38425	11.8633	Unclassified	GBIF: https://doi.org/10.15468/dl.zfaw79
Anopheles coluzzii	9.3634	0.5143	Unclassified	GBIF: https://doi.org/10.15468/dl.zfaw79
Anopheles coluzzii	9.4532	0.394	Unclassified	GBIF: https://doi.org/10.15468/dl.zfaw79
Anopheles coluzzii	9.4901	0.3763	Unclassified	GBIF: https://doi.org/10.15468/dl.zfaw79
Anopheles coluzzii	9.6786	4.0792	Unclassified	GBIF: https://doi.org/10.15468/dl.zfaw79
Anopheles coluzzii	9.7705	4.0278	Unclassified	GBIF: https://doi.org/10.15468/dl.zfaw79
Anopheles coluzzii	9.824	3.9995	Unclassified	GBIF: https://doi.org/10.15468/dl.zfaw79

Anopheles coluzzii	11.55	3.9	Unclassified	GBIF: https://doi.org/10.15468/dl.zfaw79
Anopheles coluzzii	11.59	-0.19	Unclassified	GBIF: https://doi.org/10.15468/dl.zfaw79
Anopheles coluzzii	11.6	-0.2	Unclassified	GBIF: https://doi.org/10.15468/dl.zfaw79
Anopheles coluzzii	18.5405	4.33108	Unclassified	GBIF: https://doi.org/10.15468/dl.zfaw79
Anopheles coluzzii	18.5468	4.43042	Unclassified	GBIF: https://doi.org/10.15468/dl.zfaw79
Anopheles coluzzii	18.5667	4.3549	Unclassified	GBIF: https://doi.org/10.15468/dl.zfaw79
Anopheles coluzzii	18.6083	4.37667	Unclassified	GBIF: https://doi.org/10.15468/dl.zfaw79
Anopheles comorensis	45.14743	-12.8206	Unclassified	GBIF: https://doi.org/10.15468/dl.zfaw79
Anopheles corethroides	145.933304	-17.133301	Unclassified	GBIF: https://doi.org/10.15468/dl.zfaw79
Anopheles corethroides	152.888611	-27.396667	Unclassified	GBIF: https://doi.org/10.15468/dl.zfaw79
Anopheles corethroides	152.949997	-27.166667	Unclassified	GBIF: https://doi.org/10.15468/dl.zfaw79
Anopheles corethroides	153.050003	-25.466667	Unclassified	GBIF: https://doi.org/10.15468/dl.zfaw79
Anopheles costai	-72.99	-7.44	Unclassified	GBIF: https://doi.org/10.15468/dl.zfaw79
Anopheles costai	-72.99	-7.6	Unclassified	GBIF: https://doi.org/10.15468/dl.zfaw79
Anopheles costai	-72.96	-7.6	Unclassified	GBIF: https://doi.org/10.15468/dl.zfaw79
Anopheles costai	-72.88	-7.62	Unclassified	GBIF: https://doi.org/10.15468/dl.zfaw79
Anopheles costai	-72.87	-7.62	Unclassified	GBIF: https://doi.org/10.15468/dl.zfaw79
Anopheles costai	-72.82	-7.6	Unclassified	GBIF: https://doi.org/10.15468/dl.zfaw79
Anopheles costai	-72.74	-7.55	Unclassified	GBIF: https://doi.org/10.15468/dl.zfaw79
Anopheles costai	-72.71	-7.72	Unclassified	GBIF: https://doi.org/10.15468/dl.zfaw79
Anopheles costai	-72.7	-7.47	Unclassified	GBIF: https://doi.org/10.15468/dl.zfaw79
Anopheles costai	-72.68	-7.77	Unclassified	GBIF: https://doi.org/10.15468/dl.zfaw79
Anopheles costai	-72.65	-7.29	Unclassified	GBIF: https://doi.org/10.15468/dl.zfaw79
Anopheles costai	-72.64	-7.33	Unclassified	GBIF: https://doi.org/10.15468/dl.zfaw79
Anopheles costai	-70.38	-3.77	Unclassified	GBIF: https://doi.org/10.15468/dl.zfaw79
Anopheles costai	-67.07	-0.12	Unclassified	GBIF: https://doi.org/10.15468/dl.zfaw79
Anopheles costai	-66.96	-0.15	Unclassified	GBIF: https://doi.org/10.15468/dl.zfaw79
Anopheles costai	-66.84	-10	Unclassified	GBIF: https://doi.org/10.15468/dl.zfaw79
Anopheles costai	-66.82	-10.01	Unclassified	GBIF: https://doi.org/10.15468/dl.zfaw79
Anopheles costai	-66.79	-10	Unclassified	GBIF: https://doi.org/10.15468/dl.zfaw79
Anopheles costai	-64.81	1.15	Unclassified	GBIF: https://doi.org/10.15468/dl.zfaw79
Anopheles costai	-64.68	-7.41	Unclassified	GBIF: https://doi.org/10.15468/dl.zfaw79
Anopheles costai	-64.66	-7.38	Unclassified	GBIF: https://doi.org/10.15468/dl.zfaw79
Anopheles costai	-63.89	3.28	Unclassified	GBIF: https://doi.org/10.15468/dl.zfaw79
Anopheles costai	-63.77	3.39	Unclassified	GBIF: https://doi.org/10.15468/dl.zfaw79
Anopheles costai	-63.64	1.84	Unclassified	GBIF: https://doi.org/10.15468/dl.zfaw79
Anopheles costai	-63.62	1.77	Unclassified	GBIF: https://doi.org/10.15468/dl.zfaw79
Anopheles costai	-63.61	1.82	Unclassified	GBIF: https://doi.org/10.15468/dl.zfaw79
Anopheles costai	-62.28	-9.26	Unclassified	GBIF: https://doi.org/10.15468/dl.zfaw79
Anopheles costai	-62.26	-9.22	Unclassified	GBIF: https://doi.org/10.15468/dl.zfaw79
Anopheles costai	-62.25	-9.25	Unclassified	GBIF: https://doi.org/10.15468/dl.zfaw79
Anopheles costai	-62.23	-9.19	Unclassified	GBIF: https://doi.org/10.15468/dl.zfaw79

Anopheles costai	-60.82	-7.6	Unclassified	GBIF: https://doi.org/10.15468/dl.zfaw79
Anopheles costai	-60.31	-1.54	Unclassified	GBIF: https://doi.org/10.15468/dl.zfaw79
Anopheles costai	-60.27	-1.52	Unclassified	GBIF: https://doi.org/10.15468/dl.zfaw79
Anopheles costai	-60.24	-1.49	Unclassified	GBIF: https://doi.org/10.15468/dl.zfaw79
Anopheles costai	-59.14	-3.05	Unclassified	GBIF: https://doi.org/10.15468/dl.zfaw79
Anopheles costai	-53.323	5.2575	Unclassified	GBIF: https://doi.org/10.15468/dl.zfaw79
Anopheles costai	-53.24036	3.91451	Unclassified	GBIF: https://doi.org/10.15468/dl.zfaw79
Anopheles costai	-53.23477	3.92404	Unclassified	GBIF: https://doi.org/10.15468/dl.zfaw79
Anopheles costai	-52.76686	4.93226	Unclassified	GBIF: https://doi.org/10.15468/dl.zfaw79
Anopheles costai	-48.8293	-25.4754	Unclassified	GBIF: https://doi.org/10.15468/dl.zfaw79
Anopheles costalis	18.45	-34.05	Unclassified	GBIF: https://doi.org/10.15468/dl.zfaw79
Anopheles costalis	18.56	-32.84	Unclassified	GBIF: https://doi.org/10.15468/dl.zfaw79
Anopheles coustani	-16.278611	16.199722	Unclassified	GBIF: https://doi.org/10.15468/dl.zfaw79
Anopheles coustani	-9.320214	6.18407	Unclassified	GBIF: https://doi.org/10.15468/dl.zfaw79
Anopheles coustani	-7.23	14.17	Unclassified	GBIF: https://doi.org/10.15468/dl.zfaw79
Anopheles coustani	-7.21	13.66	Unclassified	GBIF: https://doi.org/10.15468/dl.zfaw79
Anopheles coustani	-7.04	13.62	Unclassified	GBIF: https://doi.org/10.15468/dl.zfaw79
Anopheles coustani	-6.34	13.91	Unclassified	GBIF: https://doi.org/10.15468/dl.zfaw79
Anopheles coustani	-4.52002	11.236	Unclassified	GBIF: https://doi.org/10.15468/dl.zfaw79
Anopheles coustani	-4.49909	11.2396	Unclassified	GBIF: https://doi.org/10.15468/dl.zfaw79
Anopheles coustani	-4.47924	11.2359	Unclassified	GBIF: https://doi.org/10.15468/dl.zfaw79
Anopheles coustani	-4.4769	11.2346	Unclassified	GBIF: https://doi.org/10.15468/dl.zfaw79
Anopheles coustani	3.33254	7.18376	Unclassified	GBIF: https://doi.org/10.15468/dl.zfaw79
Anopheles coustani	3.33254	6.58381	Unclassified	GBIF: https://doi.org/10.15468/dl.zfaw79
Anopheles coustani	3.39921	6.45049	Unclassified	GBIF: https://doi.org/10.15468/dl.zfaw79
Anopheles coustani	3.89922	7.43373	Unclassified	GBIF: https://doi.org/10.15468/dl.zfaw79
Anopheles coustani	3.9	7.4	Unclassified	GBIF: https://doi.org/10.15468/dl.zfaw79
Anopheles coustani	3.93255	6.81712	Unclassified	GBIF: https://doi.org/10.15468/dl.zfaw79
Anopheles coustani	3.98255	6.58381	Unclassified	GBIF: https://doi.org/10.15468/dl.zfaw79
Anopheles coustani	4.18254	12.46661	Unclassified	GBIF: https://doi.org/10.15468/dl.zfaw79
Anopheles coustani	4.48256	7.7837	Unclassified	GBIF: https://doi.org/10.15468/dl.zfaw79
Anopheles coustani	4.58256	8.48364	Unclassified	GBIF: https://doi.org/10.15468/dl.zfaw79
Anopheles coustani	4.74923	7.63372	Unclassified	GBIF: https://doi.org/10.15468/dl.zfaw79
Anopheles coustani	5.0659	9.30023	Unclassified	GBIF: https://doi.org/10.15468/dl.zfaw79
Anopheles coustani	5.48257	10.3668	Unclassified	GBIF: https://doi.org/10.15468/dl.zfaw79
Anopheles coustani	5.69925	5.90055	Unclassified	GBIF: https://doi.org/10.15468/dl.zfaw79
Anopheles coustani	6.06592	7.8337	Unclassified	GBIF: https://doi.org/10.15468/dl.zfaw79
Anopheles coustani	6.11591	9.08358	Unclassified	GBIF: https://doi.org/10.15468/dl.zfaw79
Anopheles coustani	6.14925	6.75047	Unclassified	GBIF: https://doi.org/10.15468/dl.zfaw79
Anopheles coustani	6.16591	9.81685	Unclassified	GBIF: https://doi.org/10.15468/dl.zfaw79
Anopheles coustani	6.36593	4.684	Unclassified	GBIF: https://doi.org/10.15468/dl.zfaw79
Anopheles coustani	6.38259	8.58363	Unclassified	GBIF: https://doi.org/10.15468/dl.zfaw79

Anopheles coustani	6.53259	9.61687	Unclassified	GBIF: https://doi.org/10.15468/dl.zfaw79
Anopheles coustani	6.53259	5.55058	Unclassified	GBIF: https://doi.org/10.15468/dl.zfaw79
Anopheles coustani	6.69925	12.16664	Unclassified	GBIF: https://doi.org/10.15468/dl.zfaw79
Anopheles coustani	6.73259	7.80037	Unclassified	GBIF: https://doi.org/10.15468/dl.zfaw79
Anopheles coustani	6.7826	6.16719	Unclassified	GBIF: https://doi.org/10.15468/dl.zfaw79
Anopheles coustani	7.01594	4.85065	Unclassified	GBIF: https://doi.org/10.15468/dl.zfaw79
Anopheles coustani	7.16593	9.16691	Unclassified	GBIF: https://doi.org/10.15468/dl.zfaw79
Anopheles coustani	7.44927	10.50013	Unclassified	GBIF: https://doi.org/10.15468/dl.zfaw79
Anopheles coustani	7.4826	10.63345	Unclassified	GBIF: https://doi.org/10.15468/dl.zfaw79
Anopheles coustani	7.54928	6.4505	Unclassified	GBIF: https://doi.org/10.15468/dl.zfaw79
Anopheles coustani	7.6826	13.01657	Unclassified	GBIF: https://doi.org/10.15468/dl.zfaw79
Anopheles coustani	7.71594	11.08341	Unclassified	GBIF: https://doi.org/10.15468/dl.zfaw79
Anopheles coustani	7.86594	8.83361	Unclassified	GBIF: https://doi.org/10.15468/dl.zfaw79
Anopheles coustani	8.23261	10.83343	Unclassified	GBIF: https://doi.org/10.15468/dl.zfaw79
Anopheles coustani	8.34929	4.98397	Unclassified	GBIF: https://doi.org/10.15468/dl.zfaw79
Anopheles coustani	8.36595	10.95009	Unclassified	GBIF: https://doi.org/10.15468/dl.zfaw79
Anopheles coustani	8.53261	12.04999	Unclassified	GBIF: https://doi.org/10.15468/dl.zfaw79
Anopheles coustani	8.61596	7.68372	Unclassified	GBIF: https://doi.org/10.15468/dl.zfaw79
Anopheles coustani	8.6993	5.96721	Unclassified	GBIF: https://doi.org/10.15468/dl.zfaw79
Anopheles coustani	8.73263	7.73371	Unclassified	GBIF: https://doi.org/10.15468/dl.zfaw79
Anopheles coustani	8.89929	9.86685	Unclassified	GBIF: https://doi.org/10.15468/dl.zfaw79
Anopheles coustani	9.19931	4.01739	Unclassified	GBIF: https://doi.org/10.15468/dl.zfaw79
Anopheles coustani	9.81597	10.28348	Unclassified	GBIF: https://doi.org/10.15468/dl.zfaw79
Anopheles coustani	10.14932	5.96721	Unclassified	GBIF: https://doi.org/10.15468/dl.zfaw79
Anopheles coustani	10.19931	11.83335	Unclassified	GBIF: https://doi.org/10.15468/dl.zfaw79
Anopheles coustani	10.19932	5.7839	Unclassified	GBIF: https://doi.org/10.15468/dl.zfaw79
Anopheles coustani	10.33264	12.29997	Unclassified	GBIF: https://doi.org/10.15468/dl.zfaw79
Anopheles coustani	10.66599	6.20053	Unclassified	GBIF: https://doi.org/10.15468/dl.zfaw79
Anopheles coustani	11.519852	3.898465	Unclassified	GBIF: https://doi.org/10.15468/dl.zfaw79
Anopheles coustani	11.9054	4.45253	Unclassified	GBIF: https://doi.org/10.15468/dl.zfaw79
Anopheles coustani	13.00286	-1.82201	Unclassified	GBIF: https://doi.org/10.15468/dl.zfaw79
Anopheles coustani	13.08269	11.85002	Unclassified	GBIF: https://doi.org/10.15468/dl.zfaw79
Anopheles coustani	13.3501	-0.9329	Unclassified	GBIF: https://doi.org/10.15468/dl.zfaw79
Anopheles coustani	13.49	-1.63	Unclassified	GBIF: https://doi.org/10.15468/dl.zfaw79
Anopheles coustani	13.58	-1.62	Unclassified	GBIF: https://doi.org/10.15468/dl.zfaw79
Anopheles coustani	13.69937	11.51672	Unclassified	GBIF: https://doi.org/10.15468/dl.zfaw79
Anopheles coustani	14.16604	12.56663	Unclassified	GBIF: https://doi.org/10.15468/dl.zfaw79
Anopheles coustani	14.25	-17.5	Unclassified	GBIF: https://doi.org/10.15468/dl.zfaw79
Anopheles coustani	18.9683	-34.3217	Unclassified	GBIF: https://doi.org/10.15468/dl.zfaw79
Anopheles coustani	19.783333	-34.083333	Unclassified	GBIF: https://doi.org/10.15468/dl.zfaw79
Anopheles coustani	22.096867	-34.077905	Unclassified	GBIF: https://doi.org/10.15468/dl.zfaw79
Anopheles coustani	26.5333	-33.3	Unclassified	GBIF: https://doi.org/10.15468/dl.zfaw79

Anopheles coustani	27.4	-32.883333	Unclassified	GBIF: https://doi.org/10.15468/dl.zfaw79
Anopheles coustani	27.755213	-13.162833	Unclassified	GBIF: https://doi.org/10.15468/dl.zfaw79
Anopheles coustani	28.2	-25.75	Unclassified	GBIF: https://doi.org/10.15468/dl.zfaw79
Anopheles coustani	28.2667	-23.8917	Unclassified	GBIF: https://doi.org/10.15468/dl.zfaw79
Anopheles coustani	28.33694	-25.846982	Unclassified	GBIF: https://doi.org/10.15468/dl.zfaw79
Anopheles coustani	30.167017	-25.581738	Unclassified	GBIF: https://doi.org/10.15468/dl.zfaw79
Anopheles coustani	30.520713	-23.993834	Unclassified	GBIF: https://doi.org/10.15468/dl.zfaw79
Anopheles coustani	30.773822	-25.088341	Unclassified	GBIF: https://doi.org/10.15468/dl.zfaw79
Anopheles coustani	30.977778	-29.890556	Unclassified	GBIF: https://doi.org/10.15468/dl.zfaw79
Anopheles coustani	31	-29.883333	Unclassified	GBIF: https://doi.org/10.15468/dl.zfaw79
Anopheles coustani	31.130479	-17.747714	Unclassified	GBIF: https://doi.org/10.15468/dl.zfaw79
Anopheles coustani	31.58	-24.985	Unclassified	GBIF: https://doi.org/10.15468/dl.zfaw79
Anopheles coustani	31.855	-24.785	Unclassified	GBIF: https://doi.org/10.15468/dl.zfaw79
Anopheles coustani	31.897222	-28.746111	Unclassified	GBIF: https://doi.org/10.15468/dl.zfaw79
Anopheles coustani	32.278612	-26.930937	Unclassified	GBIF: https://doi.org/10.15468/dl.zfaw79
Anopheles coustani	32.424557	-28.382399	Unclassified	GBIF: https://doi.org/10.15468/dl.zfaw79
Anopheles coustani	32.424587	-28.382455	Unclassified	GBIF: https://doi.org/10.15468/dl.zfaw79
Anopheles coustani	32.424604	-28.382402	Unclassified	GBIF: https://doi.org/10.15468/dl.zfaw79
Anopheles coustani	32.424631	-28.38238	Unclassified	GBIF: https://doi.org/10.15468/dl.zfaw79
Anopheles coustani	32.424658	-28.382179	Unclassified	GBIF: https://doi.org/10.15468/dl.zfaw79
Anopheles coustani	32.424691	-28.3823	Unclassified	GBIF: https://doi.org/10.15468/dl.zfaw79
Anopheles coustani	32.424704	-28.38237	Unclassified	GBIF: https://doi.org/10.15468/dl.zfaw79
Anopheles coustani	32.424737	-28.382436	Unclassified	GBIF: https://doi.org/10.15468/dl.zfaw79
Anopheles coustani	32.424748	-28.382435	Unclassified	GBIF: https://doi.org/10.15468/dl.zfaw79
Anopheles coustani	32.424863	-28.382287	Unclassified	GBIF: https://doi.org/10.15468/dl.zfaw79
Anopheles coustani	32.424866	-28.382256	Unclassified	GBIF: https://doi.org/10.15468/dl.zfaw79
Anopheles coustani	32.42488	-28.382189	Unclassified	GBIF: https://doi.org/10.15468/dl.zfaw79
Anopheles coustani	32.42494	-28.382348	Unclassified	GBIF: https://doi.org/10.15468/dl.zfaw79
Anopheles coustani	32.424979	-28.382311	Unclassified	GBIF: https://doi.org/10.15468/dl.zfaw79
Anopheles coustani	34.299404	-14.569052	Unclassified	GBIF: https://doi.org/10.15468/dl.zfaw79
Anopheles coustani	35.247914	-13.302493	Unclassified	GBIF: https://doi.org/10.15468/dl.zfaw79
Anopheles coustani	35.528348	-14.871516	Unclassified	GBIF: https://doi.org/10.15468/dl.zfaw79
Anopheles coustani	44.883333	-17.433333	Unclassified	GBIF: https://doi.org/10.15468/dl.zfaw79
Anopheles coustani	45.14743	-12.8206	Unclassified	GBIF: https://doi.org/10.15468/dl.zfaw79
Anopheles coustani	46.901619	-19.908685	Unclassified	GBIF: https://doi.org/10.15468/dl.zfaw79
Anopheles coustani	55.2697	-21.0097	Unclassified	GBIF: https://doi.org/10.15468/dl.zfaw79
Anopheles coustani	55.283889	-20.986199	Unclassified	GBIF: https://doi.org/10.15468/dl.zfaw79
Anopheles coustani	55.285669	-20.987864	Unclassified	GBIF: https://doi.org/10.15468/dl.zfaw79
Anopheles coustani	55.53241	-21.13317	Unclassified	GBIF: https://doi.org/10.15468/dl.zfaw79
Anopheles cracens	102.05	4.18	Unclassified	GBIF: https://doi.org/10.15468/dl.zfaw79
Anopheles cracens	102.574818	14.044675	Unclassified	GBIF: https://doi.org/10.15468/dl.zfaw79
Anopheles crawfordi	98.477806	18.669139	Unclassified	GBIF: https://doi.org/10.15468/dl.zfaw79

Anopheles crawfordi	98.480167	18.642528	Unclassified	GBIF: https://doi.org/10.15468/dl.zfaw79
Anopheles crawfordi	98.90558	18.91036	Unclassified	GBIF: https://doi.org/10.15468/dl.zfaw79
Anopheles crawfordi	98.96256	18.87695	Unclassified	GBIF: https://doi.org/10.15468/dl.zfaw79
Anopheles crawfordi	99.496263	11.223328	Unclassified	GBIF: https://doi.org/10.15468/dl.zfaw79
Anopheles crawfordi	99.690027	8.027579	Unclassified	GBIF: https://doi.org/10.15468/dl.zfaw79
Anopheles crawfordi	99.8965	19.00492	Unclassified	GBIF: https://doi.org/10.15468/dl.zfaw79
Anopheles crawfordi	99.94302	18.82847	Unclassified	GBIF: https://doi.org/10.15468/dl.zfaw79
Anopheles crawfordi	99.9938	18.74959	Unclassified	GBIF: https://doi.org/10.15468/dl.zfaw79
Anopheles crawfordi	101.58015	21.47004	Unclassified	GBIF: https://doi.org/10.15468/dl.zfaw79
Anopheles crawfordi	101.633333	3.216667	Unclassified	GBIF: https://doi.org/10.15468/dl.zfaw79
Anopheles crawfordi	102.03256	12.60537	Unclassified	GBIF: https://doi.org/10.15468/dl.zfaw79
Anopheles crawfordi	104.375223	14.528899	Unclassified	GBIF: https://doi.org/10.15468/dl.zfaw79
Anopheles crawfordi	105.039734	12.699187	Unclassified	GBIF: https://doi.org/10.15468/dl.zfaw79
Anopheles crawfordi	105.91339	16.517347	Unclassified	GBIF: https://doi.org/10.15468/dl.zfaw79
Anopheles cristatus	123.874992	8.102778	Unclassified	GBIF: https://doi.org/10.15468/dl.zfaw79
Anopheles cristipalpis	8.3	9.583333	Unclassified	GBIF: https://doi.org/10.15468/dl.zfaw79
Anopheles crucians	-123.663679	39.70489	Unclassified	GBIF: https://doi.org/10.15468/dl.zfaw79
Anopheles crucians	-119.706206	34.394854	Unclassified	GBIF: https://doi.org/10.15468/dl.zfaw79
Anopheles crucians	-101.593055	17.656944	Unclassified	GBIF: https://doi.org/10.15468/dl.zfaw79
Anopheles crucians	-101.514444	27.8775	Unclassified	GBIF: https://doi.org/10.15468/dl.zfaw79
Anopheles crucians	-101.446944	27.878611	Unclassified	GBIF: https://doi.org/10.15468/dl.zfaw79
Anopheles crucians	-100.177222	22.246667	Unclassified	GBIF: https://doi.org/10.15468/dl.zfaw79
Anopheles crucians	-100.000661	25.705472	Unclassified	GBIF: https://doi.org/10.15468/dl.zfaw79
Anopheles crucians	-99.467222	17.360556	Unclassified	GBIF: https://doi.org/10.15468/dl.zfaw79
Anopheles crucians	-99.391667	21.920278	Unclassified	GBIF: https://doi.org/10.15468/dl.zfaw79
Anopheles crucians	-99.264722	24.948889	Unclassified	GBIF: https://doi.org/10.15468/dl.zfaw79
Anopheles crucians	-99.064195	35.413237	Unclassified	GBIF: https://doi.org/10.15468/dl.zfaw79
Anopheles crucians	-98.471655	29.44565	Unclassified	GBIF: https://doi.org/10.15468/dl.zfaw79
Anopheles crucians	-97.868611	22.255278	Unclassified	GBIF: https://doi.org/10.15468/dl.zfaw79
Anopheles crucians	-97.826985	30.24481	Unclassified	GBIF: https://doi.org/10.15468/dl.zfaw79
Anopheles crucians	-97.7425	24.527528	Unclassified	GBIF: https://doi.org/10.15468/dl.zfaw79
Anopheles crucians	-97.692778	25.940556	Unclassified	GBIF: https://doi.org/10.15468/dl.zfaw79
Anopheles crucians	-97.654183	33.394333	Unclassified	GBIF: https://doi.org/10.15468/dl.zfaw79
Anopheles crucians	-97.637624	26.135658	Unclassified	GBIF: https://doi.org/10.15468/dl.zfaw79
Anopheles crucians	-97.608	33.399	Unclassified	GBIF: https://doi.org/10.15468/dl.zfaw79
Anopheles crucians	-97.60799	33.399233	Unclassified	GBIF: https://doi.org/10.15468/dl.zfaw79
Anopheles crucians	-97.586518	33.330827	Unclassified	GBIF: https://doi.org/10.15468/dl.zfaw79
Anopheles crucians	-97.583553	33.372972	Unclassified	GBIF: https://doi.org/10.15468/dl.zfaw79
Anopheles crucians	-97.581961	29.586953	Unclassified	GBIF: https://doi.org/10.15468/dl.zfaw79
Anopheles crucians	-97.581	33.376	Unclassified	GBIF: https://doi.org/10.15468/dl.zfaw79
Anopheles crucians	-97.580944	33.376482	Unclassified	GBIF: https://doi.org/10.15468/dl.zfaw79
Anopheles crucians	-97.58	29.59	Unclassified	GBIF: https://doi.org/10.15468/dl.zfaw79

Anopheles crucians	-97.572997	33.389372	Unclassified	GBIF: https://doi.org/10.15468/dl.zfaw79
Anopheles crucians	-97.57	33.40123	Unclassified	GBIF: https://doi.org/10.15468/dl.zfaw79
Anopheles crucians	-97.544189	25.405926	Unclassified	GBIF: https://doi.org/10.15468/dl.zfaw79
Anopheles crucians	-97.504167	25.879722	Unclassified	GBIF: https://doi.org/10.15468/dl.zfaw79
Anopheles crucians	-97.4875	32.5901	Unclassified	GBIF: https://doi.org/10.15468/dl.zfaw79
Anopheles crucians	-97.423611	25.7625	Unclassified	GBIF: https://doi.org/10.15468/dl.zfaw79
Anopheles crucians	-97.32056	32.72528	Unclassified	GBIF: https://doi.org/10.15468/dl.zfaw79
Anopheles crucians	-97.1869	32.9354	Unclassified	GBIF: https://doi.org/10.15468/dl.zfaw79
Anopheles crucians	-97.1824	32.9419	Unclassified	GBIF: https://doi.org/10.15468/dl.zfaw79
Anopheles crucians	-97.1623	32.9182	Unclassified	GBIF: https://doi.org/10.15468/dl.zfaw79
Anopheles crucians	-97.1137	32.952	Unclassified	GBIF: https://doi.org/10.15468/dl.zfaw79
Anopheles crucians	-97.1046	32.664	Unclassified	GBIF: https://doi.org/10.15468/dl.zfaw79
Anopheles crucians	-97.095	32.9411	Unclassified	GBIF: https://doi.org/10.15468/dl.zfaw79
Anopheles crucians	-97.04056	37.06889	Unclassified	GBIF: https://doi.org/10.15468/dl.zfaw79
Anopheles crucians	-96.946111	19.590278	Unclassified	GBIF: https://doi.org/10.15468/dl.zfaw79
Anopheles crucians	-96.9275	19.54	Unclassified	GBIF: https://doi.org/10.15468/dl.zfaw79
Anopheles crucians	-96.78	30.53	Unclassified	GBIF: https://doi.org/10.15468/dl.zfaw79
Anopheles crucians	-96.33	31.18	Unclassified	GBIF: https://doi.org/10.15468/dl.zfaw79
Anopheles crucians	-96.12	29.77	Unclassified	GBIF: https://doi.org/10.15468/dl.zfaw79
Anopheles crucians	-95.357	28.959	Unclassified	GBIF: https://doi.org/10.15468/dl.zfaw79
Anopheles crucians	-95.041316	29.383567	Unclassified	GBIF: https://doi.org/10.15468/dl.zfaw79
Anopheles crucians	-94.85167	39.31444	Unclassified	GBIF: https://doi.org/10.15468/dl.zfaw79
Anopheles crucians	-94.716389	17.963889	Unclassified	GBIF: https://doi.org/10.15468/dl.zfaw79
Anopheles crucians	-94.70417	37.17083	Unclassified	GBIF: https://doi.org/10.15468/dl.zfaw79
Anopheles crucians	-94.674167	18.023889	Unclassified	GBIF: https://doi.org/10.15468/dl.zfaw79
Anopheles crucians	-94.36778	36.86889	Unclassified	GBIF: https://doi.org/10.15468/dl.zfaw79
Anopheles crucians	-94.306045	35.312314	Unclassified	GBIF: https://doi.org/10.15468/dl.zfaw79
Anopheles crucians	-93.69	41.58	Unclassified	GBIF: https://doi.org/10.15468/dl.zfaw79
Anopheles crucians	-93.289053	30.846306	Unclassified	GBIF: https://doi.org/10.15468/dl.zfaw79
Anopheles crucians	-93.19411	31.081868	Unclassified	GBIF: https://doi.org/10.15468/dl.zfaw79
Anopheles crucians	-93.112804	30.336634	Unclassified	GBIF: https://doi.org/10.15468/dl.zfaw79
Anopheles crucians	-92.883333	15.195278	Unclassified	GBIF: https://doi.org/10.15468/dl.zfaw79
Anopheles crucians	-92.842139	15.177111	Unclassified	GBIF: https://doi.org/10.15468/dl.zfaw79
Anopheles crucians	-92.839944	15.176472	Unclassified	GBIF: https://doi.org/10.15468/dl.zfaw79
Anopheles crucians	-92.839722	15.176389	Unclassified	GBIF: https://doi.org/10.15468/dl.zfaw79
Anopheles crucians	-92.669313	31.390456	Unclassified	GBIF: https://doi.org/10.15468/dl.zfaw79
Anopheles crucians	-92.65	18.41	Unclassified	GBIF: https://doi.org/10.15468/dl.zfaw79
Anopheles crucians	-92.6479	18.4073	Unclassified	GBIF: https://doi.org/10.15468/dl.zfaw79
Anopheles crucians	-92.6478	18.4073	Unclassified	GBIF: https://doi.org/10.15468/dl.zfaw79
Anopheles crucians	-92.5266	31.074532	Unclassified	GBIF: https://doi.org/10.15468/dl.zfaw79
Anopheles crucians	-92.46093	31.306601	Unclassified	GBIF: https://doi.org/10.15468/dl.zfaw79
Anopheles crucians	-92.04583	32.501783	Unclassified	GBIF: https://doi.org/10.15468/dl.zfaw79

Anopheles crucians	-91.731444	16.126806	Unclassified	GBIF: https://doi.org/10.15468/dl.zfaw79
Anopheles crucians	-91.727861	16.117944	Unclassified	GBIF: https://doi.org/10.15468/dl.zfaw79
Anopheles crucians	-91.667944	16.080389	Unclassified	GBIF: https://doi.org/10.15468/dl.zfaw79
Anopheles crucians	-91.629472	16.097389	Unclassified	GBIF: https://doi.org/10.15468/dl.zfaw79
Anopheles crucians	-91.628889	16.096667	Unclassified	GBIF: https://doi.org/10.15468/dl.zfaw79
Anopheles crucians	-91.570996	34.594649	Unclassified	GBIF: https://doi.org/10.15468/dl.zfaw79
Anopheles crucians	-91.154551	30.450746	Unclassified	GBIF: https://doi.org/10.15468/dl.zfaw79
Anopheles crucians	-91.048333	33.398611	Unclassified	GBIF: https://doi.org/10.15468/dl.zfaw79
Anopheles crucians	-90.9	33.3	Unclassified	GBIF: https://doi.org/10.15468/dl.zfaw79
Anopheles crucians	-90.7575	34.773611	Unclassified	GBIF: https://doi.org/10.15468/dl.zfaw79
Anopheles crucians	-90.531667	19.842222	Unclassified	GBIF: https://doi.org/10.15468/dl.zfaw79
Anopheles crucians	-90.391139	20.870139	Unclassified	GBIF: https://doi.org/10.15468/dl.zfaw79
Anopheles crucians	-90.383333	20.858333	Unclassified	GBIF: https://doi.org/10.15468/dl.zfaw79
Anopheles crucians	-90.380278	20.912778	Unclassified	GBIF: https://doi.org/10.15468/dl.zfaw79
Anopheles crucians	-90.376555	20.856861	Unclassified	GBIF: https://doi.org/10.15468/dl.zfaw79
Anopheles crucians	-90.356667	20.959722	Unclassified	GBIF: https://doi.org/10.15468/dl.zfaw79
Anopheles crucians	-90.355	20.977222	Unclassified	GBIF: https://doi.org/10.15468/dl.zfaw79
Anopheles crucians	-90.271389	20.845	Unclassified	GBIF: https://doi.org/10.15468/dl.zfaw79
Anopheles crucians	-90.261111	20.848889	Unclassified	GBIF: https://doi.org/10.15468/dl.zfaw79
Anopheles crucians	-90.133333	38.616667	Unclassified	GBIF: https://doi.org/10.15468/dl.zfaw79
Anopheles crucians	-90.11111	38.88028	Unclassified	GBIF: https://doi.org/10.15468/dl.zfaw79
Anopheles crucians	-90.06083	40.30001	Unclassified	GBIF: https://doi.org/10.15468/dl.zfaw79
Anopheles crucians	-90.060639	45.804898	Unclassified	GBIF: https://doi.org/10.15468/dl.zfaw79
Anopheles crucians	-90.046097	45.785148	Unclassified	GBIF: https://doi.org/10.15468/dl.zfaw79
Anopheles crucians	-89.96639	36.55694	Unclassified	GBIF: https://doi.org/10.15468/dl.zfaw79
Anopheles crucians	-89.883341	30.033003	Unclassified	GBIF: https://doi.org/10.15468/dl.zfaw79
Anopheles crucians	-89.862778	38.539444	Unclassified	GBIF: https://doi.org/10.15468/dl.zfaw79
Anopheles crucians	-89.663333	21.284444	Unclassified	GBIF: https://doi.org/10.15468/dl.zfaw79
Anopheles crucians	-89.621667	20.967778	Unclassified	GBIF: https://doi.org/10.15468/dl.zfaw79
Anopheles crucians	-89.562691	45.49649	Unclassified	GBIF: https://doi.org/10.15468/dl.zfaw79
Anopheles crucians	-89.562247	45.499533	Unclassified	GBIF: https://doi.org/10.15468/dl.zfaw79
Anopheles crucians	-89.52778	36.58639	Unclassified	GBIF: https://doi.org/10.15468/dl.zfaw79
Anopheles crucians	-89.517762	46.219949	Unclassified	GBIF: https://doi.org/10.15468/dl.zfaw79
Anopheles crucians	-89.509409	46.243091	Unclassified	GBIF: https://doi.org/10.15468/dl.zfaw79
Anopheles crucians	-89.453611	37.545556	Unclassified	GBIF: https://doi.org/10.15468/dl.zfaw79
Anopheles crucians	-89.45361	37.54556	Unclassified	GBIF: https://doi.org/10.15468/dl.zfaw79
Anopheles crucians	-89.426144	37.553982	Unclassified	GBIF: https://doi.org/10.15468/dl.zfaw79
Anopheles crucians	-89.417778	18.588611	Unclassified	GBIF: https://doi.org/10.15468/dl.zfaw79
Anopheles crucians	-89.4	43.1	Unclassified	GBIF: https://doi.org/10.15468/dl.zfaw79
Anopheles crucians	-89.08	37.721	Unclassified	GBIF: https://doi.org/10.15468/dl.zfaw79
Anopheles crucians	-89.0275	37.80306	Unclassified	GBIF: https://doi.org/10.15468/dl.zfaw79
Anopheles crucians	-88.96665	17.11747	Unclassified	GBIF: https://doi.org/10.15468/dl.zfaw79

Anopheles crucians	-88.93331	16.23418	Unclassified	GBIF: https://doi.org/10.15468/dl.zfaw79
Anopheles crucians	-88.71664	17.1508	Unclassified	GBIF: https://doi.org/10.15468/dl.zfaw79
Anopheles crucians	-88.64997	18.18408	Unclassified	GBIF: https://doi.org/10.15468/dl.zfaw79
Anopheles crucians	-88.64997	18.16741	Unclassified	GBIF: https://doi.org/10.15468/dl.zfaw79
Anopheles crucians	-88.636715	36.741724	Unclassified	GBIF: https://doi.org/10.15468/dl.zfaw79
Anopheles crucians	-88.5376	17.7772	Unclassified	GBIF: https://doi.org/10.15468/dl.zfaw79
Anopheles crucians	-88.529	18.424	Unclassified	GBIF: https://doi.org/10.15468/dl.zfaw79
Anopheles crucians	-88.524	18.445	Unclassified	GBIF: https://doi.org/10.15468/dl.zfaw79
Anopheles crucians	-88.518056	18.463694	Unclassified	GBIF: https://doi.org/10.15468/dl.zfaw79
Anopheles crucians	-88.51664	17.55078	Unclassified	GBIF: https://doi.org/10.15468/dl.zfaw79
Anopheles crucians	-88.516	18.464	Unclassified	GBIF: https://doi.org/10.15468/dl.zfaw79
Anopheles crucians	-88.49997	17.33412	Unclassified	GBIF: https://doi.org/10.15468/dl.zfaw79
Anopheles crucians	-88.46664	18.45073	Unclassified	GBIF: https://doi.org/10.15468/dl.zfaw79
Anopheles crucians	-88.44997	18.43407	Unclassified	GBIF: https://doi.org/10.15468/dl.zfaw79
Anopheles crucians	-88.41663	17.51745	Unclassified	GBIF: https://doi.org/10.15468/dl.zfaw79
Anopheles crucians	-88.39997	18.4674	Unclassified	GBIF: https://doi.org/10.15468/dl.zfaw79
Anopheles crucians	-88.39997	17.46745	Unclassified	GBIF: https://doi.org/10.15468/dl.zfaw79
Anopheles crucians	-88.39997	17.48412	Unclassified	GBIF: https://doi.org/10.15468/dl.zfaw79
Anopheles crucians	-88.39761	17.96801	Unclassified	GBIF: https://doi.org/10.15468/dl.zfaw79
Anopheles crucians	-88.388742	36.235732	Unclassified	GBIF: https://doi.org/10.15468/dl.zfaw79
Anopheles crucians	-88.3833	17.63411	Unclassified	GBIF: https://doi.org/10.15468/dl.zfaw79
Anopheles crucians	-88.3333	17.13413	Unclassified	GBIF: https://doi.org/10.15468/dl.zfaw79
Anopheles crucians	-88.31663	17.48412	Unclassified	GBIF: https://doi.org/10.15468/dl.zfaw79
Anopheles crucians	-88.264167	21.055278	Unclassified	GBIF: https://doi.org/10.15468/dl.zfaw79
Anopheles crucians	-88.24996	17.58411	Unclassified	GBIF: https://doi.org/10.15468/dl.zfaw79
Anopheles crucians	-88.24996	17.48412	Unclassified	GBIF: https://doi.org/10.15468/dl.zfaw79
Anopheles crucians	-88.199023	31.816997	Unclassified	GBIF: https://doi.org/10.15468/dl.zfaw79
Anopheles crucians	-88.199	31.817	Unclassified	GBIF: https://doi.org/10.15468/dl.zfaw79
Anopheles crucians	-88.19	31.81	Unclassified	GBIF: https://doi.org/10.15468/dl.zfaw79
Anopheles crucians	-88.18987	31.820535	Unclassified	GBIF: https://doi.org/10.15468/dl.zfaw79
Anopheles crucians	-88.189759	31.80954	Unclassified	GBIF: https://doi.org/10.15468/dl.zfaw79
Anopheles crucians	-88.189346	31.82551	Unclassified	GBIF: https://doi.org/10.15468/dl.zfaw79
Anopheles crucians	-88.189	31.826	Unclassified	GBIF: https://doi.org/10.15468/dl.zfaw79
Anopheles crucians	-88.189	31.813	Unclassified	GBIF: https://doi.org/10.15468/dl.zfaw79
Anopheles crucians	-88.188829	31.813052	Unclassified	GBIF: https://doi.org/10.15468/dl.zfaw79
Anopheles crucians	-88.187	31.81	Unclassified	GBIF: https://doi.org/10.15468/dl.zfaw79
Anopheles crucians	-88.186612	31.810405	Unclassified	GBIF: https://doi.org/10.15468/dl.zfaw79
Anopheles crucians	-88.186406	31.818782	Unclassified	GBIF: https://doi.org/10.15468/dl.zfaw79
Anopheles crucians	-88.186	31.819	Unclassified	GBIF: https://doi.org/10.15468/dl.zfaw79
Anopheles crucians	-88.184494	31.81609	Unclassified	GBIF: https://doi.org/10.15468/dl.zfaw79
Anopheles crucians	-88.184	31.816	Unclassified	GBIF: https://doi.org/10.15468/dl.zfaw79
Anopheles crucians	-88.162778	21.58	Unclassified	GBIF: https://doi.org/10.15468/dl.zfaw79

Anopheles crucians	-88.161181	31.853861	Unclassified	GBIF: https://doi.org/10.15468/dl.zfaw79
Anopheles crucians	-88.146389	21.596111	Unclassified	GBIF: https://doi.org/10.15468/dl.zfaw79
Anopheles crucians	-88.063	38.3686	Unclassified	GBIF: https://doi.org/10.15468/dl.zfaw79
Anopheles crucians	-87.817	32.528	Unclassified	GBIF: https://doi.org/10.15468/dl.zfaw79
Anopheles crucians	-87.816945	32.528483	Unclassified	GBIF: https://doi.org/10.15468/dl.zfaw79
Anopheles crucians	-87.816	32.53	Unclassified	GBIF: https://doi.org/10.15468/dl.zfaw79
Anopheles crucians	-87.815715	32.530107	Unclassified	GBIF: https://doi.org/10.15468/dl.zfaw79
Anopheles crucians	-87.814	32.537	Unclassified	GBIF: https://doi.org/10.15468/dl.zfaw79
Anopheles crucians	-87.814	32.532	Unclassified	GBIF: https://doi.org/10.15468/dl.zfaw79
Anopheles crucians	-87.813573	32.537048	Unclassified	GBIF: https://doi.org/10.15468/dl.zfaw79
Anopheles crucians	-87.813507	32.532456	Unclassified	GBIF: https://doi.org/10.15468/dl.zfaw79
Anopheles crucians	-87.812333	32.539695	Unclassified	GBIF: https://doi.org/10.15468/dl.zfaw79
Anopheles crucians	-87.812	32.54	Unclassified	GBIF: https://doi.org/10.15468/dl.zfaw79
Anopheles crucians	-87.81078	32.542638	Unclassified	GBIF: https://doi.org/10.15468/dl.zfaw79
Anopheles crucians	-87.803877	32.541727	Unclassified	GBIF: https://doi.org/10.15468/dl.zfaw79
Anopheles crucians	-87.700555	21.482778	Unclassified	GBIF: https://doi.org/10.15468/dl.zfaw79
Anopheles crucians	-87.692778	21.519167	Unclassified	GBIF: https://doi.org/10.15468/dl.zfaw79
Anopheles crucians	-87.678611	21.509722	Unclassified	GBIF: https://doi.org/10.15468/dl.zfaw79
Anopheles crucians	-87.450338	32.907758	Unclassified	GBIF: https://doi.org/10.15468/dl.zfaw79
Anopheles crucians	-87.45	32.908	Unclassified	GBIF: https://doi.org/10.15468/dl.zfaw79
Anopheles crucians	-87.439	32.881	Unclassified	GBIF: https://doi.org/10.15468/dl.zfaw79
Anopheles crucians	-87.438992	32.881294	Unclassified	GBIF: https://doi.org/10.15468/dl.zfaw79
Anopheles crucians	-87.428416	32.948435	Unclassified	GBIF: https://doi.org/10.15468/dl.zfaw79
Anopheles crucians	-87.426	32.9	Unclassified	GBIF: https://doi.org/10.15468/dl.zfaw79
Anopheles crucians	-87.425914	32.89975	Unclassified	GBIF: https://doi.org/10.15468/dl.zfaw79
Anopheles crucians	-87.41908	30.31256	Unclassified	GBIF: https://doi.org/10.15468/dl.zfaw79
Anopheles crucians	-87.41506	30.31246	Unclassified	GBIF: https://doi.org/10.15468/dl.zfaw79
Anopheles crucians	-87.413474	32.914334	Unclassified	GBIF: https://doi.org/10.15468/dl.zfaw79
Anopheles crucians	-87.413	32.897	Unclassified	GBIF: https://doi.org/10.15468/dl.zfaw79
Anopheles crucians	-87.413	32.914	Unclassified	GBIF: https://doi.org/10.15468/dl.zfaw79
Anopheles crucians	-87.412758	32.896817	Unclassified	GBIF: https://doi.org/10.15468/dl.zfaw79
Anopheles crucians	-87.409	32.974	Unclassified	GBIF: https://doi.org/10.15468/dl.zfaw79
Anopheles crucians	-87.408875	32.97357	Unclassified	GBIF: https://doi.org/10.15468/dl.zfaw79
Anopheles crucians	-87.406101	32.916037	Unclassified	GBIF: https://doi.org/10.15468/dl.zfaw79
Anopheles crucians	-87.393259	32.95047	Unclassified	GBIF: https://doi.org/10.15468/dl.zfaw79
Anopheles crucians	-87.297195	30.348273	Unclassified	GBIF: https://doi.org/10.15468/dl.zfaw79
Anopheles crucians	-87.050472	20.648972	Unclassified	GBIF: https://doi.org/10.15468/dl.zfaw79
Anopheles crucians	-86.941667	20.516667	Unclassified	GBIF: https://doi.org/10.15468/dl.zfaw79
Anopheles crucians	-86.833611	21.161111	Unclassified	GBIF: https://doi.org/10.15468/dl.zfaw79
Anopheles crucians	-86.5015	39.1749	Unclassified	GBIF: https://doi.org/10.15468/dl.zfaw79
Anopheles crucians	-86.3963	30.3972	Unclassified	GBIF: https://doi.org/10.15468/dl.zfaw79
Anopheles crucians	-86.3594	30.3806	Unclassified	GBIF: https://doi.org/10.15468/dl.zfaw79

Anopheles crucians	-86.3593	30.3813	Unclassified	GBIF: https://doi.org/10.15468/dl.zfaw79
Anopheles crucians	-86.3215	30.3736	Unclassified	GBIF: https://doi.org/10.15468/dl.zfaw79
Anopheles crucians	-86.3158	30.4025	Unclassified	GBIF: https://doi.org/10.15468/dl.zfaw79
Anopheles crucians	-86.2935	30.3818	Unclassified	GBIF: https://doi.org/10.15468/dl.zfaw79
Anopheles crucians	-86.2655	30.3717	Unclassified	GBIF: https://doi.org/10.15468/dl.zfaw79
Anopheles crucians	-86.243	30.415	Unclassified	GBIF: https://doi.org/10.15468/dl.zfaw79
Anopheles crucians	-86.2404	30.3551	Unclassified	GBIF: https://doi.org/10.15468/dl.zfaw79
Anopheles crucians	-86.2297	30.4095	Unclassified	GBIF: https://doi.org/10.15468/dl.zfaw79
Anopheles crucians	-86.2261	30.365	Unclassified	GBIF: https://doi.org/10.15468/dl.zfaw79
Anopheles crucians	-86.2179	30.3904	Unclassified	GBIF: https://doi.org/10.15468/dl.zfaw79
Anopheles crucians	-86.2178	30.3908	Unclassified	GBIF: https://doi.org/10.15468/dl.zfaw79
Anopheles crucians	-86.2123	30.3496	Unclassified	GBIF: https://doi.org/10.15468/dl.zfaw79
Anopheles crucians	-86.196	30.3408	Unclassified	GBIF: https://doi.org/10.15468/dl.zfaw79
Anopheles crucians	-86.1853	30.3746	Unclassified	GBIF: https://doi.org/10.15468/dl.zfaw79
Anopheles crucians	-86.1582	30.3295	Unclassified	GBIF: https://doi.org/10.15468/dl.zfaw79
Anopheles crucians	-86.1523	30.3776	Unclassified	GBIF: https://doi.org/10.15468/dl.zfaw79
Anopheles crucians	-86.1198	30.3708	Unclassified	GBIF: https://doi.org/10.15468/dl.zfaw79
Anopheles crucians	-86.1149	30.3178	Unclassified	GBIF: https://doi.org/10.15468/dl.zfaw79
Anopheles crucians	-86.1138	39.8428	Unclassified	GBIF: https://doi.org/10.15468/dl.zfaw79
Anopheles crucians	-86.0957	30.3612	Unclassified	GBIF: https://doi.org/10.15468/dl.zfaw79
Anopheles crucians	-86.089	30.3892	Unclassified	GBIF: https://doi.org/10.15468/dl.zfaw79
Anopheles crucians	-86.088	30.3139	Unclassified	GBIF: https://doi.org/10.15468/dl.zfaw79
Anopheles crucians	-86.0694	30.3469	Unclassified	GBIF: https://doi.org/10.15468/dl.zfaw79
Anopheles crucians	-86.0245	30.2923	Unclassified	GBIF: https://doi.org/10.15468/dl.zfaw79
Anopheles crucians	-86.01659	16.01753	Unclassified	GBIF: https://doi.org/10.15468/dl.zfaw79
Anopheles crucians	-86.001	30.278	Unclassified	GBIF: https://doi.org/10.15468/dl.zfaw79
Anopheles crucians	-85.9961	30.274	Unclassified	GBIF: https://doi.org/10.15468/dl.zfaw79
Anopheles crucians	-85.9932	39.6672	Unclassified	GBIF: https://doi.org/10.15468/dl.zfaw79
Anopheles crucians	-85.9929	39.8411	Unclassified	GBIF: https://doi.org/10.15468/dl.zfaw79
Anopheles crucians	-85.9596	39.6867	Unclassified	GBIF: https://doi.org/10.15468/dl.zfaw79
Anopheles crucians	-84.96889	32.35222	Unclassified	GBIF: https://doi.org/10.15468/dl.zfaw79
Anopheles crucians	-84.495	31.198	Unclassified	GBIF: https://doi.org/10.15468/dl.zfaw79
Anopheles crucians	-84.494913	31.198457	Unclassified	GBIF: https://doi.org/10.15468/dl.zfaw79
Anopheles crucians	-84.492063	45.472944	Unclassified	GBIF: https://doi.org/10.15468/dl.zfaw79
Anopheles crucians	-84.482202	31.182724	Unclassified	GBIF: https://doi.org/10.15468/dl.zfaw79
Anopheles crucians	-84.482	31.183	Unclassified	GBIF: https://doi.org/10.15468/dl.zfaw79
Anopheles crucians	-84.478587	42.730868	Unclassified	GBIF: https://doi.org/10.15468/dl.zfaw79
Anopheles crucians	-84.477492	31.203214	Unclassified	GBIF: https://doi.org/10.15468/dl.zfaw79
Anopheles crucians	-84.477	31.192	Unclassified	GBIF: https://doi.org/10.15468/dl.zfaw79
Anopheles crucians	-84.477	31.183	Unclassified	GBIF: https://doi.org/10.15468/dl.zfaw79
Anopheles crucians	-84.476815	31.191885	Unclassified	GBIF: https://doi.org/10.15468/dl.zfaw79
Anopheles crucians	-84.476777	31.183411	Unclassified	GBIF: https://doi.org/10.15468/dl.zfaw79

Anopheles crucians	-84.471253	31.212842	Unclassified	GBIF: https://doi.org/10.15468/dl.zfaw79
Anopheles crucians	-84.471	31.213	Unclassified	GBIF: https://doi.org/10.15468/dl.zfaw79
Anopheles crucians	-84.470462	31.19332	Unclassified	GBIF: https://doi.org/10.15468/dl.zfaw79
Anopheles crucians	-84.470371	31.175208	Unclassified	GBIF: https://doi.org/10.15468/dl.zfaw79
Anopheles crucians	-84.47	31.193	Unclassified	GBIF: https://doi.org/10.15468/dl.zfaw79
Anopheles crucians	-84.468623	31.194839	Unclassified	GBIF: https://doi.org/10.15468/dl.zfaw79
Anopheles crucians	-84.462668	31.180212	Unclassified	GBIF: https://doi.org/10.15468/dl.zfaw79
Anopheles crucians	-84.459306	31.199348	Unclassified	GBIF: https://doi.org/10.15468/dl.zfaw79
Anopheles crucians	-84.459	31.199	Unclassified	GBIF: https://doi.org/10.15468/dl.zfaw79
Anopheles crucians	-84.432	35.924	Unclassified	GBIF: https://doi.org/10.15468/dl.zfaw79
Anopheles crucians	-84.431614	35.923786	Unclassified	GBIF: https://doi.org/10.15468/dl.zfaw79
Anopheles crucians	-84.410099	35.949784	Unclassified	GBIF: https://doi.org/10.15468/dl.zfaw79
Anopheles crucians	-84.403739	35.951294	Unclassified	GBIF: https://doi.org/10.15468/dl.zfaw79
Anopheles crucians	-84.337343	35.937899	Unclassified	GBIF: https://doi.org/10.15468/dl.zfaw79
Anopheles crucians	-84.3137	35.919546	Unclassified	GBIF: https://doi.org/10.15468/dl.zfaw79
Anopheles crucians	-84.19704	32.03997	Unclassified	GBIF: https://doi.org/10.15468/dl.zfaw79
Anopheles crucians	-84.157852	30.419598	Unclassified	GBIF: https://doi.org/10.15468/dl.zfaw79
Anopheles crucians	-83.67	41.69	Unclassified	GBIF: https://doi.org/10.15468/dl.zfaw79
Anopheles crucians	-83.593	35.665	Unclassified	GBIF: https://doi.org/10.15468/dl.zfaw79
Anopheles crucians	-83.592585	35.665495	Unclassified	GBIF: https://doi.org/10.15468/dl.zfaw79
Anopheles crucians	-83.579185	35.653289	Unclassified	GBIF: https://doi.org/10.15468/dl.zfaw79
Anopheles crucians	-83.579	35.653	Unclassified	GBIF: https://doi.org/10.15468/dl.zfaw79
Anopheles crucians	-83.50195	35.68896	Unclassified	GBIF: https://doi.org/10.15468/dl.zfaw79
Anopheles crucians	-83.360424	35.704279	Unclassified	GBIF: https://doi.org/10.15468/dl.zfaw79
Anopheles crucians	-83.053	39.926	Unclassified	GBIF: https://doi.org/10.15468/dl.zfaw79
Anopheles crucians	-83.041389	9.991111	Unclassified	GBIF: https://doi.org/10.15468/dl.zfaw79
Anopheles crucians	-83	32	Unclassified	GBIF: https://doi.org/10.15468/dl.zfaw79
Anopheles crucians	-82.8942	40.1043	Unclassified	GBIF: https://doi.org/10.15468/dl.zfaw79
Anopheles crucians	-82.8876	39.9689	Unclassified	GBIF: https://doi.org/10.15468/dl.zfaw79
Anopheles crucians	-82.8625	34.754	Unclassified	GBIF: https://doi.org/10.15468/dl.zfaw79
Anopheles crucians	-82.783286	40.139244	Unclassified	GBIF: https://doi.org/10.15468/dl.zfaw79
Anopheles crucians	-82.6851	27.4667	Unclassified	GBIF: https://doi.org/10.15468/dl.zfaw79
Anopheles crucians	-82.6843	27.4685	Unclassified	GBIF: https://doi.org/10.15468/dl.zfaw79
Anopheles crucians	-82.684	27.468	Unclassified	GBIF: https://doi.org/10.15468/dl.zfaw79
Anopheles crucians	-82.6837	27.5111	Unclassified	GBIF: https://doi.org/10.15468/dl.zfaw79
Anopheles crucians	-82.6835	27.5111	Unclassified	GBIF: https://doi.org/10.15468/dl.zfaw79
Anopheles crucians	-82.6574	27.5031	Unclassified	GBIF: https://doi.org/10.15468/dl.zfaw79
Anopheles crucians	-82.657	27.503	Unclassified	GBIF: https://doi.org/10.15468/dl.zfaw79
Anopheles crucians	-82.6528	28.4509	Unclassified	GBIF: https://doi.org/10.15468/dl.zfaw79
Anopheles crucians	-82.6486	28.4956	Unclassified	GBIF: https://doi.org/10.15468/dl.zfaw79
Anopheles crucians	-82.6481	28.4949	Unclassified	GBIF: https://doi.org/10.15468/dl.zfaw79
Anopheles crucians	-82.6305	28.461	Unclassified	GBIF: https://doi.org/10.15468/dl.zfaw79

Anopheles crucians	-82.629	27.5322	Unclassified	GBIF: https://doi.org/10.15468/dl.zfaw79
Anopheles crucians	-82.6282	27.532	Unclassified	GBIF: https://doi.org/10.15468/dl.zfaw79
Anopheles crucians	-82.621	27.447	Unclassified	GBIF: https://doi.org/10.15468/dl.zfaw79
Anopheles crucians	-82.6205	27.4472	Unclassified	GBIF: https://doi.org/10.15468/dl.zfaw79
Anopheles crucians	-82.6178	28.5343	Unclassified	GBIF: https://doi.org/10.15468/dl.zfaw79
Anopheles crucians	-82.6165	28.4817	Unclassified	GBIF: https://doi.org/10.15468/dl.zfaw79
Anopheles crucians	-82.6134	27.4757	Unclassified	GBIF: https://doi.org/10.15468/dl.zfaw79
Anopheles crucians	-82.613	27.476	Unclassified	GBIF: https://doi.org/10.15468/dl.zfaw79
Anopheles crucians	-82.6	27.499	Unclassified	GBIF: https://doi.org/10.15468/dl.zfaw79
Anopheles crucians	-82.5932	27.5695	Unclassified	GBIF: https://doi.org/10.15468/dl.zfaw79
Anopheles crucians	-82.593	27.57	Unclassified	GBIF: https://doi.org/10.15468/dl.zfaw79
Anopheles crucians	-82.5921	27.4889	Unclassified	GBIF: https://doi.org/10.15468/dl.zfaw79
Anopheles crucians	-82.592	27.489	Unclassified	GBIF: https://doi.org/10.15468/dl.zfaw79
Anopheles crucians	-82.5912	28.6897	Unclassified	GBIF: https://doi.org/10.15468/dl.zfaw79
Anopheles crucians	-82.5905	27.5288	Unclassified	GBIF: https://doi.org/10.15468/dl.zfaw79
Anopheles crucians	-82.5881	27.5292	Unclassified	GBIF: https://doi.org/10.15468/dl.zfaw79
Anopheles crucians	-82.588	27.529	Unclassified	GBIF: https://doi.org/10.15468/dl.zfaw79
Anopheles crucians	-82.5878	27.5286	Unclassified	GBIF: https://doi.org/10.15468/dl.zfaw79
Anopheles crucians	-82.586901	28.904733	Unclassified	GBIF: https://doi.org/10.15468/dl.zfaw79
Anopheles crucians	-82.5824	27.531	Unclassified	GBIF: https://doi.org/10.15468/dl.zfaw79
Anopheles crucians	-82.582	27.531	Unclassified	GBIF: https://doi.org/10.15468/dl.zfaw79
Anopheles crucians	-82.577	28.5731	Unclassified	GBIF: https://doi.org/10.15468/dl.zfaw79
Anopheles crucians	-82.574819	27.498928	Unclassified	GBIF: https://doi.org/10.15468/dl.zfaw79
Anopheles crucians	-82.566	27.533	Unclassified	GBIF: https://doi.org/10.15468/dl.zfaw79
Anopheles crucians	-82.5657	27.5333	Unclassified	GBIF: https://doi.org/10.15468/dl.zfaw79
Anopheles crucians	-82.555	28.4927	Unclassified	GBIF: https://doi.org/10.15468/dl.zfaw79
Anopheles crucians	-82.553	27.64	Unclassified	GBIF: https://doi.org/10.15468/dl.zfaw79
Anopheles crucians	-82.5527	27.6405	Unclassified	GBIF: https://doi.org/10.15468/dl.zfaw79
Anopheles crucians	-82.5513	27.4751	Unclassified	GBIF: https://doi.org/10.15468/dl.zfaw79
Anopheles crucians	-82.551	27.475	Unclassified	GBIF: https://doi.org/10.15468/dl.zfaw79
Anopheles crucians	-82.549	27.439	Unclassified	GBIF: https://doi.org/10.15468/dl.zfaw79
Anopheles crucians	-82.5489	27.4394	Unclassified	GBIF: https://doi.org/10.15468/dl.zfaw79
Anopheles crucians	-82.548	27.5909	Unclassified	GBIF: https://doi.org/10.15468/dl.zfaw79
Anopheles crucians	-82.548	27.591	Unclassified	GBIF: https://doi.org/10.15468/dl.zfaw79
Anopheles crucians	-82.546	27.554	Unclassified	GBIF: https://doi.org/10.15468/dl.zfaw79
Anopheles crucians	-82.5455	27.554	Unclassified	GBIF: https://doi.org/10.15468/dl.zfaw79
Anopheles crucians	-82.538	27.516	Unclassified	GBIF: https://doi.org/10.15468/dl.zfaw79
Anopheles crucians	-82.5377	27.5163	Unclassified	GBIF: https://doi.org/10.15468/dl.zfaw79
Anopheles crucians	-82.536	27.603	Unclassified	GBIF: https://doi.org/10.15468/dl.zfaw79
Anopheles crucians	-82.5357	27.6031	Unclassified	GBIF: https://doi.org/10.15468/dl.zfaw79
Anopheles crucians	-82.5355	27.6031	Unclassified	GBIF: https://doi.org/10.15468/dl.zfaw79
Anopheles crucians	-82.5353	27.6164	Unclassified	GBIF: https://doi.org/10.15468/dl.zfaw79

Anopheles crucians	-82.535	27.616	Unclassified	GBIF: https://doi.org/10.15468/dl.zfaw79
Anopheles crucians	-82.534	27.49	Unclassified	GBIF: https://doi.org/10.15468/dl.zfaw79
Anopheles crucians	-82.5338	27.4899	Unclassified	GBIF: https://doi.org/10.15468/dl.zfaw79
Anopheles crucians	-82.5315	27.6023	Unclassified	GBIF: https://doi.org/10.15468/dl.zfaw79
Anopheles crucians	-82.5313	28.6385	Unclassified	GBIF: https://doi.org/10.15468/dl.zfaw79
Anopheles crucians	-82.529	27.601	Unclassified	GBIF: https://doi.org/10.15468/dl.zfaw79
Anopheles crucians	-82.525	27.427	Unclassified	GBIF: https://doi.org/10.15468/dl.zfaw79
Anopheles crucians	-82.5249	27.5469	Unclassified	GBIF: https://doi.org/10.15468/dl.zfaw79
Anopheles crucians	-82.5247	27.4266	Unclassified	GBIF: https://doi.org/10.15468/dl.zfaw79
Anopheles crucians	-82.524	27.4695	Unclassified	GBIF: https://doi.org/10.15468/dl.zfaw79
Anopheles crucians	-82.524	27.47	Unclassified	GBIF: https://doi.org/10.15468/dl.zfaw79
Anopheles crucians	-82.5236	28.6362	Unclassified	GBIF: https://doi.org/10.15468/dl.zfaw79
Anopheles crucians	-82.505278	22.893056	Unclassified	GBIF: https://doi.org/10.15468/dl.zfaw79
Anopheles crucians	-82.5045	27.4065	Unclassified	GBIF: https://doi.org/10.15468/dl.zfaw79
Anopheles crucians	-82.5041	27.5741	Unclassified	GBIF: https://doi.org/10.15468/dl.zfaw79
Anopheles crucians	-82.5041	27.4067	Unclassified	GBIF: https://doi.org/10.15468/dl.zfaw79
Anopheles crucians	-82.504	27.574	Unclassified	GBIF: https://doi.org/10.15468/dl.zfaw79
Anopheles crucians	-82.504	27.407	Unclassified	GBIF: https://doi.org/10.15468/dl.zfaw79
Anopheles crucians	-82.499	27.449	Unclassified	GBIF: https://doi.org/10.15468/dl.zfaw79
Anopheles crucians	-82.4987	27.4489	Unclassified	GBIF: https://doi.org/10.15468/dl.zfaw79
Anopheles crucians	-82.497	27.628	Unclassified	GBIF: https://doi.org/10.15468/dl.zfaw79
Anopheles crucians	-82.4968	27.628	Unclassified	GBIF: https://doi.org/10.15468/dl.zfaw79
Anopheles crucians	-82.4885	28.4344	Unclassified	GBIF: https://doi.org/10.15468/dl.zfaw79
Anopheles crucians	-82.4853	28.4633	Unclassified	GBIF: https://doi.org/10.15468/dl.zfaw79
Anopheles crucians	-82.484	27.578	Unclassified	GBIF: https://doi.org/10.15468/dl.zfaw79
Anopheles crucians	-82.4837	27.5784	Unclassified	GBIF: https://doi.org/10.15468/dl.zfaw79
Anopheles crucians	-82.4804	27.4813	Unclassified	GBIF: https://doi.org/10.15468/dl.zfaw79
Anopheles crucians	-82.4803	27.565	Unclassified	GBIF: https://doi.org/10.15468/dl.zfaw79
Anopheles crucians	-82.48	27.565	Unclassified	GBIF: https://doi.org/10.15468/dl.zfaw79
Anopheles crucians	-82.48	27.42	Unclassified	GBIF: https://doi.org/10.15468/dl.zfaw79
Anopheles crucians	-82.48	27.481	Unclassified	GBIF: https://doi.org/10.15468/dl.zfaw79
Anopheles crucians	-82.4798	27.4197	Unclassified	GBIF: https://doi.org/10.15468/dl.zfaw79
Anopheles crucians	-82.468	27.5161	Unclassified	GBIF: https://doi.org/10.15468/dl.zfaw79
Anopheles crucians	-82.4646	28.4401	Unclassified	GBIF: https://doi.org/10.15468/dl.zfaw79
Anopheles crucians	-82.4529	28.5292	Unclassified	GBIF: https://doi.org/10.15468/dl.zfaw79
Anopheles crucians	-82.439	27.439	Unclassified	GBIF: https://doi.org/10.15468/dl.zfaw79
Anopheles crucians	-82.4387	27.4389	Unclassified	GBIF: https://doi.org/10.15468/dl.zfaw79
Anopheles crucians	-82.4386	27.4389	Unclassified	GBIF: https://doi.org/10.15468/dl.zfaw79
Anopheles crucians	-82.436	27.442	Unclassified	GBIF: https://doi.org/10.15468/dl.zfaw79
Anopheles crucians	-82.4358	27.4424	Unclassified	GBIF: https://doi.org/10.15468/dl.zfaw79
Anopheles crucians	-82.4336	28.6227	Unclassified	GBIF: https://doi.org/10.15468/dl.zfaw79
Anopheles crucians	-82.432	27.513	Unclassified	GBIF: https://doi.org/10.15468/dl.zfaw79

Anopheles crucians	-82.4319	27.5126	Unclassified	GBIF: https://doi.org/10.15468/dl.zfaw79
Anopheles crucians	-82.4319	28.4846	Unclassified	GBIF: https://doi.org/10.15468/dl.zfaw79
Anopheles crucians	-82.4316	28.4799	Unclassified	GBIF: https://doi.org/10.15468/dl.zfaw79
Anopheles crucians	-82.4262	27.4195	Unclassified	GBIF: https://doi.org/10.15468/dl.zfaw79
Anopheles crucians	-82.426	27.419	Unclassified	GBIF: https://doi.org/10.15468/dl.zfaw79
Anopheles crucians	-82.425	27.565	Unclassified	GBIF: https://doi.org/10.15468/dl.zfaw79
Anopheles crucians	-82.4249	27.565	Unclassified	GBIF: https://doi.org/10.15468/dl.zfaw79
Anopheles crucians	-82.42	27.575	Unclassified	GBIF: https://doi.org/10.15468/dl.zfaw79
Anopheles crucians	-82.4197	27.5751	Unclassified	GBIF: https://doi.org/10.15468/dl.zfaw79
Anopheles crucians	-82.4171	27.6395	Unclassified	GBIF: https://doi.org/10.15468/dl.zfaw79
Anopheles crucians	-82.4171	27.6392	Unclassified	GBIF: https://doi.org/10.15468/dl.zfaw79
Anopheles crucians	-82.417	27.639	Unclassified	GBIF: https://doi.org/10.15468/dl.zfaw79
Anopheles crucians	-82.4135	28.4742	Unclassified	GBIF: https://doi.org/10.15468/dl.zfaw79
Anopheles crucians	-82.409	28.4774	Unclassified	GBIF: https://doi.org/10.15468/dl.zfaw79
Anopheles crucians	-82.402	27.485	Unclassified	GBIF: https://doi.org/10.15468/dl.zfaw79
Anopheles crucians	-82.4016	27.4846	Unclassified	GBIF: https://doi.org/10.15468/dl.zfaw79
Anopheles crucians	-82.3911	27.4357	Unclassified	GBIF: https://doi.org/10.15468/dl.zfaw79
Anopheles crucians	-82.3911	27.4358	Unclassified	GBIF: https://doi.org/10.15468/dl.zfaw79
Anopheles crucians	-82.3891	34.8463	Unclassified	GBIF: https://doi.org/10.15468/dl.zfaw79
Anopheles crucians	-82.3848	28.571	Unclassified	GBIF: https://doi.org/10.15468/dl.zfaw79
Anopheles crucians	-82.37955	27.66654	Unclassified	GBIF: https://doi.org/10.15468/dl.zfaw79
Anopheles crucians	-82.377	27.427	Unclassified	GBIF: https://doi.org/10.15468/dl.zfaw79
Anopheles crucians	-82.3766	27.4265	Unclassified	GBIF: https://doi.org/10.15468/dl.zfaw79
Anopheles crucians	-82.366	29.633	Unclassified	GBIF: https://doi.org/10.15468/dl.zfaw79
Anopheles crucians	-82.362	27.489	Unclassified	GBIF: https://doi.org/10.15468/dl.zfaw79
Anopheles crucians	-82.3617	27.4894	Unclassified	GBIF: https://doi.org/10.15468/dl.zfaw79
Anopheles crucians	-82.36	28.5739	Unclassified	GBIF: https://doi.org/10.15468/dl.zfaw79
Anopheles crucians	-82.3557	27.5098	Unclassified	GBIF: https://doi.org/10.15468/dl.zfaw79
Anopheles crucians	-82.355	27.578	Unclassified	GBIF: https://doi.org/10.15468/dl.zfaw79
Anopheles crucians	-82.3548	27.5776	Unclassified	GBIF: https://doi.org/10.15468/dl.zfaw79
Anopheles crucians	-82.3539	28.5431	Unclassified	GBIF: https://doi.org/10.15468/dl.zfaw79
Anopheles crucians	-82.3534	27.5491	Unclassified	GBIF: https://doi.org/10.15468/dl.zfaw79
Anopheles crucians	-82.353	27.549	Unclassified	GBIF: https://doi.org/10.15468/dl.zfaw79
Anopheles crucians	-82.3524	28.5725	Unclassified	GBIF: https://doi.org/10.15468/dl.zfaw79
Anopheles crucians	-82.3284	27.4276	Unclassified	GBIF: https://doi.org/10.15468/dl.zfaw79
Anopheles crucians	-82.327489	29.672369	Unclassified	GBIF: https://doi.org/10.15468/dl.zfaw79
Anopheles crucians	-82.324	27.4746	Unclassified	GBIF: https://doi.org/10.15468/dl.zfaw79
Anopheles crucians	-82.324	27.475	Unclassified	GBIF: https://doi.org/10.15468/dl.zfaw79
Anopheles crucians	-82.3103	27.4982	Unclassified	GBIF: https://doi.org/10.15468/dl.zfaw79
Anopheles crucians	-82.3086	27.4967	Unclassified	GBIF: https://doi.org/10.15468/dl.zfaw79
Anopheles crucians	-82.304	27.4078	Unclassified	GBIF: https://doi.org/10.15468/dl.zfaw79
Anopheles crucians	-82.304	27.408	Unclassified	GBIF: https://doi.org/10.15468/dl.zfaw79

Anopheles crucians	-82.304	27.464	Unclassified	GBIF: https://doi.org/10.15468/dl.zfaw79
Anopheles crucians	-82.3038	27.4636	Unclassified	GBIF: https://doi.org/10.15468/dl.zfaw79
Anopheles crucians	-82.3	32.38	Unclassified	GBIF: https://doi.org/10.15468/dl.zfaw79
Anopheles crucians	-82.2905	28.4455	Unclassified	GBIF: https://doi.org/10.15468/dl.zfaw79
Anopheles crucians	-82.2892	28.5284	Unclassified	GBIF: https://doi.org/10.15468/dl.zfaw79
Anopheles crucians	-82.288	27.412	Unclassified	GBIF: https://doi.org/10.15468/dl.zfaw79
Anopheles crucians	-82.2876	27.4122	Unclassified	GBIF: https://doi.org/10.15468/dl.zfaw79
Anopheles crucians	-82.282569	27.235394	Unclassified	GBIF: https://doi.org/10.15468/dl.zfaw79
Anopheles crucians	-82.2729	28.6632	Unclassified	GBIF: https://doi.org/10.15468/dl.zfaw79
Anopheles crucians	-82.2642	28.6428	Unclassified	GBIF: https://doi.org/10.15468/dl.zfaw79
Anopheles crucians	-82.2603	27.4081	Unclassified	GBIF: https://doi.org/10.15468/dl.zfaw79
Anopheles crucians	-82.26	27.408	Unclassified	GBIF: https://doi.org/10.15468/dl.zfaw79
Anopheles crucians	-82.2435	27.3471	Unclassified	GBIF: https://doi.org/10.15468/dl.zfaw79
Anopheles crucians	-82.243	27.347	Unclassified	GBIF: https://doi.org/10.15468/dl.zfaw79
Anopheles crucians	-82.2304	27.2866	Unclassified	GBIF: https://doi.org/10.15468/dl.zfaw79
Anopheles crucians	-82.23	27.287	Unclassified	GBIF: https://doi.org/10.15468/dl.zfaw79
Anopheles crucians	-82.207	28.5137	Unclassified	GBIF: https://doi.org/10.15468/dl.zfaw79
Anopheles crucians	-82.2069	28.5108	Unclassified	GBIF: https://doi.org/10.15468/dl.zfaw79
Anopheles crucians	-82.2	27.591	Unclassified	GBIF: https://doi.org/10.15468/dl.zfaw79
Anopheles crucians	-82.1999	27.5915	Unclassified	GBIF: https://doi.org/10.15468/dl.zfaw79
Anopheles crucians	-82.1998	27.5915	Unclassified	GBIF: https://doi.org/10.15468/dl.zfaw79
Anopheles crucians	-82.1996	28.4831	Unclassified	GBIF: https://doi.org/10.15468/dl.zfaw79
Anopheles crucians	-82.1982	28.5512	Unclassified	GBIF: https://doi.org/10.15468/dl.zfaw79
Anopheles crucians	-82.1746	28.4942	Unclassified	GBIF: https://doi.org/10.15468/dl.zfaw79
Anopheles crucians	-82.17	27.471	Unclassified	GBIF: https://doi.org/10.15468/dl.zfaw79
Anopheles crucians	-82.1699	27.4708	Unclassified	GBIF: https://doi.org/10.15468/dl.zfaw79
Anopheles crucians	-82.1675	28.4819	Unclassified	GBIF: https://doi.org/10.15468/dl.zfaw79
Anopheles crucians	-82.1672	26.4846	Unclassified	GBIF: https://doi.org/10.15468/dl.zfaw79
Anopheles crucians	-82.1641	28.5418	Unclassified	GBIF: https://doi.org/10.15468/dl.zfaw79
Anopheles crucians	-82.1636	28.5384	Unclassified	GBIF: https://doi.org/10.15468/dl.zfaw79
Anopheles crucians	-82.158	27.35	Unclassified	GBIF: https://doi.org/10.15468/dl.zfaw79
Anopheles crucians	-82.1576	27.3497	Unclassified	GBIF: https://doi.org/10.15468/dl.zfaw79
Anopheles crucians	-82.1563	26.4707	Unclassified	GBIF: https://doi.org/10.15468/dl.zfaw79
Anopheles crucians	-82.138	27.571	Unclassified	GBIF: https://doi.org/10.15468/dl.zfaw79
Anopheles crucians	-82.1378	27.5709	Unclassified	GBIF: https://doi.org/10.15468/dl.zfaw79
Anopheles crucians	-82.1333	27.427	Unclassified	GBIF: https://doi.org/10.15468/dl.zfaw79
Anopheles crucians	-82.133	27.427	Unclassified	GBIF: https://doi.org/10.15468/dl.zfaw79
Anopheles crucians	-82.124	27.613	Unclassified	GBIF: https://doi.org/10.15468/dl.zfaw79
Anopheles crucians	-82.1235	27.6134	Unclassified	GBIF: https://doi.org/10.15468/dl.zfaw79
Anopheles crucians	-82.1227	27.6136	Unclassified	GBIF: https://doi.org/10.15468/dl.zfaw79
Anopheles crucians	-82.117	27.273	Unclassified	GBIF: https://doi.org/10.15468/dl.zfaw79
Anopheles crucians	-82.1165	27.273	Unclassified	GBIF: https://doi.org/10.15468/dl.zfaw79

Anopheles crucians	-82.1	32.1	Unclassified	GBIF: https://doi.org/10.15468/dl.zfaw79
Anopheles crucians	-82.0981	27.6031	Unclassified	GBIF: https://doi.org/10.15468/dl.zfaw79
Anopheles crucians	-82.098	27.603	Unclassified	GBIF: https://doi.org/10.15468/dl.zfaw79
Anopheles crucians	-82.018	29.685	Unclassified	GBIF: https://doi.org/10.15468/dl.zfaw79
Anopheles crucians	-82.017693	29.68469	Unclassified	GBIF: https://doi.org/10.15468/dl.zfaw79
Anopheles crucians	-82.016734	29.681426	Unclassified	GBIF: https://doi.org/10.15468/dl.zfaw79
Anopheles crucians	-82.0156	26.5923	Unclassified	GBIF: https://doi.org/10.15468/dl.zfaw79
Anopheles crucians	-82.01	29.701	Unclassified	GBIF: https://doi.org/10.15468/dl.zfaw79
Anopheles crucians	-81.993431	29.689282	Unclassified	GBIF: https://doi.org/10.15468/dl.zfaw79
Anopheles crucians	-81.993	29.689	Unclassified	GBIF: https://doi.org/10.15468/dl.zfaw79
Anopheles crucians	-81.989	29.706	Unclassified	GBIF: https://doi.org/10.15468/dl.zfaw79
Anopheles crucians	-81.988987	29.706016	Unclassified	GBIF: https://doi.org/10.15468/dl.zfaw79
Anopheles crucians	-81.988	29.725	Unclassified	GBIF: https://doi.org/10.15468/dl.zfaw79
Anopheles crucians	-81.987658	29.725194	Unclassified	GBIF: https://doi.org/10.15468/dl.zfaw79
Anopheles crucians	-81.985398	29.707942	Unclassified	GBIF: https://doi.org/10.15468/dl.zfaw79
Anopheles crucians	-81.985	29.708	Unclassified	GBIF: https://doi.org/10.15468/dl.zfaw79
Anopheles crucians	-81.9783	26.5377	Unclassified	GBIF: https://doi.org/10.15468/dl.zfaw79
Anopheles crucians	-81.971806	29.721861	Unclassified	GBIF: https://doi.org/10.15468/dl.zfaw79
Anopheles crucians	-81.962459	29.678605	Unclassified	GBIF: https://doi.org/10.15468/dl.zfaw79
Anopheles crucians	-81.962	29.679	Unclassified	GBIF: https://doi.org/10.15468/dl.zfaw79
Anopheles crucians	-81.962	29.687	Unclassified	GBIF: https://doi.org/10.15468/dl.zfaw79
Anopheles crucians	-81.955031	29.678667	Unclassified	GBIF: https://doi.org/10.15468/dl.zfaw79
Anopheles crucians	-81.955	29.679	Unclassified	GBIF: https://doi.org/10.15468/dl.zfaw79
Anopheles crucians	-81.955	29.704	Unclassified	GBIF: https://doi.org/10.15468/dl.zfaw79
Anopheles crucians	-81.954643	29.703835	Unclassified	GBIF: https://doi.org/10.15468/dl.zfaw79
Anopheles crucians	-81.9436	32.8061	Unclassified	GBIF: https://doi.org/10.15468/dl.zfaw79
Anopheles crucians	-81.94	29.686	Unclassified	GBIF: https://doi.org/10.15468/dl.zfaw79
Anopheles crucians	-81.939573	29.68552	Unclassified	GBIF: https://doi.org/10.15468/dl.zfaw79
Anopheles crucians	-81.9349	26.4876	Unclassified	GBIF: https://doi.org/10.15468/dl.zfaw79
Anopheles crucians	-81.92	26.5619	Unclassified	GBIF: https://doi.org/10.15468/dl.zfaw79
Anopheles crucians	-81.8974	26.541	Unclassified	GBIF: https://doi.org/10.15468/dl.zfaw79
Anopheles crucians	-81.8963	26.4805	Unclassified	GBIF: https://doi.org/10.15468/dl.zfaw79
Anopheles crucians	-81.8914	26.6784	Unclassified	GBIF: https://doi.org/10.15468/dl.zfaw79
Anopheles crucians	-81.891	26.5197	Unclassified	GBIF: https://doi.org/10.15468/dl.zfaw79
Anopheles crucians	-81.8662	26.675	Unclassified	GBIF: https://doi.org/10.15468/dl.zfaw79
Anopheles crucians	-81.8622	26.4808	Unclassified	GBIF: https://doi.org/10.15468/dl.zfaw79
Anopheles crucians	-81.854	26.5486	Unclassified	GBIF: https://doi.org/10.15468/dl.zfaw79
Anopheles crucians	-81.8441	26.6533	Unclassified	GBIF: https://doi.org/10.15468/dl.zfaw79
Anopheles crucians	-81.8285	26.4757	Unclassified	GBIF: https://doi.org/10.15468/dl.zfaw79
Anopheles crucians	-81.8261	26.485	Unclassified	GBIF: https://doi.org/10.15468/dl.zfaw79
Anopheles crucians	-81.8206	26.4707	Unclassified	GBIF: https://doi.org/10.15468/dl.zfaw79
Anopheles crucians	-81.8206	26.4755	Unclassified	GBIF: https://doi.org/10.15468/dl.zfaw79

Anopheles crucians	-81.8168	26.676	Unclassified	GBIF: https://doi.org/10.15468/dl.zfaw79
Anopheles crucians	-81.816	26.6811	Unclassified	GBIF: https://doi.org/10.15468/dl.zfaw79
Anopheles crucians	-81.813	26.6673	Unclassified	GBIF: https://doi.org/10.15468/dl.zfaw79
Anopheles crucians	-81.8123	26.615	Unclassified	GBIF: https://doi.org/10.15468/dl.zfaw79
Anopheles crucians	-81.8083	26.6559	Unclassified	GBIF: https://doi.org/10.15468/dl.zfaw79
Anopheles crucians	-81.8079	26.6512	Unclassified	GBIF: https://doi.org/10.15468/dl.zfaw79
Anopheles crucians	-81.8074	26.6535	Unclassified	GBIF: https://doi.org/10.15468/dl.zfaw79
Anopheles crucians	-81.8063	26.4851	Unclassified	GBIF: https://doi.org/10.15468/dl.zfaw79
Anopheles crucians	-81.786	26.3455	Unclassified	GBIF: https://doi.org/10.15468/dl.zfaw79
Anopheles crucians	-81.7812	26.4613	Unclassified	GBIF: https://doi.org/10.15468/dl.zfaw79
Anopheles crucians	-81.775	26.3348	Unclassified	GBIF: https://doi.org/10.15468/dl.zfaw79
Anopheles crucians	-81.7692	26.7208	Unclassified	GBIF: https://doi.org/10.15468/dl.zfaw79
Anopheles crucians	-81.7104	26.6394	Unclassified	GBIF: https://doi.org/10.15468/dl.zfaw79
Anopheles crucians	-81.6685	26.62	Unclassified	GBIF: https://doi.org/10.15468/dl.zfaw79
Anopheles crucians	-81.655651	30.332184	Unclassified	GBIF: https://doi.org/10.15468/dl.zfaw79
Anopheles crucians	-81.6528	30.0673	Unclassified	GBIF: https://doi.org/10.15468/dl.zfaw79
Anopheles crucians	-81.6524	30.0815	Unclassified	GBIF: https://doi.org/10.15468/dl.zfaw79
Anopheles crucians	-81.6523	28.5514	Unclassified	GBIF: https://doi.org/10.15468/dl.zfaw79
Anopheles crucians	-81.6519	28.551	Unclassified	GBIF: https://doi.org/10.15468/dl.zfaw79
Anopheles crucians	-81.6507	28.7661	Unclassified	GBIF: https://doi.org/10.15468/dl.zfaw79
Anopheles crucians	-81.6505	28.765	Unclassified	GBIF: https://doi.org/10.15468/dl.zfaw79
Anopheles crucians	-81.6502	28.7659	Unclassified	GBIF: https://doi.org/10.15468/dl.zfaw79
Anopheles crucians	-81.6463	30.0613	Unclassified	GBIF: https://doi.org/10.15468/dl.zfaw79
Anopheles crucians	-81.6443	28.4407	Unclassified	GBIF: https://doi.org/10.15468/dl.zfaw79
Anopheles crucians	-81.64	33.55	Unclassified	GBIF: https://doi.org/10.15468/dl.zfaw79
Anopheles crucians	-81.6387	30.0803	Unclassified	GBIF: https://doi.org/10.15468/dl.zfaw79
Anopheles crucians	-81.6364	28.7427	Unclassified	GBIF: https://doi.org/10.15468/dl.zfaw79
Anopheles crucians	-81.6342	30.1229	Unclassified	GBIF: https://doi.org/10.15468/dl.zfaw79
Anopheles crucians	-81.6339	28.7468	Unclassified	GBIF: https://doi.org/10.15468/dl.zfaw79
Anopheles crucians	-81.6324	30.1169	Unclassified	GBIF: https://doi.org/10.15468/dl.zfaw79
Anopheles crucians	-81.63	33.35	Unclassified	GBIF: https://doi.org/10.15468/dl.zfaw79
Anopheles crucians	-81.6271	28.7435	Unclassified	GBIF: https://doi.org/10.15468/dl.zfaw79
Anopheles crucians	-81.6269	28.7634	Unclassified	GBIF: https://doi.org/10.15468/dl.zfaw79
Anopheles crucians	-81.6265	28.553	Unclassified	GBIF: https://doi.org/10.15468/dl.zfaw79
Anopheles crucians	-81.6255	28.5547	Unclassified	GBIF: https://doi.org/10.15468/dl.zfaw79
Anopheles crucians	-81.6251	28.5539	Unclassified	GBIF: https://doi.org/10.15468/dl.zfaw79
Anopheles crucians	-81.6249	28.556	Unclassified	GBIF: https://doi.org/10.15468/dl.zfaw79
Anopheles crucians	-81.6247	28.5314	Unclassified	GBIF: https://doi.org/10.15468/dl.zfaw79
Anopheles crucians	-81.6237	28.4292	Unclassified	GBIF: https://doi.org/10.15468/dl.zfaw79
Anopheles crucians	-81.6228	28.4275	Unclassified	GBIF: https://doi.org/10.15468/dl.zfaw79
Anopheles crucians	-81.6205	28.5253	Unclassified	GBIF: https://doi.org/10.15468/dl.zfaw79
Anopheles crucians	-81.6185	28.5466	Unclassified	GBIF: https://doi.org/10.15468/dl.zfaw79

Anopheles crucians	-81.618	28.4313	Unclassified	GBIF: https://doi.org/10.15468/dl.zfaw79
Anopheles crucians	-81.6171	28.5538	Unclassified	GBIF: https://doi.org/10.15468/dl.zfaw79
Anopheles crucians	-81.6168	28.4731	Unclassified	GBIF: https://doi.org/10.15468/dl.zfaw79
Anopheles crucians	-81.6162	28.424	Unclassified	GBIF: https://doi.org/10.15468/dl.zfaw79
Anopheles crucians	-81.6158	28.5204	Unclassified	GBIF: https://doi.org/10.15468/dl.zfaw79
Anopheles crucians	-81.6139	30.075	Unclassified	GBIF: https://doi.org/10.15468/dl.zfaw79
Anopheles crucians	-81.6133	28.5615	Unclassified	GBIF: https://doi.org/10.15468/dl.zfaw79
Anopheles crucians	-81.6127	30.0728	Unclassified	GBIF: https://doi.org/10.15468/dl.zfaw79
Anopheles crucians	-81.6123	28.5554	Unclassified	GBIF: https://doi.org/10.15468/dl.zfaw79
Anopheles crucians	-81.6117	28.471	Unclassified	GBIF: https://doi.org/10.15468/dl.zfaw79
Anopheles crucians	-81.6101	28.4304	Unclassified	GBIF: https://doi.org/10.15468/dl.zfaw79
Anopheles crucians	-81.61	28.4304	Unclassified	GBIF: https://doi.org/10.15468/dl.zfaw79
Anopheles crucians	-81.6061	28.734	Unclassified	GBIF: https://doi.org/10.15468/dl.zfaw79
Anopheles crucians	-81.6058	28.5187	Unclassified	GBIF: https://doi.org/10.15468/dl.zfaw79
Anopheles crucians	-81.6038	28.5077	Unclassified	GBIF: https://doi.org/10.15468/dl.zfaw79
Anopheles crucians	-81.6033	28.5414	Unclassified	GBIF: https://doi.org/10.15468/dl.zfaw79
Anopheles crucians	-81.6029	26.6111	Unclassified	GBIF: https://doi.org/10.15468/dl.zfaw79
Anopheles crucians	-81.6021	28.7446	Unclassified	GBIF: https://doi.org/10.15468/dl.zfaw79
Anopheles crucians	-81.6016	28.7416	Unclassified	GBIF: https://doi.org/10.15468/dl.zfaw79
Anopheles crucians	-81.6009	30.1107	Unclassified	GBIF: https://doi.org/10.15468/dl.zfaw79
Anopheles crucians	-81.6003	28.74	Unclassified	GBIF: https://doi.org/10.15468/dl.zfaw79
Anopheles crucians	-81.6002	28.7399	Unclassified	GBIF: https://doi.org/10.15468/dl.zfaw79
Anopheles crucians	-81.6002	28.4497	Unclassified	GBIF: https://doi.org/10.15468/dl.zfaw79
Anopheles crucians	-81.5999	28.5608	Unclassified	GBIF: https://doi.org/10.15468/dl.zfaw79
Anopheles crucians	-81.599	28.5179	Unclassified	GBIF: https://doi.org/10.15468/dl.zfaw79
Anopheles crucians	-81.5981	28.4668	Unclassified	GBIF: https://doi.org/10.15468/dl.zfaw79
Anopheles crucians	-81.5899	28.5526	Unclassified	GBIF: https://doi.org/10.15468/dl.zfaw79
Anopheles crucians	-81.5894	28.464	Unclassified	GBIF: https://doi.org/10.15468/dl.zfaw79
Anopheles crucians	-81.5882	28.5364	Unclassified	GBIF: https://doi.org/10.15468/dl.zfaw79
Anopheles crucians	-81.5875	28.5014	Unclassified	GBIF: https://doi.org/10.15468/dl.zfaw79
Anopheles crucians	-81.5871	29.9891	Unclassified	GBIF: https://doi.org/10.15468/dl.zfaw79
Anopheles crucians	-81.5868	28.5576	Unclassified	GBIF: https://doi.org/10.15468/dl.zfaw79
Anopheles crucians	-81.5864	28.7165	Unclassified	GBIF: https://doi.org/10.15468/dl.zfaw79
Anopheles crucians	-81.5842	28.7166	Unclassified	GBIF: https://doi.org/10.15468/dl.zfaw79
Anopheles crucians	-81.584	28.7151	Unclassified	GBIF: https://doi.org/10.15468/dl.zfaw79
Anopheles crucians	-81.5826	28.4117	Unclassified	GBIF: https://doi.org/10.15468/dl.zfaw79
Anopheles crucians	-81.5825	28.7215	Unclassified	GBIF: https://doi.org/10.15468/dl.zfaw79
Anopheles crucians	-81.5824	29.9514	Unclassified	GBIF: https://doi.org/10.15468/dl.zfaw79
Anopheles crucians	-81.5794	28.4589	Unclassified	GBIF: https://doi.org/10.15468/dl.zfaw79
Anopheles crucians	-81.5787	28.5563	Unclassified	GBIF: https://doi.org/10.15468/dl.zfaw79
Anopheles crucians	-81.5755	29.9482	Unclassified	GBIF: https://doi.org/10.15468/dl.zfaw79
Anopheles crucians	-81.5755	28.4896	Unclassified	GBIF: https://doi.org/10.15468/dl.zfaw79

Anopheles crucians	-81.5748	28.7218	Unclassified	GBIF: https://doi.org/10.15468/dl.zfaw79
Anopheles crucians	-81.57	28.7119	Unclassified	GBIF: https://doi.org/10.15468/dl.zfaw79
Anopheles crucians	-81.5687	28.6943	Unclassified	GBIF: https://doi.org/10.15468/dl.zfaw79
Anopheles crucians	-81.5666	28.6991	Unclassified	GBIF: https://doi.org/10.15468/dl.zfaw79
Anopheles crucians	-81.5611	28.604	Unclassified	GBIF: https://doi.org/10.15468/dl.zfaw79
Anopheles crucians	-81.5611	28.5661	Unclassified	GBIF: https://doi.org/10.15468/dl.zfaw79
Anopheles crucians	-81.5609	28.5425	Unclassified	GBIF: https://doi.org/10.15468/dl.zfaw79
Anopheles crucians	-81.5607	28.4404	Unclassified	GBIF: https://doi.org/10.15468/dl.zfaw79
Anopheles crucians	-81.5599	28.5661	Unclassified	GBIF: https://doi.org/10.15468/dl.zfaw79
Anopheles crucians	-81.5555	28.5262	Unclassified	GBIF: https://doi.org/10.15468/dl.zfaw79
Anopheles crucians	-81.5552	28.7307	Unclassified	GBIF: https://doi.org/10.15468/dl.zfaw79
Anopheles crucians	-81.5522	29.8228	Unclassified	GBIF: https://doi.org/10.15468/dl.zfaw79
Anopheles crucians	-81.5522	28.7408	Unclassified	GBIF: https://doi.org/10.15468/dl.zfaw79
Anopheles crucians	-81.5506	28.7245	Unclassified	GBIF: https://doi.org/10.15468/dl.zfaw79
Anopheles crucians	-81.5498	28.7148	Unclassified	GBIF: https://doi.org/10.15468/dl.zfaw79
Anopheles crucians	-81.5497	28.6865	Unclassified	GBIF: https://doi.org/10.15468/dl.zfaw79
Anopheles crucians	-81.5493	28.6349	Unclassified	GBIF: https://doi.org/10.15468/dl.zfaw79
Anopheles crucians	-81.549	28.6351	Unclassified	GBIF: https://doi.org/10.15468/dl.zfaw79
Anopheles crucians	-81.5466	30.0474	Unclassified	GBIF: https://doi.org/10.15468/dl.zfaw79
Anopheles crucians	-81.5465	28.7101	Unclassified	GBIF: https://doi.org/10.15468/dl.zfaw79
Anopheles crucians	-81.546	28.6797	Unclassified	GBIF: https://doi.org/10.15468/dl.zfaw79
Anopheles crucians	-81.546	28.6799	Unclassified	GBIF: https://doi.org/10.15468/dl.zfaw79
Anopheles crucians	-81.5454	28.6771	Unclassified	GBIF: https://doi.org/10.15468/dl.zfaw79
Anopheles crucians	-81.5441	28.4413	Unclassified	GBIF: https://doi.org/10.15468/dl.zfaw79
Anopheles crucians	-81.544	28.6976	Unclassified	GBIF: https://doi.org/10.15468/dl.zfaw79
Anopheles crucians	-81.5427	28.4422	Unclassified	GBIF: https://doi.org/10.15468/dl.zfaw79
Anopheles crucians	-81.5427	28.671	Unclassified	GBIF: https://doi.org/10.15468/dl.zfaw79
Anopheles crucians	-81.5426	28.4423	Unclassified	GBIF: https://doi.org/10.15468/dl.zfaw79
Anopheles crucians	-81.5408	28.4971	Unclassified	GBIF: https://doi.org/10.15468/dl.zfaw79
Anopheles crucians	-81.5395	28.6978	Unclassified	GBIF: https://doi.org/10.15468/dl.zfaw79
Anopheles crucians	-81.5382	28.6979	Unclassified	GBIF: https://doi.org/10.15468/dl.zfaw79
Anopheles crucians	-81.5353	28.5664	Unclassified	GBIF: https://doi.org/10.15468/dl.zfaw79
Anopheles crucians	-81.5344	28.5834	Unclassified	GBIF: https://doi.org/10.15468/dl.zfaw79
Anopheles crucians	-81.5341	28.666	Unclassified	GBIF: https://doi.org/10.15468/dl.zfaw79
Anopheles crucians	-81.534	28.531	Unclassified	GBIF: https://doi.org/10.15468/dl.zfaw79
Anopheles crucians	-81.5332	28.5924	Unclassified	GBIF: https://doi.org/10.15468/dl.zfaw79
Anopheles crucians	-81.5326	28.4402	Unclassified	GBIF: https://doi.org/10.15468/dl.zfaw79
Anopheles crucians	-81.5323	28.6632	Unclassified	GBIF: https://doi.org/10.15468/dl.zfaw79
Anopheles crucians	-81.5303	28.6035	Unclassified	GBIF: https://doi.org/10.15468/dl.zfaw79
Anopheles crucians	-81.5297	28.4278	Unclassified	GBIF: https://doi.org/10.15468/dl.zfaw79
Anopheles crucians	-81.5296	28.688	Unclassified	GBIF: https://doi.org/10.15468/dl.zfaw79
Anopheles crucians	-81.5274	28.7355	Unclassified	GBIF: https://doi.org/10.15468/dl.zfaw79

Anopheles crucians	-81.5255	28.5948	Unclassified	GBIF: https://doi.org/10.15468/dl.zfaw79
Anopheles crucians	-81.5244	28.3888	Unclassified	GBIF: https://doi.org/10.15468/dl.zfaw79
Anopheles crucians	-81.5244	28.3886	Unclassified	GBIF: https://doi.org/10.15468/dl.zfaw79
Anopheles crucians	-81.5233	28.5539	Unclassified	GBIF: https://doi.org/10.15468/dl.zfaw79
Anopheles crucians	-81.523	28.604	Unclassified	GBIF: https://doi.org/10.15468/dl.zfaw79
Anopheles crucians	-81.5224	28.5532	Unclassified	GBIF: https://doi.org/10.15468/dl.zfaw79
Anopheles crucians	-81.5223	28.6186	Unclassified	GBIF: https://doi.org/10.15468/dl.zfaw79
Anopheles crucians	-81.5221	28.5553	Unclassified	GBIF: https://doi.org/10.15468/dl.zfaw79
Anopheles crucians	-81.522	28.6284	Unclassified	GBIF: https://doi.org/10.15468/dl.zfaw79
Anopheles crucians	-81.5217	28.6972	Unclassified	GBIF: https://doi.org/10.15468/dl.zfaw79
Anopheles crucians	-81.5213	28.535	Unclassified	GBIF: https://doi.org/10.15468/dl.zfaw79
Anopheles crucians	-81.5213	28.605	Unclassified	GBIF: https://doi.org/10.15468/dl.zfaw79
Anopheles crucians	-81.5212	28.57	Unclassified	GBIF: https://doi.org/10.15468/dl.zfaw79
Anopheles crucians	-81.521	28.5325	Unclassified	GBIF: https://doi.org/10.15468/dl.zfaw79
Anopheles crucians	-81.5206	28.6576	Unclassified	GBIF: https://doi.org/10.15468/dl.zfaw79
Anopheles crucians	-81.52	28.6069	Unclassified	GBIF: https://doi.org/10.15468/dl.zfaw79
Anopheles crucians	-81.5199	28.6071	Unclassified	GBIF: https://doi.org/10.15468/dl.zfaw79
Anopheles crucians	-81.5182	28.5837	Unclassified	GBIF: https://doi.org/10.15468/dl.zfaw79
Anopheles crucians	-81.518	28.5834	Unclassified	GBIF: https://doi.org/10.15468/dl.zfaw79
Anopheles crucians	-81.5173	29.9808	Unclassified	GBIF: https://doi.org/10.15468/dl.zfaw79
Anopheles crucians	-81.5162	28.71	Unclassified	GBIF: https://doi.org/10.15468/dl.zfaw79
Anopheles crucians	-81.5159	28.5915	Unclassified	GBIF: https://doi.org/10.15468/dl.zfaw79
Anopheles crucians	-81.5156	28.6533	Unclassified	GBIF: https://doi.org/10.15468/dl.zfaw79
Anopheles crucians	-81.5151	28.564	Unclassified	GBIF: https://doi.org/10.15468/dl.zfaw79
Anopheles crucians	-81.5147	28.6844	Unclassified	GBIF: https://doi.org/10.15468/dl.zfaw79
Anopheles crucians	-81.5134	28.7519	Unclassified	GBIF: https://doi.org/10.15468/dl.zfaw79
Anopheles crucians	-81.5132	28.5482	Unclassified	GBIF: https://doi.org/10.15468/dl.zfaw79
Anopheles crucians	-81.5111	28.6043	Unclassified	GBIF: https://doi.org/10.15468/dl.zfaw79
Anopheles crucians	-81.5107	28.5904	Unclassified	GBIF: https://doi.org/10.15468/dl.zfaw79
Anopheles crucians	-81.5106	29.8467	Unclassified	GBIF: https://doi.org/10.15468/dl.zfaw79
Anopheles crucians	-81.5106	28.5896	Unclassified	GBIF: https://doi.org/10.15468/dl.zfaw79
Anopheles crucians	-81.5104	28.6539	Unclassified	GBIF: https://doi.org/10.15468/dl.zfaw79
Anopheles crucians	-81.5089	28.5323	Unclassified	GBIF: https://doi.org/10.15468/dl.zfaw79
Anopheles crucians	-81.5079	28.7626	Unclassified	GBIF: https://doi.org/10.15468/dl.zfaw79
Anopheles crucians	-81.5064	28.691	Unclassified	GBIF: https://doi.org/10.15468/dl.zfaw79
Anopheles crucians	-81.5062	29.7256	Unclassified	GBIF: https://doi.org/10.15468/dl.zfaw79
Anopheles crucians	-81.5062	28.591	Unclassified	GBIF: https://doi.org/10.15468/dl.zfaw79
Anopheles crucians	-81.5057	28.6032	Unclassified	GBIF: https://doi.org/10.15468/dl.zfaw79
Anopheles crucians	-81.5055	28.7097	Unclassified	GBIF: https://doi.org/10.15468/dl.zfaw79
Anopheles crucians	-81.505	28.639	Unclassified	GBIF: https://doi.org/10.15468/dl.zfaw79
Anopheles crucians	-81.5049	29.7127	Unclassified	GBIF: https://doi.org/10.15468/dl.zfaw79
Anopheles crucians	-81.5036	28.6372	Unclassified	GBIF: https://doi.org/10.15468/dl.zfaw79

Anopheles crucians	-81.5033	28.4513	Unclassified	GBIF: https://doi.org/10.15468/dl.zfaw79
Anopheles crucians	-81.5023	28.7585	Unclassified	GBIF: https://doi.org/10.15468/dl.zfaw79
Anopheles crucians	-81.5023	28.5549	Unclassified	GBIF: https://doi.org/10.15468/dl.zfaw79
Anopheles crucians	-81.5023	28.7583	Unclassified	GBIF: https://doi.org/10.15468/dl.zfaw79
Anopheles crucians	-81.5021	28.686	Unclassified	GBIF: https://doi.org/10.15468/dl.zfaw79
Anopheles crucians	-81.5014	28.6278	Unclassified	GBIF: https://doi.org/10.15468/dl.zfaw79
Anopheles crucians	-81.5013	29.8409	Unclassified	GBIF: https://doi.org/10.15468/dl.zfaw79
Anopheles crucians	-81.5001	28.5151	Unclassified	GBIF: https://doi.org/10.15468/dl.zfaw79
Anopheles crucians	-81.5001	28.6311	Unclassified	GBIF: https://doi.org/10.15468/dl.zfaw79
Anopheles crucians	-81.4996	28.4517	Unclassified	GBIF: https://doi.org/10.15468/dl.zfaw79
Anopheles crucians	-81.4995	28.4518	Unclassified	GBIF: https://doi.org/10.15468/dl.zfaw79
Anopheles crucians	-81.4982	28.6818	Unclassified	GBIF: https://doi.org/10.15468/dl.zfaw79
Anopheles crucians	-81.4967	28.7602	Unclassified	GBIF: https://doi.org/10.15468/dl.zfaw79
Anopheles crucians	-81.4965	28.7603	Unclassified	GBIF: https://doi.org/10.15468/dl.zfaw79
Anopheles crucians	-81.4958	28.7601	Unclassified	GBIF: https://doi.org/10.15468/dl.zfaw79
Anopheles crucians	-81.4927	28.5904	Unclassified	GBIF: https://doi.org/10.15468/dl.zfaw79
Anopheles crucians	-81.4924	29.9138	Unclassified	GBIF: https://doi.org/10.15468/dl.zfaw79
Anopheles crucians	-81.4916	28.5935	Unclassified	GBIF: https://doi.org/10.15468/dl.zfaw79
Anopheles crucians	-81.491489	31.149953	Unclassified	GBIF: https://doi.org/10.15468/dl.zfaw79
Anopheles crucians	-81.4903	28.4087	Unclassified	GBIF: https://doi.org/10.15468/dl.zfaw79
Anopheles crucians	-81.4902	29.9074	Unclassified	GBIF: https://doi.org/10.15468/dl.zfaw79
Anopheles crucians	-81.4859	28.5363	Unclassified	GBIF: https://doi.org/10.15468/dl.zfaw79
Anopheles crucians	-81.4841	28.5101	Unclassified	GBIF: https://doi.org/10.15468/dl.zfaw79
Anopheles crucians	-81.484	28.5102	Unclassified	GBIF: https://doi.org/10.15468/dl.zfaw79
Anopheles crucians	-81.4832	28.5232	Unclassified	GBIF: https://doi.org/10.15468/dl.zfaw79
Anopheles crucians	-81.4828	28.5099	Unclassified	GBIF: https://doi.org/10.15468/dl.zfaw79
Anopheles crucians	-81.481	29.9774	Unclassified	GBIF: https://doi.org/10.15468/dl.zfaw79
Anopheles crucians	-81.481	28.5973	Unclassified	GBIF: https://doi.org/10.15468/dl.zfaw79
Anopheles crucians	-81.4808	29.9781	Unclassified	GBIF: https://doi.org/10.15468/dl.zfaw79
Anopheles crucians	-81.4807	29.9811	Unclassified	GBIF: https://doi.org/10.15468/dl.zfaw79
Anopheles crucians	-81.4807	28.536	Unclassified	GBIF: https://doi.org/10.15468/dl.zfaw79
Anopheles crucians	-81.4793	28.4791	Unclassified	GBIF: https://doi.org/10.15468/dl.zfaw79
Anopheles crucians	-81.4793	33.2874	Unclassified	GBIF: https://doi.org/10.15468/dl.zfaw79
Anopheles crucians	-81.4747	28.4816	Unclassified	GBIF: https://doi.org/10.15468/dl.zfaw79
Anopheles crucians	-81.4722	29.742	Unclassified	GBIF: https://doi.org/10.15468/dl.zfaw79
Anopheles crucians	-81.4721	29.6588	Unclassified	GBIF: https://doi.org/10.15468/dl.zfaw79
Anopheles crucians	-81.472	28.5939	Unclassified	GBIF: https://doi.org/10.15468/dl.zfaw79
Anopheles crucians	-81.4716	28.5741	Unclassified	GBIF: https://doi.org/10.15468/dl.zfaw79
Anopheles crucians	-81.4715	29.659	Unclassified	GBIF: https://doi.org/10.15468/dl.zfaw79
Anopheles crucians	-81.4715	28.5744	Unclassified	GBIF: https://doi.org/10.15468/dl.zfaw79
Anopheles crucians	-81.4709	28.6626	Unclassified	GBIF: https://doi.org/10.15468/dl.zfaw79
Anopheles crucians	-81.47	31.36	Unclassified	GBIF: https://doi.org/10.15468/dl.zfaw79

Anopheles crucians	-81.4662	28.6953	Unclassified	GBIF: https://doi.org/10.15468/dl.zfaw79
Anopheles crucians	-81.4649	29.2418	Unclassified	GBIF: https://doi.org/10.15468/dl.zfaw79
Anopheles crucians	-81.4642	28.4366	Unclassified	GBIF: https://doi.org/10.15468/dl.zfaw79
Anopheles crucians	-81.4613	29.8608	Unclassified	GBIF: https://doi.org/10.15468/dl.zfaw79
Anopheles crucians	-81.4586	28.6328	Unclassified	GBIF: https://doi.org/10.15468/dl.zfaw79
Anopheles crucians	-81.4564	28.5019	Unclassified	GBIF: https://doi.org/10.15468/dl.zfaw79
Anopheles crucians	-81.455	28.5011	Unclassified	GBIF: https://doi.org/10.15468/dl.zfaw79
Anopheles crucians	-81.4534	29.7675	Unclassified	GBIF: https://doi.org/10.15468/dl.zfaw79
Anopheles crucians	-81.4529	28.4064	Unclassified	GBIF: https://doi.org/10.15468/dl.zfaw79
Anopheles crucians	-81.446	28.4311	Unclassified	GBIF: https://doi.org/10.15468/dl.zfaw79
Anopheles crucians	-81.4454	28.5515	Unclassified	GBIF: https://doi.org/10.15468/dl.zfaw79
Anopheles crucians	-81.4449	28.5518	Unclassified	GBIF: https://doi.org/10.15468/dl.zfaw79
Anopheles crucians	-81.4444	28.5119	Unclassified	GBIF: https://doi.org/10.15468/dl.zfaw79
Anopheles crucians	-81.4441	29.6491	Unclassified	GBIF: https://doi.org/10.15468/dl.zfaw79
Anopheles crucians	-81.4439	28.551	Unclassified	GBIF: https://doi.org/10.15468/dl.zfaw79
Anopheles crucians	-81.4418	28.5403	Unclassified	GBIF: https://doi.org/10.15468/dl.zfaw79
Anopheles crucians	-81.4417	28.5439	Unclassified	GBIF: https://doi.org/10.15468/dl.zfaw79
Anopheles crucians	-81.4399	29.6345	Unclassified	GBIF: https://doi.org/10.15468/dl.zfaw79
Anopheles crucians	-81.43619	28.12505	Unclassified	GBIF: https://doi.org/10.15468/dl.zfaw79
Anopheles crucians	-81.436	28.125	Unclassified	GBIF: https://doi.org/10.15468/dl.zfaw79
Anopheles crucians	-81.4359	28.4654	Unclassified	GBIF: https://doi.org/10.15468/dl.zfaw79
Anopheles crucians	-81.4353	30.0907	Unclassified	GBIF: https://doi.org/10.15468/dl.zfaw79
Anopheles crucians	-81.4349	28.3762	Unclassified	GBIF: https://doi.org/10.15468/dl.zfaw79
Anopheles crucians	-81.4333	28.6291	Unclassified	GBIF: https://doi.org/10.15468/dl.zfaw79
Anopheles crucians	-81.4328	30.2479	Unclassified	GBIF: https://doi.org/10.15468/dl.zfaw79
Anopheles crucians	-81.4328	28.4773	Unclassified	GBIF: https://doi.org/10.15468/dl.zfaw79
Anopheles crucians	-81.4302	28.129	Unclassified	GBIF: https://doi.org/10.15468/dl.zfaw79
Anopheles crucians	-81.4293	28.3602	Unclassified	GBIF: https://doi.org/10.15468/dl.zfaw79
Anopheles crucians	-81.42833	28.08583	Unclassified	GBIF: https://doi.org/10.15468/dl.zfaw79
Anopheles crucians	-81.428	28.086	Unclassified	GBIF: https://doi.org/10.15468/dl.zfaw79
Anopheles crucians	-81.426351	28.129725	Unclassified	GBIF: https://doi.org/10.15468/dl.zfaw79
Anopheles crucians	-81.426	28.13	Unclassified	GBIF: https://doi.org/10.15468/dl.zfaw79
Anopheles crucians	-81.4254	28.4395	Unclassified	GBIF: https://doi.org/10.15468/dl.zfaw79
Anopheles crucians	-81.4231	28.4934	Unclassified	GBIF: https://doi.org/10.15468/dl.zfaw79
Anopheles crucians	-81.4224	28.4853	Unclassified	GBIF: https://doi.org/10.15468/dl.zfaw79
Anopheles crucians	-81.422	29.187	Unclassified	GBIF: https://doi.org/10.15468/dl.zfaw79
Anopheles crucians	-81.422	28.044	Unclassified	GBIF: https://doi.org/10.15468/dl.zfaw79
Anopheles crucians	-81.42	30.39	Unclassified	GBIF: https://doi.org/10.15468/dl.zfaw79
Anopheles crucians	-81.4198	28.371	Unclassified	GBIF: https://doi.org/10.15468/dl.zfaw79
Anopheles crucians	-81.4195	28.3714	Unclassified	GBIF: https://doi.org/10.15468/dl.zfaw79
Anopheles crucians	-81.419	28.086	Unclassified	GBIF: https://doi.org/10.15468/dl.zfaw79
Anopheles crucians	-81.418864	28.086413	Unclassified	GBIF: https://doi.org/10.15468/dl.zfaw79

Anopheles crucians	-81.4184	30.1252	Unclassified	GBIF: https://doi.org/10.15468/dl.zfaw79
Anopheles crucians	-81.4182	30.2362	Unclassified	GBIF: https://doi.org/10.15468/dl.zfaw79
Anopheles crucians	-81.4158	28.6076	Unclassified	GBIF: https://doi.org/10.15468/dl.zfaw79
Anopheles crucians	-81.4156	28.4972	Unclassified	GBIF: https://doi.org/10.15468/dl.zfaw79
Anopheles crucians	-81.412	28.075	Unclassified	GBIF: https://doi.org/10.15468/dl.zfaw79
Anopheles crucians	-81.412	28.08	Unclassified	GBIF: https://doi.org/10.15468/dl.zfaw79
Anopheles crucians	-81.411803	28.080203	Unclassified	GBIF: https://doi.org/10.15468/dl.zfaw79
Anopheles crucians	-81.411802	28.074669	Unclassified	GBIF: https://doi.org/10.15468/dl.zfaw79
Anopheles crucians	-81.4118	28.07467	Unclassified	GBIF: https://doi.org/10.15468/dl.zfaw79
Anopheles crucians	-81.4067	28.6273	Unclassified	GBIF: https://doi.org/10.15468/dl.zfaw79
Anopheles crucians	-81.40601	33.36684	Unclassified	GBIF: https://doi.org/10.15468/dl.zfaw79
Anopheles crucians	-81.406	28.068	Unclassified	GBIF: https://doi.org/10.15468/dl.zfaw79
Anopheles crucians	-81.405949	28.068275	Unclassified	GBIF: https://doi.org/10.15468/dl.zfaw79
Anopheles crucians	-81.4035	30.1941	Unclassified	GBIF: https://doi.org/10.15468/dl.zfaw79
Anopheles crucians	-81.402859	28.062331	Unclassified	GBIF: https://doi.org/10.15468/dl.zfaw79
Anopheles crucians	-81.402	30.2515	Unclassified	GBIF: https://doi.org/10.15468/dl.zfaw79
Anopheles crucians	-81.4009	30.2517	Unclassified	GBIF: https://doi.org/10.15468/dl.zfaw79
Anopheles crucians	-81.4008	29.8121	Unclassified	GBIF: https://doi.org/10.15468/dl.zfaw79
Anopheles crucians	-81.4006	28.6039	Unclassified	GBIF: https://doi.org/10.15468/dl.zfaw79
Anopheles crucians	-81.3991	28.3997	Unclassified	GBIF: https://doi.org/10.15468/dl.zfaw79
Anopheles crucians	-81.3978	28.6011	Unclassified	GBIF: https://doi.org/10.15468/dl.zfaw79
Anopheles crucians	-81.3971	30.1834	Unclassified	GBIF: https://doi.org/10.15468/dl.zfaw79
Anopheles crucians	-81.3963	30.1833	Unclassified	GBIF: https://doi.org/10.15468/dl.zfaw79
Anopheles crucians	-81.3962	28.3824	Unclassified	GBIF: https://doi.org/10.15468/dl.zfaw79
Anopheles crucians	-81.3955	30.1782	Unclassified	GBIF: https://doi.org/10.15468/dl.zfaw79
Anopheles crucians	-81.3935	30.0181	Unclassified	GBIF: https://doi.org/10.15468/dl.zfaw79
Anopheles crucians	-81.39347	28.08186	Unclassified	GBIF: https://doi.org/10.15468/dl.zfaw79
Anopheles crucians	-81.39347	28.081862	Unclassified	GBIF: https://doi.org/10.15468/dl.zfaw79
Anopheles crucians	-81.3921	28.4662	Unclassified	GBIF: https://doi.org/10.15468/dl.zfaw79
Anopheles crucians	-81.3921	28.4663	Unclassified	GBIF: https://doi.org/10.15468/dl.zfaw79
Anopheles crucians	-81.389999	28.056904	Unclassified	GBIF: https://doi.org/10.15468/dl.zfaw79
Anopheles crucians	-81.387	28.084	Unclassified	GBIF: https://doi.org/10.15468/dl.zfaw79
Anopheles crucians	-81.3869	28.5985	Unclassified	GBIF: https://doi.org/10.15468/dl.zfaw79
Anopheles crucians	-81.386763	28.083785	Unclassified	GBIF: https://doi.org/10.15468/dl.zfaw79
Anopheles crucians	-81.3857	28.3795	Unclassified	GBIF: https://doi.org/10.15468/dl.zfaw79
Anopheles crucians	-81.3856	28.3796	Unclassified	GBIF: https://doi.org/10.15468/dl.zfaw79
Anopheles crucians	-81.385	30.2141	Unclassified	GBIF: https://doi.org/10.15468/dl.zfaw79
Anopheles crucians	-81.3819	28.4804	Unclassified	GBIF: https://doi.org/10.15468/dl.zfaw79
Anopheles crucians	-81.3795	29.8143	Unclassified	GBIF: https://doi.org/10.15468/dl.zfaw79
Anopheles crucians	-81.3786	28.6264	Unclassified	GBIF: https://doi.org/10.15468/dl.zfaw79
Anopheles crucians	-81.3783	30.203	Unclassified	GBIF: https://doi.org/10.15468/dl.zfaw79
Anopheles crucians	-81.3758	29.9075	Unclassified	GBIF: https://doi.org/10.15468/dl.zfaw79

Anopheles crucians	-81.3748	30.03	Unclassified	GBIF: https://doi.org/10.15468/dl.zfaw79
Anopheles crucians	-81.3743	28.6369	Unclassified	GBIF: https://doi.org/10.15468/dl.zfaw79
Anopheles crucians	-81.3704	28.4575	Unclassified	GBIF: https://doi.org/10.15468/dl.zfaw79
Anopheles crucians	-81.3701	29.9996	Unclassified	GBIF: https://doi.org/10.15468/dl.zfaw79
Anopheles crucians	-81.369	28.5945	Unclassified	GBIF: https://doi.org/10.15468/dl.zfaw79
Anopheles crucians	-81.3662	30.1586	Unclassified	GBIF: https://doi.org/10.15468/dl.zfaw79
Anopheles crucians	-81.3656	29.8544	Unclassified	GBIF: https://doi.org/10.15468/dl.zfaw79
Anopheles crucians	-81.3645	30.1476	Unclassified	GBIF: https://doi.org/10.15468/dl.zfaw79
Anopheles crucians	-81.3635	28.4279	Unclassified	GBIF: https://doi.org/10.15468/dl.zfaw79
Anopheles crucians	-81.3609	28.5168	Unclassified	GBIF: https://doi.org/10.15468/dl.zfaw79
Anopheles crucians	-81.3608	28.5167	Unclassified	GBIF: https://doi.org/10.15468/dl.zfaw79
Anopheles crucians	-81.3587	28.5443	Unclassified	GBIF: https://doi.org/10.15468/dl.zfaw79
Anopheles crucians	-81.358	28.4521	Unclassified	GBIF: https://doi.org/10.15468/dl.zfaw79
Anopheles crucians	-81.358	33.244	Unclassified	GBIF: https://doi.org/10.15468/dl.zfaw79
Anopheles crucians	-81.3574	28.5824	Unclassified	GBIF: https://doi.org/10.15468/dl.zfaw79
Anopheles crucians	-81.3559	28.9887	Unclassified	GBIF: https://doi.org/10.15468/dl.zfaw79
Anopheles crucians	-81.3559	29.848	Unclassified	GBIF: https://doi.org/10.15468/dl.zfaw79
Anopheles crucians	-81.3559	29.8482	Unclassified	GBIF: https://doi.org/10.15468/dl.zfaw79
Anopheles crucians	-81.3532	29.9596	Unclassified	GBIF: https://doi.org/10.15468/dl.zfaw79
Anopheles crucians	-81.3521	28.3923	Unclassified	GBIF: https://doi.org/10.15468/dl.zfaw79
Anopheles crucians	-81.3438	29.8143	Unclassified	GBIF: https://doi.org/10.15468/dl.zfaw79
Anopheles crucians	-81.3428	29.8913	Unclassified	GBIF: https://doi.org/10.15468/dl.zfaw79
Anopheles crucians	-81.3424	28.516	Unclassified	GBIF: https://doi.org/10.15468/dl.zfaw79
Anopheles crucians	-81.3404	28.4438	Unclassified	GBIF: https://doi.org/10.15468/dl.zfaw79
Anopheles crucians	-81.3399	29.8747	Unclassified	GBIF: https://doi.org/10.15468/dl.zfaw79
Anopheles crucians	-81.3395	28.6337	Unclassified	GBIF: https://doi.org/10.15468/dl.zfaw79
Anopheles crucians	-81.3391	28.463	Unclassified	GBIF: https://doi.org/10.15468/dl.zfaw79
Anopheles crucians	-81.3387	28.4619	Unclassified	GBIF: https://doi.org/10.15468/dl.zfaw79
Anopheles crucians	-81.3377895	27.1894301	Unclassified	GBIF: https://doi.org/10.15468/dl.zfaw79
Anopheles crucians	-81.337	28.3528	Unclassified	GBIF: https://doi.org/10.15468/dl.zfaw79
Anopheles crucians	-81.3368	29.9168	Unclassified	GBIF: https://doi.org/10.15468/dl.zfaw79
Anopheles crucians	-81.3362	28.6281	Unclassified	GBIF: https://doi.org/10.15468/dl.zfaw79
Anopheles crucians	-81.3355	29.9522	Unclassified	GBIF: https://doi.org/10.15468/dl.zfaw79
Anopheles crucians	-81.3352	29.8932	Unclassified	GBIF: https://doi.org/10.15468/dl.zfaw79
Anopheles crucians	-81.3347	33.3059	Unclassified	GBIF: https://doi.org/10.15468/dl.zfaw79
Anopheles crucians	-81.3337	29.8005	Unclassified	GBIF: https://doi.org/10.15468/dl.zfaw79
Anopheles crucians	-81.333	29.8898	Unclassified	GBIF: https://doi.org/10.15468/dl.zfaw79
Anopheles crucians	-81.3329	29.6544	Unclassified	GBIF: https://doi.org/10.15468/dl.zfaw79
Anopheles crucians	-81.3311	29.9106	Unclassified	GBIF: https://doi.org/10.15468/dl.zfaw79
Anopheles crucians	-81.3297	28.4842	Unclassified	GBIF: https://doi.org/10.15468/dl.zfaw79
Anopheles crucians	-81.3289	28.4026	Unclassified	GBIF: https://doi.org/10.15468/dl.zfaw79
Anopheles crucians	-81.3289	29.8953	Unclassified	GBIF: https://doi.org/10.15468/dl.zfaw79

Anopheles crucians	-81.3275	30.0236	Unclassified	GBIF: https://doi.org/10.15468/dl.zfaw79
Anopheles crucians	-81.3267	28.3597	Unclassified	GBIF: https://doi.org/10.15468/dl.zfaw79
Anopheles crucians	-81.3265	29.9294	Unclassified	GBIF: https://doi.org/10.15468/dl.zfaw79
Anopheles crucians	-81.3248	29.7899	Unclassified	GBIF: https://doi.org/10.15468/dl.zfaw79
Anopheles crucians	-81.3248	28.3606	Unclassified	GBIF: https://doi.org/10.15468/dl.zfaw79
Anopheles crucians	-81.3247	28.5768	Unclassified	GBIF: https://doi.org/10.15468/dl.zfaw79
Anopheles crucians	-81.3222	28.8648	Unclassified	GBIF: https://doi.org/10.15468/dl.zfaw79
Anopheles crucians	-81.3192	29.8521	Unclassified	GBIF: https://doi.org/10.15468/dl.zfaw79
Anopheles crucians	-81.3187	28.5656	Unclassified	GBIF: https://doi.org/10.15468/dl.zfaw79
Anopheles crucians	-81.3173	29.9201	Unclassified	GBIF: https://doi.org/10.15468/dl.zfaw79
Anopheles crucians	-81.3171	28.5612	Unclassified	GBIF: https://doi.org/10.15468/dl.zfaw79
Anopheles crucians	-81.317	28.4609	Unclassified	GBIF: https://doi.org/10.15468/dl.zfaw79
Anopheles crucians	-81.3168	29.8933	Unclassified	GBIF: https://doi.org/10.15468/dl.zfaw79
Anopheles crucians	-81.315	29.8523	Unclassified	GBIF: https://doi.org/10.15468/dl.zfaw79
Anopheles crucians	-81.3146	29.83	Unclassified	GBIF: https://doi.org/10.15468/dl.zfaw79
Anopheles crucians	-81.3138	28.4786	Unclassified	GBIF: https://doi.org/10.15468/dl.zfaw79
Anopheles crucians	-81.3136	29.8426	Unclassified	GBIF: https://doi.org/10.15468/dl.zfaw79
Anopheles crucians	-81.313	28.5455	Unclassified	GBIF: https://doi.org/10.15468/dl.zfaw79
Anopheles crucians	-81.3125	28.8641	Unclassified	GBIF: https://doi.org/10.15468/dl.zfaw79
Anopheles crucians	-81.3117	28.5664	Unclassified	GBIF: https://doi.org/10.15468/dl.zfaw79
Anopheles crucians	-81.3107	29.8356	Unclassified	GBIF: https://doi.org/10.15468/dl.zfaw79
Anopheles crucians	-81.3042	29.0267	Unclassified	GBIF: https://doi.org/10.15468/dl.zfaw79
Anopheles crucians	-81.3036	29.8177	Unclassified	GBIF: https://doi.org/10.15468/dl.zfaw79
Anopheles crucians	-81.3035	29.7607	Unclassified	GBIF: https://doi.org/10.15468/dl.zfaw79
Anopheles crucians	-81.3034	29.7627	Unclassified	GBIF: https://doi.org/10.15468/dl.zfaw79
Anopheles crucians	-81.3012	29.1516	Unclassified	GBIF: https://doi.org/10.15468/dl.zfaw79
Anopheles crucians	-81.3008	28.5859	Unclassified	GBIF: https://doi.org/10.15468/dl.zfaw79
Anopheles crucians	-81.3004	28.9692	Unclassified	GBIF: https://doi.org/10.15468/dl.zfaw79
Anopheles crucians	-81.2965	29.9247	Unclassified	GBIF: https://doi.org/10.15468/dl.zfaw79
Anopheles crucians	-81.2957	28.4957	Unclassified	GBIF: https://doi.org/10.15468/dl.zfaw79
Anopheles crucians	-81.2956	28.3503	Unclassified	GBIF: https://doi.org/10.15468/dl.zfaw79
Anopheles crucians	-81.295	28.8742	Unclassified	GBIF: https://doi.org/10.15468/dl.zfaw79
Anopheles crucians	-81.2935	28.5141	Unclassified	GBIF: https://doi.org/10.15468/dl.zfaw79
Anopheles crucians	-81.2885	29.8877	Unclassified	GBIF: https://doi.org/10.15468/dl.zfaw79
Anopheles crucians	-81.2884	29.8877	Unclassified	GBIF: https://doi.org/10.15468/dl.zfaw79
Anopheles crucians	-81.2883	28.5345	Unclassified	GBIF: https://doi.org/10.15468/dl.zfaw79
Anopheles crucians	-81.288	29.8733	Unclassified	GBIF: https://doi.org/10.15468/dl.zfaw79
Anopheles crucians	-81.288	29.8734	Unclassified	GBIF: https://doi.org/10.15468/dl.zfaw79
Anopheles crucians	-81.2777	29.858	Unclassified	GBIF: https://doi.org/10.15468/dl.zfaw79
Anopheles crucians	-81.2752	28.4914	Unclassified	GBIF: https://doi.org/10.15468/dl.zfaw79
Anopheles crucians	-81.2745	28.5211	Unclassified	GBIF: https://doi.org/10.15468/dl.zfaw79
Anopheles crucians	-81.274	29.842	Unclassified	GBIF: https://doi.org/10.15468/dl.zfaw79

Anopheles crucians	-81.2739	29.842	Unclassified	GBIF: https://doi.org/10.15468/dl.zfaw79
Anopheles crucians	-81.2708	28.4885	Unclassified	GBIF: https://doi.org/10.15468/dl.zfaw79
Anopheles crucians	-81.2695	28.5424	Unclassified	GBIF: https://doi.org/10.15468/dl.zfaw79
Anopheles crucians	-81.2693	29.8413	Unclassified	GBIF: https://doi.org/10.15468/dl.zfaw79
Anopheles crucians	-81.2682	28.5842	Unclassified	GBIF: https://doi.org/10.15468/dl.zfaw79
Anopheles crucians	-81.2635	28.5095	Unclassified	GBIF: https://doi.org/10.15468/dl.zfaw79
Anopheles crucians	-81.263	28.4903	Unclassified	GBIF: https://doi.org/10.15468/dl.zfaw79
Anopheles crucians	-81.2626	28.4306	Unclassified	GBIF: https://doi.org/10.15468/dl.zfaw79
Anopheles crucians	-81.2624	28.4305	Unclassified	GBIF: https://doi.org/10.15468/dl.zfaw79
Anopheles crucians	-81.2605	28.3908	Unclassified	GBIF: https://doi.org/10.15468/dl.zfaw79
Anopheles crucians	-81.2602	28.3936	Unclassified	GBIF: https://doi.org/10.15468/dl.zfaw79
Anopheles crucians	-81.2583	28.5421	Unclassified	GBIF: https://doi.org/10.15468/dl.zfaw79
Anopheles crucians	-81.2562	28.3734	Unclassified	GBIF: https://doi.org/10.15468/dl.zfaw79
Anopheles crucians	-81.2546	28.5846	Unclassified	GBIF: https://doi.org/10.15468/dl.zfaw79
Anopheles crucians	-81.2545	28.5845	Unclassified	GBIF: https://doi.org/10.15468/dl.zfaw79
Anopheles crucians	-81.2455	28.482	Unclassified	GBIF: https://doi.org/10.15468/dl.zfaw79
Anopheles crucians	-81.2428	28.9775	Unclassified	GBIF: https://doi.org/10.15468/dl.zfaw79
Anopheles crucians	-81.2411	28.5859	Unclassified	GBIF: https://doi.org/10.15468/dl.zfaw79
Anopheles crucians	-81.2389	28.5769	Unclassified	GBIF: https://doi.org/10.15468/dl.zfaw79
Anopheles crucians	-81.2371	28.5146	Unclassified	GBIF: https://doi.org/10.15468/dl.zfaw79
Anopheles crucians	-81.2353	28.5914	Unclassified	GBIF: https://doi.org/10.15468/dl.zfaw79
Anopheles crucians	-81.2325	28.4118	Unclassified	GBIF: https://doi.org/10.15468/dl.zfaw79
Anopheles crucians	-81.231	29.2135	Unclassified	GBIF: https://doi.org/10.15468/dl.zfaw79
Anopheles crucians	-81.2295	28.6058	Unclassified	GBIF: https://doi.org/10.15468/dl.zfaw79
Anopheles crucians	-81.2295	28.5794	Unclassified	GBIF: https://doi.org/10.15468/dl.zfaw79
Anopheles crucians	-81.2294	28.6058	Unclassified	GBIF: https://doi.org/10.15468/dl.zfaw79
Anopheles crucians	-81.2291	28.978	Unclassified	GBIF: https://doi.org/10.15468/dl.zfaw79
Anopheles crucians	-81.2245	28.8728	Unclassified	GBIF: https://doi.org/10.15468/dl.zfaw79
Anopheles crucians	-81.2217	29.0102	Unclassified	GBIF: https://doi.org/10.15468/dl.zfaw79
Anopheles crucians	-81.2182	28.5812	Unclassified	GBIF: https://doi.org/10.15468/dl.zfaw79
Anopheles crucians	-81.2113	28.3594	Unclassified	GBIF: https://doi.org/10.15468/dl.zfaw79
Anopheles crucians	-81.2106	28.4077	Unclassified	GBIF: https://doi.org/10.15468/dl.zfaw79
Anopheles crucians	-81.2079	28.4824	Unclassified	GBIF: https://doi.org/10.15468/dl.zfaw79
Anopheles crucians	-81.2026	28.5653	Unclassified	GBIF: https://doi.org/10.15468/dl.zfaw79
Anopheles crucians	-81.197	28.5183	Unclassified	GBIF: https://doi.org/10.15468/dl.zfaw79
Anopheles crucians	-81.1942	28.5151	Unclassified	GBIF: https://doi.org/10.15468/dl.zfaw79
Anopheles crucians	-81.193	28.5925	Unclassified	GBIF: https://doi.org/10.15468/dl.zfaw79
Anopheles crucians	-81.1901	28.3789	Unclassified	GBIF: https://doi.org/10.15468/dl.zfaw79
Anopheles crucians	-81.1896	28.3809	Unclassified	GBIF: https://doi.org/10.15468/dl.zfaw79
Anopheles crucians	-81.1893	28.5722	Unclassified	GBIF: https://doi.org/10.15468/dl.zfaw79
Anopheles crucians	-81.1817	28.533	Unclassified	GBIF: https://doi.org/10.15468/dl.zfaw79
Anopheles crucians	-81.1807	28.5993	Unclassified	GBIF: https://doi.org/10.15468/dl.zfaw79

Anopheles crucians	-81.1806	28.5122	Unclassified	GBIF: https://doi.org/10.15468/dl.zfaw79
Anopheles crucians	-81.1769	28.5625	Unclassified	GBIF: https://doi.org/10.15468/dl.zfaw79
Anopheles crucians	-81.176	28.4041	Unclassified	GBIF: https://doi.org/10.15468/dl.zfaw79
Anopheles crucians	-81.1724	28.4177	Unclassified	GBIF: https://doi.org/10.15468/dl.zfaw79
Anopheles crucians	-81.1686	28.5477	Unclassified	GBIF: https://doi.org/10.15468/dl.zfaw79
Anopheles crucians	-81.1548	28.5386	Unclassified	GBIF: https://doi.org/10.15468/dl.zfaw79
Anopheles crucians	-81.1491	28.4611	Unclassified	GBIF: https://doi.org/10.15468/dl.zfaw79
Anopheles crucians	-81.1459	28.5208	Unclassified	GBIF: https://doi.org/10.15468/dl.zfaw79
Anopheles crucians	-81.14	32.78	Unclassified	GBIF: https://doi.org/10.15468/dl.zfaw79
Anopheles crucians	-81.1278	28.601	Unclassified	GBIF: https://doi.org/10.15468/dl.zfaw79
Anopheles crucians	-81.1141	29.1786	Unclassified	GBIF: https://doi.org/10.15468/dl.zfaw79
Anopheles crucians	-81.1061	29.0038	Unclassified	GBIF: https://doi.org/10.15468/dl.zfaw79
Anopheles crucians	-81.1044	28.5514	Unclassified	GBIF: https://doi.org/10.15468/dl.zfaw79
Anopheles crucians	-81.1018	29.3293	Unclassified	GBIF: https://doi.org/10.15468/dl.zfaw79
Anopheles crucians	-81.1015	28.5585	Unclassified	GBIF: https://doi.org/10.15468/dl.zfaw79
Anopheles crucians	-81.1006	28.9562	Unclassified	GBIF: https://doi.org/10.15468/dl.zfaw79
Anopheles crucians	-81.099834	32.083541	Unclassified	GBIF: https://doi.org/10.15468/dl.zfaw79
Anopheles crucians	-81.0974	28.5745	Unclassified	GBIF: https://doi.org/10.15468/dl.zfaw79
Anopheles crucians	-81.097	28.5748	Unclassified	GBIF: https://doi.org/10.15468/dl.zfaw79
Anopheles crucians	-81.0962	28.5339	Unclassified	GBIF: https://doi.org/10.15468/dl.zfaw79
Anopheles crucians	-81.0865	28.5076	Unclassified	GBIF: https://doi.org/10.15468/dl.zfaw79
Anopheles crucians	-81.0863	28.5367	Unclassified	GBIF: https://doi.org/10.15468/dl.zfaw79
Anopheles crucians	-81.0854	29.2168	Unclassified	GBIF: https://doi.org/10.15468/dl.zfaw79
Anopheles crucians	-81.0822	28.5021	Unclassified	GBIF: https://doi.org/10.15468/dl.zfaw79
Anopheles crucians	-81.0806	28.4837	Unclassified	GBIF: https://doi.org/10.15468/dl.zfaw79
Anopheles crucians	-81.08	33.99	Unclassified	GBIF: https://doi.org/10.15468/dl.zfaw79
Anopheles crucians	-81.076016	32.172653	Unclassified	GBIF: https://doi.org/10.15468/dl.zfaw79
Anopheles crucians	-81.0719	28.4896	Unclassified	GBIF: https://doi.org/10.15468/dl.zfaw79
Anopheles crucians	-81.0718	28.4898	Unclassified	GBIF: https://doi.org/10.15468/dl.zfaw79
Anopheles crucians	-81.0715	28.4648	Unclassified	GBIF: https://doi.org/10.15468/dl.zfaw79
Anopheles crucians	-81.0685	29.1109	Unclassified	GBIF: https://doi.org/10.15468/dl.zfaw79
Anopheles crucians	-81.0644	29.1183	Unclassified	GBIF: https://doi.org/10.15468/dl.zfaw79
Anopheles crucians	-81.0572	29.2339	Unclassified	GBIF: https://doi.org/10.15468/dl.zfaw79
Anopheles crucians	-81.0521	28.5876	Unclassified	GBIF: https://doi.org/10.15468/dl.zfaw79
Anopheles crucians	-81.04677	27.58201	Unclassified	GBIF: https://doi.org/10.15468/dl.zfaw79
Anopheles crucians	-81.044	29.2409	Unclassified	GBIF: https://doi.org/10.15468/dl.zfaw79
Anopheles crucians	-81.0399	29.1951	Unclassified	GBIF: https://doi.org/10.15468/dl.zfaw79
Anopheles crucians	-81.0393	29.1544	Unclassified	GBIF: https://doi.org/10.15468/dl.zfaw79
Anopheles crucians	-81.0378	29.1932	Unclassified	GBIF: https://doi.org/10.15468/dl.zfaw79
Anopheles crucians	-81.03	33.29	Unclassified	GBIF: https://doi.org/10.15468/dl.zfaw79
Anopheles crucians	-81.0281	29.0342	Unclassified	GBIF: https://doi.org/10.15468/dl.zfaw79
Anopheles crucians	-81.024	29.0505	Unclassified	GBIF: https://doi.org/10.15468/dl.zfaw79

Anopheles crucians	-81.0223	28.5647	Unclassified	GBIF: https://doi.org/10.15468/dl.zfaw79
Anopheles crucians	-81.0211	29.1709	Unclassified	GBIF: https://doi.org/10.15468/dl.zfaw79
Anopheles crucians	-81.0105	29.1544	Unclassified	GBIF: https://doi.org/10.15468/dl.zfaw79
Anopheles crucians	-81.0025	28.539	Unclassified	GBIF: https://doi.org/10.15468/dl.zfaw79
Anopheles crucians	-80.9963	28.4983	Unclassified	GBIF: https://doi.org/10.15468/dl.zfaw79
Anopheles crucians	-80.9962	28.5271	Unclassified	GBIF: https://doi.org/10.15468/dl.zfaw79
Anopheles crucians	-80.9902	29.0934	Unclassified	GBIF: https://doi.org/10.15468/dl.zfaw79
Anopheles crucians	-80.9875	29.124	Unclassified	GBIF: https://doi.org/10.15468/dl.zfaw79
Anopheles crucians	-80.978	29.1266	Unclassified	GBIF: https://doi.org/10.15468/dl.zfaw79
Anopheles crucians	-80.9697	29.049	Unclassified	GBIF: https://doi.org/10.15468/dl.zfaw79
Anopheles crucians	-80.96	24.76	Unclassified	GBIF: https://doi.org/10.15468/dl.zfaw79
Anopheles crucians	-80.949	28.9828	Unclassified	GBIF: https://doi.org/10.15468/dl.zfaw79
Anopheles crucians	-80.9463	28.9997	Unclassified	GBIF: https://doi.org/10.15468/dl.zfaw79
Anopheles crucians	-80.9194	29.0152	Unclassified	GBIF: https://doi.org/10.15468/dl.zfaw79
Anopheles crucians	-80.9176	28.9719	Unclassified	GBIF: https://doi.org/10.15468/dl.zfaw79
Anopheles crucians	-80.9114	28.9739	Unclassified	GBIF: https://doi.org/10.15468/dl.zfaw79
Anopheles crucians	-80.8924	28.9477	Unclassified	GBIF: https://doi.org/10.15468/dl.zfaw79
Anopheles crucians	-80.8742	28.8751	Unclassified	GBIF: https://doi.org/10.15468/dl.zfaw79
Anopheles crucians	-80.8471	28.8597	Unclassified	GBIF: https://doi.org/10.15468/dl.zfaw79
Anopheles crucians	-80.8	33.44	Unclassified	GBIF: https://doi.org/10.15468/dl.zfaw79
Anopheles crucians	-80.73	32.2	Unclassified	GBIF: https://doi.org/10.15468/dl.zfaw79
Anopheles crucians	-80.691	32.376	Unclassified	GBIF: https://doi.org/10.15468/dl.zfaw79
Anopheles crucians	-80.477778	25.468889	Unclassified	GBIF: https://doi.org/10.15468/dl.zfaw79
Anopheles crucians	-80.411242	27.434173	Unclassified	GBIF: https://doi.org/10.15468/dl.zfaw79
Anopheles crucians	-80.352	27.594	Unclassified	GBIF: https://doi.org/10.15468/dl.zfaw79
Anopheles crucians	-80.32	27.44	Unclassified	GBIF: https://doi.org/10.15468/dl.zfaw79
Anopheles crucians	-80	36	Unclassified	GBIF: https://doi.org/10.15468/dl.zfaw79
Anopheles crucians	-79.98	32.77	Unclassified	GBIF: https://doi.org/10.15468/dl.zfaw79
Anopheles crucians	-79.95	33.2	Unclassified	GBIF: https://doi.org/10.15468/dl.zfaw79
Anopheles crucians	-79.935304	32.807848	Unclassified	GBIF: https://doi.org/10.15468/dl.zfaw79
Anopheles crucians	-79.3	33.41	Unclassified	GBIF: https://doi.org/10.15468/dl.zfaw79
Anopheles crucians	-78.886694	33.68906	Unclassified	GBIF: https://doi.org/10.15468/dl.zfaw79
Anopheles crucians	-78.655437	35.86174	Unclassified	GBIF: https://doi.org/10.15468/dl.zfaw79
Anopheles crucians	-78.155294	38.900921	Unclassified	GBIF: https://doi.org/10.15468/dl.zfaw79
Anopheles crucians	-77.95938	39.081459	Unclassified	GBIF: https://doi.org/10.15468/dl.zfaw79
Anopheles crucians	-77.387712	34.650131	Unclassified	GBIF: https://doi.org/10.15468/dl.zfaw79
Anopheles crucians	-77.249705	38.658172	Unclassified	GBIF: https://doi.org/10.15468/dl.zfaw79
Anopheles crucians	-77.0414	38.6445	Unclassified	GBIF: https://doi.org/10.15468/dl.zfaw79
Anopheles crucians	-77.0376	38.633	Unclassified	GBIF: https://doi.org/10.15468/dl.zfaw79
Anopheles crucians	-77.0375	38.911111	Unclassified	GBIF: https://doi.org/10.15468/dl.zfaw79
Anopheles crucians	-77.0337	38.6362	Unclassified	GBIF: https://doi.org/10.15468/dl.zfaw79
Anopheles crucians	-77.0248	38.776	Unclassified	GBIF: https://doi.org/10.15468/dl.zfaw79

Anopheles crucians	-77.0227	38.7226	Unclassified	GBIF: https://doi.org/10.15468/dl.zfaw79
Anopheles crucians	-77.0185	38.7149	Unclassified	GBIF: https://doi.org/10.15468/dl.zfaw79
Anopheles crucians	-77.0175	38.775	Unclassified	GBIF: https://doi.org/10.15468/dl.zfaw79
Anopheles crucians	-77.0172	38.7755	Unclassified	GBIF: https://doi.org/10.15468/dl.zfaw79
Anopheles crucians	-77.0143	38.7133	Unclassified	GBIF: https://doi.org/10.15468/dl.zfaw79
Anopheles crucians	-77.0072	38.8105	Unclassified	GBIF: https://doi.org/10.15468/dl.zfaw79
Anopheles crucians	-77.007	38.8105	Unclassified	GBIF: https://doi.org/10.15468/dl.zfaw79
Anopheles crucians	-77.0002	38.8129	Unclassified	GBIF: https://doi.org/10.15468/dl.zfaw79
Anopheles crucians	-76.9845	38.7327	Unclassified	GBIF: https://doi.org/10.15468/dl.zfaw79
Anopheles crucians	-76.979	38.951	Unclassified	GBIF: https://doi.org/10.15468/dl.zfaw79
Anopheles crucians	-76.9743	38.9529	Unclassified	GBIF: https://doi.org/10.15468/dl.zfaw79
Anopheles crucians	-76.9738	38.9522	Unclassified	GBIF: https://doi.org/10.15468/dl.zfaw79
Anopheles crucians	-76.97363	17.99999	Unclassified	GBIF: https://doi.org/10.15468/dl.zfaw79
Anopheles crucians	-76.9736	38.9523	Unclassified	GBIF: https://doi.org/10.15468/dl.zfaw79
Anopheles crucians	-76.9736	38.9522	Unclassified	GBIF: https://doi.org/10.15468/dl.zfaw79
Anopheles crucians	-76.9734	38.9524	Unclassified	GBIF: https://doi.org/10.15468/dl.zfaw79
Anopheles crucians	-76.9633	38.797	Unclassified	GBIF: https://doi.org/10.15468/dl.zfaw79
Anopheles crucians	-76.9593	39.0175	Unclassified	GBIF: https://doi.org/10.15468/dl.zfaw79
Anopheles crucians	-76.959	38.9642	Unclassified	GBIF: https://doi.org/10.15468/dl.zfaw79
Anopheles crucians	-76.9561	38.7989	Unclassified	GBIF: https://doi.org/10.15468/dl.zfaw79
Anopheles crucians	-76.9556	38.7979	Unclassified	GBIF: https://doi.org/10.15468/dl.zfaw79
Anopheles crucians	-76.9551	39.0232	Unclassified	GBIF: https://doi.org/10.15468/dl.zfaw79
Anopheles crucians	-76.9539	38.9557	Unclassified	GBIF: https://doi.org/10.15468/dl.zfaw79
Anopheles crucians	-76.9537	38.0254	Unclassified	GBIF: https://doi.org/10.15468/dl.zfaw79
Anopheles crucians	-76.9535	39.0254	Unclassified	GBIF: https://doi.org/10.15468/dl.zfaw79
Anopheles crucians	-76.9534	39.0254	Unclassified	GBIF: https://doi.org/10.15468/dl.zfaw79
Anopheles crucians	-76.953	39.0066	Unclassified	GBIF: https://doi.org/10.15468/dl.zfaw79
Anopheles crucians	-76.9491	38.9497	Unclassified	GBIF: https://doi.org/10.15468/dl.zfaw79
Anopheles crucians	-76.9489	38.9496	Unclassified	GBIF: https://doi.org/10.15468/dl.zfaw79
Anopheles crucians	-76.9489	38.9454	Unclassified	GBIF: https://doi.org/10.15468/dl.zfaw79
Anopheles crucians	-76.9485	38.9422	Unclassified	GBIF: https://doi.org/10.15468/dl.zfaw79
Anopheles crucians	-76.9485	38.942	Unclassified	GBIF: https://doi.org/10.15468/dl.zfaw79
Anopheles crucians	-76.9454	38.932	Unclassified	GBIF: https://doi.org/10.15468/dl.zfaw79
Anopheles crucians	-76.9451	38.9332	Unclassified	GBIF: https://doi.org/10.15468/dl.zfaw79
Anopheles crucians	-76.9451	38.9387	Unclassified	GBIF: https://doi.org/10.15468/dl.zfaw79
Anopheles crucians	-76.9451	38.9386	Unclassified	GBIF: https://doi.org/10.15468/dl.zfaw79
Anopheles crucians	-76.9449	39.0062	Unclassified	GBIF: https://doi.org/10.15468/dl.zfaw79
Anopheles crucians	-76.9448	38.9336	Unclassified	GBIF: https://doi.org/10.15468/dl.zfaw79
Anopheles crucians	-76.9445	39.0064	Unclassified	GBIF: https://doi.org/10.15468/dl.zfaw79
Anopheles crucians	-76.9416	39.0057	Unclassified	GBIF: https://doi.org/10.15468/dl.zfaw79
Anopheles crucians	-76.9415	39.0058	Unclassified	GBIF: https://doi.org/10.15468/dl.zfaw79
Anopheles crucians	-76.9414	39.0068	Unclassified	GBIF: https://doi.org/10.15468/dl.zfaw79

Anopheles crucians	-76.9395	38.9385	Unclassified	GBIF: https://doi.org/10.15468/dl.zfaw79
Anopheles crucians	-76.9394	39.0033	Unclassified	GBIF: https://doi.org/10.15468/dl.zfaw79
Anopheles crucians	-76.9371	38.9463	Unclassified	GBIF: https://doi.org/10.15468/dl.zfaw79
Anopheles crucians	-76.937	38.9586	Unclassified	GBIF: https://doi.org/10.15468/dl.zfaw79
Anopheles crucians	-76.9369	38.9331	Unclassified	GBIF: https://doi.org/10.15468/dl.zfaw79
Anopheles crucians	-76.9367	38.9978	Unclassified	GBIF: https://doi.org/10.15468/dl.zfaw79
Anopheles crucians	-76.9362	39.0661	Unclassified	GBIF: https://doi.org/10.15468/dl.zfaw79
Anopheles crucians	-76.9352	39.0041	Unclassified	GBIF: https://doi.org/10.15468/dl.zfaw79
Anopheles crucians	-76.9344	39.0158	Unclassified	GBIF: https://doi.org/10.15468/dl.zfaw79
Anopheles crucians	-76.9343	39.0158	Unclassified	GBIF: https://doi.org/10.15468/dl.zfaw79
Anopheles crucians	-76.9341	38.761	Unclassified	GBIF: https://doi.org/10.15468/dl.zfaw79
Anopheles crucians	-76.9337	39.0151	Unclassified	GBIF: https://doi.org/10.15468/dl.zfaw79
Anopheles crucians	-76.9333	38.9549	Unclassified	GBIF: https://doi.org/10.15468/dl.zfaw79
Anopheles crucians	-76.9333	38.9668	Unclassified	GBIF: https://doi.org/10.15468/dl.zfaw79
Anopheles crucians	-76.933	39.0147	Unclassified	GBIF: https://doi.org/10.15468/dl.zfaw79
Anopheles crucians	-76.9329	39.0148	Unclassified	GBIF: https://doi.org/10.15468/dl.zfaw79
Anopheles crucians	-76.9318	38.9744	Unclassified	GBIF: https://doi.org/10.15468/dl.zfaw79
Anopheles crucians	-76.9311	38.9752	Unclassified	GBIF: https://doi.org/10.15468/dl.zfaw79
Anopheles crucians	-76.9308	38.9916	Unclassified	GBIF: https://doi.org/10.15468/dl.zfaw79
Anopheles crucians	-76.93	38.7612	Unclassified	GBIF: https://doi.org/10.15468/dl.zfaw79
Anopheles crucians	-76.9285	38.98	Unclassified	GBIF: https://doi.org/10.15468/dl.zfaw79
Anopheles crucians	-76.928	38.9554	Unclassified	GBIF: https://doi.org/10.15468/dl.zfaw79
Anopheles crucians	-76.9278	38.9553	Unclassified	GBIF: https://doi.org/10.15468/dl.zfaw79
Anopheles crucians	-76.9277	38.9555	Unclassified	GBIF: https://doi.org/10.15468/dl.zfaw79
Anopheles crucians	-76.9274	38.9594	Unclassified	GBIF: https://doi.org/10.15468/dl.zfaw79
Anopheles crucians	-76.9268	39.0747	Unclassified	GBIF: https://doi.org/10.15468/dl.zfaw79
Anopheles crucians	-76.9225	38.9053	Unclassified	GBIF: https://doi.org/10.15468/dl.zfaw79
Anopheles crucians	-76.9221	38.9943	Unclassified	GBIF: https://doi.org/10.15468/dl.zfaw79
Anopheles crucians	-76.9218	39.002	Unclassified	GBIF: https://doi.org/10.15468/dl.zfaw79
Anopheles crucians	-76.9205	39.0742	Unclassified	GBIF: https://doi.org/10.15468/dl.zfaw79
Anopheles crucians	-76.9189	38.9914	Unclassified	GBIF: https://doi.org/10.15468/dl.zfaw79
Anopheles crucians	-76.9174	39.0055	Unclassified	GBIF: https://doi.org/10.15468/dl.zfaw79
Anopheles crucians	-76.9171	38.9966	Unclassified	GBIF: https://doi.org/10.15468/dl.zfaw79
Anopheles crucians	-76.917	38.9765	Unclassified	GBIF: https://doi.org/10.15468/dl.zfaw79
Anopheles crucians	-76.9167	39.0054	Unclassified	GBIF: https://doi.org/10.15468/dl.zfaw79
Anopheles crucians	-76.9164	38.9869	Unclassified	GBIF: https://doi.org/10.15468/dl.zfaw79
Anopheles crucians	-76.9164	39.019	Unclassified	GBIF: https://doi.org/10.15468/dl.zfaw79
Anopheles crucians	-76.9154	38.8837	Unclassified	GBIF: https://doi.org/10.15468/dl.zfaw79
Anopheles crucians	-76.9141	39.0101	Unclassified	GBIF: https://doi.org/10.15468/dl.zfaw79
Anopheles crucians	-76.9141	39.0102	Unclassified	GBIF: https://doi.org/10.15468/dl.zfaw79
Anopheles crucians	-76.9135	38.9151	Unclassified	GBIF: https://doi.org/10.15468/dl.zfaw79
Anopheles crucians	-76.9121	38.9155	Unclassified	GBIF: https://doi.org/10.15468/dl.zfaw79

Anopheles crucians	-76.9116	38.9202	Unclassified	GBIF: https://doi.org/10.15468/dl.zfaw79
Anopheles crucians	-76.9112	38.8474	Unclassified	GBIF: https://doi.org/10.15468/dl.zfaw79
Anopheles crucians	-76.9106	38.9532	Unclassified	GBIF: https://doi.org/10.15468/dl.zfaw79
Anopheles crucians	-76.9084	38.9243	Unclassified	GBIF: https://doi.org/10.15468/dl.zfaw79
Anopheles crucians	-76.9083	38.7944	Unclassified	GBIF: https://doi.org/10.15468/dl.zfaw79
Anopheles crucians	-76.9062	38.8922	Unclassified	GBIF: https://doi.org/10.15468/dl.zfaw79
Anopheles crucians	-76.9047	38.9236	Unclassified	GBIF: https://doi.org/10.15468/dl.zfaw79
Anopheles crucians	-76.9025	38.9293	Unclassified	GBIF: https://doi.org/10.15468/dl.zfaw79
Anopheles crucians	-76.9004	38.8508	Unclassified	GBIF: https://doi.org/10.15468/dl.zfaw79
Anopheles crucians	-76.8999	38.896	Unclassified	GBIF: https://doi.org/10.15468/dl.zfaw79
Anopheles crucians	-76.8948	38.9417	Unclassified	GBIF: https://doi.org/10.15468/dl.zfaw79
Anopheles crucians	-76.8946	38.8491	Unclassified	GBIF: https://doi.org/10.15468/dl.zfaw79
Anopheles crucians	-76.8897	38.8493	Unclassified	GBIF: https://doi.org/10.15468/dl.zfaw79
Anopheles crucians	-76.88916	17.95928	Unclassified	GBIF: https://doi.org/10.15468/dl.zfaw79
Anopheles crucians	-76.8878	38.9009	Unclassified	GBIF: https://doi.org/10.15468/dl.zfaw79
Anopheles crucians	-76.8871	39.0005	Unclassified	GBIF: https://doi.org/10.15468/dl.zfaw79
Anopheles crucians	-76.8854	38.8979	Unclassified	GBIF: https://doi.org/10.15468/dl.zfaw79
Anopheles crucians	-76.8841	38.7569	Unclassified	GBIF: https://doi.org/10.15468/dl.zfaw79
Anopheles crucians	-76.8832	39.0461	Unclassified	GBIF: https://doi.org/10.15468/dl.zfaw79
Anopheles crucians	-76.8829	38.9843	Unclassified	GBIF: https://doi.org/10.15468/dl.zfaw79
Anopheles crucians	-76.8826	38.863	Unclassified	GBIF: https://doi.org/10.15468/dl.zfaw79
Anopheles crucians	-76.8816	39.0919	Unclassified	GBIF: https://doi.org/10.15468/dl.zfaw79
Anopheles crucians	-76.8808	38.9507	Unclassified	GBIF: https://doi.org/10.15468/dl.zfaw79
Anopheles crucians	-76.8801	39.0823	Unclassified	GBIF: https://doi.org/10.15468/dl.zfaw79
Anopheles crucians	-76.8801	39.0822	Unclassified	GBIF: https://doi.org/10.15468/dl.zfaw79
Anopheles crucians	-76.8799	38.9478	Unclassified	GBIF: https://doi.org/10.15468/dl.zfaw79
Anopheles crucians	-76.8792	39.0741	Unclassified	GBIF: https://doi.org/10.15468/dl.zfaw79
Anopheles crucians	-76.8783	38.8647	Unclassified	GBIF: https://doi.org/10.15468/dl.zfaw79
Anopheles crucians	-76.8782	38.8647	Unclassified	GBIF: https://doi.org/10.15468/dl.zfaw79
Anopheles crucians	-76.8769	38.9445	Unclassified	GBIF: https://doi.org/10.15468/dl.zfaw79
Anopheles crucians	-76.8769	38.9446	Unclassified	GBIF: https://doi.org/10.15468/dl.zfaw79
Anopheles crucians	-76.8742	39.0681	Unclassified	GBIF: https://doi.org/10.15468/dl.zfaw79
Anopheles crucians	-76.8738	39.0138	Unclassified	GBIF: https://doi.org/10.15468/dl.zfaw79
Anopheles crucians	-76.8733	38.976	Unclassified	GBIF: https://doi.org/10.15468/dl.zfaw79
Anopheles crucians	-76.87	36.596	Unclassified	GBIF: https://doi.org/10.15468/dl.zfaw79
Anopheles crucians	-76.8677	38.8874	Unclassified	GBIF: https://doi.org/10.15468/dl.zfaw79
Anopheles crucians	-76.8666	39.0064	Unclassified	GBIF: https://doi.org/10.15468/dl.zfaw79
Anopheles crucians	-76.8658	38.7137	Unclassified	GBIF: https://doi.org/10.15468/dl.zfaw79
Anopheles crucians	-76.8639	39.061	Unclassified	GBIF: https://doi.org/10.15468/dl.zfaw79
Anopheles crucians	-76.8637	38.8533	Unclassified	GBIF: https://doi.org/10.15468/dl.zfaw79
Anopheles crucians	-76.8619	38.9886	Unclassified	GBIF: https://doi.org/10.15468/dl.zfaw79
Anopheles crucians	-76.8599	38.984	Unclassified	GBIF: https://doi.org/10.15468/dl.zfaw79

Anopheles crucians	-76.8588	39.0726	Unclassified	GBIF: https://doi.org/10.15468/dl.zfaw79
Anopheles crucians	-76.8584	38.7572	Unclassified	GBIF: https://doi.org/10.15468/dl.zfaw79
Anopheles crucians	-76.8578	38.9532	Unclassified	GBIF: https://doi.org/10.15468/dl.zfaw79
Anopheles crucians	-76.8577	39.0975	Unclassified	GBIF: https://doi.org/10.15468/dl.zfaw79
Anopheles crucians	-76.8551	39.2298	Unclassified	GBIF: https://doi.org/10.15468/dl.zfaw79
Anopheles crucians	-76.8535	38.8944	Unclassified	GBIF: https://doi.org/10.15468/dl.zfaw79
Anopheles crucians	-76.8531	38.8943	Unclassified	GBIF: https://doi.org/10.15468/dl.zfaw79
Anopheles crucians	-76.8505	39.1035	Unclassified	GBIF: https://doi.org/10.15468/dl.zfaw79
Anopheles crucians	-76.8469	39.1062	Unclassified	GBIF: https://doi.org/10.15468/dl.zfaw79
Anopheles crucians	-76.8452	39.107	Unclassified	GBIF: https://doi.org/10.15468/dl.zfaw79
Anopheles crucians	-76.844	38.9262	Unclassified	GBIF: https://doi.org/10.15468/dl.zfaw79
Anopheles crucians	-76.8429	39.0835	Unclassified	GBIF: https://doi.org/10.15468/dl.zfaw79
Anopheles crucians	-76.8408	39.1009	Unclassified	GBIF: https://doi.org/10.15468/dl.zfaw79
Anopheles crucians	-76.8383	38.8124	Unclassified	GBIF: https://doi.org/10.15468/dl.zfaw79
Anopheles crucians	-76.8375	38.8103	Unclassified	GBIF: https://doi.org/10.15468/dl.zfaw79
Anopheles crucians	-76.8374	38.8104	Unclassified	GBIF: https://doi.org/10.15468/dl.zfaw79
Anopheles crucians	-76.8349	38.7881	Unclassified	GBIF: https://doi.org/10.15468/dl.zfaw79
Anopheles crucians	-76.8346	38.7881	Unclassified	GBIF: https://doi.org/10.15468/dl.zfaw79
Anopheles crucians	-76.8319	38.9311	Unclassified	GBIF: https://doi.org/10.15468/dl.zfaw79
Anopheles crucians	-76.8296	38.9677	Unclassified	GBIF: https://doi.org/10.15468/dl.zfaw79
Anopheles crucians	-76.8261	38.9504	Unclassified	GBIF: https://doi.org/10.15468/dl.zfaw79
Anopheles crucians	-76.8255	38.7842	Unclassified	GBIF: https://doi.org/10.15468/dl.zfaw79
Anopheles crucians	-76.8251	38.9525	Unclassified	GBIF: https://doi.org/10.15468/dl.zfaw79
Anopheles crucians	-76.8232	38.7502	Unclassified	GBIF: https://doi.org/10.15468/dl.zfaw79
Anopheles crucians	-76.8229	38.7501	Unclassified	GBIF: https://doi.org/10.15468/dl.zfaw79
Anopheles crucians	-76.8222	38.9724	Unclassified	GBIF: https://doi.org/10.15468/dl.zfaw79
Anopheles crucians	-76.822	38.9587	Unclassified	GBIF: https://doi.org/10.15468/dl.zfaw79
Anopheles crucians	-76.8208	38.8515	Unclassified	GBIF: https://doi.org/10.15468/dl.zfaw79
Anopheles crucians	-76.8205	39.0005	Unclassified	GBIF: https://doi.org/10.15468/dl.zfaw79
Anopheles crucians	-76.8202	38.9639	Unclassified	GBIF: https://doi.org/10.15468/dl.zfaw79
Anopheles crucians	-76.8198	39.0019	Unclassified	GBIF: https://doi.org/10.15468/dl.zfaw79
Anopheles crucians	-76.8196	38.9586	Unclassified	GBIF: https://doi.org/10.15468/dl.zfaw79
Anopheles crucians	-76.8193	38.96	Unclassified	GBIF: https://doi.org/10.15468/dl.zfaw79
Anopheles crucians	-76.8185	38.9697	Unclassified	GBIF: https://doi.org/10.15468/dl.zfaw79
Anopheles crucians	-76.8183	38.9698	Unclassified	GBIF: https://doi.org/10.15468/dl.zfaw79
Anopheles crucians	-76.8172	38.9666	Unclassified	GBIF: https://doi.org/10.15468/dl.zfaw79
Anopheles crucians	-76.8169	38.9668	Unclassified	GBIF: https://doi.org/10.15468/dl.zfaw79
Anopheles crucians	-76.8149	38.8144	Unclassified	GBIF: https://doi.org/10.15468/dl.zfaw79
Anopheles crucians	-76.8147	38.8198	Unclassified	GBIF: https://doi.org/10.15468/dl.zfaw79
Anopheles crucians	-76.8145	38.971	Unclassified	GBIF: https://doi.org/10.15468/dl.zfaw79
Anopheles crucians	-76.81	38.8243	Unclassified	GBIF: https://doi.org/10.15468/dl.zfaw79
Anopheles crucians	-76.8095	38.9107	Unclassified	GBIF: https://doi.org/10.15468/dl.zfaw79

Anopheles crucians	-76.8089	38.9104	Unclassified	GBIF: https://doi.org/10.15468/dl.zfaw79
Anopheles crucians	-76.8087	38.9096	Unclassified	GBIF: https://doi.org/10.15468/dl.zfaw79
Anopheles crucians	-76.8081	38.9042	Unclassified	GBIF: https://doi.org/10.15468/dl.zfaw79
Anopheles crucians	-76.8074	38.9076	Unclassified	GBIF: https://doi.org/10.15468/dl.zfaw79
Anopheles crucians	-76.8067	38.9018	Unclassified	GBIF: https://doi.org/10.15468/dl.zfaw79
Anopheles crucians	-76.806	38.9125	Unclassified	GBIF: https://doi.org/10.15468/dl.zfaw79
Anopheles crucians	-76.8059	38.8839	Unclassified	GBIF: https://doi.org/10.15468/dl.zfaw79
Anopheles crucians	-76.8059	38.8916	Unclassified	GBIF: https://doi.org/10.15468/dl.zfaw79
Anopheles crucians	-76.8054	39.0592	Unclassified	GBIF: https://doi.org/10.15468/dl.zfaw79
Anopheles crucians	-76.8028	39.0736	Unclassified	GBIF: https://doi.org/10.15468/dl.zfaw79
Anopheles crucians	-76.7994	39.0497	Unclassified	GBIF: https://doi.org/10.15468/dl.zfaw79
Anopheles crucians	-76.7981	38.9146	Unclassified	GBIF: https://doi.org/10.15468/dl.zfaw79
Anopheles crucians	-76.7959	39.054	Unclassified	GBIF: https://doi.org/10.15468/dl.zfaw79
Anopheles crucians	-76.7959	38.9706	Unclassified	GBIF: https://doi.org/10.15468/dl.zfaw79
Anopheles crucians	-76.7947	39.0469	Unclassified	GBIF: https://doi.org/10.15468/dl.zfaw79
Anopheles crucians	-76.7936	38.9198	Unclassified	GBIF: https://doi.org/10.15468/dl.zfaw79
Anopheles crucians	-76.7922	39.0526	Unclassified	GBIF: https://doi.org/10.15468/dl.zfaw79
Anopheles crucians	-76.7918	39.0452	Unclassified	GBIF: https://doi.org/10.15468/dl.zfaw79
Anopheles crucians	-76.7917	38.9324	Unclassified	GBIF: https://doi.org/10.15468/dl.zfaw79
Anopheles crucians	-76.7915	39.0453	Unclassified	GBIF: https://doi.org/10.15468/dl.zfaw79
Anopheles crucians	-76.7911	38.8493	Unclassified	GBIF: https://doi.org/10.15468/dl.zfaw79
Anopheles crucians	-76.7911	38.9685	Unclassified	GBIF: https://doi.org/10.15468/dl.zfaw79
Anopheles crucians	-76.7911	38.9686	Unclassified	GBIF: https://doi.org/10.15468/dl.zfaw79
Anopheles crucians	-76.791	39.0419	Unclassified	GBIF: https://doi.org/10.15468/dl.zfaw79
Anopheles crucians	-76.791	38.9187	Unclassified	GBIF: https://doi.org/10.15468/dl.zfaw79
Anopheles crucians	-76.7909	39.0422	Unclassified	GBIF: https://doi.org/10.15468/dl.zfaw79
Anopheles crucians	-76.7907	38.86	Unclassified	GBIF: https://doi.org/10.15468/dl.zfaw79
Anopheles crucians	-76.7903	38.8599	Unclassified	GBIF: https://doi.org/10.15468/dl.zfaw79
Anopheles crucians	-76.7901	39.0434	Unclassified	GBIF: https://doi.org/10.15468/dl.zfaw79
Anopheles crucians	-76.79	38.9523	Unclassified	GBIF: https://doi.org/10.15468/dl.zfaw79
Anopheles crucians	-76.7897	38.8594	Unclassified	GBIF: https://doi.org/10.15468/dl.zfaw79
Anopheles crucians	-76.7894	39.0446	Unclassified	GBIF: https://doi.org/10.15468/dl.zfaw79
Anopheles crucians	-76.789	39.0448	Unclassified	GBIF: https://doi.org/10.15468/dl.zfaw79
Anopheles crucians	-76.7874	38.9864	Unclassified	GBIF: https://doi.org/10.15468/dl.zfaw79
Anopheles crucians	-76.787	39.0484	Unclassified	GBIF: https://doi.org/10.15468/dl.zfaw79
Anopheles crucians	-76.787	36.682	Unclassified	GBIF: https://doi.org/10.15468/dl.zfaw79
Anopheles crucians	-76.7868	39.0426	Unclassified	GBIF: https://doi.org/10.15468/dl.zfaw79
Anopheles crucians	-76.7868	39.0485	Unclassified	GBIF: https://doi.org/10.15468/dl.zfaw79
Anopheles crucians	-76.7863	38.9393	Unclassified	GBIF: https://doi.org/10.15468/dl.zfaw79
Anopheles crucians	-76.786	38.9922	Unclassified	GBIF: https://doi.org/10.15468/dl.zfaw79
Anopheles crucians	-76.786	38.8288	Unclassified	GBIF: https://doi.org/10.15468/dl.zfaw79
Anopheles crucians	-76.7845	38.86	Unclassified	GBIF: https://doi.org/10.15468/dl.zfaw79

Anopheles crucians	-76.7842	38.9859	Unclassified	GBIF: https://doi.org/10.15468/dl.zfaw79
Anopheles crucians	-76.784	39.0394	Unclassified	GBIF: https://doi.org/10.15468/dl.zfaw79
Anopheles crucians	-76.7836	39.0423	Unclassified	GBIF: https://doi.org/10.15468/dl.zfaw79
Anopheles crucians	-76.7835	39.0477	Unclassified	GBIF: https://doi.org/10.15468/dl.zfaw79
Anopheles crucians	-76.7835	39.0422	Unclassified	GBIF: https://doi.org/10.15468/dl.zfaw79
Anopheles crucians	-76.7832	38.9872	Unclassified	GBIF: https://doi.org/10.15468/dl.zfaw79
Anopheles crucians	-76.7796	39.0404	Unclassified	GBIF: https://doi.org/10.15468/dl.zfaw79
Anopheles crucians	-76.7795	39.0407	Unclassified	GBIF: https://doi.org/10.15468/dl.zfaw79
Anopheles crucians	-76.779	39.0375	Unclassified	GBIF: https://doi.org/10.15468/dl.zfaw79
Anopheles crucians	-76.775	38.8919	Unclassified	GBIF: https://doi.org/10.15468/dl.zfaw79
Anopheles crucians	-76.7744	38.8769	Unclassified	GBIF: https://doi.org/10.15468/dl.zfaw79
Anopheles crucians	-76.7744	38.9772	Unclassified	GBIF: https://doi.org/10.15468/dl.zfaw79
Anopheles crucians	-76.7743	38.9663	Unclassified	GBIF: https://doi.org/10.15468/dl.zfaw79
Anopheles crucians	-76.7742	38.9662	Unclassified	GBIF: https://doi.org/10.15468/dl.zfaw79
Anopheles crucians	-76.7741	38.9635	Unclassified	GBIF: https://doi.org/10.15468/dl.zfaw79
Anopheles crucians	-76.7735	38.9758	Unclassified	GBIF: https://doi.org/10.15468/dl.zfaw79
Anopheles crucians	-76.7733	39.0095	Unclassified	GBIF: https://doi.org/10.15468/dl.zfaw79
Anopheles crucians	-76.7731	38.9688	Unclassified	GBIF: https://doi.org/10.15468/dl.zfaw79
Anopheles crucians	-76.7727	39.0702	Unclassified	GBIF: https://doi.org/10.15468/dl.zfaw79
Anopheles crucians	-76.7726	39.0702	Unclassified	GBIF: https://doi.org/10.15468/dl.zfaw79
Anopheles crucians	-76.7724	39.0063	Unclassified	GBIF: https://doi.org/10.15468/dl.zfaw79
Anopheles crucians	-76.7723	38.8995	Unclassified	GBIF: https://doi.org/10.15468/dl.zfaw79
Anopheles crucians	-76.7715	39.0451	Unclassified	GBIF: https://doi.org/10.15468/dl.zfaw79
Anopheles crucians	-76.7712	38.0786	Unclassified	GBIF: https://doi.org/10.15468/dl.zfaw79
Anopheles crucians	-76.7712	39.0786	Unclassified	GBIF: https://doi.org/10.15468/dl.zfaw79
Anopheles crucians	-76.7691	38.831	Unclassified	GBIF: https://doi.org/10.15468/dl.zfaw79
Anopheles crucians	-76.7689	39.0405	Unclassified	GBIF: https://doi.org/10.15468/dl.zfaw79
Anopheles crucians	-76.7688	39.0038	Unclassified	GBIF: https://doi.org/10.15468/dl.zfaw79
Anopheles crucians	-76.7687	39.0037	Unclassified	GBIF: https://doi.org/10.15468/dl.zfaw79
Anopheles crucians	-76.7659	38.8317	Unclassified	GBIF: https://doi.org/10.15468/dl.zfaw79
Anopheles crucians	-76.7653	38.832	Unclassified	GBIF: https://doi.org/10.15468/dl.zfaw79
Anopheles crucians	-76.7653	38.8319	Unclassified	GBIF: https://doi.org/10.15468/dl.zfaw79
Anopheles crucians	-76.7651	38.832	Unclassified	GBIF: https://doi.org/10.15468/dl.zfaw79
Anopheles crucians	-76.7646	38.9752	Unclassified	GBIF: https://doi.org/10.15468/dl.zfaw79
Anopheles crucians	-76.7638	38.8379	Unclassified	GBIF: https://doi.org/10.15468/dl.zfaw79
Anopheles crucians	-76.7638	38.9693	Unclassified	GBIF: https://doi.org/10.15468/dl.zfaw79
Anopheles crucians	-76.7636	38.99	Unclassified	GBIF: https://doi.org/10.15468/dl.zfaw79
Anopheles crucians	-76.7623	38.8166	Unclassified	GBIF: https://doi.org/10.15468/dl.zfaw79
Anopheles crucians	-76.7623	38.9697	Unclassified	GBIF: https://doi.org/10.15468/dl.zfaw79
Anopheles crucians	-76.7621	39.0423	Unclassified	GBIF: https://doi.org/10.15468/dl.zfaw79
Anopheles crucians	-76.7615	39.0432	Unclassified	GBIF: https://doi.org/10.15468/dl.zfaw79
Anopheles crucians	-76.7612	38.8938	Unclassified	GBIF: https://doi.org/10.15468/dl.zfaw79

Anopheles crucians	-76.7601	38.9628	Unclassified	GBIF: https://doi.org/10.15468/dl.zfaw79
Anopheles crucians	-76.7599	38.9629	Unclassified	GBIF: https://doi.org/10.15468/dl.zfaw79
Anopheles crucians	-76.7598	39.0392	Unclassified	GBIF: https://doi.org/10.15468/dl.zfaw79
Anopheles crucians	-76.7594	38.9732	Unclassified	GBIF: https://doi.org/10.15468/dl.zfaw79
Anopheles crucians	-76.7586	39.0427	Unclassified	GBIF: https://doi.org/10.15468/dl.zfaw79
Anopheles crucians	-76.7581	38.8948	Unclassified	GBIF: https://doi.org/10.15468/dl.zfaw79
Anopheles crucians	-76.758	39.0401	Unclassified	GBIF: https://doi.org/10.15468/dl.zfaw79
Anopheles crucians	-76.7563	38.9631	Unclassified	GBIF: https://doi.org/10.15468/dl.zfaw79
Anopheles crucians	-76.7562	38.966	Unclassified	GBIF: https://doi.org/10.15468/dl.zfaw79
Anopheles crucians	-76.7559	39.0394	Unclassified	GBIF: https://doi.org/10.15468/dl.zfaw79
Anopheles crucians	-76.7554	38.9642	Unclassified	GBIF: https://doi.org/10.15468/dl.zfaw79
Anopheles crucians	-76.7551	38.821	Unclassified	GBIF: https://doi.org/10.15468/dl.zfaw79
Anopheles crucians	-76.7551	38.8209	Unclassified	GBIF: https://doi.org/10.15468/dl.zfaw79
Anopheles crucians	-76.7544	38.8137	Unclassified	GBIF: https://doi.org/10.15468/dl.zfaw79
Anopheles crucians	-76.7541	38.9192	Unclassified	GBIF: https://doi.org/10.15468/dl.zfaw79
Anopheles crucians	-76.7539	38.814	Unclassified	GBIF: https://doi.org/10.15468/dl.zfaw79
Anopheles crucians	-76.753	38.9629	Unclassified	GBIF: https://doi.org/10.15468/dl.zfaw79
Anopheles crucians	-76.7524	38.8233	Unclassified	GBIF: https://doi.org/10.15468/dl.zfaw79
Anopheles crucians	-76.7521	39.0049	Unclassified	GBIF: https://doi.org/10.15468/dl.zfaw79
Anopheles crucians	-76.7514	38.9581	Unclassified	GBIF: https://doi.org/10.15468/dl.zfaw79
Anopheles crucians	-76.7513	39.0381	Unclassified	GBIF: https://doi.org/10.15468/dl.zfaw79
Anopheles crucians	-76.7513	38.9602	Unclassified	GBIF: https://doi.org/10.15468/dl.zfaw79
Anopheles crucians	-76.7512	38.9353	Unclassified	GBIF: https://doi.org/10.15468/dl.zfaw79
Anopheles crucians	-76.7511	38.9598	Unclassified	GBIF: https://doi.org/10.15468/dl.zfaw79
Anopheles crucians	-76.7511	38.9604	Unclassified	GBIF: https://doi.org/10.15468/dl.zfaw79
Anopheles crucians	-76.7506	38.9596	Unclassified	GBIF: https://doi.org/10.15468/dl.zfaw79
Anopheles crucians	-76.7505	38.9074	Unclassified	GBIF: https://doi.org/10.15468/dl.zfaw79
Anopheles crucians	-76.7502	38.9052	Unclassified	GBIF: https://doi.org/10.15468/dl.zfaw79
Anopheles crucians	-76.749817	39.082328	Unclassified	GBIF: https://doi.org/10.15468/dl.zfaw79
Anopheles crucians	-76.7497	38.9466	Unclassified	GBIF: https://doi.org/10.15468/dl.zfaw79
Anopheles crucians	-76.7494	38.9467	Unclassified	GBIF: https://doi.org/10.15468/dl.zfaw79
Anopheles crucians	-76.7481	39.0075	Unclassified	GBIF: https://doi.org/10.15468/dl.zfaw79
Anopheles crucians	-76.7478	38.9992	Unclassified	GBIF: https://doi.org/10.15468/dl.zfaw79
Anopheles crucians	-76.7478	38.8916	Unclassified	GBIF: https://doi.org/10.15468/dl.zfaw79
Anopheles crucians	-76.7475	39.0076	Unclassified	GBIF: https://doi.org/10.15468/dl.zfaw79
Anopheles crucians	-76.7474	38.9074	Unclassified	GBIF: https://doi.org/10.15468/dl.zfaw79
Anopheles crucians	-76.7454	39.0083	Unclassified	GBIF: https://doi.org/10.15468/dl.zfaw79
Anopheles crucians	-76.7451	38.9967	Unclassified	GBIF: https://doi.org/10.15468/dl.zfaw79
Anopheles crucians	-76.7444	38.9378	Unclassified	GBIF: https://doi.org/10.15468/dl.zfaw79
Anopheles crucians	-76.7444	38.9376	Unclassified	GBIF: https://doi.org/10.15468/dl.zfaw79
Anopheles crucians	-76.7437	38.8246	Unclassified	GBIF: https://doi.org/10.15468/dl.zfaw79
Anopheles crucians	-76.7422	39.0089	Unclassified	GBIF: https://doi.org/10.15468/dl.zfaw79

Anopheles crucians	-76.7411	39.0096	Unclassified	GBIF: https://doi.org/10.15468/dl.zfaw79
Anopheles crucians	-76.7409	38.8262	Unclassified	GBIF: https://doi.org/10.15468/dl.zfaw79
Anopheles crucians	-76.7389	39.0057	Unclassified	GBIF: https://doi.org/10.15468/dl.zfaw79
Anopheles crucians	-76.7387	38.8934	Unclassified	GBIF: https://doi.org/10.15468/dl.zfaw79
Anopheles crucians	-76.7385	38.9083	Unclassified	GBIF: https://doi.org/10.15468/dl.zfaw79
Anopheles crucians	-76.7383	38.9243	Unclassified	GBIF: https://doi.org/10.15468/dl.zfaw79
Anopheles crucians	-76.738	38.9085	Unclassified	GBIF: https://doi.org/10.15468/dl.zfaw79
Anopheles crucians	-76.738	38.9559	Unclassified	GBIF: https://doi.org/10.15468/dl.zfaw79
Anopheles crucians	-76.7375	38.9562	Unclassified	GBIF: https://doi.org/10.15468/dl.zfaw79
Anopheles crucians	-76.7368	38.9151	Unclassified	GBIF: https://doi.org/10.15468/dl.zfaw79
Anopheles crucians	-76.7367	39.0618	Unclassified	GBIF: https://doi.org/10.15468/dl.zfaw79
Anopheles crucians	-76.7362	38.9558	Unclassified	GBIF: https://doi.org/10.15468/dl.zfaw79
Anopheles crucians	-76.7361	39.0021	Unclassified	GBIF: https://doi.org/10.15468/dl.zfaw79
Anopheles crucians	-76.7357	39.0021	Unclassified	GBIF: https://doi.org/10.15468/dl.zfaw79
Anopheles crucians	-76.7353	38.9554	Unclassified	GBIF: https://doi.org/10.15468/dl.zfaw79
Anopheles crucians	-76.7352	39.0083	Unclassified	GBIF: https://doi.org/10.15468/dl.zfaw79
Anopheles crucians	-76.7347	39.0865	Unclassified	GBIF: https://doi.org/10.15468/dl.zfaw79
Anopheles crucians	-76.7346	38.9126	Unclassified	GBIF: https://doi.org/10.15468/dl.zfaw79
Anopheles crucians	-76.7318	38.9986	Unclassified	GBIF: https://doi.org/10.15468/dl.zfaw79
Anopheles crucians	-76.7307	38.9973	Unclassified	GBIF: https://doi.org/10.15468/dl.zfaw79
Anopheles crucians	-76.7303	38.9921	Unclassified	GBIF: https://doi.org/10.15468/dl.zfaw79
Anopheles crucians	-76.7296	38.9719	Unclassified	GBIF: https://doi.org/10.15468/dl.zfaw79
Anopheles crucians	-76.7282	38.9222	Unclassified	GBIF: https://doi.org/10.15468/dl.zfaw79
Anopheles crucians	-76.7271	38.9347	Unclassified	GBIF: https://doi.org/10.15468/dl.zfaw79
Anopheles crucians	-76.727	36.726	Unclassified	GBIF: https://doi.org/10.15468/dl.zfaw79
Anopheles crucians	-76.7254	38.9939	Unclassified	GBIF: https://doi.org/10.15468/dl.zfaw79
Anopheles crucians	-76.7254	38.9938	Unclassified	GBIF: https://doi.org/10.15468/dl.zfaw79
Anopheles crucians	-76.7243	38.9464	Unclassified	GBIF: https://doi.org/10.15468/dl.zfaw79
Anopheles crucians	-76.7227	38.9367	Unclassified	GBIF: https://doi.org/10.15468/dl.zfaw79
Anopheles crucians	-76.7217	38.9662	Unclassified	GBIF: https://doi.org/10.15468/dl.zfaw79
Anopheles crucians	-76.7211	38.99	Unclassified	GBIF: https://doi.org/10.15468/dl.zfaw79
Anopheles crucians	-76.7192	38.933	Unclassified	GBIF: https://doi.org/10.15468/dl.zfaw79
Anopheles crucians	-76.7188	38.969	Unclassified	GBIF: https://doi.org/10.15468/dl.zfaw79
Anopheles crucians	-76.713	36.645	Unclassified	GBIF: https://doi.org/10.15468/dl.zfaw79
Anopheles crucians	-76.71	38.9524	Unclassified	GBIF: https://doi.org/10.15468/dl.zfaw79
Anopheles crucians	-76.7067	38.9527	Unclassified	GBIF: https://doi.org/10.15468/dl.zfaw79
Anopheles crucians	-76.7056	38.952	Unclassified	GBIF: https://doi.org/10.15468/dl.zfaw79
Anopheles crucians	-76.705	38.9523	Unclassified	GBIF: https://doi.org/10.15468/dl.zfaw79
Anopheles crucians	-76.7004	38.9524	Unclassified	GBIF: https://doi.org/10.15468/dl.zfaw79
Anopheles crucians	-76.689	38.574	Unclassified	GBIF: https://doi.org/10.15468/dl.zfaw79
Anopheles crucians	-76.6878	38.5764	Unclassified	GBIF: https://doi.org/10.15468/dl.zfaw79
Anopheles crucians	-76.6877	38.5676	Unclassified	GBIF: https://doi.org/10.15468/dl.zfaw79

Anopheles crucians	-76.6865	38.5673	Unclassified	GBIF: https://doi.org/10.15468/dl.zfaw79
Anopheles crucians	-76.6862	38.5673	Unclassified	GBIF: https://doi.org/10.15468/dl.zfaw79
Anopheles crucians	-76.6852	38.5695	Unclassified	GBIF: https://doi.org/10.15468/dl.zfaw79
Anopheles crucians	-76.685	38.8862	Unclassified	GBIF: https://doi.org/10.15468/dl.zfaw79
Anopheles crucians	-76.6849	38.5648	Unclassified	GBIF: https://doi.org/10.15468/dl.zfaw79
Anopheles crucians	-76.6848	38.5649	Unclassified	GBIF: https://doi.org/10.15468/dl.zfaw79
Anopheles crucians	-76.6848	38.5648	Unclassified	GBIF: https://doi.org/10.15468/dl.zfaw79
Anopheles crucians	-76.684	38.5703	Unclassified	GBIF: https://doi.org/10.15468/dl.zfaw79
Anopheles crucians	-76.6838	38.5703	Unclassified	GBIF: https://doi.org/10.15468/dl.zfaw79
Anopheles crucians	-76.662	36.61	Unclassified	GBIF: https://doi.org/10.15468/dl.zfaw79
Anopheles crucians	-76.655	36.716	Unclassified	GBIF: https://doi.org/10.15468/dl.zfaw79
Anopheles crucians	-76.647	36.717	Unclassified	GBIF: https://doi.org/10.15468/dl.zfaw79
Anopheles crucians	-76.642	36.825	Unclassified	GBIF: https://doi.org/10.15468/dl.zfaw79
Anopheles crucians	-76.622	36.718	Unclassified	GBIF: https://doi.org/10.15468/dl.zfaw79
Anopheles crucians	-76.622	36.795	Unclassified	GBIF: https://doi.org/10.15468/dl.zfaw79
Anopheles crucians	-76.618	36.751	Unclassified	GBIF: https://doi.org/10.15468/dl.zfaw79
Anopheles crucians	-76.616	36.813	Unclassified	GBIF: https://doi.org/10.15468/dl.zfaw79
Anopheles crucians	-76.606	36.776	Unclassified	GBIF: https://doi.org/10.15468/dl.zfaw79
Anopheles crucians	-76.606	36.687	Unclassified	GBIF: https://doi.org/10.15468/dl.zfaw79
Anopheles crucians	-76.602	36.733	Unclassified	GBIF: https://doi.org/10.15468/dl.zfaw79
Anopheles crucians	-76.595	36.723	Unclassified	GBIF: https://doi.org/10.15468/dl.zfaw79
Anopheles crucians	-76.59	36.738	Unclassified	GBIF: https://doi.org/10.15468/dl.zfaw79
Anopheles crucians	-76.588	36.732	Unclassified	GBIF: https://doi.org/10.15468/dl.zfaw79
Anopheles crucians	-76.587	36.73	Unclassified	GBIF: https://doi.org/10.15468/dl.zfaw79
Anopheles crucians	-76.586	36.845	Unclassified	GBIF: https://doi.org/10.15468/dl.zfaw79
Anopheles crucians	-76.585	36.694	Unclassified	GBIF: https://doi.org/10.15468/dl.zfaw79
Anopheles crucians	-76.585	36.73	Unclassified	GBIF: https://doi.org/10.15468/dl.zfaw79
Anopheles crucians	-76.584	36.715	Unclassified	GBIF: https://doi.org/10.15468/dl.zfaw79
Anopheles crucians	-76.584	36.746	Unclassified	GBIF: https://doi.org/10.15468/dl.zfaw79
Anopheles crucians	-76.584	36.852	Unclassified	GBIF: https://doi.org/10.15468/dl.zfaw79
Anopheles crucians	-76.583	36.732	Unclassified	GBIF: https://doi.org/10.15468/dl.zfaw79
Anopheles crucians	-76.581	36.679	Unclassified	GBIF: https://doi.org/10.15468/dl.zfaw79
Anopheles crucians	-76.58	36.766	Unclassified	GBIF: https://doi.org/10.15468/dl.zfaw79
Anopheles crucians	-76.579	36.617	Unclassified	GBIF: https://doi.org/10.15468/dl.zfaw79
Anopheles crucians	-76.579	36.735	Unclassified	GBIF: https://doi.org/10.15468/dl.zfaw79
Anopheles crucians	-76.577	36.716	Unclassified	GBIF: https://doi.org/10.15468/dl.zfaw79
Anopheles crucians	-76.574	36.758	Unclassified	GBIF: https://doi.org/10.15468/dl.zfaw79
Anopheles crucians	-76.571	36.799	Unclassified	GBIF: https://doi.org/10.15468/dl.zfaw79
Anopheles crucians	-76.571	36.769	Unclassified	GBIF: https://doi.org/10.15468/dl.zfaw79
Anopheles crucians	-76.57	36.73	Unclassified	GBIF: https://doi.org/10.15468/dl.zfaw79
Anopheles crucians	-76.57	36.86	Unclassified	GBIF: https://doi.org/10.15468/dl.zfaw79
Anopheles crucians	-76.564206	38.88788	Unclassified	GBIF: https://doi.org/10.15468/dl.zfaw79

Anopheles crucians	-76.564	38.888	Unclassified	GBIF: https://doi.org/10.15468/dl.zfaw79
Anopheles crucians	-76.563	36.726	Unclassified	GBIF: https://doi.org/10.15468/dl.zfaw79
Anopheles crucians	-76.562	36.734	Unclassified	GBIF: https://doi.org/10.15468/dl.zfaw79
Anopheles crucians	-76.560014	38.890131	Unclassified	GBIF: https://doi.org/10.15468/dl.zfaw79
Anopheles crucians	-76.56	36.618	Unclassified	GBIF: https://doi.org/10.15468/dl.zfaw79
Anopheles crucians	-76.555264	38.886782	Unclassified	GBIF: https://doi.org/10.15468/dl.zfaw79
Anopheles crucians	-76.555	38.887	Unclassified	GBIF: https://doi.org/10.15468/dl.zfaw79
Anopheles crucians	-76.554	36.75	Unclassified	GBIF: https://doi.org/10.15468/dl.zfaw79
Anopheles crucians	-76.55	36.872	Unclassified	GBIF: https://doi.org/10.15468/dl.zfaw79
Anopheles crucians	-76.55	36.7343	Unclassified	GBIF: https://doi.org/10.15468/dl.zfaw79
Anopheles crucians	-76.549	36.715	Unclassified	GBIF: https://doi.org/10.15468/dl.zfaw79
Anopheles crucians	-76.548	38.898	Unclassified	GBIF: https://doi.org/10.15468/dl.zfaw79
Anopheles crucians	-76.547916	38.897843	Unclassified	GBIF: https://doi.org/10.15468/dl.zfaw79
Anopheles crucians	-76.547	36.644	Unclassified	GBIF: https://doi.org/10.15468/dl.zfaw79
Anopheles crucians	-76.546054	38.868356	Unclassified	GBIF: https://doi.org/10.15468/dl.zfaw79
Anopheles crucians	-76.546	38.868	Unclassified	GBIF: https://doi.org/10.15468/dl.zfaw79
Anopheles crucians	-76.546	36.728	Unclassified	GBIF: https://doi.org/10.15468/dl.zfaw79
Anopheles crucians	-76.545	38.888	Unclassified	GBIF: https://doi.org/10.15468/dl.zfaw79
Anopheles crucians	-76.544644	38.887714	Unclassified	GBIF: https://doi.org/10.15468/dl.zfaw79
Anopheles crucians	-76.542491	38.875234	Unclassified	GBIF: https://doi.org/10.15468/dl.zfaw79
Anopheles crucians	-76.542	38.875	Unclassified	GBIF: https://doi.org/10.15468/dl.zfaw79
Anopheles crucians	-76.542	36.764	Unclassified	GBIF: https://doi.org/10.15468/dl.zfaw79
Anopheles crucians	-76.541404	38.87085	Unclassified	GBIF: https://doi.org/10.15468/dl.zfaw79
Anopheles crucians	-76.541	38.871	Unclassified	GBIF: https://doi.org/10.15468/dl.zfaw79
Anopheles crucians	-76.539	36.83	Unclassified	GBIF: https://doi.org/10.15468/dl.zfaw79
Anopheles crucians	-76.538	36.747	Unclassified	GBIF: https://doi.org/10.15468/dl.zfaw79
Anopheles crucians	-76.537124	38.872876	Unclassified	GBIF: https://doi.org/10.15468/dl.zfaw79
Anopheles crucians	-76.537	38.903	Unclassified	GBIF: https://doi.org/10.15468/dl.zfaw79
Anopheles crucians	-76.537	38.873	Unclassified	GBIF: https://doi.org/10.15468/dl.zfaw79
Anopheles crucians	-76.537	36.725	Unclassified	GBIF: https://doi.org/10.15468/dl.zfaw79
Anopheles crucians	-76.536653	38.902578	Unclassified	GBIF: https://doi.org/10.15468/dl.zfaw79
Anopheles crucians	-76.536	36.901	Unclassified	GBIF: https://doi.org/10.15468/dl.zfaw79
Anopheles crucians	-76.534678	38.68623	Unclassified	GBIF: https://doi.org/10.15468/dl.zfaw79
Anopheles crucians	-76.534	36.747	Unclassified	GBIF: https://doi.org/10.15468/dl.zfaw79
Anopheles crucians	-76.533	38.874	Unclassified	GBIF: https://doi.org/10.15468/dl.zfaw79
Anopheles crucians	-76.532584	38.873772	Unclassified	GBIF: https://doi.org/10.15468/dl.zfaw79
Anopheles crucians	-76.523	36.779	Unclassified	GBIF: https://doi.org/10.15468/dl.zfaw79
Anopheles crucians	-76.523	36.838	Unclassified	GBIF: https://doi.org/10.15468/dl.zfaw79
Anopheles crucians	-76.519	36.807	Unclassified	GBIF: https://doi.org/10.15468/dl.zfaw79
Anopheles crucians	-76.518	36.732	Unclassified	GBIF: https://doi.org/10.15468/dl.zfaw79
Anopheles crucians	-76.51	36.899	Unclassified	GBIF: https://doi.org/10.15468/dl.zfaw79
Anopheles crucians	-76.509	36.825	Unclassified	GBIF: https://doi.org/10.15468/dl.zfaw79

Anopheles crucians	-76.503	36.706	Unclassified	GBIF: https://doi.org/10.15468/dl.zfaw79
Anopheles crucians	-76.502	36.862	Unclassified	GBIF: https://doi.org/10.15468/dl.zfaw79
Anopheles crucians	-76.495	36.845	Unclassified	GBIF: https://doi.org/10.15468/dl.zfaw79
Anopheles crucians	-76.49	36.795	Unclassified	GBIF: https://doi.org/10.15468/dl.zfaw79
Anopheles crucians	-76.488	36.91	Unclassified	GBIF: https://doi.org/10.15468/dl.zfaw79
Anopheles crucians	-76.483	36.915	Unclassified	GBIF: https://doi.org/10.15468/dl.zfaw79
Anopheles crucians	-76.477	36.865	Unclassified	GBIF: https://doi.org/10.15468/dl.zfaw79
Anopheles crucians	-76.475	36.847	Unclassified	GBIF: https://doi.org/10.15468/dl.zfaw79
Anopheles crucians	-76.475	36.824	Unclassified	GBIF: https://doi.org/10.15468/dl.zfaw79
Anopheles crucians	-76.462	36.82	Unclassified	GBIF: https://doi.org/10.15468/dl.zfaw79
Anopheles crucians	-76.459	36.875	Unclassified	GBIF: https://doi.org/10.15468/dl.zfaw79
Anopheles crucians	-76.4483	39.0484	Unclassified	GBIF: https://doi.org/10.15468/dl.zfaw79
Anopheles crucians	-76.445	36.853	Unclassified	GBIF: https://doi.org/10.15468/dl.zfaw79
Anopheles crucians	-76.445	36.888	Unclassified	GBIF: https://doi.org/10.15468/dl.zfaw79
Anopheles crucians	-76.434	36.861	Unclassified	GBIF: https://doi.org/10.15468/dl.zfaw79
Anopheles crucians	-76.433	36.873	Unclassified	GBIF: https://doi.org/10.15468/dl.zfaw79
Anopheles crucians	-76.419	36.885	Unclassified	GBIF: https://doi.org/10.15468/dl.zfaw79
Anopheles crucians	-76.414	36.883	Unclassified	GBIF: https://doi.org/10.15468/dl.zfaw79
Anopheles crucians	-75.491692	38.125516	Unclassified	GBIF: https://doi.org/10.15468/dl.zfaw79
Anopheles crucians	-75.453628	38.276399	Unclassified	GBIF: https://doi.org/10.15468/dl.zfaw79
Anopheles crucians	-74.962321	38.940353	Unclassified	GBIF: https://doi.org/10.15468/dl.zfaw79
Anopheles crucians	-74.924281	39.21718	Unclassified	GBIF: https://doi.org/10.15468/dl.zfaw79
Anopheles crucians	-74.912252	39.178473	Unclassified	GBIF: https://doi.org/10.15468/dl.zfaw79
Anopheles crucians	-73.093164	41.316486	Unclassified	GBIF: https://doi.org/10.15468/dl.zfaw79
Anopheles crucians	-72.894424	40.803696	Unclassified	GBIF: https://doi.org/10.15468/dl.zfaw79
Anopheles crucians	-72.847132	41.297218	Unclassified	GBIF: https://doi.org/10.15468/dl.zfaw79
Anopheles crucians	-72.272148	42.482279	Unclassified	GBIF: https://doi.org/10.15468/dl.zfaw79
Anopheles crucians	-72.236178	42.417662	Unclassified	GBIF: https://doi.org/10.15468/dl.zfaw79
Anopheles crucians	-72.236	42.418	Unclassified	GBIF: https://doi.org/10.15468/dl.zfaw79
Anopheles crucians	-72.22545	42.456233	Unclassified	GBIF: https://doi.org/10.15468/dl.zfaw79
Anopheles crucians	-72.173	42.537	Unclassified	GBIF: https://doi.org/10.15468/dl.zfaw79
Anopheles crucians	-72.17266	42.5369	Unclassified	GBIF: https://doi.org/10.15468/dl.zfaw79
Anopheles crucians	-71.9	41.05	Unclassified	GBIF: https://doi.org/10.15468/dl.zfaw79
Anopheles crucians	-71.896576	41.051346	Unclassified	GBIF: https://doi.org/10.15468/dl.zfaw79
Anopheles crucians	-71.7956	41.3546	Unclassified	GBIF: https://doi.org/10.15468/dl.zfaw79
Anopheles crucians	-71.7853	41.367	Unclassified	GBIF: https://doi.org/10.15468/dl.zfaw79
Anopheles crucians	-71.6998	41.362	Unclassified	GBIF: https://doi.org/10.15468/dl.zfaw79
Anopheles crucians	-71.5745	42.0095	Unclassified	GBIF: https://doi.org/10.15468/dl.zfaw79
Anopheles crucians	-71.4665	41.547	Unclassified	GBIF: https://doi.org/10.15468/dl.zfaw79
Anopheles crucians	-71.466	41.7933	Unclassified	GBIF: https://doi.org/10.15468/dl.zfaw79
Anopheles crucians	-71.3228	41.7426	Unclassified	GBIF: https://doi.org/10.15468/dl.zfaw79
Anopheles crucians	-70.40278	19.11548	Unclassified	GBIF: https://doi.org/10.15468/dl.zfaw79

Anopheles crucians	-70.37619	19.11748	Unclassified	GBIF: https://doi.org/10.15468/dl.zfaw79
Anopheles crucians	-70.16024	19.09458	Unclassified	GBIF: https://doi.org/10.15468/dl.zfaw79
Anopheles crucians	-69.95223	18.61862	Unclassified	GBIF: https://doi.org/10.15468/dl.zfaw79
Anopheles crucians	76.9736	38.9522	Unclassified	GBIF: https://doi.org/10.15468/dl.zfaw79
Anopheles crucians	81.3873	24.7172	Unclassified	GBIF: https://doi.org/10.15468/dl.zfaw79
Anopheles cruzii	-51.020278	-30.071389	Primary	GBIF: https://doi.org/10.15468/dl.zfaw79
Anopheles cruzii	-50.583333	-29.448056	Primary	GBIF: https://doi.org/10.15468/dl.zfaw79
Anopheles cruzii	-48.8293	-25.4754	Primary	GBIF: https://doi.org/10.15468/dl.zfaw79
Anopheles cruzii	-48.5427	-25.5128	Primary	GBIF: https://doi.org/10.15468/dl.zfaw79
Anopheles cruzii	-47.95	-25.01667	Primary	GBIF: https://doi.org/10.15468/dl.zfaw79
Anopheles cruzii	-47.95	-25.02	Primary	GBIF: https://doi.org/10.15468/dl.zfaw79
Anopheles cruzii	-47.851	-24.888	Primary	GBIF: https://doi.org/10.15468/dl.zfaw79
Anopheles cruzii	-47.85	-24.89	Primary	GBIF: https://doi.org/10.15468/dl.zfaw79
Anopheles cruzii	-47.802	-24.637	Primary	GBIF: https://doi.org/10.15468/dl.zfaw79
Anopheles cruzii	-47.8	-24.64	Primary	GBIF: https://doi.org/10.15468/dl.zfaw79
Anopheles cruzii	-47.50654	-23.97192	Primary	GBIF: https://doi.org/10.15468/dl.zfaw79
Anopheles cruzii	-47.5065	-23.9719	Primary	GBIF: https://doi.org/10.15468/dl.zfaw79
Anopheles cruzii	-45.90056	-23.65157	Primary	GBIF: https://doi.org/10.15468/dl.zfaw79
Anopheles cruzii	-45.89186	-23.65587	Primary	GBIF: https://doi.org/10.15468/dl.zfaw79
Anopheles cruzii	-45.8896	-23.65579	Primary	GBIF: https://doi.org/10.15468/dl.zfaw79
Anopheles cruzii	-42.981899	-22.537201	Primary	GBIF: https://doi.org/10.15468/dl.zfaw79
Anopheles cruzii	-42.965599	-22.412201	Primary	GBIF: https://doi.org/10.15468/dl.zfaw79
Anopheles cruzii	-41.7869	-22.3708	Primary	GBIF: https://doi.org/10.15468/dl.zfaw79
Anopheles culicifacies	54.237076	32.906025	Unclassified	GBIF: https://doi.org/10.15468/dl.zfaw79
Anopheles culicifacies	55.74	24.175	Unclassified	GBIF: https://doi.org/10.15468/dl.zfaw79
Anopheles culicifacies	55.74	24.18	Unclassified	GBIF: https://doi.org/10.15468/dl.zfaw79
Anopheles culicifacies	71.48	34.01	Unclassified	GBIF: https://doi.org/10.15468/dl.zfaw79
Anopheles culicifacies	71.482	34.006	Unclassified	GBIF: https://doi.org/10.15468/dl.zfaw79
Anopheles culicifacies	71.486	33.999	Unclassified	GBIF: https://doi.org/10.15468/dl.zfaw79
Anopheles culicifacies	71.488	33.998	Unclassified	GBIF: https://doi.org/10.15468/dl.zfaw79
Anopheles culicifacies	71.49	34	Unclassified	GBIF: https://doi.org/10.15468/dl.zfaw79
Anopheles culicifacies	72.873	22.678	Unclassified	GBIF: https://doi.org/10.15468/dl.zfaw79
Anopheles culicifacies	73.41	33.13	Unclassified	GBIF: https://doi.org/10.15468/dl.zfaw79
Anopheles culicifacies	73.414	33.129	Unclassified	GBIF: https://doi.org/10.15468/dl.zfaw79
Anopheles culicifacies	73.578	31.729	Unclassified	GBIF: https://doi.org/10.15468/dl.zfaw79
Anopheles culicifacies	73.58	31.73	Unclassified	GBIF: https://doi.org/10.15468/dl.zfaw79
Anopheles culicifacies	74.329	31.582	Unclassified	GBIF: https://doi.org/10.15468/dl.zfaw79
Anopheles culicifacies	74.33	31.58	Unclassified	GBIF: https://doi.org/10.15468/dl.zfaw79
Anopheles culicifacies	74.6	30.9167	Unclassified	GBIF: https://doi.org/10.15468/dl.zfaw79
Anopheles culicifacies	75.060505	18.903092	Unclassified	GBIF: https://doi.org/10.15468/dl.zfaw79
Anopheles culicifacies	75.9167	17.6833	Unclassified	GBIF: https://doi.org/10.15468/dl.zfaw79
Anopheles culicifacies	76.69	11.4	Unclassified	GBIF: https://doi.org/10.15468/dl.zfaw79

Anopheles culicifacies	80.66931	7.696631	Unclassified	GBIF: https://doi.org/10.15468/dl.zfaw79
Anopheles culicifacies	81.173004	23.5863	Unclassified	GBIF: https://doi.org/10.15468/dl.zfaw79
Anopheles culicifacies	84.058697	28.066881	Unclassified	GBIF: https://doi.org/10.15468/dl.zfaw79
Anopheles culicifacies	84.123711	28.101191	Unclassified	GBIF: https://doi.org/10.15468/dl.zfaw79
Anopheles culicifacies	84.141344	28.045586	Unclassified	GBIF: https://doi.org/10.15468/dl.zfaw79
Anopheles culicifacies	84.15934	27.940147	Unclassified	GBIF: https://doi.org/10.15468/dl.zfaw79
Anopheles culicifacies	84.212562	28.025753	Unclassified	GBIF: https://doi.org/10.15468/dl.zfaw79
Anopheles culicifacies	84.525259	27.985058	Unclassified	GBIF: https://doi.org/10.15468/dl.zfaw79
Anopheles culicifacies	84.7381	22.2399	Unclassified	GBIF: https://doi.org/10.15468/dl.zfaw79
Anopheles culicifacies	84.7403	22.2411	Unclassified	GBIF: https://doi.org/10.15468/dl.zfaw79
Anopheles culicifacies	84.7404	22.2411	Unclassified	GBIF: https://doi.org/10.15468/dl.zfaw79
Anopheles culicifacies	84.7431	22.2482	Unclassified	GBIF: https://doi.org/10.15468/dl.zfaw79
Anopheles culicifacies	84.7432	22.2482	Unclassified	GBIF: https://doi.org/10.15468/dl.zfaw79
Anopheles culicifacies	84.7432	22.2481	Unclassified	GBIF: https://doi.org/10.15468/dl.zfaw79
Anopheles culicifacies	84.7433	22.2481	Unclassified	GBIF: https://doi.org/10.15468/dl.zfaw79
Anopheles culicifacies	84.7444	22.2482	Unclassified	GBIF: https://doi.org/10.15468/dl.zfaw79
Anopheles culicifacies	84.826	22.035	Unclassified	GBIF: https://doi.org/10.15468/dl.zfaw79
Anopheles culicifacies	84.8263	22.0342	Unclassified	GBIF: https://doi.org/10.15468/dl.zfaw79
Anopheles culicifacies	84.827	22.0362	Unclassified	GBIF: https://doi.org/10.15468/dl.zfaw79
Anopheles culicifacies	84.8271	22.0345	Unclassified	GBIF: https://doi.org/10.15468/dl.zfaw79
Anopheles culicifacies	84.8884	22.258	Unclassified	GBIF: https://doi.org/10.15468/dl.zfaw79
Anopheles culicifacies	84.8995	22.274	Unclassified	GBIF: https://doi.org/10.15468/dl.zfaw79
Anopheles culicifacies	84.8999	22.2743	Unclassified	GBIF: https://doi.org/10.15468/dl.zfaw79
Anopheles culicifacies	84.9001	22.2745	Unclassified	GBIF: https://doi.org/10.15468/dl.zfaw79
Anopheles culicifacies	84.9003	22.2743	Unclassified	GBIF: https://doi.org/10.15468/dl.zfaw79
Anopheles culicifacies	84.9005	22.2746	Unclassified	GBIF: https://doi.org/10.15468/dl.zfaw79
Anopheles culicifacies	84.9713	22.2552	Unclassified	GBIF: https://doi.org/10.15468/dl.zfaw79
Anopheles culicifacies	84.9724	22.2144	Unclassified	GBIF: https://doi.org/10.15468/dl.zfaw79
Anopheles culicifacies	84.9724	22.2554	Unclassified	GBIF: https://doi.org/10.15468/dl.zfaw79
Anopheles culicifacies	84.9725	22.2557	Unclassified	GBIF: https://doi.org/10.15468/dl.zfaw79
Anopheles culicifacies	84.9726	22.2556	Unclassified	GBIF: https://doi.org/10.15468/dl.zfaw79
Anopheles culicifacies	84.9735	22.2092	Unclassified	GBIF: https://doi.org/10.15468/dl.zfaw79
Anopheles culicifacies	84.9737	22.2085	Unclassified	GBIF: https://doi.org/10.15468/dl.zfaw79
Anopheles culicifacies	84.9739	22.2097	Unclassified	GBIF: https://doi.org/10.15468/dl.zfaw79
Anopheles culicifacies	85.042098	27.459029	Unclassified	GBIF: https://doi.org/10.15468/dl.zfaw79
Anopheles culicifacies	97.67712	18.523387	Unclassified	GBIF: https://doi.org/10.15468/dl.zfaw79
Anopheles culicifacies	97.691357	17.886409	Unclassified	GBIF: https://doi.org/10.15468/dl.zfaw79
Anopheles culicifacies	97.78651	17.993799	Unclassified	GBIF: https://doi.org/10.15468/dl.zfaw79
Anopheles culicifacies	97.855902	18.418824	Unclassified	GBIF: https://doi.org/10.15468/dl.zfaw79
Anopheles culicifacies	97.878886	18.378295	Unclassified	GBIF: https://doi.org/10.15468/dl.zfaw79
Anopheles culicifacies	97.91318	17.570554	Unclassified	GBIF: https://doi.org/10.15468/dl.zfaw79
Anopheles culicifacies	97.96116	17.583464	Unclassified	GBIF: https://doi.org/10.15468/dl.zfaw79

Anopheles culicifacies	97.965569	19.220105	Unclassified	GBIF: https://doi.org/10.15468/dl.zfaw79
Anopheles culicifacies	98.009414	17.54756	Unclassified	GBIF: https://doi.org/10.15468/dl.zfaw79
Anopheles culicifacies	98.021648	18.739661	Unclassified	GBIF: https://doi.org/10.15468/dl.zfaw79
Anopheles culicifacies	98.037474	18.317748	Unclassified	GBIF: https://doi.org/10.15468/dl.zfaw79
Anopheles culicifacies	98.114246	18.141096	Unclassified	GBIF: https://doi.org/10.15468/dl.zfaw79
Anopheles culicifacies	98.116815	17.675359	Unclassified	GBIF: https://doi.org/10.15468/dl.zfaw79
Anopheles culicifacies	98.3922	17.182128	Unclassified	GBIF: https://doi.org/10.15468/dl.zfaw79
Anopheles culicifacies	98.5863	17.0803	Unclassified	GBIF: https://doi.org/10.15468/dl.zfaw79
Anopheles culicifacies	98.66423	18.50429	Unclassified	GBIF: https://doi.org/10.15468/dl.zfaw79
Anopheles culicifacies	98.90558	18.91308	Unclassified	GBIF: https://doi.org/10.15468/dl.zfaw79
Anopheles culicifacies	98.9417	18.81187	Unclassified	GBIF: https://doi.org/10.15468/dl.zfaw79
Anopheles culicifacies	99.080245	16.645981	Unclassified	GBIF: https://doi.org/10.15468/dl.zfaw79
Anopheles culicifacies	99.646246	13.403983	Unclassified	GBIF: https://doi.org/10.15468/dl.zfaw79
Anopheles culicifacies	99.64971	13.232429	Unclassified	GBIF: https://doi.org/10.15468/dl.zfaw79
Anopheles culicifacies	99.67948	18.66875	Unclassified	GBIF: https://doi.org/10.15468/dl.zfaw79
Anopheles culicifacies	99.74737	19.01191	Unclassified	GBIF: https://doi.org/10.15468/dl.zfaw79
Anopheles culicifacies	99.80425	18.98456	Unclassified	GBIF: https://doi.org/10.15468/dl.zfaw79
Anopheles culicifacies	99.80954	19.09659	Unclassified	GBIF: https://doi.org/10.15468/dl.zfaw79
Anopheles culicifacies	99.83018	18.46029	Unclassified	GBIF: https://doi.org/10.15468/dl.zfaw79
Anopheles culicifacies	99.86834	18.51434	Unclassified	GBIF: https://doi.org/10.15468/dl.zfaw79
Anopheles culicifacies	99.8965	19.00492	Unclassified	GBIF: https://doi.org/10.15468/dl.zfaw79
Anopheles culicifacies	99.92605	18.67673	Unclassified	GBIF: https://doi.org/10.15468/dl.zfaw79
Anopheles culicifacies	99.93694	18.92971	Unclassified	GBIF: https://doi.org/10.15468/dl.zfaw79
Anopheles culicifacies	99.94302	18.82847	Unclassified	GBIF: https://doi.org/10.15468/dl.zfaw79
Anopheles culicifacies	99.94396	18.82756	Unclassified	GBIF: https://doi.org/10.15468/dl.zfaw79
Anopheles culicifacies	99.9449	18.82665	Unclassified	GBIF: https://doi.org/10.15468/dl.zfaw79
Anopheles culicifacies	99.95563	18.70912	Unclassified	GBIF: https://doi.org/10.15468/dl.zfaw79
Anopheles culicifacies	99.95658	18.70911	Unclassified	GBIF: https://doi.org/10.15468/dl.zfaw79
Anopheles culicifacies	99.98053	18.75146	Unclassified	GBIF: https://doi.org/10.15468/dl.zfaw79
Anopheles culicifacies	99.98811	18.74962	Unclassified	GBIF: https://doi.org/10.15468/dl.zfaw79
Anopheles culicifacies	99.99189	18.74779	Unclassified	GBIF: https://doi.org/10.15468/dl.zfaw79
Anopheles culicifacies	99.99285	18.74869	Unclassified	GBIF: https://doi.org/10.15468/dl.zfaw79
Anopheles culicifacies	99.9938	18.74959	Unclassified	GBIF: https://doi.org/10.15468/dl.zfaw79
Anopheles culicifacies	101.268126	15.693213	Unclassified	GBIF: https://doi.org/10.15468/dl.zfaw79
Anopheles culicifacies	102.430589	12.356449	Unclassified	GBIF: https://doi.org/10.15468/dl.zfaw79
Anopheles culicifacies	102.48322	12.616251	Unclassified	GBIF: https://doi.org/10.15468/dl.zfaw79
Anopheles culicifacies	104.24534	16.83593	Unclassified	GBIF: https://doi.org/10.15468/dl.zfaw79
Anopheles culiciformis	76.100002	25.716666	Unclassified	GBIF: https://doi.org/10.15468/dl.zfaw79
Anopheles cydippis	11.519852	3.898465	Unclassified	GBIF: https://doi.org/10.15468/dl.zfaw79
Anopheles daciae	-8.241992	53.083214	Unclassified	GBIF: https://doi.org/10.15468/dl.zfaw79
Anopheles daciae	-4.34661	53.20205	Unclassified	GBIF: https://doi.org/10.15468/dl.zfaw79
Anopheles daciae	-4.345	53.202	Unclassified	GBIF: https://doi.org/10.15468/dl.zfaw79

Anopheles daciae	-4.10024	50.68478	Unclassified	GBIF: https://doi.org/10.15468/dl.zfaw79
Anopheles daciae	-2.728	51.182	Unclassified	GBIF: https://doi.org/10.15468/dl.zfaw79
Anopheles daciae	-2.727	51.182	Unclassified	GBIF: https://doi.org/10.15468/dl.zfaw79
Anopheles daciae	-2.723	51.182	Unclassified	GBIF: https://doi.org/10.15468/dl.zfaw79
Anopheles daciae	-2.50066	50.92697	Unclassified	GBIF: https://doi.org/10.15468/dl.zfaw79
Anopheles daciae	-2.50056	50.92691	Unclassified	GBIF: https://doi.org/10.15468/dl.zfaw79
Anopheles daciae	-2.50013	50.92692	Unclassified	GBIF: https://doi.org/10.15468/dl.zfaw79
Anopheles daciae	-0.888	51.569	Unclassified	GBIF: https://doi.org/10.15468/dl.zfaw79
Anopheles daciae	0.37876	50.93582	Unclassified	GBIF: https://doi.org/10.15468/dl.zfaw79
Anopheles daciae	0.49	51.467	Unclassified	GBIF: https://doi.org/10.15468/dl.zfaw79
Anopheles daciae	1.577	52.661	Unclassified	GBIF: https://doi.org/10.15468/dl.zfaw79
Anopheles daciae	1.577	52.566	Unclassified	GBIF: https://doi.org/10.15468/dl.zfaw79
Anopheles daciae	1.65319	52.72194	Unclassified	GBIF: https://doi.org/10.15468/dl.zfaw79
Anopheles daciae	1.655	52.722	Unclassified	GBIF: https://doi.org/10.15468/dl.zfaw79
Anopheles daciae	1.69092	52.46865	Unclassified	GBIF: https://doi.org/10.15468/dl.zfaw79
Anopheles daciae	1.69094	52.46883	Unclassified	GBIF: https://doi.org/10.15468/dl.zfaw79
Anopheles daciae	1.693	52.468	Unclassified	GBIF: https://doi.org/10.15468/dl.zfaw79
Anopheles daciae	1.90291	52.62565	Unclassified	GBIF: https://doi.org/10.15468/dl.zfaw79
Anopheles daciae	1.90292	52.62567	Unclassified	GBIF: https://doi.org/10.15468/dl.zfaw79
Anopheles daciae	3.92491	50.41203	Unclassified	GBIF: https://doi.org/10.15468/dl.zfaw79
Anopheles daciae	4.1935	51.21351	Unclassified	GBIF: https://doi.org/10.15468/dl.zfaw79
Anopheles daciae	4.21772	51.25156	Unclassified	GBIF: https://doi.org/10.15468/dl.zfaw79
Anopheles daciae	4.2765	51.27883	Unclassified	GBIF: https://doi.org/10.15468/dl.zfaw79
Anopheles daciae	5.04574	50.3392	Unclassified	GBIF: https://doi.org/10.15468/dl.zfaw79
Anopheles daciae	5.62125	50.99526	Unclassified	GBIF: https://doi.org/10.15468/dl.zfaw79
Anopheles daciae	5.69522	51.01628	Unclassified	GBIF: https://doi.org/10.15468/dl.zfaw79
Anopheles daciae	15.84	57.56	Unclassified	GBIF: https://doi.org/10.15468/dl.zfaw79
Anopheles daciae	16.46	57.8	Unclassified	GBIF: https://doi.org/10.15468/dl.zfaw79
Anopheles daciae	21.46	61.59	Unclassified	GBIF: https://doi.org/10.15468/dl.zfaw79
Anopheles daciae	21.73	61.19	Unclassified	GBIF: https://doi.org/10.15468/dl.zfaw79
Anopheles daciae	23.36	60.94	Unclassified	GBIF: https://doi.org/10.15468/dl.zfaw79
Anopheles daciae	23.6	61.31	Unclassified	GBIF: https://doi.org/10.15468/dl.zfaw79
Anopheles daciae	23.88	61.22	Unclassified	GBIF: https://doi.org/10.15468/dl.zfaw79
Anopheles daciae	24.27	61.1	Unclassified	GBIF: https://doi.org/10.15468/dl.zfaw79
Anopheles daciae	24.39	61.08	Unclassified	GBIF: https://doi.org/10.15468/dl.zfaw79
Anopheles daciae	24.4	61.07	Unclassified	GBIF: https://doi.org/10.15468/dl.zfaw79
Anopheles daciae	24.46	61.05	Unclassified	GBIF: https://doi.org/10.15468/dl.zfaw79
Anopheles daciae	24.77	61.35	Unclassified	GBIF: https://doi.org/10.15468/dl.zfaw79
Anopheles daciae	25.03	63.89	Unclassified	GBIF: https://doi.org/10.15468/dl.zfaw79
Anopheles daciae	25.45	61.99	Unclassified	GBIF: https://doi.org/10.15468/dl.zfaw79
Anopheles daciae	25.82	61.33	Unclassified	GBIF: https://doi.org/10.15468/dl.zfaw79
Anopheles daciae	30.22	52.33	Unclassified	GBIF: https://doi.org/10.15468/dl.zfaw79

Anopheles daciae	31.02	52.42	Unclassified	GBIF: https://doi.org/10.15468/dl.zfaw79
Anopheles daciae	31.68	52.48	Unclassified	GBIF: https://doi.org/10.15468/dl.zfaw79
Anopheles daciae	33.46	52.89	Unclassified	GBIF: https://doi.org/10.15468/dl.zfaw79
Anopheles daciae	34.48	53.22	Unclassified	GBIF: https://doi.org/10.15468/dl.zfaw79
Anopheles daciae	60.1	55	Unclassified	GBIF: https://doi.org/10.15468/dl.zfaw79
Anopheles daciae	69.4	53.3	Unclassified	GBIF: https://doi.org/10.15468/dl.zfaw79
Anopheles daciae	82.6	57.9	Unclassified	GBIF: https://doi.org/10.15468/dl.zfaw79
Anopheles daciae	82.8	54.8	Unclassified	GBIF: https://doi.org/10.15468/dl.zfaw79
Anopheles daciae	82.81	54.83	Unclassified	GBIF: https://doi.org/10.15468/dl.zfaw79
Anopheles daciae	86.1	57	Unclassified	GBIF: https://doi.org/10.15468/dl.zfaw79
Anopheles daciae	88.2	57.3	Unclassified	GBIF: https://doi.org/10.15468/dl.zfaw79
Anopheles darlingi	-92.952778	17.548056	Primary	GBIF: https://doi.org/10.15468/dl.zfaw79
Anopheles darlingi	-92.825555	17.5925	Primary	GBIF: https://doi.org/10.15468/dl.zfaw79
Anopheles darlingi	-91.9575	16.943611	Primary	GBIF: https://doi.org/10.15468/dl.zfaw79
Anopheles darlingi	-91.899167	16.956111	Primary	GBIF: https://doi.org/10.15468/dl.zfaw79
Anopheles darlingi	-91.155555	16.120278	Primary	GBIF: https://doi.org/10.15468/dl.zfaw79
Anopheles darlingi	-91.094444	16.0875	Primary	GBIF: https://doi.org/10.15468/dl.zfaw79
Anopheles darlingi	-90.970833	16.097222	Primary	GBIF: https://doi.org/10.15468/dl.zfaw79
Anopheles darlingi	-90.890278	16.821111	Primary	GBIF: https://doi.org/10.15468/dl.zfaw79
Anopheles darlingi	-90.878055	16.159944	Primary	GBIF: https://doi.org/10.15468/dl.zfaw79
Anopheles darlingi	-90.874167	16.117778	Primary	GBIF: https://doi.org/10.15468/dl.zfaw79
Anopheles darlingi	-90.764167	16.335	Primary	GBIF: https://doi.org/10.15468/dl.zfaw79
Anopheles darlingi	-90.650555	16.518889	Primary	GBIF: https://doi.org/10.15468/dl.zfaw79
Anopheles darlingi	-90.535833	16.269167	Primary	GBIF: https://doi.org/10.15468/dl.zfaw79
Anopheles darlingi	-90.531667	19.842222	Primary	GBIF: https://doi.org/10.15468/dl.zfaw79
Anopheles darlingi	-88.79998	17.25079	Primary	GBIF: https://doi.org/10.15468/dl.zfaw79
Anopheles darlingi	-88.61664	17.1508	Primary	GBIF: https://doi.org/10.15468/dl.zfaw79
Anopheles darlingi	-87.16792	15.68594	Primary	GBIF: https://doi.org/10.15468/dl.zfaw79
Anopheles darlingi	-77.5832	-3.314419	Primary	GBIF: https://doi.org/10.15468/dl.zfaw79
Anopheles darlingi	-76.45	6.17	Primary	GBIF: https://doi.org/10.15468/dl.zfaw79
Anopheles darlingi	-76.125	8.456389	Primary	GBIF: https://doi.org/10.15468/dl.zfaw79
Anopheles darlingi	-75.51	7.43	Primary	GBIF: https://doi.org/10.15468/dl.zfaw79
Anopheles darlingi	-75.33	6.28	Primary	GBIF: https://doi.org/10.15468/dl.zfaw79
Anopheles darlingi	-74.71111	7.60669	Primary	GBIF: https://doi.org/10.15468/dl.zfaw79
Anopheles darlingi	-74.71	7.61	Primary	GBIF: https://doi.org/10.15468/dl.zfaw79
Anopheles darlingi	-74.71	7.68	Primary	GBIF: https://doi.org/10.15468/dl.zfaw79
Anopheles darlingi	-74.70928	7.67714	Primary	GBIF: https://doi.org/10.15468/dl.zfaw79
Anopheles darlingi	-74.66389	7.49094	Primary	GBIF: https://doi.org/10.15468/dl.zfaw79
Anopheles darlingi	-74.66008	7.72008	Primary	GBIF: https://doi.org/10.15468/dl.zfaw79
Anopheles darlingi	-74.66	7.72	Primary	GBIF: https://doi.org/10.15468/dl.zfaw79
Anopheles darlingi	-74.66	7.49	Primary	GBIF: https://doi.org/10.15468/dl.zfaw79
Anopheles darlingi	-74.53	-0.1	Primary	GBIF: https://doi.org/10.15468/dl.zfaw79

Anopheles darlingi	-74.49	7.35	Primary	GBIF: https://doi.org/10.15468/dl.zfaw79
Anopheles darlingi	-73.64164	4.14812	Primary	GBIF: https://doi.org/10.15468/dl.zfaw79
Anopheles darlingi	-73.628547	4.149169	Primary	GBIF: https://doi.org/10.15468/dl.zfaw79
Anopheles darlingi	-73.61867	4.14669	Primary	GBIF: https://doi.org/10.15468/dl.zfaw79
Anopheles darlingi	-73.61867	4.31336	Primary	GBIF: https://doi.org/10.15468/dl.zfaw79
Anopheles darlingi	-73.19	4.13	Primary	GBIF: https://doi.org/10.15468/dl.zfaw79
Anopheles darlingi	-72.93171	2.65014	Primary	GBIF: https://doi.org/10.15468/dl.zfaw79
Anopheles darlingi	-72.88605	-7.6205108	Primary	GBIF: https://doi.org/10.15468/dl.zfaw79
Anopheles darlingi	-72.87	-7.626	Primary	GBIF: https://doi.org/10.15468/dl.zfaw79
Anopheles darlingi	-72.87	-7.63	Primary	GBIF: https://doi.org/10.15468/dl.zfaw79
Anopheles darlingi	-72.7030903	-7.4728368	Primary	GBIF: https://doi.org/10.15468/dl.zfaw79
Anopheles darlingi	-72.69	-7.631	Primary	GBIF: https://doi.org/10.15468/dl.zfaw79
Anopheles darlingi	-72.69	-7.63	Primary	GBIF: https://doi.org/10.15468/dl.zfaw79
Anopheles darlingi	-72.65	-7.67	Primary	GBIF: https://doi.org/10.15468/dl.zfaw79
Anopheles darlingi	-72.64451	4.187754	Primary	GBIF: https://doi.org/10.15468/dl.zfaw79
Anopheles darlingi	-69.45	2.53	Primary	GBIF: https://doi.org/10.15468/dl.zfaw79
Anopheles darlingi	-67.1278721	-9.7236312	Primary	GBIF: https://doi.org/10.15468/dl.zfaw79
Anopheles darlingi	-67.112	-9.776	Primary	GBIF: https://doi.org/10.15468/dl.zfaw79
Anopheles darlingi	-67.11	-9.78	Primary	GBIF: https://doi.org/10.15468/dl.zfaw79
Anopheles darlingi	-67.03	-9.69	Primary	GBIF: https://doi.org/10.15468/dl.zfaw79
Anopheles darlingi	-67.0052428	-0.1566223	Primary	GBIF: https://doi.org/10.15468/dl.zfaw79
Anopheles darlingi	-66.9672568	-9.9269789	Primary	GBIF: https://doi.org/10.15468/dl.zfaw79
Anopheles darlingi	-66.91072	-10.12328	Primary	GBIF: https://doi.org/10.15468/dl.zfaw79
Anopheles darlingi	-66.91	-10.12	Primary	GBIF: https://doi.org/10.15468/dl.zfaw79
Anopheles darlingi	-64.98	7.06	Primary	GBIF: https://doi.org/10.15468/dl.zfaw79
Anopheles darlingi	-64.97694	7.06194	Primary	GBIF: https://doi.org/10.15468/dl.zfaw79
Anopheles darlingi	-64.8232	6.574	Primary	GBIF: https://doi.org/10.15468/dl.zfaw79
Anopheles darlingi	-64.697963	-7.5248396	Primary	GBIF: https://doi.org/10.15468/dl.zfaw79
Anopheles darlingi	-64.68	-7.4	Primary	GBIF: https://doi.org/10.15468/dl.zfaw79
Anopheles darlingi	-64.677	-7.405	Primary	GBIF: https://doi.org/10.15468/dl.zfaw79
Anopheles darlingi	-64.663	-7.382	Primary	GBIF: https://doi.org/10.15468/dl.zfaw79
Anopheles darlingi	-64.66	-7.38	Primary	GBIF: https://doi.org/10.15468/dl.zfaw79
Anopheles darlingi	-64.227203	-12.445	Primary	GBIF: https://doi.org/10.15468/dl.zfaw79
Anopheles darlingi	-63.902557	3.241979	Primary	GBIF: https://doi.org/10.15468/dl.zfaw79
Anopheles darlingi	-63.9	3.24	Primary	GBIF: https://doi.org/10.15468/dl.zfaw79
Anopheles darlingi	-63.2153957	-7.2785534	Primary	GBIF: https://doi.org/10.15468/dl.zfaw79
Anopheles darlingi	-63.1428	-4.0822	Primary	GBIF: https://doi.org/10.15468/dl.zfaw79
Anopheles darlingi	-63.040798	-9.91333	Primary	GBIF: https://doi.org/10.15468/dl.zfaw79
Anopheles darlingi	-62.283861	-9.2392474	Primary	GBIF: https://doi.org/10.15468/dl.zfaw79
Anopheles darlingi	-60.8206936	-7.637897	Primary	GBIF: https://doi.org/10.15468/dl.zfaw79
Anopheles darlingi	-60.5	-11	Primary	GBIF: https://doi.org/10.15468/dl.zfaw79
Anopheles darlingi	-60.2082701	-1.5595684	Primary	GBIF: https://doi.org/10.15468/dl.zfaw79

Anopheles darlingi	-59.1186615	-3.0713641	Primary	GBIF: https://doi.org/10.15468/dl.zfaw79
Anopheles darlingi	-59.073	-3.094	Primary	GBIF: https://doi.org/10.15468/dl.zfaw79
Anopheles darlingi	-59.07	-3.09	Primary	GBIF: https://doi.org/10.15468/dl.zfaw79
Anopheles darlingi	-56.831111	5.585278	Primary	GBIF: https://doi.org/10.15468/dl.zfaw79
Anopheles darlingi	-56.3783	-20.240601	Primary	GBIF: https://doi.org/10.15468/dl.zfaw79
Anopheles darlingi	-54.464938	4.811023	Primary	GBIF: https://doi.org/10.15468/dl.zfaw79
Anopheles darlingi	-54.442731	4.993306	Primary	GBIF: https://doi.org/10.15468/dl.zfaw79
Anopheles darlingi	-54.435506	4.990306	Primary	GBIF: https://doi.org/10.15468/dl.zfaw79
Anopheles darlingi	-54.420585	5.041128	Primary	GBIF: https://doi.org/10.15468/dl.zfaw79
Anopheles darlingi	-54.416695	5.033348	Primary	GBIF: https://doi.org/10.15468/dl.zfaw79
Anopheles darlingi	-54.415345	4.619739	Primary	GBIF: https://doi.org/10.15468/dl.zfaw79
Anopheles darlingi	-54.41527	4.618084	Primary	GBIF: https://doi.org/10.15468/dl.zfaw79
Anopheles darlingi	-54.398333	5.608056	Primary	GBIF: https://doi.org/10.15468/dl.zfaw79
Anopheles darlingi	-54.383048	4.27418	Primary	GBIF: https://doi.org/10.15468/dl.zfaw79
Anopheles darlingi	-54.376135	5.102238	Primary	GBIF: https://doi.org/10.15468/dl.zfaw79
Anopheles darlingi	-54.366695	4.316688	Primary	GBIF: https://doi.org/10.15468/dl.zfaw79
Anopheles darlingi	-54.344163	5.155783	Primary	GBIF: https://doi.org/10.15468/dl.zfaw79
Anopheles darlingi	-54.25	-24.0667	Primary	GBIF: https://doi.org/10.15468/dl.zfaw79
Anopheles darlingi	-54.198355	3.822518	Primary	GBIF: https://doi.org/10.15468/dl.zfaw79
Anopheles darlingi	-54.192245	3.826958	Primary	GBIF: https://doi.org/10.15468/dl.zfaw79
Anopheles darlingi	-54.151439	3.80651	Primary	GBIF: https://doi.org/10.15468/dl.zfaw79
Anopheles darlingi	-54.148707	3.807339	Primary	GBIF: https://doi.org/10.15468/dl.zfaw79
Anopheles darlingi	-54.141692	3.808351	Primary	GBIF: https://doi.org/10.15468/dl.zfaw79
Anopheles darlingi	-54.070388	3.298274	Primary	GBIF: https://doi.org/10.15468/dl.zfaw79
Anopheles darlingi	-54.053915	3.383908	Primary	GBIF: https://doi.org/10.15468/dl.zfaw79
Anopheles darlingi	-54.050025	3.383348	Primary	GBIF: https://doi.org/10.15468/dl.zfaw79
Anopheles darlingi	-54.046695	3.635018	Primary	GBIF: https://doi.org/10.15468/dl.zfaw79
Anopheles darlingi	-54.045577	5.480947	Primary	GBIF: https://doi.org/10.15468/dl.zfaw79
Anopheles darlingi	-54.04329	5.484058	Primary	GBIF: https://doi.org/10.15468/dl.zfaw79
Anopheles darlingi	-54.033355	3.366688	Primary	GBIF: https://doi.org/10.15468/dl.zfaw79
Anopheles darlingi	-54.031415	3.643348	Primary	GBIF: https://doi.org/10.15468/dl.zfaw79
Anopheles darlingi	-54.028939	5.503342	Primary	GBIF: https://doi.org/10.15468/dl.zfaw79
Anopheles darlingi	-54.02004	5.50717	Primary	GBIF: https://doi.org/10.15468/dl.zfaw79
Anopheles darlingi	-54.000025	4.766684	Primary	GBIF: https://doi.org/10.15468/dl.zfaw79
Anopheles darlingi	-53.916695	3.383348	Primary	GBIF: https://doi.org/10.15468/dl.zfaw79
Anopheles darlingi	-53.83532	3.32363	Primary	GBIF: https://doi.org/10.15468/dl.zfaw79
Anopheles darlingi	-53.778092	5.66898	Primary	GBIF: https://doi.org/10.15468/dl.zfaw79
Anopheles darlingi	-53.74599	5.638017	Primary	GBIF: https://doi.org/10.15468/dl.zfaw79
Anopheles darlingi	-53.674595	5.394268	Primary	GBIF: https://doi.org/10.15468/dl.zfaw79
Anopheles darlingi	-53.629005	4.559398	Primary	GBIF: https://doi.org/10.15468/dl.zfaw79
Anopheles darlingi	-53.582045	3.602948	Primary	GBIF: https://doi.org/10.15468/dl.zfaw79
Anopheles darlingi	-53.549917	3.749968	Primary	GBIF: https://doi.org/10.15468/dl.zfaw79

Anopheles darlingi	-53.452738	5.546529	Primary	GBIF: https://doi.org/10.15468/dl.zfaw79
Anopheles darlingi	-53.450025	3.950018	Primary	GBIF: https://doi.org/10.15468/dl.zfaw79
Anopheles darlingi	-53.391025	3.866018	Primary	GBIF: https://doi.org/10.15468/dl.zfaw79
Anopheles darlingi	-53.33161	3.82207	Primary	GBIF: https://doi.org/10.15468/dl.zfaw79
Anopheles darlingi	-53.288005	4.825571	Primary	GBIF: https://doi.org/10.15468/dl.zfaw79
Anopheles darlingi	-53.2745	3.81601	Primary	GBIF: https://doi.org/10.15468/dl.zfaw79
Anopheles darlingi	-53.24036	3.91451	Primary	GBIF: https://doi.org/10.15468/dl.zfaw79
Anopheles darlingi	-53.240025	5.469148	Primary	GBIF: https://doi.org/10.15468/dl.zfaw79
Anopheles darlingi	-53.23783	5.50331	Primary	GBIF: https://doi.org/10.15468/dl.zfaw79
Anopheles darlingi	-53.23477	3.92404	Primary	GBIF: https://doi.org/10.15468/dl.zfaw79
Anopheles darlingi	-53.210985	3.623926	Primary	GBIF: https://doi.org/10.15468/dl.zfaw79
Anopheles darlingi	-53.208785	3.621228	Primary	GBIF: https://doi.org/10.15468/dl.zfaw79
Anopheles darlingi	-53.20636	3.628085	Primary	GBIF: https://doi.org/10.15468/dl.zfaw79
Anopheles darlingi	-53.205199	5.47958	Primary	GBIF: https://doi.org/10.15468/dl.zfaw79
Anopheles darlingi	-53.17007	5.52203	Primary	GBIF: https://doi.org/10.15468/dl.zfaw79
Anopheles darlingi	-53.13625	5.45004	Primary	GBIF: https://doi.org/10.15468/dl.zfaw79
Anopheles darlingi	-53.133225	4.955878	Primary	GBIF: https://doi.org/10.15468/dl.zfaw79
Anopheles darlingi	-53.132825	3.608848	Primary	GBIF: https://doi.org/10.15468/dl.zfaw79
Anopheles darlingi	-53.08936	5.42827	Primary	GBIF: https://doi.org/10.15468/dl.zfaw79
Anopheles darlingi	-53.083355	5.416688	Primary	GBIF: https://doi.org/10.15468/dl.zfaw79
Anopheles darlingi	-53.0745	5.4234	Primary	GBIF: https://doi.org/10.15468/dl.zfaw79
Anopheles darlingi	-53.05649	5.4199	Primary	GBIF: https://doi.org/10.15468/dl.zfaw79
Anopheles darlingi	-53.050025	5.062018	Primary	GBIF: https://doi.org/10.15468/dl.zfaw79
Anopheles darlingi	-53.03179	5.41668	Primary	GBIF: https://doi.org/10.15468/dl.zfaw79
Anopheles darlingi	-53.02915	5.01314	Primary	GBIF: https://doi.org/10.15468/dl.zfaw79
Anopheles darlingi	-52.99943	5.40638	Primary	GBIF: https://doi.org/10.15468/dl.zfaw79
Anopheles darlingi	-52.983355	5.383348	Primary	GBIF: https://doi.org/10.15468/dl.zfaw79
Anopheles darlingi	-52.958187	5.377037	Primary	GBIF: https://doi.org/10.15468/dl.zfaw79
Anopheles darlingi	-52.950025	5.316688	Primary	GBIF: https://doi.org/10.15468/dl.zfaw79
Anopheles darlingi	-52.900025	5.166684	Primary	GBIF: https://doi.org/10.15468/dl.zfaw79
Anopheles darlingi	-52.87724	2.24504	Primary	GBIF: https://doi.org/10.15468/dl.zfaw79
Anopheles darlingi	-52.873971	2.247312	Primary	GBIF: https://doi.org/10.15468/dl.zfaw79
Anopheles darlingi	-52.873058	2.257084	Primary	GBIF: https://doi.org/10.15468/dl.zfaw79
Anopheles darlingi	-52.866695	3.416688	Primary	GBIF: https://doi.org/10.15468/dl.zfaw79
Anopheles darlingi	-52.846531	5.347491	Primary	GBIF: https://doi.org/10.15468/dl.zfaw79
Anopheles darlingi	-52.834808	2.293751	Primary	GBIF: https://doi.org/10.15468/dl.zfaw79
Anopheles darlingi	-52.8307	5.29361	Primary	GBIF: https://doi.org/10.15468/dl.zfaw79
Anopheles darlingi	-52.756745	5.153398	Primary	GBIF: https://doi.org/10.15468/dl.zfaw79
Anopheles darlingi	-52.738605	5.115338	Primary	GBIF: https://doi.org/10.15468/dl.zfaw79
Anopheles darlingi	-52.700025	5.016688	Primary	GBIF: https://doi.org/10.15468/dl.zfaw79
Anopheles darlingi	-52.700025	4.033351	Primary	GBIF: https://doi.org/10.15468/dl.zfaw79
Anopheles darlingi	-52.67767	3.56169	Primary	GBIF: https://doi.org/10.15468/dl.zfaw79

Anopheles darlingi	-52.66862	5.16483	Primary	GBIF: https://doi.org/10.15468/dl.zfaw79
Anopheles darlingi	-52.643374	5.166931	Primary	GBIF: https://doi.org/10.15468/dl.zfaw79
Anopheles darlingi	-52.63905	5.15894	Primary	GBIF: https://doi.org/10.15468/dl.zfaw79
Anopheles darlingi	-52.593636	3.256962	Primary	GBIF: https://doi.org/10.15468/dl.zfaw79
Anopheles darlingi	-52.57423	5.03313	Primary	GBIF: https://doi.org/10.15468/dl.zfaw79
Anopheles darlingi	-52.5221	4.88575	Primary	GBIF: https://doi.org/10.15468/dl.zfaw79
Anopheles darlingi	-52.516692	2.650018	Primary	GBIF: https://doi.org/10.15468/dl.zfaw79
Anopheles darlingi	-52.516445	4.901678	Primary	GBIF: https://doi.org/10.15468/dl.zfaw79
Anopheles darlingi	-52.509685	4.832668	Primary	GBIF: https://doi.org/10.15468/dl.zfaw79
Anopheles darlingi	-52.508494	4.99794	Primary	GBIF: https://doi.org/10.15468/dl.zfaw79
Anopheles darlingi	-52.500025	4.950018	Primary	GBIF: https://doi.org/10.15468/dl.zfaw79
Anopheles darlingi	-52.500025	3.383348	Primary	GBIF: https://doi.org/10.15468/dl.zfaw79
Anopheles darlingi	-52.492926	4.892476	Primary	GBIF: https://doi.org/10.15468/dl.zfaw79
Anopheles darlingi	-52.492261	4.560134	Primary	GBIF: https://doi.org/10.15468/dl.zfaw79
Anopheles darlingi	-52.483358	2.783351	Primary	GBIF: https://doi.org/10.15468/dl.zfaw79
Anopheles darlingi	-52.483355	4.666688	Primary	GBIF: https://doi.org/10.15468/dl.zfaw79
Anopheles darlingi	-52.481135	4.561623	Primary	GBIF: https://doi.org/10.15468/dl.zfaw79
Anopheles darlingi	-52.477845	4.557297	Primary	GBIF: https://doi.org/10.15468/dl.zfaw79
Anopheles darlingi	-52.47665	4.567727	Primary	GBIF: https://doi.org/10.15468/dl.zfaw79
Anopheles darlingi	-52.476399	4.568433	Primary	GBIF: https://doi.org/10.15468/dl.zfaw79
Anopheles darlingi	-52.475633	4.564139	Primary	GBIF: https://doi.org/10.15468/dl.zfaw79
Anopheles darlingi	-52.473837	5.013996	Primary	GBIF: https://doi.org/10.15468/dl.zfaw79
Anopheles darlingi	-52.473699	4.574534	Primary	GBIF: https://doi.org/10.15468/dl.zfaw79
Anopheles darlingi	-52.47329	4.550868	Primary	GBIF: https://doi.org/10.15468/dl.zfaw79
Anopheles darlingi	-52.473105	4.552939	Primary	GBIF: https://doi.org/10.15468/dl.zfaw79
Anopheles darlingi	-52.47241	5.01171	Primary	GBIF: https://doi.org/10.15468/dl.zfaw79
Anopheles darlingi	-52.47175	4.573209	Primary	GBIF: https://doi.org/10.15468/dl.zfaw79
Anopheles darlingi	-52.470895	4.574205	Primary	GBIF: https://doi.org/10.15468/dl.zfaw79
Anopheles darlingi	-52.47027	4.572008	Primary	GBIF: https://doi.org/10.15468/dl.zfaw79
Anopheles darlingi	-52.469703	4.564142	Primary	GBIF: https://doi.org/10.15468/dl.zfaw79
Anopheles darlingi	-52.468501	4.571289	Primary	GBIF: https://doi.org/10.15468/dl.zfaw79
Anopheles darlingi	-52.467313	4.580697	Primary	GBIF: https://doi.org/10.15468/dl.zfaw79
Anopheles darlingi	-52.46505	4.968779	Primary	GBIF: https://doi.org/10.15468/dl.zfaw79
Anopheles darlingi	-52.464115	4.563611	Primary	GBIF: https://doi.org/10.15468/dl.zfaw79
Anopheles darlingi	-52.46407	4.563566	Primary	GBIF: https://doi.org/10.15468/dl.zfaw79
Anopheles darlingi	-52.464045	4.988308	Primary	GBIF: https://doi.org/10.15468/dl.zfaw79
Anopheles darlingi	-52.463631	4.560528	Primary	GBIF: https://doi.org/10.15468/dl.zfaw79
Anopheles darlingi	-52.462674	4.559987	Primary	GBIF: https://doi.org/10.15468/dl.zfaw79
Anopheles darlingi	-52.462398	4.561507	Primary	GBIF: https://doi.org/10.15468/dl.zfaw79
Anopheles darlingi	-52.461504	4.578502	Primary	GBIF: https://doi.org/10.15468/dl.zfaw79
Anopheles darlingi	-52.461363	4.579995	Primary	GBIF: https://doi.org/10.15468/dl.zfaw79
Anopheles darlingi	-52.46114	4.576432	Primary	GBIF: https://doi.org/10.15468/dl.zfaw79

Anopheles darlingi	-52.45662	4.792945	Primary	GBIF: https://doi.org/10.15468/dl.zfaw79
Anopheles darlingi	-52.45627	4.94759	Primary	GBIF: https://doi.org/10.15468/dl.zfaw79
Anopheles darlingi	-52.443289	4.83079	Primary	GBIF: https://doi.org/10.15468/dl.zfaw79
Anopheles darlingi	-52.413525	4.563701	Primary	GBIF: https://doi.org/10.15468/dl.zfaw79
Anopheles darlingi	-52.406535	4.910838	Primary	GBIF: https://doi.org/10.15468/dl.zfaw79
Anopheles darlingi	-52.40423	4.94154	Primary	GBIF: https://doi.org/10.15468/dl.zfaw79
Anopheles darlingi	-52.383355	4.883348	Primary	GBIF: https://doi.org/10.15468/dl.zfaw79
Anopheles darlingi	-52.371136	4.878629	Primary	GBIF: https://doi.org/10.15468/dl.zfaw79
Anopheles darlingi	-52.366692	3.000018	Primary	GBIF: https://doi.org/10.15468/dl.zfaw79
Anopheles darlingi	-52.360475	4.819828	Primary	GBIF: https://doi.org/10.15468/dl.zfaw79
Anopheles darlingi	-52.35913	4.90255	Primary	GBIF: https://doi.org/10.15468/dl.zfaw79
Anopheles darlingi	-52.357135	4.886868	Primary	GBIF: https://doi.org/10.15468/dl.zfaw79
Anopheles darlingi	-52.356136	4.885296	Primary	GBIF: https://doi.org/10.15468/dl.zfaw79
Anopheles darlingi	-52.3543	4.7921	Primary	GBIF: https://doi.org/10.15468/dl.zfaw79
Anopheles darlingi	-52.351905	4.788018	Primary	GBIF: https://doi.org/10.15468/dl.zfaw79
Anopheles darlingi	-52.34913	4.83642	Primary	GBIF: https://doi.org/10.15468/dl.zfaw79
Anopheles darlingi	-52.348155	4.886318	Primary	GBIF: https://doi.org/10.15468/dl.zfaw79
Anopheles darlingi	-52.346175	4.889698	Primary	GBIF: https://doi.org/10.15468/dl.zfaw79
Anopheles darlingi	-52.344908	4.820517	Primary	GBIF: https://doi.org/10.15468/dl.zfaw79
Anopheles darlingi	-52.34352	4.77908	Primary	GBIF: https://doi.org/10.15468/dl.zfaw79
Anopheles darlingi	-52.3435	4.88147	Primary	GBIF: https://doi.org/10.15468/dl.zfaw79
Anopheles darlingi	-52.34241	4.89956	Primary	GBIF: https://doi.org/10.15468/dl.zfaw79
Anopheles darlingi	-52.341575	4.823418	Primary	GBIF: https://doi.org/10.15468/dl.zfaw79
Anopheles darlingi	-52.34122	4.89382	Primary	GBIF: https://doi.org/10.15468/dl.zfaw79
Anopheles darlingi	-52.341025	4.885808	Primary	GBIF: https://doi.org/10.15468/dl.zfaw79
Anopheles darlingi	-52.340858	4.894185	Primary	GBIF: https://doi.org/10.15468/dl.zfaw79
Anopheles darlingi	-52.340187	4.795619	Primary	GBIF: https://doi.org/10.15468/dl.zfaw79
Anopheles darlingi	-52.34003	4.81928	Primary	GBIF: https://doi.org/10.15468/dl.zfaw79
Anopheles darlingi	-52.33772	4.87499	Primary	GBIF: https://doi.org/10.15468/dl.zfaw79
Anopheles darlingi	-52.33696	4.83273	Primary	GBIF: https://doi.org/10.15468/dl.zfaw79
Anopheles darlingi	-52.334255	4.788748	Primary	GBIF: https://doi.org/10.15468/dl.zfaw79
Anopheles darlingi	-52.334095	3.160908	Primary	GBIF: https://doi.org/10.15468/dl.zfaw79
Anopheles darlingi	-52.333605	3.167747	Primary	GBIF: https://doi.org/10.15468/dl.zfaw79
Anopheles darlingi	-52.333355	4.833348	Primary	GBIF: https://doi.org/10.15468/dl.zfaw79
Anopheles darlingi	-52.333355	4.850018	Primary	GBIF: https://doi.org/10.15468/dl.zfaw79
Anopheles darlingi	-52.333355	4.733348	Primary	GBIF: https://doi.org/10.15468/dl.zfaw79
Anopheles darlingi	-52.333115	4.816198	Primary	GBIF: https://doi.org/10.15468/dl.zfaw79
Anopheles darlingi	-52.332371	4.767255	Primary	GBIF: https://doi.org/10.15468/dl.zfaw79
Anopheles darlingi	-52.330987	4.850608	Primary	GBIF: https://doi.org/10.15468/dl.zfaw79
Anopheles darlingi	-52.33033	4.93461	Primary	GBIF: https://doi.org/10.15468/dl.zfaw79
Anopheles darlingi	-52.329786	3.170172	Primary	GBIF: https://doi.org/10.15468/dl.zfaw79
Anopheles darlingi	-52.328392	3.172584	Primary	GBIF: https://doi.org/10.15468/dl.zfaw79

Anopheles darlingi	-52.327445	4.726308	Primary	GBIF: https://doi.org/10.15468/dl.zfaw79
Anopheles darlingi	-52.326965	4.883998	Primary	GBIF: https://doi.org/10.15468/dl.zfaw79
Anopheles darlingi	-52.32628	4.727944	Primary	GBIF: https://doi.org/10.15468/dl.zfaw79
Anopheles darlingi	-52.32436	4.8527	Primary	GBIF: https://doi.org/10.15468/dl.zfaw79
Anopheles darlingi	-52.323296	4.936976	Primary	GBIF: https://doi.org/10.15468/dl.zfaw79
Anopheles darlingi	-52.32266	4.84545	Primary	GBIF: https://doi.org/10.15468/dl.zfaw79
Anopheles darlingi	-52.321842	3.171168	Primary	GBIF: https://doi.org/10.15468/dl.zfaw79
Anopheles darlingi	-52.32059	4.73866	Primary	GBIF: https://doi.org/10.15468/dl.zfaw79
Anopheles darlingi	-52.319958	4.943506	Primary	GBIF: https://doi.org/10.15468/dl.zfaw79
Anopheles darlingi	-52.319815	4.849048	Primary	GBIF: https://doi.org/10.15468/dl.zfaw79
Anopheles darlingi	-52.3192	4.88391	Primary	GBIF: https://doi.org/10.15468/dl.zfaw79
Anopheles darlingi	-52.316695	4.516688	Primary	GBIF: https://doi.org/10.15468/dl.zfaw79
Anopheles darlingi	-52.315858	4.873629	Primary	GBIF: https://doi.org/10.15468/dl.zfaw79
Anopheles darlingi	-52.312455	4.927798	Primary	GBIF: https://doi.org/10.15468/dl.zfaw79
Anopheles darlingi	-52.30826	4.83195	Primary	GBIF: https://doi.org/10.15468/dl.zfaw79
Anopheles darlingi	-52.307803	4.890296	Primary	GBIF: https://doi.org/10.15468/dl.zfaw79
Anopheles darlingi	-52.307335	4.833438	Primary	GBIF: https://doi.org/10.15468/dl.zfaw79
Anopheles darlingi	-52.307198	4.627222	Primary	GBIF: https://doi.org/10.15468/dl.zfaw79
Anopheles darlingi	-52.306695	4.940978	Primary	GBIF: https://doi.org/10.15468/dl.zfaw79
Anopheles darlingi	-52.30318	4.91254	Primary	GBIF: https://doi.org/10.15468/dl.zfaw79
Anopheles darlingi	-52.29797	4.90772	Primary	GBIF: https://doi.org/10.15468/dl.zfaw79
Anopheles darlingi	-52.291065	4.890268	Primary	GBIF: https://doi.org/10.15468/dl.zfaw79
Anopheles darlingi	-52.28659	4.89308	Primary	GBIF: https://doi.org/10.15468/dl.zfaw79
Anopheles darlingi	-52.28402	4.91235	Primary	GBIF: https://doi.org/10.15468/dl.zfaw79
Anopheles darlingi	-52.279846	4.717854	Primary	GBIF: https://doi.org/10.15468/dl.zfaw79
Anopheles darlingi	-52.279143	4.718157	Primary	GBIF: https://doi.org/10.15468/dl.zfaw79
Anopheles darlingi	-52.276221	4.886853	Primary	GBIF: https://doi.org/10.15468/dl.zfaw79
Anopheles darlingi	-52.27475	4.85384	Primary	GBIF: https://doi.org/10.15468/dl.zfaw79
Anopheles darlingi	-52.273358	4.897518	Primary	GBIF: https://doi.org/10.15468/dl.zfaw79
Anopheles darlingi	-52.272123	4.912182	Primary	GBIF: https://doi.org/10.15468/dl.zfaw79
Anopheles darlingi	-52.268325	3.273818	Primary	GBIF: https://doi.org/10.15468/dl.zfaw79
Anopheles darlingi	-52.262164	3.245379	Primary	GBIF: https://doi.org/10.15468/dl.zfaw79
Anopheles darlingi	-52.256515	4.857598	Primary	GBIF: https://doi.org/10.15468/dl.zfaw79
Anopheles darlingi	-52.251475	4.881148	Primary	GBIF: https://doi.org/10.15468/dl.zfaw79
Anopheles darlingi	-52.21	-3.2	Primary	GBIF: https://doi.org/10.15468/dl.zfaw79
Anopheles darlingi	-52.153995	4.315458	Primary	GBIF: https://doi.org/10.15468/dl.zfaw79
Anopheles darlingi	-52.13743	4.7904	Primary	GBIF: https://doi.org/10.15468/dl.zfaw79
Anopheles darlingi	-52.129162	4.314002	Primary	GBIF: https://doi.org/10.15468/dl.zfaw79
Anopheles darlingi	-52.037094	4.486403	Primary	GBIF: https://doi.org/10.15468/dl.zfaw79
Anopheles darlingi	-51.88333	3.8	Primary	GBIF: https://doi.org/10.15468/dl.zfaw79
Anopheles darlingi	-51.855225	3.870678	Primary	GBIF: https://doi.org/10.15468/dl.zfaw79
Anopheles darlingi	-51.838914	3.833407	Primary	GBIF: https://doi.org/10.15468/dl.zfaw79

Anopheles darlingi	-51.838469	3.839657	Primary	GBIF: https://doi.org/10.15468/dl.zfaw79
Anopheles darlingi	-51.838247	3.833546	Primary	GBIF: https://doi.org/10.15468/dl.zfaw79
Anopheles darlingi	-51.838081	3.834323	Primary	GBIF: https://doi.org/10.15468/dl.zfaw79
Anopheles darlingi	-51.838081	3.834324	Primary	GBIF: https://doi.org/10.15468/dl.zfaw79
Anopheles darlingi	-51.837719	3.837379	Primary	GBIF: https://doi.org/10.15468/dl.zfaw79
Anopheles darlingi	-51.815192	3.875351	Primary	GBIF: https://doi.org/10.15468/dl.zfaw79
Anopheles darlingi	-51.808359	3.891601	Primary	GBIF: https://doi.org/10.15468/dl.zfaw79
Anopheles darlingi	-51.806695	3.888638	Primary	GBIF: https://doi.org/10.15468/dl.zfaw79
Anopheles darlingi	-51.805914	3.898851	Primary	GBIF: https://doi.org/10.15468/dl.zfaw79
Anopheles darlingi	-51.802995	3.889048	Primary	GBIF: https://doi.org/10.15468/dl.zfaw79
Anopheles darlingi	-51.801756	3.895797	Primary	GBIF: https://doi.org/10.15468/dl.zfaw79
Anopheles darlingi	-51.801414	3.898407	Primary	GBIF: https://doi.org/10.15468/dl.zfaw79
Anopheles darlingi	-51.801158	3.888398	Primary	GBIF: https://doi.org/10.15468/dl.zfaw79
Anopheles darlingi	-51.800609	3.896323	Primary	GBIF: https://doi.org/10.15468/dl.zfaw79
Anopheles darlingi	-51.800025	3.900018	Primary	GBIF: https://doi.org/10.15468/dl.zfaw79
Anopheles darlingi	-51.799135	3.891518	Primary	GBIF: https://doi.org/10.15468/dl.zfaw79
Anopheles darlingi	-51.798581	3.895712	Primary	GBIF: https://doi.org/10.15468/dl.zfaw79
Anopheles darlingi	-51.797616	3.896421	Primary	GBIF: https://doi.org/10.15468/dl.zfaw79
Anopheles darlingi	-51.79697	3.913685	Primary	GBIF: https://doi.org/10.15468/dl.zfaw79
Anopheles darlingi	-51.795969	3.878796	Primary	GBIF: https://doi.org/10.15468/dl.zfaw79
Anopheles darlingi	-51.794692	3.888657	Primary	GBIF: https://doi.org/10.15468/dl.zfaw79
Anopheles darlingi	-51.792636	3.877351	Primary	GBIF: https://doi.org/10.15468/dl.zfaw79
Anopheles darlingi	-51.78594	3.94548	Primary	GBIF: https://doi.org/10.15468/dl.zfaw79
Anopheles darlingi	-51.775025	4.166684	Primary	GBIF: https://doi.org/10.15468/dl.zfaw79
Anopheles darlingi	-51.675168	4.051384	Primary	GBIF: https://doi.org/10.15468/dl.zfaw79
Anopheles darlingi	-50.97217	0.21314	Primary	GBIF: https://doi.org/10.15468/dl.zfaw79
Anopheles darlingi	-50.97	0.21	Primary	GBIF: https://doi.org/10.15468/dl.zfaw79
Anopheles darlingi	-50.9	0.27	Primary	GBIF: https://doi.org/10.15468/dl.zfaw79
Anopheles darlingi	-50.89814	0.27153	Primary	GBIF: https://doi.org/10.15468/dl.zfaw79
Anopheles darlingi	-50.3062419	-3.4102363	Primary	GBIF: https://doi.org/10.15468/dl.zfaw79
Anopheles darlingi	-48.504398	-1.45583	Primary	GBIF: https://doi.org/10.15468/dl.zfaw79
Anopheles darlingi	-48.408575	-1.409889	Primary	GBIF: https://doi.org/10.15468/dl.zfaw79
Anopheles darlingi	-47.32	-1.2	Primary	GBIF: https://doi.org/10.15468/dl.zfaw79
Anopheles darlingi	84.3175	43.39389	Primary	GBIF: https://doi.org/10.15468/dl.zfaw79
Anopheles darlingi	84.31778	43.39417	Primary	GBIF: https://doi.org/10.15468/dl.zfaw79
Anopheles darlingi	84.31806	43.39444	Primary	GBIF: https://doi.org/10.15468/dl.zfaw79
Anopheles darlingi	84.31833	43.39472	Primary	GBIF: https://doi.org/10.15468/dl.zfaw79
Anopheles darlingi	84.31861	43.395	Primary	GBIF: https://doi.org/10.15468/dl.zfaw79
Anopheles darlingi	125.038816	12.244553	Primary	GBIF: https://doi.org/10.15468/dl.zfaw79
Anopheles deaneorum	-67.14	-9.7	Unclassified	GBIF: https://doi.org/10.15468/dl.zfaw79
Anopheles deaneorum	-67.14	-9.69	Unclassified	GBIF: https://doi.org/10.15468/dl.zfaw79
Anopheles deaneorum	-67.14	-9.71	Unclassified	GBIF: https://doi.org/10.15468/dl.zfaw79

Anopheles deaneorum	-67.13	-9.69	Unclassified	GBIF: https://doi.org/10.15468/dl.zfaw79
Anopheles deaneorum	-67.13	-9.82	Unclassified	GBIF: https://doi.org/10.15468/dl.zfaw79
Anopheles deaneorum	-67.12	-9.74	Unclassified	GBIF: https://doi.org/10.15468/dl.zfaw79
Anopheles deaneorum	-67.11	-9.7	Unclassified	GBIF: https://doi.org/10.15468/dl.zfaw79
Anopheles deaneorum	-67.11	-9.78	Unclassified	GBIF: https://doi.org/10.15468/dl.zfaw79
Anopheles deaneorum	-67.1	-9.71	Unclassified	GBIF: https://doi.org/10.15468/dl.zfaw79
Anopheles deaneorum	-67.09	-9.71	Unclassified	GBIF: https://doi.org/10.15468/dl.zfaw79
Anopheles deaneorum	-66.91	-10.12	Unclassified	GBIF: https://doi.org/10.15468/dl.zfaw79
Anopheles deaneorum	-66.83	-10	Unclassified	GBIF: https://doi.org/10.15468/dl.zfaw79
Anopheles deaneorum	-65.33	-10.83	Unclassified	GBIF: https://doi.org/10.15468/dl.zfaw79
Anopheles deaneorum	-64.27	-12.47	Unclassified	GBIF: https://doi.org/10.15468/dl.zfaw79
Anopheles deaneorum	-63.07	-9.93	Unclassified	GBIF: https://doi.org/10.15468/dl.zfaw79
Anopheles deaneorum	-59.95	-15	Unclassified	GBIF: https://doi.org/10.15468/dl.zfaw79
Anopheles deaneorum	-59.94	-14.93	Unclassified	GBIF: https://doi.org/10.15468/dl.zfaw79
Anopheles deaneorum	-59.9	-14.98	Unclassified	GBIF: https://doi.org/10.15468/dl.zfaw79
Anopheles deaneorum	-59.87	-14.88	Unclassified	GBIF: https://doi.org/10.15468/dl.zfaw79
Anopheles deaneorum	-58.833	-27.467	Unclassified	GBIF: https://doi.org/10.15468/dl.zfaw79
Anopheles deaneorum	-55.63	-19.51	Unclassified	GBIF: https://doi.org/10.15468/dl.zfaw79
Anopheles deaneorum	-55.62	-19.52	Unclassified	GBIF: https://doi.org/10.15468/dl.zfaw79
Anopheles deaneorum	-55.61	-19.5	Unclassified	GBIF: https://doi.org/10.15468/dl.zfaw79
Anopheles deaneorum	-54.9	-10.38	Unclassified	GBIF: https://doi.org/10.15468/dl.zfaw79
Anopheles deaneorum	-54.25	-24.07	Unclassified	GBIF: https://doi.org/10.15468/dl.zfaw79
Anopheles demeilloni	13.41	-1.64	Unclassified	GBIF: https://doi.org/10.15468/dl.zfaw79
Anopheles demeilloni	30.166667	-23.833333	Unclassified	GBIF: https://doi.org/10.15468/dl.zfaw79
Anopheles demeilloni	31.03	-28.9	Unclassified	GBIF: https://doi.org/10.15468/dl.zfaw79
Anopheles demeilloni	32.032612	-25.987109	Unclassified	GBIF: https://doi.org/10.15468/dl.zfaw79
Anopheles dirus	97.95548	18.37789	Unclassified	GBIF: https://doi.org/10.15468/dl.zfaw79
Anopheles dirus	97.96386	18.55778	Unclassified	GBIF: https://doi.org/10.15468/dl.zfaw79
Anopheles dirus	98.03245	18.15237	Unclassified	GBIF: https://doi.org/10.15468/dl.zfaw79
Anopheles dirus	98.59142	8.57402	Unclassified	GBIF: https://doi.org/10.15468/dl.zfaw79
Anopheles dirus	98.8133	8.855	Unclassified	GBIF: https://doi.org/10.15468/dl.zfaw79
Anopheles dirus	98.82057	14.40306	Unclassified	GBIF: https://doi.org/10.15468/dl.zfaw79
Anopheles dirus	98.82984	14.41211	Unclassified	GBIF: https://doi.org/10.15468/dl.zfaw79
Anopheles dirus	98.87621	14.43022	Unclassified	GBIF: https://doi.org/10.15468/dl.zfaw79
Anopheles dirus	98.87621	14.42118	Unclassified	GBIF: https://doi.org/10.15468/dl.zfaw79
Anopheles dirus	98.89354	16.81698	Unclassified	GBIF: https://doi.org/10.15468/dl.zfaw79
Anopheles dirus	98.9533	9.03	Unclassified	GBIF: https://doi.org/10.15468/dl.zfaw79
Anopheles dirus	98.969	14.31271	Unclassified	GBIF: https://doi.org/10.15468/dl.zfaw79
Anopheles dirus	98.97828	14.27654	Unclassified	GBIF: https://doi.org/10.15468/dl.zfaw79
Anopheles dirus	98.97828	14.28559	Unclassified	GBIF: https://doi.org/10.15468/dl.zfaw79
Anopheles dirus	98.9789	9.23889	Unclassified	GBIF: https://doi.org/10.15468/dl.zfaw79
Anopheles dirus	98.988056	14.335333	Unclassified	GBIF: https://doi.org/10.15468/dl.zfaw79

Anopheles dirus	98.988139	14.335778	Unclassified	GBIF: https://doi.org/10.15468/dl.zfaw79
Anopheles dirus	98.988667	14.335583	Unclassified	GBIF: https://doi.org/10.15468/dl.zfaw79
Anopheles dirus	99.0437	16.79892	Unclassified	GBIF: https://doi.org/10.15468/dl.zfaw79
Anopheles dirus	99.08024	14.22228	Unclassified	GBIF: https://doi.org/10.15468/dl.zfaw79
Anopheles dirus	99.1	14.63	Unclassified	GBIF: https://doi.org/10.15468/dl.zfaw79
Anopheles dirus	99.70029	8.2836	Unclassified	GBIF: https://doi.org/10.15468/dl.zfaw79
Anopheles dirus	99.73481	8.19739	Unclassified	GBIF: https://doi.org/10.15468/dl.zfaw79
Anopheles dirus	99.73925	8.44532	Unclassified	GBIF: https://doi.org/10.15468/dl.zfaw79
Anopheles dirus	99.75597	8.43731	Unclassified	GBIF: https://doi.org/10.15468/dl.zfaw79
Anopheles dirus	99.76886	8.14009	Unclassified	GBIF: https://doi.org/10.15468/dl.zfaw79
Anopheles dirus	99.77257	8.43757	Unclassified	GBIF: https://doi.org/10.15468/dl.zfaw79
Anopheles dirus	99.77269	8.42931	Unclassified	GBIF: https://doi.org/10.15468/dl.zfaw79
Anopheles dirus	99.77397	8.3467	Unclassified	GBIF: https://doi.org/10.15468/dl.zfaw79
Anopheles dirus	99.80425	18.98456	Unclassified	GBIF: https://doi.org/10.15468/dl.zfaw79
Anopheles dirus	99.93681	18.90712	Unclassified	GBIF: https://doi.org/10.15468/dl.zfaw79
Anopheles dirus	99.94543	15.59635	Unclassified	GBIF: https://doi.org/10.15468/dl.zfaw79
Anopheles dirus	100.5788	18.51878	Unclassified	GBIF: https://doi.org/10.15468/dl.zfaw79
Anopheles dirus	100.63537	18.49119	Unclassified	GBIF: https://doi.org/10.15468/dl.zfaw79
Anopheles dirus	100.95415	14.01007	Unclassified	GBIF: https://doi.org/10.15468/dl.zfaw79
Anopheles dirus	101.02188	13.26936	Unclassified	GBIF: https://doi.org/10.15468/dl.zfaw79
Anopheles dirus	101.30242	6.25942	Unclassified	GBIF: https://doi.org/10.15468/dl.zfaw79
Anopheles dirus	101.34375	6.2512	Unclassified	GBIF: https://doi.org/10.15468/dl.zfaw79
Anopheles dirus	101.36853	6.2595	Unclassified	GBIF: https://doi.org/10.15468/dl.zfaw79
Anopheles dirus	101.48209	14.3901	Unclassified	GBIF: https://doi.org/10.15468/dl.zfaw79
Anopheles dirus	101.77356	5.94562	Unclassified	GBIF: https://doi.org/10.15468/dl.zfaw79
Anopheles dirus	101.79008	5.94563	Unclassified	GBIF: https://doi.org/10.15468/dl.zfaw79
Anopheles dirus	101.80659	5.94563	Unclassified	GBIF: https://doi.org/10.15468/dl.zfaw79
Anopheles dirus	101.875835	14.271414	Unclassified	GBIF: https://doi.org/10.15468/dl.zfaw79
Anopheles dirus	102.02336	12.60527	Unclassified	GBIF: https://doi.org/10.15468/dl.zfaw79
Anopheles dirus	102.03245	12.6144	Unclassified	GBIF: https://doi.org/10.15468/dl.zfaw79
Anopheles dirus	102.03256	12.60537	Unclassified	GBIF: https://doi.org/10.15468/dl.zfaw79
Anopheles dirus	102.15868	12.8416	Unclassified	GBIF: https://doi.org/10.15468/dl.zfaw79
Anopheles dirus	102.17138	12.52558	Unclassified	GBIF: https://doi.org/10.15468/dl.zfaw79
Anopheles dirus	102.18498	12.95931	Unclassified	GBIF: https://doi.org/10.15468/dl.zfaw79
Anopheles dirus	102.18967	12.53481	Unclassified	GBIF: https://doi.org/10.15468/dl.zfaw79
Anopheles dirus	102.19915	12.50781	Unclassified	GBIF: https://doi.org/10.15468/dl.zfaw79
Anopheles dirus	102.20335	12.96403	Unclassified	GBIF: https://doi.org/10.15468/dl.zfaw79
Anopheles dirus	102.20375	12.9279	Unclassified	GBIF: https://doi.org/10.15468/dl.zfaw79
Anopheles dirus	102.20825	12.51693	Unclassified	GBIF: https://doi.org/10.15468/dl.zfaw79
Anopheles dirus	102.21261	12.95961	Unclassified	GBIF: https://doi.org/10.15468/dl.zfaw79
Anopheles dirus	102.73	12.15	Unclassified	GBIF: https://doi.org/10.15468/dl.zfaw79
Anopheles dirus	104.8	19.03	Unclassified	GBIF: https://doi.org/10.15468/dl.zfaw79

Anopheles dirus	106	19	Unclassified	GBIF: https://doi.org/10.15468/dl.zfaw79
Anopheles dirus	106.93	11.72	Unclassified	GBIF: https://doi.org/10.15468/dl.zfaw79
Anopheles dirus	106.93	11.71	Unclassified	GBIF: https://doi.org/10.15468/dl.zfaw79
Anopheles dirus	106.95	11.71	Unclassified	GBIF: https://doi.org/10.15468/dl.zfaw79
Anopheles dirus	107.3	11.99	Unclassified	GBIF: https://doi.org/10.15468/dl.zfaw79
Anopheles dirus	107.33	11.5	Unclassified	GBIF: https://doi.org/10.15468/dl.zfaw79
Anopheles dirus	107.9	11.09	Unclassified	GBIF: https://doi.org/10.15468/dl.zfaw79
Anopheles dirus	108.01	16.03	Unclassified	GBIF: https://doi.org/10.15468/dl.zfaw79
Anopheles dirus	108.27	12.49	Unclassified	GBIF: https://doi.org/10.15468/dl.zfaw79
Anopheles dirus	108.53	12.16	Unclassified	GBIF: https://doi.org/10.15468/dl.zfaw79
Anopheles dirus	108.83	13.13	Unclassified	GBIF: https://doi.org/10.15468/dl.zfaw79
Anopheles dirus	109.08	12.37	Unclassified	GBIF: https://doi.org/10.15468/dl.zfaw79
Anopheles distinctus	23.3867	-19.1183	Unclassified	GBIF: https://doi.org/10.15468/dl.zfaw79
Anopheles distinctus	23.8683	-19.1217	Unclassified	GBIF: https://doi.org/10.15468/dl.zfaw79
Anopheles domicolus	4.04921	10.73343	Unclassified	GBIF: https://doi.org/10.15468/dl.zfaw79
Anopheles domicolus	4.18254	12.46661	Unclassified	GBIF: https://doi.org/10.15468/dl.zfaw79
Anopheles domicolus	4.23255	8.13367	Unclassified	GBIF: https://doi.org/10.15468/dl.zfaw79
Anopheles domicolus	4.48256	7.7837	Unclassified	GBIF: https://doi.org/10.15468/dl.zfaw79
Anopheles domicolus	4.58256	8.48364	Unclassified	GBIF: https://doi.org/10.15468/dl.zfaw79
Anopheles domicolus	5.48257	10.3668	Unclassified	GBIF: https://doi.org/10.15468/dl.zfaw79
Anopheles domicolus	6.155834	9.812778	Unclassified	GBIF: https://doi.org/10.15468/dl.zfaw79
Anopheles domicolus	6.16591	9.81685	Unclassified	GBIF: https://doi.org/10.15468/dl.zfaw79
Anopheles domicolus	6.21592	7.56706	Unclassified	GBIF: https://doi.org/10.15468/dl.zfaw79
Anopheles domicolus	6.73259	7.80037	Unclassified	GBIF: https://doi.org/10.15468/dl.zfaw79
Anopheles domicolus	7.44927	10.50013	Unclassified	GBIF: https://doi.org/10.15468/dl.zfaw79
Anopheles domicolus	7.4826	10.63345	Unclassified	GBIF: https://doi.org/10.15468/dl.zfaw79
Anopheles domicolus	7.86594	8.83361	Unclassified	GBIF: https://doi.org/10.15468/dl.zfaw79
Anopheles domicolus	8.89929	9.86685	Unclassified	GBIF: https://doi.org/10.15468/dl.zfaw79
Anopheles domicolus	9.74931	8.18367	Unclassified	GBIF: https://doi.org/10.15468/dl.zfaw79
Anopheles domicolus	12.04935	9.4669	Unclassified	GBIF: https://doi.org/10.15468/dl.zfaw79
Anopheles dominicanus	-70.75	19.583332	Unclassified	GBIF: https://doi.org/10.15468/dl.zfaw79
Anopheles donaldi	98.8133	8.855	Unclassified	GBIF: https://doi.org/10.15468/dl.zfaw79
Anopheles donaldi	98.9533	9.03	Unclassified	GBIF: https://doi.org/10.15468/dl.zfaw79
Anopheles donaldi	98.9789	9.23889	Unclassified	GBIF: https://doi.org/10.15468/dl.zfaw79
Anopheles donaldi	101.541033	14.491603	Unclassified	GBIF: https://doi.org/10.15468/dl.zfaw79
Anopheles donaldi	101.683613	16.171731	Unclassified	GBIF: https://doi.org/10.15468/dl.zfaw79
Anopheles donaldi	101.794338	13.49568	Unclassified	GBIF: https://doi.org/10.15468/dl.zfaw79
Anopheles donaldi	101.891961	14.260731	Unclassified	GBIF: https://doi.org/10.15468/dl.zfaw79
Anopheles donaldi	101.907779	13.227123	Unclassified	GBIF: https://doi.org/10.15468/dl.zfaw79
Anopheles donaldi	102.293068	12.053376	Unclassified	GBIF: https://doi.org/10.15468/dl.zfaw79
Anopheles donaldi	102.71928	14.24297	Unclassified	GBIF: https://doi.org/10.15468/dl.zfaw79
Anopheles donaldi	102.967142	14.369102	Unclassified	GBIF: https://doi.org/10.15468/dl.zfaw79

Anopheles donaldi	116.79	6.77	Unclassified	GBIF: https://doi.org/10.15468/dl.zfaw79
Anopheles dravidicus	73.41	33.13	Unclassified	GBIF: https://doi.org/10.15468/dl.zfaw79
Anopheles dravidicus	98.252256	18.684351	Unclassified	GBIF: https://doi.org/10.15468/dl.zfaw79
Anopheles dravidicus	98.41762	18.406039	Unclassified	GBIF: https://doi.org/10.15468/dl.zfaw79
Anopheles dravidicus	98.590972	18.521056	Unclassified	GBIF: https://doi.org/10.15468/dl.zfaw79
Anopheles dravidicus	98.685111	18.531528	Unclassified	GBIF: https://doi.org/10.15468/dl.zfaw79
Anopheles dravidicus	102.679598	12.153556	Unclassified	GBIF: https://doi.org/10.15468/dl.zfaw79
Anopheles dunhami	-72.69	-7.63	Unclassified	GBIF: https://doi.org/10.15468/dl.zfaw79
Anopheles dunhami	-64.73	-7.4	Unclassified	GBIF: https://doi.org/10.15468/dl.zfaw79
Anopheles dunhami	-64.70101	-3.36675	Unclassified	GBIF: https://doi.org/10.15468/dl.zfaw79
Anopheles dunhami	-64.700001	-3.366666	Unclassified	GBIF: https://doi.org/10.15468/dl.zfaw79
Anopheles dunhami	-64.66	-7.38	Unclassified	GBIF: https://doi.org/10.15468/dl.zfaw79
Anopheles dunhami	-56.74	-2.63	Unclassified	GBIF: https://doi.org/10.15468/dl.zfaw79
Anopheles dunhami	-56.64	-2.65	Unclassified	GBIF: https://doi.org/10.15468/dl.zfaw79
Anopheles dunhami	-56.5	-20.2	Unclassified	GBIF: https://doi.org/10.15468/dl.zfaw79
Anopheles dureni	14.06	-2.14	Unclassified	GBIF: https://doi.org/10.15468/dl.zfaw79
Anopheles dureni	14.06135	-2.13735	Unclassified	GBIF: https://doi.org/10.15468/dl.zfaw79
Anopheles earlei	-150.305159	60.384539	Unclassified	GBIF: https://doi.org/10.15468/dl.zfaw79
Anopheles earlei	-149.99116	61.76934	Unclassified	GBIF: https://doi.org/10.15468/dl.zfaw79
Anopheles earlei	-149.586567	68.636291	Unclassified	GBIF: https://doi.org/10.15468/dl.zfaw79
Anopheles earlei	-147.8477	64.8584	Unclassified	GBIF: https://doi.org/10.15468/dl.zfaw79
Anopheles earlei	-147.80276	64.85695	Unclassified	GBIF: https://doi.org/10.15468/dl.zfaw79
Anopheles earlei	-147.7486	64.874	Unclassified	GBIF: https://doi.org/10.15468/dl.zfaw79
Anopheles earlei	-147.54175	64.79724	Unclassified	GBIF: https://doi.org/10.15468/dl.zfaw79
Anopheles earlei	-147.527868	64.901099	Unclassified	GBIF: https://doi.org/10.15468/dl.zfaw79
Anopheles earlei	-147.516165	65.1542	Unclassified	GBIF: https://doi.org/10.15468/dl.zfaw79
Anopheles earlei	-147.516	65.154	Unclassified	GBIF: https://doi.org/10.15468/dl.zfaw79
Anopheles earlei	-147.515	65.162	Unclassified	GBIF: https://doi.org/10.15468/dl.zfaw79
Anopheles earlei	-147.514873	65.162259	Unclassified	GBIF: https://doi.org/10.15468/dl.zfaw79
Anopheles earlei	-147.505385	65.159013	Unclassified	GBIF: https://doi.org/10.15468/dl.zfaw79
Anopheles earlei	-147.505	65.159	Unclassified	GBIF: https://doi.org/10.15468/dl.zfaw79
Anopheles earlei	-147.50258	65.15401	Unclassified	GBIF: https://doi.org/10.15468/dl.zfaw79
Anopheles earlei	-147.495	65.166	Unclassified	GBIF: https://doi.org/10.15468/dl.zfaw79
Anopheles earlei	-147.494593	65.165769	Unclassified	GBIF: https://doi.org/10.15468/dl.zfaw79
Anopheles earlei	-147.491	65.18	Unclassified	GBIF: https://doi.org/10.15468/dl.zfaw79
Anopheles earlei	-147.490992	65.179794	Unclassified	GBIF: https://doi.org/10.15468/dl.zfaw79
Anopheles earlei	-147.489	65.161	Unclassified	GBIF: https://doi.org/10.15468/dl.zfaw79
Anopheles earlei	-147.488764	65.160949	Unclassified	GBIF: https://doi.org/10.15468/dl.zfaw79
Anopheles earlei	-147.39993	64.76771	Unclassified	GBIF: https://doi.org/10.15468/dl.zfaw79
Anopheles earlei	-145.778116	63.887112	Unclassified	GBIF: https://doi.org/10.15468/dl.zfaw79
Anopheles earlei	-145.778	63.887	Unclassified	GBIF: https://doi.org/10.15468/dl.zfaw79
Anopheles earlei	-145.4632	62.77262	Unclassified	GBIF: https://doi.org/10.15468/dl.zfaw79

Anopheles earlei	-144.083333	65.833333	Unclassified	GBIF: https://doi.org/10.15468/dl.zfaw79
Anopheles earlei	-138.4922	60.9899	Unclassified	GBIF: https://doi.org/10.15468/dl.zfaw79
Anopheles earlei	-135.332	60.878	Unclassified	GBIF: https://doi.org/10.15468/dl.zfaw79
Anopheles earlei	-135.187	60.788	Unclassified	GBIF: https://doi.org/10.15468/dl.zfaw79
Anopheles earlei	-133.60169	60.44564	Unclassified	GBIF: https://doi.org/10.15468/dl.zfaw79
Anopheles earlei	-133.55127	60.44177	Unclassified	GBIF: https://doi.org/10.15468/dl.zfaw79
Anopheles earlei	-126.10154	59.42591	Unclassified	GBIF: https://doi.org/10.15468/dl.zfaw79
Anopheles earlei	-122.314	49.144	Unclassified	GBIF: https://doi.org/10.15468/dl.zfaw79
Anopheles earlei	-122.149	51.988	Unclassified	GBIF: https://doi.org/10.15468/dl.zfaw79
Anopheles earlei	-120.671	50.164	Unclassified	GBIF: https://doi.org/10.15468/dl.zfaw79
Anopheles earlei	-120.452	50.796	Unclassified	GBIF: https://doi.org/10.15468/dl.zfaw79
Anopheles earlei	-119.55	49.182	Unclassified	GBIF: https://doi.org/10.15468/dl.zfaw79
Anopheles earlei	-116.041	50.892	Unclassified	GBIF: https://doi.org/10.15468/dl.zfaw79
Anopheles earlei	-116.04	50.89	Unclassified	GBIF: https://doi.org/10.15468/dl.zfaw79
Anopheles earlei	-115.732	48.8267	Unclassified	GBIF: https://doi.org/10.15468/dl.zfaw79
Anopheles earlei	-115.407	47.6509	Unclassified	GBIF: https://doi.org/10.15468/dl.zfaw79
Anopheles earlei	-115.053	48.8772	Unclassified	GBIF: https://doi.org/10.15468/dl.zfaw79
Anopheles earlei	-114.361	48.2046	Unclassified	GBIF: https://doi.org/10.15468/dl.zfaw79
Anopheles earlei	-114.341	48.3343	Unclassified	GBIF: https://doi.org/10.15468/dl.zfaw79
Anopheles earlei	-114.307	48.2088	Unclassified	GBIF: https://doi.org/10.15468/dl.zfaw79
Anopheles earlei	-114.301	48.2152	Unclassified	GBIF: https://doi.org/10.15468/dl.zfaw79
Anopheles earlei	-114.293	48.1949	Unclassified	GBIF: https://doi.org/10.15468/dl.zfaw79
Anopheles earlei	-114.291	48.2177	Unclassified	GBIF: https://doi.org/10.15468/dl.zfaw79
Anopheles earlei	-114.277	48.1727	Unclassified	GBIF: https://doi.org/10.15468/dl.zfaw79
Anopheles earlei	-114.275	48.2206	Unclassified	GBIF: https://doi.org/10.15468/dl.zfaw79
Anopheles earlei	-114.274	48.2209	Unclassified	GBIF: https://doi.org/10.15468/dl.zfaw79
Anopheles earlei	-114.267	48.213	Unclassified	GBIF: https://doi.org/10.15468/dl.zfaw79
Anopheles earlei	-114.266	48.2862	Unclassified	GBIF: https://doi.org/10.15468/dl.zfaw79
Anopheles earlei	-114.262	48.1593	Unclassified	GBIF: https://doi.org/10.15468/dl.zfaw79
Anopheles earlei	-114.26	48.204	Unclassified	GBIF: https://doi.org/10.15468/dl.zfaw79
Anopheles earlei	-114.258	48.1171	Unclassified	GBIF: https://doi.org/10.15468/dl.zfaw79
Anopheles earlei	-114.244	48.1337	Unclassified	GBIF: https://doi.org/10.15468/dl.zfaw79
Anopheles earlei	-114.232	48.3021	Unclassified	GBIF: https://doi.org/10.15468/dl.zfaw79
Anopheles earlei	-114.229	48.0796	Unclassified	GBIF: https://doi.org/10.15468/dl.zfaw79
Anopheles earlei	-114.169	46.0239	Unclassified	GBIF: https://doi.org/10.15468/dl.zfaw79
Anopheles earlei	-114.154	46.9143	Unclassified	GBIF: https://doi.org/10.15468/dl.zfaw79
Anopheles earlei	-114.148	47.4504	Unclassified	GBIF: https://doi.org/10.15468/dl.zfaw79
Anopheles earlei	-114.144	47.4321	Unclassified	GBIF: https://doi.org/10.15468/dl.zfaw79
Anopheles earlei	-114.129	47.5638	Unclassified	GBIF: https://doi.org/10.15468/dl.zfaw79
Anopheles earlei	-114.128	47.5646	Unclassified	GBIF: https://doi.org/10.15468/dl.zfaw79
Anopheles earlei	-114.105	46.5196	Unclassified	GBIF: https://doi.org/10.15468/dl.zfaw79
Anopheles earlei	-114.098	47.4459	Unclassified	GBIF: https://doi.org/10.15468/dl.zfaw79

Anopheles earlei	-114.097	48.0714	Unclassified	GBIF: https://doi.org/10.15468/dl.zfaw79
Anopheles earlei	-114.071	46.7755	Unclassified	GBIF: https://doi.org/10.15468/dl.zfaw79
Anopheles earlei	-114.071	46.5856	Unclassified	GBIF: https://doi.org/10.15468/dl.zfaw79
Anopheles earlei	-114.062	46.8402	Unclassified	GBIF: https://doi.org/10.15468/dl.zfaw79
Anopheles earlei	-114.059	46.6386	Unclassified	GBIF: https://doi.org/10.15468/dl.zfaw79
Anopheles earlei	-114.04505	46.69289	Unclassified	GBIF: https://doi.org/10.15468/dl.zfaw79
Anopheles earlei	-114.025214	50.970055	Unclassified	GBIF: https://doi.org/10.15468/dl.zfaw79
Anopheles earlei	-114.02153	50.959392	Unclassified	GBIF: https://doi.org/10.15468/dl.zfaw79
Anopheles earlei	-114.021469	50.959397	Unclassified	GBIF: https://doi.org/10.15468/dl.zfaw79
Anopheles earlei	-113.821	47.7479	Unclassified	GBIF: https://doi.org/10.15468/dl.zfaw79
Anopheles earlei	-113.601	46.7232	Unclassified	GBIF: https://doi.org/10.15468/dl.zfaw79
Anopheles earlei	-113.431	46.701	Unclassified	GBIF: https://doi.org/10.15468/dl.zfaw79
Anopheles earlei	-113.069	45.8506	Unclassified	GBIF: https://doi.org/10.15468/dl.zfaw79
Anopheles earlei	-113.014	46.9039	Unclassified	GBIF: https://doi.org/10.15468/dl.zfaw79
Anopheles earlei	-113.012	46.9108	Unclassified	GBIF: https://doi.org/10.15468/dl.zfaw79
Anopheles earlei	-113.01	46.9163	Unclassified	GBIF: https://doi.org/10.15468/dl.zfaw79
Anopheles earlei	-112.975	46.9678	Unclassified	GBIF: https://doi.org/10.15468/dl.zfaw79
Anopheles earlei	-112.972	46.965	Unclassified	GBIF: https://doi.org/10.15468/dl.zfaw79
Anopheles earlei	-112.945	46.1751	Unclassified	GBIF: https://doi.org/10.15468/dl.zfaw79
Anopheles earlei	-112.931	46.591	Unclassified	GBIF: https://doi.org/10.15468/dl.zfaw79
Anopheles earlei	-112.794	46.5192	Unclassified	GBIF: https://doi.org/10.15468/dl.zfaw79
Anopheles earlei	-112.79	46.1843	Unclassified	GBIF: https://doi.org/10.15468/dl.zfaw79
Anopheles earlei	-112.741	46.4984	Unclassified	GBIF: https://doi.org/10.15468/dl.zfaw79
Anopheles earlei	-112.74	45.1312	Unclassified	GBIF: https://doi.org/10.15468/dl.zfaw79
Anopheles earlei	-112.737	46.317	Unclassified	GBIF: https://doi.org/10.15468/dl.zfaw79
Anopheles earlei	-112.673	45.2117	Unclassified	GBIF: https://doi.org/10.15468/dl.zfaw79
Anopheles earlei	-112.666	45.4666	Unclassified	GBIF: https://doi.org/10.15468/dl.zfaw79
Anopheles earlei	-112.616	46.5979	Unclassified	GBIF: https://doi.org/10.15468/dl.zfaw79
Anopheles earlei	-112.535	45.9919	Unclassified	GBIF: https://doi.org/10.15468/dl.zfaw79
Anopheles earlei	-112.41	46.5565	Unclassified	GBIF: https://doi.org/10.15468/dl.zfaw79
Anopheles earlei	-112.319	45.6332	Unclassified	GBIF: https://doi.org/10.15468/dl.zfaw79
Anopheles earlei	-112.118	46.2226	Unclassified	GBIF: https://doi.org/10.15468/dl.zfaw79
Anopheles earlei	-111.978	46.6609	Unclassified	GBIF: https://doi.org/10.15468/dl.zfaw79
Anopheles earlei	-111.946	47.1097	Unclassified	GBIF: https://doi.org/10.15468/dl.zfaw79
Anopheles earlei	-111.875	46.6446	Unclassified	GBIF: https://doi.org/10.15468/dl.zfaw79
Anopheles earlei	-111.871	46.6493	Unclassified	GBIF: https://doi.org/10.15468/dl.zfaw79
Anopheles earlei	-111.586	45.8888	Unclassified	GBIF: https://doi.org/10.15468/dl.zfaw79
Anopheles earlei	-111.572	46.4132	Unclassified	GBIF: https://doi.org/10.15468/dl.zfaw79
Anopheles earlei	-111.562	46.1659	Unclassified	GBIF: https://doi.org/10.15468/dl.zfaw79
Anopheles earlei	-111.562	46.3812	Unclassified	GBIF: https://doi.org/10.15468/dl.zfaw79
Anopheles earlei	-111.56	46.3833	Unclassified	GBIF: https://doi.org/10.15468/dl.zfaw79
Anopheles earlei	-111.534	45.8922	Unclassified	GBIF: https://doi.org/10.15468/dl.zfaw79

Anopheles earlei	-111.504	46.365	Unclassified	GBIF: https://doi.org/10.15468/dl.zfaw79
Anopheles earlei	-111.497	45.9032	Unclassified	GBIF: https://doi.org/10.15468/dl.zfaw79
Anopheles earlei	-111.445	46.1721	Unclassified	GBIF: https://doi.org/10.15468/dl.zfaw79
Anopheles earlei	-111.419	46.032	Unclassified	GBIF: https://doi.org/10.15468/dl.zfaw79
Anopheles earlei	-111.09	48.3065	Unclassified	GBIF: https://doi.org/10.15468/dl.zfaw79
Anopheles earlei	-110.904	46.4378	Unclassified	GBIF: https://doi.org/10.15468/dl.zfaw79
Anopheles earlei	-110.32	46.4609	Unclassified	GBIF: https://doi.org/10.15468/dl.zfaw79
Anopheles earlei	-110.229	46.4669	Unclassified	GBIF: https://doi.org/10.15468/dl.zfaw79
Anopheles earlei	-109.971	45.8375	Unclassified	GBIF: https://doi.org/10.15468/dl.zfaw79
Anopheles earlei	-109.350332	38.277972	Unclassified	GBIF: https://doi.org/10.15468/dl.zfaw79
Anopheles earlei	-109.251	45.6284	Unclassified	GBIF: https://doi.org/10.15468/dl.zfaw79
Anopheles earlei	-109.15	52.44	Unclassified	GBIF: https://doi.org/10.15468/dl.zfaw79
Anopheles earlei	-108.95108	59.11707	Unclassified	GBIF: https://doi.org/10.15468/dl.zfaw79
Anopheles earlei	-108.767	45.6815	Unclassified	GBIF: https://doi.org/10.15468/dl.zfaw79
Anopheles earlei	-108.684	48.4909	Unclassified	GBIF: https://doi.org/10.15468/dl.zfaw79
Anopheles earlei	-108.62189	59.15806	Unclassified	GBIF: https://doi.org/10.15468/dl.zfaw79
Anopheles earlei	-108.525	45.7464	Unclassified	GBIF: https://doi.org/10.15468/dl.zfaw79
Anopheles earlei	-108.434	45.8348	Unclassified	GBIF: https://doi.org/10.15468/dl.zfaw79
Anopheles earlei	-108.266	45.7908	Unclassified	GBIF: https://doi.org/10.15468/dl.zfaw79
Anopheles earlei	-107.866	45.3349	Unclassified	GBIF: https://doi.org/10.15468/dl.zfaw79
Anopheles earlei	-107.729	48.3871	Unclassified	GBIF: https://doi.org/10.15468/dl.zfaw79
Anopheles earlei	-107.716	46.072	Unclassified	GBIF: https://doi.org/10.15468/dl.zfaw79
Anopheles earlei	-107.66	48.4177	Unclassified	GBIF: https://doi.org/10.15468/dl.zfaw79
Anopheles earlei	-107.62	45.7363	Unclassified	GBIF: https://doi.org/10.15468/dl.zfaw79
Anopheles earlei	-107.571	45.7567	Unclassified	GBIF: https://doi.org/10.15468/dl.zfaw79
Anopheles earlei	-107.466	46.1447	Unclassified	GBIF: https://doi.org/10.15468/dl.zfaw79
Anopheles earlei	-107.453	45.6032	Unclassified	GBIF: https://doi.org/10.15468/dl.zfaw79
Anopheles earlei	-107.416	45.119	Unclassified	GBIF: https://doi.org/10.15468/dl.zfaw79
Anopheles earlei	-107.363	45.3271	Unclassified	GBIF: https://doi.org/10.15468/dl.zfaw79
Anopheles earlei	-107.118	47.9571	Unclassified	GBIF: https://doi.org/10.15468/dl.zfaw79
Anopheles earlei	-107.009	48.0432	Unclassified	GBIF: https://doi.org/10.15468/dl.zfaw79
Anopheles earlei	-106.893	48.1728	Unclassified	GBIF: https://doi.org/10.15468/dl.zfaw79
Anopheles earlei	-106.733433	52.131677	Unclassified	GBIF: https://doi.org/10.15468/dl.zfaw79
Anopheles earlei	-106.647	48.1765	Unclassified	GBIF: https://doi.org/10.15468/dl.zfaw79
Anopheles earlei	-105.421	48.8531	Unclassified	GBIF: https://doi.org/10.15468/dl.zfaw79
Anopheles earlei	-105.418	48.1302	Unclassified	GBIF: https://doi.org/10.15468/dl.zfaw79
Anopheles earlei	-105.334	45.0963	Unclassified	GBIF: https://doi.org/10.15468/dl.zfaw79
Anopheles earlei	-105.324	46.7988	Unclassified	GBIF: https://doi.org/10.15468/dl.zfaw79
Anopheles earlei	-104.583	48.3678	Unclassified	GBIF: https://doi.org/10.15468/dl.zfaw79
Anopheles earlei	-104.533	48.1015	Unclassified	GBIF: https://doi.org/10.15468/dl.zfaw79
Anopheles earlei	-104.45	48.4825	Unclassified	GBIF: https://doi.org/10.15468/dl.zfaw79
Anopheles earlei	-104.359	48.4736	Unclassified	GBIF: https://doi.org/10.15468/dl.zfaw79

Anopheles earlei	-104.312	47.4646	Unclassified	GBIF: https://doi.org/10.15468/dl.zfaw79
Anopheles earlei	-104.2976	51.37152	Unclassified	GBIF: https://doi.org/10.15468/dl.zfaw79
Anopheles earlei	-104.189	47.7126	Unclassified	GBIF: https://doi.org/10.15468/dl.zfaw79
Anopheles earlei	-104.187	46.9864	Unclassified	GBIF: https://doi.org/10.15468/dl.zfaw79
Anopheles earlei	-103.79034	50.76567	Unclassified	GBIF: https://doi.org/10.15468/dl.zfaw79
Anopheles earlei	-103.256096	42.781199	Unclassified	GBIF: https://doi.org/10.15468/dl.zfaw79
Anopheles earlei	-102.433	51.217	Unclassified	GBIF: https://doi.org/10.15468/dl.zfaw79
Anopheles earlei	-102.43	51.22	Unclassified	GBIF: https://doi.org/10.15468/dl.zfaw79
Anopheles earlei	-102.43	51.21	Unclassified	GBIF: https://doi.org/10.15468/dl.zfaw79
Anopheles earlei	-100.909167	46.794829	Unclassified	GBIF: https://doi.org/10.15468/dl.zfaw79
Anopheles earlei	-99.602	49.709	Unclassified	GBIF: https://doi.org/10.15468/dl.zfaw79
Anopheles earlei	-99.60167	49.70833	Unclassified	GBIF: https://doi.org/10.15468/dl.zfaw79
Anopheles earlei	-99.05911	47.119532	Unclassified	GBIF: https://doi.org/10.15468/dl.zfaw79
Anopheles earlei	-99.059	47.12	Unclassified	GBIF: https://doi.org/10.15468/dl.zfaw79
Anopheles earlei	-97.950143	50.380807	Unclassified	GBIF: https://doi.org/10.15468/dl.zfaw79
Anopheles earlei	-97.950108	50.380882	Unclassified	GBIF: https://doi.org/10.15468/dl.zfaw79
Anopheles earlei	-97.166667	49.883333	Unclassified	GBIF: https://doi.org/10.15468/dl.zfaw79
Anopheles earlei	-97.123278	49.87944	Unclassified	GBIF: https://doi.org/10.15468/dl.zfaw79
Anopheles earlei	-95.941927	37.091735	Unclassified	GBIF: https://doi.org/10.15468/dl.zfaw79
Anopheles earlei	-95.12	43.43	Unclassified	GBIF: https://doi.org/10.15468/dl.zfaw79
Anopheles earlei	-94.169695	30.254983	Unclassified	GBIF: https://doi.org/10.15468/dl.zfaw79
Anopheles earlei	-93.58	41.54	Unclassified	GBIF: https://doi.org/10.15468/dl.zfaw79
Anopheles earlei	-93.177	46.828	Unclassified	GBIF: https://doi.org/10.15468/dl.zfaw79
Anopheles earlei	-93.177	46.836	Unclassified	GBIF: https://doi.org/10.15468/dl.zfaw79
Anopheles earlei	-93.173	46.906	Unclassified	GBIF: https://doi.org/10.15468/dl.zfaw79
Anopheles earlei	-92.953251	49.719674	Unclassified	GBIF: https://doi.org/10.15468/dl.zfaw79
Anopheles earlei	-92.727064	47.146121	Unclassified	GBIF: https://doi.org/10.15468/dl.zfaw79
Anopheles earlei	-92.727062	47.146126	Unclassified	GBIF: https://doi.org/10.15468/dl.zfaw79
Anopheles earlei	-92.6	45.6	Unclassified	GBIF: https://doi.org/10.15468/dl.zfaw79
Anopheles earlei	-92.57	45.6	Unclassified	GBIF: https://doi.org/10.15468/dl.zfaw79
Anopheles earlei	-91.85	45.86	Unclassified	GBIF: https://doi.org/10.15468/dl.zfaw79
Anopheles earlei	-91.33	45.4	Unclassified	GBIF: https://doi.org/10.15468/dl.zfaw79
Anopheles earlei	-91.31	45.41	Unclassified	GBIF: https://doi.org/10.15468/dl.zfaw79
Anopheles earlei	-91.25	45.52	Unclassified	GBIF: https://doi.org/10.15468/dl.zfaw79
Anopheles earlei	-91.24	45.51	Unclassified	GBIF: https://doi.org/10.15468/dl.zfaw79
Anopheles earlei	-91.2	45.4	Unclassified	GBIF: https://doi.org/10.15468/dl.zfaw79
Anopheles earlei	-91.2	43.7	Unclassified	GBIF: https://doi.org/10.15468/dl.zfaw79
Anopheles earlei	-91.1	45	Unclassified	GBIF: https://doi.org/10.15468/dl.zfaw79
Anopheles earlei	-90.1	44.3	Unclassified	GBIF: https://doi.org/10.15468/dl.zfaw79
Anopheles earlei	-90.060639	45.804898	Unclassified	GBIF: https://doi.org/10.15468/dl.zfaw79
Anopheles earlei	-90.049011	45.805547	Unclassified	GBIF: https://doi.org/10.15468/dl.zfaw79
Anopheles earlei	-90.049	45.806	Unclassified	GBIF: https://doi.org/10.15468/dl.zfaw79

Anopheles earlei	-90.046	45.785	Unclassified	GBIF: https://doi.org/10.15468/dl.zfaw79
Anopheles earlei	-90.04	46.2	Unclassified	GBIF: https://doi.org/10.15468/dl.zfaw79
Anopheles earlei	-90	46.1	Unclassified	GBIF: https://doi.org/10.15468/dl.zfaw79
Anopheles earlei	-89.563	45.496	Unclassified	GBIF: https://doi.org/10.15468/dl.zfaw79
Anopheles earlei	-89.537254	46.23391	Unclassified	GBIF: https://doi.org/10.15468/dl.zfaw79
Anopheles earlei	-89.527305	46.259156	Unclassified	GBIF: https://doi.org/10.15468/dl.zfaw79
Anopheles earlei	-89.509	46.243	Unclassified	GBIF: https://doi.org/10.15468/dl.zfaw79
Anopheles earlei	-89.493	46.21	Unclassified	GBIF: https://doi.org/10.15468/dl.zfaw79
Anopheles earlei	-89.492758	46.209603	Unclassified	GBIF: https://doi.org/10.15468/dl.zfaw79
Anopheles earlei	-89.4	46	Unclassified	GBIF: https://doi.org/10.15468/dl.zfaw79
Anopheles earlei	-89.3333	46.6667	Unclassified	GBIF: https://doi.org/10.15468/dl.zfaw79
Anopheles earlei	-89.2	45.9	Unclassified	GBIF: https://doi.org/10.15468/dl.zfaw79
Anopheles earlei	-88.886799	46.174498	Unclassified	GBIF: https://doi.org/10.15468/dl.zfaw79
Anopheles earlei	-88.833412	48.00044	Unclassified	GBIF: https://doi.org/10.15468/dl.zfaw79
Anopheles earlei	-88.776657	43.022236	Unclassified	GBIF: https://doi.org/10.15468/dl.zfaw79
Anopheles earlei	-88.6	43.5	Unclassified	GBIF: https://doi.org/10.15468/dl.zfaw79
Anopheles earlei	-88.4	45.81	Unclassified	GBIF: https://doi.org/10.15468/dl.zfaw79
Anopheles earlei	-88.2	44.6	Unclassified	GBIF: https://doi.org/10.15468/dl.zfaw79
Anopheles earlei	-88.14	45.91	Unclassified	GBIF: https://doi.org/10.15468/dl.zfaw79
Anopheles earlei	-87.8939	41.8238	Unclassified	GBIF: https://doi.org/10.15468/dl.zfaw79
Anopheles earlei	-87.888442	47.468794	Unclassified	GBIF: https://doi.org/10.15468/dl.zfaw79
Anopheles earlei	-87.8846	41.8246	Unclassified	GBIF: https://doi.org/10.15468/dl.zfaw79
Anopheles earlei	-87.817	41.8619	Unclassified	GBIF: https://doi.org/10.15468/dl.zfaw79
Anopheles earlei	-87.8144	41.8326	Unclassified	GBIF: https://doi.org/10.15468/dl.zfaw79
Anopheles earlei	-87.51	44.21	Unclassified	GBIF: https://doi.org/10.15468/dl.zfaw79
Anopheles earlei	-86.782679	40.794568	Unclassified	GBIF: https://doi.org/10.15468/dl.zfaw79
Anopheles earlei	-86.3172	39.739	Unclassified	GBIF: https://doi.org/10.15468/dl.zfaw79
Anopheles earlei	-86.2167	43.95	Unclassified	GBIF: https://doi.org/10.15468/dl.zfaw79
Anopheles earlei	-86.1991	39.6394	Unclassified	GBIF: https://doi.org/10.15468/dl.zfaw79
Anopheles earlei	-86.196	39.7309	Unclassified	GBIF: https://doi.org/10.15468/dl.zfaw79
Anopheles earlei	-86.144	39.6996	Unclassified	GBIF: https://doi.org/10.15468/dl.zfaw79
Anopheles earlei	-86.0697	39.81	Unclassified	GBIF: https://doi.org/10.15468/dl.zfaw79
Anopheles earlei	-85.9932	39.6672	Unclassified	GBIF: https://doi.org/10.15468/dl.zfaw79
Anopheles earlei	-85.9596	39.6867	Unclassified	GBIF: https://doi.org/10.15468/dl.zfaw79
Anopheles earlei	-85.215046	44.335287	Unclassified	GBIF: https://doi.org/10.15468/dl.zfaw79
Anopheles earlei	-84.4998	49.2501	Unclassified	GBIF: https://doi.org/10.15468/dl.zfaw79
Anopheles earlei	-84.47448	45.646956	Unclassified	GBIF: https://doi.org/10.15468/dl.zfaw79
Anopheles earlei	-84.247212	43.615582	Unclassified	GBIF: https://doi.org/10.15468/dl.zfaw79
Anopheles earlei	-83.466071	43.65363	Unclassified	GBIF: https://doi.org/10.15468/dl.zfaw79
Anopheles earlei	-81.697608	49.243355	Unclassified	GBIF: https://doi.org/10.15468/dl.zfaw79
Anopheles earlei	-81.64083	45.24967	Unclassified	GBIF: https://doi.org/10.15468/dl.zfaw79
Anopheles earlei	-81.5192	45.23492	Unclassified	GBIF: https://doi.org/10.15468/dl.zfaw79

Anopheles earlei	-81.370454	45.054743	Unclassified	GBIF: https://doi.org/10.15468/dl.zfaw79
Anopheles earlei	-81.289826	44.813618	Unclassified	GBIF: https://doi.org/10.15468/dl.zfaw79
Anopheles earlei	-81.1875	44.95667	Unclassified	GBIF: https://doi.org/10.15468/dl.zfaw79
Anopheles earlei	-76.53	42.4	Unclassified	GBIF: https://doi.org/10.15468/dl.zfaw79
Anopheles earlei	-76.467	44.3	Unclassified	GBIF: https://doi.org/10.15468/dl.zfaw79
Anopheles earlei	-76.1	45.667	Unclassified	GBIF: https://doi.org/10.15468/dl.zfaw79
Anopheles earlei	-76.017	45.417	Unclassified	GBIF: https://doi.org/10.15468/dl.zfaw79
Anopheles earlei	-75.696	45.42	Unclassified	GBIF: https://doi.org/10.15468/dl.zfaw79
Anopheles earlei	-73.58389	45.047	Unclassified	GBIF: https://doi.org/10.15468/dl.zfaw79
Anopheles earlei	-72.289535	43.702293	Unclassified	GBIF: https://doi.org/10.15468/dl.zfaw79
Anopheles earlei	-71.495918	44.894492	Unclassified	GBIF: https://doi.org/10.15468/dl.zfaw79
Anopheles earlei	-71.310978	44.869969	Unclassified	GBIF: https://doi.org/10.15468/dl.zfaw79
Anopheles earlei	-71.074227	43.033419	Unclassified	GBIF: https://doi.org/10.15468/dl.zfaw79
Anopheles earlei	-71.01145	43.123265	Unclassified	GBIF: https://doi.org/10.15468/dl.zfaw79
Anopheles earlei	-68.167	49.217	Unclassified	GBIF: https://doi.org/10.15468/dl.zfaw79
Anopheles earlei	-67.31567	47.68137	Unclassified	GBIF: https://doi.org/10.15468/dl.zfaw79
Anopheles earlei	-66.73517	45.82647	Unclassified	GBIF: https://doi.org/10.15468/dl.zfaw79
Anopheles earlei	-66.57223	45.75747	Unclassified	GBIF: https://doi.org/10.15468/dl.zfaw79
Anopheles earlei	-66.48	45.77	Unclassified	GBIF: https://doi.org/10.15468/dl.zfaw79
Anopheles earlei	-66.2	45.3	Unclassified	GBIF: https://doi.org/10.15468/dl.zfaw79
Anopheles earlei	-66.12352	45.82158	Unclassified	GBIF: https://doi.org/10.15468/dl.zfaw79
Anopheles earlei	-65.82728	45.521389	Unclassified	GBIF: https://doi.org/10.15468/dl.zfaw79
Anopheles earlei	-65.65	47.6	Unclassified	GBIF: https://doi.org/10.15468/dl.zfaw79
Anopheles earlei	-64.78	46.1	Unclassified	GBIF: https://doi.org/10.15468/dl.zfaw79
Anopheles earlei	-64.33	46.03	Unclassified	GBIF: https://doi.org/10.15468/dl.zfaw79
Anopheles eiseni	-104.756389	23.081667	Unclassified	GBIF: https://doi.org/10.15468/dl.zfaw79
Anopheles eiseni	-102.061389	19.426111	Unclassified	GBIF: https://doi.org/10.15468/dl.zfaw79
Anopheles eiseni	-100.247778	19.155556	Unclassified	GBIF: https://doi.org/10.15468/dl.zfaw79
Anopheles eiseni	-100.14	19.084722	Unclassified	GBIF: https://doi.org/10.15468/dl.zfaw79
Anopheles eiseni	-100.112778	19.081667	Unclassified	GBIF: https://doi.org/10.15468/dl.zfaw79
Anopheles eiseni	-99.911944	18.634722	Unclassified	GBIF: https://doi.org/10.15468/dl.zfaw79
Anopheles eiseni	-99.421389	17.361111	Unclassified	GBIF: https://doi.org/10.15468/dl.zfaw79
Anopheles eiseni	-99.018055	21.180278	Unclassified	GBIF: https://doi.org/10.15468/dl.zfaw79
Anopheles eiseni	-98.990278	21.386111	Unclassified	GBIF: https://doi.org/10.15468/dl.zfaw79
Anopheles eiseni	-98.925278	21.554167	Unclassified	GBIF: https://doi.org/10.15468/dl.zfaw79
Anopheles eiseni	-98.877778	21.230556	Unclassified	GBIF: https://doi.org/10.15468/dl.zfaw79
Anopheles eiseni	-98.856389	21.179444	Unclassified	GBIF: https://doi.org/10.15468/dl.zfaw79
Anopheles eiseni	-98.843055	21.013333	Unclassified	GBIF: https://doi.org/10.15468/dl.zfaw79
Anopheles eiseni	-98.836111	21.256111	Unclassified	GBIF: https://doi.org/10.15468/dl.zfaw79
Anopheles eiseni	-98.791667	21.261111	Unclassified	GBIF: https://doi.org/10.15468/dl.zfaw79
Anopheles eiseni	-98.465	18.600833	Unclassified	GBIF: https://doi.org/10.15468/dl.zfaw79
Anopheles eiseni	-97.1745578	19.6178911	Unclassified	GBIF: https://doi.org/10.15468/dl.zfaw79

Anopheles eiseni	-97.001944	18.884722	Unclassified	GBIF: https://doi.org/10.15468/dl.zfaw79
Anopheles eiseni	-96.946111	19.590278	Unclassified	GBIF: https://doi.org/10.15468/dl.zfaw79
Anopheles eiseni	-96.882778	19.688333	Unclassified	GBIF: https://doi.org/10.15468/dl.zfaw79
Anopheles eiseni	-96.873611	19.653333	Unclassified	GBIF: https://doi.org/10.15468/dl.zfaw79
Anopheles eiseni	-96.86	19.610278	Unclassified	GBIF: https://doi.org/10.15468/dl.zfaw79
Anopheles eiseni	-96.834167	19.650556	Unclassified	GBIF: https://doi.org/10.15468/dl.zfaw79
Anopheles eiseni	-96.691111	15.876667	Unclassified	GBIF: https://doi.org/10.15468/dl.zfaw79
Anopheles eiseni	-96.680833	16.005556	Unclassified	GBIF: https://doi.org/10.15468/dl.zfaw79
Anopheles eiseni	-96.679444	15.982222	Unclassified	GBIF: https://doi.org/10.15468/dl.zfaw79
Anopheles eiseni	-96.6625	15.947222	Unclassified	GBIF: https://doi.org/10.15468/dl.zfaw79
Anopheles eiseni	-96.660833	15.958611	Unclassified	GBIF: https://doi.org/10.15468/dl.zfaw79
Anopheles eiseni	-96.636667	16.011111	Unclassified	GBIF: https://doi.org/10.15468/dl.zfaw79
Anopheles eiseni	-96.493055	17.318056	Unclassified	GBIF: https://doi.org/10.15468/dl.zfaw79
Anopheles eiseni	-96.487222	17.330556	Unclassified	GBIF: https://doi.org/10.15468/dl.zfaw79
Anopheles eiseni	-96.481111	17.299167	Unclassified	GBIF: https://doi.org/10.15468/dl.zfaw79
Anopheles eiseni	-96.375555	15.909444	Unclassified	GBIF: https://doi.org/10.15468/dl.zfaw79
Anopheles eiseni	-96.360555	18.447778	Unclassified	GBIF: https://doi.org/10.15468/dl.zfaw79
Anopheles eiseni	-96.340833	17.465278	Unclassified	GBIF: https://doi.org/10.15468/dl.zfaw79
Anopheles eiseni	-95.101667	18.309722	Unclassified	GBIF: https://doi.org/10.15468/dl.zfaw79
Anopheles eiseni	-95.07	18.585	Unclassified	GBIF: https://doi.org/10.15468/dl.zfaw79
Anopheles eiseni	-94.9875	18.5525	Unclassified	GBIF: https://doi.org/10.15468/dl.zfaw79
Anopheles eiseni	-94.952222	16.845278	Unclassified	GBIF: https://doi.org/10.15468/dl.zfaw79
Anopheles eiseni	-94.691389	18.260833	Unclassified	GBIF: https://doi.org/10.15468/dl.zfaw79
Anopheles eiseni	-93.311111	17.136111	Unclassified	GBIF: https://doi.org/10.15468/dl.zfaw79
Anopheles eiseni	-93.0175	15.538333	Unclassified	GBIF: https://doi.org/10.15468/dl.zfaw79
Anopheles eiseni	-92.952778	17.548056	Unclassified	GBIF: https://doi.org/10.15468/dl.zfaw79
Anopheles eiseni	-92.825555	17.5925	Unclassified	GBIF: https://doi.org/10.15468/dl.zfaw79
Anopheles eiseni	-92.465833	15.138889	Unclassified	GBIF: https://doi.org/10.15468/dl.zfaw79
Anopheles eiseni	-92.434167	15.254444	Unclassified	GBIF: https://doi.org/10.15468/dl.zfaw79
Anopheles eiseni	-92.415	15.262222	Unclassified	GBIF: https://doi.org/10.15468/dl.zfaw79
Anopheles eiseni	-92.357778	15.180556	Unclassified	GBIF: https://doi.org/10.15468/dl.zfaw79
Anopheles eiseni	-92.32423	15.15503	Unclassified	GBIF: https://doi.org/10.15468/dl.zfaw79
Anopheles eiseni	-92.32	15.15	Unclassified	GBIF: https://doi.org/10.15468/dl.zfaw79
Anopheles eiseni	-92.285833	15.156944	Unclassified	GBIF: https://doi.org/10.15468/dl.zfaw79
Anopheles eiseni	-92.260555	14.908056	Unclassified	GBIF: https://doi.org/10.15468/dl.zfaw79
Anopheles eiseni	-92.25476	15.1307	Unclassified	GBIF: https://doi.org/10.15468/dl.zfaw79
Anopheles eiseni	-92.25	15.13	Unclassified	GBIF: https://doi.org/10.15468/dl.zfaw79
Anopheles eiseni	-92.2327059	15.2194521	Unclassified	GBIF: https://doi.org/10.15468/dl.zfaw79
Anopheles eiseni	-92.216389	15.1025	Unclassified	GBIF: https://doi.org/10.15468/dl.zfaw79
Anopheles eiseni	-91.72	16.1021	Unclassified	GBIF: https://doi.org/10.15468/dl.zfaw79
Anopheles eiseni	-91.72	16.1	Unclassified	GBIF: https://doi.org/10.15468/dl.zfaw79
Anopheles eiseni	-89.08331	16.15085	Unclassified	GBIF: https://doi.org/10.15468/dl.zfaw79

Anopheles eiseni	-88.93331	16.16752	Unclassified	GBIF: https://doi.org/10.15468/dl.zfaw79
Anopheles eiseni	-88.91664	16.20085	Unclassified	GBIF: https://doi.org/10.15468/dl.zfaw79
Anopheles eiseni	-88.73914	17.15205	Unclassified	GBIF: https://doi.org/10.15468/dl.zfaw79
Anopheles eiseni	-84.424917	10.234082	Unclassified	GBIF: https://doi.org/10.15468/dl.zfaw79
Anopheles eiseni	-84.422921	10.227921	Unclassified	GBIF: https://doi.org/10.15468/dl.zfaw79
Anopheles eiseni	-84.01352	10.42731	Unclassified	GBIF: https://doi.org/10.15468/dl.zfaw79
Anopheles eiseni	-83.96993	9.74275	Unclassified	GBIF: https://doi.org/10.15468/dl.zfaw79
Anopheles eiseni	-83.4899	8.69651	Unclassified	GBIF: https://doi.org/10.15468/dl.zfaw79
Anopheles eiseni	-83.06159	10.00797	Unclassified	GBIF: https://doi.org/10.15468/dl.zfaw79
Anopheles eiseni	-83.05203	9.93753	Unclassified	GBIF: https://doi.org/10.15468/dl.zfaw79
Anopheles eiseni	-83.01539	9.91063	Unclassified	GBIF: https://doi.org/10.15468/dl.zfaw79
Anopheles eiseni	-82.6819	8.81854	Unclassified	GBIF: https://doi.org/10.15468/dl.zfaw79
Anopheles eiseni	-82.6636	8.7915	Unclassified	GBIF: https://doi.org/10.15468/dl.zfaw79
Anopheles eiseni	-82.59568	8.84876	Unclassified	GBIF: https://doi.org/10.15468/dl.zfaw79
Anopheles eiseni	-82.43268	8.7816	Unclassified	GBIF: https://doi.org/10.15468/dl.zfaw79
Anopheles eiseni	-82.41103	9.28987	Unclassified	GBIF: https://doi.org/10.15468/dl.zfaw79
Anopheles eiseni	-80.13646	8.60425	Unclassified	GBIF: https://doi.org/10.15468/dl.zfaw79
Anopheles eiseni	-80.05294	9.27331	Unclassified	GBIF: https://doi.org/10.15468/dl.zfaw79
Anopheles eiseni	-79.975	9.2958	Unclassified	GBIF: https://doi.org/10.15468/dl.zfaw79
Anopheles eiseni	-79.96164	9.36349	Unclassified	GBIF: https://doi.org/10.15468/dl.zfaw79
Anopheles eiseni	-79.9498	9.36795	Unclassified	GBIF: https://doi.org/10.15468/dl.zfaw79
Anopheles eiseni	-79.9498	9.18463	Unclassified	GBIF: https://doi.org/10.15468/dl.zfaw79
Anopheles eiseni	-79.947751	8.631462	Unclassified	GBIF: https://doi.org/10.15468/dl.zfaw79
Anopheles eiseni	-79.916667	9.266667	Unclassified	GBIF: https://doi.org/10.15468/dl.zfaw79
Anopheles eiseni	-79.883333	9.35	Unclassified	GBIF: https://doi.org/10.15468/dl.zfaw79
Anopheles eiseni	-79.84395	9.15513	Unclassified	GBIF: https://doi.org/10.15468/dl.zfaw79
Anopheles eiseni	-79.772129	8.884647	Unclassified	GBIF: https://doi.org/10.15468/dl.zfaw79
Anopheles eiseni	-79.761713	9.121174	Unclassified	GBIF: https://doi.org/10.15468/dl.zfaw79
Anopheles eiseni	-79.70758	9.1185	Unclassified	GBIF: https://doi.org/10.15468/dl.zfaw79
Anopheles eiseni	-79.65282	9.16352	Unclassified	GBIF: https://doi.org/10.15468/dl.zfaw79
Anopheles eiseni	-79.611502	9.019601	Unclassified	GBIF: https://doi.org/10.15468/dl.zfaw79
Anopheles eiseni	-79.611341	9.019567	Unclassified	GBIF: https://doi.org/10.15468/dl.zfaw79
Anopheles eiseni	-79.611321	9.019604	Unclassified	GBIF: https://doi.org/10.15468/dl.zfaw79
Anopheles eiseni	-79.611316	9.019453	Unclassified	GBIF: https://doi.org/10.15468/dl.zfaw79
Anopheles eiseni	-79.61125	9.01952	Unclassified	GBIF: https://doi.org/10.15468/dl.zfaw79
Anopheles eiseni	-79.611054	9.019452	Unclassified	GBIF: https://doi.org/10.15468/dl.zfaw79
Anopheles eiseni	-79.5333	9.1667	Unclassified	GBIF: https://doi.org/10.15468/dl.zfaw79
Anopheles eiseni	-79.41624	9.16256	Unclassified	GBIF: https://doi.org/10.15468/dl.zfaw79
Anopheles eiseni	-77.6717	6.94616	Unclassified	GBIF: https://doi.org/10.15468/dl.zfaw79
Anopheles eiseni	-77.49975	8.01803	Unclassified	GBIF: https://doi.org/10.15468/dl.zfaw79
Anopheles eiseni	-67.8519	10.44685	Unclassified	GBIF: https://doi.org/10.15468/dl.zfaw79
Anopheles eiseni	-67.74143	10.36136	Unclassified	GBIF: https://doi.org/10.15468/dl.zfaw79

Anopheles eiseni	-67.72324	10.3432	Unclassified	GBIF: https://doi.org/10.15468/dl.zfaw79
Anopheles eiseni	-67.71411	10.34316	Unclassified	GBIF: https://doi.org/10.15468/dl.zfaw79
Anopheles eiseni	-67.69585	10.34309	Unclassified	GBIF: https://doi.org/10.15468/dl.zfaw79
Anopheles eiseni	-67.60189	10.24685	Unclassified	GBIF: https://doi.org/10.15468/dl.zfaw79
Anopheles eiseni	-67.58623	10.35167	Unclassified	GBIF: https://doi.org/10.15468/dl.zfaw79
Anopheles eiseni	-63.040798	-9.91333	Unclassified	GBIF: https://doi.org/10.15468/dl.zfaw79
Anopheles eiseni	-61.64468	10.682511	Unclassified	GBIF: https://doi.org/10.15468/dl.zfaw79
Anopheles eiseni	-61.609989	10.713191	Unclassified	GBIF: https://doi.org/10.15468/dl.zfaw79
Anopheles eiseni	-61.298692	10.717962	Unclassified	GBIF: https://doi.org/10.15468/dl.zfaw79
Anopheles eiseni	-61.297045	10.38824	Unclassified	GBIF: https://doi.org/10.15468/dl.zfaw79
Anopheles eiseni	-61.266667	10.65	Unclassified	GBIF: https://doi.org/10.15468/dl.zfaw79
Anopheles eiseni	-61.2	10.4	Unclassified	GBIF: https://doi.org/10.15468/dl.zfaw79
Anopheles eiseni	-54.383048	4.27418	Unclassified	GBIF: https://doi.org/10.15468/dl.zfaw79
Anopheles eiseni	-54.148707	3.807339	Unclassified	GBIF: https://doi.org/10.15468/dl.zfaw79
Anopheles eiseni	-54.04329	5.484058	Unclassified	GBIF: https://doi.org/10.15468/dl.zfaw79
Anopheles eiseni	-54.03466	3.63007	Unclassified	GBIF: https://doi.org/10.15468/dl.zfaw79
Anopheles eiseni	-54.033772	3.64443	Unclassified	GBIF: https://doi.org/10.15468/dl.zfaw79
Anopheles eiseni	-54.02004	5.50717	Unclassified	GBIF: https://doi.org/10.15468/dl.zfaw79
Anopheles eiseni	-54.000025	4.766684	Unclassified	GBIF: https://doi.org/10.15468/dl.zfaw79
Anopheles eiseni	-53.549917	3.749968	Unclassified	GBIF: https://doi.org/10.15468/dl.zfaw79
Anopheles eiseni	-53.41201	4.60107	Unclassified	GBIF: https://doi.org/10.15468/dl.zfaw79
Anopheles eiseni	-53.28	3.622	Unclassified	GBIF: https://doi.org/10.15468/dl.zfaw79
Anopheles eiseni	-53.28	3.62	Unclassified	GBIF: https://doi.org/10.15468/dl.zfaw79
Anopheles eiseni	-53.27958	3.622118	Unclassified	GBIF: https://doi.org/10.15468/dl.zfaw79
Anopheles eiseni	-53.24036	3.91451	Unclassified	GBIF: https://doi.org/10.15468/dl.zfaw79
Anopheles eiseni	-53.23477	3.92404	Unclassified	GBIF: https://doi.org/10.15468/dl.zfaw79
Anopheles eiseni	-53.099865	3.508858	Unclassified	GBIF: https://doi.org/10.15468/dl.zfaw79
Anopheles eiseni	-53.08	3.05	Unclassified	GBIF: https://doi.org/10.15468/dl.zfaw79
Anopheles eiseni	-53.075	3.05	Unclassified	GBIF: https://doi.org/10.15468/dl.zfaw79
Anopheles eiseni	-53.074507	3.141974	Unclassified	GBIF: https://doi.org/10.15468/dl.zfaw79
Anopheles eiseni	-53.02915	5.01314	Unclassified	GBIF: https://doi.org/10.15468/dl.zfaw79
Anopheles eiseni	-52.950025	5.316688	Unclassified	GBIF: https://doi.org/10.15468/dl.zfaw79
Anopheles eiseni	-52.87724	2.24504	Unclassified	GBIF: https://doi.org/10.15468/dl.zfaw79
Anopheles eiseni	-52.866695	3.416688	Unclassified	GBIF: https://doi.org/10.15468/dl.zfaw79
Anopheles eiseni	-52.700025	4.033351	Unclassified	GBIF: https://doi.org/10.15468/dl.zfaw79
Anopheles eiseni	-52.67012	3.61844	Unclassified	GBIF: https://doi.org/10.15468/dl.zfaw79
Anopheles eiseni	-52.483355	4.666688	Unclassified	GBIF: https://doi.org/10.15468/dl.zfaw79
Anopheles eiseni	-52.47241	5.01171	Unclassified	GBIF: https://doi.org/10.15468/dl.zfaw79
Anopheles eiseni	-52.360475	4.819828	Unclassified	GBIF: https://doi.org/10.15468/dl.zfaw79
Anopheles eiseni	-52.357135	4.886868	Unclassified	GBIF: https://doi.org/10.15468/dl.zfaw79
Anopheles eiseni	-52.33696	4.83273	Unclassified	GBIF: https://doi.org/10.15468/dl.zfaw79
Anopheles eiseni	-52.323296	4.936976	Unclassified	GBIF: https://doi.org/10.15468/dl.zfaw79

Anopheles eiseni	-52.321842	3.171168	Unclassified	GBIF: https://doi.org/10.15468/dl.zfaw79
Anopheles eiseni	-52.312455	4.927798	Unclassified	GBIF: https://doi.org/10.15468/dl.zfaw79
Anopheles eiseni	-52.306695	4.940978	Unclassified	GBIF: https://doi.org/10.15468/dl.zfaw79
Anopheles eiseni	-52.30125	4.89677	Unclassified	GBIF: https://doi.org/10.15468/dl.zfaw79
Anopheles eiseni	-52.251475	4.881148	Unclassified	GBIF: https://doi.org/10.15468/dl.zfaw79
Anopheles eiseni	-52.25125	4.88011	Unclassified	GBIF: https://doi.org/10.15468/dl.zfaw79
Anopheles eiseni	-52.129162	4.314002	Unclassified	GBIF: https://doi.org/10.15468/dl.zfaw79
Anopheles eiseni	-51.78594	3.94548	Unclassified	GBIF: https://doi.org/10.15468/dl.zfaw79
Anopheles eiseni	-43.0406	-22.6528	Unclassified	GBIF: https://doi.org/10.15468/dl.zfaw79
Anopheles eiseni	-42.965599	-22.412201	Unclassified	GBIF: https://doi.org/10.15468/dl.zfaw79
Anopheles elegans	75.9	11.75	Unclassified	GBIF: https://doi.org/10.15468/dl.zfaw79
Anopheles epiroticus	98.31549	9.250532	Unclassified	GBIF: https://doi.org/10.15468/dl.zfaw79
Anopheles epiroticus	98.320916	9.037794	Unclassified	GBIF: https://doi.org/10.15468/dl.zfaw79
Anopheles epiroticus	98.407775	9.330989	Unclassified	GBIF: https://doi.org/10.15468/dl.zfaw79
Anopheles epiroticus	98.411169	8.927604	Unclassified	GBIF: https://doi.org/10.15468/dl.zfaw79
Anopheles epiroticus	98.429807	9.220681	Unclassified	GBIF: https://doi.org/10.15468/dl.zfaw79
Anopheles epiroticus	98.438505	8.277316	Unclassified	GBIF: https://doi.org/10.15468/dl.zfaw79
Anopheles epiroticus	98.443351	8.271899	Unclassified	GBIF: https://doi.org/10.15468/dl.zfaw79
Anopheles epiroticus	98.444316	9.46322	Unclassified	GBIF: https://doi.org/10.15468/dl.zfaw79
Anopheles epiroticus	98.458192	9.843434	Unclassified	GBIF: https://doi.org/10.15468/dl.zfaw79
Anopheles epiroticus	98.46851	9.563197	Unclassified	GBIF: https://doi.org/10.15468/dl.zfaw79
Anopheles epiroticus	98.484936	9.903064	Unclassified	GBIF: https://doi.org/10.15468/dl.zfaw79
Anopheles epiroticus	98.492807	9.451781	Unclassified	GBIF: https://doi.org/10.15468/dl.zfaw79
Anopheles epiroticus	98.50142	9.454588	Unclassified	GBIF: https://doi.org/10.15468/dl.zfaw79
Anopheles epiroticus	98.565829	9.5827	Unclassified	GBIF: https://doi.org/10.15468/dl.zfaw79
Anopheles epiroticus	98.570345	9.909454	Unclassified	GBIF: https://doi.org/10.15468/dl.zfaw79
Anopheles epiroticus	98.578775	9.522252	Unclassified	GBIF: https://doi.org/10.15468/dl.zfaw79
Anopheles epiroticus	98.602055	9.529134	Unclassified	GBIF: https://doi.org/10.15468/dl.zfaw79
Anopheles epiroticus	98.606255	9.613244	Unclassified	GBIF: https://doi.org/10.15468/dl.zfaw79
Anopheles epiroticus	98.614928	9.997566	Unclassified	GBIF: https://doi.org/10.15468/dl.zfaw79
Anopheles epiroticus	98.615631	10.015679	Unclassified	GBIF: https://doi.org/10.15468/dl.zfaw79
Anopheles epiroticus	98.64058	10.056865	Unclassified	GBIF: https://doi.org/10.15468/dl.zfaw79
Anopheles epiroticus	98.670266	10.098068	Unclassified	GBIF: https://doi.org/10.15468/dl.zfaw79
Anopheles epiroticus	98.679836	10.126277	Unclassified	GBIF: https://doi.org/10.15468/dl.zfaw79
Anopheles epiroticus	98.681766	10.132158	Unclassified	GBIF: https://doi.org/10.15468/dl.zfaw79
Anopheles epiroticus	98.689023	10.131174	Unclassified	GBIF: https://doi.org/10.15468/dl.zfaw79
Anopheles epiroticus	98.757503	10.338121	Unclassified	GBIF: https://doi.org/10.15468/dl.zfaw79
Anopheles epiroticus	98.774574	10.330236	Unclassified	GBIF: https://doi.org/10.15468/dl.zfaw79
Anopheles epiroticus	98.809541	10.398834	Unclassified	GBIF: https://doi.org/10.15468/dl.zfaw79
Anopheles epiroticus	98.8133	8.855	Unclassified	GBIF: https://doi.org/10.15468/dl.zfaw79
Anopheles epiroticus	98.821815	10.473006	Unclassified	GBIF: https://doi.org/10.15468/dl.zfaw79
Anopheles epiroticus	98.832614	10.467871	Unclassified	GBIF: https://doi.org/10.15468/dl.zfaw79

Anopheles epiroticus	98.842626	10.38754	Unclassified	GBIF: https://doi.org/10.15468/dl.zfaw79
Anopheles epiroticus	98.843182	10.294795	Unclassified	GBIF: https://doi.org/10.15468/dl.zfaw79
Anopheles epiroticus	98.9533	9.03	Unclassified	GBIF: https://doi.org/10.15468/dl.zfaw79
Anopheles epiroticus	101.105607	13.65539	Unclassified	GBIF: https://doi.org/10.15468/dl.zfaw79
Anopheles epiroticus	101.916117	12.586819	Unclassified	GBIF: https://doi.org/10.15468/dl.zfaw79
Anopheles epiroticus	102.276671	12.131813	Unclassified	GBIF: https://doi.org/10.15468/dl.zfaw79
Anopheles epiroticus	102.338285	12.237169	Unclassified	GBIF: https://doi.org/10.15468/dl.zfaw79
Anopheles epiroticus	102.342697	12.023555	Unclassified	GBIF: https://doi.org/10.15468/dl.zfaw79
Anopheles epiroticus	102.34474	12.484815	Unclassified	GBIF: https://doi.org/10.15468/dl.zfaw79
Anopheles epiroticus	102.34776	11.989918	Unclassified	GBIF: https://doi.org/10.15468/dl.zfaw79
Anopheles epiroticus	102.354082	12.10275	Unclassified	GBIF: https://doi.org/10.15468/dl.zfaw79
Anopheles epiroticus	102.384203	12.026239	Unclassified	GBIF: https://doi.org/10.15468/dl.zfaw79
Anopheles epiroticus	102.392028	12.204587	Unclassified	GBIF: https://doi.org/10.15468/dl.zfaw79
Anopheles epiroticus	102.393685	11.994705	Unclassified	GBIF: https://doi.org/10.15468/dl.zfaw79
Anopheles epiroticus	102.421872	12.275075	Unclassified	GBIF: https://doi.org/10.15468/dl.zfaw79
Anopheles epiroticus	102.455168	12.189529	Unclassified	GBIF: https://doi.org/10.15468/dl.zfaw79
Anopheles epiroticus	102.492493	11.823558	Unclassified	GBIF: https://doi.org/10.15468/dl.zfaw79
Anopheles epiroticus	102.530348	12.400742	Unclassified	GBIF: https://doi.org/10.15468/dl.zfaw79
Anopheles epiroticus	102.564458	12.390208	Unclassified	GBIF: https://doi.org/10.15468/dl.zfaw79
Anopheles epiroticus	102.578558	11.67832	Unclassified	GBIF: https://doi.org/10.15468/dl.zfaw79
Anopheles epiroticus	102.582475	11.611098	Unclassified	GBIF: https://doi.org/10.15468/dl.zfaw79
Anopheles epiroticus	102.618292	12.230746	Unclassified	GBIF: https://doi.org/10.15468/dl.zfaw79
Anopheles epiroticus	102.629235	12.444094	Unclassified	GBIF: https://doi.org/10.15468/dl.zfaw79
Anopheles epiroticus	102.660827	12.199483	Unclassified	GBIF: https://doi.org/10.15468/dl.zfaw79
Anopheles epiroticus	102.666093	12.227541	Unclassified	GBIF: https://doi.org/10.15468/dl.zfaw79
Anopheles epiroticus	102.666441	12.187784	Unclassified	GBIF: https://doi.org/10.15468/dl.zfaw79
Anopheles epiroticus	102.679426	12.173435	Unclassified	GBIF: https://doi.org/10.15468/dl.zfaw79
Anopheles epiroticus	102.679598	12.153556	Unclassified	GBIF: https://doi.org/10.15468/dl.zfaw79
Anopheles epiroticus	102.701124	12.106745	Unclassified	GBIF: https://doi.org/10.15468/dl.zfaw79
Anopheles epiroticus	102.704913	12.09322	Unclassified	GBIF: https://doi.org/10.15468/dl.zfaw79
Anopheles epiroticus	102.731724	12.071752	Unclassified	GBIF: https://doi.org/10.15468/dl.zfaw79
Anopheles epiroticus	102.767958	12.020534	Unclassified	GBIF: https://doi.org/10.15468/dl.zfaw79
Anopheles epiroticus	102.778824	11.926632	Unclassified	GBIF: https://doi.org/10.15468/dl.zfaw79
Anopheles epiroticus	102.783061	11.957611	Unclassified	GBIF: https://doi.org/10.15468/dl.zfaw79
Anopheles epiroticus	102.808502	11.888004	Unclassified	GBIF: https://doi.org/10.15468/dl.zfaw79
Anopheles epiroticus	102.817793	11.873617	Unclassified	GBIF: https://doi.org/10.15468/dl.zfaw79
Anopheles epiroticus	102.829025	11.845686	Unclassified	GBIF: https://doi.org/10.15468/dl.zfaw79
Anopheles epiroticus	102.885517	11.777426	Unclassified	GBIF: https://doi.org/10.15468/dl.zfaw79
Anopheles epiroticus	102.895765	11.756714	Unclassified	GBIF: https://doi.org/10.15468/dl.zfaw79
Anopheles epiroticus	102.896373	11.769024	Unclassified	GBIF: https://doi.org/10.15468/dl.zfaw79
Anopheles epiroticus	102.906576	11.718147	Unclassified	GBIF: https://doi.org/10.15468/dl.zfaw79
Anopheles epiroticus	102.907592	11.701079	Unclassified	GBIF: https://doi.org/10.15468/dl.zfaw79

Anopheles epiroticus	102.910037	11.655242	Unclassified	GBIF: https://doi.org/10.15468/dl.zfaw79
Anopheles erythraeus	39.088608	15.449167	Unclassified	GBIF: https://doi.org/10.15468/dl.zfaw79
Anopheles evansae	-79.783333	8.880278	Unclassified	GBIF: https://doi.org/10.15468/dl.zfaw79
Anopheles evansae	-79.772129	8.884647	Unclassified	GBIF: https://doi.org/10.15468/dl.zfaw79
Anopheles evansae	-79.449306	9.04817	Unclassified	GBIF: https://doi.org/10.15468/dl.zfaw79
Anopheles evansae	-79.447833	9.048501	Unclassified	GBIF: https://doi.org/10.15468/dl.zfaw79
Anopheles evansae	-79.439986	9.07	Unclassified	GBIF: https://doi.org/10.15468/dl.zfaw79
Anopheles evansae	-79.439169	9.069383	Unclassified	GBIF: https://doi.org/10.15468/dl.zfaw79
Anopheles evansae	-79.438728	9.069225	Unclassified	GBIF: https://doi.org/10.15468/dl.zfaw79
Anopheles evansae	-65.58413	-27.33271	Unclassified	GBIF: https://doi.org/10.15468/dl.zfaw79
Anopheles evansae	-65.50079	-27.16604	Unclassified	GBIF: https://doi.org/10.15468/dl.zfaw79
Anopheles evansae	-65.38486	-10.80362	Unclassified	GBIF: https://doi.org/10.15468/dl.zfaw79
Anopheles evansae	-65.320301	-27.038434	Unclassified	GBIF: https://doi.org/10.15468/dl.zfaw79
Anopheles evansae	-64.5	-23.12	Unclassified	GBIF: https://doi.org/10.15468/dl.zfaw79
Anopheles evansae	-63.21816	-19.80366	Unclassified	GBIF: https://doi.org/10.15468/dl.zfaw79
Anopheles evansae	-56.5	-20.2	Unclassified	GBIF: https://doi.org/10.15468/dl.zfaw79
Anopheles evansae	-54.5833	-25.55	Unclassified	GBIF: https://doi.org/10.15468/dl.zfaw79
Anopheles evansae	-54.35	-24.86	Unclassified	GBIF: https://doi.org/10.15468/dl.zfaw79
Anopheles evansae	-54.25	-24.0667	Unclassified	GBIF: https://doi.org/10.15468/dl.zfaw79
Anopheles evansae	-53.860556	-27.265	Unclassified	GBIF: https://doi.org/10.15468/dl.zfaw79
Anopheles evansae	-52.738605	5.115338	Unclassified	GBIF: https://doi.org/10.15468/dl.zfaw79
Anopheles evansae	-52.466075	4.566139	Unclassified	GBIF: https://doi.org/10.15468/dl.zfaw79
Anopheles evansae	-52.3667	-24.05	Unclassified	GBIF: https://doi.org/10.15468/dl.zfaw79
Anopheles evansae	-52.2333	-23.55	Unclassified	GBIF: https://doi.org/10.15468/dl.zfaw79
Anopheles evansae	-51.79697	3.913685	Unclassified	GBIF: https://doi.org/10.15468/dl.zfaw79
Anopheles evansae	-51.237222	-30.019139	Unclassified	GBIF: https://doi.org/10.15468/dl.zfaw79
Anopheles evansae	-51.15	-23.3	Unclassified	GBIF: https://doi.org/10.15468/dl.zfaw79
Anopheles evansae	-51.11717	-20.63368	Unclassified	GBIF: https://doi.org/10.15468/dl.zfaw79
Anopheles evansae	-51.05	-23.2833	Unclassified	GBIF: https://doi.org/10.15468/dl.zfaw79
Anopheles evansae	-49.7566	-23.1976	Unclassified	GBIF: https://doi.org/10.15468/dl.zfaw79
Anopheles evansae	-48.8293	-25.4754	Unclassified	GBIF: https://doi.org/10.15468/dl.zfaw79
Anopheles evansae	-48.44	-22.07	Unclassified	GBIF: https://doi.org/10.15468/dl.zfaw79
Anopheles evansae	-48.39	-22.14	Unclassified	GBIF: https://doi.org/10.15468/dl.zfaw79
Anopheles evansae	-45.45	-22.96	Unclassified	GBIF: https://doi.org/10.15468/dl.zfaw79
Anopheles evansae	-43.84003	-22.8918	Unclassified	GBIF: https://doi.org/10.15468/dl.zfaw79
Anopheles evansae	-43.78351	-22.88373	Unclassified	GBIF: https://doi.org/10.15468/dl.zfaw79
Anopheles evansae	-43.46683	-22.88373	Unclassified	GBIF: https://doi.org/10.15468/dl.zfaw79
Anopheles evansae	-43.292801	-22.1619	Unclassified	GBIF: https://doi.org/10.15468/dl.zfaw79
Anopheles evansae	-43.2075	-22.9028	Unclassified	GBIF: https://doi.org/10.15468/dl.zfaw79
Anopheles evansae	-43.03348	-22.6504	Unclassified	GBIF: https://doi.org/10.15468/dl.zfaw79
Anopheles evansae	-42.99159	-22.91947	Unclassified	GBIF: https://doi.org/10.15468/dl.zfaw79
Anopheles evansae	-42.99067	-22.91584	Unclassified	GBIF: https://doi.org/10.15468/dl.zfaw79

Anopheles evansae	-42.653099	-22.4625	Unclassified	GBIF: https://doi.org/10.15468/dl.zfaw79
Anopheles evansae	-40.56705	-16.26713	Unclassified	GBIF: https://doi.org/10.15468/dl.zfaw79
Anopheles evansae	-40.35002	-10.73354	Unclassified	GBIF: https://doi.org/10.15468/dl.zfaw79
Anopheles evansae	-40.08334	-13.85025	Unclassified	GBIF: https://doi.org/10.15468/dl.zfaw79
Anopheles evansae	-39.95	-14.01692	Unclassified	GBIF: https://doi.org/10.15468/dl.zfaw79
Anopheles evansae	-39.53332	-15.93362	Unclassified	GBIF: https://doi.org/10.15468/dl.zfaw79
Anopheles evansae	-39.51665	-17.08364	Unclassified	GBIF: https://doi.org/10.15468/dl.zfaw79
Anopheles evansae	-39.33331	-14.30026	Unclassified	GBIF: https://doi.org/10.15468/dl.zfaw79
Anopheles evansae	-39.33331	-14.33359	Unclassified	GBIF: https://doi.org/10.15468/dl.zfaw79
Anopheles evansae	-38.93329	-4.25011	Unclassified	GBIF: https://doi.org/10.15468/dl.zfaw79
Anopheles evansae	-38.71662	-12.53356	Unclassified	GBIF: https://doi.org/10.15468/dl.zfaw79
Anopheles evansae	-38.30036	-12.85045	Unclassified	GBIF: https://doi.org/10.15468/dl.zfaw79
Anopheles evansae	-35.23314	-5.95013	Unclassified	GBIF: https://doi.org/10.15468/dl.zfaw79
Anopheles farauti	70.942154	38.569332	Unclassified	GBIF: https://doi.org/10.15468/dl.zfaw79
Anopheles farauti	125.937002	-2.20147	Unclassified	GBIF: https://doi.org/10.15468/dl.zfaw79
Anopheles farauti	128.4167	2.3333	Unclassified	GBIF: https://doi.org/10.15468/dl.zfaw79
Anopheles farauti	128.416702	2.3333	Unclassified	GBIF: https://doi.org/10.15468/dl.zfaw79
Anopheles farauti	128.44267	2.321306	Unclassified	GBIF: https://doi.org/10.15468/dl.zfaw79
Anopheles farauti	128.681527	-2.868118	Unclassified	GBIF: https://doi.org/10.15468/dl.zfaw79
Anopheles farauti	129.404032	-2.85697	Unclassified	GBIF: https://doi.org/10.15468/dl.zfaw79
Anopheles farauti	130.43	-3.17	Unclassified	GBIF: https://doi.org/10.15468/dl.zfaw79
Anopheles farauti	130.828	-12.452	Unclassified	GBIF: https://doi.org/10.15468/dl.zfaw79
Anopheles farauti	130.833298	-12.43333	Unclassified	GBIF: https://doi.org/10.15468/dl.zfaw79
Anopheles farauti	130.833333	-12.466667	Unclassified	GBIF: https://doi.org/10.15468/dl.zfaw79
Anopheles farauti	130.837	-12.444	Unclassified	GBIF: https://doi.org/10.15468/dl.zfaw79
Anopheles farauti	130.85	-12.384	Unclassified	GBIF: https://doi.org/10.15468/dl.zfaw79
Anopheles farauti	130.850006	-12.416667	Unclassified	GBIF: https://doi.org/10.15468/dl.zfaw79
Anopheles farauti	130.8667	-12.45	Unclassified	GBIF: https://doi.org/10.15468/dl.zfaw79
Anopheles farauti	130.906852	-12.38481	Unclassified	GBIF: https://doi.org/10.15468/dl.zfaw79
Anopheles farauti	131	-12.983334	Unclassified	GBIF: https://doi.org/10.15468/dl.zfaw79
Anopheles farauti	131	-12.98333	Unclassified	GBIF: https://doi.org/10.15468/dl.zfaw79
Anopheles farauti	131.100006	-13.233334	Unclassified	GBIF: https://doi.org/10.15468/dl.zfaw79
Anopheles farauti	131.10001	-13.23333	Unclassified	GBIF: https://doi.org/10.15468/dl.zfaw79
Anopheles farauti	131.255816	-0.876191	Unclassified	GBIF: https://doi.org/10.15468/dl.zfaw79
Anopheles farauti	132.866699	-12.58333	Unclassified	GBIF: https://doi.org/10.15468/dl.zfaw79
Anopheles farauti	132.8667	-12.58333	Unclassified	GBIF: https://doi.org/10.15468/dl.zfaw79
Anopheles farauti	134.217407	-5.755787	Unclassified	GBIF: https://doi.org/10.15468/dl.zfaw79
Anopheles farauti	134.733337	-14.733334	Unclassified	GBIF: https://doi.org/10.15468/dl.zfaw79
Anopheles farauti	134.73334	-14.73333	Unclassified	GBIF: https://doi.org/10.15468/dl.zfaw79
Anopheles farauti	136.753	-12.216	Unclassified	GBIF: https://doi.org/10.15468/dl.zfaw79
Anopheles farauti	136.76666	-12.16667	Unclassified	GBIF: https://doi.org/10.15468/dl.zfaw79
Anopheles farauti	136.766663	-12.166667	Unclassified	GBIF: https://doi.org/10.15468/dl.zfaw79

Anopheles farauti	136.76669	-12.18333	Unclassified	GBIF: https://doi.org/10.15468/dl.zfaw79
Anopheles farauti	136.766693	-12.18333	Unclassified	GBIF: https://doi.org/10.15468/dl.zfaw79
Anopheles farauti	138.883333	-1.966667	Unclassified	GBIF: https://doi.org/10.15468/dl.zfaw79
Anopheles farauti	139.013901	-1.939183	Unclassified	GBIF: https://doi.org/10.15468/dl.zfaw79
Anopheles farauti	140.276667	-6.080278	Unclassified	GBIF: https://doi.org/10.15468/dl.zfaw79
Anopheles farauti	140.28329	-6.08333	Unclassified	GBIF: https://doi.org/10.15468/dl.zfaw79
Anopheles farauti	140.283295	-6.083333	Unclassified	GBIF: https://doi.org/10.15468/dl.zfaw79
Anopheles farauti	140.404444	-8.495833	Unclassified	GBIF: https://doi.org/10.15468/dl.zfaw79
Anopheles farauti	140.407156	-8.500401	Unclassified	GBIF: https://doi.org/10.15468/dl.zfaw79
Anopheles farauti	141.066666	-17.666666	Unclassified	GBIF: https://doi.org/10.15468/dl.zfaw79
Anopheles farauti	141.06667	-17.66667	Unclassified	GBIF: https://doi.org/10.15468/dl.zfaw79
Anopheles farauti	141.733337	-13.366667	Unclassified	GBIF: https://doi.org/10.15468/dl.zfaw79
Anopheles farauti	141.73334	-13.36667	Unclassified	GBIF: https://doi.org/10.15468/dl.zfaw79
Anopheles farauti	141.75	-15.466666	Unclassified	GBIF: https://doi.org/10.15468/dl.zfaw79
Anopheles farauti	141.75	-15.46667	Unclassified	GBIF: https://doi.org/10.15468/dl.zfaw79
Anopheles farauti	141.88333	-12.68333	Unclassified	GBIF: https://doi.org/10.15468/dl.zfaw79
Anopheles farauti	141.883331	-12.683333	Unclassified	GBIF: https://doi.org/10.15468/dl.zfaw79
Anopheles farauti	142.116669	-10.716666	Unclassified	GBIF: https://doi.org/10.15468/dl.zfaw79
Anopheles farauti	142.11667	-10.71667	Unclassified	GBIF: https://doi.org/10.15468/dl.zfaw79
Anopheles farauti	142.21666	-9.283334	Unclassified	GBIF: https://doi.org/10.15468/dl.zfaw79
Anopheles farauti	142.21666	-9.28333	Unclassified	GBIF: https://doi.org/10.15468/dl.zfaw79
Anopheles farauti	142.28334	-10.616667	Unclassified	GBIF: https://doi.org/10.15468/dl.zfaw79
Anopheles farauti	142.28334	-10.61667	Unclassified	GBIF: https://doi.org/10.15468/dl.zfaw79
Anopheles farauti	142.350006	-10.85	Unclassified	GBIF: https://doi.org/10.15468/dl.zfaw79
Anopheles farauti	142.369	-10.848	Unclassified	GBIF: https://doi.org/10.15468/dl.zfaw79
Anopheles farauti	142.46666	-10.8	Unclassified	GBIF: https://doi.org/10.15468/dl.zfaw79
Anopheles farauti	142.467	-10.8	Unclassified	GBIF: https://doi.org/10.15468/dl.zfaw79
Anopheles farauti	142.5	-11.833333	Unclassified	GBIF: https://doi.org/10.15468/dl.zfaw79
Anopheles farauti	142.5	-11.83333	Unclassified	GBIF: https://doi.org/10.15468/dl.zfaw79
Anopheles farauti	142.52	-5.38	Unclassified	GBIF: https://doi.org/10.15468/dl.zfaw79
Anopheles farauti	142.53334	-11.733334	Unclassified	GBIF: https://doi.org/10.15468/dl.zfaw79
Anopheles farauti	142.53334	-11.73333	Unclassified	GBIF: https://doi.org/10.15468/dl.zfaw79
Anopheles farauti	142.66667	-9.38333	Unclassified	GBIF: https://doi.org/10.15468/dl.zfaw79
Anopheles farauti	142.666672	-9.383333	Unclassified	GBIF: https://doi.org/10.15468/dl.zfaw79
Anopheles farauti	142.91667	-5.83333	Unclassified	GBIF: https://doi.org/10.15468/dl.zfaw79
Anopheles farauti	143.28329	-12.7833	Unclassified	GBIF: https://doi.org/10.15468/dl.zfaw79
Anopheles farauti	143.283295	-12.7833	Unclassified	GBIF: https://doi.org/10.15468/dl.zfaw79
Anopheles farauti	143.28334	-12.733334	Unclassified	GBIF: https://doi.org/10.15468/dl.zfaw79
Anopheles farauti	143.28334	-12.73333	Unclassified	GBIF: https://doi.org/10.15468/dl.zfaw79
Anopheles farauti	143.3	-12.73333	Unclassified	GBIF: https://doi.org/10.15468/dl.zfaw79
Anopheles farauti	143.300003	-12.733334	Unclassified	GBIF: https://doi.org/10.15468/dl.zfaw79
Anopheles farauti	143.316666	-12.716666	Unclassified	GBIF: https://doi.org/10.15468/dl.zfaw79

Anopheles farauti	143.31667	-12.71667	Unclassified	GBIF: https://doi.org/10.15468/dl.zfaw79
Anopheles farauti	143.51666	-12.96667	Unclassified	GBIF: https://doi.org/10.15468/dl.zfaw79
Anopheles farauti	143.516663	-12.966666	Unclassified	GBIF: https://doi.org/10.15468/dl.zfaw79
Anopheles farauti	143.583328	-3.583333	Unclassified	GBIF: https://doi.org/10.15468/dl.zfaw79
Anopheles farauti	143.58333	-3.58333	Unclassified	GBIF: https://doi.org/10.15468/dl.zfaw79
Anopheles farauti	143.66667	-5.5	Unclassified	GBIF: https://doi.org/10.15468/dl.zfaw79
Anopheles farauti	143.666672	-5.5	Unclassified	GBIF: https://doi.org/10.15468/dl.zfaw79
Anopheles farauti	143.6667	-3.58333	Unclassified	GBIF: https://doi.org/10.15468/dl.zfaw79
Anopheles farauti	143.666702	-3.583333	Unclassified	GBIF: https://doi.org/10.15468/dl.zfaw79
Anopheles farauti	143.76666	-9.58333	Unclassified	GBIF: https://doi.org/10.15468/dl.zfaw79
Anopheles farauti	143.766663	-9.583333	Unclassified	GBIF: https://doi.org/10.15468/dl.zfaw79
Anopheles farauti	144.0629	-9.9204	Unclassified	GBIF: https://doi.org/10.15468/dl.zfaw79
Anopheles farauti	144.063	-9.92	Unclassified	GBIF: https://doi.org/10.15468/dl.zfaw79
Anopheles farauti	144.066666	-4.066667	Unclassified	GBIF: https://doi.org/10.15468/dl.zfaw79
Anopheles farauti	144.06667	-4.06667	Unclassified	GBIF: https://doi.org/10.15468/dl.zfaw79
Anopheles farauti	144.25	-5.833333	Unclassified	GBIF: https://doi.org/10.15468/dl.zfaw79
Anopheles farauti	144.25	-5.83333	Unclassified	GBIF: https://doi.org/10.15468/dl.zfaw79
Anopheles farauti	144.66667	-5.91667	Unclassified	GBIF: https://doi.org/10.15468/dl.zfaw79
Anopheles farauti	144.666672	-5.916667	Unclassified	GBIF: https://doi.org/10.15468/dl.zfaw79
Anopheles farauti	145.066666	-6.98333	Unclassified	GBIF: https://doi.org/10.15468/dl.zfaw79
Anopheles farauti	145.06667	-6.98333	Unclassified	GBIF: https://doi.org/10.15468/dl.zfaw79
Anopheles farauti	145.14999	-15.41667	Unclassified	GBIF: https://doi.org/10.15468/dl.zfaw79
Anopheles farauti	145.149994	-15.416667	Unclassified	GBIF: https://doi.org/10.15468/dl.zfaw79
Anopheles farauti	145.316666	-16.25	Unclassified	GBIF: https://doi.org/10.15468/dl.zfaw79
Anopheles farauti	145.316666	-16.299999	Unclassified	GBIF: https://doi.org/10.15468/dl.zfaw79
Anopheles farauti	145.31667	-16.25	Unclassified	GBIF: https://doi.org/10.15468/dl.zfaw79
Anopheles farauti	145.31667	-16.3	Unclassified	GBIF: https://doi.org/10.15468/dl.zfaw79
Anopheles farauti	145.350006	-15.866667	Unclassified	GBIF: https://doi.org/10.15468/dl.zfaw79
Anopheles farauti	145.35001	-15.86667	Unclassified	GBIF: https://doi.org/10.15468/dl.zfaw79
Anopheles farauti	145.366699	-25.4167	Unclassified	GBIF: https://doi.org/10.15468/dl.zfaw79
Anopheles farauti	145.3667	-25.4167	Unclassified	GBIF: https://doi.org/10.15468/dl.zfaw79
Anopheles farauti	145.3833	-6.08333	Unclassified	GBIF: https://doi.org/10.15468/dl.zfaw79
Anopheles farauti	145.409463	-16.358583	Unclassified	GBIF: https://doi.org/10.15468/dl.zfaw79
Anopheles farauti	145.449997	-16.416666	Unclassified	GBIF: https://doi.org/10.15468/dl.zfaw79
Anopheles farauti	145.4833	-16.5	Unclassified	GBIF: https://doi.org/10.15468/dl.zfaw79
Anopheles farauti	145.5	-5.45	Unclassified	GBIF: https://doi.org/10.15468/dl.zfaw79
Anopheles farauti	145.607	-17.169	Unclassified	GBIF: https://doi.org/10.15468/dl.zfaw79
Anopheles farauti	145.63333	-17.25	Unclassified	GBIF: https://doi.org/10.15468/dl.zfaw79
Anopheles farauti	145.633331	-17.25	Unclassified	GBIF: https://doi.org/10.15468/dl.zfaw79
Anopheles farauti	145.6833	-5.2833	Unclassified	GBIF: https://doi.org/10.15468/dl.zfaw79
Anopheles farauti	145.683304	-5.2833	Unclassified	GBIF: https://doi.org/10.15468/dl.zfaw79
Anopheles farauti	145.733337	-16.883333	Unclassified	GBIF: https://doi.org/10.15468/dl.zfaw79

Anopheles farauti	145.73334	-16.88333	Unclassified	GBIF: https://doi.org/10.15468/dl.zfaw79
Anopheles farauti	145.742937	-16.912298	Unclassified	GBIF: https://doi.org/10.15468/dl.zfaw79
Anopheles farauti	145.75	-5.35	Unclassified	GBIF: https://doi.org/10.15468/dl.zfaw79
Anopheles farauti	145.75	-5.3	Unclassified	GBIF: https://doi.org/10.15468/dl.zfaw79
Anopheles farauti	145.76666	-17.03333	Unclassified	GBIF: https://doi.org/10.15468/dl.zfaw79
Anopheles farauti	145.76666	-16.91667	Unclassified	GBIF: https://doi.org/10.15468/dl.zfaw79
Anopheles farauti	145.766663	-16.916666	Unclassified	GBIF: https://doi.org/10.15468/dl.zfaw79
Anopheles farauti	145.766663	-17.033333	Unclassified	GBIF: https://doi.org/10.15468/dl.zfaw79
Anopheles farauti	145.78329	-5.0833	Unclassified	GBIF: https://doi.org/10.15468/dl.zfaw79
Anopheles farauti	145.783295	-5.0833	Unclassified	GBIF: https://doi.org/10.15468/dl.zfaw79
Anopheles farauti	145.866699	-6.283333	Unclassified	GBIF: https://doi.org/10.15468/dl.zfaw79
Anopheles farauti	145.8667	-6.28333	Unclassified	GBIF: https://doi.org/10.15468/dl.zfaw79
Anopheles farauti	145.916667	-6.333333	Unclassified	GBIF: https://doi.org/10.15468/dl.zfaw79
Anopheles farauti	145.91667	-17.55	Unclassified	GBIF: https://doi.org/10.15468/dl.zfaw79
Anopheles farauti	145.91667	-6.33333	Unclassified	GBIF: https://doi.org/10.15468/dl.zfaw79
Anopheles farauti	145.916672	-17.549999	Unclassified	GBIF: https://doi.org/10.15468/dl.zfaw79
Anopheles farauti	145.9167	-17.4167	Unclassified	GBIF: https://doi.org/10.15468/dl.zfaw79
Anopheles farauti	145.916702	-17.4167	Unclassified	GBIF: https://doi.org/10.15468/dl.zfaw79
Anopheles farauti	145.966705	-17.433332	Unclassified	GBIF: https://doi.org/10.15468/dl.zfaw79
Anopheles farauti	145.96671	-17.43333	Unclassified	GBIF: https://doi.org/10.15468/dl.zfaw79
Anopheles farauti	146	-17.316668	Unclassified	GBIF: https://doi.org/10.15468/dl.zfaw79
Anopheles farauti	146	-17.333334	Unclassified	GBIF: https://doi.org/10.15468/dl.zfaw79
Anopheles farauti	146	-17.31667	Unclassified	GBIF: https://doi.org/10.15468/dl.zfaw79
Anopheles farauti	146	-17.33333	Unclassified	GBIF: https://doi.org/10.15468/dl.zfaw79
Anopheles farauti	146.01666	-18.26667	Unclassified	GBIF: https://doi.org/10.15468/dl.zfaw79
Anopheles farauti	146.016663	-18.266666	Unclassified	GBIF: https://doi.org/10.15468/dl.zfaw79
Anopheles farauti	146.02	-17.38	Unclassified	GBIF: https://doi.org/10.15468/dl.zfaw79
Anopheles farauti	146.03329	-17.56667	Unclassified	GBIF: https://doi.org/10.15468/dl.zfaw79
Anopheles farauti	146.033295	-17.566669	Unclassified	GBIF: https://doi.org/10.15468/dl.zfaw79
Anopheles farauti	146.0416	-17.375	Unclassified	GBIF: https://doi.org/10.15468/dl.zfaw79
Anopheles farauti	146.066666	-17.866667	Unclassified	GBIF: https://doi.org/10.15468/dl.zfaw79
Anopheles farauti	146.06667	-17.86667	Unclassified	GBIF: https://doi.org/10.15468/dl.zfaw79
Anopheles farauti	146.083328	-17.566668	Unclassified	GBIF: https://doi.org/10.15468/dl.zfaw79
Anopheles farauti	146.083328	-18.633333	Unclassified	GBIF: https://doi.org/10.15468/dl.zfaw79
Anopheles farauti	146.08333	-17.56667	Unclassified	GBIF: https://doi.org/10.15468/dl.zfaw79
Anopheles farauti	146.08333	-18.63333	Unclassified	GBIF: https://doi.org/10.15468/dl.zfaw79
Anopheles farauti	146.116669	-17.6	Unclassified	GBIF: https://doi.org/10.15468/dl.zfaw79
Anopheles farauti	146.11667	-17.6	Unclassified	GBIF: https://doi.org/10.15468/dl.zfaw79
Anopheles farauti	146.16388	-8.21888	Unclassified	GBIF: https://doi.org/10.15468/dl.zfaw79
Anopheles farauti	146.16667	-18.65	Unclassified	GBIF: https://doi.org/10.15468/dl.zfaw79
Anopheles farauti	146.166672	-18.65	Unclassified	GBIF: https://doi.org/10.15468/dl.zfaw79
Anopheles farauti	146.18333	-8.18333	Unclassified	GBIF: https://doi.org/10.15468/dl.zfaw79

Anopheles farauti	146.183334	-8.183333	Unclassified	GBIF: https://doi.org/10.15468/dl.zfaw79
Anopheles farauti	146.251007	-18.799	Unclassified	GBIF: https://doi.org/10.15468/dl.zfaw79
Anopheles farauti	146.25101	-18.799	Unclassified	GBIF: https://doi.org/10.15468/dl.zfaw79
Anopheles farauti	146.28	-18.73333	Unclassified	GBIF: https://doi.org/10.15468/dl.zfaw79
Anopheles farauti	146.28334	-18.583334	Unclassified	GBIF: https://doi.org/10.15468/dl.zfaw79
Anopheles farauti	146.28334	-18.58333	Unclassified	GBIF: https://doi.org/10.15468/dl.zfaw79
Anopheles farauti	146.3	-18.71667	Unclassified	GBIF: https://doi.org/10.15468/dl.zfaw79
Anopheles farauti	146.300003	-18.733334	Unclassified	GBIF: https://doi.org/10.15468/dl.zfaw79
Anopheles farauti	146.300003	-18.716667	Unclassified	GBIF: https://doi.org/10.15468/dl.zfaw79
Anopheles farauti	146.333328	-18.533333	Unclassified	GBIF: https://doi.org/10.15468/dl.zfaw79
Anopheles farauti	146.33333	-18.53333	Unclassified	GBIF: https://doi.org/10.15468/dl.zfaw79
Anopheles farauti	146.5333	-8.7667	Unclassified	GBIF: https://doi.org/10.15468/dl.zfaw79
Anopheles farauti	146.55	-18.66667	Unclassified	GBIF: https://doi.org/10.15468/dl.zfaw79
Anopheles farauti	146.550003	-18.666666	Unclassified	GBIF: https://doi.org/10.15468/dl.zfaw79
Anopheles farauti	146.561001	-6.497125	Unclassified	GBIF: https://doi.org/10.15468/dl.zfaw79
Anopheles farauti	146.583298	-8.6333	Unclassified	GBIF: https://doi.org/10.15468/dl.zfaw79
Anopheles farauti	146.5833	-8.6333	Unclassified	GBIF: https://doi.org/10.15468/dl.zfaw79
Anopheles farauti	146.583328	-8.5	Unclassified	GBIF: https://doi.org/10.15468/dl.zfaw79
Anopheles farauti	146.58333	-8.5	Unclassified	GBIF: https://doi.org/10.15468/dl.zfaw79
Anopheles farauti	146.604908	-19.156229	Unclassified	GBIF: https://doi.org/10.15468/dl.zfaw79
Anopheles farauti	146.733307	-19.450001	Unclassified	GBIF: https://doi.org/10.15468/dl.zfaw79
Anopheles farauti	146.73331	-19.45	Unclassified	GBIF: https://doi.org/10.15468/dl.zfaw79
Anopheles farauti	146.816666	-19.266666	Unclassified	GBIF: https://doi.org/10.15468/dl.zfaw79
Anopheles farauti	146.81667	-19.26667	Unclassified	GBIF: https://doi.org/10.15468/dl.zfaw79
Anopheles farauti	146.833328	-2.083333	Unclassified	GBIF: https://doi.org/10.15468/dl.zfaw79
Anopheles farauti	146.83333	-2.08333	Unclassified	GBIF: https://doi.org/10.15468/dl.zfaw79
Anopheles farauti	147	-6.716667	Unclassified	GBIF: https://doi.org/10.15468/dl.zfaw79
Anopheles farauti	147	-6.71667	Unclassified	GBIF: https://doi.org/10.15468/dl.zfaw79
Anopheles farauti	147.16667	-9.25	Unclassified	GBIF: https://doi.org/10.15468/dl.zfaw79
Anopheles farauti	147.166672	-9.25	Unclassified	GBIF: https://doi.org/10.15468/dl.zfaw79
Anopheles farauti	147.841512	-6.600603	Unclassified	GBIF: https://doi.org/10.15468/dl.zfaw79
Anopheles farauti	147.850202	-6.598758	Unclassified	GBIF: https://doi.org/10.15468/dl.zfaw79
Anopheles farauti	148.066666	-8.816667	Unclassified	GBIF: https://doi.org/10.15468/dl.zfaw79
Anopheles farauti	148.06667	-8.81667	Unclassified	GBIF: https://doi.org/10.15468/dl.zfaw79
Anopheles farauti	148.25	-8.75	Unclassified	GBIF: https://doi.org/10.15468/dl.zfaw79
Anopheles farauti	149.91667	-1.66667	Unclassified	GBIF: https://doi.org/10.15468/dl.zfaw79
Anopheles farauti	149.916672	-1.666667	Unclassified	GBIF: https://doi.org/10.15468/dl.zfaw79
Anopheles farauti	150.25	-9.333333	Unclassified	GBIF: https://doi.org/10.15468/dl.zfaw79
Anopheles farauti	150.25	-9.33333	Unclassified	GBIF: https://doi.org/10.15468/dl.zfaw79
Anopheles farauti	150.333328	-10.333333	Unclassified	GBIF: https://doi.org/10.15468/dl.zfaw79
Anopheles farauti	150.33333	-10.33333	Unclassified	GBIF: https://doi.org/10.15468/dl.zfaw79
Anopheles farauti	150.63333	-26.73333	Unclassified	GBIF: https://doi.org/10.15468/dl.zfaw79

Anopheles farauti	150.633331	-26.733334	Unclassified	GBIF: https://doi.org/10.15468/dl.zfaw79
Anopheles farauti	150.701933	-10.410705	Unclassified	GBIF: https://doi.org/10.15468/dl.zfaw79
Anopheles farauti	150.733307	-9.733334	Unclassified	GBIF: https://doi.org/10.15468/dl.zfaw79
Anopheles farauti	150.73331	-9.73333	Unclassified	GBIF: https://doi.org/10.15468/dl.zfaw79
Anopheles farauti	150.75	-2.583333	Unclassified	GBIF: https://doi.org/10.15468/dl.zfaw79
Anopheles farauti	150.8	-2.567	Unclassified	GBIF: https://doi.org/10.15468/dl.zfaw79
Anopheles farauti	151.75	-3.166667	Unclassified	GBIF: https://doi.org/10.15468/dl.zfaw79
Anopheles farauti	151.75	-3.16667	Unclassified	GBIF: https://doi.org/10.15468/dl.zfaw79
Anopheles farauti	152.066696	-4.58	Unclassified	GBIF: https://doi.org/10.15468/dl.zfaw79
Anopheles farauti	152.0667	-4.58	Unclassified	GBIF: https://doi.org/10.15468/dl.zfaw79
Anopheles farauti	152.16667	-4.16667	Unclassified	GBIF: https://doi.org/10.15468/dl.zfaw79
Anopheles farauti	152.166672	-4.166667	Unclassified	GBIF: https://doi.org/10.15468/dl.zfaw79
Anopheles farauti	152.183333	-4.216667	Unclassified	GBIF: https://doi.org/10.15468/dl.zfaw79
Anopheles farauti	152.3833	-4.3667	Unclassified	GBIF: https://doi.org/10.15468/dl.zfaw79
Anopheles farauti	152.383301	-4.3667	Unclassified	GBIF: https://doi.org/10.15468/dl.zfaw79
Anopheles farauti	152.75	-10.666667	Unclassified	GBIF: https://doi.org/10.15468/dl.zfaw79
Anopheles farauti	152.75	-10.66667	Unclassified	GBIF: https://doi.org/10.15468/dl.zfaw79
Anopheles farauti	154.25	-4.5	Unclassified	GBIF: https://doi.org/10.15468/dl.zfaw79
Anopheles farauti	154.66667	-5.5	Unclassified	GBIF: https://doi.org/10.15468/dl.zfaw79
Anopheles farauti	154.666672	-5.5	Unclassified	GBIF: https://doi.org/10.15468/dl.zfaw79
Anopheles farauti	155.26669	-5.86667	Unclassified	GBIF: https://doi.org/10.15468/dl.zfaw79
Anopheles farauti	155.266693	-5.866667	Unclassified	GBIF: https://doi.org/10.15468/dl.zfaw79
Anopheles farauti	155.63333	-6.21667	Unclassified	GBIF: https://doi.org/10.15468/dl.zfaw79
Anopheles farauti	155.633331	-6.216667	Unclassified	GBIF: https://doi.org/10.15468/dl.zfaw79
Anopheles farauti	158	-8	Unclassified	GBIF: https://doi.org/10.15468/dl.zfaw79
Anopheles farauti	159.83	-9.37	Unclassified	GBIF: https://doi.org/10.15468/dl.zfaw79
Anopheles farauti	159.88	-9.38	Unclassified	GBIF: https://doi.org/10.15468/dl.zfaw79
Anopheles farauti	159.8833	-9.4	Unclassified	GBIF: https://doi.org/10.15468/dl.zfaw79
Anopheles farauti	159.883301	-9.4	Unclassified	GBIF: https://doi.org/10.15468/dl.zfaw79
Anopheles farauti	159.8846	-9.42	Unclassified	GBIF: https://doi.org/10.15468/dl.zfaw79
Anopheles farauti	160.18	-9.62	Unclassified	GBIF: https://doi.org/10.15468/dl.zfaw79
Anopheles farauti	160.2	-9.6	Unclassified	GBIF: https://doi.org/10.15468/dl.zfaw79
Anopheles farauti	160.2	-9.533333	Unclassified	GBIF: https://doi.org/10.15468/dl.zfaw79
Anopheles farauti	160.2	-9.5333	Unclassified	GBIF: https://doi.org/10.15468/dl.zfaw79
Anopheles farauti	166.833333	-15.25	Unclassified	GBIF: https://doi.org/10.15468/dl.zfaw79
Anopheles farauti	166.841306	-15.247364	Unclassified	GBIF: https://doi.org/10.15468/dl.zfaw79
Anopheles farauti	166.9167	-15.51667	Unclassified	GBIF: https://doi.org/10.15468/dl.zfaw79
Anopheles farauti	166.916702	-15.51667	Unclassified	GBIF: https://doi.org/10.15468/dl.zfaw79
Anopheles farauti	167.100006	-15.4	Unclassified	GBIF: https://doi.org/10.15468/dl.zfaw79
Anopheles farauti	167.10001	-15.4	Unclassified	GBIF: https://doi.org/10.15468/dl.zfaw79
Anopheles farauti	167.14999	-15.55	Unclassified	GBIF: https://doi.org/10.15468/dl.zfaw79
Anopheles farauti	167.149994	-15.55	Unclassified	GBIF: https://doi.org/10.15468/dl.zfaw79

Anopheles farauti	168.25	-16.683331	Unclassified	GBIF: https://doi.org/10.15468/dl.zfaw79
Anopheles farauti	168.25	-16.68333	Unclassified	GBIF: https://doi.org/10.15468/dl.zfaw79
Anopheles farauti	168.4252	-17.652	Unclassified	GBIF: https://doi.org/10.15468/dl.zfaw79
Anopheles farauti	168.51669	-17.66667	Unclassified	GBIF: https://doi.org/10.15468/dl.zfaw79
Anopheles farauti	168.516693	-17.66667	Unclassified	GBIF: https://doi.org/10.15468/dl.zfaw79
Anopheles farauti	169.733337	-20.233334	Unclassified	GBIF: https://doi.org/10.15468/dl.zfaw79
Anopheles farauti	169.767	-20.23333	Unclassified	GBIF: https://doi.org/10.15468/dl.zfaw79
Anopheles fausti	-110.856389	30.778056	Unclassified	GBIF: https://doi.org/10.15468/dl.zfaw79
Anopheles fausti	-96.340833	17.465278	Unclassified	GBIF: https://doi.org/10.15468/dl.zfaw79
Anopheles filipinae	120.40565	16.240515	Unclassified	GBIF: https://doi.org/10.15468/dl.zfaw79
Anopheles flavicosta	-4.52002	11.236	Unclassified	GBIF: https://doi.org/10.15468/dl.zfaw79
Anopheles flavicosta	-4.50953	11.2379	Unclassified	GBIF: https://doi.org/10.15468/dl.zfaw79
Anopheles flavicosta	-4.50952	11.2384	Unclassified	GBIF: https://doi.org/10.15468/dl.zfaw79
Anopheles flavicosta	-4.49909	11.2396	Unclassified	GBIF: https://doi.org/10.15468/dl.zfaw79
Anopheles flavicosta	4.18254	12.46661	Unclassified	GBIF: https://doi.org/10.15468/dl.zfaw79
Anopheles flavicosta	5.0659	9.30023	Unclassified	GBIF: https://doi.org/10.15468/dl.zfaw79
Anopheles flavicosta	6.16591	9.81685	Unclassified	GBIF: https://doi.org/10.15468/dl.zfaw79
Anopheles flavicosta	6.38259	8.58363	Unclassified	GBIF: https://doi.org/10.15468/dl.zfaw79
Anopheles flavicosta	6.416667	8.6	Unclassified	GBIF: https://doi.org/10.15468/dl.zfaw79
Anopheles flavicosta	6.73259	7.80037	Unclassified	GBIF: https://doi.org/10.15468/dl.zfaw79
Anopheles flavicosta	7.44927	10.50013	Unclassified	GBIF: https://doi.org/10.15468/dl.zfaw79
Anopheles flavicosta	7.4826	10.63345	Unclassified	GBIF: https://doi.org/10.15468/dl.zfaw79
Anopheles flavicosta	7.54928	6.4505	Unclassified	GBIF: https://doi.org/10.15468/dl.zfaw79
Anopheles flavicosta	7.86594	8.83361	Unclassified	GBIF: https://doi.org/10.15468/dl.zfaw79
Anopheles flavicosta	8.61596	7.68372	Unclassified	GBIF: https://doi.org/10.15468/dl.zfaw79
Anopheles flavicosta	10.33264	12.29997	Unclassified	GBIF: https://doi.org/10.15468/dl.zfaw79
Anopheles flavicosta	12.04935	9.4669	Unclassified	GBIF: https://doi.org/10.15468/dl.zfaw79
Anopheles flavirostris	120.40565	16.240515	Unclassified	GBIF: https://doi.org/10.15468/dl.zfaw79
Anopheles flavirostris	120.420556	16.21	Unclassified	GBIF: https://doi.org/10.15468/dl.zfaw79
Anopheles flavirostris	121.065918	12.347433	Unclassified	GBIF: https://doi.org/10.15468/dl.zfaw79
Anopheles flavirostris	121.067613	12.352748	Unclassified	GBIF: https://doi.org/10.15468/dl.zfaw79
Anopheles flavirostris	124.751667	11.245556	Unclassified	GBIF: https://doi.org/10.15468/dl.zfaw79
Anopheles flavirostris	124.757122	11.243803	Unclassified	GBIF: https://doi.org/10.15468/dl.zfaw79
Anopheles flavirostris	124.883694	10.138439	Unclassified	GBIF: https://doi.org/10.15468/dl.zfaw79
Anopheles flavirostris	124.884981	10.138764	Unclassified	GBIF: https://doi.org/10.15468/dl.zfaw79
Anopheles fluminensis	-76.163853	-0.634005	Unclassified	GBIF: https://doi.org/10.15468/dl.zfaw79
Anopheles fluminensis	-76.160425	-0.637495	Unclassified	GBIF: https://doi.org/10.15468/dl.zfaw79
Anopheles fluminensis	-76.150089	-0.637087	Unclassified	GBIF: https://doi.org/10.15468/dl.zfaw79
Anopheles fluminensis	-76.147785	-0.625633	Unclassified	GBIF: https://doi.org/10.15468/dl.zfaw79
Anopheles fluminensis	-76.147343	-0.63753	Unclassified	GBIF: https://doi.org/10.15468/dl.zfaw79
Anopheles fluminensis	-76.145528	-0.630164	Unclassified	GBIF: https://doi.org/10.15468/dl.zfaw79
Anopheles fluminensis	-76.145504	-0.630179	Unclassified	GBIF: https://doi.org/10.15468/dl.zfaw79

Anopheles fluminensis	-76.145393	-0.630607	Unclassified	GBIF: https://doi.org/10.15468/dl.zfaw79
Anopheles fluminensis	-72.68	-7.77	Unclassified	GBIF: https://doi.org/10.15468/dl.zfaw79
Anopheles fluminensis	-66.86	-9.95	Unclassified	GBIF: https://doi.org/10.15468/dl.zfaw79
Anopheles fluminensis	-66.84	-10	Unclassified	GBIF: https://doi.org/10.15468/dl.zfaw79
Anopheles fluminensis	-66.82	-10.01	Unclassified	GBIF: https://doi.org/10.15468/dl.zfaw79
Anopheles fluminensis	-63.89	3.28	Unclassified	GBIF: https://doi.org/10.15468/dl.zfaw79
Anopheles fluminensis	-63.77	3.39	Unclassified	GBIF: https://doi.org/10.15468/dl.zfaw79
Anopheles fluminensis	-61.297045	10.38824	Unclassified	GBIF: https://doi.org/10.15468/dl.zfaw79
Anopheles fluminensis	-61.175559	10.305938	Unclassified	GBIF: https://doi.org/10.15468/dl.zfaw79
Anopheles fluminensis	-61.075996	10.423877	Unclassified	GBIF: https://doi.org/10.15468/dl.zfaw79
Anopheles fluminensis	-53.860556	-27.265	Unclassified	GBIF: https://doi.org/10.15468/dl.zfaw79
Anopheles fluminensis	-52.2333	-23.55	Unclassified	GBIF: https://doi.org/10.15468/dl.zfaw79
Anopheles fluminensis	-51.05	-23.2833	Unclassified	GBIF: https://doi.org/10.15468/dl.zfaw79
Anopheles fluminensis	-48.8293	-25.4754	Unclassified	GBIF: https://doi.org/10.15468/dl.zfaw79
Anopheles fluminensis	-41.899998	-21.2	Unclassified	GBIF: https://doi.org/10.15468/dl.zfaw79
Anopheles fluviatilis	81.9	18.4	Unclassified	GBIF: https://doi.org/10.15468/dl.zfaw79
Anopheles fluviatilis	82.7	18.8	Unclassified	GBIF: https://doi.org/10.15468/dl.zfaw79
Anopheles fluviatilis	83	19.75	Unclassified	GBIF: https://doi.org/10.15468/dl.zfaw79
Anopheles fluviatilis	84	22.1	Unclassified	GBIF: https://doi.org/10.15468/dl.zfaw79
Anopheles fluviatilis	84.2333	20.4667	Unclassified	GBIF: https://doi.org/10.15468/dl.zfaw79
Anopheles fluviatilis	84.826	22.035	Unclassified	GBIF: https://doi.org/10.15468/dl.zfaw79
Anopheles fluviatilis	84.8263	22.0342	Unclassified	GBIF: https://doi.org/10.15468/dl.zfaw79
Anopheles fluviatilis	84.827	22.0362	Unclassified	GBIF: https://doi.org/10.15468/dl.zfaw79
Anopheles fluviatilis	84.8271	22.0345	Unclassified	GBIF: https://doi.org/10.15468/dl.zfaw79
Anopheles fluviatilis	84.9713	22.2552	Unclassified	GBIF: https://doi.org/10.15468/dl.zfaw79
Anopheles fluviatilis	84.9724	22.2144	Unclassified	GBIF: https://doi.org/10.15468/dl.zfaw79
Anopheles fluviatilis	84.9724	22.2554	Unclassified	GBIF: https://doi.org/10.15468/dl.zfaw79
Anopheles fluviatilis	84.9725	22.2557	Unclassified	GBIF: https://doi.org/10.15468/dl.zfaw79
Anopheles fluviatilis	84.9726	22.2556	Unclassified	GBIF: https://doi.org/10.15468/dl.zfaw79
Anopheles fluviatilis	84.9735	22.2092	Unclassified	GBIF: https://doi.org/10.15468/dl.zfaw79
Anopheles fluviatilis	84.9736	22.208	Unclassified	GBIF: https://doi.org/10.15468/dl.zfaw79
Anopheles fluviatilis	84.9737	22.2085	Unclassified	GBIF: https://doi.org/10.15468/dl.zfaw79
Anopheles fluviatilis	84.9739	22.2097	Unclassified	GBIF: https://doi.org/10.15468/dl.zfaw79
Anopheles fluviatilis	85.1	20.85	Unclassified	GBIF: https://doi.org/10.15468/dl.zfaw79
Anopheles fluviatilis	85.48	21.56	Unclassified	GBIF: https://doi.org/10.15468/dl.zfaw79
Anopheles fluviatilis	85.5	21.5	Unclassified	GBIF: https://doi.org/10.15468/dl.zfaw79
Anopheles fluviatilis	120.40565	16.240515	Unclassified	GBIF: https://doi.org/10.15468/dl.zfaw79
Anopheles fontenillei	11.59	-0.17	Unclassified	GBIF: https://doi.org/10.15468/dl.zfaw79
Anopheles fontenillei	11.60041	-0.19773	Unclassified	GBIF: https://doi.org/10.15468/dl.zfaw79
Anopheles fontenillei	11.60197	-0.20336	Unclassified	GBIF: https://doi.org/10.15468/dl.zfaw79
Anopheles fontenillei	11.60281	-0.20356	Unclassified	GBIF: https://doi.org/10.15468/dl.zfaw79
Anopheles fontenillei	11.98	-0.28	Unclassified	GBIF: https://doi.org/10.15468/dl.zfaw79

Anopheles forattinii	-76.15	-0.63	Unclassified	GBIF: https://doi.org/10.15468/dl.zfaw79
Anopheles forattinii	-72.64	-7.33	Unclassified	GBIF: https://doi.org/10.15468/dl.zfaw79
Anopheles forattinii	-72.63879	-7.32804	Unclassified	GBIF: https://doi.org/10.15468/dl.zfaw79
Anopheles forattinii	-64.26667	-12.453432	Unclassified	GBIF: https://doi.org/10.15468/dl.zfaw79
Anopheles forattinii	-53.24036	3.91451	Unclassified	GBIF: https://doi.org/10.15468/dl.zfaw79
Anopheles forattinii	-53.23477	3.92404	Unclassified	GBIF: https://doi.org/10.15468/dl.zfaw79
Anopheles fragilis	99.76886	8.14009	Unclassified	GBIF: https://doi.org/10.15468/dl.zfaw79
Anopheles fragilis	101.486	3.841183	Unclassified	GBIF: https://doi.org/10.15468/dl.zfaw79
Anopheles fragilis	102.342697	12.023555	Unclassified	GBIF: https://doi.org/10.15468/dl.zfaw79
Anopheles fragilis	102.666441	12.187784	Unclassified	GBIF: https://doi.org/10.15468/dl.zfaw79
Anopheles fragilis	103.81026	1.352825	Unclassified	GBIF: https://doi.org/10.15468/dl.zfaw79
Anopheles franciscanus	-123.752245	39.896539	Unclassified	GBIF: https://doi.org/10.15468/dl.zfaw79
Anopheles franciscanus	-123.4903	38.9159	Unclassified	GBIF: https://doi.org/10.15468/dl.zfaw79
Anopheles franciscanus	-123.478	38.9176	Unclassified	GBIF: https://doi.org/10.15468/dl.zfaw79
Anopheles franciscanus	-122.782123	38.611756	Unclassified	GBIF: https://doi.org/10.15468/dl.zfaw79
Anopheles franciscanus	-122.782044	38.611987	Unclassified	GBIF: https://doi.org/10.15468/dl.zfaw79
Anopheles franciscanus	-122.781937	38.611966	Unclassified	GBIF: https://doi.org/10.15468/dl.zfaw79
Anopheles franciscanus	-122.640236	38.93574	Unclassified	GBIF: https://doi.org/10.15468/dl.zfaw79
Anopheles franciscanus	-122.492335	37.619973	Unclassified	GBIF: https://doi.org/10.15468/dl.zfaw79
Anopheles franciscanus	-122.451091	38.581296	Unclassified	GBIF: https://doi.org/10.15468/dl.zfaw79
Anopheles franciscanus	-122.272535	37.775519	Unclassified	GBIF: https://doi.org/10.15468/dl.zfaw79
Anopheles franciscanus	-122.238577	40.180587	Unclassified	GBIF: https://doi.org/10.15468/dl.zfaw79
Anopheles franciscanus	-121.997031	45.827907	Unclassified	GBIF: https://doi.org/10.15468/dl.zfaw79
Anopheles franciscanus	-121.983875	39.759103	Unclassified	GBIF: https://doi.org/10.15468/dl.zfaw79
Anopheles franciscanus	-121.962181	37.760761	Unclassified	GBIF: https://doi.org/10.15468/dl.zfaw79
Anopheles franciscanus	-121.930021	37.394318	Unclassified	GBIF: https://doi.org/10.15468/dl.zfaw79
Anopheles franciscanus	-121.893611	36.603056	Unclassified	GBIF: https://doi.org/10.15468/dl.zfaw79
Anopheles franciscanus	-121.820389	36.31229	Unclassified	GBIF: https://doi.org/10.15468/dl.zfaw79
Anopheles franciscanus	-121.812149	36.333312	Unclassified	GBIF: https://doi.org/10.15468/dl.zfaw79
Anopheles franciscanus	-121.773333	38.678611	Unclassified	GBIF: https://doi.org/10.15468/dl.zfaw79
Anopheles franciscanus	-121.201476	36.353278	Unclassified	GBIF: https://doi.org/10.15468/dl.zfaw79
Anopheles franciscanus	-121.144413	36.497087	Unclassified	GBIF: https://doi.org/10.15468/dl.zfaw79
Anopheles franciscanus	-120.487088	37.29834	Unclassified	GBIF: https://doi.org/10.15468/dl.zfaw79
Anopheles franciscanus	-120.094936	37.403228	Unclassified	GBIF: https://doi.org/10.15468/dl.zfaw79
Anopheles franciscanus	-120.094872	37.403227	Unclassified	GBIF: https://doi.org/10.15468/dl.zfaw79
Anopheles franciscanus	-120.094846	37.403275	Unclassified	GBIF: https://doi.org/10.15468/dl.zfaw79
Anopheles franciscanus	-120.09465	37.402942	Unclassified	GBIF: https://doi.org/10.15468/dl.zfaw79
Anopheles franciscanus	-119.75	37.109	Unclassified	GBIF: https://doi.org/10.15468/dl.zfaw79
Anopheles franciscanus	-119.749511	37.109009	Unclassified	GBIF: https://doi.org/10.15468/dl.zfaw79
Anopheles franciscanus	-119.7411	37.082013	Unclassified	GBIF: https://doi.org/10.15468/dl.zfaw79
Anopheles franciscanus	-119.741	37.082	Unclassified	GBIF: https://doi.org/10.15468/dl.zfaw79
Anopheles franciscanus	-119.73524	37.096437	Unclassified	GBIF: https://doi.org/10.15468/dl.zfaw79

Anopheles franciscanus	-119.735	37.096	Unclassified	GBIF: https://doi.org/10.15468/dl.zfaw79
Anopheles franciscanus	-119.733	37.087	Unclassified	GBIF: https://doi.org/10.15468/dl.zfaw79
Anopheles franciscanus	-119.732525	37.086793	Unclassified	GBIF: https://doi.org/10.15468/dl.zfaw79
Anopheles franciscanus	-119.73228	37.10878	Unclassified	GBIF: https://doi.org/10.15468/dl.zfaw79
Anopheles franciscanus	-119.728	37.082	Unclassified	GBIF: https://doi.org/10.15468/dl.zfaw79
Anopheles franciscanus	-119.727648	37.081507	Unclassified	GBIF: https://doi.org/10.15468/dl.zfaw79
Anopheles franciscanus	-119.726034	37.092865	Unclassified	GBIF: https://doi.org/10.15468/dl.zfaw79
Anopheles franciscanus	-119.726	37.093	Unclassified	GBIF: https://doi.org/10.15468/dl.zfaw79
Anopheles franciscanus	-119.724	37.09	Unclassified	GBIF: https://doi.org/10.15468/dl.zfaw79
Anopheles franciscanus	-119.723575	37.089955	Unclassified	GBIF: https://doi.org/10.15468/dl.zfaw79
Anopheles franciscanus	-119.723127	37.085395	Unclassified	GBIF: https://doi.org/10.15468/dl.zfaw79
Anopheles franciscanus	-119.723	37.085	Unclassified	GBIF: https://doi.org/10.15468/dl.zfaw79
Anopheles franciscanus	-119.721285	37.101331	Unclassified	GBIF: https://doi.org/10.15468/dl.zfaw79
Anopheles franciscanus	-119.721	37.101	Unclassified	GBIF: https://doi.org/10.15468/dl.zfaw79
Anopheles franciscanus	-119.273022	37.031666	Unclassified	GBIF: https://doi.org/10.15468/dl.zfaw79
Anopheles franciscanus	-119.273	37.032	Unclassified	GBIF: https://doi.org/10.15468/dl.zfaw79
Anopheles franciscanus	-119.266198	37.028829	Unclassified	GBIF: https://doi.org/10.15468/dl.zfaw79
Anopheles franciscanus	-119.266	37.029	Unclassified	GBIF: https://doi.org/10.15468/dl.zfaw79
Anopheles franciscanus	-119.26219	37.03337	Unclassified	GBIF: https://doi.org/10.15468/dl.zfaw79
Anopheles franciscanus	-119.262184	37.031342	Unclassified	GBIF: https://doi.org/10.15468/dl.zfaw79
Anopheles franciscanus	-119.262	37.031	Unclassified	GBIF: https://doi.org/10.15468/dl.zfaw79
Anopheles franciscanus	-119.251043	37.031894	Unclassified	GBIF: https://doi.org/10.15468/dl.zfaw79
Anopheles franciscanus	-119.251	37.032	Unclassified	GBIF: https://doi.org/10.15468/dl.zfaw79
Anopheles franciscanus	-119.247486	37.034666	Unclassified	GBIF: https://doi.org/10.15468/dl.zfaw79
Anopheles franciscanus	-119.247	37.035	Unclassified	GBIF: https://doi.org/10.15468/dl.zfaw79
Anopheles franciscanus	-119.149829	34.283058	Unclassified	GBIF: https://doi.org/10.15468/dl.zfaw79
Anopheles franciscanus	-119.018712	35.37315	Unclassified	GBIF: https://doi.org/10.15468/dl.zfaw79
Anopheles franciscanus	-119.013482	36.980194	Unclassified	GBIF: https://doi.org/10.15468/dl.zfaw79
Anopheles franciscanus	-119.00602	37.00583	Unclassified	GBIF: https://doi.org/10.15468/dl.zfaw79
Anopheles franciscanus	-118.615634	34.486632	Unclassified	GBIF: https://doi.org/10.15468/dl.zfaw79
Anopheles franciscanus	-118.613559	34.250982	Unclassified	GBIF: https://doi.org/10.15468/dl.zfaw79
Anopheles franciscanus	-118.452139	33.401173	Unclassified	GBIF: https://doi.org/10.15468/dl.zfaw79
Anopheles franciscanus	-118.11242	34.550016	Unclassified	GBIF: https://doi.org/10.15468/dl.zfaw79
Anopheles franciscanus	-117.70139	33.758165	Unclassified	GBIF: https://doi.org/10.15468/dl.zfaw79
Anopheles franciscanus	-117.701368	33.758032	Unclassified	GBIF: https://doi.org/10.15468/dl.zfaw79
Anopheles franciscanus	-117.701195	33.75702	Unclassified	GBIF: https://doi.org/10.15468/dl.zfaw79
Anopheles franciscanus	-117.652	33.927	Unclassified	GBIF: https://doi.org/10.15468/dl.zfaw79
Anopheles franciscanus	-117.635	33.912	Unclassified	GBIF: https://doi.org/10.15468/dl.zfaw79
Anopheles franciscanus	-117.632	33.9183	Unclassified	GBIF: https://doi.org/10.15468/dl.zfaw79
Anopheles franciscanus	-117.626	33.9143	Unclassified	GBIF: https://doi.org/10.15468/dl.zfaw79
Anopheles franciscanus	-117.625	33.9177	Unclassified	GBIF: https://doi.org/10.15468/dl.zfaw79
Anopheles franciscanus	-117.62	33.9231	Unclassified	GBIF: https://doi.org/10.15468/dl.zfaw79

Anopheles franciscanus	-117.62	33.918	Unclassified	GBIF: https://doi.org/10.15468/dl.zfaw79
Anopheles franciscanus	-117.619	33.8862	Unclassified	GBIF: https://doi.org/10.15468/dl.zfaw79
Anopheles franciscanus	-117.609	33.8621	Unclassified	GBIF: https://doi.org/10.15468/dl.zfaw79
Anopheles franciscanus	-117.608	33.855	Unclassified	GBIF: https://doi.org/10.15468/dl.zfaw79
Anopheles franciscanus	-117.606	33.9099	Unclassified	GBIF: https://doi.org/10.15468/dl.zfaw79
Anopheles franciscanus	-117.606	33.8694	Unclassified	GBIF: https://doi.org/10.15468/dl.zfaw79
Anopheles franciscanus	-117.604	33.94	Unclassified	GBIF: https://doi.org/10.15468/dl.zfaw79
Anopheles franciscanus	-117.602	33.8934	Unclassified	GBIF: https://doi.org/10.15468/dl.zfaw79
Anopheles franciscanus	-117.601	33.9285	Unclassified	GBIF: https://doi.org/10.15468/dl.zfaw79
Anopheles franciscanus	-117.601	33.8923	Unclassified	GBIF: https://doi.org/10.15468/dl.zfaw79
Anopheles franciscanus	-117.601	33.9267	Unclassified	GBIF: https://doi.org/10.15468/dl.zfaw79
Anopheles franciscanus	-117.599	33.868	Unclassified	GBIF: https://doi.org/10.15468/dl.zfaw79
Anopheles franciscanus	-117.598	33.8953	Unclassified	GBIF: https://doi.org/10.15468/dl.zfaw79
Anopheles franciscanus	-117.596	33.8989	Unclassified	GBIF: https://doi.org/10.15468/dl.zfaw79
Anopheles franciscanus	-117.596	33.929	Unclassified	GBIF: https://doi.org/10.15468/dl.zfaw79
Anopheles franciscanus	-117.593	33.9324	Unclassified	GBIF: https://doi.org/10.15468/dl.zfaw79
Anopheles franciscanus	-117.589	33.8433	Unclassified	GBIF: https://doi.org/10.15468/dl.zfaw79
Anopheles franciscanus	-117.587	33.9024	Unclassified	GBIF: https://doi.org/10.15468/dl.zfaw79
Anopheles franciscanus	-117.582	33.9429	Unclassified	GBIF: https://doi.org/10.15468/dl.zfaw79
Anopheles franciscanus	-117.578	33.9481	Unclassified	GBIF: https://doi.org/10.15468/dl.zfaw79
Anopheles franciscanus	-117.577	33.9229	Unclassified	GBIF: https://doi.org/10.15468/dl.zfaw79
Anopheles franciscanus	-117.576	33.9248	Unclassified	GBIF: https://doi.org/10.15468/dl.zfaw79
Anopheles franciscanus	-117.576	33.8394	Unclassified	GBIF: https://doi.org/10.15468/dl.zfaw79
Anopheles franciscanus	-117.576	33.8916	Unclassified	GBIF: https://doi.org/10.15468/dl.zfaw79
Anopheles franciscanus	-117.568	33.9403	Unclassified	GBIF: https://doi.org/10.15468/dl.zfaw79
Anopheles franciscanus	-117.565	33.8231	Unclassified	GBIF: https://doi.org/10.15468/dl.zfaw79
Anopheles franciscanus	-117.551	33.9145	Unclassified	GBIF: https://doi.org/10.15468/dl.zfaw79
Anopheles franciscanus	-117.545	33.9012	Unclassified	GBIF: https://doi.org/10.15468/dl.zfaw79
Anopheles franciscanus	-117.536	33.8602	Unclassified	GBIF: https://doi.org/10.15468/dl.zfaw79
Anopheles franciscanus	-117.533	33.8588	Unclassified	GBIF: https://doi.org/10.15468/dl.zfaw79
Anopheles franciscanus	-117.523	33.968	Unclassified	GBIF: https://doi.org/10.15468/dl.zfaw79
Anopheles franciscanus	-117.496	33.9643	Unclassified	GBIF: https://doi.org/10.15468/dl.zfaw79
Anopheles franciscanus	-117.426	33.976	Unclassified	GBIF: https://doi.org/10.15468/dl.zfaw79
Anopheles franciscanus	-117.421	33.9652	Unclassified	GBIF: https://doi.org/10.15468/dl.zfaw79
Anopheles franciscanus	-117.417	33.982	Unclassified	GBIF: https://doi.org/10.15468/dl.zfaw79
Anopheles franciscanus	-117.397	33.6921	Unclassified	GBIF: https://doi.org/10.15468/dl.zfaw79
Anopheles franciscanus	-117.395	33.9733	Unclassified	GBIF: https://doi.org/10.15468/dl.zfaw79
Anopheles franciscanus	-117.387	33.7076	Unclassified	GBIF: https://doi.org/10.15468/dl.zfaw79
Anopheles franciscanus	-117.386	33.9396	Unclassified	GBIF: https://doi.org/10.15468/dl.zfaw79
Anopheles franciscanus	-117.358	33.7016	Unclassified	GBIF: https://doi.org/10.15468/dl.zfaw79
Anopheles franciscanus	-117.356	33.6977	Unclassified	GBIF: https://doi.org/10.15468/dl.zfaw79
Anopheles franciscanus	-117.355	33.6982	Unclassified	GBIF: https://doi.org/10.15468/dl.zfaw79

Anopheles franciscanus	-117.352	33.6928	Unclassified	GBIF: https://doi.org/10.15468/dl.zfaw79
Anopheles franciscanus	-117.351	33.6944	Unclassified	GBIF: https://doi.org/10.15468/dl.zfaw79
Anopheles franciscanus	-117.35	33.6986	Unclassified	GBIF: https://doi.org/10.15468/dl.zfaw79
Anopheles franciscanus	-117.344	33.6828	Unclassified	GBIF: https://doi.org/10.15468/dl.zfaw79
Anopheles franciscanus	-117.342	33.6819	Unclassified	GBIF: https://doi.org/10.15468/dl.zfaw79
Anopheles franciscanus	-117.323	33.8764	Unclassified	GBIF: https://doi.org/10.15468/dl.zfaw79
Anopheles franciscanus	-117.318	33.6315	Unclassified	GBIF: https://doi.org/10.15468/dl.zfaw79
Anopheles franciscanus	-117.308	33.6222	Unclassified	GBIF: https://doi.org/10.15468/dl.zfaw79
Anopheles franciscanus	-117.307	33.6491	Unclassified	GBIF: https://doi.org/10.15468/dl.zfaw79
Anopheles franciscanus	-117.291	33.6709	Unclassified	GBIF: https://doi.org/10.15468/dl.zfaw79
Anopheles franciscanus	-117.271	33.6941	Unclassified	GBIF: https://doi.org/10.15468/dl.zfaw79
Anopheles franciscanus	-117.271	33.672	Unclassified	GBIF: https://doi.org/10.15468/dl.zfaw79
Anopheles franciscanus	-117.248	33.6597	Unclassified	GBIF: https://doi.org/10.15468/dl.zfaw79
Anopheles franciscanus	-117.03	33.16	Unclassified	GBIF: https://doi.org/10.15468/dl.zfaw79
Anopheles franciscanus	-116.757	36.9117	Unclassified	GBIF: https://doi.org/10.15468/dl.zfaw79
Anopheles franciscanus	-116.757	36.9113	Unclassified	GBIF: https://doi.org/10.15468/dl.zfaw79
Anopheles franciscanus	-116.755	36.9091	Unclassified	GBIF: https://doi.org/10.15468/dl.zfaw79
Anopheles franciscanus	-116.754	36.8949	Unclassified	GBIF: https://doi.org/10.15468/dl.zfaw79
Anopheles franciscanus	-116.754	36.8948	Unclassified	GBIF: https://doi.org/10.15468/dl.zfaw79
Anopheles franciscanus	-116.754	36.9145	Unclassified	GBIF: https://doi.org/10.15468/dl.zfaw79
Anopheles franciscanus	-116.754	36.8146	Unclassified	GBIF: https://doi.org/10.15468/dl.zfaw79
Anopheles franciscanus	-116.752	36.915	Unclassified	GBIF: https://doi.org/10.15468/dl.zfaw79
Anopheles franciscanus	-116.751	36.8913	Unclassified	GBIF: https://doi.org/10.15468/dl.zfaw79
Anopheles franciscanus	-116.751	36.8915	Unclassified	GBIF: https://doi.org/10.15468/dl.zfaw79
Anopheles franciscanus	-116.75	36.8912	Unclassified	GBIF: https://doi.org/10.15468/dl.zfaw79
Anopheles franciscanus	-116.717	36.9484	Unclassified	GBIF: https://doi.org/10.15468/dl.zfaw79
Anopheles franciscanus	-116.717	36.9485	Unclassified	GBIF: https://doi.org/10.15468/dl.zfaw79
Anopheles franciscanus	-116.717	36.9483	Unclassified	GBIF: https://doi.org/10.15468/dl.zfaw79
Anopheles franciscanus	-116.452	36.5442	Unclassified	GBIF: https://doi.org/10.15468/dl.zfaw79
Anopheles franciscanus	-116.451	36.5341	Unclassified	GBIF: https://doi.org/10.15468/dl.zfaw79
Anopheles franciscanus	-116.451	36.5452	Unclassified	GBIF: https://doi.org/10.15468/dl.zfaw79
Anopheles franciscanus	-116.451	36.5454	Unclassified	GBIF: https://doi.org/10.15468/dl.zfaw79
Anopheles franciscanus	-116.45	36.5329	Unclassified	GBIF: https://doi.org/10.15468/dl.zfaw79
Anopheles franciscanus	-116.4359	33.1233	Unclassified	GBIF: https://doi.org/10.15468/dl.zfaw79
Anopheles franciscanus	-116.43	36.5654	Unclassified	GBIF: https://doi.org/10.15468/dl.zfaw79
Anopheles franciscanus	-116.42483	31.753655	Unclassified	GBIF: https://doi.org/10.15468/dl.zfaw79
Anopheles franciscanus	-116.40601	31.552385	Unclassified	GBIF: https://doi.org/10.15468/dl.zfaw79
Anopheles franciscanus	-116.353	36.444	Unclassified	GBIF: https://doi.org/10.15468/dl.zfaw79
Anopheles franciscanus	-116.351	36.4449	Unclassified	GBIF: https://doi.org/10.15468/dl.zfaw79
Anopheles franciscanus	-116.329	36.4132	Unclassified	GBIF: https://doi.org/10.15468/dl.zfaw79
Anopheles franciscanus	-116.31	36.4286	Unclassified	GBIF: https://doi.org/10.15468/dl.zfaw79
Anopheles franciscanus	-116.303	36.4028	Unclassified	GBIF: https://doi.org/10.15468/dl.zfaw79

Anopheles franciscanus	-116.302	36.4022	Unclassified	GBIF: https://doi.org/10.15468/dl.zfaw79
Anopheles franciscanus	-116.263	37.1637	Unclassified	GBIF: https://doi.org/10.15468/dl.zfaw79
Anopheles franciscanus	-116.195	36.2514	Unclassified	GBIF: https://doi.org/10.15468/dl.zfaw79
Anopheles franciscanus	-116.194	36.2448	Unclassified	GBIF: https://doi.org/10.15468/dl.zfaw79
Anopheles franciscanus	-116.18	36.241	Unclassified	GBIF: https://doi.org/10.15468/dl.zfaw79
Anopheles franciscanus	-115.99	36.2031	Unclassified	GBIF: https://doi.org/10.15468/dl.zfaw79
Anopheles franciscanus	-115.97	36.801	Unclassified	GBIF: https://doi.org/10.15468/dl.zfaw79
Anopheles franciscanus	-115.906218	32.676345	Unclassified	GBIF: https://doi.org/10.15468/dl.zfaw79
Anopheles franciscanus	-115.642	36.1101	Unclassified	GBIF: https://doi.org/10.15468/dl.zfaw79
Anopheles franciscanus	-115.615	35.1226	Unclassified	GBIF: https://doi.org/10.15468/dl.zfaw79
Anopheles franciscanus	-115.397	36.0484	Unclassified	GBIF: https://doi.org/10.15468/dl.zfaw79
Anopheles franciscanus	-115.394	36.0484	Unclassified	GBIF: https://doi.org/10.15468/dl.zfaw79
Anopheles franciscanus	-115.394	36.0788	Unclassified	GBIF: https://doi.org/10.15468/dl.zfaw79
Anopheles franciscanus	-115.394	36.0489	Unclassified	GBIF: https://doi.org/10.15468/dl.zfaw79
Anopheles franciscanus	-115.272	36.3314	Unclassified	GBIF: https://doi.org/10.15468/dl.zfaw79
Anopheles franciscanus	-115.24	36.0256	Unclassified	GBIF: https://doi.org/10.15468/dl.zfaw79
Anopheles franciscanus	-115.238	36.0254	Unclassified	GBIF: https://doi.org/10.15468/dl.zfaw79
Anopheles franciscanus	-115.235	36.0254	Unclassified	GBIF: https://doi.org/10.15468/dl.zfaw79
Anopheles franciscanus	-115.235	36.0251	Unclassified	GBIF: https://doi.org/10.15468/dl.zfaw79
Anopheles franciscanus	-115.234	36.0256	Unclassified	GBIF: https://doi.org/10.15468/dl.zfaw79
Anopheles franciscanus	-115.234	36.0255	Unclassified	GBIF: https://doi.org/10.15468/dl.zfaw79
Anopheles franciscanus	-115.233	37.5321	Unclassified	GBIF: https://doi.org/10.15468/dl.zfaw79
Anopheles franciscanus	-115.233	37.5326	Unclassified	GBIF: https://doi.org/10.15468/dl.zfaw79
Anopheles franciscanus	-115.221	37.5799	Unclassified	GBIF: https://doi.org/10.15468/dl.zfaw79
Anopheles franciscanus	-115.221	37.5199	Unclassified	GBIF: https://doi.org/10.15468/dl.zfaw79
Anopheles franciscanus	-115.131	37.3118	Unclassified	GBIF: https://doi.org/10.15468/dl.zfaw79
Anopheles franciscanus	-115.126	37.312	Unclassified	GBIF: https://doi.org/10.15468/dl.zfaw79
Anopheles franciscanus	-115.125	37.3109	Unclassified	GBIF: https://doi.org/10.15468/dl.zfaw79
Anopheles franciscanus	-115.125	37.3108	Unclassified	GBIF: https://doi.org/10.15468/dl.zfaw79
Anopheles franciscanus	-115.122	37.2666	Unclassified	GBIF: https://doi.org/10.15468/dl.zfaw79
Anopheles franciscanus	-115.117	37.2751	Unclassified	GBIF: https://doi.org/10.15468/dl.zfaw79
Anopheles franciscanus	-115.117	37.275	Unclassified	GBIF: https://doi.org/10.15468/dl.zfaw79
Anopheles franciscanus	-115.07	37.1629	Unclassified	GBIF: https://doi.org/10.15468/dl.zfaw79
Anopheles franciscanus	-115.054	36.0471	Unclassified	GBIF: https://doi.org/10.15468/dl.zfaw79
Anopheles franciscanus	-115.054	36.0337	Unclassified	GBIF: https://doi.org/10.15468/dl.zfaw79
Anopheles franciscanus	-115.054	36.0364	Unclassified	GBIF: https://doi.org/10.15468/dl.zfaw79
Anopheles franciscanus	-115.054	36.0375	Unclassified	GBIF: https://doi.org/10.15468/dl.zfaw79
Anopheles franciscanus	-115.054	36.0371	Unclassified	GBIF: https://doi.org/10.15468/dl.zfaw79
Anopheles franciscanus	-115.054	36.0818	Unclassified	GBIF: https://doi.org/10.15468/dl.zfaw79
Anopheles franciscanus	-115.053	36.0337	Unclassified	GBIF: https://doi.org/10.15468/dl.zfaw79
Anopheles franciscanus	-115.053	36.0388	Unclassified	GBIF: https://doi.org/10.15468/dl.zfaw79
Anopheles franciscanus	-115.053	36.0386	Unclassified	GBIF: https://doi.org/10.15468/dl.zfaw79

Anopheles franciscanus	-115.052	36.046	Unclassified	GBIF: https://doi.org/10.15468/dl.zfaw79
Anopheles franciscanus	-115.052	36.0318	Unclassified	GBIF: https://doi.org/10.15468/dl.zfaw79
Anopheles franciscanus	-115.052	36.0369	Unclassified	GBIF: https://doi.org/10.15468/dl.zfaw79
Anopheles franciscanus	-115.051	36.0387	Unclassified	GBIF: https://doi.org/10.15468/dl.zfaw79
Anopheles franciscanus	-115.047	36.2164	Unclassified	GBIF: https://doi.org/10.15468/dl.zfaw79
Anopheles franciscanus	-115.038	36.111	Unclassified	GBIF: https://doi.org/10.15468/dl.zfaw79
Anopheles franciscanus	-115.035	36.0756	Unclassified	GBIF: https://doi.org/10.15468/dl.zfaw79
Anopheles franciscanus	-115.033	36.0723	Unclassified	GBIF: https://doi.org/10.15468/dl.zfaw79
Anopheles franciscanus	-115.032	36.0997	Unclassified	GBIF: https://doi.org/10.15468/dl.zfaw79
Anopheles franciscanus	-115.032	36.0742	Unclassified	GBIF: https://doi.org/10.15468/dl.zfaw79
Anopheles franciscanus	-115.031	36.0742	Unclassified	GBIF: https://doi.org/10.15468/dl.zfaw79
Anopheles franciscanus	-115.031	36.0737	Unclassified	GBIF: https://doi.org/10.15468/dl.zfaw79
Anopheles franciscanus	-115.03	36.0742	Unclassified	GBIF: https://doi.org/10.15468/dl.zfaw79
Anopheles franciscanus	-115.029	36.0743	Unclassified	GBIF: https://doi.org/10.15468/dl.zfaw79
Anopheles franciscanus	-115.029	36.0719	Unclassified	GBIF: https://doi.org/10.15468/dl.zfaw79
Anopheles franciscanus	-115.029	36.0713	Unclassified	GBIF: https://doi.org/10.15468/dl.zfaw79
Anopheles franciscanus	-115.026	36.1006	Unclassified	GBIF: https://doi.org/10.15468/dl.zfaw79
Anopheles franciscanus	-115.026	36.1007	Unclassified	GBIF: https://doi.org/10.15468/dl.zfaw79
Anopheles franciscanus	-115.026	36.0741	Unclassified	GBIF: https://doi.org/10.15468/dl.zfaw79
Anopheles franciscanus	-115.025	36.1024	Unclassified	GBIF: https://doi.org/10.15468/dl.zfaw79
Anopheles franciscanus	-115.025	36.1031	Unclassified	GBIF: https://doi.org/10.15468/dl.zfaw79
Anopheles franciscanus	-115.024	36.1062	Unclassified	GBIF: https://doi.org/10.15468/dl.zfaw79
Anopheles franciscanus	-115.024	36.1059	Unclassified	GBIF: https://doi.org/10.15468/dl.zfaw79
Anopheles franciscanus	-115.023	36.1019	Unclassified	GBIF: https://doi.org/10.15468/dl.zfaw79
Anopheles franciscanus	-115.023	36.1018	Unclassified	GBIF: https://doi.org/10.15468/dl.zfaw79
Anopheles franciscanus	-115.023	36.1006	Unclassified	GBIF: https://doi.org/10.15468/dl.zfaw79
Anopheles franciscanus	-115.023	36.0921	Unclassified	GBIF: https://doi.org/10.15468/dl.zfaw79
Anopheles franciscanus	-115.023	36.0986	Unclassified	GBIF: https://doi.org/10.15468/dl.zfaw79
Anopheles franciscanus	-115.022	36.0917	Unclassified	GBIF: https://doi.org/10.15468/dl.zfaw79
Anopheles franciscanus	-115.021	36.092	Unclassified	GBIF: https://doi.org/10.15468/dl.zfaw79
Anopheles franciscanus	-115.021	36.1005	Unclassified	GBIF: https://doi.org/10.15468/dl.zfaw79
Anopheles franciscanus	-115.021	36.1008	Unclassified	GBIF: https://doi.org/10.15468/dl.zfaw79
Anopheles franciscanus	-115.021	36.0924	Unclassified	GBIF: https://doi.org/10.15468/dl.zfaw79
Anopheles franciscanus	-115.021	36.0913	Unclassified	GBIF: https://doi.org/10.15468/dl.zfaw79
Anopheles franciscanus	-115.02	36.1026	Unclassified	GBIF: https://doi.org/10.15468/dl.zfaw79
Anopheles franciscanus	-115.02	36.0976	Unclassified	GBIF: https://doi.org/10.15468/dl.zfaw79
Anopheles franciscanus	-115.02	36.1027	Unclassified	GBIF: https://doi.org/10.15468/dl.zfaw79
Anopheles franciscanus	-115.02	36.0887	Unclassified	GBIF: https://doi.org/10.15468/dl.zfaw79
Anopheles franciscanus	-115.02	36.1039	Unclassified	GBIF: https://doi.org/10.15468/dl.zfaw79
Anopheles franciscanus	-115.02	36.0888	Unclassified	GBIF: https://doi.org/10.15468/dl.zfaw79
Anopheles franciscanus	-115.02	36.104	Unclassified	GBIF: https://doi.org/10.15468/dl.zfaw79
Anopheles franciscanus	-115.019	36.1002	Unclassified	GBIF: https://doi.org/10.15468/dl.zfaw79

Anopheles franciscanus	-115.019	36.0996	Unclassified	GBIF: https://doi.org/10.15468/dl.zfaw79
Anopheles franciscanus	-115.019	36.1001	Unclassified	GBIF: https://doi.org/10.15468/dl.zfaw79
Anopheles franciscanus	-115.019	36.9959	Unclassified	GBIF: https://doi.org/10.15468/dl.zfaw79
Anopheles franciscanus	-115.018	36.1023	Unclassified	GBIF: https://doi.org/10.15468/dl.zfaw79
Anopheles franciscanus	-115.018	36.0942	Unclassified	GBIF: https://doi.org/10.15468/dl.zfaw79
Anopheles franciscanus	-115.017	36.0924	Unclassified	GBIF: https://doi.org/10.15468/dl.zfaw79
Anopheles franciscanus	-115.017	36.0906	Unclassified	GBIF: https://doi.org/10.15468/dl.zfaw79
Anopheles franciscanus	-115.017	36.0807	Unclassified	GBIF: https://doi.org/10.15468/dl.zfaw79
Anopheles franciscanus	-115.017	36.0943	Unclassified	GBIF: https://doi.org/10.15468/dl.zfaw79
Anopheles franciscanus	-115.017	36.0905	Unclassified	GBIF: https://doi.org/10.15468/dl.zfaw79
Anopheles franciscanus	-115.017	36.0944	Unclassified	GBIF: https://doi.org/10.15468/dl.zfaw79
Anopheles franciscanus	-115.015	36.0929	Unclassified	GBIF: https://doi.org/10.15468/dl.zfaw79
Anopheles franciscanus	-115.014	36.0927	Unclassified	GBIF: https://doi.org/10.15468/dl.zfaw79
Anopheles franciscanus	-115.012	36.0789	Unclassified	GBIF: https://doi.org/10.15468/dl.zfaw79
Anopheles franciscanus	-115.009	36.0796	Unclassified	GBIF: https://doi.org/10.15468/dl.zfaw79
Anopheles franciscanus	-115.005	36.0779	Unclassified	GBIF: https://doi.org/10.15468/dl.zfaw79
Anopheles franciscanus	-115.002	36.0754	Unclassified	GBIF: https://doi.org/10.15468/dl.zfaw79
Anopheles franciscanus	-115.002	36.0785	Unclassified	GBIF: https://doi.org/10.15468/dl.zfaw79
Anopheles franciscanus	-115.002	36.0742	Unclassified	GBIF: https://doi.org/10.15468/dl.zfaw79
Anopheles franciscanus	-115.002	36.0808	Unclassified	GBIF: https://doi.org/10.15468/dl.zfaw79
Anopheles franciscanus	-115	36.0809	Unclassified	GBIF: https://doi.org/10.15468/dl.zfaw79
Anopheles franciscanus	-115	36.0808	Unclassified	GBIF: https://doi.org/10.15468/dl.zfaw79
Anopheles franciscanus	-114.998	36.0772	Unclassified	GBIF: https://doi.org/10.15468/dl.zfaw79
Anopheles franciscanus	-114.998	36.0773	Unclassified	GBIF: https://doi.org/10.15468/dl.zfaw79
Anopheles franciscanus	-114.998	36.0771	Unclassified	GBIF: https://doi.org/10.15468/dl.zfaw79
Anopheles franciscanus	-114.998	36.0788	Unclassified	GBIF: https://doi.org/10.15468/dl.zfaw79
Anopheles franciscanus	-114.989	36.0721	Unclassified	GBIF: https://doi.org/10.15468/dl.zfaw79
Anopheles franciscanus	-114.974	36.0894	Unclassified	GBIF: https://doi.org/10.15468/dl.zfaw79
Anopheles franciscanus	-114.968	36.0915	Unclassified	GBIF: https://doi.org/10.15468/dl.zfaw79
Anopheles franciscanus	-114.967	36.0914	Unclassified	GBIF: https://doi.org/10.15468/dl.zfaw79
Anopheles franciscanus	-114.958	36.0857	Unclassified	GBIF: https://doi.org/10.15468/dl.zfaw79
Anopheles franciscanus	-114.95	35.9978	Unclassified	GBIF: https://doi.org/10.15468/dl.zfaw79
Anopheles franciscanus	-114.942	36.0788	Unclassified	GBIF: https://doi.org/10.15468/dl.zfaw79
Anopheles franciscanus	-114.937	36.1038	Unclassified	GBIF: https://doi.org/10.15468/dl.zfaw79
Anopheles franciscanus	-114.936	36.1044	Unclassified	GBIF: https://doi.org/10.15468/dl.zfaw79
Anopheles franciscanus	-114.935	36.0865	Unclassified	GBIF: https://doi.org/10.15468/dl.zfaw79
Anopheles franciscanus	-114.927	36.0864	Unclassified	GBIF: https://doi.org/10.15468/dl.zfaw79
Anopheles franciscanus	-114.77	36.5441	Unclassified	GBIF: https://doi.org/10.15468/dl.zfaw79
Anopheles franciscanus	-114.717	36.7214	Unclassified	GBIF: https://doi.org/10.15468/dl.zfaw79
Anopheles franciscanus	-114.717	36.7261	Unclassified	GBIF: https://doi.org/10.15468/dl.zfaw79
Anopheles franciscanus	-114.716	36.7191	Unclassified	GBIF: https://doi.org/10.15468/dl.zfaw79
Anopheles franciscanus	-114.695	36.7106	Unclassified	GBIF: https://doi.org/10.15468/dl.zfaw79

Anopheles franciscanus	-114.694	36.7099	Unclassified	GBIF: https://doi.org/10.15468/dl.zfaw79
Anopheles franciscanus	-114.685	36.6772	Unclassified	GBIF: https://doi.org/10.15468/dl.zfaw79
Anopheles franciscanus	-114.684	36.684	Unclassified	GBIF: https://doi.org/10.15468/dl.zfaw79
Anopheles franciscanus	-114.681	36.7061	Unclassified	GBIF: https://doi.org/10.15468/dl.zfaw79
Anopheles franciscanus	-114.681	36.7036	Unclassified	GBIF: https://doi.org/10.15468/dl.zfaw79
Anopheles franciscanus	-114.68	36.7038	Unclassified	GBIF: https://doi.org/10.15468/dl.zfaw79
Anopheles franciscanus	-114.678	36.6986	Unclassified	GBIF: https://doi.org/10.15468/dl.zfaw79
Anopheles franciscanus	-114.662	36.6783	Unclassified	GBIF: https://doi.org/10.15468/dl.zfaw79
Anopheles franciscanus	-114.648	35.1074	Unclassified	GBIF: https://doi.org/10.15468/dl.zfaw79
Anopheles franciscanus	-114.647	35.1021	Unclassified	GBIF: https://doi.org/10.15468/dl.zfaw79
Anopheles franciscanus	-114.646	35.1145	Unclassified	GBIF: https://doi.org/10.15468/dl.zfaw79
Anopheles franciscanus	-114.642	35.1098	Unclassified	GBIF: https://doi.org/10.15468/dl.zfaw79
Anopheles franciscanus	-114.633356	32.75883	Unclassified	GBIF: https://doi.org/10.15468/dl.zfaw79
Anopheles franciscanus	-114.592	36.6731	Unclassified	GBIF: https://doi.org/10.15468/dl.zfaw79
Anopheles franciscanus	-114.592	36.6854	Unclassified	GBIF: https://doi.org/10.15468/dl.zfaw79
Anopheles franciscanus	-114.59	36.6713	Unclassified	GBIF: https://doi.org/10.15468/dl.zfaw79
Anopheles franciscanus	-114.589	36.6713	Unclassified	GBIF: https://doi.org/10.15468/dl.zfaw79
Anopheles franciscanus	-114.586	36.6737	Unclassified	GBIF: https://doi.org/10.15468/dl.zfaw79
Anopheles franciscanus	-114.585	36.6687	Unclassified	GBIF: https://doi.org/10.15468/dl.zfaw79
Anopheles franciscanus	-114.582	36.6701	Unclassified	GBIF: https://doi.org/10.15468/dl.zfaw79
Anopheles franciscanus	-114.582	36.6705	Unclassified	GBIF: https://doi.org/10.15468/dl.zfaw79
Anopheles franciscanus	-114.526	37.6116	Unclassified	GBIF: https://doi.org/10.15468/dl.zfaw79
Anopheles franciscanus	-114.525	37.6112	Unclassified	GBIF: https://doi.org/10.15468/dl.zfaw79
Anopheles franciscanus	-114.515	37.6173	Unclassified	GBIF: https://doi.org/10.15468/dl.zfaw79
Anopheles franciscanus	-114.509	37.6199	Unclassified	GBIF: https://doi.org/10.15468/dl.zfaw79
Anopheles franciscanus	-114.489	36.6082	Unclassified	GBIF: https://doi.org/10.15468/dl.zfaw79
Anopheles franciscanus	-114.483	36.6277	Unclassified	GBIF: https://doi.org/10.15468/dl.zfaw79
Anopheles franciscanus	-114.482	36.6266	Unclassified	GBIF: https://doi.org/10.15468/dl.zfaw79
Anopheles franciscanus	-114.481	36.6206	Unclassified	GBIF: https://doi.org/10.15468/dl.zfaw79
Anopheles franciscanus	-114.481	36.6208	Unclassified	GBIF: https://doi.org/10.15468/dl.zfaw79
Anopheles franciscanus	-114.481	36.6207	Unclassified	GBIF: https://doi.org/10.15468/dl.zfaw79
Anopheles franciscanus	-114.445	36.5472	Unclassified	GBIF: https://doi.org/10.15468/dl.zfaw79
Anopheles franciscanus	-114.443	36.5462	Unclassified	GBIF: https://doi.org/10.15468/dl.zfaw79
Anopheles franciscanus	-114.439	36.5419	Unclassified	GBIF: https://doi.org/10.15468/dl.zfaw79
Anopheles franciscanus	-114.439	36.542	Unclassified	GBIF: https://doi.org/10.15468/dl.zfaw79
Anopheles franciscanus	-114.437	36.5519	Unclassified	GBIF: https://doi.org/10.15468/dl.zfaw79
Anopheles franciscanus	-114.429	36.5355	Unclassified	GBIF: https://doi.org/10.15468/dl.zfaw79
Anopheles franciscanus	-114.428	36.5266	Unclassified	GBIF: https://doi.org/10.15468/dl.zfaw79
Anopheles franciscanus	-114.422	36.5151	Unclassified	GBIF: https://doi.org/10.15468/dl.zfaw79
Anopheles franciscanus	-114.421	36.5178	Unclassified	GBIF: https://doi.org/10.15468/dl.zfaw79
Anopheles franciscanus	-114.392	37.7903	Unclassified	GBIF: https://doi.org/10.15468/dl.zfaw79
Anopheles franciscanus	-114.385	36.0621	Unclassified	GBIF: https://doi.org/10.15468/dl.zfaw79

Anopheles franciscanus	-114.382	37.8074	Unclassified	GBIF: https://doi.org/10.15468/dl.zfaw79
Anopheles franciscanus	-114.381	37.8067	Unclassified	GBIF: https://doi.org/10.15468/dl.zfaw79
Anopheles franciscanus	-114.381	37.8066	Unclassified	GBIF: https://doi.org/10.15468/dl.zfaw79
Anopheles franciscanus	-114.37	35.0447	Unclassified	GBIF: https://doi.org/10.15468/dl.zfaw79
Anopheles franciscanus	-114.365	35.0447	Unclassified	GBIF: https://doi.org/10.15468/dl.zfaw79
Anopheles franciscanus	-114.305	37.37	Unclassified	GBIF: https://doi.org/10.15468/dl.zfaw79
Anopheles franciscanus	-114.295	37.3915	Unclassified	GBIF: https://doi.org/10.15468/dl.zfaw79
Anopheles franciscanus	-114.295	37.3916	Unclassified	GBIF: https://doi.org/10.15468/dl.zfaw79
Anopheles franciscanus	-114.187	38.0263	Unclassified	GBIF: https://doi.org/10.15468/dl.zfaw79
Anopheles franciscanus	-114.162	36.8026	Unclassified	GBIF: https://doi.org/10.15468/dl.zfaw79
Anopheles franciscanus	-114.147	36.7635	Unclassified	GBIF: https://doi.org/10.15468/dl.zfaw79
Anopheles franciscanus	-114.132	36.7713	Unclassified	GBIF: https://doi.org/10.15468/dl.zfaw79
Anopheles franciscanus	-114.13	36.78	Unclassified	GBIF: https://doi.org/10.15468/dl.zfaw79
Anopheles franciscanus	-114.115	36.7921	Unclassified	GBIF: https://doi.org/10.15468/dl.zfaw79
Anopheles franciscanus	-114.114	36.793	Unclassified	GBIF: https://doi.org/10.15468/dl.zfaw79
Anopheles franciscanus	-114.101	36.7961	Unclassified	GBIF: https://doi.org/10.15468/dl.zfaw79
Anopheles franciscanus	-114.097	36.8079	Unclassified	GBIF: https://doi.org/10.15468/dl.zfaw79
Anopheles franciscanus	-114.091	36.7896	Unclassified	GBIF: https://doi.org/10.15468/dl.zfaw79
Anopheles franciscanus	-114.084	36.794	Unclassified	GBIF: https://doi.org/10.15468/dl.zfaw79
Anopheles franciscanus	-114.084	36.7929	Unclassified	GBIF: https://doi.org/10.15468/dl.zfaw79
Anopheles franciscanus	-114.083	36.794	Unclassified	GBIF: https://doi.org/10.15468/dl.zfaw79
Anopheles franciscanus	-114.08	36.7945	Unclassified	GBIF: https://doi.org/10.15468/dl.zfaw79
Anopheles franciscanus	-114.08	36.7962	Unclassified	GBIF: https://doi.org/10.15468/dl.zfaw79
Anopheles franciscanus	-114.079	36.7968	Unclassified	GBIF: https://doi.org/10.15468/dl.zfaw79
Anopheles franciscanus	-114.077	36.7986	Unclassified	GBIF: https://doi.org/10.15468/dl.zfaw79
Anopheles franciscanus	-114.076	36.8033	Unclassified	GBIF: https://doi.org/10.15468/dl.zfaw79
Anopheles franciscanus	-114.075	36.7981	Unclassified	GBIF: https://doi.org/10.15468/dl.zfaw79
Anopheles franciscanus	-114.075	36.798	Unclassified	GBIF: https://doi.org/10.15468/dl.zfaw79
Anopheles franciscanus	-114.069	36.8009	Unclassified	GBIF: https://doi.org/10.15468/dl.zfaw79
Anopheles franciscanus	-114.067	36.8007	Unclassified	GBIF: https://doi.org/10.15468/dl.zfaw79
Anopheles franciscanus	-114.067	36.8008	Unclassified	GBIF: https://doi.org/10.15468/dl.zfaw79
Anopheles franciscanus	-114.066	36.8007	Unclassified	GBIF: https://doi.org/10.15468/dl.zfaw79
Anopheles franciscanus	-114.066	36.8005	Unclassified	GBIF: https://doi.org/10.15468/dl.zfaw79
Anopheles franciscanus	-114.066	36.8077	Unclassified	GBIF: https://doi.org/10.15468/dl.zfaw79
Anopheles franciscanus	-114.064	36.8161	Unclassified	GBIF: https://doi.org/10.15468/dl.zfaw79
Anopheles franciscanus	-114.064	36.816	Unclassified	GBIF: https://doi.org/10.15468/dl.zfaw79
Anopheles franciscanus	-114.063	36.8163	Unclassified	GBIF: https://doi.org/10.15468/dl.zfaw79
Anopheles franciscanus	-114.062	36.8022	Unclassified	GBIF: https://doi.org/10.15468/dl.zfaw79
Anopheles franciscanus	-114.062	36.802	Unclassified	GBIF: https://doi.org/10.15468/dl.zfaw79
Anopheles franciscanus	-114.062	36.8029	Unclassified	GBIF: https://doi.org/10.15468/dl.zfaw79
Anopheles franciscanus	-114.062	36.8025	Unclassified	GBIF: https://doi.org/10.15468/dl.zfaw79
Anopheles franciscanus	-114.06	36.8169	Unclassified	GBIF: https://doi.org/10.15468/dl.zfaw79

Anopheles franciscanus	-114.034	36.481	Unclassified	GBIF: https://doi.org/10.15468/dl.zfaw79
Anopheles franciscanus	-112.64641	34.629015	Unclassified	GBIF: https://doi.org/10.15468/dl.zfaw79
Anopheles franciscanus	-112.545944	34.546158	Unclassified	GBIF: https://doi.org/10.15468/dl.zfaw79
Anopheles franciscanus	-112.022	34.75	Unclassified	GBIF: https://doi.org/10.15468/dl.zfaw79
Anopheles franciscanus	-112.013	34.754	Unclassified	GBIF: https://doi.org/10.15468/dl.zfaw79
Anopheles franciscanus	-111.971442	26.881458	Unclassified	GBIF: https://doi.org/10.15468/dl.zfaw79
Anopheles franciscanus	-111.886482	33.438842	Unclassified	GBIF: https://doi.org/10.15468/dl.zfaw79
Anopheles franciscanus	-111.844008	26.03639	Unclassified	GBIF: https://doi.org/10.15468/dl.zfaw79
Anopheles franciscanus	-111.837306	26.036959	Unclassified	GBIF: https://doi.org/10.15468/dl.zfaw79
Anopheles franciscanus	-111.564566	26.010519	Unclassified	GBIF: https://doi.org/10.15468/dl.zfaw79
Anopheles franciscanus	-111.23701	31.461652	Unclassified	GBIF: https://doi.org/10.15468/dl.zfaw79
Anopheles franciscanus	-111.087482	31.402633	Unclassified	GBIF: https://doi.org/10.15468/dl.zfaw79
Anopheles franciscanus	-111.021	32.28	Unclassified	GBIF: https://doi.org/10.15468/dl.zfaw79
Anopheles franciscanus	-110.90327	31.805422	Unclassified	GBIF: https://doi.org/10.15468/dl.zfaw79
Anopheles franciscanus	-110.903	31.805	Unclassified	GBIF: https://doi.org/10.15468/dl.zfaw79
Anopheles franciscanus	-110.894336	31.849247	Unclassified	GBIF: https://doi.org/10.15468/dl.zfaw79
Anopheles franciscanus	-110.894	31.849	Unclassified	GBIF: https://doi.org/10.15468/dl.zfaw79
Anopheles franciscanus	-110.883327	31.794002	Unclassified	GBIF: https://doi.org/10.15468/dl.zfaw79
Anopheles franciscanus	-110.883	31.794	Unclassified	GBIF: https://doi.org/10.15468/dl.zfaw79
Anopheles franciscanus	-110.879072	31.843809	Unclassified	GBIF: https://doi.org/10.15468/dl.zfaw79
Anopheles franciscanus	-110.879	31.844	Unclassified	GBIF: https://doi.org/10.15468/dl.zfaw79
Anopheles franciscanus	-110.869	31.888	Unclassified	GBIF: https://doi.org/10.15468/dl.zfaw79
Anopheles franciscanus	-110.868606	31.887663	Unclassified	GBIF: https://doi.org/10.15468/dl.zfaw79
Anopheles franciscanus	-110.83549	31.91068	Unclassified	GBIF: https://doi.org/10.15468/dl.zfaw79
Anopheles franciscanus	-110.833873	31.800204	Unclassified	GBIF: https://doi.org/10.15468/dl.zfaw79
Anopheles franciscanus	-110.833375	31.900587	Unclassified	GBIF: https://doi.org/10.15468/dl.zfaw79
Anopheles franciscanus	-110.833	31.901	Unclassified	GBIF: https://doi.org/10.15468/dl.zfaw79
Anopheles franciscanus	-110.814125	31.851864	Unclassified	GBIF: https://doi.org/10.15468/dl.zfaw79
Anopheles franciscanus	-110.813994	32.35255	Unclassified	GBIF: https://doi.org/10.15468/dl.zfaw79
Anopheles franciscanus	-110.813433	31.877763	Unclassified	GBIF: https://doi.org/10.15468/dl.zfaw79
Anopheles franciscanus	-110.813	31.878	Unclassified	GBIF: https://doi.org/10.15468/dl.zfaw79
Anopheles franciscanus	-110.334701	34.955294	Unclassified	GBIF: https://doi.org/10.15468/dl.zfaw79
Anopheles franciscanus	-110.3067	31.4492	Unclassified	GBIF: https://doi.org/10.15468/dl.zfaw79
Anopheles franciscanus	-110.306103	31.449369	Unclassified	GBIF: https://doi.org/10.15468/dl.zfaw79
Anopheles franciscanus	-109.980296	31.918911	Unclassified	GBIF: https://doi.org/10.15468/dl.zfaw79
Anopheles franciscanus	-109.73336	23.340555	Unclassified	GBIF: https://doi.org/10.15468/dl.zfaw79
Anopheles franciscanus	-109.426013	38.263925	Unclassified	GBIF: https://doi.org/10.15468/dl.zfaw79
Anopheles franciscanus	-109.41782	38.262994	Unclassified	GBIF: https://doi.org/10.15468/dl.zfaw79
Anopheles franciscanus	-109.401	38.235	Unclassified	GBIF: https://doi.org/10.15468/dl.zfaw79
Anopheles franciscanus	-109.400608	38.23527	Unclassified	GBIF: https://doi.org/10.15468/dl.zfaw79
Anopheles franciscanus	-109.39442	38.221342	Unclassified	GBIF: https://doi.org/10.15468/dl.zfaw79
Anopheles franciscanus	-109.394	38.221	Unclassified	GBIF: https://doi.org/10.15468/dl.zfaw79

Anopheles franciscanus	-109.350332	38.277972	Unclassified	GBIF: https://doi.org/10.15468/dl.zfaw79
Anopheles franciscanus	-109.03117	32.053076	Unclassified	GBIF: https://doi.org/10.15468/dl.zfaw79
Anopheles franciscanus	-106.863663	32.609219	Unclassified	GBIF: https://doi.org/10.15468/dl.zfaw79
Anopheles franciscanus	-106.85916	32.607928	Unclassified	GBIF: https://doi.org/10.15468/dl.zfaw79
Anopheles franciscanus	-106.843741	32.605275	Unclassified	GBIF: https://doi.org/10.15468/dl.zfaw79
Anopheles franciscanus	-106.842542	32.590694	Unclassified	GBIF: https://doi.org/10.15468/dl.zfaw79
Anopheles franciscanus	-106.840847	32.634737	Unclassified	GBIF: https://doi.org/10.15468/dl.zfaw79
Anopheles franciscanus	-106.818828	32.605277	Unclassified	GBIF: https://doi.org/10.15468/dl.zfaw79
Anopheles franciscanus	-106.814391	32.607504	Unclassified	GBIF: https://doi.org/10.15468/dl.zfaw79
Anopheles franciscanus	-106.813332	32.570443	Unclassified	GBIF: https://doi.org/10.15468/dl.zfaw79
Anopheles franciscanus	-106.813	32.57	Unclassified	GBIF: https://doi.org/10.15468/dl.zfaw79
Anopheles franciscanus	-106.803214	32.608205	Unclassified	GBIF: https://doi.org/10.15468/dl.zfaw79
Anopheles franciscanus	-106.803	32.608	Unclassified	GBIF: https://doi.org/10.15468/dl.zfaw79
Anopheles franciscanus	-106.80028	32.284513	Unclassified	GBIF: https://doi.org/10.15468/dl.zfaw79
Anopheles franciscanus	-105.471739	28.273111	Unclassified	GBIF: https://doi.org/10.15468/dl.zfaw79
Anopheles franciscanus	-104.714539	40.85555	Unclassified	GBIF: https://doi.org/10.15468/dl.zfaw79
Anopheles franciscanus	-104.19	30.85	Unclassified	GBIF: https://doi.org/10.15468/dl.zfaw79
Anopheles franciscanus	-103.79	30.86	Unclassified	GBIF: https://doi.org/10.15468/dl.zfaw79
Anopheles franciscanus	-103.67	30.51	Unclassified	GBIF: https://doi.org/10.15468/dl.zfaw79
Anopheles franciscanus	-103.61	29.17	Unclassified	GBIF: https://doi.org/10.15468/dl.zfaw79
Anopheles franciscanus	-103.34	29.28	Unclassified	GBIF: https://doi.org/10.15468/dl.zfaw79
Anopheles franciscanus	-103.12	29.36	Unclassified	GBIF: https://doi.org/10.15468/dl.zfaw79
Anopheles franciscanus	-102.406083	21.035278	Unclassified	GBIF: https://doi.org/10.15468/dl.zfaw79
Anopheles franciscanus	-101.21	33.19	Unclassified	GBIF: https://doi.org/10.15468/dl.zfaw79
Anopheles franciscanus	-100.516667	21.2975	Unclassified	GBIF: https://doi.org/10.15468/dl.zfaw79
Anopheles franciscanus	-99.56	18.81	Unclassified	GBIF: https://doi.org/10.15468/dl.zfaw79
Anopheles franciscanus	-99.297417	25.726583	Unclassified	GBIF: https://doi.org/10.15468/dl.zfaw79
Anopheles franciscanus	-98.979444	23.42	Unclassified	GBIF: https://doi.org/10.15468/dl.zfaw79
Anopheles franciscanus	-98.913889	23.5125	Unclassified	GBIF: https://doi.org/10.15468/dl.zfaw79
Anopheles franciscanus	-98.674697	23.444894	Unclassified	GBIF: https://doi.org/10.15468/dl.zfaw79
Anopheles franciscanus	-97.479631	25.941271	Unclassified	GBIF: https://doi.org/10.15468/dl.zfaw79
Anopheles franciscanus	-96.927187	18.880769	Unclassified	GBIF: https://doi.org/10.15468/dl.zfaw79
Anopheles franciscanus	-96.926389	15.888333	Unclassified	GBIF: https://doi.org/10.15468/dl.zfaw79
Anopheles franciscanus	-96.5405	17.276556	Unclassified	GBIF: https://doi.org/10.15468/dl.zfaw79
Anopheles franciscanus	-96.493305	17.319	Unclassified	GBIF: https://doi.org/10.15468/dl.zfaw79
Anopheles franciscanus	-96.491667	17.325	Unclassified	GBIF: https://doi.org/10.15468/dl.zfaw79
Anopheles franciscanus	-96.3625	16.922222	Unclassified	GBIF: https://doi.org/10.15468/dl.zfaw79
Anopheles franciscanus	-96.330278	16.924722	Unclassified	GBIF: https://doi.org/10.15468/dl.zfaw79
Anopheles franciscanus	-96.330278	16.925028	Unclassified	GBIF: https://doi.org/10.15468/dl.zfaw79
Anopheles franciscanus	-96.101139	16.657667	Unclassified	GBIF: https://doi.org/10.15468/dl.zfaw79
Anopheles franciscanus	-95.638778	15.979639	Unclassified	GBIF: https://doi.org/10.15468/dl.zfaw79
Anopheles franciscanus	-95.599444	16.411944	Unclassified	GBIF: https://doi.org/10.15468/dl.zfaw79

Anopheles franciscanus	-95.599167	16.412222	Unclassified	GBIF: https://doi.org/10.15468/dl.zfaw79
Anopheles franciscanus	-95.536667	15.968333	Unclassified	GBIF: https://doi.org/10.15468/dl.zfaw79
Anopheles franciscanus	-95.406	16.091306	Unclassified	GBIF: https://doi.org/10.15468/dl.zfaw79
Anopheles franciscanus	-91.62925	16.097361	Unclassified	GBIF: https://doi.org/10.15468/dl.zfaw79
Anopheles franciscanus	-80	9	Unclassified	GBIF: https://doi.org/10.15468/dl.zfaw79
Anopheles franciscanus	-64.728052	-21.525701	Unclassified	GBIF: https://doi.org/10.15468/dl.zfaw79
Anopheles franciscanus	0	0	Unclassified	GBIF: https://doi.org/10.15468/dl.zfaw79
Anopheles franciscoi	121.048756	14.419291	Unclassified	GBIF: https://doi.org/10.15468/dl.zfaw79
Anopheles franciscoi	123.994019	12.769742	Unclassified	GBIF: https://doi.org/10.15468/dl.zfaw79
Anopheles franciscoi	124.203611	7.2575	Unclassified	GBIF: https://doi.org/10.15468/dl.zfaw79
Anopheles franciscoi	124.244131	7.212787	Unclassified	GBIF: https://doi.org/10.15468/dl.zfaw79
Anopheles franciscoi	124.244848	7.210794	Unclassified	GBIF: https://doi.org/10.15468/dl.zfaw79
Anopheles franciscoi	124.244989	7.208729	Unclassified	GBIF: https://doi.org/10.15468/dl.zfaw79
Anopheles franciscoi	124.246289	7.21592	Unclassified	GBIF: https://doi.org/10.15468/dl.zfaw79
Anopheles freeborni	-123.4722	38.9128	Unclassified	GBIF: https://doi.org/10.15468/dl.zfaw79
Anopheles freeborni	-122.980832	39.175168	Unclassified	GBIF: https://doi.org/10.15468/dl.zfaw79
Anopheles freeborni	-122.83992	38.394213	Unclassified	GBIF: https://doi.org/10.15468/dl.zfaw79
Anopheles freeborni	-122.781926	38.612008	Unclassified	GBIF: https://doi.org/10.15468/dl.zfaw79
Anopheles freeborni	-122.781818	38.612224	Unclassified	GBIF: https://doi.org/10.15468/dl.zfaw79
Anopheles freeborni	-122.636222	49.242078	Unclassified	GBIF: https://doi.org/10.15468/dl.zfaw79
Anopheles freeborni	-122.278048	37.779215	Unclassified	GBIF: https://doi.org/10.15468/dl.zfaw79
Anopheles freeborni	-122.277284	37.777428	Unclassified	GBIF: https://doi.org/10.15468/dl.zfaw79
Anopheles freeborni	-122.0525	39.933611	Unclassified	GBIF: https://doi.org/10.15468/dl.zfaw79
Anopheles freeborni	-122.031073	37.977978	Unclassified	GBIF: https://doi.org/10.15468/dl.zfaw79
Anopheles freeborni	-121.983875	39.759103	Unclassified	GBIF: https://doi.org/10.15468/dl.zfaw79
Anopheles freeborni	-121.814814	37.242144	Unclassified	GBIF: https://doi.org/10.15468/dl.zfaw79
Anopheles freeborni	-121.756905	38.565158	Unclassified	GBIF: https://doi.org/10.15468/dl.zfaw79
Anopheles freeborni	-121.740517	38.544906	Unclassified	GBIF: https://doi.org/10.15468/dl.zfaw79
Anopheles freeborni	-121.732404	39.087231	Unclassified	GBIF: https://doi.org/10.15468/dl.zfaw79
Anopheles freeborni	-121.723611	38.553611	Unclassified	GBIF: https://doi.org/10.15468/dl.zfaw79
Anopheles freeborni	-121.664062	38.453139	Unclassified	GBIF: https://doi.org/10.15468/dl.zfaw79
Anopheles freeborni	-121.587175	38.572072	Unclassified	GBIF: https://doi.org/10.15468/dl.zfaw79
Anopheles freeborni	-121.548536	39.071536	Unclassified	GBIF: https://doi.org/10.15468/dl.zfaw79
Anopheles freeborni	-121.547943	39.071442	Unclassified	GBIF: https://doi.org/10.15468/dl.zfaw79
Anopheles freeborni	-121.533856	39.329056	Unclassified	GBIF: https://doi.org/10.15468/dl.zfaw79
Anopheles freeborni	-121.508994	40.829684	Unclassified	GBIF: https://doi.org/10.15468/dl.zfaw79
Anopheles freeborni	-121.503216	39.472765	Unclassified	GBIF: https://doi.org/10.15468/dl.zfaw79
Anopheles freeborni	-121.487557	38.576589	Unclassified	GBIF: https://doi.org/10.15468/dl.zfaw79
Anopheles freeborni	-121.478029	38.625347	Unclassified	GBIF: https://doi.org/10.15468/dl.zfaw79
Anopheles freeborni	-121.438239	41.004056	Unclassified	GBIF: https://doi.org/10.15468/dl.zfaw79
Anopheles freeborni	-121.385962	44.01501	Unclassified	GBIF: https://doi.org/10.15468/dl.zfaw79
Anopheles freeborni	-121.38035	39.363989	Unclassified	GBIF: https://doi.org/10.15468/dl.zfaw79

Anopheles freeborni	-121.348445	38.680409	Unclassified	GBIF: https://doi.org/10.15468/dl.zfaw79
Anopheles freeborni	-121.007	40.29972	Unclassified	GBIF: https://doi.org/10.15468/dl.zfaw79
Anopheles freeborni	-120.996878	37.639097	Unclassified	GBIF: https://doi.org/10.15468/dl.zfaw79
Anopheles freeborni	-120.671	50.164	Unclassified	GBIF: https://doi.org/10.15468/dl.zfaw79
Anopheles freeborni	-120.62556	36.98611	Unclassified	GBIF: https://doi.org/10.15468/dl.zfaw79
Anopheles freeborni	-120.505899	46.602071	Unclassified	GBIF: https://doi.org/10.15468/dl.zfaw79
Anopheles freeborni	-120.36639	39.58972	Unclassified	GBIF: https://doi.org/10.15468/dl.zfaw79
Anopheles freeborni	-120.355006	39.559744	Unclassified	GBIF: https://doi.org/10.15468/dl.zfaw79
Anopheles freeborni	-119.843637	36.858892	Unclassified	GBIF: https://doi.org/10.15468/dl.zfaw79
Anopheles freeborni	-119.785214	36.888879	Unclassified	GBIF: https://doi.org/10.15468/dl.zfaw79
Anopheles freeborni	-119.749511	37.109009	Unclassified	GBIF: https://doi.org/10.15468/dl.zfaw79
Anopheles freeborni	-119.401756	49.946793	Unclassified	GBIF: https://doi.org/10.15468/dl.zfaw79
Anopheles freeborni	-119.398813	49.953066	Unclassified	GBIF: https://doi.org/10.15468/dl.zfaw79
Anopheles freeborni	-118.777374	39.473529	Unclassified	GBIF: https://doi.org/10.15468/dl.zfaw79
Anopheles freeborni	-118.406083	37.432458	Unclassified	GBIF: https://doi.org/10.15468/dl.zfaw79
Anopheles freeborni	-118.289546	37.164931	Unclassified	GBIF: https://doi.org/10.15468/dl.zfaw79
Anopheles freeborni	-118.236318	36.802705	Unclassified	GBIF: https://doi.org/10.15468/dl.zfaw79
Anopheles freeborni	-118.200095	36.80271	Unclassified	GBIF: https://doi.org/10.15468/dl.zfaw79
Anopheles freeborni	-117.76328	33.608613	Unclassified	GBIF: https://doi.org/10.15468/dl.zfaw79
Anopheles freeborni	-117.755638	33.680078	Unclassified	GBIF: https://doi.org/10.15468/dl.zfaw79
Anopheles freeborni	-117.256794	33.078084	Unclassified	GBIF: https://doi.org/10.15468/dl.zfaw79
Anopheles freeborni	-117.19289	32.87733	Unclassified	GBIF: https://doi.org/10.15468/dl.zfaw79
Anopheles freeborni	-117.155	33.042	Unclassified	GBIF: https://doi.org/10.15468/dl.zfaw79
Anopheles freeborni	-116.940183	32.729028	Unclassified	GBIF: https://doi.org/10.15468/dl.zfaw79
Anopheles freeborni	-116.92919	33.799741	Unclassified	GBIF: https://doi.org/10.15468/dl.zfaw79
Anopheles freeborni	-116.757	36.9113	Unclassified	GBIF: https://doi.org/10.15468/dl.zfaw79
Anopheles freeborni	-116.757	36.9116	Unclassified	GBIF: https://doi.org/10.15468/dl.zfaw79
Anopheles freeborni	-116.757	36.9117	Unclassified	GBIF: https://doi.org/10.15468/dl.zfaw79
Anopheles freeborni	-116.754	36.8948	Unclassified	GBIF: https://doi.org/10.15468/dl.zfaw79
Anopheles freeborni	-116.754	36.9145	Unclassified	GBIF: https://doi.org/10.15468/dl.zfaw79
Anopheles freeborni	-116.754	36.9102	Unclassified	GBIF: https://doi.org/10.15468/dl.zfaw79
Anopheles freeborni	-116.754	36.8949	Unclassified	GBIF: https://doi.org/10.15468/dl.zfaw79
Anopheles freeborni	-116.752	36.8833	Unclassified	GBIF: https://doi.org/10.15468/dl.zfaw79
Anopheles freeborni	-116.751	36.8913	Unclassified	GBIF: https://doi.org/10.15468/dl.zfaw79
Anopheles freeborni	-116.751	36.8962	Unclassified	GBIF: https://doi.org/10.15468/dl.zfaw79
Anopheles freeborni	-116.751	36.8915	Unclassified	GBIF: https://doi.org/10.15468/dl.zfaw79
Anopheles freeborni	-116.75	36.8912	Unclassified	GBIF: https://doi.org/10.15468/dl.zfaw79
Anopheles freeborni	-116.717	36.9483	Unclassified	GBIF: https://doi.org/10.15468/dl.zfaw79
Anopheles freeborni	-116.717	36.9484	Unclassified	GBIF: https://doi.org/10.15468/dl.zfaw79
Anopheles freeborni	-116.717	36.9485	Unclassified	GBIF: https://doi.org/10.15468/dl.zfaw79
Anopheles freeborni	-116.697958	43.546839	Unclassified	GBIF: https://doi.org/10.15468/dl.zfaw79
Anopheles freeborni	-116.511	43.638	Unclassified	GBIF: https://doi.org/10.15468/dl.zfaw79

Anopheles freeborni	-116.51	43.6728	Unclassified	GBIF: https://doi.org/10.15468/dl.zfaw79
Anopheles freeborni	-116.509	43.6762	Unclassified	GBIF: https://doi.org/10.15468/dl.zfaw79
Anopheles freeborni	-116.508	43.676	Unclassified	GBIF: https://doi.org/10.15468/dl.zfaw79
Anopheles freeborni	-116.508	43.6858	Unclassified	GBIF: https://doi.org/10.15468/dl.zfaw79
Anopheles freeborni	-116.505	43.7038	Unclassified	GBIF: https://doi.org/10.15468/dl.zfaw79
Anopheles freeborni	-116.5	43.682	Unclassified	GBIF: https://doi.org/10.15468/dl.zfaw79
Anopheles freeborni	-116.499	43.6821	Unclassified	GBIF: https://doi.org/10.15468/dl.zfaw79
Anopheles freeborni	-116.498	43.6833	Unclassified	GBIF: https://doi.org/10.15468/dl.zfaw79
Anopheles freeborni	-116.496	43.6849	Unclassified	GBIF: https://doi.org/10.15468/dl.zfaw79
Anopheles freeborni	-116.494	43.6764	Unclassified	GBIF: https://doi.org/10.15468/dl.zfaw79
Anopheles freeborni	-116.493	43.6796	Unclassified	GBIF: https://doi.org/10.15468/dl.zfaw79
Anopheles freeborni	-116.493	43.6736	Unclassified	GBIF: https://doi.org/10.15468/dl.zfaw79
Anopheles freeborni	-116.493	43.7069	Unclassified	GBIF: https://doi.org/10.15468/dl.zfaw79
Anopheles freeborni	-116.492	43.7062	Unclassified	GBIF: https://doi.org/10.15468/dl.zfaw79
Anopheles freeborni	-116.48	43.7112	Unclassified	GBIF: https://doi.org/10.15468/dl.zfaw79
Anopheles freeborni	-116.478	43.744	Unclassified	GBIF: https://doi.org/10.15468/dl.zfaw79
Anopheles freeborni	-116.477	43.6787	Unclassified	GBIF: https://doi.org/10.15468/dl.zfaw79
Anopheles freeborni	-116.477	43.698	Unclassified	GBIF: https://doi.org/10.15468/dl.zfaw79
Anopheles freeborni	-116.476	43.6981	Unclassified	GBIF: https://doi.org/10.15468/dl.zfaw79
Anopheles freeborni	-116.473	43.5681	Unclassified	GBIF: https://doi.org/10.15468/dl.zfaw79
Anopheles freeborni	-116.473	43.7346	Unclassified	GBIF: https://doi.org/10.15468/dl.zfaw79
Anopheles freeborni	-116.471	43.5864	Unclassified	GBIF: https://doi.org/10.15468/dl.zfaw79
Anopheles freeborni	-116.468	43.7	Unclassified	GBIF: https://doi.org/10.15468/dl.zfaw79
Anopheles freeborni	-116.467	43.6583	Unclassified	GBIF: https://doi.org/10.15468/dl.zfaw79
Anopheles freeborni	-116.464	43.6396	Unclassified	GBIF: https://doi.org/10.15468/dl.zfaw79
Anopheles freeborni	-116.461	43.5425	Unclassified	GBIF: https://doi.org/10.15468/dl.zfaw79
Anopheles freeborni	-116.458	43.5145	Unclassified	GBIF: https://doi.org/10.15468/dl.zfaw79
Anopheles freeborni	-116.458	43.6252	Unclassified	GBIF: https://doi.org/10.15468/dl.zfaw79
Anopheles freeborni	-116.458	43.6151	Unclassified	GBIF: https://doi.org/10.15468/dl.zfaw79
Anopheles freeborni	-116.455	43.5583	Unclassified	GBIF: https://doi.org/10.15468/dl.zfaw79
Anopheles freeborni	-116.451	43.6894	Unclassified	GBIF: https://doi.org/10.15468/dl.zfaw79
Anopheles freeborni	-116.451	36.5341	Unclassified	GBIF: https://doi.org/10.15468/dl.zfaw79
Anopheles freeborni	-116.45	43.5073	Unclassified	GBIF: https://doi.org/10.15468/dl.zfaw79
Anopheles freeborni	-116.45	36.5329	Unclassified	GBIF: https://doi.org/10.15468/dl.zfaw79
Anopheles freeborni	-116.448	43.6659	Unclassified	GBIF: https://doi.org/10.15468/dl.zfaw79
Anopheles freeborni	-116.446	43.6164	Unclassified	GBIF: https://doi.org/10.15468/dl.zfaw79
Anopheles freeborni	-116.443	43.6865	Unclassified	GBIF: https://doi.org/10.15468/dl.zfaw79
Anopheles freeborni	-116.443	43.5284	Unclassified	GBIF: https://doi.org/10.15468/dl.zfaw79
Anopheles freeborni	-116.443	43.6144	Unclassified	GBIF: https://doi.org/10.15468/dl.zfaw79
Anopheles freeborni	-116.442	43.6122	Unclassified	GBIF: https://doi.org/10.15468/dl.zfaw79
Anopheles freeborni	-116.44	43.6679	Unclassified	GBIF: https://doi.org/10.15468/dl.zfaw79
Anopheles freeborni	-116.439	43.486	Unclassified	GBIF: https://doi.org/10.15468/dl.zfaw79

Anopheles freeborni	-116.438	43.4932	Unclassified	GBIF: https://doi.org/10.15468/dl.zfaw79
Anopheles freeborni	-116.433	43.6921	Unclassified	GBIF: https://doi.org/10.15468/dl.zfaw79
Anopheles freeborni	-116.433	43.6562	Unclassified	GBIF: https://doi.org/10.15468/dl.zfaw79
Anopheles freeborni	-116.43	36.5654	Unclassified	GBIF: https://doi.org/10.15468/dl.zfaw79
Anopheles freeborni	-116.429	43.6355	Unclassified	GBIF: https://doi.org/10.15468/dl.zfaw79
Anopheles freeborni	-116.429	43.4922	Unclassified	GBIF: https://doi.org/10.15468/dl.zfaw79
Anopheles freeborni	-116.424	43.7065	Unclassified	GBIF: https://doi.org/10.15468/dl.zfaw79
Anopheles freeborni	-116.423	43.4836	Unclassified	GBIF: https://doi.org/10.15468/dl.zfaw79
Anopheles freeborni	-116.423	43.7245	Unclassified	GBIF: https://doi.org/10.15468/dl.zfaw79
Anopheles freeborni	-116.423	43.5799	Unclassified	GBIF: https://doi.org/10.15468/dl.zfaw79
Anopheles freeborni	-116.422	43.482	Unclassified	GBIF: https://doi.org/10.15468/dl.zfaw79
Anopheles freeborni	-116.421	43.658	Unclassified	GBIF: https://doi.org/10.15468/dl.zfaw79
Anopheles freeborni	-116.42	43.5359	Unclassified	GBIF: https://doi.org/10.15468/dl.zfaw79
Anopheles freeborni	-116.418	43.6833	Unclassified	GBIF: https://doi.org/10.15468/dl.zfaw79
Anopheles freeborni	-116.417	43.5531	Unclassified	GBIF: https://doi.org/10.15468/dl.zfaw79
Anopheles freeborni	-116.414	43.6391	Unclassified	GBIF: https://doi.org/10.15468/dl.zfaw79
Anopheles freeborni	-116.412	43.4794	Unclassified	GBIF: https://doi.org/10.15468/dl.zfaw79
Anopheles freeborni	-116.412	43.6513	Unclassified	GBIF: https://doi.org/10.15468/dl.zfaw79
Anopheles freeborni	-116.411	43.5987	Unclassified	GBIF: https://doi.org/10.15468/dl.zfaw79
Anopheles freeborni	-116.411	43.6379	Unclassified	GBIF: https://doi.org/10.15468/dl.zfaw79
Anopheles freeborni	-116.41	43.4801	Unclassified	GBIF: https://doi.org/10.15468/dl.zfaw79
Anopheles freeborni	-116.41	43.7134	Unclassified	GBIF: https://doi.org/10.15468/dl.zfaw79
Anopheles freeborni	-116.409	43.6848	Unclassified	GBIF: https://doi.org/10.15468/dl.zfaw79
Anopheles freeborni	-116.408	43.5188	Unclassified	GBIF: https://doi.org/10.15468/dl.zfaw79
Anopheles freeborni	-116.407	43.6723	Unclassified	GBIF: https://doi.org/10.15468/dl.zfaw79
Anopheles freeborni	-116.407	43.5941	Unclassified	GBIF: https://doi.org/10.15468/dl.zfaw79
Anopheles freeborni	-116.405	43.6845	Unclassified	GBIF: https://doi.org/10.15468/dl.zfaw79
Anopheles freeborni	-116.405	43.6098	Unclassified	GBIF: https://doi.org/10.15468/dl.zfaw79
Anopheles freeborni	-116.404	43.599	Unclassified	GBIF: https://doi.org/10.15468/dl.zfaw79
Anopheles freeborni	-116.404	43.6528	Unclassified	GBIF: https://doi.org/10.15468/dl.zfaw79
Anopheles freeborni	-116.403	43.6783	Unclassified	GBIF: https://doi.org/10.15468/dl.zfaw79
Anopheles freeborni	-116.403	43.678	Unclassified	GBIF: https://doi.org/10.15468/dl.zfaw79
Anopheles freeborni	-116.401	43.6973	Unclassified	GBIF: https://doi.org/10.15468/dl.zfaw79
Anopheles freeborni	-116.401	43.4927	Unclassified	GBIF: https://doi.org/10.15468/dl.zfaw79
Anopheles freeborni	-116.399	43.7212	Unclassified	GBIF: https://doi.org/10.15468/dl.zfaw79
Anopheles freeborni	-116.396	43.6857	Unclassified	GBIF: https://doi.org/10.15468/dl.zfaw79
Anopheles freeborni	-116.394	43.472	Unclassified	GBIF: https://doi.org/10.15468/dl.zfaw79
Anopheles freeborni	-116.394	43.6963	Unclassified	GBIF: https://doi.org/10.15468/dl.zfaw79
Anopheles freeborni	-116.393	43.6757	Unclassified	GBIF: https://doi.org/10.15468/dl.zfaw79
Anopheles freeborni	-116.392	43.6855	Unclassified	GBIF: https://doi.org/10.15468/dl.zfaw79
Anopheles freeborni	-116.389	43.6459	Unclassified	GBIF: https://doi.org/10.15468/dl.zfaw79
Anopheles freeborni	-116.388	43.6882	Unclassified	GBIF: https://doi.org/10.15468/dl.zfaw79

Anopheles freeborni	-116.388	43.6985	Unclassified	GBIF: https://doi.org/10.15468/dl.zfaw79
Anopheles freeborni	-116.388	43.686	Unclassified	GBIF: https://doi.org/10.15468/dl.zfaw79
Anopheles freeborni	-116.387	43.6929	Unclassified	GBIF: https://doi.org/10.15468/dl.zfaw79
Anopheles freeborni	-116.387	43.6766	Unclassified	GBIF: https://doi.org/10.15468/dl.zfaw79
Anopheles freeborni	-116.386	43.5507	Unclassified	GBIF: https://doi.org/10.15468/dl.zfaw79
Anopheles freeborni	-116.386	43.7343	Unclassified	GBIF: https://doi.org/10.15468/dl.zfaw79
Anopheles freeborni	-116.384	43.6964	Unclassified	GBIF: https://doi.org/10.15468/dl.zfaw79
Anopheles freeborni	-116.382	43.6988	Unclassified	GBIF: https://doi.org/10.15468/dl.zfaw79
Anopheles freeborni	-116.381	43.6841	Unclassified	GBIF: https://doi.org/10.15468/dl.zfaw79
Anopheles freeborni	-116.375	43.7154	Unclassified	GBIF: https://doi.org/10.15468/dl.zfaw79
Anopheles freeborni	-116.374	43.6764	Unclassified	GBIF: https://doi.org/10.15468/dl.zfaw79
Anopheles freeborni	-116.374	43.6942	Unclassified	GBIF: https://doi.org/10.15468/dl.zfaw79
Anopheles freeborni	-116.374	43.701	Unclassified	GBIF: https://doi.org/10.15468/dl.zfaw79
Anopheles freeborni	-116.373	43.6712	Unclassified	GBIF: https://doi.org/10.15468/dl.zfaw79
Anopheles freeborni	-116.372	43.4793	Unclassified	GBIF: https://doi.org/10.15468/dl.zfaw79
Anopheles freeborni	-116.37	43.712	Unclassified	GBIF: https://doi.org/10.15468/dl.zfaw79
Anopheles freeborni	-116.37	43.5995	Unclassified	GBIF: https://doi.org/10.15468/dl.zfaw79
Anopheles freeborni	-116.37	43.6931	Unclassified	GBIF: https://doi.org/10.15468/dl.zfaw79
Anopheles freeborni	-116.369	43.6855	Unclassified	GBIF: https://doi.org/10.15468/dl.zfaw79
Anopheles freeborni	-116.369	43.5175	Unclassified	GBIF: https://doi.org/10.15468/dl.zfaw79
Anopheles freeborni	-116.367	43.6753	Unclassified	GBIF: https://doi.org/10.15468/dl.zfaw79
Anopheles freeborni	-116.363	43.6564	Unclassified	GBIF: https://doi.org/10.15468/dl.zfaw79
Anopheles freeborni	-116.362	43.6993	Unclassified	GBIF: https://doi.org/10.15468/dl.zfaw79
Anopheles freeborni	-116.362	43.6991	Unclassified	GBIF: https://doi.org/10.15468/dl.zfaw79
Anopheles freeborni	-116.361	43.6027	Unclassified	GBIF: https://doi.org/10.15468/dl.zfaw79
Anopheles freeborni	-116.359	43.6889	Unclassified	GBIF: https://doi.org/10.15468/dl.zfaw79
Anopheles freeborni	-116.359	43.489	Unclassified	GBIF: https://doi.org/10.15468/dl.zfaw79
Anopheles freeborni	-116.359	43.661	Unclassified	GBIF: https://doi.org/10.15468/dl.zfaw79
Anopheles freeborni	-116.357	43.7021	Unclassified	GBIF: https://doi.org/10.15468/dl.zfaw79
Anopheles freeborni	-116.356	43.6806	Unclassified	GBIF: https://doi.org/10.15468/dl.zfaw79
Anopheles freeborni	-116.356	43.6679	Unclassified	GBIF: https://doi.org/10.15468/dl.zfaw79
Anopheles freeborni	-116.353	43.6524	Unclassified	GBIF: https://doi.org/10.15468/dl.zfaw79
Anopheles freeborni	-116.353	36.444	Unclassified	GBIF: https://doi.org/10.15468/dl.zfaw79
Anopheles freeborni	-116.352	43.6862	Unclassified	GBIF: https://doi.org/10.15468/dl.zfaw79
Anopheles freeborni	-116.351	36.4449	Unclassified	GBIF: https://doi.org/10.15468/dl.zfaw79
Anopheles freeborni	-116.351	43.6859	Unclassified	GBIF: https://doi.org/10.15468/dl.zfaw79
Anopheles freeborni	-116.35	43.6791	Unclassified	GBIF: https://doi.org/10.15468/dl.zfaw79
Anopheles freeborni	-116.347	43.6869	Unclassified	GBIF: https://doi.org/10.15468/dl.zfaw79
Anopheles freeborni	-116.346	43.6708	Unclassified	GBIF: https://doi.org/10.15468/dl.zfaw79
Anopheles freeborni	-116.346	43.7082	Unclassified	GBIF: https://doi.org/10.15468/dl.zfaw79
Anopheles freeborni	-116.346	43.5027	Unclassified	GBIF: https://doi.org/10.15468/dl.zfaw79
Anopheles freeborni	-116.345	43.5129	Unclassified	GBIF: https://doi.org/10.15468/dl.zfaw79

Anopheles freeborni	-116.345	43.6162	Unclassified	GBIF: https://doi.org/10.15468/dl.zfaw79
Anopheles freeborni	-116.344	43.6811	Unclassified	GBIF: https://doi.org/10.15468/dl.zfaw79
Anopheles freeborni	-116.344	43.5246	Unclassified	GBIF: https://doi.org/10.15468/dl.zfaw79
Anopheles freeborni	-116.344	43.6018	Unclassified	GBIF: https://doi.org/10.15468/dl.zfaw79
Anopheles freeborni	-116.343	43.6976	Unclassified	GBIF: https://doi.org/10.15468/dl.zfaw79
Anopheles freeborni	-116.34	43.5924	Unclassified	GBIF: https://doi.org/10.15468/dl.zfaw79
Anopheles freeborni	-116.34	43.5392	Unclassified	GBIF: https://doi.org/10.15468/dl.zfaw79
Anopheles freeborni	-116.337	43.6779	Unclassified	GBIF: https://doi.org/10.15468/dl.zfaw79
Anopheles freeborni	-116.335	43.5029	Unclassified	GBIF: https://doi.org/10.15468/dl.zfaw79
Anopheles freeborni	-116.335	43.6847	Unclassified	GBIF: https://doi.org/10.15468/dl.zfaw79
Anopheles freeborni	-116.333	43.6562	Unclassified	GBIF: https://doi.org/10.15468/dl.zfaw79
Anopheles freeborni	-116.332	43.5115	Unclassified	GBIF: https://doi.org/10.15468/dl.zfaw79
Anopheles freeborni	-116.332	43.6851	Unclassified	GBIF: https://doi.org/10.15468/dl.zfaw79
Anopheles freeborni	-116.33	36.4202	Unclassified	GBIF: https://doi.org/10.15468/dl.zfaw79
Anopheles freeborni	-116.33	43.5388	Unclassified	GBIF: https://doi.org/10.15468/dl.zfaw79
Anopheles freeborni	-116.329	36.4132	Unclassified	GBIF: https://doi.org/10.15468/dl.zfaw79
Anopheles freeborni	-116.329	43.63	Unclassified	GBIF: https://doi.org/10.15468/dl.zfaw79
Anopheles freeborni	-116.328	36.4195	Unclassified	GBIF: https://doi.org/10.15468/dl.zfaw79
Anopheles freeborni	-116.328	43.7163	Unclassified	GBIF: https://doi.org/10.15468/dl.zfaw79
Anopheles freeborni	-116.326	36.4793	Unclassified	GBIF: https://doi.org/10.15468/dl.zfaw79
Anopheles freeborni	-116.326	36.4792	Unclassified	GBIF: https://doi.org/10.15468/dl.zfaw79
Anopheles freeborni	-116.325	43.5284	Unclassified	GBIF: https://doi.org/10.15468/dl.zfaw79
Anopheles freeborni	-116.325	43.6526	Unclassified	GBIF: https://doi.org/10.15468/dl.zfaw79
Anopheles freeborni	-116.323	43.5249	Unclassified	GBIF: https://doi.org/10.15468/dl.zfaw79
Anopheles freeborni	-116.322	43.527	Unclassified	GBIF: https://doi.org/10.15468/dl.zfaw79
Anopheles freeborni	-116.316	43.7009	Unclassified	GBIF: https://doi.org/10.15468/dl.zfaw79
Anopheles freeborni	-116.314	43.6887	Unclassified	GBIF: https://doi.org/10.15468/dl.zfaw79
Anopheles freeborni	-116.314	43.657	Unclassified	GBIF: https://doi.org/10.15468/dl.zfaw79
Anopheles freeborni	-116.312	43.6801	Unclassified	GBIF: https://doi.org/10.15468/dl.zfaw79
Anopheles freeborni	-116.31	43.6457	Unclassified	GBIF: https://doi.org/10.15468/dl.zfaw79
Anopheles freeborni	-116.31	36.4286	Unclassified	GBIF: https://doi.org/10.15468/dl.zfaw79
Anopheles freeborni	-116.31	36.4315	Unclassified	GBIF: https://doi.org/10.15468/dl.zfaw79
Anopheles freeborni	-116.309	43.5886	Unclassified	GBIF: https://doi.org/10.15468/dl.zfaw79
Anopheles freeborni	-116.307	43.6756	Unclassified	GBIF: https://doi.org/10.15468/dl.zfaw79
Anopheles freeborni	-116.303	36.4028	Unclassified	GBIF: https://doi.org/10.15468/dl.zfaw79
Anopheles freeborni	-116.302	36.4022	Unclassified	GBIF: https://doi.org/10.15468/dl.zfaw79
Anopheles freeborni	-116.302	36.4027	Unclassified	GBIF: https://doi.org/10.15468/dl.zfaw79
Anopheles freeborni	-116.3	43.6515	Unclassified	GBIF: https://doi.org/10.15468/dl.zfaw79
Anopheles freeborni	-116.299	43.6663	Unclassified	GBIF: https://doi.org/10.15468/dl.zfaw79
Anopheles freeborni	-116.297	43.6492	Unclassified	GBIF: https://doi.org/10.15468/dl.zfaw79
Anopheles freeborni	-116.296	43.6621	Unclassified	GBIF: https://doi.org/10.15468/dl.zfaw79
Anopheles freeborni	-116.295	43.6669	Unclassified	GBIF: https://doi.org/10.15468/dl.zfaw79

Anopheles freeborni	-116.294	43.6523	Unclassified	GBIF: https://doi.org/10.15468/dl.zfaw79
Anopheles freeborni	-116.294	43.6715	Unclassified	GBIF: https://doi.org/10.15468/dl.zfaw79
Anopheles freeborni	-116.294	43.6283	Unclassified	GBIF: https://doi.org/10.15468/dl.zfaw79
Anopheles freeborni	-116.292	43.5875	Unclassified	GBIF: https://doi.org/10.15468/dl.zfaw79
Anopheles freeborni	-116.289	43.6648	Unclassified	GBIF: https://doi.org/10.15468/dl.zfaw79
Anopheles freeborni	-116.288	43.5844	Unclassified	GBIF: https://doi.org/10.15468/dl.zfaw79
Anopheles freeborni	-116.285	43.6502	Unclassified	GBIF: https://doi.org/10.15468/dl.zfaw79
Anopheles freeborni	-116.283	43.5631	Unclassified	GBIF: https://doi.org/10.15468/dl.zfaw79
Anopheles freeborni	-116.282	43.6632	Unclassified	GBIF: https://doi.org/10.15468/dl.zfaw79
Anopheles freeborni	-116.281	43.6604	Unclassified	GBIF: https://doi.org/10.15468/dl.zfaw79
Anopheles freeborni	-116.281	43.6605	Unclassified	GBIF: https://doi.org/10.15468/dl.zfaw79
Anopheles freeborni	-116.28	43.6444	Unclassified	GBIF: https://doi.org/10.15468/dl.zfaw79
Anopheles freeborni	-116.279	43.6454	Unclassified	GBIF: https://doi.org/10.15468/dl.zfaw79
Anopheles freeborni	-116.277	43.645	Unclassified	GBIF: https://doi.org/10.15468/dl.zfaw79
Anopheles freeborni	-116.275	43.6439	Unclassified	GBIF: https://doi.org/10.15468/dl.zfaw79
Anopheles freeborni	-116.274	43.4904	Unclassified	GBIF: https://doi.org/10.15468/dl.zfaw79
Anopheles freeborni	-116.274	43.532	Unclassified	GBIF: https://doi.org/10.15468/dl.zfaw79
Anopheles freeborni	-116.271	43.6236	Unclassified	GBIF: https://doi.org/10.15468/dl.zfaw79
Anopheles freeborni	-116.27	43.5652	Unclassified	GBIF: https://doi.org/10.15468/dl.zfaw79
Anopheles freeborni	-116.265	43.6807	Unclassified	GBIF: https://doi.org/10.15468/dl.zfaw79
Anopheles freeborni	-116.264	43.5229	Unclassified	GBIF: https://doi.org/10.15468/dl.zfaw79
Anopheles freeborni	-116.26	43.6483	Unclassified	GBIF: https://doi.org/10.15468/dl.zfaw79
Anopheles freeborni	-116.258	43.7262	Unclassified	GBIF: https://doi.org/10.15468/dl.zfaw79
Anopheles freeborni	-116.255	43.6432	Unclassified	GBIF: https://doi.org/10.15468/dl.zfaw79
Anopheles freeborni	-116.255	43.7246	Unclassified	GBIF: https://doi.org/10.15468/dl.zfaw79
Anopheles freeborni	-116.254	43.7215	Unclassified	GBIF: https://doi.org/10.15468/dl.zfaw79
Anopheles freeborni	-116.254	43.726	Unclassified	GBIF: https://doi.org/10.15468/dl.zfaw79
Anopheles freeborni	-116.253	43.589	Unclassified	GBIF: https://doi.org/10.15468/dl.zfaw79
Anopheles freeborni	-116.252	43.6452	Unclassified	GBIF: https://doi.org/10.15468/dl.zfaw79
Anopheles freeborni	-116.252	43.7222	Unclassified	GBIF: https://doi.org/10.15468/dl.zfaw79
Anopheles freeborni	-116.251	43.6451	Unclassified	GBIF: https://doi.org/10.15468/dl.zfaw79
Anopheles freeborni	-116.25	43.7196	Unclassified	GBIF: https://doi.org/10.15468/dl.zfaw79
Anopheles freeborni	-116.248	43.641	Unclassified	GBIF: https://doi.org/10.15468/dl.zfaw79
Anopheles freeborni	-116.247	43.7218	Unclassified	GBIF: https://doi.org/10.15468/dl.zfaw79
Anopheles freeborni	-116.246	43.7218	Unclassified	GBIF: https://doi.org/10.15468/dl.zfaw79
Anopheles freeborni	-116.241	43.6478	Unclassified	GBIF: https://doi.org/10.15468/dl.zfaw79
Anopheles freeborni	-116.24	43.632	Unclassified	GBIF: https://doi.org/10.15468/dl.zfaw79
Anopheles freeborni	-116.233	43.6061	Unclassified	GBIF: https://doi.org/10.15468/dl.zfaw79
Anopheles freeborni	-116.231	43.6288	Unclassified	GBIF: https://doi.org/10.15468/dl.zfaw79
Anopheles freeborni	-116.228	43.6199	Unclassified	GBIF: https://doi.org/10.15468/dl.zfaw79
Anopheles freeborni	-116.225	43.6145	Unclassified	GBIF: https://doi.org/10.15468/dl.zfaw79
Anopheles freeborni	-116.222	43.6107	Unclassified	GBIF: https://doi.org/10.15468/dl.zfaw79

Anopheles freeborni	-116.216	43.6112	Unclassified	GBIF: https://doi.org/10.15468/dl.zfaw79
Anopheles freeborni	-116.204	43.6076	Unclassified	GBIF: https://doi.org/10.15468/dl.zfaw79
Anopheles freeborni	-116.203	43.6363	Unclassified	GBIF: https://doi.org/10.15468/dl.zfaw79
Anopheles freeborni	-116.198	43.6368	Unclassified	GBIF: https://doi.org/10.15468/dl.zfaw79
Anopheles freeborni	-116.196	43.6067	Unclassified	GBIF: https://doi.org/10.15468/dl.zfaw79
Anopheles freeborni	-116.195	36.2514	Unclassified	GBIF: https://doi.org/10.15468/dl.zfaw79
Anopheles freeborni	-116.194	36.2448	Unclassified	GBIF: https://doi.org/10.15468/dl.zfaw79
Anopheles freeborni	-116.185	43.5784	Unclassified	GBIF: https://doi.org/10.15468/dl.zfaw79
Anopheles freeborni	-116.184	43.5968	Unclassified	GBIF: https://doi.org/10.15468/dl.zfaw79
Anopheles freeborni	-116.183	43.6199	Unclassified	GBIF: https://doi.org/10.15468/dl.zfaw79
Anopheles freeborni	-116.183	43.5979	Unclassified	GBIF: https://doi.org/10.15468/dl.zfaw79
Anopheles freeborni	-116.181	43.6025	Unclassified	GBIF: https://doi.org/10.15468/dl.zfaw79
Anopheles freeborni	-116.18	36.241	Unclassified	GBIF: https://doi.org/10.15468/dl.zfaw79
Anopheles freeborni	-116.176	43.6017	Unclassified	GBIF: https://doi.org/10.15468/dl.zfaw79
Anopheles freeborni	-116.169	43.5654	Unclassified	GBIF: https://doi.org/10.15468/dl.zfaw79
Anopheles freeborni	-116.161	43.5819	Unclassified	GBIF: https://doi.org/10.15468/dl.zfaw79
Anopheles freeborni	-116.16	43.6084	Unclassified	GBIF: https://doi.org/10.15468/dl.zfaw79
Anopheles freeborni	-116.158	43.4625	Unclassified	GBIF: https://doi.org/10.15468/dl.zfaw79
Anopheles freeborni	-116.154	43.5751	Unclassified	GBIF: https://doi.org/10.15468/dl.zfaw79
Anopheles freeborni	-116.152	43.5741	Unclassified	GBIF: https://doi.org/10.15468/dl.zfaw79
Anopheles freeborni	-116.149	43.572	Unclassified	GBIF: https://doi.org/10.15468/dl.zfaw79
Anopheles freeborni	-116.142	43.5726	Unclassified	GBIF: https://doi.org/10.15468/dl.zfaw79
Anopheles freeborni	-116.142	43.5778	Unclassified	GBIF: https://doi.org/10.15468/dl.zfaw79
Anopheles freeborni	-116.141	43.5735	Unclassified	GBIF: https://doi.org/10.15468/dl.zfaw79
Anopheles freeborni	-116.139	43.5805	Unclassified	GBIF: https://doi.org/10.15468/dl.zfaw79
Anopheles freeborni	-116.137	43.5671	Unclassified	GBIF: https://doi.org/10.15468/dl.zfaw79
Anopheles freeborni	-116.133	43.5773	Unclassified	GBIF: https://doi.org/10.15468/dl.zfaw79
Anopheles freeborni	-116.132	43.5659	Unclassified	GBIF: https://doi.org/10.15468/dl.zfaw79
Anopheles freeborni	-116.131	43.566	Unclassified	GBIF: https://doi.org/10.15468/dl.zfaw79
Anopheles freeborni	-116.119	43.5534	Unclassified	GBIF: https://doi.org/10.15468/dl.zfaw79
Anopheles freeborni	-116.117	43.5527	Unclassified	GBIF: https://doi.org/10.15468/dl.zfaw79
Anopheles freeborni	-116.117	43.5647	Unclassified	GBIF: https://doi.org/10.15468/dl.zfaw79
Anopheles freeborni	-116.108	43.5466	Unclassified	GBIF: https://doi.org/10.15468/dl.zfaw79
Anopheles freeborni	-116.096	43.548	Unclassified	GBIF: https://doi.org/10.15468/dl.zfaw79
Anopheles freeborni	-115.467	36.0651	Unclassified	GBIF: https://doi.org/10.15468/dl.zfaw79
Anopheles freeborni	-115.407	47.6509	Unclassified	GBIF: https://doi.org/10.15468/dl.zfaw79
Anopheles freeborni	-115.394	36.0489	Unclassified	GBIF: https://doi.org/10.15468/dl.zfaw79
Anopheles freeborni	-115.394	36.0484	Unclassified	GBIF: https://doi.org/10.15468/dl.zfaw79
Anopheles freeborni	-115.394	36.0788	Unclassified	GBIF: https://doi.org/10.15468/dl.zfaw79
Anopheles freeborni	-115.352	36.4361	Unclassified	GBIF: https://doi.org/10.15468/dl.zfaw79
Anopheles freeborni	-115.269	36.3227	Unclassified	GBIF: https://doi.org/10.15468/dl.zfaw79
Anopheles freeborni	-115.242	36.0245	Unclassified	GBIF: https://doi.org/10.15468/dl.zfaw79

Anopheles freeborni	-115.24	36.0256	Unclassified	GBIF: https://doi.org/10.15468/dl.zfaw79
Anopheles freeborni	-115.238	36.0254	Unclassified	GBIF: https://doi.org/10.15468/dl.zfaw79
Anopheles freeborni	-115.235	36.0254	Unclassified	GBIF: https://doi.org/10.15468/dl.zfaw79
Anopheles freeborni	-115.234	36.0256	Unclassified	GBIF: https://doi.org/10.15468/dl.zfaw79
Anopheles freeborni	-115.234	36.0255	Unclassified	GBIF: https://doi.org/10.15468/dl.zfaw79
Anopheles freeborni	-115.233	37.5326	Unclassified	GBIF: https://doi.org/10.15468/dl.zfaw79
Anopheles freeborni	-115.233	37.5325	Unclassified	GBIF: https://doi.org/10.15468/dl.zfaw79
Anopheles freeborni	-115.233	37.5323	Unclassified	GBIF: https://doi.org/10.15468/dl.zfaw79
Anopheles freeborni	-115.233	37.5321	Unclassified	GBIF: https://doi.org/10.15468/dl.zfaw79
Anopheles freeborni	-115.221	37.5799	Unclassified	GBIF: https://doi.org/10.15468/dl.zfaw79
Anopheles freeborni	-115.146	36.1102	Unclassified	GBIF: https://doi.org/10.15468/dl.zfaw79
Anopheles freeborni	-115.14	37.3157	Unclassified	GBIF: https://doi.org/10.15468/dl.zfaw79
Anopheles freeborni	-115.133	37.3113	Unclassified	GBIF: https://doi.org/10.15468/dl.zfaw79
Anopheles freeborni	-115.131	37.3111	Unclassified	GBIF: https://doi.org/10.15468/dl.zfaw79
Anopheles freeborni	-115.131	37.3118	Unclassified	GBIF: https://doi.org/10.15468/dl.zfaw79
Anopheles freeborni	-115.131	37.3448	Unclassified	GBIF: https://doi.org/10.15468/dl.zfaw79
Anopheles freeborni	-115.13	37.3116	Unclassified	GBIF: https://doi.org/10.15468/dl.zfaw79
Anopheles freeborni	-115.129	37.3099	Unclassified	GBIF: https://doi.org/10.15468/dl.zfaw79
Anopheles freeborni	-115.126	37.3119	Unclassified	GBIF: https://doi.org/10.15468/dl.zfaw79
Anopheles freeborni	-115.125	37.3108	Unclassified	GBIF: https://doi.org/10.15468/dl.zfaw79
Anopheles freeborni	-115.125	37.3109	Unclassified	GBIF: https://doi.org/10.15468/dl.zfaw79
Anopheles freeborni	-115.122	37.2666	Unclassified	GBIF: https://doi.org/10.15468/dl.zfaw79
Anopheles freeborni	-115.118	37.2713	Unclassified	GBIF: https://doi.org/10.15468/dl.zfaw79
Anopheles freeborni	-115.11	36.0717	Unclassified	GBIF: https://doi.org/10.15468/dl.zfaw79
Anopheles freeborni	-115.109	36.0706	Unclassified	GBIF: https://doi.org/10.15468/dl.zfaw79
Anopheles freeborni	-115.108	36.09	Unclassified	GBIF: https://doi.org/10.15468/dl.zfaw79
Anopheles freeborni	-115.108	36.0701	Unclassified	GBIF: https://doi.org/10.15468/dl.zfaw79
Anopheles freeborni	-115.108	36.0699	Unclassified	GBIF: https://doi.org/10.15468/dl.zfaw79
Anopheles freeborni	-115.108	36.3071	Unclassified	GBIF: https://doi.org/10.15468/dl.zfaw79
Anopheles freeborni	-115.108	36.07	Unclassified	GBIF: https://doi.org/10.15468/dl.zfaw79
Anopheles freeborni	-115.107	36.0704	Unclassified	GBIF: https://doi.org/10.15468/dl.zfaw79
Anopheles freeborni	-115.106	36.0708	Unclassified	GBIF: https://doi.org/10.15468/dl.zfaw79
Anopheles freeborni	-115.105	36.0633	Unclassified	GBIF: https://doi.org/10.15468/dl.zfaw79
Anopheles freeborni	-115.084	36.0433	Unclassified	GBIF: https://doi.org/10.15468/dl.zfaw79
Anopheles freeborni	-115.084	36.0431	Unclassified	GBIF: https://doi.org/10.15468/dl.zfaw79
Anopheles freeborni	-115.084	36.0434	Unclassified	GBIF: https://doi.org/10.15468/dl.zfaw79
Anopheles freeborni	-115.084	36.0432	Unclassified	GBIF: https://doi.org/10.15468/dl.zfaw79
Anopheles freeborni	-115.076	38.4212	Unclassified	GBIF: https://doi.org/10.15468/dl.zfaw79
Anopheles freeborni	-115.073	37.1818	Unclassified	GBIF: https://doi.org/10.15468/dl.zfaw79
Anopheles freeborni	-115.072	37.156	Unclassified	GBIF: https://doi.org/10.15468/dl.zfaw79
Anopheles freeborni	-115.07	37.1629	Unclassified	GBIF: https://doi.org/10.15468/dl.zfaw79
Anopheles freeborni	-115.063	36.0657	Unclassified	GBIF: https://doi.org/10.15468/dl.zfaw79

Anopheles freeborni	-115.063	36.0774	Unclassified	GBIF: https://doi.org/10.15468/dl.zfaw79
Anopheles freeborni	-115.063	36.0801	Unclassified	GBIF: https://doi.org/10.15468/dl.zfaw79
Anopheles freeborni	-115.062	36.08	Unclassified	GBIF: https://doi.org/10.15468/dl.zfaw79
Anopheles freeborni	-115.061	36.0452	Unclassified	GBIF: https://doi.org/10.15468/dl.zfaw79
Anopheles freeborni	-115.06	36.0789	Unclassified	GBIF: https://doi.org/10.15468/dl.zfaw79
Anopheles freeborni	-115.06	36.0444	Unclassified	GBIF: https://doi.org/10.15468/dl.zfaw79
Anopheles freeborni	-115.058	36.0458	Unclassified	GBIF: https://doi.org/10.15468/dl.zfaw79
Anopheles freeborni	-115.058	36.046	Unclassified	GBIF: https://doi.org/10.15468/dl.zfaw79
Anopheles freeborni	-115.056	36.0465	Unclassified	GBIF: https://doi.org/10.15468/dl.zfaw79
Anopheles freeborni	-115.056	36.0463	Unclassified	GBIF: https://doi.org/10.15468/dl.zfaw79
Anopheles freeborni	-115.055	36.0472	Unclassified	GBIF: https://doi.org/10.15468/dl.zfaw79
Anopheles freeborni	-115.055	36.0506	Unclassified	GBIF: https://doi.org/10.15468/dl.zfaw79
Anopheles freeborni	-115.055	36.047	Unclassified	GBIF: https://doi.org/10.15468/dl.zfaw79
Anopheles freeborni	-115.055	36.0473	Unclassified	GBIF: https://doi.org/10.15468/dl.zfaw79
Anopheles freeborni	-115.055	36.0371	Unclassified	GBIF: https://doi.org/10.15468/dl.zfaw79
Anopheles freeborni	-115.055	36.0377	Unclassified	GBIF: https://doi.org/10.15468/dl.zfaw79
Anopheles freeborni	-115.055	36.0476	Unclassified	GBIF: https://doi.org/10.15468/dl.zfaw79
Anopheles freeborni	-115.055	36.0478	Unclassified	GBIF: https://doi.org/10.15468/dl.zfaw79
Anopheles freeborni	-115.055	36.0461	Unclassified	GBIF: https://doi.org/10.15468/dl.zfaw79
Anopheles freeborni	-115.055	36.0773	Unclassified	GBIF: https://doi.org/10.15468/dl.zfaw79
Anopheles freeborni	-115.054	36.0818	Unclassified	GBIF: https://doi.org/10.15468/dl.zfaw79
Anopheles freeborni	-115.054	36.0471	Unclassified	GBIF: https://doi.org/10.15468/dl.zfaw79
Anopheles freeborni	-115.054	36.0375	Unclassified	GBIF: https://doi.org/10.15468/dl.zfaw79
Anopheles freeborni	-115.054	36.0336	Unclassified	GBIF: https://doi.org/10.15468/dl.zfaw79
Anopheles freeborni	-115.054	36.0373	Unclassified	GBIF: https://doi.org/10.15468/dl.zfaw79
Anopheles freeborni	-115.054	36.037	Unclassified	GBIF: https://doi.org/10.15468/dl.zfaw79
Anopheles freeborni	-115.054	36.0371	Unclassified	GBIF: https://doi.org/10.15468/dl.zfaw79
Anopheles freeborni	-115.054	36.0372	Unclassified	GBIF: https://doi.org/10.15468/dl.zfaw79
Anopheles freeborni	-115.054	36.0763	Unclassified	GBIF: https://doi.org/10.15468/dl.zfaw79
Anopheles freeborni	-115.054	36.0342	Unclassified	GBIF: https://doi.org/10.15468/dl.zfaw79
Anopheles freeborni	-115.054	36.05	Unclassified	GBIF: https://doi.org/10.15468/dl.zfaw79
Anopheles freeborni	-115.053	36.0391	Unclassified	GBIF: https://doi.org/10.15468/dl.zfaw79
Anopheles freeborni	-115.053	36.0386	Unclassified	GBIF: https://doi.org/10.15468/dl.zfaw79
Anopheles freeborni	-115.053	36.0456	Unclassified	GBIF: https://doi.org/10.15468/dl.zfaw79
Anopheles freeborni	-115.053	48.8772	Unclassified	GBIF: https://doi.org/10.15468/dl.zfaw79
Anopheles freeborni	-115.053	36.0468	Unclassified	GBIF: https://doi.org/10.15468/dl.zfaw79
Anopheles freeborni	-115.053	36.0477	Unclassified	GBIF: https://doi.org/10.15468/dl.zfaw79
Anopheles freeborni	-115.053	36.0388	Unclassified	GBIF: https://doi.org/10.15468/dl.zfaw79
Anopheles freeborni	-115.053	36.0482	Unclassified	GBIF: https://doi.org/10.15468/dl.zfaw79
Anopheles freeborni	-115.052	36.0459	Unclassified	GBIF: https://doi.org/10.15468/dl.zfaw79
Anopheles freeborni	-115.052	36.0373	Unclassified	GBIF: https://doi.org/10.15468/dl.zfaw79
Anopheles freeborni	-115.052	36.046	Unclassified	GBIF: https://doi.org/10.15468/dl.zfaw79

Anopheles freeborni	-115.052	36.0361	Unclassified	GBIF: https://doi.org/10.15468/dl.zfaw79
Anopheles freeborni	-115.052	36.0458	Unclassified	GBIF: https://doi.org/10.15468/dl.zfaw79
Anopheles freeborni	-115.052	36.0359	Unclassified	GBIF: https://doi.org/10.15468/dl.zfaw79
Anopheles freeborni	-115.052	36.0363	Unclassified	GBIF: https://doi.org/10.15468/dl.zfaw79
Anopheles freeborni	-115.051	36.034	Unclassified	GBIF: https://doi.org/10.15468/dl.zfaw79
Anopheles freeborni	-115.051	36.047	Unclassified	GBIF: https://doi.org/10.15468/dl.zfaw79
Anopheles freeborni	-115.038	36.1111	Unclassified	GBIF: https://doi.org/10.15468/dl.zfaw79
Anopheles freeborni	-115.035	36.1039	Unclassified	GBIF: https://doi.org/10.15468/dl.zfaw79
Anopheles freeborni	-115.034	36.1039	Unclassified	GBIF: https://doi.org/10.15468/dl.zfaw79
Anopheles freeborni	-115.033	36.0723	Unclassified	GBIF: https://doi.org/10.15468/dl.zfaw79
Anopheles freeborni	-115.031	36.0737	Unclassified	GBIF: https://doi.org/10.15468/dl.zfaw79
Anopheles freeborni	-115.031	36.0839	Unclassified	GBIF: https://doi.org/10.15468/dl.zfaw79
Anopheles freeborni	-115.031	36.0741	Unclassified	GBIF: https://doi.org/10.15468/dl.zfaw79
Anopheles freeborni	-115.031	36.0742	Unclassified	GBIF: https://doi.org/10.15468/dl.zfaw79
Anopheles freeborni	-115.029	36.1033	Unclassified	GBIF: https://doi.org/10.15468/dl.zfaw79
Anopheles freeborni	-115.029	36.0719	Unclassified	GBIF: https://doi.org/10.15468/dl.zfaw79
Anopheles freeborni	-115.029	36.0713	Unclassified	GBIF: https://doi.org/10.15468/dl.zfaw79
Anopheles freeborni	-115.029	36.1006	Unclassified	GBIF: https://doi.org/10.15468/dl.zfaw79
Anopheles freeborni	-115.026	36.1007	Unclassified	GBIF: https://doi.org/10.15468/dl.zfaw79
Anopheles freeborni	-115.026	36.1006	Unclassified	GBIF: https://doi.org/10.15468/dl.zfaw79
Anopheles freeborni	-115.026	36.1005	Unclassified	GBIF: https://doi.org/10.15468/dl.zfaw79
Anopheles freeborni	-115.026	36.0738	Unclassified	GBIF: https://doi.org/10.15468/dl.zfaw79
Anopheles freeborni	-115.025	36.1024	Unclassified	GBIF: https://doi.org/10.15468/dl.zfaw79
Anopheles freeborni	-115.025	36.1031	Unclassified	GBIF: https://doi.org/10.15468/dl.zfaw79
Anopheles freeborni	-115.024	36.106	Unclassified	GBIF: https://doi.org/10.15468/dl.zfaw79
Anopheles freeborni	-115.024	36.1062	Unclassified	GBIF: https://doi.org/10.15468/dl.zfaw79
Anopheles freeborni	-115.024	36.1059	Unclassified	GBIF: https://doi.org/10.15468/dl.zfaw79
Anopheles freeborni	-115.023	36.0986	Unclassified	GBIF: https://doi.org/10.15468/dl.zfaw79
Anopheles freeborni	-115.023	36.1019	Unclassified	GBIF: https://doi.org/10.15468/dl.zfaw79
Anopheles freeborni	-115.023	36.1006	Unclassified	GBIF: https://doi.org/10.15468/dl.zfaw79
Anopheles freeborni	-115.023	36.1075	Unclassified	GBIF: https://doi.org/10.15468/dl.zfaw79
Anopheles freeborni	-115.023	36.1018	Unclassified	GBIF: https://doi.org/10.15468/dl.zfaw79
Anopheles freeborni	-115.022	36.1004	Unclassified	GBIF: https://doi.org/10.15468/dl.zfaw79
Anopheles freeborni	-115.022	36.0917	Unclassified	GBIF: https://doi.org/10.15468/dl.zfaw79
Anopheles freeborni	-115.022	36.1006	Unclassified	GBIF: https://doi.org/10.15468/dl.zfaw79
Anopheles freeborni	-115.022	36.1049	Unclassified	GBIF: https://doi.org/10.15468/dl.zfaw79
Anopheles freeborni	-115.021	36.0913	Unclassified	GBIF: https://doi.org/10.15468/dl.zfaw79
Anopheles freeborni	-115.021	36.1005	Unclassified	GBIF: https://doi.org/10.15468/dl.zfaw79
Anopheles freeborni	-115.021	36.092	Unclassified	GBIF: https://doi.org/10.15468/dl.zfaw79
Anopheles freeborni	-115.021	36.1047	Unclassified	GBIF: https://doi.org/10.15468/dl.zfaw79
Anopheles freeborni	-115.021	36.1008	Unclassified	GBIF: https://doi.org/10.15468/dl.zfaw79
Anopheles freeborni	-115.021	36.097	Unclassified	GBIF: https://doi.org/10.15468/dl.zfaw79

Anopheles freeborni	-115.02	36.1026	Unclassified	GBIF: https://doi.org/10.15468/dl.zfaw79
Anopheles freeborni	-115.02	36.1027	Unclassified	GBIF: https://doi.org/10.15468/dl.zfaw79
Anopheles freeborni	-115.02	36.104	Unclassified	GBIF: https://doi.org/10.15468/dl.zfaw79
Anopheles freeborni	-115.02	36.1039	Unclassified	GBIF: https://doi.org/10.15468/dl.zfaw79
Anopheles freeborni	-115.02	36.1007	Unclassified	GBIF: https://doi.org/10.15468/dl.zfaw79
Anopheles freeborni	-115.02	36.0976	Unclassified	GBIF: https://doi.org/10.15468/dl.zfaw79
Anopheles freeborni	-115.02	36.1047	Unclassified	GBIF: https://doi.org/10.15468/dl.zfaw79
Anopheles freeborni	-115.02	36.0888	Unclassified	GBIF: https://doi.org/10.15468/dl.zfaw79
Anopheles freeborni	-115.02	36.1021	Unclassified	GBIF: https://doi.org/10.15468/dl.zfaw79
Anopheles freeborni	-115.019	36.1001	Unclassified	GBIF: https://doi.org/10.15468/dl.zfaw79
Anopheles freeborni	-115.019	36.1002	Unclassified	GBIF: https://doi.org/10.15468/dl.zfaw79
Anopheles freeborni	-115.019	36.9959	Unclassified	GBIF: https://doi.org/10.15468/dl.zfaw79
Anopheles freeborni	-115.019	36.1006	Unclassified	GBIF: https://doi.org/10.15468/dl.zfaw79
Anopheles freeborni	-115.019	36.1026	Unclassified	GBIF: https://doi.org/10.15468/dl.zfaw79
Anopheles freeborni	-115.019	36.0996	Unclassified	GBIF: https://doi.org/10.15468/dl.zfaw79
Anopheles freeborni	-115.019	36.1019	Unclassified	GBIF: https://doi.org/10.15468/dl.zfaw79
Anopheles freeborni	-115.019	36.1034	Unclassified	GBIF: https://doi.org/10.15468/dl.zfaw79
Anopheles freeborni	-115.018	36.1023	Unclassified	GBIF: https://doi.org/10.15468/dl.zfaw79
Anopheles freeborni	-115.018	36.0942	Unclassified	GBIF: https://doi.org/10.15468/dl.zfaw79
Anopheles freeborni	-115.017	36.0943	Unclassified	GBIF: https://doi.org/10.15468/dl.zfaw79
Anopheles freeborni	-115.017	36.0807	Unclassified	GBIF: https://doi.org/10.15468/dl.zfaw79
Anopheles freeborni	-115.017	36.0906	Unclassified	GBIF: https://doi.org/10.15468/dl.zfaw79
Anopheles freeborni	-115.017	36.0933	Unclassified	GBIF: https://doi.org/10.15468/dl.zfaw79
Anopheles freeborni	-115.017	36.0905	Unclassified	GBIF: https://doi.org/10.15468/dl.zfaw79
Anopheles freeborni	-115.015	36.0929	Unclassified	GBIF: https://doi.org/10.15468/dl.zfaw79
Anopheles freeborni	-115.014	36.0927	Unclassified	GBIF: https://doi.org/10.15468/dl.zfaw79
Anopheles freeborni	-115.013	36.0826	Unclassified	GBIF: https://doi.org/10.15468/dl.zfaw79
Anopheles freeborni	-115.009	36.0794	Unclassified	GBIF: https://doi.org/10.15468/dl.zfaw79
Anopheles freeborni	-115.003	36.0971	Unclassified	GBIF: https://doi.org/10.15468/dl.zfaw79
Anopheles freeborni	-115.003	36.059	Unclassified	GBIF: https://doi.org/10.15468/dl.zfaw79
Anopheles freeborni	-115.002	36.0786	Unclassified	GBIF: https://doi.org/10.15468/dl.zfaw79
Anopheles freeborni	-115	36.0808	Unclassified	GBIF: https://doi.org/10.15468/dl.zfaw79
Anopheles freeborni	-115	36.0809	Unclassified	GBIF: https://doi.org/10.15468/dl.zfaw79
Anopheles freeborni	-114.991	36.0796	Unclassified	GBIF: https://doi.org/10.15468/dl.zfaw79
Anopheles freeborni	-114.991	36.0555	Unclassified	GBIF: https://doi.org/10.15468/dl.zfaw79
Anopheles freeborni	-114.975	36.0893	Unclassified	GBIF: https://doi.org/10.15468/dl.zfaw79
Anopheles freeborni	-114.974	36.0893	Unclassified	GBIF: https://doi.org/10.15468/dl.zfaw79
Anopheles freeborni	-114.973	36.0898	Unclassified	GBIF: https://doi.org/10.15468/dl.zfaw79
Anopheles freeborni	-114.967	36.0912	Unclassified	GBIF: https://doi.org/10.15468/dl.zfaw79
Anopheles freeborni	-114.958	36.0866	Unclassified	GBIF: https://doi.org/10.15468/dl.zfaw79
Anopheles freeborni	-114.937	36.1038	Unclassified	GBIF: https://doi.org/10.15468/dl.zfaw79
Anopheles freeborni	-114.937	36.103	Unclassified	GBIF: https://doi.org/10.15468/dl.zfaw79

Anopheles freeborni	-114.936	36.1038	Unclassified	GBIF: https://doi.org/10.15468/dl.zfaw79
Anopheles freeborni	-114.928	36.1029	Unclassified	GBIF: https://doi.org/10.15468/dl.zfaw79
Anopheles freeborni	-114.872	39.2638	Unclassified	GBIF: https://doi.org/10.15468/dl.zfaw79
Anopheles freeborni	-114.869	36.1287	Unclassified	GBIF: https://doi.org/10.15468/dl.zfaw79
Anopheles freeborni	-114.77	36.5441	Unclassified	GBIF: https://doi.org/10.15468/dl.zfaw79
Anopheles freeborni	-114.717	36.7263	Unclassified	GBIF: https://doi.org/10.15468/dl.zfaw79
Anopheles freeborni	-114.717	36.726	Unclassified	GBIF: https://doi.org/10.15468/dl.zfaw79
Anopheles freeborni	-114.716	36.7191	Unclassified	GBIF: https://doi.org/10.15468/dl.zfaw79
Anopheles freeborni	-114.716	36.7189	Unclassified	GBIF: https://doi.org/10.15468/dl.zfaw79
Anopheles freeborni	-114.695	36.7106	Unclassified	GBIF: https://doi.org/10.15468/dl.zfaw79
Anopheles freeborni	-114.694	36.7106	Unclassified	GBIF: https://doi.org/10.15468/dl.zfaw79
Anopheles freeborni	-114.694	36.7099	Unclassified	GBIF: https://doi.org/10.15468/dl.zfaw79
Anopheles freeborni	-114.685	36.6402	Unclassified	GBIF: https://doi.org/10.15468/dl.zfaw79
Anopheles freeborni	-114.681	36.7036	Unclassified	GBIF: https://doi.org/10.15468/dl.zfaw79
Anopheles freeborni	-114.672	36.5679	Unclassified	GBIF: https://doi.org/10.15468/dl.zfaw79
Anopheles freeborni	-114.663	36.6785	Unclassified	GBIF: https://doi.org/10.15468/dl.zfaw79
Anopheles freeborni	-114.662	36.6781	Unclassified	GBIF: https://doi.org/10.15468/dl.zfaw79
Anopheles freeborni	-114.654	36.6656	Unclassified	GBIF: https://doi.org/10.15468/dl.zfaw79
Anopheles freeborni	-114.586	36.6737	Unclassified	GBIF: https://doi.org/10.15468/dl.zfaw79
Anopheles freeborni	-114.582	36.6705	Unclassified	GBIF: https://doi.org/10.15468/dl.zfaw79
Anopheles freeborni	-114.582	36.6704	Unclassified	GBIF: https://doi.org/10.15468/dl.zfaw79
Anopheles freeborni	-114.582	36.6701	Unclassified	GBIF: https://doi.org/10.15468/dl.zfaw79
Anopheles freeborni	-114.526	37.6113	Unclassified	GBIF: https://doi.org/10.15468/dl.zfaw79
Anopheles freeborni	-114.526	37.6109	Unclassified	GBIF: https://doi.org/10.15468/dl.zfaw79
Anopheles freeborni	-114.526	37.6111	Unclassified	GBIF: https://doi.org/10.15468/dl.zfaw79
Anopheles freeborni	-114.525	37.6112	Unclassified	GBIF: https://doi.org/10.15468/dl.zfaw79
Anopheles freeborni	-114.519	37.6155	Unclassified	GBIF: https://doi.org/10.15468/dl.zfaw79
Anopheles freeborni	-114.515	37.6166	Unclassified	GBIF: https://doi.org/10.15468/dl.zfaw79
Anopheles freeborni	-114.515	37.6167	Unclassified	GBIF: https://doi.org/10.15468/dl.zfaw79
Anopheles freeborni	-114.515	37.6173	Unclassified	GBIF: https://doi.org/10.15468/dl.zfaw79
Anopheles freeborni	-114.514	37.6174	Unclassified	GBIF: https://doi.org/10.15468/dl.zfaw79
Anopheles freeborni	-114.509	37.6199	Unclassified	GBIF: https://doi.org/10.15468/dl.zfaw79
Anopheles freeborni	-114.497	37.6543	Unclassified	GBIF: https://doi.org/10.15468/dl.zfaw79
Anopheles freeborni	-114.497	37.6545	Unclassified	GBIF: https://doi.org/10.15468/dl.zfaw79
Anopheles freeborni	-114.497	37.6544	Unclassified	GBIF: https://doi.org/10.15468/dl.zfaw79
Anopheles freeborni	-114.497	37.6546	Unclassified	GBIF: https://doi.org/10.15468/dl.zfaw79
Anopheles freeborni	-114.497	37.6542	Unclassified	GBIF: https://doi.org/10.15468/dl.zfaw79
Anopheles freeborni	-114.481	36.6206	Unclassified	GBIF: https://doi.org/10.15468/dl.zfaw79
Anopheles freeborni	-114.45	36.5551	Unclassified	GBIF: https://doi.org/10.15468/dl.zfaw79
Anopheles freeborni	-114.443	36.5461	Unclassified	GBIF: https://doi.org/10.15468/dl.zfaw79
Anopheles freeborni	-114.443	36.5462	Unclassified	GBIF: https://doi.org/10.15468/dl.zfaw79
Anopheles freeborni	-114.439	36.5419	Unclassified	GBIF: https://doi.org/10.15468/dl.zfaw79

Anopheles freeborni	-114.439	36.542	Unclassified	GBIF: https://doi.org/10.15468/dl.zfaw79
Anopheles freeborni	-114.437	36.5519	Unclassified	GBIF: https://doi.org/10.15468/dl.zfaw79
Anopheles freeborni	-114.429	36.5355	Unclassified	GBIF: https://doi.org/10.15468/dl.zfaw79
Anopheles freeborni	-114.429	36.5354	Unclassified	GBIF: https://doi.org/10.15468/dl.zfaw79
Anopheles freeborni	-114.428	36.5266	Unclassified	GBIF: https://doi.org/10.15468/dl.zfaw79
Anopheles freeborni	-114.426	36.5217	Unclassified	GBIF: https://doi.org/10.15468/dl.zfaw79
Anopheles freeborni	-114.422	36.5151	Unclassified	GBIF: https://doi.org/10.15468/dl.zfaw79
Anopheles freeborni	-114.421	36.5149	Unclassified	GBIF: https://doi.org/10.15468/dl.zfaw79
Anopheles freeborni	-114.421	36.5156	Unclassified	GBIF: https://doi.org/10.15468/dl.zfaw79
Anopheles freeborni	-114.415807	36.440631	Unclassified	GBIF: https://doi.org/10.15468/dl.zfaw79
Anopheles freeborni	-114.392	37.7903	Unclassified	GBIF: https://doi.org/10.15468/dl.zfaw79
Anopheles freeborni	-114.382	37.807	Unclassified	GBIF: https://doi.org/10.15468/dl.zfaw79
Anopheles freeborni	-114.382	37.8075	Unclassified	GBIF: https://doi.org/10.15468/dl.zfaw79
Anopheles freeborni	-114.382	37.8069	Unclassified	GBIF: https://doi.org/10.15468/dl.zfaw79
Anopheles freeborni	-114.381	37.8066	Unclassified	GBIF: https://doi.org/10.15468/dl.zfaw79
Anopheles freeborni	-114.381	37.7903	Unclassified	GBIF: https://doi.org/10.15468/dl.zfaw79
Anopheles freeborni	-114.341	48.3343	Unclassified	GBIF: https://doi.org/10.15468/dl.zfaw79
Anopheles freeborni	-114.313	37.367	Unclassified	GBIF: https://doi.org/10.15468/dl.zfaw79
Anopheles freeborni	-114.313	37.364	Unclassified	GBIF: https://doi.org/10.15468/dl.zfaw79
Anopheles freeborni	-114.307	48.2088	Unclassified	GBIF: https://doi.org/10.15468/dl.zfaw79
Anopheles freeborni	-114.305	37.37	Unclassified	GBIF: https://doi.org/10.15468/dl.zfaw79
Anopheles freeborni	-114.295	37.3916	Unclassified	GBIF: https://doi.org/10.15468/dl.zfaw79
Anopheles freeborni	-114.285	36.3714	Unclassified	GBIF: https://doi.org/10.15468/dl.zfaw79
Anopheles freeborni	-114.279	47.4708	Unclassified	GBIF: https://doi.org/10.15468/dl.zfaw79
Anopheles freeborni	-114.277	48.1727	Unclassified	GBIF: https://doi.org/10.15468/dl.zfaw79
Anopheles freeborni	-114.233	37.4725	Unclassified	GBIF: https://doi.org/10.15468/dl.zfaw79
Anopheles freeborni	-114.225	37.4824	Unclassified	GBIF: https://doi.org/10.15468/dl.zfaw79
Anopheles freeborni	-114.216	37.983	Unclassified	GBIF: https://doi.org/10.15468/dl.zfaw79
Anopheles freeborni	-114.169	46.0239	Unclassified	GBIF: https://doi.org/10.15468/dl.zfaw79
Anopheles freeborni	-114.162529	46.242519	Unclassified	GBIF: https://doi.org/10.15468/dl.zfaw79
Anopheles freeborni	-114.154	46.9143	Unclassified	GBIF: https://doi.org/10.15468/dl.zfaw79
Anopheles freeborni	-114.153	36.7669	Unclassified	GBIF: https://doi.org/10.15468/dl.zfaw79
Anopheles freeborni	-114.152	36.7679	Unclassified	GBIF: https://doi.org/10.15468/dl.zfaw79
Anopheles freeborni	-114.148	47.4504	Unclassified	GBIF: https://doi.org/10.15468/dl.zfaw79
Anopheles freeborni	-114.147	36.7635	Unclassified	GBIF: https://doi.org/10.15468/dl.zfaw79
Anopheles freeborni	-114.144	47.4321	Unclassified	GBIF: https://doi.org/10.15468/dl.zfaw79
Anopheles freeborni	-114.137	47.4456	Unclassified	GBIF: https://doi.org/10.15468/dl.zfaw79
Anopheles freeborni	-114.13	36.78	Unclassified	GBIF: https://doi.org/10.15468/dl.zfaw79
Anopheles freeborni	-114.13	36.7747	Unclassified	GBIF: https://doi.org/10.15468/dl.zfaw79
Anopheles freeborni	-114.129	47.5638	Unclassified	GBIF: https://doi.org/10.15468/dl.zfaw79
Anopheles freeborni	-114.129	36.7734	Unclassified	GBIF: https://doi.org/10.15468/dl.zfaw79
Anopheles freeborni	-114.128	36.7744	Unclassified	GBIF: https://doi.org/10.15468/dl.zfaw79

Anopheles freeborni	-114.128	47.5646	Unclassified	GBIF: https://doi.org/10.15468/dl.zfaw79
Anopheles freeborni	-114.126	37.5859	Unclassified	GBIF: https://doi.org/10.15468/dl.zfaw79
Anopheles freeborni	-114.115	36.7921	Unclassified	GBIF: https://doi.org/10.15468/dl.zfaw79
Anopheles freeborni	-114.114	36.793	Unclassified	GBIF: https://doi.org/10.15468/dl.zfaw79
Anopheles freeborni	-114.107	37.2441	Unclassified	GBIF: https://doi.org/10.15468/dl.zfaw79
Anopheles freeborni	-114.105	46.5196	Unclassified	GBIF: https://doi.org/10.15468/dl.zfaw79
Anopheles freeborni	-114.101	36.7961	Unclassified	GBIF: https://doi.org/10.15468/dl.zfaw79
Anopheles freeborni	-114.101	36.7962	Unclassified	GBIF: https://doi.org/10.15468/dl.zfaw79
Anopheles freeborni	-114.098	47.4459	Unclassified	GBIF: https://doi.org/10.15468/dl.zfaw79
Anopheles freeborni	-114.097	48.0714	Unclassified	GBIF: https://doi.org/10.15468/dl.zfaw79
Anopheles freeborni	-114.093	36.7895	Unclassified	GBIF: https://doi.org/10.15468/dl.zfaw79
Anopheles freeborni	-114.091	36.7896	Unclassified	GBIF: https://doi.org/10.15468/dl.zfaw79
Anopheles freeborni	-114.085	36.7932	Unclassified	GBIF: https://doi.org/10.15468/dl.zfaw79
Anopheles freeborni	-114.084	36.794	Unclassified	GBIF: https://doi.org/10.15468/dl.zfaw79
Anopheles freeborni	-114.083	36.794	Unclassified	GBIF: https://doi.org/10.15468/dl.zfaw79
Anopheles freeborni	-114.074	46.4986	Unclassified	GBIF: https://doi.org/10.15468/dl.zfaw79
Anopheles freeborni	-114.071	46.5856	Unclassified	GBIF: https://doi.org/10.15468/dl.zfaw79
Anopheles freeborni	-114.067	36.8	Unclassified	GBIF: https://doi.org/10.15468/dl.zfaw79
Anopheles freeborni	-114.066	36.8077	Unclassified	GBIF: https://doi.org/10.15468/dl.zfaw79
Anopheles freeborni	-114.066	36.8003	Unclassified	GBIF: https://doi.org/10.15468/dl.zfaw79
Anopheles freeborni	-114.064	36.8161	Unclassified	GBIF: https://doi.org/10.15468/dl.zfaw79
Anopheles freeborni	-114.063	36.8163	Unclassified	GBIF: https://doi.org/10.15468/dl.zfaw79
Anopheles freeborni	-114.062	46.8402	Unclassified	GBIF: https://doi.org/10.15468/dl.zfaw79
Anopheles freeborni	-114.062	36.8025	Unclassified	GBIF: https://doi.org/10.15468/dl.zfaw79
Anopheles freeborni	-114.062	36.8024	Unclassified	GBIF: https://doi.org/10.15468/dl.zfaw79
Anopheles freeborni	-114.062	36.8027	Unclassified	GBIF: https://doi.org/10.15468/dl.zfaw79
Anopheles freeborni	-114.059	46.6386	Unclassified	GBIF: https://doi.org/10.15468/dl.zfaw79
Anopheles freeborni	-114.034	36.481	Unclassified	GBIF: https://doi.org/10.15468/dl.zfaw79
Anopheles freeborni	-113.821	47.7479	Unclassified	GBIF: https://doi.org/10.15468/dl.zfaw79
Anopheles freeborni	-113.807	47.7517	Unclassified	GBIF: https://doi.org/10.15468/dl.zfaw79
Anopheles freeborni	-113.601	46.7232	Unclassified	GBIF: https://doi.org/10.15468/dl.zfaw79
Anopheles freeborni	-113.147	46.6595	Unclassified	GBIF: https://doi.org/10.15468/dl.zfaw79
Anopheles freeborni	-113.014	46.9039	Unclassified	GBIF: https://doi.org/10.15468/dl.zfaw79
Anopheles freeborni	-113.012	46.9108	Unclassified	GBIF: https://doi.org/10.15468/dl.zfaw79
Anopheles freeborni	-112.975	46.9678	Unclassified	GBIF: https://doi.org/10.15468/dl.zfaw79
Anopheles freeborni	-112.972	46.965	Unclassified	GBIF: https://doi.org/10.15468/dl.zfaw79
Anopheles freeborni	-112.931	46.591	Unclassified	GBIF: https://doi.org/10.15468/dl.zfaw79
Anopheles freeborni	-112.577055	28.700744	Unclassified	GBIF: https://doi.org/10.15468/dl.zfaw79
Anopheles freeborni	-112.1903	35.24944	Unclassified	GBIF: https://doi.org/10.15468/dl.zfaw79
Anopheles freeborni	-112.122	40.7948	Unclassified	GBIF: https://doi.org/10.15468/dl.zfaw79
Anopheles freeborni	-112.114	40.8263	Unclassified	GBIF: https://doi.org/10.15468/dl.zfaw79
Anopheles freeborni	-112.109	40.7844	Unclassified	GBIF: https://doi.org/10.15468/dl.zfaw79

Anopheles freeborni	-112.101	40.816	Unclassified	GBIF: https://doi.org/10.15468/dl.zfaw79
Anopheles freeborni	-112.083	40.7689	Unclassified	GBIF: https://doi.org/10.15468/dl.zfaw79
Anopheles freeborni	-112.069	40.8241	Unclassified	GBIF: https://doi.org/10.15468/dl.zfaw79
Anopheles freeborni	-112.062	40.7877	Unclassified	GBIF: https://doi.org/10.15468/dl.zfaw79
Anopheles freeborni	-112.059	40.8243	Unclassified	GBIF: https://doi.org/10.15468/dl.zfaw79
Anopheles freeborni	-112.05	40.8021	Unclassified	GBIF: https://doi.org/10.15468/dl.zfaw79
Anopheles freeborni	-112.032	40.831	Unclassified	GBIF: https://doi.org/10.15468/dl.zfaw79
Anopheles freeborni	-112.029	40.7787	Unclassified	GBIF: https://doi.org/10.15468/dl.zfaw79
Anopheles freeborni	-112.029	40.846	Unclassified	GBIF: https://doi.org/10.15468/dl.zfaw79
Anopheles freeborni	-112.023	40.8699	Unclassified	GBIF: https://doi.org/10.15468/dl.zfaw79
Anopheles freeborni	-112.017	40.8067	Unclassified	GBIF: https://doi.org/10.15468/dl.zfaw79
Anopheles freeborni	-112.016816	40.806833	Unclassified	GBIF: https://doi.org/10.15468/dl.zfaw79
Anopheles freeborni	-112.015	40.822	Unclassified	GBIF: https://doi.org/10.15468/dl.zfaw79
Anopheles freeborni	-112.014242	40.792855	Unclassified	GBIF: https://doi.org/10.15468/dl.zfaw79
Anopheles freeborni	-112.012	40.7933	Unclassified	GBIF: https://doi.org/10.15468/dl.zfaw79
Anopheles freeborni	-112.007	40.8077	Unclassified	GBIF: https://doi.org/10.15468/dl.zfaw79
Anopheles freeborni	-112.004	40.7542	Unclassified	GBIF: https://doi.org/10.15468/dl.zfaw79
Anopheles freeborni	-112.003	40.8445	Unclassified	GBIF: https://doi.org/10.15468/dl.zfaw79
Anopheles freeborni	-111.986	40.8299	Unclassified	GBIF: https://doi.org/10.15468/dl.zfaw79
Anopheles freeborni	-111.982	40.8459	Unclassified	GBIF: https://doi.org/10.15468/dl.zfaw79
Anopheles freeborni	-111.978	46.6609	Unclassified	GBIF: https://doi.org/10.15468/dl.zfaw79
Anopheles freeborni	-111.977	40.7715	Unclassified	GBIF: https://doi.org/10.15468/dl.zfaw79
Anopheles freeborni	-111.97	40.8418	Unclassified	GBIF: https://doi.org/10.15468/dl.zfaw79
Anopheles freeborni	-111.962	40.8469	Unclassified	GBIF: https://doi.org/10.15468/dl.zfaw79
Anopheles freeborni	-111.953	40.8421	Unclassified	GBIF: https://doi.org/10.15468/dl.zfaw79
Anopheles freeborni	-111.946	47.1097	Unclassified	GBIF: https://doi.org/10.15468/dl.zfaw79
Anopheles freeborni	-111.944	40.8265	Unclassified	GBIF: https://doi.org/10.15468/dl.zfaw79
Anopheles freeborni	-111.944	46.6683	Unclassified	GBIF: https://doi.org/10.15468/dl.zfaw79
Anopheles freeborni	-111.942	40.8157	Unclassified	GBIF: https://doi.org/10.15468/dl.zfaw79
Anopheles freeborni	-111.94	40.8086	Unclassified	GBIF: https://doi.org/10.15468/dl.zfaw79
Anopheles freeborni	-111.937	40.8001	Unclassified	GBIF: https://doi.org/10.15468/dl.zfaw79
Anopheles freeborni	-111.937	40.8124	Unclassified	GBIF: https://doi.org/10.15468/dl.zfaw79
Anopheles freeborni	-111.932	40.7276	Unclassified	GBIF: https://doi.org/10.15468/dl.zfaw79
Anopheles freeborni	-111.923	40.7723	Unclassified	GBIF: https://doi.org/10.15468/dl.zfaw79
Anopheles freeborni	-111.875	46.6446	Unclassified	GBIF: https://doi.org/10.15468/dl.zfaw79
Anopheles freeborni	-111.873	40.7104	Unclassified	GBIF: https://doi.org/10.15468/dl.zfaw79
Anopheles freeborni	-111.861	40.75	Unclassified	GBIF: https://doi.org/10.15468/dl.zfaw79
Anopheles freeborni	-111.7269	34.91238	Unclassified	GBIF: https://doi.org/10.15468/dl.zfaw79
Anopheles freeborni	-111.726	46.8522	Unclassified	GBIF: https://doi.org/10.15468/dl.zfaw79
Anopheles freeborni	-111.705528	40.2384	Unclassified	GBIF: https://doi.org/10.15468/dl.zfaw79
Anopheles freeborni	-111.66	45.8391	Unclassified	GBIF: https://doi.org/10.15468/dl.zfaw79
Anopheles freeborni	-111.56	46.3833	Unclassified	GBIF: https://doi.org/10.15468/dl.zfaw79

Anopheles freeborni	-111.497	45.9032	Unclassified	GBIF: https://doi.org/10.15468/dl.zfaw79
Anopheles freeborni	-111.484	46.3846	Unclassified	GBIF: https://doi.org/10.15468/dl.zfaw79
Anopheles freeborni	-110.856389	30.778056	Unclassified	GBIF: https://doi.org/10.15468/dl.zfaw79
Anopheles freeborni	-110.294517	31.967861	Unclassified	GBIF: https://doi.org/10.15468/dl.zfaw79
Anopheles freeborni	-109.971	45.8375	Unclassified	GBIF: https://doi.org/10.15468/dl.zfaw79
Anopheles freeborni	-109.713	48.561	Unclassified	GBIF: https://doi.org/10.15468/dl.zfaw79
Anopheles freeborni	-109.400608	38.23527	Unclassified	GBIF: https://doi.org/10.15468/dl.zfaw79
Anopheles freeborni	-109.39442	38.221342	Unclassified	GBIF: https://doi.org/10.15468/dl.zfaw79
Anopheles freeborni	-109.39148	38.230243	Unclassified	GBIF: https://doi.org/10.15468/dl.zfaw79
Anopheles freeborni	-109.388273	38.248283	Unclassified	GBIF: https://doi.org/10.15468/dl.zfaw79
Anopheles freeborni	-109.38162	38.25792	Unclassified	GBIF: https://doi.org/10.15468/dl.zfaw79
Anopheles freeborni	-109.350332	38.277972	Unclassified	GBIF: https://doi.org/10.15468/dl.zfaw79
Anopheles freeborni	-109.35	38.278	Unclassified	GBIF: https://doi.org/10.15468/dl.zfaw79
Anopheles freeborni	-109.3122	31.51056	Unclassified	GBIF: https://doi.org/10.15468/dl.zfaw79
Anopheles freeborni	-109.305	46.2985	Unclassified	GBIF: https://doi.org/10.15468/dl.zfaw79
Anopheles freeborni	-109.251	45.6284	Unclassified	GBIF: https://doi.org/10.15468/dl.zfaw79
Anopheles freeborni	-108.874496	31.524546	Unclassified	GBIF: https://doi.org/10.15468/dl.zfaw79
Anopheles freeborni	-108.857	45.6686	Unclassified	GBIF: https://doi.org/10.15468/dl.zfaw79
Anopheles freeborni	-108.767	45.6815	Unclassified	GBIF: https://doi.org/10.15468/dl.zfaw79
Anopheles freeborni	-108.684	48.4909	Unclassified	GBIF: https://doi.org/10.15468/dl.zfaw79
Anopheles freeborni	-108.528	45.4367	Unclassified	GBIF: https://doi.org/10.15468/dl.zfaw79
Anopheles freeborni	-108.525	45.7464	Unclassified	GBIF: https://doi.org/10.15468/dl.zfaw79
Anopheles freeborni	-108.434	45.8348	Unclassified	GBIF: https://doi.org/10.15468/dl.zfaw79
Anopheles freeborni	-108.266	45.7908	Unclassified	GBIF: https://doi.org/10.15468/dl.zfaw79
Anopheles freeborni	-108.245	45.9227	Unclassified	GBIF: https://doi.org/10.15468/dl.zfaw79
Anopheles freeborni	-107.729	48.3871	Unclassified	GBIF: https://doi.org/10.15468/dl.zfaw79
Anopheles freeborni	-107.716	46.072	Unclassified	GBIF: https://doi.org/10.15468/dl.zfaw79
Anopheles freeborni	-107.66	48.4177	Unclassified	GBIF: https://doi.org/10.15468/dl.zfaw79
Anopheles freeborni	-107.59	45.7313	Unclassified	GBIF: https://doi.org/10.15468/dl.zfaw79
Anopheles freeborni	-107.571	45.7567	Unclassified	GBIF: https://doi.org/10.15468/dl.zfaw79
Anopheles freeborni	-107.453	45.6032	Unclassified	GBIF: https://doi.org/10.15468/dl.zfaw79
Anopheles freeborni	-107.416	45.119	Unclassified	GBIF: https://doi.org/10.15468/dl.zfaw79
Anopheles freeborni	-107.363	45.3271	Unclassified	GBIF: https://doi.org/10.15468/dl.zfaw79
Anopheles freeborni	-107.145	45.9524	Unclassified	GBIF: https://doi.org/10.15468/dl.zfaw79
Anopheles freeborni	-107.117	45.8341	Unclassified	GBIF: https://doi.org/10.15468/dl.zfaw79
Anopheles freeborni	-106.649	48.2009	Unclassified	GBIF: https://doi.org/10.15468/dl.zfaw79
Anopheles freeborni	-106.647	48.1765	Unclassified	GBIF: https://doi.org/10.15468/dl.zfaw79
Anopheles freeborni	-105.418	48.1302	Unclassified	GBIF: https://doi.org/10.15468/dl.zfaw79
Anopheles freeborni	-104.583	48.3678	Unclassified	GBIF: https://doi.org/10.15468/dl.zfaw79
Anopheles freeborni	-104.45	48.4825	Unclassified	GBIF: https://doi.org/10.15468/dl.zfaw79
Anopheles freeborni	-101.672361	19.236778	Unclassified	GBIF: https://doi.org/10.15468/dl.zfaw79
Anopheles freeborni	-100.374472	19.539472	Unclassified	GBIF: https://doi.org/10.15468/dl.zfaw79

Anopheles freetownensis	3.33254	7.18376	Unclassified	GBIF: https://doi.org/10.15468/dl.zfaw79
Anopheles freetownensis	3.33254	6.58381	Unclassified	GBIF: https://doi.org/10.15468/dl.zfaw79
Anopheles freetownensis	3.39921	6.45049	Unclassified	GBIF: https://doi.org/10.15468/dl.zfaw79
Anopheles freetownensis	6.21592	7.56706	Unclassified	GBIF: https://doi.org/10.15468/dl.zfaw79
Anopheles freetownensis	6.73259	7.80037	Unclassified	GBIF: https://doi.org/10.15468/dl.zfaw79
Anopheles freetownensis	6.7826	6.16719	Unclassified	GBIF: https://doi.org/10.15468/dl.zfaw79
Anopheles freetownensis	8.89929	9.86685	Unclassified	GBIF: https://doi.org/10.15468/dl.zfaw79
Anopheles freetownensis	9.28264	5.71724	Unclassified	GBIF: https://doi.org/10.15468/dl.zfaw79
Anopheles funestus	-17.47	14.71	Unclassified	GBIF: https://doi.org/10.15468/dl.zfaw79
Anopheles funestus	-17.283333	14.766667	Unclassified	GBIF: https://doi.org/10.15468/dl.zfaw79
Anopheles funestus	-10.353433	6.240148	Unclassified	GBIF: https://doi.org/10.15468/dl.zfaw79
Anopheles funestus	-7.1	13.85	Unclassified	GBIF: https://doi.org/10.15468/dl.zfaw79
Anopheles funestus	-5.779912	9.167013	Unclassified	GBIF: https://doi.org/10.15468/dl.zfaw79
Anopheles funestus	-5.779229	9.167393	Unclassified	GBIF: https://doi.org/10.15468/dl.zfaw79
Anopheles funestus	-5.779149	9.167072	Unclassified	GBIF: https://doi.org/10.15468/dl.zfaw79
Anopheles funestus	-5.778431	9.16805	Unclassified	GBIF: https://doi.org/10.15468/dl.zfaw79
Anopheles funestus	-5.778425	9.168052	Unclassified	GBIF: https://doi.org/10.15468/dl.zfaw79
Anopheles funestus	-5.761149	9.255368	Unclassified	GBIF: https://doi.org/10.15468/dl.zfaw79
Anopheles funestus	-5.761018	9.256659	Unclassified	GBIF: https://doi.org/10.15468/dl.zfaw79
Anopheles funestus	-5.760093	9.255427	Unclassified	GBIF: https://doi.org/10.15468/dl.zfaw79
Anopheles funestus	-5.759284	9.254708	Unclassified	GBIF: https://doi.org/10.15468/dl.zfaw79
Anopheles funestus	-5.757217	9.210645	Unclassified	GBIF: https://doi.org/10.15468/dl.zfaw79
Anopheles funestus	-5.756871	9.210201	Unclassified	GBIF: https://doi.org/10.15468/dl.zfaw79
Anopheles funestus	-5.756218	9.210008	Unclassified	GBIF: https://doi.org/10.15468/dl.zfaw79
Anopheles funestus	-5.748389	9.059743	Unclassified	GBIF: https://doi.org/10.15468/dl.zfaw79
Anopheles funestus	-5.748355	9.060641	Unclassified	GBIF: https://doi.org/10.15468/dl.zfaw79
Anopheles funestus	-5.748215	9.060273	Unclassified	GBIF: https://doi.org/10.15468/dl.zfaw79
Anopheles funestus	-5.747999	9.060559	Unclassified	GBIF: https://doi.org/10.15468/dl.zfaw79
Anopheles funestus	-5.740081	9.235433	Unclassified	GBIF: https://doi.org/10.15468/dl.zfaw79
Anopheles funestus	-5.740062	9.235212	Unclassified	GBIF: https://doi.org/10.15468/dl.zfaw79
Anopheles funestus	-5.739909	9.23508	Unclassified	GBIF: https://doi.org/10.15468/dl.zfaw79
Anopheles funestus	-5.739506	9.234096	Unclassified	GBIF: https://doi.org/10.15468/dl.zfaw79
Anopheles funestus	-5.738114	9.234081	Unclassified	GBIF: https://doi.org/10.15468/dl.zfaw79
Anopheles funestus	-5.710799	8.991622	Unclassified	GBIF: https://doi.org/10.15468/dl.zfaw79
Anopheles funestus	-5.688478	9.161241	Unclassified	GBIF: https://doi.org/10.15468/dl.zfaw79
Anopheles funestus	-5.687875	9.160922	Unclassified	GBIF: https://doi.org/10.15468/dl.zfaw79
Anopheles funestus	-5.687781	9.159902	Unclassified	GBIF: https://doi.org/10.15468/dl.zfaw79
Anopheles funestus	-5.687131	9.160263	Unclassified	GBIF: https://doi.org/10.15468/dl.zfaw79
Anopheles funestus	-5.66878	9.237231	Unclassified	GBIF: https://doi.org/10.15468/dl.zfaw79
Anopheles funestus	-5.668521	9.23779	Unclassified	GBIF: https://doi.org/10.15468/dl.zfaw79
Anopheles funestus	-5.668484	9.237798	Unclassified	GBIF: https://doi.org/10.15468/dl.zfaw79
Anopheles funestus	-5.668395	9.236691	Unclassified	GBIF: https://doi.org/10.15468/dl.zfaw79

Anopheles funestus	-5.667802	9.236155	Unclassified	GBIF: https://doi.org/10.15468/dl.zfaw79
Anopheles funestus	-5.653322	8.948459	Unclassified	GBIF: https://doi.org/10.15468/dl.zfaw79
Anopheles funestus	-5.652115	8.948168	Unclassified	GBIF: https://doi.org/10.15468/dl.zfaw79
Anopheles funestus	-5.652092	8.948175	Unclassified	GBIF: https://doi.org/10.15468/dl.zfaw79
Anopheles funestus	-5.617148	9.258641	Unclassified	GBIF: https://doi.org/10.15468/dl.zfaw79
Anopheles funestus	-5.614409	9.251595	Unclassified	GBIF: https://doi.org/10.15468/dl.zfaw79
Anopheles funestus	-5.614276	9.252568	Unclassified	GBIF: https://doi.org/10.15468/dl.zfaw79
Anopheles funestus	-5.614248	9.252597	Unclassified	GBIF: https://doi.org/10.15468/dl.zfaw79
Anopheles funestus	-5.613993	9.251237	Unclassified	GBIF: https://doi.org/10.15468/dl.zfaw79
Anopheles funestus	-5.611362	9.326845	Unclassified	GBIF: https://doi.org/10.15468/dl.zfaw79
Anopheles funestus	-5.610996	9.295945	Unclassified	GBIF: https://doi.org/10.15468/dl.zfaw79
Anopheles funestus	-5.609751	9.296192	Unclassified	GBIF: https://doi.org/10.15468/dl.zfaw79
Anopheles funestus	-5.606884	9.186423	Unclassified	GBIF: https://doi.org/10.15468/dl.zfaw79
Anopheles funestus	-5.60686	9.186424	Unclassified	GBIF: https://doi.org/10.15468/dl.zfaw79
Anopheles funestus	-5.606308	9.325325	Unclassified	GBIF: https://doi.org/10.15468/dl.zfaw79
Anopheles funestus	-5.605534	9.186001	Unclassified	GBIF: https://doi.org/10.15468/dl.zfaw79
Anopheles funestus	-5.605161	9.188004	Unclassified	GBIF: https://doi.org/10.15468/dl.zfaw79
Anopheles funestus	-5.604381	9.186696	Unclassified	GBIF: https://doi.org/10.15468/dl.zfaw79
Anopheles funestus	-5.5896	9.353077	Unclassified	GBIF: https://doi.org/10.15468/dl.zfaw79
Anopheles funestus	-5.589579	9.353049	Unclassified	GBIF: https://doi.org/10.15468/dl.zfaw79
Anopheles funestus	-5.588385	9.352951	Unclassified	GBIF: https://doi.org/10.15468/dl.zfaw79
Anopheles funestus	-5.587175	9.351393	Unclassified	GBIF: https://doi.org/10.15468/dl.zfaw79
Anopheles funestus	-5.586832	9.353458	Unclassified	GBIF: https://doi.org/10.15468/dl.zfaw79
Anopheles funestus	-5.584309	9.278933	Unclassified	GBIF: https://doi.org/10.15468/dl.zfaw79
Anopheles funestus	-5.569895	9.397348	Unclassified	GBIF: https://doi.org/10.15468/dl.zfaw79
Anopheles funestus	-5.568234	9.297857	Unclassified	GBIF: https://doi.org/10.15468/dl.zfaw79
Anopheles funestus	-5.567198	9.298328	Unclassified	GBIF: https://doi.org/10.15468/dl.zfaw79
Anopheles funestus	-5.566653	9.299306	Unclassified	GBIF: https://doi.org/10.15468/dl.zfaw79
Anopheles funestus	-5.535889	9.282598	Unclassified	GBIF: https://doi.org/10.15468/dl.zfaw79
Anopheles funestus	-5.53528	9.281796	Unclassified	GBIF: https://doi.org/10.15468/dl.zfaw79
Anopheles funestus	-5.534768	9.282752	Unclassified	GBIF: https://doi.org/10.15468/dl.zfaw79
Anopheles funestus	-5.523485	9.288249	Unclassified	GBIF: https://doi.org/10.15468/dl.zfaw79
Anopheles funestus	-5.516588	9.486683	Unclassified	GBIF: https://doi.org/10.15468/dl.zfaw79
Anopheles funestus	-5.514441	9.440569	Unclassified	GBIF: https://doi.org/10.15468/dl.zfaw79
Anopheles funestus	-5.514386	9.253341	Unclassified	GBIF: https://doi.org/10.15468/dl.zfaw79
Anopheles funestus	-5.513939	9.440282	Unclassified	GBIF: https://doi.org/10.15468/dl.zfaw79
Anopheles funestus	-5.51303	9.44086	Unclassified	GBIF: https://doi.org/10.15468/dl.zfaw79
Anopheles funestus	-5.500091	8.98903	Unclassified	GBIF: https://doi.org/10.15468/dl.zfaw79
Anopheles funestus	-5.4999	8.988799	Unclassified	GBIF: https://doi.org/10.15468/dl.zfaw79
Anopheles funestus	-5.499599	8.989674	Unclassified	GBIF: https://doi.org/10.15468/dl.zfaw79
Anopheles funestus	-5.499375	9.39459	Unclassified	GBIF: https://doi.org/10.15468/dl.zfaw79
Anopheles funestus	-5.499347	8.989034	Unclassified	GBIF: https://doi.org/10.15468/dl.zfaw79

Anopheles funestus	-5.499	9.394457	Unclassified	GBIF: https://doi.org/10.15468/dl.zfaw79
Anopheles funestus	-5.498557	9.394168	Unclassified	GBIF: https://doi.org/10.15468/dl.zfaw79
Anopheles funestus	-5.488005	9.506761	Unclassified	GBIF: https://doi.org/10.15468/dl.zfaw79
Anopheles funestus	-5.487461	9.03183	Unclassified	GBIF: https://doi.org/10.15468/dl.zfaw79
Anopheles funestus	-5.487444	9.031841	Unclassified	GBIF: https://doi.org/10.15468/dl.zfaw79
Anopheles funestus	-5.486642	9.033241	Unclassified	GBIF: https://doi.org/10.15468/dl.zfaw79
Anopheles funestus	-5.486623	9.033267	Unclassified	GBIF: https://doi.org/10.15468/dl.zfaw79
Anopheles funestus	-5.486363	9.031165	Unclassified	GBIF: https://doi.org/10.15468/dl.zfaw79
Anopheles funestus	-5.486232	9.032412	Unclassified	GBIF: https://doi.org/10.15468/dl.zfaw79
Anopheles funestus	-5.486216	9.03242	Unclassified	GBIF: https://doi.org/10.15468/dl.zfaw79
Anopheles funestus	-5.486212	9.032418	Unclassified	GBIF: https://doi.org/10.15468/dl.zfaw79
Anopheles funestus	-4.52002	11.236	Unclassified	GBIF: https://doi.org/10.15468/dl.zfaw79
Anopheles funestus	-4.51156	11.2365	Unclassified	GBIF: https://doi.org/10.15468/dl.zfaw79
Anopheles funestus	-4.50952	11.2384	Unclassified	GBIF: https://doi.org/10.15468/dl.zfaw79
Anopheles funestus	-4.5006	11.239	Unclassified	GBIF: https://doi.org/10.15468/dl.zfaw79
Anopheles funestus	-4.49909	11.2396	Unclassified	GBIF: https://doi.org/10.15468/dl.zfaw79
Anopheles funestus	-4.47924	11.2359	Unclassified	GBIF: https://doi.org/10.15468/dl.zfaw79
Anopheles funestus	-4.47916	11.2314	Unclassified	GBIF: https://doi.org/10.15468/dl.zfaw79
Anopheles funestus	-4.4769	11.2346	Unclassified	GBIF: https://doi.org/10.15468/dl.zfaw79
Anopheles funestus	-3.552445	10.705937	Unclassified	GBIF: https://doi.org/10.15468/dl.zfaw79
Anopheles funestus	-3.486203	10.746854	Unclassified	GBIF: https://doi.org/10.15468/dl.zfaw79
Anopheles funestus	-3.479202	10.795241	Unclassified	GBIF: https://doi.org/10.15468/dl.zfaw79
Anopheles funestus	-3.478258	10.794586	Unclassified	GBIF: https://doi.org/10.15468/dl.zfaw79
Anopheles funestus	-3.478155	10.794589	Unclassified	GBIF: https://doi.org/10.15468/dl.zfaw79
Anopheles funestus	-3.477808	10.793409	Unclassified	GBIF: https://doi.org/10.15468/dl.zfaw79
Anopheles funestus	-3.477669	10.795122	Unclassified	GBIF: https://doi.org/10.15468/dl.zfaw79
Anopheles funestus	-3.477531	10.795137	Unclassified	GBIF: https://doi.org/10.15468/dl.zfaw79
Anopheles funestus	-3.475028	10.858615	Unclassified	GBIF: https://doi.org/10.15468/dl.zfaw79
Anopheles funestus	-3.472521	10.858118	Unclassified	GBIF: https://doi.org/10.15468/dl.zfaw79
Anopheles funestus	-3.454403	10.919474	Unclassified	GBIF: https://doi.org/10.15468/dl.zfaw79
Anopheles funestus	-3.454059	10.919467	Unclassified	GBIF: https://doi.org/10.15468/dl.zfaw79
Anopheles funestus	-3.453431	10.919607	Unclassified	GBIF: https://doi.org/10.15468/dl.zfaw79
Anopheles funestus	-3.453351	10.922379	Unclassified	GBIF: https://doi.org/10.15468/dl.zfaw79
Anopheles funestus	-3.453336	10.922344	Unclassified	GBIF: https://doi.org/10.15468/dl.zfaw79
Anopheles funestus	-3.453267	10.922382	Unclassified	GBIF: https://doi.org/10.15468/dl.zfaw79
Anopheles funestus	-3.453218	10.921763	Unclassified	GBIF: https://doi.org/10.15468/dl.zfaw79
Anopheles funestus	-3.453175	10.921755	Unclassified	GBIF: https://doi.org/10.15468/dl.zfaw79
Anopheles funestus	-3.453166	10.919507	Unclassified	GBIF: https://doi.org/10.15468/dl.zfaw79
Anopheles funestus	-3.453155	10.921741	Unclassified	GBIF: https://doi.org/10.15468/dl.zfaw79
Anopheles funestus	-3.452867	10.920293	Unclassified	GBIF: https://doi.org/10.15468/dl.zfaw79
Anopheles funestus	-3.452863	10.920315	Unclassified	GBIF: https://doi.org/10.15468/dl.zfaw79
Anopheles funestus	-3.452819	10.920313	Unclassified	GBIF: https://doi.org/10.15468/dl.zfaw79

Anopheles funestus	-3.452748	10.920485	Unclassified	GBIF: https://doi.org/10.15468/dl.zfaw79
Anopheles funestus	-3.452722	10.920495	Unclassified	GBIF: https://doi.org/10.15468/dl.zfaw79
Anopheles funestus	-3.452468	10.922771	Unclassified	GBIF: https://doi.org/10.15468/dl.zfaw79
Anopheles funestus	-3.452459	10.922811	Unclassified	GBIF: https://doi.org/10.15468/dl.zfaw79
Anopheles funestus	-3.452442	10.922821	Unclassified	GBIF: https://doi.org/10.15468/dl.zfaw79
Anopheles funestus	-3.403911	10.794422	Unclassified	GBIF: https://doi.org/10.15468/dl.zfaw79
Anopheles funestus	-3.395308	10.80704	Unclassified	GBIF: https://doi.org/10.15468/dl.zfaw79
Anopheles funestus	-3.393933	10.808007	Unclassified	GBIF: https://doi.org/10.15468/dl.zfaw79
Anopheles funestus	-3.393422	10.808199	Unclassified	GBIF: https://doi.org/10.15468/dl.zfaw79
Anopheles funestus	-3.390866	10.903137	Unclassified	GBIF: https://doi.org/10.15468/dl.zfaw79
Anopheles funestus	-3.387575	10.914597	Unclassified	GBIF: https://doi.org/10.15468/dl.zfaw79
Anopheles funestus	-3.387539	10.914582	Unclassified	GBIF: https://doi.org/10.15468/dl.zfaw79
Anopheles funestus	-3.387539	10.914585	Unclassified	GBIF: https://doi.org/10.15468/dl.zfaw79
Anopheles funestus	-3.387528	10.914534	Unclassified	GBIF: https://doi.org/10.15468/dl.zfaw79
Anopheles funestus	-3.386642	10.913802	Unclassified	GBIF: https://doi.org/10.15468/dl.zfaw79
Anopheles funestus	-3.38664	10.913732	Unclassified	GBIF: https://doi.org/10.15468/dl.zfaw79
Anopheles funestus	-3.386599	10.913746	Unclassified	GBIF: https://doi.org/10.15468/dl.zfaw79
Anopheles funestus	-3.385274	10.913341	Unclassified	GBIF: https://doi.org/10.15468/dl.zfaw79
Anopheles funestus	-3.385214	10.91331	Unclassified	GBIF: https://doi.org/10.15468/dl.zfaw79
Anopheles funestus	-3.385207	10.913335	Unclassified	GBIF: https://doi.org/10.15468/dl.zfaw79
Anopheles funestus	-3.384336	10.988661	Unclassified	GBIF: https://doi.org/10.15468/dl.zfaw79
Anopheles funestus	-3.384312	10.988624	Unclassified	GBIF: https://doi.org/10.15468/dl.zfaw79
Anopheles funestus	-3.384298	10.988608	Unclassified	GBIF: https://doi.org/10.15468/dl.zfaw79
Anopheles funestus	-3.384285	10.98821	Unclassified	GBIF: https://doi.org/10.15468/dl.zfaw79
Anopheles funestus	-3.384278	10.98826	Unclassified	GBIF: https://doi.org/10.15468/dl.zfaw79
Anopheles funestus	-3.384267	10.988254	Unclassified	GBIF: https://doi.org/10.15468/dl.zfaw79
Anopheles funestus	-3.384254	10.988247	Unclassified	GBIF: https://doi.org/10.15468/dl.zfaw79
Anopheles funestus	-3.384245	10.988181	Unclassified	GBIF: https://doi.org/10.15468/dl.zfaw79
Anopheles funestus	-3.384194	10.915213	Unclassified	GBIF: https://doi.org/10.15468/dl.zfaw79
Anopheles funestus	-3.384187	10.915262	Unclassified	GBIF: https://doi.org/10.15468/dl.zfaw79
Anopheles funestus	-3.38348	10.987875	Unclassified	GBIF: https://doi.org/10.15468/dl.zfaw79
Anopheles funestus	-3.383415	10.987845	Unclassified	GBIF: https://doi.org/10.15468/dl.zfaw79
Anopheles funestus	-3.383253	10.987842	Unclassified	GBIF: https://doi.org/10.15468/dl.zfaw79
Anopheles funestus	-3.383227	10.987826	Unclassified	GBIF: https://doi.org/10.15468/dl.zfaw79
Anopheles funestus	-3.340978	10.78277	Unclassified	GBIF: https://doi.org/10.15468/dl.zfaw79
Anopheles funestus	-3.340175	10.783398	Unclassified	GBIF: https://doi.org/10.15468/dl.zfaw79
Anopheles funestus	-3.340162	10.783423	Unclassified	GBIF: https://doi.org/10.15468/dl.zfaw79
Anopheles funestus	-3.332104	10.854664	Unclassified	GBIF: https://doi.org/10.15468/dl.zfaw79
Anopheles funestus	-3.331869	10.856224	Unclassified	GBIF: https://doi.org/10.15468/dl.zfaw79
Anopheles funestus	-3.33127	10.827468	Unclassified	GBIF: https://doi.org/10.15468/dl.zfaw79
Anopheles funestus	-3.33093	10.825562	Unclassified	GBIF: https://doi.org/10.15468/dl.zfaw79
Anopheles funestus	-3.330877	10.825572	Unclassified	GBIF: https://doi.org/10.15468/dl.zfaw79

Anopheles funestus	-3.330863	10.825548	Unclassified	GBIF: https://doi.org/10.15468/dl.zfaw79
Anopheles funestus	-3.330809	10.855686	Unclassified	GBIF: https://doi.org/10.15468/dl.zfaw79
Anopheles funestus	-3.330592	10.824709	Unclassified	GBIF: https://doi.org/10.15468/dl.zfaw79
Anopheles funestus	-3.3305	10.824746	Unclassified	GBIF: https://doi.org/10.15468/dl.zfaw79
Anopheles funestus	-3.330086	10.825608	Unclassified	GBIF: https://doi.org/10.15468/dl.zfaw79
Anopheles funestus	-3.330063	10.825624	Unclassified	GBIF: https://doi.org/10.15468/dl.zfaw79
Anopheles funestus	-3.329987	10.892908	Unclassified	GBIF: https://doi.org/10.15468/dl.zfaw79
Anopheles funestus	-3.329959	10.89294	Unclassified	GBIF: https://doi.org/10.15468/dl.zfaw79
Anopheles funestus	-3.32995	10.856014	Unclassified	GBIF: https://doi.org/10.15468/dl.zfaw79
Anopheles funestus	-3.329923	10.855973	Unclassified	GBIF: https://doi.org/10.15468/dl.zfaw79
Anopheles funestus	-3.329912	10.891961	Unclassified	GBIF: https://doi.org/10.15468/dl.zfaw79
Anopheles funestus	-3.329907	10.89193	Unclassified	GBIF: https://doi.org/10.15468/dl.zfaw79
Anopheles funestus	-3.329896	10.89194	Unclassified	GBIF: https://doi.org/10.15468/dl.zfaw79
Anopheles funestus	-3.329525	10.89252	Unclassified	GBIF: https://doi.org/10.15468/dl.zfaw79
Anopheles funestus	-3.3295	10.892524	Unclassified	GBIF: https://doi.org/10.15468/dl.zfaw79
Anopheles funestus	-3.329497	10.892524	Unclassified	GBIF: https://doi.org/10.15468/dl.zfaw79
Anopheles funestus	-3.328952	10.892462	Unclassified	GBIF: https://doi.org/10.15468/dl.zfaw79
Anopheles funestus	-3.32895	10.892451	Unclassified	GBIF: https://doi.org/10.15468/dl.zfaw79
Anopheles funestus	-3.328943	10.8925	Unclassified	GBIF: https://doi.org/10.15468/dl.zfaw79
Anopheles funestus	-3.324404	10.890882	Unclassified	GBIF: https://doi.org/10.15468/dl.zfaw79
Anopheles funestus	-3.323908	10.891245	Unclassified	GBIF: https://doi.org/10.15468/dl.zfaw79
Anopheles funestus	-3.323332	10.89123	Unclassified	GBIF: https://doi.org/10.15468/dl.zfaw79
Anopheles funestus	-3.322304	10.891803	Unclassified	GBIF: https://doi.org/10.15468/dl.zfaw79
Anopheles funestus	-3.321167	10.852973	Unclassified	GBIF: https://doi.org/10.15468/dl.zfaw79
Anopheles funestus	-3.320809	10.853105	Unclassified	GBIF: https://doi.org/10.15468/dl.zfaw79
Anopheles funestus	-3.316595	10.708401	Unclassified	GBIF: https://doi.org/10.15468/dl.zfaw79
Anopheles funestus	-3.31581	10.852056	Unclassified	GBIF: https://doi.org/10.15468/dl.zfaw79
Anopheles funestus	-3.315796	10.851316	Unclassified	GBIF: https://doi.org/10.15468/dl.zfaw79
Anopheles funestus	-3.31579	10.851355	Unclassified	GBIF: https://doi.org/10.15468/dl.zfaw79
Anopheles funestus	-3.315789	10.851345	Unclassified	GBIF: https://doi.org/10.15468/dl.zfaw79
Anopheles funestus	-3.31578	10.852071	Unclassified	GBIF: https://doi.org/10.15468/dl.zfaw79
Anopheles funestus	-3.315769	10.852078	Unclassified	GBIF: https://doi.org/10.15468/dl.zfaw79
Anopheles funestus	-3.315761	10.849326	Unclassified	GBIF: https://doi.org/10.15468/dl.zfaw79
Anopheles funestus	-3.31576	10.851339	Unclassified	GBIF: https://doi.org/10.15468/dl.zfaw79
Anopheles funestus	-3.315749	10.849297	Unclassified	GBIF: https://doi.org/10.15468/dl.zfaw79
Anopheles funestus	-3.315739	10.84928	Unclassified	GBIF: https://doi.org/10.15468/dl.zfaw79
Anopheles funestus	-3.315715	10.852331	Unclassified	GBIF: https://doi.org/10.15468/dl.zfaw79
Anopheles funestus	-3.315689	10.852419	Unclassified	GBIF: https://doi.org/10.15468/dl.zfaw79
Anopheles funestus	-3.315687	10.852369	Unclassified	GBIF: https://doi.org/10.15468/dl.zfaw79
Anopheles funestus	-3.315665	10.852385	Unclassified	GBIF: https://doi.org/10.15468/dl.zfaw79
Anopheles funestus	-3.314068	10.708081	Unclassified	GBIF: https://doi.org/10.15468/dl.zfaw79
Anopheles funestus	-3.314048	10.708092	Unclassified	GBIF: https://doi.org/10.15468/dl.zfaw79

Anopheles funestus	-3.313999	10.854832	Unclassified	GBIF: https://doi.org/10.15468/dl.zfaw79
Anopheles funestus	-3.296218	10.669313	Unclassified	GBIF: https://doi.org/10.15468/dl.zfaw79
Anopheles funestus	-3.295863	10.66832	Unclassified	GBIF: https://doi.org/10.15468/dl.zfaw79
Anopheles funestus	-3.295841	10.668278	Unclassified	GBIF: https://doi.org/10.15468/dl.zfaw79
Anopheles funestus	-3.28905	10.761338	Unclassified	GBIF: https://doi.org/10.15468/dl.zfaw79
Anopheles funestus	-3.287936	10.711344	Unclassified	GBIF: https://doi.org/10.15468/dl.zfaw79
Anopheles funestus	-3.287418	10.711288	Unclassified	GBIF: https://doi.org/10.15468/dl.zfaw79
Anopheles funestus	-3.287375	10.711306	Unclassified	GBIF: https://doi.org/10.15468/dl.zfaw79
Anopheles funestus	-3.287029	10.713	Unclassified	GBIF: https://doi.org/10.15468/dl.zfaw79
Anopheles funestus	-3.28698	10.712808	Unclassified	GBIF: https://doi.org/10.15468/dl.zfaw79
Anopheles funestus	-3.25589	10.745253	Unclassified	GBIF: https://doi.org/10.15468/dl.zfaw79
Anopheles funestus	-3.19443	10.963547	Unclassified	GBIF: https://doi.org/10.15468/dl.zfaw79
Anopheles funestus	-3.19441	10.962663	Unclassified	GBIF: https://doi.org/10.15468/dl.zfaw79
Anopheles funestus	-3.194278	10.974277	Unclassified	GBIF: https://doi.org/10.15468/dl.zfaw79
Anopheles funestus	-3.194094	10.963303	Unclassified	GBIF: https://doi.org/10.15468/dl.zfaw79
Anopheles funestus	-3.192815	10.963358	Unclassified	GBIF: https://doi.org/10.15468/dl.zfaw79
Anopheles funestus	-3.191295	10.964066	Unclassified	GBIF: https://doi.org/10.15468/dl.zfaw79
Anopheles funestus	-3.190162	10.96256	Unclassified	GBIF: https://doi.org/10.15468/dl.zfaw79
Anopheles funestus	-3.18912	10.961106	Unclassified	GBIF: https://doi.org/10.15468/dl.zfaw79
Anopheles funestus	-3.189106	10.961109	Unclassified	GBIF: https://doi.org/10.15468/dl.zfaw79
Anopheles funestus	-3.166412	10.737555	Unclassified	GBIF: https://doi.org/10.15468/dl.zfaw79
Anopheles funestus	-3.165726	10.737976	Unclassified	GBIF: https://doi.org/10.15468/dl.zfaw79
Anopheles funestus	-3.165469	10.737956	Unclassified	GBIF: https://doi.org/10.15468/dl.zfaw79
Anopheles funestus	1.99834	10.9721	Unclassified	GBIF: https://doi.org/10.15468/dl.zfaw79
Anopheles funestus	2.114	7.267	Unclassified	GBIF: https://doi.org/10.15468/dl.zfaw79
Anopheles funestus	2.116	7.261	Unclassified	GBIF: https://doi.org/10.15468/dl.zfaw79
Anopheles funestus	2.118	7.284	Unclassified	GBIF: https://doi.org/10.15468/dl.zfaw79
Anopheles funestus	2.122	7.253	Unclassified	GBIF: https://doi.org/10.15468/dl.zfaw79
Anopheles funestus	2.13	7.26	Unclassified	GBIF: https://doi.org/10.15468/dl.zfaw79
Anopheles funestus	2.136	7.26	Unclassified	GBIF: https://doi.org/10.15468/dl.zfaw79
Anopheles funestus	2.151	7.209	Unclassified	GBIF: https://doi.org/10.15468/dl.zfaw79
Anopheles funestus	2.157	7.209	Unclassified	GBIF: https://doi.org/10.15468/dl.zfaw79
Anopheles funestus	2.185	7.78491	Unclassified	GBIF: https://doi.org/10.15468/dl.zfaw79
Anopheles funestus	2.35	7.207	Unclassified	GBIF: https://doi.org/10.15468/dl.zfaw79
Anopheles funestus	2.351	7.211	Unclassified	GBIF: https://doi.org/10.15468/dl.zfaw79
Anopheles funestus	2.42	7.2845	Unclassified	GBIF: https://doi.org/10.15468/dl.zfaw79
Anopheles funestus	2.42	7.222	Unclassified	GBIF: https://doi.org/10.15468/dl.zfaw79
Anopheles funestus	2.484	7.036	Unclassified	GBIF: https://doi.org/10.15468/dl.zfaw79
Anopheles funestus	2.484	7.04	Unclassified	GBIF: https://doi.org/10.15468/dl.zfaw79
Anopheles funestus	2.485	8.03361	Unclassified	GBIF: https://doi.org/10.15468/dl.zfaw79
Anopheles funestus	2.494	7.025	Unclassified	GBIF: https://doi.org/10.15468/dl.zfaw79
Anopheles funestus	2.498	7.031	Unclassified	GBIF: https://doi.org/10.15468/dl.zfaw79

Anopheles funestus	2.503	7.035	Unclassified	GBIF: https://doi.org/10.15468/dl.zfaw79
Anopheles funestus	2.504	7.089	Unclassified	GBIF: https://doi.org/10.15468/dl.zfaw79
Anopheles funestus	2.504	7.086	Unclassified	GBIF: https://doi.org/10.15468/dl.zfaw79
Anopheles funestus	2.504	7.04	Unclassified	GBIF: https://doi.org/10.15468/dl.zfaw79
Anopheles funestus	3.9	7.4	Unclassified	GBIF: https://doi.org/10.15468/dl.zfaw79
Anopheles funestus	4.74	10.84	Unclassified	GBIF: https://doi.org/10.15468/dl.zfaw79
Anopheles funestus	9.83333	5.25	Unclassified	GBIF: https://doi.org/10.15468/dl.zfaw79
Anopheles funestus	10.0667	5.45	Unclassified	GBIF: https://doi.org/10.15468/dl.zfaw79
Anopheles funestus	11.519852	3.898465	Unclassified	GBIF: https://doi.org/10.15468/dl.zfaw79
Anopheles funestus	11.59	-0.19	Unclassified	GBIF: https://doi.org/10.15468/dl.zfaw79
Anopheles funestus	11.9054	4.45253	Unclassified	GBIF: https://doi.org/10.15468/dl.zfaw79
Anopheles funestus	13.4341	14.9205	Unclassified	GBIF: https://doi.org/10.15468/dl.zfaw79
Anopheles funestus	13.4347	14.9209	Unclassified	GBIF: https://doi.org/10.15468/dl.zfaw79
Anopheles funestus	13.4348	14.9208	Unclassified	GBIF: https://doi.org/10.15468/dl.zfaw79
Anopheles funestus	13.4349	14.9211	Unclassified	GBIF: https://doi.org/10.15468/dl.zfaw79
Anopheles funestus	13.49	-1.63	Unclassified	GBIF: https://doi.org/10.15468/dl.zfaw79
Anopheles funestus	13.58	-1.62	Unclassified	GBIF: https://doi.org/10.15468/dl.zfaw79
Anopheles funestus	14.25	-17.5	Unclassified	GBIF: https://doi.org/10.15468/dl.zfaw79
Anopheles funestus	15.272778	-4.326944	Unclassified	GBIF: https://doi.org/10.15468/dl.zfaw79
Anopheles funestus	15.281111	-4.413611	Unclassified	GBIF: https://doi.org/10.15468/dl.zfaw79
Anopheles funestus	15.32	-4.3475	Unclassified	GBIF: https://doi.org/10.15468/dl.zfaw79
Anopheles funestus	15.324167	-4.416389	Unclassified	GBIF: https://doi.org/10.15468/dl.zfaw79
Anopheles funestus	15.338056	-4.349722	Unclassified	GBIF: https://doi.org/10.15468/dl.zfaw79
Anopheles funestus	15.346389	-4.413056	Unclassified	GBIF: https://doi.org/10.15468/dl.zfaw79
Anopheles funestus	15.353889	-4.476389	Unclassified	GBIF: https://doi.org/10.15468/dl.zfaw79
Anopheles funestus	15.413333	-4.414167	Unclassified	GBIF: https://doi.org/10.15468/dl.zfaw79
Anopheles funestus	17	-18.8	Unclassified	GBIF: https://doi.org/10.15468/dl.zfaw79
Anopheles funestus	17.761713	2.104093	Unclassified	GBIF: https://doi.org/10.15468/dl.zfaw79
Anopheles funestus	18.5405	4.33108	Unclassified	GBIF: https://doi.org/10.15468/dl.zfaw79
Anopheles funestus	18.5468	4.43042	Unclassified	GBIF: https://doi.org/10.15468/dl.zfaw79
Anopheles funestus	18.5667	4.3549	Unclassified	GBIF: https://doi.org/10.15468/dl.zfaw79
Anopheles funestus	18.6083	4.37667	Unclassified	GBIF: https://doi.org/10.15468/dl.zfaw79
Anopheles funestus	23.3867	-19.1183	Unclassified	GBIF: https://doi.org/10.15468/dl.zfaw79
Anopheles funestus	23.8683	-19.1217	Unclassified	GBIF: https://doi.org/10.15468/dl.zfaw79
Anopheles funestus	28.8	-2.24	Unclassified	GBIF: https://doi.org/10.15468/dl.zfaw79
Anopheles funestus	29.7	0.750025	Unclassified	GBIF: https://doi.org/10.15468/dl.zfaw79
Anopheles funestus	29.7001	0.750102	Unclassified	GBIF: https://doi.org/10.15468/dl.zfaw79
Anopheles funestus	29.7003	-0.748889	Unclassified	GBIF: https://doi.org/10.15468/dl.zfaw79
Anopheles funestus	29.7827	-0.885652	Unclassified	GBIF: https://doi.org/10.15468/dl.zfaw79
Anopheles funestus	30.015	-0.85	Unclassified	GBIF: https://doi.org/10.15468/dl.zfaw79
Anopheles funestus	30.520713	-23.993834	Unclassified	GBIF: https://doi.org/10.15468/dl.zfaw79
Anopheles funestus	30.89	2.81	Unclassified	GBIF: https://doi.org/10.15468/dl.zfaw79

Anopheles funestus	32.14	2.24	Unclassified	GBIF: https://doi.org/10.15468/dl.zfaw79
Anopheles funestus	32.251389	-27.7125	Unclassified	GBIF: https://doi.org/10.15468/dl.zfaw79
Anopheles funestus	32.278612	-26.930937	Unclassified	GBIF: https://doi.org/10.15468/dl.zfaw79
Anopheles funestus	32.573292	-25.969141	Unclassified	GBIF: https://doi.org/10.15468/dl.zfaw79
Anopheles funestus	33.226	0.443034	Unclassified	GBIF: https://doi.org/10.15468/dl.zfaw79
Anopheles funestus	33.226	0.443036	Unclassified	GBIF: https://doi.org/10.15468/dl.zfaw79
Anopheles funestus	33.226	0.443006	Unclassified	GBIF: https://doi.org/10.15468/dl.zfaw79
Anopheles funestus	33.226	0.44303	Unclassified	GBIF: https://doi.org/10.15468/dl.zfaw79
Anopheles funestus	33.226	0.443026	Unclassified	GBIF: https://doi.org/10.15468/dl.zfaw79
Anopheles funestus	33.226	0.443028	Unclassified	GBIF: https://doi.org/10.15468/dl.zfaw79
Anopheles funestus	33.226	0.44301	Unclassified	GBIF: https://doi.org/10.15468/dl.zfaw79
Anopheles funestus	33.226	0.443023	Unclassified	GBIF: https://doi.org/10.15468/dl.zfaw79
Anopheles funestus	33.226	0.443033	Unclassified	GBIF: https://doi.org/10.15468/dl.zfaw79
Anopheles funestus	33.226	0.443047	Unclassified	GBIF: https://doi.org/10.15468/dl.zfaw79
Anopheles funestus	33.226	0.443049	Unclassified	GBIF: https://doi.org/10.15468/dl.zfaw79
Anopheles funestus	33.226	0.443048	Unclassified	GBIF: https://doi.org/10.15468/dl.zfaw79
Anopheles funestus	33.226	0.443008	Unclassified	GBIF: https://doi.org/10.15468/dl.zfaw79
Anopheles funestus	33.226	0.443029	Unclassified	GBIF: https://doi.org/10.15468/dl.zfaw79
Anopheles funestus	33.226	0.443038	Unclassified	GBIF: https://doi.org/10.15468/dl.zfaw79
Anopheles funestus	33.226	0.443009	Unclassified	GBIF: https://doi.org/10.15468/dl.zfaw79
Anopheles funestus	33.226	0.443032	Unclassified	GBIF: https://doi.org/10.15468/dl.zfaw79
Anopheles funestus	33.226	0.443017	Unclassified	GBIF: https://doi.org/10.15468/dl.zfaw79
Anopheles funestus	33.226	0.443037	Unclassified	GBIF: https://doi.org/10.15468/dl.zfaw79
Anopheles funestus	33.226	0.44302	Unclassified	GBIF: https://doi.org/10.15468/dl.zfaw79
Anopheles funestus	33.226	0.443012	Unclassified	GBIF: https://doi.org/10.15468/dl.zfaw79
Anopheles funestus	33.226	0.443022	Unclassified	GBIF: https://doi.org/10.15468/dl.zfaw79
Anopheles funestus	33.226	0.443007	Unclassified	GBIF: https://doi.org/10.15468/dl.zfaw79
Anopheles funestus	33.226	0.443035	Unclassified	GBIF: https://doi.org/10.15468/dl.zfaw79
Anopheles funestus	33.226	0.443002	Unclassified	GBIF: https://doi.org/10.15468/dl.zfaw79
Anopheles funestus	33.226	0.443019	Unclassified	GBIF: https://doi.org/10.15468/dl.zfaw79
Anopheles funestus	33.226	0.443039	Unclassified	GBIF: https://doi.org/10.15468/dl.zfaw79
Anopheles funestus	33.226	0.443031	Unclassified	GBIF: https://doi.org/10.15468/dl.zfaw79
Anopheles funestus	33.2261	0.443096	Unclassified	GBIF: https://doi.org/10.15468/dl.zfaw79
Anopheles funestus	33.2261	0.443072	Unclassified	GBIF: https://doi.org/10.15468/dl.zfaw79
Anopheles funestus	33.2261	0.4431	Unclassified	GBIF: https://doi.org/10.15468/dl.zfaw79
Anopheles funestus	33.2261	0.44308	Unclassified	GBIF: https://doi.org/10.15468/dl.zfaw79
Anopheles funestus	33.2261	0.443052	Unclassified	GBIF: https://doi.org/10.15468/dl.zfaw79
Anopheles funestus	33.2261	0.443053	Unclassified	GBIF: https://doi.org/10.15468/dl.zfaw79
Anopheles funestus	33.2261	0.44305	Unclassified	GBIF: https://doi.org/10.15468/dl.zfaw79
Anopheles funestus	33.2261	0.443093	Unclassified	GBIF: https://doi.org/10.15468/dl.zfaw79
Anopheles funestus	33.2261	0.443063	Unclassified	GBIF: https://doi.org/10.15468/dl.zfaw79
Anopheles funestus	33.2261	0.44306	Unclassified	GBIF: https://doi.org/10.15468/dl.zfaw79

Anopheles funestus	33.2261	0.443071	Unclassified	GBIF: https://doi.org/10.15468/dl.zfaw79
Anopheles funestus	33.2261	0.443079	Unclassified	GBIF: https://doi.org/10.15468/dl.zfaw79
Anopheles funestus	33.2261	0.443058	Unclassified	GBIF: https://doi.org/10.15468/dl.zfaw79
Anopheles funestus	33.2261	0.443069	Unclassified	GBIF: https://doi.org/10.15468/dl.zfaw79
Anopheles funestus	33.2261	0.443082	Unclassified	GBIF: https://doi.org/10.15468/dl.zfaw79
Anopheles funestus	33.2261	0.44307	Unclassified	GBIF: https://doi.org/10.15468/dl.zfaw79
Anopheles funestus	33.2261	0.443111	Unclassified	GBIF: https://doi.org/10.15468/dl.zfaw79
Anopheles funestus	33.2261	0.443062	Unclassified	GBIF: https://doi.org/10.15468/dl.zfaw79
Anopheles funestus	33.2261	0.443061	Unclassified	GBIF: https://doi.org/10.15468/dl.zfaw79
Anopheles funestus	33.2261	0.44311	Unclassified	GBIF: https://doi.org/10.15468/dl.zfaw79
Anopheles funestus	33.2261	0.443057	Unclassified	GBIF: https://doi.org/10.15468/dl.zfaw79
Anopheles funestus	33.2261	0.443094	Unclassified	GBIF: https://doi.org/10.15468/dl.zfaw79
Anopheles funestus	33.2261	0.443108	Unclassified	GBIF: https://doi.org/10.15468/dl.zfaw79
Anopheles funestus	33.2367	0.447222	Unclassified	GBIF: https://doi.org/10.15468/dl.zfaw79
Anopheles funestus	33.7276	1.66042	Unclassified	GBIF: https://doi.org/10.15468/dl.zfaw79
Anopheles funestus	33.7366	1.71742	Unclassified	GBIF: https://doi.org/10.15468/dl.zfaw79
Anopheles funestus	34	-13.5	Unclassified	GBIF: https://doi.org/10.15468/dl.zfaw79
Anopheles funestus	34.026	0.770014	Unclassified	GBIF: https://doi.org/10.15468/dl.zfaw79
Anopheles funestus	34.026	0.770038	Unclassified	GBIF: https://doi.org/10.15468/dl.zfaw79
Anopheles funestus	34.026	0.770025	Unclassified	GBIF: https://doi.org/10.15468/dl.zfaw79
Anopheles funestus	34.026	0.77001	Unclassified	GBIF: https://doi.org/10.15468/dl.zfaw79
Anopheles funestus	34.026	0.770048	Unclassified	GBIF: https://doi.org/10.15468/dl.zfaw79
Anopheles funestus	34.026	0.770026	Unclassified	GBIF: https://doi.org/10.15468/dl.zfaw79
Anopheles funestus	34.026	0.77002	Unclassified	GBIF: https://doi.org/10.15468/dl.zfaw79
Anopheles funestus	34.026	0.770029	Unclassified	GBIF: https://doi.org/10.15468/dl.zfaw79
Anopheles funestus	34.026	0.770009	Unclassified	GBIF: https://doi.org/10.15468/dl.zfaw79
Anopheles funestus	34.026	0.770015	Unclassified	GBIF: https://doi.org/10.15468/dl.zfaw79
Anopheles funestus	34.026	0.770005	Unclassified	GBIF: https://doi.org/10.15468/dl.zfaw79
Anopheles funestus	34.026	0.770035	Unclassified	GBIF: https://doi.org/10.15468/dl.zfaw79
Anopheles funestus	34.026	0.770031	Unclassified	GBIF: https://doi.org/10.15468/dl.zfaw79
Anopheles funestus	34.026	0.770039	Unclassified	GBIF: https://doi.org/10.15468/dl.zfaw79
Anopheles funestus	34.026	0.770027	Unclassified	GBIF: https://doi.org/10.15468/dl.zfaw79
Anopheles funestus	34.026	0.770043	Unclassified	GBIF: https://doi.org/10.15468/dl.zfaw79
Anopheles funestus	34.026	0.770041	Unclassified	GBIF: https://doi.org/10.15468/dl.zfaw79
Anopheles funestus	34.026	0.770032	Unclassified	GBIF: https://doi.org/10.15468/dl.zfaw79
Anopheles funestus	34.026	0.770044	Unclassified	GBIF: https://doi.org/10.15468/dl.zfaw79
Anopheles funestus	34.026	0.770037	Unclassified	GBIF: https://doi.org/10.15468/dl.zfaw79
Anopheles funestus	34.026	0.770011	Unclassified	GBIF: https://doi.org/10.15468/dl.zfaw79
Anopheles funestus	34.026	0.770007	Unclassified	GBIF: https://doi.org/10.15468/dl.zfaw79
Anopheles funestus	34.026	0.770036	Unclassified	GBIF: https://doi.org/10.15468/dl.zfaw79
Anopheles funestus	34.026	0.770018	Unclassified	GBIF: https://doi.org/10.15468/dl.zfaw79
Anopheles funestus	34.026	0.770012	Unclassified	GBIF: https://doi.org/10.15468/dl.zfaw79

Anopheles funestus	34.026	0.770028	Unclassified	GBIF: https://doi.org/10.15468/dl.zfaw79
Anopheles funestus	34.026	0.770033	Unclassified	GBIF: https://doi.org/10.15468/dl.zfaw79
Anopheles funestus	34.026	0.770019	Unclassified	GBIF: https://doi.org/10.15468/dl.zfaw79
Anopheles funestus	34.026	0.770042	Unclassified	GBIF: https://doi.org/10.15468/dl.zfaw79
Anopheles funestus	34.026	0.770008	Unclassified	GBIF: https://doi.org/10.15468/dl.zfaw79
Anopheles funestus	34.026	0.770006	Unclassified	GBIF: https://doi.org/10.15468/dl.zfaw79
Anopheles funestus	34.026	0.770003	Unclassified	GBIF: https://doi.org/10.15468/dl.zfaw79
Anopheles funestus	34.026	0.770024	Unclassified	GBIF: https://doi.org/10.15468/dl.zfaw79
Anopheles funestus	34.026	0.770017	Unclassified	GBIF: https://doi.org/10.15468/dl.zfaw79
Anopheles funestus	34.026	0.770047	Unclassified	GBIF: https://doi.org/10.15468/dl.zfaw79
Anopheles funestus	34.026	0.770004	Unclassified	GBIF: https://doi.org/10.15468/dl.zfaw79
Anopheles funestus	34.026	0.770013	Unclassified	GBIF: https://doi.org/10.15468/dl.zfaw79
Anopheles funestus	34.026	0.770045	Unclassified	GBIF: https://doi.org/10.15468/dl.zfaw79
Anopheles funestus	34.026	0.77003	Unclassified	GBIF: https://doi.org/10.15468/dl.zfaw79
Anopheles funestus	34.026	0.77004	Unclassified	GBIF: https://doi.org/10.15468/dl.zfaw79
Anopheles funestus	34.026	0.770022	Unclassified	GBIF: https://doi.org/10.15468/dl.zfaw79
Anopheles funestus	34.026	0.770023	Unclassified	GBIF: https://doi.org/10.15468/dl.zfaw79
Anopheles funestus	34.026	0.770021	Unclassified	GBIF: https://doi.org/10.15468/dl.zfaw79
Anopheles funestus	34.026	0.770034	Unclassified	GBIF: https://doi.org/10.15468/dl.zfaw79
Anopheles funestus	34.026	0.770002	Unclassified	GBIF: https://doi.org/10.15468/dl.zfaw79
Anopheles funestus	34.026	0.770046	Unclassified	GBIF: https://doi.org/10.15468/dl.zfaw79
Anopheles funestus	34.026	0.770016	Unclassified	GBIF: https://doi.org/10.15468/dl.zfaw79
Anopheles funestus	34.026	0.770001	Unclassified	GBIF: https://doi.org/10.15468/dl.zfaw79
Anopheles funestus	34.0261	0.77	Unclassified	GBIF: https://doi.org/10.15468/dl.zfaw79
Anopheles funestus	34.0261	0.770058	Unclassified	GBIF: https://doi.org/10.15468/dl.zfaw79
Anopheles funestus	34.0261	0.770113	Unclassified	GBIF: https://doi.org/10.15468/dl.zfaw79
Anopheles funestus	34.0261	0.770075	Unclassified	GBIF: https://doi.org/10.15468/dl.zfaw79
Anopheles funestus	34.0261	0.7701	Unclassified	GBIF: https://doi.org/10.15468/dl.zfaw79
Anopheles funestus	34.0261	0.770054	Unclassified	GBIF: https://doi.org/10.15468/dl.zfaw79
Anopheles funestus	34.0261	0.770073	Unclassified	GBIF: https://doi.org/10.15468/dl.zfaw79
Anopheles funestus	34.0261	0.77007	Unclassified	GBIF: https://doi.org/10.15468/dl.zfaw79
Anopheles funestus	34.0261	0.770053	Unclassified	GBIF: https://doi.org/10.15468/dl.zfaw79
Anopheles funestus	34.0261	0.770088	Unclassified	GBIF: https://doi.org/10.15468/dl.zfaw79
Anopheles funestus	34.0261	0.770098	Unclassified	GBIF: https://doi.org/10.15468/dl.zfaw79
Anopheles funestus	34.0261	0.77009	Unclassified	GBIF: https://doi.org/10.15468/dl.zfaw79
Anopheles funestus	34.0261	0.770085	Unclassified	GBIF: https://doi.org/10.15468/dl.zfaw79
Anopheles funestus	34.0261	0.770106	Unclassified	GBIF: https://doi.org/10.15468/dl.zfaw79
Anopheles funestus	34.0261	0.770087	Unclassified	GBIF: https://doi.org/10.15468/dl.zfaw79
Anopheles funestus	34.0261	0.770077	Unclassified	GBIF: https://doi.org/10.15468/dl.zfaw79
Anopheles funestus	34.0261	0.770114	Unclassified	GBIF: https://doi.org/10.15468/dl.zfaw79
Anopheles funestus	34.0261	0.770072	Unclassified	GBIF: https://doi.org/10.15468/dl.zfaw79
Anopheles funestus	34.0261	0.77005	Unclassified	GBIF: https://doi.org/10.15468/dl.zfaw79

Anopheles funestus	34.0261	0.770107	Unclassified	GBIF: https://doi.org/10.15468/dl.zfaw79
Anopheles funestus	34.0261	0.770052	Unclassified	GBIF: https://doi.org/10.15468/dl.zfaw79
Anopheles funestus	34.0261	0.770056	Unclassified	GBIF: https://doi.org/10.15468/dl.zfaw79
Anopheles funestus	34.0261	0.770066	Unclassified	GBIF: https://doi.org/10.15468/dl.zfaw79
Anopheles funestus	34.0261	0.770091	Unclassified	GBIF: https://doi.org/10.15468/dl.zfaw79
Anopheles funestus	34.0261	0.770092	Unclassified	GBIF: https://doi.org/10.15468/dl.zfaw79
Anopheles funestus	34.0261	0.770063	Unclassified	GBIF: https://doi.org/10.15468/dl.zfaw79
Anopheles funestus	34.0261	0.770069	Unclassified	GBIF: https://doi.org/10.15468/dl.zfaw79
Anopheles funestus	34.0261	0.770082	Unclassified	GBIF: https://doi.org/10.15468/dl.zfaw79
Anopheles funestus	34.0261	0.770074	Unclassified	GBIF: https://doi.org/10.15468/dl.zfaw79
Anopheles funestus	34.0261	0.770089	Unclassified	GBIF: https://doi.org/10.15468/dl.zfaw79
Anopheles funestus	34.0261	0.770057	Unclassified	GBIF: https://doi.org/10.15468/dl.zfaw79
Anopheles funestus	34.0261	0.770061	Unclassified	GBIF: https://doi.org/10.15468/dl.zfaw79
Anopheles funestus	34.0261	0.770083	Unclassified	GBIF: https://doi.org/10.15468/dl.zfaw79
Anopheles funestus	34.0261	0.770062	Unclassified	GBIF: https://doi.org/10.15468/dl.zfaw79
Anopheles funestus	34.0261	0.770095	Unclassified	GBIF: https://doi.org/10.15468/dl.zfaw79
Anopheles funestus	34.0261	0.770084	Unclassified	GBIF: https://doi.org/10.15468/dl.zfaw79
Anopheles funestus	34.0261	0.770116	Unclassified	GBIF: https://doi.org/10.15468/dl.zfaw79
Anopheles funestus	34.0261	0.770071	Unclassified	GBIF: https://doi.org/10.15468/dl.zfaw79
Anopheles funestus	34.0261	0.77011	Unclassified	GBIF: https://doi.org/10.15468/dl.zfaw79
Anopheles funestus	34.0261	0.770055	Unclassified	GBIF: https://doi.org/10.15468/dl.zfaw79
Anopheles funestus	34.0261	0.770112	Unclassified	GBIF: https://doi.org/10.15468/dl.zfaw79
Anopheles funestus	34.0261	0.770065	Unclassified	GBIF: https://doi.org/10.15468/dl.zfaw79
Anopheles funestus	34.0261	0.77008	Unclassified	GBIF: https://doi.org/10.15468/dl.zfaw79
Anopheles funestus	34.0261	0.77006	Unclassified	GBIF: https://doi.org/10.15468/dl.zfaw79
Anopheles funestus	34.0261	0.770051	Unclassified	GBIF: https://doi.org/10.15468/dl.zfaw79
Anopheles funestus	34.0261	0.770059	Unclassified	GBIF: https://doi.org/10.15468/dl.zfaw79
Anopheles funestus	34.0261	0.770097	Unclassified	GBIF: https://doi.org/10.15468/dl.zfaw79
Anopheles funestus	34.0261	0.770067	Unclassified	GBIF: https://doi.org/10.15468/dl.zfaw79
Anopheles funestus	34.0261	0.770118	Unclassified	GBIF: https://doi.org/10.15468/dl.zfaw79
Anopheles funestus	34.0261	0.770049	Unclassified	GBIF: https://doi.org/10.15468/dl.zfaw79
Anopheles funestus	34.0261	0.770096	Unclassified	GBIF: https://doi.org/10.15468/dl.zfaw79
Anopheles funestus	34.0261	0.770086	Unclassified	GBIF: https://doi.org/10.15468/dl.zfaw79
Anopheles funestus	34.0261	0.770094	Unclassified	GBIF: https://doi.org/10.15468/dl.zfaw79
Anopheles funestus	34.0261	0.770109	Unclassified	GBIF: https://doi.org/10.15468/dl.zfaw79
Anopheles funestus	34.0261	0.770079	Unclassified	GBIF: https://doi.org/10.15468/dl.zfaw79
Anopheles funestus	34.0261	0.770081	Unclassified	GBIF: https://doi.org/10.15468/dl.zfaw79
Anopheles funestus	34.0261	0.770101	Unclassified	GBIF: https://doi.org/10.15468/dl.zfaw79
Anopheles funestus	34.0261	0.770093	Unclassified	GBIF: https://doi.org/10.15468/dl.zfaw79
Anopheles funestus	34.0261	0.770099	Unclassified	GBIF: https://doi.org/10.15468/dl.zfaw79
Anopheles funestus	34.0261	0.770076	Unclassified	GBIF: https://doi.org/10.15468/dl.zfaw79
Anopheles funestus	34.0261	0.770064	Unclassified	GBIF: https://doi.org/10.15468/dl.zfaw79

Anopheles funestus	34.0261	0.770103	Unclassified	GBIF: https://doi.org/10.15468/dl.zfaw79
Anopheles funestus	34.0261	0.770108	Unclassified	GBIF: https://doi.org/10.15468/dl.zfaw79
Anopheles funestus	34.0261	0.770115	Unclassified	GBIF: https://doi.org/10.15468/dl.zfaw79
Anopheles funestus	34.0261	0.770111	Unclassified	GBIF: https://doi.org/10.15468/dl.zfaw79
Anopheles funestus	34.0261	0.770117	Unclassified	GBIF: https://doi.org/10.15468/dl.zfaw79
Anopheles funestus	34.0261	0.770105	Unclassified	GBIF: https://doi.org/10.15468/dl.zfaw79
Anopheles funestus	34.0261	0.770078	Unclassified	GBIF: https://doi.org/10.15468/dl.zfaw79
Anopheles funestus	34.0261	0.770068	Unclassified	GBIF: https://doi.org/10.15468/dl.zfaw79
Anopheles funestus	34.0261	0.770104	Unclassified	GBIF: https://doi.org/10.15468/dl.zfaw79
Anopheles funestus	34.05	0.26	Unclassified	GBIF: https://doi.org/10.15468/dl.zfaw79
Anopheles funestus	34.06	-0.07	Unclassified	GBIF: https://doi.org/10.15468/dl.zfaw79
Anopheles funestus	34.1	0.21	Unclassified	GBIF: https://doi.org/10.15468/dl.zfaw79
Anopheles funestus	34.1847	0.681882	Unclassified	GBIF: https://doi.org/10.15468/dl.zfaw79
Anopheles funestus	34.2	-0.43	Unclassified	GBIF: https://doi.org/10.15468/dl.zfaw79
Anopheles funestus	34.27	-0.13	Unclassified	GBIF: https://doi.org/10.15468/dl.zfaw79
Anopheles funestus	34.31	0.63	Unclassified	GBIF: https://doi.org/10.15468/dl.zfaw79
Anopheles funestus	34.5	0.50002	Unclassified	GBIF: https://doi.org/10.15468/dl.zfaw79
Anopheles funestus	34.5	0.50003	Unclassified	GBIF: https://doi.org/10.15468/dl.zfaw79
Anopheles funestus	34.5	0.50004	Unclassified	GBIF: https://doi.org/10.15468/dl.zfaw79
Anopheles funestus	34.5	0.50001	Unclassified	GBIF: https://doi.org/10.15468/dl.zfaw79
Anopheles funestus	34.5	0.50005	Unclassified	GBIF: https://doi.org/10.15468/dl.zfaw79
Anopheles funestus	34.5001	0.50008	Unclassified	GBIF: https://doi.org/10.15468/dl.zfaw79
Anopheles funestus	34.5001	0.50006	Unclassified	GBIF: https://doi.org/10.15468/dl.zfaw79
Anopheles funestus	34.5001	0.50009	Unclassified	GBIF: https://doi.org/10.15468/dl.zfaw79
Anopheles funestus	34.5001	0.50013	Unclassified	GBIF: https://doi.org/10.15468/dl.zfaw79
Anopheles funestus	34.5001	0.50011	Unclassified	GBIF: https://doi.org/10.15468/dl.zfaw79
Anopheles funestus	34.5001	0.50014	Unclassified	GBIF: https://doi.org/10.15468/dl.zfaw79
Anopheles funestus	34.5001	0.5001	Unclassified	GBIF: https://doi.org/10.15468/dl.zfaw79
Anopheles funestus	34.5001	0.50015	Unclassified	GBIF: https://doi.org/10.15468/dl.zfaw79
Anopheles funestus	34.5001	0.50012	Unclassified	GBIF: https://doi.org/10.15468/dl.zfaw79
Anopheles funestus	34.5001	0.50007	Unclassified	GBIF: https://doi.org/10.15468/dl.zfaw79
Anopheles funestus	34.5002	0.50019	Unclassified	GBIF: https://doi.org/10.15468/dl.zfaw79
Anopheles funestus	34.5002	0.50024	Unclassified	GBIF: https://doi.org/10.15468/dl.zfaw79
Anopheles funestus	34.5002	0.50022	Unclassified	GBIF: https://doi.org/10.15468/dl.zfaw79
Anopheles funestus	34.5002	0.50016	Unclassified	GBIF: https://doi.org/10.15468/dl.zfaw79
Anopheles funestus	34.5002	0.50023	Unclassified	GBIF: https://doi.org/10.15468/dl.zfaw79
Anopheles funestus	34.5002	0.5002	Unclassified	GBIF: https://doi.org/10.15468/dl.zfaw79
Anopheles funestus	34.5002	0.50021	Unclassified	GBIF: https://doi.org/10.15468/dl.zfaw79
Anopheles funestus	34.5002	0.50018	Unclassified	GBIF: https://doi.org/10.15468/dl.zfaw79
Anopheles funestus	34.5002	0.50017	Unclassified	GBIF: https://doi.org/10.15468/dl.zfaw79
Anopheles funestus	34.5003	0.50034	Unclassified	GBIF: https://doi.org/10.15468/dl.zfaw79
Anopheles funestus	34.5003	0.50025	Unclassified	GBIF: https://doi.org/10.15468/dl.zfaw79

Anopheles funestus	34.5003	0.50026	Unclassified	GBIF: https://doi.org/10.15468/dl.zfaw79
Anopheles funestus	34.5003	0.50027	Unclassified	GBIF: https://doi.org/10.15468/dl.zfaw79
Anopheles funestus	34.5003	0.50033	Unclassified	GBIF: https://doi.org/10.15468/dl.zfaw79
Anopheles funestus	34.5003	0.50028	Unclassified	GBIF: https://doi.org/10.15468/dl.zfaw79
Anopheles funestus	34.5003	0.50031	Unclassified	GBIF: https://doi.org/10.15468/dl.zfaw79
Anopheles funestus	34.5003	0.50032	Unclassified	GBIF: https://doi.org/10.15468/dl.zfaw79
Anopheles funestus	34.5003	0.50029	Unclassified	GBIF: https://doi.org/10.15468/dl.zfaw79
Anopheles funestus	34.5003	0.5003	Unclassified	GBIF: https://doi.org/10.15468/dl.zfaw79
Anopheles funestus	34.5004	0.50035	Unclassified	GBIF: https://doi.org/10.15468/dl.zfaw79
Anopheles funestus	34.5004	0.50037	Unclassified	GBIF: https://doi.org/10.15468/dl.zfaw79
Anopheles funestus	34.5004	0.50041	Unclassified	GBIF: https://doi.org/10.15468/dl.zfaw79
Anopheles funestus	34.5004	0.50039	Unclassified	GBIF: https://doi.org/10.15468/dl.zfaw79
Anopheles funestus	34.5004	0.50044	Unclassified	GBIF: https://doi.org/10.15468/dl.zfaw79
Anopheles funestus	34.5004	0.50043	Unclassified	GBIF: https://doi.org/10.15468/dl.zfaw79
Anopheles funestus	34.5004	0.50036	Unclassified	GBIF: https://doi.org/10.15468/dl.zfaw79
Anopheles funestus	34.5004	0.5004	Unclassified	GBIF: https://doi.org/10.15468/dl.zfaw79
Anopheles funestus	34.5004	0.50042	Unclassified	GBIF: https://doi.org/10.15468/dl.zfaw79
Anopheles funestus	34.5004	0.50038	Unclassified	GBIF: https://doi.org/10.15468/dl.zfaw79
Anopheles funestus	34.5005	0.50046	Unclassified	GBIF: https://doi.org/10.15468/dl.zfaw79
Anopheles funestus	34.5005	0.50048	Unclassified	GBIF: https://doi.org/10.15468/dl.zfaw79
Anopheles funestus	34.5005	0.50047	Unclassified	GBIF: https://doi.org/10.15468/dl.zfaw79
Anopheles funestus	34.5005	0.50051	Unclassified	GBIF: https://doi.org/10.15468/dl.zfaw79
Anopheles funestus	34.5005	0.5005	Unclassified	GBIF: https://doi.org/10.15468/dl.zfaw79
Anopheles funestus	34.5005	0.50049	Unclassified	GBIF: https://doi.org/10.15468/dl.zfaw79
Anopheles funestus	34.5005	0.50045	Unclassified	GBIF: https://doi.org/10.15468/dl.zfaw79
Anopheles funestus	34.5005	0.50052	Unclassified	GBIF: https://doi.org/10.15468/dl.zfaw79
Anopheles funestus	34.5006	0.50058	Unclassified	GBIF: https://doi.org/10.15468/dl.zfaw79
Anopheles funestus	34.5007	0.5007	Unclassified	GBIF: https://doi.org/10.15468/dl.zfaw79
Anopheles funestus	34.5007	0.50067	Unclassified	GBIF: https://doi.org/10.15468/dl.zfaw79
Anopheles funestus	34.5007	0.50066	Unclassified	GBIF: https://doi.org/10.15468/dl.zfaw79
Anopheles funestus	34.5007	0.50072	Unclassified	GBIF: https://doi.org/10.15468/dl.zfaw79
Anopheles funestus	34.5008	0.50078	Unclassified	GBIF: https://doi.org/10.15468/dl.zfaw79
Anopheles funestus	34.5008	0.50083	Unclassified	GBIF: https://doi.org/10.15468/dl.zfaw79
Anopheles funestus	34.5008	0.50084	Unclassified	GBIF: https://doi.org/10.15468/dl.zfaw79
Anopheles funestus	34.5008	0.50081	Unclassified	GBIF: https://doi.org/10.15468/dl.zfaw79
Anopheles funestus	34.5008	0.50077	Unclassified	GBIF: https://doi.org/10.15468/dl.zfaw79
Anopheles funestus	34.5008	0.50082	Unclassified	GBIF: https://doi.org/10.15468/dl.zfaw79
Anopheles funestus	34.5009	0.50087	Unclassified	GBIF: https://doi.org/10.15468/dl.zfaw79
Anopheles funestus	34.5009	0.50089	Unclassified	GBIF: https://doi.org/10.15468/dl.zfaw79
Anopheles funestus	34.92	0.17	Unclassified	GBIF: https://doi.org/10.15468/dl.zfaw79
Anopheles funestus	34.96	0.8	Unclassified	GBIF: https://doi.org/10.15468/dl.zfaw79
Anopheles funestus	35.31	-23.72	Unclassified	GBIF: https://doi.org/10.15468/dl.zfaw79

Anopheles funestus	35.528479	-14.873062	Unclassified	GBIF: https://doi.org/10.15468/dl.zfaw79
Anopheles funestus	36	-16.9	Unclassified	GBIF: https://doi.org/10.15468/dl.zfaw79
Anopheles funestus	36.08	0.45	Unclassified	GBIF: https://doi.org/10.15468/dl.zfaw79
Anopheles funestus	36.1	0.46	Unclassified	GBIF: https://doi.org/10.15468/dl.zfaw79
Anopheles funestus	36.36	-8.16668	Unclassified	GBIF: https://doi.org/10.15468/dl.zfaw79
Anopheles funestus	36.3623	-8.17202	Unclassified	GBIF: https://doi.org/10.15468/dl.zfaw79
Anopheles funestus	36.3627	-8.15718	Unclassified	GBIF: https://doi.org/10.15468/dl.zfaw79
Anopheles funestus	36.3644	-8.15527	Unclassified	GBIF: https://doi.org/10.15468/dl.zfaw79
Anopheles funestus	36.365	-8.16375	Unclassified	GBIF: https://doi.org/10.15468/dl.zfaw79
Anopheles funestus	36.3681	-8.1713	Unclassified	GBIF: https://doi.org/10.15468/dl.zfaw79
Anopheles funestus	36.3694	-8.15503	Unclassified	GBIF: https://doi.org/10.15468/dl.zfaw79
Anopheles funestus	36.3715	-8.16998	Unclassified	GBIF: https://doi.org/10.15468/dl.zfaw79
Anopheles funestus	36.3729	-8.1803	Unclassified	GBIF: https://doi.org/10.15468/dl.zfaw79
Anopheles funestus	36.3807	-8.18135	Unclassified	GBIF: https://doi.org/10.15468/dl.zfaw79
Anopheles funestus	36.384	-8.1683	Unclassified	GBIF: https://doi.org/10.15468/dl.zfaw79
Anopheles funestus	36.386	-8.18618	Unclassified	GBIF: https://doi.org/10.15468/dl.zfaw79
Anopheles funestus	36.387	-8.18568	Unclassified	GBIF: https://doi.org/10.15468/dl.zfaw79
Anopheles funestus	36.3872	-8.1701	Unclassified	GBIF: https://doi.org/10.15468/dl.zfaw79
Anopheles funestus	36.3876	-8.18327	Unclassified	GBIF: https://doi.org/10.15468/dl.zfaw79
Anopheles funestus	36.3876	-8.2072	Unclassified	GBIF: https://doi.org/10.15468/dl.zfaw79
Anopheles funestus	36.3883	-8.17953	Unclassified	GBIF: https://doi.org/10.15468/dl.zfaw79
Anopheles funestus	36.3885	-8.1748	Unclassified	GBIF: https://doi.org/10.15468/dl.zfaw79
Anopheles funestus	36.3893	-8.17788	Unclassified	GBIF: https://doi.org/10.15468/dl.zfaw79
Anopheles funestus	36.3907	-8.15435	Unclassified	GBIF: https://doi.org/10.15468/dl.zfaw79
Anopheles funestus	36.3908	-8.17755	Unclassified	GBIF: https://doi.org/10.15468/dl.zfaw79
Anopheles funestus	36.3917	-8.15608	Unclassified	GBIF: https://doi.org/10.15468/dl.zfaw79
Anopheles funestus	36.3919	-8.17193	Unclassified	GBIF: https://doi.org/10.15468/dl.zfaw79
Anopheles funestus	36.3919	-8.17112	Unclassified	GBIF: https://doi.org/10.15468/dl.zfaw79
Anopheles funestus	36.3938	-8.15408	Unclassified	GBIF: https://doi.org/10.15468/dl.zfaw79
Anopheles funestus	36.3942	-8.15687	Unclassified	GBIF: https://doi.org/10.15468/dl.zfaw79
Anopheles funestus	36.3943	-8.15422	Unclassified	GBIF: https://doi.org/10.15468/dl.zfaw79
Anopheles funestus	36.3943	-8.15415	Unclassified	GBIF: https://doi.org/10.15468/dl.zfaw79
Anopheles funestus	36.3949	-8.1661	Unclassified	GBIF: https://doi.org/10.15468/dl.zfaw79
Anopheles funestus	36.3952	-8.15354	Unclassified	GBIF: https://doi.org/10.15468/dl.zfaw79
Anopheles funestus	36.3953	-8.167	Unclassified	GBIF: https://doi.org/10.15468/dl.zfaw79
Anopheles funestus	36.3954	-8.167	Unclassified	GBIF: https://doi.org/10.15468/dl.zfaw79
Anopheles funestus	36.3956	-8.15948	Unclassified	GBIF: https://doi.org/10.15468/dl.zfaw79
Anopheles funestus	36.3956	-8.15543	Unclassified	GBIF: https://doi.org/10.15468/dl.zfaw79
Anopheles funestus	36.3956	-8.15978	Unclassified	GBIF: https://doi.org/10.15468/dl.zfaw79
Anopheles funestus	36.3958	-8.15662	Unclassified	GBIF: https://doi.org/10.15468/dl.zfaw79
Anopheles funestus	36.3959	-8.15885	Unclassified	GBIF: https://doi.org/10.15468/dl.zfaw79
Anopheles funestus	36.3963	-8.15737	Unclassified	GBIF: https://doi.org/10.15468/dl.zfaw79

Anopheles funestus	36.3975	-8.15263	Unclassified	GBIF: https://doi.org/10.15468/dl.zfaw79
Anopheles funestus	36.3976	-8.15592	Unclassified	GBIF: https://doi.org/10.15468/dl.zfaw79
Anopheles funestus	36.3979	-8.15625	Unclassified	GBIF: https://doi.org/10.15468/dl.zfaw79
Anopheles funestus	36.3986	-8.15317	Unclassified	GBIF: https://doi.org/10.15468/dl.zfaw79
Anopheles funestus	36.3991	-8.15375	Unclassified	GBIF: https://doi.org/10.15468/dl.zfaw79
Anopheles funestus	36.3992	-8.15548	Unclassified	GBIF: https://doi.org/10.15468/dl.zfaw79
Anopheles funestus	36.3994	-8.15638	Unclassified	GBIF: https://doi.org/10.15468/dl.zfaw79
Anopheles funestus	36.3996	-8.15255	Unclassified	GBIF: https://doi.org/10.15468/dl.zfaw79
Anopheles funestus	36.3998	-8.16152	Unclassified	GBIF: https://doi.org/10.15468/dl.zfaw79
Anopheles funestus	36.4002	-8.15532	Unclassified	GBIF: https://doi.org/10.15468/dl.zfaw79
Anopheles funestus	36.401	-8.23767	Unclassified	GBIF: https://doi.org/10.15468/dl.zfaw79
Anopheles funestus	36.4013	-8.23812	Unclassified	GBIF: https://doi.org/10.15468/dl.zfaw79
Anopheles funestus	36.4025	-8.15143	Unclassified	GBIF: https://doi.org/10.15468/dl.zfaw79
Anopheles funestus	36.4027	-8.1721	Unclassified	GBIF: https://doi.org/10.15468/dl.zfaw79
Anopheles funestus	36.4037	-8.1729	Unclassified	GBIF: https://doi.org/10.15468/dl.zfaw79
Anopheles funestus	36.4038	-8.1729	Unclassified	GBIF: https://doi.org/10.15468/dl.zfaw79
Anopheles funestus	36.405	-8.15203	Unclassified	GBIF: https://doi.org/10.15468/dl.zfaw79
Anopheles funestus	36.409	-8.1473	Unclassified	GBIF: https://doi.org/10.15468/dl.zfaw79
Anopheles funestus	36.4123	-8.1633	Unclassified	GBIF: https://doi.org/10.15468/dl.zfaw79
Anopheles funestus	36.4173	-8.14743	Unclassified	GBIF: https://doi.org/10.15468/dl.zfaw79
Anopheles funestus	36.4175	-8.1482	Unclassified	GBIF: https://doi.org/10.15468/dl.zfaw79
Anopheles funestus	36.4178	-8.22123	Unclassified	GBIF: https://doi.org/10.15468/dl.zfaw79
Anopheles funestus	36.4191	-8.22228	Unclassified	GBIF: https://doi.org/10.15468/dl.zfaw79
Anopheles funestus	36.4193	-8.14423	Unclassified	GBIF: https://doi.org/10.15468/dl.zfaw79
Anopheles funestus	36.4209	-8.14275	Unclassified	GBIF: https://doi.org/10.15468/dl.zfaw79
Anopheles funestus	36.4223	-8.1768	Unclassified	GBIF: https://doi.org/10.15468/dl.zfaw79
Anopheles funestus	36.424	-8.1382	Unclassified	GBIF: https://doi.org/10.15468/dl.zfaw79
Anopheles funestus	36.4442	-8.2197	Unclassified	GBIF: https://doi.org/10.15468/dl.zfaw79
Anopheles funestus	36.4464	-8.1836	Unclassified	GBIF: https://doi.org/10.15468/dl.zfaw79
Anopheles funestus	36.4809	-8.2098	Unclassified	GBIF: https://doi.org/10.15468/dl.zfaw79
Anopheles funestus	36.4871	-8.08422	Unclassified	GBIF: https://doi.org/10.15468/dl.zfaw79
Anopheles funestus	36.4891	-8.07934	Unclassified	GBIF: https://doi.org/10.15468/dl.zfaw79
Anopheles funestus	36.4897	-8.2256	Unclassified	GBIF: https://doi.org/10.15468/dl.zfaw79
Anopheles funestus	36.4948	-8.08026	Unclassified	GBIF: https://doi.org/10.15468/dl.zfaw79
Anopheles funestus	36.4989	-8.08888	Unclassified	GBIF: https://doi.org/10.15468/dl.zfaw79
Anopheles funestus	36.499	-8.09943	Unclassified	GBIF: https://doi.org/10.15468/dl.zfaw79
Anopheles funestus	36.4999	-8.09793	Unclassified	GBIF: https://doi.org/10.15468/dl.zfaw79
Anopheles funestus	36.5013	-8.09985	Unclassified	GBIF: https://doi.org/10.15468/dl.zfaw79
Anopheles funestus	36.5022	-8.09957	Unclassified	GBIF: https://doi.org/10.15468/dl.zfaw79
Anopheles funestus	36.506	-8.10022	Unclassified	GBIF: https://doi.org/10.15468/dl.zfaw79
Anopheles funestus	36.5065	-8.0972	Unclassified	GBIF: https://doi.org/10.15468/dl.zfaw79
Anopheles funestus	36.5071	-8.10013	Unclassified	GBIF: https://doi.org/10.15468/dl.zfaw79

Anopheles funestus	36.5084	-8.1015	Unclassified	GBIF: https://doi.org/10.15468/dl.zfaw79
Anopheles funestus	36.5088	-8.08348	Unclassified	GBIF: https://doi.org/10.15468/dl.zfaw79
Anopheles funestus	36.5088	-8.10113	Unclassified	GBIF: https://doi.org/10.15468/dl.zfaw79
Anopheles funestus	36.5089	-8.10205	Unclassified	GBIF: https://doi.org/10.15468/dl.zfaw79
Anopheles funestus	36.509	-8.1024	Unclassified	GBIF: https://doi.org/10.15468/dl.zfaw79
Anopheles funestus	36.509	-8.09308	Unclassified	GBIF: https://doi.org/10.15468/dl.zfaw79
Anopheles funestus	36.5092	-8.10068	Unclassified	GBIF: https://doi.org/10.15468/dl.zfaw79
Anopheles funestus	36.5093	-8.09962	Unclassified	GBIF: https://doi.org/10.15468/dl.zfaw79
Anopheles funestus	36.5093	-8.1007	Unclassified	GBIF: https://doi.org/10.15468/dl.zfaw79
Anopheles funestus	36.5097	-8.09598	Unclassified	GBIF: https://doi.org/10.15468/dl.zfaw79
Anopheles funestus	36.5098	-8.09598	Unclassified	GBIF: https://doi.org/10.15468/dl.zfaw79
Anopheles funestus	36.51	-8.10127	Unclassified	GBIF: https://doi.org/10.15468/dl.zfaw79
Anopheles funestus	36.5103	-8.10002	Unclassified	GBIF: https://doi.org/10.15468/dl.zfaw79
Anopheles funestus	36.5103	-8.09778	Unclassified	GBIF: https://doi.org/10.15468/dl.zfaw79
Anopheles funestus	36.5104	-8.09688	Unclassified	GBIF: https://doi.org/10.15468/dl.zfaw79
Anopheles funestus	36.5105	-8.09998	Unclassified	GBIF: https://doi.org/10.15468/dl.zfaw79
Anopheles funestus	36.5107	-8.10348	Unclassified	GBIF: https://doi.org/10.15468/dl.zfaw79
Anopheles funestus	36.5108	-8.10037	Unclassified	GBIF: https://doi.org/10.15468/dl.zfaw79
Anopheles funestus	36.511	-8.09795	Unclassified	GBIF: https://doi.org/10.15468/dl.zfaw79
Anopheles funestus	36.511	-8.10002	Unclassified	GBIF: https://doi.org/10.15468/dl.zfaw79
Anopheles funestus	36.5111	-8.09627	Unclassified	GBIF: https://doi.org/10.15468/dl.zfaw79
Anopheles funestus	36.5111	-8.10248	Unclassified	GBIF: https://doi.org/10.15468/dl.zfaw79
Anopheles funestus	36.5111	-8.098	Unclassified	GBIF: https://doi.org/10.15468/dl.zfaw79
Anopheles funestus	36.5114	-8.09942	Unclassified	GBIF: https://doi.org/10.15468/dl.zfaw79
Anopheles funestus	36.5115	-8.10038	Unclassified	GBIF: https://doi.org/10.15468/dl.zfaw79
Anopheles funestus	36.5116	-8.0941	Unclassified	GBIF: https://doi.org/10.15468/dl.zfaw79
Anopheles funestus	36.5122	-8.10003	Unclassified	GBIF: https://doi.org/10.15468/dl.zfaw79
Anopheles funestus	36.5128	-8.09644	Unclassified	GBIF: https://doi.org/10.15468/dl.zfaw79
Anopheles funestus	36.5131	-8.1007	Unclassified	GBIF: https://doi.org/10.15468/dl.zfaw79
Anopheles funestus	36.5155	-8.10778	Unclassified	GBIF: https://doi.org/10.15468/dl.zfaw79
Anopheles funestus	36.5168	-8.1001	Unclassified	GBIF: https://doi.org/10.15468/dl.zfaw79
Anopheles funestus	36.5173	-8.09877	Unclassified	GBIF: https://doi.org/10.15468/dl.zfaw79
Anopheles funestus	36.5177	-8.09588	Unclassified	GBIF: https://doi.org/10.15468/dl.zfaw79
Anopheles funestus	36.5178	-8.09894	Unclassified	GBIF: https://doi.org/10.15468/dl.zfaw79
Anopheles funestus	36.5182	-8.09975	Unclassified	GBIF: https://doi.org/10.15468/dl.zfaw79
Anopheles funestus	36.5189	-8.09853	Unclassified	GBIF: https://doi.org/10.15468/dl.zfaw79
Anopheles funestus	36.5199	-8.10857	Unclassified	GBIF: https://doi.org/10.15468/dl.zfaw79
Anopheles funestus	36.5201	-8.09785	Unclassified	GBIF: https://doi.org/10.15468/dl.zfaw79
Anopheles funestus	36.5202	-8.10823	Unclassified	GBIF: https://doi.org/10.15468/dl.zfaw79
Anopheles funestus	36.5202	-8.0986	Unclassified	GBIF: https://doi.org/10.15468/dl.zfaw79
Anopheles funestus	36.5202	-8.1081	Unclassified	GBIF: https://doi.org/10.15468/dl.zfaw79
Anopheles funestus	36.5205	-8.1017	Unclassified	GBIF: https://doi.org/10.15468/dl.zfaw79

Anopheles funestus	36.5206	-8.10062	Unclassified	GBIF: https://doi.org/10.15468/dl.zfaw79
Anopheles funestus	36.5208	-8.1011	Unclassified	GBIF: https://doi.org/10.15468/dl.zfaw79
Anopheles funestus	36.5209	-8.09753	Unclassified	GBIF: https://doi.org/10.15468/dl.zfaw79
Anopheles funestus	36.521	-8.0977	Unclassified	GBIF: https://doi.org/10.15468/dl.zfaw79
Anopheles funestus	36.5214	-8.1098	Unclassified	GBIF: https://doi.org/10.15468/dl.zfaw79
Anopheles funestus	36.5227	-8.09815	Unclassified	GBIF: https://doi.org/10.15468/dl.zfaw79
Anopheles funestus	36.5232	-8.09953	Unclassified	GBIF: https://doi.org/10.15468/dl.zfaw79
Anopheles funestus	36.5234	-8.108	Unclassified	GBIF: https://doi.org/10.15468/dl.zfaw79
Anopheles funestus	36.524	-8.10003	Unclassified	GBIF: https://doi.org/10.15468/dl.zfaw79
Anopheles funestus	36.524	-8.08778	Unclassified	GBIF: https://doi.org/10.15468/dl.zfaw79
Anopheles funestus	36.5243	-8.10058	Unclassified	GBIF: https://doi.org/10.15468/dl.zfaw79
Anopheles funestus	36.5264	-8.09977	Unclassified	GBIF: https://doi.org/10.15468/dl.zfaw79
Anopheles funestus	36.5283	-8.09658	Unclassified	GBIF: https://doi.org/10.15468/dl.zfaw79
Anopheles funestus	36.5283	-8.10648	Unclassified	GBIF: https://doi.org/10.15468/dl.zfaw79
Anopheles funestus	36.5289	-8.09548	Unclassified	GBIF: https://doi.org/10.15468/dl.zfaw79
Anopheles funestus	36.5296	-8.0651	Unclassified	GBIF: https://doi.org/10.15468/dl.zfaw79
Anopheles funestus	36.5308	-8.06853	Unclassified	GBIF: https://doi.org/10.15468/dl.zfaw79
Anopheles funestus	36.5323	-8.09362	Unclassified	GBIF: https://doi.org/10.15468/dl.zfaw79
Anopheles funestus	36.5336	-8.06665	Unclassified	GBIF: https://doi.org/10.15468/dl.zfaw79
Anopheles funestus	36.549	-8.06653	Unclassified	GBIF: https://doi.org/10.15468/dl.zfaw79
Anopheles funestus	36.5505	-8.11457	Unclassified	GBIF: https://doi.org/10.15468/dl.zfaw79
Anopheles funestus	36.5521	-8.11192	Unclassified	GBIF: https://doi.org/10.15468/dl.zfaw79
Anopheles funestus	36.5587	-8.13008	Unclassified	GBIF: https://doi.org/10.15468/dl.zfaw79
Anopheles funestus	36.6202	-8.41346	Unclassified	GBIF: https://doi.org/10.15468/dl.zfaw79
Anopheles funestus	36.6224	-8.41348	Unclassified	GBIF: https://doi.org/10.15468/dl.zfaw79
Anopheles funestus	36.6255	-8.41094	Unclassified	GBIF: https://doi.org/10.15468/dl.zfaw79
Anopheles funestus	36.6257	-8.41097	Unclassified	GBIF: https://doi.org/10.15468/dl.zfaw79
Anopheles funestus	36.6262	-8.41262	Unclassified	GBIF: https://doi.org/10.15468/dl.zfaw79
Anopheles funestus	36.6264	-8.4106	Unclassified	GBIF: https://doi.org/10.15468/dl.zfaw79
Anopheles funestus	36.6268	-8.41439	Unclassified	GBIF: https://doi.org/10.15468/dl.zfaw79
Anopheles funestus	36.6271	-8.41431	Unclassified	GBIF: https://doi.org/10.15468/dl.zfaw79
Anopheles funestus	36.6275	-8.40995	Unclassified	GBIF: https://doi.org/10.15468/dl.zfaw79
Anopheles funestus	36.6276	-8.41333	Unclassified	GBIF: https://doi.org/10.15468/dl.zfaw79
Anopheles funestus	36.6278	-8.40854	Unclassified	GBIF: https://doi.org/10.15468/dl.zfaw79
Anopheles funestus	36.6279	-8.40844	Unclassified	GBIF: https://doi.org/10.15468/dl.zfaw79
Anopheles funestus	36.6279	-8.41206	Unclassified	GBIF: https://doi.org/10.15468/dl.zfaw79
Anopheles funestus	36.6279	-8.41268	Unclassified	GBIF: https://doi.org/10.15468/dl.zfaw79
Anopheles funestus	36.6281	-8.40974	Unclassified	GBIF: https://doi.org/10.15468/dl.zfaw79
Anopheles funestus	36.6284	-8.4114	Unclassified	GBIF: https://doi.org/10.15468/dl.zfaw79
Anopheles funestus	36.6284	-8.4119	Unclassified	GBIF: https://doi.org/10.15468/dl.zfaw79
Anopheles funestus	36.6285	-8.41076	Unclassified	GBIF: https://doi.org/10.15468/dl.zfaw79
Anopheles funestus	36.6285	-8.40835	Unclassified	GBIF: https://doi.org/10.15468/dl.zfaw79

Anopheles funestus	36.6287	-8.41079	Unclassified	GBIF: https://doi.org/10.15468/dl.zfaw79
Anopheles funestus	36.6289	-8.41265	Unclassified	GBIF: https://doi.org/10.15468/dl.zfaw79
Anopheles funestus	36.629	-8.4089	Unclassified	GBIF: https://doi.org/10.15468/dl.zfaw79
Anopheles funestus	36.6296	-8.40965	Unclassified	GBIF: https://doi.org/10.15468/dl.zfaw79
Anopheles funestus	36.6297	-8.41156	Unclassified	GBIF: https://doi.org/10.15468/dl.zfaw79
Anopheles funestus	36.6299	-8.40894	Unclassified	GBIF: https://doi.org/10.15468/dl.zfaw79
Anopheles funestus	36.6299	-8.40929	Unclassified	GBIF: https://doi.org/10.15468/dl.zfaw79
Anopheles funestus	36.6302	-8.40816	Unclassified	GBIF: https://doi.org/10.15468/dl.zfaw79
Anopheles funestus	36.6304	-8.40884	Unclassified	GBIF: https://doi.org/10.15468/dl.zfaw79
Anopheles funestus	36.6304	-8.40857	Unclassified	GBIF: https://doi.org/10.15468/dl.zfaw79
Anopheles funestus	36.6306	-8.40676	Unclassified	GBIF: https://doi.org/10.15468/dl.zfaw79
Anopheles funestus	36.6306	-8.40844	Unclassified	GBIF: https://doi.org/10.15468/dl.zfaw79
Anopheles funestus	36.631	-8.41112	Unclassified	GBIF: https://doi.org/10.15468/dl.zfaw79
Anopheles funestus	36.6314	-8.41114	Unclassified	GBIF: https://doi.org/10.15468/dl.zfaw79
Anopheles funestus	36.6315	-8.41109	Unclassified	GBIF: https://doi.org/10.15468/dl.zfaw79
Anopheles funestus	36.6321	-8.40645	Unclassified	GBIF: https://doi.org/10.15468/dl.zfaw79
Anopheles funestus	36.683	-8.14607	Unclassified	GBIF: https://doi.org/10.15468/dl.zfaw79
Anopheles funestus	36.6908	-8.4796	Unclassified	GBIF: https://doi.org/10.15468/dl.zfaw79
Anopheles funestus	36.691	-8.47978	Unclassified	GBIF: https://doi.org/10.15468/dl.zfaw79
Anopheles funestus	36.6914	-8.48052	Unclassified	GBIF: https://doi.org/10.15468/dl.zfaw79
Anopheles funestus	36.6915	-8.48041	Unclassified	GBIF: https://doi.org/10.15468/dl.zfaw79
Anopheles funestus	36.6917	-8.47891	Unclassified	GBIF: https://doi.org/10.15468/dl.zfaw79
Anopheles funestus	36.6918	-8.48184	Unclassified	GBIF: https://doi.org/10.15468/dl.zfaw79
Anopheles funestus	36.6919	-8.47922	Unclassified	GBIF: https://doi.org/10.15468/dl.zfaw79
Anopheles funestus	36.692	-8.47982	Unclassified	GBIF: https://doi.org/10.15468/dl.zfaw79
Anopheles funestus	36.692	-8.47976	Unclassified	GBIF: https://doi.org/10.15468/dl.zfaw79
Anopheles funestus	36.6924	-8.48137	Unclassified	GBIF: https://doi.org/10.15468/dl.zfaw79
Anopheles funestus	36.6926	-8.48814	Unclassified	GBIF: https://doi.org/10.15468/dl.zfaw79
Anopheles funestus	36.6929	-8.48803	Unclassified	GBIF: https://doi.org/10.15468/dl.zfaw79
Anopheles funestus	36.6929	-8.48245	Unclassified	GBIF: https://doi.org/10.15468/dl.zfaw79
Anopheles funestus	36.6932	-8.4814	Unclassified	GBIF: https://doi.org/10.15468/dl.zfaw79
Anopheles funestus	36.6934	-8.48719	Unclassified	GBIF: https://doi.org/10.15468/dl.zfaw79
Anopheles funestus	36.6935	-8.48621	Unclassified	GBIF: https://doi.org/10.15468/dl.zfaw79
Anopheles funestus	36.6937	-8.48751	Unclassified	GBIF: https://doi.org/10.15468/dl.zfaw79
Anopheles funestus	36.6939	-8.4863	Unclassified	GBIF: https://doi.org/10.15468/dl.zfaw79
Anopheles funestus	36.694	-8.48647	Unclassified	GBIF: https://doi.org/10.15468/dl.zfaw79
Anopheles funestus	36.6941	-8.48609	Unclassified	GBIF: https://doi.org/10.15468/dl.zfaw79
Anopheles funestus	36.6941	-8.48625	Unclassified	GBIF: https://doi.org/10.15468/dl.zfaw79
Anopheles funestus	36.6941	-8.48635	Unclassified	GBIF: https://doi.org/10.15468/dl.zfaw79
Anopheles funestus	36.6942	-8.48729	Unclassified	GBIF: https://doi.org/10.15468/dl.zfaw79
Anopheles funestus	36.6945	-8.48585	Unclassified	GBIF: https://doi.org/10.15468/dl.zfaw79
Anopheles funestus	36.6946	-8.48736	Unclassified	GBIF: https://doi.org/10.15468/dl.zfaw79

Anopheles funestus	36.6947	-8.49933	Unclassified	GBIF: https://doi.org/10.15468/dl.zfaw79
Anopheles funestus	36.6948	-8.48607	Unclassified	GBIF: https://doi.org/10.15468/dl.zfaw79
Anopheles funestus	36.6948	-8.48604	Unclassified	GBIF: https://doi.org/10.15468/dl.zfaw79
Anopheles funestus	36.6949	-8.49962	Unclassified	GBIF: https://doi.org/10.15468/dl.zfaw79
Anopheles funestus	36.6949	-8.50027	Unclassified	GBIF: https://doi.org/10.15468/dl.zfaw79
Anopheles funestus	36.6949	-8.50019	Unclassified	GBIF: https://doi.org/10.15468/dl.zfaw79
Anopheles funestus	36.6949	-8.48582	Unclassified	GBIF: https://doi.org/10.15468/dl.zfaw79
Anopheles funestus	36.6949	-8.48945	Unclassified	GBIF: https://doi.org/10.15468/dl.zfaw79
Anopheles funestus	36.6949	-8.50037	Unclassified	GBIF: https://doi.org/10.15468/dl.zfaw79
Anopheles funestus	36.6949	-8.48596	Unclassified	GBIF: https://doi.org/10.15468/dl.zfaw79
Anopheles funestus	36.6953	-8.4898	Unclassified	GBIF: https://doi.org/10.15468/dl.zfaw79
Anopheles funestus	36.6953	-8.5202	Unclassified	GBIF: https://doi.org/10.15468/dl.zfaw79
Anopheles funestus	36.6954	-8.51941	Unclassified	GBIF: https://doi.org/10.15468/dl.zfaw79
Anopheles funestus	36.6958	-8.50051	Unclassified	GBIF: https://doi.org/10.15468/dl.zfaw79
Anopheles funestus	36.6958	-8.50058	Unclassified	GBIF: https://doi.org/10.15468/dl.zfaw79
Anopheles funestus	36.6959	-8.52399	Unclassified	GBIF: https://doi.org/10.15468/dl.zfaw79
Anopheles funestus	36.696	-8.51981	Unclassified	GBIF: https://doi.org/10.15468/dl.zfaw79
Anopheles funestus	36.696	-8.5202	Unclassified	GBIF: https://doi.org/10.15468/dl.zfaw79
Anopheles funestus	36.6962	-8.51988	Unclassified	GBIF: https://doi.org/10.15468/dl.zfaw79
Anopheles funestus	36.6968	-8.51633	Unclassified	GBIF: https://doi.org/10.15468/dl.zfaw79
Anopheles funestus	36.6969	-8.5165	Unclassified	GBIF: https://doi.org/10.15468/dl.zfaw79
Anopheles funestus	36.697	-8.52527	Unclassified	GBIF: https://doi.org/10.15468/dl.zfaw79
Anopheles funestus	36.6971	-8.52871	Unclassified	GBIF: https://doi.org/10.15468/dl.zfaw79
Anopheles funestus	36.6972	-8.50757	Unclassified	GBIF: https://doi.org/10.15468/dl.zfaw79
Anopheles funestus	36.6973	-8.53006	Unclassified	GBIF: https://doi.org/10.15468/dl.zfaw79
Anopheles funestus	36.6973	-8.52484	Unclassified	GBIF: https://doi.org/10.15468/dl.zfaw79
Anopheles funestus	36.6973	-8.52486	Unclassified	GBIF: https://doi.org/10.15468/dl.zfaw79
Anopheles funestus	36.6973	-8.52953	Unclassified	GBIF: https://doi.org/10.15468/dl.zfaw79
Anopheles funestus	36.6974	-8.53049	Unclassified	GBIF: https://doi.org/10.15468/dl.zfaw79
Anopheles funestus	36.6978	-8.5073	Unclassified	GBIF: https://doi.org/10.15468/dl.zfaw79
Anopheles funestus	36.698	-8.51669	Unclassified	GBIF: https://doi.org/10.15468/dl.zfaw79
Anopheles funestus	36.6981	-8.50765	Unclassified	GBIF: https://doi.org/10.15468/dl.zfaw79
Anopheles funestus	36.6984	-8.51681	Unclassified	GBIF: https://doi.org/10.15468/dl.zfaw79
Anopheles funestus	36.6984	-8.50681	Unclassified	GBIF: https://doi.org/10.15468/dl.zfaw79
Anopheles funestus	36.6987	-8.52804	Unclassified	GBIF: https://doi.org/10.15468/dl.zfaw79
Anopheles funestus	36.6998	-8.51657	Unclassified	GBIF: https://doi.org/10.15468/dl.zfaw79
Anopheles funestus	36.6998	-8.51674	Unclassified	GBIF: https://doi.org/10.15468/dl.zfaw79
Anopheles funestus	36.6999	-8.51649	Unclassified	GBIF: https://doi.org/10.15468/dl.zfaw79
Anopheles funestus	36.6999	-8.51629	Unclassified	GBIF: https://doi.org/10.15468/dl.zfaw79
Anopheles funestus	36.7	-8.51646	Unclassified	GBIF: https://doi.org/10.15468/dl.zfaw79
Anopheles funestus	36.7	-8.51642	Unclassified	GBIF: https://doi.org/10.15468/dl.zfaw79
Anopheles funestus	36.7001	-8.51633	Unclassified	GBIF: https://doi.org/10.15468/dl.zfaw79

Anopheles funestus	36.7003	-8.51647	Unclassified	GBIF: https://doi.org/10.15468/dl.zfaw79
Anopheles funestus	36.7005	-8.51308	Unclassified	GBIF: https://doi.org/10.15468/dl.zfaw79
Anopheles funestus	36.701	-8.5127	Unclassified	GBIF: https://doi.org/10.15468/dl.zfaw79
Anopheles funestus	36.7012	-8.53656	Unclassified	GBIF: https://doi.org/10.15468/dl.zfaw79
Anopheles funestus	36.7013	-8.51293	Unclassified	GBIF: https://doi.org/10.15468/dl.zfaw79
Anopheles funestus	36.7017	-8.53766	Unclassified	GBIF: https://doi.org/10.15468/dl.zfaw79
Anopheles funestus	36.7019	-8.53768	Unclassified	GBIF: https://doi.org/10.15468/dl.zfaw79
Anopheles funestus	36.7021	-8.5376	Unclassified	GBIF: https://doi.org/10.15468/dl.zfaw79
Anopheles funestus	36.7026	-8.53627	Unclassified	GBIF: https://doi.org/10.15468/dl.zfaw79
Anopheles funestus	36.7027	-8.5365	Unclassified	GBIF: https://doi.org/10.15468/dl.zfaw79
Anopheles funestus	36.7028	-8.53652	Unclassified	GBIF: https://doi.org/10.15468/dl.zfaw79
Anopheles funestus	36.7215	-8.61411	Unclassified	GBIF: https://doi.org/10.15468/dl.zfaw79
Anopheles funestus	36.7217	-8.61201	Unclassified	GBIF: https://doi.org/10.15468/dl.zfaw79
Anopheles funestus	36.7217	-8.61079	Unclassified	GBIF: https://doi.org/10.15468/dl.zfaw79
Anopheles funestus	36.7221	-8.61036	Unclassified	GBIF: https://doi.org/10.15468/dl.zfaw79
Anopheles funestus	36.7224	-8.60932	Unclassified	GBIF: https://doi.org/10.15468/dl.zfaw79
Anopheles funestus	36.7232	-8.60933	Unclassified	GBIF: https://doi.org/10.15468/dl.zfaw79
Anopheles funestus	36.7246	-8.60832	Unclassified	GBIF: https://doi.org/10.15468/dl.zfaw79
Anopheles funestus	36.7248	-8.60828	Unclassified	GBIF: https://doi.org/10.15468/dl.zfaw79
Anopheles funestus	36.7248	-8.60858	Unclassified	GBIF: https://doi.org/10.15468/dl.zfaw79
Anopheles funestus	36.7252	-8.60745	Unclassified	GBIF: https://doi.org/10.15468/dl.zfaw79
Anopheles funestus	36.7252	-8.60697	Unclassified	GBIF: https://doi.org/10.15468/dl.zfaw79
Anopheles funestus	36.7253	-8.86173	Unclassified	GBIF: https://doi.org/10.15468/dl.zfaw79
Anopheles funestus	36.7255	-8.60777	Unclassified	GBIF: https://doi.org/10.15468/dl.zfaw79
Anopheles funestus	36.7255	-8.60405	Unclassified	GBIF: https://doi.org/10.15468/dl.zfaw79
Anopheles funestus	36.7256	-8.60432	Unclassified	GBIF: https://doi.org/10.15468/dl.zfaw79
Anopheles funestus	36.7256	-8.86295	Unclassified	GBIF: https://doi.org/10.15468/dl.zfaw79
Anopheles funestus	36.7257	-8.60684	Unclassified	GBIF: https://doi.org/10.15468/dl.zfaw79
Anopheles funestus	36.726	-8.60634	Unclassified	GBIF: https://doi.org/10.15468/dl.zfaw79
Anopheles funestus	36.7262	-8.60262	Unclassified	GBIF: https://doi.org/10.15468/dl.zfaw79
Anopheles funestus	36.7263	-8.86336	Unclassified	GBIF: https://doi.org/10.15468/dl.zfaw79
Anopheles funestus	36.7264	-8.60774	Unclassified	GBIF: https://doi.org/10.15468/dl.zfaw79
Anopheles funestus	36.7269	-8.86636	Unclassified	GBIF: https://doi.org/10.15468/dl.zfaw79
Anopheles funestus	36.727	-8.86573	Unclassified	GBIF: https://doi.org/10.15468/dl.zfaw79
Anopheles funestus	36.7272	-8.86899	Unclassified	GBIF: https://doi.org/10.15468/dl.zfaw79
Anopheles funestus	36.7273	-8.87002	Unclassified	GBIF: https://doi.org/10.15468/dl.zfaw79
Anopheles funestus	36.7273	-8.86715	Unclassified	GBIF: https://doi.org/10.15468/dl.zfaw79
Anopheles funestus	36.7281	-8.87319	Unclassified	GBIF: https://doi.org/10.15468/dl.zfaw79
Anopheles funestus	36.7284	-8.87186	Unclassified	GBIF: https://doi.org/10.15468/dl.zfaw79
Anopheles funestus	36.7285	-8.60201	Unclassified	GBIF: https://doi.org/10.15468/dl.zfaw79
Anopheles funestus	36.7286	-8.87508	Unclassified	GBIF: https://doi.org/10.15468/dl.zfaw79
Anopheles funestus	36.7288	-8.87782	Unclassified	GBIF: https://doi.org/10.15468/dl.zfaw79

Anopheles funestus	36.7289	-8.87616	Unclassified	GBIF: https://doi.org/10.15468/dl.zfaw79
Anopheles funestus	36.729	-8.87831	Unclassified	GBIF: https://doi.org/10.15468/dl.zfaw79
Anopheles funestus	36.7291	-8.87648	Unclassified	GBIF: https://doi.org/10.15468/dl.zfaw79
Anopheles funestus	36.7294	-8.87979	Unclassified	GBIF: https://doi.org/10.15468/dl.zfaw79
Anopheles funestus	36.7297	-8.87908	Unclassified	GBIF: https://doi.org/10.15468/dl.zfaw79
Anopheles funestus	36.7298	-8.60247	Unclassified	GBIF: https://doi.org/10.15468/dl.zfaw79
Anopheles funestus	36.7299	-8.87975	Unclassified	GBIF: https://doi.org/10.15468/dl.zfaw79
Anopheles funestus	36.73	-8.88001	Unclassified	GBIF: https://doi.org/10.15468/dl.zfaw79
Anopheles funestus	36.73	-8.87801	Unclassified	GBIF: https://doi.org/10.15468/dl.zfaw79
Anopheles funestus	36.7301	-8.88224	Unclassified	GBIF: https://doi.org/10.15468/dl.zfaw79
Anopheles funestus	36.7301	-8.6021	Unclassified	GBIF: https://doi.org/10.15468/dl.zfaw79
Anopheles funestus	36.7306	-8.60129	Unclassified	GBIF: https://doi.org/10.15468/dl.zfaw79
Anopheles funestus	36.7308	-8.88076	Unclassified	GBIF: https://doi.org/10.15468/dl.zfaw79
Anopheles funestus	36.7308	-8.88333	Unclassified	GBIF: https://doi.org/10.15468/dl.zfaw79
Anopheles funestus	36.7308	-8.88222	Unclassified	GBIF: https://doi.org/10.15468/dl.zfaw79
Anopheles funestus	36.7313	-8.59897	Unclassified	GBIF: https://doi.org/10.15468/dl.zfaw79
Anopheles funestus	36.7316	-8.8882	Unclassified	GBIF: https://doi.org/10.15468/dl.zfaw79
Anopheles funestus	36.7316	-8.59583	Unclassified	GBIF: https://doi.org/10.15468/dl.zfaw79
Anopheles funestus	36.7318	-8.88838	Unclassified	GBIF: https://doi.org/10.15468/dl.zfaw79
Anopheles funestus	36.732	-8.59858	Unclassified	GBIF: https://doi.org/10.15468/dl.zfaw79
Anopheles funestus	36.7322	-8.60105	Unclassified	GBIF: https://doi.org/10.15468/dl.zfaw79
Anopheles funestus	36.7325	-8.59644	Unclassified	GBIF: https://doi.org/10.15468/dl.zfaw79
Anopheles funestus	36.7329	-8.89041	Unclassified	GBIF: https://doi.org/10.15468/dl.zfaw79
Anopheles funestus	36.7333	-8.89005	Unclassified	GBIF: https://doi.org/10.15468/dl.zfaw79
Anopheles funestus	36.7341	-8.89544	Unclassified	GBIF: https://doi.org/10.15468/dl.zfaw79
Anopheles funestus	36.7342	-8.89517	Unclassified	GBIF: https://doi.org/10.15468/dl.zfaw79
Anopheles funestus	36.7344	-8.8955	Unclassified	GBIF: https://doi.org/10.15468/dl.zfaw79
Anopheles funestus	36.7351	-8.89701	Unclassified	GBIF: https://doi.org/10.15468/dl.zfaw79
Anopheles funestus	36.7364	-8.89992	Unclassified	GBIF: https://doi.org/10.15468/dl.zfaw79
Anopheles funestus	36.7368	-8.90165	Unclassified	GBIF: https://doi.org/10.15468/dl.zfaw79
Anopheles funestus	36.7369	-8.90213	Unclassified	GBIF: https://doi.org/10.15468/dl.zfaw79
Anopheles funestus	36.7537	-8.97952	Unclassified	GBIF: https://doi.org/10.15468/dl.zfaw79
Anopheles funestus	36.7582	-8.94925	Unclassified	GBIF: https://doi.org/10.15468/dl.zfaw79
Anopheles funestus	36.7583	-8.95011	Unclassified	GBIF: https://doi.org/10.15468/dl.zfaw79
Anopheles funestus	36.7583	-8.97886	Unclassified	GBIF: https://doi.org/10.15468/dl.zfaw79
Anopheles funestus	36.7583	-8.95023	Unclassified	GBIF: https://doi.org/10.15468/dl.zfaw79
Anopheles funestus	36.7585	-8.9497	Unclassified	GBIF: https://doi.org/10.15468/dl.zfaw79
Anopheles funestus	36.7586	-8.94952	Unclassified	GBIF: https://doi.org/10.15468/dl.zfaw79
Anopheles funestus	36.7586	-8.94677	Unclassified	GBIF: https://doi.org/10.15468/dl.zfaw79
Anopheles funestus	36.7586	-8.94939	Unclassified	GBIF: https://doi.org/10.15468/dl.zfaw79
Anopheles funestus	36.7588	-8.94777	Unclassified	GBIF: https://doi.org/10.15468/dl.zfaw79
Anopheles funestus	36.7588	-8.94926	Unclassified	GBIF: https://doi.org/10.15468/dl.zfaw79

Anopheles funestus	36.7588	-8.94738	Unclassified	GBIF: https://doi.org/10.15468/dl.zfaw79
Anopheles funestus	36.7588	-8.98038	Unclassified	GBIF: https://doi.org/10.15468/dl.zfaw79
Anopheles funestus	36.7588	-8.9503	Unclassified	GBIF: https://doi.org/10.15468/dl.zfaw79
Anopheles funestus	36.7589	-8.94892	Unclassified	GBIF: https://doi.org/10.15468/dl.zfaw79
Anopheles funestus	36.7589	-8.94754	Unclassified	GBIF: https://doi.org/10.15468/dl.zfaw79
Anopheles funestus	36.759	-8.97857	Unclassified	GBIF: https://doi.org/10.15468/dl.zfaw79
Anopheles funestus	36.7592	-8.9797	Unclassified	GBIF: https://doi.org/10.15468/dl.zfaw79
Anopheles funestus	36.7593	-8.97919	Unclassified	GBIF: https://doi.org/10.15468/dl.zfaw79
Anopheles funestus	36.7594	-8.97905	Unclassified	GBIF: https://doi.org/10.15468/dl.zfaw79
Anopheles funestus	36.7595	-8.97895	Unclassified	GBIF: https://doi.org/10.15468/dl.zfaw79
Anopheles funestus	36.7596	-8.97864	Unclassified	GBIF: https://doi.org/10.15468/dl.zfaw79
Anopheles funestus	36.7596	-8.96189	Unclassified	GBIF: https://doi.org/10.15468/dl.zfaw79
Anopheles funestus	36.7596	-8.97968	Unclassified	GBIF: https://doi.org/10.15468/dl.zfaw79
Anopheles funestus	36.7598	-8.94694	Unclassified	GBIF: https://doi.org/10.15468/dl.zfaw79
Anopheles funestus	36.7598	-8.94554	Unclassified	GBIF: https://doi.org/10.15468/dl.zfaw79
Anopheles funestus	36.7598	-8.97867	Unclassified	GBIF: https://doi.org/10.15468/dl.zfaw79
Anopheles funestus	36.7599	-8.96194	Unclassified	GBIF: https://doi.org/10.15468/dl.zfaw79
Anopheles funestus	36.76	-8.97795	Unclassified	GBIF: https://doi.org/10.15468/dl.zfaw79
Anopheles funestus	36.76	-8.97787	Unclassified	GBIF: https://doi.org/10.15468/dl.zfaw79
Anopheles funestus	36.7601	-8.97771	Unclassified	GBIF: https://doi.org/10.15468/dl.zfaw79
Anopheles funestus	36.7602	-8.97755	Unclassified	GBIF: https://doi.org/10.15468/dl.zfaw79
Anopheles funestus	36.7603	-8.96996	Unclassified	GBIF: https://doi.org/10.15468/dl.zfaw79
Anopheles funestus	36.7603	-8.97055	Unclassified	GBIF: https://doi.org/10.15468/dl.zfaw79
Anopheles funestus	36.7604	-8.95647	Unclassified	GBIF: https://doi.org/10.15468/dl.zfaw79
Anopheles funestus	36.7605	-8.97616	Unclassified	GBIF: https://doi.org/10.15468/dl.zfaw79
Anopheles funestus	36.7605	-8.96944	Unclassified	GBIF: https://doi.org/10.15468/dl.zfaw79
Anopheles funestus	36.7606	-8.96154	Unclassified	GBIF: https://doi.org/10.15468/dl.zfaw79
Anopheles funestus	36.7606	-8.971	Unclassified	GBIF: https://doi.org/10.15468/dl.zfaw79
Anopheles funestus	36.7606	-8.97091	Unclassified	GBIF: https://doi.org/10.15468/dl.zfaw79
Anopheles funestus	36.7606	-8.9614	Unclassified	GBIF: https://doi.org/10.15468/dl.zfaw79
Anopheles funestus	36.7606	-8.96226	Unclassified	GBIF: https://doi.org/10.15468/dl.zfaw79
Anopheles funestus	36.7606	-8.97067	Unclassified	GBIF: https://doi.org/10.15468/dl.zfaw79
Anopheles funestus	36.7607	-8.96221	Unclassified	GBIF: https://doi.org/10.15468/dl.zfaw79
Anopheles funestus	36.7607	-8.96222	Unclassified	GBIF: https://doi.org/10.15468/dl.zfaw79
Anopheles funestus	36.7608	-8.96845	Unclassified	GBIF: https://doi.org/10.15468/dl.zfaw79
Anopheles funestus	36.7608	-8.96141	Unclassified	GBIF: https://doi.org/10.15468/dl.zfaw79
Anopheles funestus	36.7609	-8.97112	Unclassified	GBIF: https://doi.org/10.15468/dl.zfaw79
Anopheles funestus	36.7609	-8.96313	Unclassified	GBIF: https://doi.org/10.15468/dl.zfaw79
Anopheles funestus	36.7609	-8.96219	Unclassified	GBIF: https://doi.org/10.15468/dl.zfaw79
Anopheles funestus	36.7609	-8.97061	Unclassified	GBIF: https://doi.org/10.15468/dl.zfaw79
Anopheles funestus	36.7609	-8.97426	Unclassified	GBIF: https://doi.org/10.15468/dl.zfaw79
Anopheles funestus	36.7609	-8.95698	Unclassified	GBIF: https://doi.org/10.15468/dl.zfaw79

Anopheles funestus	36.761	-8.96127	Unclassified	GBIF: https://doi.org/10.15468/dl.zfaw79
Anopheles funestus	36.761	-8.96253	Unclassified	GBIF: https://doi.org/10.15468/dl.zfaw79
Anopheles funestus	36.7611	-8.9629	Unclassified	GBIF: https://doi.org/10.15468/dl.zfaw79
Anopheles funestus	36.7611	-8.96687	Unclassified	GBIF: https://doi.org/10.15468/dl.zfaw79
Anopheles funestus	36.7611	-8.97405	Unclassified	GBIF: https://doi.org/10.15468/dl.zfaw79
Anopheles funestus	36.7617	-8.9413	Unclassified	GBIF: https://doi.org/10.15468/dl.zfaw79
Anopheles funestus	36.762	-8.94141	Unclassified	GBIF: https://doi.org/10.15468/dl.zfaw79
Anopheles funestus	36.7623	-8.94154	Unclassified	GBIF: https://doi.org/10.15468/dl.zfaw79
Anopheles funestus	36.7624	-8.9427	Unclassified	GBIF: https://doi.org/10.15468/dl.zfaw79
Anopheles funestus	36.7625	-8.94399	Unclassified	GBIF: https://doi.org/10.15468/dl.zfaw79
Anopheles funestus	36.7626	-8.94097	Unclassified	GBIF: https://doi.org/10.15468/dl.zfaw79
Anopheles funestus	36.7626	-8.94084	Unclassified	GBIF: https://doi.org/10.15468/dl.zfaw79
Anopheles funestus	36.7626	-8.94384	Unclassified	GBIF: https://doi.org/10.15468/dl.zfaw79
Anopheles funestus	36.7629	-8.94396	Unclassified	GBIF: https://doi.org/10.15468/dl.zfaw79
Anopheles funestus	36.7629	-8.94436	Unclassified	GBIF: https://doi.org/10.15468/dl.zfaw79
Anopheles funestus	36.7629	-8.94523	Unclassified	GBIF: https://doi.org/10.15468/dl.zfaw79
Anopheles funestus	36.763	-8.94315	Unclassified	GBIF: https://doi.org/10.15468/dl.zfaw79
Anopheles funestus	36.763	-8.93949	Unclassified	GBIF: https://doi.org/10.15468/dl.zfaw79
Anopheles funestus	36.7631	-8.93964	Unclassified	GBIF: https://doi.org/10.15468/dl.zfaw79
Anopheles funestus	36.7633	-8.94438	Unclassified	GBIF: https://doi.org/10.15468/dl.zfaw79
Anopheles funestus	36.7633	-8.94359	Unclassified	GBIF: https://doi.org/10.15468/dl.zfaw79
Anopheles funestus	36.7633	-8.93885	Unclassified	GBIF: https://doi.org/10.15468/dl.zfaw79
Anopheles funestus	36.7633	-8.94135	Unclassified	GBIF: https://doi.org/10.15468/dl.zfaw79
Anopheles funestus	36.7634	-8.94563	Unclassified	GBIF: https://doi.org/10.15468/dl.zfaw79
Anopheles funestus	36.7634	-8.93906	Unclassified	GBIF: https://doi.org/10.15468/dl.zfaw79
Anopheles funestus	36.7635	-8.94143	Unclassified	GBIF: https://doi.org/10.15468/dl.zfaw79
Anopheles funestus	36.7635	-8.93915	Unclassified	GBIF: https://doi.org/10.15468/dl.zfaw79
Anopheles funestus	36.7635	-8.93905	Unclassified	GBIF: https://doi.org/10.15468/dl.zfaw79
Anopheles funestus	36.7635	-8.94458	Unclassified	GBIF: https://doi.org/10.15468/dl.zfaw79
Anopheles funestus	36.7636	-8.94478	Unclassified	GBIF: https://doi.org/10.15468/dl.zfaw79
Anopheles funestus	36.7639	-8.93876	Unclassified	GBIF: https://doi.org/10.15468/dl.zfaw79
Anopheles funestus	36.7639	-8.94364	Unclassified	GBIF: https://doi.org/10.15468/dl.zfaw79
Anopheles funestus	36.7642	-8.93836	Unclassified	GBIF: https://doi.org/10.15468/dl.zfaw79
Anopheles funestus	36.7645	-8.94355	Unclassified	GBIF: https://doi.org/10.15468/dl.zfaw79
Anopheles funestus	36.7821	-9.07734	Unclassified	GBIF: https://doi.org/10.15468/dl.zfaw79
Anopheles funestus	36.7826	-9.07687	Unclassified	GBIF: https://doi.org/10.15468/dl.zfaw79
Anopheles funestus	36.7827	-9.07739	Unclassified	GBIF: https://doi.org/10.15468/dl.zfaw79
Anopheles funestus	36.7829	-9.0767	Unclassified	GBIF: https://doi.org/10.15468/dl.zfaw79
Anopheles funestus	36.7831	-9.07527	Unclassified	GBIF: https://doi.org/10.15468/dl.zfaw79
Anopheles funestus	36.7832	-9.07705	Unclassified	GBIF: https://doi.org/10.15468/dl.zfaw79
Anopheles funestus	36.7834	-9.07646	Unclassified	GBIF: https://doi.org/10.15468/dl.zfaw79
Anopheles funestus	36.7834	-9.07759	Unclassified	GBIF: https://doi.org/10.15468/dl.zfaw79

Anopheles funestus	36.7835	-9.07558	Unclassified	GBIF: https://doi.org/10.15468/dl.zfaw79
Anopheles funestus	36.7838	-9.07466	Unclassified	GBIF: https://doi.org/10.15468/dl.zfaw79
Anopheles funestus	36.7847	-9.0738	Unclassified	GBIF: https://doi.org/10.15468/dl.zfaw79
Anopheles funestus	36.7848	-9.07434	Unclassified	GBIF: https://doi.org/10.15468/dl.zfaw79
Anopheles funestus	36.7849	-9.07358	Unclassified	GBIF: https://doi.org/10.15468/dl.zfaw79
Anopheles funestus	36.785	-8.92968	Unclassified	GBIF: https://doi.org/10.15468/dl.zfaw79
Anopheles funestus	36.7851	-9.07644	Unclassified	GBIF: https://doi.org/10.15468/dl.zfaw79
Anopheles funestus	36.7852	-9.0769	Unclassified	GBIF: https://doi.org/10.15468/dl.zfaw79
Anopheles funestus	36.7854	-9.07348	Unclassified	GBIF: https://doi.org/10.15468/dl.zfaw79
Anopheles funestus	36.7856	-9.07356	Unclassified	GBIF: https://doi.org/10.15468/dl.zfaw79
Anopheles funestus	36.7859	-9.07388	Unclassified	GBIF: https://doi.org/10.15468/dl.zfaw79
Anopheles funestus	36.7859	-9.0737	Unclassified	GBIF: https://doi.org/10.15468/dl.zfaw79
Anopheles funestus	36.7859	-9.07451	Unclassified	GBIF: https://doi.org/10.15468/dl.zfaw79
Anopheles funestus	36.7859	-9.07729	Unclassified	GBIF: https://doi.org/10.15468/dl.zfaw79
Anopheles funestus	36.786	-9.07408	Unclassified	GBIF: https://doi.org/10.15468/dl.zfaw79
Anopheles funestus	36.7866	-9.07505	Unclassified	GBIF: https://doi.org/10.15468/dl.zfaw79
Anopheles funestus	36.787	-9.07426	Unclassified	GBIF: https://doi.org/10.15468/dl.zfaw79
Anopheles funestus	36.7882	-9.07257	Unclassified	GBIF: https://doi.org/10.15468/dl.zfaw79
Anopheles funestus	36.7885	-9.07051	Unclassified	GBIF: https://doi.org/10.15468/dl.zfaw79
Anopheles funestus	36.7887	-9.07042	Unclassified	GBIF: https://doi.org/10.15468/dl.zfaw79
Anopheles funestus	36.789	-9.07032	Unclassified	GBIF: https://doi.org/10.15468/dl.zfaw79
Anopheles funestus	36.7929	-8.92785	Unclassified	GBIF: https://doi.org/10.15468/dl.zfaw79
Anopheles funestus	36.7929	-8.92721	Unclassified	GBIF: https://doi.org/10.15468/dl.zfaw79
Anopheles funestus	36.7932	-8.92722	Unclassified	GBIF: https://doi.org/10.15468/dl.zfaw79
Anopheles funestus	36.7932	-8.92747	Unclassified	GBIF: https://doi.org/10.15468/dl.zfaw79
Anopheles funestus	36.7933	-9.06546	Unclassified	GBIF: https://doi.org/10.15468/dl.zfaw79
Anopheles funestus	36.7934	-8.92783	Unclassified	GBIF: https://doi.org/10.15468/dl.zfaw79
Anopheles funestus	36.7934	-8.92707	Unclassified	GBIF: https://doi.org/10.15468/dl.zfaw79
Anopheles funestus	36.7935	-8.92746	Unclassified	GBIF: https://doi.org/10.15468/dl.zfaw79
Anopheles funestus	36.7936	-9.06553	Unclassified	GBIF: https://doi.org/10.15468/dl.zfaw79
Anopheles funestus	36.7936	-8.92743	Unclassified	GBIF: https://doi.org/10.15468/dl.zfaw79
Anopheles funestus	36.7937	-8.92736	Unclassified	GBIF: https://doi.org/10.15468/dl.zfaw79
Anopheles funestus	36.7937	-9.06571	Unclassified	GBIF: https://doi.org/10.15468/dl.zfaw79
Anopheles funestus	36.7938	-8.85932	Unclassified	GBIF: https://doi.org/10.15468/dl.zfaw79
Anopheles funestus	36.7939	-8.86088	Unclassified	GBIF: https://doi.org/10.15468/dl.zfaw79
Anopheles funestus	36.7939	-8.92751	Unclassified	GBIF: https://doi.org/10.15468/dl.zfaw79
Anopheles funestus	36.7942	-8.92715	Unclassified	GBIF: https://doi.org/10.15468/dl.zfaw79
Anopheles funestus	36.7942	-8.8591	Unclassified	GBIF: https://doi.org/10.15468/dl.zfaw79
Anopheles funestus	36.7943	-8.92795	Unclassified	GBIF: https://doi.org/10.15468/dl.zfaw79
Anopheles funestus	36.7944	-8.86067	Unclassified	GBIF: https://doi.org/10.15468/dl.zfaw79
Anopheles funestus	36.7944	-8.86049	Unclassified	GBIF: https://doi.org/10.15468/dl.zfaw79
Anopheles funestus	36.7944	-8.86068	Unclassified	GBIF: https://doi.org/10.15468/dl.zfaw79

Anopheles funestus	36.7945	-8.86044	Unclassified	GBIF: https://doi.org/10.15468/dl.zfaw79
Anopheles funestus	36.7945	-8.86145	Unclassified	GBIF: https://doi.org/10.15468/dl.zfaw79
Anopheles funestus	36.7945	-8.85907	Unclassified	GBIF: https://doi.org/10.15468/dl.zfaw79
Anopheles funestus	36.7946	-8.86239	Unclassified	GBIF: https://doi.org/10.15468/dl.zfaw79
Anopheles funestus	36.7947	-8.86247	Unclassified	GBIF: https://doi.org/10.15468/dl.zfaw79
Anopheles funestus	36.7948	-8.85937	Unclassified	GBIF: https://doi.org/10.15468/dl.zfaw79
Anopheles funestus	36.7948	-8.86027	Unclassified	GBIF: https://doi.org/10.15468/dl.zfaw79
Anopheles funestus	36.7949	-8.8596	Unclassified	GBIF: https://doi.org/10.15468/dl.zfaw79
Anopheles funestus	36.795	-8.85911	Unclassified	GBIF: https://doi.org/10.15468/dl.zfaw79
Anopheles funestus	36.7952	-8.86009	Unclassified	GBIF: https://doi.org/10.15468/dl.zfaw79
Anopheles funestus	36.7952	-8.92645	Unclassified	GBIF: https://doi.org/10.15468/dl.zfaw79
Anopheles funestus	36.7952	-8.92633	Unclassified	GBIF: https://doi.org/10.15468/dl.zfaw79
Anopheles funestus	36.7952	-8.92602	Unclassified	GBIF: https://doi.org/10.15468/dl.zfaw79
Anopheles funestus	36.7952	-8.86047	Unclassified	GBIF: https://doi.org/10.15468/dl.zfaw79
Anopheles funestus	36.7953	-8.85946	Unclassified	GBIF: https://doi.org/10.15468/dl.zfaw79
Anopheles funestus	36.7955	-8.8606	Unclassified	GBIF: https://doi.org/10.15468/dl.zfaw79
Anopheles funestus	36.7955	-8.86062	Unclassified	GBIF: https://doi.org/10.15468/dl.zfaw79
Anopheles funestus	36.7956	-8.85999	Unclassified	GBIF: https://doi.org/10.15468/dl.zfaw79
Anopheles funestus	36.7956	-8.92642	Unclassified	GBIF: https://doi.org/10.15468/dl.zfaw79
Anopheles funestus	36.7957	-8.92671	Unclassified	GBIF: https://doi.org/10.15468/dl.zfaw79
Anopheles funestus	36.7957	-8.92614	Unclassified	GBIF: https://doi.org/10.15468/dl.zfaw79
Anopheles funestus	36.7957	-8.92884	Unclassified	GBIF: https://doi.org/10.15468/dl.zfaw79
Anopheles funestus	36.7959	-8.9263	Unclassified	GBIF: https://doi.org/10.15468/dl.zfaw79
Anopheles funestus	36.7959	-8.92469	Unclassified	GBIF: https://doi.org/10.15468/dl.zfaw79
Anopheles funestus	36.7959	-8.92805	Unclassified	GBIF: https://doi.org/10.15468/dl.zfaw79
Anopheles funestus	36.796	-8.92727	Unclassified	GBIF: https://doi.org/10.15468/dl.zfaw79
Anopheles funestus	36.796	-8.92653	Unclassified	GBIF: https://doi.org/10.15468/dl.zfaw79
Anopheles funestus	36.7961	-8.85901	Unclassified	GBIF: https://doi.org/10.15468/dl.zfaw79
Anopheles funestus	36.7962	-8.92703	Unclassified	GBIF: https://doi.org/10.15468/dl.zfaw79
Anopheles funestus	36.7964	-8.92563	Unclassified	GBIF: https://doi.org/10.15468/dl.zfaw79
Anopheles funestus	36.7966	-8.92609	Unclassified	GBIF: https://doi.org/10.15468/dl.zfaw79
Anopheles funestus	36.7971	-8.8577	Unclassified	GBIF: https://doi.org/10.15468/dl.zfaw79
Anopheles funestus	36.7978	-8.85704	Unclassified	GBIF: https://doi.org/10.15468/dl.zfaw79
Anopheles funestus	36.7979	-8.85906	Unclassified	GBIF: https://doi.org/10.15468/dl.zfaw79
Anopheles funestus	36.7979	-8.92526	Unclassified	GBIF: https://doi.org/10.15468/dl.zfaw79
Anopheles funestus	36.798	-8.92528	Unclassified	GBIF: https://doi.org/10.15468/dl.zfaw79
Anopheles funestus	36.7981	-8.85908	Unclassified	GBIF: https://doi.org/10.15468/dl.zfaw79
Anopheles funestus	36.7981	-8.92494	Unclassified	GBIF: https://doi.org/10.15468/dl.zfaw79
Anopheles funestus	36.7981	-8.85903	Unclassified	GBIF: https://doi.org/10.15468/dl.zfaw79
Anopheles funestus	36.7982	-8.85949	Unclassified	GBIF: https://doi.org/10.15468/dl.zfaw79
Anopheles funestus	36.7983	-8.92521	Unclassified	GBIF: https://doi.org/10.15468/dl.zfaw79
Anopheles funestus	36.7986	-8.92577	Unclassified	GBIF: https://doi.org/10.15468/dl.zfaw79

Anopheles funestus	36.799	-8.92581	Unclassified	GBIF: https://doi.org/10.15468/dl.zfaw79
Anopheles funestus	36.7991	-8.85892	Unclassified	GBIF: https://doi.org/10.15468/dl.zfaw79
Anopheles funestus	36.7993	-8.85772	Unclassified	GBIF: https://doi.org/10.15468/dl.zfaw79
Anopheles funestus	36.7999	-8.85777	Unclassified	GBIF: https://doi.org/10.15468/dl.zfaw79
Anopheles funestus	36.8005	-8.85862	Unclassified	GBIF: https://doi.org/10.15468/dl.zfaw79
Anopheles funestus	36.8009	-8.85853	Unclassified	GBIF: https://doi.org/10.15468/dl.zfaw79
Anopheles funestus	36.801	-8.8584	Unclassified	GBIF: https://doi.org/10.15468/dl.zfaw79
Anopheles funestus	36.8207	-8.94651	Unclassified	GBIF: https://doi.org/10.15468/dl.zfaw79
Anopheles funestus	36.8227	-8.94298	Unclassified	GBIF: https://doi.org/10.15468/dl.zfaw79
Anopheles funestus	36.8228	-8.94604	Unclassified	GBIF: https://doi.org/10.15468/dl.zfaw79
Anopheles funestus	36.8236	-8.94522	Unclassified	GBIF: https://doi.org/10.15468/dl.zfaw79
Anopheles funestus	36.8238	-8.94484	Unclassified	GBIF: https://doi.org/10.15468/dl.zfaw79
Anopheles funestus	36.8238	-8.9465	Unclassified	GBIF: https://doi.org/10.15468/dl.zfaw79
Anopheles funestus	36.8241	-8.94541	Unclassified	GBIF: https://doi.org/10.15468/dl.zfaw79
Anopheles funestus	36.8244	-8.94496	Unclassified	GBIF: https://doi.org/10.15468/dl.zfaw79
Anopheles funestus	36.8245	-8.94524	Unclassified	GBIF: https://doi.org/10.15468/dl.zfaw79
Anopheles funestus	36.8246	-8.9449	Unclassified	GBIF: https://doi.org/10.15468/dl.zfaw79
Anopheles funestus	36.8246	-8.94519	Unclassified	GBIF: https://doi.org/10.15468/dl.zfaw79
Anopheles funestus	36.8277	-8.94283	Unclassified	GBIF: https://doi.org/10.15468/dl.zfaw79
Anopheles funestus	36.828	-8.94255	Unclassified	GBIF: https://doi.org/10.15468/dl.zfaw79
Anopheles funestus	36.8282	-8.94079	Unclassified	GBIF: https://doi.org/10.15468/dl.zfaw79
Anopheles funestus	36.8283	-8.94098	Unclassified	GBIF: https://doi.org/10.15468/dl.zfaw79
Anopheles funestus	36.8284	-8.94036	Unclassified	GBIF: https://doi.org/10.15468/dl.zfaw79
Anopheles funestus	36.8285	-8.94045	Unclassified	GBIF: https://doi.org/10.15468/dl.zfaw79
Anopheles funestus	36.8297	-8.94003	Unclassified	GBIF: https://doi.org/10.15468/dl.zfaw79
Anopheles funestus	36.8298	-9.05593	Unclassified	GBIF: https://doi.org/10.15468/dl.zfaw79
Anopheles funestus	36.8299	-8.9398	Unclassified	GBIF: https://doi.org/10.15468/dl.zfaw79
Anopheles funestus	36.8299	-9.05602	Unclassified	GBIF: https://doi.org/10.15468/dl.zfaw79
Anopheles funestus	36.83	-8.97644	Unclassified	GBIF: https://doi.org/10.15468/dl.zfaw79
Anopheles funestus	36.8301	-9.05644	Unclassified	GBIF: https://doi.org/10.15468/dl.zfaw79
Anopheles funestus	36.8301	-8.97724	Unclassified	GBIF: https://doi.org/10.15468/dl.zfaw79
Anopheles funestus	36.8303	-8.97838	Unclassified	GBIF: https://doi.org/10.15468/dl.zfaw79
Anopheles funestus	36.8303	-9.05621	Unclassified	GBIF: https://doi.org/10.15468/dl.zfaw79
Anopheles funestus	36.8304	-8.97701	Unclassified	GBIF: https://doi.org/10.15468/dl.zfaw79
Anopheles funestus	36.8304	-8.97848	Unclassified	GBIF: https://doi.org/10.15468/dl.zfaw79
Anopheles funestus	36.8304	-8.97853	Unclassified	GBIF: https://doi.org/10.15468/dl.zfaw79
Anopheles funestus	36.8304	-8.97823	Unclassified	GBIF: https://doi.org/10.15468/dl.zfaw79
Anopheles funestus	36.8304	-8.97886	Unclassified	GBIF: https://doi.org/10.15468/dl.zfaw79
Anopheles funestus	36.8305	-8.98102	Unclassified	GBIF: https://doi.org/10.15468/dl.zfaw79
Anopheles funestus	36.8305	-8.93661	Unclassified	GBIF: https://doi.org/10.15468/dl.zfaw79
Anopheles funestus	36.8305	-8.977	Unclassified	GBIF: https://doi.org/10.15468/dl.zfaw79
Anopheles funestus	36.8305	-8.93707	Unclassified	GBIF: https://doi.org/10.15468/dl.zfaw79

Anopheles funestus	36.8306	-8.93868	Unclassified	GBIF: https://doi.org/10.15468/dl.zfaw79
Anopheles funestus	36.8307	-8.93798	Unclassified	GBIF: https://doi.org/10.15468/dl.zfaw79
Anopheles funestus	36.8309	-8.93835	Unclassified	GBIF: https://doi.org/10.15468/dl.zfaw79
Anopheles funestus	36.8309	-8.93771	Unclassified	GBIF: https://doi.org/10.15468/dl.zfaw79
Anopheles funestus	36.8311	-8.93834	Unclassified	GBIF: https://doi.org/10.15468/dl.zfaw79
Anopheles funestus	36.8312	-9.05636	Unclassified	GBIF: https://doi.org/10.15468/dl.zfaw79
Anopheles funestus	36.8312	-8.98139	Unclassified	GBIF: https://doi.org/10.15468/dl.zfaw79
Anopheles funestus	36.8312	-9.05634	Unclassified	GBIF: https://doi.org/10.15468/dl.zfaw79
Anopheles funestus	36.8314	-9.05547	Unclassified	GBIF: https://doi.org/10.15468/dl.zfaw79
Anopheles funestus	36.8315	-8.97386	Unclassified	GBIF: https://doi.org/10.15468/dl.zfaw79
Anopheles funestus	36.8315	-9.05545	Unclassified	GBIF: https://doi.org/10.15468/dl.zfaw79
Anopheles funestus	36.8315	-8.97399	Unclassified	GBIF: https://doi.org/10.15468/dl.zfaw79
Anopheles funestus	36.8318	-8.97471	Unclassified	GBIF: https://doi.org/10.15468/dl.zfaw79
Anopheles funestus	36.8318	-8.97923	Unclassified	GBIF: https://doi.org/10.15468/dl.zfaw79
Anopheles funestus	36.8319	-8.96922	Unclassified	GBIF: https://doi.org/10.15468/dl.zfaw79
Anopheles funestus	36.8319	-9.05633	Unclassified	GBIF: https://doi.org/10.15468/dl.zfaw79
Anopheles funestus	36.832	-8.97227	Unclassified	GBIF: https://doi.org/10.15468/dl.zfaw79
Anopheles funestus	36.832	-8.95112	Unclassified	GBIF: https://doi.org/10.15468/dl.zfaw79
Anopheles funestus	36.832	-8.94806	Unclassified	GBIF: https://doi.org/10.15468/dl.zfaw79
Anopheles funestus	36.8321	-9.05573	Unclassified	GBIF: https://doi.org/10.15468/dl.zfaw79
Anopheles funestus	36.8321	-8.94788	Unclassified	GBIF: https://doi.org/10.15468/dl.zfaw79
Anopheles funestus	36.8321	-8.97456	Unclassified	GBIF: https://doi.org/10.15468/dl.zfaw79
Anopheles funestus	36.8322	-9.05489	Unclassified	GBIF: https://doi.org/10.15468/dl.zfaw79
Anopheles funestus	36.8322	-8.94498	Unclassified	GBIF: https://doi.org/10.15468/dl.zfaw79
Anopheles funestus	36.8322	-8.94797	Unclassified	GBIF: https://doi.org/10.15468/dl.zfaw79
Anopheles funestus	36.8323	-9.05496	Unclassified	GBIF: https://doi.org/10.15468/dl.zfaw79
Anopheles funestus	36.8324	-8.97219	Unclassified	GBIF: https://doi.org/10.15468/dl.zfaw79
Anopheles funestus	36.8324	-9.05467	Unclassified	GBIF: https://doi.org/10.15468/dl.zfaw79
Anopheles funestus	36.8324	-8.94414	Unclassified	GBIF: https://doi.org/10.15468/dl.zfaw79
Anopheles funestus	36.8324	-8.94741	Unclassified	GBIF: https://doi.org/10.15468/dl.zfaw79
Anopheles funestus	36.8326	-8.96994	Unclassified	GBIF: https://doi.org/10.15468/dl.zfaw79
Anopheles funestus	36.8326	-8.97997	Unclassified	GBIF: https://doi.org/10.15468/dl.zfaw79
Anopheles funestus	36.8327	-8.9469	Unclassified	GBIF: https://doi.org/10.15468/dl.zfaw79
Anopheles funestus	36.8327	-9.05633	Unclassified	GBIF: https://doi.org/10.15468/dl.zfaw79
Anopheles funestus	36.8328	-8.94812	Unclassified	GBIF: https://doi.org/10.15468/dl.zfaw79
Anopheles funestus	36.8329	-9.05728	Unclassified	GBIF: https://doi.org/10.15468/dl.zfaw79
Anopheles funestus	36.833	-9.05507	Unclassified	GBIF: https://doi.org/10.15468/dl.zfaw79
Anopheles funestus	36.833	-9.05463	Unclassified	GBIF: https://doi.org/10.15468/dl.zfaw79
Anopheles funestus	36.833	-9.05706	Unclassified	GBIF: https://doi.org/10.15468/dl.zfaw79
Anopheles funestus	36.8331	-8.98039	Unclassified	GBIF: https://doi.org/10.15468/dl.zfaw79
Anopheles funestus	36.8331	-8.9477	Unclassified	GBIF: https://doi.org/10.15468/dl.zfaw79
Anopheles funestus	36.8331	-9.0569	Unclassified	GBIF: https://doi.org/10.15468/dl.zfaw79

Anopheles funestus	36.8331	-9.05568	Unclassified	GBIF: https://doi.org/10.15468/dl.zfaw79
Anopheles funestus	36.8332	-9.05468	Unclassified	GBIF: https://doi.org/10.15468/dl.zfaw79
Anopheles funestus	36.8332	-9.05526	Unclassified	GBIF: https://doi.org/10.15468/dl.zfaw79
Anopheles funestus	36.8332	-9.05433	Unclassified	GBIF: https://doi.org/10.15468/dl.zfaw79
Anopheles funestus	36.8332	-9.05529	Unclassified	GBIF: https://doi.org/10.15468/dl.zfaw79
Anopheles funestus	36.8332	-9.05689	Unclassified	GBIF: https://doi.org/10.15468/dl.zfaw79
Anopheles funestus	36.8333	-9.05465	Unclassified	GBIF: https://doi.org/10.15468/dl.zfaw79
Anopheles funestus	36.8333	-9.05435	Unclassified	GBIF: https://doi.org/10.15468/dl.zfaw79
Anopheles funestus	36.8333	-8.97923	Unclassified	GBIF: https://doi.org/10.15468/dl.zfaw79
Anopheles funestus	36.8333	-9.05458	Unclassified	GBIF: https://doi.org/10.15468/dl.zfaw79
Anopheles funestus	36.8333	-9.05462	Unclassified	GBIF: https://doi.org/10.15468/dl.zfaw79
Anopheles funestus	36.8334	-9.05579	Unclassified	GBIF: https://doi.org/10.15468/dl.zfaw79
Anopheles funestus	36.8334	-9.05633	Unclassified	GBIF: https://doi.org/10.15468/dl.zfaw79
Anopheles funestus	36.8335	-9.05468	Unclassified	GBIF: https://doi.org/10.15468/dl.zfaw79
Anopheles funestus	36.8337	-9.05624	Unclassified	GBIF: https://doi.org/10.15468/dl.zfaw79
Anopheles funestus	36.8343	-8.9694	Unclassified	GBIF: https://doi.org/10.15468/dl.zfaw79
Anopheles funestus	36.8343	-9.05317	Unclassified	GBIF: https://doi.org/10.15468/dl.zfaw79
Anopheles funestus	36.8345	-8.97002	Unclassified	GBIF: https://doi.org/10.15468/dl.zfaw79
Anopheles funestus	36.8391	-9.00898	Unclassified	GBIF: https://doi.org/10.15468/dl.zfaw79
Anopheles funestus	36.8395	-9.00987	Unclassified	GBIF: https://doi.org/10.15468/dl.zfaw79
Anopheles funestus	36.8403	-9.00864	Unclassified	GBIF: https://doi.org/10.15468/dl.zfaw79
Anopheles funestus	36.8403	-9.0091	Unclassified	GBIF: https://doi.org/10.15468/dl.zfaw79
Anopheles funestus	36.8404	-9.0097	Unclassified	GBIF: https://doi.org/10.15468/dl.zfaw79
Anopheles funestus	36.8408	-9.01154	Unclassified	GBIF: https://doi.org/10.15468/dl.zfaw79
Anopheles funestus	36.8409	-9.00861	Unclassified	GBIF: https://doi.org/10.15468/dl.zfaw79
Anopheles funestus	36.8409	-9.01049	Unclassified	GBIF: https://doi.org/10.15468/dl.zfaw79
Anopheles funestus	36.8411	-9.01087	Unclassified	GBIF: https://doi.org/10.15468/dl.zfaw79
Anopheles funestus	36.8415	-9.01008	Unclassified	GBIF: https://doi.org/10.15468/dl.zfaw79
Anopheles funestus	36.8415	-9.00913	Unclassified	GBIF: https://doi.org/10.15468/dl.zfaw79
Anopheles funestus	36.8417	-9.00938	Unclassified	GBIF: https://doi.org/10.15468/dl.zfaw79
Anopheles funestus	36.8418	-9.01017	Unclassified	GBIF: https://doi.org/10.15468/dl.zfaw79
Anopheles funestus	36.842	-9.01096	Unclassified	GBIF: https://doi.org/10.15468/dl.zfaw79
Anopheles funestus	36.8421	-9.01353	Unclassified	GBIF: https://doi.org/10.15468/dl.zfaw79
Anopheles funestus	36.8424	-9.0113	Unclassified	GBIF: https://doi.org/10.15468/dl.zfaw79
Anopheles funestus	36.8424	-9.01108	Unclassified	GBIF: https://doi.org/10.15468/dl.zfaw79
Anopheles funestus	36.8425	-9.01315	Unclassified	GBIF: https://doi.org/10.15468/dl.zfaw79
Anopheles funestus	36.8425	-9.0116	Unclassified	GBIF: https://doi.org/10.15468/dl.zfaw79
Anopheles funestus	36.8426	-9.01142	Unclassified	GBIF: https://doi.org/10.15468/dl.zfaw79
Anopheles funestus	36.8427	-9.01066	Unclassified	GBIF: https://doi.org/10.15468/dl.zfaw79
Anopheles funestus	36.8428	-9.01075	Unclassified	GBIF: https://doi.org/10.15468/dl.zfaw79
Anopheles funestus	36.843	-9.01133	Unclassified	GBIF: https://doi.org/10.15468/dl.zfaw79
Anopheles funestus	36.843	-9.01287	Unclassified	GBIF: https://doi.org/10.15468/dl.zfaw79

Anopheles funestus	36.8431	-9.01073	Unclassified	GBIF: https://doi.org/10.15468/dl.zfaw79
Anopheles funestus	36.8432	-9.011	Unclassified	GBIF: https://doi.org/10.15468/dl.zfaw79
Anopheles funestus	36.8432	-9.01347	Unclassified	GBIF: https://doi.org/10.15468/dl.zfaw79
Anopheles funestus	36.8432	-9.01382	Unclassified	GBIF: https://doi.org/10.15468/dl.zfaw79
Anopheles funestus	36.8432	-9.01217	Unclassified	GBIF: https://doi.org/10.15468/dl.zfaw79
Anopheles funestus	36.8433	-9.01365	Unclassified	GBIF: https://doi.org/10.15468/dl.zfaw79
Anopheles funestus	36.8433	-9.01003	Unclassified	GBIF: https://doi.org/10.15468/dl.zfaw79
Anopheles funestus	36.8433	-9.00995	Unclassified	GBIF: https://doi.org/10.15468/dl.zfaw79
Anopheles funestus	36.8434	-9.01277	Unclassified	GBIF: https://doi.org/10.15468/dl.zfaw79
Anopheles funestus	36.8434	-9.01093	Unclassified	GBIF: https://doi.org/10.15468/dl.zfaw79
Anopheles funestus	36.8435	-9.01364	Unclassified	GBIF: https://doi.org/10.15468/dl.zfaw79
Anopheles funestus	36.8436	-9.01295	Unclassified	GBIF: https://doi.org/10.15468/dl.zfaw79
Anopheles funestus	36.8438	-9.01186	Unclassified	GBIF: https://doi.org/10.15468/dl.zfaw79
Anopheles funestus	36.8438	-9.01369	Unclassified	GBIF: https://doi.org/10.15468/dl.zfaw79
Anopheles funestus	36.8438	-9.01185	Unclassified	GBIF: https://doi.org/10.15468/dl.zfaw79
Anopheles funestus	36.8439	-9.01133	Unclassified	GBIF: https://doi.org/10.15468/dl.zfaw79
Anopheles funestus	36.8439	-9.01086	Unclassified	GBIF: https://doi.org/10.15468/dl.zfaw79
Anopheles funestus	36.844	-9.01039	Unclassified	GBIF: https://doi.org/10.15468/dl.zfaw79
Anopheles funestus	36.844	-9.01083	Unclassified	GBIF: https://doi.org/10.15468/dl.zfaw79
Anopheles funestus	36.8441	-9.01097	Unclassified	GBIF: https://doi.org/10.15468/dl.zfaw79
Anopheles funestus	36.8442	-9.01051	Unclassified	GBIF: https://doi.org/10.15468/dl.zfaw79
Anopheles funestus	36.8442	-9.01055	Unclassified	GBIF: https://doi.org/10.15468/dl.zfaw79
Anopheles funestus	36.8443	-9.01071	Unclassified	GBIF: https://doi.org/10.15468/dl.zfaw79
Anopheles funestus	36.8445	-9.00996	Unclassified	GBIF: https://doi.org/10.15468/dl.zfaw79
Anopheles funestus	36.8445	-9.00941	Unclassified	GBIF: https://doi.org/10.15468/dl.zfaw79
Anopheles funestus	36.8446	-9.00973	Unclassified	GBIF: https://doi.org/10.15468/dl.zfaw79
Anopheles funestus	36.8446	-9.00318	Unclassified	GBIF: https://doi.org/10.15468/dl.zfaw79
Anopheles funestus	36.8447	-9.01019	Unclassified	GBIF: https://doi.org/10.15468/dl.zfaw79
Anopheles funestus	36.8448	-9.00997	Unclassified	GBIF: https://doi.org/10.15468/dl.zfaw79
Anopheles funestus	36.8449	-9.00902	Unclassified	GBIF: https://doi.org/10.15468/dl.zfaw79
Anopheles funestus	36.8449	-9.01038	Unclassified	GBIF: https://doi.org/10.15468/dl.zfaw79
Anopheles funestus	36.845	-9.0096	Unclassified	GBIF: https://doi.org/10.15468/dl.zfaw79
Anopheles funestus	36.845	-9.00961	Unclassified	GBIF: https://doi.org/10.15468/dl.zfaw79
Anopheles funestus	36.8451	-9.00894	Unclassified	GBIF: https://doi.org/10.15468/dl.zfaw79
Anopheles funestus	36.8452	-9.00895	Unclassified	GBIF: https://doi.org/10.15468/dl.zfaw79
Anopheles funestus	36.8454	-9.00923	Unclassified	GBIF: https://doi.org/10.15468/dl.zfaw79
Anopheles funestus	36.8454	-9.00909	Unclassified	GBIF: https://doi.org/10.15468/dl.zfaw79
Anopheles funestus	36.8456	-9.00863	Unclassified	GBIF: https://doi.org/10.15468/dl.zfaw79
Anopheles funestus	36.8461	-9.00832	Unclassified	GBIF: https://doi.org/10.15468/dl.zfaw79
Anopheles funestus	37.4301	5.51846	Unclassified	GBIF: https://doi.org/10.15468/dl.zfaw79
Anopheles funestus	37.4303	5.5157	Unclassified	GBIF: https://doi.org/10.15468/dl.zfaw79
Anopheles funestus	37.4303	5.51721	Unclassified	GBIF: https://doi.org/10.15468/dl.zfaw79

Anopheles funestus	37.4304	5.51648	Unclassified	GBIF: https://doi.org/10.15468/dl.zfaw79
Anopheles funestus	37.4305	5.5176	Unclassified	GBIF: https://doi.org/10.15468/dl.zfaw79
Anopheles funestus	37.4309	5.51644	Unclassified	GBIF: https://doi.org/10.15468/dl.zfaw79
Anopheles funestus	37.4312	5.51697	Unclassified	GBIF: https://doi.org/10.15468/dl.zfaw79
Anopheles funestus	37.4312	5.51601	Unclassified	GBIF: https://doi.org/10.15468/dl.zfaw79
Anopheles funestus	37.4313	5.51762	Unclassified	GBIF: https://doi.org/10.15468/dl.zfaw79
Anopheles funestus	37.4313	5.51647	Unclassified	GBIF: https://doi.org/10.15468/dl.zfaw79
Anopheles funestus	37.4313	5.51626	Unclassified	GBIF: https://doi.org/10.15468/dl.zfaw79
Anopheles funestus	37.4313	5.51629	Unclassified	GBIF: https://doi.org/10.15468/dl.zfaw79
Anopheles funestus	37.4316	5.51642	Unclassified	GBIF: https://doi.org/10.15468/dl.zfaw79
Anopheles funestus	37.4317	5.51757	Unclassified	GBIF: https://doi.org/10.15468/dl.zfaw79
Anopheles funestus	37.4318	5.51684	Unclassified	GBIF: https://doi.org/10.15468/dl.zfaw79
Anopheles funestus	37.432	5.51643	Unclassified	GBIF: https://doi.org/10.15468/dl.zfaw79
Anopheles funestus	37.432	5.51729	Unclassified	GBIF: https://doi.org/10.15468/dl.zfaw79
Anopheles funestus	37.4321	5.51708	Unclassified	GBIF: https://doi.org/10.15468/dl.zfaw79
Anopheles funestus	37.4322	5.51498	Unclassified	GBIF: https://doi.org/10.15468/dl.zfaw79
Anopheles funestus	37.4323	5.51624	Unclassified	GBIF: https://doi.org/10.15468/dl.zfaw79
Anopheles funestus	37.4324	5.51647	Unclassified	GBIF: https://doi.org/10.15468/dl.zfaw79
Anopheles funestus	37.4326	5.51801	Unclassified	GBIF: https://doi.org/10.15468/dl.zfaw79
Anopheles funestus	37.4326	5.51766	Unclassified	GBIF: https://doi.org/10.15468/dl.zfaw79
Anopheles funestus	37.4326	5.51786	Unclassified	GBIF: https://doi.org/10.15468/dl.zfaw79
Anopheles funestus	37.4327	5.5178	Unclassified	GBIF: https://doi.org/10.15468/dl.zfaw79
Anopheles funestus	37.4327	5.5183	Unclassified	GBIF: https://doi.org/10.15468/dl.zfaw79
Anopheles funestus	37.4328	5.51531	Unclassified	GBIF: https://doi.org/10.15468/dl.zfaw79
Anopheles funestus	37.4328	5.51681	Unclassified	GBIF: https://doi.org/10.15468/dl.zfaw79
Anopheles funestus	37.4329	5.51614	Unclassified	GBIF: https://doi.org/10.15468/dl.zfaw79
Anopheles funestus	37.4331	5.51625	Unclassified	GBIF: https://doi.org/10.15468/dl.zfaw79
Anopheles funestus	37.4336	5.51701	Unclassified	GBIF: https://doi.org/10.15468/dl.zfaw79
Anopheles funestus	37.4336	5.51662	Unclassified	GBIF: https://doi.org/10.15468/dl.zfaw79
Anopheles funestus	37.4337	5.51759	Unclassified	GBIF: https://doi.org/10.15468/dl.zfaw79
Anopheles funestus	37.4337	5.51662	Unclassified	GBIF: https://doi.org/10.15468/dl.zfaw79
Anopheles funestus	37.4337	5.51614	Unclassified	GBIF: https://doi.org/10.15468/dl.zfaw79
Anopheles funestus	37.434	5.51612	Unclassified	GBIF: https://doi.org/10.15468/dl.zfaw79
Anopheles funestus	37.434	5.51763	Unclassified	GBIF: https://doi.org/10.15468/dl.zfaw79
Anopheles funestus	37.4342	5.51604	Unclassified	GBIF: https://doi.org/10.15468/dl.zfaw79
Anopheles funestus	37.4349	5.51631	Unclassified	GBIF: https://doi.org/10.15468/dl.zfaw79
Anopheles funestus	37.4351	5.51692	Unclassified	GBIF: https://doi.org/10.15468/dl.zfaw79
Anopheles funestus	37.4351	5.51556	Unclassified	GBIF: https://doi.org/10.15468/dl.zfaw79
Anopheles funestus	37.4352	5.51757	Unclassified	GBIF: https://doi.org/10.15468/dl.zfaw79
Anopheles funestus	37.4352	5.51648	Unclassified	GBIF: https://doi.org/10.15468/dl.zfaw79
Anopheles funestus	37.4353	5.5169	Unclassified	GBIF: https://doi.org/10.15468/dl.zfaw79
Anopheles funestus	37.4355	5.51566	Unclassified	GBIF: https://doi.org/10.15468/dl.zfaw79

Anopheles funestus	37.4356	5.5156	Unclassified	GBIF: https://doi.org/10.15468/dl.zfaw79
Anopheles funestus	37.4357	5.51643	Unclassified	GBIF: https://doi.org/10.15468/dl.zfaw79
Anopheles funestus	37.4359	5.51639	Unclassified	GBIF: https://doi.org/10.15468/dl.zfaw79
Anopheles funestus	37.4362	5.51563	Unclassified	GBIF: https://doi.org/10.15468/dl.zfaw79
Anopheles funestus	37.4366	5.51619	Unclassified	GBIF: https://doi.org/10.15468/dl.zfaw79
Anopheles funestus	37.4366	5.51635	Unclassified	GBIF: https://doi.org/10.15468/dl.zfaw79
Anopheles funestus	37.4367	5.51664	Unclassified	GBIF: https://doi.org/10.15468/dl.zfaw79
Anopheles funestus	37.4383	5.51589	Unclassified	GBIF: https://doi.org/10.15468/dl.zfaw79
Anopheles funestus	37.4385	5.51576	Unclassified	GBIF: https://doi.org/10.15468/dl.zfaw79
Anopheles funestus	37.4385	5.51542	Unclassified	GBIF: https://doi.org/10.15468/dl.zfaw79
Anopheles funestus	37.4386	5.51565	Unclassified	GBIF: https://doi.org/10.15468/dl.zfaw79
Anopheles funestus	37.4387	5.51507	Unclassified	GBIF: https://doi.org/10.15468/dl.zfaw79
Anopheles funestus	37.4387	5.51553	Unclassified	GBIF: https://doi.org/10.15468/dl.zfaw79
Anopheles funestus	37.4389	5.51578	Unclassified	GBIF: https://doi.org/10.15468/dl.zfaw79
Anopheles funestus	37.4391	5.5152	Unclassified	GBIF: https://doi.org/10.15468/dl.zfaw79
Anopheles funestus	37.4391	5.5157	Unclassified	GBIF: https://doi.org/10.15468/dl.zfaw79
Anopheles funestus	37.4393	5.5158	Unclassified	GBIF: https://doi.org/10.15468/dl.zfaw79
Anopheles funestus	37.4396	5.51509	Unclassified	GBIF: https://doi.org/10.15468/dl.zfaw79
Anopheles funestus	37.4398	5.51556	Unclassified	GBIF: https://doi.org/10.15468/dl.zfaw79
Anopheles funestus	37.4399	5.51493	Unclassified	GBIF: https://doi.org/10.15468/dl.zfaw79
Anopheles funestus	37.44	5.51503	Unclassified	GBIF: https://doi.org/10.15468/dl.zfaw79
Anopheles funestus	37.4415	5.51978	Unclassified	GBIF: https://doi.org/10.15468/dl.zfaw79
Anopheles funestus	37.4466	5.51414	Unclassified	GBIF: https://doi.org/10.15468/dl.zfaw79
Anopheles funestus	37.4539	5.51241	Unclassified	GBIF: https://doi.org/10.15468/dl.zfaw79
Anopheles funestus	37.4541	5.51111	Unclassified	GBIF: https://doi.org/10.15468/dl.zfaw79
Anopheles funestus	37.4542	5.5123	Unclassified	GBIF: https://doi.org/10.15468/dl.zfaw79
Anopheles funestus	37.4543	5.51165	Unclassified	GBIF: https://doi.org/10.15468/dl.zfaw79
Anopheles funestus	37.4543	5.51281	Unclassified	GBIF: https://doi.org/10.15468/dl.zfaw79
Anopheles funestus	37.4543	5.51241	Unclassified	GBIF: https://doi.org/10.15468/dl.zfaw79
Anopheles funestus	37.4543	5.51147	Unclassified	GBIF: https://doi.org/10.15468/dl.zfaw79
Anopheles funestus	37.4544	5.51196	Unclassified	GBIF: https://doi.org/10.15468/dl.zfaw79
Anopheles funestus	37.4544	5.51087	Unclassified	GBIF: https://doi.org/10.15468/dl.zfaw79
Anopheles funestus	37.4544	5.51197	Unclassified	GBIF: https://doi.org/10.15468/dl.zfaw79
Anopheles funestus	37.4545	5.51169	Unclassified	GBIF: https://doi.org/10.15468/dl.zfaw79
Anopheles funestus	37.4545	5.51276	Unclassified	GBIF: https://doi.org/10.15468/dl.zfaw79
Anopheles funestus	37.4545	5.51097	Unclassified	GBIF: https://doi.org/10.15468/dl.zfaw79
Anopheles funestus	37.4546	5.5105	Unclassified	GBIF: https://doi.org/10.15468/dl.zfaw79
Anopheles funestus	37.4547	5.5124	Unclassified	GBIF: https://doi.org/10.15468/dl.zfaw79
Anopheles funestus	37.4548	5.51278	Unclassified	GBIF: https://doi.org/10.15468/dl.zfaw79
Anopheles funestus	37.4549	5.51282	Unclassified	GBIF: https://doi.org/10.15468/dl.zfaw79
Anopheles funestus	37.455	5.51253	Unclassified	GBIF: https://doi.org/10.15468/dl.zfaw79
Anopheles funestus	37.4551	5.51196	Unclassified	GBIF: https://doi.org/10.15468/dl.zfaw79

Anopheles funestus	37.4551	5.51181	Unclassified	GBIF: https://doi.org/10.15468/dl.zfaw79
Anopheles funestus	37.4551	5.51166	Unclassified	GBIF: https://doi.org/10.15468/dl.zfaw79
Anopheles funestus	37.4552	5.51189	Unclassified	GBIF: https://doi.org/10.15468/dl.zfaw79
Anopheles funestus	37.4554	5.51143	Unclassified	GBIF: https://doi.org/10.15468/dl.zfaw79
Anopheles funestus	37.4554	5.51122	Unclassified	GBIF: https://doi.org/10.15468/dl.zfaw79
Anopheles funestus	37.4555	5.51172	Unclassified	GBIF: https://doi.org/10.15468/dl.zfaw79
Anopheles funestus	37.4556	5.51169	Unclassified	GBIF: https://doi.org/10.15468/dl.zfaw79
Anopheles funestus	37.4556	5.5123	Unclassified	GBIF: https://doi.org/10.15468/dl.zfaw79
Anopheles funestus	37.4557	5.51243	Unclassified	GBIF: https://doi.org/10.15468/dl.zfaw79
Anopheles funestus	37.4559	5.51215	Unclassified	GBIF: https://doi.org/10.15468/dl.zfaw79
Anopheles funestus	37.456	5.51251	Unclassified	GBIF: https://doi.org/10.15468/dl.zfaw79
Anopheles funestus	37.4561	5.51134	Unclassified	GBIF: https://doi.org/10.15468/dl.zfaw79
Anopheles funestus	37.4561	5.51232	Unclassified	GBIF: https://doi.org/10.15468/dl.zfaw79
Anopheles funestus	37.4562	5.51246	Unclassified	GBIF: https://doi.org/10.15468/dl.zfaw79
Anopheles funestus	37.4565	5.51175	Unclassified	GBIF: https://doi.org/10.15468/dl.zfaw79
Anopheles funestus	37.4565	5.51205	Unclassified	GBIF: https://doi.org/10.15468/dl.zfaw79
Anopheles funestus	37.4565	5.51191	Unclassified	GBIF: https://doi.org/10.15468/dl.zfaw79
Anopheles funestus	37.4566	5.51149	Unclassified	GBIF: https://doi.org/10.15468/dl.zfaw79
Anopheles funestus	37.4567	5.51185	Unclassified	GBIF: https://doi.org/10.15468/dl.zfaw79
Anopheles funestus	37.4569	5.51201	Unclassified	GBIF: https://doi.org/10.15468/dl.zfaw79
Anopheles funestus	37.457	5.5118	Unclassified	GBIF: https://doi.org/10.15468/dl.zfaw79
Anopheles funestus	37.457	5.51137	Unclassified	GBIF: https://doi.org/10.15468/dl.zfaw79
Anopheles funestus	37.4571	5.51201	Unclassified	GBIF: https://doi.org/10.15468/dl.zfaw79
Anopheles funestus	37.4571	5.51179	Unclassified	GBIF: https://doi.org/10.15468/dl.zfaw79
Anopheles funestus	37.4576	5.51188	Unclassified	GBIF: https://doi.org/10.15468/dl.zfaw79
Anopheles funestus	37.4576	5.51112	Unclassified	GBIF: https://doi.org/10.15468/dl.zfaw79
Anopheles funestus	37.4579	5.51158	Unclassified	GBIF: https://doi.org/10.15468/dl.zfaw79
Anopheles funestus	37.4581	5.51188	Unclassified	GBIF: https://doi.org/10.15468/dl.zfaw79
Anopheles funestus	37.4583	5.51134	Unclassified	GBIF: https://doi.org/10.15468/dl.zfaw79
Anopheles funestus	37.4607	5.51123	Unclassified	GBIF: https://doi.org/10.15468/dl.zfaw79
Anopheles funestus	37.6053	5.33419	Unclassified	GBIF: https://doi.org/10.15468/dl.zfaw79
Anopheles funestus	37.6053	5.33426	Unclassified	GBIF: https://doi.org/10.15468/dl.zfaw79
Anopheles funestus	37.6069	5.30044	Unclassified	GBIF: https://doi.org/10.15468/dl.zfaw79
Anopheles funestus	37.607	5.30164	Unclassified	GBIF: https://doi.org/10.15468/dl.zfaw79
Anopheles funestus	37.6102	5.30562	Unclassified	GBIF: https://doi.org/10.15468/dl.zfaw79
Anopheles funestus	37.6113	5.30453	Unclassified	GBIF: https://doi.org/10.15468/dl.zfaw79
Anopheles funestus	37.612	5.30552	Unclassified	GBIF: https://doi.org/10.15468/dl.zfaw79
Anopheles funestus	37.6121	5.3139	Unclassified	GBIF: https://doi.org/10.15468/dl.zfaw79
Anopheles funestus	37.6122	5.31304	Unclassified	GBIF: https://doi.org/10.15468/dl.zfaw79
Anopheles funestus	39.49	-4.41	Unclassified	GBIF: https://doi.org/10.15468/dl.zfaw79
Anopheles funestus	39.75	-3.61	Unclassified	GBIF: https://doi.org/10.15468/dl.zfaw79
Anopheles funestus	45.14743	-12.8206	Unclassified	GBIF: https://doi.org/10.15468/dl.zfaw79

Anopheles funestus	48.1667	-19.3667	Unclassified	GBIF: https://doi.org/10.15468/dl.zfaw79
Anopheles gabaldoni	-99.467222	17.360556	Unclassified	GBIF: https://doi.org/10.15468/dl.zfaw79
Anopheles gabaldoni	-93.192778	15.57	Unclassified	GBIF: https://doi.org/10.15468/dl.zfaw79
Anopheles gabaldoni	-92.952778	17.548056	Unclassified	GBIF: https://doi.org/10.15468/dl.zfaw79
Anopheles gabaldoni	-92.928055	17.990556	Unclassified	GBIF: https://doi.org/10.15468/dl.zfaw79
Anopheles gabaldoni	-92.92	17.592222	Unclassified	GBIF: https://doi.org/10.15468/dl.zfaw79
Anopheles gabaldoni	-92.749722	17.546389	Unclassified	GBIF: https://doi.org/10.15468/dl.zfaw79
Anopheles gabaldoni	-92.333889	17.555556	Unclassified	GBIF: https://doi.org/10.15468/dl.zfaw79
Anopheles gabaldoni	-92.284167	17.569444	Unclassified	GBIF: https://doi.org/10.15468/dl.zfaw79
Anopheles gabaldoni	-92.153333	17.459444	Unclassified	GBIF: https://doi.org/10.15468/dl.zfaw79
Anopheles gabaldoni	-91.426389	17.470833	Unclassified	GBIF: https://doi.org/10.15468/dl.zfaw79
Anopheles gabaldoni	-88.99998	16.21752	Unclassified	GBIF: https://doi.org/10.15468/dl.zfaw79
Anopheles gabonensis	11.66	-0.33	Unclassified	GBIF: https://doi.org/10.15468/dl.zfaw79
Anopheles gabonensis	11.70677	-0.31264	Unclassified	GBIF: https://doi.org/10.15468/dl.zfaw79
Anopheles gabonensis	11.71	-0.31	Unclassified	GBIF: https://doi.org/10.15468/dl.zfaw79
Anopheles gabonensis	12.96	-1.76	Unclassified	GBIF: https://doi.org/10.15468/dl.zfaw79
Anopheles galvaoi	-56.3783	-20.240601	Unclassified	GBIF: https://doi.org/10.15468/dl.zfaw79
Anopheles galvaoi	-54.35303	-24.86297	Unclassified	GBIF: https://doi.org/10.15468/dl.zfaw79
Anopheles galvaoi	-54.35	-24.86	Unclassified	GBIF: https://doi.org/10.15468/dl.zfaw79
Anopheles galvaoi	-54.25	-24.0667	Unclassified	GBIF: https://doi.org/10.15468/dl.zfaw79
Anopheles galvaoi	-52.2333	-23.55	Unclassified	GBIF: https://doi.org/10.15468/dl.zfaw79
Anopheles galvaoi	-48.39172	-22.13469	Unclassified	GBIF: https://doi.org/10.15468/dl.zfaw79
Anopheles galvaoi	-48.39108	-22.13531	Unclassified	GBIF: https://doi.org/10.15468/dl.zfaw79
Anopheles galvaoi	-48.39	-22.13	Unclassified	GBIF: https://doi.org/10.15468/dl.zfaw79
Anopheles galvaoi	-48.39	-22.14	Unclassified	GBIF: https://doi.org/10.15468/dl.zfaw79
Anopheles galvaoi	-48.13211	-24.514444	Unclassified	GBIF: https://doi.org/10.15468/dl.zfaw79
Anopheles galvaoi	-48.1321	-24.5144	Unclassified	GBIF: https://doi.org/10.15468/dl.zfaw79
Anopheles galvaoi	-43.292801	-22.1619	Unclassified	GBIF: https://doi.org/10.15468/dl.zfaw79
Anopheles galvaoi	-41.7869	-22.3708	Unclassified	GBIF: https://doi.org/10.15468/dl.zfaw79
Anopheles gambiae	-40.56705	-16.26713	Unclassified	GBIF: https://doi.org/10.15468/dl.zfaw79
Anopheles gambiae	-38.33369	-12.90045	Unclassified	GBIF: https://doi.org/10.15468/dl.zfaw79
Anopheles gambiae	-17.47	14.71	Unclassified	GBIF: https://doi.org/10.15468/dl.zfaw79
Anopheles gambiae	-17.45	14.69	Unclassified	GBIF: https://doi.org/10.15468/dl.zfaw79
Anopheles gambiae	-17.44	14.72	Unclassified	GBIF: https://doi.org/10.15468/dl.zfaw79
Anopheles gambiae	-17.283333	14.766667	Unclassified	GBIF: https://doi.org/10.15468/dl.zfaw79
Anopheles gambiae	-17.05	14.95	Unclassified	GBIF: https://doi.org/10.15468/dl.zfaw79
Anopheles gambiae	-17	14.58333	Unclassified	GBIF: https://doi.org/10.15468/dl.zfaw79
Anopheles gambiae	-16.86667	14.81667	Unclassified	GBIF: https://doi.org/10.15468/dl.zfaw79
Anopheles gambiae	-16.75	13.33333	Unclassified	GBIF: https://doi.org/10.15468/dl.zfaw79
Anopheles gambiae	-16.71667	13.26667	Unclassified	GBIF: https://doi.org/10.15468/dl.zfaw79
Anopheles gambiae	-16.66667	14.53333	Unclassified	GBIF: https://doi.org/10.15468/dl.zfaw79
Anopheles gambiae	-16.6	13.36667	Unclassified	GBIF: https://doi.org/10.15468/dl.zfaw79

Anopheles gambiae	-16.5928	13.2806	Unclassified	GBIF: https://doi.org/10.15468/dl.zfaw79
Anopheles gambiae	-16.5919	13.2256	Unclassified	GBIF: https://doi.org/10.15468/dl.zfaw79
Anopheles gambiae	-16.5761	13.1794	Unclassified	GBIF: https://doi.org/10.15468/dl.zfaw79
Anopheles gambiae	-16.5	16.01667	Unclassified	GBIF: https://doi.org/10.15468/dl.zfaw79
Anopheles gambiae	-16.38333	13.68333	Unclassified	GBIF: https://doi.org/10.15468/dl.zfaw79
Anopheles gambiae	-16.36667	12.8	Unclassified	GBIF: https://doi.org/10.15468/dl.zfaw79
Anopheles gambiae	-16.36667	13.25	Unclassified	GBIF: https://doi.org/10.15468/dl.zfaw79
Anopheles gambiae	-16.33333	12.41667	Unclassified	GBIF: https://doi.org/10.15468/dl.zfaw79
Anopheles gambiae	-16.33333	13.86667	Unclassified	GBIF: https://doi.org/10.15468/dl.zfaw79
Anopheles gambiae	-16.33333	12.66667	Unclassified	GBIF: https://doi.org/10.15468/dl.zfaw79
Anopheles gambiae	-16.28333	13.46667	Unclassified	GBIF: https://doi.org/10.15468/dl.zfaw79
Anopheles gambiae	-16.278611	16.199722	Unclassified	GBIF: https://doi.org/10.15468/dl.zfaw79
Anopheles gambiae	-16.25	14.26667	Unclassified	GBIF: https://doi.org/10.15468/dl.zfaw79
Anopheles gambiae	-16.0836	13.6183	Unclassified	GBIF: https://doi.org/10.15468/dl.zfaw79
Anopheles gambiae	-16.06667	14.16667	Unclassified	GBIF: https://doi.org/10.15468/dl.zfaw79
Anopheles gambiae	-16	13.33333	Unclassified	GBIF: https://doi.org/10.15468/dl.zfaw79
Anopheles gambiae	-15.91667	13.91667	Unclassified	GBIF: https://doi.org/10.15468/dl.zfaw79
Anopheles gambiae	-15.81667	11.93333	Unclassified	GBIF: https://doi.org/10.15468/dl.zfaw79
Anopheles gambiae	-15.81667	12.3	Unclassified	GBIF: https://doi.org/10.15468/dl.zfaw79
Anopheles gambiae	-15.8	13.36667	Unclassified	GBIF: https://doi.org/10.15468/dl.zfaw79
Anopheles gambiae	-15.78333	13.48333	Unclassified	GBIF: https://doi.org/10.15468/dl.zfaw79
Anopheles gambiae	-15.75	13.41667	Unclassified	GBIF: https://doi.org/10.15468/dl.zfaw79
Anopheles gambiae	-15.73333	11.23333	Unclassified	GBIF: https://doi.org/10.15468/dl.zfaw79
Anopheles gambiae	-15.71667	13.43333	Unclassified	GBIF: https://doi.org/10.15468/dl.zfaw79
Anopheles gambiae	-15.66667	12.93333	Unclassified	GBIF: https://doi.org/10.15468/dl.zfaw79
Anopheles gambiae	-15.65	11.86667	Unclassified	GBIF: https://doi.org/10.15468/dl.zfaw79
Anopheles gambiae	-15.6	16.476667	Unclassified	GBIF: https://doi.org/10.15468/dl.zfaw79
Anopheles gambiae	-15.58333	13.5	Unclassified	GBIF: https://doi.org/10.15468/dl.zfaw79
Anopheles gambiae	-15.56667	13.48333	Unclassified	GBIF: https://doi.org/10.15468/dl.zfaw79
Anopheles gambiae	-15.56667	13.56667	Unclassified	GBIF: https://doi.org/10.15468/dl.zfaw79
Anopheles gambiae	-15.53333	13.3	Unclassified	GBIF: https://doi.org/10.15468/dl.zfaw79
Anopheles gambiae	-15.53333	13.2	Unclassified	GBIF: https://doi.org/10.15468/dl.zfaw79
Anopheles gambiae	-15.5	12.73333	Unclassified	GBIF: https://doi.org/10.15468/dl.zfaw79
Anopheles gambiae	-15.5	13.5	Unclassified	GBIF: https://doi.org/10.15468/dl.zfaw79
Anopheles gambiae	-15.5	11.56667	Unclassified	GBIF: https://doi.org/10.15468/dl.zfaw79
Anopheles gambiae	-15.26667	11.2	Unclassified	GBIF: https://doi.org/10.15468/dl.zfaw79
Anopheles gambiae	-15.16667	11.21667	Unclassified	GBIF: https://doi.org/10.15468/dl.zfaw79
Anopheles gambiae	-15.03333	16.58333	Unclassified	GBIF: https://doi.org/10.15468/dl.zfaw79
Anopheles gambiae	-15	13.6	Unclassified	GBIF: https://doi.org/10.15468/dl.zfaw79
Anopheles gambiae	-15	11.9	Unclassified	GBIF: https://doi.org/10.15468/dl.zfaw79
Anopheles gambiae	-15	11.33333	Unclassified	GBIF: https://doi.org/10.15468/dl.zfaw79
Anopheles gambiae	-14.93333	13.58333	Unclassified	GBIF: https://doi.org/10.15468/dl.zfaw79

Anopheles gambiae	-14.91667	12.93333	Unclassified	GBIF: https://doi.org/10.15468/dl.zfaw79
Anopheles gambiae	-14.88333	15.28333	Unclassified	GBIF: https://doi.org/10.15468/dl.zfaw79
Anopheles gambiae	-14.85	13.48333	Unclassified	GBIF: https://doi.org/10.15468/dl.zfaw79
Anopheles gambiae	-14.83333	13.51667	Unclassified	GBIF: https://doi.org/10.15468/dl.zfaw79
Anopheles gambiae	-14.81667	13.13333	Unclassified	GBIF: https://doi.org/10.15468/dl.zfaw79
Anopheles gambiae	-14.76667	17.66667	Unclassified	GBIF: https://doi.org/10.15468/dl.zfaw79
Anopheles gambiae	-14.75	16.55	Unclassified	GBIF: https://doi.org/10.15468/dl.zfaw79
Anopheles gambiae	-14.75	12.25	Unclassified	GBIF: https://doi.org/10.15468/dl.zfaw79
Anopheles gambiae	-14.7	16.01667	Unclassified	GBIF: https://doi.org/10.15468/dl.zfaw79
Anopheles gambiae	-14.66667	13.43333	Unclassified	GBIF: https://doi.org/10.15468/dl.zfaw79
Anopheles gambiae	-14.66667	12.13333	Unclassified	GBIF: https://doi.org/10.15468/dl.zfaw79
Anopheles gambiae	-14.65	16.51667	Unclassified	GBIF: https://doi.org/10.15468/dl.zfaw79
Anopheles gambiae	-14.55	12.28333	Unclassified	GBIF: https://doi.org/10.15468/dl.zfaw79
Anopheles gambiae	-14.51667	16.51667	Unclassified	GBIF: https://doi.org/10.15468/dl.zfaw79
Anopheles gambiae	-14.48333	12.41667	Unclassified	GBIF: https://doi.org/10.15468/dl.zfaw79
Anopheles gambiae	-14.45	12.33333	Unclassified	GBIF: https://doi.org/10.15468/dl.zfaw79
Anopheles gambiae	-14.25	13.38333	Unclassified	GBIF: https://doi.org/10.15468/dl.zfaw79
Anopheles gambiae	-14.25	13.33333	Unclassified	GBIF: https://doi.org/10.15468/dl.zfaw79
Anopheles gambiae	-14.23333	16.08333	Unclassified	GBIF: https://doi.org/10.15468/dl.zfaw79
Anopheles gambiae	-14.18333	12.55	Unclassified	GBIF: https://doi.org/10.15468/dl.zfaw79
Anopheles gambiae	-13.98333	12.38333	Unclassified	GBIF: https://doi.org/10.15468/dl.zfaw79
Anopheles gambiae	-13.98333	11.71667	Unclassified	GBIF: https://doi.org/10.15468/dl.zfaw79
Anopheles gambiae	-13.96667	17.03333	Unclassified	GBIF: https://doi.org/10.15468/dl.zfaw79
Anopheles gambiae	-13.88333	11.8	Unclassified	GBIF: https://doi.org/10.15468/dl.zfaw79
Anopheles gambiae	-13.71667	9.5	Unclassified	GBIF: https://doi.org/10.15468/dl.zfaw79
Anopheles gambiae	-13.66667	15.65	Unclassified	GBIF: https://doi.org/10.15468/dl.zfaw79
Anopheles gambiae	-13.55	16.16667	Unclassified	GBIF: https://doi.org/10.15468/dl.zfaw79
Anopheles gambiae	-13.33333	13.35	Unclassified	GBIF: https://doi.org/10.15468/dl.zfaw79
Anopheles gambiae	-13.28333	8.46667	Unclassified	GBIF: https://doi.org/10.15468/dl.zfaw79
Anopheles gambiae	-13.28333	8.5	Unclassified	GBIF: https://doi.org/10.15468/dl.zfaw79
Anopheles gambiae	-13.13333	16.43333	Unclassified	GBIF: https://doi.org/10.15468/dl.zfaw79
Anopheles gambiae	-13.1	16.25	Unclassified	GBIF: https://doi.org/10.15468/dl.zfaw79
Anopheles gambiae	-12.83333	8.33333	Unclassified	GBIF: https://doi.org/10.15468/dl.zfaw79
Anopheles gambiae	-11.7	7.96667	Unclassified	GBIF: https://doi.org/10.15468/dl.zfaw79
Anopheles gambiae	-11.55	13.75	Unclassified	GBIF: https://doi.org/10.15468/dl.zfaw79
Anopheles gambiae	-11.46667	14.43333	Unclassified	GBIF: https://doi.org/10.15468/dl.zfaw79
Anopheles gambiae	-11.37	6.75	Unclassified	GBIF: https://doi.org/10.15468/dl.zfaw79
Anopheles gambiae	-11.36667	12.7	Unclassified	GBIF: https://doi.org/10.15468/dl.zfaw79
Anopheles gambiae	-10.91667	14.46667	Unclassified	GBIF: https://doi.org/10.15468/dl.zfaw79
Anopheles gambiae	-10.83333	13.81667	Unclassified	GBIF: https://doi.org/10.15468/dl.zfaw79
Anopheles gambiae	-10.77373	6.31093	Unclassified	GBIF: https://doi.org/10.15468/dl.zfaw79
Anopheles gambiae	-10.75	13.45	Unclassified	GBIF: https://doi.org/10.15468/dl.zfaw79

Anopheles gambiae	-10.73333	6.33333	Unclassified	GBIF: https://doi.org/10.15468/dl.zfaw79
Anopheles gambiae	-10.58333	15.06667	Unclassified	GBIF: https://doi.org/10.15468/dl.zfaw79
Anopheles gambiae	-10.5	6.5	Unclassified	GBIF: https://doi.org/10.15468/dl.zfaw79
Anopheles gambiae	-10.5	13.25	Unclassified	GBIF: https://doi.org/10.15468/dl.zfaw79
Anopheles gambiae	-10.45	13.16667	Unclassified	GBIF: https://doi.org/10.15468/dl.zfaw79
Anopheles gambiae	-10.4	13.05	Unclassified	GBIF: https://doi.org/10.15468/dl.zfaw79
Anopheles gambiae	-10.36667	12.91667	Unclassified	GBIF: https://doi.org/10.15468/dl.zfaw79
Anopheles gambiae	-10.353433	6.240148	Unclassified	GBIF: https://doi.org/10.15468/dl.zfaw79
Anopheles gambiae	-9.75	14.55	Unclassified	GBIF: https://doi.org/10.15468/dl.zfaw79
Anopheles gambiae	-9.5	12.91667	Unclassified	GBIF: https://doi.org/10.15468/dl.zfaw79
Anopheles gambiae	-9.320214	6.18407	Unclassified	GBIF: https://doi.org/10.15468/dl.zfaw79
Anopheles gambiae	-9.13333	15.06667	Unclassified	GBIF: https://doi.org/10.15468/dl.zfaw79
Anopheles gambiae	-9.08333	7.15	Unclassified	GBIF: https://doi.org/10.15468/dl.zfaw79
Anopheles gambiae	-8.33333	6.58333	Unclassified	GBIF: https://doi.org/10.15468/dl.zfaw79
Anopheles gambiae	-8.33333	13.36667	Unclassified	GBIF: https://doi.org/10.15468/dl.zfaw79
Anopheles gambiae	-8.31667	7.5	Unclassified	GBIF: https://doi.org/10.15468/dl.zfaw79
Anopheles gambiae	-8.31667	11.41667	Unclassified	GBIF: https://doi.org/10.15468/dl.zfaw79
Anopheles gambiae	-8.29	12.17	Unclassified	GBIF: https://doi.org/10.15468/dl.zfaw79
Anopheles gambiae	-8.28333	11.7	Unclassified	GBIF: https://doi.org/10.15468/dl.zfaw79
Anopheles gambiae	-8.26667	12.41667	Unclassified	GBIF: https://doi.org/10.15468/dl.zfaw79
Anopheles gambiae	-8.26667	12.8	Unclassified	GBIF: https://doi.org/10.15468/dl.zfaw79
Anopheles gambiae	-8.21667	12.5	Unclassified	GBIF: https://doi.org/10.15468/dl.zfaw79
Anopheles gambiae	-8.18333	11.66667	Unclassified	GBIF: https://doi.org/10.15468/dl.zfaw79
Anopheles gambiae	-8.16667	7.45	Unclassified	GBIF: https://doi.org/10.15468/dl.zfaw79
Anopheles gambiae	-8.08333	13.91667	Unclassified	GBIF: https://doi.org/10.15468/dl.zfaw79
Anopheles gambiae	-8.05	12.8	Unclassified	GBIF: https://doi.org/10.15468/dl.zfaw79
Anopheles gambiae	-8	12.46667	Unclassified	GBIF: https://doi.org/10.15468/dl.zfaw79
Anopheles gambiae	-7.98333	12.66667	Unclassified	GBIF: https://doi.org/10.15468/dl.zfaw79
Anopheles gambiae	-7.95	12.68333	Unclassified	GBIF: https://doi.org/10.15468/dl.zfaw79
Anopheles gambiae	-7.93333	12.03333	Unclassified	GBIF: https://doi.org/10.15468/dl.zfaw79
Anopheles gambiae	-7.91667	12.51667	Unclassified	GBIF: https://doi.org/10.15468/dl.zfaw79
Anopheles gambiae	-7.75	14.33333	Unclassified	GBIF: https://doi.org/10.15468/dl.zfaw79
Anopheles gambiae	-7.71667	11.78333	Unclassified	GBIF: https://doi.org/10.15468/dl.zfaw79
Anopheles gambiae	-7.63333	12.91667	Unclassified	GBIF: https://doi.org/10.15468/dl.zfaw79
Anopheles gambiae	-7.61667	7.51667	Unclassified	GBIF: https://doi.org/10.15468/dl.zfaw79
Anopheles gambiae	-7.55	11.36667	Unclassified	GBIF: https://doi.org/10.15468/dl.zfaw79
Anopheles gambiae	-7.53333	4.45	Unclassified	GBIF: https://doi.org/10.15468/dl.zfaw79
Anopheles gambiae	-7.51667	4.41667	Unclassified	GBIF: https://doi.org/10.15468/dl.zfaw79
Anopheles gambiae	-7.46667	8.6	Unclassified	GBIF: https://doi.org/10.15468/dl.zfaw79
Anopheles gambiae	-7.46667	11.41667	Unclassified	GBIF: https://doi.org/10.15468/dl.zfaw79
Anopheles gambiae	-7.46667	14.56667	Unclassified	GBIF: https://doi.org/10.15468/dl.zfaw79
Anopheles gambiae	-7.41667	14.58333	Unclassified	GBIF: https://doi.org/10.15468/dl.zfaw79

Anopheles gambiae	-7.33333	11.36667	Unclassified	GBIF: https://doi.org/10.15468/dl.zfaw79
Anopheles gambiae	-7.3	13.86667	Unclassified	GBIF: https://doi.org/10.15468/dl.zfaw79
Anopheles gambiae	-7.28333	13.86667	Unclassified	GBIF: https://doi.org/10.15468/dl.zfaw79
Anopheles gambiae	-7.21667	6.48333	Unclassified	GBIF: https://doi.org/10.15468/dl.zfaw79
Anopheles gambiae	-7.17	13.55	Unclassified	GBIF: https://doi.org/10.15468/dl.zfaw79
Anopheles gambiae	-7.1	13.85	Unclassified	GBIF: https://doi.org/10.15468/dl.zfaw79
Anopheles gambiae	-6.98333	10.86667	Unclassified	GBIF: https://doi.org/10.15468/dl.zfaw79
Anopheles gambiae	-6.96667	9.7	Unclassified	GBIF: https://doi.org/10.15468/dl.zfaw79
Anopheles gambiae	-6.81667	12.51667	Unclassified	GBIF: https://doi.org/10.15468/dl.zfaw79
Anopheles gambiae	-6.81667	12.56667	Unclassified	GBIF: https://doi.org/10.15468/dl.zfaw79
Anopheles gambiae	-6.8	6.4	Unclassified	GBIF: https://doi.org/10.15468/dl.zfaw79
Anopheles gambiae	-6.78333	12.43333	Unclassified	GBIF: https://doi.org/10.15468/dl.zfaw79
Anopheles gambiae	-6.76667	11.41667	Unclassified	GBIF: https://doi.org/10.15468/dl.zfaw79
Anopheles gambiae	-6.68333	12.33333	Unclassified	GBIF: https://doi.org/10.15468/dl.zfaw79
Anopheles gambiae	-6.63333	13.01667	Unclassified	GBIF: https://doi.org/10.15468/dl.zfaw79
Anopheles gambiae	-6.46667	6.93333	Unclassified	GBIF: https://doi.org/10.15468/dl.zfaw79
Anopheles gambiae	-6.45	9.23333	Unclassified	GBIF: https://doi.org/10.15468/dl.zfaw79
Anopheles gambiae	-6.45	13.3	Unclassified	GBIF: https://doi.org/10.15468/dl.zfaw79
Anopheles gambiae	-6.35	13.4	Unclassified	GBIF: https://doi.org/10.15468/dl.zfaw79
Anopheles gambiae	-6.3	13.46667	Unclassified	GBIF: https://doi.org/10.15468/dl.zfaw79
Anopheles gambiae	-6.21667	13.33333	Unclassified	GBIF: https://doi.org/10.15468/dl.zfaw79
Anopheles gambiae	-6.21667	5.38333	Unclassified	GBIF: https://doi.org/10.15468/dl.zfaw79
Anopheles gambiae	-6.18333	14.88333	Unclassified	GBIF: https://doi.org/10.15468/dl.zfaw79
Anopheles gambiae	-6.16667	10.75	Unclassified	GBIF: https://doi.org/10.15468/dl.zfaw79
Anopheles gambiae	-6.13333	4.96667	Unclassified	GBIF: https://doi.org/10.15468/dl.zfaw79
Anopheles gambiae	-6.08333	13.68333	Unclassified	GBIF: https://doi.org/10.15468/dl.zfaw79
Anopheles gambiae	-6.08333	13.7	Unclassified	GBIF: https://doi.org/10.15468/dl.zfaw79
Anopheles gambiae	-6.06667	13.63333	Unclassified	GBIF: https://doi.org/10.15468/dl.zfaw79
Anopheles gambiae	-6.03333	14.13333	Unclassified	GBIF: https://doi.org/10.15468/dl.zfaw79
Anopheles gambiae	-6.01667	14.73333	Unclassified	GBIF: https://doi.org/10.15468/dl.zfaw79
Anopheles gambiae	-6	14.78333	Unclassified	GBIF: https://doi.org/10.15468/dl.zfaw79
Anopheles gambiae	-5.96667	13.65	Unclassified	GBIF: https://doi.org/10.15468/dl.zfaw79
Anopheles gambiae	-5.95	10.91667	Unclassified	GBIF: https://doi.org/10.15468/dl.zfaw79
Anopheles gambiae	-5.91667	13.21667	Unclassified	GBIF: https://doi.org/10.15468/dl.zfaw79
Anopheles gambiae	-5.78333	12.8	Unclassified	GBIF: https://doi.org/10.15468/dl.zfaw79
Anopheles gambiae	-5.78022	9.167032	Unclassified	GBIF: https://doi.org/10.15468/dl.zfaw79
Anopheles gambiae	-5.780209	9.167037	Unclassified	GBIF: https://doi.org/10.15468/dl.zfaw79
Anopheles gambiae	-5.779912	9.167013	Unclassified	GBIF: https://doi.org/10.15468/dl.zfaw79
Anopheles gambiae	-5.779374	9.167517	Unclassified	GBIF: https://doi.org/10.15468/dl.zfaw79
Anopheles gambiae	-5.77936	9.167506	Unclassified	GBIF: https://doi.org/10.15468/dl.zfaw79
Anopheles gambiae	-5.779229	9.167393	Unclassified	GBIF: https://doi.org/10.15468/dl.zfaw79
Anopheles gambiae	-5.779149	9.167072	Unclassified	GBIF: https://doi.org/10.15468/dl.zfaw79

Anopheles gambiae	-5.77879	9.166932	Unclassified	GBIF: https://doi.org/10.15468/dl.zfaw79
Anopheles gambiae	-5.778734	9.166936	Unclassified	GBIF: https://doi.org/10.15468/dl.zfaw79
Anopheles gambiae	-5.778703	9.166935	Unclassified	GBIF: https://doi.org/10.15468/dl.zfaw79
Anopheles gambiae	-5.778683	9.166938	Unclassified	GBIF: https://doi.org/10.15468/dl.zfaw79
Anopheles gambiae	-5.778431	9.16805	Unclassified	GBIF: https://doi.org/10.15468/dl.zfaw79
Anopheles gambiae	-5.778425	9.168052	Unclassified	GBIF: https://doi.org/10.15468/dl.zfaw79
Anopheles gambiae	-5.778418	9.168054	Unclassified	GBIF: https://doi.org/10.15468/dl.zfaw79
Anopheles gambiae	-5.761173	9.255355	Unclassified	GBIF: https://doi.org/10.15468/dl.zfaw79
Anopheles gambiae	-5.761159	9.255373	Unclassified	GBIF: https://doi.org/10.15468/dl.zfaw79
Anopheles gambiae	-5.761154	9.255394	Unclassified	GBIF: https://doi.org/10.15468/dl.zfaw79
Anopheles gambiae	-5.761149	9.255368	Unclassified	GBIF: https://doi.org/10.15468/dl.zfaw79
Anopheles gambiae	-5.761138	9.255358	Unclassified	GBIF: https://doi.org/10.15468/dl.zfaw79
Anopheles gambiae	-5.76113	9.255374	Unclassified	GBIF: https://doi.org/10.15468/dl.zfaw79
Anopheles gambiae	-5.761128	9.255361	Unclassified	GBIF: https://doi.org/10.15468/dl.zfaw79
Anopheles gambiae	-5.761043	9.256652	Unclassified	GBIF: https://doi.org/10.15468/dl.zfaw79
Anopheles gambiae	-5.761025	9.256667	Unclassified	GBIF: https://doi.org/10.15468/dl.zfaw79
Anopheles gambiae	-5.761025	9.256636	Unclassified	GBIF: https://doi.org/10.15468/dl.zfaw79
Anopheles gambiae	-5.761023	9.256631	Unclassified	GBIF: https://doi.org/10.15468/dl.zfaw79
Anopheles gambiae	-5.761018	9.256659	Unclassified	GBIF: https://doi.org/10.15468/dl.zfaw79
Anopheles gambiae	-5.761	9.256662	Unclassified	GBIF: https://doi.org/10.15468/dl.zfaw79
Anopheles gambiae	-5.760115	9.255453	Unclassified	GBIF: https://doi.org/10.15468/dl.zfaw79
Anopheles gambiae	-5.760093	9.255427	Unclassified	GBIF: https://doi.org/10.15468/dl.zfaw79
Anopheles gambiae	-5.76009	9.255415	Unclassified	GBIF: https://doi.org/10.15468/dl.zfaw79
Anopheles gambiae	-5.760085	9.255423	Unclassified	GBIF: https://doi.org/10.15468/dl.zfaw79
Anopheles gambiae	-5.760082	9.255452	Unclassified	GBIF: https://doi.org/10.15468/dl.zfaw79
Anopheles gambiae	-5.759303	9.25474	Unclassified	GBIF: https://doi.org/10.15468/dl.zfaw79
Anopheles gambiae	-5.759295	9.254716	Unclassified	GBIF: https://doi.org/10.15468/dl.zfaw79
Anopheles gambiae	-5.759284	9.254708	Unclassified	GBIF: https://doi.org/10.15468/dl.zfaw79
Anopheles gambiae	-5.759272	9.254687	Unclassified	GBIF: https://doi.org/10.15468/dl.zfaw79
Anopheles gambiae	-5.759272	9.25472	Unclassified	GBIF: https://doi.org/10.15468/dl.zfaw79
Anopheles gambiae	-5.757217	9.210645	Unclassified	GBIF: https://doi.org/10.15468/dl.zfaw79
Anopheles gambiae	-5.757197	9.210644	Unclassified	GBIF: https://doi.org/10.15468/dl.zfaw79
Anopheles gambiae	-5.757192	9.210644	Unclassified	GBIF: https://doi.org/10.15468/dl.zfaw79
Anopheles gambiae	-5.757192	9.21066	Unclassified	GBIF: https://doi.org/10.15468/dl.zfaw79
Anopheles gambiae	-5.756871	9.210201	Unclassified	GBIF: https://doi.org/10.15468/dl.zfaw79
Anopheles gambiae	-5.756844	9.210213	Unclassified	GBIF: https://doi.org/10.15468/dl.zfaw79
Anopheles gambiae	-5.756829	9.210139	Unclassified	GBIF: https://doi.org/10.15468/dl.zfaw79
Anopheles gambiae	-5.756821	9.210185	Unclassified	GBIF: https://doi.org/10.15468/dl.zfaw79
Anopheles gambiae	-5.756814	9.210204	Unclassified	GBIF: https://doi.org/10.15468/dl.zfaw79
Anopheles gambiae	-5.756797	9.210179	Unclassified	GBIF: https://doi.org/10.15468/dl.zfaw79
Anopheles gambiae	-5.75628	9.210724	Unclassified	GBIF: https://doi.org/10.15468/dl.zfaw79
Anopheles gambiae	-5.756273	9.210743	Unclassified	GBIF: https://doi.org/10.15468/dl.zfaw79

Anopheles gambiae	-5.75627	9.210729	Unclassified	GBIF: https://doi.org/10.15468/dl.zfaw79
Anopheles gambiae	-5.756221	9.210022	Unclassified	GBIF: https://doi.org/10.15468/dl.zfaw79
Anopheles gambiae	-5.756218	9.210008	Unclassified	GBIF: https://doi.org/10.15468/dl.zfaw79
Anopheles gambiae	-5.756201	9.210018	Unclassified	GBIF: https://doi.org/10.15468/dl.zfaw79
Anopheles gambiae	-5.7562	9.20999	Unclassified	GBIF: https://doi.org/10.15468/dl.zfaw79
Anopheles gambiae	-5.756195	9.210037	Unclassified	GBIF: https://doi.org/10.15468/dl.zfaw79
Anopheles gambiae	-5.756174	9.210009	Unclassified	GBIF: https://doi.org/10.15468/dl.zfaw79
Anopheles gambiae	-5.751178	8.884021	Unclassified	GBIF: https://doi.org/10.15468/dl.zfaw79
Anopheles gambiae	-5.751124	8.883995	Unclassified	GBIF: https://doi.org/10.15468/dl.zfaw79
Anopheles gambiae	-5.751123	8.884024	Unclassified	GBIF: https://doi.org/10.15468/dl.zfaw79
Anopheles gambiae	-5.750521	8.885467	Unclassified	GBIF: https://doi.org/10.15468/dl.zfaw79
Anopheles gambiae	-5.750488	8.885481	Unclassified	GBIF: https://doi.org/10.15468/dl.zfaw79
Anopheles gambiae	-5.750474	8.885456	Unclassified	GBIF: https://doi.org/10.15468/dl.zfaw79
Anopheles gambiae	-5.75043	8.884102	Unclassified	GBIF: https://doi.org/10.15468/dl.zfaw79
Anopheles gambiae	-5.75041	8.884148	Unclassified	GBIF: https://doi.org/10.15468/dl.zfaw79
Anopheles gambiae	-5.75	10.93333	Unclassified	GBIF: https://doi.org/10.15468/dl.zfaw79
Anopheles gambiae	-5.749422	8.884662	Unclassified	GBIF: https://doi.org/10.15468/dl.zfaw79
Anopheles gambiae	-5.749404	8.884662	Unclassified	GBIF: https://doi.org/10.15468/dl.zfaw79
Anopheles gambiae	-5.749401	8.884694	Unclassified	GBIF: https://doi.org/10.15468/dl.zfaw79
Anopheles gambiae	-5.748971	9.060317	Unclassified	GBIF: https://doi.org/10.15468/dl.zfaw79
Anopheles gambiae	-5.7484	9.059754	Unclassified	GBIF: https://doi.org/10.15468/dl.zfaw79
Anopheles gambiae	-5.748392	9.059769	Unclassified	GBIF: https://doi.org/10.15468/dl.zfaw79
Anopheles gambiae	-5.748389	9.059743	Unclassified	GBIF: https://doi.org/10.15468/dl.zfaw79
Anopheles gambiae	-5.748381	9.059729	Unclassified	GBIF: https://doi.org/10.15468/dl.zfaw79
Anopheles gambiae	-5.748377	9.059728	Unclassified	GBIF: https://doi.org/10.15468/dl.zfaw79
Anopheles gambiae	-5.748355	9.060641	Unclassified	GBIF: https://doi.org/10.15468/dl.zfaw79
Anopheles gambiae	-5.748329	9.060099	Unclassified	GBIF: https://doi.org/10.15468/dl.zfaw79
Anopheles gambiae	-5.748228	9.060728	Unclassified	GBIF: https://doi.org/10.15468/dl.zfaw79
Anopheles gambiae	-5.748215	9.060273	Unclassified	GBIF: https://doi.org/10.15468/dl.zfaw79
Anopheles gambiae	-5.748212	9.060345	Unclassified	GBIF: https://doi.org/10.15468/dl.zfaw79
Anopheles gambiae	-5.748197	9.060688	Unclassified	GBIF: https://doi.org/10.15468/dl.zfaw79
Anopheles gambiae	-5.748191	9.06072	Unclassified	GBIF: https://doi.org/10.15468/dl.zfaw79
Anopheles gambiae	-5.748185	9.06028	Unclassified	GBIF: https://doi.org/10.15468/dl.zfaw79
Anopheles gambiae	-5.748172	9.060685	Unclassified	GBIF: https://doi.org/10.15468/dl.zfaw79
Anopheles gambiae	-5.748167	9.060317	Unclassified	GBIF: https://doi.org/10.15468/dl.zfaw79
Anopheles gambiae	-5.747999	9.060559	Unclassified	GBIF: https://doi.org/10.15468/dl.zfaw79
Anopheles gambiae	-5.747959	9.060533	Unclassified	GBIF: https://doi.org/10.15468/dl.zfaw79
Anopheles gambiae	-5.747939	9.060538	Unclassified	GBIF: https://doi.org/10.15468/dl.zfaw79
Anopheles gambiae	-5.747937	9.060545	Unclassified	GBIF: https://doi.org/10.15468/dl.zfaw79
Anopheles gambiae	-5.740081	9.235433	Unclassified	GBIF: https://doi.org/10.15468/dl.zfaw79
Anopheles gambiae	-5.740076	9.235419	Unclassified	GBIF: https://doi.org/10.15468/dl.zfaw79
Anopheles gambiae	-5.740062	9.235212	Unclassified	GBIF: https://doi.org/10.15468/dl.zfaw79

Anopheles gambiae	-5.739909	9.23508	Unclassified	GBIF: https://doi.org/10.15468/dl.zfaw79
Anopheles gambiae	-5.739851	9.23497	Unclassified	GBIF: https://doi.org/10.15468/dl.zfaw79
Anopheles gambiae	-5.739793	9.235023	Unclassified	GBIF: https://doi.org/10.15468/dl.zfaw79
Anopheles gambiae	-5.739785	9.23504	Unclassified	GBIF: https://doi.org/10.15468/dl.zfaw79
Anopheles gambiae	-5.739717	9.235201	Unclassified	GBIF: https://doi.org/10.15468/dl.zfaw79
Anopheles gambiae	-5.73969	9.234908	Unclassified	GBIF: https://doi.org/10.15468/dl.zfaw79
Anopheles gambiae	-5.739602	9.23413	Unclassified	GBIF: https://doi.org/10.15468/dl.zfaw79
Anopheles gambiae	-5.739575	9.2341	Unclassified	GBIF: https://doi.org/10.15468/dl.zfaw79
Anopheles gambiae	-5.739545	9.234149	Unclassified	GBIF: https://doi.org/10.15468/dl.zfaw79
Anopheles gambiae	-5.739538	9.234096	Unclassified	GBIF: https://doi.org/10.15468/dl.zfaw79
Anopheles gambiae	-5.739512	9.234063	Unclassified	GBIF: https://doi.org/10.15468/dl.zfaw79
Anopheles gambiae	-5.739506	9.234096	Unclassified	GBIF: https://doi.org/10.15468/dl.zfaw79
Anopheles gambiae	-5.7395	9.23408	Unclassified	GBIF: https://doi.org/10.15468/dl.zfaw79
Anopheles gambiae	-5.73899	9.234947	Unclassified	GBIF: https://doi.org/10.15468/dl.zfaw79
Anopheles gambiae	-5.738183	9.234103	Unclassified	GBIF: https://doi.org/10.15468/dl.zfaw79
Anopheles gambiae	-5.738157	9.234128	Unclassified	GBIF: https://doi.org/10.15468/dl.zfaw79
Anopheles gambiae	-5.738153	9.234166	Unclassified	GBIF: https://doi.org/10.15468/dl.zfaw79
Anopheles gambiae	-5.738152	9.234097	Unclassified	GBIF: https://doi.org/10.15468/dl.zfaw79
Anopheles gambiae	-5.73815	9.234117	Unclassified	GBIF: https://doi.org/10.15468/dl.zfaw79
Anopheles gambiae	-5.738114	9.234081	Unclassified	GBIF: https://doi.org/10.15468/dl.zfaw79
Anopheles gambiae	-5.737891	9.234798	Unclassified	GBIF: https://doi.org/10.15468/dl.zfaw79
Anopheles gambiae	-5.711436	8.991644	Unclassified	GBIF: https://doi.org/10.15468/dl.zfaw79
Anopheles gambiae	-5.711436	8.9917	Unclassified	GBIF: https://doi.org/10.15468/dl.zfaw79
Anopheles gambiae	-5.711433	8.991674	Unclassified	GBIF: https://doi.org/10.15468/dl.zfaw79
Anopheles gambiae	-5.711422	8.991668	Unclassified	GBIF: https://doi.org/10.15468/dl.zfaw79
Anopheles gambiae	-5.710868	8.991617	Unclassified	GBIF: https://doi.org/10.15468/dl.zfaw79
Anopheles gambiae	-5.710836	8.991616	Unclassified	GBIF: https://doi.org/10.15468/dl.zfaw79
Anopheles gambiae	-5.710836	8.991587	Unclassified	GBIF: https://doi.org/10.15468/dl.zfaw79
Anopheles gambiae	-5.710814	8.991646	Unclassified	GBIF: https://doi.org/10.15468/dl.zfaw79
Anopheles gambiae	-5.710799	8.991622	Unclassified	GBIF: https://doi.org/10.15468/dl.zfaw79
Anopheles gambiae	-5.710629	8.991153	Unclassified	GBIF: https://doi.org/10.15468/dl.zfaw79
Anopheles gambiae	-5.710587	8.991091	Unclassified	GBIF: https://doi.org/10.15468/dl.zfaw79
Anopheles gambiae	-5.710585	8.991108	Unclassified	GBIF: https://doi.org/10.15468/dl.zfaw79
Anopheles gambiae	-5.710578	8.991117	Unclassified	GBIF: https://doi.org/10.15468/dl.zfaw79
Anopheles gambiae	-5.710549	8.991094	Unclassified	GBIF: https://doi.org/10.15468/dl.zfaw79
Anopheles gambiae	-5.709842	8.991595	Unclassified	GBIF: https://doi.org/10.15468/dl.zfaw79
Anopheles gambiae	-5.709838	8.991563	Unclassified	GBIF: https://doi.org/10.15468/dl.zfaw79
Anopheles gambiae	-5.709821	8.991553	Unclassified	GBIF: https://doi.org/10.15468/dl.zfaw79
Anopheles gambiae	-5.709808	8.991549	Unclassified	GBIF: https://doi.org/10.15468/dl.zfaw79
Anopheles gambiae	-5.7	11.46667	Unclassified	GBIF: https://doi.org/10.15468/dl.zfaw79
Anopheles gambiae	-5.688522	9.161182	Unclassified	GBIF: https://doi.org/10.15468/dl.zfaw79
Anopheles gambiae	-5.68848	9.161236	Unclassified	GBIF: https://doi.org/10.15468/dl.zfaw79

Anopheles gambiae	-5.688478	9.161241	Unclassified	GBIF: https://doi.org/10.15468/dl.zfaw79
Anopheles gambiae	-5.688469	9.161255	Unclassified	GBIF: https://doi.org/10.15468/dl.zfaw79
Anopheles gambiae	-5.688465	9.161274	Unclassified	GBIF: https://doi.org/10.15468/dl.zfaw79
Anopheles gambiae	-5.687897	9.160935	Unclassified	GBIF: https://doi.org/10.15468/dl.zfaw79
Anopheles gambiae	-5.687875	9.160922	Unclassified	GBIF: https://doi.org/10.15468/dl.zfaw79
Anopheles gambiae	-5.687869	9.160929	Unclassified	GBIF: https://doi.org/10.15468/dl.zfaw79
Anopheles gambiae	-5.687791	9.159889	Unclassified	GBIF: https://doi.org/10.15468/dl.zfaw79
Anopheles gambiae	-5.687781	9.159902	Unclassified	GBIF: https://doi.org/10.15468/dl.zfaw79
Anopheles gambiae	-5.687779	9.159899	Unclassified	GBIF: https://doi.org/10.15468/dl.zfaw79
Anopheles gambiae	-5.687778	9.159933	Unclassified	GBIF: https://doi.org/10.15468/dl.zfaw79
Anopheles gambiae	-5.68777	9.159892	Unclassified	GBIF: https://doi.org/10.15468/dl.zfaw79
Anopheles gambiae	-5.687142	9.160252	Unclassified	GBIF: https://doi.org/10.15468/dl.zfaw79
Anopheles gambiae	-5.68714	9.160268	Unclassified	GBIF: https://doi.org/10.15468/dl.zfaw79
Anopheles gambiae	-5.687131	9.160263	Unclassified	GBIF: https://doi.org/10.15468/dl.zfaw79
Anopheles gambiae	-5.687114	9.160227	Unclassified	GBIF: https://doi.org/10.15468/dl.zfaw79
Anopheles gambiae	-5.66878	9.237231	Unclassified	GBIF: https://doi.org/10.15468/dl.zfaw79
Anopheles gambiae	-5.668774	9.237268	Unclassified	GBIF: https://doi.org/10.15468/dl.zfaw79
Anopheles gambiae	-5.668768	9.237244	Unclassified	GBIF: https://doi.org/10.15468/dl.zfaw79
Anopheles gambiae	-5.668763	9.237229	Unclassified	GBIF: https://doi.org/10.15468/dl.zfaw79
Anopheles gambiae	-5.66874	9.237223	Unclassified	GBIF: https://doi.org/10.15468/dl.zfaw79
Anopheles gambiae	-5.668542	9.236578	Unclassified	GBIF: https://doi.org/10.15468/dl.zfaw79
Anopheles gambiae	-5.668529	9.236651	Unclassified	GBIF: https://doi.org/10.15468/dl.zfaw79
Anopheles gambiae	-5.668521	9.23663	Unclassified	GBIF: https://doi.org/10.15468/dl.zfaw79
Anopheles gambiae	-5.668521	9.236631	Unclassified	GBIF: https://doi.org/10.15468/dl.zfaw79
Anopheles gambiae	-5.668521	9.23779	Unclassified	GBIF: https://doi.org/10.15468/dl.zfaw79
Anopheles gambiae	-5.668519	9.236654	Unclassified	GBIF: https://doi.org/10.15468/dl.zfaw79
Anopheles gambiae	-5.668484	9.237798	Unclassified	GBIF: https://doi.org/10.15468/dl.zfaw79
Anopheles gambiae	-5.668482	9.237775	Unclassified	GBIF: https://doi.org/10.15468/dl.zfaw79
Anopheles gambiae	-5.66847	9.237793	Unclassified	GBIF: https://doi.org/10.15468/dl.zfaw79
Anopheles gambiae	-5.668462	9.237755	Unclassified	GBIF: https://doi.org/10.15468/dl.zfaw79
Anopheles gambiae	-5.668395	9.236691	Unclassified	GBIF: https://doi.org/10.15468/dl.zfaw79
Anopheles gambiae	-5.667822	9.23615	Unclassified	GBIF: https://doi.org/10.15468/dl.zfaw79
Anopheles gambiae	-5.667802	9.236155	Unclassified	GBIF: https://doi.org/10.15468/dl.zfaw79
Anopheles gambiae	-5.667792	9.236133	Unclassified	GBIF: https://doi.org/10.15468/dl.zfaw79
Anopheles gambiae	-5.667783	9.236149	Unclassified	GBIF: https://doi.org/10.15468/dl.zfaw79
Anopheles gambiae	-5.667778	9.23622	Unclassified	GBIF: https://doi.org/10.15468/dl.zfaw79
Anopheles gambiae	-5.66774	9.237004	Unclassified	GBIF: https://doi.org/10.15468/dl.zfaw79
Anopheles gambiae	-5.653322	8.948459	Unclassified	GBIF: https://doi.org/10.15468/dl.zfaw79
Anopheles gambiae	-5.653316	8.948413	Unclassified	GBIF: https://doi.org/10.15468/dl.zfaw79
Anopheles gambiae	-5.653268	8.948403	Unclassified	GBIF: https://doi.org/10.15468/dl.zfaw79
Anopheles gambiae	-5.653266	8.948469	Unclassified	GBIF: https://doi.org/10.15468/dl.zfaw79
Anopheles gambiae	-5.652866	8.949516	Unclassified	GBIF: https://doi.org/10.15468/dl.zfaw79

Anopheles gambiae	-5.652802	8.949554	Unclassified	GBIF: https://doi.org/10.15468/dl.zfaw79
Anopheles gambiae	-5.652783	8.949523	Unclassified	GBIF: https://doi.org/10.15468/dl.zfaw79
Anopheles gambiae	-5.652115	8.948168	Unclassified	GBIF: https://doi.org/10.15468/dl.zfaw79
Anopheles gambiae	-5.652101	8.948199	Unclassified	GBIF: https://doi.org/10.15468/dl.zfaw79
Anopheles gambiae	-5.652092	8.948175	Unclassified	GBIF: https://doi.org/10.15468/dl.zfaw79
Anopheles gambiae	-5.652092	8.948231	Unclassified	GBIF: https://doi.org/10.15468/dl.zfaw79
Anopheles gambiae	-5.651703	8.949318	Unclassified	GBIF: https://doi.org/10.15468/dl.zfaw79
Anopheles gambiae	-5.651657	8.949303	Unclassified	GBIF: https://doi.org/10.15468/dl.zfaw79
Anopheles gambiae	-5.651652	8.949291	Unclassified	GBIF: https://doi.org/10.15468/dl.zfaw79
Anopheles gambiae	-5.63333	9.45	Unclassified	GBIF: https://doi.org/10.15468/dl.zfaw79
Anopheles gambiae	-5.63333	11.3	Unclassified	GBIF: https://doi.org/10.15468/dl.zfaw79
Anopheles gambiae	-5.618515	9.25808	Unclassified	GBIF: https://doi.org/10.15468/dl.zfaw79
Anopheles gambiae	-5.618506	9.258062	Unclassified	GBIF: https://doi.org/10.15468/dl.zfaw79
Anopheles gambiae	-5.618501	9.258067	Unclassified	GBIF: https://doi.org/10.15468/dl.zfaw79
Anopheles gambiae	-5.618493	9.258067	Unclassified	GBIF: https://doi.org/10.15468/dl.zfaw79
Anopheles gambiae	-5.618458	9.257513	Unclassified	GBIF: https://doi.org/10.15468/dl.zfaw79
Anopheles gambiae	-5.618426	9.257508	Unclassified	GBIF: https://doi.org/10.15468/dl.zfaw79
Anopheles gambiae	-5.618424	9.257512	Unclassified	GBIF: https://doi.org/10.15468/dl.zfaw79
Anopheles gambiae	-5.618422	9.257481	Unclassified	GBIF: https://doi.org/10.15468/dl.zfaw79
Anopheles gambiae	-5.618396	9.257524	Unclassified	GBIF: https://doi.org/10.15468/dl.zfaw79
Anopheles gambiae	-5.617662	9.256227	Unclassified	GBIF: https://doi.org/10.15468/dl.zfaw79
Anopheles gambiae	-5.617656	9.256245	Unclassified	GBIF: https://doi.org/10.15468/dl.zfaw79
Anopheles gambiae	-5.617656	9.256214	Unclassified	GBIF: https://doi.org/10.15468/dl.zfaw79
Anopheles gambiae	-5.617631	9.256271	Unclassified	GBIF: https://doi.org/10.15468/dl.zfaw79
Anopheles gambiae	-5.617164	9.258627	Unclassified	GBIF: https://doi.org/10.15468/dl.zfaw79
Anopheles gambiae	-5.617162	9.258655	Unclassified	GBIF: https://doi.org/10.15468/dl.zfaw79
Anopheles gambiae	-5.617148	9.258641	Unclassified	GBIF: https://doi.org/10.15468/dl.zfaw79
Anopheles gambiae	-5.617145	9.258635	Unclassified	GBIF: https://doi.org/10.15468/dl.zfaw79
Anopheles gambiae	-5.617128	9.258666	Unclassified	GBIF: https://doi.org/10.15468/dl.zfaw79
Anopheles gambiae	-5.614412	9.251566	Unclassified	GBIF: https://doi.org/10.15468/dl.zfaw79
Anopheles gambiae	-5.614409	9.251595	Unclassified	GBIF: https://doi.org/10.15468/dl.zfaw79
Anopheles gambiae	-5.614406	9.251623	Unclassified	GBIF: https://doi.org/10.15468/dl.zfaw79
Anopheles gambiae	-5.614304	9.252539	Unclassified	GBIF: https://doi.org/10.15468/dl.zfaw79
Anopheles gambiae	-5.614276	9.252568	Unclassified	GBIF: https://doi.org/10.15468/dl.zfaw79
Anopheles gambiae	-5.614248	9.252597	Unclassified	GBIF: https://doi.org/10.15468/dl.zfaw79
Anopheles gambiae	-5.614039	9.251281	Unclassified	GBIF: https://doi.org/10.15468/dl.zfaw79
Anopheles gambiae	-5.613993	9.251237	Unclassified	GBIF: https://doi.org/10.15468/dl.zfaw79
Anopheles gambiae	-5.613946	9.251192	Unclassified	GBIF: https://doi.org/10.15468/dl.zfaw79
Anopheles gambiae	-5.613869	9.251788	Unclassified	GBIF: https://doi.org/10.15468/dl.zfaw79
Anopheles gambiae	-5.613851	9.251793	Unclassified	GBIF: https://doi.org/10.15468/dl.zfaw79
Anopheles gambiae	-5.613833	9.251798	Unclassified	GBIF: https://doi.org/10.15468/dl.zfaw79
Anopheles gambiae	-5.611362	9.326845	Unclassified	GBIF: https://doi.org/10.15468/dl.zfaw79

Anopheles gambiae	-5.611026	9.295963	Unclassified	GBIF: https://doi.org/10.15468/dl.zfaw79
Anopheles gambiae	-5.611	9.295939	Unclassified	GBIF: https://doi.org/10.15468/dl.zfaw79
Anopheles gambiae	-5.610998	9.295943	Unclassified	GBIF: https://doi.org/10.15468/dl.zfaw79
Anopheles gambiae	-5.610996	9.295945	Unclassified	GBIF: https://doi.org/10.15468/dl.zfaw79
Anopheles gambiae	-5.610987	9.295943	Unclassified	GBIF: https://doi.org/10.15468/dl.zfaw79
Anopheles gambiae	-5.610981	9.295961	Unclassified	GBIF: https://doi.org/10.15468/dl.zfaw79
Anopheles gambiae	-5.610639	9.327927	Unclassified	GBIF: https://doi.org/10.15468/dl.zfaw79
Anopheles gambiae	-5.610639	9.327938	Unclassified	GBIF: https://doi.org/10.15468/dl.zfaw79
Anopheles gambiae	-5.610638	9.327949	Unclassified	GBIF: https://doi.org/10.15468/dl.zfaw79
Anopheles gambiae	-5.610627	9.294486	Unclassified	GBIF: https://doi.org/10.15468/dl.zfaw79
Anopheles gambiae	-5.610591	9.294421	Unclassified	GBIF: https://doi.org/10.15468/dl.zfaw79
Anopheles gambiae	-5.610587	9.294459	Unclassified	GBIF: https://doi.org/10.15468/dl.zfaw79
Anopheles gambiae	-5.610585	9.294448	Unclassified	GBIF: https://doi.org/10.15468/dl.zfaw79
Anopheles gambiae	-5.610582	9.294411	Unclassified	GBIF: https://doi.org/10.15468/dl.zfaw79
Anopheles gambiae	-5.610569	9.294453	Unclassified	GBIF: https://doi.org/10.15468/dl.zfaw79
Anopheles gambiae	-5.610559	9.294423	Unclassified	GBIF: https://doi.org/10.15468/dl.zfaw79
Anopheles gambiae	-5.610008	9.297116	Unclassified	GBIF: https://doi.org/10.15468/dl.zfaw79
Anopheles gambiae	-5.610003	9.297115	Unclassified	GBIF: https://doi.org/10.15468/dl.zfaw79
Anopheles gambiae	-5.609981	9.297148	Unclassified	GBIF: https://doi.org/10.15468/dl.zfaw79
Anopheles gambiae	-5.609976	9.297136	Unclassified	GBIF: https://doi.org/10.15468/dl.zfaw79
Anopheles gambiae	-5.609969	9.297172	Unclassified	GBIF: https://doi.org/10.15468/dl.zfaw79
Anopheles gambiae	-5.609964	9.297192	Unclassified	GBIF: https://doi.org/10.15468/dl.zfaw79
Anopheles gambiae	-5.609825	9.296094	Unclassified	GBIF: https://doi.org/10.15468/dl.zfaw79
Anopheles gambiae	-5.609765	9.29623	Unclassified	GBIF: https://doi.org/10.15468/dl.zfaw79
Anopheles gambiae	-5.609751	9.296192	Unclassified	GBIF: https://doi.org/10.15468/dl.zfaw79
Anopheles gambiae	-5.609736	9.296228	Unclassified	GBIF: https://doi.org/10.15468/dl.zfaw79
Anopheles gambiae	-5.609729	9.296244	Unclassified	GBIF: https://doi.org/10.15468/dl.zfaw79
Anopheles gambiae	-5.609727	9.296226	Unclassified	GBIF: https://doi.org/10.15468/dl.zfaw79
Anopheles gambiae	-5.609717	9.296232	Unclassified	GBIF: https://doi.org/10.15468/dl.zfaw79
Anopheles gambiae	-5.609294	9.326399	Unclassified	GBIF: https://doi.org/10.15468/dl.zfaw79
Anopheles gambiae	-5.609289	9.326395	Unclassified	GBIF: https://doi.org/10.15468/dl.zfaw79
Anopheles gambiae	-5.609283	9.32639	Unclassified	GBIF: https://doi.org/10.15468/dl.zfaw79
Anopheles gambiae	-5.608625	9.327883	Unclassified	GBIF: https://doi.org/10.15468/dl.zfaw79
Anopheles gambiae	-5.60861	9.327875	Unclassified	GBIF: https://doi.org/10.15468/dl.zfaw79
Anopheles gambiae	-5.608595	9.327866	Unclassified	GBIF: https://doi.org/10.15468/dl.zfaw79
Anopheles gambiae	-5.606912	9.186423	Unclassified	GBIF: https://doi.org/10.15468/dl.zfaw79
Anopheles gambiae	-5.606884	9.186423	Unclassified	GBIF: https://doi.org/10.15468/dl.zfaw79
Anopheles gambiae	-5.60686	9.186424	Unclassified	GBIF: https://doi.org/10.15468/dl.zfaw79
Anopheles gambiae	-5.606842	9.186437	Unclassified	GBIF: https://doi.org/10.15468/dl.zfaw79
Anopheles gambiae	-5.606349	9.325325	Unclassified	GBIF: https://doi.org/10.15468/dl.zfaw79
Anopheles gambiae	-5.60632	9.325318	Unclassified	GBIF: https://doi.org/10.15468/dl.zfaw79
Anopheles gambiae	-5.606311	9.325334	Unclassified	GBIF: https://doi.org/10.15468/dl.zfaw79

Anopheles gambiae	-5.606308	9.325325	Unclassified	GBIF: https://doi.org/10.15468/dl.zfaw79
Anopheles gambiae	-5.606301	9.325347	Unclassified	GBIF: https://doi.org/10.15468/dl.zfaw79
Anopheles gambiae	-5.606288	9.325316	Unclassified	GBIF: https://doi.org/10.15468/dl.zfaw79
Anopheles gambiae	-5.606275	9.325311	Unclassified	GBIF: https://doi.org/10.15468/dl.zfaw79
Anopheles gambiae	-5.606271	9.186539	Unclassified	GBIF: https://doi.org/10.15468/dl.zfaw79
Anopheles gambiae	-5.606258	9.186535	Unclassified	GBIF: https://doi.org/10.15468/dl.zfaw79
Anopheles gambiae	-5.606246	9.18655	Unclassified	GBIF: https://doi.org/10.15468/dl.zfaw79
Anopheles gambiae	-5.606181	9.323405	Unclassified	GBIF: https://doi.org/10.15468/dl.zfaw79
Anopheles gambiae	-5.606157	9.323317	Unclassified	GBIF: https://doi.org/10.15468/dl.zfaw79
Anopheles gambiae	-5.606152	9.323367	Unclassified	GBIF: https://doi.org/10.15468/dl.zfaw79
Anopheles gambiae	-5.60615	9.323356	Unclassified	GBIF: https://doi.org/10.15468/dl.zfaw79
Anopheles gambiae	-5.606147	9.323375	Unclassified	GBIF: https://doi.org/10.15468/dl.zfaw79
Anopheles gambiae	-5.606128	9.323391	Unclassified	GBIF: https://doi.org/10.15468/dl.zfaw79
Anopheles gambiae	-5.605703	9.327494	Unclassified	GBIF: https://doi.org/10.15468/dl.zfaw79
Anopheles gambiae	-5.605692	9.327529	Unclassified	GBIF: https://doi.org/10.15468/dl.zfaw79
Anopheles gambiae	-5.605684	9.327505	Unclassified	GBIF: https://doi.org/10.15468/dl.zfaw79
Anopheles gambiae	-5.605683	9.327515	Unclassified	GBIF: https://doi.org/10.15468/dl.zfaw79
Anopheles gambiae	-5.605678	9.327542	Unclassified	GBIF: https://doi.org/10.15468/dl.zfaw79
Anopheles gambiae	-5.605675	9.327501	Unclassified	GBIF: https://doi.org/10.15468/dl.zfaw79
Anopheles gambiae	-5.605667	9.327522	Unclassified	GBIF: https://doi.org/10.15468/dl.zfaw79
Anopheles gambiae	-5.605534	9.186001	Unclassified	GBIF: https://doi.org/10.15468/dl.zfaw79
Anopheles gambiae	-5.605525	9.186011	Unclassified	GBIF: https://doi.org/10.15468/dl.zfaw79
Anopheles gambiae	-5.605499	9.186006	Unclassified	GBIF: https://doi.org/10.15468/dl.zfaw79
Anopheles gambiae	-5.605484	9.186014	Unclassified	GBIF: https://doi.org/10.15468/dl.zfaw79
Anopheles gambiae	-5.605422	9.186231	Unclassified	GBIF: https://doi.org/10.15468/dl.zfaw79
Anopheles gambiae	-5.605419	9.186237	Unclassified	GBIF: https://doi.org/10.15468/dl.zfaw79
Anopheles gambiae	-5.605388	9.186283	Unclassified	GBIF: https://doi.org/10.15468/dl.zfaw79
Anopheles gambiae	-5.605165	9.188016	Unclassified	GBIF: https://doi.org/10.15468/dl.zfaw79
Anopheles gambiae	-5.605161	9.188004	Unclassified	GBIF: https://doi.org/10.15468/dl.zfaw79
Anopheles gambiae	-5.605151	9.187994	Unclassified	GBIF: https://doi.org/10.15468/dl.zfaw79
Anopheles gambiae	-5.605145	9.18802	Unclassified	GBIF: https://doi.org/10.15468/dl.zfaw79
Anopheles gambiae	-5.605143	9.188022	Unclassified	GBIF: https://doi.org/10.15468/dl.zfaw79
Anopheles gambiae	-5.604381	9.186696	Unclassified	GBIF: https://doi.org/10.15468/dl.zfaw79
Anopheles gambiae	-5.604367	9.186717	Unclassified	GBIF: https://doi.org/10.15468/dl.zfaw79
Anopheles gambiae	-5.604361	9.186701	Unclassified	GBIF: https://doi.org/10.15468/dl.zfaw79
Anopheles gambiae	-5.604357	9.186722	Unclassified	GBIF: https://doi.org/10.15468/dl.zfaw79
Anopheles gambiae	-5.604355	9.186758	Unclassified	GBIF: https://doi.org/10.15468/dl.zfaw79
Anopheles gambiae	-5.603134	9.327117	Unclassified	GBIF: https://doi.org/10.15468/dl.zfaw79
Anopheles gambiae	-5.603104	9.327091	Unclassified	GBIF: https://doi.org/10.15468/dl.zfaw79
Anopheles gambiae	-5.603098	9.327075	Unclassified	GBIF: https://doi.org/10.15468/dl.zfaw79
Anopheles gambiae	-5.603093	9.327074	Unclassified	GBIF: https://doi.org/10.15468/dl.zfaw79
Anopheles gambiae	-5.603075	9.327073	Unclassified	GBIF: https://doi.org/10.15468/dl.zfaw79

Anopheles gambiae	-5.603074	9.327061	Unclassified	GBIF: https://doi.org/10.15468/dl.zfaw79
Anopheles gambiae	-5.5896	9.353064	Unclassified	GBIF: https://doi.org/10.15468/dl.zfaw79
Anopheles gambiae	-5.5896	9.353077	Unclassified	GBIF: https://doi.org/10.15468/dl.zfaw79
Anopheles gambiae	-5.589588	9.353045	Unclassified	GBIF: https://doi.org/10.15468/dl.zfaw79
Anopheles gambiae	-5.589583	9.353041	Unclassified	GBIF: https://doi.org/10.15468/dl.zfaw79
Anopheles gambiae	-5.589579	9.353049	Unclassified	GBIF: https://doi.org/10.15468/dl.zfaw79
Anopheles gambiae	-5.589576	9.353055	Unclassified	GBIF: https://doi.org/10.15468/dl.zfaw79
Anopheles gambiae	-5.589554	9.353037	Unclassified	GBIF: https://doi.org/10.15468/dl.zfaw79
Anopheles gambiae	-5.589552	9.353027	Unclassified	GBIF: https://doi.org/10.15468/dl.zfaw79
Anopheles gambiae	-5.588412	9.352941	Unclassified	GBIF: https://doi.org/10.15468/dl.zfaw79
Anopheles gambiae	-5.588393	9.352947	Unclassified	GBIF: https://doi.org/10.15468/dl.zfaw79
Anopheles gambiae	-5.588393	9.352932	Unclassified	GBIF: https://doi.org/10.15468/dl.zfaw79
Anopheles gambiae	-5.588391	9.352972	Unclassified	GBIF: https://doi.org/10.15468/dl.zfaw79
Anopheles gambiae	-5.588385	9.352951	Unclassified	GBIF: https://doi.org/10.15468/dl.zfaw79
Anopheles gambiae	-5.588378	9.352953	Unclassified	GBIF: https://doi.org/10.15468/dl.zfaw79
Anopheles gambiae	-5.58837	9.352946	Unclassified	GBIF: https://doi.org/10.15468/dl.zfaw79
Anopheles gambiae	-5.588357	9.352961	Unclassified	GBIF: https://doi.org/10.15468/dl.zfaw79
Anopheles gambiae	-5.587198	9.351439	Unclassified	GBIF: https://doi.org/10.15468/dl.zfaw79
Anopheles gambiae	-5.587187	9.351349	Unclassified	GBIF: https://doi.org/10.15468/dl.zfaw79
Anopheles gambiae	-5.587179	9.351418	Unclassified	GBIF: https://doi.org/10.15468/dl.zfaw79
Anopheles gambiae	-5.587175	9.351393	Unclassified	GBIF: https://doi.org/10.15468/dl.zfaw79
Anopheles gambiae	-5.587175	9.351377	Unclassified	GBIF: https://doi.org/10.15468/dl.zfaw79
Anopheles gambiae	-5.587173	9.351402	Unclassified	GBIF: https://doi.org/10.15468/dl.zfaw79
Anopheles gambiae	-5.587167	9.351382	Unclassified	GBIF: https://doi.org/10.15468/dl.zfaw79
Anopheles gambiae	-5.586841	9.353462	Unclassified	GBIF: https://doi.org/10.15468/dl.zfaw79
Anopheles gambiae	-5.586839	9.353444	Unclassified	GBIF: https://doi.org/10.15468/dl.zfaw79
Anopheles gambiae	-5.586836	9.353487	Unclassified	GBIF: https://doi.org/10.15468/dl.zfaw79
Anopheles gambiae	-5.586835	9.353476	Unclassified	GBIF: https://doi.org/10.15468/dl.zfaw79
Anopheles gambiae	-5.586832	9.353458	Unclassified	GBIF: https://doi.org/10.15468/dl.zfaw79
Anopheles gambiae	-5.586832	9.353438	Unclassified	GBIF: https://doi.org/10.15468/dl.zfaw79
Anopheles gambiae	-5.58683	9.353454	Unclassified	GBIF: https://doi.org/10.15468/dl.zfaw79
Anopheles gambiae	-5.586812	9.353442	Unclassified	GBIF: https://doi.org/10.15468/dl.zfaw79
Anopheles gambiae	-5.584327	9.278919	Unclassified	GBIF: https://doi.org/10.15468/dl.zfaw79
Anopheles gambiae	-5.584318	9.278908	Unclassified	GBIF: https://doi.org/10.15468/dl.zfaw79
Anopheles gambiae	-5.584318	9.278902	Unclassified	GBIF: https://doi.org/10.15468/dl.zfaw79
Anopheles gambiae	-5.584316	9.27892	Unclassified	GBIF: https://doi.org/10.15468/dl.zfaw79
Anopheles gambiae	-5.584312	9.278941	Unclassified	GBIF: https://doi.org/10.15468/dl.zfaw79
Anopheles gambiae	-5.584311	9.278919	Unclassified	GBIF: https://doi.org/10.15468/dl.zfaw79
Anopheles gambiae	-5.584309	9.278933	Unclassified	GBIF: https://doi.org/10.15468/dl.zfaw79
Anopheles gambiae	-5.58333	12.21667	Unclassified	GBIF: https://doi.org/10.15468/dl.zfaw79
Anopheles gambiae	-5.583271	9.279775	Unclassified	GBIF: https://doi.org/10.15468/dl.zfaw79
Anopheles gambiae	-5.583236	9.27985	Unclassified	GBIF: https://doi.org/10.15468/dl.zfaw79

Anopheles gambiae	-5.583211	9.279842	Unclassified	GBIF: https://doi.org/10.15468/dl.zfaw79
Anopheles gambiae	-5.583193	9.279842	Unclassified	GBIF: https://doi.org/10.15468/dl.zfaw79
Anopheles gambiae	-5.583192	9.279868	Unclassified	GBIF: https://doi.org/10.15468/dl.zfaw79
Anopheles gambiae	-5.583183	9.279827	Unclassified	GBIF: https://doi.org/10.15468/dl.zfaw79
Anopheles gambiae	-5.581828	9.279254	Unclassified	GBIF: https://doi.org/10.15468/dl.zfaw79
Anopheles gambiae	-5.581782	9.279268	Unclassified	GBIF: https://doi.org/10.15468/dl.zfaw79
Anopheles gambiae	-5.58177	9.279226	Unclassified	GBIF: https://doi.org/10.15468/dl.zfaw79
Anopheles gambiae	-5.581767	9.279262	Unclassified	GBIF: https://doi.org/10.15468/dl.zfaw79
Anopheles gambiae	-5.581748	9.279259	Unclassified	GBIF: https://doi.org/10.15468/dl.zfaw79
Anopheles gambiae	-5.581733	9.279249	Unclassified	GBIF: https://doi.org/10.15468/dl.zfaw79
Anopheles gambiae	-5.581431	9.27938	Unclassified	GBIF: https://doi.org/10.15468/dl.zfaw79
Anopheles gambiae	-5.580081	9.279904	Unclassified	GBIF: https://doi.org/10.15468/dl.zfaw79
Anopheles gambiae	-5.579754	9.280038	Unclassified	GBIF: https://doi.org/10.15468/dl.zfaw79
Anopheles gambiae	-5.579752	9.280037	Unclassified	GBIF: https://doi.org/10.15468/dl.zfaw79
Anopheles gambiae	-5.579741	9.280029	Unclassified	GBIF: https://doi.org/10.15468/dl.zfaw79
Anopheles gambiae	-5.579731	9.280032	Unclassified	GBIF: https://doi.org/10.15468/dl.zfaw79
Anopheles gambiae	-5.579727	9.280025	Unclassified	GBIF: https://doi.org/10.15468/dl.zfaw79
Anopheles gambiae	-5.572192	9.398714	Unclassified	GBIF: https://doi.org/10.15468/dl.zfaw79
Anopheles gambiae	-5.572132	9.398719	Unclassified	GBIF: https://doi.org/10.15468/dl.zfaw79
Anopheles gambiae	-5.572131	9.398706	Unclassified	GBIF: https://doi.org/10.15468/dl.zfaw79
Anopheles gambiae	-5.572123	9.398704	Unclassified	GBIF: https://doi.org/10.15468/dl.zfaw79
Anopheles gambiae	-5.572105	9.398717	Unclassified	GBIF: https://doi.org/10.15468/dl.zfaw79
Anopheles gambiae	-5.571877	9.3979	Unclassified	GBIF: https://doi.org/10.15468/dl.zfaw79
Anopheles gambiae	-5.571858	9.397916	Unclassified	GBIF: https://doi.org/10.15468/dl.zfaw79
Anopheles gambiae	-5.571802	9.39787	Unclassified	GBIF: https://doi.org/10.15468/dl.zfaw79
Anopheles gambiae	-5.57176	9.399092	Unclassified	GBIF: https://doi.org/10.15468/dl.zfaw79
Anopheles gambiae	-5.571752	9.397859	Unclassified	GBIF: https://doi.org/10.15468/dl.zfaw79
Anopheles gambiae	-5.571746	9.39781	Unclassified	GBIF: https://doi.org/10.15468/dl.zfaw79
Anopheles gambiae	-5.571743	9.399109	Unclassified	GBIF: https://doi.org/10.15468/dl.zfaw79
Anopheles gambiae	-5.571728	9.397846	Unclassified	GBIF: https://doi.org/10.15468/dl.zfaw79
Anopheles gambiae	-5.571724	9.399129	Unclassified	GBIF: https://doi.org/10.15468/dl.zfaw79
Anopheles gambiae	-5.571713	9.39909	Unclassified	GBIF: https://doi.org/10.15468/dl.zfaw79
Anopheles gambiae	-5.571699	9.399135	Unclassified	GBIF: https://doi.org/10.15468/dl.zfaw79
Anopheles gambiae	-5.571474	9.398735	Unclassified	GBIF: https://doi.org/10.15468/dl.zfaw79
Anopheles gambiae	-5.570204	9.396859	Unclassified	GBIF: https://doi.org/10.15468/dl.zfaw79
Anopheles gambiae	-5.569917	9.397328	Unclassified	GBIF: https://doi.org/10.15468/dl.zfaw79
Anopheles gambiae	-5.569897	9.397337	Unclassified	GBIF: https://doi.org/10.15468/dl.zfaw79
Anopheles gambiae	-5.569895	9.397348	Unclassified	GBIF: https://doi.org/10.15468/dl.zfaw79
Anopheles gambiae	-5.569882	9.397355	Unclassified	GBIF: https://doi.org/10.15468/dl.zfaw79
Anopheles gambiae	-5.568261	9.29786	Unclassified	GBIF: https://doi.org/10.15468/dl.zfaw79
Anopheles gambiae	-5.568252	9.297859	Unclassified	GBIF: https://doi.org/10.15468/dl.zfaw79
Anopheles gambiae	-5.568234	9.297857	Unclassified	GBIF: https://doi.org/10.15468/dl.zfaw79

Anopheles gambiae	-5.568229	9.297843	Unclassified	GBIF: https://doi.org/10.15468/dl.zfaw79
Anopheles gambiae	-5.56822	9.297876	Unclassified	GBIF: https://doi.org/10.15468/dl.zfaw79
Anopheles gambiae	-5.567933	9.2969	Unclassified	GBIF: https://doi.org/10.15468/dl.zfaw79
Anopheles gambiae	-5.567927	9.296889	Unclassified	GBIF: https://doi.org/10.15468/dl.zfaw79
Anopheles gambiae	-5.567923	9.296866	Unclassified	GBIF: https://doi.org/10.15468/dl.zfaw79
Anopheles gambiae	-5.567919	9.296887	Unclassified	GBIF: https://doi.org/10.15468/dl.zfaw79
Anopheles gambiae	-5.567901	9.296939	Unclassified	GBIF: https://doi.org/10.15468/dl.zfaw79
Anopheles gambiae	-5.567202	9.29834	Unclassified	GBIF: https://doi.org/10.15468/dl.zfaw79
Anopheles gambiae	-5.567198	9.298328	Unclassified	GBIF: https://doi.org/10.15468/dl.zfaw79
Anopheles gambiae	-5.567192	9.298326	Unclassified	GBIF: https://doi.org/10.15468/dl.zfaw79
Anopheles gambiae	-5.567186	9.2983	Unclassified	GBIF: https://doi.org/10.15468/dl.zfaw79
Anopheles gambiae	-5.566826	9.391943	Unclassified	GBIF: https://doi.org/10.15468/dl.zfaw79
Anopheles gambiae	-5.566816	9.391947	Unclassified	GBIF: https://doi.org/10.15468/dl.zfaw79
Anopheles gambiae	-5.566787	9.391945	Unclassified	GBIF: https://doi.org/10.15468/dl.zfaw79
Anopheles gambiae	-5.566717	9.299286	Unclassified	GBIF: https://doi.org/10.15468/dl.zfaw79
Anopheles gambiae	-5.566714	9.393027	Unclassified	GBIF: https://doi.org/10.15468/dl.zfaw79
Anopheles gambiae	-5.566699	9.39305	Unclassified	GBIF: https://doi.org/10.15468/dl.zfaw79
Anopheles gambiae	-5.566694	9.393031	Unclassified	GBIF: https://doi.org/10.15468/dl.zfaw79
Anopheles gambiae	-5.566669	9.393017	Unclassified	GBIF: https://doi.org/10.15468/dl.zfaw79
Anopheles gambiae	-5.566657	9.299324	Unclassified	GBIF: https://doi.org/10.15468/dl.zfaw79
Anopheles gambiae	-5.566653	9.299306	Unclassified	GBIF: https://doi.org/10.15468/dl.zfaw79
Anopheles gambiae	-5.56664	9.299307	Unclassified	GBIF: https://doi.org/10.15468/dl.zfaw79
Anopheles gambiae	-5.565804	9.393049	Unclassified	GBIF: https://doi.org/10.15468/dl.zfaw79
Anopheles gambiae	-5.5658	9.393036	Unclassified	GBIF: https://doi.org/10.15468/dl.zfaw79
Anopheles gambiae	-5.565791	9.393012	Unclassified	GBIF: https://doi.org/10.15468/dl.zfaw79
Anopheles gambiae	-5.565379	9.392026	Unclassified	GBIF: https://doi.org/10.15468/dl.zfaw79
Anopheles gambiae	-5.565354	9.392018	Unclassified	GBIF: https://doi.org/10.15468/dl.zfaw79
Anopheles gambiae	-5.565347	9.392012	Unclassified	GBIF: https://doi.org/10.15468/dl.zfaw79
Anopheles gambiae	-5.549452	9.271673	Unclassified	GBIF: https://doi.org/10.15468/dl.zfaw79
Anopheles gambiae	-5.549444	9.271675	Unclassified	GBIF: https://doi.org/10.15468/dl.zfaw79
Anopheles gambiae	-5.549419	9.271672	Unclassified	GBIF: https://doi.org/10.15468/dl.zfaw79
Anopheles gambiae	-5.549406	9.271693	Unclassified	GBIF: https://doi.org/10.15468/dl.zfaw79
Anopheles gambiae	-5.549253	9.272441	Unclassified	GBIF: https://doi.org/10.15468/dl.zfaw79
Anopheles gambiae	-5.549252	9.272499	Unclassified	GBIF: https://doi.org/10.15468/dl.zfaw79
Anopheles gambiae	-5.549241	9.272438	Unclassified	GBIF: https://doi.org/10.15468/dl.zfaw79
Anopheles gambiae	-5.54882	9.271236	Unclassified	GBIF: https://doi.org/10.15468/dl.zfaw79
Anopheles gambiae	-5.548806	9.271249	Unclassified	GBIF: https://doi.org/10.15468/dl.zfaw79
Anopheles gambiae	-5.548406	9.271905	Unclassified	GBIF: https://doi.org/10.15468/dl.zfaw79
Anopheles gambiae	-5.548401	9.271929	Unclassified	GBIF: https://doi.org/10.15468/dl.zfaw79
Anopheles gambiae	-5.548395	9.271932	Unclassified	GBIF: https://doi.org/10.15468/dl.zfaw79
Anopheles gambiae	-5.54838	9.271931	Unclassified	GBIF: https://doi.org/10.15468/dl.zfaw79
Anopheles gambiae	-5.536304	9.281444	Unclassified	GBIF: https://doi.org/10.15468/dl.zfaw79

Anopheles gambiae	-5.536284	9.281451	Unclassified	GBIF: https://doi.org/10.15468/dl.zfaw79
Anopheles gambiae	-5.536265	9.28146	Unclassified	GBIF: https://doi.org/10.15468/dl.zfaw79
Anopheles gambiae	-5.536258	9.281447	Unclassified	GBIF: https://doi.org/10.15468/dl.zfaw79
Anopheles gambiae	-5.53589	9.282552	Unclassified	GBIF: https://doi.org/10.15468/dl.zfaw79
Anopheles gambiae	-5.535889	9.282598	Unclassified	GBIF: https://doi.org/10.15468/dl.zfaw79
Anopheles gambiae	-5.535873	9.282567	Unclassified	GBIF: https://doi.org/10.15468/dl.zfaw79
Anopheles gambiae	-5.53587	9.282604	Unclassified	GBIF: https://doi.org/10.15468/dl.zfaw79
Anopheles gambiae	-5.535867	9.282582	Unclassified	GBIF: https://doi.org/10.15468/dl.zfaw79
Anopheles gambiae	-5.53583	9.282585	Unclassified	GBIF: https://doi.org/10.15468/dl.zfaw79
Anopheles gambiae	-5.53528	9.281796	Unclassified	GBIF: https://doi.org/10.15468/dl.zfaw79
Anopheles gambiae	-5.53527	9.281792	Unclassified	GBIF: https://doi.org/10.15468/dl.zfaw79
Anopheles gambiae	-5.535249	9.281816	Unclassified	GBIF: https://doi.org/10.15468/dl.zfaw79
Anopheles gambiae	-5.535228	9.281804	Unclassified	GBIF: https://doi.org/10.15468/dl.zfaw79
Anopheles gambiae	-5.534783	9.282717	Unclassified	GBIF: https://doi.org/10.15468/dl.zfaw79
Anopheles gambiae	-5.534768	9.282752	Unclassified	GBIF: https://doi.org/10.15468/dl.zfaw79
Anopheles gambiae	-5.53476	9.28275	Unclassified	GBIF: https://doi.org/10.15468/dl.zfaw79
Anopheles gambiae	-5.534754	9.282776	Unclassified	GBIF: https://doi.org/10.15468/dl.zfaw79
Anopheles gambiae	-5.525526	9.288469	Unclassified	GBIF: https://doi.org/10.15468/dl.zfaw79
Anopheles gambiae	-5.523949	9.289397	Unclassified	GBIF: https://doi.org/10.15468/dl.zfaw79
Anopheles gambiae	-5.523586	9.287433	Unclassified	GBIF: https://doi.org/10.15468/dl.zfaw79
Anopheles gambiae	-5.523485	9.288249	Unclassified	GBIF: https://doi.org/10.15468/dl.zfaw79
Anopheles gambiae	-5.517241	9.484894	Unclassified	GBIF: https://doi.org/10.15468/dl.zfaw79
Anopheles gambiae	-5.517052	9.484694	Unclassified	GBIF: https://doi.org/10.15468/dl.zfaw79
Anopheles gambiae	-5.517045	9.484642	Unclassified	GBIF: https://doi.org/10.15468/dl.zfaw79
Anopheles gambiae	-5.516949	9.484657	Unclassified	GBIF: https://doi.org/10.15468/dl.zfaw79
Anopheles gambiae	-5.516703	9.484562	Unclassified	GBIF: https://doi.org/10.15468/dl.zfaw79
Anopheles gambiae	-5.516694	9.484588	Unclassified	GBIF: https://doi.org/10.15468/dl.zfaw79
Anopheles gambiae	-5.516694	9.484635	Unclassified	GBIF: https://doi.org/10.15468/dl.zfaw79
Anopheles gambiae	-5.51667	13	Unclassified	GBIF: https://doi.org/10.15468/dl.zfaw79
Anopheles gambiae	-5.51667	9.36667	Unclassified	GBIF: https://doi.org/10.15468/dl.zfaw79
Anopheles gambiae	-5.516597	9.486687	Unclassified	GBIF: https://doi.org/10.15468/dl.zfaw79
Anopheles gambiae	-5.516588	9.486683	Unclassified	GBIF: https://doi.org/10.15468/dl.zfaw79
Anopheles gambiae	-5.516582	9.486688	Unclassified	GBIF: https://doi.org/10.15468/dl.zfaw79
Anopheles gambiae	-5.516565	9.486682	Unclassified	GBIF: https://doi.org/10.15468/dl.zfaw79
Anopheles gambiae	-5.516431	9.485977	Unclassified	GBIF: https://doi.org/10.15468/dl.zfaw79
Anopheles gambiae	-5.516398	9.48578	Unclassified	GBIF: https://doi.org/10.15468/dl.zfaw79
Anopheles gambiae	-5.516389	9.485852	Unclassified	GBIF: https://doi.org/10.15468/dl.zfaw79
Anopheles gambiae	-5.516367	9.485807	Unclassified	GBIF: https://doi.org/10.15468/dl.zfaw79
Anopheles gambiae	-5.516364	9.485884	Unclassified	GBIF: https://doi.org/10.15468/dl.zfaw79
Anopheles gambiae	-5.515894	9.253188	Unclassified	GBIF: https://doi.org/10.15468/dl.zfaw79
Anopheles gambiae	-5.515879	9.253172	Unclassified	GBIF: https://doi.org/10.15468/dl.zfaw79
Anopheles gambiae	-5.515872	9.253205	Unclassified	GBIF: https://doi.org/10.15468/dl.zfaw79

Anopheles gambiae	-5.515652	9.254035	Unclassified	GBIF: https://doi.org/10.15468/dl.zfaw79
Anopheles gambiae	-5.515632	9.254004	Unclassified	GBIF: https://doi.org/10.15468/dl.zfaw79
Anopheles gambiae	-5.514449	9.440538	Unclassified	GBIF: https://doi.org/10.15468/dl.zfaw79
Anopheles gambiae	-5.514441	9.440569	Unclassified	GBIF: https://doi.org/10.15468/dl.zfaw79
Anopheles gambiae	-5.514426	9.440584	Unclassified	GBIF: https://doi.org/10.15468/dl.zfaw79
Anopheles gambiae	-5.514424	9.440589	Unclassified	GBIF: https://doi.org/10.15468/dl.zfaw79
Anopheles gambiae	-5.514423	9.440627	Unclassified	GBIF: https://doi.org/10.15468/dl.zfaw79
Anopheles gambiae	-5.514406	9.440621	Unclassified	GBIF: https://doi.org/10.15468/dl.zfaw79
Anopheles gambiae	-5.514392	9.253341	Unclassified	GBIF: https://doi.org/10.15468/dl.zfaw79
Anopheles gambiae	-5.514392	9.253333	Unclassified	GBIF: https://doi.org/10.15468/dl.zfaw79
Anopheles gambiae	-5.514386	9.253341	Unclassified	GBIF: https://doi.org/10.15468/dl.zfaw79
Anopheles gambiae	-5.51438	9.253368	Unclassified	GBIF: https://doi.org/10.15468/dl.zfaw79
Anopheles gambiae	-5.513976	9.440286	Unclassified	GBIF: https://doi.org/10.15468/dl.zfaw79
Anopheles gambiae	-5.513976	9.440288	Unclassified	GBIF: https://doi.org/10.15468/dl.zfaw79
Anopheles gambiae	-5.513951	9.440292	Unclassified	GBIF: https://doi.org/10.15468/dl.zfaw79
Anopheles gambiae	-5.513946	9.440321	Unclassified	GBIF: https://doi.org/10.15468/dl.zfaw79
Anopheles gambiae	-5.513939	9.440282	Unclassified	GBIF: https://doi.org/10.15468/dl.zfaw79
Anopheles gambiae	-5.513919	9.440294	Unclassified	GBIF: https://doi.org/10.15468/dl.zfaw79
Anopheles gambiae	-5.513528	9.440772	Unclassified	GBIF: https://doi.org/10.15468/dl.zfaw79
Anopheles gambiae	-5.513524	9.440765	Unclassified	GBIF: https://doi.org/10.15468/dl.zfaw79
Anopheles gambiae	-5.513519	9.440745	Unclassified	GBIF: https://doi.org/10.15468/dl.zfaw79
Anopheles gambiae	-5.513511	9.440765	Unclassified	GBIF: https://doi.org/10.15468/dl.zfaw79
Anopheles gambiae	-5.513508	9.440792	Unclassified	GBIF: https://doi.org/10.15468/dl.zfaw79
Anopheles gambiae	-5.513502	9.440762	Unclassified	GBIF: https://doi.org/10.15468/dl.zfaw79
Anopheles gambiae	-5.513464	9.253545	Unclassified	GBIF: https://doi.org/10.15468/dl.zfaw79
Anopheles gambiae	-5.513457	9.253561	Unclassified	GBIF: https://doi.org/10.15468/dl.zfaw79
Anopheles gambiae	-5.513295	9.391305	Unclassified	GBIF: https://doi.org/10.15468/dl.zfaw79
Anopheles gambiae	-5.513281	9.391278	Unclassified	GBIF: https://doi.org/10.15468/dl.zfaw79
Anopheles gambiae	-5.513059	9.440866	Unclassified	GBIF: https://doi.org/10.15468/dl.zfaw79
Anopheles gambiae	-5.51305	9.440883	Unclassified	GBIF: https://doi.org/10.15468/dl.zfaw79
Anopheles gambiae	-5.51303	9.44086	Unclassified	GBIF: https://doi.org/10.15468/dl.zfaw79
Anopheles gambiae	-5.513028	9.440838	Unclassified	GBIF: https://doi.org/10.15468/dl.zfaw79
Anopheles gambiae	-5.513024	9.440864	Unclassified	GBIF: https://doi.org/10.15468/dl.zfaw79
Anopheles gambiae	-5.512982	9.440851	Unclassified	GBIF: https://doi.org/10.15468/dl.zfaw79
Anopheles gambiae	-5.51273	9.389976	Unclassified	GBIF: https://doi.org/10.15468/dl.zfaw79
Anopheles gambiae	-5.512722	9.390073	Unclassified	GBIF: https://doi.org/10.15468/dl.zfaw79
Anopheles gambiae	-5.512442	9.391955	Unclassified	GBIF: https://doi.org/10.15468/dl.zfaw79
Anopheles gambiae	-5.512426	9.39194	Unclassified	GBIF: https://doi.org/10.15468/dl.zfaw79
Anopheles gambiae	-5.512126	9.392539	Unclassified	GBIF: https://doi.org/10.15468/dl.zfaw79
Anopheles gambiae	-5.500125	8.989074	Unclassified	GBIF: https://doi.org/10.15468/dl.zfaw79
Anopheles gambiae	-5.500097	8.988988	Unclassified	GBIF: https://doi.org/10.15468/dl.zfaw79
Anopheles gambiae	-5.500095	8.98903	Unclassified	GBIF: https://doi.org/10.15468/dl.zfaw79

Anopheles gambiae	-5.500091	8.98903	Unclassified	GBIF: https://doi.org/10.15468/dl.zfaw79
Anopheles gambiae	-5.500079	8.989065	Unclassified	GBIF: https://doi.org/10.15468/dl.zfaw79
Anopheles gambiae	-5.5	10.83333	Unclassified	GBIF: https://doi.org/10.15468/dl.zfaw79
Anopheles gambiae	-5.49994	8.988825	Unclassified	GBIF: https://doi.org/10.15468/dl.zfaw79
Anopheles gambiae	-5.499925	8.988805	Unclassified	GBIF: https://doi.org/10.15468/dl.zfaw79
Anopheles gambiae	-5.499924	8.988782	Unclassified	GBIF: https://doi.org/10.15468/dl.zfaw79
Anopheles gambiae	-5.499923	8.98879	Unclassified	GBIF: https://doi.org/10.15468/dl.zfaw79
Anopheles gambiae	-5.49992	8.988784	Unclassified	GBIF: https://doi.org/10.15468/dl.zfaw79
Anopheles gambiae	-5.4999	8.988799	Unclassified	GBIF: https://doi.org/10.15468/dl.zfaw79
Anopheles gambiae	-5.49962	8.989709	Unclassified	GBIF: https://doi.org/10.15468/dl.zfaw79
Anopheles gambiae	-5.499619	8.989675	Unclassified	GBIF: https://doi.org/10.15468/dl.zfaw79
Anopheles gambiae	-5.499601	8.989688	Unclassified	GBIF: https://doi.org/10.15468/dl.zfaw79
Anopheles gambiae	-5.499599	8.989674	Unclassified	GBIF: https://doi.org/10.15468/dl.zfaw79
Anopheles gambiae	-5.499586	8.989688	Unclassified	GBIF: https://doi.org/10.15468/dl.zfaw79
Anopheles gambiae	-5.499524	8.989301	Unclassified	GBIF: https://doi.org/10.15468/dl.zfaw79
Anopheles gambiae	-5.499459	9.394624	Unclassified	GBIF: https://doi.org/10.15468/dl.zfaw79
Anopheles gambiae	-5.499404	9.394611	Unclassified	GBIF: https://doi.org/10.15468/dl.zfaw79
Anopheles gambiae	-5.499375	9.39459	Unclassified	GBIF: https://doi.org/10.15468/dl.zfaw79
Anopheles gambiae	-5.49935	8.989059	Unclassified	GBIF: https://doi.org/10.15468/dl.zfaw79
Anopheles gambiae	-5.499347	8.989034	Unclassified	GBIF: https://doi.org/10.15468/dl.zfaw79
Anopheles gambiae	-5.499346	8.989075	Unclassified	GBIF: https://doi.org/10.15468/dl.zfaw79
Anopheles gambiae	-5.499345	8.989057	Unclassified	GBIF: https://doi.org/10.15468/dl.zfaw79
Anopheles gambiae	-5.499338	8.989101	Unclassified	GBIF: https://doi.org/10.15468/dl.zfaw79
Anopheles gambiae	-5.499338	8.989107	Unclassified	GBIF: https://doi.org/10.15468/dl.zfaw79
Anopheles gambiae	-5.499126	9.394285	Unclassified	GBIF: https://doi.org/10.15468/dl.zfaw79
Anopheles gambiae	-5.499	9.394457	Unclassified	GBIF: https://doi.org/10.15468/dl.zfaw79
Anopheles gambiae	-5.49897	9.394478	Unclassified	GBIF: https://doi.org/10.15468/dl.zfaw79
Anopheles gambiae	-5.498792	9.394007	Unclassified	GBIF: https://doi.org/10.15468/dl.zfaw79
Anopheles gambiae	-5.498631	9.394096	Unclassified	GBIF: https://doi.org/10.15468/dl.zfaw79
Anopheles gambiae	-5.498557	9.394168	Unclassified	GBIF: https://doi.org/10.15468/dl.zfaw79
Anopheles gambiae	-5.488259	9.504935	Unclassified	GBIF: https://doi.org/10.15468/dl.zfaw79
Anopheles gambiae	-5.488252	9.504933	Unclassified	GBIF: https://doi.org/10.15468/dl.zfaw79
Anopheles gambiae	-5.488252	9.504971	Unclassified	GBIF: https://doi.org/10.15468/dl.zfaw79
Anopheles gambiae	-5.48824	9.50491	Unclassified	GBIF: https://doi.org/10.15468/dl.zfaw79
Anopheles gambiae	-5.48811	9.504183	Unclassified	GBIF: https://doi.org/10.15468/dl.zfaw79
Anopheles gambiae	-5.488097	9.504177	Unclassified	GBIF: https://doi.org/10.15468/dl.zfaw79
Anopheles gambiae	-5.488093	9.504188	Unclassified	GBIF: https://doi.org/10.15468/dl.zfaw79
Anopheles gambiae	-5.488083	9.504213	Unclassified	GBIF: https://doi.org/10.15468/dl.zfaw79
Anopheles gambiae	-5.488082	9.504184	Unclassified	GBIF: https://doi.org/10.15468/dl.zfaw79
Anopheles gambiae	-5.488043	9.50415	Unclassified	GBIF: https://doi.org/10.15468/dl.zfaw79
Anopheles gambiae	-5.488043	9.506759	Unclassified	GBIF: https://doi.org/10.15468/dl.zfaw79
Anopheles gambiae	-5.488017	9.506761	Unclassified	GBIF: https://doi.org/10.15468/dl.zfaw79

Anopheles gambiae	-5.488006	9.506775	Unclassified	GBIF: https://doi.org/10.15468/dl.zfaw79
Anopheles gambiae	-5.488005	9.506761	Unclassified	GBIF: https://doi.org/10.15468/dl.zfaw79
Anopheles gambiae	-5.487461	9.03183	Unclassified	GBIF: https://doi.org/10.15468/dl.zfaw79
Anopheles gambiae	-5.487457	9.031848	Unclassified	GBIF: https://doi.org/10.15468/dl.zfaw79
Anopheles gambiae	-5.487454	9.031845	Unclassified	GBIF: https://doi.org/10.15468/dl.zfaw79
Anopheles gambiae	-5.487446	9.031844	Unclassified	GBIF: https://doi.org/10.15468/dl.zfaw79
Anopheles gambiae	-5.487444	9.031841	Unclassified	GBIF: https://doi.org/10.15468/dl.zfaw79
Anopheles gambiae	-5.487397	9.031841	Unclassified	GBIF: https://doi.org/10.15468/dl.zfaw79
Anopheles gambiae	-5.487037	9.505247	Unclassified	GBIF: https://doi.org/10.15468/dl.zfaw79
Anopheles gambiae	-5.487017	9.505225	Unclassified	GBIF: https://doi.org/10.15468/dl.zfaw79
Anopheles gambiae	-5.487016	9.505217	Unclassified	GBIF: https://doi.org/10.15468/dl.zfaw79
Anopheles gambiae	-5.486658	9.033219	Unclassified	GBIF: https://doi.org/10.15468/dl.zfaw79
Anopheles gambiae	-5.486652	9.03326	Unclassified	GBIF: https://doi.org/10.15468/dl.zfaw79
Anopheles gambiae	-5.486646	9.033238	Unclassified	GBIF: https://doi.org/10.15468/dl.zfaw79
Anopheles gambiae	-5.486642	9.033241	Unclassified	GBIF: https://doi.org/10.15468/dl.zfaw79
Anopheles gambiae	-5.486623	9.033267	Unclassified	GBIF: https://doi.org/10.15468/dl.zfaw79
Anopheles gambiae	-5.486405	9.03115	Unclassified	GBIF: https://doi.org/10.15468/dl.zfaw79
Anopheles gambiae	-5.486383	9.031171	Unclassified	GBIF: https://doi.org/10.15468/dl.zfaw79
Anopheles gambiae	-5.486374	9.0312	Unclassified	GBIF: https://doi.org/10.15468/dl.zfaw79
Anopheles gambiae	-5.486373	9.031158	Unclassified	GBIF: https://doi.org/10.15468/dl.zfaw79
Anopheles gambiae	-5.486364	9.031134	Unclassified	GBIF: https://doi.org/10.15468/dl.zfaw79
Anopheles gambiae	-5.486363	9.031165	Unclassified	GBIF: https://doi.org/10.15468/dl.zfaw79
Anopheles gambiae	-5.486353	9.031134	Unclassified	GBIF: https://doi.org/10.15468/dl.zfaw79
Anopheles gambiae	-5.48625	9.032432	Unclassified	GBIF: https://doi.org/10.15468/dl.zfaw79
Anopheles gambiae	-5.486236	9.032424	Unclassified	GBIF: https://doi.org/10.15468/dl.zfaw79
Anopheles gambiae	-5.486232	9.032412	Unclassified	GBIF: https://doi.org/10.15468/dl.zfaw79
Anopheles gambiae	-5.486216	9.03242	Unclassified	GBIF: https://doi.org/10.15468/dl.zfaw79
Anopheles gambiae	-5.486212	9.032418	Unclassified	GBIF: https://doi.org/10.15468/dl.zfaw79
Anopheles gambiae	-5.486172	9.032396	Unclassified	GBIF: https://doi.org/10.15468/dl.zfaw79
Anopheles gambiae	-5.472067	9.449943	Unclassified	GBIF: https://doi.org/10.15468/dl.zfaw79
Anopheles gambiae	-5.472017	9.449983	Unclassified	GBIF: https://doi.org/10.15468/dl.zfaw79
Anopheles gambiae	-5.472013	9.44992	Unclassified	GBIF: https://doi.org/10.15468/dl.zfaw79
Anopheles gambiae	-5.472009	9.449934	Unclassified	GBIF: https://doi.org/10.15468/dl.zfaw79
Anopheles gambiae	-5.472007	9.449966	Unclassified	GBIF: https://doi.org/10.15468/dl.zfaw79
Anopheles gambiae	-5.471982	9.449938	Unclassified	GBIF: https://doi.org/10.15468/dl.zfaw79
Anopheles gambiae	-5.471982	9.449953	Unclassified	GBIF: https://doi.org/10.15468/dl.zfaw79
Anopheles gambiae	-5.471737	9.450453	Unclassified	GBIF: https://doi.org/10.15468/dl.zfaw79
Anopheles gambiae	-5.471727	9.450405	Unclassified	GBIF: https://doi.org/10.15468/dl.zfaw79
Anopheles gambiae	-5.471699	9.450409	Unclassified	GBIF: https://doi.org/10.15468/dl.zfaw79
Anopheles gambiae	-5.471699	9.450436	Unclassified	GBIF: https://doi.org/10.15468/dl.zfaw79
Anopheles gambiae	-5.471686	9.450424	Unclassified	GBIF: https://doi.org/10.15468/dl.zfaw79
Anopheles gambiae	-5.471684	9.450406	Unclassified	GBIF: https://doi.org/10.15468/dl.zfaw79

Anopheles gambiae	-5.471678	9.450427	Unclassified	GBIF: https://doi.org/10.15468/dl.zfaw79
Anopheles gambiae	-5.470364	9.44996	Unclassified	GBIF: https://doi.org/10.15468/dl.zfaw79
Anopheles gambiae	-5.470317	9.449936	Unclassified	GBIF: https://doi.org/10.15468/dl.zfaw79
Anopheles gambiae	-5.470309	9.449927	Unclassified	GBIF: https://doi.org/10.15468/dl.zfaw79
Anopheles gambiae	-5.4703	9.449943	Unclassified	GBIF: https://doi.org/10.15468/dl.zfaw79
Anopheles gambiae	-5.470295	9.449947	Unclassified	GBIF: https://doi.org/10.15468/dl.zfaw79
Anopheles gambiae	-5.47028	9.449922	Unclassified	GBIF: https://doi.org/10.15468/dl.zfaw79
Anopheles gambiae	-5.470268	9.449965	Unclassified	GBIF: https://doi.org/10.15468/dl.zfaw79
Anopheles gambiae	-5.470267	9.449943	Unclassified	GBIF: https://doi.org/10.15468/dl.zfaw79
Anopheles gambiae	-5.470129	9.448957	Unclassified	GBIF: https://doi.org/10.15468/dl.zfaw79
Anopheles gambiae	-5.470124	9.448971	Unclassified	GBIF: https://doi.org/10.15468/dl.zfaw79
Anopheles gambiae	-5.470122	9.448938	Unclassified	GBIF: https://doi.org/10.15468/dl.zfaw79
Anopheles gambiae	-5.470119	9.449011	Unclassified	GBIF: https://doi.org/10.15468/dl.zfaw79
Anopheles gambiae	-5.470118	9.44895	Unclassified	GBIF: https://doi.org/10.15468/dl.zfaw79
Anopheles gambiae	-5.470118	9.448981	Unclassified	GBIF: https://doi.org/10.15468/dl.zfaw79
Anopheles gambiae	-5.470115	9.448979	Unclassified	GBIF: https://doi.org/10.15468/dl.zfaw79
Anopheles gambiae	-5.4	14.1	Unclassified	GBIF: https://doi.org/10.15468/dl.zfaw79
Anopheles gambiae	-5.36667	11.05	Unclassified	GBIF: https://doi.org/10.15468/dl.zfaw79
Anopheles gambiae	-5.3	13.11667	Unclassified	GBIF: https://doi.org/10.15468/dl.zfaw79
Anopheles gambiae	-5.16667	9.5	Unclassified	GBIF: https://doi.org/10.15468/dl.zfaw79
Anopheles gambiae	-5.15	12.65	Unclassified	GBIF: https://doi.org/10.15468/dl.zfaw79
Anopheles gambiae	-5.03333	12.71667	Unclassified	GBIF: https://doi.org/10.15468/dl.zfaw79
Anopheles gambiae	-5.03333	12.26667	Unclassified	GBIF: https://doi.org/10.15468/dl.zfaw79
Anopheles gambiae	-5	7.7	Unclassified	GBIF: https://doi.org/10.15468/dl.zfaw79
Anopheles gambiae	-4.96667	11.16667	Unclassified	GBIF: https://doi.org/10.15468/dl.zfaw79
Anopheles gambiae	-4.95	8.16667	Unclassified	GBIF: https://doi.org/10.15468/dl.zfaw79
Anopheles gambiae	-4.93333	11.08333	Unclassified	GBIF: https://doi.org/10.15468/dl.zfaw79
Anopheles gambiae	-4.93333	13.26667	Unclassified	GBIF: https://doi.org/10.15468/dl.zfaw79
Anopheles gambiae	-4.91667	10.28333	Unclassified	GBIF: https://doi.org/10.15468/dl.zfaw79
Anopheles gambiae	-4.91667	13.28333	Unclassified	GBIF: https://doi.org/10.15468/dl.zfaw79
Anopheles gambiae	-4.88333	13.23333	Unclassified	GBIF: https://doi.org/10.15468/dl.zfaw79
Anopheles gambiae	-4.86667	9.21667	Unclassified	GBIF: https://doi.org/10.15468/dl.zfaw79
Anopheles gambiae	-4.85	13.21667	Unclassified	GBIF: https://doi.org/10.15468/dl.zfaw79
Anopheles gambiae	-4.76667	6.71667	Unclassified	GBIF: https://doi.org/10.15468/dl.zfaw79
Anopheles gambiae	-4.75	10.6	Unclassified	GBIF: https://doi.org/10.15468/dl.zfaw79
Anopheles gambiae	-4.68333	11.7	Unclassified	GBIF: https://doi.org/10.15468/dl.zfaw79
Anopheles gambiae	-4.65	6.35	Unclassified	GBIF: https://doi.org/10.15468/dl.zfaw79
Anopheles gambiae	-4.55	11.58333	Unclassified	GBIF: https://doi.org/10.15468/dl.zfaw79
Anopheles gambiae	-4.52305	11.2352	Unclassified	GBIF: https://doi.org/10.15468/dl.zfaw79
Anopheles gambiae	-4.52299	11.2351	Unclassified	GBIF: https://doi.org/10.15468/dl.zfaw79
Anopheles gambiae	-4.52115	11.2357	Unclassified	GBIF: https://doi.org/10.15468/dl.zfaw79
Anopheles gambiae	-4.52113	11.2385	Unclassified	GBIF: https://doi.org/10.15468/dl.zfaw79

Anopheles gambiae	-4.52013	11.2372	Unclassified	GBIF: https://doi.org/10.15468/dl.zfaw79
Anopheles gambiae	-4.52002	11.236	Unclassified	GBIF: https://doi.org/10.15468/dl.zfaw79
Anopheles gambiae	-4.51963	11.2373	Unclassified	GBIF: https://doi.org/10.15468/dl.zfaw79
Anopheles gambiae	-4.51739	11.2364	Unclassified	GBIF: https://doi.org/10.15468/dl.zfaw79
Anopheles gambiae	-4.51733	11.2363	Unclassified	GBIF: https://doi.org/10.15468/dl.zfaw79
Anopheles gambiae	-4.51684	11.236	Unclassified	GBIF: https://doi.org/10.15468/dl.zfaw79
Anopheles gambiae	-4.51683	11.236	Unclassified	GBIF: https://doi.org/10.15468/dl.zfaw79
Anopheles gambiae	-4.51667	11.88333	Unclassified	GBIF: https://doi.org/10.15468/dl.zfaw79
Anopheles gambiae	-4.51566	11.2362	Unclassified	GBIF: https://doi.org/10.15468/dl.zfaw79
Anopheles gambiae	-4.5144	11.2384	Unclassified	GBIF: https://doi.org/10.15468/dl.zfaw79
Anopheles gambiae	-4.5132	11.2366	Unclassified	GBIF: https://doi.org/10.15468/dl.zfaw79
Anopheles gambiae	-4.51156	11.2365	Unclassified	GBIF: https://doi.org/10.15468/dl.zfaw79
Anopheles gambiae	-4.51146	11.2364	Unclassified	GBIF: https://doi.org/10.15468/dl.zfaw79
Anopheles gambiae	-4.51102	11.2374	Unclassified	GBIF: https://doi.org/10.15468/dl.zfaw79
Anopheles gambiae	-4.50974	11.238	Unclassified	GBIF: https://doi.org/10.15468/dl.zfaw79
Anopheles gambiae	-4.50964	11.2379	Unclassified	GBIF: https://doi.org/10.15468/dl.zfaw79
Anopheles gambiae	-4.5096	11.2346	Unclassified	GBIF: https://doi.org/10.15468/dl.zfaw79
Anopheles gambiae	-4.50953	11.2379	Unclassified	GBIF: https://doi.org/10.15468/dl.zfaw79
Anopheles gambiae	-4.50953	11.2386	Unclassified	GBIF: https://doi.org/10.15468/dl.zfaw79
Anopheles gambiae	-4.50952	11.2384	Unclassified	GBIF: https://doi.org/10.15468/dl.zfaw79
Anopheles gambiae	-4.5092	11.2405	Unclassified	GBIF: https://doi.org/10.15468/dl.zfaw79
Anopheles gambiae	-4.50909	11.2382	Unclassified	GBIF: https://doi.org/10.15468/dl.zfaw79
Anopheles gambiae	-4.5025	11.2398	Unclassified	GBIF: https://doi.org/10.15468/dl.zfaw79
Anopheles gambiae	-4.50185	11.2397	Unclassified	GBIF: https://doi.org/10.15468/dl.zfaw79
Anopheles gambiae	-4.50128	11.2367	Unclassified	GBIF: https://doi.org/10.15468/dl.zfaw79
Anopheles gambiae	-4.5006	11.239	Unclassified	GBIF: https://doi.org/10.15468/dl.zfaw79
Anopheles gambiae	-4.50037	11.236	Unclassified	GBIF: https://doi.org/10.15468/dl.zfaw79
Anopheles gambiae	-4.50015	11.2373	Unclassified	GBIF: https://doi.org/10.15468/dl.zfaw79
Anopheles gambiae	-4.50008	11.2374	Unclassified	GBIF: https://doi.org/10.15468/dl.zfaw79
Anopheles gambiae	-4.5	6.88333	Unclassified	GBIF: https://doi.org/10.15468/dl.zfaw79
Anopheles gambiae	-4.49909	11.2396	Unclassified	GBIF: https://doi.org/10.15468/dl.zfaw79
Anopheles gambiae	-4.49744	11.2408	Unclassified	GBIF: https://doi.org/10.15468/dl.zfaw79
Anopheles gambiae	-4.49743	11.2382	Unclassified	GBIF: https://doi.org/10.15468/dl.zfaw79
Anopheles gambiae	-4.49678	11.2427	Unclassified	GBIF: https://doi.org/10.15468/dl.zfaw79
Anopheles gambiae	-4.49528	11.2421	Unclassified	GBIF: https://doi.org/10.15468/dl.zfaw79
Anopheles gambiae	-4.49525	11.242	Unclassified	GBIF: https://doi.org/10.15468/dl.zfaw79
Anopheles gambiae	-4.483	11.2375	Unclassified	GBIF: https://doi.org/10.15468/dl.zfaw79
Anopheles gambiae	-4.48239	11.2372	Unclassified	GBIF: https://doi.org/10.15468/dl.zfaw79
Anopheles gambiae	-4.47924	11.2359	Unclassified	GBIF: https://doi.org/10.15468/dl.zfaw79
Anopheles gambiae	-4.47916	11.2314	Unclassified	GBIF: https://doi.org/10.15468/dl.zfaw79
Anopheles gambiae	-4.47907	11.236	Unclassified	GBIF: https://doi.org/10.15468/dl.zfaw79
Anopheles gambiae	-4.47788	11.235	Unclassified	GBIF: https://doi.org/10.15468/dl.zfaw79

Anopheles gambiae	-4.47732	11.2337	Unclassified	GBIF: https://doi.org/10.15468/dl.zfaw79
Anopheles gambiae	-4.47732	11.235	Unclassified	GBIF: https://doi.org/10.15468/dl.zfaw79
Anopheles gambiae	-4.47729	11.235	Unclassified	GBIF: https://doi.org/10.15468/dl.zfaw79
Anopheles gambiae	-4.4771	11.2348	Unclassified	GBIF: https://doi.org/10.15468/dl.zfaw79
Anopheles gambiae	-4.4769	11.2346	Unclassified	GBIF: https://doi.org/10.15468/dl.zfaw79
Anopheles gambiae	-4.47457	11.238	Unclassified	GBIF: https://doi.org/10.15468/dl.zfaw79
Anopheles gambiae	-4.4745	11.2379	Unclassified	GBIF: https://doi.org/10.15468/dl.zfaw79
Anopheles gambiae	-4.47448	11.2379	Unclassified	GBIF: https://doi.org/10.15468/dl.zfaw79
Anopheles gambiae	-4.47437	11.2379	Unclassified	GBIF: https://doi.org/10.15468/dl.zfaw79
Anopheles gambiae	-4.47401	11.2375	Unclassified	GBIF: https://doi.org/10.15468/dl.zfaw79
Anopheles gambiae	-4.47319	11.2356	Unclassified	GBIF: https://doi.org/10.15468/dl.zfaw79
Anopheles gambiae	-4.47318	11.2357	Unclassified	GBIF: https://doi.org/10.15468/dl.zfaw79
Anopheles gambiae	-4.47317	11.2358	Unclassified	GBIF: https://doi.org/10.15468/dl.zfaw79
Anopheles gambiae	-4.47312	11.2353	Unclassified	GBIF: https://doi.org/10.15468/dl.zfaw79
Anopheles gambiae	-4.47308	11.2353	Unclassified	GBIF: https://doi.org/10.15468/dl.zfaw79
Anopheles gambiae	-4.47288	11.2366	Unclassified	GBIF: https://doi.org/10.15468/dl.zfaw79
Anopheles gambiae	-4.4725	11.2347	Unclassified	GBIF: https://doi.org/10.15468/dl.zfaw79
Anopheles gambiae	-4.47247	11.2347	Unclassified	GBIF: https://doi.org/10.15468/dl.zfaw79
Anopheles gambiae	-4.46667	11.26667	Unclassified	GBIF: https://doi.org/10.15468/dl.zfaw79
Anopheles gambiae	-4.43333	7.11667	Unclassified	GBIF: https://doi.org/10.15468/dl.zfaw79
Anopheles gambiae	-4.41667	11.38333	Unclassified	GBIF: https://doi.org/10.15468/dl.zfaw79
Anopheles gambiae	-4.41667	8.86667	Unclassified	GBIF: https://doi.org/10.15468/dl.zfaw79
Anopheles gambiae	-4.38333	11.38333	Unclassified	GBIF: https://doi.org/10.15468/dl.zfaw79
Anopheles gambiae	-4.33333	13.86667	Unclassified	GBIF: https://doi.org/10.15468/dl.zfaw79
Anopheles gambiae	-4.31667	11.31667	Unclassified	GBIF: https://doi.org/10.15468/dl.zfaw79
Anopheles gambiae	-4.31	11.16	Unclassified	GBIF: https://doi.org/10.15468/dl.zfaw79
Anopheles gambiae	-4.3	11.18333	Unclassified	GBIF: https://doi.org/10.15468/dl.zfaw79
Anopheles gambiae	-4.26667	14.51667	Unclassified	GBIF: https://doi.org/10.15468/dl.zfaw79
Anopheles gambiae	-4.25	11.48333	Unclassified	GBIF: https://doi.org/10.15468/dl.zfaw79
Anopheles gambiae	-4.23333	11.15	Unclassified	GBIF: https://doi.org/10.15468/dl.zfaw79
Anopheles gambiae	-4.2	11.13333	Unclassified	GBIF: https://doi.org/10.15468/dl.zfaw79
Anopheles gambiae	-4.18333	11.18333	Unclassified	GBIF: https://doi.org/10.15468/dl.zfaw79
Anopheles gambiae	-4.16667	11.15	Unclassified	GBIF: https://doi.org/10.15468/dl.zfaw79
Anopheles gambiae	-4.15	12.16667	Unclassified	GBIF: https://doi.org/10.15468/dl.zfaw79
Anopheles gambiae	-4.15	8.51667	Unclassified	GBIF: https://doi.org/10.15468/dl.zfaw79
Anopheles gambiae	-4.11667	14.55	Unclassified	GBIF: https://doi.org/10.15468/dl.zfaw79
Anopheles gambiae	-4.11667	5.36667	Unclassified	GBIF: https://doi.org/10.15468/dl.zfaw79
Anopheles gambiae	-4.1	8.05	Unclassified	GBIF: https://doi.org/10.15468/dl.zfaw79
Anopheles gambiae	-4.06	11.02	Unclassified	GBIF: https://doi.org/10.15468/dl.zfaw79
Anopheles gambiae	-4.05	11.06667	Unclassified	GBIF: https://doi.org/10.15468/dl.zfaw79
Anopheles gambiae	-4.01667	5.31667	Unclassified	GBIF: https://doi.org/10.15468/dl.zfaw79
Anopheles gambiae	-4	11.51667	Unclassified	GBIF: https://doi.org/10.15468/dl.zfaw79

Anopheles gambiae	-4	11.65	Unclassified	GBIF: https://doi.org/10.15468/dl.zfaw79
Anopheles gambiae	-3.93333	8.61667	Unclassified	GBIF: https://doi.org/10.15468/dl.zfaw79
Anopheles gambiae	-3.86667	10.76667	Unclassified	GBIF: https://doi.org/10.15468/dl.zfaw79
Anopheles gambiae	-3.86667	10.86667	Unclassified	GBIF: https://doi.org/10.15468/dl.zfaw79
Anopheles gambiae	-3.86667	11.23333	Unclassified	GBIF: https://doi.org/10.15468/dl.zfaw79
Anopheles gambiae	-3.81667	11.7	Unclassified	GBIF: https://doi.org/10.15468/dl.zfaw79
Anopheles gambiae	-3.78333	14.41667	Unclassified	GBIF: https://doi.org/10.15468/dl.zfaw79
Anopheles gambiae	-3.75	14.18333	Unclassified	GBIF: https://doi.org/10.15468/dl.zfaw79
Anopheles gambiae	-3.75	9.16667	Unclassified	GBIF: https://doi.org/10.15468/dl.zfaw79
Anopheles gambiae	-3.66667	6.58333	Unclassified	GBIF: https://doi.org/10.15468/dl.zfaw79
Anopheles gambiae	-3.63333	11.3	Unclassified	GBIF: https://doi.org/10.15468/dl.zfaw79
Anopheles gambiae	-3.61667	14.41667	Unclassified	GBIF: https://doi.org/10.15468/dl.zfaw79
Anopheles gambiae	-3.6	14.33333	Unclassified	GBIF: https://doi.org/10.15468/dl.zfaw79
Anopheles gambiae	-3.56667	14.4	Unclassified	GBIF: https://doi.org/10.15468/dl.zfaw79
Anopheles gambiae	-3.553674	10.706287	Unclassified	GBIF: https://doi.org/10.15468/dl.zfaw79
Anopheles gambiae	-3.553645	10.706175	Unclassified	GBIF: https://doi.org/10.15468/dl.zfaw79
Anopheles gambiae	-3.553343	10.70501	Unclassified	GBIF: https://doi.org/10.15468/dl.zfaw79
Anopheles gambiae	-3.553273	10.705211	Unclassified	GBIF: https://doi.org/10.15468/dl.zfaw79
Anopheles gambiae	-3.553114	10.704534	Unclassified	GBIF: https://doi.org/10.15468/dl.zfaw79
Anopheles gambiae	-3.553094	10.704497	Unclassified	GBIF: https://doi.org/10.15468/dl.zfaw79
Anopheles gambiae	-3.552666	10.705635	Unclassified	GBIF: https://doi.org/10.15468/dl.zfaw79
Anopheles gambiae	-3.552627	10.705975	Unclassified	GBIF: https://doi.org/10.15468/dl.zfaw79
Anopheles gambiae	-3.55	11.43333	Unclassified	GBIF: https://doi.org/10.15468/dl.zfaw79
Anopheles gambiae	-3.525304	10.694035	Unclassified	GBIF: https://doi.org/10.15468/dl.zfaw79
Anopheles gambiae	-3.525258	10.694028	Unclassified	GBIF: https://doi.org/10.15468/dl.zfaw79
Anopheles gambiae	-3.523798	10.694465	Unclassified	GBIF: https://doi.org/10.15468/dl.zfaw79
Anopheles gambiae	-3.523797	10.69716	Unclassified	GBIF: https://doi.org/10.15468/dl.zfaw79
Anopheles gambiae	-3.523778	10.69473	Unclassified	GBIF: https://doi.org/10.15468/dl.zfaw79
Anopheles gambiae	-3.523777	10.694708	Unclassified	GBIF: https://doi.org/10.15468/dl.zfaw79
Anopheles gambiae	-3.52369	10.694744	Unclassified	GBIF: https://doi.org/10.15468/dl.zfaw79
Anopheles gambiae	-3.52292	10.695174	Unclassified	GBIF: https://doi.org/10.15468/dl.zfaw79
Anopheles gambiae	-3.52	11.49	Unclassified	GBIF: https://doi.org/10.15468/dl.zfaw79
Anopheles gambiae	-3.51667	11.13333	Unclassified	GBIF: https://doi.org/10.15468/dl.zfaw79
Anopheles gambiae	-3.487221	10.74687	Unclassified	GBIF: https://doi.org/10.15468/dl.zfaw79
Anopheles gambiae	-3.487121	10.746348	Unclassified	GBIF: https://doi.org/10.15468/dl.zfaw79
Anopheles gambiae	-3.486374	10.746505	Unclassified	GBIF: https://doi.org/10.15468/dl.zfaw79
Anopheles gambiae	-3.486374	10.74652	Unclassified	GBIF: https://doi.org/10.15468/dl.zfaw79
Anopheles gambiae	-3.486362	10.746527	Unclassified	GBIF: https://doi.org/10.15468/dl.zfaw79
Anopheles gambiae	-3.486271	10.746853	Unclassified	GBIF: https://doi.org/10.15468/dl.zfaw79
Anopheles gambiae	-3.486179	10.746839	Unclassified	GBIF: https://doi.org/10.15468/dl.zfaw79
Anopheles gambiae	-3.48333	16.36667	Unclassified	GBIF: https://doi.org/10.15468/dl.zfaw79
Anopheles gambiae	-3.479202	10.795241	Unclassified	GBIF: https://doi.org/10.15468/dl.zfaw79

Anopheles gambiae	-3.479137	10.79523	Unclassified	GBIF: https://doi.org/10.15468/dl.zfaw79
Anopheles gambiae	-3.478208	10.7946	Unclassified	GBIF: https://doi.org/10.15468/dl.zfaw79
Anopheles gambiae	-3.477823	10.79342	Unclassified	GBIF: https://doi.org/10.15468/dl.zfaw79
Anopheles gambiae	-3.477817	10.793442	Unclassified	GBIF: https://doi.org/10.15468/dl.zfaw79
Anopheles gambiae	-3.477808	10.793409	Unclassified	GBIF: https://doi.org/10.15468/dl.zfaw79
Anopheles gambiae	-3.477669	10.795122	Unclassified	GBIF: https://doi.org/10.15468/dl.zfaw79
Anopheles gambiae	-3.477567	10.795097	Unclassified	GBIF: https://doi.org/10.15468/dl.zfaw79
Anopheles gambiae	-3.477545	10.795111	Unclassified	GBIF: https://doi.org/10.15468/dl.zfaw79
Anopheles gambiae	-3.475168	10.858775	Unclassified	GBIF: https://doi.org/10.15468/dl.zfaw79
Anopheles gambiae	-3.4728	10.857876	Unclassified	GBIF: https://doi.org/10.15468/dl.zfaw79
Anopheles gambiae	-3.472757	10.857889	Unclassified	GBIF: https://doi.org/10.15468/dl.zfaw79
Anopheles gambiae	-3.472688	10.858547	Unclassified	GBIF: https://doi.org/10.15468/dl.zfaw79
Anopheles gambiae	-3.472538	10.858087	Unclassified	GBIF: https://doi.org/10.15468/dl.zfaw79
Anopheles gambiae	-3.472536	10.858099	Unclassified	GBIF: https://doi.org/10.15468/dl.zfaw79
Anopheles gambiae	-3.454403	10.919474	Unclassified	GBIF: https://doi.org/10.15468/dl.zfaw79
Anopheles gambiae	-3.453448	10.919592	Unclassified	GBIF: https://doi.org/10.15468/dl.zfaw79
Anopheles gambiae	-3.453352	10.922321	Unclassified	GBIF: https://doi.org/10.15468/dl.zfaw79
Anopheles gambiae	-3.453351	10.922379	Unclassified	GBIF: https://doi.org/10.15468/dl.zfaw79
Anopheles gambiae	-3.453336	10.922344	Unclassified	GBIF: https://doi.org/10.15468/dl.zfaw79
Anopheles gambiae	-3.453327	10.921791	Unclassified	GBIF: https://doi.org/10.15468/dl.zfaw79
Anopheles gambiae	-3.453218	10.921763	Unclassified	GBIF: https://doi.org/10.15468/dl.zfaw79
Anopheles gambiae	-3.453194	10.919507	Unclassified	GBIF: https://doi.org/10.15468/dl.zfaw79
Anopheles gambiae	-3.453175	10.921755	Unclassified	GBIF: https://doi.org/10.15468/dl.zfaw79
Anopheles gambiae	-3.453171	10.92178	Unclassified	GBIF: https://doi.org/10.15468/dl.zfaw79
Anopheles gambiae	-3.453155	10.921741	Unclassified	GBIF: https://doi.org/10.15468/dl.zfaw79
Anopheles gambiae	-3.453132	10.920139	Unclassified	GBIF: https://doi.org/10.15468/dl.zfaw79
Anopheles gambiae	-3.453089	10.921504	Unclassified	GBIF: https://doi.org/10.15468/dl.zfaw79
Anopheles gambiae	-3.452867	10.920293	Unclassified	GBIF: https://doi.org/10.15468/dl.zfaw79
Anopheles gambiae	-3.452863	10.920315	Unclassified	GBIF: https://doi.org/10.15468/dl.zfaw79
Anopheles gambiae	-3.452838	10.92036	Unclassified	GBIF: https://doi.org/10.15468/dl.zfaw79
Anopheles gambiae	-3.452748	10.920485	Unclassified	GBIF: https://doi.org/10.15468/dl.zfaw79
Anopheles gambiae	-3.452722	10.920495	Unclassified	GBIF: https://doi.org/10.15468/dl.zfaw79
Anopheles gambiae	-3.452622	10.922154	Unclassified	GBIF: https://doi.org/10.15468/dl.zfaw79
Anopheles gambiae	-3.452468	10.922771	Unclassified	GBIF: https://doi.org/10.15468/dl.zfaw79
Anopheles gambiae	-3.452465	10.922756	Unclassified	GBIF: https://doi.org/10.15468/dl.zfaw79
Anopheles gambiae	-3.452442	10.922821	Unclassified	GBIF: https://doi.org/10.15468/dl.zfaw79
Anopheles gambiae	-3.45	6.71667	Unclassified	GBIF: https://doi.org/10.15468/dl.zfaw79
Anopheles gambiae	-3.45	6.7	Unclassified	GBIF: https://doi.org/10.15468/dl.zfaw79
Anopheles gambiae	-3.447251	10.8158	Unclassified	GBIF: https://doi.org/10.15468/dl.zfaw79
Anopheles gambiae	-3.446506	10.816349	Unclassified	GBIF: https://doi.org/10.15468/dl.zfaw79
Anopheles gambiae	-3.446161	10.815725	Unclassified	GBIF: https://doi.org/10.15468/dl.zfaw79
Anopheles gambiae	-3.446081	10.815455	Unclassified	GBIF: https://doi.org/10.15468/dl.zfaw79

Anopheles gambiae	-3.446055	10.815437	Unclassified	GBIF: https://doi.org/10.15468/dl.zfaw79
Anopheles gambiae	-3.446023	10.815473	Unclassified	GBIF: https://doi.org/10.15468/dl.zfaw79
Anopheles gambiae	-3.446004	10.81544	Unclassified	GBIF: https://doi.org/10.15468/dl.zfaw79
Anopheles gambiae	-3.445984	10.815444	Unclassified	GBIF: https://doi.org/10.15468/dl.zfaw79
Anopheles gambiae	-3.445563	10.814736	Unclassified	GBIF: https://doi.org/10.15468/dl.zfaw79
Anopheles gambiae	-3.445551	10.8147	Unclassified	GBIF: https://doi.org/10.15468/dl.zfaw79
Anopheles gambiae	-3.445545	10.814746	Unclassified	GBIF: https://doi.org/10.15468/dl.zfaw79
Anopheles gambiae	-3.444903	10.814357	Unclassified	GBIF: https://doi.org/10.15468/dl.zfaw79
Anopheles gambiae	-3.444826	10.814347	Unclassified	GBIF: https://doi.org/10.15468/dl.zfaw79
Anopheles gambiae	-3.44193	10.823981	Unclassified	GBIF: https://doi.org/10.15468/dl.zfaw79
Anopheles gambiae	-3.43333	14.4	Unclassified	GBIF: https://doi.org/10.15468/dl.zfaw79
Anopheles gambiae	-3.418135	10.75436	Unclassified	GBIF: https://doi.org/10.15468/dl.zfaw79
Anopheles gambiae	-3.417735	10.754262	Unclassified	GBIF: https://doi.org/10.15468/dl.zfaw79
Anopheles gambiae	-3.417711	10.754363	Unclassified	GBIF: https://doi.org/10.15468/dl.zfaw79
Anopheles gambiae	-3.417289	10.75448	Unclassified	GBIF: https://doi.org/10.15468/dl.zfaw79
Anopheles gambiae	-3.41667	13.16667	Unclassified	GBIF: https://doi.org/10.15468/dl.zfaw79
Anopheles gambiae	-3.403983	10.794352	Unclassified	GBIF: https://doi.org/10.15468/dl.zfaw79
Anopheles gambiae	-3.400056	10.746463	Unclassified	GBIF: https://doi.org/10.15468/dl.zfaw79
Anopheles gambiae	-3.399661	10.743503	Unclassified	GBIF: https://doi.org/10.15468/dl.zfaw79
Anopheles gambiae	-3.399188	10.744949	Unclassified	GBIF: https://doi.org/10.15468/dl.zfaw79
Anopheles gambiae	-3.398909	10.746243	Unclassified	GBIF: https://doi.org/10.15468/dl.zfaw79
Anopheles gambiae	-3.398598	10.744867	Unclassified	GBIF: https://doi.org/10.15468/dl.zfaw79
Anopheles gambiae	-3.398204	10.744567	Unclassified	GBIF: https://doi.org/10.15468/dl.zfaw79
Anopheles gambiae	-3.398181	10.745831	Unclassified	GBIF: https://doi.org/10.15468/dl.zfaw79
Anopheles gambiae	-3.39768	10.744274	Unclassified	GBIF: https://doi.org/10.15468/dl.zfaw79
Anopheles gambiae	-3.39461	10.80788	Unclassified	GBIF: https://doi.org/10.15468/dl.zfaw79
Anopheles gambiae	-3.393985	10.807926	Unclassified	GBIF: https://doi.org/10.15468/dl.zfaw79
Anopheles gambiae	-3.393884	10.805911	Unclassified	GBIF: https://doi.org/10.15468/dl.zfaw79
Anopheles gambiae	-3.393422	10.808199	Unclassified	GBIF: https://doi.org/10.15468/dl.zfaw79
Anopheles gambiae	-3.393395	10.80714	Unclassified	GBIF: https://doi.org/10.15468/dl.zfaw79
Anopheles gambiae	-3.393386	10.808234	Unclassified	GBIF: https://doi.org/10.15468/dl.zfaw79
Anopheles gambiae	-3.387539	10.914582	Unclassified	GBIF: https://doi.org/10.15468/dl.zfaw79
Anopheles gambiae	-3.387473	10.914349	Unclassified	GBIF: https://doi.org/10.15468/dl.zfaw79
Anopheles gambiae	-3.386719	10.915201	Unclassified	GBIF: https://doi.org/10.15468/dl.zfaw79
Anopheles gambiae	-3.386599	10.913746	Unclassified	GBIF: https://doi.org/10.15468/dl.zfaw79
Anopheles gambiae	-3.385578	10.912662	Unclassified	GBIF: https://doi.org/10.15468/dl.zfaw79
Anopheles gambiae	-3.384354	10.988509	Unclassified	GBIF: https://doi.org/10.15468/dl.zfaw79
Anopheles gambiae	-3.384336	10.988661	Unclassified	GBIF: https://doi.org/10.15468/dl.zfaw79
Anopheles gambiae	-3.384312	10.988624	Unclassified	GBIF: https://doi.org/10.15468/dl.zfaw79
Anopheles gambiae	-3.384052	10.98831	Unclassified	GBIF: https://doi.org/10.15468/dl.zfaw79
Anopheles gambiae	-3.383707	10.988053	Unclassified	GBIF: https://doi.org/10.15468/dl.zfaw79
Anopheles gambiae	-3.38333	16.25	Unclassified	GBIF: https://doi.org/10.15468/dl.zfaw79

Anopheles gambiae	-3.383304	10.987865	Unclassified	GBIF: https://doi.org/10.15468/dl.zfaw79
Anopheles gambiae	-3.383283	10.987871	Unclassified	GBIF: https://doi.org/10.15468/dl.zfaw79
Anopheles gambiae	-3.35	13.15	Unclassified	GBIF: https://doi.org/10.15468/dl.zfaw79
Anopheles gambiae	-3.342176	10.781939	Unclassified	GBIF: https://doi.org/10.15468/dl.zfaw79
Anopheles gambiae	-3.342055	10.781851	Unclassified	GBIF: https://doi.org/10.15468/dl.zfaw79
Anopheles gambiae	-3.342048	10.78183	Unclassified	GBIF: https://doi.org/10.15468/dl.zfaw79
Anopheles gambiae	-3.342034	10.781841	Unclassified	GBIF: https://doi.org/10.15468/dl.zfaw79
Anopheles gambiae	-3.342008	10.78191	Unclassified	GBIF: https://doi.org/10.15468/dl.zfaw79
Anopheles gambiae	-3.341794	10.781939	Unclassified	GBIF: https://doi.org/10.15468/dl.zfaw79
Anopheles gambiae	-3.341612	10.781839	Unclassified	GBIF: https://doi.org/10.15468/dl.zfaw79
Anopheles gambiae	-3.341607	10.781834	Unclassified	GBIF: https://doi.org/10.15468/dl.zfaw79
Anopheles gambiae	-3.341232	10.782837	Unclassified	GBIF: https://doi.org/10.15468/dl.zfaw79
Anopheles gambiae	-3.340978	10.78277	Unclassified	GBIF: https://doi.org/10.15468/dl.zfaw79
Anopheles gambiae	-3.340944	10.782752	Unclassified	GBIF: https://doi.org/10.15468/dl.zfaw79
Anopheles gambiae	-3.340556	10.783444	Unclassified	GBIF: https://doi.org/10.15468/dl.zfaw79
Anopheles gambiae	-3.340141	10.783414	Unclassified	GBIF: https://doi.org/10.15468/dl.zfaw79
Anopheles gambiae	-3.33333	9.8	Unclassified	GBIF: https://doi.org/10.15468/dl.zfaw79
Anopheles gambiae	-3.332104	10.854664	Unclassified	GBIF: https://doi.org/10.15468/dl.zfaw79
Anopheles gambiae	-3.332069	10.854748	Unclassified	GBIF: https://doi.org/10.15468/dl.zfaw79
Anopheles gambiae	-3.331869	10.856224	Unclassified	GBIF: https://doi.org/10.15468/dl.zfaw79
Anopheles gambiae	-3.331853	10.856236	Unclassified	GBIF: https://doi.org/10.15468/dl.zfaw79
Anopheles gambiae	-3.330952	10.826591	Unclassified	GBIF: https://doi.org/10.15468/dl.zfaw79
Anopheles gambiae	-3.330898	10.825726	Unclassified	GBIF: https://doi.org/10.15468/dl.zfaw79
Anopheles gambiae	-3.330889	10.825541	Unclassified	GBIF: https://doi.org/10.15468/dl.zfaw79
Anopheles gambiae	-3.330863	10.825548	Unclassified	GBIF: https://doi.org/10.15468/dl.zfaw79
Anopheles gambiae	-3.33082	10.855686	Unclassified	GBIF: https://doi.org/10.15468/dl.zfaw79
Anopheles gambiae	-3.330592	10.824709	Unclassified	GBIF: https://doi.org/10.15468/dl.zfaw79
Anopheles gambiae	-3.330557	10.824723	Unclassified	GBIF: https://doi.org/10.15468/dl.zfaw79
Anopheles gambiae	-3.330532	10.824689	Unclassified	GBIF: https://doi.org/10.15468/dl.zfaw79
Anopheles gambiae	-3.3305	10.824746	Unclassified	GBIF: https://doi.org/10.15468/dl.zfaw79
Anopheles gambiae	-3.330331	10.825128	Unclassified	GBIF: https://doi.org/10.15468/dl.zfaw79
Anopheles gambiae	-3.330326	10.825814	Unclassified	GBIF: https://doi.org/10.15468/dl.zfaw79
Anopheles gambiae	-3.330063	10.825624	Unclassified	GBIF: https://doi.org/10.15468/dl.zfaw79
Anopheles gambiae	-3.330062	10.82558	Unclassified	GBIF: https://doi.org/10.15468/dl.zfaw79
Anopheles gambiae	-3.329992	10.892918	Unclassified	GBIF: https://doi.org/10.15468/dl.zfaw79
Anopheles gambiae	-3.32995	10.856014	Unclassified	GBIF: https://doi.org/10.15468/dl.zfaw79
Anopheles gambiae	-3.329923	10.855973	Unclassified	GBIF: https://doi.org/10.15468/dl.zfaw79
Anopheles gambiae	-3.329921	10.891975	Unclassified	GBIF: https://doi.org/10.15468/dl.zfaw79
Anopheles gambiae	-3.329896	10.89194	Unclassified	GBIF: https://doi.org/10.15468/dl.zfaw79
Anopheles gambiae	-3.329804	10.824567	Unclassified	GBIF: https://doi.org/10.15468/dl.zfaw79
Anopheles gambiae	-3.329531	10.892519	Unclassified	GBIF: https://doi.org/10.15468/dl.zfaw79
Anopheles gambiae	-3.329525	10.89252	Unclassified	GBIF: https://doi.org/10.15468/dl.zfaw79

Anopheles gambiae	-3.329497	10.892524	Unclassified	GBIF: https://doi.org/10.15468/dl.zfaw79
Anopheles gambiae	-3.329295	10.822287	Unclassified	GBIF: https://doi.org/10.15468/dl.zfaw79
Anopheles gambiae	-3.328943	10.892494	Unclassified	GBIF: https://doi.org/10.15468/dl.zfaw79
Anopheles gambiae	-3.327488	10.894405	Unclassified	GBIF: https://doi.org/10.15468/dl.zfaw79
Anopheles gambiae	-3.32624	10.891821	Unclassified	GBIF: https://doi.org/10.15468/dl.zfaw79
Anopheles gambiae	-3.326126	10.891635	Unclassified	GBIF: https://doi.org/10.15468/dl.zfaw79
Anopheles gambiae	-3.32612	10.891774	Unclassified	GBIF: https://doi.org/10.15468/dl.zfaw79
Anopheles gambiae	-3.324404	10.890882	Unclassified	GBIF: https://doi.org/10.15468/dl.zfaw79
Anopheles gambiae	-3.323908	10.891245	Unclassified	GBIF: https://doi.org/10.15468/dl.zfaw79
Anopheles gambiae	-3.323332	10.89123	Unclassified	GBIF: https://doi.org/10.15468/dl.zfaw79
Anopheles gambiae	-3.322304	10.891803	Unclassified	GBIF: https://doi.org/10.15468/dl.zfaw79
Anopheles gambiae	-3.321167	10.852973	Unclassified	GBIF: https://doi.org/10.15468/dl.zfaw79
Anopheles gambiae	-3.320809	10.853105	Unclassified	GBIF: https://doi.org/10.15468/dl.zfaw79
Anopheles gambiae	-3.32079	10.852812	Unclassified	GBIF: https://doi.org/10.15468/dl.zfaw79
Anopheles gambiae	-3.320505	10.85249	Unclassified	GBIF: https://doi.org/10.15468/dl.zfaw79
Anopheles gambiae	-3.31667	16.31667	Unclassified	GBIF: https://doi.org/10.15468/dl.zfaw79
Anopheles gambiae	-3.31581	10.852056	Unclassified	GBIF: https://doi.org/10.15468/dl.zfaw79
Anopheles gambiae	-3.315796	10.851316	Unclassified	GBIF: https://doi.org/10.15468/dl.zfaw79
Anopheles gambiae	-3.31579	10.851355	Unclassified	GBIF: https://doi.org/10.15468/dl.zfaw79
Anopheles gambiae	-3.315789	10.851345	Unclassified	GBIF: https://doi.org/10.15468/dl.zfaw79
Anopheles gambiae	-3.31578	10.852071	Unclassified	GBIF: https://doi.org/10.15468/dl.zfaw79
Anopheles gambiae	-3.315769	10.852078	Unclassified	GBIF: https://doi.org/10.15468/dl.zfaw79
Anopheles gambiae	-3.31576	10.851339	Unclassified	GBIF: https://doi.org/10.15468/dl.zfaw79
Anopheles gambiae	-3.315739	10.84928	Unclassified	GBIF: https://doi.org/10.15468/dl.zfaw79
Anopheles gambiae	-3.315715	10.852331	Unclassified	GBIF: https://doi.org/10.15468/dl.zfaw79
Anopheles gambiae	-3.315689	10.852419	Unclassified	GBIF: https://doi.org/10.15468/dl.zfaw79
Anopheles gambiae	-3.315687	10.852369	Unclassified	GBIF: https://doi.org/10.15468/dl.zfaw79
Anopheles gambiae	-3.314975	10.708011	Unclassified	GBIF: https://doi.org/10.15468/dl.zfaw79
Anopheles gambiae	-3.313999	10.854832	Unclassified	GBIF: https://doi.org/10.15468/dl.zfaw79
Anopheles gambiae	-3.313989	10.708101	Unclassified	GBIF: https://doi.org/10.15468/dl.zfaw79
Anopheles gambiae	-3.3	14.46667	Unclassified	GBIF: https://doi.org/10.15468/dl.zfaw79
Anopheles gambiae	-3.296257	10.6693	Unclassified	GBIF: https://doi.org/10.15468/dl.zfaw79
Anopheles gambiae	-3.296218	10.669313	Unclassified	GBIF: https://doi.org/10.15468/dl.zfaw79
Anopheles gambiae	-3.295975	10.669052	Unclassified	GBIF: https://doi.org/10.15468/dl.zfaw79
Anopheles gambiae	-3.295841	10.668278	Unclassified	GBIF: https://doi.org/10.15468/dl.zfaw79
Anopheles gambiae	-3.29583	10.668199	Unclassified	GBIF: https://doi.org/10.15468/dl.zfaw79
Anopheles gambiae	-3.295752	10.667907	Unclassified	GBIF: https://doi.org/10.15468/dl.zfaw79
Anopheles gambiae	-3.2952	10.668362	Unclassified	GBIF: https://doi.org/10.15468/dl.zfaw79
Anopheles gambiae	-3.295173	10.668426	Unclassified	GBIF: https://doi.org/10.15468/dl.zfaw79
Anopheles gambiae	-3.289781	10.989137	Unclassified	GBIF: https://doi.org/10.15468/dl.zfaw79
Anopheles gambiae	-3.289593	10.989876	Unclassified	GBIF: https://doi.org/10.15468/dl.zfaw79
Anopheles gambiae	-3.288112	10.712303	Unclassified	GBIF: https://doi.org/10.15468/dl.zfaw79

Anopheles gambiae	-3.287936	10.711344	Unclassified	GBIF: https://doi.org/10.15468/dl.zfaw79
Anopheles gambiae	-3.2879	10.711338	Unclassified	GBIF: https://doi.org/10.15468/dl.zfaw79
Anopheles gambiae	-3.287418	10.711288	Unclassified	GBIF: https://doi.org/10.15468/dl.zfaw79
Anopheles gambiae	-3.287385	10.711307	Unclassified	GBIF: https://doi.org/10.15468/dl.zfaw79
Anopheles gambiae	-3.287375	10.7113	Unclassified	GBIF: https://doi.org/10.15468/dl.zfaw79
Anopheles gambiae	-3.28722	10.761557	Unclassified	GBIF: https://doi.org/10.15468/dl.zfaw79
Anopheles gambiae	-3.28713	10.762614	Unclassified	GBIF: https://doi.org/10.15468/dl.zfaw79
Anopheles gambiae	-3.287029	10.713	Unclassified	GBIF: https://doi.org/10.15468/dl.zfaw79
Anopheles gambiae	-3.286987	10.712953	Unclassified	GBIF: https://doi.org/10.15468/dl.zfaw79
Anopheles gambiae	-3.286984	10.71299	Unclassified	GBIF: https://doi.org/10.15468/dl.zfaw79
Anopheles gambiae	-3.284901	10.760504	Unclassified	GBIF: https://doi.org/10.15468/dl.zfaw79
Anopheles gambiae	-3.284695	10.761533	Unclassified	GBIF: https://doi.org/10.15468/dl.zfaw79
Anopheles gambiae	-3.284679	10.761879	Unclassified	GBIF: https://doi.org/10.15468/dl.zfaw79
Anopheles gambiae	-3.256245	10.749499	Unclassified	GBIF: https://doi.org/10.15468/dl.zfaw79
Anopheles gambiae	-3.256221	10.749552	Unclassified	GBIF: https://doi.org/10.15468/dl.zfaw79
Anopheles gambiae	-3.256147	10.749527	Unclassified	GBIF: https://doi.org/10.15468/dl.zfaw79
Anopheles gambiae	-3.256134	10.749538	Unclassified	GBIF: https://doi.org/10.15468/dl.zfaw79
Anopheles gambiae	-3.255973	10.745221	Unclassified	GBIF: https://doi.org/10.15468/dl.zfaw79
Anopheles gambiae	-3.255915	10.745237	Unclassified	GBIF: https://doi.org/10.15468/dl.zfaw79
Anopheles gambiae	-3.255906	10.745241	Unclassified	GBIF: https://doi.org/10.15468/dl.zfaw79
Anopheles gambiae	-3.25589	10.745253	Unclassified	GBIF: https://doi.org/10.15468/dl.zfaw79
Anopheles gambiae	-3.255249	10.749508	Unclassified	GBIF: https://doi.org/10.15468/dl.zfaw79
Anopheles gambiae	-3.255217	10.749533	Unclassified	GBIF: https://doi.org/10.15468/dl.zfaw79
Anopheles gambiae	-3.255191	10.749473	Unclassified	GBIF: https://doi.org/10.15468/dl.zfaw79
Anopheles gambiae	-3.255155	10.749496	Unclassified	GBIF: https://doi.org/10.15468/dl.zfaw79
Anopheles gambiae	-3.255154	10.749507	Unclassified	GBIF: https://doi.org/10.15468/dl.zfaw79
Anopheles gambiae	-3.23333	16.08333	Unclassified	GBIF: https://doi.org/10.15468/dl.zfaw79
Anopheles gambiae	-3.212989	10.738675	Unclassified	GBIF: https://doi.org/10.15468/dl.zfaw79
Anopheles gambiae	-3.212977	10.739225	Unclassified	GBIF: https://doi.org/10.15468/dl.zfaw79
Anopheles gambiae	-3.212783	10.738704	Unclassified	GBIF: https://doi.org/10.15468/dl.zfaw79
Anopheles gambiae	-3.212624	10.739121	Unclassified	GBIF: https://doi.org/10.15468/dl.zfaw79
Anopheles gambiae	-3.208781	10.742146	Unclassified	GBIF: https://doi.org/10.15468/dl.zfaw79
Anopheles gambiae	-3.208712	10.742072	Unclassified	GBIF: https://doi.org/10.15468/dl.zfaw79
Anopheles gambiae	-3.208352	10.741617	Unclassified	GBIF: https://doi.org/10.15468/dl.zfaw79
Anopheles gambiae	-3.208278	10.741602	Unclassified	GBIF: https://doi.org/10.15468/dl.zfaw79
Anopheles gambiae	-3.208146	10.742015	Unclassified	GBIF: https://doi.org/10.15468/dl.zfaw79
Anopheles gambiae	-3.208086	10.742054	Unclassified	GBIF: https://doi.org/10.15468/dl.zfaw79
Anopheles gambiae	-3.202365	10.69502	Unclassified	GBIF: https://doi.org/10.15468/dl.zfaw79
Anopheles gambiae	-3.202034	10.693052	Unclassified	GBIF: https://doi.org/10.15468/dl.zfaw79
Anopheles gambiae	-3.202032	10.69303	Unclassified	GBIF: https://doi.org/10.15468/dl.zfaw79
Anopheles gambiae	-3.2	8.68333	Unclassified	GBIF: https://doi.org/10.15468/dl.zfaw79
Anopheles gambiae	-3.2	11	Unclassified	GBIF: https://doi.org/10.15468/dl.zfaw79

Anopheles gambiae	-3.2	10.3	Unclassified	GBIF: https://doi.org/10.15468/dl.zfaw79
Anopheles gambiae	-3.196218	10.972128	Unclassified	GBIF: https://doi.org/10.15468/dl.zfaw79
Anopheles gambiae	-3.194461	10.962637	Unclassified	GBIF: https://doi.org/10.15468/dl.zfaw79
Anopheles gambiae	-3.19443	10.963547	Unclassified	GBIF: https://doi.org/10.15468/dl.zfaw79
Anopheles gambiae	-3.194416	10.960994	Unclassified	GBIF: https://doi.org/10.15468/dl.zfaw79
Anopheles gambiae	-3.19441	10.962663	Unclassified	GBIF: https://doi.org/10.15468/dl.zfaw79
Anopheles gambiae	-3.194278	10.974277	Unclassified	GBIF: https://doi.org/10.15468/dl.zfaw79
Anopheles gambiae	-3.192815	10.963358	Unclassified	GBIF: https://doi.org/10.15468/dl.zfaw79
Anopheles gambiae	-3.191842	10.963724	Unclassified	GBIF: https://doi.org/10.15468/dl.zfaw79
Anopheles gambiae	-3.191202	10.964034	Unclassified	GBIF: https://doi.org/10.15468/dl.zfaw79
Anopheles gambiae	-3.190162	10.96256	Unclassified	GBIF: https://doi.org/10.15468/dl.zfaw79
Anopheles gambiae	-3.189137	10.961069	Unclassified	GBIF: https://doi.org/10.15468/dl.zfaw79
Anopheles gambiae	-3.189124	10.961074	Unclassified	GBIF: https://doi.org/10.15468/dl.zfaw79
Anopheles gambiae	-3.18912	10.961106	Unclassified	GBIF: https://doi.org/10.15468/dl.zfaw79
Anopheles gambiae	-3.18911	10.961097	Unclassified	GBIF: https://doi.org/10.15468/dl.zfaw79
Anopheles gambiae	-3.166412	10.737555	Unclassified	GBIF: https://doi.org/10.15468/dl.zfaw79
Anopheles gambiae	-3.166394	10.737602	Unclassified	GBIF: https://doi.org/10.15468/dl.zfaw79
Anopheles gambiae	-3.165782	10.738518	Unclassified	GBIF: https://doi.org/10.15468/dl.zfaw79
Anopheles gambiae	-3.165778	10.738544	Unclassified	GBIF: https://doi.org/10.15468/dl.zfaw79
Anopheles gambiae	-3.165712	10.737985	Unclassified	GBIF: https://doi.org/10.15468/dl.zfaw79
Anopheles gambiae	-3.165707	10.738	Unclassified	GBIF: https://doi.org/10.15468/dl.zfaw79
Anopheles gambiae	-3.165506	10.737966	Unclassified	GBIF: https://doi.org/10.15468/dl.zfaw79
Anopheles gambiae	-3.165469	10.737956	Unclassified	GBIF: https://doi.org/10.15468/dl.zfaw79
Anopheles gambiae	-3.1	16.08333	Unclassified	GBIF: https://doi.org/10.15468/dl.zfaw79
Anopheles gambiae	-2.96667	15	Unclassified	GBIF: https://doi.org/10.15468/dl.zfaw79
Anopheles gambiae	-2.9	11.78333	Unclassified	GBIF: https://doi.org/10.15468/dl.zfaw79
Anopheles gambiae	-2.88333	9.25	Unclassified	GBIF: https://doi.org/10.15468/dl.zfaw79
Anopheles gambiae	-2.83333	16.71667	Unclassified	GBIF: https://doi.org/10.15468/dl.zfaw79
Anopheles gambiae	-2.7167	8.1667	Unclassified	GBIF: https://doi.org/10.15468/dl.zfaw79
Anopheles gambiae	-2.7	9.45	Unclassified	GBIF: https://doi.org/10.15468/dl.zfaw79
Anopheles gambiae	-2.61667	8.63333	Unclassified	GBIF: https://doi.org/10.15468/dl.zfaw79
Anopheles gambiae	-2.61667	12.3	Unclassified	GBIF: https://doi.org/10.15468/dl.zfaw79
Anopheles gambiae	-2.47	12.32	Unclassified	GBIF: https://doi.org/10.15468/dl.zfaw79
Anopheles gambiae	-2.45	12.21667	Unclassified	GBIF: https://doi.org/10.15468/dl.zfaw79
Anopheles gambiae	-2.41	13.57	Unclassified	GBIF: https://doi.org/10.15468/dl.zfaw79
Anopheles gambiae	-2.38333	12.25	Unclassified	GBIF: https://doi.org/10.15468/dl.zfaw79
Anopheles gambiae	-2.33333	13.51667	Unclassified	GBIF: https://doi.org/10.15468/dl.zfaw79
Anopheles gambiae	-2.25	12.01667	Unclassified	GBIF: https://doi.org/10.15468/dl.zfaw79
Anopheles gambiae	-2.13333	11.11667	Unclassified	GBIF: https://doi.org/10.15468/dl.zfaw79
Anopheles gambiae	-1.8	12.3	Unclassified	GBIF: https://doi.org/10.15468/dl.zfaw79
Anopheles gambiae	-1.75	12.41667	Unclassified	GBIF: https://doi.org/10.15468/dl.zfaw79
Anopheles gambiae	-1.66667	12.33333	Unclassified	GBIF: https://doi.org/10.15468/dl.zfaw79

Anopheles gambiae	-1.66667	11.33333	Unclassified	GBIF: https://doi.org/10.15468/dl.zfaw79
Anopheles gambiae	-1.63333	14.15	Unclassified	GBIF: https://doi.org/10.15468/dl.zfaw79
Anopheles gambiae	-1.58333	12.36667	Unclassified	GBIF: https://doi.org/10.15468/dl.zfaw79
Anopheles gambiae	-1.53	13.33	Unclassified	GBIF: https://doi.org/10.15468/dl.zfaw79
Anopheles gambiae	-1.48333	14.08333	Unclassified	GBIF: https://doi.org/10.15468/dl.zfaw79
Anopheles gambiae	-1.34	12.07	Unclassified	GBIF: https://doi.org/10.15468/dl.zfaw79
Anopheles gambiae	-1.33333	15.8	Unclassified	GBIF: https://doi.org/10.15468/dl.zfaw79
Anopheles gambiae	-1.26667	11.83333	Unclassified	GBIF: https://doi.org/10.15468/dl.zfaw79
Anopheles gambiae	-1.15	13.06667	Unclassified	GBIF: https://doi.org/10.15468/dl.zfaw79
Anopheles gambiae	-0.33333	16.91667	Unclassified	GBIF: https://doi.org/10.15468/dl.zfaw79
Anopheles gambiae	-0.31667	11.9	Unclassified	GBIF: https://doi.org/10.15468/dl.zfaw79
Anopheles gambiae	-0.03333	14.05	Unclassified	GBIF: https://doi.org/10.15468/dl.zfaw79
Anopheles gambiae	0.11667	17.33333	Unclassified	GBIF: https://doi.org/10.15468/dl.zfaw79
Anopheles gambiae	0.117	5.883	Unclassified	GBIF: https://doi.org/10.15468/dl.zfaw79
Anopheles gambiae	0.11701	5.88301	Unclassified	GBIF: https://doi.org/10.15468/dl.zfaw79
Anopheles gambiae	0.18333	5.81667	Unclassified	GBIF: https://doi.org/10.15468/dl.zfaw79
Anopheles gambiae	0.2	10.66667	Unclassified	GBIF: https://doi.org/10.15468/dl.zfaw79
Anopheles gambiae	0.21667	11.81667	Unclassified	GBIF: https://doi.org/10.15468/dl.zfaw79
Anopheles gambiae	0.23333	6.95	Unclassified	GBIF: https://doi.org/10.15468/dl.zfaw79
Anopheles gambiae	0.25	6.91667	Unclassified	GBIF: https://doi.org/10.15468/dl.zfaw79
Anopheles gambiae	0.33333	6.58333	Unclassified	GBIF: https://doi.org/10.15468/dl.zfaw79
Anopheles gambiae	0.38333	10.73333	Unclassified	GBIF: https://doi.org/10.15468/dl.zfaw79
Anopheles gambiae	0.41667	10.16667	Unclassified	GBIF: https://doi.org/10.15468/dl.zfaw79
Anopheles gambiae	0.46667	12.25	Unclassified	GBIF: https://doi.org/10.15468/dl.zfaw79
Anopheles gambiae	0.5	10.5	Unclassified	GBIF: https://doi.org/10.15468/dl.zfaw79
Anopheles gambiae	0.586	5.781	Unclassified	GBIF: https://doi.org/10.15468/dl.zfaw79
Anopheles gambiae	0.58601	5.78101	Unclassified	GBIF: https://doi.org/10.15468/dl.zfaw79
Anopheles gambiae	0.61667	18.06667	Unclassified	GBIF: https://doi.org/10.15468/dl.zfaw79
Anopheles gambiae	0.66667	7.03333	Unclassified	GBIF: https://doi.org/10.15468/dl.zfaw79
Anopheles gambiae	0.708	9.376	Unclassified	GBIF: https://doi.org/10.15468/dl.zfaw79
Anopheles gambiae	0.70801	9.37601	Unclassified	GBIF: https://doi.org/10.15468/dl.zfaw79
Anopheles gambiae	0.75	7.16667	Unclassified	GBIF: https://doi.org/10.15468/dl.zfaw79
Anopheles gambiae	0.83333	19.33333	Unclassified	GBIF: https://doi.org/10.15468/dl.zfaw79
Anopheles gambiae	0.9	6.56667	Unclassified	GBIF: https://doi.org/10.15468/dl.zfaw79
Anopheles gambiae	0.93333	14.73333	Unclassified	GBIF: https://doi.org/10.15468/dl.zfaw79
Anopheles gambiae	0.93333	7.63333	Unclassified	GBIF: https://doi.org/10.15468/dl.zfaw79
Anopheles gambiae	0.96667	11.05	Unclassified	GBIF: https://doi.org/10.15468/dl.zfaw79
Anopheles gambiae	1.032	9.563	Unclassified	GBIF: https://doi.org/10.15468/dl.zfaw79
Anopheles gambiae	1.03201	9.56301	Unclassified	GBIF: https://doi.org/10.15468/dl.zfaw79
Anopheles gambiae	1.03333	12.46667	Unclassified	GBIF: https://doi.org/10.15468/dl.zfaw79
Anopheles gambiae	1.070225	10.821468	Unclassified	GBIF: https://doi.org/10.15468/dl.zfaw79
Anopheles gambiae	1.070225	10.479186	Unclassified	GBIF: https://doi.org/10.15468/dl.zfaw79

Anopheles gambiae	1.100053	10.183217	Unclassified	GBIF: https://doi.org/10.15468/dl.zfaw79
Anopheles gambiae	1.13333	7.18333	Unclassified	GBIF: https://doi.org/10.15468/dl.zfaw79
Anopheles gambiae	1.18333	6.86667	Unclassified	GBIF: https://doi.org/10.15468/dl.zfaw79
Anopheles gambiae	1.245	6.534	Unclassified	GBIF: https://doi.org/10.15468/dl.zfaw79
Anopheles gambiae	1.24501	6.53401	Unclassified	GBIF: https://doi.org/10.15468/dl.zfaw79
Anopheles gambiae	1.33333	18.41667	Unclassified	GBIF: https://doi.org/10.15468/dl.zfaw79
Anopheles gambiae	1.378001	10.498435	Unclassified	GBIF: https://doi.org/10.15468/dl.zfaw79
Anopheles gambiae	1.383541	10.251408	Unclassified	GBIF: https://doi.org/10.15468/dl.zfaw79
Anopheles gambiae	1.420338	10.965512	Unclassified	GBIF: https://doi.org/10.15468/dl.zfaw79
Anopheles gambiae	1.431776	9.621699	Unclassified	GBIF: https://doi.org/10.15468/dl.zfaw79
Anopheles gambiae	1.5	6.25	Unclassified	GBIF: https://doi.org/10.15468/dl.zfaw79
Anopheles gambiae	1.51667	6.23333	Unclassified	GBIF: https://doi.org/10.15468/dl.zfaw79
Anopheles gambiae	1.53333	6.2	Unclassified	GBIF: https://doi.org/10.15468/dl.zfaw79
Anopheles gambiae	1.544632	9.881787	Unclassified	GBIF: https://doi.org/10.15468/dl.zfaw79
Anopheles gambiae	1.616557	11.303739	Unclassified	GBIF: https://doi.org/10.15468/dl.zfaw79
Anopheles gambiae	1.665161	9.710668	Unclassified	GBIF: https://doi.org/10.15468/dl.zfaw79
Anopheles gambiae	1.66667	7.83333	Unclassified	GBIF: https://doi.org/10.15468/dl.zfaw79
Anopheles gambiae	1.667509	11.370685	Unclassified	GBIF: https://doi.org/10.15468/dl.zfaw79
Anopheles gambiae	1.667645	6.581237	Unclassified	GBIF: https://doi.org/10.15468/dl.zfaw79
Anopheles gambiae	1.67317	6.94103	Unclassified	GBIF: https://doi.org/10.15468/dl.zfaw79
Anopheles gambiae	1.68333	8.6	Unclassified	GBIF: https://doi.org/10.15468/dl.zfaw79
Anopheles gambiae	1.701118	10.792699	Unclassified	GBIF: https://doi.org/10.15468/dl.zfaw79
Anopheles gambiae	1.703533	11.402647	Unclassified	GBIF: https://doi.org/10.15468/dl.zfaw79
Anopheles gambiae	1.703843	10.836197	Unclassified	GBIF: https://doi.org/10.15468/dl.zfaw79
Anopheles gambiae	1.714034	11.00589	Unclassified	GBIF: https://doi.org/10.15468/dl.zfaw79
Anopheles gambiae	1.71618	6.8991	Unclassified	GBIF: https://doi.org/10.15468/dl.zfaw79
Anopheles gambiae	1.71714	6.645852	Unclassified	GBIF: https://doi.org/10.15468/dl.zfaw79
Anopheles gambiae	1.718009	11.002679	Unclassified	GBIF: https://doi.org/10.15468/dl.zfaw79
Anopheles gambiae	1.722393	10.932028	Unclassified	GBIF: https://doi.org/10.15468/dl.zfaw79
Anopheles gambiae	1.737336	10.790329	Unclassified	GBIF: https://doi.org/10.15468/dl.zfaw79
Anopheles gambiae	1.742677	7.200417	Unclassified	GBIF: https://doi.org/10.15468/dl.zfaw79
Anopheles gambiae	1.782231	10.766118	Unclassified	GBIF: https://doi.org/10.15468/dl.zfaw79
Anopheles gambiae	1.782796	10.432187	Unclassified	GBIF: https://doi.org/10.15468/dl.zfaw79
Anopheles gambiae	1.78333	7.65	Unclassified	GBIF: https://doi.org/10.15468/dl.zfaw79
Anopheles gambiae	1.791098	10.736402	Unclassified	GBIF: https://doi.org/10.15468/dl.zfaw79
Anopheles gambiae	1.798892	10.706577	Unclassified	GBIF: https://doi.org/10.15468/dl.zfaw79
Anopheles gambiae	1.8	12.15	Unclassified	GBIF: https://doi.org/10.15468/dl.zfaw79
Anopheles gambiae	1.806164	6.788782	Unclassified	GBIF: https://doi.org/10.15468/dl.zfaw79
Anopheles gambiae	1.80672	6.276375	Unclassified	GBIF: https://doi.org/10.15468/dl.zfaw79
Anopheles gambiae	1.809261	10.876923	Unclassified	GBIF: https://doi.org/10.15468/dl.zfaw79
Anopheles gambiae	1.80951	10.698325	Unclassified	GBIF: https://doi.org/10.15468/dl.zfaw79
Anopheles gambiae	1.812788	10.7737	Unclassified	GBIF: https://doi.org/10.15468/dl.zfaw79

Anopheles gambiae	1.815344	10.888701	Unclassified	GBIF: https://doi.org/10.15468/dl.zfaw79
Anopheles gambiae	1.820961	6.891788	Unclassified	GBIF: https://doi.org/10.15468/dl.zfaw79
Anopheles gambiae	1.82184	8.966664	Unclassified	GBIF: https://doi.org/10.15468/dl.zfaw79
Anopheles gambiae	1.824425	7.075	Unclassified	GBIF: https://doi.org/10.15468/dl.zfaw79
Anopheles gambiae	1.836815	10.674056	Unclassified	GBIF: https://doi.org/10.15468/dl.zfaw79
Anopheles gambiae	1.843678	11.334202	Unclassified	GBIF: https://doi.org/10.15468/dl.zfaw79
Anopheles gambiae	1.863731	6.544869	Unclassified	GBIF: https://doi.org/10.15468/dl.zfaw79
Anopheles gambiae	1.869567	7.866489	Unclassified	GBIF: https://doi.org/10.15468/dl.zfaw79
Anopheles gambiae	1.88538	6.40966	Unclassified	GBIF: https://doi.org/10.15468/dl.zfaw79
Anopheles gambiae	1.9	6.85	Unclassified	GBIF: https://doi.org/10.15468/dl.zfaw79
Anopheles gambiae	1.90453	10.704394	Unclassified	GBIF: https://doi.org/10.15468/dl.zfaw79
Anopheles gambiae	1.930118	10.77192	Unclassified	GBIF: https://doi.org/10.15468/dl.zfaw79
Anopheles gambiae	1.93404	6.63625	Unclassified	GBIF: https://doi.org/10.15468/dl.zfaw79
Anopheles gambiae	1.93416	7.34464	Unclassified	GBIF: https://doi.org/10.15468/dl.zfaw79
Anopheles gambiae	1.964502	10.677505	Unclassified	GBIF: https://doi.org/10.15468/dl.zfaw79
Anopheles gambiae	1.964645	7.069011	Unclassified	GBIF: https://doi.org/10.15468/dl.zfaw79
Anopheles gambiae	1.968125	6.521662	Unclassified	GBIF: https://doi.org/10.15468/dl.zfaw79
Anopheles gambiae	1.96839	6.58927	Unclassified	GBIF: https://doi.org/10.15468/dl.zfaw79
Anopheles gambiae	1.968794	11.152095	Unclassified	GBIF: https://doi.org/10.15468/dl.zfaw79
Anopheles gambiae	1.970425	11.146781	Unclassified	GBIF: https://doi.org/10.15468/dl.zfaw79
Anopheles gambiae	1.972308	10.83356	Unclassified	GBIF: https://doi.org/10.15468/dl.zfaw79
Anopheles gambiae	1.972922	6.874445	Unclassified	GBIF: https://doi.org/10.15468/dl.zfaw79
Anopheles gambiae	1.976907	7.399119	Unclassified	GBIF: https://doi.org/10.15468/dl.zfaw79
Anopheles gambiae	1.9772	7.0811	Unclassified	GBIF: https://doi.org/10.15468/dl.zfaw79
Anopheles gambiae	1.98288	7.165446	Unclassified	GBIF: https://doi.org/10.15468/dl.zfaw79
Anopheles gambiae	1.986864	11.113346	Unclassified	GBIF: https://doi.org/10.15468/dl.zfaw79
Anopheles gambiae	1.99834	10.9721	Unclassified	GBIF: https://doi.org/10.15468/dl.zfaw79
Anopheles gambiae	1.998343	10.972115	Unclassified	GBIF: https://doi.org/10.15468/dl.zfaw79
Anopheles gambiae	2	7.23333	Unclassified	GBIF: https://doi.org/10.15468/dl.zfaw79
Anopheles gambiae	2.000428	10.230895	Unclassified	GBIF: https://doi.org/10.15468/dl.zfaw79
Anopheles gambiae	2.00043	10.2309	Unclassified	GBIF: https://doi.org/10.15468/dl.zfaw79
Anopheles gambiae	2.004257	11.126861	Unclassified	GBIF: https://doi.org/10.15468/dl.zfaw79
Anopheles gambiae	2.007703	10.737536	Unclassified	GBIF: https://doi.org/10.15468/dl.zfaw79
Anopheles gambiae	2.013701	6.445031	Unclassified	GBIF: https://doi.org/10.15468/dl.zfaw79
Anopheles gambiae	2.01667	8.31667	Unclassified	GBIF: https://doi.org/10.15468/dl.zfaw79
Anopheles gambiae	2.03333	7.5	Unclassified	GBIF: https://doi.org/10.15468/dl.zfaw79
Anopheles gambiae	2.03527	7.314335	Unclassified	GBIF: https://doi.org/10.15468/dl.zfaw79
Anopheles gambiae	2.061312	10.919731	Unclassified	GBIF: https://doi.org/10.15468/dl.zfaw79
Anopheles gambiae	2.069533	10.503028	Unclassified	GBIF: https://doi.org/10.15468/dl.zfaw79
Anopheles gambiae	2.07151	7.18012	Unclassified	GBIF: https://doi.org/10.15468/dl.zfaw79
Anopheles gambiae	2.07795	10.956947	Unclassified	GBIF: https://doi.org/10.15468/dl.zfaw79
Anopheles gambiae	2.078225	10.083646	Unclassified	GBIF: https://doi.org/10.15468/dl.zfaw79

Anopheles gambiae	2.080011	6.36986	Unclassified	GBIF: https://doi.org/10.15468/dl.zfaw79
Anopheles gambiae	2.080063	11.019172	Unclassified	GBIF: https://doi.org/10.15468/dl.zfaw79
Anopheles gambiae	2.0808	7.184004	Unclassified	GBIF: https://doi.org/10.15468/dl.zfaw79
Anopheles gambiae	2.082229	10.193671	Unclassified	GBIF: https://doi.org/10.15468/dl.zfaw79
Anopheles gambiae	2.08333	13.53333	Unclassified	GBIF: https://doi.org/10.15468/dl.zfaw79
Anopheles gambiae	2.084662	10.816691	Unclassified	GBIF: https://doi.org/10.15468/dl.zfaw79
Anopheles gambiae	2.08536	6.366615	Unclassified	GBIF: https://doi.org/10.15468/dl.zfaw79
Anopheles gambiae	2.085987	10.825673	Unclassified	GBIF: https://doi.org/10.15468/dl.zfaw79
Anopheles gambiae	2.088643	10.668004	Unclassified	GBIF: https://doi.org/10.15468/dl.zfaw79
Anopheles gambiae	2.089037	10.47609	Unclassified	GBIF: https://doi.org/10.15468/dl.zfaw79
Anopheles gambiae	2.089815	10.382092	Unclassified	GBIF: https://doi.org/10.15468/dl.zfaw79
Anopheles gambiae	2.094539	10.816786	Unclassified	GBIF: https://doi.org/10.15468/dl.zfaw79
Anopheles gambiae	2.097009	10.759451	Unclassified	GBIF: https://doi.org/10.15468/dl.zfaw79
Anopheles gambiae	2.101	7.236	Unclassified	GBIF: https://doi.org/10.15468/dl.zfaw79
Anopheles gambiae	2.102	7.279	Unclassified	GBIF: https://doi.org/10.15468/dl.zfaw79
Anopheles gambiae	2.110088	10.214117	Unclassified	GBIF: https://doi.org/10.15468/dl.zfaw79
Anopheles gambiae	2.111121	10.240532	Unclassified	GBIF: https://doi.org/10.15468/dl.zfaw79
Anopheles gambiae	2.1123	7.2103	Unclassified	GBIF: https://doi.org/10.15468/dl.zfaw79
Anopheles gambiae	2.1123	7.2055	Unclassified	GBIF: https://doi.org/10.15468/dl.zfaw79
Anopheles gambiae	2.114	7.267	Unclassified	GBIF: https://doi.org/10.15468/dl.zfaw79
Anopheles gambiae	2.116	7.261	Unclassified	GBIF: https://doi.org/10.15468/dl.zfaw79
Anopheles gambiae	2.11606	10.238647	Unclassified	GBIF: https://doi.org/10.15468/dl.zfaw79
Anopheles gambiae	2.118	7.284	Unclassified	GBIF: https://doi.org/10.15468/dl.zfaw79
Anopheles gambiae	2.122	7.253	Unclassified	GBIF: https://doi.org/10.15468/dl.zfaw79
Anopheles gambiae	2.122566	10.620368	Unclassified	GBIF: https://doi.org/10.15468/dl.zfaw79
Anopheles gambiae	2.123923	6.672633	Unclassified	GBIF: https://doi.org/10.15468/dl.zfaw79
Anopheles gambiae	2.125088	9.868283	Unclassified	GBIF: https://doi.org/10.15468/dl.zfaw79
Anopheles gambiae	2.140419	9.868089	Unclassified	GBIF: https://doi.org/10.15468/dl.zfaw79
Anopheles gambiae	2.153621	10.817644	Unclassified	GBIF: https://doi.org/10.15468/dl.zfaw79
Anopheles gambiae	2.15896	6.66233	Unclassified	GBIF: https://doi.org/10.15468/dl.zfaw79
Anopheles gambiae	2.160172	10.769394	Unclassified	GBIF: https://doi.org/10.15468/dl.zfaw79
Anopheles gambiae	2.162895	10.353473	Unclassified	GBIF: https://doi.org/10.15468/dl.zfaw79
Anopheles gambiae	2.169382	6.528158	Unclassified	GBIF: https://doi.org/10.15468/dl.zfaw79
Anopheles gambiae	2.182448	10.682195	Unclassified	GBIF: https://doi.org/10.15468/dl.zfaw79
Anopheles gambiae	2.185	7.78491	Unclassified	GBIF: https://doi.org/10.15468/dl.zfaw79
Anopheles gambiae	2.186538	6.824885	Unclassified	GBIF: https://doi.org/10.15468/dl.zfaw79
Anopheles gambiae	2.190897	7.220267	Unclassified	GBIF: https://doi.org/10.15468/dl.zfaw79
Anopheles gambiae	2.196614	10.705036	Unclassified	GBIF: https://doi.org/10.15468/dl.zfaw79
Anopheles gambiae	2.2	8.21667	Unclassified	GBIF: https://doi.org/10.15468/dl.zfaw79
Anopheles gambiae	2.203066	7.01729	Unclassified	GBIF: https://doi.org/10.15468/dl.zfaw79
Anopheles gambiae	2.20307	7.01729	Unclassified	GBIF: https://doi.org/10.15468/dl.zfaw79
Anopheles gambiae	2.207735	7.205107	Unclassified	GBIF: https://doi.org/10.15468/dl.zfaw79

Anopheles gambiae	2.209028	6.388861	Unclassified	GBIF: https://doi.org/10.15468/dl.zfaw79
Anopheles gambiae	2.215392	11.009224	Unclassified	GBIF: https://doi.org/10.15468/dl.zfaw79
Anopheles gambiae	2.215897	11.008503	Unclassified	GBIF: https://doi.org/10.15468/dl.zfaw79
Anopheles gambiae	2.21667	7.68333	Unclassified	GBIF: https://doi.org/10.15468/dl.zfaw79
Anopheles gambiae	2.229136	8.168117	Unclassified	GBIF: https://doi.org/10.15468/dl.zfaw79
Anopheles gambiae	2.23333	6.61667	Unclassified	GBIF: https://doi.org/10.15468/dl.zfaw79
Anopheles gambiae	2.234783	10.830002	Unclassified	GBIF: https://doi.org/10.15468/dl.zfaw79
Anopheles gambiae	2.235351	10.833814	Unclassified	GBIF: https://doi.org/10.15468/dl.zfaw79
Anopheles gambiae	2.244512	10.374981	Unclassified	GBIF: https://doi.org/10.15468/dl.zfaw79
Anopheles gambiae	2.246915	10.359875	Unclassified	GBIF: https://doi.org/10.15468/dl.zfaw79
Anopheles gambiae	2.250963	10.358211	Unclassified	GBIF: https://doi.org/10.15468/dl.zfaw79
Anopheles gambiae	2.251118	10.952699	Unclassified	GBIF: https://doi.org/10.15468/dl.zfaw79
Anopheles gambiae	2.252289	10.203056	Unclassified	GBIF: https://doi.org/10.15468/dl.zfaw79
Anopheles gambiae	2.253785	10.57653	Unclassified	GBIF: https://doi.org/10.15468/dl.zfaw79
Anopheles gambiae	2.254203	10.494477	Unclassified	GBIF: https://doi.org/10.15468/dl.zfaw79
Anopheles gambiae	2.256284	9.990865	Unclassified	GBIF: https://doi.org/10.15468/dl.zfaw79
Anopheles gambiae	2.257111	6.413865	Unclassified	GBIF: https://doi.org/10.15468/dl.zfaw79
Anopheles gambiae	2.266978	9.902223	Unclassified	GBIF: https://doi.org/10.15468/dl.zfaw79
Anopheles gambiae	2.267	6.45243	Unclassified	GBIF: https://doi.org/10.15468/dl.zfaw79
Anopheles gambiae	2.268118	11.322699	Unclassified	GBIF: https://doi.org/10.15468/dl.zfaw79
Anopheles gambiae	2.268218	6.386814	Unclassified	GBIF: https://doi.org/10.15468/dl.zfaw79
Anopheles gambiae	2.269355	7.690645	Unclassified	GBIF: https://doi.org/10.15468/dl.zfaw79
Anopheles gambiae	2.269841	9.912335	Unclassified	GBIF: https://doi.org/10.15468/dl.zfaw79
Anopheles gambiae	2.271753	9.712536	Unclassified	GBIF: https://doi.org/10.15468/dl.zfaw79
Anopheles gambiae	2.274288	10.202922	Unclassified	GBIF: https://doi.org/10.15468/dl.zfaw79
Anopheles gambiae	2.274539	10.315615	Unclassified	GBIF: https://doi.org/10.15468/dl.zfaw79
Anopheles gambiae	2.283011	10.447031	Unclassified	GBIF: https://doi.org/10.15468/dl.zfaw79
Anopheles gambiae	2.28335	6.38649	Unclassified	GBIF: https://doi.org/10.15468/dl.zfaw79
Anopheles gambiae	2.2906	6.418725	Unclassified	GBIF: https://doi.org/10.15468/dl.zfaw79
Anopheles gambiae	2.297312	10.210667	Unclassified	GBIF: https://doi.org/10.15468/dl.zfaw79
Anopheles gambiae	2.3	6.88333	Unclassified	GBIF: https://doi.org/10.15468/dl.zfaw79
Anopheles gambiae	2.301118	9.652699	Unclassified	GBIF: https://doi.org/10.15468/dl.zfaw79
Anopheles gambiae	2.301666	6.38639	Unclassified	GBIF: https://doi.org/10.15468/dl.zfaw79
Anopheles gambiae	2.304783	10.191112	Unclassified	GBIF: https://doi.org/10.15468/dl.zfaw79
Anopheles gambiae	2.306228	6.41537	Unclassified	GBIF: https://doi.org/10.15468/dl.zfaw79
Anopheles gambiae	2.316	7.207	Unclassified	GBIF: https://doi.org/10.15468/dl.zfaw79
Anopheles gambiae	2.31775	6.42879	Unclassified	GBIF: https://doi.org/10.15468/dl.zfaw79
Anopheles gambiae	2.321	7.209	Unclassified	GBIF: https://doi.org/10.15468/dl.zfaw79
Anopheles gambiae	2.322368	9.958642	Unclassified	GBIF: https://doi.org/10.15468/dl.zfaw79
Anopheles gambiae	2.32782	6.375353	Unclassified	GBIF: https://doi.org/10.15468/dl.zfaw79
Anopheles gambiae	2.329986	6.406409	Unclassified	GBIF: https://doi.org/10.15468/dl.zfaw79
Anopheles gambiae	2.331118	10.082699	Unclassified	GBIF: https://doi.org/10.15468/dl.zfaw79

Anopheles gambiae	2.332907	6.730784	Unclassified	GBIF: https://doi.org/10.15468/dl.zfaw79
Anopheles gambiae	2.33545	9.608558	Unclassified	GBIF: https://doi.org/10.15468/dl.zfaw79
Anopheles gambiae	2.33575	9.18852	Unclassified	GBIF: https://doi.org/10.15468/dl.zfaw79
Anopheles gambiae	2.339644	10.995341	Unclassified	GBIF: https://doi.org/10.15468/dl.zfaw79
Anopheles gambiae	2.342	7.213	Unclassified	GBIF: https://doi.org/10.15468/dl.zfaw79
Anopheles gambiae	2.344134	6.3857	Unclassified	GBIF: https://doi.org/10.15468/dl.zfaw79
Anopheles gambiae	2.3444	6.43097	Unclassified	GBIF: https://doi.org/10.15468/dl.zfaw79
Anopheles gambiae	2.349145	9.296952	Unclassified	GBIF: https://doi.org/10.15468/dl.zfaw79
Anopheles gambiae	2.351394	9.355919	Unclassified	GBIF: https://doi.org/10.15468/dl.zfaw79
Anopheles gambiae	2.351978	10.225725	Unclassified	GBIF: https://doi.org/10.15468/dl.zfaw79
Anopheles gambiae	2.353145	7.214672	Unclassified	GBIF: https://doi.org/10.15468/dl.zfaw79
Anopheles gambiae	2.35424	6.45386	Unclassified	GBIF: https://doi.org/10.15468/dl.zfaw79
Anopheles gambiae	2.354706	6.518871	Unclassified	GBIF: https://doi.org/10.15468/dl.zfaw79
Anopheles gambiae	2.35567	7.457635	Unclassified	GBIF: https://doi.org/10.15468/dl.zfaw79
Anopheles gambiae	2.356338	7.361585	Unclassified	GBIF: https://doi.org/10.15468/dl.zfaw79
Anopheles gambiae	2.3568	6.61338	Unclassified	GBIF: https://doi.org/10.15468/dl.zfaw79
Anopheles gambiae	2.358888	9.540389	Unclassified	GBIF: https://doi.org/10.15468/dl.zfaw79
Anopheles gambiae	2.35942	7.639393	Unclassified	GBIF: https://doi.org/10.15468/dl.zfaw79
Anopheles gambiae	2.360398	8.515252	Unclassified	GBIF: https://doi.org/10.15468/dl.zfaw79
Anopheles gambiae	2.360513	9.532755	Unclassified	GBIF: https://doi.org/10.15468/dl.zfaw79
Anopheles gambiae	2.3629	6.39148	Unclassified	GBIF: https://doi.org/10.15468/dl.zfaw79
Anopheles gambiae	2.362966	10.228235	Unclassified	GBIF: https://doi.org/10.15468/dl.zfaw79
Anopheles gambiae	2.36389	6.38375	Unclassified	GBIF: https://doi.org/10.15468/dl.zfaw79
Anopheles gambiae	2.36454	6.51595	Unclassified	GBIF: https://doi.org/10.15468/dl.zfaw79
Anopheles gambiae	2.365505	10.229711	Unclassified	GBIF: https://doi.org/10.15468/dl.zfaw79
Anopheles gambiae	2.365555	6.393082	Unclassified	GBIF: https://doi.org/10.15468/dl.zfaw79
Anopheles gambiae	2.367528	10.230683	Unclassified	GBIF: https://doi.org/10.15468/dl.zfaw79
Anopheles gambiae	2.367957	10.228293	Unclassified	GBIF: https://doi.org/10.15468/dl.zfaw79
Anopheles gambiae	2.367979	10.260695	Unclassified	GBIF: https://doi.org/10.15468/dl.zfaw79
Anopheles gambiae	2.368577	6.36101	Unclassified	GBIF: https://doi.org/10.15468/dl.zfaw79
Anopheles gambiae	2.368901	10.232016	Unclassified	GBIF: https://doi.org/10.15468/dl.zfaw79
Anopheles gambiae	2.36974	10.232335	Unclassified	GBIF: https://doi.org/10.15468/dl.zfaw79
Anopheles gambiae	2.369741	10.232336	Unclassified	GBIF: https://doi.org/10.15468/dl.zfaw79
Anopheles gambiae	2.369742	10.232337	Unclassified	GBIF: https://doi.org/10.15468/dl.zfaw79
Anopheles gambiae	2.369743	10.232338	Unclassified	GBIF: https://doi.org/10.15468/dl.zfaw79
Anopheles gambiae	2.369744	10.232339	Unclassified	GBIF: https://doi.org/10.15468/dl.zfaw79
Anopheles gambiae	2.369745	10.23234	Unclassified	GBIF: https://doi.org/10.15468/dl.zfaw79
Anopheles gambiae	2.369746	10.232341	Unclassified	GBIF: https://doi.org/10.15468/dl.zfaw79
Anopheles gambiae	2.369747	10.232342	Unclassified	GBIF: https://doi.org/10.15468/dl.zfaw79
Anopheles gambiae	2.369753	10.232348	Unclassified	GBIF: https://doi.org/10.15468/dl.zfaw79
Anopheles gambiae	2.369759	10.232354	Unclassified	GBIF: https://doi.org/10.15468/dl.zfaw79
Anopheles gambiae	2.370336	7.286118	Unclassified	GBIF: https://doi.org/10.15468/dl.zfaw79

Anopheles gambiae	2.372077	10.2332	Unclassified	GBIF: https://doi.org/10.15468/dl.zfaw79
Anopheles gambiae	2.372843	8.25037	Unclassified	GBIF: https://doi.org/10.15468/dl.zfaw79
Anopheles gambiae	2.375119	11.266559	Unclassified	GBIF: https://doi.org/10.15468/dl.zfaw79
Anopheles gambiae	2.37548	10.318528	Unclassified	GBIF: https://doi.org/10.15468/dl.zfaw79
Anopheles gambiae	2.378055	6.38921	Unclassified	GBIF: https://doi.org/10.15468/dl.zfaw79
Anopheles gambiae	2.381082	7.113431	Unclassified	GBIF: https://doi.org/10.15468/dl.zfaw79
Anopheles gambiae	2.381118	9.572699	Unclassified	GBIF: https://doi.org/10.15468/dl.zfaw79
Anopheles gambiae	2.382073	7.219811	Unclassified	GBIF: https://doi.org/10.15468/dl.zfaw79
Anopheles gambiae	2.383927	10.182364	Unclassified	GBIF: https://doi.org/10.15468/dl.zfaw79
Anopheles gambiae	2.386481	7.396785	Unclassified	GBIF: https://doi.org/10.15468/dl.zfaw79
Anopheles gambiae	2.38863	6.379177	Unclassified	GBIF: https://doi.org/10.15468/dl.zfaw79
Anopheles gambiae	2.388816	10.190611	Unclassified	GBIF: https://doi.org/10.15468/dl.zfaw79
Anopheles gambiae	2.394289	6.39252	Unclassified	GBIF: https://doi.org/10.15468/dl.zfaw79
Anopheles gambiae	2.394321	10.853746	Unclassified	GBIF: https://doi.org/10.15468/dl.zfaw79
Anopheles gambiae	2.394807	10.856332	Unclassified	GBIF: https://doi.org/10.15468/dl.zfaw79
Anopheles gambiae	2.396479	7.198953	Unclassified	GBIF: https://doi.org/10.15468/dl.zfaw79
Anopheles gambiae	2.397144	10.995174	Unclassified	GBIF: https://doi.org/10.15468/dl.zfaw79
Anopheles gambiae	2.39937	10.940976	Unclassified	GBIF: https://doi.org/10.15468/dl.zfaw79
Anopheles gambiae	2.399818	6.397883	Unclassified	GBIF: https://doi.org/10.15468/dl.zfaw79
Anopheles gambiae	2.4	7.26667	Unclassified	GBIF: https://doi.org/10.15468/dl.zfaw79
Anopheles gambiae	2.400537	6.496973	Unclassified	GBIF: https://doi.org/10.15468/dl.zfaw79
Anopheles gambiae	2.400539	6.49697	Unclassified	GBIF: https://doi.org/10.15468/dl.zfaw79
Anopheles gambiae	2.402	7.202	Unclassified	GBIF: https://doi.org/10.15468/dl.zfaw79
Anopheles gambiae	2.402102	6.368819	Unclassified	GBIF: https://doi.org/10.15468/dl.zfaw79
Anopheles gambiae	2.403394	7.469922	Unclassified	GBIF: https://doi.org/10.15468/dl.zfaw79
Anopheles gambiae	2.403538	7.470004	Unclassified	GBIF: https://doi.org/10.15468/dl.zfaw79
Anopheles gambiae	2.404481	10.379116	Unclassified	GBIF: https://doi.org/10.15468/dl.zfaw79
Anopheles gambiae	2.404814	7.470767	Unclassified	GBIF: https://doi.org/10.15468/dl.zfaw79
Anopheles gambiae	2.405305	6.39289	Unclassified	GBIF: https://doi.org/10.15468/dl.zfaw79
Anopheles gambiae	2.40559	6.36648	Unclassified	GBIF: https://doi.org/10.15468/dl.zfaw79
Anopheles gambiae	2.407	7.207	Unclassified	GBIF: https://doi.org/10.15468/dl.zfaw79
Anopheles gambiae	2.407013	10.121451	Unclassified	GBIF: https://doi.org/10.15468/dl.zfaw79
Anopheles gambiae	2.408556	6.356606	Unclassified	GBIF: https://doi.org/10.15468/dl.zfaw79
Anopheles gambiae	2.410341	10.278695	Unclassified	GBIF: https://doi.org/10.15468/dl.zfaw79
Anopheles gambiae	2.413573	6.372798	Unclassified	GBIF: https://doi.org/10.15468/dl.zfaw79
Anopheles gambiae	2.41606	7.424792	Unclassified	GBIF: https://doi.org/10.15468/dl.zfaw79
Anopheles gambiae	2.41833	6.36536	Unclassified	GBIF: https://doi.org/10.15468/dl.zfaw79
Anopheles gambiae	2.42	7.222	Unclassified	GBIF: https://doi.org/10.15468/dl.zfaw79
Anopheles gambiae	2.42001	7.436614	Unclassified	GBIF: https://doi.org/10.15468/dl.zfaw79
Anopheles gambiae	2.420816	7.57306	Unclassified	GBIF: https://doi.org/10.15468/dl.zfaw79
Anopheles gambiae	2.421	7.0963	Unclassified	GBIF: https://doi.org/10.15468/dl.zfaw79
Anopheles gambiae	2.421	7.0988	Unclassified	GBIF: https://doi.org/10.15468/dl.zfaw79

Anopheles gambiae	2.422536	7.459809	Unclassified	GBIF: https://doi.org/10.15468/dl.zfaw79
Anopheles gambiae	2.423011	10.335973	Unclassified	GBIF: https://doi.org/10.15468/dl.zfaw79
Anopheles gambiae	2.423392	7.370088	Unclassified	GBIF: https://doi.org/10.15468/dl.zfaw79
Anopheles gambiae	2.424928	6.363348	Unclassified	GBIF: https://doi.org/10.15468/dl.zfaw79
Anopheles gambiae	2.425092	7.18378	Unclassified	GBIF: https://doi.org/10.15468/dl.zfaw79
Anopheles gambiae	2.42515	7.35867	Unclassified	GBIF: https://doi.org/10.15468/dl.zfaw79
Anopheles gambiae	2.425251	6.367695	Unclassified	GBIF: https://doi.org/10.15468/dl.zfaw79
Anopheles gambiae	2.425366	7.426924	Unclassified	GBIF: https://doi.org/10.15468/dl.zfaw79
Anopheles gambiae	2.42854	6.38347	Unclassified	GBIF: https://doi.org/10.15468/dl.zfaw79
Anopheles gambiae	2.429143	10.257977	Unclassified	GBIF: https://doi.org/10.15468/dl.zfaw79
Anopheles gambiae	2.429297	11.090846	Unclassified	GBIF: https://doi.org/10.15468/dl.zfaw79
Anopheles gambiae	2.429983	10.424725	Unclassified	GBIF: https://doi.org/10.15468/dl.zfaw79
Anopheles gambiae	2.431392	9.534752	Unclassified	GBIF: https://doi.org/10.15468/dl.zfaw79
Anopheles gambiae	2.432031	7.469367	Unclassified	GBIF: https://doi.org/10.15468/dl.zfaw79
Anopheles gambiae	2.436017	6.369314	Unclassified	GBIF: https://doi.org/10.15468/dl.zfaw79
Anopheles gambiae	2.436029	6.362597	Unclassified	GBIF: https://doi.org/10.15468/dl.zfaw79
Anopheles gambiae	2.439782	7.396223	Unclassified	GBIF: https://doi.org/10.15468/dl.zfaw79
Anopheles gambiae	2.440586	11.096143	Unclassified	GBIF: https://doi.org/10.15468/dl.zfaw79
Anopheles gambiae	2.440952	7.364283	Unclassified	GBIF: https://doi.org/10.15468/dl.zfaw79
Anopheles gambiae	2.44365	6.37798	Unclassified	GBIF: https://doi.org/10.15468/dl.zfaw79
Anopheles gambiae	2.4439	6.3943	Unclassified	GBIF: https://doi.org/10.15468/dl.zfaw79
Anopheles gambiae	2.444811	7.112501	Unclassified	GBIF: https://doi.org/10.15468/dl.zfaw79
Anopheles gambiae	2.44537	10.831006	Unclassified	GBIF: https://doi.org/10.15468/dl.zfaw79
Anopheles gambiae	2.445812	10.211975	Unclassified	GBIF: https://doi.org/10.15468/dl.zfaw79
Anopheles gambiae	2.449116	7.405228	Unclassified	GBIF: https://doi.org/10.15468/dl.zfaw79
Anopheles gambiae	2.45601	8.517613	Unclassified	GBIF: https://doi.org/10.15468/dl.zfaw79
Anopheles gambiae	2.458347	6.391753	Unclassified	GBIF: https://doi.org/10.15468/dl.zfaw79
Anopheles gambiae	2.458618	10.230999	Unclassified	GBIF: https://doi.org/10.15468/dl.zfaw79
Anopheles gambiae	2.459283	7.371783	Unclassified	GBIF: https://doi.org/10.15468/dl.zfaw79
Anopheles gambiae	2.460588	7.527007	Unclassified	GBIF: https://doi.org/10.15468/dl.zfaw79
Anopheles gambiae	2.463361	9.688334	Unclassified	GBIF: https://doi.org/10.15468/dl.zfaw79
Anopheles gambiae	2.46442	6.36483	Unclassified	GBIF: https://doi.org/10.15468/dl.zfaw79
Anopheles gambiae	2.46472	6.365	Unclassified	GBIF: https://doi.org/10.15468/dl.zfaw79
Anopheles gambiae	2.465503	7.039169	Unclassified	GBIF: https://doi.org/10.15468/dl.zfaw79
Anopheles gambiae	2.466714	6.890962	Unclassified	GBIF: https://doi.org/10.15468/dl.zfaw79
Anopheles gambiae	2.470535	7.527558	Unclassified	GBIF: https://doi.org/10.15468/dl.zfaw79
Anopheles gambiae	2.471787	10.136697	Unclassified	GBIF: https://doi.org/10.15468/dl.zfaw79
Anopheles gambiae	2.473041	11.326254	Unclassified	GBIF: https://doi.org/10.15468/dl.zfaw79
Anopheles gambiae	2.473842	10.429147	Unclassified	GBIF: https://doi.org/10.15468/dl.zfaw79
Anopheles gambiae	2.474448	10.272005	Unclassified	GBIF: https://doi.org/10.15468/dl.zfaw79
Anopheles gambiae	2.47745	10.238648	Unclassified	GBIF: https://doi.org/10.15468/dl.zfaw79
Anopheles gambiae	2.479257	10.412447	Unclassified	GBIF: https://doi.org/10.15468/dl.zfaw79

Anopheles gambiae	2.481461	10.413388	Unclassified	GBIF: https://doi.org/10.15468/dl.zfaw79
Anopheles gambiae	2.483033	10.332731	Unclassified	GBIF: https://doi.org/10.15468/dl.zfaw79
Anopheles gambiae	2.483591	7.082361	Unclassified	GBIF: https://doi.org/10.15468/dl.zfaw79
Anopheles gambiae	2.485	8.03361	Unclassified	GBIF: https://doi.org/10.15468/dl.zfaw79
Anopheles gambiae	2.485725	7.970865	Unclassified	GBIF: https://doi.org/10.15468/dl.zfaw79
Anopheles gambiae	2.487533	7.321814	Unclassified	GBIF: https://doi.org/10.15468/dl.zfaw79
Anopheles gambiae	2.490065	7.057009	Unclassified	GBIF: https://doi.org/10.15468/dl.zfaw79
Anopheles gambiae	2.492502	7.520976	Unclassified	GBIF: https://doi.org/10.15468/dl.zfaw79
Anopheles gambiae	2.494	7.025	Unclassified	GBIF: https://doi.org/10.15468/dl.zfaw79
Anopheles gambiae	2.494418	6.796871	Unclassified	GBIF: https://doi.org/10.15468/dl.zfaw79
Anopheles gambiae	2.498	7.031	Unclassified	GBIF: https://doi.org/10.15468/dl.zfaw79
Anopheles gambiae	2.503	7.035	Unclassified	GBIF: https://doi.org/10.15468/dl.zfaw79
Anopheles gambiae	2.504	7.04	Unclassified	GBIF: https://doi.org/10.15468/dl.zfaw79
Anopheles gambiae	2.505865	7.387258	Unclassified	GBIF: https://doi.org/10.15468/dl.zfaw79
Anopheles gambiae	2.510033	7.377618	Unclassified	GBIF: https://doi.org/10.15468/dl.zfaw79
Anopheles gambiae	2.515448	6.566207	Unclassified	GBIF: https://doi.org/10.15468/dl.zfaw79
Anopheles gambiae	2.51641	6.69813	Unclassified	GBIF: https://doi.org/10.15468/dl.zfaw79
Anopheles gambiae	2.516411	6.698138	Unclassified	GBIF: https://doi.org/10.15468/dl.zfaw79
Anopheles gambiae	2.51667	6.4	Unclassified	GBIF: https://doi.org/10.15468/dl.zfaw79
Anopheles gambiae	2.517367	7.357676	Unclassified	GBIF: https://doi.org/10.15468/dl.zfaw79
Anopheles gambiae	2.521289	7.400446	Unclassified	GBIF: https://doi.org/10.15468/dl.zfaw79
Anopheles gambiae	2.522536	7.390148	Unclassified	GBIF: https://doi.org/10.15468/dl.zfaw79
Anopheles gambiae	2.524621	7.050855	Unclassified	GBIF: https://doi.org/10.15468/dl.zfaw79
Anopheles gambiae	2.525098	6.812699	Unclassified	GBIF: https://doi.org/10.15468/dl.zfaw79
Anopheles gambiae	2.52759	8.448384	Unclassified	GBIF: https://doi.org/10.15468/dl.zfaw79
Anopheles gambiae	2.528864	7.303366	Unclassified	GBIF: https://doi.org/10.15468/dl.zfaw79
Anopheles gambiae	2.531642	7.42234	Unclassified	GBIF: https://doi.org/10.15468/dl.zfaw79
Anopheles gambiae	2.53628	6.984393	Unclassified	GBIF: https://doi.org/10.15468/dl.zfaw79
Anopheles gambiae	2.539583	6.484872	Unclassified	GBIF: https://doi.org/10.15468/dl.zfaw79
Anopheles gambiae	2.539992	6.803979	Unclassified	GBIF: https://doi.org/10.15468/dl.zfaw79
Anopheles gambiae	2.541758	7.985217	Unclassified	GBIF: https://doi.org/10.15468/dl.zfaw79
Anopheles gambiae	2.545615	11.363062	Unclassified	GBIF: https://doi.org/10.15468/dl.zfaw79
Anopheles gambiae	2.546564	9.211232	Unclassified	GBIF: https://doi.org/10.15468/dl.zfaw79
Anopheles gambiae	2.55031	8.906223	Unclassified	GBIF: https://doi.org/10.15468/dl.zfaw79
Anopheles gambiae	2.552755	7.063948	Unclassified	GBIF: https://doi.org/10.15468/dl.zfaw79
Anopheles gambiae	2.554757	10.370618	Unclassified	GBIF: https://doi.org/10.15468/dl.zfaw79
Anopheles gambiae	2.560089	7.492647	Unclassified	GBIF: https://doi.org/10.15468/dl.zfaw79
Anopheles gambiae	2.57334	7.458645	Unclassified	GBIF: https://doi.org/10.15468/dl.zfaw79
Anopheles gambiae	2.58131	6.39684	Unclassified	GBIF: https://doi.org/10.15468/dl.zfaw79
Anopheles gambiae	2.582978	10.505362	Unclassified	GBIF: https://doi.org/10.15468/dl.zfaw79
Anopheles gambiae	2.585314	6.922873	Unclassified	GBIF: https://doi.org/10.15468/dl.zfaw79
Anopheles gambiae	2.586115	7.129813	Unclassified	GBIF: https://doi.org/10.15468/dl.zfaw79

Anopheles gambiae	2.588095	7.41057	Unclassified	GBIF: https://doi.org/10.15468/dl.zfaw79
Anopheles gambiae	2.590119	7.231589	Unclassified	GBIF: https://doi.org/10.15468/dl.zfaw79
Anopheles gambiae	2.590957	8.932313	Unclassified	GBIF: https://doi.org/10.15468/dl.zfaw79
Anopheles gambiae	2.591449	11.191449	Unclassified	GBIF: https://doi.org/10.15468/dl.zfaw79
Anopheles gambiae	2.595726	7.057722	Unclassified	GBIF: https://doi.org/10.15468/dl.zfaw79
Anopheles gambiae	2.598304	7.011825	Unclassified	GBIF: https://doi.org/10.15468/dl.zfaw79
Anopheles gambiae	2.601979	8.099641	Unclassified	GBIF: https://doi.org/10.15468/dl.zfaw79
Anopheles gambiae	2.60389	7.36028	Unclassified	GBIF: https://doi.org/10.15468/dl.zfaw79
Anopheles gambiae	2.605752	8.055452	Unclassified	GBIF: https://doi.org/10.15468/dl.zfaw79
Anopheles gambiae	2.60948	9.602056	Unclassified	GBIF: https://doi.org/10.15468/dl.zfaw79
Anopheles gambiae	2.609937	6.575072	Unclassified	GBIF: https://doi.org/10.15468/dl.zfaw79
Anopheles gambiae	2.61	7.0036	Unclassified	GBIF: https://doi.org/10.15468/dl.zfaw79
Anopheles gambiae	2.610287	10.118063	Unclassified	GBIF: https://doi.org/10.15468/dl.zfaw79
Anopheles gambiae	2.61098	10.059104	Unclassified	GBIF: https://doi.org/10.15468/dl.zfaw79
Anopheles gambiae	2.615892	7.062062	Unclassified	GBIF: https://doi.org/10.15468/dl.zfaw79
Anopheles gambiae	2.618336	9.943119	Unclassified	GBIF: https://doi.org/10.15468/dl.zfaw79
Anopheles gambiae	2.625336	6.499072	Unclassified	GBIF: https://doi.org/10.15468/dl.zfaw79
Anopheles gambiae	2.626282	6.498036	Unclassified	GBIF: https://doi.org/10.15468/dl.zfaw79
Anopheles gambiae	2.627826	9.340016	Unclassified	GBIF: https://doi.org/10.15468/dl.zfaw79
Anopheles gambiae	2.6285	6.49825	Unclassified	GBIF: https://doi.org/10.15468/dl.zfaw79
Anopheles gambiae	2.63333	7.91667	Unclassified	GBIF: https://doi.org/10.15468/dl.zfaw79
Anopheles gambiae	2.636947	7.867309	Unclassified	GBIF: https://doi.org/10.15468/dl.zfaw79
Anopheles gambiae	2.637336	9.624557	Unclassified	GBIF: https://doi.org/10.15468/dl.zfaw79
Anopheles gambiae	2.640176	7.280586	Unclassified	GBIF: https://doi.org/10.15468/dl.zfaw79
Anopheles gambiae	2.646612	11.437866	Unclassified	GBIF: https://doi.org/10.15468/dl.zfaw79
Anopheles gambiae	2.649559	10.270591	Unclassified	GBIF: https://doi.org/10.15468/dl.zfaw79
Anopheles gambiae	2.65	8.28333	Unclassified	GBIF: https://doi.org/10.15468/dl.zfaw79
Anopheles gambiae	2.65161	6.55717	Unclassified	GBIF: https://doi.org/10.15468/dl.zfaw79
Anopheles gambiae	2.653199	11.529533	Unclassified	GBIF: https://doi.org/10.15468/dl.zfaw79
Anopheles gambiae	2.66177	7.114763	Unclassified	GBIF: https://doi.org/10.15468/dl.zfaw79
Anopheles gambiae	2.661899	9.515254	Unclassified	GBIF: https://doi.org/10.15468/dl.zfaw79
Anopheles gambiae	2.66212	10.182258	Unclassified	GBIF: https://doi.org/10.15468/dl.zfaw79
Anopheles gambiae	2.664606	6.560953	Unclassified	GBIF: https://doi.org/10.15468/dl.zfaw79
Anopheles gambiae	2.665312	7.660782	Unclassified	GBIF: https://doi.org/10.15468/dl.zfaw79
Anopheles gambiae	2.667777	7.598679	Unclassified	GBIF: https://doi.org/10.15468/dl.zfaw79
Anopheles gambiae	2.676064	7.380751	Unclassified	GBIF: https://doi.org/10.15468/dl.zfaw79
Anopheles gambiae	2.676551	6.744032	Unclassified	GBIF: https://doi.org/10.15468/dl.zfaw79
Anopheles gambiae	2.677922	7.155308	Unclassified	GBIF: https://doi.org/10.15468/dl.zfaw79
Anopheles gambiae	2.683067	7.058841	Unclassified	GBIF: https://doi.org/10.15468/dl.zfaw79
Anopheles gambiae	2.688955	11.511759	Unclassified	GBIF: https://doi.org/10.15468/dl.zfaw79
Anopheles gambiae	2.691205	7.699514	Unclassified	GBIF: https://doi.org/10.15468/dl.zfaw79
Anopheles gambiae	2.693338	6.493028	Unclassified	GBIF: https://doi.org/10.15468/dl.zfaw79

Anopheles gambiae	2.695325	6.490042	Unclassified	GBIF: https://doi.org/10.15468/dl.zfaw79
Anopheles gambiae	2.696094	7.065919	Unclassified	GBIF: https://doi.org/10.15468/dl.zfaw79
Anopheles gambiae	2.696927	7.083169	Unclassified	GBIF: https://doi.org/10.15468/dl.zfaw79
Anopheles gambiae	2.703948	6.902007	Unclassified	GBIF: https://doi.org/10.15468/dl.zfaw79
Anopheles gambiae	2.706586	7.643227	Unclassified	GBIF: https://doi.org/10.15468/dl.zfaw79
Anopheles gambiae	2.710792	6.644475	Unclassified	GBIF: https://doi.org/10.15468/dl.zfaw79
Anopheles gambiae	2.711229	6.966456	Unclassified	GBIF: https://doi.org/10.15468/dl.zfaw79
Anopheles gambiae	2.712142	11.916227	Unclassified	GBIF: https://doi.org/10.15468/dl.zfaw79
Anopheles gambiae	2.718456	10.940306	Unclassified	GBIF: https://doi.org/10.15468/dl.zfaw79
Anopheles gambiae	2.719282	6.911642	Unclassified	GBIF: https://doi.org/10.15468/dl.zfaw79
Anopheles gambiae	2.725197	7.705557	Unclassified	GBIF: https://doi.org/10.15468/dl.zfaw79
Anopheles gambiae	2.726066	7.637588	Unclassified	GBIF: https://doi.org/10.15468/dl.zfaw79
Anopheles gambiae	2.7272	7.124338	Unclassified	GBIF: https://doi.org/10.15468/dl.zfaw79
Anopheles gambiae	2.729095	10.852273	Unclassified	GBIF: https://doi.org/10.15468/dl.zfaw79
Anopheles gambiae	2.734839	7.719895	Unclassified	GBIF: https://doi.org/10.15468/dl.zfaw79
Anopheles gambiae	2.735893	9.976279	Unclassified	GBIF: https://doi.org/10.15468/dl.zfaw79
Anopheles gambiae	2.750743	10.253959	Unclassified	GBIF: https://doi.org/10.15468/dl.zfaw79
Anopheles gambiae	2.75928	11.0863	Unclassified	GBIF: https://doi.org/10.15468/dl.zfaw79
Anopheles gambiae	2.761118	9.672699	Unclassified	GBIF: https://doi.org/10.15468/dl.zfaw79
Anopheles gambiae	2.765861	9.955896	Unclassified	GBIF: https://doi.org/10.15468/dl.zfaw79
Anopheles gambiae	2.766311	9.954223	Unclassified	GBIF: https://doi.org/10.15468/dl.zfaw79
Anopheles gambiae	2.771288	11.287729	Unclassified	GBIF: https://doi.org/10.15468/dl.zfaw79
Anopheles gambiae	2.781282	10.68359	Unclassified	GBIF: https://doi.org/10.15468/dl.zfaw79
Anopheles gambiae	2.922232	10.828148	Unclassified	GBIF: https://doi.org/10.15468/dl.zfaw79
Anopheles gambiae	2.925837	11.582314	Unclassified	GBIF: https://doi.org/10.15468/dl.zfaw79
Anopheles gambiae	2.940209	11.131597	Unclassified	GBIF: https://doi.org/10.15468/dl.zfaw79
Anopheles gambiae	2.96667	6.41667	Unclassified	GBIF: https://doi.org/10.15468/dl.zfaw79
Anopheles gambiae	2.980285	9.725445	Unclassified	GBIF: https://doi.org/10.15468/dl.zfaw79
Anopheles gambiae	2.984448	11.706029	Unclassified	GBIF: https://doi.org/10.15468/dl.zfaw79
Anopheles gambiae	2.987226	9.352531	Unclassified	GBIF: https://doi.org/10.15468/dl.zfaw79
Anopheles gambiae	2.996725	11.66962	Unclassified	GBIF: https://doi.org/10.15468/dl.zfaw79
Anopheles gambiae	3	6.41667	Unclassified	GBIF: https://doi.org/10.15468/dl.zfaw79
Anopheles gambiae	3.002922	11.701476	Unclassified	GBIF: https://doi.org/10.15468/dl.zfaw79
Anopheles gambiae	3.020617	11.724975	Unclassified	GBIF: https://doi.org/10.15468/dl.zfaw79
Anopheles gambiae	3.025334	11.856117	Unclassified	GBIF: https://doi.org/10.15468/dl.zfaw79
Anopheles gambiae	3.029524	9.605063	Unclassified	GBIF: https://doi.org/10.15468/dl.zfaw79
Anopheles gambiae	3.03009	11.807227	Unclassified	GBIF: https://doi.org/10.15468/dl.zfaw79
Anopheles gambiae	3.052227	11.859618	Unclassified	GBIF: https://doi.org/10.15468/dl.zfaw79
Anopheles gambiae	3.099199	11.816979	Unclassified	GBIF: https://doi.org/10.15468/dl.zfaw79
Anopheles gambiae	3.103336	9.549147	Unclassified	GBIF: https://doi.org/10.15468/dl.zfaw79
Anopheles gambiae	3.124759	11.809506	Unclassified	GBIF: https://doi.org/10.15468/dl.zfaw79
Anopheles gambiae	3.134426	11.902481	Unclassified	GBIF: https://doi.org/10.15468/dl.zfaw79

Anopheles gambiae	3.137808	11.818538	Unclassified	GBIF: https://doi.org/10.15468/dl.zfaw79
Anopheles gambiae	3.138414	11.822238	Unclassified	GBIF: https://doi.org/10.15468/dl.zfaw79
Anopheles gambiae	3.138947	10.271695	Unclassified	GBIF: https://doi.org/10.15468/dl.zfaw79
Anopheles gambiae	3.139267	11.822915	Unclassified	GBIF: https://doi.org/10.15468/dl.zfaw79
Anopheles gambiae	3.1682	12.063923	Unclassified	GBIF: https://doi.org/10.15468/dl.zfaw79
Anopheles gambiae	3.171891	11.854033	Unclassified	GBIF: https://doi.org/10.15468/dl.zfaw79
Anopheles gambiae	3.17267	11.888779	Unclassified	GBIF: https://doi.org/10.15468/dl.zfaw79
Anopheles gambiae	3.175392	11.978585	Unclassified	GBIF: https://doi.org/10.15468/dl.zfaw79
Anopheles gambiae	3.201257	10.476058	Unclassified	GBIF: https://doi.org/10.15468/dl.zfaw79
Anopheles gambiae	3.222483	11.95953	Unclassified	GBIF: https://doi.org/10.15468/dl.zfaw79
Anopheles gambiae	3.227615	11.905142	Unclassified	GBIF: https://doi.org/10.15468/dl.zfaw79
Anopheles gambiae	3.237642	12.00706	Unclassified	GBIF: https://doi.org/10.15468/dl.zfaw79
Anopheles gambiae	3.253331	9.985374	Unclassified	GBIF: https://doi.org/10.15468/dl.zfaw79
Anopheles gambiae	3.254117	10.277979	Unclassified	GBIF: https://doi.org/10.15468/dl.zfaw79
Anopheles gambiae	3.255482	9.72892	Unclassified	GBIF: https://doi.org/10.15468/dl.zfaw79
Anopheles gambiae	3.259892	11.659446	Unclassified	GBIF: https://doi.org/10.15468/dl.zfaw79
Anopheles gambiae	3.28333	6.43333	Unclassified	GBIF: https://doi.org/10.15468/dl.zfaw79
Anopheles gambiae	3.290451	9.866116	Unclassified	GBIF: https://doi.org/10.15468/dl.zfaw79
Anopheles gambiae	3.322422	9.918837	Unclassified	GBIF: https://doi.org/10.15468/dl.zfaw79
Anopheles gambiae	3.33642	9.866039	Unclassified	GBIF: https://doi.org/10.15468/dl.zfaw79
Anopheles gambiae	3.339017	10.310299	Unclassified	GBIF: https://doi.org/10.15468/dl.zfaw79
Anopheles gambiae	3.366964	10.742929	Unclassified	GBIF: https://doi.org/10.15468/dl.zfaw79
Anopheles gambiae	3.37556	10.757562	Unclassified	GBIF: https://doi.org/10.15468/dl.zfaw79
Anopheles gambiae	3.379274	10.288073	Unclassified	GBIF: https://doi.org/10.15468/dl.zfaw79
Anopheles gambiae	3.381118	9.822699	Unclassified	GBIF: https://doi.org/10.15468/dl.zfaw79
Anopheles gambiae	3.38425	11.8633	Unclassified	GBIF: https://doi.org/10.15468/dl.zfaw79
Anopheles gambiae	3.399756	10.272423	Unclassified	GBIF: https://doi.org/10.15468/dl.zfaw79
Anopheles gambiae	3.420675	11.305085	Unclassified	GBIF: https://doi.org/10.15468/dl.zfaw79
Anopheles gambiae	3.424451	11.336842	Unclassified	GBIF: https://doi.org/10.15468/dl.zfaw79
Anopheles gambiae	3.425927	11.512948	Unclassified	GBIF: https://doi.org/10.15468/dl.zfaw79
Anopheles gambiae	3.435616	10.929169	Unclassified	GBIF: https://doi.org/10.15468/dl.zfaw79
Anopheles gambiae	3.446808	9.971453	Unclassified	GBIF: https://doi.org/10.15468/dl.zfaw79
Anopheles gambiae	3.478534	11.52312	Unclassified	GBIF: https://doi.org/10.15468/dl.zfaw79
Anopheles gambiae	3.484118	10.422699	Unclassified	GBIF: https://doi.org/10.15468/dl.zfaw79
Anopheles gambiae	3.498149	11.046338	Unclassified	GBIF: https://doi.org/10.15468/dl.zfaw79
Anopheles gambiae	3.524478	10.887419	Unclassified	GBIF: https://doi.org/10.15468/dl.zfaw79
Anopheles gambiae	3.556731	11.641978	Unclassified	GBIF: https://doi.org/10.15468/dl.zfaw79
Anopheles gambiae	3.576785	10.720728	Unclassified	GBIF: https://doi.org/10.15468/dl.zfaw79
Anopheles gambiae	3.695206	10.928699	Unclassified	GBIF: https://doi.org/10.15468/dl.zfaw79
Anopheles gambiae	3.9	7.4	Unclassified	GBIF: https://doi.org/10.15468/dl.zfaw79
Anopheles gambiae	3.93333	7.38333	Unclassified	GBIF: https://doi.org/10.15468/dl.zfaw79
Anopheles gambiae	4.18333	12.5	Unclassified	GBIF: https://doi.org/10.15468/dl.zfaw79

Anopheles gambiae	4.74	10.84	Unclassified	GBIF: https://doi.org/10.15468/dl.zfaw79
Anopheles gambiae	5.68333	6.31667	Unclassified	GBIF: https://doi.org/10.15468/dl.zfaw79
Anopheles gambiae	5.76667	5.91667	Unclassified	GBIF: https://doi.org/10.15468/dl.zfaw79
Anopheles gambiae	6.26667	7.01667	Unclassified	GBIF: https://doi.org/10.15468/dl.zfaw79
Anopheles gambiae	6.65	0.2	Unclassified	GBIF: https://doi.org/10.15468/dl.zfaw79
Anopheles gambiae	7.1	9.16667	Unclassified	GBIF: https://doi.org/10.15468/dl.zfaw79
Anopheles gambiae	7.23333	10.38333	Unclassified	GBIF: https://doi.org/10.15468/dl.zfaw79
Anopheles gambiae	7.33333	10.5	Unclassified	GBIF: https://doi.org/10.15468/dl.zfaw79
Anopheles gambiae	7.48333	6.33333	Unclassified	GBIF: https://doi.org/10.15468/dl.zfaw79
Anopheles gambiae	7.65	11.8	Unclassified	GBIF: https://doi.org/10.15468/dl.zfaw79
Anopheles gambiae	7.7	6.76667	Unclassified	GBIF: https://doi.org/10.15468/dl.zfaw79
Anopheles gambiae	7.71667	6.3	Unclassified	GBIF: https://doi.org/10.15468/dl.zfaw79
Anopheles gambiae	8.36667	4.93333	Unclassified	GBIF: https://doi.org/10.15468/dl.zfaw79
Anopheles gambiae	8.56667	3.46667	Unclassified	GBIF: https://doi.org/10.15468/dl.zfaw79
Anopheles gambiae	8.58333	10.5	Unclassified	GBIF: https://doi.org/10.15468/dl.zfaw79
Anopheles gambiae	8.66667	3.63333	Unclassified	GBIF: https://doi.org/10.15468/dl.zfaw79
Anopheles gambiae	8.75	12.33333	Unclassified	GBIF: https://doi.org/10.15468/dl.zfaw79
Anopheles gambiae	8.8	3.75	Unclassified	GBIF: https://doi.org/10.15468/dl.zfaw79
Anopheles gambiae	8.83333	9.91667	Unclassified	GBIF: https://doi.org/10.15468/dl.zfaw79
Anopheles gambiae	8.899	39.912	Unclassified	GBIF: https://doi.org/10.15468/dl.zfaw79
Anopheles gambiae	8.89901	39.912	Unclassified	GBIF: https://doi.org/10.15468/dl.zfaw79
Anopheles gambiae	8.89902	39.912	Unclassified	GBIF: https://doi.org/10.15468/dl.zfaw79
Anopheles gambiae	8.89903	39.912	Unclassified	GBIF: https://doi.org/10.15468/dl.zfaw79
Anopheles gambiae	8.993	40.156	Unclassified	GBIF: https://doi.org/10.15468/dl.zfaw79
Anopheles gambiae	8.99301	40.156	Unclassified	GBIF: https://doi.org/10.15468/dl.zfaw79
Anopheles gambiae	8.99303	40.156	Unclassified	GBIF: https://doi.org/10.15468/dl.zfaw79
Anopheles gambiae	9.58333	10.25	Unclassified	GBIF: https://doi.org/10.15468/dl.zfaw79
Anopheles gambiae	9.6	4.65	Unclassified	GBIF: https://doi.org/10.15468/dl.zfaw79
Anopheles gambiae	9.71667	4.06667	Unclassified	GBIF: https://doi.org/10.15468/dl.zfaw79
Anopheles gambiae	9.76791	4.04341	Unclassified	GBIF: https://doi.org/10.15468/dl.zfaw79
Anopheles gambiae	9.83333	5.25	Unclassified	GBIF: https://doi.org/10.15468/dl.zfaw79
Anopheles gambiae	9.83333	10.33333	Unclassified	GBIF: https://doi.org/10.15468/dl.zfaw79
Anopheles gambiae	9.91667	10.16667	Unclassified	GBIF: https://doi.org/10.15468/dl.zfaw79
Anopheles gambiae	10.03333	5.46667	Unclassified	GBIF: https://doi.org/10.15468/dl.zfaw79
Anopheles gambiae	10.0667	5.45	Unclassified	GBIF: https://doi.org/10.15468/dl.zfaw79
Anopheles gambiae	10.0833	5.6	Unclassified	GBIF: https://doi.org/10.15468/dl.zfaw79
Anopheles gambiae	10.21667	-0.68333	Unclassified	GBIF: https://doi.org/10.15468/dl.zfaw79
Anopheles gambiae	10.8	3.66667	Unclassified	GBIF: https://doi.org/10.15468/dl.zfaw79
Anopheles gambiae	11	4.2	Unclassified	GBIF: https://doi.org/10.15468/dl.zfaw79
Anopheles gambiae	11.3	4.1	Unclassified	GBIF: https://doi.org/10.15468/dl.zfaw79
Anopheles gambiae	11.46667	3.83333	Unclassified	GBIF: https://doi.org/10.15468/dl.zfaw79
Anopheles gambiae	11.5	3.95	Unclassified	GBIF: https://doi.org/10.15468/dl.zfaw79

Anopheles gambiae	11.51667	3.85	Unclassified	GBIF: https://doi.org/10.15468/dl.zfaw79
Anopheles gambiae	11.519852	3.898465	Unclassified	GBIF: https://doi.org/10.15468/dl.zfaw79
Anopheles gambiae	11.53333	3.66667	Unclassified	GBIF: https://doi.org/10.15468/dl.zfaw79
Anopheles gambiae	11.55	4.16667	Unclassified	GBIF: https://doi.org/10.15468/dl.zfaw79
Anopheles gambiae	11.61667	4.23333	Unclassified	GBIF: https://doi.org/10.15468/dl.zfaw79
Anopheles gambiae	11.9054	4.45253	Unclassified	GBIF: https://doi.org/10.15468/dl.zfaw79
Anopheles gambiae	12.96	-1.76	Unclassified	GBIF: https://doi.org/10.15468/dl.zfaw79
Anopheles gambiae	13.35	-8.78333	Unclassified	GBIF: https://doi.org/10.15468/dl.zfaw79
Anopheles gambiae	13.4341	14.9205	Unclassified	GBIF: https://doi.org/10.15468/dl.zfaw79
Anopheles gambiae	13.4347	14.9209	Unclassified	GBIF: https://doi.org/10.15468/dl.zfaw79
Anopheles gambiae	13.4348	14.9208	Unclassified	GBIF: https://doi.org/10.15468/dl.zfaw79
Anopheles gambiae	13.4349	14.9211	Unclassified	GBIF: https://doi.org/10.15468/dl.zfaw79
Anopheles gambiae	13.49	-1.63	Unclassified	GBIF: https://doi.org/10.15468/dl.zfaw79
Anopheles gambiae	13.98333	9.75	Unclassified	GBIF: https://doi.org/10.15468/dl.zfaw79
Anopheles gambiae	14.25	-17.5	Unclassified	GBIF: https://doi.org/10.15468/dl.zfaw79
Anopheles gambiae	15.11667	-4.23333	Unclassified	GBIF: https://doi.org/10.15468/dl.zfaw79
Anopheles gambiae	15.15	-4.36667	Unclassified	GBIF: https://doi.org/10.15468/dl.zfaw79
Anopheles gambiae	15.272778	-4.326944	Unclassified	GBIF: https://doi.org/10.15468/dl.zfaw79
Anopheles gambiae	15.281111	-4.413611	Unclassified	GBIF: https://doi.org/10.15468/dl.zfaw79
Anopheles gambiae	15.32	-4.3475	Unclassified	GBIF: https://doi.org/10.15468/dl.zfaw79
Anopheles gambiae	15.324167	-4.416389	Unclassified	GBIF: https://doi.org/10.15468/dl.zfaw79
Anopheles gambiae	15.33333	-4.33333	Unclassified	GBIF: https://doi.org/10.15468/dl.zfaw79
Anopheles gambiae	15.338056	-4.349722	Unclassified	GBIF: https://doi.org/10.15468/dl.zfaw79
Anopheles gambiae	15.346389	-4.413056	Unclassified	GBIF: https://doi.org/10.15468/dl.zfaw79
Anopheles gambiae	15.353889	-4.476389	Unclassified	GBIF: https://doi.org/10.15468/dl.zfaw79
Anopheles gambiae	15.36667	-4.33333	Unclassified	GBIF: https://doi.org/10.15468/dl.zfaw79
Anopheles gambiae	15.413333	-4.414167	Unclassified	GBIF: https://doi.org/10.15468/dl.zfaw79
Anopheles gambiae	17	-18.8	Unclassified	GBIF: https://doi.org/10.15468/dl.zfaw79
Anopheles gambiae	17.328288	2.709585	Unclassified	GBIF: https://doi.org/10.15468/dl.zfaw79
Anopheles gambiae	18.05	-3.58333	Unclassified	GBIF: https://doi.org/10.15468/dl.zfaw79
Anopheles gambiae	18.5405	4.33108	Unclassified	GBIF: https://doi.org/10.15468/dl.zfaw79
Anopheles gambiae	18.5468	4.43042	Unclassified	GBIF: https://doi.org/10.15468/dl.zfaw79
Anopheles gambiae	18.5667	4.3549	Unclassified	GBIF: https://doi.org/10.15468/dl.zfaw79
Anopheles gambiae	18.6083	4.37667	Unclassified	GBIF: https://doi.org/10.15468/dl.zfaw79
Anopheles gambiae	18.61667	4.38333	Unclassified	GBIF: https://doi.org/10.15468/dl.zfaw79
Anopheles gambiae	20.86667	-7.36667	Unclassified	GBIF: https://doi.org/10.15468/dl.zfaw79
Anopheles gambiae	20.9667	-28.7	Unclassified	GBIF: https://doi.org/10.15468/dl.zfaw79
Anopheles gambiae	21.2	-7.48333	Unclassified	GBIF: https://doi.org/10.15468/dl.zfaw79
Anopheles gambiae	21.75	-18.08333	Unclassified	GBIF: https://doi.org/10.15468/dl.zfaw79
Anopheles gambiae	22.722124	-18.875305	Unclassified	GBIF: https://doi.org/10.15468/dl.zfaw79
Anopheles gambiae	23.8	-30.98333	Unclassified	GBIF: https://doi.org/10.15468/dl.zfaw79
Anopheles gambiae	26.6	-18.05	Unclassified	GBIF: https://doi.org/10.15468/dl.zfaw79

Anopheles gambiae	28.43333	-10.55	Unclassified	GBIF: https://doi.org/10.15468/dl.zfaw79
Anopheles gambiae	28.8333	-16.05	Unclassified	GBIF: https://doi.org/10.15468/dl.zfaw79
Anopheles gambiae	29	-2.83333	Unclassified	GBIF: https://doi.org/10.15468/dl.zfaw79
Anopheles gambiae	29.2933	-3.1875	Unclassified	GBIF: https://doi.org/10.15468/dl.zfaw79
Anopheles gambiae	29.36667	-17.91667	Unclassified	GBIF: https://doi.org/10.15468/dl.zfaw79
Anopheles gambiae	29.7	0.750025	Unclassified	GBIF: https://doi.org/10.15468/dl.zfaw79
Anopheles gambiae	29.7	0.75004	Unclassified	GBIF: https://doi.org/10.15468/dl.zfaw79
Anopheles gambiae	29.7	0.750043	Unclassified	GBIF: https://doi.org/10.15468/dl.zfaw79
Anopheles gambiae	29.7	0.750034	Unclassified	GBIF: https://doi.org/10.15468/dl.zfaw79
Anopheles gambiae	29.7	0.750039	Unclassified	GBIF: https://doi.org/10.15468/dl.zfaw79
Anopheles gambiae	29.7	0.750012	Unclassified	GBIF: https://doi.org/10.15468/dl.zfaw79
Anopheles gambiae	29.7	0.750031	Unclassified	GBIF: https://doi.org/10.15468/dl.zfaw79
Anopheles gambiae	29.7	0.750038	Unclassified	GBIF: https://doi.org/10.15468/dl.zfaw79
Anopheles gambiae	29.7	0.750018	Unclassified	GBIF: https://doi.org/10.15468/dl.zfaw79
Anopheles gambiae	29.7	0.750037	Unclassified	GBIF: https://doi.org/10.15468/dl.zfaw79
Anopheles gambiae	29.7	0.750028	Unclassified	GBIF: https://doi.org/10.15468/dl.zfaw79
Anopheles gambiae	29.7	0.750022	Unclassified	GBIF: https://doi.org/10.15468/dl.zfaw79
Anopheles gambiae	29.7	0.750023	Unclassified	GBIF: https://doi.org/10.15468/dl.zfaw79
Anopheles gambiae	29.7	0.750005	Unclassified	GBIF: https://doi.org/10.15468/dl.zfaw79
Anopheles gambiae	29.7	0.750027	Unclassified	GBIF: https://doi.org/10.15468/dl.zfaw79
Anopheles gambiae	29.7	0.750001	Unclassified	GBIF: https://doi.org/10.15468/dl.zfaw79
Anopheles gambiae	29.7	0.750019	Unclassified	GBIF: https://doi.org/10.15468/dl.zfaw79
Anopheles gambiae	29.7	0.750026	Unclassified	GBIF: https://doi.org/10.15468/dl.zfaw79
Anopheles gambiae	29.7	0.750045	Unclassified	GBIF: https://doi.org/10.15468/dl.zfaw79
Anopheles gambiae	29.7	0.75003	Unclassified	GBIF: https://doi.org/10.15468/dl.zfaw79
Anopheles gambiae	29.7	0.750015	Unclassified	GBIF: https://doi.org/10.15468/dl.zfaw79
Anopheles gambiae	29.7	0.750024	Unclassified	GBIF: https://doi.org/10.15468/dl.zfaw79
Anopheles gambiae	29.7	0.750048	Unclassified	GBIF: https://doi.org/10.15468/dl.zfaw79
Anopheles gambiae	29.7	0.750006	Unclassified	GBIF: https://doi.org/10.15468/dl.zfaw79
Anopheles gambiae	29.7	0.750049	Unclassified	GBIF: https://doi.org/10.15468/dl.zfaw79
Anopheles gambiae	29.7	0.750014	Unclassified	GBIF: https://doi.org/10.15468/dl.zfaw79
Anopheles gambiae	29.7	0.750003	Unclassified	GBIF: https://doi.org/10.15468/dl.zfaw79
Anopheles gambiae	29.7	0.750044	Unclassified	GBIF: https://doi.org/10.15468/dl.zfaw79
Anopheles gambiae	29.7	0.750041	Unclassified	GBIF: https://doi.org/10.15468/dl.zfaw79
Anopheles gambiae	29.7	0.750036	Unclassified	GBIF: https://doi.org/10.15468/dl.zfaw79
Anopheles gambiae	29.7	0.750017	Unclassified	GBIF: https://doi.org/10.15468/dl.zfaw79
Anopheles gambiae	29.7	0.750013	Unclassified	GBIF: https://doi.org/10.15468/dl.zfaw79
Anopheles gambiae	29.7	0.750032	Unclassified	GBIF: https://doi.org/10.15468/dl.zfaw79
Anopheles gambiae	29.7	0.75002	Unclassified	GBIF: https://doi.org/10.15468/dl.zfaw79
Anopheles gambiae	29.7	0.750002	Unclassified	GBIF: https://doi.org/10.15468/dl.zfaw79
Anopheles gambiae	29.7	0.750016	Unclassified	GBIF: https://doi.org/10.15468/dl.zfaw79
Anopheles gambiae	29.7	0.750011	Unclassified	GBIF: https://doi.org/10.15468/dl.zfaw79

Anopheles gambiae	29.7	0.750029	Unclassified	GBIF: https://doi.org/10.15468/dl.zfaw79
Anopheles gambiae	29.7	0.75001	Unclassified	GBIF: https://doi.org/10.15468/dl.zfaw79
Anopheles gambiae	29.7	0.750047	Unclassified	GBIF: https://doi.org/10.15468/dl.zfaw79
Anopheles gambiae	29.7	0.750035	Unclassified	GBIF: https://doi.org/10.15468/dl.zfaw79
Anopheles gambiae	29.7	0.750008	Unclassified	GBIF: https://doi.org/10.15468/dl.zfaw79
Anopheles gambiae	29.7	0.750042	Unclassified	GBIF: https://doi.org/10.15468/dl.zfaw79
Anopheles gambiae	29.7	0.750007	Unclassified	GBIF: https://doi.org/10.15468/dl.zfaw79
Anopheles gambiae	29.7	0.750004	Unclassified	GBIF: https://doi.org/10.15468/dl.zfaw79
Anopheles gambiae	29.7	0.750021	Unclassified	GBIF: https://doi.org/10.15468/dl.zfaw79
Anopheles gambiae	29.7	0.750033	Unclassified	GBIF: https://doi.org/10.15468/dl.zfaw79
Anopheles gambiae	29.7	0.750009	Unclassified	GBIF: https://doi.org/10.15468/dl.zfaw79
Anopheles gambiae	29.7	0.750046	Unclassified	GBIF: https://doi.org/10.15468/dl.zfaw79
Anopheles gambiae	29.7001	0.750108	Unclassified	GBIF: https://doi.org/10.15468/dl.zfaw79
Anopheles gambiae	29.7001	0.750065	Unclassified	GBIF: https://doi.org/10.15468/dl.zfaw79
Anopheles gambiae	29.7001	0.750064	Unclassified	GBIF: https://doi.org/10.15468/dl.zfaw79
Anopheles gambiae	29.7001	0.750112	Unclassified	GBIF: https://doi.org/10.15468/dl.zfaw79
Anopheles gambiae	29.7001	0.750059	Unclassified	GBIF: https://doi.org/10.15468/dl.zfaw79
Anopheles gambiae	29.7001	0.750113	Unclassified	GBIF: https://doi.org/10.15468/dl.zfaw79
Anopheles gambiae	29.7001	0.750053	Unclassified	GBIF: https://doi.org/10.15468/dl.zfaw79
Anopheles gambiae	29.7001	0.750083	Unclassified	GBIF: https://doi.org/10.15468/dl.zfaw79
Anopheles gambiae	29.7001	0.750107	Unclassified	GBIF: https://doi.org/10.15468/dl.zfaw79
Anopheles gambiae	29.7001	0.750096	Unclassified	GBIF: https://doi.org/10.15468/dl.zfaw79
Anopheles gambiae	29.7001	0.750114	Unclassified	GBIF: https://doi.org/10.15468/dl.zfaw79
Anopheles gambiae	29.7001	0.750117	Unclassified	GBIF: https://doi.org/10.15468/dl.zfaw79
Anopheles gambiae	29.7001	0.750056	Unclassified	GBIF: https://doi.org/10.15468/dl.zfaw79
Anopheles gambiae	29.7001	0.75006	Unclassified	GBIF: https://doi.org/10.15468/dl.zfaw79
Anopheles gambiae	29.7001	0.750078	Unclassified	GBIF: https://doi.org/10.15468/dl.zfaw79
Anopheles gambiae	29.7001	0.750052	Unclassified	GBIF: https://doi.org/10.15468/dl.zfaw79
Anopheles gambiae	29.7001	0.750055	Unclassified	GBIF: https://doi.org/10.15468/dl.zfaw79
Anopheles gambiae	29.7001	0.750084	Unclassified	GBIF: https://doi.org/10.15468/dl.zfaw79
Anopheles gambiae	29.7001	0.750097	Unclassified	GBIF: https://doi.org/10.15468/dl.zfaw79
Anopheles gambiae	29.7001	0.750058	Unclassified	GBIF: https://doi.org/10.15468/dl.zfaw79
Anopheles gambiae	29.7001	0.750054	Unclassified	GBIF: https://doi.org/10.15468/dl.zfaw79
Anopheles gambiae	29.7001	0.750081	Unclassified	GBIF: https://doi.org/10.15468/dl.zfaw79
Anopheles gambiae	29.7001	0.750092	Unclassified	GBIF: https://doi.org/10.15468/dl.zfaw79
Anopheles gambiae	29.7001	0.75005	Unclassified	GBIF: https://doi.org/10.15468/dl.zfaw79
Anopheles gambiae	29.7001	0.750103	Unclassified	GBIF: https://doi.org/10.15468/dl.zfaw79
Anopheles gambiae	29.7001	0.750073	Unclassified	GBIF: https://doi.org/10.15468/dl.zfaw79
Anopheles gambiae	29.7001	0.750085	Unclassified	GBIF: https://doi.org/10.15468/dl.zfaw79
Anopheles gambiae	29.7001	0.750098	Unclassified	GBIF: https://doi.org/10.15468/dl.zfaw79
Anopheles gambiae	29.7001	0.750075	Unclassified	GBIF: https://doi.org/10.15468/dl.zfaw79
Anopheles gambiae	29.7001	0.750104	Unclassified	GBIF: https://doi.org/10.15468/dl.zfaw79

Anopheles gambiae	29.7001	0.750086	Unclassified	GBIF: https://doi.org/10.15468/dl.zfaw79
Anopheles gambiae	29.7001	0.750051	Unclassified	GBIF: https://doi.org/10.15468/dl.zfaw79
Anopheles gambiae	29.7001	0.750099	Unclassified	GBIF: https://doi.org/10.15468/dl.zfaw79
Anopheles gambiae	29.7001	0.750057	Unclassified	GBIF: https://doi.org/10.15468/dl.zfaw79
Anopheles gambiae	29.7001	0.750067	Unclassified	GBIF: https://doi.org/10.15468/dl.zfaw79
Anopheles gambiae	29.7001	0.750072	Unclassified	GBIF: https://doi.org/10.15468/dl.zfaw79
Anopheles gambiae	29.7001	0.750101	Unclassified	GBIF: https://doi.org/10.15468/dl.zfaw79
Anopheles gambiae	29.7001	0.750082	Unclassified	GBIF: https://doi.org/10.15468/dl.zfaw79
Anopheles gambiae	29.7001	0.750077	Unclassified	GBIF: https://doi.org/10.15468/dl.zfaw79
Anopheles gambiae	29.7001	0.750068	Unclassified	GBIF: https://doi.org/10.15468/dl.zfaw79
Anopheles gambiae	29.7001	0.750076	Unclassified	GBIF: https://doi.org/10.15468/dl.zfaw79
Anopheles gambiae	29.7001	0.750063	Unclassified	GBIF: https://doi.org/10.15468/dl.zfaw79
Anopheles gambiae	29.7001	0.750116	Unclassified	GBIF: https://doi.org/10.15468/dl.zfaw79
Anopheles gambiae	29.7001	0.75007	Unclassified	GBIF: https://doi.org/10.15468/dl.zfaw79
Anopheles gambiae	29.7001	0.750066	Unclassified	GBIF: https://doi.org/10.15468/dl.zfaw79
Anopheles gambiae	29.7001	0.750102	Unclassified	GBIF: https://doi.org/10.15468/dl.zfaw79
Anopheles gambiae	29.7001	0.750094	Unclassified	GBIF: https://doi.org/10.15468/dl.zfaw79
Anopheles gambiae	29.7001	0.750062	Unclassified	GBIF: https://doi.org/10.15468/dl.zfaw79
Anopheles gambiae	29.7001	0.750091	Unclassified	GBIF: https://doi.org/10.15468/dl.zfaw79
Anopheles gambiae	29.7001	0.750079	Unclassified	GBIF: https://doi.org/10.15468/dl.zfaw79
Anopheles gambiae	29.7001	0.750093	Unclassified	GBIF: https://doi.org/10.15468/dl.zfaw79
Anopheles gambiae	29.7001	0.75008	Unclassified	GBIF: https://doi.org/10.15468/dl.zfaw79
Anopheles gambiae	29.7001	0.750071	Unclassified	GBIF: https://doi.org/10.15468/dl.zfaw79
Anopheles gambiae	29.7001	0.7501	Unclassified	GBIF: https://doi.org/10.15468/dl.zfaw79
Anopheles gambiae	29.7001	0.750106	Unclassified	GBIF: https://doi.org/10.15468/dl.zfaw79
Anopheles gambiae	29.7001	0.750087	Unclassified	GBIF: https://doi.org/10.15468/dl.zfaw79
Anopheles gambiae	29.7001	0.750061	Unclassified	GBIF: https://doi.org/10.15468/dl.zfaw79
Anopheles gambiae	29.7001	0.750105	Unclassified	GBIF: https://doi.org/10.15468/dl.zfaw79
Anopheles gambiae	29.7001	0.750115	Unclassified	GBIF: https://doi.org/10.15468/dl.zfaw79
Anopheles gambiae	29.7001	0.750074	Unclassified	GBIF: https://doi.org/10.15468/dl.zfaw79
Anopheles gambiae	29.7001	0.750089	Unclassified	GBIF: https://doi.org/10.15468/dl.zfaw79
Anopheles gambiae	29.7001	0.75009	Unclassified	GBIF: https://doi.org/10.15468/dl.zfaw79
Anopheles gambiae	29.7003	-0.748889	Unclassified	GBIF: https://doi.org/10.15468/dl.zfaw79
Anopheles gambiae	29.7814	-0.885566	Unclassified	GBIF: https://doi.org/10.15468/dl.zfaw79
Anopheles gambiae	29.7827	-0.885652	Unclassified	GBIF: https://doi.org/10.15468/dl.zfaw79
Anopheles gambiae	29.88333	-0.13333	Unclassified	GBIF: https://doi.org/10.15468/dl.zfaw79
Anopheles gambiae	30	-3.33333	Unclassified	GBIF: https://doi.org/10.15468/dl.zfaw79
Anopheles gambiae	30.015	-0.85	Unclassified	GBIF: https://doi.org/10.15468/dl.zfaw79
Anopheles gambiae	30.026	-2.015	Unclassified	GBIF: https://doi.org/10.15468/dl.zfaw79
Anopheles gambiae	30.064	-1.887	Unclassified	GBIF: https://doi.org/10.15468/dl.zfaw79
Anopheles gambiae	30.115	-1.967	Unclassified	GBIF: https://doi.org/10.15468/dl.zfaw79
Anopheles gambiae	30.13333	0.66667	Unclassified	GBIF: https://doi.org/10.15468/dl.zfaw79

Anopheles gambiae	30.18333	0.8	Unclassified	GBIF: https://doi.org/10.15468/dl.zfaw79
Anopheles gambiae	30.23333	0.9	Unclassified	GBIF: https://doi.org/10.15468/dl.zfaw79
Anopheles gambiae	30.33333	-15.66667	Unclassified	GBIF: https://doi.org/10.15468/dl.zfaw79
Anopheles gambiae	30.51667	-23	Unclassified	GBIF: https://doi.org/10.15468/dl.zfaw79
Anopheles gambiae	30.58333	-22.75	Unclassified	GBIF: https://doi.org/10.15468/dl.zfaw79
Anopheles gambiae	30.58333	-23.5	Unclassified	GBIF: https://doi.org/10.15468/dl.zfaw79
Anopheles gambiae	30.977778	-29.890556	Unclassified	GBIF: https://doi.org/10.15468/dl.zfaw79
Anopheles gambiae	31	-29.883333	Unclassified	GBIF: https://doi.org/10.15468/dl.zfaw79
Anopheles gambiae	31.0396	-22.8896	Unclassified	GBIF: https://doi.org/10.15468/dl.zfaw79
Anopheles gambiae	31.11667	-23.98333	Unclassified	GBIF: https://doi.org/10.15468/dl.zfaw79
Anopheles gambiae	31.56667	-0.63333	Unclassified	GBIF: https://doi.org/10.15468/dl.zfaw79
Anopheles gambiae	31.63333	-25.91667	Unclassified	GBIF: https://doi.org/10.15468/dl.zfaw79
Anopheles gambiae	31.66667	-21.41667	Unclassified	GBIF: https://doi.org/10.15468/dl.zfaw79
Anopheles gambiae	31.7827	-24.6314	Unclassified	GBIF: https://doi.org/10.15468/dl.zfaw79
Anopheles gambiae	31.8	-26.41667	Unclassified	GBIF: https://doi.org/10.15468/dl.zfaw79
Anopheles gambiae	31.88	0.31	Unclassified	GBIF: https://doi.org/10.15468/dl.zfaw79
Anopheles gambiae	31.91667	-21.28333	Unclassified	GBIF: https://doi.org/10.15468/dl.zfaw79
Anopheles gambiae	31.91667	-17.33333	Unclassified	GBIF: https://doi.org/10.15468/dl.zfaw79
Anopheles gambiae	31.93333	-26.83333	Unclassified	GBIF: https://doi.org/10.15468/dl.zfaw79
Anopheles gambiae	32.023894	-20.639587	Unclassified	GBIF: https://doi.org/10.15468/dl.zfaw79
Anopheles gambiae	32.05	0.35	Unclassified	GBIF: https://doi.org/10.15468/dl.zfaw79
Anopheles gambiae	32.088611	-27.561667	Unclassified	GBIF: https://doi.org/10.15468/dl.zfaw79
Anopheles gambiae	32.1	0.06	Unclassified	GBIF: https://doi.org/10.15468/dl.zfaw79
Anopheles gambiae	32.18333	-20.91667	Unclassified	GBIF: https://doi.org/10.15468/dl.zfaw79
Anopheles gambiae	32.24	-0.44	Unclassified	GBIF: https://doi.org/10.15468/dl.zfaw79
Anopheles gambiae	32.25	-27.7	Unclassified	GBIF: https://doi.org/10.15468/dl.zfaw79
Anopheles gambiae	32.251389	-27.7125	Unclassified	GBIF: https://doi.org/10.15468/dl.zfaw79
Anopheles gambiae	32.278612	-26.930937	Unclassified	GBIF: https://doi.org/10.15468/dl.zfaw79
Anopheles gambiae	32.35	-0.24	Unclassified	GBIF: https://doi.org/10.15468/dl.zfaw79
Anopheles gambiae	32.4	-0.26	Unclassified	GBIF: https://doi.org/10.15468/dl.zfaw79
Anopheles gambiae	32.45	0.06	Unclassified	GBIF: https://doi.org/10.15468/dl.zfaw79
Anopheles gambiae	32.5	-0.44	Unclassified	GBIF: https://doi.org/10.15468/dl.zfaw79
Anopheles gambiae	32.5	-27	Unclassified	GBIF: https://doi.org/10.15468/dl.zfaw79
Anopheles gambiae	32.55	-27.05	Unclassified	GBIF: https://doi.org/10.15468/dl.zfaw79
Anopheles gambiae	32.573292	-25.969141	Unclassified	GBIF: https://doi.org/10.15468/dl.zfaw79
Anopheles gambiae	32.6	-0.09	Unclassified	GBIF: https://doi.org/10.15468/dl.zfaw79
Anopheles gambiae	32.66667	-25.76667	Unclassified	GBIF: https://doi.org/10.15468/dl.zfaw79
Anopheles gambiae	32.83333	2.7	Unclassified	GBIF: https://doi.org/10.15468/dl.zfaw79
Anopheles gambiae	32.86667	-25.25	Unclassified	GBIF: https://doi.org/10.15468/dl.zfaw79
Anopheles gambiae	33	-25.05	Unclassified	GBIF: https://doi.org/10.15468/dl.zfaw79
Anopheles gambiae	33	-2.83333	Unclassified	GBIF: https://doi.org/10.15468/dl.zfaw79
Anopheles gambiae	33.08333	0.83333	Unclassified	GBIF: https://doi.org/10.15468/dl.zfaw79

Anopheles gambiae	33.15	0.28	Unclassified	GBIF: https://doi.org/10.15468/dl.zfaw79
Anopheles gambiae	33.214	0.448213	Unclassified	GBIF: https://doi.org/10.15468/dl.zfaw79
Anopheles gambiae	33.226	0.443037	Unclassified	GBIF: https://doi.org/10.15468/dl.zfaw79
Anopheles gambiae	33.226	0.443036	Unclassified	GBIF: https://doi.org/10.15468/dl.zfaw79
Anopheles gambiae	33.226	0.443034	Unclassified	GBIF: https://doi.org/10.15468/dl.zfaw79
Anopheles gambiae	33.226	0.443035	Unclassified	GBIF: https://doi.org/10.15468/dl.zfaw79
Anopheles gambiae	33.226	0.443032	Unclassified	GBIF: https://doi.org/10.15468/dl.zfaw79
Anopheles gambiae	33.226	0.443009	Unclassified	GBIF: https://doi.org/10.15468/dl.zfaw79
Anopheles gambiae	33.226	0.443011	Unclassified	GBIF: https://doi.org/10.15468/dl.zfaw79
Anopheles gambiae	33.226	0.443029	Unclassified	GBIF: https://doi.org/10.15468/dl.zfaw79
Anopheles gambiae	33.226	0.443002	Unclassified	GBIF: https://doi.org/10.15468/dl.zfaw79
Anopheles gambiae	33.226	0.443017	Unclassified	GBIF: https://doi.org/10.15468/dl.zfaw79
Anopheles gambiae	33.226	0.443041	Unclassified	GBIF: https://doi.org/10.15468/dl.zfaw79
Anopheles gambiae	33.226	0.443049	Unclassified	GBIF: https://doi.org/10.15468/dl.zfaw79
Anopheles gambiae	33.226	0.443047	Unclassified	GBIF: https://doi.org/10.15468/dl.zfaw79
Anopheles gambiae	33.226	0.443008	Unclassified	GBIF: https://doi.org/10.15468/dl.zfaw79
Anopheles gambiae	33.226	0.443033	Unclassified	GBIF: https://doi.org/10.15468/dl.zfaw79
Anopheles gambiae	33.226	0.443042	Unclassified	GBIF: https://doi.org/10.15468/dl.zfaw79
Anopheles gambiae	33.226	0.443004	Unclassified	GBIF: https://doi.org/10.15468/dl.zfaw79
Anopheles gambiae	33.226	0.44303	Unclassified	GBIF: https://doi.org/10.15468/dl.zfaw79
Anopheles gambiae	33.226	0.443025	Unclassified	GBIF: https://doi.org/10.15468/dl.zfaw79
Anopheles gambiae	33.226	0.443026	Unclassified	GBIF: https://doi.org/10.15468/dl.zfaw79
Anopheles gambiae	33.226	0.443038	Unclassified	GBIF: https://doi.org/10.15468/dl.zfaw79
Anopheles gambiae	33.226	0.443018	Unclassified	GBIF: https://doi.org/10.15468/dl.zfaw79
Anopheles gambiae	33.226	0.44304	Unclassified	GBIF: https://doi.org/10.15468/dl.zfaw79
Anopheles gambiae	33.226	0.443023	Unclassified	GBIF: https://doi.org/10.15468/dl.zfaw79
Anopheles gambiae	33.226	0.44301	Unclassified	GBIF: https://doi.org/10.15468/dl.zfaw79
Anopheles gambiae	33.226	0.443028	Unclassified	GBIF: https://doi.org/10.15468/dl.zfaw79
Anopheles gambiae	33.226	0.443048	Unclassified	GBIF: https://doi.org/10.15468/dl.zfaw79
Anopheles gambiae	33.226	0.443024	Unclassified	GBIF: https://doi.org/10.15468/dl.zfaw79
Anopheles gambiae	33.226	0.443027	Unclassified	GBIF: https://doi.org/10.15468/dl.zfaw79
Anopheles gambiae	33.226	0.443019	Unclassified	GBIF: https://doi.org/10.15468/dl.zfaw79
Anopheles gambiae	33.226	0.443001	Unclassified	GBIF: https://doi.org/10.15468/dl.zfaw79
Anopheles gambiae	33.226	0.443044	Unclassified	GBIF: https://doi.org/10.15468/dl.zfaw79
Anopheles gambiae	33.226	0.443016	Unclassified	GBIF: https://doi.org/10.15468/dl.zfaw79
Anopheles gambiae	33.226	0.443012	Unclassified	GBIF: https://doi.org/10.15468/dl.zfaw79
Anopheles gambiae	33.226	0.443022	Unclassified	GBIF: https://doi.org/10.15468/dl.zfaw79
Anopheles gambiae	33.226	0.443014	Unclassified	GBIF: https://doi.org/10.15468/dl.zfaw79
Anopheles gambiae	33.226	0.443045	Unclassified	GBIF: https://doi.org/10.15468/dl.zfaw79
Anopheles gambiae	33.226	0.443007	Unclassified	GBIF: https://doi.org/10.15468/dl.zfaw79
Anopheles gambiae	33.226	0.443031	Unclassified	GBIF: https://doi.org/10.15468/dl.zfaw79
Anopheles gambiae	33.226	0.443003	Unclassified	GBIF: https://doi.org/10.15468/dl.zfaw79

Anopheles gambiae	33.226	0.443006	Unclassified	GBIF: https://doi.org/10.15468/dl.zfaw79
Anopheles gambiae	33.226	0.443043	Unclassified	GBIF: https://doi.org/10.15468/dl.zfaw79
Anopheles gambiae	33.226	0.44302	Unclassified	GBIF: https://doi.org/10.15468/dl.zfaw79
Anopheles gambiae	33.226	0.443046	Unclassified	GBIF: https://doi.org/10.15468/dl.zfaw79
Anopheles gambiae	33.226	0.443039	Unclassified	GBIF: https://doi.org/10.15468/dl.zfaw79
Anopheles gambiae	33.226	0.443015	Unclassified	GBIF: https://doi.org/10.15468/dl.zfaw79
Anopheles gambiae	33.2261	0.443066	Unclassified	GBIF: https://doi.org/10.15468/dl.zfaw79
Anopheles gambiae	33.2261	0.44308	Unclassified	GBIF: https://doi.org/10.15468/dl.zfaw79
Anopheles gambiae	33.2261	0.443058	Unclassified	GBIF: https://doi.org/10.15468/dl.zfaw79
Anopheles gambiae	33.2261	0.443099	Unclassified	GBIF: https://doi.org/10.15468/dl.zfaw79
Anopheles gambiae	33.2261	0.4431	Unclassified	GBIF: https://doi.org/10.15468/dl.zfaw79
Anopheles gambiae	33.2261	0.443094	Unclassified	GBIF: https://doi.org/10.15468/dl.zfaw79
Anopheles gambiae	33.2261	0.443096	Unclassified	GBIF: https://doi.org/10.15468/dl.zfaw79
Anopheles gambiae	33.2261	0.443095	Unclassified	GBIF: https://doi.org/10.15468/dl.zfaw79
Anopheles gambiae	33.2261	0.443061	Unclassified	GBIF: https://doi.org/10.15468/dl.zfaw79
Anopheles gambiae	33.2261	0.443112	Unclassified	GBIF: https://doi.org/10.15468/dl.zfaw79
Anopheles gambiae	33.2261	0.443104	Unclassified	GBIF: https://doi.org/10.15468/dl.zfaw79
Anopheles gambiae	33.2261	0.443053	Unclassified	GBIF: https://doi.org/10.15468/dl.zfaw79
Anopheles gambiae	33.2261	0.443064	Unclassified	GBIF: https://doi.org/10.15468/dl.zfaw79
Anopheles gambiae	33.2261	0.443107	Unclassified	GBIF: https://doi.org/10.15468/dl.zfaw79
Anopheles gambiae	33.2261	0.443072	Unclassified	GBIF: https://doi.org/10.15468/dl.zfaw79
Anopheles gambiae	33.2261	0.443071	Unclassified	GBIF: https://doi.org/10.15468/dl.zfaw79
Anopheles gambiae	33.2261	0.443113	Unclassified	GBIF: https://doi.org/10.15468/dl.zfaw79
Anopheles gambiae	33.2261	0.443062	Unclassified	GBIF: https://doi.org/10.15468/dl.zfaw79
Anopheles gambiae	33.2261	0.443079	Unclassified	GBIF: https://doi.org/10.15468/dl.zfaw79
Anopheles gambiae	33.2261	0.443115	Unclassified	GBIF: https://doi.org/10.15468/dl.zfaw79
Anopheles gambiae	33.2261	0.443105	Unclassified	GBIF: https://doi.org/10.15468/dl.zfaw79
Anopheles gambiae	33.2261	0.44306	Unclassified	GBIF: https://doi.org/10.15468/dl.zfaw79
Anopheles gambiae	33.2261	0.443108	Unclassified	GBIF: https://doi.org/10.15468/dl.zfaw79
Anopheles gambiae	33.2261	0.443073	Unclassified	GBIF: https://doi.org/10.15468/dl.zfaw79
Anopheles gambiae	33.2261	0.443055	Unclassified	GBIF: https://doi.org/10.15468/dl.zfaw79
Anopheles gambiae	33.2261	0.443098	Unclassified	GBIF: https://doi.org/10.15468/dl.zfaw79
Anopheles gambiae	33.2261	0.44311	Unclassified	GBIF: https://doi.org/10.15468/dl.zfaw79
Anopheles gambiae	33.2261	0.443106	Unclassified	GBIF: https://doi.org/10.15468/dl.zfaw79
Anopheles gambiae	33.2261	0.443092	Unclassified	GBIF: https://doi.org/10.15468/dl.zfaw79
Anopheles gambiae	33.2261	0.443078	Unclassified	GBIF: https://doi.org/10.15468/dl.zfaw79
Anopheles gambiae	33.2261	0.443093	Unclassified	GBIF: https://doi.org/10.15468/dl.zfaw79
Anopheles gambiae	33.2261	0.443087	Unclassified	GBIF: https://doi.org/10.15468/dl.zfaw79
Anopheles gambiae	33.2261	0.44305	Unclassified	GBIF: https://doi.org/10.15468/dl.zfaw79
Anopheles gambiae	33.2261	0.443052	Unclassified	GBIF: https://doi.org/10.15468/dl.zfaw79
Anopheles gambiae	33.2261	0.443057	Unclassified	GBIF: https://doi.org/10.15468/dl.zfaw79
Anopheles gambiae	33.2261	0.443059	Unclassified	GBIF: https://doi.org/10.15468/dl.zfaw79

Anopheles gambiae	33.2261	0.443067	Unclassified	GBIF: https://doi.org/10.15468/dl.zfaw79
Anopheles gambiae	33.2261	0.44309	Unclassified	GBIF: https://doi.org/10.15468/dl.zfaw79
Anopheles gambiae	33.2261	0.443068	Unclassified	GBIF: https://doi.org/10.15468/dl.zfaw79
Anopheles gambiae	33.2261	0.443081	Unclassified	GBIF: https://doi.org/10.15468/dl.zfaw79
Anopheles gambiae	33.2261	0.443069	Unclassified	GBIF: https://doi.org/10.15468/dl.zfaw79
Anopheles gambiae	33.2261	0.443103	Unclassified	GBIF: https://doi.org/10.15468/dl.zfaw79
Anopheles gambiae	33.2261	0.443102	Unclassified	GBIF: https://doi.org/10.15468/dl.zfaw79
Anopheles gambiae	33.2261	0.443114	Unclassified	GBIF: https://doi.org/10.15468/dl.zfaw79
Anopheles gambiae	33.2261	0.443083	Unclassified	GBIF: https://doi.org/10.15468/dl.zfaw79
Anopheles gambiae	33.2261	0.443111	Unclassified	GBIF: https://doi.org/10.15468/dl.zfaw79
Anopheles gambiae	33.2261	0.443101	Unclassified	GBIF: https://doi.org/10.15468/dl.zfaw79
Anopheles gambiae	33.2261	0.443082	Unclassified	GBIF: https://doi.org/10.15468/dl.zfaw79
Anopheles gambiae	33.2261	0.443109	Unclassified	GBIF: https://doi.org/10.15468/dl.zfaw79
Anopheles gambiae	33.2261	0.443118	Unclassified	GBIF: https://doi.org/10.15468/dl.zfaw79
Anopheles gambiae	33.2261	0.443084	Unclassified	GBIF: https://doi.org/10.15468/dl.zfaw79
Anopheles gambiae	33.2261	0.443051	Unclassified	GBIF: https://doi.org/10.15468/dl.zfaw79
Anopheles gambiae	33.2261	0.443077	Unclassified	GBIF: https://doi.org/10.15468/dl.zfaw79
Anopheles gambiae	33.2261	0.443116	Unclassified	GBIF: https://doi.org/10.15468/dl.zfaw79
Anopheles gambiae	33.2261	0.443065	Unclassified	GBIF: https://doi.org/10.15468/dl.zfaw79
Anopheles gambiae	33.2261	0.443089	Unclassified	GBIF: https://doi.org/10.15468/dl.zfaw79
Anopheles gambiae	33.2261	0.443074	Unclassified	GBIF: https://doi.org/10.15468/dl.zfaw79
Anopheles gambiae	33.2261	0.443063	Unclassified	GBIF: https://doi.org/10.15468/dl.zfaw79
Anopheles gambiae	33.2261	0.443097	Unclassified	GBIF: https://doi.org/10.15468/dl.zfaw79
Anopheles gambiae	33.2261	0.443085	Unclassified	GBIF: https://doi.org/10.15468/dl.zfaw79
Anopheles gambiae	33.2367	0.447222	Unclassified	GBIF: https://doi.org/10.15468/dl.zfaw79
Anopheles gambiae	33.41667	1.1	Unclassified	GBIF: https://doi.org/10.15468/dl.zfaw79
Anopheles gambiae	33.51667	-16.16667	Unclassified	GBIF: https://doi.org/10.15468/dl.zfaw79
Anopheles gambiae	33.7276	1.66042	Unclassified	GBIF: https://doi.org/10.15468/dl.zfaw79
Anopheles gambiae	33.7366	1.71742	Unclassified	GBIF: https://doi.org/10.15468/dl.zfaw79
Anopheles gambiae	33.86667	-9.58333	Unclassified	GBIF: https://doi.org/10.15468/dl.zfaw79
Anopheles gambiae	33.99	-0.45	Unclassified	GBIF: https://doi.org/10.15468/dl.zfaw79
Anopheles gambiae	34	0	Unclassified	GBIF: https://doi.org/10.15468/dl.zfaw79
Anopheles gambiae	34	-0.06667	Unclassified	GBIF: https://doi.org/10.15468/dl.zfaw79
Anopheles gambiae	34.01	-0.03	Unclassified	GBIF: https://doi.org/10.15468/dl.zfaw79
Anopheles gambiae	34.026	0.770004	Unclassified	GBIF: https://doi.org/10.15468/dl.zfaw79
Anopheles gambiae	34.026	0.770032	Unclassified	GBIF: https://doi.org/10.15468/dl.zfaw79
Anopheles gambiae	34.026	0.770041	Unclassified	GBIF: https://doi.org/10.15468/dl.zfaw79
Anopheles gambiae	34.026	0.770003	Unclassified	GBIF: https://doi.org/10.15468/dl.zfaw79
Anopheles gambiae	34.026	0.770031	Unclassified	GBIF: https://doi.org/10.15468/dl.zfaw79
Anopheles gambiae	34.026	0.770009	Unclassified	GBIF: https://doi.org/10.15468/dl.zfaw79
Anopheles gambiae	34.026	0.770012	Unclassified	GBIF: https://doi.org/10.15468/dl.zfaw79
Anopheles gambiae	34.026	0.77001	Unclassified	GBIF: https://doi.org/10.15468/dl.zfaw79

Anopheles gambiae	34.026	0.770033	Unclassified	GBIF: https://doi.org/10.15468/dl.zfaw79
Anopheles gambiae	34.026	0.770011	Unclassified	GBIF: https://doi.org/10.15468/dl.zfaw79
Anopheles gambiae	34.026	0.770013	Unclassified	GBIF: https://doi.org/10.15468/dl.zfaw79
Anopheles gambiae	34.026	0.770018	Unclassified	GBIF: https://doi.org/10.15468/dl.zfaw79
Anopheles gambiae	34.026	0.770029	Unclassified	GBIF: https://doi.org/10.15468/dl.zfaw79
Anopheles gambiae	34.026	0.770024	Unclassified	GBIF: https://doi.org/10.15468/dl.zfaw79
Anopheles gambiae	34.026	0.770005	Unclassified	GBIF: https://doi.org/10.15468/dl.zfaw79
Anopheles gambiae	34.026	0.770045	Unclassified	GBIF: https://doi.org/10.15468/dl.zfaw79
Anopheles gambiae	34.026	0.770042	Unclassified	GBIF: https://doi.org/10.15468/dl.zfaw79
Anopheles gambiae	34.026	0.770022	Unclassified	GBIF: https://doi.org/10.15468/dl.zfaw79
Anopheles gambiae	34.026	0.770035	Unclassified	GBIF: https://doi.org/10.15468/dl.zfaw79
Anopheles gambiae	34.026	0.770015	Unclassified	GBIF: https://doi.org/10.15468/dl.zfaw79
Anopheles gambiae	34.026	0.770027	Unclassified	GBIF: https://doi.org/10.15468/dl.zfaw79
Anopheles gambiae	34.026	0.770016	Unclassified	GBIF: https://doi.org/10.15468/dl.zfaw79
Anopheles gambiae	34.026	0.770025	Unclassified	GBIF: https://doi.org/10.15468/dl.zfaw79
Anopheles gambiae	34.026	0.770007	Unclassified	GBIF: https://doi.org/10.15468/dl.zfaw79
Anopheles gambiae	34.026	0.770019	Unclassified	GBIF: https://doi.org/10.15468/dl.zfaw79
Anopheles gambiae	34.026	0.77004	Unclassified	GBIF: https://doi.org/10.15468/dl.zfaw79
Anopheles gambiae	34.026	0.770039	Unclassified	GBIF: https://doi.org/10.15468/dl.zfaw79
Anopheles gambiae	34.026	0.77002	Unclassified	GBIF: https://doi.org/10.15468/dl.zfaw79
Anopheles gambiae	34.026	0.770038	Unclassified	GBIF: https://doi.org/10.15468/dl.zfaw79
Anopheles gambiae	34.026	0.770006	Unclassified	GBIF: https://doi.org/10.15468/dl.zfaw79
Anopheles gambiae	34.026	0.770028	Unclassified	GBIF: https://doi.org/10.15468/dl.zfaw79
Anopheles gambiae	34.026	0.770023	Unclassified	GBIF: https://doi.org/10.15468/dl.zfaw79
Anopheles gambiae	34.026	0.770021	Unclassified	GBIF: https://doi.org/10.15468/dl.zfaw79
Anopheles gambiae	34.026	0.770043	Unclassified	GBIF: https://doi.org/10.15468/dl.zfaw79
Anopheles gambiae	34.026	0.770046	Unclassified	GBIF: https://doi.org/10.15468/dl.zfaw79
Anopheles gambiae	34.026	0.770044	Unclassified	GBIF: https://doi.org/10.15468/dl.zfaw79
Anopheles gambiae	34.026	0.770037	Unclassified	GBIF: https://doi.org/10.15468/dl.zfaw79
Anopheles gambiae	34.026	0.770048	Unclassified	GBIF: https://doi.org/10.15468/dl.zfaw79
Anopheles gambiae	34.026	0.770002	Unclassified	GBIF: https://doi.org/10.15468/dl.zfaw79
Anopheles gambiae	34.026	0.770034	Unclassified	GBIF: https://doi.org/10.15468/dl.zfaw79
Anopheles gambiae	34.026	0.770014	Unclassified	GBIF: https://doi.org/10.15468/dl.zfaw79
Anopheles gambiae	34.026	0.770047	Unclassified	GBIF: https://doi.org/10.15468/dl.zfaw79
Anopheles gambiae	34.026	0.770008	Unclassified	GBIF: https://doi.org/10.15468/dl.zfaw79
Anopheles gambiae	34.026	0.770001	Unclassified	GBIF: https://doi.org/10.15468/dl.zfaw79
Anopheles gambiae	34.026	0.770036	Unclassified	GBIF: https://doi.org/10.15468/dl.zfaw79
Anopheles gambiae	34.026	0.770026	Unclassified	GBIF: https://doi.org/10.15468/dl.zfaw79
Anopheles gambiae	34.026	0.77003	Unclassified	GBIF: https://doi.org/10.15468/dl.zfaw79
Anopheles gambiae	34.026	0.770017	Unclassified	GBIF: https://doi.org/10.15468/dl.zfaw79
Anopheles gambiae	34.0261	0.77	Unclassified	GBIF: https://doi.org/10.15468/dl.zfaw79
Anopheles gambiae	34.0261	0.770116	Unclassified	GBIF: https://doi.org/10.15468/dl.zfaw79

Anopheles gambiae	34.0261	0.770098	Unclassified	GBIF: https://doi.org/10.15468/dl.zfaw79
Anopheles gambiae	34.0261	0.770094	Unclassified	GBIF: https://doi.org/10.15468/dl.zfaw79
Anopheles gambiae	34.0261	0.77008	Unclassified	GBIF: https://doi.org/10.15468/dl.zfaw79
Anopheles gambiae	34.0261	0.770103	Unclassified	GBIF: https://doi.org/10.15468/dl.zfaw79
Anopheles gambiae	34.0261	0.770117	Unclassified	GBIF: https://doi.org/10.15468/dl.zfaw79
Anopheles gambiae	34.0261	0.770104	Unclassified	GBIF: https://doi.org/10.15468/dl.zfaw79
Anopheles gambiae	34.0261	0.770089	Unclassified	GBIF: https://doi.org/10.15468/dl.zfaw79
Anopheles gambiae	34.0261	0.770118	Unclassified	GBIF: https://doi.org/10.15468/dl.zfaw79
Anopheles gambiae	34.0261	0.77007	Unclassified	GBIF: https://doi.org/10.15468/dl.zfaw79
Anopheles gambiae	34.0261	0.770075	Unclassified	GBIF: https://doi.org/10.15468/dl.zfaw79
Anopheles gambiae	34.0261	0.770067	Unclassified	GBIF: https://doi.org/10.15468/dl.zfaw79
Anopheles gambiae	34.0261	0.770073	Unclassified	GBIF: https://doi.org/10.15468/dl.zfaw79
Anopheles gambiae	34.0261	0.770083	Unclassified	GBIF: https://doi.org/10.15468/dl.zfaw79
Anopheles gambiae	34.0261	0.770115	Unclassified	GBIF: https://doi.org/10.15468/dl.zfaw79
Anopheles gambiae	34.0261	0.770081	Unclassified	GBIF: https://doi.org/10.15468/dl.zfaw79
Anopheles gambiae	34.0261	0.770087	Unclassified	GBIF: https://doi.org/10.15468/dl.zfaw79
Anopheles gambiae	34.0261	0.770101	Unclassified	GBIF: https://doi.org/10.15468/dl.zfaw79
Anopheles gambiae	34.0261	0.770088	Unclassified	GBIF: https://doi.org/10.15468/dl.zfaw79
Anopheles gambiae	34.0261	0.770108	Unclassified	GBIF: https://doi.org/10.15468/dl.zfaw79
Anopheles gambiae	34.0261	0.77011	Unclassified	GBIF: https://doi.org/10.15468/dl.zfaw79
Anopheles gambiae	34.0261	0.770076	Unclassified	GBIF: https://doi.org/10.15468/dl.zfaw79
Anopheles gambiae	34.0261	0.770082	Unclassified	GBIF: https://doi.org/10.15468/dl.zfaw79
Anopheles gambiae	34.0261	0.770109	Unclassified	GBIF: https://doi.org/10.15468/dl.zfaw79
Anopheles gambiae	34.0261	0.770105	Unclassified	GBIF: https://doi.org/10.15468/dl.zfaw79
Anopheles gambiae	34.0261	0.7701	Unclassified	GBIF: https://doi.org/10.15468/dl.zfaw79
Anopheles gambiae	34.0261	0.770114	Unclassified	GBIF: https://doi.org/10.15468/dl.zfaw79
Anopheles gambiae	34.0261	0.77009	Unclassified	GBIF: https://doi.org/10.15468/dl.zfaw79
Anopheles gambiae	34.0261	0.770111	Unclassified	GBIF: https://doi.org/10.15468/dl.zfaw79
Anopheles gambiae	34.0261	0.770074	Unclassified	GBIF: https://doi.org/10.15468/dl.zfaw79
Anopheles gambiae	34.0261	0.770055	Unclassified	GBIF: https://doi.org/10.15468/dl.zfaw79
Anopheles gambiae	34.0261	0.770059	Unclassified	GBIF: https://doi.org/10.15468/dl.zfaw79
Anopheles gambiae	34.0261	0.770069	Unclassified	GBIF: https://doi.org/10.15468/dl.zfaw79
Anopheles gambiae	34.0261	0.770113	Unclassified	GBIF: https://doi.org/10.15468/dl.zfaw79
Anopheles gambiae	34.0261	0.770056	Unclassified	GBIF: https://doi.org/10.15468/dl.zfaw79
Anopheles gambiae	34.0261	0.770057	Unclassified	GBIF: https://doi.org/10.15468/dl.zfaw79
Anopheles gambiae	34.0261	0.770068	Unclassified	GBIF: https://doi.org/10.15468/dl.zfaw79
Anopheles gambiae	34.0261	0.770085	Unclassified	GBIF: https://doi.org/10.15468/dl.zfaw79
Anopheles gambiae	34.0261	0.770097	Unclassified	GBIF: https://doi.org/10.15468/dl.zfaw79
Anopheles gambiae	34.0261	0.770112	Unclassified	GBIF: https://doi.org/10.15468/dl.zfaw79
Anopheles gambiae	34.0261	0.77005	Unclassified	GBIF: https://doi.org/10.15468/dl.zfaw79
Anopheles gambiae	34.0261	0.770107	Unclassified	GBIF: https://doi.org/10.15468/dl.zfaw79
Anopheles gambiae	34.0261	0.770091	Unclassified	GBIF: https://doi.org/10.15468/dl.zfaw79

Anopheles gambiae	34.0261	0.770053	Unclassified	GBIF: https://doi.org/10.15468/dl.zfaw79
Anopheles gambiae	34.0261	0.770072	Unclassified	GBIF: https://doi.org/10.15468/dl.zfaw79
Anopheles gambiae	34.0261	0.770052	Unclassified	GBIF: https://doi.org/10.15468/dl.zfaw79
Anopheles gambiae	34.0261	0.770049	Unclassified	GBIF: https://doi.org/10.15468/dl.zfaw79
Anopheles gambiae	34.0261	0.770096	Unclassified	GBIF: https://doi.org/10.15468/dl.zfaw79
Anopheles gambiae	34.0261	0.770077	Unclassified	GBIF: https://doi.org/10.15468/dl.zfaw79
Anopheles gambiae	34.0261	0.770054	Unclassified	GBIF: https://doi.org/10.15468/dl.zfaw79
Anopheles gambiae	34.0261	0.770066	Unclassified	GBIF: https://doi.org/10.15468/dl.zfaw79
Anopheles gambiae	34.0261	0.770064	Unclassified	GBIF: https://doi.org/10.15468/dl.zfaw79
Anopheles gambiae	34.0261	0.770092	Unclassified	GBIF: https://doi.org/10.15468/dl.zfaw79
Anopheles gambiae	34.0261	0.770062	Unclassified	GBIF: https://doi.org/10.15468/dl.zfaw79
Anopheles gambiae	34.0261	0.770099	Unclassified	GBIF: https://doi.org/10.15468/dl.zfaw79
Anopheles gambiae	34.0261	0.770071	Unclassified	GBIF: https://doi.org/10.15468/dl.zfaw79
Anopheles gambiae	34.0261	0.770079	Unclassified	GBIF: https://doi.org/10.15468/dl.zfaw79
Anopheles gambiae	34.0261	0.770065	Unclassified	GBIF: https://doi.org/10.15468/dl.zfaw79
Anopheles gambiae	34.0261	0.770051	Unclassified	GBIF: https://doi.org/10.15468/dl.zfaw79
Anopheles gambiae	34.0261	0.770061	Unclassified	GBIF: https://doi.org/10.15468/dl.zfaw79
Anopheles gambiae	34.0261	0.77006	Unclassified	GBIF: https://doi.org/10.15468/dl.zfaw79
Anopheles gambiae	34.0261	0.770095	Unclassified	GBIF: https://doi.org/10.15468/dl.zfaw79
Anopheles gambiae	34.0261	0.770106	Unclassified	GBIF: https://doi.org/10.15468/dl.zfaw79
Anopheles gambiae	34.0261	0.770084	Unclassified	GBIF: https://doi.org/10.15468/dl.zfaw79
Anopheles gambiae	34.0261	0.770086	Unclassified	GBIF: https://doi.org/10.15468/dl.zfaw79
Anopheles gambiae	34.0261	0.770078	Unclassified	GBIF: https://doi.org/10.15468/dl.zfaw79
Anopheles gambiae	34.0261	0.770093	Unclassified	GBIF: https://doi.org/10.15468/dl.zfaw79
Anopheles gambiae	34.0261	0.770058	Unclassified	GBIF: https://doi.org/10.15468/dl.zfaw79
Anopheles gambiae	34.0261	0.770063	Unclassified	GBIF: https://doi.org/10.15468/dl.zfaw79
Anopheles gambiae	34.0261	0.770102	Unclassified	GBIF: https://doi.org/10.15468/dl.zfaw79
Anopheles gambiae	34.05	0.1	Unclassified	GBIF: https://doi.org/10.15468/dl.zfaw79
Anopheles gambiae	34.11	0.01	Unclassified	GBIF: https://doi.org/10.15468/dl.zfaw79
Anopheles gambiae	34.14	-0.16	Unclassified	GBIF: https://doi.org/10.15468/dl.zfaw79
Anopheles gambiae	34.15	0.51	Unclassified	GBIF: https://doi.org/10.15468/dl.zfaw79
Anopheles gambiae	34.1847	0.681882	Unclassified	GBIF: https://doi.org/10.15468/dl.zfaw79
Anopheles gambiae	34.2	-0.42	Unclassified	GBIF: https://doi.org/10.15468/dl.zfaw79
Anopheles gambiae	34.21	-0.43	Unclassified	GBIF: https://doi.org/10.15468/dl.zfaw79
Anopheles gambiae	34.22	0.61	Unclassified	GBIF: https://doi.org/10.15468/dl.zfaw79
Anopheles gambiae	34.23	0.58	Unclassified	GBIF: https://doi.org/10.15468/dl.zfaw79
Anopheles gambiae	34.25	-0.66667	Unclassified	GBIF: https://doi.org/10.15468/dl.zfaw79
Anopheles gambiae	34.34	0.71	Unclassified	GBIF: https://doi.org/10.15468/dl.zfaw79
Anopheles gambiae	34.35	-9.55	Unclassified	GBIF: https://doi.org/10.15468/dl.zfaw79
Anopheles gambiae	34.38333	-0.16667	Unclassified	GBIF: https://doi.org/10.15468/dl.zfaw79
Anopheles gambiae	34.41	0.02	Unclassified	GBIF: https://doi.org/10.15468/dl.zfaw79
Anopheles gambiae	34.42	0.62	Unclassified	GBIF: https://doi.org/10.15468/dl.zfaw79

Anopheles gambiae	34.5	0.50003	Unclassified	GBIF: https://doi.org/10.15468/dl.zfaw79
Anopheles gambiae	34.5	0.50005	Unclassified	GBIF: https://doi.org/10.15468/dl.zfaw79
Anopheles gambiae	34.5	0.50001	Unclassified	GBIF: https://doi.org/10.15468/dl.zfaw79
Anopheles gambiae	34.5	0.50004	Unclassified	GBIF: https://doi.org/10.15468/dl.zfaw79
Anopheles gambiae	34.5	0.50002	Unclassified	GBIF: https://doi.org/10.15468/dl.zfaw79
Anopheles gambiae	34.5	-18.96667	Unclassified	GBIF: https://doi.org/10.15468/dl.zfaw79
Anopheles gambiae	34.5001	0.50009	Unclassified	GBIF: https://doi.org/10.15468/dl.zfaw79
Anopheles gambiae	34.5001	0.50013	Unclassified	GBIF: https://doi.org/10.15468/dl.zfaw79
Anopheles gambiae	34.5001	0.50011	Unclassified	GBIF: https://doi.org/10.15468/dl.zfaw79
Anopheles gambiae	34.5001	0.50008	Unclassified	GBIF: https://doi.org/10.15468/dl.zfaw79
Anopheles gambiae	34.5001	0.50014	Unclassified	GBIF: https://doi.org/10.15468/dl.zfaw79
Anopheles gambiae	34.5001	0.5001	Unclassified	GBIF: https://doi.org/10.15468/dl.zfaw79
Anopheles gambiae	34.5001	0.50015	Unclassified	GBIF: https://doi.org/10.15468/dl.zfaw79
Anopheles gambiae	34.5001	0.50012	Unclassified	GBIF: https://doi.org/10.15468/dl.zfaw79
Anopheles gambiae	34.5001	0.50006	Unclassified	GBIF: https://doi.org/10.15468/dl.zfaw79
Anopheles gambiae	34.5001	0.50007	Unclassified	GBIF: https://doi.org/10.15468/dl.zfaw79
Anopheles gambiae	34.5002	0.50022	Unclassified	GBIF: https://doi.org/10.15468/dl.zfaw79
Anopheles gambiae	34.5002	0.5002	Unclassified	GBIF: https://doi.org/10.15468/dl.zfaw79
Anopheles gambiae	34.5002	0.50023	Unclassified	GBIF: https://doi.org/10.15468/dl.zfaw79
Anopheles gambiae	34.5002	0.50024	Unclassified	GBIF: https://doi.org/10.15468/dl.zfaw79
Anopheles gambiae	34.5002	0.50019	Unclassified	GBIF: https://doi.org/10.15468/dl.zfaw79
Anopheles gambiae	34.5002	0.50017	Unclassified	GBIF: https://doi.org/10.15468/dl.zfaw79
Anopheles gambiae	34.5002	0.50016	Unclassified	GBIF: https://doi.org/10.15468/dl.zfaw79
Anopheles gambiae	34.5002	0.50021	Unclassified	GBIF: https://doi.org/10.15468/dl.zfaw79
Anopheles gambiae	34.5002	0.50018	Unclassified	GBIF: https://doi.org/10.15468/dl.zfaw79
Anopheles gambiae	34.5003	0.50034	Unclassified	GBIF: https://doi.org/10.15468/dl.zfaw79
Anopheles gambiae	34.5003	0.50026	Unclassified	GBIF: https://doi.org/10.15468/dl.zfaw79
Anopheles gambiae	34.5003	0.50025	Unclassified	GBIF: https://doi.org/10.15468/dl.zfaw79
Anopheles gambiae	34.5003	0.50032	Unclassified	GBIF: https://doi.org/10.15468/dl.zfaw79
Anopheles gambiae	34.5003	0.50033	Unclassified	GBIF: https://doi.org/10.15468/dl.zfaw79
Anopheles gambiae	34.5003	0.50027	Unclassified	GBIF: https://doi.org/10.15468/dl.zfaw79
Anopheles gambiae	34.5003	0.5003	Unclassified	GBIF: https://doi.org/10.15468/dl.zfaw79
Anopheles gambiae	34.5003	0.50031	Unclassified	GBIF: https://doi.org/10.15468/dl.zfaw79
Anopheles gambiae	34.5003	0.50029	Unclassified	GBIF: https://doi.org/10.15468/dl.zfaw79
Anopheles gambiae	34.5003	0.50028	Unclassified	GBIF: https://doi.org/10.15468/dl.zfaw79
Anopheles gambiae	34.5004	0.50042	Unclassified	GBIF: https://doi.org/10.15468/dl.zfaw79
Anopheles gambiae	34.5004	0.50037	Unclassified	GBIF: https://doi.org/10.15468/dl.zfaw79
Anopheles gambiae	34.5004	0.50036	Unclassified	GBIF: https://doi.org/10.15468/dl.zfaw79
Anopheles gambiae	34.5004	0.50039	Unclassified	GBIF: https://doi.org/10.15468/dl.zfaw79
Anopheles gambiae	34.5004	0.50044	Unclassified	GBIF: https://doi.org/10.15468/dl.zfaw79
Anopheles gambiae	34.5004	0.50035	Unclassified	GBIF: https://doi.org/10.15468/dl.zfaw79
Anopheles gambiae	34.5004	0.50041	Unclassified	GBIF: https://doi.org/10.15468/dl.zfaw79

Anopheles gambiae	34.5004	0.50038	Unclassified	GBIF: https://doi.org/10.15468/dl.zfaw79
Anopheles gambiae	34.5004	0.50043	Unclassified	GBIF: https://doi.org/10.15468/dl.zfaw79
Anopheles gambiae	34.5004	0.5004	Unclassified	GBIF: https://doi.org/10.15468/dl.zfaw79
Anopheles gambiae	34.5005	0.50048	Unclassified	GBIF: https://doi.org/10.15468/dl.zfaw79
Anopheles gambiae	34.5005	0.50047	Unclassified	GBIF: https://doi.org/10.15468/dl.zfaw79
Anopheles gambiae	34.5005	0.50049	Unclassified	GBIF: https://doi.org/10.15468/dl.zfaw79
Anopheles gambiae	34.5005	0.50046	Unclassified	GBIF: https://doi.org/10.15468/dl.zfaw79
Anopheles gambiae	34.5005	0.5005	Unclassified	GBIF: https://doi.org/10.15468/dl.zfaw79
Anopheles gambiae	34.5005	0.50051	Unclassified	GBIF: https://doi.org/10.15468/dl.zfaw79
Anopheles gambiae	34.5005	0.50052	Unclassified	GBIF: https://doi.org/10.15468/dl.zfaw79
Anopheles gambiae	34.5005	0.50045	Unclassified	GBIF: https://doi.org/10.15468/dl.zfaw79
Anopheles gambiae	34.5006	0.50063	Unclassified	GBIF: https://doi.org/10.15468/dl.zfaw79
Anopheles gambiae	34.5006	0.50064	Unclassified	GBIF: https://doi.org/10.15468/dl.zfaw79
Anopheles gambiae	34.5006	0.50059	Unclassified	GBIF: https://doi.org/10.15468/dl.zfaw79
Anopheles gambiae	34.5006	0.50062	Unclassified	GBIF: https://doi.org/10.15468/dl.zfaw79
Anopheles gambiae	34.5006	0.50056	Unclassified	GBIF: https://doi.org/10.15468/dl.zfaw79
Anopheles gambiae	34.5007	0.50071	Unclassified	GBIF: https://doi.org/10.15468/dl.zfaw79
Anopheles gambiae	34.5007	0.50073	Unclassified	GBIF: https://doi.org/10.15468/dl.zfaw79
Anopheles gambiae	34.5007	0.50068	Unclassified	GBIF: https://doi.org/10.15468/dl.zfaw79
Anopheles gambiae	34.5007	0.50072	Unclassified	GBIF: https://doi.org/10.15468/dl.zfaw79
Anopheles gambiae	34.5007	0.50074	Unclassified	GBIF: https://doi.org/10.15468/dl.zfaw79
Anopheles gambiae	34.5007	0.5007	Unclassified	GBIF: https://doi.org/10.15468/dl.zfaw79
Anopheles gambiae	34.5008	0.50084	Unclassified	GBIF: https://doi.org/10.15468/dl.zfaw79
Anopheles gambiae	34.5008	0.50078	Unclassified	GBIF: https://doi.org/10.15468/dl.zfaw79
Anopheles gambiae	34.5008	0.50081	Unclassified	GBIF: https://doi.org/10.15468/dl.zfaw79
Anopheles gambiae	34.5008	0.50077	Unclassified	GBIF: https://doi.org/10.15468/dl.zfaw79
Anopheles gambiae	34.5008	0.50083	Unclassified	GBIF: https://doi.org/10.15468/dl.zfaw79
Anopheles gambiae	34.5008	0.50082	Unclassified	GBIF: https://doi.org/10.15468/dl.zfaw79
Anopheles gambiae	34.5008	0.50075	Unclassified	GBIF: https://doi.org/10.15468/dl.zfaw79
Anopheles gambiae	34.5009	0.50086	Unclassified	GBIF: https://doi.org/10.15468/dl.zfaw79
Anopheles gambiae	34.5009	0.50087	Unclassified	GBIF: https://doi.org/10.15468/dl.zfaw79
Anopheles gambiae	34.5009	0.50088	Unclassified	GBIF: https://doi.org/10.15468/dl.zfaw79
Anopheles gambiae	34.5009	0.50085	Unclassified	GBIF: https://doi.org/10.15468/dl.zfaw79
Anopheles gambiae	34.61	-0.44	Unclassified	GBIF: https://doi.org/10.15468/dl.zfaw79
Anopheles gambiae	34.66667	0.78333	Unclassified	GBIF: https://doi.org/10.15468/dl.zfaw79
Anopheles gambiae	34.68333	-0.06667	Unclassified	GBIF: https://doi.org/10.15468/dl.zfaw79
Anopheles gambiae	34.74	0.16	Unclassified	GBIF: https://doi.org/10.15468/dl.zfaw79
Anopheles gambiae	34.75	-12.75	Unclassified	GBIF: https://doi.org/10.15468/dl.zfaw79
Anopheles gambiae	34.81667	-13.46667	Unclassified	GBIF: https://doi.org/10.15468/dl.zfaw79
Anopheles gambiae	34.8717	-13.4217	Unclassified	GBIF: https://doi.org/10.15468/dl.zfaw79
Anopheles gambiae	34.93	-0.16	Unclassified	GBIF: https://doi.org/10.15468/dl.zfaw79
Anopheles gambiae	34.94	-0.13	Unclassified	GBIF: https://doi.org/10.15468/dl.zfaw79

Anopheles gambiae	35.114853	-15.065292	Unclassified	GBIF: https://doi.org/10.15468/dl.zfaw79
Anopheles gambiae	35.13333	-13	Unclassified	GBIF: https://doi.org/10.15468/dl.zfaw79
Anopheles gambiae	35.26667	-13.48333	Unclassified	GBIF: https://doi.org/10.15468/dl.zfaw79
Anopheles gambiae	35.31	-23.72	Unclassified	GBIF: https://doi.org/10.15468/dl.zfaw79
Anopheles gambiae	35.46667	-18.28333	Unclassified	GBIF: https://doi.org/10.15468/dl.zfaw79
Anopheles gambiae	35.5	-1.5	Unclassified	GBIF: https://doi.org/10.15468/dl.zfaw79
Anopheles gambiae	35.528479	-14.873062	Unclassified	GBIF: https://doi.org/10.15468/dl.zfaw79
Anopheles gambiae	35.83333	-7.13333	Unclassified	GBIF: https://doi.org/10.15468/dl.zfaw79
Anopheles gambiae	35.93333	-11.16667	Unclassified	GBIF: https://doi.org/10.15468/dl.zfaw79
Anopheles gambiae	36	-16.9	Unclassified	GBIF: https://doi.org/10.15468/dl.zfaw79
Anopheles gambiae	36.02	0.65	Unclassified	GBIF: https://doi.org/10.15468/dl.zfaw79
Anopheles gambiae	36.03	0.62	Unclassified	GBIF: https://doi.org/10.15468/dl.zfaw79
Anopheles gambiae	36.1	0.46	Unclassified	GBIF: https://doi.org/10.15468/dl.zfaw79
Anopheles gambiae	36.36	-8.16668	Unclassified	GBIF: https://doi.org/10.15468/dl.zfaw79
Anopheles gambiae	36.3623	-8.17202	Unclassified	GBIF: https://doi.org/10.15468/dl.zfaw79
Anopheles gambiae	36.3627	-8.15718	Unclassified	GBIF: https://doi.org/10.15468/dl.zfaw79
Anopheles gambiae	36.3644	-8.15527	Unclassified	GBIF: https://doi.org/10.15468/dl.zfaw79
Anopheles gambiae	36.365	-8.16375	Unclassified	GBIF: https://doi.org/10.15468/dl.zfaw79
Anopheles gambiae	36.3681	-8.1713	Unclassified	GBIF: https://doi.org/10.15468/dl.zfaw79
Anopheles gambiae	36.3694	-8.15503	Unclassified	GBIF: https://doi.org/10.15468/dl.zfaw79
Anopheles gambiae	36.3715	-8.16998	Unclassified	GBIF: https://doi.org/10.15468/dl.zfaw79
Anopheles gambiae	36.3807	-8.18135	Unclassified	GBIF: https://doi.org/10.15468/dl.zfaw79
Anopheles gambiae	36.384	-8.1683	Unclassified	GBIF: https://doi.org/10.15468/dl.zfaw79
Anopheles gambiae	36.386	-8.18618	Unclassified	GBIF: https://doi.org/10.15468/dl.zfaw79
Anopheles gambiae	36.387	-8.18568	Unclassified	GBIF: https://doi.org/10.15468/dl.zfaw79
Anopheles gambiae	36.3876	-8.18327	Unclassified	GBIF: https://doi.org/10.15468/dl.zfaw79
Anopheles gambiae	36.3883	-8.17953	Unclassified	GBIF: https://doi.org/10.15468/dl.zfaw79
Anopheles gambiae	36.3893	-8.17788	Unclassified	GBIF: https://doi.org/10.15468/dl.zfaw79
Anopheles gambiae	36.3907	-8.15435	Unclassified	GBIF: https://doi.org/10.15468/dl.zfaw79
Anopheles gambiae	36.3908	-8.17755	Unclassified	GBIF: https://doi.org/10.15468/dl.zfaw79
Anopheles gambiae	36.3916	-8.22153	Unclassified	GBIF: https://doi.org/10.15468/dl.zfaw79
Anopheles gambiae	36.3917	-8.15608	Unclassified	GBIF: https://doi.org/10.15468/dl.zfaw79
Anopheles gambiae	36.3919	-8.17112	Unclassified	GBIF: https://doi.org/10.15468/dl.zfaw79
Anopheles gambiae	36.3919	-8.17193	Unclassified	GBIF: https://doi.org/10.15468/dl.zfaw79
Anopheles gambiae	36.3927	-8.17417	Unclassified	GBIF: https://doi.org/10.15468/dl.zfaw79
Anopheles gambiae	36.3938	-8.15408	Unclassified	GBIF: https://doi.org/10.15468/dl.zfaw79
Anopheles gambiae	36.3942	-8.15687	Unclassified	GBIF: https://doi.org/10.15468/dl.zfaw79
Anopheles gambiae	36.3943	-8.15415	Unclassified	GBIF: https://doi.org/10.15468/dl.zfaw79
Anopheles gambiae	36.3943	-8.15422	Unclassified	GBIF: https://doi.org/10.15468/dl.zfaw79
Anopheles gambiae	36.3952	-8.15473	Unclassified	GBIF: https://doi.org/10.15468/dl.zfaw79
Anopheles gambiae	36.3952	-8.15354	Unclassified	GBIF: https://doi.org/10.15468/dl.zfaw79
Anopheles gambiae	36.3955	-8.15603	Unclassified	GBIF: https://doi.org/10.15468/dl.zfaw79

Anopheles gambiae	36.3956	-8.15978	Unclassified	GBIF: https://doi.org/10.15468/dl.zfaw79
Anopheles gambiae	36.3956	-8.15948	Unclassified	GBIF: https://doi.org/10.15468/dl.zfaw79
Anopheles gambiae	36.3956	-8.15543	Unclassified	GBIF: https://doi.org/10.15468/dl.zfaw79
Anopheles gambiae	36.3958	-8.15662	Unclassified	GBIF: https://doi.org/10.15468/dl.zfaw79
Anopheles gambiae	36.3959	-8.15885	Unclassified	GBIF: https://doi.org/10.15468/dl.zfaw79
Anopheles gambiae	36.3959	-8.15623	Unclassified	GBIF: https://doi.org/10.15468/dl.zfaw79
Anopheles gambiae	36.3962	-8.15373	Unclassified	GBIF: https://doi.org/10.15468/dl.zfaw79
Anopheles gambiae	36.3963	-8.15737	Unclassified	GBIF: https://doi.org/10.15468/dl.zfaw79
Anopheles gambiae	36.3963	-8.15403	Unclassified	GBIF: https://doi.org/10.15468/dl.zfaw79
Anopheles gambiae	36.3966	-8.1542	Unclassified	GBIF: https://doi.org/10.15468/dl.zfaw79
Anopheles gambiae	36.3968	-8.15583	Unclassified	GBIF: https://doi.org/10.15468/dl.zfaw79
Anopheles gambiae	36.397	-8.19995	Unclassified	GBIF: https://doi.org/10.15468/dl.zfaw79
Anopheles gambiae	36.3971	-8.15353	Unclassified	GBIF: https://doi.org/10.15468/dl.zfaw79
Anopheles gambiae	36.3972	-8.15433	Unclassified	GBIF: https://doi.org/10.15468/dl.zfaw79
Anopheles gambiae	36.3974	-8.15251	Unclassified	GBIF: https://doi.org/10.15468/dl.zfaw79
Anopheles gambiae	36.3975	-8.15263	Unclassified	GBIF: https://doi.org/10.15468/dl.zfaw79
Anopheles gambiae	36.3975	-8.1525	Unclassified	GBIF: https://doi.org/10.15468/dl.zfaw79
Anopheles gambiae	36.3975	-8.15543	Unclassified	GBIF: https://doi.org/10.15468/dl.zfaw79
Anopheles gambiae	36.3976	-8.15592	Unclassified	GBIF: https://doi.org/10.15468/dl.zfaw79
Anopheles gambiae	36.3976	-8.15645	Unclassified	GBIF: https://doi.org/10.15468/dl.zfaw79
Anopheles gambiae	36.3979	-8.15916	Unclassified	GBIF: https://doi.org/10.15468/dl.zfaw79
Anopheles gambiae	36.3979	-8.15625	Unclassified	GBIF: https://doi.org/10.15468/dl.zfaw79
Anopheles gambiae	36.3979	-8.15883	Unclassified	GBIF: https://doi.org/10.15468/dl.zfaw79
Anopheles gambiae	36.3982	-8.15192	Unclassified	GBIF: https://doi.org/10.15468/dl.zfaw79
Anopheles gambiae	36.3982	-8.15523	Unclassified	GBIF: https://doi.org/10.15468/dl.zfaw79
Anopheles gambiae	36.3983	-8.15672	Unclassified	GBIF: https://doi.org/10.15468/dl.zfaw79
Anopheles gambiae	36.3986	-8.15187	Unclassified	GBIF: https://doi.org/10.15468/dl.zfaw79
Anopheles gambiae	36.3991	-8.15375	Unclassified	GBIF: https://doi.org/10.15468/dl.zfaw79
Anopheles gambiae	36.3992	-8.15252	Unclassified	GBIF: https://doi.org/10.15468/dl.zfaw79
Anopheles gambiae	36.3992	-8.15548	Unclassified	GBIF: https://doi.org/10.15468/dl.zfaw79
Anopheles gambiae	36.3993	-8.22125	Unclassified	GBIF: https://doi.org/10.15468/dl.zfaw79
Anopheles gambiae	36.3994	-8.15638	Unclassified	GBIF: https://doi.org/10.15468/dl.zfaw79
Anopheles gambiae	36.3996	-8.15255	Unclassified	GBIF: https://doi.org/10.15468/dl.zfaw79
Anopheles gambiae	36.3998	-8.16152	Unclassified	GBIF: https://doi.org/10.15468/dl.zfaw79
Anopheles gambiae	36.4001	-8.1516	Unclassified	GBIF: https://doi.org/10.15468/dl.zfaw79
Anopheles gambiae	36.4002	-8.15532	Unclassified	GBIF: https://doi.org/10.15468/dl.zfaw79
Anopheles gambiae	36.4005	-8.15118	Unclassified	GBIF: https://doi.org/10.15468/dl.zfaw79
Anopheles gambiae	36.4007	-8.15705	Unclassified	GBIF: https://doi.org/10.15468/dl.zfaw79
Anopheles gambiae	36.401	-8.23767	Unclassified	GBIF: https://doi.org/10.15468/dl.zfaw79
Anopheles gambiae	36.401	-8.22303	Unclassified	GBIF: https://doi.org/10.15468/dl.zfaw79
Anopheles gambiae	36.4013	-8.23812	Unclassified	GBIF: https://doi.org/10.15468/dl.zfaw79
Anopheles gambiae	36.4015	-8.15153	Unclassified	GBIF: https://doi.org/10.15468/dl.zfaw79

Anopheles gambiae	36.4025	-8.15143	Unclassified	GBIF: https://doi.org/10.15468/dl.zfaw79
Anopheles gambiae	36.4029	-8.2021	Unclassified	GBIF: https://doi.org/10.15468/dl.zfaw79
Anopheles gambiae	36.4047	-8.20558	Unclassified	GBIF: https://doi.org/10.15468/dl.zfaw79
Anopheles gambiae	36.405	-8.15203	Unclassified	GBIF: https://doi.org/10.15468/dl.zfaw79
Anopheles gambiae	36.4173	-8.14743	Unclassified	GBIF: https://doi.org/10.15468/dl.zfaw79
Anopheles gambiae	36.4178	-8.22123	Unclassified	GBIF: https://doi.org/10.15468/dl.zfaw79
Anopheles gambiae	36.4191	-8.22228	Unclassified	GBIF: https://doi.org/10.15468/dl.zfaw79
Anopheles gambiae	36.4193	-8.14423	Unclassified	GBIF: https://doi.org/10.15468/dl.zfaw79
Anopheles gambiae	36.4193	-8.1442	Unclassified	GBIF: https://doi.org/10.15468/dl.zfaw79
Anopheles gambiae	36.4209	-8.14275	Unclassified	GBIF: https://doi.org/10.15468/dl.zfaw79
Anopheles gambiae	36.424	-8.1382	Unclassified	GBIF: https://doi.org/10.15468/dl.zfaw79
Anopheles gambiae	36.4891	-8.07934	Unclassified	GBIF: https://doi.org/10.15468/dl.zfaw79
Anopheles gambiae	36.4948	-8.08026	Unclassified	GBIF: https://doi.org/10.15468/dl.zfaw79
Anopheles gambiae	36.4989	-8.08888	Unclassified	GBIF: https://doi.org/10.15468/dl.zfaw79
Anopheles gambiae	36.499	-8.09943	Unclassified	GBIF: https://doi.org/10.15468/dl.zfaw79
Anopheles gambiae	36.4999	-8.09793	Unclassified	GBIF: https://doi.org/10.15468/dl.zfaw79
Anopheles gambiae	36.5022	-8.09957	Unclassified	GBIF: https://doi.org/10.15468/dl.zfaw79
Anopheles gambiae	36.5065	-8.0972	Unclassified	GBIF: https://doi.org/10.15468/dl.zfaw79
Anopheles gambiae	36.5071	-8.10013	Unclassified	GBIF: https://doi.org/10.15468/dl.zfaw79
Anopheles gambiae	36.5083	-8.10252	Unclassified	GBIF: https://doi.org/10.15468/dl.zfaw79
Anopheles gambiae	36.5084	-8.1015	Unclassified	GBIF: https://doi.org/10.15468/dl.zfaw79
Anopheles gambiae	36.5086	-8.10095	Unclassified	GBIF: https://doi.org/10.15468/dl.zfaw79
Anopheles gambiae	36.5088	-8.10113	Unclassified	GBIF: https://doi.org/10.15468/dl.zfaw79
Anopheles gambiae	36.5088	-8.08348	Unclassified	GBIF: https://doi.org/10.15468/dl.zfaw79
Anopheles gambiae	36.5089	-8.10205	Unclassified	GBIF: https://doi.org/10.15468/dl.zfaw79
Anopheles gambiae	36.5089	-8.10162	Unclassified	GBIF: https://doi.org/10.15468/dl.zfaw79
Anopheles gambiae	36.509	-8.1024	Unclassified	GBIF: https://doi.org/10.15468/dl.zfaw79
Anopheles gambiae	36.509	-8.09308	Unclassified	GBIF: https://doi.org/10.15468/dl.zfaw79
Anopheles gambiae	36.5091	-8.10053	Unclassified	GBIF: https://doi.org/10.15468/dl.zfaw79
Anopheles gambiae	36.5092	-8.10068	Unclassified	GBIF: https://doi.org/10.15468/dl.zfaw79
Anopheles gambiae	36.5093	-8.09962	Unclassified	GBIF: https://doi.org/10.15468/dl.zfaw79
Anopheles gambiae	36.5093	-8.1007	Unclassified	GBIF: https://doi.org/10.15468/dl.zfaw79
Anopheles gambiae	36.5097	-8.09598	Unclassified	GBIF: https://doi.org/10.15468/dl.zfaw79
Anopheles gambiae	36.5098	-8.09598	Unclassified	GBIF: https://doi.org/10.15468/dl.zfaw79
Anopheles gambiae	36.51	-8.10127	Unclassified	GBIF: https://doi.org/10.15468/dl.zfaw79
Anopheles gambiae	36.5102	-8.10118	Unclassified	GBIF: https://doi.org/10.15468/dl.zfaw79
Anopheles gambiae	36.5103	-8.09778	Unclassified	GBIF: https://doi.org/10.15468/dl.zfaw79
Anopheles gambiae	36.5103	-8.10002	Unclassified	GBIF: https://doi.org/10.15468/dl.zfaw79
Anopheles gambiae	36.5104	-8.09688	Unclassified	GBIF: https://doi.org/10.15468/dl.zfaw79
Anopheles gambiae	36.5105	-8.09998	Unclassified	GBIF: https://doi.org/10.15468/dl.zfaw79
Anopheles gambiae	36.5107	-8.09993	Unclassified	GBIF: https://doi.org/10.15468/dl.zfaw79
Anopheles gambiae	36.5107	-8.10348	Unclassified	GBIF: https://doi.org/10.15468/dl.zfaw79

Anopheles gambiae	36.5108	-8.10322	Unclassified	GBIF: https://doi.org/10.15468/dl.zfaw79
Anopheles gambiae	36.5108	-8.10037	Unclassified	GBIF: https://doi.org/10.15468/dl.zfaw79
Anopheles gambiae	36.511	-8.10002	Unclassified	GBIF: https://doi.org/10.15468/dl.zfaw79
Anopheles gambiae	36.511	-8.09795	Unclassified	GBIF: https://doi.org/10.15468/dl.zfaw79
Anopheles gambiae	36.5111	-8.09627	Unclassified	GBIF: https://doi.org/10.15468/dl.zfaw79
Anopheles gambiae	36.5111	-8.10248	Unclassified	GBIF: https://doi.org/10.15468/dl.zfaw79
Anopheles gambiae	36.5111	-8.098	Unclassified	GBIF: https://doi.org/10.15468/dl.zfaw79
Anopheles gambiae	36.5114	-8.09942	Unclassified	GBIF: https://doi.org/10.15468/dl.zfaw79
Anopheles gambiae	36.5115	-8.10038	Unclassified	GBIF: https://doi.org/10.15468/dl.zfaw79
Anopheles gambiae	36.5116	-8.0941	Unclassified	GBIF: https://doi.org/10.15468/dl.zfaw79
Anopheles gambiae	36.5121	-8.09948	Unclassified	GBIF: https://doi.org/10.15468/dl.zfaw79
Anopheles gambiae	36.5122	-8.10003	Unclassified	GBIF: https://doi.org/10.15468/dl.zfaw79
Anopheles gambiae	36.5128	-8.09644	Unclassified	GBIF: https://doi.org/10.15468/dl.zfaw79
Anopheles gambiae	36.5131	-8.1007	Unclassified	GBIF: https://doi.org/10.15468/dl.zfaw79
Anopheles gambiae	36.5133	-8.09995	Unclassified	GBIF: https://doi.org/10.15468/dl.zfaw79
Anopheles gambiae	36.5155	-8.10778	Unclassified	GBIF: https://doi.org/10.15468/dl.zfaw79
Anopheles gambiae	36.5168	-8.1001	Unclassified	GBIF: https://doi.org/10.15468/dl.zfaw79
Anopheles gambiae	36.5173	-8.09877	Unclassified	GBIF: https://doi.org/10.15468/dl.zfaw79
Anopheles gambiae	36.5177	-8.09588	Unclassified	GBIF: https://doi.org/10.15468/dl.zfaw79
Anopheles gambiae	36.5178	-8.09894	Unclassified	GBIF: https://doi.org/10.15468/dl.zfaw79
Anopheles gambiae	36.5182	-8.09975	Unclassified	GBIF: https://doi.org/10.15468/dl.zfaw79
Anopheles gambiae	36.5189	-8.09853	Unclassified	GBIF: https://doi.org/10.15468/dl.zfaw79
Anopheles gambiae	36.5199	-8.10857	Unclassified	GBIF: https://doi.org/10.15468/dl.zfaw79
Anopheles gambiae	36.5202	-8.0986	Unclassified	GBIF: https://doi.org/10.15468/dl.zfaw79
Anopheles gambiae	36.5202	-8.10823	Unclassified	GBIF: https://doi.org/10.15468/dl.zfaw79
Anopheles gambiae	36.5202	-8.1081	Unclassified	GBIF: https://doi.org/10.15468/dl.zfaw79
Anopheles gambiae	36.5205	-8.1017	Unclassified	GBIF: https://doi.org/10.15468/dl.zfaw79
Anopheles gambiae	36.5206	-8.10062	Unclassified	GBIF: https://doi.org/10.15468/dl.zfaw79
Anopheles gambiae	36.5208	-8.1011	Unclassified	GBIF: https://doi.org/10.15468/dl.zfaw79
Anopheles gambiae	36.5209	-8.09753	Unclassified	GBIF: https://doi.org/10.15468/dl.zfaw79
Anopheles gambiae	36.521	-8.0977	Unclassified	GBIF: https://doi.org/10.15468/dl.zfaw79
Anopheles gambiae	36.5214	-8.1098	Unclassified	GBIF: https://doi.org/10.15468/dl.zfaw79
Anopheles gambiae	36.5227	-8.09815	Unclassified	GBIF: https://doi.org/10.15468/dl.zfaw79
Anopheles gambiae	36.5232	-8.09953	Unclassified	GBIF: https://doi.org/10.15468/dl.zfaw79
Anopheles gambiae	36.5234	-8.108	Unclassified	GBIF: https://doi.org/10.15468/dl.zfaw79
Anopheles gambiae	36.524	-8.08778	Unclassified	GBIF: https://doi.org/10.15468/dl.zfaw79
Anopheles gambiae	36.524	-8.10003	Unclassified	GBIF: https://doi.org/10.15468/dl.zfaw79
Anopheles gambiae	36.5243	-8.10058	Unclassified	GBIF: https://doi.org/10.15468/dl.zfaw79
Anopheles gambiae	36.5264	-8.09977	Unclassified	GBIF: https://doi.org/10.15468/dl.zfaw79
Anopheles gambiae	36.5283	-8.10648	Unclassified	GBIF: https://doi.org/10.15468/dl.zfaw79
Anopheles gambiae	36.5283	-8.09658	Unclassified	GBIF: https://doi.org/10.15468/dl.zfaw79
Anopheles gambiae	36.5289	-8.09548	Unclassified	GBIF: https://doi.org/10.15468/dl.zfaw79

Anopheles gambiae	36.5296	-8.0651	Unclassified	GBIF: https://doi.org/10.15468/dl.zfaw79
Anopheles gambiae	36.5308	-8.06853	Unclassified	GBIF: https://doi.org/10.15468/dl.zfaw79
Anopheles gambiae	36.5323	-8.09362	Unclassified	GBIF: https://doi.org/10.15468/dl.zfaw79
Anopheles gambiae	36.5336	-8.06665	Unclassified	GBIF: https://doi.org/10.15468/dl.zfaw79
Anopheles gambiae	36.5343	-8.11875	Unclassified	GBIF: https://doi.org/10.15468/dl.zfaw79
Anopheles gambiae	36.549	-8.06653	Unclassified	GBIF: https://doi.org/10.15468/dl.zfaw79
Anopheles gambiae	36.5505	-8.11457	Unclassified	GBIF: https://doi.org/10.15468/dl.zfaw79
Anopheles gambiae	36.5521	-8.11192	Unclassified	GBIF: https://doi.org/10.15468/dl.zfaw79
Anopheles gambiae	36.5587	-8.13008	Unclassified	GBIF: https://doi.org/10.15468/dl.zfaw79
Anopheles gambiae	36.683	-8.14607	Unclassified	GBIF: https://doi.org/10.15468/dl.zfaw79
Anopheles gambiae	36.68333	-8.16667	Unclassified	GBIF: https://doi.org/10.15468/dl.zfaw79
Anopheles gambiae	36.7166	8.96713	Unclassified	GBIF: https://doi.org/10.15468/dl.zfaw79
Anopheles gambiae	36.73333	-17.83333	Unclassified	GBIF: https://doi.org/10.15468/dl.zfaw79
Anopheles gambiae	36.8093	8.97825	Unclassified	GBIF: https://doi.org/10.15468/dl.zfaw79
Anopheles gambiae	36.8676	8.96192	Unclassified	GBIF: https://doi.org/10.15468/dl.zfaw79
Anopheles gambiae	36.93333	-6.75	Unclassified	GBIF: https://doi.org/10.15468/dl.zfaw79
Anopheles gambiae	37.4301	5.51846	Unclassified	GBIF: https://doi.org/10.15468/dl.zfaw79
Anopheles gambiae	37.4303	5.51721	Unclassified	GBIF: https://doi.org/10.15468/dl.zfaw79
Anopheles gambiae	37.4306	5.5181	Unclassified	GBIF: https://doi.org/10.15468/dl.zfaw79
Anopheles gambiae	37.4311	5.51649	Unclassified	GBIF: https://doi.org/10.15468/dl.zfaw79
Anopheles gambiae	37.4312	5.51697	Unclassified	GBIF: https://doi.org/10.15468/dl.zfaw79
Anopheles gambiae	37.4312	5.51601	Unclassified	GBIF: https://doi.org/10.15468/dl.zfaw79
Anopheles gambiae	37.4313	5.51762	Unclassified	GBIF: https://doi.org/10.15468/dl.zfaw79
Anopheles gambiae	37.4313	5.51647	Unclassified	GBIF: https://doi.org/10.15468/dl.zfaw79
Anopheles gambiae	37.4316	5.51642	Unclassified	GBIF: https://doi.org/10.15468/dl.zfaw79
Anopheles gambiae	37.4316	5.5178	Unclassified	GBIF: https://doi.org/10.15468/dl.zfaw79
Anopheles gambiae	37.4316	5.51694	Unclassified	GBIF: https://doi.org/10.15468/dl.zfaw79
Anopheles gambiae	37.4316	5.5159	Unclassified	GBIF: https://doi.org/10.15468/dl.zfaw79
Anopheles gambiae	37.4317	5.51462	Unclassified	GBIF: https://doi.org/10.15468/dl.zfaw79
Anopheles gambiae	37.4318	5.51684	Unclassified	GBIF: https://doi.org/10.15468/dl.zfaw79
Anopheles gambiae	37.4318	5.51867	Unclassified	GBIF: https://doi.org/10.15468/dl.zfaw79
Anopheles gambiae	37.432	5.51703	Unclassified	GBIF: https://doi.org/10.15468/dl.zfaw79
Anopheles gambiae	37.4321	5.51708	Unclassified	GBIF: https://doi.org/10.15468/dl.zfaw79
Anopheles gambiae	37.4321	5.51565	Unclassified	GBIF: https://doi.org/10.15468/dl.zfaw79
Anopheles gambiae	37.4322	5.51773	Unclassified	GBIF: https://doi.org/10.15468/dl.zfaw79
Anopheles gambiae	37.4323	5.51624	Unclassified	GBIF: https://doi.org/10.15468/dl.zfaw79
Anopheles gambiae	37.4325	5.51803	Unclassified	GBIF: https://doi.org/10.15468/dl.zfaw79
Anopheles gambiae	37.4325	5.5176	Unclassified	GBIF: https://doi.org/10.15468/dl.zfaw79
Anopheles gambiae	37.4326	5.51766	Unclassified	GBIF: https://doi.org/10.15468/dl.zfaw79
Anopheles gambiae	37.4326	5.51786	Unclassified	GBIF: https://doi.org/10.15468/dl.zfaw79
Anopheles gambiae	37.4327	5.5183	Unclassified	GBIF: https://doi.org/10.15468/dl.zfaw79
Anopheles gambiae	37.4328	5.51531	Unclassified	GBIF: https://doi.org/10.15468/dl.zfaw79

Anopheles gambiae	37.4328	5.51681	Unclassified	GBIF: https://doi.org/10.15468/dl.zfaw79
Anopheles gambiae	37.4328	5.51743	Unclassified	GBIF: https://doi.org/10.15468/dl.zfaw79
Anopheles gambiae	37.4329	5.51562	Unclassified	GBIF: https://doi.org/10.15468/dl.zfaw79
Anopheles gambiae	37.4331	5.51625	Unclassified	GBIF: https://doi.org/10.15468/dl.zfaw79
Anopheles gambiae	37.4333	5.51625	Unclassified	GBIF: https://doi.org/10.15468/dl.zfaw79
Anopheles gambiae	37.4336	5.51701	Unclassified	GBIF: https://doi.org/10.15468/dl.zfaw79
Anopheles gambiae	37.4336	5.51684	Unclassified	GBIF: https://doi.org/10.15468/dl.zfaw79
Anopheles gambiae	37.4337	5.51614	Unclassified	GBIF: https://doi.org/10.15468/dl.zfaw79
Anopheles gambiae	37.4337	5.51759	Unclassified	GBIF: https://doi.org/10.15468/dl.zfaw79
Anopheles gambiae	37.4339	5.51514	Unclassified	GBIF: https://doi.org/10.15468/dl.zfaw79
Anopheles gambiae	37.434	5.51593	Unclassified	GBIF: https://doi.org/10.15468/dl.zfaw79
Anopheles gambiae	37.434	5.51612	Unclassified	GBIF: https://doi.org/10.15468/dl.zfaw79
Anopheles gambiae	37.4343	5.51616	Unclassified	GBIF: https://doi.org/10.15468/dl.zfaw79
Anopheles gambiae	37.4344	5.51751	Unclassified	GBIF: https://doi.org/10.15468/dl.zfaw79
Anopheles gambiae	37.4347	5.51576	Unclassified	GBIF: https://doi.org/10.15468/dl.zfaw79
Anopheles gambiae	37.4348	5.51693	Unclassified	GBIF: https://doi.org/10.15468/dl.zfaw79
Anopheles gambiae	37.4349	5.51672	Unclassified	GBIF: https://doi.org/10.15468/dl.zfaw79
Anopheles gambiae	37.4351	5.51556	Unclassified	GBIF: https://doi.org/10.15468/dl.zfaw79
Anopheles gambiae	37.4351	5.51692	Unclassified	GBIF: https://doi.org/10.15468/dl.zfaw79
Anopheles gambiae	37.4352	5.51648	Unclassified	GBIF: https://doi.org/10.15468/dl.zfaw79
Anopheles gambiae	37.4352	5.51757	Unclassified	GBIF: https://doi.org/10.15468/dl.zfaw79
Anopheles gambiae	37.4353	5.5169	Unclassified	GBIF: https://doi.org/10.15468/dl.zfaw79
Anopheles gambiae	37.4355	5.51566	Unclassified	GBIF: https://doi.org/10.15468/dl.zfaw79
Anopheles gambiae	37.4356	5.5156	Unclassified	GBIF: https://doi.org/10.15468/dl.zfaw79
Anopheles gambiae	37.4357	5.51643	Unclassified	GBIF: https://doi.org/10.15468/dl.zfaw79
Anopheles gambiae	37.4358	5.51669	Unclassified	GBIF: https://doi.org/10.15468/dl.zfaw79
Anopheles gambiae	37.4362	5.51563	Unclassified	GBIF: https://doi.org/10.15468/dl.zfaw79
Anopheles gambiae	37.4366	5.51635	Unclassified	GBIF: https://doi.org/10.15468/dl.zfaw79
Anopheles gambiae	37.4366	5.51619	Unclassified	GBIF: https://doi.org/10.15468/dl.zfaw79
Anopheles gambiae	37.4367	5.51664	Unclassified	GBIF: https://doi.org/10.15468/dl.zfaw79
Anopheles gambiae	37.4383	5.51589	Unclassified	GBIF: https://doi.org/10.15468/dl.zfaw79
Anopheles gambiae	37.4383	5.51578	Unclassified	GBIF: https://doi.org/10.15468/dl.zfaw79
Anopheles gambiae	37.4385	5.51542	Unclassified	GBIF: https://doi.org/10.15468/dl.zfaw79
Anopheles gambiae	37.4385	5.51576	Unclassified	GBIF: https://doi.org/10.15468/dl.zfaw79
Anopheles gambiae	37.4386	5.51565	Unclassified	GBIF: https://doi.org/10.15468/dl.zfaw79
Anopheles gambiae	37.4386	5.51539	Unclassified	GBIF: https://doi.org/10.15468/dl.zfaw79
Anopheles gambiae	37.4387	5.51507	Unclassified	GBIF: https://doi.org/10.15468/dl.zfaw79
Anopheles gambiae	37.4387	5.51553	Unclassified	GBIF: https://doi.org/10.15468/dl.zfaw79
Anopheles gambiae	37.4388	5.51533	Unclassified	GBIF: https://doi.org/10.15468/dl.zfaw79
Anopheles gambiae	37.4389	5.51569	Unclassified	GBIF: https://doi.org/10.15468/dl.zfaw79
Anopheles gambiae	37.4389	5.51578	Unclassified	GBIF: https://doi.org/10.15468/dl.zfaw79
Anopheles gambiae	37.439	5.51531	Unclassified	GBIF: https://doi.org/10.15468/dl.zfaw79

Anopheles gambiae	37.4391	5.5157	Unclassified	GBIF: https://doi.org/10.15468/dl.zfaw79
Anopheles gambiae	37.4393	5.5158	Unclassified	GBIF: https://doi.org/10.15468/dl.zfaw79
Anopheles gambiae	37.4394	5.51522	Unclassified	GBIF: https://doi.org/10.15468/dl.zfaw79
Anopheles gambiae	37.4398	5.51556	Unclassified	GBIF: https://doi.org/10.15468/dl.zfaw79
Anopheles gambiae	37.4399	5.51493	Unclassified	GBIF: https://doi.org/10.15468/dl.zfaw79
Anopheles gambiae	37.44	5.51503	Unclassified	GBIF: https://doi.org/10.15468/dl.zfaw79
Anopheles gambiae	37.4466	5.51414	Unclassified	GBIF: https://doi.org/10.15468/dl.zfaw79
Anopheles gambiae	37.4534	5.51304	Unclassified	GBIF: https://doi.org/10.15468/dl.zfaw79
Anopheles gambiae	37.4536	5.51321	Unclassified	GBIF: https://doi.org/10.15468/dl.zfaw79
Anopheles gambiae	37.4539	5.51241	Unclassified	GBIF: https://doi.org/10.15468/dl.zfaw79
Anopheles gambiae	37.454	5.51306	Unclassified	GBIF: https://doi.org/10.15468/dl.zfaw79
Anopheles gambiae	37.454	5.51177	Unclassified	GBIF: https://doi.org/10.15468/dl.zfaw79
Anopheles gambiae	37.4541	5.51169	Unclassified	GBIF: https://doi.org/10.15468/dl.zfaw79
Anopheles gambiae	37.4541	5.51111	Unclassified	GBIF: https://doi.org/10.15468/dl.zfaw79
Anopheles gambiae	37.4542	5.51244	Unclassified	GBIF: https://doi.org/10.15468/dl.zfaw79
Anopheles gambiae	37.4542	5.5123	Unclassified	GBIF: https://doi.org/10.15468/dl.zfaw79
Anopheles gambiae	37.4543	5.51281	Unclassified	GBIF: https://doi.org/10.15468/dl.zfaw79
Anopheles gambiae	37.4543	5.51165	Unclassified	GBIF: https://doi.org/10.15468/dl.zfaw79
Anopheles gambiae	37.4543	5.51241	Unclassified	GBIF: https://doi.org/10.15468/dl.zfaw79
Anopheles gambiae	37.4543	5.5126	Unclassified	GBIF: https://doi.org/10.15468/dl.zfaw79
Anopheles gambiae	37.4543	5.51147	Unclassified	GBIF: https://doi.org/10.15468/dl.zfaw79
Anopheles gambiae	37.4544	5.51196	Unclassified	GBIF: https://doi.org/10.15468/dl.zfaw79
Anopheles gambiae	37.4544	5.51087	Unclassified	GBIF: https://doi.org/10.15468/dl.zfaw79
Anopheles gambiae	37.4544	5.51316	Unclassified	GBIF: https://doi.org/10.15468/dl.zfaw79
Anopheles gambiae	37.4545	5.51097	Unclassified	GBIF: https://doi.org/10.15468/dl.zfaw79
Anopheles gambiae	37.4545	5.51169	Unclassified	GBIF: https://doi.org/10.15468/dl.zfaw79
Anopheles gambiae	37.4545	5.51276	Unclassified	GBIF: https://doi.org/10.15468/dl.zfaw79
Anopheles gambiae	37.4545	5.51158	Unclassified	GBIF: https://doi.org/10.15468/dl.zfaw79
Anopheles gambiae	37.4546	5.5105	Unclassified	GBIF: https://doi.org/10.15468/dl.zfaw79
Anopheles gambiae	37.4546	5.51243	Unclassified	GBIF: https://doi.org/10.15468/dl.zfaw79
Anopheles gambiae	37.4546	5.51204	Unclassified	GBIF: https://doi.org/10.15468/dl.zfaw79
Anopheles gambiae	37.4547	5.5124	Unclassified	GBIF: https://doi.org/10.15468/dl.zfaw79
Anopheles gambiae	37.4547	5.5123	Unclassified	GBIF: https://doi.org/10.15468/dl.zfaw79
Anopheles gambiae	37.4547	5.51249	Unclassified	GBIF: https://doi.org/10.15468/dl.zfaw79
Anopheles gambiae	37.4548	5.51278	Unclassified	GBIF: https://doi.org/10.15468/dl.zfaw79
Anopheles gambiae	37.4548	5.51062	Unclassified	GBIF: https://doi.org/10.15468/dl.zfaw79
Anopheles gambiae	37.4549	5.51282	Unclassified	GBIF: https://doi.org/10.15468/dl.zfaw79
Anopheles gambiae	37.455	5.51253	Unclassified	GBIF: https://doi.org/10.15468/dl.zfaw79
Anopheles gambiae	37.4551	5.51181	Unclassified	GBIF: https://doi.org/10.15468/dl.zfaw79
Anopheles gambiae	37.4551	5.51166	Unclassified	GBIF: https://doi.org/10.15468/dl.zfaw79
Anopheles gambiae	37.4551	5.51196	Unclassified	GBIF: https://doi.org/10.15468/dl.zfaw79
Anopheles gambiae	37.4552	5.51189	Unclassified	GBIF: https://doi.org/10.15468/dl.zfaw79

Anopheles gambiae	37.4553	5.51109	Unclassified	GBIF: https://doi.org/10.15468/dl.zfaw79
Anopheles gambiae	37.4554	5.51143	Unclassified	GBIF: https://doi.org/10.15468/dl.zfaw79
Anopheles gambiae	37.4554	5.51122	Unclassified	GBIF: https://doi.org/10.15468/dl.zfaw79
Anopheles gambiae	37.4555	5.51141	Unclassified	GBIF: https://doi.org/10.15468/dl.zfaw79
Anopheles gambiae	37.4555	5.51172	Unclassified	GBIF: https://doi.org/10.15468/dl.zfaw79
Anopheles gambiae	37.4555	5.51205	Unclassified	GBIF: https://doi.org/10.15468/dl.zfaw79
Anopheles gambiae	37.4556	5.51169	Unclassified	GBIF: https://doi.org/10.15468/dl.zfaw79
Anopheles gambiae	37.4556	5.5123	Unclassified	GBIF: https://doi.org/10.15468/dl.zfaw79
Anopheles gambiae	37.4556	5.51216	Unclassified	GBIF: https://doi.org/10.15468/dl.zfaw79
Anopheles gambiae	37.4557	5.51243	Unclassified	GBIF: https://doi.org/10.15468/dl.zfaw79
Anopheles gambiae	37.4558	5.51138	Unclassified	GBIF: https://doi.org/10.15468/dl.zfaw79
Anopheles gambiae	37.4558	5.51143	Unclassified	GBIF: https://doi.org/10.15468/dl.zfaw79
Anopheles gambiae	37.4559	5.51129	Unclassified	GBIF: https://doi.org/10.15468/dl.zfaw79
Anopheles gambiae	37.4559	5.51215	Unclassified	GBIF: https://doi.org/10.15468/dl.zfaw79
Anopheles gambiae	37.456	5.51251	Unclassified	GBIF: https://doi.org/10.15468/dl.zfaw79
Anopheles gambiae	37.4561	5.51232	Unclassified	GBIF: https://doi.org/10.15468/dl.zfaw79
Anopheles gambiae	37.4561	5.51134	Unclassified	GBIF: https://doi.org/10.15468/dl.zfaw79
Anopheles gambiae	37.4561	5.51112	Unclassified	GBIF: https://doi.org/10.15468/dl.zfaw79
Anopheles gambiae	37.4561	5.51145	Unclassified	GBIF: https://doi.org/10.15468/dl.zfaw79
Anopheles gambiae	37.4561	5.51205	Unclassified	GBIF: https://doi.org/10.15468/dl.zfaw79
Anopheles gambiae	37.4562	5.51246	Unclassified	GBIF: https://doi.org/10.15468/dl.zfaw79
Anopheles gambiae	37.4562	5.51175	Unclassified	GBIF: https://doi.org/10.15468/dl.zfaw79
Anopheles gambiae	37.4563	5.51231	Unclassified	GBIF: https://doi.org/10.15468/dl.zfaw79
Anopheles gambiae	37.4563	5.51148	Unclassified	GBIF: https://doi.org/10.15468/dl.zfaw79
Anopheles gambiae	37.4564	5.51222	Unclassified	GBIF: https://doi.org/10.15468/dl.zfaw79
Anopheles gambiae	37.4564	5.51152	Unclassified	GBIF: https://doi.org/10.15468/dl.zfaw79
Anopheles gambiae	37.4564	5.51198	Unclassified	GBIF: https://doi.org/10.15468/dl.zfaw79
Anopheles gambiae	37.4565	5.51169	Unclassified	GBIF: https://doi.org/10.15468/dl.zfaw79
Anopheles gambiae	37.4565	5.51175	Unclassified	GBIF: https://doi.org/10.15468/dl.zfaw79
Anopheles gambiae	37.4565	5.51191	Unclassified	GBIF: https://doi.org/10.15468/dl.zfaw79
Anopheles gambiae	37.4565	5.51217	Unclassified	GBIF: https://doi.org/10.15468/dl.zfaw79
Anopheles gambiae	37.4565	5.51205	Unclassified	GBIF: https://doi.org/10.15468/dl.zfaw79
Anopheles gambiae	37.4566	5.51149	Unclassified	GBIF: https://doi.org/10.15468/dl.zfaw79
Anopheles gambiae	37.4567	5.51185	Unclassified	GBIF: https://doi.org/10.15468/dl.zfaw79
Anopheles gambiae	37.4568	5.51113	Unclassified	GBIF: https://doi.org/10.15468/dl.zfaw79
Anopheles gambiae	37.4569	5.51201	Unclassified	GBIF: https://doi.org/10.15468/dl.zfaw79
Anopheles gambiae	37.457	5.5118	Unclassified	GBIF: https://doi.org/10.15468/dl.zfaw79
Anopheles gambiae	37.457	5.51137	Unclassified	GBIF: https://doi.org/10.15468/dl.zfaw79
Anopheles gambiae	37.4571	5.51179	Unclassified	GBIF: https://doi.org/10.15468/dl.zfaw79
Anopheles gambiae	37.4571	5.51201	Unclassified	GBIF: https://doi.org/10.15468/dl.zfaw79
Anopheles gambiae	37.4572	5.51141	Unclassified	GBIF: https://doi.org/10.15468/dl.zfaw79
Anopheles gambiae	37.4572	5.5115	Unclassified	GBIF: https://doi.org/10.15468/dl.zfaw79

Anopheles gambiae	37.4573	5.51203	Unclassified	GBIF: https://doi.org/10.15468/dl.zfaw79
Anopheles gambiae	37.4574	5.51176	Unclassified	GBIF: https://doi.org/10.15468/dl.zfaw79
Anopheles gambiae	37.4576	5.51112	Unclassified	GBIF: https://doi.org/10.15468/dl.zfaw79
Anopheles gambiae	37.4576	5.51188	Unclassified	GBIF: https://doi.org/10.15468/dl.zfaw79
Anopheles gambiae	37.4579	5.51158	Unclassified	GBIF: https://doi.org/10.15468/dl.zfaw79
Anopheles gambiae	37.4581	5.51188	Unclassified	GBIF: https://doi.org/10.15468/dl.zfaw79
Anopheles gambiae	37.4583	5.51134	Unclassified	GBIF: https://doi.org/10.15468/dl.zfaw79
Anopheles gambiae	37.4607	5.51123	Unclassified	GBIF: https://doi.org/10.15468/dl.zfaw79
Anopheles gambiae	37.53333	-3.3	Unclassified	GBIF: https://doi.org/10.15468/dl.zfaw79
Anopheles gambiae	37.6053	5.33426	Unclassified	GBIF: https://doi.org/10.15468/dl.zfaw79
Anopheles gambiae	37.6053	5.33419	Unclassified	GBIF: https://doi.org/10.15468/dl.zfaw79
Anopheles gambiae	37.6066	5.30055	Unclassified	GBIF: https://doi.org/10.15468/dl.zfaw79
Anopheles gambiae	37.6069	5.30185	Unclassified	GBIF: https://doi.org/10.15468/dl.zfaw79
Anopheles gambiae	37.6069	5.30044	Unclassified	GBIF: https://doi.org/10.15468/dl.zfaw79
Anopheles gambiae	37.607	5.30164	Unclassified	GBIF: https://doi.org/10.15468/dl.zfaw79
Anopheles gambiae	37.6098	5.3051	Unclassified	GBIF: https://doi.org/10.15468/dl.zfaw79
Anopheles gambiae	37.6102	5.30562	Unclassified	GBIF: https://doi.org/10.15468/dl.zfaw79
Anopheles gambiae	37.6113	5.30453	Unclassified	GBIF: https://doi.org/10.15468/dl.zfaw79
Anopheles gambiae	37.6114	5.30391	Unclassified	GBIF: https://doi.org/10.15468/dl.zfaw79
Anopheles gambiae	37.612	5.30552	Unclassified	GBIF: https://doi.org/10.15468/dl.zfaw79
Anopheles gambiae	37.6121	5.3139	Unclassified	GBIF: https://doi.org/10.15468/dl.zfaw79
Anopheles gambiae	37.6122	5.31304	Unclassified	GBIF: https://doi.org/10.15468/dl.zfaw79
Anopheles gambiae	37.66667	-3.38333	Unclassified	GBIF: https://doi.org/10.15468/dl.zfaw79
Anopheles gambiae	37.66667	-6.81667	Unclassified	GBIF: https://doi.org/10.15468/dl.zfaw79
Anopheles gambiae	38	-5	Unclassified	GBIF: https://doi.org/10.15468/dl.zfaw79
Anopheles gambiae	38.01667	-4.66667	Unclassified	GBIF: https://doi.org/10.15468/dl.zfaw79
Anopheles gambiae	38.03333	-5.41667	Unclassified	GBIF: https://doi.org/10.15468/dl.zfaw79
Anopheles gambiae	38.06667	-4.38333	Unclassified	GBIF: https://doi.org/10.15468/dl.zfaw79
Anopheles gambiae	38.16667	-15	Unclassified	GBIF: https://doi.org/10.15468/dl.zfaw79
Anopheles gambiae	38.28333	-4.88333	Unclassified	GBIF: https://doi.org/10.15468/dl.zfaw79
Anopheles gambiae	38.41667	-5.16667	Unclassified	GBIF: https://doi.org/10.15468/dl.zfaw79
Anopheles gambiae	38.55	-5.08333	Unclassified	GBIF: https://doi.org/10.15468/dl.zfaw79
Anopheles gambiae	38.55	-5.3	Unclassified	GBIF: https://doi.org/10.15468/dl.zfaw79
Anopheles gambiae	38.6	-5.15	Unclassified	GBIF: https://doi.org/10.15468/dl.zfaw79
Anopheles gambiae	38.76667	-5.16667	Unclassified	GBIF: https://doi.org/10.15468/dl.zfaw79
Anopheles gambiae	38.83333	-5.18333	Unclassified	GBIF: https://doi.org/10.15468/dl.zfaw79
Anopheles gambiae	38.91667	-6.43333	Unclassified	GBIF: https://doi.org/10.15468/dl.zfaw79
Anopheles gambiae	38.98333	-13.25	Unclassified	GBIF: https://doi.org/10.15468/dl.zfaw79
Anopheles gambiae	39.08333	-5.11667	Unclassified	GBIF: https://doi.org/10.15468/dl.zfaw79
Anopheles gambiae	39.15	-4.86667	Unclassified	GBIF: https://doi.org/10.15468/dl.zfaw79
Anopheles gambiae	39.16667	-4.83333	Unclassified	GBIF: https://doi.org/10.15468/dl.zfaw79
Anopheles gambiae	39.2	-6.16667	Unclassified	GBIF: https://doi.org/10.15468/dl.zfaw79

Anopheles gambiae	39.21667	-4.6	Unclassified	GBIF: https://doi.org/10.15468/dl.zfaw79
Anopheles gambiae	39.23333	-4.6	Unclassified	GBIF: https://doi.org/10.15468/dl.zfaw79
Anopheles gambiae	39.25	-15.96667	Unclassified	GBIF: https://doi.org/10.15468/dl.zfaw79
Anopheles gambiae	39.25	-6.75	Unclassified	GBIF: https://doi.org/10.15468/dl.zfaw79
Anopheles gambiae	39.3	-6.85	Unclassified	GBIF: https://doi.org/10.15468/dl.zfaw79
Anopheles gambiae	39.47	-4.17	Unclassified	GBIF: https://doi.org/10.15468/dl.zfaw79
Anopheles gambiae	39.5	-11.5	Unclassified	GBIF: https://doi.org/10.15468/dl.zfaw79
Anopheles gambiae	39.51667	-4.06667	Unclassified	GBIF: https://doi.org/10.15468/dl.zfaw79
Anopheles gambiae	39.63333	-1.25	Unclassified	GBIF: https://doi.org/10.15468/dl.zfaw79
Anopheles gambiae	39.75	-13.75	Unclassified	GBIF: https://doi.org/10.15468/dl.zfaw79
Anopheles gambiae	39.75	-5	Unclassified	GBIF: https://doi.org/10.15468/dl.zfaw79
Anopheles gambiae	39.83333	-3.61667	Unclassified	GBIF: https://doi.org/10.15468/dl.zfaw79
Anopheles gambiae	39.91	-3.89	Unclassified	GBIF: https://doi.org/10.15468/dl.zfaw79
Anopheles gambiae	39.96667	-3.25	Unclassified	GBIF: https://doi.org/10.15468/dl.zfaw79
Anopheles gambiae	40	-3.1	Unclassified	GBIF: https://doi.org/10.15468/dl.zfaw79
Anopheles gambiae	40.13333	-12	Unclassified	GBIF: https://doi.org/10.15468/dl.zfaw79
Anopheles gambiae	40.25	-11.25	Unclassified	GBIF: https://doi.org/10.15468/dl.zfaw79
Anopheles gambiae	40.28333	-14.21667	Unclassified	GBIF: https://doi.org/10.15468/dl.zfaw79
Anopheles gambiae	40.5	-10.75	Unclassified	GBIF: https://doi.org/10.15468/dl.zfaw79
Anopheles gambiae	40.5	-13.13333	Unclassified	GBIF: https://doi.org/10.15468/dl.zfaw79
Anopheles gambiae	40.83955	-20.74312	Unclassified	GBIF: https://doi.org/10.15468/dl.zfaw79
Anopheles gambiae	43.26667	-11.66667	Unclassified	GBIF: https://doi.org/10.15468/dl.zfaw79
Anopheles gambiae	43.66667	-12.3	Unclassified	GBIF: https://doi.org/10.15468/dl.zfaw79
Anopheles gambiae	43.68333	-23.33333	Unclassified	GBIF: https://doi.org/10.15468/dl.zfaw79
Anopheles gambiae	43.75	-21.73333	Unclassified	GBIF: https://doi.org/10.15468/dl.zfaw79
Anopheles gambiae	44.5	-12.2	Unclassified	GBIF: https://doi.org/10.15468/dl.zfaw79
Anopheles gambiae	44.78333	-19.11667	Unclassified	GBIF: https://doi.org/10.15468/dl.zfaw79
Anopheles gambiae	44.9	-17.81667	Unclassified	GBIF: https://doi.org/10.15468/dl.zfaw79
Anopheles gambiae	45.14743	-12.8206	Unclassified	GBIF: https://doi.org/10.15468/dl.zfaw79
Anopheles gambiae	45.45	-22.53333	Unclassified	GBIF: https://doi.org/10.15468/dl.zfaw79
Anopheles gambiae	45.48333	-19.51667	Unclassified	GBIF: https://doi.org/10.15468/dl.zfaw79
Anopheles gambiae	45.65	-17.66667	Unclassified	GBIF: https://doi.org/10.15468/dl.zfaw79
Anopheles gambiae	46.66667	-16.08333	Unclassified	GBIF: https://doi.org/10.15468/dl.zfaw79
Anopheles gambiae	46.71667	-16.43333	Unclassified	GBIF: https://doi.org/10.15468/dl.zfaw79
Anopheles gambiae	46.83333	-16.95	Unclassified	GBIF: https://doi.org/10.15468/dl.zfaw79
Anopheles gambiae	46.86667	-24.81667	Unclassified	GBIF: https://doi.org/10.15468/dl.zfaw79
Anopheles gambiae	46.86667	-24.85	Unclassified	GBIF: https://doi.org/10.15468/dl.zfaw79
Anopheles gambiae	46.86667	-24.88333	Unclassified	GBIF: https://doi.org/10.15468/dl.zfaw79
Anopheles gambiae	46.91667	-17.55	Unclassified	GBIF: https://doi.org/10.15468/dl.zfaw79
Anopheles gambiae	46.95	-17.3	Unclassified	GBIF: https://doi.org/10.15468/dl.zfaw79
Anopheles gambiae	46.966667	-16.133333	Unclassified	GBIF: https://doi.org/10.15468/dl.zfaw79
Anopheles gambiae	47	-24.81667	Unclassified	GBIF: https://doi.org/10.15468/dl.zfaw79

Anopheles gambiae	47.01667	-23.58333	Unclassified	GBIF: https://doi.org/10.15468/dl.zfaw79
Anopheles gambiae	47.05	-16.33333	Unclassified	GBIF: https://doi.org/10.15468/dl.zfaw79
Anopheles gambiae	47.26667	-23.41667	Unclassified	GBIF: https://doi.org/10.15468/dl.zfaw79
Anopheles gambiae	47.33333	-22.83333	Unclassified	GBIF: https://doi.org/10.15468/dl.zfaw79
Anopheles gambiae	47.35	-16.33333	Unclassified	GBIF: https://doi.org/10.15468/dl.zfaw79
Anopheles gambiae	47.43333	-21.55	Unclassified	GBIF: https://doi.org/10.15468/dl.zfaw79
Anopheles gambiae	47.45	-21.86667	Unclassified	GBIF: https://doi.org/10.15468/dl.zfaw79
Anopheles gambiae	47.46667	-22.8	Unclassified	GBIF: https://doi.org/10.15468/dl.zfaw79
Anopheles gambiae	47.58333	-18.95	Unclassified	GBIF: https://doi.org/10.15468/dl.zfaw79
Anopheles gambiae	47.58333	-23.35	Unclassified	GBIF: https://doi.org/10.15468/dl.zfaw79
Anopheles gambiae	47.65	-21.31667	Unclassified	GBIF: https://doi.org/10.15468/dl.zfaw79
Anopheles gambiae	47.65	-16.05	Unclassified	GBIF: https://doi.org/10.15468/dl.zfaw79
Anopheles gambiae	47.66667	-15.51667	Unclassified	GBIF: https://doi.org/10.15468/dl.zfaw79
Anopheles gambiae	47.68333	-18.96667	Unclassified	GBIF: https://doi.org/10.15468/dl.zfaw79
Anopheles gambiae	47.83333	-14.63333	Unclassified	GBIF: https://doi.org/10.15468/dl.zfaw79
Anopheles gambiae	47.83333	-22.83333	Unclassified	GBIF: https://doi.org/10.15468/dl.zfaw79
Anopheles gambiae	47.85	-21.81667	Unclassified	GBIF: https://doi.org/10.15468/dl.zfaw79
Anopheles gambiae	47.85	-22.35	Unclassified	GBIF: https://doi.org/10.15468/dl.zfaw79
Anopheles gambiae	47.86667	-21.96667	Unclassified	GBIF: https://doi.org/10.15468/dl.zfaw79
Anopheles gambiae	47.86667	-22	Unclassified	GBIF: https://doi.org/10.15468/dl.zfaw79
Anopheles gambiae	47.86667	-18.18333	Unclassified	GBIF: https://doi.org/10.15468/dl.zfaw79
Anopheles gambiae	48	-22.15	Unclassified	GBIF: https://doi.org/10.15468/dl.zfaw79
Anopheles gambiae	48	-14.83333	Unclassified	GBIF: https://doi.org/10.15468/dl.zfaw79
Anopheles gambiae	48	-19.15	Unclassified	GBIF: https://doi.org/10.15468/dl.zfaw79
Anopheles gambiae	48.01667	-19.15	Unclassified	GBIF: https://doi.org/10.15468/dl.zfaw79
Anopheles gambiae	48.05	-19.13333	Unclassified	GBIF: https://doi.org/10.15468/dl.zfaw79
Anopheles gambiae	48.1	-19.08333	Unclassified	GBIF: https://doi.org/10.15468/dl.zfaw79
Anopheles gambiae	48.1	-14.16667	Unclassified	GBIF: https://doi.org/10.15468/dl.zfaw79
Anopheles gambiae	48.1	-19.03333	Unclassified	GBIF: https://doi.org/10.15468/dl.zfaw79
Anopheles gambiae	48.11667	-19.01667	Unclassified	GBIF: https://doi.org/10.15468/dl.zfaw79
Anopheles gambiae	48.1667	-19.3667	Unclassified	GBIF: https://doi.org/10.15468/dl.zfaw79
Anopheles gambiae	48.18333	-19.4	Unclassified	GBIF: https://doi.org/10.15468/dl.zfaw79
Anopheles gambiae	48.2	-19.06667	Unclassified	GBIF: https://doi.org/10.15468/dl.zfaw79
Anopheles gambiae	48.2	-19.36667	Unclassified	GBIF: https://doi.org/10.15468/dl.zfaw79
Anopheles gambiae	48.21667	-13.35	Unclassified	GBIF: https://doi.org/10.15468/dl.zfaw79
Anopheles gambiae	48.33333	-21.21667	Unclassified	GBIF: https://doi.org/10.15468/dl.zfaw79
Anopheles gambiae	48.33333	-19	Unclassified	GBIF: https://doi.org/10.15468/dl.zfaw79
Anopheles gambiae	48.45	-13.66667	Unclassified	GBIF: https://doi.org/10.15468/dl.zfaw79
Anopheles gambiae	48.46667	-18.9	Unclassified	GBIF: https://doi.org/10.15468/dl.zfaw79
Anopheles gambiae	48.46667	-17.81667	Unclassified	GBIF: https://doi.org/10.15468/dl.zfaw79
Anopheles gambiae	48.51667	-20.6	Unclassified	GBIF: https://doi.org/10.15468/dl.zfaw79
Anopheles gambiae	48.53333	-18.91667	Unclassified	GBIF: https://doi.org/10.15468/dl.zfaw79

Anopheles gambiae	48.6	-18.98333	Unclassified	GBIF: https://doi.org/10.15468/dl.zfaw79
Anopheles gambiae	48.63333	-14.05	Unclassified	GBIF: https://doi.org/10.15468/dl.zfaw79
Anopheles gambiae	48.75	-18.96667	Unclassified	GBIF: https://doi.org/10.15468/dl.zfaw79
Anopheles gambiae	48.83333	-18.91667	Unclassified	GBIF: https://doi.org/10.15468/dl.zfaw79
Anopheles gambiae	48.85	-18.86667	Unclassified	GBIF: https://doi.org/10.15468/dl.zfaw79
Anopheles gambiae	48.85	-18.88333	Unclassified	GBIF: https://doi.org/10.15468/dl.zfaw79
Anopheles gambiae	48.85	-18.9	Unclassified	GBIF: https://doi.org/10.15468/dl.zfaw79
Anopheles gambiae	48.85	-18.91667	Unclassified	GBIF: https://doi.org/10.15468/dl.zfaw79
Anopheles gambiae	48.95	-18.98333	Unclassified	GBIF: https://doi.org/10.15468/dl.zfaw79
Anopheles gambiae	49	-18.83333	Unclassified	GBIF: https://doi.org/10.15468/dl.zfaw79
Anopheles gambiae	49.05	-13.16667	Unclassified	GBIF: https://doi.org/10.15468/dl.zfaw79
Anopheles gambiae	49.18333	-17.41667	Unclassified	GBIF: https://doi.org/10.15468/dl.zfaw79
Anopheles gambiae	49.2	-12.7	Unclassified	GBIF: https://doi.org/10.15468/dl.zfaw79
Anopheles gambiae	49.28333	-12.31667	Unclassified	GBIF: https://doi.org/10.15468/dl.zfaw79
Anopheles gambiae	49.31667	-18.05	Unclassified	GBIF: https://doi.org/10.15468/dl.zfaw79
Anopheles gambiae	49.38333	-18.16667	Unclassified	GBIF: https://doi.org/10.15468/dl.zfaw79
Anopheles gambiae	49.41667	-17.35	Unclassified	GBIF: https://doi.org/10.15468/dl.zfaw79
Anopheles gambiae	49.42	-17.81	Unclassified	GBIF: https://doi.org/10.15468/dl.zfaw79
Anopheles gambiae	49.58333	-16.88333	Unclassified	GBIF: https://doi.org/10.15468/dl.zfaw79
Anopheles gambiae	49.66667	-14.65	Unclassified	GBIF: https://doi.org/10.15468/dl.zfaw79
Anopheles gambiae	49.76667	-16.16667	Unclassified	GBIF: https://doi.org/10.15468/dl.zfaw79
Anopheles gambiae	49.95	-16.91667	Unclassified	GBIF: https://doi.org/10.15468/dl.zfaw79
Anopheles gambiae	50.26667	-14.88333	Unclassified	GBIF: https://doi.org/10.15468/dl.zfaw79
Anopheles gambiae	50.280691	-14.902474	Unclassified	GBIF: https://doi.org/10.15468/dl.zfaw79
Anopheles gambiae	57.5	-20.25	Unclassified	GBIF: https://doi.org/10.15468/dl.zfaw79
Anopheles georgianus	-83.559883	30.784919	Unclassified	GBIF: https://doi.org/10.15468/dl.zfaw79
Anopheles georgianus	-82.324	27.4746	Unclassified	GBIF: https://doi.org/10.15468/dl.zfaw79
Anopheles georgianus	-81.64	33.55	Unclassified	GBIF: https://doi.org/10.15468/dl.zfaw79
Anopheles gigas	98.492213	18.071814	Unclassified	GBIF: https://doi.org/10.15468/dl.zfaw79
Anopheles gigas	98.795965	8.284143	Unclassified	GBIF: https://doi.org/10.15468/dl.zfaw79
Anopheles gigas	101.104708	13.235179	Unclassified	GBIF: https://doi.org/10.15468/dl.zfaw79
Anopheles gigas	101.501371	13.239733	Unclassified	GBIF: https://doi.org/10.15468/dl.zfaw79
Anopheles gigas	120.615559	16.396666	Unclassified	GBIF: https://doi.org/10.15468/dl.zfaw79
Anopheles gilesi	-44.566668	-17.900001	Unclassified	GBIF: https://doi.org/10.15468/dl.zfaw79
Anopheles goeldii	-64.74	-7.35	Unclassified	GBIF: https://doi.org/10.15468/dl.zfaw79
Anopheles goeldii	-63.61	1.82	Unclassified	GBIF: https://doi.org/10.15468/dl.zfaw79
Anopheles goeldii	-62.26	-9.22	Unclassified	GBIF: https://doi.org/10.15468/dl.zfaw79
Anopheles goeldii	-60.31	-1.54	Unclassified	GBIF: https://doi.org/10.15468/dl.zfaw79
Anopheles goeldii	-60.27	-1.52	Unclassified	GBIF: https://doi.org/10.15468/dl.zfaw79
Anopheles goeldii	-60.24	-1.49	Unclassified	GBIF: https://doi.org/10.15468/dl.zfaw79
Anopheles goeldii	-58.39	-3.15	Unclassified	GBIF: https://doi.org/10.15468/dl.zfaw79
Anopheles goeldii	-55.02	-2.75	Unclassified	GBIF: https://doi.org/10.15468/dl.zfaw79

Anopheles goeldii	-53.59	-2.08	Unclassified	GBIF: https://doi.org/10.15468/dl.zfaw79
Anopheles grabhamii	-81.40359	19.37796	Unclassified	GBIF: https://doi.org/10.15468/dl.zfaw79
Anopheles grabhamii	-81.39974	19.36442	Unclassified	GBIF: https://doi.org/10.15468/dl.zfaw79
Anopheles grabhamii	-81.32536	24.69459	Unclassified	GBIF: https://doi.org/10.15468/dl.zfaw79
Anopheles grabhamii	-80.96	24.76	Unclassified	GBIF: https://doi.org/10.15468/dl.zfaw79
Anopheles grabhamii	-78.00354	18.11689	Unclassified	GBIF: https://doi.org/10.15468/dl.zfaw79
Anopheles grabhamii	-76.91266	18.03765	Unclassified	GBIF: https://doi.org/10.15468/dl.zfaw79
Anopheles grabhamii	-76.90773	18.01782	Unclassified	GBIF: https://doi.org/10.15468/dl.zfaw79
Anopheles grabhamii	-76.81	18.018056	Unclassified	GBIF: https://doi.org/10.15468/dl.zfaw79
Anopheles grabhamii	-76.2905	17.94611	Unclassified	GBIF: https://doi.org/10.15468/dl.zfaw79
Anopheles grabhamii	-76.27154	17.93448	Unclassified	GBIF: https://doi.org/10.15468/dl.zfaw79
Anopheles grabhamii	-70.18082	18.50346	Unclassified	GBIF: https://doi.org/10.15468/dl.zfaw79
Anopheles grabhamii	-70.14472	18.48652	Unclassified	GBIF: https://doi.org/10.15468/dl.zfaw79
Anopheles grabhamii	-70.14377	18.48652	Unclassified	GBIF: https://doi.org/10.15468/dl.zfaw79
Anopheles grabhamii	-70.14376	18.48472	Unclassified	GBIF: https://doi.org/10.15468/dl.zfaw79
Anopheles grabhamii	-70.12085	18.45774	Unclassified	GBIF: https://doi.org/10.15468/dl.zfaw79
Anopheles grabhamii	-70.11145	18.46864	Unclassified	GBIF: https://doi.org/10.15468/dl.zfaw79
Anopheles grabhamii	-69.99777	18.46204	Unclassified	GBIF: https://doi.org/10.15468/dl.zfaw79
Anopheles grabhamii	-69.96575	18.49474	Unclassified	GBIF: https://doi.org/10.15468/dl.zfaw79
Anopheles grabhamii	-69.94594	18.5093	Unclassified	GBIF: https://doi.org/10.15468/dl.zfaw79
Anopheles grabhamii	-69.84729	18.48266	Unclassified	GBIF: https://doi.org/10.15468/dl.zfaw79
Anopheles grabhamii	-69.34451	19.03464	Unclassified	GBIF: https://doi.org/10.15468/dl.zfaw79
Anopheles grabhamii	-69.34391	19.20545	Unclassified	GBIF: https://doi.org/10.15468/dl.zfaw79
Anopheles grabhamii	-67.16617	18.234	Unclassified	GBIF: https://doi.org/10.15468/dl.zfaw79
Anopheles grabhamii	-67.070481	18.036123	Unclassified	GBIF: https://doi.org/10.15468/dl.zfaw79
Anopheles grabhamii	-66.872022	17.982274	Unclassified	GBIF: https://doi.org/10.15468/dl.zfaw79
Anopheles grabhamii	-66.872	17.982	Unclassified	GBIF: https://doi.org/10.15468/dl.zfaw79
Anopheles grabhamii	-66.868	17.976	Unclassified	GBIF: https://doi.org/10.15468/dl.zfaw79
Anopheles grabhamii	-66.867896	17.975642	Unclassified	GBIF: https://doi.org/10.15468/dl.zfaw79
Anopheles grabhamii	-66.841744	17.962708	Unclassified	GBIF: https://doi.org/10.15468/dl.zfaw79
Anopheles grabhamii	-66.835854	17.953607	Unclassified	GBIF: https://doi.org/10.15468/dl.zfaw79
Anopheles grabhamii	-66.829438	17.970605	Unclassified	GBIF: https://doi.org/10.15468/dl.zfaw79
Anopheles grabhamii	-66.71616	18.46732	Unclassified	GBIF: https://doi.org/10.15468/dl.zfaw79
Anopheles grabhamii	-66.5	18.25	Unclassified	GBIF: https://doi.org/10.15468/dl.zfaw79
Anopheles grabhamii	-66.3875	18.446111	Unclassified	GBIF: https://doi.org/10.15468/dl.zfaw79
Anopheles grabhamii	-66.16615	18.46732	Unclassified	GBIF: https://doi.org/10.15468/dl.zfaw79
Anopheles grabhamii	-66.11615	18.45066	Unclassified	GBIF: https://doi.org/10.15468/dl.zfaw79
Anopheles grabhamii	-66.08281	18.43399	Unclassified	GBIF: https://doi.org/10.15468/dl.zfaw79
Anopheles grabhamii	-66.04948	18.41732	Unclassified	GBIF: https://doi.org/10.15468/dl.zfaw79
Anopheles grabhamii	-65.99948	18.45066	Unclassified	GBIF: https://doi.org/10.15468/dl.zfaw79
Anopheles grabhamii	-65.96615	18.26733	Unclassified	GBIF: https://doi.org/10.15468/dl.zfaw79
Anopheles grabhamii	-65.819719	18.321047	Unclassified	GBIF: https://doi.org/10.15468/dl.zfaw79

Anopheles grabhamii	-65.284224	18.299756	Unclassified	GBIF: https://doi.org/10.15468/dl.zfaw79
Anopheles grabhamii	-64.894167	18.341111	Unclassified	GBIF: https://doi.org/10.15468/dl.zfaw79
Anopheles grabhamii	-64.87859	18.32073	Unclassified	GBIF: https://doi.org/10.15468/dl.zfaw79
Anopheles grabhamii	-64.75036	18.36261	Unclassified	GBIF: https://doi.org/10.15468/dl.zfaw79
Anopheles grabhamii	81.3873	24.7172	Unclassified	GBIF: https://doi.org/10.15468/dl.zfaw79
Anopheles greeni	117.87	4.16	Unclassified	GBIF: https://doi.org/10.15468/dl.zfaw79
Anopheles guarani	-54.59	-25.48	Unclassified	GBIF: https://doi.org/10.15468/dl.zfaw79
Anopheles guarani	-54.58667	-25.48056	Unclassified	GBIF: https://doi.org/10.15468/dl.zfaw79
Anopheles guarao	-63.101946	10.117777	Unclassified	GBIF: https://doi.org/10.15468/dl.zfaw79
Anopheles hackeri	97.996656	19.01668	Unclassified	GBIF: https://doi.org/10.15468/dl.zfaw79
Anopheles hackeri	98.844326	16.04819	Unclassified	GBIF: https://doi.org/10.15468/dl.zfaw79
Anopheles hackeri	101.234592	14.56598	Unclassified	GBIF: https://doi.org/10.15468/dl.zfaw79
Anopheles hackeri	101.36853	6.2595	Unclassified	GBIF: https://doi.org/10.15468/dl.zfaw79
Anopheles hackeri	101.77356	5.94562	Unclassified	GBIF: https://doi.org/10.15468/dl.zfaw79
Anopheles hackeri	101.80659	5.94563	Unclassified	GBIF: https://doi.org/10.15468/dl.zfaw79
Anopheles hackeri	104.22271	14.615236	Unclassified	GBIF: https://doi.org/10.15468/dl.zfaw79
Anopheles hackeri	116.8333	-1.2833	Unclassified	GBIF: https://doi.org/10.15468/dl.zfaw79
Anopheles hancocki	-10.353433	6.240148	Unclassified	GBIF: https://doi.org/10.15468/dl.zfaw79
Anopheles hancocki	3.01587	6.90045	Unclassified	GBIF: https://doi.org/10.15468/dl.zfaw79
Anopheles hancocki	4.48256	7.7837	Unclassified	GBIF: https://doi.org/10.15468/dl.zfaw79
Anopheles hancocki	4.58256	8.48364	Unclassified	GBIF: https://doi.org/10.15468/dl.zfaw79
Anopheles hancocki	6.73259	7.80037	Unclassified	GBIF: https://doi.org/10.15468/dl.zfaw79
Anopheles hancocki	6.7826	6.16719	Unclassified	GBIF: https://doi.org/10.15468/dl.zfaw79
Anopheles hancocki	7.29927	6.21719	Unclassified	GBIF: https://doi.org/10.15468/dl.zfaw79
Anopheles hancocki	7.36594	6.96712	Unclassified	GBIF: https://doi.org/10.15468/dl.zfaw79
Anopheles hancocki	7.44927	10.50013	Unclassified	GBIF: https://doi.org/10.15468/dl.zfaw79
Anopheles hancocki	7.4826	10.63345	Unclassified	GBIF: https://doi.org/10.15468/dl.zfaw79
Anopheles hancocki	7.54928	6.4505	Unclassified	GBIF: https://doi.org/10.15468/dl.zfaw79
Anopheles hancocki	8.34929	4.98397	Unclassified	GBIF: https://doi.org/10.15468/dl.zfaw79
Anopheles hancocki	9.23264	4.15071	Unclassified	GBIF: https://doi.org/10.15468/dl.zfaw79
Anopheles hancocki	9.34931	4.10072	Unclassified	GBIF: https://doi.org/10.15468/dl.zfaw79
Anopheles hancocki	10.0833	5.6	Unclassified	GBIF: https://doi.org/10.15468/dl.zfaw79
Anopheles hancocki	11.519852	3.898465	Unclassified	GBIF: https://doi.org/10.15468/dl.zfaw79
Anopheles hancocki	12.96	-1.76	Unclassified	GBIF: https://doi.org/10.15468/dl.zfaw79
Anopheles hancocki	13	-1.82	Unclassified	GBIF: https://doi.org/10.15468/dl.zfaw79
Anopheles hancocki	13.49	-1.63	Unclassified	GBIF: https://doi.org/10.15468/dl.zfaw79
Anopheles hancocki	13.58	-1.62	Unclassified	GBIF: https://doi.org/10.15468/dl.zfaw79
Anopheles hargreavesi	3.01587	6.90045	Unclassified	GBIF: https://doi.org/10.15468/dl.zfaw79
Anopheles hargreavesi	3.28254	6.46716	Unclassified	GBIF: https://doi.org/10.15468/dl.zfaw79
Anopheles hargreavesi	3.33254	7.18376	Unclassified	GBIF: https://doi.org/10.15468/dl.zfaw79
Anopheles hargreavesi	3.39921	6.45049	Unclassified	GBIF: https://doi.org/10.15468/dl.zfaw79
Anopheles hargreavesi	3.89922	7.43373	Unclassified	GBIF: https://doi.org/10.15468/dl.zfaw79

Anopheles hargreavesi	3.93255	6.81712	Unclassified	GBIF: https://doi.org/10.15468/dl.zfaw79
Anopheles hargreavesi	4.23255	8.13367	Unclassified	GBIF: https://doi.org/10.15468/dl.zfaw79
Anopheles hargreavesi	4.48256	7.7837	Unclassified	GBIF: https://doi.org/10.15468/dl.zfaw79
Anopheles hargreavesi	4.81589	9.13358	Unclassified	GBIF: https://doi.org/10.15468/dl.zfaw79
Anopheles hargreavesi	5.69925	5.90055	Unclassified	GBIF: https://doi.org/10.15468/dl.zfaw79
Anopheles hargreavesi	5.73258	5.51725	Unclassified	GBIF: https://doi.org/10.15468/dl.zfaw79
Anopheles hargreavesi	6.01591	9.10025	Unclassified	GBIF: https://doi.org/10.15468/dl.zfaw79
Anopheles hargreavesi	6.11591	9.08358	Unclassified	GBIF: https://doi.org/10.15468/dl.zfaw79
Anopheles hargreavesi	6.14925	6.75047	Unclassified	GBIF: https://doi.org/10.15468/dl.zfaw79
Anopheles hargreavesi	6.36593	4.684	Unclassified	GBIF: https://doi.org/10.15468/dl.zfaw79
Anopheles hargreavesi	7.01594	5.50059	Unclassified	GBIF: https://doi.org/10.15468/dl.zfaw79
Anopheles hargreavesi	7.01594	4.85065	Unclassified	GBIF: https://doi.org/10.15468/dl.zfaw79
Anopheles hargreavesi	7.54928	6.4505	Unclassified	GBIF: https://doi.org/10.15468/dl.zfaw79
Anopheles hargreavesi	8.34929	4.98397	Unclassified	GBIF: https://doi.org/10.15468/dl.zfaw79
Anopheles hargreavesi	9.23264	4.15071	Unclassified	GBIF: https://doi.org/10.15468/dl.zfaw79
Anopheles hargreavesi	9.28264	5.71724	Unclassified	GBIF: https://doi.org/10.15468/dl.zfaw79
Anopheles hargreavesi	9.34931	4.10072	Unclassified	GBIF: https://doi.org/10.15468/dl.zfaw79
Anopheles hargreavesi	10.09932	6.0672	Unclassified	GBIF: https://doi.org/10.15468/dl.zfaw79
Anopheles harperi	34.999996	1.016667	Unclassified	GBIF: https://doi.org/10.15468/dl.zfaw79
Anopheles harrisoni	96.27	22.2	Unclassified	GBIF: https://doi.org/10.15468/dl.zfaw79
Anopheles harrisoni	98.55	14.85	Unclassified	GBIF: https://doi.org/10.15468/dl.zfaw79
Anopheles harrisoni	98.988056	14.335333	Unclassified	GBIF: https://doi.org/10.15468/dl.zfaw79
Anopheles harrisoni	98.988139	14.335778	Unclassified	GBIF: https://doi.org/10.15468/dl.zfaw79
Anopheles harrisoni	98.988333	14.335556	Unclassified	GBIF: https://doi.org/10.15468/dl.zfaw79
Anopheles harrisoni	98.988389	14.33525	Unclassified	GBIF: https://doi.org/10.15468/dl.zfaw79
Anopheles harrisoni	98.988528	14.335889	Unclassified	GBIF: https://doi.org/10.15468/dl.zfaw79
Anopheles harrisoni	98.988667	14.335583	Unclassified	GBIF: https://doi.org/10.15468/dl.zfaw79
Anopheles harrisoni	99.017	14.283	Unclassified	GBIF: https://doi.org/10.15468/dl.zfaw79
Anopheles harrisoni	99.04167	14.6125	Unclassified	GBIF: https://doi.org/10.15468/dl.zfaw79
Anopheles harrisoni	99.1075	14.7075	Unclassified	GBIF: https://doi.org/10.15468/dl.zfaw79
Anopheles harrisoni	99.5	22.3	Unclassified	GBIF: https://doi.org/10.15468/dl.zfaw79
Anopheles harrisoni	99.78333	13.9	Unclassified	GBIF: https://doi.org/10.15468/dl.zfaw79
Anopheles harrisoni	100.8	24.4	Unclassified	GBIF: https://doi.org/10.15468/dl.zfaw79
Anopheles harrisoni	100.9	22.7	Unclassified	GBIF: https://doi.org/10.15468/dl.zfaw79
Anopheles harrisoni	101.2	21.9	Unclassified	GBIF: https://doi.org/10.15468/dl.zfaw79
Anopheles harrisoni	101.58333	21.38333	Unclassified	GBIF: https://doi.org/10.15468/dl.zfaw79
Anopheles harrisoni	102	23.5	Unclassified	GBIF: https://doi.org/10.15468/dl.zfaw79
Anopheles harrisoni	103.9	27.7	Unclassified	GBIF: https://doi.org/10.15468/dl.zfaw79
Anopheles harrisoni	104	29.2	Unclassified	GBIF: https://doi.org/10.15468/dl.zfaw79
Anopheles harrisoni	104.03333	20.88333	Unclassified	GBIF: https://doi.org/10.15468/dl.zfaw79
Anopheles harrisoni	104.05	20.91667	Unclassified	GBIF: https://doi.org/10.15468/dl.zfaw79
Anopheles harrisoni	104.08333	21.06667	Unclassified	GBIF: https://doi.org/10.15468/dl.zfaw79

Anopheles harrisoni	104.08425	22.50231	Unclassified	GBIF: https://doi.org/10.15468/dl.zfaw79
Anopheles harrisoni	104.12556	21.14953	Unclassified	GBIF: https://doi.org/10.15468/dl.zfaw79
Anopheles harrisoni	104.2	28.1	Unclassified	GBIF: https://doi.org/10.15468/dl.zfaw79
Anopheles harrisoni	104.45717	22.64597	Unclassified	GBIF: https://doi.org/10.15468/dl.zfaw79
Anopheles harrisoni	104.60181	20.83478	Unclassified	GBIF: https://doi.org/10.15468/dl.zfaw79
Anopheles harrisoni	104.7	18.86667	Unclassified	GBIF: https://doi.org/10.15468/dl.zfaw79
Anopheles harrisoni	105.05	22.81667	Unclassified	GBIF: https://doi.org/10.15468/dl.zfaw79
Anopheles harrisoni	105.1667	20.6333	Unclassified	GBIF: https://doi.org/10.15468/dl.zfaw79
Anopheles harrisoni	105.18333	20.5	Unclassified	GBIF: https://doi.org/10.15468/dl.zfaw79
Anopheles harrisoni	105.26667	20.61667	Unclassified	GBIF: https://doi.org/10.15468/dl.zfaw79
Anopheles harrisoni	105.46667	18.38333	Unclassified	GBIF: https://doi.org/10.15468/dl.zfaw79
Anopheles harrisoni	105.48644	19.48	Unclassified	GBIF: https://doi.org/10.15468/dl.zfaw79
Anopheles harrisoni	105.6	20.23333	Unclassified	GBIF: https://doi.org/10.15468/dl.zfaw79
Anopheles harrisoni	105.6	25.95	Unclassified	GBIF: https://doi.org/10.15468/dl.zfaw79
Anopheles harrisoni	105.8	24.6	Unclassified	GBIF: https://doi.org/10.15468/dl.zfaw79
Anopheles harrisoni	106.1	25.2	Unclassified	GBIF: https://doi.org/10.15468/dl.zfaw79
Anopheles harrisoni	106.42211	22.34225	Unclassified	GBIF: https://doi.org/10.15468/dl.zfaw79
Anopheles harrisoni	106.46667	22.25	Unclassified	GBIF: https://doi.org/10.15468/dl.zfaw79
Anopheles harrisoni	106.5	24.36667	Unclassified	GBIF: https://doi.org/10.15468/dl.zfaw79
Anopheles harrisoni	106.55	22.51667	Unclassified	GBIF: https://doi.org/10.15468/dl.zfaw79
Anopheles harrisoni	106.56667	22.48333	Unclassified	GBIF: https://doi.org/10.15468/dl.zfaw79
Anopheles harrisoni	107.16175	21.32022	Unclassified	GBIF: https://doi.org/10.15468/dl.zfaw79
Anopheles harrisoni	107.2285	21.42167	Unclassified	GBIF: https://doi.org/10.15468/dl.zfaw79
Anopheles harrisoni	107.29928	21.3775	Unclassified	GBIF: https://doi.org/10.15468/dl.zfaw79
Anopheles harrisoni	110	25.8	Unclassified	GBIF: https://doi.org/10.15468/dl.zfaw79
Anopheles hectoris	-92.844167	16.710833	Unclassified	GBIF: https://doi.org/10.15468/dl.zfaw79
Anopheles hectoris	-92.826944	16.713333	Unclassified	GBIF: https://doi.org/10.15468/dl.zfaw79
Anopheles hectoris	-92.723639	16.765278	Unclassified	GBIF: https://doi.org/10.15468/dl.zfaw79
Anopheles hectoris	-92.723028	16.765861	Unclassified	GBIF: https://doi.org/10.15468/dl.zfaw79
Anopheles hectoris	-92.680833	16.76	Unclassified	GBIF: https://doi.org/10.15468/dl.zfaw79
Anopheles hectoris	-92.680805	16.76	Unclassified	GBIF: https://doi.org/10.15468/dl.zfaw79
Anopheles hectoris	-92.638333	16.736667	Unclassified	GBIF: https://doi.org/10.15468/dl.zfaw79
Anopheles hectoris	-92.6335	16.7508	Unclassified	GBIF: https://doi.org/10.15468/dl.zfaw79
Anopheles hectoris	-92.63306	16.738	Unclassified	GBIF: https://doi.org/10.15468/dl.zfaw79
Anopheles hectoris	-92.617778	16.709194	Unclassified	GBIF: https://doi.org/10.15468/dl.zfaw79
Anopheles hectoris	-92.509667	16.583333	Unclassified	GBIF: https://doi.org/10.15468/dl.zfaw79
Anopheles hectoris	-92.48625	16.559417	Unclassified	GBIF: https://doi.org/10.15468/dl.zfaw79
Anopheles hectoris	-92.473611	16.539444	Unclassified	GBIF: https://doi.org/10.15468/dl.zfaw79
Anopheles hectoris	-92.340833	15.251111	Unclassified	GBIF: https://doi.org/10.15468/dl.zfaw79
Anopheles hectoris	-92.283889	15.175	Unclassified	GBIF: https://doi.org/10.15468/dl.zfaw79
Anopheles hectoris	-92.282222	15.263889	Unclassified	GBIF: https://doi.org/10.15468/dl.zfaw79
Anopheles hectoris	-92.269444	15.332222	Unclassified	GBIF: https://doi.org/10.15468/dl.zfaw79

Anopheles hectoris	-92.090278	15.086111	Unclassified	GBIF: https://doi.org/10.15468/dl.zfaw79
Anopheles hectoris	-91.731444	16.126806	Unclassified	GBIF: https://doi.org/10.15468/dl.zfaw79
Anopheles hectoris	-91.730111	16.1145	Unclassified	GBIF: https://doi.org/10.15468/dl.zfaw79
Anopheles hectoris	-91.727861	16.117944	Unclassified	GBIF: https://doi.org/10.15468/dl.zfaw79
Anopheles hectoris	-91.677472	16.078	Unclassified	GBIF: https://doi.org/10.15468/dl.zfaw79
Anopheles hectoris	-91.667944	16.080389	Unclassified	GBIF: https://doi.org/10.15468/dl.zfaw79
Anopheles hectoris	-91.629472	16.097389	Unclassified	GBIF: https://doi.org/10.15468/dl.zfaw79
Anopheles hectoris	-91.629083	16.097139	Unclassified	GBIF: https://doi.org/10.15468/dl.zfaw79
Anopheles hectoris	-91.628472	16.097417	Unclassified	GBIF: https://doi.org/10.15468/dl.zfaw79
Anopheles hectoris	-91.22523	14.81122	Unclassified	GBIF: https://doi.org/10.15468/dl.zfaw79
Anopheles hectoris	-91.16082	14.73842	Unclassified	GBIF: https://doi.org/10.15468/dl.zfaw79
Anopheles hectoris	-90.94903	14.54687	Unclassified	GBIF: https://doi.org/10.15468/dl.zfaw79
Anopheles hectoris	-90.51229	14.61491	Unclassified	GBIF: https://doi.org/10.15468/dl.zfaw79
Anopheles hectoris	-90.50578	14.6689	Unclassified	GBIF: https://doi.org/10.15468/dl.zfaw79
Anopheles heiheensis	127.466663	50.266668	Unclassified	GBIF: https://doi.org/10.15468/dl.zfaw79
Anopheles hermsi	-117.652	33.927	Unclassified	GBIF: https://doi.org/10.15468/dl.zfaw79
Anopheles hermsi	-117.637	33.9146	Unclassified	GBIF: https://doi.org/10.15468/dl.zfaw79
Anopheles hermsi	-117.636	33.9117	Unclassified	GBIF: https://doi.org/10.15468/dl.zfaw79
Anopheles hermsi	-117.635	33.912	Unclassified	GBIF: https://doi.org/10.15468/dl.zfaw79
Anopheles hermsi	-117.633	33.9083	Unclassified	GBIF: https://doi.org/10.15468/dl.zfaw79
Anopheles hermsi	-117.632	33.9183	Unclassified	GBIF: https://doi.org/10.15468/dl.zfaw79
Anopheles hermsi	-117.632	33.9146	Unclassified	GBIF: https://doi.org/10.15468/dl.zfaw79
Anopheles hermsi	-117.626	33.9143	Unclassified	GBIF: https://doi.org/10.15468/dl.zfaw79
Anopheles hermsi	-117.625	33.9177	Unclassified	GBIF: https://doi.org/10.15468/dl.zfaw79
Anopheles hermsi	-117.621	33.9178	Unclassified	GBIF: https://doi.org/10.15468/dl.zfaw79
Anopheles hermsi	-117.62	33.918	Unclassified	GBIF: https://doi.org/10.15468/dl.zfaw79
Anopheles hermsi	-117.62	33.9231	Unclassified	GBIF: https://doi.org/10.15468/dl.zfaw79
Anopheles hermsi	-117.619	33.8862	Unclassified	GBIF: https://doi.org/10.15468/dl.zfaw79
Anopheles hermsi	-117.615	33.9233	Unclassified	GBIF: https://doi.org/10.15468/dl.zfaw79
Anopheles hermsi	-117.609	33.8621	Unclassified	GBIF: https://doi.org/10.15468/dl.zfaw79
Anopheles hermsi	-117.606	33.9099	Unclassified	GBIF: https://doi.org/10.15468/dl.zfaw79
Anopheles hermsi	-117.604	33.94	Unclassified	GBIF: https://doi.org/10.15468/dl.zfaw79
Anopheles hermsi	-117.603	33.9053	Unclassified	GBIF: https://doi.org/10.15468/dl.zfaw79
Anopheles hermsi	-117.603	33.9393	Unclassified	GBIF: https://doi.org/10.15468/dl.zfaw79
Anopheles hermsi	-117.602	33.8934	Unclassified	GBIF: https://doi.org/10.15468/dl.zfaw79
Anopheles hermsi	-117.601	33.9285	Unclassified	GBIF: https://doi.org/10.15468/dl.zfaw79
Anopheles hermsi	-117.601	33.9267	Unclassified	GBIF: https://doi.org/10.15468/dl.zfaw79
Anopheles hermsi	-117.601	33.8923	Unclassified	GBIF: https://doi.org/10.15468/dl.zfaw79
Anopheles hermsi	-117.599	33.8989	Unclassified	GBIF: https://doi.org/10.15468/dl.zfaw79
Anopheles hermsi	-117.598	33.8953	Unclassified	GBIF: https://doi.org/10.15468/dl.zfaw79
Anopheles hermsi	-117.596	33.8989	Unclassified	GBIF: https://doi.org/10.15468/dl.zfaw79
Anopheles hermsi	-117.596	33.9354	Unclassified	GBIF: https://doi.org/10.15468/dl.zfaw79

Anopheles hermsi	-117.596	33.929	Unclassified	GBIF: https://doi.org/10.15468/dl.zfaw79
Anopheles hermsi	-117.594	33.9652	Unclassified	GBIF: https://doi.org/10.15468/dl.zfaw79
Anopheles hermsi	-117.593	33.9324	Unclassified	GBIF: https://doi.org/10.15468/dl.zfaw79
Anopheles hermsi	-117.592	33.9705	Unclassified	GBIF: https://doi.org/10.15468/dl.zfaw79
Anopheles hermsi	-117.592	33.9409	Unclassified	GBIF: https://doi.org/10.15468/dl.zfaw79
Anopheles hermsi	-117.591	33.9375	Unclassified	GBIF: https://doi.org/10.15468/dl.zfaw79
Anopheles hermsi	-117.59	33.9404	Unclassified	GBIF: https://doi.org/10.15468/dl.zfaw79
Anopheles hermsi	-117.59	33.9507	Unclassified	GBIF: https://doi.org/10.15468/dl.zfaw79
Anopheles hermsi	-117.589	33.8433	Unclassified	GBIF: https://doi.org/10.15468/dl.zfaw79
Anopheles hermsi	-117.587	33.9024	Unclassified	GBIF: https://doi.org/10.15468/dl.zfaw79
Anopheles hermsi	-117.586	33.879	Unclassified	GBIF: https://doi.org/10.15468/dl.zfaw79
Anopheles hermsi	-117.586	33.9435	Unclassified	GBIF: https://doi.org/10.15468/dl.zfaw79
Anopheles hermsi	-117.585	33.9417	Unclassified	GBIF: https://doi.org/10.15468/dl.zfaw79
Anopheles hermsi	-117.582	33.9429	Unclassified	GBIF: https://doi.org/10.15468/dl.zfaw79
Anopheles hermsi	-117.582	33.9446	Unclassified	GBIF: https://doi.org/10.15468/dl.zfaw79
Anopheles hermsi	-117.582	33.9428	Unclassified	GBIF: https://doi.org/10.15468/dl.zfaw79
Anopheles hermsi	-117.582	33.9301	Unclassified	GBIF: https://doi.org/10.15468/dl.zfaw79
Anopheles hermsi	-117.581	33.9458	Unclassified	GBIF: https://doi.org/10.15468/dl.zfaw79
Anopheles hermsi	-117.58	33.9247	Unclassified	GBIF: https://doi.org/10.15468/dl.zfaw79
Anopheles hermsi	-117.579	33.9294	Unclassified	GBIF: https://doi.org/10.15468/dl.zfaw79
Anopheles hermsi	-117.578	33.9481	Unclassified	GBIF: https://doi.org/10.15468/dl.zfaw79
Anopheles hermsi	-117.577	33.9224	Unclassified	GBIF: https://doi.org/10.15468/dl.zfaw79
Anopheles hermsi	-117.577	33.9229	Unclassified	GBIF: https://doi.org/10.15468/dl.zfaw79
Anopheles hermsi	-117.577	33.8955	Unclassified	GBIF: https://doi.org/10.15468/dl.zfaw79
Anopheles hermsi	-117.576	33.9248	Unclassified	GBIF: https://doi.org/10.15468/dl.zfaw79
Anopheles hermsi	-117.576	33.8767	Unclassified	GBIF: https://doi.org/10.15468/dl.zfaw79
Anopheles hermsi	-117.576	33.8916	Unclassified	GBIF: https://doi.org/10.15468/dl.zfaw79
Anopheles hermsi	-117.574	33.9441	Unclassified	GBIF: https://doi.org/10.15468/dl.zfaw79
Anopheles hermsi	-117.574	33.9468	Unclassified	GBIF: https://doi.org/10.15468/dl.zfaw79
Anopheles hermsi	-117.572	33.9447	Unclassified	GBIF: https://doi.org/10.15468/dl.zfaw79
Anopheles hermsi	-117.572	33.9217	Unclassified	GBIF: https://doi.org/10.15468/dl.zfaw79
Anopheles hermsi	-117.572	33.9468	Unclassified	GBIF: https://doi.org/10.15468/dl.zfaw79
Anopheles hermsi	-117.572	33.9483	Unclassified	GBIF: https://doi.org/10.15468/dl.zfaw79
Anopheles hermsi	-117.571	33.9473	Unclassified	GBIF: https://doi.org/10.15468/dl.zfaw79
Anopheles hermsi	-117.571	33.922	Unclassified	GBIF: https://doi.org/10.15468/dl.zfaw79
Anopheles hermsi	-117.569	33.9266	Unclassified	GBIF: https://doi.org/10.15468/dl.zfaw79
Anopheles hermsi	-117.569	33.9408	Unclassified	GBIF: https://doi.org/10.15468/dl.zfaw79
Anopheles hermsi	-117.568	33.9403	Unclassified	GBIF: https://doi.org/10.15468/dl.zfaw79
Anopheles hermsi	-117.567	33.9475	Unclassified	GBIF: https://doi.org/10.15468/dl.zfaw79
Anopheles hermsi	-117.566	33.9508	Unclassified	GBIF: https://doi.org/10.15468/dl.zfaw79
Anopheles hermsi	-117.563	33.9429	Unclassified	GBIF: https://doi.org/10.15468/dl.zfaw79
Anopheles hermsi	-117.563	33.9442	Unclassified	GBIF: https://doi.org/10.15468/dl.zfaw79

Anopheles hermsi	-117.561	33.9451	Unclassified	GBIF: https://doi.org/10.15468/dl.zfaw79
Anopheles hermsi	-117.554	33.905	Unclassified	GBIF: https://doi.org/10.15468/dl.zfaw79
Anopheles hermsi	-117.554	33.9028	Unclassified	GBIF: https://doi.org/10.15468/dl.zfaw79
Anopheles hermsi	-117.545	33.9012	Unclassified	GBIF: https://doi.org/10.15468/dl.zfaw79
Anopheles hermsi	-117.544	33.9003	Unclassified	GBIF: https://doi.org/10.15468/dl.zfaw79
Anopheles hermsi	-117.543	33.8902	Unclassified	GBIF: https://doi.org/10.15468/dl.zfaw79
Anopheles hermsi	-117.54	33.9059	Unclassified	GBIF: https://doi.org/10.15468/dl.zfaw79
Anopheles hermsi	-117.536	33.8602	Unclassified	GBIF: https://doi.org/10.15468/dl.zfaw79
Anopheles hermsi	-117.533	33.8588	Unclassified	GBIF: https://doi.org/10.15468/dl.zfaw79
Anopheles hermsi	-117.533	33.9537	Unclassified	GBIF: https://doi.org/10.15468/dl.zfaw79
Anopheles hermsi	-117.533	33.8584	Unclassified	GBIF: https://doi.org/10.15468/dl.zfaw79
Anopheles hermsi	-117.528	33.9264	Unclassified	GBIF: https://doi.org/10.15468/dl.zfaw79
Anopheles hermsi	-117.527	33.9261	Unclassified	GBIF: https://doi.org/10.15468/dl.zfaw79
Anopheles hermsi	-117.524	33.9258	Unclassified	GBIF: https://doi.org/10.15468/dl.zfaw79
Anopheles hermsi	-117.523	33.968	Unclassified	GBIF: https://doi.org/10.15468/dl.zfaw79
Anopheles hermsi	-117.506	33.9746	Unclassified	GBIF: https://doi.org/10.15468/dl.zfaw79
Anopheles hermsi	-117.505	33.9358	Unclassified	GBIF: https://doi.org/10.15468/dl.zfaw79
Anopheles hermsi	-117.502	33.8068	Unclassified	GBIF: https://doi.org/10.15468/dl.zfaw79
Anopheles hermsi	-117.501	33.885	Unclassified	GBIF: https://doi.org/10.15468/dl.zfaw79
Anopheles hermsi	-117.5	33.9741	Unclassified	GBIF: https://doi.org/10.15468/dl.zfaw79
Anopheles hermsi	-117.5	33.9737	Unclassified	GBIF: https://doi.org/10.15468/dl.zfaw79
Anopheles hermsi	-117.497	33.9618	Unclassified	GBIF: https://doi.org/10.15468/dl.zfaw79
Anopheles hermsi	-117.496	33.963	Unclassified	GBIF: https://doi.org/10.15468/dl.zfaw79
Anopheles hermsi	-117.496	33.9643	Unclassified	GBIF: https://doi.org/10.15468/dl.zfaw79
Anopheles hermsi	-117.491	33.7666	Unclassified	GBIF: https://doi.org/10.15468/dl.zfaw79
Anopheles hermsi	-117.489	33.9617	Unclassified	GBIF: https://doi.org/10.15468/dl.zfaw79
Anopheles hermsi	-117.488	33.964	Unclassified	GBIF: https://doi.org/10.15468/dl.zfaw79
Anopheles hermsi	-117.484	33.9863	Unclassified	GBIF: https://doi.org/10.15468/dl.zfaw79
Anopheles hermsi	-117.484	33.9882	Unclassified	GBIF: https://doi.org/10.15468/dl.zfaw79
Anopheles hermsi	-117.481	33.9896	Unclassified	GBIF: https://doi.org/10.15468/dl.zfaw79
Anopheles hermsi	-117.479	33.9917	Unclassified	GBIF: https://doi.org/10.15468/dl.zfaw79
Anopheles hermsi	-117.471	33.9994	Unclassified	GBIF: https://doi.org/10.15468/dl.zfaw79
Anopheles hermsi	-117.469	33.9577	Unclassified	GBIF: https://doi.org/10.15468/dl.zfaw79
Anopheles hermsi	-117.469	33.7598	Unclassified	GBIF: https://doi.org/10.15468/dl.zfaw79
Anopheles hermsi	-117.458	33.8776	Unclassified	GBIF: https://doi.org/10.15468/dl.zfaw79
Anopheles hermsi	-117.451	33.9171	Unclassified	GBIF: https://doi.org/10.15468/dl.zfaw79
Anopheles hermsi	-117.446	33.9149	Unclassified	GBIF: https://doi.org/10.15468/dl.zfaw79
Anopheles hermsi	-117.434	33.9826	Unclassified	GBIF: https://doi.org/10.15468/dl.zfaw79
Anopheles hermsi	-117.433	33.9788	Unclassified	GBIF: https://doi.org/10.15468/dl.zfaw79
Anopheles hermsi	-117.431	33.9667	Unclassified	GBIF: https://doi.org/10.15468/dl.zfaw79
Anopheles hermsi	-117.427	33.9636	Unclassified	GBIF: https://doi.org/10.15468/dl.zfaw79
Anopheles hermsi	-117.426	33.976	Unclassified	GBIF: https://doi.org/10.15468/dl.zfaw79

Anopheles hermsi	-117.423	33.9777	Unclassified	GBIF: https://doi.org/10.15468/dl.zfaw79
Anopheles hermsi	-117.423	33.9775	Unclassified	GBIF: https://doi.org/10.15468/dl.zfaw79
Anopheles hermsi	-117.421	33.9811	Unclassified	GBIF: https://doi.org/10.15468/dl.zfaw79
Anopheles hermsi	-117.421	33.9652	Unclassified	GBIF: https://doi.org/10.15468/dl.zfaw79
Anopheles hermsi	-117.417	33.982	Unclassified	GBIF: https://doi.org/10.15468/dl.zfaw79
Anopheles hermsi	-117.417	33.9811	Unclassified	GBIF: https://doi.org/10.15468/dl.zfaw79
Anopheles hermsi	-117.417	33.9819	Unclassified	GBIF: https://doi.org/10.15468/dl.zfaw79
Anopheles hermsi	-117.417	33.9803	Unclassified	GBIF: https://doi.org/10.15468/dl.zfaw79
Anopheles hermsi	-117.4	33.9591	Unclassified	GBIF: https://doi.org/10.15468/dl.zfaw79
Anopheles hermsi	-117.397	33.6921	Unclassified	GBIF: https://doi.org/10.15468/dl.zfaw79
Anopheles hermsi	-117.396	33.8762	Unclassified	GBIF: https://doi.org/10.15468/dl.zfaw79
Anopheles hermsi	-117.395	33.9733	Unclassified	GBIF: https://doi.org/10.15468/dl.zfaw79
Anopheles hermsi	-117.39	33.9762	Unclassified	GBIF: https://doi.org/10.15468/dl.zfaw79
Anopheles hermsi	-117.387	33.7076	Unclassified	GBIF: https://doi.org/10.15468/dl.zfaw79
Anopheles hermsi	-117.376	33.9338	Unclassified	GBIF: https://doi.org/10.15468/dl.zfaw79
Anopheles hermsi	-117.373	33.9974	Unclassified	GBIF: https://doi.org/10.15468/dl.zfaw79
Anopheles hermsi	-117.368	33.86	Unclassified	GBIF: https://doi.org/10.15468/dl.zfaw79
Anopheles hermsi	-117.365	33.8911	Unclassified	GBIF: https://doi.org/10.15468/dl.zfaw79
Anopheles hermsi	-117.358	33.7016	Unclassified	GBIF: https://doi.org/10.15468/dl.zfaw79
Anopheles hermsi	-117.356	33.6977	Unclassified	GBIF: https://doi.org/10.15468/dl.zfaw79
Anopheles hermsi	-117.355	33.6982	Unclassified	GBIF: https://doi.org/10.15468/dl.zfaw79
Anopheles hermsi	-117.353	34.0105	Unclassified	GBIF: https://doi.org/10.15468/dl.zfaw79
Anopheles hermsi	-117.352	33.6928	Unclassified	GBIF: https://doi.org/10.15468/dl.zfaw79
Anopheles hermsi	-117.351	33.6944	Unclassified	GBIF: https://doi.org/10.15468/dl.zfaw79
Anopheles hermsi	-117.351	33.9533	Unclassified	GBIF: https://doi.org/10.15468/dl.zfaw79
Anopheles hermsi	-117.351	33.9569	Unclassified	GBIF: https://doi.org/10.15468/dl.zfaw79
Anopheles hermsi	-117.35	33.6986	Unclassified	GBIF: https://doi.org/10.15468/dl.zfaw79
Anopheles hermsi	-117.35	33.9608	Unclassified	GBIF: https://doi.org/10.15468/dl.zfaw79
Anopheles hermsi	-117.349	33.6957	Unclassified	GBIF: https://doi.org/10.15468/dl.zfaw79
Anopheles hermsi	-117.348	33.949	Unclassified	GBIF: https://doi.org/10.15468/dl.zfaw79
Anopheles hermsi	-117.344	33.9356	Unclassified	GBIF: https://doi.org/10.15468/dl.zfaw79
Anopheles hermsi	-117.344	33.6828	Unclassified	GBIF: https://doi.org/10.15468/dl.zfaw79
Anopheles hermsi	-117.342	33.6819	Unclassified	GBIF: https://doi.org/10.15468/dl.zfaw79
Anopheles hermsi	-117.329	33.8885	Unclassified	GBIF: https://doi.org/10.15468/dl.zfaw79
Anopheles hermsi	-117.329	34.0159	Unclassified	GBIF: https://doi.org/10.15468/dl.zfaw79
Anopheles hermsi	-117.323	33.8764	Unclassified	GBIF: https://doi.org/10.15468/dl.zfaw79
Anopheles hermsi	-117.319	33.8811	Unclassified	GBIF: https://doi.org/10.15468/dl.zfaw79
Anopheles hermsi	-117.318	33.6315	Unclassified	GBIF: https://doi.org/10.15468/dl.zfaw79
Anopheles hermsi	-117.311	33.9571	Unclassified	GBIF: https://doi.org/10.15468/dl.zfaw79
Anopheles hermsi	-117.308	33.6222	Unclassified	GBIF: https://doi.org/10.15468/dl.zfaw79
Anopheles hermsi	-117.307	33.6491	Unclassified	GBIF: https://doi.org/10.15468/dl.zfaw79
Anopheles hermsi	-117.3	33.8874	Unclassified	GBIF: https://doi.org/10.15468/dl.zfaw79

Anopheles hermsi	-117.291	33.6709	Unclassified	GBIF: https://doi.org/10.15468/dl.zfaw79
Anopheles hermsi	-117.271	33.672	Unclassified	GBIF: https://doi.org/10.15468/dl.zfaw79
Anopheles hermsi	-117.249	33.6611	Unclassified	GBIF: https://doi.org/10.15468/dl.zfaw79
Anopheles hermsi	-117.248	33.6597	Unclassified	GBIF: https://doi.org/10.15468/dl.zfaw79
Anopheles hermsi	-117.247	33.66	Unclassified	GBIF: https://doi.org/10.15468/dl.zfaw79
Anopheles hermsi	-117.247	33.6603	Unclassified	GBIF: https://doi.org/10.15468/dl.zfaw79
Anopheles hermsi	-117.24	33.657	Unclassified	GBIF: https://doi.org/10.15468/dl.zfaw79
Anopheles hermsi	-117.236	33.6805	Unclassified	GBIF: https://doi.org/10.15468/dl.zfaw79
Anopheles hermsi	-117.219484	32.897823	Unclassified	GBIF: https://doi.org/10.15468/dl.zfaw79
Anopheles hermsi	-116.92919	33.799741	Unclassified	GBIF: https://doi.org/10.15468/dl.zfaw79
Anopheles hermsi	-116.374024	31.715331	Unclassified	GBIF: https://doi.org/10.15468/dl.zfaw79
Anopheles hermsi	-115.870406	30.733297	Unclassified	GBIF: https://doi.org/10.15468/dl.zfaw79
Anopheles hermsi	-109.205977	28.758946	Unclassified	GBIF: https://doi.org/10.15468/dl.zfaw79
Anopheles hermsi	-109.20323	28.730049	Unclassified	GBIF: https://doi.org/10.15468/dl.zfaw79
Anopheles hermsi	-109.20323	28.725232	Unclassified	GBIF: https://doi.org/10.15468/dl.zfaw79
Anopheles hermsi	-109.200484	28.725232	Unclassified	GBIF: https://doi.org/10.15468/dl.zfaw79
Anopheles hermsi	-109.200484	28.72764	Unclassified	GBIF: https://doi.org/10.15468/dl.zfaw79
Anopheles hermsi	-109.200484	28.746907	Unclassified	GBIF: https://doi.org/10.15468/dl.zfaw79
Anopheles hermsi	-109.200484	28.718006	Unclassified	GBIF: https://doi.org/10.15468/dl.zfaw79
Anopheles hermsi	-109.200484	28.751723	Unclassified	GBIF: https://doi.org/10.15468/dl.zfaw79
Anopheles hermsi	-109.200484	28.734866	Unclassified	GBIF: https://doi.org/10.15468/dl.zfaw79
Anopheles hermsi	-109.200484	28.74209	Unclassified	GBIF: https://doi.org/10.15468/dl.zfaw79
Anopheles hermsi	-109.200484	28.739682	Unclassified	GBIF: https://doi.org/10.15468/dl.zfaw79
Anopheles hermsi	-109.19911	28.756539	Unclassified	GBIF: https://doi.org/10.15468/dl.zfaw79
Anopheles hermsi	-109.197737	28.746907	Unclassified	GBIF: https://doi.org/10.15468/dl.zfaw79
Anopheles hermsi	-109.197737	28.713188	Unclassified	GBIF: https://doi.org/10.15468/dl.zfaw79
Anopheles hermsi	-109.197737	28.720414	Unclassified	GBIF: https://doi.org/10.15468/dl.zfaw79
Anopheles hermsi	-109.197737	28.725232	Unclassified	GBIF: https://doi.org/10.15468/dl.zfaw79
Anopheles hermsi	-109.197737	28.718006	Unclassified	GBIF: https://doi.org/10.15468/dl.zfaw79
Anopheles hermsi	-109.197737	28.737274	Unclassified	GBIF: https://doi.org/10.15468/dl.zfaw79
Anopheles hermsi	-109.197737	28.710779	Unclassified	GBIF: https://doi.org/10.15468/dl.zfaw79
Anopheles hermsi	-109.197737	28.72764	Unclassified	GBIF: https://doi.org/10.15468/dl.zfaw79
Anopheles hermsi	-109.197737	28.730049	Unclassified	GBIF: https://doi.org/10.15468/dl.zfaw79
Anopheles hermsi	-109.197737	28.744499	Unclassified	GBIF: https://doi.org/10.15468/dl.zfaw79
Anopheles hermsi	-109.197737	28.756539	Unclassified	GBIF: https://doi.org/10.15468/dl.zfaw79
Anopheles hermsi	-109.197737	28.768577	Unclassified	GBIF: https://doi.org/10.15468/dl.zfaw79
Anopheles hermsi	-98.150882	24.854906	Unclassified	GBIF: https://doi.org/10.15468/dl.zfaw79
Anopheles hervyi	9.2	13.699999	Unclassified	GBIF: https://doi.org/10.15468/dl.zfaw79
Anopheles hilli	117.03333	-31.2	Unclassified	GBIF: https://doi.org/10.15468/dl.zfaw79
Anopheles hilli	117.033333	-31.200001	Unclassified	GBIF: https://doi.org/10.15468/dl.zfaw79
Anopheles hilli	122.166667	-17.316667	Unclassified	GBIF: https://doi.org/10.15468/dl.zfaw79
Anopheles hilli	128.100006	-15.466666	Unclassified	GBIF: https://doi.org/10.15468/dl.zfaw79

Anopheles hilli	128.10001	-15.46667	Unclassified	GBIF: https://doi.org/10.15468/dl.zfaw79
Anopheles hilli	128.35	-15.5	Unclassified	GBIF: https://doi.org/10.15468/dl.zfaw79
Anopheles hilli	129.55	-14.1	Unclassified	GBIF: https://doi.org/10.15468/dl.zfaw79
Anopheles hilli	129.550003	-14.1	Unclassified	GBIF: https://doi.org/10.15468/dl.zfaw79
Anopheles hilli	130.6833	-13.75	Unclassified	GBIF: https://doi.org/10.15468/dl.zfaw79
Anopheles hilli	130.683304	-13.75	Unclassified	GBIF: https://doi.org/10.15468/dl.zfaw79
Anopheles hilli	130.828	-12.452	Unclassified	GBIF: https://doi.org/10.15468/dl.zfaw79
Anopheles hilli	130.83	-12.45	Unclassified	GBIF: https://doi.org/10.15468/dl.zfaw79
Anopheles hilli	130.833333	-12.466667	Unclassified	GBIF: https://doi.org/10.15468/dl.zfaw79
Anopheles hilli	130.85	-12.384	Unclassified	GBIF: https://doi.org/10.15468/dl.zfaw79
Anopheles hilli	130.850006	-12.416667	Unclassified	GBIF: https://doi.org/10.15468/dl.zfaw79
Anopheles hilli	130.8667	-12.45	Unclassified	GBIF: https://doi.org/10.15468/dl.zfaw79
Anopheles hilli	130.904401	-12.364106	Unclassified	GBIF: https://doi.org/10.15468/dl.zfaw79
Anopheles hilli	130.91667	-12.41667	Unclassified	GBIF: https://doi.org/10.15468/dl.zfaw79
Anopheles hilli	130.916672	-12.416667	Unclassified	GBIF: https://doi.org/10.15468/dl.zfaw79
Anopheles hilli	130.920405	-12.394625	Unclassified	GBIF: https://doi.org/10.15468/dl.zfaw79
Anopheles hilli	131.100006	-13.233334	Unclassified	GBIF: https://doi.org/10.15468/dl.zfaw79
Anopheles hilli	131.10001	-13.23333	Unclassified	GBIF: https://doi.org/10.15468/dl.zfaw79
Anopheles hilli	131.105	-13.23999	Unclassified	GBIF: https://doi.org/10.15468/dl.zfaw79
Anopheles hilli	131.11	-13.24	Unclassified	GBIF: https://doi.org/10.15468/dl.zfaw79
Anopheles hilli	134.733337	-14.733334	Unclassified	GBIF: https://doi.org/10.15468/dl.zfaw79
Anopheles hilli	134.73334	-14.73333	Unclassified	GBIF: https://doi.org/10.15468/dl.zfaw79
Anopheles hilli	136.46666	-13.9	Unclassified	GBIF: https://doi.org/10.15468/dl.zfaw79
Anopheles hilli	136.662	-15.603	Unclassified	GBIF: https://doi.org/10.15468/dl.zfaw79
Anopheles hilli	136.71666	-12.183333	Unclassified	GBIF: https://doi.org/10.15468/dl.zfaw79
Anopheles hilli	136.71666	-12.18333	Unclassified	GBIF: https://doi.org/10.15468/dl.zfaw79
Anopheles hilli	136.753	-12.216	Unclassified	GBIF: https://doi.org/10.15468/dl.zfaw79
Anopheles hilli	136.76666	-12.16667	Unclassified	GBIF: https://doi.org/10.15468/dl.zfaw79
Anopheles hilli	136.766663	-12.166667	Unclassified	GBIF: https://doi.org/10.15468/dl.zfaw79
Anopheles hilli	136.76669	-12.18333	Unclassified	GBIF: https://doi.org/10.15468/dl.zfaw79
Anopheles hilli	136.766693	-12.18333	Unclassified	GBIF: https://doi.org/10.15468/dl.zfaw79
Anopheles hilli	136.816667	-15.733333	Unclassified	GBIF: https://doi.org/10.15468/dl.zfaw79
Anopheles hilli	141.066666	-17.666666	Unclassified	GBIF: https://doi.org/10.15468/dl.zfaw79
Anopheles hilli	141.06667	-17.66667	Unclassified	GBIF: https://doi.org/10.15468/dl.zfaw79
Anopheles hilli	142.116669	-10.116667	Unclassified	GBIF: https://doi.org/10.15468/dl.zfaw79
Anopheles hilli	142.11667	-10.11667	Unclassified	GBIF: https://doi.org/10.15468/dl.zfaw79
Anopheles hilli	142.21666	-9.283334	Unclassified	GBIF: https://doi.org/10.15468/dl.zfaw79
Anopheles hilli	142.21666	-10.583333	Unclassified	GBIF: https://doi.org/10.15468/dl.zfaw79
Anopheles hilli	142.21666	-9.28333	Unclassified	GBIF: https://doi.org/10.15468/dl.zfaw79
Anopheles hilli	142.21666	-10.58333	Unclassified	GBIF: https://doi.org/10.15468/dl.zfaw79
Anopheles hilli	142.28334	-10.616667	Unclassified	GBIF: https://doi.org/10.15468/dl.zfaw79
Anopheles hilli	142.28334	-10.61667	Unclassified	GBIF: https://doi.org/10.15468/dl.zfaw79

Anopheles hilli	142.46666	-10.8	Unclassified	GBIF: https://doi.org/10.15468/dl.zfaw79
Anopheles hilli	142.467	-10.8	Unclassified	GBIF: https://doi.org/10.15468/dl.zfaw79
Anopheles hilli	142.66667	-9.38333	Unclassified	GBIF: https://doi.org/10.15468/dl.zfaw79
Anopheles hilli	142.666672	-9.383333	Unclassified	GBIF: https://doi.org/10.15468/dl.zfaw79
Anopheles hilli	143.466705	-13.7667	Unclassified	GBIF: https://doi.org/10.15468/dl.zfaw79
Anopheles hilli	143.46671	-13.7667	Unclassified	GBIF: https://doi.org/10.15468/dl.zfaw79
Anopheles hilli	143.66667	-14.06667	Unclassified	GBIF: https://doi.org/10.15468/dl.zfaw79
Anopheles hilli	143.666672	-14.066667	Unclassified	GBIF: https://doi.org/10.15468/dl.zfaw79
Anopheles hilli	146.203	-18.289	Unclassified	GBIF: https://doi.org/10.15468/dl.zfaw79
Anopheles hilli	146.28	-18.73333	Unclassified	GBIF: https://doi.org/10.15468/dl.zfaw79
Anopheles hilli	146.300003	-18.733334	Unclassified	GBIF: https://doi.org/10.15468/dl.zfaw79
Anopheles hilli	146.333328	-18.533333	Unclassified	GBIF: https://doi.org/10.15468/dl.zfaw79
Anopheles hilli	146.33333	-18.53333	Unclassified	GBIF: https://doi.org/10.15468/dl.zfaw79
Anopheles hilli	146.5	-18.7	Unclassified	GBIF: https://doi.org/10.15468/dl.zfaw79
Anopheles hilli	146.816666	-19.266666	Unclassified	GBIF: https://doi.org/10.15468/dl.zfaw79
Anopheles hilli	146.81667	-19.26667	Unclassified	GBIF: https://doi.org/10.15468/dl.zfaw79
Anopheles hilli	146.816696	-19.26667	Unclassified	GBIF: https://doi.org/10.15468/dl.zfaw79
Anopheles hilli	146.8167	-19.26667	Unclassified	GBIF: https://doi.org/10.15468/dl.zfaw79
Anopheles hilli	148.16667	-20.01667	Unclassified	GBIF: https://doi.org/10.15468/dl.zfaw79
Anopheles hilli	148.166672	-20.016666	Unclassified	GBIF: https://doi.org/10.15468/dl.zfaw79
Anopheles hilli	150.333328	-10.333333	Unclassified	GBIF: https://doi.org/10.15468/dl.zfaw79
Anopheles hilli	150.33333	-10.33333	Unclassified	GBIF: https://doi.org/10.15468/dl.zfaw79
Anopheles hilli	151.116669	-23.833334	Unclassified	GBIF: https://doi.org/10.15468/dl.zfaw79
Anopheles hilli	151.11667	-23.83333	Unclassified	GBIF: https://doi.org/10.15468/dl.zfaw79
Anopheles hilli	151.26666	-23.85	Unclassified	GBIF: https://doi.org/10.15468/dl.zfaw79
Anopheles hilli	151.266663	-23.85	Unclassified	GBIF: https://doi.org/10.15468/dl.zfaw79
Anopheles hilli	151.333328	-23.933332	Unclassified	GBIF: https://doi.org/10.15468/dl.zfaw79
Anopheles hilli	151.33333	-23.93333	Unclassified	GBIF: https://doi.org/10.15468/dl.zfaw79
Anopheles hilli	152.25	-24.85	Unclassified	GBIF: https://doi.org/10.15468/dl.zfaw79
Anopheles hilli	152.350006	-24.866667	Unclassified	GBIF: https://doi.org/10.15468/dl.zfaw79
Anopheles hilli	152.35001	-24.86667	Unclassified	GBIF: https://doi.org/10.15468/dl.zfaw79
Anopheles hilli	153.13333	-27.45	Unclassified	GBIF: https://doi.org/10.15468/dl.zfaw79
Anopheles hilli	153.133331	-27.450001	Unclassified	GBIF: https://doi.org/10.15468/dl.zfaw79
Anopheles hilli	153.402	-27.483	Unclassified	GBIF: https://doi.org/10.15468/dl.zfaw79
Anopheles hilli	153.41667	-27.48333	Unclassified	GBIF: https://doi.org/10.15468/dl.zfaw79
Anopheles hilli	153.41667	-27.96667	Unclassified	GBIF: https://doi.org/10.15468/dl.zfaw79
Anopheles hilli	153.416672	-27.966667	Unclassified	GBIF: https://doi.org/10.15468/dl.zfaw79
Anopheles hinesorum	142.66667	-9.38333	Unclassified	GBIF: https://doi.org/10.15468/dl.zfaw79
Anopheles hinesorum	142.666672	-9.383333	Unclassified	GBIF: https://doi.org/10.15468/dl.zfaw79
Anopheles hinesorum	145.12883	-4.550967	Unclassified	GBIF: https://doi.org/10.15468/dl.zfaw79
Anopheles hinesorum	145.316666	-16.25	Unclassified	GBIF: https://doi.org/10.15468/dl.zfaw79
Anopheles hinesorum	145.31667	-16.25	Unclassified	GBIF: https://doi.org/10.15468/dl.zfaw79

Anopheles hinesorum	145.76666	-16.91667	Unclassified	GBIF: https://doi.org/10.15468/dl.zfaw79
Anopheles hinesorum	145.766663	-16.916666	Unclassified	GBIF: https://doi.org/10.15468/dl.zfaw79
Anopheles hinesorum	145.966705	-17.433332	Unclassified	GBIF: https://doi.org/10.15468/dl.zfaw79
Anopheles hinesorum	145.96671	-17.43333	Unclassified	GBIF: https://doi.org/10.15468/dl.zfaw79
Anopheles hinesorum	146.066666	-17.866667	Unclassified	GBIF: https://doi.org/10.15468/dl.zfaw79
Anopheles hinesorum	146.06667	-17.86667	Unclassified	GBIF: https://doi.org/10.15468/dl.zfaw79
Anopheles hinesorum	146.083328	-17.566668	Unclassified	GBIF: https://doi.org/10.15468/dl.zfaw79
Anopheles hinesorum	146.08333	-17.56667	Unclassified	GBIF: https://doi.org/10.15468/dl.zfaw79
Anopheles hinesorum	146.1	-17.566667	Unclassified	GBIF: https://doi.org/10.15468/dl.zfaw79
Anopheles hinesorum	146.116669	-17.6	Unclassified	GBIF: https://doi.org/10.15468/dl.zfaw79
Anopheles hinesorum	146.11667	-17.6	Unclassified	GBIF: https://doi.org/10.15468/dl.zfaw79
Anopheles hinesorum	159.83	-9.37	Unclassified	GBIF: https://doi.org/10.15468/dl.zfaw79
Anopheles hinesorum	159.88	-9.38	Unclassified	GBIF: https://doi.org/10.15468/dl.zfaw79
Anopheles hinesorum	159.8833	-9.4	Unclassified	GBIF: https://doi.org/10.15468/dl.zfaw79
Anopheles hinesorum	159.8846	-9.42	Unclassified	GBIF: https://doi.org/10.15468/dl.zfaw79
Anopheles hinesorum	160.031	-9.405	Unclassified	GBIF: https://doi.org/10.15468/dl.zfaw79
Anopheles hinesorum	160.883301	-9.4	Unclassified	GBIF: https://doi.org/10.15468/dl.zfaw79
Anopheles hodgkini	98.49348	8.48945	Unclassified	GBIF: https://doi.org/10.15468/dl.zfaw79
Anopheles hodgkini	98.62446	8.58293	Unclassified	GBIF: https://doi.org/10.15468/dl.zfaw79
Anopheles hodgkini	98.8133	8.855	Unclassified	GBIF: https://doi.org/10.15468/dl.zfaw79
Anopheles hodgkini	98.97828	14.27654	Unclassified	GBIF: https://doi.org/10.15468/dl.zfaw79
Anopheles hodgkini	99.92632	9.56368	Unclassified	GBIF: https://doi.org/10.15468/dl.zfaw79
Anopheles hodgkini	99.92635	9.57272	Unclassified	GBIF: https://doi.org/10.15468/dl.zfaw79
Anopheles hodgkini	99.93536	9.53653	Unclassified	GBIF: https://doi.org/10.15468/dl.zfaw79
Anopheles hodgkini	99.93539	9.54557	Unclassified	GBIF: https://doi.org/10.15468/dl.zfaw79
Anopheles hodgkini	99.93541	9.55461	Unclassified	GBIF: https://doi.org/10.15468/dl.zfaw79
Anopheles hodgkini	101.39096	14.53559	Unclassified	GBIF: https://doi.org/10.15468/dl.zfaw79
Anopheles hodgkini	101.4474	14.60728	Unclassified	GBIF: https://doi.org/10.15468/dl.zfaw79
Anopheles hodgkini	101.794338	13.49568	Unclassified	GBIF: https://doi.org/10.15468/dl.zfaw79
Anopheles hodgkini	101.82424	14.33206	Unclassified	GBIF: https://doi.org/10.15468/dl.zfaw79
Anopheles hodgkini	101.8335	14.33195	Unclassified	GBIF: https://doi.org/10.15468/dl.zfaw79
Anopheles hodgkini	101.88815	14.25904	Unclassified	GBIF: https://doi.org/10.15468/dl.zfaw79
Anopheles hodgkini	102.661542	14.162085	Unclassified	GBIF: https://doi.org/10.15468/dl.zfaw79
Anopheles hodgkini	103.012442	14.383042	Unclassified	GBIF: https://doi.org/10.15468/dl.zfaw79
Anopheles hodgkini	104.369222	14.519333	Unclassified	GBIF: https://doi.org/10.15468/dl.zfaw79
Anopheles hodgkini	104.369222	14.521444	Unclassified	GBIF: https://doi.org/10.15468/dl.zfaw79
Anopheles hodgkini	105.459964	15.132515	Unclassified	GBIF: https://doi.org/10.15468/dl.zfaw79
Anopheles homunculus	-73.61867	4.14669	Unclassified	GBIF: https://doi.org/10.15468/dl.zfaw79
Anopheles homunculus	-61.31597	10.72592	Unclassified	GBIF: https://doi.org/10.15468/dl.zfaw79
Anopheles homunculus	-61.298692	10.717962	Unclassified	GBIF: https://doi.org/10.15468/dl.zfaw79
Anopheles homunculus	-61.2631	10.37305	Unclassified	GBIF: https://doi.org/10.15468/dl.zfaw79
Anopheles homunculus	-61.2	10.4	Unclassified	GBIF: https://doi.org/10.15468/dl.zfaw79

Anopheles homunculus	-61.17	10.47	Unclassified	GBIF: https://doi.org/10.15468/dl.zfaw79
Anopheles homunculus	-61.16084	10.67984	Unclassified	GBIF: https://doi.org/10.15468/dl.zfaw79
Anopheles homunculus	-61.16068	10.70696	Unclassified	GBIF: https://doi.org/10.15468/dl.zfaw79
Anopheles homunculus	-61	11	Unclassified	GBIF: https://doi.org/10.15468/dl.zfaw79
Anopheles homunculus	-48.8293	-25.4754	Unclassified	GBIF: https://doi.org/10.15468/dl.zfaw79
Anopheles homunculus	-48.5427	-25.5128	Unclassified	GBIF: https://doi.org/10.15468/dl.zfaw79
Anopheles hughi	30.547768	-22.808308	Unclassified	GBIF: https://doi.org/10.15468/dl.zfaw79
Anopheles hunteri	105.164167	-2.064444	Unclassified	GBIF: https://doi.org/10.15468/dl.zfaw79
Anopheles hyrcanus	2.045278	49.413612	Unclassified	GBIF: https://doi.org/10.15468/dl.zfaw79
Anopheles hyrcanus	16.484541	48.194892	Unclassified	GBIF: https://doi.org/10.15468/dl.zfaw79
Anopheles hyrcanus	16.766116	47.769099	Unclassified	GBIF: https://doi.org/10.15468/dl.zfaw79
Anopheles hyrcanus	16.766236	47.769053	Unclassified	GBIF: https://doi.org/10.15468/dl.zfaw79
Anopheles hyrcanus	17.581823	46.755663	Unclassified	GBIF: https://doi.org/10.15468/dl.zfaw79
Anopheles hyrcanus	20.883435	47.612127	Unclassified	GBIF: https://doi.org/10.15468/dl.zfaw79
Anopheles hyrcanus	21.545323	47.468836	Unclassified	GBIF: https://doi.org/10.15468/dl.zfaw79
Anopheles hyrcanus	21.772222	41.716389	Unclassified	GBIF: https://doi.org/10.15468/dl.zfaw79
Anopheles hyrcanus	21.969591	51.444094	Unclassified	GBIF: https://doi.org/10.15468/dl.zfaw79
Anopheles hyrcanus	22.693652	40.730494	Unclassified	GBIF: https://doi.org/10.15468/dl.zfaw79
Anopheles hyrcanus	22.693928	40.730382	Unclassified	GBIF: https://doi.org/10.15468/dl.zfaw79
Anopheles hyrcanus	22.8582	40.688	Unclassified	GBIF: https://doi.org/10.15468/dl.zfaw79
Anopheles hyrcanus	28.640798	44.086321	Unclassified	GBIF: https://doi.org/10.15468/dl.zfaw79
Anopheles hyrcanus	29.218895	44.992763	Unclassified	GBIF: https://doi.org/10.15468/dl.zfaw79
Anopheles hyrcanus	29.374061	45.133232	Unclassified	GBIF: https://doi.org/10.15468/dl.zfaw79
Anopheles hyrcanus	29.52	45.15	Unclassified	GBIF: https://doi.org/10.15468/dl.zfaw79
Anopheles hyrcanus	30.20254	46.469552	Unclassified	GBIF: https://doi.org/10.15468/dl.zfaw79
Anopheles hyrcanus	30.217518	46.460576	Unclassified	GBIF: https://doi.org/10.15468/dl.zfaw79
Anopheles hyrcanus	30.248647	46.434434	Unclassified	GBIF: https://doi.org/10.15468/dl.zfaw79
Anopheles hyrcanus	30.257596	47.425157	Unclassified	GBIF: https://doi.org/10.15468/dl.zfaw79
Anopheles hyrcanus	30.257596	46.426746	Unclassified	GBIF: https://doi.org/10.15468/dl.zfaw79
Anopheles hyrcanus	30.423187	46.196734	Unclassified	GBIF: https://doi.org/10.15468/dl.zfaw79
Anopheles hyrcanus	30.561477	46.349917	Unclassified	GBIF: https://doi.org/10.15468/dl.zfaw79
Anopheles hyrcanus	30.561922	46.350012	Unclassified	GBIF: https://doi.org/10.15468/dl.zfaw79
Anopheles hyrcanus	30.573667	46.34967	Unclassified	GBIF: https://doi.org/10.15468/dl.zfaw79
Anopheles hyrcanus	30.579027	46.352008	Unclassified	GBIF: https://doi.org/10.15468/dl.zfaw79
Anopheles hyrcanus	30.582815	46.411009	Unclassified	GBIF: https://doi.org/10.15468/dl.zfaw79
Anopheles hyrcanus	30.588995	46.4029	Unclassified	GBIF: https://doi.org/10.15468/dl.zfaw79
Anopheles hyrcanus	30.59706	46.409883	Unclassified	GBIF: https://doi.org/10.15468/dl.zfaw79
Anopheles hyrcanus	30.638961	46.386715	Unclassified	GBIF: https://doi.org/10.15468/dl.zfaw79
Anopheles hyrcanus	31.994583	46.975033	Unclassified	GBIF: https://doi.org/10.15468/dl.zfaw79
Anopheles hyrcanus	32.26944	46.530043	Unclassified	GBIF: https://doi.org/10.15468/dl.zfaw79
Anopheles hyrcanus	32.722877	46.663592	Unclassified	GBIF: https://doi.org/10.15468/dl.zfaw79
Anopheles hyrcanus	32.723924	46.664251	Unclassified	GBIF: https://doi.org/10.15468/dl.zfaw79

Anopheles hyrcanus	81.25015	43.88003	Unclassified	GBIF: https://doi.org/10.15468/dl.zfaw79
Anopheles hyrcanus	83.30015	43.52003	Unclassified	GBIF: https://doi.org/10.15468/dl.zfaw79
Anopheles hyrcanus	95.323611	5.549444	Unclassified	GBIF: https://doi.org/10.15468/dl.zfaw79
Anopheles hyrcanus	98.492807	9.451781	Unclassified	GBIF: https://doi.org/10.15468/dl.zfaw79
Anopheles hyrcanus	98.785	1.736667	Unclassified	GBIF: https://doi.org/10.15468/dl.zfaw79
Anopheles hyrcanus	98.847827	9.891034	Unclassified	GBIF: https://doi.org/10.15468/dl.zfaw79
Anopheles hyrcanus	98.9533	9.03	Unclassified	GBIF: https://doi.org/10.15468/dl.zfaw79
Anopheles hyrcanus	98.958039	10.515357	Unclassified	GBIF: https://doi.org/10.15468/dl.zfaw79
Anopheles hyrcanus	99.056241	9.893162	Unclassified	GBIF: https://doi.org/10.15468/dl.zfaw79
Anopheles hyrcanus	99.062369	10.537866	Unclassified	GBIF: https://doi.org/10.15468/dl.zfaw79
Anopheles hyrcanus	99.065485	10.492812	Unclassified	GBIF: https://doi.org/10.15468/dl.zfaw79
Anopheles hyrcanus	99.074596	10.375233	Unclassified	GBIF: https://doi.org/10.15468/dl.zfaw79
Anopheles hyrcanus	99.081334	9.951966	Unclassified	GBIF: https://doi.org/10.15468/dl.zfaw79
Anopheles hyrcanus	99.082009	10.772276	Unclassified	GBIF: https://doi.org/10.15468/dl.zfaw79
Anopheles hyrcanus	99.116314	10.798231	Unclassified	GBIF: https://doi.org/10.15468/dl.zfaw79
Anopheles hyrcanus	99.119962	10.511094	Unclassified	GBIF: https://doi.org/10.15468/dl.zfaw79
Anopheles hyrcanus	99.135314	9.793432	Unclassified	GBIF: https://doi.org/10.15468/dl.zfaw79
Anopheles hyrcanus	99.181219	10.671592	Unclassified	GBIF: https://doi.org/10.15468/dl.zfaw79
Anopheles hyrcanus	99.23568	10.871475	Unclassified	GBIF: https://doi.org/10.15468/dl.zfaw79
Anopheles hyrcanus	99.257729	10.888848	Unclassified	GBIF: https://doi.org/10.15468/dl.zfaw79
Anopheles hyrcanus	99.2725	1.378056	Unclassified	GBIF: https://doi.org/10.15468/dl.zfaw79
Anopheles hyrcanus	99.28559	10.923141	Unclassified	GBIF: https://doi.org/10.15468/dl.zfaw79
Anopheles hyrcanus	99.347572	10.921539	Unclassified	GBIF: https://doi.org/10.15468/dl.zfaw79
Anopheles hyrcanus	99.359444	0.798056	Unclassified	GBIF: https://doi.org/10.15468/dl.zfaw79
Anopheles hyrcanus	99.360871	10.673497	Unclassified	GBIF: https://doi.org/10.15468/dl.zfaw79
Anopheles hyrcanus	99.503618	10.914247	Unclassified	GBIF: https://doi.org/10.15468/dl.zfaw79
Anopheles hyrcanus	100.012778	0.537222	Unclassified	GBIF: https://doi.org/10.15468/dl.zfaw79
Anopheles hyrcanus	100.3675	-0.300278	Unclassified	GBIF: https://doi.org/10.15468/dl.zfaw79
Anopheles hyrcanus	102.73	12.15	Unclassified	GBIF: https://doi.org/10.15468/dl.zfaw79
Anopheles hyrcanus	102.735	12.155	Unclassified	GBIF: https://doi.org/10.15468/dl.zfaw79
Anopheles hyrcanus	103.1408	5.3302	Unclassified	GBIF: https://doi.org/10.15468/dl.zfaw79
Anopheles hyrcanus	105.164167	-2.064444	Unclassified	GBIF: https://doi.org/10.15468/dl.zfaw79
Anopheles hyrcanus	106.795015	-6.145732	Unclassified	GBIF: https://doi.org/10.15468/dl.zfaw79
Anopheles hyrcanus	106.842727	-6.214125	Unclassified	GBIF: https://doi.org/10.15468/dl.zfaw79
Anopheles hyrcanus	106.845278	-6.204167	Unclassified	GBIF: https://doi.org/10.15468/dl.zfaw79
Anopheles hyrcanus	106.846655	-6.18272	Unclassified	GBIF: https://doi.org/10.15468/dl.zfaw79
Anopheles hyrcanus	106.879827	-6.197469	Unclassified	GBIF: https://doi.org/10.15468/dl.zfaw79
Anopheles hyrcanus	108.216667	-7.349722	Unclassified	GBIF: https://doi.org/10.15468/dl.zfaw79
Anopheles hyrcanus	108.510278	-6.576111	Unclassified	GBIF: https://doi.org/10.15468/dl.zfaw79
Anopheles hyrcanus	108.551667	-6.731667	Unclassified	GBIF: https://doi.org/10.15468/dl.zfaw79
Anopheles hyrcanus	109.023056	-7.697778	Unclassified	GBIF: https://doi.org/10.15468/dl.zfaw79
Anopheles hyrcanus	109.146389	-7.684283	Unclassified	GBIF: https://doi.org/10.15468/dl.zfaw79

Anopheles hyrcanus	109.173139	-7.53588	Unclassified	GBIF: https://doi.org/10.15468/dl.zfaw79
Anopheles hyrcanus	110.333	1.4167	Unclassified	GBIF: https://doi.org/10.15468/dl.zfaw79
Anopheles hyrcanus	110.818812	-7.559209	Unclassified	GBIF: https://doi.org/10.15468/dl.zfaw79
Anopheles hyrcanus	112.749444	-7.256111	Unclassified	GBIF: https://doi.org/10.15468/dl.zfaw79
Anopheles hyrcanus	115.2167	5.35	Unclassified	GBIF: https://doi.org/10.15468/dl.zfaw79
Anopheles hyrcanus	119.432222	-5.122778	Unclassified	GBIF: https://doi.org/10.15468/dl.zfaw79
Anopheles hyrcanus	119.966516	-1.170781	Unclassified	GBIF: https://doi.org/10.15468/dl.zfaw79
Anopheles hyrcanus	120.99897	14.598251	Unclassified	GBIF: https://doi.org/10.15468/dl.zfaw79
Anopheles hyrcanus	122.038574	-5.309061	Unclassified	GBIF: https://doi.org/10.15468/dl.zfaw79
Anopheles hyrcanus	124.9961	10.772604	Unclassified	GBIF: https://doi.org/10.15468/dl.zfaw79
Anopheles hyrcanus	126.383333	7.05	Unclassified	GBIF: https://doi.org/10.15468/dl.zfaw79
Anopheles hyrcanus	126.780278	-3.389444	Unclassified	GBIF: https://doi.org/10.15468/dl.zfaw79
Anopheles hyrcanus	131.222984	-4.015743	Unclassified	GBIF: https://doi.org/10.15468/dl.zfaw79
Anopheles implexus	7.44927	10.50013	Unclassified	GBIF: https://doi.org/10.15468/dl.zfaw79
Anopheles implexus	10.39932	6.16719	Unclassified	GBIF: https://doi.org/10.15468/dl.zfaw79
Anopheles implexus	11.66	-0.33	Unclassified	GBIF: https://doi.org/10.15468/dl.zfaw79
Anopheles implexus	11.70677	-0.31264	Unclassified	GBIF: https://doi.org/10.15468/dl.zfaw79
Anopheles implexus	11.71	-0.31	Unclassified	GBIF: https://doi.org/10.15468/dl.zfaw79
Anopheles implexus	32.34372	1.282173	Unclassified	GBIF: https://doi.org/10.15468/dl.zfaw79
Anopheles implexus	32.4667	0.05	Unclassified	GBIF: https://doi.org/10.15468/dl.zfaw79
Anopheles implexus	34.86667	0.24167	Unclassified	GBIF: https://doi.org/10.15468/dl.zfaw79
Anopheles indefinitus	96.6625	5.1325	Unclassified	GBIF: https://doi.org/10.15468/dl.zfaw79
Anopheles indefinitus	97.975556	4.473056	Unclassified	GBIF: https://doi.org/10.15468/dl.zfaw79
Anopheles indefinitus	99.272222	1.376944	Unclassified	GBIF: https://doi.org/10.15468/dl.zfaw79
Anopheles indefinitus	99.2725	1.378056	Unclassified	GBIF: https://doi.org/10.15468/dl.zfaw79
Anopheles indefinitus	99.338339	13.039994	Unclassified	GBIF: https://doi.org/10.15468/dl.zfaw79
Anopheles indefinitus	99.600483	12.815447	Unclassified	GBIF: https://doi.org/10.15468/dl.zfaw79
Anopheles indefinitus	99.653077	12.777445	Unclassified	GBIF: https://doi.org/10.15468/dl.zfaw79
Anopheles indefinitus	99.670083	13.218098	Unclassified	GBIF: https://doi.org/10.15468/dl.zfaw79
Anopheles indefinitus	99.744448	13.199366	Unclassified	GBIF: https://doi.org/10.15468/dl.zfaw79
Anopheles indefinitus	99.95373	18.70823	Unclassified	GBIF: https://doi.org/10.15468/dl.zfaw79
Anopheles indefinitus	100.405278	-0.465556	Unclassified	GBIF: https://doi.org/10.15468/dl.zfaw79
Anopheles indefinitus	100.85772	14.66424	Unclassified	GBIF: https://doi.org/10.15468/dl.zfaw79
Anopheles indefinitus	100.95415	14.01007	Unclassified	GBIF: https://doi.org/10.15468/dl.zfaw79
Anopheles indefinitus	103.38384	15.05659	Unclassified	GBIF: https://doi.org/10.15468/dl.zfaw79
Anopheles indefinitus	103.38386	15.05388	Unclassified	GBIF: https://doi.org/10.15468/dl.zfaw79
Anopheles indefinitus	106.78333	-6.58333	Unclassified	GBIF: https://doi.org/10.15468/dl.zfaw79
Anopheles indefinitus	106.83528	-6.151389	Unclassified	GBIF: https://doi.org/10.15468/dl.zfaw79
Anopheles indefinitus	106.835281	-6.151389	Unclassified	GBIF: https://doi.org/10.15468/dl.zfaw79
Anopheles indefinitus	110.403333	-7.261667	Unclassified	GBIF: https://doi.org/10.15468/dl.zfaw79
Anopheles indefinitus	112.631944	-7.961667	Unclassified	GBIF: https://doi.org/10.15468/dl.zfaw79
Anopheles indefinitus	118.675905	9.792584	Unclassified	GBIF: https://doi.org/10.15468/dl.zfaw79

Anopheles indefinitus	118.818458	9.952099	Unclassified	GBIF: https://doi.org/10.15468/dl.zfaw79
Anopheles indefinitus	120.554726	15.183742	Unclassified	GBIF: https://doi.org/10.15468/dl.zfaw79
Anopheles indefinitus	120.898727	23.719677	Unclassified	GBIF: https://doi.org/10.15468/dl.zfaw79
Anopheles indefinitus	121.065918	12.347433	Unclassified	GBIF: https://doi.org/10.15468/dl.zfaw79
Anopheles indefinitus	121.067613	12.352748	Unclassified	GBIF: https://doi.org/10.15468/dl.zfaw79
Anopheles indefinitus	124.674744	11.292469	Unclassified	GBIF: https://doi.org/10.15468/dl.zfaw79
Anopheles indefinitus	124.785886	7.235703	Unclassified	GBIF: https://doi.org/10.15468/dl.zfaw79
Anopheles indefinitus	124.788633	7.20028	Unclassified	GBIF: https://doi.org/10.15468/dl.zfaw79
Anopheles indefinitus	124.788633	7.237065	Unclassified	GBIF: https://doi.org/10.15468/dl.zfaw79
Anopheles indefinitus	124.850379	11.205105	Unclassified	GBIF: https://doi.org/10.15468/dl.zfaw79
Anopheles indefinitus	124.961687	11.254339	Unclassified	GBIF: https://doi.org/10.15468/dl.zfaw79
Anopheles ininii	-56.86813	2.31337	Unclassified	GBIF: https://doi.org/10.15468/dl.zfaw79
Anopheles ininii	-54.435506	4.990306	Unclassified	GBIF: https://doi.org/10.15468/dl.zfaw79
Anopheles ininii	-54.415345	4.619739	Unclassified	GBIF: https://doi.org/10.15468/dl.zfaw79
Anopheles ininii	-54.41527	4.618084	Unclassified	GBIF: https://doi.org/10.15468/dl.zfaw79
Anopheles ininii	-54.383048	4.27418	Unclassified	GBIF: https://doi.org/10.15468/dl.zfaw79
Anopheles ininii	-53.66711	-3.20039	Unclassified	GBIF: https://doi.org/10.15468/dl.zfaw79
Anopheles ininii	-53.63378	-3.20039	Unclassified	GBIF: https://doi.org/10.15468/dl.zfaw79
Anopheles ininii	-53.55044	-3.20039	Unclassified	GBIF: https://doi.org/10.15468/dl.zfaw79
Anopheles ininii	-53.549917	3.749968	Unclassified	GBIF: https://doi.org/10.15468/dl.zfaw79
Anopheles ininii	-53.288005	4.825571	Unclassified	GBIF: https://doi.org/10.15468/dl.zfaw79
Anopheles ininii	-53.24036	3.91451	Unclassified	GBIF: https://doi.org/10.15468/dl.zfaw79
Anopheles ininii	-53.23477	3.92404	Unclassified	GBIF: https://doi.org/10.15468/dl.zfaw79
Anopheles ininii	-53.207695	3.594195	Unclassified	GBIF: https://doi.org/10.15468/dl.zfaw79
Anopheles ininii	-53.20636	3.628085	Unclassified	GBIF: https://doi.org/10.15468/dl.zfaw79
Anopheles ininii	-53.02915	5.01314	Unclassified	GBIF: https://doi.org/10.15468/dl.zfaw79
Anopheles ininii	-52.700025	4.033351	Unclassified	GBIF: https://doi.org/10.15468/dl.zfaw79
Anopheles ininii	-52.47665	4.567727	Unclassified	GBIF: https://doi.org/10.15468/dl.zfaw79
Anopheles ininii	-52.473185	4.57464	Unclassified	GBIF: https://doi.org/10.15468/dl.zfaw79
Anopheles ininii	-52.46114	4.576432	Unclassified	GBIF: https://doi.org/10.15468/dl.zfaw79
Anopheles ininii	-52.413525	4.563701	Unclassified	GBIF: https://doi.org/10.15468/dl.zfaw79
Anopheles ininii	-52.333605	3.167747	Unclassified	GBIF: https://doi.org/10.15468/dl.zfaw79
Anopheles ininii	-51.88333	3.8	Unclassified	GBIF: https://doi.org/10.15468/dl.zfaw79
Anopheles ininii	-51.815192	3.875351	Unclassified	GBIF: https://doi.org/10.15468/dl.zfaw79
Anopheles ininii	-51.805914	3.898851	Unclassified	GBIF: https://doi.org/10.15468/dl.zfaw79
Anopheles ininii	-51.801158	3.888398	Unclassified	GBIF: https://doi.org/10.15468/dl.zfaw79
Anopheles ininii	-49.53376	-4.53373	Unclassified	GBIF: https://doi.org/10.15468/dl.zfaw79
Anopheles ininii	-49.11709	-5.3504	Unclassified	GBIF: https://doi.org/10.15468/dl.zfaw79
Anopheles ininii	-48.40336	-1.44613	Unclassified	GBIF: https://doi.org/10.15468/dl.zfaw79
Anopheles insulaeflorum	75.9	11.74	Unclassified	GBIF: https://doi.org/10.15468/dl.zfaw79
Anopheles insulaeflorum	98.82057	14.40306	Unclassified	GBIF: https://doi.org/10.15468/dl.zfaw79
Anopheles insulaeflorum	98.86693	14.43926	Unclassified	GBIF: https://doi.org/10.15468/dl.zfaw79

Anopheles insulaeflorum	99.89454	18.99228	Unclassified	GBIF: https://doi.org/10.15468/dl.zfaw79
Anopheles insulaeflorum	99.93681	18.90712	Unclassified	GBIF: https://doi.org/10.15468/dl.zfaw79
Anopheles insulaeflorum	100.4927	18.42009	Unclassified	GBIF: https://doi.org/10.15468/dl.zfaw79
Anopheles insulaeflorum	100.79171	18.95053	Unclassified	GBIF: https://doi.org/10.15468/dl.zfaw79
Anopheles insulaeflorum	101.82418	14.32755	Unclassified	GBIF: https://doi.org/10.15468/dl.zfaw79
Anopheles insulaeflorum	102.19879	12.95946	Unclassified	GBIF: https://doi.org/10.15468/dl.zfaw79
Anopheles insulaeflorum	102.2034	12.95951	Unclassified	GBIF: https://doi.org/10.15468/dl.zfaw79
Anopheles insulaeflorum	121.067613	12.352748	Unclassified	GBIF: https://doi.org/10.15468/dl.zfaw79
Anopheles insulaeflorum	128.19	-3.058889	Unclassified	GBIF: https://doi.org/10.15468/dl.zfaw79
Anopheles intermedius	-58.30039	5.96627	Unclassified	GBIF: https://doi.org/10.15468/dl.zfaw79
Anopheles intermedius	-58.1682	6.79681	Unclassified	GBIF: https://doi.org/10.15468/dl.zfaw79
Anopheles intermedius	-54.415345	4.619739	Unclassified	GBIF: https://doi.org/10.15468/dl.zfaw79
Anopheles intermedius	-54.383048	4.27418	Unclassified	GBIF: https://doi.org/10.15468/dl.zfaw79
Anopheles intermedius	-54.192245	3.826958	Unclassified	GBIF: https://doi.org/10.15468/dl.zfaw79
Anopheles intermedius	-54.151439	3.80651	Unclassified	GBIF: https://doi.org/10.15468/dl.zfaw79
Anopheles intermedius	-54.148707	3.807339	Unclassified	GBIF: https://doi.org/10.15468/dl.zfaw79
Anopheles intermedius	-54.141692	3.808351	Unclassified	GBIF: https://doi.org/10.15468/dl.zfaw79
Anopheles intermedius	-54.070388	3.298274	Unclassified	GBIF: https://doi.org/10.15468/dl.zfaw79
Anopheles intermedius	-54.053915	3.383908	Unclassified	GBIF: https://doi.org/10.15468/dl.zfaw79
Anopheles intermedius	-54.050025	3.383348	Unclassified	GBIF: https://doi.org/10.15468/dl.zfaw79
Anopheles intermedius	-54.046695	3.635018	Unclassified	GBIF: https://doi.org/10.15468/dl.zfaw79
Anopheles intermedius	-54.033355	3.366688	Unclassified	GBIF: https://doi.org/10.15468/dl.zfaw79
Anopheles intermedius	-54.031415	3.643348	Unclassified	GBIF: https://doi.org/10.15468/dl.zfaw79
Anopheles intermedius	-54.000025	4.766684	Unclassified	GBIF: https://doi.org/10.15468/dl.zfaw79
Anopheles intermedius	-53.916695	3.383348	Unclassified	GBIF: https://doi.org/10.15468/dl.zfaw79
Anopheles intermedius	-53.549917	3.749968	Unclassified	GBIF: https://doi.org/10.15468/dl.zfaw79
Anopheles intermedius	-53.24036	3.91451	Unclassified	GBIF: https://doi.org/10.15468/dl.zfaw79
Anopheles intermedius	-53.23477	3.92404	Unclassified	GBIF: https://doi.org/10.15468/dl.zfaw79
Anopheles intermedius	-53.20944	3.623127	Unclassified	GBIF: https://doi.org/10.15468/dl.zfaw79
Anopheles intermedius	-52.900025	5.166684	Unclassified	GBIF: https://doi.org/10.15468/dl.zfaw79
Anopheles intermedius	-52.700025	4.033351	Unclassified	GBIF: https://doi.org/10.15468/dl.zfaw79
Anopheles intermedius	-52.66885	5.15907	Unclassified	GBIF: https://doi.org/10.15468/dl.zfaw79
Anopheles intermedius	-52.62876	4.02668	Unclassified	GBIF: https://doi.org/10.15468/dl.zfaw79
Anopheles intermedius	-52.593636	3.256962	Unclassified	GBIF: https://doi.org/10.15468/dl.zfaw79
Anopheles intermedius	-52.47665	4.567727	Unclassified	GBIF: https://doi.org/10.15468/dl.zfaw79
Anopheles intermedius	-52.476399	4.568433	Unclassified	GBIF: https://doi.org/10.15468/dl.zfaw79
Anopheles intermedius	-52.473699	4.574534	Unclassified	GBIF: https://doi.org/10.15468/dl.zfaw79
Anopheles intermedius	-52.47329	4.550868	Unclassified	GBIF: https://doi.org/10.15468/dl.zfaw79
Anopheles intermedius	-52.473191	4.550624	Unclassified	GBIF: https://doi.org/10.15468/dl.zfaw79
Anopheles intermedius	-52.473106	4.549104	Unclassified	GBIF: https://doi.org/10.15468/dl.zfaw79
Anopheles intermedius	-52.470895	4.574205	Unclassified	GBIF: https://doi.org/10.15468/dl.zfaw79
Anopheles intermedius	-52.47027	4.572008	Unclassified	GBIF: https://doi.org/10.15468/dl.zfaw79

Anopheles intermedius	-52.469703	4.564142	Unclassified	GBIF: https://doi.org/10.15468/dl.zfaw79
Anopheles intermedius	-52.468501	4.571289	Unclassified	GBIF: https://doi.org/10.15468/dl.zfaw79
Anopheles intermedius	-52.463631	4.560528	Unclassified	GBIF: https://doi.org/10.15468/dl.zfaw79
Anopheles intermedius	-52.462674	4.559987	Unclassified	GBIF: https://doi.org/10.15468/dl.zfaw79
Anopheles intermedius	-52.46114	4.576432	Unclassified	GBIF: https://doi.org/10.15468/dl.zfaw79
Anopheles intermedius	-52.45662	4.792945	Unclassified	GBIF: https://doi.org/10.15468/dl.zfaw79
Anopheles intermedius	-52.43459	4.7801	Unclassified	GBIF: https://doi.org/10.15468/dl.zfaw79
Anopheles intermedius	-52.434423	4.780484	Unclassified	GBIF: https://doi.org/10.15468/dl.zfaw79
Anopheles intermedius	-52.357135	4.886868	Unclassified	GBIF: https://doi.org/10.15468/dl.zfaw79
Anopheles intermedius	-52.34352	4.77908	Unclassified	GBIF: https://doi.org/10.15468/dl.zfaw79
Anopheles intermedius	-52.334095	3.160908	Unclassified	GBIF: https://doi.org/10.15468/dl.zfaw79
Anopheles intermedius	-52.333605	3.167747	Unclassified	GBIF: https://doi.org/10.15468/dl.zfaw79
Anopheles intermedius	-52.330987	4.850608	Unclassified	GBIF: https://doi.org/10.15468/dl.zfaw79
Anopheles intermedius	-52.329786	3.170172	Unclassified	GBIF: https://doi.org/10.15468/dl.zfaw79
Anopheles intermedius	-52.323296	4.936976	Unclassified	GBIF: https://doi.org/10.15468/dl.zfaw79
Anopheles intermedius	-52.312455	4.927798	Unclassified	GBIF: https://doi.org/10.15468/dl.zfaw79
Anopheles intermedius	-52.291065	4.890268	Unclassified	GBIF: https://doi.org/10.15468/dl.zfaw79
Anopheles intermedius	-52.276221	4.886853	Unclassified	GBIF: https://doi.org/10.15468/dl.zfaw79
Anopheles intermedius	-52.037094	4.486403	Unclassified	GBIF: https://doi.org/10.15468/dl.zfaw79
Anopheles intermedius	-51.79697	3.913685	Unclassified	GBIF: https://doi.org/10.15468/dl.zfaw79
Anopheles intermedius	-51.677335	4.039038	Unclassified	GBIF: https://doi.org/10.15468/dl.zfaw79
Anopheles intermedius	-48.40336	-1.44613	Unclassified	GBIF: https://doi.org/10.15468/dl.zfaw79
Anopheles interruptus	98.425492	19.365813	Unclassified	GBIF: https://doi.org/10.15468/dl.zfaw79
Anopheles interruptus	101.77356	5.94562	Unclassified	GBIF: https://doi.org/10.15468/dl.zfaw79
Anopheles introlatus	100.01919	6.87593	Unclassified	GBIF: https://doi.org/10.15468/dl.zfaw79
Anopheles introlatus	101.36853	6.2595	Unclassified	GBIF: https://doi.org/10.15468/dl.zfaw79
Anopheles introlatus	102.735	12.155	Unclassified	GBIF: https://doi.org/10.15468/dl.zfaw79
Anopheles introlatus	105.539903	15.572432	Unclassified	GBIF: https://doi.org/10.15468/dl.zfaw79
Anopheles irenicus	159.83	-9.37	Unclassified	GBIF: https://doi.org/10.15468/dl.zfaw79
Anopheles irenicus	159.88	-9.38	Unclassified	GBIF: https://doi.org/10.15468/dl.zfaw79
Anopheles jamesii	81.277411	28.635302	Unclassified	GBIF: https://doi.org/10.15468/dl.zfaw79
Anopheles jamesii	85.002554	27.296121	Unclassified	GBIF: https://doi.org/10.15468/dl.zfaw79
Anopheles jamesii	92.66	11.74	Unclassified	GBIF: https://doi.org/10.15468/dl.zfaw79
Anopheles jamesii	97.91318	17.570554	Unclassified	GBIF: https://doi.org/10.15468/dl.zfaw79
Anopheles jamesii	98.036012	17.506116	Unclassified	GBIF: https://doi.org/10.15468/dl.zfaw79
Anopheles jamesii	98.062728	17.439362	Unclassified	GBIF: https://doi.org/10.15468/dl.zfaw79
Anopheles jamesii	98.114246	18.141096	Unclassified	GBIF: https://doi.org/10.15468/dl.zfaw79
Anopheles jamesii	98.249585	8.587786	Unclassified	GBIF: https://doi.org/10.15468/dl.zfaw79
Anopheles jamesii	98.256188	17.311012	Unclassified	GBIF: https://doi.org/10.15468/dl.zfaw79
Anopheles jamesii	98.391201	8.333326	Unclassified	GBIF: https://doi.org/10.15468/dl.zfaw79
Anopheles jamesii	98.458192	9.843434	Unclassified	GBIF: https://doi.org/10.15468/dl.zfaw79
Anopheles jamesii	98.492807	9.451781	Unclassified	GBIF: https://doi.org/10.15468/dl.zfaw79

Anopheles jamesii	98.564605	14.636853	Unclassified	GBIF: https://doi.org/10.15468/dl.zfaw79
Anopheles jamesii	98.585361	9.514689	Unclassified	GBIF: https://doi.org/10.15468/dl.zfaw79
Anopheles jamesii	98.653801	16.033791	Unclassified	GBIF: https://doi.org/10.15468/dl.zfaw79
Anopheles jamesii	98.656019	16.371895	Unclassified	GBIF: https://doi.org/10.15468/dl.zfaw79
Anopheles jamesii	98.65907	8.29664	Unclassified	GBIF: https://doi.org/10.15468/dl.zfaw79
Anopheles jamesii	98.659846	8.533548	Unclassified	GBIF: https://doi.org/10.15468/dl.zfaw79
Anopheles jamesii	98.668094	16.42977	Unclassified	GBIF: https://doi.org/10.15468/dl.zfaw79
Anopheles jamesii	98.703903	16.286991	Unclassified	GBIF: https://doi.org/10.15468/dl.zfaw79
Anopheles jamesii	98.873906	10.718134	Unclassified	GBIF: https://doi.org/10.15468/dl.zfaw79
Anopheles jamesii	98.9533	9.03	Unclassified	GBIF: https://doi.org/10.15468/dl.zfaw79
Anopheles jamesii	99.173763	13.987069	Unclassified	GBIF: https://doi.org/10.15468/dl.zfaw79
Anopheles jamesii	99.278195	10.798224	Unclassified	GBIF: https://doi.org/10.15468/dl.zfaw79
Anopheles jamesii	99.64971	13.232429	Unclassified	GBIF: https://doi.org/10.15468/dl.zfaw79
Anopheles jamesii	100.72826	14.61101	Unclassified	GBIF: https://doi.org/10.15468/dl.zfaw79
Anopheles jamesii	100.85772	14.66424	Unclassified	GBIF: https://doi.org/10.15468/dl.zfaw79
Anopheles jamesii	100.89131	14.68476	Unclassified	GBIF: https://doi.org/10.15468/dl.zfaw79
Anopheles jamesii	100.946318	17.74737	Unclassified	GBIF: https://doi.org/10.15468/dl.zfaw79
Anopheles jamesii	100.991387	13.253259	Unclassified	GBIF: https://doi.org/10.15468/dl.zfaw79
Anopheles jamesii	101.0847	12.8573	Unclassified	GBIF: https://doi.org/10.15468/dl.zfaw79
Anopheles jamesii	101.104708	13.235179	Unclassified	GBIF: https://doi.org/10.15468/dl.zfaw79
Anopheles jamesii	101.501371	13.239733	Unclassified	GBIF: https://doi.org/10.15468/dl.zfaw79
Anopheles jamesii	101.569413	13.04301	Unclassified	GBIF: https://doi.org/10.15468/dl.zfaw79
Anopheles jamesii	101.67662	13.072638	Unclassified	GBIF: https://doi.org/10.15468/dl.zfaw79
Anopheles jamesii	101.742215	13.41702	Unclassified	GBIF: https://doi.org/10.15468/dl.zfaw79
Anopheles jamesii	101.742333	13.015038	Unclassified	GBIF: https://doi.org/10.15468/dl.zfaw79
Anopheles jamesii	101.879012	13.063376	Unclassified	GBIF: https://doi.org/10.15468/dl.zfaw79
Anopheles jamesii	101.907779	13.227123	Unclassified	GBIF: https://doi.org/10.15468/dl.zfaw79
Anopheles jamesii	102.04314	12.48356	Unclassified	GBIF: https://doi.org/10.15468/dl.zfaw79
Anopheles jamesii	102.069882	12.964291	Unclassified	GBIF: https://doi.org/10.15468/dl.zfaw79
Anopheles jamesii	102.207242	13.116654	Unclassified	GBIF: https://doi.org/10.15468/dl.zfaw79
Anopheles jamesii	102.26383	12.817941	Unclassified	GBIF: https://doi.org/10.15468/dl.zfaw79
Anopheles jamesii	102.269907	12.864386	Unclassified	GBIF: https://doi.org/10.15468/dl.zfaw79
Anopheles jamesii	102.279299	12.7615	Unclassified	GBIF: https://doi.org/10.15468/dl.zfaw79
Anopheles jamesii	102.34776	11.989918	Unclassified	GBIF: https://doi.org/10.15468/dl.zfaw79
Anopheles jamesii	102.35176	16.72826	Unclassified	GBIF: https://doi.org/10.15468/dl.zfaw79
Anopheles jamesii	102.365687	12.684681	Unclassified	GBIF: https://doi.org/10.15468/dl.zfaw79
Anopheles jamesii	102.381435	12.215942	Unclassified	GBIF: https://doi.org/10.15468/dl.zfaw79
Anopheles jamesii	102.384203	12.026239	Unclassified	GBIF: https://doi.org/10.15468/dl.zfaw79
Anopheles jamesii	102.392028	12.204587	Unclassified	GBIF: https://doi.org/10.15468/dl.zfaw79
Anopheles jamesii	102.393685	11.994705	Unclassified	GBIF: https://doi.org/10.15468/dl.zfaw79
Anopheles jamesii	102.421872	12.275075	Unclassified	GBIF: https://doi.org/10.15468/dl.zfaw79
Anopheles jamesii	102.462267	12.595272	Unclassified	GBIF: https://doi.org/10.15468/dl.zfaw79

Anopheles jamesii	102.472119	12.62247	Unclassified	GBIF: https://doi.org/10.15468/dl.zfaw79
Anopheles jamesii	102.48322	12.616251	Unclassified	GBIF: https://doi.org/10.15468/dl.zfaw79
Anopheles jamesii	102.492493	11.823558	Unclassified	GBIF: https://doi.org/10.15468/dl.zfaw79
Anopheles jamesii	102.530348	12.400742	Unclassified	GBIF: https://doi.org/10.15468/dl.zfaw79
Anopheles jamesii	102.545352	11.713025	Unclassified	GBIF: https://doi.org/10.15468/dl.zfaw79
Anopheles jamesii	102.550476	12.606033	Unclassified	GBIF: https://doi.org/10.15468/dl.zfaw79
Anopheles jamesii	102.564458	12.390208	Unclassified	GBIF: https://doi.org/10.15468/dl.zfaw79
Anopheles jamesii	102.578558	11.67832	Unclassified	GBIF: https://doi.org/10.15468/dl.zfaw79
Anopheles jamesii	102.580471	12.546671	Unclassified	GBIF: https://doi.org/10.15468/dl.zfaw79
Anopheles jamesii	102.589953	12.516034	Unclassified	GBIF: https://doi.org/10.15468/dl.zfaw79
Anopheles jamesii	102.618292	12.230746	Unclassified	GBIF: https://doi.org/10.15468/dl.zfaw79
Anopheles jamesii	102.629235	12.444094	Unclassified	GBIF: https://doi.org/10.15468/dl.zfaw79
Anopheles jamesii	102.632075	12.435083	Unclassified	GBIF: https://doi.org/10.15468/dl.zfaw79
Anopheles jamesii	102.6369	12.409824	Unclassified	GBIF: https://doi.org/10.15468/dl.zfaw79
Anopheles jamesii	102.680198	12.474401	Unclassified	GBIF: https://doi.org/10.15468/dl.zfaw79
Anopheles jamesii	102.692968	12.387497	Unclassified	GBIF: https://doi.org/10.15468/dl.zfaw79
Anopheles jamesii	102.73	12.15	Unclassified	GBIF: https://doi.org/10.15468/dl.zfaw79
Anopheles jamesii	102.778824	11.926632	Unclassified	GBIF: https://doi.org/10.15468/dl.zfaw79
Anopheles jamesii	102.817793	11.873617	Unclassified	GBIF: https://doi.org/10.15468/dl.zfaw79
Anopheles jamesii	102.829025	11.845686	Unclassified	GBIF: https://doi.org/10.15468/dl.zfaw79
Anopheles jamesii	103.128814	14.371502	Unclassified	GBIF: https://doi.org/10.15468/dl.zfaw79
Anopheles jamesii	103.233552	16.935962	Unclassified	GBIF: https://doi.org/10.15468/dl.zfaw79
Anopheles jamesii	103.444973	14.449552	Unclassified	GBIF: https://doi.org/10.15468/dl.zfaw79
Anopheles jamesii	103.847257	14.480587	Unclassified	GBIF: https://doi.org/10.15468/dl.zfaw79
Anopheles jamesii	104.208742	14.503051	Unclassified	GBIF: https://doi.org/10.15468/dl.zfaw79
Anopheles jamesii	104.363153	14.530674	Unclassified	GBIF: https://doi.org/10.15468/dl.zfaw79
Anopheles jamesii	104.369222	14.519333	Unclassified	GBIF: https://doi.org/10.15468/dl.zfaw79
Anopheles jamesii	104.369222	14.521444	Unclassified	GBIF: https://doi.org/10.15468/dl.zfaw79
Anopheles jamesii	104.369639	14.520556	Unclassified	GBIF: https://doi.org/10.15468/dl.zfaw79
Anopheles jamesii	104.375223	14.528899	Unclassified	GBIF: https://doi.org/10.15468/dl.zfaw79
Anopheles jamesii	104.542243	16.818064	Unclassified	GBIF: https://doi.org/10.15468/dl.zfaw79
Anopheles jamesii	104.69114	38.073254	Unclassified	GBIF: https://doi.org/10.15468/dl.zfaw79
Anopheles jamesii	104.769864	16.22415	Unclassified	GBIF: https://doi.org/10.15468/dl.zfaw79
Anopheles jamesii	104.844444	14.473205	Unclassified	GBIF: https://doi.org/10.15468/dl.zfaw79
Anopheles jamesii	104.92	13.77	Unclassified	GBIF: https://doi.org/10.15468/dl.zfaw79
Anopheles jamesii	105.137188	14.552966	Unclassified	GBIF: https://doi.org/10.15468/dl.zfaw79
Anopheles jamesii	105.311586	15.697635	Unclassified	GBIF: https://doi.org/10.15468/dl.zfaw79
Anopheles jamesii	105.506659	15.695174	Unclassified	GBIF: https://doi.org/10.15468/dl.zfaw79
Anopheles janconnae	-61.233	1.829	Unclassified	GBIF: https://doi.org/10.15468/dl.zfaw79
Anopheles janconnae	-61.23	1.83	Unclassified	GBIF: https://doi.org/10.15468/dl.zfaw79
Anopheles janconnae	-61.224	1.744	Unclassified	GBIF: https://doi.org/10.15468/dl.zfaw79
Anopheles janconnae	-61.22	1.74	Unclassified	GBIF: https://doi.org/10.15468/dl.zfaw79

Anopheles janconnae	-61.152	1.757	Unclassified	GBIF: https://doi.org/10.15468/dl.zfaw79
Anopheles janconnae	-61.15	1.76	Unclassified	GBIF: https://doi.org/10.15468/dl.zfaw79
Anopheles janconnae	-61.12	2.27	Unclassified	GBIF: https://doi.org/10.15468/dl.zfaw79
Anopheles janconnae	-61.116	2.266	Unclassified	GBIF: https://doi.org/10.15468/dl.zfaw79
Anopheles janconnae	-61	3.77	Unclassified	GBIF: https://doi.org/10.15468/dl.zfaw79
Anopheles janconnae	-60.996	3.771	Unclassified	GBIF: https://doi.org/10.15468/dl.zfaw79
Anopheles janconnae	-60.88	2.59	Unclassified	GBIF: https://doi.org/10.15468/dl.zfaw79
Anopheles janconnae	-60.877	2.586	Unclassified	GBIF: https://doi.org/10.15468/dl.zfaw79
Anopheles janconnae	-60.851	3.283	Unclassified	GBIF: https://doi.org/10.15468/dl.zfaw79
Anopheles janconnae	-60.85	3.28	Unclassified	GBIF: https://doi.org/10.15468/dl.zfaw79
Anopheles janconnae	-60.83	3.12	Unclassified	GBIF: https://doi.org/10.15468/dl.zfaw79
Anopheles janconnae	-60.829	3.123	Unclassified	GBIF: https://doi.org/10.15468/dl.zfaw79
Anopheles janconnae	-60.782	2.893	Unclassified	GBIF: https://doi.org/10.15468/dl.zfaw79
Anopheles janconnae	-60.78	2.89	Unclassified	GBIF: https://doi.org/10.15468/dl.zfaw79
Anopheles janconnae	-60.776	3.021	Unclassified	GBIF: https://doi.org/10.15468/dl.zfaw79
Anopheles janconnae	-60.742	2.648	Unclassified	GBIF: https://doi.org/10.15468/dl.zfaw79
Anopheles janconnae	-60.74	2.65	Unclassified	GBIF: https://doi.org/10.15468/dl.zfaw79
Anopheles janconnae	-60.71	2.78	Unclassified	GBIF: https://doi.org/10.15468/dl.zfaw79
Anopheles janconnae	-60.71	2.85	Unclassified	GBIF: https://doi.org/10.15468/dl.zfaw79
Anopheles janconnae	-60.709	2.778	Unclassified	GBIF: https://doi.org/10.15468/dl.zfaw79
Anopheles janconnae	-60.707	2.848	Unclassified	GBIF: https://doi.org/10.15468/dl.zfaw79
Anopheles janconnae	-60.705	2.758	Unclassified	GBIF: https://doi.org/10.15468/dl.zfaw79
Anopheles janconnae	-60.7	2.8	Unclassified	GBIF: https://doi.org/10.15468/dl.zfaw79
Anopheles janconnae	-60.7	2.76	Unclassified	GBIF: https://doi.org/10.15468/dl.zfaw79
Anopheles janconnae	-60.696	2.8	Unclassified	GBIF: https://doi.org/10.15468/dl.zfaw79
Anopheles janconnae	-60.67	0.25	Unclassified	GBIF: https://doi.org/10.15468/dl.zfaw79
Anopheles janconnae	-60.669	0.246	Unclassified	GBIF: https://doi.org/10.15468/dl.zfaw79
Anopheles janconnae	-60.66	2.792	Unclassified	GBIF: https://doi.org/10.15468/dl.zfaw79
Anopheles janconnae	-60.66	2.79	Unclassified	GBIF: https://doi.org/10.15468/dl.zfaw79
Anopheles janconnae	-60.65	2.85	Unclassified	GBIF: https://doi.org/10.15468/dl.zfaw79
Anopheles janconnae	-60.646	2.854	Unclassified	GBIF: https://doi.org/10.15468/dl.zfaw79
Anopheles janconnae	-60.64	2.794	Unclassified	GBIF: https://doi.org/10.15468/dl.zfaw79
Anopheles janconnae	-60.64	2.79	Unclassified	GBIF: https://doi.org/10.15468/dl.zfaw79
Anopheles janconnae	-60.635	2.793	Unclassified	GBIF: https://doi.org/10.15468/dl.zfaw79
Anopheles janconnae	-60.63	2.79	Unclassified	GBIF: https://doi.org/10.15468/dl.zfaw79
Anopheles janconnae	-60.44	1.19	Unclassified	GBIF: https://doi.org/10.15468/dl.zfaw79
Anopheles janconnae	-60.436	1.185	Unclassified	GBIF: https://doi.org/10.15468/dl.zfaw79
Anopheles janconnae	-60.404	0.985	Unclassified	GBIF: https://doi.org/10.15468/dl.zfaw79
Anopheles janconnae	-60.4	0.98	Unclassified	GBIF: https://doi.org/10.15468/dl.zfaw79
Anopheles janconnae	-60.39	1.053	Unclassified	GBIF: https://doi.org/10.15468/dl.zfaw79
Anopheles janconnae	-60.39	1.05	Unclassified	GBIF: https://doi.org/10.15468/dl.zfaw79
Anopheles janconnae	-54.752	-2.434	Unclassified	GBIF: https://doi.org/10.15468/dl.zfaw79

Anopheles janconnae	-54.75	-2.43	Unclassified	GBIF: https://doi.org/10.15468/dl.zfaw79
Anopheles janconnae	-51.07	0.03	Unclassified	GBIF: https://doi.org/10.15468/dl.zfaw79
Anopheles janconnae	-51.066	0.034	Unclassified	GBIF: https://doi.org/10.15468/dl.zfaw79
Anopheles jebudensis	3.33254	6.58381	Unclassified	GBIF: https://doi.org/10.15468/dl.zfaw79
Anopheles jebudensis	3.93255	6.81712	Unclassified	GBIF: https://doi.org/10.15468/dl.zfaw79
Anopheles jebudensis	12.96	-1.76	Unclassified	GBIF: https://doi.org/10.15468/dl.zfaw79
Anopheles jebudensis	13	-1.82	Unclassified	GBIF: https://doi.org/10.15468/dl.zfaw79
Anopheles jeyporiensis	83.985567	28.209583	Unclassified	GBIF: https://doi.org/10.15468/dl.zfaw79
Anopheles jeyporiensis	85.570255	27.704429	Unclassified	GBIF: https://doi.org/10.15468/dl.zfaw79
Anopheles jeyporiensis	92.26	21.8	Unclassified	GBIF: https://doi.org/10.15468/dl.zfaw79
Anopheles jeyporiensis	92.2601	21.8004	Unclassified	GBIF: https://doi.org/10.15468/dl.zfaw79
Anopheles jeyporiensis	97.96386	18.55778	Unclassified	GBIF: https://doi.org/10.15468/dl.zfaw79
Anopheles jeyporiensis	98.0871	18.54576	Unclassified	GBIF: https://doi.org/10.15468/dl.zfaw79
Anopheles jeyporiensis	98.844326	16.04819	Unclassified	GBIF: https://doi.org/10.15468/dl.zfaw79
Anopheles jeyporiensis	98.90558	18.91036	Unclassified	GBIF: https://doi.org/10.15468/dl.zfaw79
Anopheles jeyporiensis	98.96256	18.87514	Unclassified	GBIF: https://doi.org/10.15468/dl.zfaw79
Anopheles jeyporiensis	99.163427	10.537782	Unclassified	GBIF: https://doi.org/10.15468/dl.zfaw79
Anopheles jeyporiensis	104.69114	38.073254	Unclassified	GBIF: https://doi.org/10.15468/dl.zfaw79
Anopheles jeyporiensis	105.91339	16.517347	Unclassified	GBIF: https://doi.org/10.15468/dl.zfaw79
Anopheles judithae	-110.856389	30.778056	Unclassified	GBIF: https://doi.org/10.15468/dl.zfaw79
Anopheles karwari	75.9	11.74	Unclassified	GBIF: https://doi.org/10.15468/dl.zfaw79
Anopheles karwari	85.002554	27.296121	Unclassified	GBIF: https://doi.org/10.15468/dl.zfaw79
Anopheles karwari	85.570255	27.704429	Unclassified	GBIF: https://doi.org/10.15468/dl.zfaw79
Anopheles karwari	97.900708	17.607539	Unclassified	GBIF: https://doi.org/10.15468/dl.zfaw79
Anopheles karwari	98.279913	8.599055	Unclassified	GBIF: https://doi.org/10.15468/dl.zfaw79
Anopheles karwari	98.342768	8.511432	Unclassified	GBIF: https://doi.org/10.15468/dl.zfaw79
Anopheles karwari	98.785	1.736667	Unclassified	GBIF: https://doi.org/10.15468/dl.zfaw79
Anopheles karwari	98.96256	18.87695	Unclassified	GBIF: https://doi.org/10.15468/dl.zfaw79
Anopheles karwari	99.077778	1.559722	Unclassified	GBIF: https://doi.org/10.15468/dl.zfaw79
Anopheles karwari	99.316465	16.600952	Unclassified	GBIF: https://doi.org/10.15468/dl.zfaw79
Anopheles karwari	99.469207	7.674829	Unclassified	GBIF: https://doi.org/10.15468/dl.zfaw79
Anopheles karwari	100.129167	0.186111	Unclassified	GBIF: https://doi.org/10.15468/dl.zfaw79
Anopheles karwari	100.85772	14.66424	Unclassified	GBIF: https://doi.org/10.15468/dl.zfaw79
Anopheles karwari	100.95415	14.01007	Unclassified	GBIF: https://doi.org/10.15468/dl.zfaw79
Anopheles karwari	101.50725	14.60882	Unclassified	GBIF: https://doi.org/10.15468/dl.zfaw79
Anopheles karwari	101.89313	14.28608	Unclassified	GBIF: https://doi.org/10.15468/dl.zfaw79
Anopheles karwari	102.03245	12.6144	Unclassified	GBIF: https://doi.org/10.15468/dl.zfaw79
Anopheles karwari	102.05249	12.47011	Unclassified	GBIF: https://doi.org/10.15468/dl.zfaw79
Anopheles karwari	102.207242	13.116654	Unclassified	GBIF: https://doi.org/10.15468/dl.zfaw79
Anopheles karwari	102.943857	14.279438	Unclassified	GBIF: https://doi.org/10.15468/dl.zfaw79
Anopheles karwari	103.81026	1.352825	Unclassified	GBIF: https://doi.org/10.15468/dl.zfaw79
Anopheles karwari	104.063014	14.433857	Unclassified	GBIF: https://doi.org/10.15468/dl.zfaw79

Anopheles karwari	104.208742	14.503051	Unclassified	GBIF: https://doi.org/10.15468/dl.zfaw79
Anopheles karwari	104.211418	14.506937	Unclassified	GBIF: https://doi.org/10.15468/dl.zfaw79
Anopheles karwari	104.22271	14.615236	Unclassified	GBIF: https://doi.org/10.15468/dl.zfaw79
Anopheles karwari	104.312034	14.554037	Unclassified	GBIF: https://doi.org/10.15468/dl.zfaw79
Anopheles karwari	104.320394	16.718235	Unclassified	GBIF: https://doi.org/10.15468/dl.zfaw79
Anopheles karwari	104.363153	14.530674	Unclassified	GBIF: https://doi.org/10.15468/dl.zfaw79
Anopheles karwari	104.375223	14.528899	Unclassified	GBIF: https://doi.org/10.15468/dl.zfaw79
Anopheles karwari	104.691789	14.480704	Unclassified	GBIF: https://doi.org/10.15468/dl.zfaw79
Anopheles karwari	104.844444	14.473205	Unclassified	GBIF: https://doi.org/10.15468/dl.zfaw79
Anopheles karwari	104.991524	14.483618	Unclassified	GBIF: https://doi.org/10.15468/dl.zfaw79
Anopheles karwari	105.137874	14.427188	Unclassified	GBIF: https://doi.org/10.15468/dl.zfaw79
Anopheles karwari	105.311586	15.697635	Unclassified	GBIF: https://doi.org/10.15468/dl.zfaw79
Anopheles karwari	105.36191	15.656875	Unclassified	GBIF: https://doi.org/10.15468/dl.zfaw79
Anopheles karwari	105.374034	14.591819	Unclassified	GBIF: https://doi.org/10.15468/dl.zfaw79
Anopheles karwari	105.506659	15.695174	Unclassified	GBIF: https://doi.org/10.15468/dl.zfaw79
Anopheles karwari	107.948889	-2.841944	Unclassified	GBIF: https://doi.org/10.15468/dl.zfaw79
Anopheles karwari	110.333	1.4167	Unclassified	GBIF: https://doi.org/10.15468/dl.zfaw79
Anopheles karwari	115.2167	5.35	Unclassified	GBIF: https://doi.org/10.15468/dl.zfaw79
Anopheles karwari	119.432222	-5.122778	Unclassified	GBIF: https://doi.org/10.15468/dl.zfaw79
Anopheles karwari	119.533333	-3.066667	Unclassified	GBIF: https://doi.org/10.15468/dl.zfaw79
Anopheles karwari	119.62	-2.37	Unclassified	GBIF: https://doi.org/10.15468/dl.zfaw79
Anopheles karwari	140.669012	-2.591744	Unclassified	GBIF: https://doi.org/10.15468/dl.zfaw79
Anopheles karwari	143.05	-3.63333	Unclassified	GBIF: https://doi.org/10.15468/dl.zfaw79
Anopheles keniensis	37.684796	-3.400394	Unclassified	GBIF: https://doi.org/10.15468/dl.zfaw79
Anopheles kleini	126.63758	37.76688	Unclassified	GBIF: https://doi.org/10.15468/dl.zfaw79
Anopheles kleini	126.65192	37.71319	Unclassified	GBIF: https://doi.org/10.15468/dl.zfaw79
Anopheles kleini	126.69833	37.66833	Unclassified	GBIF: https://doi.org/10.15468/dl.zfaw79
Anopheles kleini	126.71667	37.81667	Unclassified	GBIF: https://doi.org/10.15468/dl.zfaw79
Anopheles kleini	126.72362	37.93156	Unclassified	GBIF: https://doi.org/10.15468/dl.zfaw79
Anopheles kleini	126.73367	37.90517	Unclassified	GBIF: https://doi.org/10.15468/dl.zfaw79
Anopheles kleini	126.7373	37.90802	Unclassified	GBIF: https://doi.org/10.15468/dl.zfaw79
Anopheles kleini	126.74159	37.91911	Unclassified	GBIF: https://doi.org/10.15468/dl.zfaw79
Anopheles kleini	126.74172	37.92204	Unclassified	GBIF: https://doi.org/10.15468/dl.zfaw79
Anopheles kleini	126.75861	37.87944	Unclassified	GBIF: https://doi.org/10.15468/dl.zfaw79
Anopheles kleini	126.75911	37.87861	Unclassified	GBIF: https://doi.org/10.15468/dl.zfaw79
Anopheles kleini	126.79071	37.92292	Unclassified	GBIF: https://doi.org/10.15468/dl.zfaw79
Anopheles kleini	126.79646	37.90391	Unclassified	GBIF: https://doi.org/10.15468/dl.zfaw79
Anopheles kleini	126.80189	37.89508	Unclassified	GBIF: https://doi.org/10.15468/dl.zfaw79
Anopheles kleini	126.8032	35.12422	Unclassified	GBIF: https://doi.org/10.15468/dl.zfaw79
Anopheles kleini	126.82128	37.89816	Unclassified	GBIF: https://doi.org/10.15468/dl.zfaw79
Anopheles kleini	126.82971	37.89038	Unclassified	GBIF: https://doi.org/10.15468/dl.zfaw79
Anopheles kleini	126.8324	37.89359	Unclassified	GBIF: https://doi.org/10.15468/dl.zfaw79

Anopheles kleini	126.93028	35.21139	Unclassified	GBIF: https://doi.org/10.15468/dl.zfaw79
Anopheles kleini	126.9491	37.97792	Unclassified	GBIF: https://doi.org/10.15468/dl.zfaw79
Anopheles kleini	126.98611	37.99222	Unclassified	GBIF: https://doi.org/10.15468/dl.zfaw79
Anopheles kleini	126.99102	38.01699	Unclassified	GBIF: https://doi.org/10.15468/dl.zfaw79
Anopheles kleini	126.99399	38.0443	Unclassified	GBIF: https://doi.org/10.15468/dl.zfaw79
Anopheles kleini	127.02803	35.88808	Unclassified	GBIF: https://doi.org/10.15468/dl.zfaw79
Anopheles kleini	127.03031	36.95795	Unclassified	GBIF: https://doi.org/10.15468/dl.zfaw79
Anopheles kleini	127.07473	38.07828	Unclassified	GBIF: https://doi.org/10.15468/dl.zfaw79
Anopheles kleini	127.07506	37.92381	Unclassified	GBIF: https://doi.org/10.15468/dl.zfaw79
Anopheles kleini	127.08333	34.76044	Unclassified	GBIF: https://doi.org/10.15468/dl.zfaw79
Anopheles kleini	127.08675	37.90717	Unclassified	GBIF: https://doi.org/10.15468/dl.zfaw79
Anopheles kleini	127.0897	37.92309	Unclassified	GBIF: https://doi.org/10.15468/dl.zfaw79
Anopheles kleini	127.15597	37.37981	Unclassified	GBIF: https://doi.org/10.15468/dl.zfaw79
Anopheles kleini	127.23071	38.03663	Unclassified	GBIF: https://doi.org/10.15468/dl.zfaw79
Anopheles kleini	127.31845	38.06074	Unclassified	GBIF: https://doi.org/10.15468/dl.zfaw79
Anopheles kleini	127.31883	37.90394	Unclassified	GBIF: https://doi.org/10.15468/dl.zfaw79
Anopheles kleini	127.38639	37.66639	Unclassified	GBIF: https://doi.org/10.15468/dl.zfaw79
Anopheles kleini	127.38644	37.66653	Unclassified	GBIF: https://doi.org/10.15468/dl.zfaw79
Anopheles kleini	127.52522	38.04956	Unclassified	GBIF: https://doi.org/10.15468/dl.zfaw79
Anopheles kleini	127.52564	38.06811	Unclassified	GBIF: https://doi.org/10.15468/dl.zfaw79
Anopheles kleini	127.52703	38.08531	Unclassified	GBIF: https://doi.org/10.15468/dl.zfaw79
Anopheles kleini	127.70719	37.86369	Unclassified	GBIF: https://doi.org/10.15468/dl.zfaw79
Anopheles kleini	127.78336	37.87008	Unclassified	GBIF: https://doi.org/10.15468/dl.zfaw79
Anopheles kleini	127.95293	37.39079	Unclassified	GBIF: https://doi.org/10.15468/dl.zfaw79
Anopheles kleini	127.96047	37.12158	Unclassified	GBIF: https://doi.org/10.15468/dl.zfaw79
Anopheles kleini	127.97342	37.45056	Unclassified	GBIF: https://doi.org/10.15468/dl.zfaw79
Anopheles kleini	128.24349	37.14442	Unclassified	GBIF: https://doi.org/10.15468/dl.zfaw79
Anopheles kleini	129.07467	36.40253	Unclassified	GBIF: https://doi.org/10.15468/dl.zfaw79
Anopheles kleini	129.19161	37.13942	Unclassified	GBIF: https://doi.org/10.15468/dl.zfaw79
Anopheles kleini	129.22419	35.2685	Unclassified	GBIF: https://doi.org/10.15468/dl.zfaw79
Anopheles kleini	129.226	35.23981	Unclassified	GBIF: https://doi.org/10.15468/dl.zfaw79
Anopheles kleini	129.24178	35.80222	Unclassified	GBIF: https://doi.org/10.15468/dl.zfaw79
Anopheles kleini	129.37517	36.54125	Unclassified	GBIF: https://doi.org/10.15468/dl.zfaw79
Anopheles kleini	129.39436	36.9015	Unclassified	GBIF: https://doi.org/10.15468/dl.zfaw79
Anopheles kleini	129.43772	46.82447	Unclassified	GBIF: https://doi.org/10.15468/dl.zfaw79
Anopheles kleini	129.462	36.76947	Unclassified	GBIF: https://doi.org/10.15468/dl.zfaw79
Anopheles kochi	90.72	27.59	Unclassified	GBIF: https://doi.org/10.15468/dl.zfaw79
Anopheles kochi	92.26	21.8	Unclassified	GBIF: https://doi.org/10.15468/dl.zfaw79
Anopheles kochi	92.66	11.74	Unclassified	GBIF: https://doi.org/10.15468/dl.zfaw79
Anopheles kochi	97.179722	3.261389	Unclassified	GBIF: https://doi.org/10.15468/dl.zfaw79
Anopheles kochi	97.383333	4.416111	Unclassified	GBIF: https://doi.org/10.15468/dl.zfaw79
Anopheles kochi	97.67712	18.523387	Unclassified	GBIF: https://doi.org/10.15468/dl.zfaw79

Anopheles kochi	97.725302	18.41257	Unclassified	GBIF: https://doi.org/10.15468/dl.zfaw79
Anopheles kochi	97.82409	18.311524	Unclassified	GBIF: https://doi.org/10.15468/dl.zfaw79
Anopheles kochi	97.826148	18.754803	Unclassified	GBIF: https://doi.org/10.15468/dl.zfaw79
Anopheles kochi	97.84472	18.533523	Unclassified	GBIF: https://doi.org/10.15468/dl.zfaw79
Anopheles kochi	97.84906	17.873848	Unclassified	GBIF: https://doi.org/10.15468/dl.zfaw79
Anopheles kochi	97.850588	18.64742	Unclassified	GBIF: https://doi.org/10.15468/dl.zfaw79
Anopheles kochi	97.878886	18.378295	Unclassified	GBIF: https://doi.org/10.15468/dl.zfaw79
Anopheles kochi	97.884694	18.210241	Unclassified	GBIF: https://doi.org/10.15468/dl.zfaw79
Anopheles kochi	97.891199	19.198597	Unclassified	GBIF: https://doi.org/10.15468/dl.zfaw79
Anopheles kochi	97.900873	17.889511	Unclassified	GBIF: https://doi.org/10.15468/dl.zfaw79
Anopheles kochi	97.904747	18.479659	Unclassified	GBIF: https://doi.org/10.15468/dl.zfaw79
Anopheles kochi	97.911314	18.034168	Unclassified	GBIF: https://doi.org/10.15468/dl.zfaw79
Anopheles kochi	97.911956	18.387524	Unclassified	GBIF: https://doi.org/10.15468/dl.zfaw79
Anopheles kochi	97.928027	18.082159	Unclassified	GBIF: https://doi.org/10.15468/dl.zfaw79
Anopheles kochi	97.94749	19.070902	Unclassified	GBIF: https://doi.org/10.15468/dl.zfaw79
Anopheles kochi	97.969765	18.376097	Unclassified	GBIF: https://doi.org/10.15468/dl.zfaw79
Anopheles kochi	97.975556	4.473056	Unclassified	GBIF: https://doi.org/10.15468/dl.zfaw79
Anopheles kochi	97.980989	18.489337	Unclassified	GBIF: https://doi.org/10.15468/dl.zfaw79
Anopheles kochi	97.988361	18.453693	Unclassified	GBIF: https://doi.org/10.15468/dl.zfaw79
Anopheles kochi	97.992498	18.332777	Unclassified	GBIF: https://doi.org/10.15468/dl.zfaw79
Anopheles kochi	97.993719	17.995753	Unclassified	GBIF: https://doi.org/10.15468/dl.zfaw79
Anopheles kochi	98.00489	18.350394	Unclassified	GBIF: https://doi.org/10.15468/dl.zfaw79
Anopheles kochi	98.016401	18.297805	Unclassified	GBIF: https://doi.org/10.15468/dl.zfaw79
Anopheles kochi	98.021648	18.739661	Unclassified	GBIF: https://doi.org/10.15468/dl.zfaw79
Anopheles kochi	98.055716	17.702336	Unclassified	GBIF: https://doi.org/10.15468/dl.zfaw79
Anopheles kochi	98.072219	18.155207	Unclassified	GBIF: https://doi.org/10.15468/dl.zfaw79
Anopheles kochi	98.076833	17.752515	Unclassified	GBIF: https://doi.org/10.15468/dl.zfaw79
Anopheles kochi	98.08554	18.190335	Unclassified	GBIF: https://doi.org/10.15468/dl.zfaw79
Anopheles kochi	98.09158	18.250193	Unclassified	GBIF: https://doi.org/10.15468/dl.zfaw79
Anopheles kochi	98.126585	18.470102	Unclassified	GBIF: https://doi.org/10.15468/dl.zfaw79
Anopheles kochi	98.127542	18.149959	Unclassified	GBIF: https://doi.org/10.15468/dl.zfaw79
Anopheles kochi	98.138216	18.191664	Unclassified	GBIF: https://doi.org/10.15468/dl.zfaw79
Anopheles kochi	98.153831	18.106116	Unclassified	GBIF: https://doi.org/10.15468/dl.zfaw79
Anopheles kochi	98.161384	19.501423	Unclassified	GBIF: https://doi.org/10.15468/dl.zfaw79
Anopheles kochi	98.167296	18.040862	Unclassified	GBIF: https://doi.org/10.15468/dl.zfaw79
Anopheles kochi	98.20166	17.900948	Unclassified	GBIF: https://doi.org/10.15468/dl.zfaw79
Anopheles kochi	98.206252	18.445299	Unclassified	GBIF: https://doi.org/10.15468/dl.zfaw79
Anopheles kochi	98.243856	17.914949	Unclassified	GBIF: https://doi.org/10.15468/dl.zfaw79
Anopheles kochi	98.259209	19.493173	Unclassified	GBIF: https://doi.org/10.15468/dl.zfaw79
Anopheles kochi	98.293339	19.455937	Unclassified	GBIF: https://doi.org/10.15468/dl.zfaw79
Anopheles kochi	98.37308	8.28871	Unclassified	GBIF: https://doi.org/10.15468/dl.zfaw79
Anopheles kochi	98.391201	8.333326	Unclassified	GBIF: https://doi.org/10.15468/dl.zfaw79

Anopheles kochi	98.42031	8.300807	Unclassified	GBIF: https://doi.org/10.15468/dl.zfaw79
Anopheles kochi	98.428423	18.570699	Unclassified	GBIF: https://doi.org/10.15468/dl.zfaw79
Anopheles kochi	98.43	8.56	Unclassified	GBIF: https://doi.org/10.15468/dl.zfaw79
Anopheles kochi	98.459221	18.420279	Unclassified	GBIF: https://doi.org/10.15468/dl.zfaw79
Anopheles kochi	98.490191	17.788002	Unclassified	GBIF: https://doi.org/10.15468/dl.zfaw79
Anopheles kochi	98.49315	8.50597	Unclassified	GBIF: https://doi.org/10.15468/dl.zfaw79
Anopheles kochi	98.49348	8.48945	Unclassified	GBIF: https://doi.org/10.15468/dl.zfaw79
Anopheles kochi	98.527969	17.01478	Unclassified	GBIF: https://doi.org/10.15468/dl.zfaw79
Anopheles kochi	98.55041	8.54843	Unclassified	GBIF: https://doi.org/10.15468/dl.zfaw79
Anopheles kochi	98.62446	8.58293	Unclassified	GBIF: https://doi.org/10.15468/dl.zfaw79
Anopheles kochi	98.6747	18.48171	Unclassified	GBIF: https://doi.org/10.15468/dl.zfaw79
Anopheles kochi	98.785	1.736667	Unclassified	GBIF: https://doi.org/10.15468/dl.zfaw79
Anopheles kochi	98.8133	8.855	Unclassified	GBIF: https://doi.org/10.15468/dl.zfaw79
Anopheles kochi	98.833532	18.796849	Unclassified	GBIF: https://doi.org/10.15468/dl.zfaw79
Anopheles kochi	98.85765	14.43925	Unclassified	GBIF: https://doi.org/10.15468/dl.zfaw79
Anopheles kochi	98.87621	14.43022	Unclassified	GBIF: https://doi.org/10.15468/dl.zfaw79
Anopheles kochi	98.90387	18.57506	Unclassified	GBIF: https://doi.org/10.15468/dl.zfaw79
Anopheles kochi	98.90558	18.91036	Unclassified	GBIF: https://doi.org/10.15468/dl.zfaw79
Anopheles kochi	98.921222	18.805417	Unclassified	GBIF: https://doi.org/10.15468/dl.zfaw79
Anopheles kochi	98.925657	18.734034	Unclassified	GBIF: https://doi.org/10.15468/dl.zfaw79
Anopheles kochi	98.928393	18.735514	Unclassified	GBIF: https://doi.org/10.15468/dl.zfaw79
Anopheles kochi	98.93	19.15	Unclassified	GBIF: https://doi.org/10.15468/dl.zfaw79
Anopheles kochi	98.930533	18.73501	Unclassified	GBIF: https://doi.org/10.15468/dl.zfaw79
Anopheles kochi	98.93293	19.38666	Unclassified	GBIF: https://doi.org/10.15468/dl.zfaw79
Anopheles kochi	98.938908	16.062086	Unclassified	GBIF: https://doi.org/10.15468/dl.zfaw79
Anopheles kochi	98.94455	18.81187	Unclassified	GBIF: https://doi.org/10.15468/dl.zfaw79
Anopheles kochi	98.96256	18.87695	Unclassified	GBIF: https://doi.org/10.15468/dl.zfaw79
Anopheles kochi	98.98257	19.16918	Unclassified	GBIF: https://doi.org/10.15468/dl.zfaw79
Anopheles kochi	98.998904	14.49628	Unclassified	GBIF: https://doi.org/10.15468/dl.zfaw79
Anopheles kochi	99.013478	14.077114	Unclassified	GBIF: https://doi.org/10.15468/dl.zfaw79
Anopheles kochi	99.12109	18.948726	Unclassified	GBIF: https://doi.org/10.15468/dl.zfaw79
Anopheles kochi	99.178938	-1.627301	Unclassified	GBIF: https://doi.org/10.15468/dl.zfaw79
Anopheles kochi	99.24081	18.92017	Unclassified	GBIF: https://doi.org/10.15468/dl.zfaw79
Anopheles kochi	99.272222	1.376944	Unclassified	GBIF: https://doi.org/10.15468/dl.zfaw79
Anopheles kochi	99.2725	1.378056	Unclassified	GBIF: https://doi.org/10.15468/dl.zfaw79
Anopheles kochi	99.296171	13.616696	Unclassified	GBIF: https://doi.org/10.15468/dl.zfaw79
Anopheles kochi	99.359444	0.798056	Unclassified	GBIF: https://doi.org/10.15468/dl.zfaw79
Anopheles kochi	99.41	7.66	Unclassified	GBIF: https://doi.org/10.15468/dl.zfaw79
Anopheles kochi	99.496263	11.223328	Unclassified	GBIF: https://doi.org/10.15468/dl.zfaw79
Anopheles kochi	99.580969	20.243946	Unclassified	GBIF: https://doi.org/10.15468/dl.zfaw79
Anopheles kochi	99.70165	8.19688	Unclassified	GBIF: https://doi.org/10.15468/dl.zfaw79
Anopheles kochi	99.73277	8.16845	Unclassified	GBIF: https://doi.org/10.15468/dl.zfaw79

Anopheles kochi	99.76458	8.14828	Unclassified	GBIF: https://doi.org/10.15468/dl.zfaw79
Anopheles kochi	99.81042	8.13246	Unclassified	GBIF: https://doi.org/10.15468/dl.zfaw79
Anopheles kochi	99.86396	8.43068	Unclassified	GBIF: https://doi.org/10.15468/dl.zfaw79
Anopheles kochi	99.8965	19.00492	Unclassified	GBIF: https://doi.org/10.15468/dl.zfaw79
Anopheles kochi	99.91839	19.01114	Unclassified	GBIF: https://doi.org/10.15468/dl.zfaw79
Anopheles kochi	99.92632	9.56368	Unclassified	GBIF: https://doi.org/10.15468/dl.zfaw79
Anopheles kochi	99.93529	9.50939	Unclassified	GBIF: https://doi.org/10.15468/dl.zfaw79
Anopheles kochi	99.94432	9.48224	Unclassified	GBIF: https://doi.org/10.15468/dl.zfaw79
Anopheles kochi	99.94447	9.5365	Unclassified	GBIF: https://doi.org/10.15468/dl.zfaw79
Anopheles kochi	99.95338	9.46413	Unclassified	GBIF: https://doi.org/10.15468/dl.zfaw79
Anopheles kochi	100.03533	9.45485	Unclassified	GBIF: https://doi.org/10.15468/dl.zfaw79
Anopheles kochi	100.3675	-0.300278	Unclassified	GBIF: https://doi.org/10.15468/dl.zfaw79
Anopheles kochi	100.60612	18.40109	Unclassified	GBIF: https://doi.org/10.15468/dl.zfaw79
Anopheles kochi	100.63537	18.49119	Unclassified	GBIF: https://doi.org/10.15468/dl.zfaw79
Anopheles kochi	100.81	18.78	Unclassified	GBIF: https://doi.org/10.15468/dl.zfaw79
Anopheles kochi	100.85772	14.66424	Unclassified	GBIF: https://doi.org/10.15468/dl.zfaw79
Anopheles kochi	100.89131	14.68476	Unclassified	GBIF: https://doi.org/10.15468/dl.zfaw79
Anopheles kochi	100.89854	14.6612	Unclassified	GBIF: https://doi.org/10.15468/dl.zfaw79
Anopheles kochi	101.39096	14.53559	Unclassified	GBIF: https://doi.org/10.15468/dl.zfaw79
Anopheles kochi	101.50725	14.60882	Unclassified	GBIF: https://doi.org/10.15468/dl.zfaw79
Anopheles kochi	101.80659	5.94563	Unclassified	GBIF: https://doi.org/10.15468/dl.zfaw79
Anopheles kochi	101.86975	14.2683	Unclassified	GBIF: https://doi.org/10.15468/dl.zfaw79
Anopheles kochi	101.8966	14.19572	Unclassified	GBIF: https://doi.org/10.15468/dl.zfaw79
Anopheles kochi	102.03256	12.60537	Unclassified	GBIF: https://doi.org/10.15468/dl.zfaw79
Anopheles kochi	102.08925	12.47051	Unclassified	GBIF: https://doi.org/10.15468/dl.zfaw79
Anopheles kochi	102.3425	-1.218611	Unclassified	GBIF: https://doi.org/10.15468/dl.zfaw79
Anopheles kochi	102.34305	16.64641	Unclassified	GBIF: https://doi.org/10.15468/dl.zfaw79
Anopheles kochi	102.941229	5.522312	Unclassified	GBIF: https://doi.org/10.15468/dl.zfaw79
Anopheles kochi	103.81026	1.352825	Unclassified	GBIF: https://doi.org/10.15468/dl.zfaw79
Anopheles kochi	104.275563	17.480126	Unclassified	GBIF: https://doi.org/10.15468/dl.zfaw79
Anopheles kochi	104.69114	38.073254	Unclassified	GBIF: https://doi.org/10.15468/dl.zfaw79
Anopheles kochi	105.23	14.53	Unclassified	GBIF: https://doi.org/10.15468/dl.zfaw79
Anopheles kochi	105.338703	14.558419	Unclassified	GBIF: https://doi.org/10.15468/dl.zfaw79
Anopheles kochi	106.78333	-6.58333	Unclassified	GBIF: https://doi.org/10.15468/dl.zfaw79
Anopheles kochi	106.845278	-6.204167	Unclassified	GBIF: https://doi.org/10.15468/dl.zfaw79
Anopheles kochi	106.932333	-6.680078	Unclassified	GBIF: https://doi.org/10.15468/dl.zfaw79
Anopheles kochi	107	13.73	Unclassified	GBIF: https://doi.org/10.15468/dl.zfaw79
Anopheles kochi	107.6525	-2.738611	Unclassified	GBIF: https://doi.org/10.15468/dl.zfaw79
Anopheles kochi	108.905278	-7.724444	Unclassified	GBIF: https://doi.org/10.15468/dl.zfaw79
Anopheles kochi	109.023056	-7.697778	Unclassified	GBIF: https://doi.org/10.15468/dl.zfaw79
Anopheles kochi	109.173139	-7.53588	Unclassified	GBIF: https://doi.org/10.15468/dl.zfaw79
Anopheles kochi	109.514722	-7.603333	Unclassified	GBIF: https://doi.org/10.15468/dl.zfaw79

Anopheles kochi	110.333	1.4167	Unclassified	GBIF: https://doi.org/10.15468/dl.zfaw79
Anopheles kochi	110.410278	-7.270833	Unclassified	GBIF: https://doi.org/10.15468/dl.zfaw79
Anopheles kochi	110.563056	-7.698333	Unclassified	GBIF: https://doi.org/10.15468/dl.zfaw79
Anopheles kochi	115.2167	5.35	Unclassified	GBIF: https://doi.org/10.15468/dl.zfaw79
Anopheles kochi	116.79	5.69	Unclassified	GBIF: https://doi.org/10.15468/dl.zfaw79
Anopheles kochi	116.854444	-1.2375	Unclassified	GBIF: https://doi.org/10.15468/dl.zfaw79
Anopheles kochi	119.65	-5.14	Unclassified	GBIF: https://doi.org/10.15468/dl.zfaw79
Anopheles kochi	119.844007	16.194132	Unclassified	GBIF: https://doi.org/10.15468/dl.zfaw79
Anopheles kochi	119.872251	-0.819373	Unclassified	GBIF: https://doi.org/10.15468/dl.zfaw79
Anopheles kochi	119.966516	-1.170781	Unclassified	GBIF: https://doi.org/10.15468/dl.zfaw79
Anopheles kochi	119.968603	-1.171993	Unclassified	GBIF: https://doi.org/10.15468/dl.zfaw79
Anopheles kochi	121.067613	12.352748	Unclassified	GBIF: https://doi.org/10.15468/dl.zfaw79
Anopheles kochi	121.0833	14.9	Unclassified	GBIF: https://doi.org/10.15468/dl.zfaw79
Anopheles kochi	121.124203	14.626047	Unclassified	GBIF: https://doi.org/10.15468/dl.zfaw79
Anopheles kochi	121.124426	14.625222	Unclassified	GBIF: https://doi.org/10.15468/dl.zfaw79
Anopheles kochi	121.124599	14.62471	Unclassified	GBIF: https://doi.org/10.15468/dl.zfaw79
Anopheles kochi	121.124744	14.624497	Unclassified	GBIF: https://doi.org/10.15468/dl.zfaw79
Anopheles kochi	121.125228	14.624753	Unclassified	GBIF: https://doi.org/10.15468/dl.zfaw79
Anopheles kochi	121.125601	14.62508	Unclassified	GBIF: https://doi.org/10.15468/dl.zfaw79
Anopheles kochi	122.038574	-5.309061	Unclassified	GBIF: https://doi.org/10.15468/dl.zfaw79
Anopheles kochi	124.674744	11.292469	Unclassified	GBIF: https://doi.org/10.15468/dl.zfaw79
Anopheles kochi	124.961687	11.254339	Unclassified	GBIF: https://doi.org/10.15468/dl.zfaw79
Anopheles koliensis	11.43243	55.76478	Unclassified	GBIF: https://doi.org/10.15468/dl.zfaw79
Anopheles koliensis	133.599137	-2.099277	Unclassified	GBIF: https://doi.org/10.15468/dl.zfaw79
Anopheles koliensis	137.89999	-1.48	Unclassified	GBIF: https://doi.org/10.15468/dl.zfaw79
Anopheles koliensis	138.883333	-1.966667	Unclassified	GBIF: https://doi.org/10.15468/dl.zfaw79
Anopheles koliensis	139.012967	-1.938879	Unclassified	GBIF: https://doi.org/10.15468/dl.zfaw79
Anopheles koliensis	139.01331	-1.938707	Unclassified	GBIF: https://doi.org/10.15468/dl.zfaw79
Anopheles koliensis	139.013353	-1.938855	Unclassified	GBIF: https://doi.org/10.15468/dl.zfaw79
Anopheles koliensis	140.276667	-6.080278	Unclassified	GBIF: https://doi.org/10.15468/dl.zfaw79
Anopheles koliensis	140.306489	-6.097564	Unclassified	GBIF: https://doi.org/10.15468/dl.zfaw79
Anopheles koliensis	140.404444	-8.495833	Unclassified	GBIF: https://doi.org/10.15468/dl.zfaw79
Anopheles koliensis	140.668998	-2.591601	Unclassified	GBIF: https://doi.org/10.15468/dl.zfaw79
Anopheles koliensis	140.7	-2.5333	Unclassified	GBIF: https://doi.org/10.15468/dl.zfaw79
Anopheles koliensis	141.1441	-4.1038	Unclassified	GBIF: https://doi.org/10.15468/dl.zfaw79
Anopheles koliensis	141.53329	-7.1333	Unclassified	GBIF: https://doi.org/10.15468/dl.zfaw79
Anopheles koliensis	142.0429	-3.8191	Unclassified	GBIF: https://doi.org/10.15468/dl.zfaw79
Anopheles koliensis	142.3265	-4.3125	Unclassified	GBIF: https://doi.org/10.15468/dl.zfaw79
Anopheles koliensis	143.03329	-4	Unclassified	GBIF: https://doi.org/10.15468/dl.zfaw79
Anopheles koliensis	143.05	-3.63333	Unclassified	GBIF: https://doi.org/10.15468/dl.zfaw79
Anopheles koliensis	143.274	-4.5914	Unclassified	GBIF: https://doi.org/10.15468/dl.zfaw79
Anopheles koliensis	143.515	-4.1875	Unclassified	GBIF: https://doi.org/10.15468/dl.zfaw79

Anopheles koliensis	143.5633	-4.0915	Unclassified	GBIF: https://doi.org/10.15468/dl.zfaw79
Anopheles koliensis	143.6667	-3.58333	Unclassified	GBIF: https://doi.org/10.15468/dl.zfaw79
Anopheles koliensis	143.71671	-3.6167	Unclassified	GBIF: https://doi.org/10.15468/dl.zfaw79
Anopheles koliensis	143.9272	-3.9566	Unclassified	GBIF: https://doi.org/10.15468/dl.zfaw79
Anopheles koliensis	144.4958	-4.719	Unclassified	GBIF: https://doi.org/10.15468/dl.zfaw79
Anopheles koliensis	144.5847	-4.5503	Unclassified	GBIF: https://doi.org/10.15468/dl.zfaw79
Anopheles koliensis	145.4598	-5.239	Unclassified	GBIF: https://doi.org/10.15468/dl.zfaw79
Anopheles koliensis	145.6667	-5	Unclassified	GBIF: https://doi.org/10.15468/dl.zfaw79
Anopheles koliensis	145.67	-5.37	Unclassified	GBIF: https://doi.org/10.15468/dl.zfaw79
Anopheles koliensis	145.6833	-5.2833	Unclassified	GBIF: https://doi.org/10.15468/dl.zfaw79
Anopheles koliensis	145.72	-5.22	Unclassified	GBIF: https://doi.org/10.15468/dl.zfaw79
Anopheles koliensis	145.7264	-5.4142	Unclassified	GBIF: https://doi.org/10.15468/dl.zfaw79
Anopheles koliensis	145.7381	-5.8854	Unclassified	GBIF: https://doi.org/10.15468/dl.zfaw79
Anopheles koliensis	145.75	-5.3	Unclassified	GBIF: https://doi.org/10.15468/dl.zfaw79
Anopheles koliensis	145.75	-5.35	Unclassified	GBIF: https://doi.org/10.15468/dl.zfaw79
Anopheles koliensis	145.75	-5.3667	Unclassified	GBIF: https://doi.org/10.15468/dl.zfaw79
Anopheles koliensis	145.7715	-7.9692	Unclassified	GBIF: https://doi.org/10.15468/dl.zfaw79
Anopheles koliensis	145.78329	-5.0833	Unclassified	GBIF: https://doi.org/10.15468/dl.zfaw79
Anopheles koliensis	145.8265	-5.4777	Unclassified	GBIF: https://doi.org/10.15468/dl.zfaw79
Anopheles koliensis	146.3396	-7.8547	Unclassified	GBIF: https://doi.org/10.15468/dl.zfaw79
Anopheles koliensis	146.3913	-7.863	Unclassified	GBIF: https://doi.org/10.15468/dl.zfaw79
Anopheles koliensis	146.531	-7.8405	Unclassified	GBIF: https://doi.org/10.15468/dl.zfaw79
Anopheles koliensis	146.76669	-6.58333	Unclassified	GBIF: https://doi.org/10.15468/dl.zfaw79
Anopheles koliensis	146.8209	-6.5761	Unclassified	GBIF: https://doi.org/10.15468/dl.zfaw79
Anopheles koliensis	146.8472	-8.8376	Unclassified	GBIF: https://doi.org/10.15468/dl.zfaw79
Anopheles koliensis	146.9404	-5.9077	Unclassified	GBIF: https://doi.org/10.15468/dl.zfaw79
Anopheles koliensis	147	-6.71667	Unclassified	GBIF: https://doi.org/10.15468/dl.zfaw79
Anopheles koliensis	147.0786	-5.9592	Unclassified	GBIF: https://doi.org/10.15468/dl.zfaw79
Anopheles koliensis	147.2295	-9.297	Unclassified	GBIF: https://doi.org/10.15468/dl.zfaw79
Anopheles koliensis	147.2465	-7.4852	Unclassified	GBIF: https://doi.org/10.15468/dl.zfaw79
Anopheles koliensis	148.571	-9.2104	Unclassified	GBIF: https://doi.org/10.15468/dl.zfaw79
Anopheles koliensis	150.25	-10.3167	Unclassified	GBIF: https://doi.org/10.15468/dl.zfaw79
Anopheles koliensis	150.467	-9.583	Unclassified	GBIF: https://doi.org/10.15468/dl.zfaw79
Anopheles koliensis	155.26669	-5.86667	Unclassified	GBIF: https://doi.org/10.15468/dl.zfaw79
Anopheles koliensis	160.2	-9.6	Unclassified	GBIF: https://doi.org/10.15468/dl.zfaw79
Anopheles koliensis	160.2	-9.5333	Unclassified	GBIF: https://doi.org/10.15468/dl.zfaw79
Anopheles kompi	-79.783333	8.880278	Unclassified	GBIF: https://doi.org/10.15468/dl.zfaw79
Anopheles kompi	-54.383048	4.27418	Unclassified	GBIF: https://doi.org/10.15468/dl.zfaw79
Anopheles kompi	-54.07664	3.67331	Unclassified	GBIF: https://doi.org/10.15468/dl.zfaw79
Anopheles kompi	-54.04329	5.484058	Unclassified	GBIF: https://doi.org/10.15468/dl.zfaw79
Anopheles kompi	-54.031415	3.643348	Unclassified	GBIF: https://doi.org/10.15468/dl.zfaw79
Anopheles kompi	-54.02004	5.50717	Unclassified	GBIF: https://doi.org/10.15468/dl.zfaw79

Anopheles kompi	-53.24036	3.91451	Unclassified	GBIF: https://doi.org/10.15468/dl.zfaw79
Anopheles kompi	-53.23477	3.92404	Unclassified	GBIF: https://doi.org/10.15468/dl.zfaw79
Anopheles kompi	-53.20944	3.623127	Unclassified	GBIF: https://doi.org/10.15468/dl.zfaw79
Anopheles kompi	-53.050025	5.062018	Unclassified	GBIF: https://doi.org/10.15468/dl.zfaw79
Anopheles kompi	-52.643374	5.166931	Unclassified	GBIF: https://doi.org/10.15468/dl.zfaw79
Anopheles kompi	-52.57423	5.03313	Unclassified	GBIF: https://doi.org/10.15468/dl.zfaw79
Anopheles kompi	-52.473837	5.013996	Unclassified	GBIF: https://doi.org/10.15468/dl.zfaw79
Anopheles kompi	-52.47241	5.01171	Unclassified	GBIF: https://doi.org/10.15468/dl.zfaw79
Anopheles kompi	-52.47027	4.572008	Unclassified	GBIF: https://doi.org/10.15468/dl.zfaw79
Anopheles kompi	-52.32628	4.727944	Unclassified	GBIF: https://doi.org/10.15468/dl.zfaw79
Anopheles kompi	-52.316695	4.516688	Unclassified	GBIF: https://doi.org/10.15468/dl.zfaw79
Anopheles kompi	-52.291065	4.890268	Unclassified	GBIF: https://doi.org/10.15468/dl.zfaw79
Anopheles kompi	-52.272123	4.912182	Unclassified	GBIF: https://doi.org/10.15468/dl.zfaw79
Anopheles kompi	-52.259305	4.911678	Unclassified	GBIF: https://doi.org/10.15468/dl.zfaw79
Anopheles kompi	-52.129162	4.314002	Unclassified	GBIF: https://doi.org/10.15468/dl.zfaw79
Anopheles kompi	-51.801158	3.888398	Unclassified	GBIF: https://doi.org/10.15468/dl.zfaw79
Anopheles kompi	-48.44	-22.08	Unclassified	GBIF: https://doi.org/10.15468/dl.zfaw79
Anopheles kompi	-48.43736	-22.07569	Unclassified	GBIF: https://doi.org/10.15468/dl.zfaw79
Anopheles konderi	-76.991	-0.472	Unclassified	GBIF: https://doi.org/10.15468/dl.zfaw79
Anopheles konderi	-76.182	-0.638	Unclassified	GBIF: https://doi.org/10.15468/dl.zfaw79
Anopheles konderi	-76.18	-0.64	Unclassified	GBIF: https://doi.org/10.15468/dl.zfaw79
Anopheles konderi	-76.16	-0.64	Unclassified	GBIF: https://doi.org/10.15468/dl.zfaw79
Anopheles konderi	-76.15	-0.63	Unclassified	GBIF: https://doi.org/10.15468/dl.zfaw79
Anopheles konderi	-75.5103	-0.7991	Unclassified	GBIF: https://doi.org/10.15468/dl.zfaw79
Anopheles konderi	-73.8419	0.8699	Unclassified	GBIF: https://doi.org/10.15468/dl.zfaw79
Anopheles konderi	-73.279	-3.733	Unclassified	GBIF: https://doi.org/10.15468/dl.zfaw79
Anopheles konderi	-69.167	-11.917	Unclassified	GBIF: https://doi.org/10.15468/dl.zfaw79
Anopheles konderi	-68.9	-9.5	Unclassified	GBIF: https://doi.org/10.15468/dl.zfaw79
Anopheles konderi	-68.656898	-9.06556	Unclassified	GBIF: https://doi.org/10.15468/dl.zfaw79
Anopheles konderi	-67.85	-10.01	Unclassified	GBIF: https://doi.org/10.15468/dl.zfaw79
Anopheles konderi	-67.81	-10.24	Unclassified	GBIF: https://doi.org/10.15468/dl.zfaw79
Anopheles konderi	-67.22	-10.13	Unclassified	GBIF: https://doi.org/10.15468/dl.zfaw79
Anopheles konderi	-67.14	-9.68	Unclassified	GBIF: https://doi.org/10.15468/dl.zfaw79
Anopheles konderi	-67.13	-9.68	Unclassified	GBIF: https://doi.org/10.15468/dl.zfaw79
Anopheles konderi	-66.864	-9.951	Unclassified	GBIF: https://doi.org/10.15468/dl.zfaw79
Anopheles konderi	-66.86	-9.95	Unclassified	GBIF: https://doi.org/10.15468/dl.zfaw79
Anopheles konderi	-66.82	-10.01	Unclassified	GBIF: https://doi.org/10.15468/dl.zfaw79
Anopheles konderi	-66.817	-10.011	Unclassified	GBIF: https://doi.org/10.15468/dl.zfaw79
Anopheles konderi	-66.791	-10.003	Unclassified	GBIF: https://doi.org/10.15468/dl.zfaw79
Anopheles konderi	-66.79	-10	Unclassified	GBIF: https://doi.org/10.15468/dl.zfaw79
Anopheles konderi	-66.789	-10.003	Unclassified	GBIF: https://doi.org/10.15468/dl.zfaw79
Anopheles konderi	-63.9039	-8.76194	Unclassified	GBIF: https://doi.org/10.15468/dl.zfaw79

Anopheles konderi	-63.86	-8.72	Unclassified	GBIF: https://doi.org/10.15468/dl.zfaw79
Anopheles konderi	-63.82	-0.14	Unclassified	GBIF: https://doi.org/10.15468/dl.zfaw79
Anopheles konderi	-63.8167	0.1434	Unclassified	GBIF: https://doi.org/10.15468/dl.zfaw79
Anopheles konderi	-63.8167	0.1433	Unclassified	GBIF: https://doi.org/10.15468/dl.zfaw79
Anopheles konderi	-63.695599	-8.80972	Unclassified	GBIF: https://doi.org/10.15468/dl.zfaw79
Anopheles konderi	-63.24	-10.3	Unclassified	GBIF: https://doi.org/10.15468/dl.zfaw79
Anopheles konderi	-63.141399	-4.085	Unclassified	GBIF: https://doi.org/10.15468/dl.zfaw79
Anopheles konderi	-63.08	-4.1244	Unclassified	GBIF: https://doi.org/10.15468/dl.zfaw79
Anopheles konderi	-63.08	-4.12	Unclassified	GBIF: https://doi.org/10.15468/dl.zfaw79
Anopheles konderi	-59.75	-4.2684	Unclassified	GBIF: https://doi.org/10.15468/dl.zfaw79
Anopheles konderi	-59.75	-4.27	Unclassified	GBIF: https://doi.org/10.15468/dl.zfaw79
Anopheles konderi	-59.75	-4.26	Unclassified	GBIF: https://doi.org/10.15468/dl.zfaw79
Anopheles konderi	-59.132	-3.699	Unclassified	GBIF: https://doi.org/10.15468/dl.zfaw79
Anopheles konderi	-59.1319	-3.69917	Unclassified	GBIF: https://doi.org/10.15468/dl.zfaw79
Anopheles konderi	-59.13	-3.7	Unclassified	GBIF: https://doi.org/10.15468/dl.zfaw79
Anopheles konderi	-57.13	-1	Unclassified	GBIF: https://doi.org/10.15468/dl.zfaw79
Anopheles konderi	-57.1266	1.002	Unclassified	GBIF: https://doi.org/10.15468/dl.zfaw79
Anopheles konderi	-56.503773	-20.197704	Unclassified	GBIF: https://doi.org/10.15468/dl.zfaw79
Anopheles konderi	-56.50287	-20.197557	Unclassified	GBIF: https://doi.org/10.15468/dl.zfaw79
Anopheles konderi	-56.5	-20.2	Unclassified	GBIF: https://doi.org/10.15468/dl.zfaw79
Anopheles konderi	-54.35	-24.86	Unclassified	GBIF: https://doi.org/10.15468/dl.zfaw79
Anopheles konderi	-53.62	-23.06	Unclassified	GBIF: https://doi.org/10.15468/dl.zfaw79
Anopheles konderi	-53.36	-22.85	Unclassified	GBIF: https://doi.org/10.15468/dl.zfaw79
Anopheles konderi	-53.27	-22.77	Unclassified	GBIF: https://doi.org/10.15468/dl.zfaw79
Anopheles konderi	-51.066	0.034	Unclassified	GBIF: https://doi.org/10.15468/dl.zfaw79
Anopheles konderi	-51.05	0.03	Unclassified	GBIF: https://doi.org/10.15468/dl.zfaw79
Anopheles konderi	-50.97217	0.21314	Unclassified	GBIF: https://doi.org/10.15468/dl.zfaw79
Anopheles konderi	-50.97	0.21	Unclassified	GBIF: https://doi.org/10.15468/dl.zfaw79
Anopheles konderi	-50.902	0.2912	Unclassified	GBIF: https://doi.org/10.15468/dl.zfaw79
Anopheles konderi	-50.9	0.27	Unclassified	GBIF: https://doi.org/10.15468/dl.zfaw79
Anopheles konderi	-50.9	0.29	Unclassified	GBIF: https://doi.org/10.15468/dl.zfaw79
Anopheles konderi	-40.072201	-19.3911	Unclassified	GBIF: https://doi.org/10.15468/dl.zfaw79
Anopheles koreicus	126.315079	34.613931	Unclassified	GBIF: https://doi.org/10.15468/dl.zfaw79
Anopheles koreicus	126.346934	36.399859	Unclassified	GBIF: https://doi.org/10.15468/dl.zfaw79
Anopheles koreicus	126.353384	36.34235	Unclassified	GBIF: https://doi.org/10.15468/dl.zfaw79
Anopheles koreicus	126.357691	36.481654	Unclassified	GBIF: https://doi.org/10.15468/dl.zfaw79
Anopheles koreicus	126.365599	34.579388	Unclassified	GBIF: https://doi.org/10.15468/dl.zfaw79
Anopheles koreicus	126.36942	34.605255	Unclassified	GBIF: https://doi.org/10.15468/dl.zfaw79
Anopheles koreicus	126.383946	34.650879	Unclassified	GBIF: https://doi.org/10.15468/dl.zfaw79
Anopheles koreicus	126.389225	36.372872	Unclassified	GBIF: https://doi.org/10.15468/dl.zfaw79
Anopheles koreicus	126.394224	36.475314	Unclassified	GBIF: https://doi.org/10.15468/dl.zfaw79
Anopheles koreicus	126.398495	36.421756	Unclassified	GBIF: https://doi.org/10.15468/dl.zfaw79

Anopheles koreicus	126.4078	34.695114	Unclassified	GBIF: https://doi.org/10.15468/dl.zfaw79
Anopheles koreicus	126.424573	36.428993	Unclassified	GBIF: https://doi.org/10.15468/dl.zfaw79
Anopheles koreicus	126.433417	34.583814	Unclassified	GBIF: https://doi.org/10.15468/dl.zfaw79
Anopheles koreicus	126.440414	34.732822	Unclassified	GBIF: https://doi.org/10.15468/dl.zfaw79
Anopheles koreicus	126.466861	34.595833	Unclassified	GBIF: https://doi.org/10.15468/dl.zfaw79
Anopheles koreicus	126.487039	34.56469	Unclassified	GBIF: https://doi.org/10.15468/dl.zfaw79
Anopheles koreicus	126.494288	36.457605	Unclassified	GBIF: https://doi.org/10.15468/dl.zfaw79
Anopheles koreicus	126.786886	34.613928	Unclassified	GBIF: https://doi.org/10.15468/dl.zfaw79
Anopheles koreicus	126.93	37.54972	Unclassified	GBIF: https://doi.org/10.15468/dl.zfaw79
Anopheles koreicus	126.9491	37.97792	Unclassified	GBIF: https://doi.org/10.15468/dl.zfaw79
Anopheles koreicus	127.07506	37.92381	Unclassified	GBIF: https://doi.org/10.15468/dl.zfaw79
Anopheles koreicus	127.08917	37.90417	Unclassified	GBIF: https://doi.org/10.15468/dl.zfaw79
Anopheles koreicus	127.08972	37.90417	Unclassified	GBIF: https://doi.org/10.15468/dl.zfaw79
Anopheles koreicus	127.26153	37.96286	Unclassified	GBIF: https://doi.org/10.15468/dl.zfaw79
Anopheles koreicus	127.385256	35.590708	Unclassified	GBIF: https://doi.org/10.15468/dl.zfaw79
Anopheles koreicus	127.38644	37.66653	Unclassified	GBIF: https://doi.org/10.15468/dl.zfaw79
Anopheles koreicus	127.52564	38.06811	Unclassified	GBIF: https://doi.org/10.15468/dl.zfaw79
Anopheles koreicus	127.52703	38.08531	Unclassified	GBIF: https://doi.org/10.15468/dl.zfaw79
Anopheles koreicus	127.70719	37.86369	Unclassified	GBIF: https://doi.org/10.15468/dl.zfaw79
Anopheles koreicus	127.78053	37.87364	Unclassified	GBIF: https://doi.org/10.15468/dl.zfaw79
Anopheles koreicus	127.800725	37.221975	Unclassified	GBIF: https://doi.org/10.15468/dl.zfaw79
Anopheles koreicus	127.88581	37.83886	Unclassified	GBIF: https://doi.org/10.15468/dl.zfaw79
Anopheles koreicus	127.912247	35.862061	Unclassified	GBIF: https://doi.org/10.15468/dl.zfaw79
Anopheles koreicus	128.112397	37.113117	Unclassified	GBIF: https://doi.org/10.15468/dl.zfaw79
Anopheles koreicus	128.142236	34.998742	Unclassified	GBIF: https://doi.org/10.15468/dl.zfaw79
Anopheles koreicus	128.182443	34.83821	Unclassified	GBIF: https://doi.org/10.15468/dl.zfaw79
Anopheles koreicus	128.55439	34.903545	Unclassified	GBIF: https://doi.org/10.15468/dl.zfaw79
Anopheles koreicus	128.682495	34.98246	Unclassified	GBIF: https://doi.org/10.15468/dl.zfaw79
Anopheles koreicus	128.694803	34.826244	Unclassified	GBIF: https://doi.org/10.15468/dl.zfaw79
Anopheles koreicus	128.842767	36.813772	Unclassified	GBIF: https://doi.org/10.15468/dl.zfaw79
Anopheles koreicus	129.19161	37.13942	Unclassified	GBIF: https://doi.org/10.15468/dl.zfaw79
Anopheles koreicus	129.295	34.569	Unclassified	GBIF: https://doi.org/10.15468/dl.zfaw79
Anopheles koreicus	129.323	34.645	Unclassified	GBIF: https://doi.org/10.15468/dl.zfaw79
Anopheles koreicus	129.883	32.75	Unclassified	GBIF: https://doi.org/10.15468/dl.zfaw79
Anopheles koreicus	135.413	34.196	Unclassified	GBIF: https://doi.org/10.15468/dl.zfaw79
Anopheles koreicus	135.428	34.196	Unclassified	GBIF: https://doi.org/10.15468/dl.zfaw79
Anopheles kweiyangensis	103.08015	29.98004	Unclassified	GBIF: https://doi.org/10.15468/dl.zfaw79
Anopheles kweiyangensis	103.50015	30.20004	Unclassified	GBIF: https://doi.org/10.15468/dl.zfaw79
Anopheles kweiyangensis	103.73015	29.57004	Unclassified	GBIF: https://doi.org/10.15468/dl.zfaw79
Anopheles kweiyangensis	105.63015	25.95004	Unclassified	GBIF: https://doi.org/10.15468/dl.zfaw79
Anopheles kweiyangensis	108.73015	26.92004	Unclassified	GBIF: https://doi.org/10.15468/dl.zfaw79
Anopheles kweiyangensis	114.12015	32.10004	Unclassified	GBIF: https://doi.org/10.15468/dl.zfaw79

Anopheles kweiyangensis	122.10015	30.02004	Unclassified	GBIF: https://doi.org/10.15468/dl.zfaw79
Anopheles kweiyangensis	123.27015	40.29003	Unclassified	GBIF: https://doi.org/10.15468/dl.zfaw79
Anopheles kyondawensis	97.996656	19.01668	Unclassified	GBIF: https://doi.org/10.15468/dl.zfaw79
Anopheles kyondawensis	103.920707	14.47843	Unclassified	GBIF: https://doi.org/10.15468/dl.zfaw79
Anopheles labranchiae	-6.75	37.16	Unclassified	GBIF: https://doi.org/10.15468/dl.zfaw79
Anopheles labranchiae	-6.5	33.2833	Unclassified	GBIF: https://doi.org/10.15468/dl.zfaw79
Anopheles labranchiae	-6.5	33.28	Unclassified	GBIF: https://doi.org/10.15468/dl.zfaw79
Anopheles labranchiae	-6.15	35.1833	Unclassified	GBIF: https://doi.org/10.15468/dl.zfaw79
Anopheles labranchiae	-6.15	35.18	Unclassified	GBIF: https://doi.org/10.15468/dl.zfaw79
Anopheles labranchiae	-6.07	35.12	Unclassified	GBIF: https://doi.org/10.15468/dl.zfaw79
Anopheles labranchiae	-6.067	35.117	Unclassified	GBIF: https://doi.org/10.15468/dl.zfaw79
Anopheles labranchiae	-4.88333	34.9167	Unclassified	GBIF: https://doi.org/10.15468/dl.zfaw79
Anopheles labranchiae	-4.88	34.92	Unclassified	GBIF: https://doi.org/10.15468/dl.zfaw79
Anopheles labranchiae	8.5	36.85	Unclassified	GBIF: https://doi.org/10.15468/dl.zfaw79
Anopheles labranchiae	12.763658	42.98201	Unclassified	GBIF: https://doi.org/10.15468/dl.zfaw79
Anopheles laneanus	-45.52	-22.76	Unclassified	GBIF: https://doi.org/10.15468/dl.zfaw79
Anopheles laneanus	-45.515	-22.759	Unclassified	GBIF: https://doi.org/10.15468/dl.zfaw79
Anopheles lanei	-45.52417	-22.76389	Unclassified	GBIF: https://doi.org/10.15468/dl.zfaw79
Anopheles lanei	-45.52	-22.76	Unclassified	GBIF: https://doi.org/10.15468/dl.zfaw79
Anopheles leesoni	3.89922	7.43373	Unclassified	GBIF: https://doi.org/10.15468/dl.zfaw79
Anopheles leesoni	4.48256	7.7837	Unclassified	GBIF: https://doi.org/10.15468/dl.zfaw79
Anopheles leesoni	6.73259	7.80037	Unclassified	GBIF: https://doi.org/10.15468/dl.zfaw79
Anopheles leesoni	7.44927	10.50013	Unclassified	GBIF: https://doi.org/10.15468/dl.zfaw79
Anopheles leesoni	31.4567	-23.1117	Unclassified	GBIF: https://doi.org/10.15468/dl.zfaw79
Anopheles leesoni	35.3133	-21.9517	Unclassified	GBIF: https://doi.org/10.15468/dl.zfaw79
Anopheles leesoni	36.3934	-8.1756	Unclassified	GBIF: https://doi.org/10.15468/dl.zfaw79
Anopheles lepidotus	-76.145	-0.638	Unclassified	GBIF: https://doi.org/10.15468/dl.zfaw79
Anopheles lepidotus	-76.14	-0.64	Unclassified	GBIF: https://doi.org/10.15468/dl.zfaw79
Anopheles lepidotus	-73.566669	4.249604	Unclassified	GBIF: https://doi.org/10.15468/dl.zfaw79
Anopheles lepidotus	-63.89	3.28	Unclassified	GBIF: https://doi.org/10.15468/dl.zfaw79
Anopheles lesteri	81.173004	23.5863	Unclassified	GBIF: https://doi.org/10.15468/dl.zfaw79
Anopheles lesteri	97.962888	19.493886	Unclassified	GBIF: https://doi.org/10.15468/dl.zfaw79
Anopheles lesteri	98.505187	18.163134	Unclassified	GBIF: https://doi.org/10.15468/dl.zfaw79
Anopheles lesteri	98.6705	59.039433	Unclassified	GBIF: https://doi.org/10.15468/dl.zfaw79
Anopheles lesteri	98.90558	18.91308	Unclassified	GBIF: https://doi.org/10.15468/dl.zfaw79
Anopheles lesteri	99.24081	18.92017	Unclassified	GBIF: https://doi.org/10.15468/dl.zfaw79
Anopheles lesteri	99.92632	9.56368	Unclassified	GBIF: https://doi.org/10.15468/dl.zfaw79
Anopheles lesteri	99.92635	9.57272	Unclassified	GBIF: https://doi.org/10.15468/dl.zfaw79
Anopheles lesteri	99.93536	9.53653	Unclassified	GBIF: https://doi.org/10.15468/dl.zfaw79
Anopheles lesteri	99.93541	9.55461	Unclassified	GBIF: https://doi.org/10.15468/dl.zfaw79
Anopheles lesteri	101.446078	3.045163	Unclassified	GBIF: https://doi.org/10.15468/dl.zfaw79
Anopheles lesteri	104.07015	30.67004	Unclassified	GBIF: https://doi.org/10.15468/dl.zfaw79

Anopheles lesteri	105.08015	27.80004	Unclassified	GBIF: https://doi.org/10.15468/dl.zfaw79
Anopheles lesteri	109.97015	22.28004	Unclassified	GBIF: https://doi.org/10.15468/dl.zfaw79
Anopheles lesteri	110.75015	19.62004	Unclassified	GBIF: https://doi.org/10.15468/dl.zfaw79
Anopheles lesteri	111.12015	33.85004	Unclassified	GBIF: https://doi.org/10.15468/dl.zfaw79
Anopheles lesteri	113.25015	23.12004	Unclassified	GBIF: https://doi.org/10.15468/dl.zfaw79
Anopheles lesteri	113.40015	32.36004	Unclassified	GBIF: https://doi.org/10.15468/dl.zfaw79
Anopheles lesteri	113.57015	22.28004	Unclassified	GBIF: https://doi.org/10.15468/dl.zfaw79
Anopheles lesteri	114.12015	32.10004	Unclassified	GBIF: https://doi.org/10.15468/dl.zfaw79
Anopheles lesteri	116.682358	23.334454	Unclassified	GBIF: https://doi.org/10.15468/dl.zfaw79
Anopheles lesteri	117.106018	23.40497	Unclassified	GBIF: https://doi.org/10.15468/dl.zfaw79
Anopheles lesteri	118.466663	31.866665	Unclassified	GBIF: https://doi.org/10.15468/dl.zfaw79
Anopheles lesteri	119.14015	33.50004	Unclassified	GBIF: https://doi.org/10.15468/dl.zfaw79
Anopheles lesteri	120.29015	31.58004	Unclassified	GBIF: https://doi.org/10.15468/dl.zfaw79
Anopheles lesteri	121.067613	12.352748	Unclassified	GBIF: https://doi.org/10.15468/dl.zfaw79
Anopheles lesteri	121.123909	14.625601	Unclassified	GBIF: https://doi.org/10.15468/dl.zfaw79
Anopheles lesteri	121.124021	14.624775	Unclassified	GBIF: https://doi.org/10.15468/dl.zfaw79
Anopheles lesteri	121.124705	14.626286	Unclassified	GBIF: https://doi.org/10.15468/dl.zfaw79
Anopheles lesteri	121.50015	39.77003	Unclassified	GBIF: https://doi.org/10.15468/dl.zfaw79
Anopheles lesteri	122.99015	39.70003	Unclassified	GBIF: https://doi.org/10.15468/dl.zfaw79
Anopheles lesteri	123.41015	42.50003	Unclassified	GBIF: https://doi.org/10.15468/dl.zfaw79
Anopheles lesteri	123.43015	41.79003	Unclassified	GBIF: https://doi.org/10.15468/dl.zfaw79
Anopheles lesteri	124.23	24.49	Unclassified	GBIF: https://doi.org/10.15468/dl.zfaw79
Anopheles lesteri	126.63758	37.76688	Unclassified	GBIF: https://doi.org/10.15468/dl.zfaw79
Anopheles lesteri	126.65192	37.71319	Unclassified	GBIF: https://doi.org/10.15468/dl.zfaw79
Anopheles lesteri	126.69833	37.66833	Unclassified	GBIF: https://doi.org/10.15468/dl.zfaw79
Anopheles lesteri	126.71667	37.81667	Unclassified	GBIF: https://doi.org/10.15468/dl.zfaw79
Anopheles lesteri	126.72362	37.93156	Unclassified	GBIF: https://doi.org/10.15468/dl.zfaw79
Anopheles lesteri	126.75911	37.87861	Unclassified	GBIF: https://doi.org/10.15468/dl.zfaw79
Anopheles lesteri	126.76889	37.68667	Unclassified	GBIF: https://doi.org/10.15468/dl.zfaw79
Anopheles lesteri	126.80287	37.75586	Unclassified	GBIF: https://doi.org/10.15468/dl.zfaw79
Anopheles lesteri	126.85	36.96667	Unclassified	GBIF: https://doi.org/10.15468/dl.zfaw79
Anopheles lesteri	126.85611	37.98722	Unclassified	GBIF: https://doi.org/10.15468/dl.zfaw79
Anopheles lesteri	126.93	37.54972	Unclassified	GBIF: https://doi.org/10.15468/dl.zfaw79
Anopheles lesteri	127.07028	37.92639	Unclassified	GBIF: https://doi.org/10.15468/dl.zfaw79
Anopheles lesteri	127.08972	37.90417	Unclassified	GBIF: https://doi.org/10.15468/dl.zfaw79
Anopheles lesteri	127.10611	36.96861	Unclassified	GBIF: https://doi.org/10.15468/dl.zfaw79
Anopheles lesteri	127.23071	38.03663	Unclassified	GBIF: https://doi.org/10.15468/dl.zfaw79
Anopheles lesteri	127.38644	37.66653	Unclassified	GBIF: https://doi.org/10.15468/dl.zfaw79
Anopheles lesteri	127.762245	36.402386	Unclassified	GBIF: https://doi.org/10.15468/dl.zfaw79
Anopheles letifer	103.81026	1.352825	Unclassified	GBIF: https://doi.org/10.15468/dl.zfaw79
Anopheles leucosphyrus	98.70905	3.408338	Unclassified	GBIF: https://doi.org/10.15468/dl.zfaw79
Anopheles leucosphyrus	98.807778	3.531111	Unclassified	GBIF: https://doi.org/10.15468/dl.zfaw79

Anopheles leucosphyrus	99.359444	0.798056	Unclassified	GBIF: https://doi.org/10.15468/dl.zfaw79
Anopheles leucosphyrus	102.451667	-1.477222	Unclassified	GBIF: https://doi.org/10.15468/dl.zfaw79
Anopheles leucosphyrus	115.2167	5.35	Unclassified	GBIF: https://doi.org/10.15468/dl.zfaw79
Anopheles leucosphyrus	116.854444	-1.2375	Unclassified	GBIF: https://doi.org/10.15468/dl.zfaw79
Anopheles leucosphyrus	119.966516	-1.170781	Unclassified	GBIF: https://doi.org/10.15468/dl.zfaw79
Anopheles leucosphyrus	122.024251	-5.239455	Unclassified	GBIF: https://doi.org/10.15468/dl.zfaw79
Anopheles leucosphyrus	122.038574	-5.309061	Unclassified	GBIF: https://doi.org/10.15468/dl.zfaw79
Anopheles liangshanensis	98.47015	25.03004	Unclassified	GBIF: https://doi.org/10.15468/dl.zfaw79
Anopheles liangshanensis	98.68015	24.58004	Unclassified	GBIF: https://doi.org/10.15468/dl.zfaw79
Anopheles liangshanensis	102.27015	27.90004	Unclassified	GBIF: https://doi.org/10.15468/dl.zfaw79
Anopheles liangshanensis	102.85015	28.02004	Unclassified	GBIF: https://doi.org/10.15468/dl.zfaw79
Anopheles liangshanensis	104.07015	30.67004	Unclassified	GBIF: https://doi.org/10.15468/dl.zfaw79
Anopheles liangshanensis	104.69114	38.073254	Unclassified	GBIF: https://doi.org/10.15468/dl.zfaw79
Anopheles limosus	123.539197	10.785489	Unclassified	GBIF: https://doi.org/10.15468/dl.zfaw79
Anopheles lindesayi	76.95	30.8833	Unclassified	GBIF: https://doi.org/10.15468/dl.zfaw79
Anopheles lindesayi	81.173004	23.5863	Unclassified	GBIF: https://doi.org/10.15468/dl.zfaw79
Anopheles lindesayi	84.23799	28.019412	Unclassified	GBIF: https://doi.org/10.15468/dl.zfaw79
Anopheles lindesayi	84.453007	28.058517	Unclassified	GBIF: https://doi.org/10.15468/dl.zfaw79
Anopheles lindesayi	84.847769	27.584379	Unclassified	GBIF: https://doi.org/10.15468/dl.zfaw79
Anopheles lindesayi	84.848305	27.584495	Unclassified	GBIF: https://doi.org/10.15468/dl.zfaw79
Anopheles lindesayi	85.002554	27.296121	Unclassified	GBIF: https://doi.org/10.15468/dl.zfaw79
Anopheles lindesayi	85.003813	27.437133	Unclassified	GBIF: https://doi.org/10.15468/dl.zfaw79
Anopheles lindesayi	98.474611	18.549889	Unclassified	GBIF: https://doi.org/10.15468/dl.zfaw79
Anopheles lindesayi	98.476556	18.540472	Unclassified	GBIF: https://doi.org/10.15468/dl.zfaw79
Anopheles lindesayi	100.78557	16.821839	Unclassified	GBIF: https://doi.org/10.15468/dl.zfaw79
Anopheles lindesayi	104.69114	38.073254	Unclassified	GBIF: https://doi.org/10.15468/dl.zfaw79
Anopheles lindesayi	120.830977	23.632146	Unclassified	GBIF: https://doi.org/10.15468/dl.zfaw79
Anopheles lindesayi	120.96051	23.697807	Unclassified	GBIF: https://doi.org/10.15468/dl.zfaw79
Anopheles lindesayi	126.53	37.74	Unclassified	GBIF: https://doi.org/10.15468/dl.zfaw79
Anopheles lindesayi	126.82566	37.89404	Unclassified	GBIF: https://doi.org/10.15468/dl.zfaw79
Anopheles lindesayi	126.828	37.8911	Unclassified	GBIF: https://doi.org/10.15468/dl.zfaw79
Anopheles lindesayi	127.05139	37.44042	Unclassified	GBIF: https://doi.org/10.15468/dl.zfaw79
Anopheles lindesayi	127.07506	37.92381	Unclassified	GBIF: https://doi.org/10.15468/dl.zfaw79
Anopheles lindesayi	127.08917	37.90417	Unclassified	GBIF: https://doi.org/10.15468/dl.zfaw79
Anopheles lindesayi	127.26153	37.96286	Unclassified	GBIF: https://doi.org/10.15468/dl.zfaw79
Anopheles lindesayi	127.52703	38.08531	Unclassified	GBIF: https://doi.org/10.15468/dl.zfaw79
Anopheles lindesayi	127.57058	38.05442	Unclassified	GBIF: https://doi.org/10.15468/dl.zfaw79
Anopheles lindesayi	127.88678	36.44167	Unclassified	GBIF: https://doi.org/10.15468/dl.zfaw79
Anopheles lindesayi	127.95644	37.40658	Unclassified	GBIF: https://doi.org/10.15468/dl.zfaw79
Anopheles lindesayi	128.0045	36.47881	Unclassified	GBIF: https://doi.org/10.15468/dl.zfaw79
Anopheles lindesayi	135.413	34.196	Unclassified	GBIF: https://doi.org/10.15468/dl.zfaw79
Anopheles lindesayi	135.428	34.196	Unclassified	GBIF: https://doi.org/10.15468/dl.zfaw79

Anopheles lindesayi	135.768333	35.011667	Unclassified	GBIF: https://doi.org/10.15468/dl.zfaw79
Anopheles lindesayi	136.8343	36.1468	Unclassified	GBIF: https://doi.org/10.15468/dl.zfaw79
Anopheles lindesayi	137.46935	36.767387	Unclassified	GBIF: https://doi.org/10.15468/dl.zfaw79
Anopheles lindesayi	137.6215	36.0458	Unclassified	GBIF: https://doi.org/10.15468/dl.zfaw79
Anopheles lindesayi	139.04	35.86	Unclassified	GBIF: https://doi.org/10.15468/dl.zfaw79
Anopheles lindesayi	139.865	37.32	Unclassified	GBIF: https://doi.org/10.15468/dl.zfaw79
Anopheles lindesayi	139.865	37.321	Unclassified	GBIF: https://doi.org/10.15468/dl.zfaw79
Anopheles lindesayi	140.338	40.63	Unclassified	GBIF: https://doi.org/10.15468/dl.zfaw79
Anopheles listeri	18.75	-32.97	Unclassified	GBIF: https://doi.org/10.15468/dl.zfaw79
Anopheles listeri	30.45	-28.73	Unclassified	GBIF: https://doi.org/10.15468/dl.zfaw79
Anopheles litoralis	120.982559	14.700557	Unclassified	GBIF: https://doi.org/10.15468/dl.zfaw79
Anopheles litoralis	120.983441	14.700601	Unclassified	GBIF: https://doi.org/10.15468/dl.zfaw79
Anopheles litoralis	120.983785	14.700269	Unclassified	GBIF: https://doi.org/10.15468/dl.zfaw79
Anopheles litoralis	120.984128	14.700933	Unclassified	GBIF: https://doi.org/10.15468/dl.zfaw79
Anopheles litoralis	120.984341	14.701443	Unclassified	GBIF: https://doi.org/10.15468/dl.zfaw79
Anopheles litoralis	121.065918	12.347433	Unclassified	GBIF: https://doi.org/10.15468/dl.zfaw79
Anopheles litoralis	124.675556	11.299167	Unclassified	GBIF: https://doi.org/10.15468/dl.zfaw79
Anopheles longipalpis	28.2667	-23.8917	Unclassified	GBIF: https://doi.org/10.15468/dl.zfaw79
Anopheles longipalpis	35.63	0.64	Unclassified	GBIF: https://doi.org/10.15468/dl.zfaw79
Anopheles longirostris	128.4167	2.3333	Unclassified	GBIF: https://doi.org/10.15468/dl.zfaw79
Anopheles longirostris	131.255816	-0.876191	Unclassified	GBIF: https://doi.org/10.15468/dl.zfaw79
Anopheles longirostris	133.599137	-2.099277	Unclassified	GBIF: https://doi.org/10.15468/dl.zfaw79
Anopheles longirostris	136.671726	-2.845024	Unclassified	GBIF: https://doi.org/10.15468/dl.zfaw79
Anopheles longirostris	138.080353	-4.269928	Unclassified	GBIF: https://doi.org/10.15468/dl.zfaw79
Anopheles longirostris	140.276667	-6.080278	Unclassified	GBIF: https://doi.org/10.15468/dl.zfaw79
Anopheles longirostris	142.38333	-11.03333	Unclassified	GBIF: https://doi.org/10.15468/dl.zfaw79
Anopheles longirostris	143.01669	-3.8833	Unclassified	GBIF: https://doi.org/10.15468/dl.zfaw79
Anopheles longirostris	143.05	-3.66667	Unclassified	GBIF: https://doi.org/10.15468/dl.zfaw79
Anopheles longirostris	143.51669	-6.66667	Unclassified	GBIF: https://doi.org/10.15468/dl.zfaw79
Anopheles longirostris	143.6667	-3.58333	Unclassified	GBIF: https://doi.org/10.15468/dl.zfaw79
Anopheles longirostris	145.05	-7.65	Unclassified	GBIF: https://doi.org/10.15468/dl.zfaw79
Anopheles longirostris	145.67	-5.37	Unclassified	GBIF: https://doi.org/10.15468/dl.zfaw79
Anopheles longirostris	145.6833	-5.2833	Unclassified	GBIF: https://doi.org/10.15468/dl.zfaw79
Anopheles longirostris	145.71666	-16.81667	Unclassified	GBIF: https://doi.org/10.15468/dl.zfaw79
Anopheles longirostris	146.81667	-19.26667	Unclassified	GBIF: https://doi.org/10.15468/dl.zfaw79
Anopheles longirostris	146.83333	-7.21667	Unclassified	GBIF: https://doi.org/10.15468/dl.zfaw79
Anopheles longirostris	147	-6.71667	Unclassified	GBIF: https://doi.org/10.15468/dl.zfaw79
Anopheles longirostris	147.795868	-6.596245	Unclassified	GBIF: https://doi.org/10.15468/dl.zfaw79
Anopheles longirostris	147.819654	-6.594865	Unclassified	GBIF: https://doi.org/10.15468/dl.zfaw79
Anopheles longirostris	147.853302	-6.598434	Unclassified	GBIF: https://doi.org/10.15468/dl.zfaw79
Anopheles longirostris	149.565168	-10.146721	Unclassified	GBIF: https://doi.org/10.15468/dl.zfaw79
Anopheles longirostris	149.578901	-10.07412	Unclassified	GBIF: https://doi.org/10.15468/dl.zfaw79

Anopheles longirostris	149.598127	-10.081828	Unclassified	GBIF: https://doi.org/10.15468/dl.zfaw79
Anopheles longirostris	149.675036	-10.174721	Unclassified	GBIF: https://doi.org/10.15468/dl.zfaw79
Anopheles longirostris	150.33333	-10.33333	Unclassified	GBIF: https://doi.org/10.15468/dl.zfaw79
Anopheles lovettae	38.633333	-5.1	Unclassified	GBIF: https://doi.org/10.15468/dl.zfaw79
Anopheles ludlowae	95.323611	5.549444	Unclassified	GBIF: https://doi.org/10.15468/dl.zfaw79
Anopheles ludlowae	97.798611	2.283611	Unclassified	GBIF: https://doi.org/10.15468/dl.zfaw79
Anopheles ludlowae	98.688611	3.784722	Unclassified	GBIF: https://doi.org/10.15468/dl.zfaw79
Anopheles ludlowae	98.785	1.736667	Unclassified	GBIF: https://doi.org/10.15468/dl.zfaw79
Anopheles ludlowae	99.359444	0.798056	Unclassified	GBIF: https://doi.org/10.15468/dl.zfaw79
Anopheles ludlowae	100.012778	0.537222	Unclassified	GBIF: https://doi.org/10.15468/dl.zfaw79
Anopheles ludlowae	105.164167	-2.064444	Unclassified	GBIF: https://doi.org/10.15468/dl.zfaw79
Anopheles ludlowae	106.795015	-6.145732	Unclassified	GBIF: https://doi.org/10.15468/dl.zfaw79
Anopheles ludlowae	106.798653	-6.127175	Unclassified	GBIF: https://doi.org/10.15468/dl.zfaw79
Anopheles ludlowae	106.845278	-6.204167	Unclassified	GBIF: https://doi.org/10.15468/dl.zfaw79
Anopheles ludlowae	106.871328	-6.132434	Unclassified	GBIF: https://doi.org/10.15468/dl.zfaw79
Anopheles ludlowae	109.023056	-7.697778	Unclassified	GBIF: https://doi.org/10.15468/dl.zfaw79
Anopheles ludlowae	109.13714	-7.681584	Unclassified	GBIF: https://doi.org/10.15468/dl.zfaw79
Anopheles ludlowae	109.146389	-7.684283	Unclassified	GBIF: https://doi.org/10.15468/dl.zfaw79
Anopheles ludlowae	110.438056	-7.004722	Unclassified	GBIF: https://doi.org/10.15468/dl.zfaw79
Anopheles ludlowae	112.681532	-7.376416	Unclassified	GBIF: https://doi.org/10.15468/dl.zfaw79
Anopheles ludlowae	112.749444	-7.256111	Unclassified	GBIF: https://doi.org/10.15468/dl.zfaw79
Anopheles ludlowae	115.090278	-8.3325	Unclassified	GBIF: https://doi.org/10.15468/dl.zfaw79
Anopheles ludlowae	118.789356	9.919192	Unclassified	GBIF: https://doi.org/10.15468/dl.zfaw79
Anopheles ludlowae	120.089019	-4.680028	Unclassified	GBIF: https://doi.org/10.15468/dl.zfaw79
Anopheles ludlowae	120.814829	23.729419	Unclassified	GBIF: https://doi.org/10.15468/dl.zfaw79
Anopheles ludlowae	120.834043	23.680942	Unclassified	GBIF: https://doi.org/10.15468/dl.zfaw79
Anopheles ludlowae	120.838541	23.702754	Unclassified	GBIF: https://doi.org/10.15468/dl.zfaw79
Anopheles ludlowae	121.065918	12.347433	Unclassified	GBIF: https://doi.org/10.15468/dl.zfaw79
Anopheles ludlowae	121.067613	12.352748	Unclassified	GBIF: https://doi.org/10.15468/dl.zfaw79
Anopheles ludlowae	121.121442	14.624818	Unclassified	GBIF: https://doi.org/10.15468/dl.zfaw79
Anopheles ludlowae	121.123147	14.624823	Unclassified	GBIF: https://doi.org/10.15468/dl.zfaw79
Anopheles ludlowae	121.123867	14.626041	Unclassified	GBIF: https://doi.org/10.15468/dl.zfaw79
Anopheles ludlowae	121.124977	14.625484	Unclassified	GBIF: https://doi.org/10.15468/dl.zfaw79
Anopheles ludlowae	121.127447	14.625879	Unclassified	GBIF: https://doi.org/10.15468/dl.zfaw79
Anopheles lungae	155.32	-6.65	Unclassified	GBIF: https://doi.org/10.15468/dl.zfaw79
Anopheles lungae	156.66667	-7.75	Unclassified	GBIF: https://doi.org/10.15468/dl.zfaw79
Anopheles lungae	160.2	-9.5333	Unclassified	GBIF: https://doi.org/10.15468/dl.zfaw79
Anopheles lutzii	-50.22	-29.66	Unclassified	GBIF: https://doi.org/10.15468/dl.zfaw79
Anopheles lutzii	-48.8293	-25.4754	Unclassified	GBIF: https://doi.org/10.15468/dl.zfaw79
Anopheles lutzii	-47.95	-24.75	Unclassified	GBIF: https://doi.org/10.15468/dl.zfaw79
Anopheles lutzii	-47.94907	-24.74958	Unclassified	GBIF: https://doi.org/10.15468/dl.zfaw79
Anopheles lutzii	-47.57	-24.45	Unclassified	GBIF: https://doi.org/10.15468/dl.zfaw79

Anopheles lutzii	-45.59	-22.73	Unclassified	GBIF: https://doi.org/10.15468/dl.zfaw79
Anopheles lutzii	-41.7869	-22.3708	Unclassified	GBIF: https://doi.org/10.15468/dl.zfaw79
Anopheles macarthuri	98.565348	8.531644	Unclassified	GBIF: https://doi.org/10.15468/dl.zfaw79
Anopheles macarthuri	99.111026	16.427828	Unclassified	GBIF: https://doi.org/10.15468/dl.zfaw79
Anopheles macarthuri	99.37382	15.121545	Unclassified	GBIF: https://doi.org/10.15468/dl.zfaw79
Anopheles macarthuri	99.580969	20.243946	Unclassified	GBIF: https://doi.org/10.15468/dl.zfaw79
Anopheles macarthuri	99.73925	8.44532	Unclassified	GBIF: https://doi.org/10.15468/dl.zfaw79
Anopheles macarthuri	99.75069	7.92094	Unclassified	GBIF: https://doi.org/10.15468/dl.zfaw79
Anopheles macarthuri	99.75597	8.43731	Unclassified	GBIF: https://doi.org/10.15468/dl.zfaw79
Anopheles macarthuri	99.77269	8.42931	Unclassified	GBIF: https://doi.org/10.15468/dl.zfaw79
Anopheles macarthuri	101.30242	6.25942	Unclassified	GBIF: https://doi.org/10.15468/dl.zfaw79
Anopheles macarthuri	101.34375	6.2512	Unclassified	GBIF: https://doi.org/10.15468/dl.zfaw79
Anopheles macarthuri	101.36853	6.2595	Unclassified	GBIF: https://doi.org/10.15468/dl.zfaw79
Anopheles macarthuri	101.38507	6.25125	Unclassified	GBIF: https://doi.org/10.15468/dl.zfaw79
Anopheles macarthuri	101.637721	6.030815	Unclassified	GBIF: https://doi.org/10.15468/dl.zfaw79
Anopheles macarthuri	101.77356	5.94562	Unclassified	GBIF: https://doi.org/10.15468/dl.zfaw79
Anopheles macarthuri	101.79008	5.94563	Unclassified	GBIF: https://doi.org/10.15468/dl.zfaw79
Anopheles macarthuri	101.80659	5.94563	Unclassified	GBIF: https://doi.org/10.15468/dl.zfaw79
Anopheles macarthuri	102.287214	12.881731	Unclassified	GBIF: https://doi.org/10.15468/dl.zfaw79
Anopheles macarthuri	102.817793	11.873617	Unclassified	GBIF: https://doi.org/10.15468/dl.zfaw79
Anopheles macarthuri	105.192871	14.453702	Unclassified	GBIF: https://doi.org/10.15468/dl.zfaw79
Anopheles macarthuri	117.6917	4.949	Unclassified	GBIF: https://doi.org/10.15468/dl.zfaw79
Anopheles maculatus	-84.4	33.77	Unclassified	GBIF: https://doi.org/10.15468/dl.zfaw79
Anopheles maculatus	71.133331	32.849998	Unclassified	GBIF: https://doi.org/10.15468/dl.zfaw79
Anopheles maculatus	72.550003	35.049999	Unclassified	GBIF: https://doi.org/10.15468/dl.zfaw79
Anopheles maculatus	80.66931	7.696631	Unclassified	GBIF: https://doi.org/10.15468/dl.zfaw79
Anopheles maculatus	92.66	11.74	Unclassified	GBIF: https://doi.org/10.15468/dl.zfaw79
Anopheles maculatus	95.31	5.83	Unclassified	GBIF: https://doi.org/10.15468/dl.zfaw79
Anopheles maculatus	95.847212	27.329005	Unclassified	GBIF: https://doi.org/10.15468/dl.zfaw79
Anopheles maculatus	97.95596	18.29656	Unclassified	GBIF: https://doi.org/10.15468/dl.zfaw79
Anopheles maculatus	97.96	19.3	Unclassified	GBIF: https://doi.org/10.15468/dl.zfaw79
Anopheles maculatus	97.96386	18.55778	Unclassified	GBIF: https://doi.org/10.15468/dl.zfaw79
Anopheles maculatus	98.04195	18.14338	Unclassified	GBIF: https://doi.org/10.15468/dl.zfaw79
Anopheles maculatus	98.0871	18.54576	Unclassified	GBIF: https://doi.org/10.15468/dl.zfaw79
Anopheles maculatus	98.08935	18.11649	Unclassified	GBIF: https://doi.org/10.15468/dl.zfaw79
Anopheles maculatus	98.37818	18.49362	Unclassified	GBIF: https://doi.org/10.15468/dl.zfaw79
Anopheles maculatus	98.503333	3.195	Unclassified	GBIF: https://doi.org/10.15468/dl.zfaw79
Anopheles maculatus	98.584222	18.513639	Unclassified	GBIF: https://doi.org/10.15468/dl.zfaw79
Anopheles maculatus	98.594444	18.513111	Unclassified	GBIF: https://doi.org/10.15468/dl.zfaw79
Anopheles maculatus	98.66423	18.50429	Unclassified	GBIF: https://doi.org/10.15468/dl.zfaw79
Anopheles maculatus	98.67941	18.49347	Unclassified	GBIF: https://doi.org/10.15468/dl.zfaw79
Anopheles maculatus	98.785	1.736667	Unclassified	GBIF: https://doi.org/10.15468/dl.zfaw79

Anopheles maculatus	98.8133	8.855	Unclassified	GBIF: https://doi.org/10.15468/dl.zfaw79
Anopheles maculatus	98.83937	18.789043	Unclassified	GBIF: https://doi.org/10.15468/dl.zfaw79
Anopheles maculatus	98.844978	18.71642	Unclassified	GBIF: https://doi.org/10.15468/dl.zfaw79
Anopheles maculatus	98.89989	18.90584	Unclassified	GBIF: https://doi.org/10.15468/dl.zfaw79
Anopheles maculatus	98.90083	18.90584	Unclassified	GBIF: https://doi.org/10.15468/dl.zfaw79
Anopheles maculatus	98.90558	18.91308	Unclassified	GBIF: https://doi.org/10.15468/dl.zfaw79
Anopheles maculatus	98.90558	18.91036	Unclassified	GBIF: https://doi.org/10.15468/dl.zfaw79
Anopheles maculatus	98.94046	16.83508	Unclassified	GBIF: https://doi.org/10.15468/dl.zfaw79
Anopheles maculatus	98.94834	18.81187	Unclassified	GBIF: https://doi.org/10.15468/dl.zfaw79
Anopheles maculatus	98.9533	9.03	Unclassified	GBIF: https://doi.org/10.15468/dl.zfaw79
Anopheles maculatus	98.97827	14.30367	Unclassified	GBIF: https://doi.org/10.15468/dl.zfaw79
Anopheles maculatus	98.97828	14.28559	Unclassified	GBIF: https://doi.org/10.15468/dl.zfaw79
Anopheles maculatus	98.9789	9.23889	Unclassified	GBIF: https://doi.org/10.15468/dl.zfaw79
Anopheles maculatus	98.98257	19.16918	Unclassified	GBIF: https://doi.org/10.15468/dl.zfaw79
Anopheles maculatus	98.988493	18.797168	Unclassified	GBIF: https://doi.org/10.15468/dl.zfaw79
Anopheles maculatus	99.034395	10.322782	Unclassified	GBIF: https://doi.org/10.15468/dl.zfaw79
Anopheles maculatus	99.0437	16.79892	Unclassified	GBIF: https://doi.org/10.15468/dl.zfaw79
Anopheles maculatus	99.05309	16.80344	Unclassified	GBIF: https://doi.org/10.15468/dl.zfaw79
Anopheles maculatus	99.06171	14.24037	Unclassified	GBIF: https://doi.org/10.15468/dl.zfaw79
Anopheles maculatus	99.06171	14.24941	Unclassified	GBIF: https://doi.org/10.15468/dl.zfaw79
Anopheles maculatus	99.077778	1.559722	Unclassified	GBIF: https://doi.org/10.15468/dl.zfaw79
Anopheles maculatus	99.08024	14.22228	Unclassified	GBIF: https://doi.org/10.15468/dl.zfaw79
Anopheles maculatus	99.130313	10.358031	Unclassified	GBIF: https://doi.org/10.15468/dl.zfaw79
Anopheles maculatus	99.239482	10.955789	Unclassified	GBIF: https://doi.org/10.15468/dl.zfaw79
Anopheles maculatus	99.24081	18.92017	Unclassified	GBIF: https://doi.org/10.15468/dl.zfaw79
Anopheles maculatus	99.276019	10.901084	Unclassified	GBIF: https://doi.org/10.15468/dl.zfaw79
Anopheles maculatus	99.359444	0.798056	Unclassified	GBIF: https://doi.org/10.15468/dl.zfaw79
Anopheles maculatus	99.76886	8.14009	Unclassified	GBIF: https://doi.org/10.15468/dl.zfaw79
Anopheles maculatus	99.80954	19.09659	Unclassified	GBIF: https://doi.org/10.15468/dl.zfaw79
Anopheles maculatus	99.89309	18.72027	Unclassified	GBIF: https://doi.org/10.15468/dl.zfaw79
Anopheles maculatus	99.8965	19.00492	Unclassified	GBIF: https://doi.org/10.15468/dl.zfaw79
Anopheles maculatus	99.90211	18.98772	Unclassified	GBIF: https://doi.org/10.15468/dl.zfaw79
Anopheles maculatus	99.93536	9.53653	Unclassified	GBIF: https://doi.org/10.15468/dl.zfaw79
Anopheles maculatus	99.93539	9.54557	Unclassified	GBIF: https://doi.org/10.15468/dl.zfaw79
Anopheles maculatus	99.93694	18.92971	Unclassified	GBIF: https://doi.org/10.15468/dl.zfaw79
Anopheles maculatus	99.93699	18.93875	Unclassified	GBIF: https://doi.org/10.15468/dl.zfaw79
Anopheles maculatus	99.94447	9.5365	Unclassified	GBIF: https://doi.org/10.15468/dl.zfaw79
Anopheles maculatus	99.94543	15.59635	Unclassified	GBIF: https://doi.org/10.15468/dl.zfaw79
Anopheles maculatus	99.96259	9.50028	Unclassified	GBIF: https://doi.org/10.15468/dl.zfaw79
Anopheles maculatus	99.96629	9.52107	Unclassified	GBIF: https://doi.org/10.15468/dl.zfaw79
Anopheles maculatus	99.97175	9.51834	Unclassified	GBIF: https://doi.org/10.15468/dl.zfaw79
Anopheles maculatus	99.98086	9.51831	Unclassified	GBIF: https://doi.org/10.15468/dl.zfaw79

Anopheles maculatus	99.98345	9.46857	Unclassified	GBIF: https://doi.org/10.15468/dl.zfaw79
Anopheles maculatus	99.98346	9.47038	Unclassified	GBIF: https://doi.org/10.15468/dl.zfaw79
Anopheles maculatus	99.98439	9.47851	Unclassified	GBIF: https://doi.org/10.15468/dl.zfaw79
Anopheles maculatus	99.98904	9.50925	Unclassified	GBIF: https://doi.org/10.15468/dl.zfaw79
Anopheles maculatus	99.98906	9.51648	Unclassified	GBIF: https://doi.org/10.15468/dl.zfaw79
Anopheles maculatus	99.98987	9.48211	Unclassified	GBIF: https://doi.org/10.15468/dl.zfaw79
Anopheles maculatus	99.98989	9.49116	Unclassified	GBIF: https://doi.org/10.15468/dl.zfaw79
Anopheles maculatus	100.00801	9.45493	Unclassified	GBIF: https://doi.org/10.15468/dl.zfaw79
Anopheles maculatus	100.00803	9.46397	Unclassified	GBIF: https://doi.org/10.15468/dl.zfaw79
Anopheles maculatus	100.00805	9.4685	Unclassified	GBIF: https://doi.org/10.15468/dl.zfaw79
Anopheles maculatus	100.012778	0.537222	Unclassified	GBIF: https://doi.org/10.15468/dl.zfaw79
Anopheles maculatus	100.03533	9.45485	Unclassified	GBIF: https://doi.org/10.15468/dl.zfaw79
Anopheles maculatus	100.05447	9.75323	Unclassified	GBIF: https://doi.org/10.15468/dl.zfaw79
Anopheles maculatus	100.129167	0.186111	Unclassified	GBIF: https://doi.org/10.15468/dl.zfaw79
Anopheles maculatus	100.3675	-0.300278	Unclassified	GBIF: https://doi.org/10.15468/dl.zfaw79
Anopheles maculatus	100.4927	18.42009	Unclassified	GBIF: https://doi.org/10.15468/dl.zfaw79
Anopheles maculatus	100.5788	18.51878	Unclassified	GBIF: https://doi.org/10.15468/dl.zfaw79
Anopheles maculatus	100.58727	18.41028	Unclassified	GBIF: https://doi.org/10.15468/dl.zfaw79
Anopheles maculatus	100.60612	18.40109	Unclassified	GBIF: https://doi.org/10.15468/dl.zfaw79
Anopheles maculatus	100.6073	18.52757	Unclassified	GBIF: https://doi.org/10.15468/dl.zfaw79
Anopheles maculatus	100.63537	18.49119	Unclassified	GBIF: https://doi.org/10.15468/dl.zfaw79
Anopheles maculatus	100.85772	14.66424	Unclassified	GBIF: https://doi.org/10.15468/dl.zfaw79
Anopheles maculatus	100.93455	13.19321	Unclassified	GBIF: https://doi.org/10.15468/dl.zfaw79
Anopheles maculatus	100.95415	14.01007	Unclassified	GBIF: https://doi.org/10.15468/dl.zfaw79
Anopheles maculatus	101.05673	14.60383	Unclassified	GBIF: https://doi.org/10.15468/dl.zfaw79
Anopheles maculatus	101.61	3.109	Unclassified	GBIF: https://doi.org/10.15468/dl.zfaw79
Anopheles maculatus	101.86975	14.2683	Unclassified	GBIF: https://doi.org/10.15468/dl.zfaw79
Anopheles maculatus	101.8966	14.19572	Unclassified	GBIF: https://doi.org/10.15468/dl.zfaw79
Anopheles maculatus	101.90621	14.2227	Unclassified	GBIF: https://doi.org/10.15468/dl.zfaw79
Anopheles maculatus	101.92694	14.39404	Unclassified	GBIF: https://doi.org/10.15468/dl.zfaw79
Anopheles maculatus	102.26657	16.75379	Unclassified	GBIF: https://doi.org/10.15468/dl.zfaw79
Anopheles maculatus	102.34293	16.65544	Unclassified	GBIF: https://doi.org/10.15468/dl.zfaw79
Anopheles maculatus	102.35176	16.72826	Unclassified	GBIF: https://doi.org/10.15468/dl.zfaw79
Anopheles maculatus	102.35635	16.63303	Unclassified	GBIF: https://doi.org/10.15468/dl.zfaw79
Anopheles maculatus	103.57	12.31	Unclassified	GBIF: https://doi.org/10.15468/dl.zfaw79
Anopheles maculatus	104.69114	38.073254	Unclassified	GBIF: https://doi.org/10.15468/dl.zfaw79
Anopheles maculatus	106.78333	-6.58333	Unclassified	GBIF: https://doi.org/10.15468/dl.zfaw79
Anopheles maculatus	106.8325	-6.152778	Unclassified	GBIF: https://doi.org/10.15468/dl.zfaw79
Anopheles maculatus	106.932333	-6.680078	Unclassified	GBIF: https://doi.org/10.15468/dl.zfaw79
Anopheles maculatus	107.616397	-6.874231	Unclassified	GBIF: https://doi.org/10.15468/dl.zfaw79
Anopheles maculatus	108.53	12.16	Unclassified	GBIF: https://doi.org/10.15468/dl.zfaw79
Anopheles maculatus	108.59	13.37	Unclassified	GBIF: https://doi.org/10.15468/dl.zfaw79

Anopheles maculatus	109.02	13.22	Unclassified	GBIF: https://doi.org/10.15468/dl.zfaw79
Anopheles maculatus	109.173139	-7.53588	Unclassified	GBIF: https://doi.org/10.15468/dl.zfaw79
Anopheles maculatus	110.05	-7.75	Unclassified	GBIF: https://doi.org/10.15468/dl.zfaw79
Anopheles maculatus	110.333	1.4167	Unclassified	GBIF: https://doi.org/10.15468/dl.zfaw79
Anopheles maculatus	110.49	-7.34	Unclassified	GBIF: https://doi.org/10.15468/dl.zfaw79
Anopheles maculatus	110.563056	-7.698333	Unclassified	GBIF: https://doi.org/10.15468/dl.zfaw79
Anopheles maculatus	115.2167	5.35	Unclassified	GBIF: https://doi.org/10.15468/dl.zfaw79
Anopheles maculatus	116.682358	23.334454	Unclassified	GBIF: https://doi.org/10.15468/dl.zfaw79
Anopheles maculatus	116.79	6.77	Unclassified	GBIF: https://doi.org/10.15468/dl.zfaw79
Anopheles maculatus	117.87	4.16	Unclassified	GBIF: https://doi.org/10.15468/dl.zfaw79
Anopheles maculatus	119.42	-9.64	Unclassified	GBIF: https://doi.org/10.15468/dl.zfaw79
Anopheles maculatus	119.52	-1.04	Unclassified	GBIF: https://doi.org/10.15468/dl.zfaw79
Anopheles maculatus	119.7	-5.15	Unclassified	GBIF: https://doi.org/10.15468/dl.zfaw79
Anopheles maculatus	119.85	-0.64	Unclassified	GBIF: https://doi.org/10.15468/dl.zfaw79
Anopheles maculatus	120.467506	-8.612898	Unclassified	GBIF: https://doi.org/10.15468/dl.zfaw79
Anopheles maculatus	120.6	16.41667	Unclassified	GBIF: https://doi.org/10.15468/dl.zfaw79
Anopheles maculatus	120.810688	23.697086	Unclassified	GBIF: https://doi.org/10.15468/dl.zfaw79
Anopheles maculatus	120.830624	23.740411	Unclassified	GBIF: https://doi.org/10.15468/dl.zfaw79
Anopheles maculatus	120.845318	23.703724	Unclassified	GBIF: https://doi.org/10.15468/dl.zfaw79
Anopheles maculatus	120.866944	23.816303	Unclassified	GBIF: https://doi.org/10.15468/dl.zfaw79
Anopheles maculatus	120.8706	23.727738	Unclassified	GBIF: https://doi.org/10.15468/dl.zfaw79
Anopheles maculatus	120.876254	23.668577	Unclassified	GBIF: https://doi.org/10.15468/dl.zfaw79
Anopheles maculatus	120.879555	23.671002	Unclassified	GBIF: https://doi.org/10.15468/dl.zfaw79
Anopheles maculatus	121.065918	12.347433	Unclassified	GBIF: https://doi.org/10.15468/dl.zfaw79
Anopheles maculatus	121.121337	14.624184	Unclassified	GBIF: https://doi.org/10.15468/dl.zfaw79
Anopheles maculatus	121.124389	14.625048	Unclassified	GBIF: https://doi.org/10.15468/dl.zfaw79
Anopheles maculatus	121.252199	16.568399	Unclassified	GBIF: https://doi.org/10.15468/dl.zfaw79
Anopheles maculatus	123.606667	-10.173333	Unclassified	GBIF: https://doi.org/10.15468/dl.zfaw79
Anopheles maculatus	124.5	11.366667	Unclassified	GBIF: https://doi.org/10.15468/dl.zfaw79
Anopheles maculatus	124.757122	11.243803	Unclassified	GBIF: https://doi.org/10.15468/dl.zfaw79
Anopheles maculipalpis	3.93255	6.81712	Unclassified	GBIF: https://doi.org/10.15468/dl.zfaw79
Anopheles maculipalpis	4.18254	12.46661	Unclassified	GBIF: https://doi.org/10.15468/dl.zfaw79
Anopheles maculipalpis	4.58256	8.48364	Unclassified	GBIF: https://doi.org/10.15468/dl.zfaw79
Anopheles maculipalpis	5.24922	13.01656	Unclassified	GBIF: https://doi.org/10.15468/dl.zfaw79
Anopheles maculipalpis	6.16591	9.81685	Unclassified	GBIF: https://doi.org/10.15468/dl.zfaw79
Anopheles maculipalpis	6.73259	7.80037	Unclassified	GBIF: https://doi.org/10.15468/dl.zfaw79
Anopheles maculipalpis	7.16593	9.16691	Unclassified	GBIF: https://doi.org/10.15468/dl.zfaw79
Anopheles maculipalpis	7.25	10.25	Unclassified	GBIF: https://doi.org/10.15468/dl.zfaw79
Anopheles maculipalpis	7.44927	10.50013	Unclassified	GBIF: https://doi.org/10.15468/dl.zfaw79
Anopheles maculipalpis	7.4826	10.63345	Unclassified	GBIF: https://doi.org/10.15468/dl.zfaw79
Anopheles maculipalpis	7.54928	6.4505	Unclassified	GBIF: https://doi.org/10.15468/dl.zfaw79
Anopheles maculipalpis	7.86594	8.83361	Unclassified	GBIF: https://doi.org/10.15468/dl.zfaw79

Anopheles maculipalpis	8.36595	10.95009	Unclassified	GBIF: https://doi.org/10.15468/dl.zfaw79
Anopheles maculipalpis	8.61596	7.68372	Unclassified	GBIF: https://doi.org/10.15468/dl.zfaw79
Anopheles maculipalpis	8.89929	9.86685	Unclassified	GBIF: https://doi.org/10.15468/dl.zfaw79
Anopheles maculipalpis	11.59	-0.19	Unclassified	GBIF: https://doi.org/10.15468/dl.zfaw79
Anopheles maculipalpis	11.59327	-0.19124	Unclassified	GBIF: https://doi.org/10.15468/dl.zfaw79
Anopheles maculipalpis	11.6	-0.2	Unclassified	GBIF: https://doi.org/10.15468/dl.zfaw79
Anopheles maculipalpis	12.48269	9.23358	Unclassified	GBIF: https://doi.org/10.15468/dl.zfaw79
Anopheles maculipalpis	28.82	-2.24	Unclassified	GBIF: https://doi.org/10.15468/dl.zfaw79
Anopheles maculipalpis	28.822	2.243	Unclassified	GBIF: https://doi.org/10.15468/dl.zfaw79
Anopheles maculipalpis	30.15	-23.833333	Unclassified	GBIF: https://doi.org/10.15468/dl.zfaw79
Anopheles maculipalpis	33.473	-9.6963	Unclassified	GBIF: https://doi.org/10.15468/dl.zfaw79
Anopheles maculipalpis	33.5761	-12.6655	Unclassified	GBIF: https://doi.org/10.15468/dl.zfaw79
Anopheles maculipalpis	34.0055	-13.9541	Unclassified	GBIF: https://doi.org/10.15468/dl.zfaw79
Anopheles maculipalpis	35.528348	-14.871516	Unclassified	GBIF: https://doi.org/10.15468/dl.zfaw79
Anopheles maculipalpis	45.14743	-12.8206	Unclassified	GBIF: https://doi.org/10.15468/dl.zfaw79
Anopheles maculipalpis	46.366667	-19.583333	Unclassified	GBIF: https://doi.org/10.15468/dl.zfaw79
Anopheles maculipennis	-115.766667	49.516667	Unclassified	GBIF: https://doi.org/10.15468/dl.zfaw79
Anopheles maculipennis	-101.609444	19.515833	Unclassified	GBIF: https://doi.org/10.15468/dl.zfaw79
Anopheles maculipennis	-101.066667	20.372222	Unclassified	GBIF: https://doi.org/10.15468/dl.zfaw79
Anopheles maculipennis	-99.60167	49.70833	Unclassified	GBIF: https://doi.org/10.15468/dl.zfaw79
Anopheles maculipennis	-99.183333	19.482778	Unclassified	GBIF: https://doi.org/10.15468/dl.zfaw79
Anopheles maculipennis	-99.093055	19.358333	Unclassified	GBIF: https://doi.org/10.15468/dl.zfaw79
Anopheles maculipennis	-89.71083	45.87108	Unclassified	GBIF: https://doi.org/10.15468/dl.zfaw79
Anopheles maculipennis	-87.6075	45.415278	Unclassified	GBIF: https://doi.org/10.15468/dl.zfaw79
Anopheles maculipennis	-85.50028	44.25028	Unclassified	GBIF: https://doi.org/10.15468/dl.zfaw79
Anopheles maculipennis	-84.651944	44.298333	Unclassified	GBIF: https://doi.org/10.15468/dl.zfaw79
Anopheles maculipennis	-84.55	42.7167	Unclassified	GBIF: https://doi.org/10.15468/dl.zfaw79
Anopheles maculipennis	-84.478587	42.730868	Unclassified	GBIF: https://doi.org/10.15468/dl.zfaw79
Anopheles maculipennis	-84.474444	45.646944	Unclassified	GBIF: https://doi.org/10.15468/dl.zfaw79
Anopheles maculipennis	-74.44389	43.85528	Unclassified	GBIF: https://doi.org/10.15468/dl.zfaw79
Anopheles maculipennis	-6.372222	39.475278	Unclassified	GBIF: https://doi.org/10.15468/dl.zfaw79
Anopheles maculipennis	-6.230278	39.864722	Unclassified	GBIF: https://doi.org/10.15468/dl.zfaw79
Anopheles maculipennis	-4.830278	39.962778	Unclassified	GBIF: https://doi.org/10.15468/dl.zfaw79
Anopheles maculipennis	-4.779167	37.888611	Unclassified	GBIF: https://doi.org/10.15468/dl.zfaw79
Anopheles maculipennis	-3.82324	50.05436	Unclassified	GBIF: https://doi.org/10.15468/dl.zfaw79
Anopheles maculipennis	-3.82316	50.05439	Unclassified	GBIF: https://doi.org/10.15468/dl.zfaw79
Anopheles maculipennis	-3.622778	37.025	Unclassified	GBIF: https://doi.org/10.15468/dl.zfaw79
Anopheles maculipennis	-2.50066	50.92697	Unclassified	GBIF: https://doi.org/10.15468/dl.zfaw79
Anopheles maculipennis	-2.50056	50.92691	Unclassified	GBIF: https://doi.org/10.15468/dl.zfaw79
Anopheles maculipennis	-2.50013	50.92692	Unclassified	GBIF: https://doi.org/10.15468/dl.zfaw79
Anopheles maculipennis	-1.32386	45.97605	Unclassified	GBIF: https://doi.org/10.15468/dl.zfaw79
Anopheles maculipennis	-1.30563	45.93966	Unclassified	GBIF: https://doi.org/10.15468/dl.zfaw79

Anopheles maculipennis	-1.25749	45.90998	Unclassified	GBIF: https://doi.org/10.15468/dl.zfaw79
Anopheles maculipennis	-1.24107	45.8623	Unclassified	GBIF: https://doi.org/10.15468/dl.zfaw79
Anopheles maculipennis	-1.21646	45.88134	Unclassified	GBIF: https://doi.org/10.15468/dl.zfaw79
Anopheles maculipennis	-1.19082	45.75932	Unclassified	GBIF: https://doi.org/10.15468/dl.zfaw79
Anopheles maculipennis	-1.1735	46.16242	Unclassified	GBIF: https://doi.org/10.15468/dl.zfaw79
Anopheles maculipennis	-1.16992	45.70639	Unclassified	GBIF: https://doi.org/10.15468/dl.zfaw79
Anopheles maculipennis	-1.16425	46.45106	Unclassified	GBIF: https://doi.org/10.15468/dl.zfaw79
Anopheles maculipennis	-1.11418	46.13445	Unclassified	GBIF: https://doi.org/10.15468/dl.zfaw79
Anopheles maculipennis	-1.10572	46.10681	Unclassified	GBIF: https://doi.org/10.15468/dl.zfaw79
Anopheles maculipennis	-1.08866	46.15805	Unclassified	GBIF: https://doi.org/10.15468/dl.zfaw79
Anopheles maculipennis	-1.08303	46.07222	Unclassified	GBIF: https://doi.org/10.15468/dl.zfaw79
Anopheles maculipennis	-1.07835	45.94427	Unclassified	GBIF: https://doi.org/10.15468/dl.zfaw79
Anopheles maculipennis	-1.07453	45.83521	Unclassified	GBIF: https://doi.org/10.15468/dl.zfaw79
Anopheles maculipennis	-1.05406	45.79461	Unclassified	GBIF: https://doi.org/10.15468/dl.zfaw79
Anopheles maculipennis	-1.05196	45.88864	Unclassified	GBIF: https://doi.org/10.15468/dl.zfaw79
Anopheles maculipennis	-1.03464	46.03977	Unclassified	GBIF: https://doi.org/10.15468/dl.zfaw79
Anopheles maculipennis	-1.028	45.7564	Unclassified	GBIF: https://doi.org/10.15468/dl.zfaw79
Anopheles maculipennis	-1.02777	45.9435	Unclassified	GBIF: https://doi.org/10.15468/dl.zfaw79
Anopheles maculipennis	-1.02738	45.9882	Unclassified	GBIF: https://doi.org/10.15468/dl.zfaw79
Anopheles maculipennis	-1.00625	45.67339	Unclassified	GBIF: https://doi.org/10.15468/dl.zfaw79
Anopheles maculipennis	-1.00276	45.90977	Unclassified	GBIF: https://doi.org/10.15468/dl.zfaw79
Anopheles maculipennis	-0.98648	45.97359	Unclassified	GBIF: https://doi.org/10.15468/dl.zfaw79
Anopheles maculipennis	-0.97958	46.3119	Unclassified	GBIF: https://doi.org/10.15468/dl.zfaw79
Anopheles maculipennis	-0.9722	45.94288	Unclassified	GBIF: https://doi.org/10.15468/dl.zfaw79
Anopheles maculipennis	-0.95335	45.8676	Unclassified	GBIF: https://doi.org/10.15468/dl.zfaw79
Anopheles maculipennis	-0.893554	35.704773	Unclassified	GBIF: https://doi.org/10.15468/dl.zfaw79
Anopheles maculipennis	-0.88574	45.55691	Unclassified	GBIF: https://doi.org/10.15468/dl.zfaw79
Anopheles maculipennis	-0.87454	45.96376	Unclassified	GBIF: https://doi.org/10.15468/dl.zfaw79
Anopheles maculipennis	-0.804254	51.089088	Unclassified	GBIF: https://doi.org/10.15468/dl.zfaw79
Anopheles maculipennis	-0.71381	45.87244	Unclassified	GBIF: https://doi.org/10.15468/dl.zfaw79
Anopheles maculipennis	-0.67722	45.88655	Unclassified	GBIF: https://doi.org/10.15468/dl.zfaw79
Anopheles maculipennis	-0.38754	46.07346	Unclassified	GBIF: https://doi.org/10.15468/dl.zfaw79
Anopheles maculipennis	0.226583	50.943389	Unclassified	GBIF: https://doi.org/10.15468/dl.zfaw79
Anopheles maculipennis	0.281957	52.400172	Unclassified	GBIF: https://doi.org/10.15468/dl.zfaw79
Anopheles maculipennis	0.37832	50.93337	Unclassified	GBIF: https://doi.org/10.15468/dl.zfaw79
Anopheles maculipennis	0.37876	50.93582	Unclassified	GBIF: https://doi.org/10.15468/dl.zfaw79
Anopheles maculipennis	0.37943	50.93471	Unclassified	GBIF: https://doi.org/10.15468/dl.zfaw79
Anopheles maculipennis	0.91149	45.27581	Unclassified	GBIF: https://doi.org/10.15468/dl.zfaw79
Anopheles maculipennis	1.04461	43.20719	Unclassified	GBIF: https://doi.org/10.15468/dl.zfaw79
Anopheles maculipennis	1.68143	44.62284	Unclassified	GBIF: https://doi.org/10.15468/dl.zfaw79
Anopheles maculipennis	1.70038	44.60754	Unclassified	GBIF: https://doi.org/10.15468/dl.zfaw79
Anopheles maculipennis	1.90291	52.62565	Unclassified	GBIF: https://doi.org/10.15468/dl.zfaw79

Anopheles maculipennis	1.90292	52.62567	Unclassified	GBIF: https://doi.org/10.15468/dl.zfaw79
Anopheles maculipennis	1.90297	52.62567	Unclassified	GBIF: https://doi.org/10.15468/dl.zfaw79
Anopheles maculipennis	1.945312	27.922369	Unclassified	GBIF: https://doi.org/10.15468/dl.zfaw79
Anopheles maculipennis	2.045278	49.413612	Unclassified	GBIF: https://doi.org/10.15468/dl.zfaw79
Anopheles maculipennis	2.3227	50.00488	Unclassified	GBIF: https://doi.org/10.15468/dl.zfaw79
Anopheles maculipennis	2.384766	28	Unclassified	GBIF: https://doi.org/10.15468/dl.zfaw79
Anopheles maculipennis	2.474722	48.90361	Unclassified	GBIF: https://doi.org/10.15468/dl.zfaw79
Anopheles maculipennis	2.74068	51.041939	Unclassified	GBIF: https://doi.org/10.15468/dl.zfaw79
Anopheles maculipennis	2.91382	48.12171	Unclassified	GBIF: https://doi.org/10.15468/dl.zfaw79
Anopheles maculipennis	3	28	Unclassified	GBIF: https://doi.org/10.15468/dl.zfaw79
Anopheles maculipennis	3.02395	42.47816	Unclassified	GBIF: https://doi.org/10.15468/dl.zfaw79
Anopheles maculipennis	3.1699	50.76562	Unclassified	GBIF: https://doi.org/10.15468/dl.zfaw79
Anopheles maculipennis	3.251229	51.338514	Unclassified	GBIF: https://doi.org/10.15468/dl.zfaw79
Anopheles maculipennis	3.27088	50.833752	Unclassified	GBIF: https://doi.org/10.15468/dl.zfaw79
Anopheles maculipennis	3.27243	51.27351	Unclassified	GBIF: https://doi.org/10.15468/dl.zfaw79
Anopheles maculipennis	3.41928	50.638851	Unclassified	GBIF: https://doi.org/10.15468/dl.zfaw79
Anopheles maculipennis	3.59848	50.762798	Unclassified	GBIF: https://doi.org/10.15468/dl.zfaw79
Anopheles maculipennis	3.65568	50.48383	Unclassified	GBIF: https://doi.org/10.15468/dl.zfaw79
Anopheles maculipennis	3.677257	51.022093	Unclassified	GBIF: https://doi.org/10.15468/dl.zfaw79
Anopheles maculipennis	3.69062	50.44035	Unclassified	GBIF: https://doi.org/10.15468/dl.zfaw79
Anopheles maculipennis	3.74423	50.751901	Unclassified	GBIF: https://doi.org/10.15468/dl.zfaw79
Anopheles maculipennis	3.749266	51.066617	Unclassified	GBIF: https://doi.org/10.15468/dl.zfaw79
Anopheles maculipennis	3.75315	49.89022	Unclassified	GBIF: https://doi.org/10.15468/dl.zfaw79
Anopheles maculipennis	3.819823	51.02118	Unclassified	GBIF: https://doi.org/10.15468/dl.zfaw79
Anopheles maculipennis	3.820617	51.066137	Unclassified	GBIF: https://doi.org/10.15468/dl.zfaw79
Anopheles maculipennis	3.83611	51.11228	Unclassified	GBIF: https://doi.org/10.15468/dl.zfaw79
Anopheles maculipennis	3.83629	50.864208	Unclassified	GBIF: https://doi.org/10.15468/dl.zfaw79
Anopheles maculipennis	3.92491	50.41203	Unclassified	GBIF: https://doi.org/10.15468/dl.zfaw79
Anopheles maculipennis	3.963313	51.065049	Unclassified	GBIF: https://doi.org/10.15468/dl.zfaw79
Anopheles maculipennis	4.06593	51.042309	Unclassified	GBIF: https://doi.org/10.15468/dl.zfaw79
Anopheles maculipennis	4.180249	51.704616	Unclassified	GBIF: https://doi.org/10.15468/dl.zfaw79
Anopheles maculipennis	4.1935	51.21351	Unclassified	GBIF: https://doi.org/10.15468/dl.zfaw79
Anopheles maculipennis	4.19417	51.10524	Unclassified	GBIF: https://doi.org/10.15468/dl.zfaw79
Anopheles maculipennis	4.20551	51.06319	Unclassified	GBIF: https://doi.org/10.15468/dl.zfaw79
Anopheles maculipennis	4.20923	50.56591	Unclassified	GBIF: https://doi.org/10.15468/dl.zfaw79
Anopheles maculipennis	4.21772	51.25156	Unclassified	GBIF: https://doi.org/10.15468/dl.zfaw79
Anopheles maculipennis	4.246791	51.667168	Unclassified	GBIF: https://doi.org/10.15468/dl.zfaw79
Anopheles maculipennis	4.25157	51.361626	Unclassified	GBIF: https://doi.org/10.15468/dl.zfaw79
Anopheles maculipennis	4.2765	51.27883	Unclassified	GBIF: https://doi.org/10.15468/dl.zfaw79
Anopheles maculipennis	4.29872	51.268799	Unclassified	GBIF: https://doi.org/10.15468/dl.zfaw79
Anopheles maculipennis	4.36848	51.16473	Unclassified	GBIF: https://doi.org/10.15468/dl.zfaw79
Anopheles maculipennis	4.37289	50.87615	Unclassified	GBIF: https://doi.org/10.15468/dl.zfaw79

Anopheles maculipennis	4.383314	50.791038	Unclassified	GBIF: https://doi.org/10.15468/dl.zfaw79
Anopheles maculipennis	4.384642	50.835989	Unclassified	GBIF: https://doi.org/10.15468/dl.zfaw79
Anopheles maculipennis	4.405637	52.029152	Unclassified	GBIF: https://doi.org/10.15468/dl.zfaw79
Anopheles maculipennis	4.411739	51.977122	Unclassified	GBIF: https://doi.org/10.15468/dl.zfaw79
Anopheles maculipennis	4.418174	51.968304	Unclassified	GBIF: https://doi.org/10.15468/dl.zfaw79
Anopheles maculipennis	4.42	51.22	Unclassified	GBIF: https://doi.org/10.15468/dl.zfaw79
Anopheles maculipennis	4.42	51.220001	Unclassified	GBIF: https://doi.org/10.15468/dl.zfaw79
Anopheles maculipennis	4.42983	51.423611	Unclassified	GBIF: https://doi.org/10.15468/dl.zfaw79
Anopheles maculipennis	4.432774	52.178064	Unclassified	GBIF: https://doi.org/10.15468/dl.zfaw79
Anopheles maculipennis	4.455626	50.835124	Unclassified	GBIF: https://doi.org/10.15468/dl.zfaw79
Anopheles maculipennis	4.457026	50.880074	Unclassified	GBIF: https://doi.org/10.15468/dl.zfaw79
Anopheles maculipennis	4.47132	50.46976	Unclassified	GBIF: https://doi.org/10.15468/dl.zfaw79
Anopheles maculipennis	4.5	51.9	Unclassified	GBIF: https://doi.org/10.15468/dl.zfaw79
Anopheles maculipennis	4.51494	49.985889	Unclassified	GBIF: https://doi.org/10.15468/dl.zfaw79
Anopheles maculipennis	4.51725	51.000629	Unclassified	GBIF: https://doi.org/10.15468/dl.zfaw79
Anopheles maculipennis	4.542687	51.999897	Unclassified	GBIF: https://doi.org/10.15468/dl.zfaw79
Anopheles maculipennis	4.56994	50.947449	Unclassified	GBIF: https://doi.org/10.15468/dl.zfaw79
Anopheles maculipennis	4.59191	50.76918	Unclassified	GBIF: https://doi.org/10.15468/dl.zfaw79
Anopheles maculipennis	4.600152	52.387589	Unclassified	GBIF: https://doi.org/10.15468/dl.zfaw79
Anopheles maculipennis	4.617952	51.998705	Unclassified	GBIF: https://doi.org/10.15468/dl.zfaw79
Anopheles maculipennis	4.643056	46.669167	Unclassified	GBIF: https://doi.org/10.15468/dl.zfaw79
Anopheles maculipennis	4.643676	52.621369	Unclassified	GBIF: https://doi.org/10.15468/dl.zfaw79
Anopheles maculipennis	4.665356	50.742383	Unclassified	GBIF: https://doi.org/10.15468/dl.zfaw79
Anopheles maculipennis	4.739245	51.532016	Unclassified	GBIF: https://doi.org/10.15468/dl.zfaw79
Anopheles maculipennis	4.79333	53.097878	Unclassified	GBIF: https://doi.org/10.15468/dl.zfaw79
Anopheles maculipennis	4.793851	51.544261	Unclassified	GBIF: https://doi.org/10.15468/dl.zfaw79
Anopheles maculipennis	4.79908	51.071232	Unclassified	GBIF: https://doi.org/10.15468/dl.zfaw79
Anopheles maculipennis	4.8	51.07	Unclassified	GBIF: https://doi.org/10.15468/dl.zfaw79
Anopheles maculipennis	4.84673	50.541431	Unclassified	GBIF: https://doi.org/10.15468/dl.zfaw79
Anopheles maculipennis	4.84879	51.628213	Unclassified	GBIF: https://doi.org/10.15468/dl.zfaw79
Anopheles maculipennis	4.93018	51.196499	Unclassified	GBIF: https://doi.org/10.15468/dl.zfaw79
Anopheles maculipennis	4.937851	51.618611	Unclassified	GBIF: https://doi.org/10.15468/dl.zfaw79
Anopheles maculipennis	4.95421	51.245739	Unclassified	GBIF: https://doi.org/10.15468/dl.zfaw79
Anopheles maculipennis	4.955578	51.749534	Unclassified	GBIF: https://doi.org/10.15468/dl.zfaw79
Anopheles maculipennis	4.978475	50.311804	Unclassified	GBIF: https://doi.org/10.15468/dl.zfaw79
Anopheles maculipennis	4.99479	51.42572	Unclassified	GBIF: https://doi.org/10.15468/dl.zfaw79
Anopheles maculipennis	5.00615	50.315843	Unclassified	GBIF: https://doi.org/10.15468/dl.zfaw79
Anopheles maculipennis	5.035719	50.327532	Unclassified	GBIF: https://doi.org/10.15468/dl.zfaw79
Anopheles maculipennis	5.04574	50.3392	Unclassified	GBIF: https://doi.org/10.15468/dl.zfaw79
Anopheles maculipennis	5.04774	51.28685	Unclassified	GBIF: https://doi.org/10.15468/dl.zfaw79
Anopheles maculipennis	5.07164	50.33586	Unclassified	GBIF: https://doi.org/10.15468/dl.zfaw79
Anopheles maculipennis	5.08706	51.031181	Unclassified	GBIF: https://doi.org/10.15468/dl.zfaw79

Anopheles maculipennis	5.09425	50.483158	Unclassified	GBIF: https://doi.org/10.15468/dl.zfaw79
Anopheles maculipennis	5.11055	51.184864	Unclassified	GBIF: https://doi.org/10.15468/dl.zfaw79
Anopheles maculipennis	5.11516	50.998459	Unclassified	GBIF: https://doi.org/10.15468/dl.zfaw79
Anopheles maculipennis	5.14074	51.032089	Unclassified	GBIF: https://doi.org/10.15468/dl.zfaw79
Anopheles maculipennis	5.15545	50.193069	Unclassified	GBIF: https://doi.org/10.15468/dl.zfaw79
Anopheles maculipennis	5.166295	52.352475	Unclassified	GBIF: https://doi.org/10.15468/dl.zfaw79
Anopheles maculipennis	5.17407	51.15456	Unclassified	GBIF: https://doi.org/10.15468/dl.zfaw79
Anopheles maculipennis	5.186295	51.273408	Unclassified	GBIF: https://doi.org/10.15468/dl.zfaw79
Anopheles maculipennis	5.21916	50.493839	Unclassified	GBIF: https://doi.org/10.15468/dl.zfaw79
Anopheles maculipennis	5.22	50.49	Unclassified	GBIF: https://doi.org/10.15468/dl.zfaw79
Anopheles maculipennis	5.237478	51.725173	Unclassified	GBIF: https://doi.org/10.15468/dl.zfaw79
Anopheles maculipennis	5.25564	51.924781	Unclassified	GBIF: https://doi.org/10.15468/dl.zfaw79
Anopheles maculipennis	5.25975	50.58533	Unclassified	GBIF: https://doi.org/10.15468/dl.zfaw79
Anopheles maculipennis	5.30223	50.975208	Unclassified	GBIF: https://doi.org/10.15468/dl.zfaw79
Anopheles maculipennis	5.303462	51.568631	Unclassified	GBIF: https://doi.org/10.15468/dl.zfaw79
Anopheles maculipennis	5.324865	49.741642	Unclassified	GBIF: https://doi.org/10.15468/dl.zfaw79
Anopheles maculipennis	5.33971	51.154549	Unclassified	GBIF: https://doi.org/10.15468/dl.zfaw79
Anopheles maculipennis	5.380351	52.365667	Unclassified	GBIF: https://doi.org/10.15468/dl.zfaw79
Anopheles maculipennis	5.41562	50.9347	Unclassified	GBIF: https://doi.org/10.15468/dl.zfaw79
Anopheles maculipennis	5.43139	50.63778	Unclassified	GBIF: https://doi.org/10.15468/dl.zfaw79
Anopheles maculipennis	5.465106	52.45584	Unclassified	GBIF: https://doi.org/10.15468/dl.zfaw79
Anopheles maculipennis	5.514375	52.368547	Unclassified	GBIF: https://doi.org/10.15468/dl.zfaw79
Anopheles maculipennis	5.52234	50.876019	Unclassified	GBIF: https://doi.org/10.15468/dl.zfaw79
Anopheles maculipennis	5.528944	52.388034	Unclassified	GBIF: https://doi.org/10.15468/dl.zfaw79
Anopheles maculipennis	5.60128	50.636768	Unclassified	GBIF: https://doi.org/10.15468/dl.zfaw79
Anopheles maculipennis	5.60537	51.346014	Unclassified	GBIF: https://doi.org/10.15468/dl.zfaw79
Anopheles maculipennis	5.618601	50.992042	Unclassified	GBIF: https://doi.org/10.15468/dl.zfaw79
Anopheles maculipennis	5.618889	50.995	Unclassified	GBIF: https://doi.org/10.15468/dl.zfaw79
Anopheles maculipennis	5.619013	50.996013	Unclassified	GBIF: https://doi.org/10.15468/dl.zfaw79
Anopheles maculipennis	5.619639	52.394163	Unclassified	GBIF: https://doi.org/10.15468/dl.zfaw79
Anopheles maculipennis	5.61986	50.996262	Unclassified	GBIF: https://doi.org/10.15468/dl.zfaw79
Anopheles maculipennis	5.62125	50.99526	Unclassified	GBIF: https://doi.org/10.15468/dl.zfaw79
Anopheles maculipennis	5.629263	51.431629	Unclassified	GBIF: https://doi.org/10.15468/dl.zfaw79
Anopheles maculipennis	5.651487	50.634172	Unclassified	GBIF: https://doi.org/10.15468/dl.zfaw79
Anopheles maculipennis	5.6541	50.423229	Unclassified	GBIF: https://doi.org/10.15468/dl.zfaw79
Anopheles maculipennis	5.661653	50.813837	Unclassified	GBIF: https://doi.org/10.15468/dl.zfaw79
Anopheles maculipennis	5.66941	51.038521	Unclassified	GBIF: https://doi.org/10.15468/dl.zfaw79
Anopheles maculipennis	5.67	51.04	Unclassified	GBIF: https://doi.org/10.15468/dl.zfaw79
Anopheles maculipennis	5.67007	49.63533	Unclassified	GBIF: https://doi.org/10.15468/dl.zfaw79
Anopheles maculipennis	5.67582	50.929001	Unclassified	GBIF: https://doi.org/10.15468/dl.zfaw79
Anopheles maculipennis	5.681096	51.717753	Unclassified	GBIF: https://doi.org/10.15468/dl.zfaw79
Anopheles maculipennis	5.685177	51.70462	Unclassified	GBIF: https://doi.org/10.15468/dl.zfaw79

Anopheles maculipennis	5.68738	50.53413	Unclassified	GBIF: https://doi.org/10.15468/dl.zfaw79
Anopheles maculipennis	5.6891	51.01456	Unclassified	GBIF: https://doi.org/10.15468/dl.zfaw79
Anopheles maculipennis	5.69522	51.01628	Unclassified	GBIF: https://doi.org/10.15468/dl.zfaw79
Anopheles maculipennis	5.697141	51.014011	Unclassified	GBIF: https://doi.org/10.15468/dl.zfaw79
Anopheles maculipennis	5.726969	52.527503	Unclassified	GBIF: https://doi.org/10.15468/dl.zfaw79
Anopheles maculipennis	5.742086	51.741008	Unclassified	GBIF: https://doi.org/10.15468/dl.zfaw79
Anopheles maculipennis	5.75789	51.015499	Unclassified	GBIF: https://doi.org/10.15468/dl.zfaw79
Anopheles maculipennis	5.778409	51.722179	Unclassified	GBIF: https://doi.org/10.15468/dl.zfaw79
Anopheles maculipennis	5.82689	45.82792	Unclassified	GBIF: https://doi.org/10.15468/dl.zfaw79
Anopheles maculipennis	5.873522	51.652178	Unclassified	GBIF: https://doi.org/10.15468/dl.zfaw79
Anopheles maculipennis	5.889546	53.176778	Unclassified	GBIF: https://doi.org/10.15468/dl.zfaw79
Anopheles maculipennis	5.946278	52.340888	Unclassified	GBIF: https://doi.org/10.15468/dl.zfaw79
Anopheles maculipennis	5.98312	51.608849	Unclassified	GBIF: https://doi.org/10.15468/dl.zfaw79
Anopheles maculipennis	6.021725	50.446361	Unclassified	GBIF: https://doi.org/10.15468/dl.zfaw79
Anopheles maculipennis	6.024182	51.3768	Unclassified	GBIF: https://doi.org/10.15468/dl.zfaw79
Anopheles maculipennis	6.030856	52.986611	Unclassified	GBIF: https://doi.org/10.15468/dl.zfaw79
Anopheles maculipennis	6.048707	53.163614	Unclassified	GBIF: https://doi.org/10.15468/dl.zfaw79
Anopheles maculipennis	6.050142	53.164603	Unclassified	GBIF: https://doi.org/10.15468/dl.zfaw79
Anopheles maculipennis	6.07321	50.63235	Unclassified	GBIF: https://doi.org/10.15468/dl.zfaw79
Anopheles maculipennis	6.09002	50.719719	Unclassified	GBIF: https://doi.org/10.15468/dl.zfaw79
Anopheles maculipennis	6.098344	52.013531	Unclassified	GBIF: https://doi.org/10.15468/dl.zfaw79
Anopheles maculipennis	6.104256	52.037045	Unclassified	GBIF: https://doi.org/10.15468/dl.zfaw79
Anopheles maculipennis	6.110003	51.271613	Unclassified	GBIF: https://doi.org/10.15468/dl.zfaw79
Anopheles maculipennis	6.11033	50.67555	Unclassified	GBIF: https://doi.org/10.15468/dl.zfaw79
Anopheles maculipennis	6.11618	50.682968	Unclassified	GBIF: https://doi.org/10.15468/dl.zfaw79
Anopheles maculipennis	6.123644	52.06389	Unclassified	GBIF: https://doi.org/10.15468/dl.zfaw79
Anopheles maculipennis	6.143794	53.053555	Unclassified	GBIF: https://doi.org/10.15468/dl.zfaw79
Anopheles maculipennis	6.155528	52.306818	Unclassified	GBIF: https://doi.org/10.15468/dl.zfaw79
Anopheles maculipennis	6.16321	51.990829	Unclassified	GBIF: https://doi.org/10.15468/dl.zfaw79
Anopheles maculipennis	6.169819	53.185952	Unclassified	GBIF: https://doi.org/10.15468/dl.zfaw79
Anopheles maculipennis	6.176233	52.068943	Unclassified	GBIF: https://doi.org/10.15468/dl.zfaw79
Anopheles maculipennis	6.183384	51.979009	Unclassified	GBIF: https://doi.org/10.15468/dl.zfaw79
Anopheles maculipennis	6.183691	52.299171	Unclassified	GBIF: https://doi.org/10.15468/dl.zfaw79
Anopheles maculipennis	6.189497	52.377148	Unclassified	GBIF: https://doi.org/10.15468/dl.zfaw79
Anopheles maculipennis	6.195231	52.53853	Unclassified	GBIF: https://doi.org/10.15468/dl.zfaw79
Anopheles maculipennis	6.206617	52.31016	Unclassified	GBIF: https://doi.org/10.15468/dl.zfaw79
Anopheles maculipennis	6.210903	52.011179	Unclassified	GBIF: https://doi.org/10.15468/dl.zfaw79
Anopheles maculipennis	6.219647	52.520116	Unclassified	GBIF: https://doi.org/10.15468/dl.zfaw79
Anopheles maculipennis	6.22	43.37	Unclassified	GBIF: https://doi.org/10.15468/dl.zfaw79
Anopheles maculipennis	6.225399	52.030761	Unclassified	GBIF: https://doi.org/10.15468/dl.zfaw79
Anopheles maculipennis	6.22645	51.869048	Unclassified	GBIF: https://doi.org/10.15468/dl.zfaw79
Anopheles maculipennis	6.227922	52.02544	Unclassified	GBIF: https://doi.org/10.15468/dl.zfaw79

Anopheles maculipennis	6.241997	52.152076	Unclassified	GBIF: https://doi.org/10.15468/dl.zfaw79
Anopheles maculipennis	6.269138	53.211565	Unclassified	GBIF: https://doi.org/10.15468/dl.zfaw79
Anopheles maculipennis	6.29456	50.43258	Unclassified	GBIF: https://doi.org/10.15468/dl.zfaw79
Anopheles maculipennis	6.304335	52.362472	Unclassified	GBIF: https://doi.org/10.15468/dl.zfaw79
Anopheles maculipennis	6.311906	52.218512	Unclassified	GBIF: https://doi.org/10.15468/dl.zfaw79
Anopheles maculipennis	6.313246	52.307575	Unclassified	GBIF: https://doi.org/10.15468/dl.zfaw79
Anopheles maculipennis	6.334845	52.395488	Unclassified	GBIF: https://doi.org/10.15468/dl.zfaw79
Anopheles maculipennis	6.354943	52.23929	Unclassified	GBIF: https://doi.org/10.15468/dl.zfaw79
Anopheles maculipennis	6.356537	52.500472	Unclassified	GBIF: https://doi.org/10.15468/dl.zfaw79
Anopheles maculipennis	6.375015	52.483781	Unclassified	GBIF: https://doi.org/10.15468/dl.zfaw79
Anopheles maculipennis	6.378925	52.244574	Unclassified	GBIF: https://doi.org/10.15468/dl.zfaw79
Anopheles maculipennis	6.380319	52.287346	Unclassified	GBIF: https://doi.org/10.15468/dl.zfaw79
Anopheles maculipennis	6.386215	52.243164	Unclassified	GBIF: https://doi.org/10.15468/dl.zfaw79
Anopheles maculipennis	6.390442	51.865065	Unclassified	GBIF: https://doi.org/10.15468/dl.zfaw79
Anopheles maculipennis	6.394381	52.293429	Unclassified	GBIF: https://doi.org/10.15468/dl.zfaw79
Anopheles maculipennis	6.395363	52.394808	Unclassified	GBIF: https://doi.org/10.15468/dl.zfaw79
Anopheles maculipennis	6.421779	51.965019	Unclassified	GBIF: https://doi.org/10.15468/dl.zfaw79
Anopheles maculipennis	6.442276	52.456453	Unclassified	GBIF: https://doi.org/10.15468/dl.zfaw79
Anopheles maculipennis	6.473698	52.125899	Unclassified	GBIF: https://doi.org/10.15468/dl.zfaw79
Anopheles maculipennis	6.478862	52.217625	Unclassified	GBIF: https://doi.org/10.15468/dl.zfaw79
Anopheles maculipennis	6.505725	52.238407	Unclassified	GBIF: https://doi.org/10.15468/dl.zfaw79
Anopheles maculipennis	6.53784	52.490474	Unclassified	GBIF: https://doi.org/10.15468/dl.zfaw79
Anopheles maculipennis	6.549711	52.016409	Unclassified	GBIF: https://doi.org/10.15468/dl.zfaw79
Anopheles maculipennis	6.616153	51.940226	Unclassified	GBIF: https://doi.org/10.15468/dl.zfaw79
Anopheles maculipennis	6.662472	52.036815	Unclassified	GBIF: https://doi.org/10.15468/dl.zfaw79
Anopheles maculipennis	6.697309	52.206145	Unclassified	GBIF: https://doi.org/10.15468/dl.zfaw79
Anopheles maculipennis	6.757622	50.949635	Unclassified	GBIF: https://doi.org/10.15468/dl.zfaw79
Anopheles maculipennis	6.758823	50.949728	Unclassified	GBIF: https://doi.org/10.15468/dl.zfaw79
Anopheles maculipennis	6.815785	53.039824	Unclassified	GBIF: https://doi.org/10.15468/dl.zfaw79
Anopheles maculipennis	6.857187	52.333434	Unclassified	GBIF: https://doi.org/10.15468/dl.zfaw79
Anopheles maculipennis	6.922927	52.373382	Unclassified	GBIF: https://doi.org/10.15468/dl.zfaw79
Anopheles maculipennis	6.925196	52.406189	Unclassified	GBIF: https://doi.org/10.15468/dl.zfaw79
Anopheles maculipennis	6.941757	52.294257	Unclassified	GBIF: https://doi.org/10.15468/dl.zfaw79
Anopheles maculipennis	6.943072	52.187322	Unclassified	GBIF: https://doi.org/10.15468/dl.zfaw79
Anopheles maculipennis	6.95501	52.375649	Unclassified	GBIF: https://doi.org/10.15468/dl.zfaw79
Anopheles maculipennis	6.959854	52.410768	Unclassified	GBIF: https://doi.org/10.15468/dl.zfaw79
Anopheles maculipennis	6.962759	52.324227	Unclassified	GBIF: https://doi.org/10.15468/dl.zfaw79
Anopheles maculipennis	7.13604	52.946005	Unclassified	GBIF: https://doi.org/10.15468/dl.zfaw79
Anopheles maculipennis	7.528333	48.017502	Unclassified	GBIF: https://doi.org/10.15468/dl.zfaw79
Anopheles maculipennis	8.370466	49.218774	Unclassified	GBIF: https://doi.org/10.15468/dl.zfaw79
Anopheles maculipennis	8.698014	49.228861	Unclassified	GBIF: https://doi.org/10.15468/dl.zfaw79
Anopheles maculipennis	9.252924	48.911404	Unclassified	GBIF: https://doi.org/10.15468/dl.zfaw79

Anopheles maculipennis	9.52909	37.045968	Unclassified	GBIF: https://doi.org/10.15468/dl.zfaw79
Anopheles maculipennis	9.533082	37.079317	Unclassified	GBIF: https://doi.org/10.15468/dl.zfaw79
Anopheles maculipennis	10.022549	59.976086	Unclassified	GBIF: https://doi.org/10.15468/dl.zfaw79
Anopheles maculipennis	10.79408	59.29218	Unclassified	GBIF: https://doi.org/10.15468/dl.zfaw79
Anopheles maculipennis	11.00222	59.4296	Unclassified	GBIF: https://doi.org/10.15468/dl.zfaw79
Anopheles maculipennis	11.01237	59.04038	Unclassified	GBIF: https://doi.org/10.15468/dl.zfaw79
Anopheles maculipennis	11.932753	52.524195	Unclassified	GBIF: https://doi.org/10.15468/dl.zfaw79
Anopheles maculipennis	12.090905	55.675858	Unclassified	GBIF: https://doi.org/10.15468/dl.zfaw79
Anopheles maculipennis	12.091233	55.678136	Unclassified	GBIF: https://doi.org/10.15468/dl.zfaw79
Anopheles maculipennis	13.54375	57.84921	Unclassified	GBIF: https://doi.org/10.15468/dl.zfaw79
Anopheles maculipennis	13.576441	52.519199	Unclassified	GBIF: https://doi.org/10.15468/dl.zfaw79
Anopheles maculipennis	13.789022	51.128989	Unclassified	GBIF: https://doi.org/10.15468/dl.zfaw79
Anopheles maculipennis	14.067977	40.837928	Unclassified	GBIF: https://doi.org/10.15468/dl.zfaw79
Anopheles maculipennis	14.069192	40.839032	Unclassified	GBIF: https://doi.org/10.15468/dl.zfaw79
Anopheles maculipennis	14.26812	40.851798	Unclassified	GBIF: https://doi.org/10.15468/dl.zfaw79
Anopheles maculipennis	14.98526	59.52024	Unclassified	GBIF: https://doi.org/10.15468/dl.zfaw79
Anopheles maculipennis	15.00095	56.1726	Unclassified	GBIF: https://doi.org/10.15468/dl.zfaw79
Anopheles maculipennis	16.04	49.223333	Unclassified	GBIF: https://doi.org/10.15468/dl.zfaw79
Anopheles maculipennis	16.40648	56.42313	Unclassified	GBIF: https://doi.org/10.15468/dl.zfaw79
Anopheles maculipennis	17.02679	61.99008	Unclassified	GBIF: https://doi.org/10.15468/dl.zfaw79
Anopheles maculipennis	17.564167	43.017222	Unclassified	GBIF: https://doi.org/10.15468/dl.zfaw79
Anopheles maculipennis	17.56449	59.84283	Unclassified	GBIF: https://doi.org/10.15468/dl.zfaw79
Anopheles maculipennis	17.79651	62.92545	Unclassified	GBIF: https://doi.org/10.15468/dl.zfaw79
Anopheles maculipennis	19.18472	47.5186	Unclassified	GBIF: https://doi.org/10.15468/dl.zfaw79
Anopheles maculipennis	19.49	60.14	Unclassified	GBIF: https://doi.org/10.15468/dl.zfaw79
Anopheles maculipennis	19.51	60.13	Unclassified	GBIF: https://doi.org/10.15468/dl.zfaw79
Anopheles maculipennis	19.529544	41.000099	Unclassified	GBIF: https://doi.org/10.15468/dl.zfaw79
Anopheles maculipennis	20.363056	66.678889	Unclassified	GBIF: https://doi.org/10.15468/dl.zfaw79
Anopheles maculipennis	20.548666	66.676244	Unclassified	GBIF: https://doi.org/10.15468/dl.zfaw79
Anopheles maculipennis	20.8052	37.774	Unclassified	GBIF: https://doi.org/10.15468/dl.zfaw79
Anopheles maculipennis	20.974643	46.333069	Unclassified	GBIF: https://doi.org/10.15468/dl.zfaw79
Anopheles maculipennis	20.983614	46.321649	Unclassified	GBIF: https://doi.org/10.15468/dl.zfaw79
Anopheles maculipennis	21.041126	46.280333	Unclassified	GBIF: https://doi.org/10.15468/dl.zfaw79
Anopheles maculipennis	21.054094	46.262186	Unclassified	GBIF: https://doi.org/10.15468/dl.zfaw79
Anopheles maculipennis	21.679133	60.169563	Unclassified	GBIF: https://doi.org/10.15468/dl.zfaw79
Anopheles maculipennis	21.73	61.19	Unclassified	GBIF: https://doi.org/10.15468/dl.zfaw79
Anopheles maculipennis	21.79	60.65	Unclassified	GBIF: https://doi.org/10.15468/dl.zfaw79
Anopheles maculipennis	21.82	60.5	Unclassified	GBIF: https://doi.org/10.15468/dl.zfaw79
Anopheles maculipennis	21.923415	60.613993	Unclassified	GBIF: https://doi.org/10.15468/dl.zfaw79
Anopheles maculipennis	22.200524	60.427962	Unclassified	GBIF: https://doi.org/10.15468/dl.zfaw79
Anopheles maculipennis	22.405267	60.285624	Unclassified	GBIF: https://doi.org/10.15468/dl.zfaw79
Anopheles maculipennis	22.492	58.254	Unclassified	GBIF: https://doi.org/10.15468/dl.zfaw79

Anopheles maculipennis	23.36	60.94	Unclassified	GBIF: https://doi.org/10.15468/dl.zfaw79
Anopheles maculipennis	23.8844	58.9678	Unclassified	GBIF: https://doi.org/10.15468/dl.zfaw79
Anopheles maculipennis	24.39	61.07	Unclassified	GBIF: https://doi.org/10.15468/dl.zfaw79
Anopheles maculipennis	24.610475	60.160616	Unclassified	GBIF: https://doi.org/10.15468/dl.zfaw79
Anopheles maculipennis	24.84416	59.47013	Unclassified	GBIF: https://doi.org/10.15468/dl.zfaw79
Anopheles maculipennis	25.09998	60.262809	Unclassified	GBIF: https://doi.org/10.15468/dl.zfaw79
Anopheles maculipennis	25.105165	60.173105	Unclassified	GBIF: https://doi.org/10.15468/dl.zfaw79
Anopheles maculipennis	25.45	61.99	Unclassified	GBIF: https://doi.org/10.15468/dl.zfaw79
Anopheles maculipennis	26.17	60.91	Unclassified	GBIF: https://doi.org/10.15468/dl.zfaw79
Anopheles maculipennis	26.351967	60.903084	Unclassified	GBIF: https://doi.org/10.15468/dl.zfaw79
Anopheles maculipennis	26.683556	58.352861	Unclassified	GBIF: https://doi.org/10.15468/dl.zfaw79
Anopheles maculipennis	26.6944	58.396	Unclassified	GBIF: https://doi.org/10.15468/dl.zfaw79
Anopheles maculipennis	26.69806	58.39111	Unclassified	GBIF: https://doi.org/10.15468/dl.zfaw79
Anopheles maculipennis	26.7	58.35	Unclassified	GBIF: https://doi.org/10.15468/dl.zfaw79
Anopheles maculipennis	26.70556	58.385	Unclassified	GBIF: https://doi.org/10.15468/dl.zfaw79
Anopheles maculipennis	26.72389	58.34889	Unclassified	GBIF: https://doi.org/10.15468/dl.zfaw79
Anopheles maculipennis	26.72472	58.34944	Unclassified	GBIF: https://doi.org/10.15468/dl.zfaw79
Anopheles maculipennis	26.72889	58.39583	Unclassified	GBIF: https://doi.org/10.15468/dl.zfaw79
Anopheles maculipennis	26.73	58.39	Unclassified	GBIF: https://doi.org/10.15468/dl.zfaw79
Anopheles maculipennis	26.734722	58.394444	Unclassified	GBIF: https://doi.org/10.15468/dl.zfaw79
Anopheles maculipennis	26.734722	58.394447	Unclassified	GBIF: https://doi.org/10.15468/dl.zfaw79
Anopheles maculipennis	26.783333	58.35	Unclassified	GBIF: https://doi.org/10.15468/dl.zfaw79
Anopheles maculipennis	26.816667	58.325	Unclassified	GBIF: https://doi.org/10.15468/dl.zfaw79
Anopheles maculipennis	27.181835	69.084568	Unclassified	GBIF: https://doi.org/10.15468/dl.zfaw79
Anopheles maculipennis	27.184538	69.085053	Unclassified	GBIF: https://doi.org/10.15468/dl.zfaw79
Anopheles maculipennis	27.185291	69.078856	Unclassified	GBIF: https://doi.org/10.15468/dl.zfaw79
Anopheles maculipennis	27.187567	69.085009	Unclassified	GBIF: https://doi.org/10.15468/dl.zfaw79
Anopheles maculipennis	27.678115	63.071718	Unclassified	GBIF: https://doi.org/10.15468/dl.zfaw79
Anopheles maculipennis	28.07511	68.822115	Unclassified	GBIF: https://doi.org/10.15468/dl.zfaw79
Anopheles maculipennis	28.122024	68.795732	Unclassified	GBIF: https://doi.org/10.15468/dl.zfaw79
Anopheles maculipennis	30.253047	46.431781	Unclassified	GBIF: https://doi.org/10.15468/dl.zfaw79
Anopheles maculipennis	30.257596	47.425057	Unclassified	GBIF: https://doi.org/10.15468/dl.zfaw79
Anopheles maculipennis	30.260319	46.411309	Unclassified	GBIF: https://doi.org/10.15468/dl.zfaw79
Anopheles maculipennis	30.582815	46.411009	Unclassified	GBIF: https://doi.org/10.15468/dl.zfaw79
Anopheles maculipennis	32.26944	46.530043	Unclassified	GBIF: https://doi.org/10.15468/dl.zfaw79
Anopheles maculipennis	34.59837	64.95553	Unclassified	GBIF: https://doi.org/10.15468/dl.zfaw79
Anopheles maculipennis	36.305439	49.262712	Unclassified	GBIF: https://doi.org/10.15468/dl.zfaw79
Anopheles maculipennis	53.684025	63.562626	Unclassified	GBIF: https://doi.org/10.15468/dl.zfaw79
Anopheles maculipes	-54.000025	4.766684	Unclassified	GBIF: https://doi.org/10.15468/dl.zfaw79
Anopheles maculipes	-53.24036	3.91451	Unclassified	GBIF: https://doi.org/10.15468/dl.zfaw79
Anopheles maculipes	-53.23477	3.92404	Unclassified	GBIF: https://doi.org/10.15468/dl.zfaw79
Anopheles maculipes	-53.02915	5.01314	Unclassified	GBIF: https://doi.org/10.15468/dl.zfaw79

Anopheles maculipes	-52.486147	4.558239	Unclassified	GBIF: https://doi.org/10.15468/dl.zfaw79
Anopheles maculipes	-52.48462	4.560602	Unclassified	GBIF: https://doi.org/10.15468/dl.zfaw79
Anopheles maculipes	-52.481135	4.561623	Unclassified	GBIF: https://doi.org/10.15468/dl.zfaw79
Anopheles maculipes	-52.476399	4.568397	Unclassified	GBIF: https://doi.org/10.15468/dl.zfaw79
Anopheles maculipes	-52.476399	4.568433	Unclassified	GBIF: https://doi.org/10.15468/dl.zfaw79
Anopheles maculipes	-52.473064	4.550579	Unclassified	GBIF: https://doi.org/10.15468/dl.zfaw79
Anopheles maculipes	-52.333605	3.167747	Unclassified	GBIF: https://doi.org/10.15468/dl.zfaw79
Anopheles maculipes	-52.323296	4.936976	Unclassified	GBIF: https://doi.org/10.15468/dl.zfaw79
Anopheles majidi	85.016353	27.439336	Unclassified	GBIF: https://doi.org/10.15468/dl.zfaw79
Anopheles majidi	85.570255	27.704429	Unclassified	GBIF: https://doi.org/10.15468/dl.zfaw79
Anopheles malefactor	-89.114361	18.599444	Unclassified	GBIF: https://doi.org/10.15468/dl.zfaw79
Anopheles malefactor	-84.33	9.51	Unclassified	GBIF: https://doi.org/10.15468/dl.zfaw79
Anopheles malefactor	-79.916667	9.266667	Unclassified	GBIF: https://doi.org/10.15468/dl.zfaw79
Anopheles malefactor	-77.39	8.07	Unclassified	GBIF: https://doi.org/10.15468/dl.zfaw79
Anopheles malefactor	-76.87	7.8	Unclassified	GBIF: https://doi.org/10.15468/dl.zfaw79
Anopheles malefactor	-76.75	6.2833	Unclassified	GBIF: https://doi.org/10.15468/dl.zfaw79
Anopheles malefactor	-76.75	6.28	Unclassified	GBIF: https://doi.org/10.15468/dl.zfaw79
Anopheles malefactor	-76.69	5.13	Unclassified	GBIF: https://doi.org/10.15468/dl.zfaw79
Anopheles malefactor	-75.85	7.7167	Unclassified	GBIF: https://doi.org/10.15468/dl.zfaw79
Anopheles malefactor	-75.85	7.72	Unclassified	GBIF: https://doi.org/10.15468/dl.zfaw79
Anopheles malefactor	-72.72	8.63	Unclassified	GBIF: https://doi.org/10.15468/dl.zfaw79
Anopheles malefactor	-72.7	-7.79	Unclassified	GBIF: https://doi.org/10.15468/dl.zfaw79
Anopheles malefactor	-72.7	-7.47	Unclassified	GBIF: https://doi.org/10.15468/dl.zfaw79
Anopheles malefactor	-72.67	8.57	Unclassified	GBIF: https://doi.org/10.15468/dl.zfaw79
Anopheles malefactor	-72.667	8.5692	Unclassified	GBIF: https://doi.org/10.15468/dl.zfaw79
Anopheles malefactor	-72.64451	4.187754	Unclassified	GBIF: https://doi.org/10.15468/dl.zfaw79
Anopheles malefactor	-66.82	-10.01	Unclassified	GBIF: https://doi.org/10.15468/dl.zfaw79
Anopheles mangyanus	121.065918	12.347433	Unclassified	GBIF: https://doi.org/10.15468/dl.zfaw79
Anopheles mangyanus	124.757122	11.243803	Unclassified	GBIF: https://doi.org/10.15468/dl.zfaw79
Anopheles marajoara	-64.267	-12.467	Unclassified	GBIF: https://doi.org/10.15468/dl.zfaw79
Anopheles marajoara	-60.24	-1.49	Unclassified	GBIF: https://doi.org/10.15468/dl.zfaw79
Anopheles marajoara	-55.83	-4.17	Unclassified	GBIF: https://doi.org/10.15468/dl.zfaw79
Anopheles marajoara	-55.02	-2.75	Unclassified	GBIF: https://doi.org/10.15468/dl.zfaw79
Anopheles marajoara	-54.9	-10.38	Unclassified	GBIF: https://doi.org/10.15468/dl.zfaw79
Anopheles marajoara	-54.66	-2.48	Unclassified	GBIF: https://doi.org/10.15468/dl.zfaw79
Anopheles marajoara	-54.59	-2.55	Unclassified	GBIF: https://doi.org/10.15468/dl.zfaw79
Anopheles marajoara	-54.42	-9.99	Unclassified	GBIF: https://doi.org/10.15468/dl.zfaw79
Anopheles marajoara	-54.031415	3.643348	Unclassified	GBIF: https://doi.org/10.15468/dl.zfaw79
Anopheles marajoara	-53.549917	3.749968	Unclassified	GBIF: https://doi.org/10.15468/dl.zfaw79
Anopheles marajoara	-53.24036	3.91451	Unclassified	GBIF: https://doi.org/10.15468/dl.zfaw79
Anopheles marajoara	-53.23477	3.92404	Unclassified	GBIF: https://doi.org/10.15468/dl.zfaw79
Anopheles marajoara	-52.268325	3.273818	Unclassified	GBIF: https://doi.org/10.15468/dl.zfaw79

Anopheles marajoara	-51.07	0.03	Unclassified	GBIF: https://doi.org/10.15468/dl.zfaw79
Anopheles marajoara	-51.05	-0.14	Unclassified	GBIF: https://doi.org/10.15468/dl.zfaw79
Anopheles marajoara	-51.05	0.14	Unclassified	GBIF: https://doi.org/10.15468/dl.zfaw79
Anopheles marajoara	-51.04	-0.15	Unclassified	GBIF: https://doi.org/10.15468/dl.zfaw79
Anopheles marajoara	-50.97	0.21	Unclassified	GBIF: https://doi.org/10.15468/dl.zfaw79
Anopheles marajoara	-50.97	-0.21	Unclassified	GBIF: https://doi.org/10.15468/dl.zfaw79
Anopheles marajoara	-50.35	-19.52	Unclassified	GBIF: https://doi.org/10.15468/dl.zfaw79
Anopheles marajoara	-49.5	-1	Unclassified	GBIF: https://doi.org/10.15468/dl.zfaw79
Anopheles marajoara	-49.22	-5.64	Unclassified	GBIF: https://doi.org/10.15468/dl.zfaw79
Anopheles marajoara	-49.22	-3.85	Unclassified	GBIF: https://doi.org/10.15468/dl.zfaw79
Anopheles marajoara	-49.219	-3.853	Unclassified	GBIF: https://doi.org/10.15468/dl.zfaw79
Anopheles marajoara	-49.202	-5.365	Unclassified	GBIF: https://doi.org/10.15468/dl.zfaw79
Anopheles marajoara	-49.113	-4.47	Unclassified	GBIF: https://doi.org/10.15468/dl.zfaw79
Anopheles marajoara	-48.92	-5.28	Unclassified	GBIF: https://doi.org/10.15468/dl.zfaw79
Anopheles marajoara	-47.18	-1.4	Unclassified	GBIF: https://doi.org/10.15468/dl.zfaw79
Anopheles marshallii	3.33254	7.18376	Unclassified	GBIF: https://doi.org/10.15468/dl.zfaw79
Anopheles marshallii	3.89922	7.43373	Unclassified	GBIF: https://doi.org/10.15468/dl.zfaw79
Anopheles marshallii	5.0659	9.30023	Unclassified	GBIF: https://doi.org/10.15468/dl.zfaw79
Anopheles marshallii	6.73259	7.80037	Unclassified	GBIF: https://doi.org/10.15468/dl.zfaw79
Anopheles marshallii	7.54928	6.4505	Unclassified	GBIF: https://doi.org/10.15468/dl.zfaw79
Anopheles marshallii	7.71594	11.08341	Unclassified	GBIF: https://doi.org/10.15468/dl.zfaw79
Anopheles marshallii	11.59	-0.19	Unclassified	GBIF: https://doi.org/10.15468/dl.zfaw79
Anopheles marshallii	12.96	-1.76	Unclassified	GBIF: https://doi.org/10.15468/dl.zfaw79
Anopheles marshallii	14.07	-2.11	Unclassified	GBIF: https://doi.org/10.15468/dl.zfaw79
Anopheles marshallii	28.1825	-25.671111	Unclassified	GBIF: https://doi.org/10.15468/dl.zfaw79
Anopheles marshallii	28.2	-25.75	Unclassified	GBIF: https://doi.org/10.15468/dl.zfaw79
Anopheles marshallii	28.2667	-23.8917	Unclassified	GBIF: https://doi.org/10.15468/dl.zfaw79
Anopheles marshallii	30.85	-29.766667	Unclassified	GBIF: https://doi.org/10.15468/dl.zfaw79
Anopheles marshallii	31.05	-29.854167	Unclassified	GBIF: https://doi.org/10.15468/dl.zfaw79
Anopheles marshallii	32.168889	-27.321944	Unclassified	GBIF: https://doi.org/10.15468/dl.zfaw79
Anopheles marshallii	32.573292	-25.969141	Unclassified	GBIF: https://doi.org/10.15468/dl.zfaw79
Anopheles marteri	2.691979	42.244414	Unclassified	GBIF: https://doi.org/10.15468/dl.zfaw79
Anopheles marteri	24.958107	35.382106	Unclassified	GBIF: https://doi.org/10.15468/dl.zfaw79
Anopheles martinius	59.6	42.46	Unclassified	GBIF: https://doi.org/10.15468/dl.zfaw79
Anopheles mascarensis	45.14743	-12.8206	Unclassified	GBIF: https://doi.org/10.15468/dl.zfaw79
Anopheles mascarensis	47.96	-22.02	Unclassified	GBIF: https://doi.org/10.15468/dl.zfaw79
Anopheles mascarensis	48.1667	-19.3667	Unclassified	GBIF: https://doi.org/10.15468/dl.zfaw79
Anopheles mattogrossensis	-77.5832	-3.314419	Secondary	GBIF: https://doi.org/10.15468/dl.zfaw79
Anopheles mattogrossensis	-74.11416	-8.522718	Secondary	GBIF: https://doi.org/10.15468/dl.zfaw79
Anopheles mattogrossensis	-69.95035	4.14969	Secondary	GBIF: https://doi.org/10.15468/dl.zfaw79
Anopheles mattogrossensis	-69.76	-2.9	Secondary	GBIF: https://doi.org/10.15468/dl.zfaw79
Anopheles mattogrossensis	-69.7581	-2.8956	Secondary	GBIF: https://doi.org/10.15468/dl.zfaw79

Anopheles mattogrossensis	-63.040798	-9.91333	Secondary	GBIF: https://doi.org/10.15468/dl.zfaw79
Anopheles mattogrossensis	-51.066399	-0.366667	Secondary	GBIF: https://doi.org/10.15468/dl.zfaw79
Anopheles medialis	-63.894543	3.279717	Unclassified	GBIF: https://doi.org/10.15468/dl.zfaw79
Anopheles medialis	-63.89	3.28	Unclassified	GBIF: https://doi.org/10.15468/dl.zfaw79
Anopheles medialis	-63.79	3.3	Unclassified	GBIF: https://doi.org/10.15468/dl.zfaw79
Anopheles medialis	-63.787624	3.30083	Unclassified	GBIF: https://doi.org/10.15468/dl.zfaw79
Anopheles medialis	-63.64	1.84	Unclassified	GBIF: https://doi.org/10.15468/dl.zfaw79
Anopheles medialis	-63.63843	1.835648	Unclassified	GBIF: https://doi.org/10.15468/dl.zfaw79
Anopheles medialis	-63.62	1.77	Unclassified	GBIF: https://doi.org/10.15468/dl.zfaw79
Anopheles medialis	-63.618893	1.7653	Unclassified	GBIF: https://doi.org/10.15468/dl.zfaw79
Anopheles medialis	-63.61	1.82	Unclassified	GBIF: https://doi.org/10.15468/dl.zfaw79
Anopheles medialis	-63.608437	1.822008	Unclassified	GBIF: https://doi.org/10.15468/dl.zfaw79
Anopheles medialis	-63.6	1.72	Unclassified	GBIF: https://doi.org/10.15468/dl.zfaw79
Anopheles medialis	-63.595516	1.724896	Unclassified	GBIF: https://doi.org/10.15468/dl.zfaw79
Anopheles medialis	-63.534576	1.661081	Unclassified	GBIF: https://doi.org/10.15468/dl.zfaw79
Anopheles medialis	-63.53	1.66	Unclassified	GBIF: https://doi.org/10.15468/dl.zfaw79
Anopheles medialis	-48.8293	-25.4754	Unclassified	GBIF: https://doi.org/10.15468/dl.zfaw79
Anopheles medialis	-47.88333	-24.71667	Unclassified	GBIF: https://doi.org/10.15468/dl.zfaw79
Anopheles medialis	-47.88	-24.72	Unclassified	GBIF: https://doi.org/10.15468/dl.zfaw79
Anopheles mediopunctatus	-73.64164	4.14812	Unclassified	GBIF: https://doi.org/10.15468/dl.zfaw79
Anopheles mediopunctatus	-73.61867	4.14669	Unclassified	GBIF: https://doi.org/10.15468/dl.zfaw79
Anopheles mediopunctatus	-73.2505	-3.76707	Unclassified	GBIF: https://doi.org/10.15468/dl.zfaw79
Anopheles mediopunctatus	-61.275691	10.554713	Unclassified	GBIF: https://doi.org/10.15468/dl.zfaw79
Anopheles mediopunctatus	-61.271348	10.51485	Unclassified	GBIF: https://doi.org/10.15468/dl.zfaw79
Anopheles mediopunctatus	-61.2631	10.37305	Unclassified	GBIF: https://doi.org/10.15468/dl.zfaw79
Anopheles mediopunctatus	-61.2	10.4	Unclassified	GBIF: https://doi.org/10.15468/dl.zfaw79
Anopheles mediopunctatus	-61	11	Unclassified	GBIF: https://doi.org/10.15468/dl.zfaw79
Anopheles mediopunctatus	-54.415345	4.619739	Unclassified	GBIF: https://doi.org/10.15468/dl.zfaw79
Anopheles mediopunctatus	-54.41527	4.618084	Unclassified	GBIF: https://doi.org/10.15468/dl.zfaw79
Anopheles mediopunctatus	-54.398333	5.608056	Unclassified	GBIF: https://doi.org/10.15468/dl.zfaw79
Anopheles mediopunctatus	-54.383048	4.27418	Unclassified	GBIF: https://doi.org/10.15468/dl.zfaw79
Anopheles mediopunctatus	-54.151439	3.80651	Unclassified	GBIF: https://doi.org/10.15468/dl.zfaw79
Anopheles mediopunctatus	-54.148707	3.807339	Unclassified	GBIF: https://doi.org/10.15468/dl.zfaw79
Anopheles mediopunctatus	-54.141692	3.808351	Unclassified	GBIF: https://doi.org/10.15468/dl.zfaw79
Anopheles mediopunctatus	-54.07664	3.67331	Unclassified	GBIF: https://doi.org/10.15468/dl.zfaw79
Anopheles mediopunctatus	-54.04329	5.484058	Unclassified	GBIF: https://doi.org/10.15468/dl.zfaw79
Anopheles mediopunctatus	-54.031415	3.643348	Unclassified	GBIF: https://doi.org/10.15468/dl.zfaw79
Anopheles mediopunctatus	-54.02004	5.50717	Unclassified	GBIF: https://doi.org/10.15468/dl.zfaw79
Anopheles mediopunctatus	-54.000025	4.766684	Unclassified	GBIF: https://doi.org/10.15468/dl.zfaw79
Anopheles mediopunctatus	-53.833358	3.333351	Unclassified	GBIF: https://doi.org/10.15468/dl.zfaw79
Anopheles mediopunctatus	-53.674595	5.394268	Unclassified	GBIF: https://doi.org/10.15468/dl.zfaw79
Anopheles mediopunctatus	-53.582045	3.602948	Unclassified	GBIF: https://doi.org/10.15468/dl.zfaw79

Anopheles mediopunctatus	-53.210985	3.623926	Unclassified	GBIF: https://doi.org/10.15468/dl.zfaw79
Anopheles mediopunctatus	-53.08936	5.42827	Unclassified	GBIF: https://doi.org/10.15468/dl.zfaw79
Anopheles mediopunctatus	-53.083355	5.416688	Unclassified	GBIF: https://doi.org/10.15468/dl.zfaw79
Anopheles mediopunctatus	-53.050025	5.062018	Unclassified	GBIF: https://doi.org/10.15468/dl.zfaw79
Anopheles mediopunctatus	-53.02915	5.01314	Unclassified	GBIF: https://doi.org/10.15468/dl.zfaw79
Anopheles mediopunctatus	-52.900025	5.166684	Unclassified	GBIF: https://doi.org/10.15468/dl.zfaw79
Anopheles mediopunctatus	-52.87724	2.24504	Unclassified	GBIF: https://doi.org/10.15468/dl.zfaw79
Anopheles mediopunctatus	-52.873742	2.247535	Unclassified	GBIF: https://doi.org/10.15468/dl.zfaw79
Anopheles mediopunctatus	-52.738605	5.115338	Unclassified	GBIF: https://doi.org/10.15468/dl.zfaw79
Anopheles mediopunctatus	-52.700025	4.033351	Unclassified	GBIF: https://doi.org/10.15468/dl.zfaw79
Anopheles mediopunctatus	-52.700025	4.091684	Unclassified	GBIF: https://doi.org/10.15468/dl.zfaw79
Anopheles mediopunctatus	-52.66885	5.15907	Unclassified	GBIF: https://doi.org/10.15468/dl.zfaw79
Anopheles mediopunctatus	-52.643374	5.166931	Unclassified	GBIF: https://doi.org/10.15468/dl.zfaw79
Anopheles mediopunctatus	-52.57423	5.03313	Unclassified	GBIF: https://doi.org/10.15468/dl.zfaw79
Anopheles mediopunctatus	-52.499664	4.557415	Unclassified	GBIF: https://doi.org/10.15468/dl.zfaw79
Anopheles mediopunctatus	-52.497411	4.553404	Unclassified	GBIF: https://doi.org/10.15468/dl.zfaw79
Anopheles mediopunctatus	-52.492926	4.892476	Unclassified	GBIF: https://doi.org/10.15468/dl.zfaw79
Anopheles mediopunctatus	-52.483355	4.666688	Unclassified	GBIF: https://doi.org/10.15468/dl.zfaw79
Anopheles mediopunctatus	-52.47665	4.567727	Unclassified	GBIF: https://doi.org/10.15468/dl.zfaw79
Anopheles mediopunctatus	-52.476399	4.568397	Unclassified	GBIF: https://doi.org/10.15468/dl.zfaw79
Anopheles mediopunctatus	-52.473837	5.013996	Unclassified	GBIF: https://doi.org/10.15468/dl.zfaw79
Anopheles mediopunctatus	-52.473699	4.574534	Unclassified	GBIF: https://doi.org/10.15468/dl.zfaw79
Anopheles mediopunctatus	-52.473105	4.552939	Unclassified	GBIF: https://doi.org/10.15468/dl.zfaw79
Anopheles mediopunctatus	-52.47241	5.01171	Unclassified	GBIF: https://doi.org/10.15468/dl.zfaw79
Anopheles mediopunctatus	-52.471795	4.573254	Unclassified	GBIF: https://doi.org/10.15468/dl.zfaw79
Anopheles mediopunctatus	-52.47027	4.572008	Unclassified	GBIF: https://doi.org/10.15468/dl.zfaw79
Anopheles mediopunctatus	-52.464115	4.563611	Unclassified	GBIF: https://doi.org/10.15468/dl.zfaw79
Anopheles mediopunctatus	-52.462674	4.559987	Unclassified	GBIF: https://doi.org/10.15468/dl.zfaw79
Anopheles mediopunctatus	-52.46114	4.576432	Unclassified	GBIF: https://doi.org/10.15468/dl.zfaw79
Anopheles mediopunctatus	-52.434423	4.780484	Unclassified	GBIF: https://doi.org/10.15468/dl.zfaw79
Anopheles mediopunctatus	-52.360475	4.819828	Unclassified	GBIF: https://doi.org/10.15468/dl.zfaw79
Anopheles mediopunctatus	-52.35913	4.90255	Unclassified	GBIF: https://doi.org/10.15468/dl.zfaw79
Anopheles mediopunctatus	-52.357135	4.886868	Unclassified	GBIF: https://doi.org/10.15468/dl.zfaw79
Anopheles mediopunctatus	-52.35125	4.88011	Unclassified	GBIF: https://doi.org/10.15468/dl.zfaw79
Anopheles mediopunctatus	-52.34352	4.77908	Unclassified	GBIF: https://doi.org/10.15468/dl.zfaw79
Anopheles mediopunctatus	-52.33696	4.83273	Unclassified	GBIF: https://doi.org/10.15468/dl.zfaw79
Anopheles mediopunctatus	-52.334495	4.941608	Unclassified	GBIF: https://doi.org/10.15468/dl.zfaw79
Anopheles mediopunctatus	-52.334095	3.160908	Unclassified	GBIF: https://doi.org/10.15468/dl.zfaw79
Anopheles mediopunctatus	-52.333605	3.167747	Unclassified	GBIF: https://doi.org/10.15468/dl.zfaw79
Anopheles mediopunctatus	-52.330987	4.850608	Unclassified	GBIF: https://doi.org/10.15468/dl.zfaw79
Anopheles mediopunctatus	-52.32628	4.727944	Unclassified	GBIF: https://doi.org/10.15468/dl.zfaw79
Anopheles mediopunctatus	-52.323296	4.936976	Unclassified	GBIF: https://doi.org/10.15468/dl.zfaw79

Anopheles mediopunctatus	-52.31792	4.89677	Unclassified	GBIF: https://doi.org/10.15468/dl.zfaw79
Anopheles mediopunctatus	-52.316695	4.516688	Unclassified	GBIF: https://doi.org/10.15468/dl.zfaw79
Anopheles mediopunctatus	-52.312455	4.927798	Unclassified	GBIF: https://doi.org/10.15468/dl.zfaw79
Anopheles mediopunctatus	-52.30318	4.91254	Unclassified	GBIF: https://doi.org/10.15468/dl.zfaw79
Anopheles mediopunctatus	-52.30125	4.89677	Unclassified	GBIF: https://doi.org/10.15468/dl.zfaw79
Anopheles mediopunctatus	-52.291065	4.890268	Unclassified	GBIF: https://doi.org/10.15468/dl.zfaw79
Anopheles mediopunctatus	-52.28659	4.89308	Unclassified	GBIF: https://doi.org/10.15468/dl.zfaw79
Anopheles mediopunctatus	-52.276221	4.886853	Unclassified	GBIF: https://doi.org/10.15468/dl.zfaw79
Anopheles mediopunctatus	-52.251475	4.881148	Unclassified	GBIF: https://doi.org/10.15468/dl.zfaw79
Anopheles mediopunctatus	-52.13743	4.7904	Unclassified	GBIF: https://doi.org/10.15468/dl.zfaw79
Anopheles mediopunctatus	-52.129162	4.314002	Unclassified	GBIF: https://doi.org/10.15468/dl.zfaw79
Anopheles mediopunctatus	-52.037094	4.486403	Unclassified	GBIF: https://doi.org/10.15468/dl.zfaw79
Anopheles mediopunctatus	-51.88333	3.8	Unclassified	GBIF: https://doi.org/10.15468/dl.zfaw79
Anopheles mediopunctatus	-51.855225	3.870678	Unclassified	GBIF: https://doi.org/10.15468/dl.zfaw79
Anopheles mediopunctatus	-51.815192	3.875351	Unclassified	GBIF: https://doi.org/10.15468/dl.zfaw79
Anopheles mediopunctatus	-51.808359	3.891601	Unclassified	GBIF: https://doi.org/10.15468/dl.zfaw79
Anopheles mediopunctatus	-51.801158	3.888398	Unclassified	GBIF: https://doi.org/10.15468/dl.zfaw79
Anopheles mediopunctatus	-51.800609	3.896323	Unclassified	GBIF: https://doi.org/10.15468/dl.zfaw79
Anopheles mediopunctatus	-51.79697	3.913685	Unclassified	GBIF: https://doi.org/10.15468/dl.zfaw79
Anopheles mediopunctatus	-51.775025	4.166684	Unclassified	GBIF: https://doi.org/10.15468/dl.zfaw79
Anopheles mediopunctatus	-51.677335	4.039038	Unclassified	GBIF: https://doi.org/10.15468/dl.zfaw79
Anopheles mediopunctatus	-48.8293	-25.4754	Unclassified	GBIF: https://doi.org/10.15468/dl.zfaw79
Anopheles mediopunctatus	-48.5427	-25.5128	Unclassified	GBIF: https://doi.org/10.15468/dl.zfaw79
Anopheles mediopunctatus	-46.1833	-1.5	Unclassified	GBIF: https://doi.org/10.15468/dl.zfaw79
Anopheles melanoon	4.630278	43.678612	Unclassified	GBIF: https://doi.org/10.15468/dl.zfaw79
Anopheles melanoon	8.665	45.148	Unclassified	GBIF: https://doi.org/10.15468/dl.zfaw79
Anopheles melanoon	9.168	45.143	Unclassified	GBIF: https://doi.org/10.15468/dl.zfaw79
Anopheles melanoon	48.923	41.52	Unclassified	GBIF: https://doi.org/10.15468/dl.zfaw79
Anopheles melas	-17.11667	14.55	Unclassified	GBIF: https://doi.org/10.15468/dl.zfaw79
Anopheles melas	-16.75	13.33333	Unclassified	GBIF: https://doi.org/10.15468/dl.zfaw79
Anopheles melas	-16.65	13.46667	Unclassified	GBIF: https://doi.org/10.15468/dl.zfaw79
Anopheles melas	-16.6	13.36667	Unclassified	GBIF: https://doi.org/10.15468/dl.zfaw79
Anopheles melas	-16.5928	13.2806	Unclassified	GBIF: https://doi.org/10.15468/dl.zfaw79
Anopheles melas	-16.5919	13.2256	Unclassified	GBIF: https://doi.org/10.15468/dl.zfaw79
Anopheles melas	-16.5761	13.1794	Unclassified	GBIF: https://doi.org/10.15468/dl.zfaw79
Anopheles melas	-16.5	16.01667	Unclassified	GBIF: https://doi.org/10.15468/dl.zfaw79
Anopheles melas	-16.38333	13.68333	Unclassified	GBIF: https://doi.org/10.15468/dl.zfaw79
Anopheles melas	-16.36667	13.25	Unclassified	GBIF: https://doi.org/10.15468/dl.zfaw79
Anopheles melas	-16.36667	12.8	Unclassified	GBIF: https://doi.org/10.15468/dl.zfaw79
Anopheles melas	-16.33333	12.41667	Unclassified	GBIF: https://doi.org/10.15468/dl.zfaw79
Anopheles melas	-16.33333	13.86667	Unclassified	GBIF: https://doi.org/10.15468/dl.zfaw79
Anopheles melas	-16.3	12.76667	Unclassified	GBIF: https://doi.org/10.15468/dl.zfaw79

Anopheles melas	-16.3	12.8	Unclassified	GBIF: https://doi.org/10.15468/dl.zfaw79
Anopheles melas	-16.28333	13.46667	Unclassified	GBIF: https://doi.org/10.15468/dl.zfaw79
Anopheles melas	-16	13.33333	Unclassified	GBIF: https://doi.org/10.15468/dl.zfaw79
Anopheles melas	-15.91667	13.91667	Unclassified	GBIF: https://doi.org/10.15468/dl.zfaw79
Anopheles melas	-15.81667	11.93333	Unclassified	GBIF: https://doi.org/10.15468/dl.zfaw79
Anopheles melas	-15.81667	12.3	Unclassified	GBIF: https://doi.org/10.15468/dl.zfaw79
Anopheles melas	-15.8	13.36667	Unclassified	GBIF: https://doi.org/10.15468/dl.zfaw79
Anopheles melas	-15.78333	13.48333	Unclassified	GBIF: https://doi.org/10.15468/dl.zfaw79
Anopheles melas	-15.75	13.41667	Unclassified	GBIF: https://doi.org/10.15468/dl.zfaw79
Anopheles melas	-15.73333	11.23333	Unclassified	GBIF: https://doi.org/10.15468/dl.zfaw79
Anopheles melas	-15.71667	13.43333	Unclassified	GBIF: https://doi.org/10.15468/dl.zfaw79
Anopheles melas	-15.7	16.41667	Unclassified	GBIF: https://doi.org/10.15468/dl.zfaw79
Anopheles melas	-15.66667	12.93333	Unclassified	GBIF: https://doi.org/10.15468/dl.zfaw79
Anopheles melas	-15.65	11.86667	Unclassified	GBIF: https://doi.org/10.15468/dl.zfaw79
Anopheles melas	-15.61667	13.41667	Unclassified	GBIF: https://doi.org/10.15468/dl.zfaw79
Anopheles melas	-15.58333	13.5	Unclassified	GBIF: https://doi.org/10.15468/dl.zfaw79
Anopheles melas	-15.56667	13.48333	Unclassified	GBIF: https://doi.org/10.15468/dl.zfaw79
Anopheles melas	-15.53333	13.3	Unclassified	GBIF: https://doi.org/10.15468/dl.zfaw79
Anopheles melas	-15.36667	12.28333	Unclassified	GBIF: https://doi.org/10.15468/dl.zfaw79
Anopheles melas	-15.26667	11.2	Unclassified	GBIF: https://doi.org/10.15468/dl.zfaw79
Anopheles melas	-15.25	12.45	Unclassified	GBIF: https://doi.org/10.15468/dl.zfaw79
Anopheles melas	-15.16667	11.21667	Unclassified	GBIF: https://doi.org/10.15468/dl.zfaw79
Anopheles melas	-15	11.33333	Unclassified	GBIF: https://doi.org/10.15468/dl.zfaw79
Anopheles melas	-15	13.6	Unclassified	GBIF: https://doi.org/10.15468/dl.zfaw79
Anopheles melas	-15	11.9	Unclassified	GBIF: https://doi.org/10.15468/dl.zfaw79
Anopheles melas	-14.83333	13.51667	Unclassified	GBIF: https://doi.org/10.15468/dl.zfaw79
Anopheles melas	-14.81667	13.13333	Unclassified	GBIF: https://doi.org/10.15468/dl.zfaw79
Anopheles melas	-14.03333	10.2	Unclassified	GBIF: https://doi.org/10.15468/dl.zfaw79
Anopheles melas	-13.71667	9.5	Unclassified	GBIF: https://doi.org/10.15468/dl.zfaw79
Anopheles melas	-13.28333	8.5	Unclassified	GBIF: https://doi.org/10.15468/dl.zfaw79
Anopheles melas	-10.73333	6.33333	Unclassified	GBIF: https://doi.org/10.15468/dl.zfaw79
Anopheles melas	-10.38333	6.16667	Unclassified	GBIF: https://doi.org/10.15468/dl.zfaw79
Anopheles melas	-10.33333	6.31667	Unclassified	GBIF: https://doi.org/10.15468/dl.zfaw79
Anopheles melas	-9.320214	6.18407	Unclassified	GBIF: https://doi.org/10.15468/dl.zfaw79
Anopheles melas	-7.51667	4.41667	Unclassified	GBIF: https://doi.org/10.15468/dl.zfaw79
Anopheles melas	-6.61667	4.75	Unclassified	GBIF: https://doi.org/10.15468/dl.zfaw79
Anopheles melas	-6.16667	4.83333	Unclassified	GBIF: https://doi.org/10.15468/dl.zfaw79
Anopheles melas	-6.13333	4.96667	Unclassified	GBIF: https://doi.org/10.15468/dl.zfaw79
Anopheles melas	-6.1	4.93333	Unclassified	GBIF: https://doi.org/10.15468/dl.zfaw79
Anopheles melas	-5	7.7	Unclassified	GBIF: https://doi.org/10.15468/dl.zfaw79
Anopheles melas	-4.01667	5.31667	Unclassified	GBIF: https://doi.org/10.15468/dl.zfaw79
Anopheles melas	-1.75	4.93333	Unclassified	GBIF: https://doi.org/10.15468/dl.zfaw79

Anopheles melas	-0.25	5.55	Unclassified	GBIF: https://doi.org/10.15468/dl.zfaw79
Anopheles melas	0.586	5.781	Unclassified	GBIF: https://doi.org/10.15468/dl.zfaw79
Anopheles melas	0.58601	5.78101	Unclassified	GBIF: https://doi.org/10.15468/dl.zfaw79
Anopheles melas	1.1	6.08333	Unclassified	GBIF: https://doi.org/10.15468/dl.zfaw79
Anopheles melas	1.53333	6.2	Unclassified	GBIF: https://doi.org/10.15468/dl.zfaw79
Anopheles melas	2.51667	6.4	Unclassified	GBIF: https://doi.org/10.15468/dl.zfaw79
Anopheles melas	3.28333	6.43333	Unclassified	GBIF: https://doi.org/10.15468/dl.zfaw79
Anopheles melas	9.93333	2.93333	Unclassified	GBIF: https://doi.org/10.15468/dl.zfaw79
Anopheles melas	12.45	-5.96667	Unclassified	GBIF: https://doi.org/10.15468/dl.zfaw79
Anopheles melas	13.16667	-9.3	Unclassified	GBIF: https://doi.org/10.15468/dl.zfaw79
Anopheles melas	13.35	-8.78333	Unclassified	GBIF: https://doi.org/10.15468/dl.zfaw79
Anopheles melas	13.56667	-12.33333	Unclassified	GBIF: https://doi.org/10.15468/dl.zfaw79
Anopheles meraukensis	113.768	-24.657	Unclassified	GBIF: https://doi.org/10.15468/dl.zfaw79
Anopheles meraukensis	113.81	-23.93	Unclassified	GBIF: https://doi.org/10.15468/dl.zfaw79
Anopheles meraukensis	126.63333	-14.3	Unclassified	GBIF: https://doi.org/10.15468/dl.zfaw79
Anopheles meraukensis	126.633331	-14.3	Unclassified	GBIF: https://doi.org/10.15468/dl.zfaw79
Anopheles meraukensis	130.6833	-13.75	Unclassified	GBIF: https://doi.org/10.15468/dl.zfaw79
Anopheles meraukensis	130.683304	-13.75	Unclassified	GBIF: https://doi.org/10.15468/dl.zfaw79
Anopheles meraukensis	130.828	-12.452	Unclassified	GBIF: https://doi.org/10.15468/dl.zfaw79
Anopheles meraukensis	130.83	-12.45	Unclassified	GBIF: https://doi.org/10.15468/dl.zfaw79
Anopheles meraukensis	130.833298	-12.45	Unclassified	GBIF: https://doi.org/10.15468/dl.zfaw79
Anopheles meraukensis	130.8333	-12.45	Unclassified	GBIF: https://doi.org/10.15468/dl.zfaw79
Anopheles meraukensis	130.833333	-12.466667	Unclassified	GBIF: https://doi.org/10.15468/dl.zfaw79
Anopheles meraukensis	130.85	-12.384	Unclassified	GBIF: https://doi.org/10.15468/dl.zfaw79
Anopheles meraukensis	130.850006	-12.416667	Unclassified	GBIF: https://doi.org/10.15468/dl.zfaw79
Anopheles meraukensis	130.8667	-12.45	Unclassified	GBIF: https://doi.org/10.15468/dl.zfaw79
Anopheles meraukensis	130.899994	-12.4	Unclassified	GBIF: https://doi.org/10.15468/dl.zfaw79
Anopheles meraukensis	130.91667	-12.41667	Unclassified	GBIF: https://doi.org/10.15468/dl.zfaw79
Anopheles meraukensis	130.916672	-12.416667	Unclassified	GBIF: https://doi.org/10.15468/dl.zfaw79
Anopheles meraukensis	131.025219	-12.435145	Unclassified	GBIF: https://doi.org/10.15468/dl.zfaw79
Anopheles meraukensis	131.03334	-13.066667	Unclassified	GBIF: https://doi.org/10.15468/dl.zfaw79
Anopheles meraukensis	131.03334	-13.06667	Unclassified	GBIF: https://doi.org/10.15468/dl.zfaw79
Anopheles meraukensis	131.100006	-13.233334	Unclassified	GBIF: https://doi.org/10.15468/dl.zfaw79
Anopheles meraukensis	131.10001	-13.23333	Unclassified	GBIF: https://doi.org/10.15468/dl.zfaw79
Anopheles meraukensis	131.105	-13.23999	Unclassified	GBIF: https://doi.org/10.15468/dl.zfaw79
Anopheles meraukensis	131.11	-13.24	Unclassified	GBIF: https://doi.org/10.15468/dl.zfaw79
Anopheles meraukensis	132.36667	-13.35	Unclassified	GBIF: https://doi.org/10.15468/dl.zfaw79
Anopheles meraukensis	132.866699	-12.58333	Unclassified	GBIF: https://doi.org/10.15468/dl.zfaw79
Anopheles meraukensis	132.8667	-12.58333	Unclassified	GBIF: https://doi.org/10.15468/dl.zfaw79
Anopheles meraukensis	134.733337	-14.733334	Unclassified	GBIF: https://doi.org/10.15468/dl.zfaw79
Anopheles meraukensis	134.73334	-14.73333	Unclassified	GBIF: https://doi.org/10.15468/dl.zfaw79
Anopheles meraukensis	136.6	-13.93304	Unclassified	GBIF: https://doi.org/10.15468/dl.zfaw79

Anopheles meraukensis	140.404444	-8.495833	Unclassified	GBIF: https://doi.org/10.15468/dl.zfaw79
Anopheles meraukensis	141.75	-15.466666	Unclassified	GBIF: https://doi.org/10.15468/dl.zfaw79
Anopheles meraukensis	141.75	-15.46667	Unclassified	GBIF: https://doi.org/10.15468/dl.zfaw79
Anopheles meraukensis	141.88333	-12.68333	Unclassified	GBIF: https://doi.org/10.15468/dl.zfaw79
Anopheles meraukensis	141.883331	-12.683333	Unclassified	GBIF: https://doi.org/10.15468/dl.zfaw79
Anopheles meraukensis	142.21666	-10.583333	Unclassified	GBIF: https://doi.org/10.15468/dl.zfaw79
Anopheles meraukensis	142.21666	-10.58333	Unclassified	GBIF: https://doi.org/10.15468/dl.zfaw79
Anopheles meraukensis	142.26666	-10.18333	Unclassified	GBIF: https://doi.org/10.15468/dl.zfaw79
Anopheles meraukensis	142.266663	-10.183333	Unclassified	GBIF: https://doi.org/10.15468/dl.zfaw79
Anopheles meraukensis	142.53334	-11.733334	Unclassified	GBIF: https://doi.org/10.15468/dl.zfaw79
Anopheles meraukensis	142.53334	-11.73333	Unclassified	GBIF: https://doi.org/10.15468/dl.zfaw79
Anopheles meraukensis	143.51666	-12.96667	Unclassified	GBIF: https://doi.org/10.15468/dl.zfaw79
Anopheles meraukensis	143.516663	-12.966666	Unclassified	GBIF: https://doi.org/10.15468/dl.zfaw79
Anopheles meraukensis	145.333328	-16.683332	Unclassified	GBIF: https://doi.org/10.15468/dl.zfaw79
Anopheles meraukensis	145.33333	-16.68333	Unclassified	GBIF: https://doi.org/10.15468/dl.zfaw79
Anopheles meraukensis	145.4833	-16.5	Unclassified	GBIF: https://doi.org/10.15468/dl.zfaw79
Anopheles meraukensis	146.333328	-18.533333	Unclassified	GBIF: https://doi.org/10.15468/dl.zfaw79
Anopheles meraukensis	146.33333	-18.53333	Unclassified	GBIF: https://doi.org/10.15468/dl.zfaw79
Anopheles meraukensis	146.816666	-19.266666	Unclassified	GBIF: https://doi.org/10.15468/dl.zfaw79
Anopheles meraukensis	146.81667	-19.26667	Unclassified	GBIF: https://doi.org/10.15468/dl.zfaw79
Anopheles meraukensis	147.199997	-19.9	Unclassified	GBIF: https://doi.org/10.15468/dl.zfaw79
Anopheles meraukensis	147.2	-19.9	Unclassified	GBIF: https://doi.org/10.15468/dl.zfaw79
Anopheles meraukensis	147.21666	-19.783333	Unclassified	GBIF: https://doi.org/10.15468/dl.zfaw79
Anopheles meraukensis	147.21666	-19.78333	Unclassified	GBIF: https://doi.org/10.15468/dl.zfaw79
Anopheles meraukensis	147.39999	-19.56667	Unclassified	GBIF: https://doi.org/10.15468/dl.zfaw79
Anopheles meraukensis	147.399994	-19.566668	Unclassified	GBIF: https://doi.org/10.15468/dl.zfaw79
Anopheles meraukensis	149.18333	-21.1	Unclassified	GBIF: https://doi.org/10.15468/dl.zfaw79
Anopheles meraukensis	149.183334	-21.1	Unclassified	GBIF: https://doi.org/10.15468/dl.zfaw79
Anopheles meraukensis	149.199997	-21.133333	Unclassified	GBIF: https://doi.org/10.15468/dl.zfaw79
Anopheles meraukensis	152.983337	-27.316668	Unclassified	GBIF: https://doi.org/10.15468/dl.zfaw79
Anopheles meraukensis	152.98334	-27.31667	Unclassified	GBIF: https://doi.org/10.15468/dl.zfaw79
Anopheles meraukensis	153	-26.283333	Unclassified	GBIF: https://doi.org/10.15468/dl.zfaw79
Anopheles meraukensis	153	-26.28333	Unclassified	GBIF: https://doi.org/10.15468/dl.zfaw79
Anopheles meraukensis	153.116699	-26.66667	Unclassified	GBIF: https://doi.org/10.15468/dl.zfaw79
Anopheles meraukensis	153.13333	-27.48333	Unclassified	GBIF: https://doi.org/10.15468/dl.zfaw79
Anopheles meraukensis	153.133331	-27.483334	Unclassified	GBIF: https://doi.org/10.15468/dl.zfaw79
Anopheles meraukensis	153.16667	-27.05	Unclassified	GBIF: https://doi.org/10.15468/dl.zfaw79
Anopheles meraukensis	153.166672	-27.049999	Unclassified	GBIF: https://doi.org/10.15468/dl.zfaw79
Anopheles merus	25.66667	-17.83333	Unclassified	GBIF: https://doi.org/10.15468/dl.zfaw79
Anopheles merus	26.31667	-18.33333	Unclassified	GBIF: https://doi.org/10.15468/dl.zfaw79
Anopheles merus	28.9432	-18.2203	Unclassified	GBIF: https://doi.org/10.15468/dl.zfaw79
Anopheles merus	30.638974	-25.067747	Unclassified	GBIF: https://doi.org/10.15468/dl.zfaw79

Anopheles merus	30.68333	-23.6	Unclassified	GBIF: https://doi.org/10.15468/dl.zfaw79
Anopheles merus	30.8892	-23.5362	Unclassified	GBIF: https://doi.org/10.15468/dl.zfaw79
Anopheles merus	30.9	-23.43333	Unclassified	GBIF: https://doi.org/10.15468/dl.zfaw79
Anopheles merus	31.25	-23.01667	Unclassified	GBIF: https://doi.org/10.15468/dl.zfaw79
Anopheles merus	31.33333	-29.33333	Unclassified	GBIF: https://doi.org/10.15468/dl.zfaw79
Anopheles merus	31.43333	-23.4	Unclassified	GBIF: https://doi.org/10.15468/dl.zfaw79
Anopheles merus	31.58333	-24.98333	Unclassified	GBIF: https://doi.org/10.15468/dl.zfaw79
Anopheles merus	31.63333	-21.11667	Unclassified	GBIF: https://doi.org/10.15468/dl.zfaw79
Anopheles merus	31.83333	-26.83333	Unclassified	GBIF: https://doi.org/10.15468/dl.zfaw79
Anopheles merus	31.93333	-26.83333	Unclassified	GBIF: https://doi.org/10.15468/dl.zfaw79
Anopheles merus	31.93333	-25.43333	Unclassified	GBIF: https://doi.org/10.15468/dl.zfaw79
Anopheles merus	32	-28.53333	Unclassified	GBIF: https://doi.org/10.15468/dl.zfaw79
Anopheles merus	32.05	-25.71667	Unclassified	GBIF: https://doi.org/10.15468/dl.zfaw79
Anopheles merus	32.16667	-27.83333	Unclassified	GBIF: https://doi.org/10.15468/dl.zfaw79
Anopheles merus	32.26667	-27.38333	Unclassified	GBIF: https://doi.org/10.15468/dl.zfaw79
Anopheles merus	32.28333	-27.08333	Unclassified	GBIF: https://doi.org/10.15468/dl.zfaw79
Anopheles merus	32.3	-27.56667	Unclassified	GBIF: https://doi.org/10.15468/dl.zfaw79
Anopheles merus	32.31667	-27.03333	Unclassified	GBIF: https://doi.org/10.15468/dl.zfaw79
Anopheles merus	32.33333	-26.3	Unclassified	GBIF: https://doi.org/10.15468/dl.zfaw79
Anopheles merus	32.33333	-26.53333	Unclassified	GBIF: https://doi.org/10.15468/dl.zfaw79
Anopheles merus	32.5	-27	Unclassified	GBIF: https://doi.org/10.15468/dl.zfaw79
Anopheles merus	32.55	-27.05	Unclassified	GBIF: https://doi.org/10.15468/dl.zfaw79
Anopheles merus	32.55	-25.5	Unclassified	GBIF: https://doi.org/10.15468/dl.zfaw79
Anopheles merus	32.56667	-26.83333	Unclassified	GBIF: https://doi.org/10.15468/dl.zfaw79
Anopheles merus	32.61667	-24.7	Unclassified	GBIF: https://doi.org/10.15468/dl.zfaw79
Anopheles merus	32.66667	-25.9	Unclassified	GBIF: https://doi.org/10.15468/dl.zfaw79
Anopheles merus	32.66667	-25.76667	Unclassified	GBIF: https://doi.org/10.15468/dl.zfaw79
Anopheles merus	32.83333	-26.91667	Unclassified	GBIF: https://doi.org/10.15468/dl.zfaw79
Anopheles merus	33	-25.05	Unclassified	GBIF: https://doi.org/10.15468/dl.zfaw79
Anopheles merus	34.5	-18.96667	Unclassified	GBIF: https://doi.org/10.15468/dl.zfaw79
Anopheles merus	35	-21	Unclassified	GBIF: https://doi.org/10.15468/dl.zfaw79
Anopheles merus	36.73333	-17.83333	Unclassified	GBIF: https://doi.org/10.15468/dl.zfaw79
Anopheles merus	38	-5	Unclassified	GBIF: https://doi.org/10.15468/dl.zfaw79
Anopheles merus	38.01667	-4.66667	Unclassified	GBIF: https://doi.org/10.15468/dl.zfaw79
Anopheles merus	38.16667	-17.23333	Unclassified	GBIF: https://doi.org/10.15468/dl.zfaw79
Anopheles merus	38.55	-5.08333	Unclassified	GBIF: https://doi.org/10.15468/dl.zfaw79
Anopheles merus	38.55	-5.3	Unclassified	GBIF: https://doi.org/10.15468/dl.zfaw79
Anopheles merus	38.91667	-6.43333	Unclassified	GBIF: https://doi.org/10.15468/dl.zfaw79
Anopheles merus	38.95	-6.45	Unclassified	GBIF: https://doi.org/10.15468/dl.zfaw79
Anopheles merus	39.08333	-5.11667	Unclassified	GBIF: https://doi.org/10.15468/dl.zfaw79
Anopheles merus	39.15	-4.86667	Unclassified	GBIF: https://doi.org/10.15468/dl.zfaw79
Anopheles merus	39.2	-6.16667	Unclassified	GBIF: https://doi.org/10.15468/dl.zfaw79

Anopheles merus	39.21667	-4.6	Unclassified	GBIF: https://doi.org/10.15468/dl.zfaw79
Anopheles merus	39.23333	-4.6	Unclassified	GBIF: https://doi.org/10.15468/dl.zfaw79
Anopheles merus	39.3	-6.85	Unclassified	GBIF: https://doi.org/10.15468/dl.zfaw79
Anopheles merus	39.47	-4.17	Unclassified	GBIF: https://doi.org/10.15468/dl.zfaw79
Anopheles merus	39.75	-5	Unclassified	GBIF: https://doi.org/10.15468/dl.zfaw79
Anopheles merus	39.83333	-3.61667	Unclassified	GBIF: https://doi.org/10.15468/dl.zfaw79
Anopheles merus	39.91	-3.89	Unclassified	GBIF: https://doi.org/10.15468/dl.zfaw79
Anopheles merus	39.96667	-3.25	Unclassified	GBIF: https://doi.org/10.15468/dl.zfaw79
Anopheles merus	40	-0.1	Unclassified	GBIF: https://doi.org/10.15468/dl.zfaw79
Anopheles merus	40.11	-3.25	Unclassified	GBIF: https://doi.org/10.15468/dl.zfaw79
Anopheles merus	40.28333	-14.21667	Unclassified	GBIF: https://doi.org/10.15468/dl.zfaw79
Anopheles merus	40.5	-10.75	Unclassified	GBIF: https://doi.org/10.15468/dl.zfaw79
Anopheles merus	42.46667	-0.41667	Unclassified	GBIF: https://doi.org/10.15468/dl.zfaw79
Anopheles merus	43.68333	-23.33333	Unclassified	GBIF: https://doi.org/10.15468/dl.zfaw79
Anopheles merus	45.14743	-12.8206	Unclassified	GBIF: https://doi.org/10.15468/dl.zfaw79
Anopheles merus	57.5	-20.25	Unclassified	GBIF: https://doi.org/10.15468/dl.zfaw79
Anopheles messeae	-5.447585	56.543239	Unclassified	GBIF: https://doi.org/10.15468/dl.zfaw79
Anopheles messeae	-4.721552	51.65776	Unclassified	GBIF: https://doi.org/10.15468/dl.zfaw79
Anopheles messeae	-4.329487	53.339129	Unclassified	GBIF: https://doi.org/10.15468/dl.zfaw79
Anopheles messeae	-4.295652	53.128501	Unclassified	GBIF: https://doi.org/10.15468/dl.zfaw79
Anopheles messeae	-4.174845	53.252144	Unclassified	GBIF: https://doi.org/10.15468/dl.zfaw79
Anopheles messeae	-3.82324	50.05436	Unclassified	GBIF: https://doi.org/10.15468/dl.zfaw79
Anopheles messeae	-3.82316	50.05439	Unclassified	GBIF: https://doi.org/10.15468/dl.zfaw79
Anopheles messeae	-3.73056	53.219419	Unclassified	GBIF: https://doi.org/10.15468/dl.zfaw79
Anopheles messeae	-3.475	50.675	Unclassified	GBIF: https://doi.org/10.15468/dl.zfaw79
Anopheles messeae	-3.47417	50.67793	Unclassified	GBIF: https://doi.org/10.15468/dl.zfaw79
Anopheles messeae	-3.474	50.676	Unclassified	GBIF: https://doi.org/10.15468/dl.zfaw79
Anopheles messeae	-3.473	50.677	Unclassified	GBIF: https://doi.org/10.15468/dl.zfaw79
Anopheles messeae	-3.3131	52.5121	Unclassified	GBIF: https://doi.org/10.15468/dl.zfaw79
Anopheles messeae	-3.018242	53.055939	Unclassified	GBIF: https://doi.org/10.15468/dl.zfaw79
Anopheles messeae	-3.000379	53.021014	Unclassified	GBIF: https://doi.org/10.15468/dl.zfaw79
Anopheles messeae	-2.994307	53.030567	Unclassified	GBIF: https://doi.org/10.15468/dl.zfaw79
Anopheles messeae	-2.986238	53.026544	Unclassified	GBIF: https://doi.org/10.15468/dl.zfaw79
Anopheles messeae	-2.83041	51.575773	Unclassified	GBIF: https://doi.org/10.15468/dl.zfaw79
Anopheles messeae	-2.830394	51.574874	Unclassified	GBIF: https://doi.org/10.15468/dl.zfaw79
Anopheles messeae	-2.812161	51.414006	Unclassified	GBIF: https://doi.org/10.15468/dl.zfaw79
Anopheles messeae	-2.728	51.182	Unclassified	GBIF: https://doi.org/10.15468/dl.zfaw79
Anopheles messeae	-2.727	51.182	Unclassified	GBIF: https://doi.org/10.15468/dl.zfaw79
Anopheles messeae	-2.723	51.182	Unclassified	GBIF: https://doi.org/10.15468/dl.zfaw79
Anopheles messeae	-2.50066	50.92697	Unclassified	GBIF: https://doi.org/10.15468/dl.zfaw79
Anopheles messeae	-2.50056	50.92691	Unclassified	GBIF: https://doi.org/10.15468/dl.zfaw79
Anopheles messeae	-2.50013	50.92692	Unclassified	GBIF: https://doi.org/10.15468/dl.zfaw79

Anopheles messeae	-2.36667	51.7333	Unclassified	GBIF: https://doi.org/10.15468/dl.zfaw79
Anopheles messeae	-2.265542	51.712381	Unclassified	GBIF: https://doi.org/10.15468/dl.zfaw79
Anopheles messeae	-1.564855	54.542529	Unclassified	GBIF: https://doi.org/10.15468/dl.zfaw79
Anopheles messeae	-1.444964	54.064137	Unclassified	GBIF: https://doi.org/10.15468/dl.zfaw79
Anopheles messeae	-1.39	51.72	Unclassified	GBIF: https://doi.org/10.15468/dl.zfaw79
Anopheles messeae	-0.888	51.569	Unclassified	GBIF: https://doi.org/10.15468/dl.zfaw79
Anopheles messeae	-0.63	51.33	Unclassified	GBIF: https://doi.org/10.15468/dl.zfaw79
Anopheles messeae	-0.48	51.37	Unclassified	GBIF: https://doi.org/10.15468/dl.zfaw79
Anopheles messeae	-0.4	51.95	Unclassified	GBIF: https://doi.org/10.15468/dl.zfaw79
Anopheles messeae	-0.2425	52.647691	Unclassified	GBIF: https://doi.org/10.15468/dl.zfaw79
Anopheles messeae	0.09	52.42	Unclassified	GBIF: https://doi.org/10.15468/dl.zfaw79
Anopheles messeae	0.279576	52.310595	Unclassified	GBIF: https://doi.org/10.15468/dl.zfaw79
Anopheles messeae	0.28638	52.31451	Unclassified	GBIF: https://doi.org/10.15468/dl.zfaw79
Anopheles messeae	0.37832	50.93337	Unclassified	GBIF: https://doi.org/10.15468/dl.zfaw79
Anopheles messeae	0.37876	50.93582	Unclassified	GBIF: https://doi.org/10.15468/dl.zfaw79
Anopheles messeae	0.37943	50.93471	Unclassified	GBIF: https://doi.org/10.15468/dl.zfaw79
Anopheles messeae	0.478	51.454	Unclassified	GBIF: https://doi.org/10.15468/dl.zfaw79
Anopheles messeae	0.483	51.478	Unclassified	GBIF: https://doi.org/10.15468/dl.zfaw79
Anopheles messeae	0.49	51.467	Unclassified	GBIF: https://doi.org/10.15468/dl.zfaw79
Anopheles messeae	0.539194	50.938316	Unclassified	GBIF: https://doi.org/10.15468/dl.zfaw79
Anopheles messeae	0.79	51.37	Unclassified	GBIF: https://doi.org/10.15468/dl.zfaw79
Anopheles messeae	1.577	52.661	Unclassified	GBIF: https://doi.org/10.15468/dl.zfaw79
Anopheles messeae	1.577	52.566	Unclassified	GBIF: https://doi.org/10.15468/dl.zfaw79
Anopheles messeae	1.69092	52.46865	Unclassified	GBIF: https://doi.org/10.15468/dl.zfaw79
Anopheles messeae	1.69094	52.46883	Unclassified	GBIF: https://doi.org/10.15468/dl.zfaw79
Anopheles messeae	1.693	52.468	Unclassified	GBIF: https://doi.org/10.15468/dl.zfaw79
Anopheles messeae	1.90291	52.62565	Unclassified	GBIF: https://doi.org/10.15468/dl.zfaw79
Anopheles messeae	1.90292	52.62567	Unclassified	GBIF: https://doi.org/10.15468/dl.zfaw79
Anopheles messeae	4.07	51.04	Unclassified	GBIF: https://doi.org/10.15468/dl.zfaw79
Anopheles messeae	4.52	51	Unclassified	GBIF: https://doi.org/10.15468/dl.zfaw79
Anopheles messeae	4.8	51.07	Unclassified	GBIF: https://doi.org/10.15468/dl.zfaw79
Anopheles messeae	4.95	52.4	Unclassified	GBIF: https://doi.org/10.15468/dl.zfaw79
Anopheles messeae	5.149431	52.078177	Unclassified	GBIF: https://doi.org/10.15468/dl.zfaw79
Anopheles messeae	5.22	50.49	Unclassified	GBIF: https://doi.org/10.15468/dl.zfaw79
Anopheles messeae	5.225254	51.68318	Unclassified	GBIF: https://doi.org/10.15468/dl.zfaw79
Anopheles messeae	5.244773	52.051097	Unclassified	GBIF: https://doi.org/10.15468/dl.zfaw79
Anopheles messeae	5.25155	51.351533	Unclassified	GBIF: https://doi.org/10.15468/dl.zfaw79
Anopheles messeae	5.252009	51.487622	Unclassified	GBIF: https://doi.org/10.15468/dl.zfaw79
Anopheles messeae	5.459292	51.631666	Unclassified	GBIF: https://doi.org/10.15468/dl.zfaw79
Anopheles messeae	5.596527	51.37394	Unclassified	GBIF: https://doi.org/10.15468/dl.zfaw79
Anopheles messeae	5.742086	51.741008	Unclassified	GBIF: https://doi.org/10.15468/dl.zfaw79
Anopheles messeae	5.76	51.02	Unclassified	GBIF: https://doi.org/10.15468/dl.zfaw79

Anopheles messeae	5.87529	51.447488	Unclassified	GBIF: https://doi.org/10.15468/dl.zfaw79
Anopheles messeae	11.058881	60.253885	Unclassified	GBIF: https://doi.org/10.15468/dl.zfaw79
Anopheles messeae	12.00262	59.71311	Unclassified	GBIF: https://doi.org/10.15468/dl.zfaw79
Anopheles messeae	13.32023	59.50213	Unclassified	GBIF: https://doi.org/10.15468/dl.zfaw79
Anopheles messeae	13.63532	59.37585	Unclassified	GBIF: https://doi.org/10.15468/dl.zfaw79
Anopheles messeae	15.69072	56.28054	Unclassified	GBIF: https://doi.org/10.15468/dl.zfaw79
Anopheles messeae	15.84	57.56	Unclassified	GBIF: https://doi.org/10.15468/dl.zfaw79
Anopheles messeae	16.46	57.8	Unclassified	GBIF: https://doi.org/10.15468/dl.zfaw79
Anopheles messeae	16.52	60.73	Unclassified	GBIF: https://doi.org/10.15468/dl.zfaw79
Anopheles messeae	16.52423	59.70103	Unclassified	GBIF: https://doi.org/10.15468/dl.zfaw79
Anopheles messeae	16.68	59.48	Unclassified	GBIF: https://doi.org/10.15468/dl.zfaw79
Anopheles messeae	17.72764	59.72776	Unclassified	GBIF: https://doi.org/10.15468/dl.zfaw79
Anopheles messeae	17.83798	59.11879	Unclassified	GBIF: https://doi.org/10.15468/dl.zfaw79
Anopheles messeae	18.09077	59.51068	Unclassified	GBIF: https://doi.org/10.15468/dl.zfaw79
Anopheles messeae	18.80859	59.86263	Unclassified	GBIF: https://doi.org/10.15468/dl.zfaw79
Anopheles messeae	19.48	60.14	Unclassified	GBIF: https://doi.org/10.15468/dl.zfaw79
Anopheles messeae	19.49	60.16	Unclassified	GBIF: https://doi.org/10.15468/dl.zfaw79
Anopheles messeae	19.83	60.28	Unclassified	GBIF: https://doi.org/10.15468/dl.zfaw79
Anopheles messeae	21.37	60.55	Unclassified	GBIF: https://doi.org/10.15468/dl.zfaw79
Anopheles messeae	21.46	61.59	Unclassified	GBIF: https://doi.org/10.15468/dl.zfaw79
Anopheles messeae	21.82	60.5	Unclassified	GBIF: https://doi.org/10.15468/dl.zfaw79
Anopheles messeae	22.31	60.56	Unclassified	GBIF: https://doi.org/10.15468/dl.zfaw79
Anopheles messeae	23.25	59.84	Unclassified	GBIF: https://doi.org/10.15468/dl.zfaw79
Anopheles messeae	25.33	58.34	Unclassified	GBIF: https://doi.org/10.15468/dl.zfaw79
Anopheles messeae	25.334444	58.335833	Unclassified	GBIF: https://doi.org/10.15468/dl.zfaw79
Anopheles messeae	25.334446	58.33583	Unclassified	GBIF: https://doi.org/10.15468/dl.zfaw79
Anopheles messeae	25.45	61.99	Unclassified	GBIF: https://doi.org/10.15468/dl.zfaw79
Anopheles messeae	25.52	58.08	Unclassified	GBIF: https://doi.org/10.15468/dl.zfaw79
Anopheles messeae	25.82	61.33	Unclassified	GBIF: https://doi.org/10.15468/dl.zfaw79
Anopheles messeae	26.59	60.9	Unclassified	GBIF: https://doi.org/10.15468/dl.zfaw79
Anopheles messeae	26.679167	58.353611	Unclassified	GBIF: https://doi.org/10.15468/dl.zfaw79
Anopheles messeae	26.679167	58.35361	Unclassified	GBIF: https://doi.org/10.15468/dl.zfaw79
Anopheles messeae	26.68	58.35	Unclassified	GBIF: https://doi.org/10.15468/dl.zfaw79
Anopheles messeae	26.716667	58.357222	Unclassified	GBIF: https://doi.org/10.15468/dl.zfaw79
Anopheles messeae	26.716667	58.35722	Unclassified	GBIF: https://doi.org/10.15468/dl.zfaw79
Anopheles messeae	26.72	58.35	Unclassified	GBIF: https://doi.org/10.15468/dl.zfaw79
Anopheles messeae	27.52	53.84	Unclassified	GBIF: https://doi.org/10.15468/dl.zfaw79
Anopheles messeae	27.87	52.21	Unclassified	GBIF: https://doi.org/10.15468/dl.zfaw79
Anopheles messeae	28.18	61.57	Unclassified	GBIF: https://doi.org/10.15468/dl.zfaw79
Anopheles messeae	29.27	52.07	Unclassified	GBIF: https://doi.org/10.15468/dl.zfaw79
Anopheles messeae	29.52	45.15	Unclassified	GBIF: https://doi.org/10.15468/dl.zfaw79
Anopheles messeae	29.63	45.06	Unclassified	GBIF: https://doi.org/10.15468/dl.zfaw79

Anopheles messeae	29.67	62.63	Unclassified	GBIF: https://doi.org/10.15468/dl.zfaw79
Anopheles messeae	30.140078	69.26724	Unclassified	GBIF: https://doi.org/10.15468/dl.zfaw79
Anopheles messeae	30.257596	47.425257	Unclassified	GBIF: https://doi.org/10.15468/dl.zfaw79
Anopheles messeae	30.257596	46.425017	Unclassified	GBIF: https://doi.org/10.15468/dl.zfaw79
Anopheles messeae	30.257596	46.428303	Unclassified	GBIF: https://doi.org/10.15468/dl.zfaw79
Anopheles messeae	30.26206	46.411307	Unclassified	GBIF: https://doi.org/10.15468/dl.zfaw79
Anopheles messeae	30.582815	46.411009	Unclassified	GBIF: https://doi.org/10.15468/dl.zfaw79
Anopheles messeae	32.272375	46.238521	Unclassified	GBIF: https://doi.org/10.15468/dl.zfaw79
Anopheles messeae	32.556117	46.520186	Unclassified	GBIF: https://doi.org/10.15468/dl.zfaw79
Anopheles messeae	34.71	53.17	Unclassified	GBIF: https://doi.org/10.15468/dl.zfaw79
Anopheles messeae	34.76333	64.53032	Unclassified	GBIF: https://doi.org/10.15468/dl.zfaw79
Anopheles messeae	34.94	53.92	Unclassified	GBIF: https://doi.org/10.15468/dl.zfaw79
Anopheles messeae	37.01	56.13	Unclassified	GBIF: https://doi.org/10.15468/dl.zfaw79
Anopheles messeae	37.23	44.54	Unclassified	GBIF: https://doi.org/10.15468/dl.zfaw79
Anopheles messeae	40.51575	64.53991	Unclassified	GBIF: https://doi.org/10.15468/dl.zfaw79
Anopheles messeae	44.42	53.03	Unclassified	GBIF: https://doi.org/10.15468/dl.zfaw79
Anopheles messeae	45.05	48.3	Unclassified	GBIF: https://doi.org/10.15468/dl.zfaw79
Anopheles messeae	46.42	47.33	Unclassified	GBIF: https://doi.org/10.15468/dl.zfaw79
Anopheles messeae	47.35	45.43	Unclassified	GBIF: https://doi.org/10.15468/dl.zfaw79
Anopheles messeae	53.684025	63.562626	Unclassified	GBIF: https://doi.org/10.15468/dl.zfaw79
Anopheles messeae	60.1	55	Unclassified	GBIF: https://doi.org/10.15468/dl.zfaw79
Anopheles messeae	69.4	53.3	Unclassified	GBIF: https://doi.org/10.15468/dl.zfaw79
Anopheles messeae	82.6	57.9	Unclassified	GBIF: https://doi.org/10.15468/dl.zfaw79
Anopheles messeae	82.8	54.8	Unclassified	GBIF: https://doi.org/10.15468/dl.zfaw79
Anopheles messeae	82.81	54.83	Unclassified	GBIF: https://doi.org/10.15468/dl.zfaw79
Anopheles messeae	82.82	54.92	Unclassified	GBIF: https://doi.org/10.15468/dl.zfaw79
Anopheles messeae	84.3	49.7	Unclassified	GBIF: https://doi.org/10.15468/dl.zfaw79
Anopheles messeae	86.1	57	Unclassified	GBIF: https://doi.org/10.15468/dl.zfaw79
Anopheles messeae	114.9	60.7	Unclassified	GBIF: https://doi.org/10.15468/dl.zfaw79
Anopheles milloti	44.862599	-20.492464	Unclassified	GBIF: https://doi.org/10.15468/dl.zfaw79
Anopheles milloti	46.7	-15.883333	Unclassified	GBIF: https://doi.org/10.15468/dl.zfaw79
Anopheles minimus	85.570255	27.704429	Unclassified	GBIF: https://doi.org/10.15468/dl.zfaw79
Anopheles minimus	97.55084	18.422576	Unclassified	GBIF: https://doi.org/10.15468/dl.zfaw79
Anopheles minimus	97.67712	18.523387	Unclassified	GBIF: https://doi.org/10.15468/dl.zfaw79
Anopheles minimus	97.682293	18.341793	Unclassified	GBIF: https://doi.org/10.15468/dl.zfaw79
Anopheles minimus	97.684865	18.377053	Unclassified	GBIF: https://doi.org/10.15468/dl.zfaw79
Anopheles minimus	97.688304	18.072015	Unclassified	GBIF: https://doi.org/10.15468/dl.zfaw79
Anopheles minimus	97.691357	17.886409	Unclassified	GBIF: https://doi.org/10.15468/dl.zfaw79
Anopheles minimus	97.696452	17.834933	Unclassified	GBIF: https://doi.org/10.15468/dl.zfaw79
Anopheles minimus	97.724506	17.870362	Unclassified	GBIF: https://doi.org/10.15468/dl.zfaw79
Anopheles minimus	97.725302	18.41257	Unclassified	GBIF: https://doi.org/10.15468/dl.zfaw79
Anopheles minimus	97.744867	17.927427	Unclassified	GBIF: https://doi.org/10.15468/dl.zfaw79

Anopheles minimus	97.756216	17.889755	Unclassified	GBIF: https://doi.org/10.15468/dl.zfaw79
Anopheles minimus	97.762666	18.251192	Unclassified	GBIF: https://doi.org/10.15468/dl.zfaw79
Anopheles minimus	97.772716	17.858923	Unclassified	GBIF: https://doi.org/10.15468/dl.zfaw79
Anopheles minimus	97.78651	17.993799	Unclassified	GBIF: https://doi.org/10.15468/dl.zfaw79
Anopheles minimus	97.799208	18.674208	Unclassified	GBIF: https://doi.org/10.15468/dl.zfaw79
Anopheles minimus	97.804777	18.142886	Unclassified	GBIF: https://doi.org/10.15468/dl.zfaw79
Anopheles minimus	97.809343	18.851386	Unclassified	GBIF: https://doi.org/10.15468/dl.zfaw79
Anopheles minimus	97.816897	17.850059	Unclassified	GBIF: https://doi.org/10.15468/dl.zfaw79
Anopheles minimus	97.821452	19.419831	Unclassified	GBIF: https://doi.org/10.15468/dl.zfaw79
Anopheles minimus	97.82409	18.311524	Unclassified	GBIF: https://doi.org/10.15468/dl.zfaw79
Anopheles minimus	97.824277	18.17254	Unclassified	GBIF: https://doi.org/10.15468/dl.zfaw79
Anopheles minimus	97.826148	18.754803	Unclassified	GBIF: https://doi.org/10.15468/dl.zfaw79
Anopheles minimus	97.834183	18.689791	Unclassified	GBIF: https://doi.org/10.15468/dl.zfaw79
Anopheles minimus	97.839458	18.611205	Unclassified	GBIF: https://doi.org/10.15468/dl.zfaw79
Anopheles minimus	97.84472	18.533523	Unclassified	GBIF: https://doi.org/10.15468/dl.zfaw79
Anopheles minimus	97.846079	19.438964	Unclassified	GBIF: https://doi.org/10.15468/dl.zfaw79
Anopheles minimus	97.846468	17.83678	Unclassified	GBIF: https://doi.org/10.15468/dl.zfaw79
Anopheles minimus	97.847584	18.810962	Unclassified	GBIF: https://doi.org/10.15468/dl.zfaw79
Anopheles minimus	97.84906	17.873848	Unclassified	GBIF: https://doi.org/10.15468/dl.zfaw79
Anopheles minimus	97.850588	18.64742	Unclassified	GBIF: https://doi.org/10.15468/dl.zfaw79
Anopheles minimus	97.853575	18.906785	Unclassified	GBIF: https://doi.org/10.15468/dl.zfaw79
Anopheles minimus	97.855902	18.418824	Unclassified	GBIF: https://doi.org/10.15468/dl.zfaw79
Anopheles minimus	97.86669	18.933071	Unclassified	GBIF: https://doi.org/10.15468/dl.zfaw79
Anopheles minimus	97.871028	18.998232	Unclassified	GBIF: https://doi.org/10.15468/dl.zfaw79
Anopheles minimus	97.876015	19.379515	Unclassified	GBIF: https://doi.org/10.15468/dl.zfaw79
Anopheles minimus	97.878886	18.378295	Unclassified	GBIF: https://doi.org/10.15468/dl.zfaw79
Anopheles minimus	97.880463	18.427103	Unclassified	GBIF: https://doi.org/10.15468/dl.zfaw79
Anopheles minimus	97.884694	18.210241	Unclassified	GBIF: https://doi.org/10.15468/dl.zfaw79
Anopheles minimus	97.886859	17.714105	Unclassified	GBIF: https://doi.org/10.15468/dl.zfaw79
Anopheles minimus	97.891199	19.198597	Unclassified	GBIF: https://doi.org/10.15468/dl.zfaw79
Anopheles minimus	97.891864	18.272638	Unclassified	GBIF: https://doi.org/10.15468/dl.zfaw79
Anopheles minimus	97.896788	18.539256	Unclassified	GBIF: https://doi.org/10.15468/dl.zfaw79
Anopheles minimus	97.896951	18.513954	Unclassified	GBIF: https://doi.org/10.15468/dl.zfaw79
Anopheles minimus	97.898893	18.800428	Unclassified	GBIF: https://doi.org/10.15468/dl.zfaw79
Anopheles minimus	97.898905	18.798621	Unclassified	GBIF: https://doi.org/10.15468/dl.zfaw79
Anopheles minimus	97.900708	17.607539	Unclassified	GBIF: https://doi.org/10.15468/dl.zfaw79
Anopheles minimus	97.900873	17.889511	Unclassified	GBIF: https://doi.org/10.15468/dl.zfaw79
Anopheles minimus	97.904747	18.479659	Unclassified	GBIF: https://doi.org/10.15468/dl.zfaw79
Anopheles minimus	97.904764	18.476948	Unclassified	GBIF: https://doi.org/10.15468/dl.zfaw79
Anopheles minimus	97.911314	18.034168	Unclassified	GBIF: https://doi.org/10.15468/dl.zfaw79
Anopheles minimus	97.911956	18.387524	Unclassified	GBIF: https://doi.org/10.15468/dl.zfaw79
Anopheles minimus	97.91318	17.570554	Unclassified	GBIF: https://doi.org/10.15468/dl.zfaw79

Anopheles minimus	97.913223	17.563324	Unclassified	GBIF: https://doi.org/10.15468/dl.zfaw79
Anopheles minimus	97.915494	18.132221	Unclassified	GBIF: https://doi.org/10.15468/dl.zfaw79
Anopheles minimus	97.915758	17.772104	Unclassified	GBIF: https://doi.org/10.15468/dl.zfaw79
Anopheles minimus	97.918359	18.146971	Unclassified	GBIF: https://doi.org/10.15468/dl.zfaw79
Anopheles minimus	97.924769	19.057216	Unclassified	GBIF: https://doi.org/10.15468/dl.zfaw79
Anopheles minimus	97.924988	17.960138	Unclassified	GBIF: https://doi.org/10.15468/dl.zfaw79
Anopheles minimus	97.926781	18.741853	Unclassified	GBIF: https://doi.org/10.15468/dl.zfaw79
Anopheles minimus	97.928655	18.745478	Unclassified	GBIF: https://doi.org/10.15468/dl.zfaw79
Anopheles minimus	97.930401	19.068093	Unclassified	GBIF: https://doi.org/10.15468/dl.zfaw79
Anopheles minimus	97.932839	18.53133	Unclassified	GBIF: https://doi.org/10.15468/dl.zfaw79
Anopheles minimus	97.933273	17.678956	Unclassified	GBIF: https://doi.org/10.15468/dl.zfaw79
Anopheles minimus	97.934013	18.029775	Unclassified	GBIF: https://doi.org/10.15468/dl.zfaw79
Anopheles minimus	97.935662	19.158621	Unclassified	GBIF: https://doi.org/10.15468/dl.zfaw79
Anopheles minimus	97.935824	18.81239	Unclassified	GBIF: https://doi.org/10.15468/dl.zfaw79
Anopheles minimus	97.938127	18.131016	Unclassified	GBIF: https://doi.org/10.15468/dl.zfaw79
Anopheles minimus	97.939412	18.231336	Unclassified	GBIF: https://doi.org/10.15468/dl.zfaw79
Anopheles minimus	97.93968	18.650655	Unclassified	GBIF: https://doi.org/10.15468/dl.zfaw79
Anopheles minimus	97.939742	17.832553	Unclassified	GBIF: https://doi.org/10.15468/dl.zfaw79
Anopheles minimus	97.939987	18.753675	Unclassified	GBIF: https://doi.org/10.15468/dl.zfaw79
Anopheles minimus	97.940201	18.719337	Unclassified	GBIF: https://doi.org/10.15468/dl.zfaw79
Anopheles minimus	97.941246	17.947884	Unclassified	GBIF: https://doi.org/10.15468/dl.zfaw79
Anopheles minimus	97.942017	18.112963	Unclassified	GBIF: https://doi.org/10.15468/dl.zfaw79
Anopheles minimus	97.944509	19.240768	Unclassified	GBIF: https://doi.org/10.15468/dl.zfaw79
Anopheles minimus	97.945471	18.938958	Unclassified	GBIF: https://doi.org/10.15468/dl.zfaw79
Anopheles minimus	97.948904	18.382309	Unclassified	GBIF: https://doi.org/10.15468/dl.zfaw79
Anopheles minimus	97.949883	19.589592	Unclassified	GBIF: https://doi.org/10.15468/dl.zfaw79
Anopheles minimus	97.954144	18.484729	Unclassified	GBIF: https://doi.org/10.15468/dl.zfaw79
Anopheles minimus	97.960051	18.558135	Unclassified	GBIF: https://doi.org/10.15468/dl.zfaw79
Anopheles minimus	97.960614	18.164577	Unclassified	GBIF: https://doi.org/10.15468/dl.zfaw79
Anopheles minimus	97.96116	17.583464	Unclassified	GBIF: https://doi.org/10.15468/dl.zfaw79
Anopheles minimus	97.961414	17.922707	Unclassified	GBIF: https://doi.org/10.15468/dl.zfaw79
Anopheles minimus	97.962888	19.493886	Unclassified	GBIF: https://doi.org/10.15468/dl.zfaw79
Anopheles minimus	97.965569	19.220105	Unclassified	GBIF: https://doi.org/10.15468/dl.zfaw79
Anopheles minimus	97.979977	18.251273	Unclassified	GBIF: https://doi.org/10.15468/dl.zfaw79
Anopheles minimus	97.982524	19.235492	Unclassified	GBIF: https://doi.org/10.15468/dl.zfaw79
Anopheles minimus	97.98278	19.205743	Unclassified	GBIF: https://doi.org/10.15468/dl.zfaw79
Anopheles minimus	97.982849	18.328586	Unclassified	GBIF: https://doi.org/10.15468/dl.zfaw79
Anopheles minimus	97.992498	18.332777	Unclassified	GBIF: https://doi.org/10.15468/dl.zfaw79
Anopheles minimus	97.993719	17.995753	Unclassified	GBIF: https://doi.org/10.15468/dl.zfaw79
Anopheles minimus	97.996656	19.01668	Unclassified	GBIF: https://doi.org/10.15468/dl.zfaw79
Anopheles minimus	98.000497	17.78334	Unclassified	GBIF: https://doi.org/10.15468/dl.zfaw79
Anopheles minimus	98.004753	18.860719	Unclassified	GBIF: https://doi.org/10.15468/dl.zfaw79

Anopheles minimus	98.00489	18.350394	Unclassified	GBIF: https://doi.org/10.15468/dl.zfaw79
Anopheles minimus	98.006081	18.421649	Unclassified	GBIF: https://doi.org/10.15468/dl.zfaw79
Anopheles minimus	98.016401	18.297805	Unclassified	GBIF: https://doi.org/10.15468/dl.zfaw79
Anopheles minimus	98.019926	17.867552	Unclassified	GBIF: https://doi.org/10.15468/dl.zfaw79
Anopheles minimus	98.021648	18.739661	Unclassified	GBIF: https://doi.org/10.15468/dl.zfaw79
Anopheles minimus	98.022862	19.291325	Unclassified	GBIF: https://doi.org/10.15468/dl.zfaw79
Anopheles minimus	98.036012	17.506116	Unclassified	GBIF: https://doi.org/10.15468/dl.zfaw79
Anopheles minimus	98.037481	18.791254	Unclassified	GBIF: https://doi.org/10.15468/dl.zfaw79
Anopheles minimus	98.039303	18.636731	Unclassified	GBIF: https://doi.org/10.15468/dl.zfaw79
Anopheles minimus	98.040266	19.710568	Unclassified	GBIF: https://doi.org/10.15468/dl.zfaw79
Anopheles minimus	98.042889	17.451921	Unclassified	GBIF: https://doi.org/10.15468/dl.zfaw79
Anopheles minimus	98.043789	18.681939	Unclassified	GBIF: https://doi.org/10.15468/dl.zfaw79
Anopheles minimus	98.051291	17.689577	Unclassified	GBIF: https://doi.org/10.15468/dl.zfaw79
Anopheles minimus	98.054507	18.819829	Unclassified	GBIF: https://doi.org/10.15468/dl.zfaw79
Anopheles minimus	98.055716	17.702336	Unclassified	GBIF: https://doi.org/10.15468/dl.zfaw79
Anopheles minimus	98.056148	19.78454	Unclassified	GBIF: https://doi.org/10.15468/dl.zfaw79
Anopheles minimus	98.062728	17.439362	Unclassified	GBIF: https://doi.org/10.15468/dl.zfaw79
Anopheles minimus	98.065015	19.677655	Unclassified	GBIF: https://doi.org/10.15468/dl.zfaw79
Anopheles minimus	98.068329	18.702848	Unclassified	GBIF: https://doi.org/10.15468/dl.zfaw79
Anopheles minimus	98.070217	17.692326	Unclassified	GBIF: https://doi.org/10.15468/dl.zfaw79
Anopheles minimus	98.077687	18.373036	Unclassified	GBIF: https://doi.org/10.15468/dl.zfaw79
Anopheles minimus	98.081706	19.205361	Unclassified	GBIF: https://doi.org/10.15468/dl.zfaw79
Anopheles minimus	98.0852	18.562855	Unclassified	GBIF: https://doi.org/10.15468/dl.zfaw79
Anopheles minimus	98.08554	18.190335	Unclassified	GBIF: https://doi.org/10.15468/dl.zfaw79
Anopheles minimus	98.0871	18.54576	Unclassified	GBIF: https://doi.org/10.15468/dl.zfaw79
Anopheles minimus	98.088941	17.658207	Unclassified	GBIF: https://doi.org/10.15468/dl.zfaw79
Anopheles minimus	98.08935	18.11649	Unclassified	GBIF: https://doi.org/10.15468/dl.zfaw79
Anopheles minimus	98.095186	19.648125	Unclassified	GBIF: https://doi.org/10.15468/dl.zfaw79
Anopheles minimus	98.099076	17.515451	Unclassified	GBIF: https://doi.org/10.15468/dl.zfaw79
Anopheles minimus	98.099094	19.718793	Unclassified	GBIF: https://doi.org/10.15468/dl.zfaw79
Anopheles minimus	98.1	24.2	Unclassified	GBIF: https://doi.org/10.15468/dl.zfaw79
Anopheles minimus	98.101065	19.624267	Unclassified	GBIF: https://doi.org/10.15468/dl.zfaw79
Anopheles minimus	98.104769	19.757131	Unclassified	GBIF: https://doi.org/10.15468/dl.zfaw79
Anopheles minimus	98.105715	19.690714	Unclassified	GBIF: https://doi.org/10.15468/dl.zfaw79
Anopheles minimus	98.112886	18.835889	Unclassified	GBIF: https://doi.org/10.15468/dl.zfaw79
Anopheles minimus	98.113234	17.702604	Unclassified	GBIF: https://doi.org/10.15468/dl.zfaw79
Anopheles minimus	98.114246	18.141096	Unclassified	GBIF: https://doi.org/10.15468/dl.zfaw79
Anopheles minimus	98.115839	18.447453	Unclassified	GBIF: https://doi.org/10.15468/dl.zfaw79
Anopheles minimus	98.116084	19.533555	Unclassified	GBIF: https://doi.org/10.15468/dl.zfaw79
Anopheles minimus	98.116815	17.675359	Unclassified	GBIF: https://doi.org/10.15468/dl.zfaw79
Anopheles minimus	98.126585	18.470102	Unclassified	GBIF: https://doi.org/10.15468/dl.zfaw79
Anopheles minimus	98.138165	19.608595	Unclassified	GBIF: https://doi.org/10.15468/dl.zfaw79

Anopheles minimus	98.139303	18.561896	Unclassified	GBIF: https://doi.org/10.15468/dl.zfaw79
Anopheles minimus	98.140662	19.662243	Unclassified	GBIF: https://doi.org/10.15468/dl.zfaw79
Anopheles minimus	98.143289	17.358119	Unclassified	GBIF: https://doi.org/10.15468/dl.zfaw79
Anopheles minimus	98.145751	17.404486	Unclassified	GBIF: https://doi.org/10.15468/dl.zfaw79
Anopheles minimus	98.151179	17.582298	Unclassified	GBIF: https://doi.org/10.15468/dl.zfaw79
Anopheles minimus	98.161384	19.501423	Unclassified	GBIF: https://doi.org/10.15468/dl.zfaw79
Anopheles minimus	98.161811	19.551702	Unclassified	GBIF: https://doi.org/10.15468/dl.zfaw79
Anopheles minimus	98.163519	18.589571	Unclassified	GBIF: https://doi.org/10.15468/dl.zfaw79
Anopheles minimus	98.163604	17.293081	Unclassified	GBIF: https://doi.org/10.15468/dl.zfaw79
Anopheles minimus	98.167633	17.326326	Unclassified	GBIF: https://doi.org/10.15468/dl.zfaw79
Anopheles minimus	98.182382	17.359504	Unclassified	GBIF: https://doi.org/10.15468/dl.zfaw79
Anopheles minimus	98.189487	18.350047	Unclassified	GBIF: https://doi.org/10.15468/dl.zfaw79
Anopheles minimus	98.191787	19.688528	Unclassified	GBIF: https://doi.org/10.15468/dl.zfaw79
Anopheles minimus	98.20281	18.10796	Unclassified	GBIF: https://doi.org/10.15468/dl.zfaw79
Anopheles minimus	98.209376	18.758623	Unclassified	GBIF: https://doi.org/10.15468/dl.zfaw79
Anopheles minimus	98.227472	17.223143	Unclassified	GBIF: https://doi.org/10.15468/dl.zfaw79
Anopheles minimus	98.228235	17.36258	Unclassified	GBIF: https://doi.org/10.15468/dl.zfaw79
Anopheles minimus	98.2331	17.22678	Unclassified	GBIF: https://doi.org/10.15468/dl.zfaw79
Anopheles minimus	98.252256	18.684351	Unclassified	GBIF: https://doi.org/10.15468/dl.zfaw79
Anopheles minimus	98.256188	17.311012	Unclassified	GBIF: https://doi.org/10.15468/dl.zfaw79
Anopheles minimus	98.259209	19.493173	Unclassified	GBIF: https://doi.org/10.15468/dl.zfaw79
Anopheles minimus	98.259625	8.750061	Unclassified	GBIF: https://doi.org/10.15468/dl.zfaw79
Anopheles minimus	98.262847	19.326369	Unclassified	GBIF: https://doi.org/10.15468/dl.zfaw79
Anopheles minimus	98.264829	19.538395	Unclassified	GBIF: https://doi.org/10.15468/dl.zfaw79
Anopheles minimus	98.265175	19.415867	Unclassified	GBIF: https://doi.org/10.15468/dl.zfaw79
Anopheles minimus	98.270636	17.248612	Unclassified	GBIF: https://doi.org/10.15468/dl.zfaw79
Anopheles minimus	98.279571	19.574489	Unclassified	GBIF: https://doi.org/10.15468/dl.zfaw79
Anopheles minimus	98.279913	8.599055	Unclassified	GBIF: https://doi.org/10.15468/dl.zfaw79
Anopheles minimus	98.280448	8.795324	Unclassified	GBIF: https://doi.org/10.15468/dl.zfaw79
Anopheles minimus	98.2807	19.379758	Unclassified	GBIF: https://doi.org/10.15468/dl.zfaw79
Anopheles minimus	98.293339	19.455937	Unclassified	GBIF: https://doi.org/10.15468/dl.zfaw79
Anopheles minimus	98.29335	19.120191	Unclassified	GBIF: https://doi.org/10.15468/dl.zfaw79
Anopheles minimus	98.301521	17.2903	Unclassified	GBIF: https://doi.org/10.15468/dl.zfaw79
Anopheles minimus	98.303117	8.329563	Unclassified	GBIF: https://doi.org/10.15468/dl.zfaw79
Anopheles minimus	98.306828	19.584663	Unclassified	GBIF: https://doi.org/10.15468/dl.zfaw79
Anopheles minimus	98.320916	9.037794	Unclassified	GBIF: https://doi.org/10.15468/dl.zfaw79
Anopheles minimus	98.330041	17.209048	Unclassified	GBIF: https://doi.org/10.15468/dl.zfaw79
Anopheles minimus	98.343518	18.661537	Unclassified	GBIF: https://doi.org/10.15468/dl.zfaw79
Anopheles minimus	98.356373	8.525927	Unclassified	GBIF: https://doi.org/10.15468/dl.zfaw79
Anopheles minimus	98.366079	19.216506	Unclassified	GBIF: https://doi.org/10.15468/dl.zfaw79
Anopheles minimus	98.368106	14.679777	Unclassified	GBIF: https://doi.org/10.15468/dl.zfaw79
Anopheles minimus	98.369411	17.247138	Unclassified	GBIF: https://doi.org/10.15468/dl.zfaw79

Anopheles minimus	98.37394	9.175242	Unclassified	GBIF: https://doi.org/10.15468/dl.zfaw79
Anopheles minimus	98.375289	8.610302	Unclassified	GBIF: https://doi.org/10.15468/dl.zfaw79
Anopheles minimus	98.37818	18.49362	Unclassified	GBIF: https://doi.org/10.15468/dl.zfaw79
Anopheles minimus	98.38406	8.253719	Unclassified	GBIF: https://doi.org/10.15468/dl.zfaw79
Anopheles minimus	98.390204	19.381061	Unclassified	GBIF: https://doi.org/10.15468/dl.zfaw79
Anopheles minimus	98.391201	8.333326	Unclassified	GBIF: https://doi.org/10.15468/dl.zfaw79
Anopheles minimus	98.3922	17.182128	Unclassified	GBIF: https://doi.org/10.15468/dl.zfaw79
Anopheles minimus	98.393148	8.248306	Unclassified	GBIF: https://doi.org/10.15468/dl.zfaw79
Anopheles minimus	98.399541	19.431699	Unclassified	GBIF: https://doi.org/10.15468/dl.zfaw79
Anopheles minimus	98.404426	19.398279	Unclassified	GBIF: https://doi.org/10.15468/dl.zfaw79
Anopheles minimus	98.404687	8.422892	Unclassified	GBIF: https://doi.org/10.15468/dl.zfaw79
Anopheles minimus	98.407486	8.374053	Unclassified	GBIF: https://doi.org/10.15468/dl.zfaw79
Anopheles minimus	98.407775	9.330989	Unclassified	GBIF: https://doi.org/10.15468/dl.zfaw79
Anopheles minimus	98.411169	8.927604	Unclassified	GBIF: https://doi.org/10.15468/dl.zfaw79
Anopheles minimus	98.414655	9.17601	Unclassified	GBIF: https://doi.org/10.15468/dl.zfaw79
Anopheles minimus	98.415368	8.251888	Unclassified	GBIF: https://doi.org/10.15468/dl.zfaw79
Anopheles minimus	98.415571	8.433762	Unclassified	GBIF: https://doi.org/10.15468/dl.zfaw79
Anopheles minimus	98.41762	18.406039	Unclassified	GBIF: https://doi.org/10.15468/dl.zfaw79
Anopheles minimus	98.420755	9.042557	Unclassified	GBIF: https://doi.org/10.15468/dl.zfaw79
Anopheles minimus	98.421046	9.008118	Unclassified	GBIF: https://doi.org/10.15468/dl.zfaw79
Anopheles minimus	98.421094	8.97827	Unclassified	GBIF: https://doi.org/10.15468/dl.zfaw79
Anopheles minimus	98.42475	8.591151	Unclassified	GBIF: https://doi.org/10.15468/dl.zfaw79
Anopheles minimus	98.425492	19.365813	Unclassified	GBIF: https://doi.org/10.15468/dl.zfaw79
Anopheles minimus	98.428512	19.31883	Unclassified	GBIF: https://doi.org/10.15468/dl.zfaw79
Anopheles minimus	98.430765	17.098101	Unclassified	GBIF: https://doi.org/10.15468/dl.zfaw79
Anopheles minimus	98.431969	8.402129	Unclassified	GBIF: https://doi.org/10.15468/dl.zfaw79
Anopheles minimus	98.438505	8.277316	Unclassified	GBIF: https://doi.org/10.15468/dl.zfaw79
Anopheles minimus	98.440819	8.563142	Unclassified	GBIF: https://doi.org/10.15468/dl.zfaw79
Anopheles minimus	98.449181	19.400227	Unclassified	GBIF: https://doi.org/10.15468/dl.zfaw79
Anopheles minimus	98.450802	9.140403	Unclassified	GBIF: https://doi.org/10.15468/dl.zfaw79
Anopheles minimus	98.454249	19.308065	Unclassified	GBIF: https://doi.org/10.15468/dl.zfaw79
Anopheles minimus	98.454464	17.504818	Unclassified	GBIF: https://doi.org/10.15468/dl.zfaw79
Anopheles minimus	98.459221	18.420279	Unclassified	GBIF: https://doi.org/10.15468/dl.zfaw79
Anopheles minimus	98.45925	9.17141	Unclassified	GBIF: https://doi.org/10.15468/dl.zfaw79
Anopheles minimus	98.460114	8.411213	Unclassified	GBIF: https://doi.org/10.15468/dl.zfaw79
Anopheles minimus	98.461775	17.120149	Unclassified	GBIF: https://doi.org/10.15468/dl.zfaw79
Anopheles minimus	98.462582	8.492644	Unclassified	GBIF: https://doi.org/10.15468/dl.zfaw79
Anopheles minimus	98.46274	17.176905	Unclassified	GBIF: https://doi.org/10.15468/dl.zfaw79
Anopheles minimus	98.464262	8.501555	Unclassified	GBIF: https://doi.org/10.15468/dl.zfaw79
Anopheles minimus	98.466359	8.493532	Unclassified	GBIF: https://doi.org/10.15468/dl.zfaw79
Anopheles minimus	98.469358	8.44988	Unclassified	GBIF: https://doi.org/10.15468/dl.zfaw79
Anopheles minimus	98.471898	8.429319	Unclassified	GBIF: https://doi.org/10.15468/dl.zfaw79

Anopheles minimus	98.476391	8.465506	Unclassified	GBIF: https://doi.org/10.15468/dl.zfaw79
Anopheles minimus	98.477145	19.291865	Unclassified	GBIF: https://doi.org/10.15468/dl.zfaw79
Anopheles minimus	98.481216	9.170429	Unclassified	GBIF: https://doi.org/10.15468/dl.zfaw79
Anopheles minimus	98.485906	8.819177	Unclassified	GBIF: https://doi.org/10.15468/dl.zfaw79
Anopheles minimus	98.487822	8.747725	Unclassified	GBIF: https://doi.org/10.15468/dl.zfaw79
Anopheles minimus	98.492188	17.040433	Unclassified	GBIF: https://doi.org/10.15468/dl.zfaw79
Anopheles minimus	98.4925	16.981409	Unclassified	GBIF: https://doi.org/10.15468/dl.zfaw79
Anopheles minimus	98.498753	8.511742	Unclassified	GBIF: https://doi.org/10.15468/dl.zfaw79
Anopheles minimus	98.505023	19.515158	Unclassified	GBIF: https://doi.org/10.15468/dl.zfaw79
Anopheles minimus	98.507149	14.740746	Unclassified	GBIF: https://doi.org/10.15468/dl.zfaw79
Anopheles minimus	98.510952	16.715117	Unclassified	GBIF: https://doi.org/10.15468/dl.zfaw79
Anopheles minimus	98.511546	8.533747	Unclassified	GBIF: https://doi.org/10.15468/dl.zfaw79
Anopheles minimus	98.512302	16.9438	Unclassified	GBIF: https://doi.org/10.15468/dl.zfaw79
Anopheles minimus	98.517059	17.055351	Unclassified	GBIF: https://doi.org/10.15468/dl.zfaw79
Anopheles minimus	98.526925	19.521542	Unclassified	GBIF: https://doi.org/10.15468/dl.zfaw79
Anopheles minimus	98.527557	16.828149	Unclassified	GBIF: https://doi.org/10.15468/dl.zfaw79
Anopheles minimus	98.527969	17.01478	Unclassified	GBIF: https://doi.org/10.15468/dl.zfaw79
Anopheles minimus	98.528075	8.546977	Unclassified	GBIF: https://doi.org/10.15468/dl.zfaw79
Anopheles minimus	98.532184	14.559572	Unclassified	GBIF: https://doi.org/10.15468/dl.zfaw79
Anopheles minimus	98.539884	17.069085	Unclassified	GBIF: https://doi.org/10.15468/dl.zfaw79
Anopheles minimus	98.545316	8.566897	Unclassified	GBIF: https://doi.org/10.15468/dl.zfaw79
Anopheles minimus	98.555653	17.025727	Unclassified	GBIF: https://doi.org/10.15468/dl.zfaw79
Anopheles minimus	98.557819	14.670573	Unclassified	GBIF: https://doi.org/10.15468/dl.zfaw79
Anopheles minimus	98.561985	16.958394	Unclassified	GBIF: https://doi.org/10.15468/dl.zfaw79
Anopheles minimus	98.564605	14.636853	Unclassified	GBIF: https://doi.org/10.15468/dl.zfaw79
Anopheles minimus	98.565348	8.531644	Unclassified	GBIF: https://doi.org/10.15468/dl.zfaw79
Anopheles minimus	98.573818	17.124742	Unclassified	GBIF: https://doi.org/10.15468/dl.zfaw79
Anopheles minimus	98.574929	17.05002	Unclassified	GBIF: https://doi.org/10.15468/dl.zfaw79
Anopheles minimus	98.578127	16.879784	Unclassified	GBIF: https://doi.org/10.15468/dl.zfaw79
Anopheles minimus	98.58263	16.966572	Unclassified	GBIF: https://doi.org/10.15468/dl.zfaw79
Anopheles minimus	98.5863	17.0803	Unclassified	GBIF: https://doi.org/10.15468/dl.zfaw79
Anopheles minimus	98.588792	17.155507	Unclassified	GBIF: https://doi.org/10.15468/dl.zfaw79
Anopheles minimus	98.59043	17.121171	Unclassified	GBIF: https://doi.org/10.15468/dl.zfaw79
Anopheles minimus	98.590765	8.555189	Unclassified	GBIF: https://doi.org/10.15468/dl.zfaw79
Anopheles minimus	98.595281	14.678629	Unclassified	GBIF: https://doi.org/10.15468/dl.zfaw79
Anopheles minimus	98.601544	16.028639	Unclassified	GBIF: https://doi.org/10.15468/dl.zfaw79
Anopheles minimus	98.605142	17.026647	Unclassified	GBIF: https://doi.org/10.15468/dl.zfaw79
Anopheles minimus	98.605882	8.556309	Unclassified	GBIF: https://doi.org/10.15468/dl.zfaw79
Anopheles minimus	98.610329	16.742447	Unclassified	GBIF: https://doi.org/10.15468/dl.zfaw79
Anopheles minimus	98.614228	17.131151	Unclassified	GBIF: https://doi.org/10.15468/dl.zfaw79
Anopheles minimus	98.615168	17.131153	Unclassified	GBIF: https://doi.org/10.15468/dl.zfaw79
Anopheles minimus	98.615629	14.940706	Unclassified	GBIF: https://doi.org/10.15468/dl.zfaw79

Anopheles minimus	98.620219	14.974166	Unclassified	GBIF: https://doi.org/10.15468/dl.zfaw79
Anopheles minimus	98.623709	16.700189	Unclassified	GBIF: https://doi.org/10.15468/dl.zfaw79
Anopheles minimus	98.624785	18.817759	Unclassified	GBIF: https://doi.org/10.15468/dl.zfaw79
Anopheles minimus	98.629776	16.985793	Unclassified	GBIF: https://doi.org/10.15468/dl.zfaw79
Anopheles minimus	98.631538	15.939734	Unclassified	GBIF: https://doi.org/10.15468/dl.zfaw79
Anopheles minimus	98.6327	14.743635	Unclassified	GBIF: https://doi.org/10.15468/dl.zfaw79
Anopheles minimus	98.635298	19.646503	Unclassified	GBIF: https://doi.org/10.15468/dl.zfaw79
Anopheles minimus	98.635782	17.166444	Unclassified	GBIF: https://doi.org/10.15468/dl.zfaw79
Anopheles minimus	98.639253	16.955513	Unclassified	GBIF: https://doi.org/10.15468/dl.zfaw79
Anopheles minimus	98.64074	8.559761	Unclassified	GBIF: https://doi.org/10.15468/dl.zfaw79
Anopheles minimus	98.645267	17.123072	Unclassified	GBIF: https://doi.org/10.15468/dl.zfaw79
Anopheles minimus	98.647403	16.476744	Unclassified	GBIF: https://doi.org/10.15468/dl.zfaw79
Anopheles minimus	98.647404	16.47584	Unclassified	GBIF: https://doi.org/10.15468/dl.zfaw79
Anopheles minimus	98.650163	16.504772	Unclassified	GBIF: https://doi.org/10.15468/dl.zfaw79
Anopheles minimus	98.653037	16.469522	Unclassified	GBIF: https://doi.org/10.15468/dl.zfaw79
Anopheles minimus	98.653801	16.033791	Unclassified	GBIF: https://doi.org/10.15468/dl.zfaw79
Anopheles minimus	98.654738	16.987161	Unclassified	GBIF: https://doi.org/10.15468/dl.zfaw79
Anopheles minimus	98.655272	8.567915	Unclassified	GBIF: https://doi.org/10.15468/dl.zfaw79
Anopheles minimus	98.656019	16.371895	Unclassified	GBIF: https://doi.org/10.15468/dl.zfaw79
Anopheles minimus	98.659846	8.533548	Unclassified	GBIF: https://doi.org/10.15468/dl.zfaw79
Anopheles minimus	98.662471	14.716557	Unclassified	GBIF: https://doi.org/10.15468/dl.zfaw79
Anopheles minimus	98.66423	18.50429	Unclassified	GBIF: https://doi.org/10.15468/dl.zfaw79
Anopheles minimus	98.6667	14.65	Unclassified	GBIF: https://doi.org/10.15468/dl.zfaw79
Anopheles minimus	98.668094	16.42977	Unclassified	GBIF: https://doi.org/10.15468/dl.zfaw79
Anopheles minimus	98.668136	14.655988	Unclassified	GBIF: https://doi.org/10.15468/dl.zfaw79
Anopheles minimus	98.668184	16.737221	Unclassified	GBIF: https://doi.org/10.15468/dl.zfaw79
Anopheles minimus	98.669202	16.328524	Unclassified	GBIF: https://doi.org/10.15468/dl.zfaw79
Anopheles minimus	98.673505	14.79252	Unclassified	GBIF: https://doi.org/10.15468/dl.zfaw79
Anopheles minimus	98.6747	18.48171	Unclassified	GBIF: https://doi.org/10.15468/dl.zfaw79
Anopheles minimus	98.676111	16.677475	Unclassified	GBIF: https://doi.org/10.15468/dl.zfaw79
Anopheles minimus	98.67657	18.49346	Unclassified	GBIF: https://doi.org/10.15468/dl.zfaw79
Anopheles minimus	98.67941	18.49347	Unclassified	GBIF: https://doi.org/10.15468/dl.zfaw79
Anopheles minimus	98.680047	15.98863	Unclassified	GBIF: https://doi.org/10.15468/dl.zfaw79
Anopheles minimus	98.6813	18.49347	Unclassified	GBIF: https://doi.org/10.15468/dl.zfaw79
Anopheles minimus	98.684802	16.523811	Unclassified	GBIF: https://doi.org/10.15468/dl.zfaw79
Anopheles minimus	98.689719	16.378275	Unclassified	GBIF: https://doi.org/10.15468/dl.zfaw79
Anopheles minimus	98.692486	16.405399	Unclassified	GBIF: https://doi.org/10.15468/dl.zfaw79
Anopheles minimus	98.693435	16.397265	Unclassified	GBIF: https://doi.org/10.15468/dl.zfaw79
Anopheles minimus	98.696078	16.504844	Unclassified	GBIF: https://doi.org/10.15468/dl.zfaw79
Anopheles minimus	98.700735	16.522931	Unclassified	GBIF: https://doi.org/10.15468/dl.zfaw79
Anopheles minimus	98.703514	14.590937	Unclassified	GBIF: https://doi.org/10.15468/dl.zfaw79
Anopheles minimus	98.703903	16.286991	Unclassified	GBIF: https://doi.org/10.15468/dl.zfaw79

Anopheles minimus	98.710439	16.297848	Unclassified	GBIF: https://doi.org/10.15468/dl.zfaw79
Anopheles minimus	98.71649	14.608132	Unclassified	GBIF: https://doi.org/10.15468/dl.zfaw79
Anopheles minimus	98.719566	16.461485	Unclassified	GBIF: https://doi.org/10.15468/dl.zfaw79
Anopheles minimus	98.719946	14.674302	Unclassified	GBIF: https://doi.org/10.15468/dl.zfaw79
Anopheles minimus	98.720646	16.361143	Unclassified	GBIF: https://doi.org/10.15468/dl.zfaw79
Anopheles minimus	98.720925	14.670656	Unclassified	GBIF: https://doi.org/10.15468/dl.zfaw79
Anopheles minimus	98.72352	16.315042	Unclassified	GBIF: https://doi.org/10.15468/dl.zfaw79
Anopheles minimus	98.723911	14.613566	Unclassified	GBIF: https://doi.org/10.15468/dl.zfaw79
Anopheles minimus	98.730117	18.827883	Unclassified	GBIF: https://doi.org/10.15468/dl.zfaw79
Anopheles minimus	98.731831	16.398221	Unclassified	GBIF: https://doi.org/10.15468/dl.zfaw79
Anopheles minimus	98.732199	16.809535	Unclassified	GBIF: https://doi.org/10.15468/dl.zfaw79
Anopheles minimus	98.733785	16.33856	Unclassified	GBIF: https://doi.org/10.15468/dl.zfaw79
Anopheles minimus	98.737832	16.807734	Unclassified	GBIF: https://doi.org/10.15468/dl.zfaw79
Anopheles minimus	98.739244	16.457895	Unclassified	GBIF: https://doi.org/10.15468/dl.zfaw79
Anopheles minimus	98.739665	14.639804	Unclassified	GBIF: https://doi.org/10.15468/dl.zfaw79
Anopheles minimus	98.743875	16.414867	Unclassified	GBIF: https://doi.org/10.15468/dl.zfaw79
Anopheles minimus	98.745317	14.570191	Unclassified	GBIF: https://doi.org/10.15468/dl.zfaw79
Anopheles minimus	98.746245	14.570192	Unclassified	GBIF: https://doi.org/10.15468/dl.zfaw79
Anopheles minimus	98.751836	14.55121	Unclassified	GBIF: https://doi.org/10.15468/dl.zfaw79
Anopheles minimus	98.752905	14.475528	Unclassified	GBIF: https://doi.org/10.15468/dl.zfaw79
Anopheles minimus	98.753768	16.080034	Unclassified	GBIF: https://doi.org/10.15468/dl.zfaw79
Anopheles minimus	98.754286	14.714072	Unclassified	GBIF: https://doi.org/10.15468/dl.zfaw79
Anopheles minimus	98.755966	16.619599	Unclassified	GBIF: https://doi.org/10.15468/dl.zfaw79
Anopheles minimus	98.760464	16.365653	Unclassified	GBIF: https://doi.org/10.15468/dl.zfaw79
Anopheles minimus	98.763621	16.426822	Unclassified	GBIF: https://doi.org/10.15468/dl.zfaw79
Anopheles minimus	98.7676	16.38704	Unclassified	GBIF: https://doi.org/10.15468/dl.zfaw79
Anopheles minimus	98.767819	16.057449	Unclassified	GBIF: https://doi.org/10.15468/dl.zfaw79
Anopheles minimus	98.769608	16.918056	Unclassified	GBIF: https://doi.org/10.15468/dl.zfaw79
Anopheles minimus	98.773157	16.566119	Unclassified	GBIF: https://doi.org/10.15468/dl.zfaw79
Anopheles minimus	98.777978	16.987669	Unclassified	GBIF: https://doi.org/10.15468/dl.zfaw79
Anopheles minimus	98.781407	16.426601	Unclassified	GBIF: https://doi.org/10.15468/dl.zfaw79
Anopheles minimus	98.787977	16.445064	Unclassified	GBIF: https://doi.org/10.15468/dl.zfaw79
Anopheles minimus	98.789505	16.674094	Unclassified	GBIF: https://doi.org/10.15468/dl.zfaw79
Anopheles minimus	98.793828	18.802629	Unclassified	GBIF: https://doi.org/10.15468/dl.zfaw79
Anopheles minimus	98.799834	16.418367	Unclassified	GBIF: https://doi.org/10.15468/dl.zfaw79
Anopheles minimus	98.80316	14.501957	Unclassified	GBIF: https://doi.org/10.15468/dl.zfaw79
Anopheles minimus	98.806739	16.394307	Unclassified	GBIF: https://doi.org/10.15468/dl.zfaw79
Anopheles minimus	98.809225	16.741822	Unclassified	GBIF: https://doi.org/10.15468/dl.zfaw79
Anopheles minimus	98.8133	8.855	Unclassified	GBIF: https://doi.org/10.15468/dl.zfaw79
Anopheles minimus	98.832576	14.575694	Unclassified	GBIF: https://doi.org/10.15468/dl.zfaw79
Anopheles minimus	98.83265	16.776194	Unclassified	GBIF: https://doi.org/10.15468/dl.zfaw79
Anopheles minimus	98.83333	14.56667	Unclassified	GBIF: https://doi.org/10.15468/dl.zfaw79

Anopheles minimus	98.834309	14.498046	Unclassified	GBIF: https://doi.org/10.15468/dl.zfaw79
Anopheles minimus	98.836142	16.435759	Unclassified	GBIF: https://doi.org/10.15468/dl.zfaw79
Anopheles minimus	98.838156	14.19671	Unclassified	GBIF: https://doi.org/10.15468/dl.zfaw79
Anopheles minimus	98.85765	14.43021	Unclassified	GBIF: https://doi.org/10.15468/dl.zfaw79
Anopheles minimus	98.86224	16.066571	Unclassified	GBIF: https://doi.org/10.15468/dl.zfaw79
Anopheles minimus	98.87541	18.764716	Unclassified	GBIF: https://doi.org/10.15468/dl.zfaw79
Anopheles minimus	98.8842	18.747948	Unclassified	GBIF: https://doi.org/10.15468/dl.zfaw79
[truncated: 547,556 more chars]
